# Establishing the Proteome of Normal Human Cerebrospinal Fluid

**DOI:** 10.1371/journal.pone.0010980

**Published:** 2010-06-11

**Authors:** Steven E. Schutzer, Tao Liu, Benjamin H. Natelson, Thomas E. Angel, Athena A. Schepmoes, Samuel O. Purvine, Kim K. Hixson, Mary S. Lipton, David G. Camp, Patricia K. Coyle, Richard D. Smith, Jonas Bergquist

**Affiliations:** 1 Departments of Medicine and Neurology, University of Medicine and Dentistry of New Jersey-New Jersey Medical School, Newark, New Jersey, United States of America; 2 Biological Sciences Division, Pacific Northwest National Laboratory, Richland, Washington, United States of America; 3 Department of Neurology, State University of New York-Stony Brook, Stony Brook, New York, United States of America; 4 Uppsala University, Department of Physical and Analytical Chemistry, Uppsala, Sweden; University of Nebraska, United States of America

## Abstract

**Background:**

Knowledge of the entire protein content, the proteome, of normal human cerebrospinal fluid (CSF) would enable insights into neurologic and psychiatric disorders. Until now technologic hurdles and access to true normal samples hindered attaining this goal.

**Methods and Principal Findings:**

We applied immunoaffinity separation and high sensitivity and resolution liquid chromatography-mass spectrometry to examine CSF from healthy normal individuals. 2630 proteins in CSF from normal subjects were identified, of which 56% were CSF-specific, not found in the much larger set of 3654 proteins we have identified in plasma. We also examined CSF from groups of subjects previously examined by others as surrogates for normals where neurologic symptoms warranted a lumbar puncture but where clinical laboratory were reported as normal. We found statistically significant differences between their CSF proteins and our non-neurological normals. We also examined CSF from 10 volunteer subjects who had lumbar punctures at least 4 weeks apart and found that there was little variability in CSF proteins in an individual as compared to subject to subject.

**Conclusions:**

Our results represent the most comprehensive characterization of true normal CSF to date. This normal CSF proteome establishes a comparative standard and basis for investigations into a variety of diseases with neurological and psychiatric features.

## Introduction

Knowledge of the entire protein content, the proteome, of normal human cerebrospinal fluid (CSF) would provide a critical standard to allow meaningful comparisons with and between neurologic and psychiatric disorders. CSF contains both cellular and soluble components providing insights into processes occurring in the central nervous system (CNS). As much as 30 to 40% of CSF is formed by the extracellular fluid of the brain and spinal cord. CSF contains both normal and disease specific components, and provides an accessible liquid window into the brain. In fact, recent data suggest CSF may provide more relevant evidence for initial or propagating pathology than the brain parenchyma itself in certain neuropsychiatric diseases[1]. Comprehensive characterization of the normal CSF proteome would facilitate identification of disease-specific markers[2]. Knowledge of which proteins are present, absent, or of changed concentrations may lead to diagnostic, prognostic, or disease-activity biomarkers as well as provide insights into disease etiology and pathogenesis. An advantage of a full proteome analysis is the ability to identify not just one but a multitude of proteins at a single instance. We had a unique opportunity to generate what may be the most comprehensive database of true normal CSF proteins to date. We were able to do this because we had sufficient numbers and total volume of true normal CSF samples to employ immunoaffinity depletion followed by extensive fractionation and high-resolution liquid chromatography (LC) separation and mass spectrometry (MS) analysis. The combination of our normal CSF samples, including a set of serial CSF samples, and advanced technology contribute to the uniqueness and value of our study.

Until recently, technological limitations have prevented full characterization of the CSF proteome. Comprehensive analysis of CSF has been challenged by low protein levels (0.3 to 0.7 mg/ml) compared to plasma, protein concentration variability up to twelve orders of magnitude, potential masking of brain-specific proteins by highly abundant proteins[3], and limited access to an adequate number of appropriate biological samples. Despite some of these limitations, a number of earlier studies have provided increasing levels of characterization of the CSF proteome. For the most part, these studies have used pooled samples from patient populations with diseases or from people with normal CSF clinical laboratory values (chemistries, cell counts, and microbiology) who underwent lumbar puncture for investigation of neurological complaints. These CSF samples were used as substitutes or surrogates for true normals (healthy volunteers) due to lack of availability of such normal CSF samples. Sickmann et al.,[4] used two-dimensional polyacrylamide gel electrophoresis (2D-PAGE) followed by mass spectrometry (MS) to identify close to 70 CSF proteins. Yuan et al.,[5] used matrix-assisted laser desorption ionization (MALDI) MS to identify 22 proteins in desalted CSF. Wenner et al.,[6] used 2D liquid chromatography (LC) coupled to tandem mass spectrometry (2D-LC-MS/MS) to identify 249 proteins in pooled CSF. Maccarrone et al.[7], used immunodepletion techniques and shotgun LC-MS/MS, to identify more than 100 proteins in CSF from a patient with normal pressure hydrocephalus. More recently Zougman et al.[8], using LC-MS/MS, reported 798 proteins in a pool of 6 patients with neurologic complaints that warranted a lumbar puncture, but whose subsequent clinical CSF laboratory values were reported as normal; for the purposes of this paper we term these types of patients as neurologic surrogate-normals. A notable exception to use of surrogates was the work by Zhang et al.[9], who used 2D-LC-MS/MS to identify 315 proteins in pools of CSF comparing healthy younger versus older individuals. Subsequently, with Xu et al. [10], they analyzed CSF from the younger group with two different LC-MS/MS platforms and identified a combined total of 915 proteins. Pan et al., reported a total of 2594 CSF protein identifications from different combined (cumulative) results of several CSF studies with a focus on neurodegenerative diseases such as Alzheimer's disease[11].

Towards our principal purpose of establishing a comprehensive list or proteome of normal CSF, we have prepared CSF samples from healthy normal people for analysis by using immunoaffinity depletion of abundant proteins (with masking potential) to enhance coverage and detection of low abundance proteins[12]. We then analyzed the samples employing high throughput, high sensitivity, and high resolution nanocapillary liquid chromatography-mass spectrometry (LC-MS[13] and LC-MS/MS[12]). We used the pre-fractionation (immunoaffinity depletion chromatography) and ultra-high resolution nanocapillary LC separations to effectively reduce the sample complexity and concentration dynamic range (thereby reducing or eliminating the “masking” effect[14]), high efficiency ion transmission technologies (e.g., electrodynamic ion funnel[15]) for highly sensitive global MS analysis, and the accurate mass and time (AMT) tag strategy[16] for high-throughput analysis (e.g. of individual CSF samples) and accurate quantitation. Our general approach for AMT tag generation and application has been successfully implemented for whole microbial[17] and mammalian tissue[13] and plasma proteomes[12], [18], but has not been previously applied to CSF from normal subjects.

We examined pooled CSF from 11 normal healthy volunteers (8 women and 3 men, aged 24 to 55, median  = 28 years) who reported their health as excellent or good and were taking no medications. Standard clinical laboratory testing on their CSF was normal (none had more than 3 white blood cells/mm^3^ and protein levels ranged from 14 to 40 mg/dl with a median of 25 mg/dl). We also examined pairs of individual serial CSF samples, obtained at least 4 weeks apart, from 10 additional normal healthy volunteers to assess the potential variability of particular CSF protein levels in an individual from one time point to another.

To illustrate the utility of such a normal database and how one clinical condition might be compared to another we began to analyze and compare one set of CSF samples to another set processed in the same manner. We were particularly interested in seeing if there might be significant differences among different surrogate-normal groups.

## Materials and Methods

### Cerebrospinal Fluid (CSF) specimens

All specimens had normal clinical laboratory values with respect to microbiology, chemistry (including protein levels), and cell counts (red blood cells were 0–10/mm^3^and white blood cells were 0–5/mm^3^). Four sets of different types of normal CSF samples were analyzed. The first set, designated as true (healthy) normals was comprised of pooled CSF from 11 healthy normal individual volunteers from the United States (8 women and 3 men; aged 24 to 55 years with a median age of 28 years) was used for the comprehensive analysis using immunoaffinity depletion and 2D-LC-MS/MS. A second set, also true normals included pairs of serial CSF aliquots taken at least 4 weeks apart from 10 healthy volunteers from the United States (age 37–44 years; 5 males and 5 females). A third set, designated as non-neurologic surrogate-normals, was a pool of 200 subjects from Sweden (all without a neurologic or psychiatric disease, most who underwent lumbar puncture for non diagnostic reasons; over 90% were undergoing spinal anesthesia in preparation for orthopedic surgery (e.g. limbs-knees and hips)). Ages ranged from 16 to 65 years with a median of 44 years; 50∶50 female:male. They were used in the direct LC-MS analysis using the AMT tag approach. These samples were collected on ice and cells removed by centrifugation[19]. A fourth set, designated as neurologic surrogate-normals consisted of a pool of CSF from 10 people from Sweden with headaches (age 18–35 years; 8 female and 2 male) who had a lumbar puncture to investigate possible CNS infection, and who had normal CSF clinical laboratory values (hence designation surrogate-normal), was collected following the same protocol as the third set and analyzed in the same fashion as the second and third sets of normal CSF. CSF from this group was also subjected to centrifugation to remove cells. CSF from this group was collected following the same protocol as the pool of the 200 non-neurological surrogate-normals. Approval for the conduct of this study was obtained from our Institutional Review Boards in accordance with federal regulations. The protein concentrations were determined by Coomassie Plus protein assay (Pierce, Rockford, IL) using a bovine serum albumin standard.

### Immunoaffinity depletion of 14 high-abundance CSF proteins

A total of 18 mL of the pooled CSF sample (from the 11 healthy volunteers) was subjected to the separations of 14 high-abundance proteins (albumin, IgG, α_1_-antitrypsin, IgA, IgM, transferrin, haptoglobin, α_1_-acid glycoprotein, α_2_-macroglobulin, apolipoprotein A-I, apolipoprotein A-II, fibrinogen, C3 and apolipoprotein B) using a 12.7×79.0 mm Seppro® IgY14 LC10 affinity LC column (Sigma, St Louis, MO) on an Agilent 1100 series HPLC system (Agilent, Palo Alto, CA), followed the protocols described previously[20].

### Protein digestion

The CSF proteins were incubated in 8 M urea and 10 mM dithiothreitol at 37°C for 60 min, followed by alkylation with 40 mM iodoacetamide in the dark for 30 min at room temperature. The samples were diluted 10-fold with 50 mM ammonium bicarbonate (pH 8) and 1 mM CaCl_2_, and digested for 3 h at 37°C using sequencing grade, modified porcine trypsin (Promega, Madison, WI) at a trypsin/protein ratio of 1∶50. Sample cleanup was achieved using a 1-mL SPE C18 column (Supelco, Bellefonte, PA) as described previously[21]. The final peptide concentration was determined by BCA assay (Pierce). All tryptic digests were snap frozen in liquid nitrogen and stored at -80°C.

### Strong cation exchange (SCX) fractionation

For tryptic digests of the IgY14 bound and flow-through fractions (the first set normal CSF pool from BN), 300 µg of tryptic peptides from CSF samples were resuspended in 300 µL 10 mM ammonium formate, 25% acetonitrile and fractionated by strong cation exchange chromatography as described previously[21]. A total of 30 fractions were collected for each sample with each fraction being lyophilized prior to reversed-phase LC-MS/MS analysis.

### Reversed-phase capillary LC-MS/MS and LC-MS analysis

The SCX fractions were analyzed using a custom-built automated four-column high pressure nanocapillary LC system coupled on-line to either a linear ion trap mass spectrometer (LTQ; ThermoFisher) or a linear quadrupole ion trap-orbitrap mass spectrometer (LTQ-Orbitrap, ThermoFisher), both modified in-house with an electrodynamic ion funnel[15], via an electrospray ionization interface manufactured in-house. The reversed-phase separation was performed as described previously[21]. To analyze the SCX fractions of the IgY14 bound fraction, the LTQ mass spectrometer was operated in a data-dependent MS/MS mode (m/z 400–2000) in which a full MS scan was followed by 10 MS/MS scans. The ten most intensive precursor ions were dynamically selected in the order of highest intensity to lowest intensity and subjected to collision-induced dissociation using a normalized collision energy setting of 35% and a dynamic exclusion duration of 1 min. The heated capillary was maintained at 200°C, while the ESI voltage was kept at 2.2 kV. The SCX fractions of the IgY14 flow-through fraction, which are enriched with lower abundance proteins, were analyzed by the LTQ-Orbitrap instrument operated in a data-dependent MS/MS mode with survey full scan MS spectra (m/z 400–2000) acquired in the orbitrap with resolution of 30,000 at m/z 400 (ion accumulation target: 1,000,000), followed by MS/MS of the 10 most intense ions. In the case of label-free quantitation using the unfractionated CSF samples (the second and third set of normal CSF and the headache CSF), the LTQ-Orbitrap MS was operated in the data dependent mode with survey full scan spectra (m/z 400–2000) acquired in the orbitrap with resolution of 60,000 at m/z 400 (accumulation target: 1,000,000). The six most intense ions were sequentially isolated for fragmentation and detection in the linear ion trap.

### Data analysis

The LTQ LC-MS/MS raw data were converted into .dta file by Extract_MSn (version 3.0) in Bioworks Cluster 3.2 (Thermo) and the SEQUEST algorithm (version 27 revision 12) was used to independently search all the MS/MS spectra against the human International Protein Index (IPI) database with a total of 69,731 total protein entries (Version 3.40, released at February 7, 2008, available on-line at www.ebi.ac.uk/ipi). The search parameters used were: 3-Da tolerance for precursor ion masses and 1-Da tolerance for fragment ion masses with no enzyme restraint and a maximum of 2 missed tryptic cleavages. Static carboxyamidomethylation of cysteine and dynamic oxidation of methionine were used during the database search. LTQ-Orbitrap MS/MS data were first processed by an in-house software DeconMSn[22] to accurately determine the monoisotopic mass and charge state of parent ions, followed by SEQUEST search against the IPI database in the same fashion, except that 0.1-Da tolerance for precursor ion masses and 1-Da tolerance for fragment ion masses were used. A set of criteria considering the cross correlation score (Xcorr) and delta correlation (ΔCn) values along with tryptic cleavage and charge states were developed using the decoy database approach and applied for filtering the raw data to limit false positive identifications to <1% at the peptide level[23]–[25]. For the LTQ-Orbitrap data, the distribution of mass deviation (from the theoretical masses) was first determined as having a standard deviation (σ) of 2.05 part per million (ppm), and peptide identifications with mass error of greater than 3σ were filtered out[23], [25], [26]. In general, slightly lower Xcorr cutoff values were used when combined with ΔCn and the mass error constraint to achieve the same level of false positive rate (<1%). For peptides identified by both LTQ-Orbitrap (IgY14 flow-through fraction) and LTQ (IgY14 bound fraction) analyses, the database matching scores are shown only for the LTQ-Orbitrap analysis, along with their mass errors (Table S1).

The AMT tag strategy[16] was used for identifying and quantifying LC-MS features measured by LTQ-Orbitrap. The filtered MS/MS peptide identifications obtained from the LTQ and LTQ-Orbitrap analyses of CSF samples were included in an AMT tag database with their theoretical mass and normalized elution time (NET; from 0 to 1) recorded. LC-MS datasets were then analyzed by in-house software VIPER[27] that detects features in mass–NET space and assigned them to peptides in the AMT tag database[28]. A 11-Da shift strategy analogous to the decoy database approach used for LC-MS/MS identification of peptides was applied for estimating the false discovery rate of the AMT analysis as previously described[13]. A false positive rate of <4% was estimated for each of the LC-MS data sets. The resulting lists of peptides from 2D-LC-MS/MS or direct LC-MS analysis was further analyzed by ProteinProphet software[29] to remove redundancy in protein identification as described previously^1^.

Data normalization and quantification of the changes in protein abundance between the normal and headache CSF samples were performed and visualized using in-house software DAnTE[30]. Briefly, peptide intensities from the LC-MS analyses were log2 transformed and normalized using a mean central tendency procedure. Peptide abundances were then “rolled up” to the protein level employing the R-rollup method (based on trends observed at peptide level) implemented in DAnTE. ANOVA and clustering analyses were also performed using DAnTE.

Gene ontology annotation was performed using a software tool STRAP[31]. The final distribution charts were generated using Excel.

## Results

Here we present a comprehensive analysis of the CSF proteome from healthy normal individuals providing the foundation for future investigation on this biological fluid which may be highly reflective of the status of the brain and central nervous system. Our primary goal was to provide a comprehensive coverage of CSF proteins from normal healthy individuals. From the pool of 11 CSF samples from healthy volunteers, we identified with high confidence a total of 19,051 tryptic peptides, covering 2630 non-redundant proteins, with 1506 having at least two peptide identifications (see Table S1). The immunoaffinity-based partitioning generated a separate bound fraction consisting of the 14 most abundant proteins and their potential associated proteins, and a flow-through fraction enriched with the less abundant proteins in CSF. Similar to plasma, the bound fraction represents approximately 95% of the total protein mass (Figure S1). Both fractions were subjected to 2D-LC-MS/MS analysis.

This set of 2630 CSF proteins, and a comprehensive set of 3654 proteins from our previous plasma database[21], showed very similar distribution of gene ontology terms in biological process and molecular function, but different distributions by cellular component: approximately a total of 35% of the CSF proteins are from plasma membrane, cell surface or extracellular space, while plasma have a total of 28% of proteins in those three categories; there are also less CSF proteins in the nucleus and cytoplasm (10% versus 15% and 11% versus 16%, respectively; Figures S2, S3, and S4). Importantly, nearly 56% of the proteins are CSF-specific and are not present in our larger plasma database of 3654 proteins, also analyzed by LC-MS/MS [21], (see Table S2). This is notable because the acquisition and analysis conditions likely favored the set of plasma proteins as opposed to the CSF proteins. This is because more proteins were likely available for detection in the plasma of burn patients due to severe tissue leakage, and there was the additional dimension of sample fractionation via enrichment of cysteinyl and N-linked glycopeptides. We point out that this is not a head-to-head comparison because of the differences in sample type and conditions, extensiveness of fractionation and MS instrumentation. It was beyond the scope of this initial study to determine what CSF proteins are not detectable in plasma under normal conditions, or vice versa.

**Figure 1:**
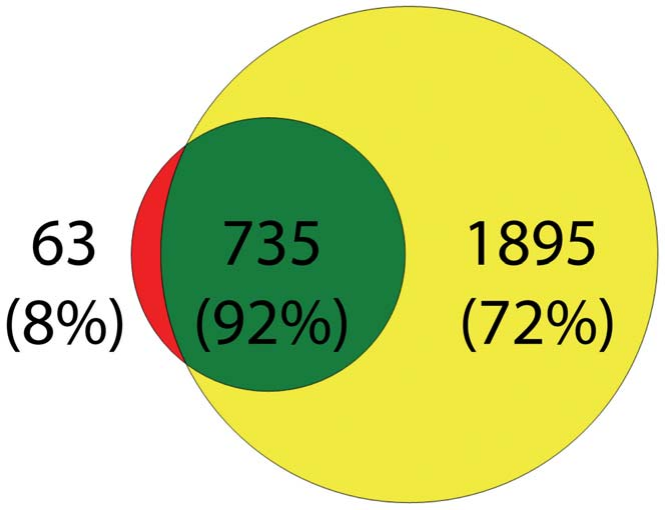
Venn diagram showing the amount of overlap of our dataset with a comparable dataset of proteins detected in the CSF of “normal clinical value” or “neurologic surrogate-normal” individuals who required a lumbar puncture for clinical reasons as reported by Zougman et al[**8**]. The large circle represents the **2630** proteins observed in our comprehensive dataset of proteins detected in the CSF of normal individuals. The small circle represents the **798** proteins identified in the analysis by Zougman et al.

Comparison between proteins detected in this study and those (which we have termed neurologic surrogate-normals) from the CSF study by Zougman et al., reveals a 92% overlap (see Figure 1 and Table S2).

**Figure 2:**
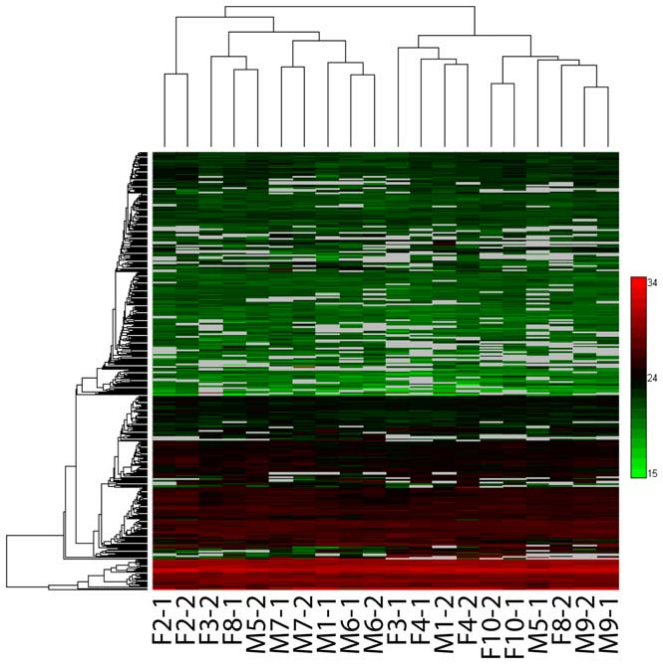
Unsupervised hierarchical clustering of all proteins identified and quantified in direct LC-MS analyses of CSF samples from 10 normal healthy individuals (5 males and 5 females; 37–44 years old; each has two longitudinal samples collected at least 4 weeks apart). Log2 transformed protein abundances were used. M: male; F: female; numbers right after the hyphen indicate the two serial samples from the same individual.

In order to assess the CSF protein variability from serial sample collections, we next examined individual (non-pooled) samples from another group of 10 healthy volunteers (5 male, 5 female; age range 37–44 years old) who had two CSF samples obtained at least 4 weeks apart using the AMT tag approach. Inter-subject differences were far greater than intra-subject differences (Figure 2). We performed statistical tests of variance of differences (ANOVA) for these data sets based on different factors (e.g., subject, gender, and time of sampling), followed by unsupervised hierarchical clustering analysis of the statistically significant proteins (p-value <0.01). It is clear that human heterogeneity is the major factor responsible for inter-sample differences; clustering of the “significant” proteins could not distinguish corresponding groups based on the other factors we defined in the ANOVA analysis (i.e., gender and time of sampling; see Figures S6 and S7), except for “subject” (Figure S5).

**Figure 3:**
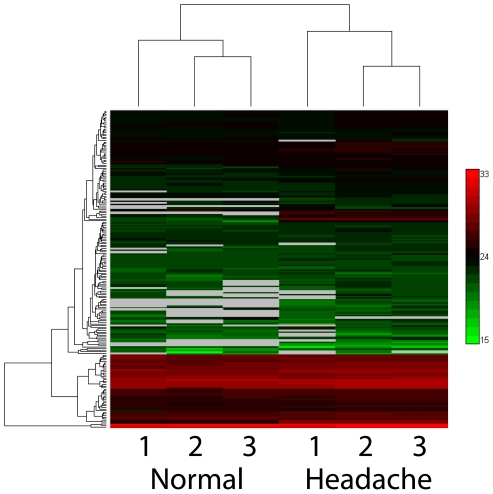
Unsupervised hierarchical clustering of all proteins identified and quantified in direct replicate LC-MS analyses of pooled CSF from non-neurologic surrogate-normal individuals (n = 200) and neurologic surrogate-normal (headache) patients (n = 10). Log2 transformed protein abundances were used.

As an example of how CSF proteomic databases may be used to better understand disease states, we compared the proteomes of two similarly processed (see Methods) pooled samples of patients using the AMT tag strategy[16]. The first set, considered as neurologic surrogate-normals was a pool of 10 headache patients. CSF had been obtained to evaluate the possibility of a CNS infection or bleed but all clinical results were normal. The second set, considered as non-neurologic surrogate-normals, was a pool of 200 subjects (without a neurologic disease, who underwent lumbar puncture for non diagnostic reasons; over 90% were undergoing spinal anesthesia in preparation for orthopedic surgery (limbs-knees and hips)). We found significantly distinct results between each group. Specifically, we identified 191±7 and 211±8 non-redundant proteins from the 3 replicates of each data set. Statistical analysis comparing these CSF data sets showed that the neurologic surrogate-normal CSF pool had distinctive quantitative differences compared to the non-neurologic surrogate normal pool (22 proteins with p-value <0.01 by ANOVA; see Table S3). Unsupervised hierarchical clustering of abundances of all proteins clearly separates these two groups (Figure 3). One interesting difference was our identification and quantification of certain hemoglobin isoforms, which were among significantly changed proteins identified by our statistical analysis (Table S3), in our neurologic surrogate-normal (headache) samples; Although Zougman et al[8] previously identified these same proteins in a qualitative analysis of their neurologic surrogate-normal samples, we were able to discern that these isoforms were increased by about ten-fold on average. The differences found in this study suggest it would be attractive to extend these studies with immunoaffinity depletion applied to different defined categories of headache subjects.

## Discussion

This study provides the most comprehensive CSF protein coverage and list reported to date for healthy normal individuals including serial lumbar punctures. The protein set has immediate utility for investigators interested in using CSF to study neurological or psychiatric diseases. It will serve as a normative base to which disease states may be compared. Our study also suggests CSF protein variability over a short time is relatively limited in an individual. If this observation is supported by larger scale studies, it would further facilitate the utility of disease-state sample comparative analyses.

The other major previous investigations of CSF from healthy individuals were published by Zhang et al [9] and updated by part of that group, Xu et al[10]. What began as detection of approximately 315 proteins was expanded to 915 using different mass spectrometry methods. Interestingly Xu et al[10] stated that they believed their coverage of the normal CSF was insufficient because they were unable to detect two well known CSF proteins, α-synuclein[5] and gelsolin[32]. Our methods and approach differed from theirs, and included a rigorous separation of abundant from less abundant proteins to mitigate the masking effect of the most abundant proteins, as well as high-resolution LC coupled to MS/MS analysis for highly efficient peptide identification. We identified 2630 proteins in total, including α-synuclein and gelsolin.

Because of the challenge in obtaining CSF from healthy people, most previous studies may have used CSF from “surrogate-normals,” that is CSF collected from people with neurological complaints such as headache but with normal clinical CSF laboratory values.

We compared proteomes of two different surrogate-normal groups. We found significant differences between the two groups. This study supports the potential usefulness of the normal human CSF proteome data library as an invaluable tool in investigating pathophysiological abnormalities in neurological and psychiatric disorders.

Proteomic databases can be used in several ways. One of our own perspectives on using these proteomic databases for studying diseases involves a stepwise strategy. The first step would be a comparison of pooled samples representative of the disease to normal subjects or a comparator disease. The second step involves the selection of specific candidate proteins. The selection of candidate proteins is not likely to be predicted in advance and may require bioinformatic strategies and knowledge related to the disease under study. A third step would involve analysis of the individual samples contributing to the pool to ascertain how many of the samples actually contained one or more of the candidate proteins. This step provides a check in the event that a single individual in the pool disproportionately contributes a protein compared to other subjects. We would subject the results to statistical analyses. In the case of a search for biomarker proteins we strive to select those that meet clinically useful criteria, such as presence, absence or relative abundance in a large percentage of disease subjects and not so in most subjects without the disease under consideration. The fourth step would involve verification of the previous results using independent individual samples with the same disease. A final validation step may involve analyzing a larger number of subjects with the disease and controls using assays targeted to the candidate proteins. In contrast to the discovery phases, it would be advantageous, if feasible, to use assay platforms already having wide clinical use. Immunobased assays such as ELISA and Western blots may serve this purpose being relatively inexpensive. Steps 3 and 4 will likely employ a type of mass spectrometry which targets selected candidate proteins, such as Multiple Reaction Monitoring (MRM) using triple quadrupole instrumentation.

The availability of the data presented here, detailing the normal human CSF proteome, should prove to be a critical base on which to compare proteins, both qualitatively and quantitatively, in studies of patients with a variety of neurological or psychiatric diseases.

## Supporting Information

Figure S1 Immunoaffinity depletion of plasma and CSF samples using the IgY14 LC10 column.
(0.30 MB TIF)

Figure S2 Comparison of the distributions of the gene ontology terms for all proteins identified from the healthy normal CSF sample and those for the 3654 plasma proteins reported by us previously (Text Reference 21). Biological process.
(0.74 MB TIF)

Figure S3 Comparison of the distributions of the gene ontology terms for all proteins identified from the healthy normal CSF sample and those for the 3654 plasma proteins reported by us previously (text reference 21). Cellular component.
(0.85 MB TIF)

Figure S4 Comparison of the distributions of the gene ontology terms for all proteins identified from the healthy normal CSF sample and those for the 3654 plasma proteins reported by us previously (text reference 21). Molecular function.
(0.71 MB TIF)

Figure S5 Unsupervised hierarchical clustering analysis of 88 proteins found to be present at significantly different levels (p-values <0.01; ANOVA was performed based on individual differences) comparing serial CSF samples from 10 individuals (5 males and 5 females; 37–44 years old; each has two longitudinal samples collected at least 4 weeks apart). Log2 transformed protein abundances were used. M: male; F: female; numbers right after the hyphen indicate the two serial samples from the same individual.
(0.31 MB TIF)

Figure S6 Unsupervised hierarchical clustering analysis of 9 proteins found to be present at significantly different levels (p-values <0.01; ANOVA was based on gender differences) comparing serial CSF samples from 10 individuals (5 males and 5 females; 37–44 years old; each has two longitudinal samples collected at least 4 weeks apart). Log2 transformed protein abundances were used. M: male; F: female; numbers right after the hyphen indicate the two serial samples from the same individual.
(0.28 MB TIF)

Figure S7 Unsupervised hierarchical clustering analysis of 2 proteins found to be present at significantly different levels (p-values <0.01; ANOVA was based on differences in the time of sampling, i.e., visit 1 vs. visit 2) comparing serial CSF samples from 10 individuals (5 males and 5 females; 37–44 years old; each has two longitudinal samples collected at least 4 weeks apart). Log2 transformed protein abundances were used. M: male; F: female; numbers right after the hyphen indicate the two serial samples from the same individual.
(0.26 MB TIF)

Table S1 Peptides detected in CSF from healthy normal individuals using immunoaffinity depletion and 2D-LC-MS/MS.
(2.36 MB PDF)

Table S2 Analysis of overlap between proteins identified in normal CSF, plasma and previous CSF (neurologic surrogate-normal) proteomic study.
(0.24 MB PDF)

Table S3 Proteins identified and quantified from direct LC-MS analysis of CSF from non-neurologic and neurologic (headache) surrogate-normals.
(0.02 MB PDF)
