# Supplementary figures and images for: Establishing the Proteome of Normal Human Cerebrospinal Fluid
Source: PLoS One. 2010 Jun 11;5(6):e10980. doi: 10.1371/journal.pone.0010980 (PMC2881861; doi:10.1371/journal.pone.0010980)

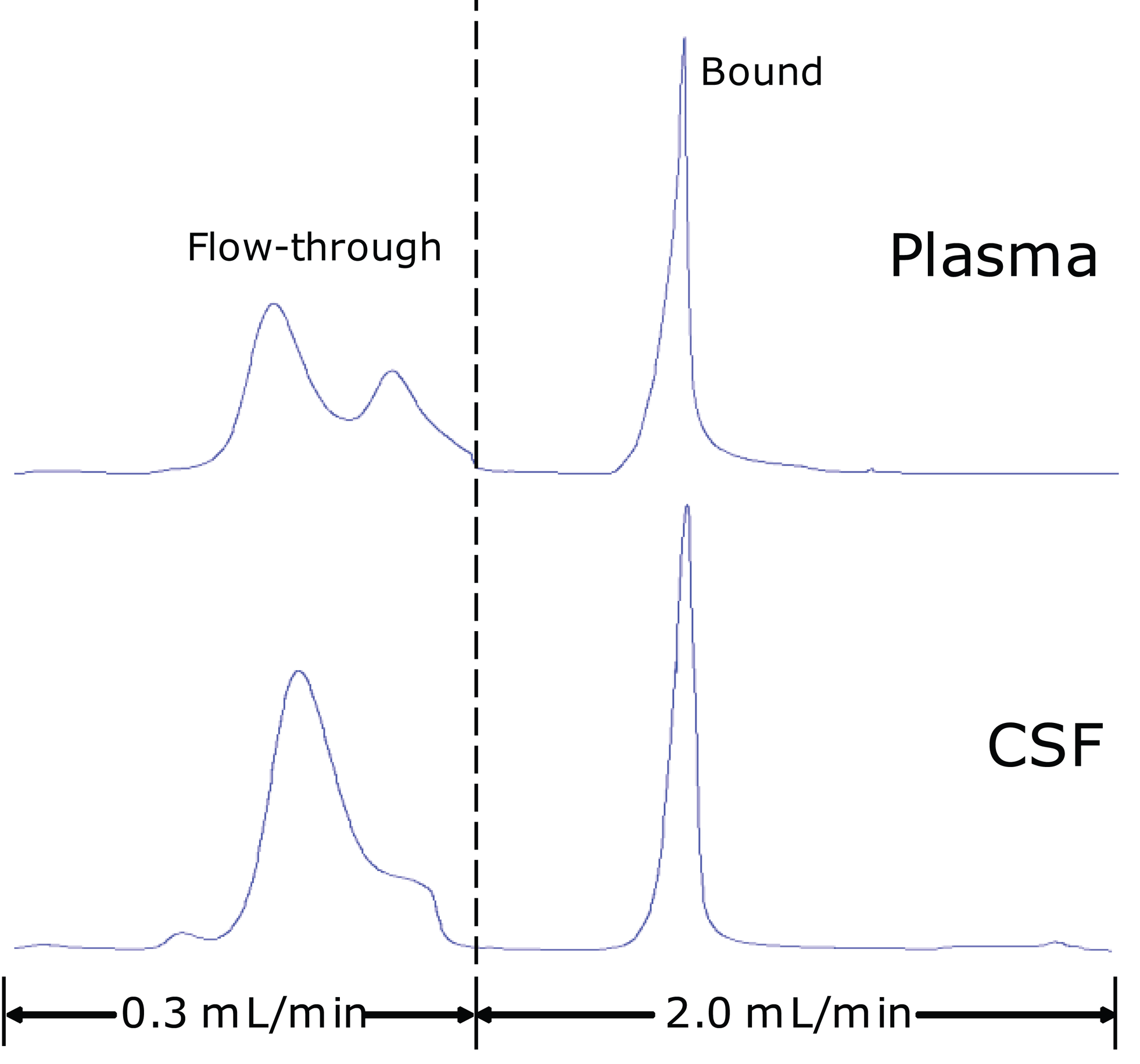

Supplement: Figure S1 — Immunoaffinity depletion of plasma and CSF samples using the IgY14 LC10 column. (0.30 MB TIF) [file pone.0010980.s001.tif]

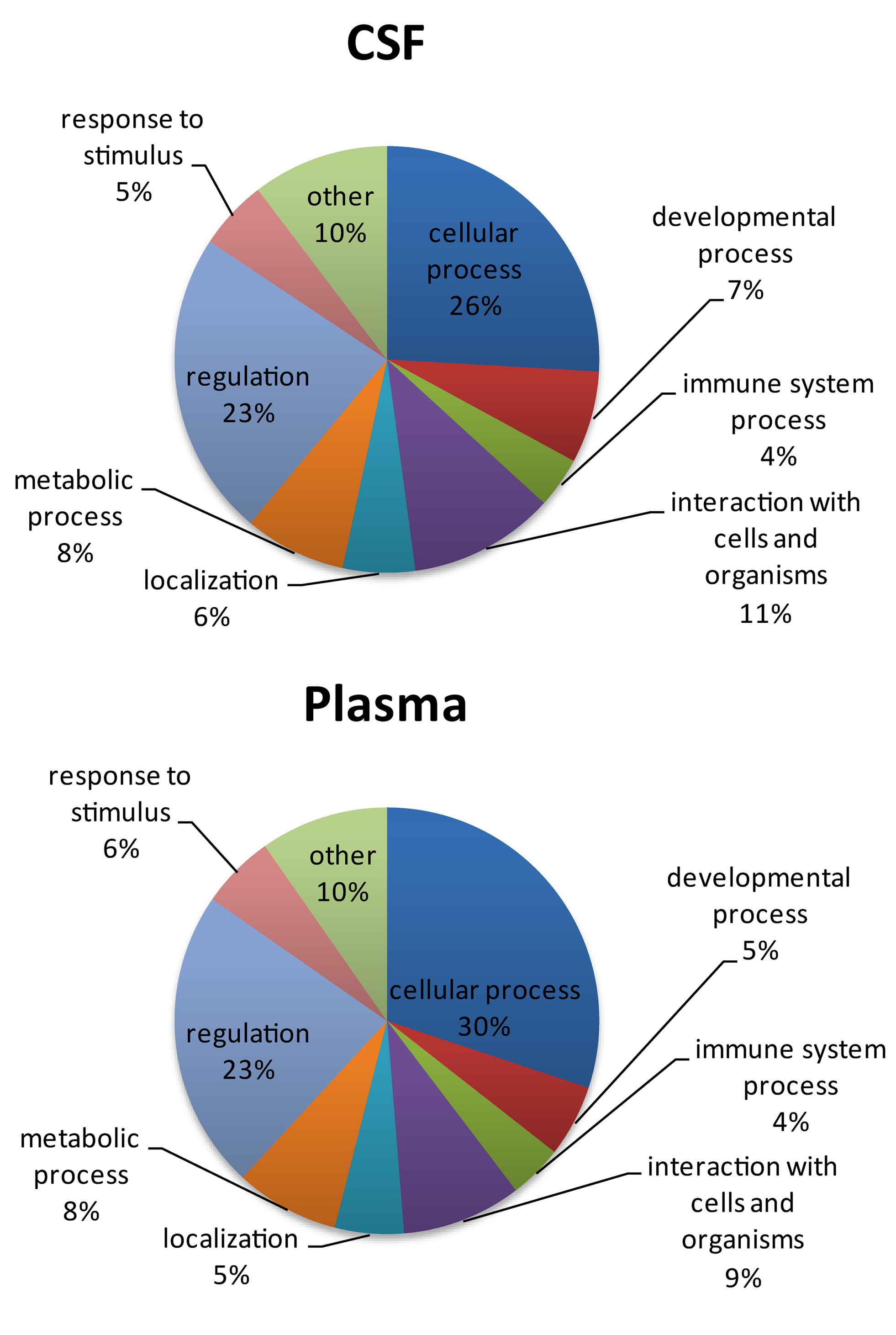

Supplement: Figure S2 — Comparison of the distributions of the gene ontology terms for all proteins identified from the healthy normal CSF sample and those for the 3654 plasma proteins reported by us previously (Text Reference 21). Biological process. (0.74 MB TIF) [file pone.0010980.s002.tif]

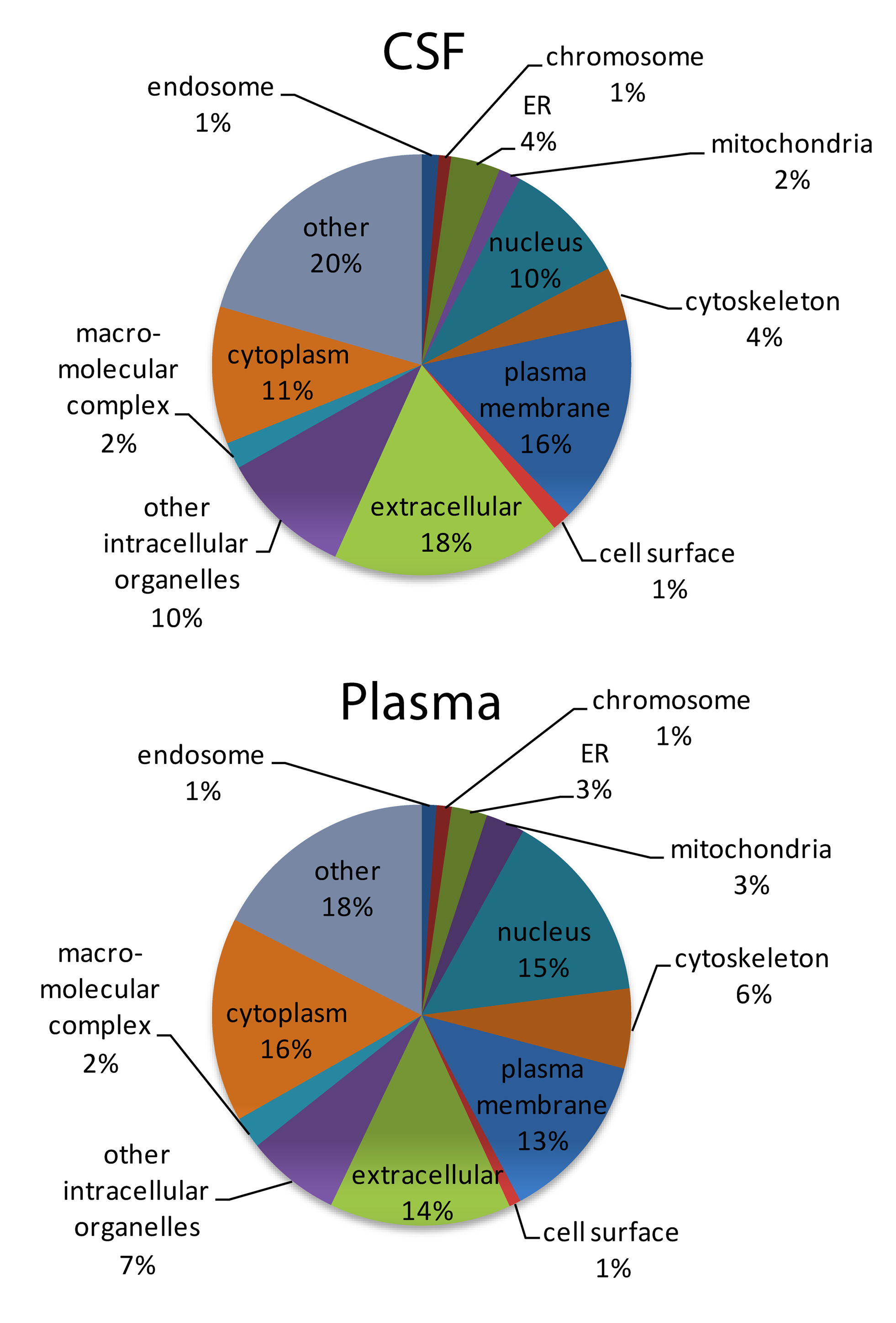

Supplement: Figure S3 — Comparison of the distributions of the gene ontology terms for all proteins identified from the healthy normal CSF sample and those for the 3654 plasma proteins reported by us previously (text reference 21). Cellular component. (0.85 MB TIF) [file pone.0010980.s003.tif]

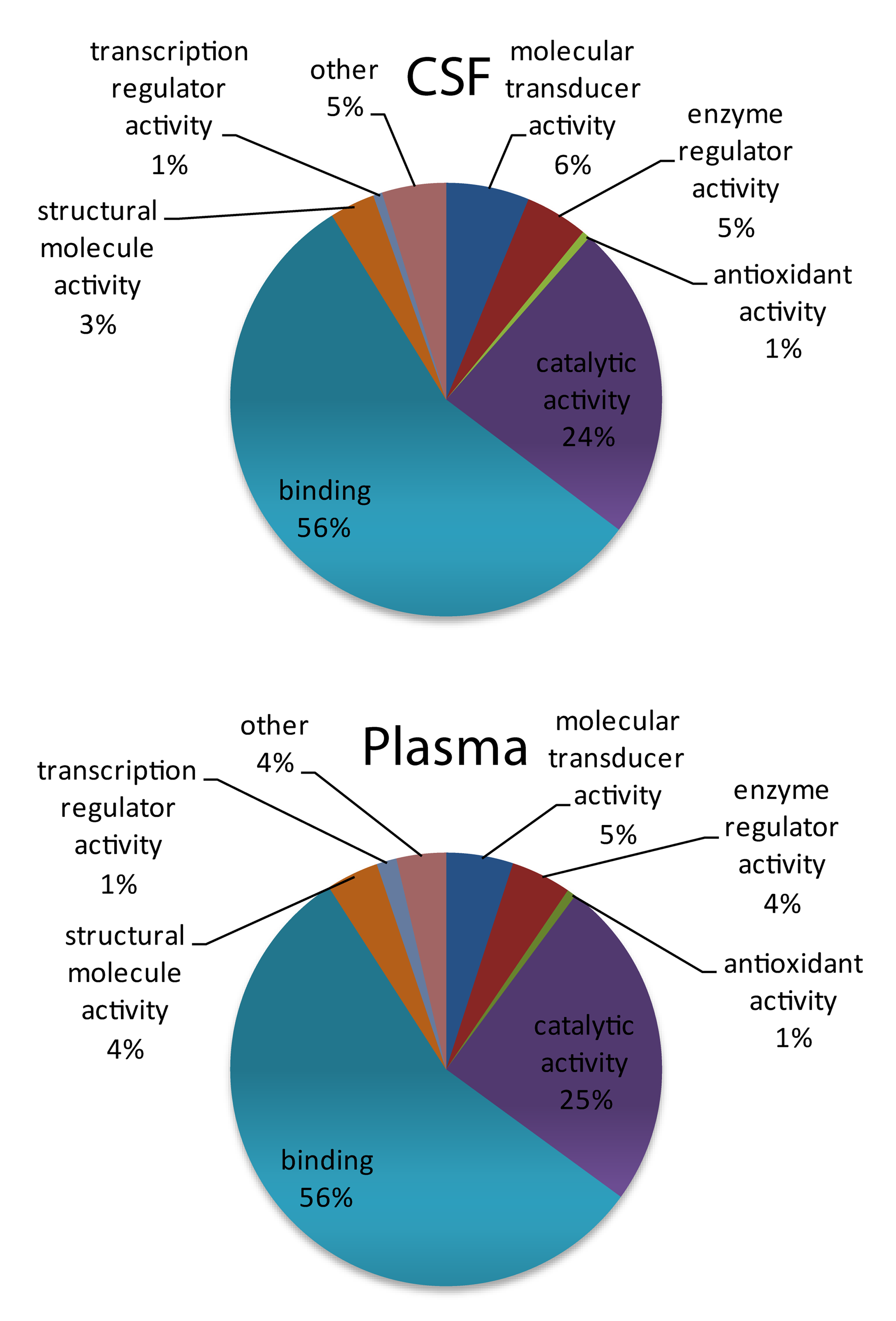

Supplement: Figure S4 — Comparison of the distributions of the gene ontology terms for all proteins identified from the healthy normal CSF sample and those for the 3654 plasma proteins reported by us previously (text reference 21). Molecular function. (0.71 MB TIF) [file pone.0010980.s004.tif]

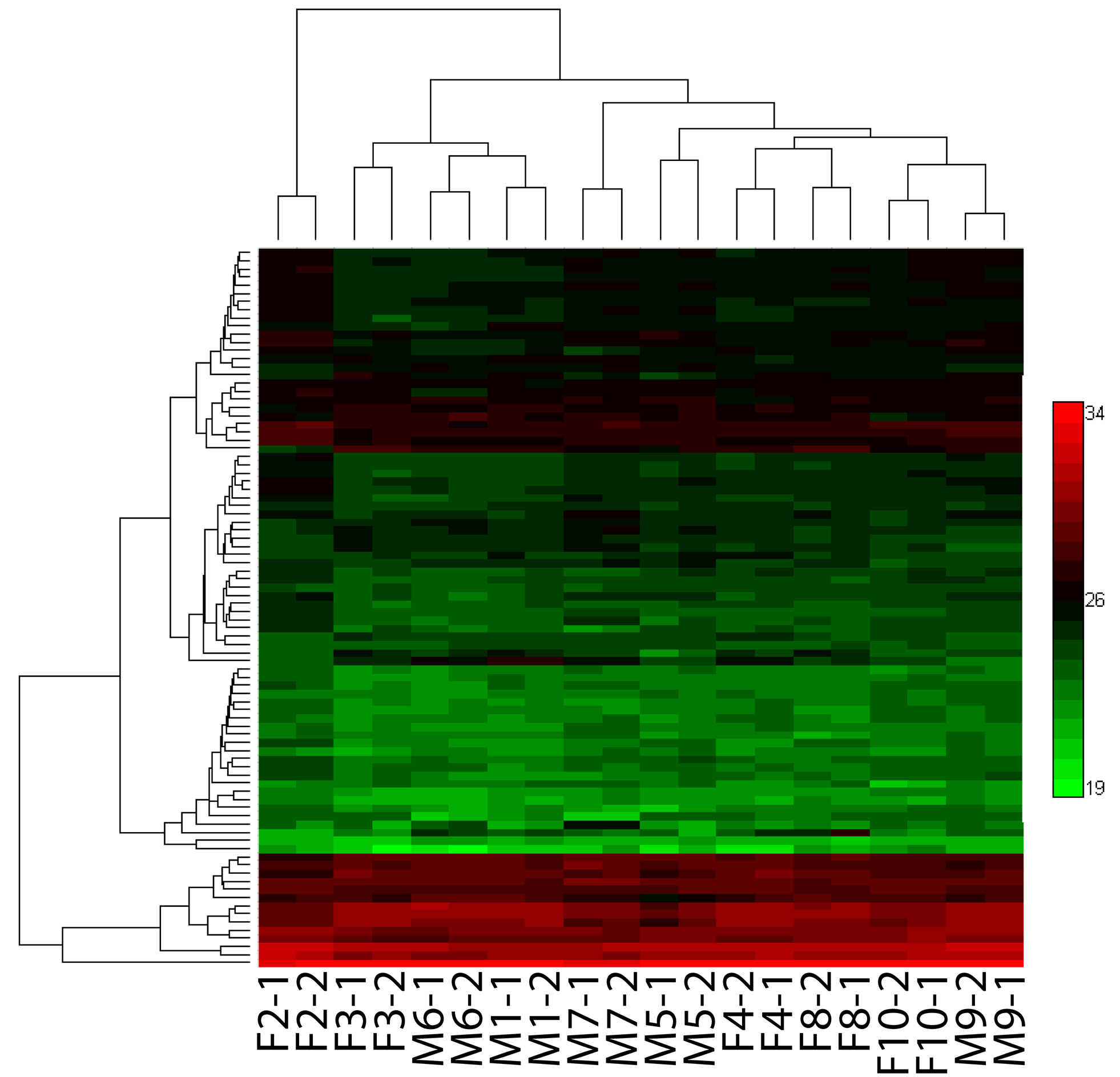

Supplement: Figure S5 — Unsupervised hierarchical clustering analysis of 88 proteins found to be present at significantly different levels (p-values <0.01; ANOVA was performed based on individual differences) comparing serial CSF samples from 10 individuals (5 males and 5 females; 37–44 years old; each has two longitudinal samples collected at least 4 weeks apart). Log2 transformed protein abundances were used. M: male; F: female; numbers right after the hyphen indicate the two serial samples from the same individual. (0.31 MB TIF) [file pone.0010980.s005.tif]

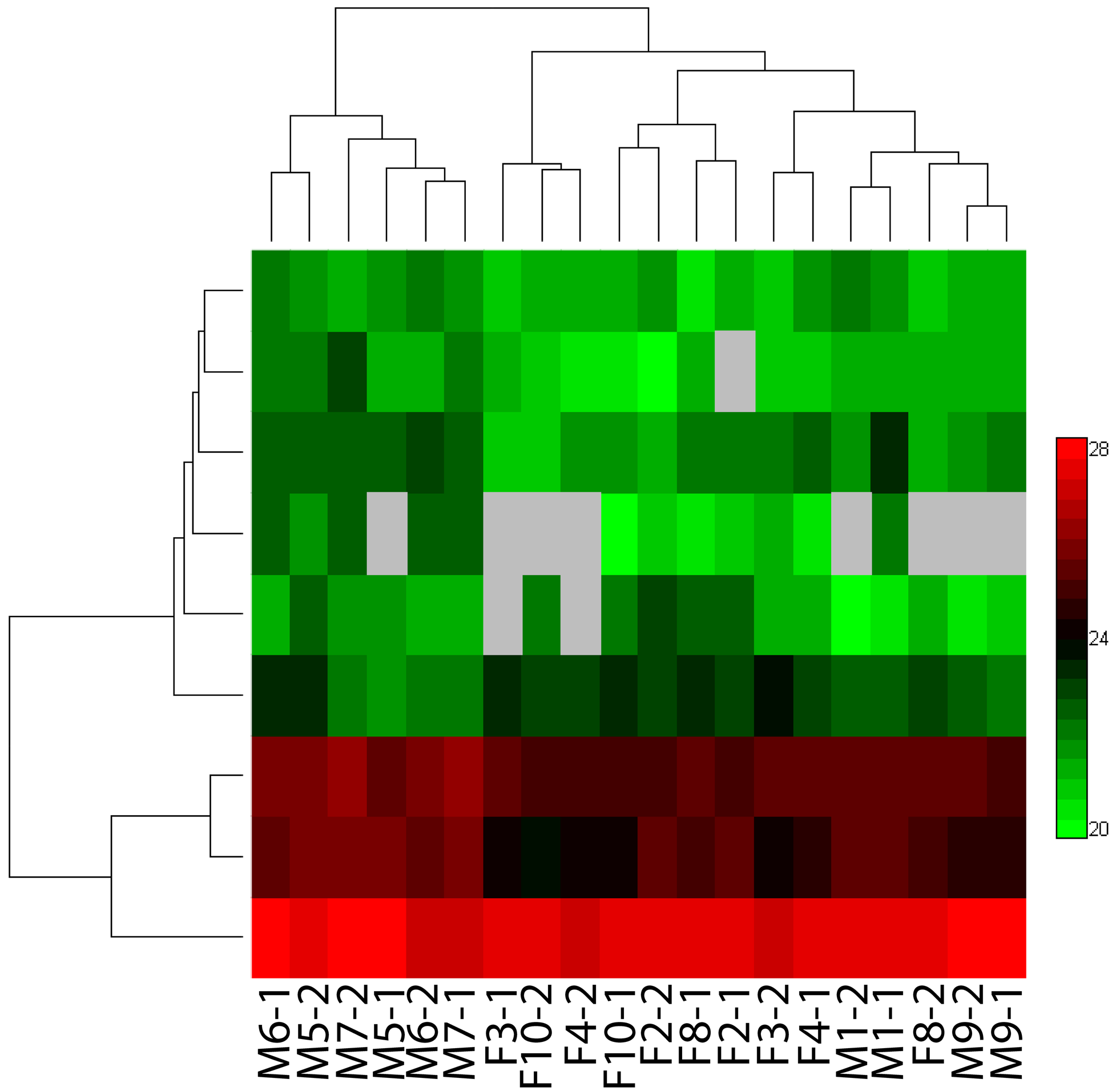

Supplement: Figure S6 — Unsupervised hierarchical clustering analysis of 9 proteins found to be present at significantly different levels (p-values <0.01; ANOVA was based on gender differences) comparing serial CSF samples from 10 individuals (5 males and 5 females; 37–44 years old; each has two longitudinal samples collected at least 4 weeks apart). Log2 transformed protein abundances were used. M: male; F: female; numbers right after the hyphen indicate the two serial samples from the same individual. (0.28 MB TIF) [file pone.0010980.s006.tif]

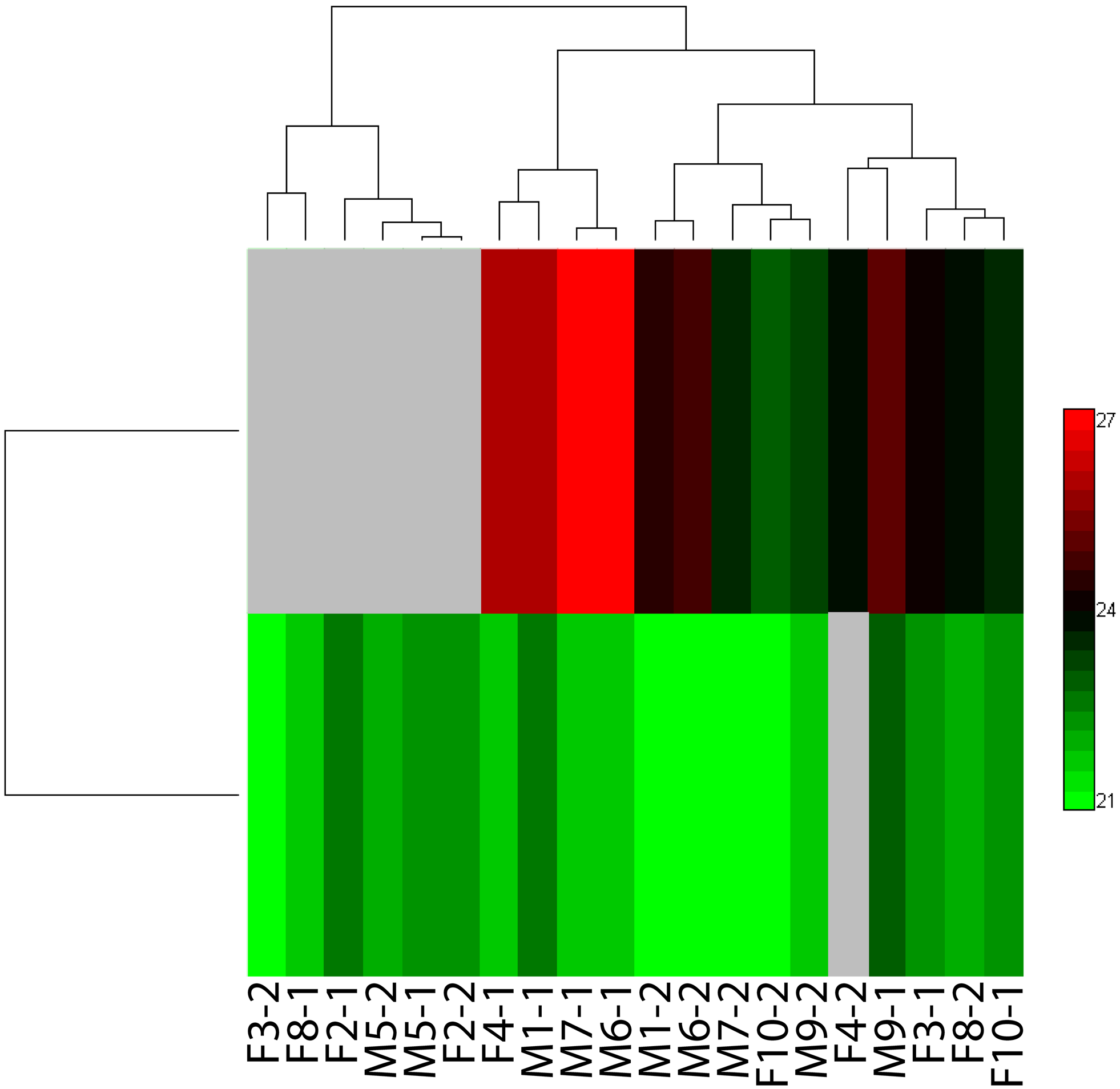

Supplement: Figure S7 — Unsupervised hierarchical clustering analysis of 2 proteins found to be present at significantly different levels (p-values <0.01; ANOVA was based on differences in the time of sampling, i.e., visit 1 vs. visit 2) comparing serial CSF samples from 10 individuals (5 males and 5 females; 37–44 years old; each has two longitudinal samples collected at least 4 weeks apart). Log2 transformed protein abundances were used. M: male; F: female; numbers right after the hyphen indicate the two serial samples from the same individual. (0.26 MB TIF) [file pone.0010980.s007.tif]
