# Supplementary material for: Establishing the Proteome of Normal Human Cerebrospinal Fluid
Source: PLoS One. 2010 Jun 11;5(6):e10980. doi: 10.1371/journal.pone.0010980 (PMC2881861; doi:10.1371/journal.pone.0010980)
Supplement: Table S1 — Peptides detected in CSF from healthy normal individuals using immunoaffinity depletion and 2D-LC-MS/MS. (2.36 MB PDF) [file pone.0010980.s008.pdf]

Supplementary Table 1. Peptides detected in CSF from healthy normal individuals using immunoaffinity depletion and 2D-LC-MS/MS.						
**IPI**	**Protein name**	**Peptide Sequence**	**Charge State**	**XCorr**	**DeltaCn**	**Mass Error (ppm)**
IPI00000027	Pituitary adenylate cyclase-activating polypeptide precursor	R.FPGIRPEEEAYGEDGNPLPDFDGSEPPGAGSPASAPR.A	4	3.89	0.30	-1.67
IPI00000044	Platelet-derived growth factor B chain precursor	A.EGDPIPEELYEM*LSDHSIR.S	2	3.62	0.49	-2.66
IPI00000044	Platelet-derived growth factor B chain precursor	R.SFDDLQR.L	1	1.69	0.06	-3.38
IPI00000044	Platelet-derived growth factor B chain precursor	R.SHSGGELESLAR.G	1	2.84	0.27	-3.62
IPI00000044	Platelet-derived growth factor B chain precursor	R.SHSGGELESLAR.G	2	2.93	0.42	-1.85
IPI00000044	Platelet-derived growth factor B chain precursor	R.SHSGGELESLAR.G	3	2.51	0.07	-3.61
IPI00000070	Low-density lipoprotein receptor precursor	R.NEFQCQDGK.C	2	2.52	0.25	-3.05
IPI00000076	Beta-nerve growth factor precursor	R.SAPAAAIAAR.V	2	2.33	0.15	0.10
IPI00000087	Sodium channel subunit beta-2 precursor	K.YDVSVM*LR.N	2	2.56	0.22	-2.01
IPI00000087	Sodium channel subunit beta-2 precursor	R.NVQPEDEGIYNCYIM*NPPDR.H	2	4.83	0.57	-4.61
IPI00000104	Isoform 1 of mRNA-capping enzyme	R.WLNCPR.R	2	1.52	0.21	
IPI00000130	Somatostatin precursor	A.ELLSEPNQTENDALEPEDLSQAAEQDEM*RLELQR.S	3	3.94	0.32	-3.54
IPI00000130	Somatostatin precursor	E.LLSEPNQTENDALEPEDLSQAAEQDEM*RLELQR.S	3	4.91	0.48	0.32
IPI00000130	Somatostatin precursor	F.LAELLSEPNQTENDALEPEDLSQAAEQDEM*RLELQR.S	3	4.20	0.43	-3.19
IPI00000130	Somatostatin precursor	K.SLAAAAGK.Q	1	1.90	0.20	-1.34
IPI00000130	Somatostatin precursor	K.SLAAAAGKQELAK.Y	2	3.06	0.25	-0.59
IPI00000130	Somatostatin precursor	K.YFLAELLSEPNQTENDALEPEDLSQAAEQDEM*RLELQR.S	3	4.57	0.43	-3.87
IPI00000130	Somatostatin precursor	K.YFLAELLSEPNQTENDALEPEDLSQAAEQDEM*RLELQR.S	4	5.10	0.44	-4.13
IPI00000130	Somatostatin precursor	L.AELLSEPNQTENDALEPEDLSQAAEQDEM*RLELQR.S	3	4.63	0.51	-2.19
IPI00000130	Somatostatin precursor	L.LSEPNQTENDALEPEDLSQAAEQDEM*R.L	3	4.96	0.52	-1.84
IPI00000130	Somatostatin precursor	L.LSEPNQTENDALEPEDLSQAAEQDEM*RLELQR.S	3	4.31	0.48	-3.33
IPI00000130	Somatostatin precursor	L.SEPNQTENDALEPEDLSQAAEQDEM*RLELQR.S	3	4.67	0.51	-1.17
IPI00000130	Somatostatin precursor	R.SANSNPAM*APR.E	2	3.50	0.24	-3.09
IPI00000130	Somatostatin precursor	R.SANSNPAM*APRE.R	2	3.34	0.36	-3.26
IPI00000130	Somatostatin precursor	Y.FLAELLSEPNQTENDALEPEDLSQAAEQDEM*RLELQR.S	3	4.73	0.44	-4.20
IPI00000137	N-acetylglucosamine-1-phosphotransferase subunit gamma precursor	K.CFSLVESTYK.Y	2	3.06	0.44	-2.94
IPI00000137	N-acetylglucosamine-1-phosphotransferase subunit gamma precursor	K.M*KVVEEPNAFGVNNPFLPQASR.L	3	4.78	0.37	-2.86
IPI00000137	N-acetylglucosamine-1-phosphotransferase subunit gamma precursor	K.RDPSPVSGPVHLFR.L	2	3.74	0.41	-4.61
IPI00000137	N-acetylglucosamine-1-phosphotransferase subunit gamma precursor	K.RDPSPVSGPVHLFR.L	3	5.30	0.50	-3.20
IPI00000137	N-acetylglucosamine-1-phosphotransferase subunit gamma precursor	K.TPEENEPTQLEGGPDSLGFETLENCRK.A	3	4.56	0.43	-2.36
IPI00000137	N-acetylglucosamine-1-phosphotransferase subunit gamma precursor	K.VVEEPNAFGVNNPFLPQASR.L	2	5.50	0.49	-3.34

IPI00000137	N-acetylglucosamine-1-phosphotransferase subunit gamma precursor	K.VVEEPNAFGVNNPFLPQASR.L	3	2.68	0.14	-3.04
IPI00000137	N-acetylglucosamine-1-phosphotransferase subunit gamma precursor	R.DPSPVSGPVHLFR.L	2	2.53	0.20	-3.16
IPI00000137	N-acetylglucosamine-1-phosphotransferase subunit gamma precursor	R.QWDQVEQDLADELITPQGHEK.L	3	4.52	0.31	-4.62
IPI00000137	N-acetylglucosamine-1-phosphotransferase subunit gamma precursor	R.TLFEDAGYLK.T	2	2.64	0.11	-2.98
IPI00000137	N-acetylglucosamine-1-phosphotransferase subunit gamma precursor	R.TLFEDAGYLKTPEENEPTQLEGGPDSLGFETLENCRK.A	4	5.04	0.37	-2.69
IPI00000138	Alpha-1,3-mannosyl-glycoprotein 2-beta-N-acetylglucosaminyltransferase	K.EQM*VDASRPELLYR.T	3	2.41	0.14	-0.64
IPI00000138	Alpha-1,3-mannosyl-glycoprotein 2-beta-N-acetylglucosaminyltransferase	K.LNQQFVHFTQLDLSYLQR.E	3	3.33	0.24	-2.34
IPI00000138	Alpha-1,3-mannosyl-glycoprotein 2-beta-N-acetylglucosaminyltransferase	R.FPAAVVVEDDLEVAPDFFEYFR.A	3	3.49	0.34	-4.69
IPI00000138	Alpha-1,3-mannosyl-glycoprotein 2-beta-N-acetylglucosaminyltransferase	R.GIVTFQFR.G	2	2.68	0.25	-1.50
IPI00000138	Alpha-1,3-mannosyl-glycoprotein 2-beta-N-acetylglucosaminyltransferase	R.GLLQQIGDALSSQR.G	2	3.72	0.41	-5.25
IPI00000138	Alpha-1,3-mannosyl-glycoprotein 2-beta-N-acetylglucosaminyltransferase	R.VYGAPQLQVEK.V	2	3.02	0.17	-3.78
IPI00000138	Alpha-1,3-mannosyl-glycoprotein 2-beta-N-acetylglucosaminyltransferase	R.WALGQVFR.Q	2	1.86	0.13	-0.54
IPI00000144	Oxytocin-neurophysin 1 precursor	R.CFGPNICCAEELGCFVGTAEALR.C	2	5.44	0.57	-2.33
IPI00000144	Oxytocin-neurophysin 1 precursor	R.CFGPNICCAEELGCFVGTAEALR.C	3	3.95	0.30	-2.92
IPI00000160	Proopiomelanocortin preproprotein	R.LREGDGPDGPADDGAGAQADLEHSLLVAAEK.K	3	4.94	0.46	-1.97
IPI00000160	Proopiomelanocortin preproprotein	R.LREGDGPDGPADDGAGAQADLEHSLLVAAEK.K	4	4.42	0.34	-2.45
IPI00000190	CD81 antigen	K.QFYDQALQQAVVDDDANNAK.A	3	4.48	0.39	-4.07
IPI00000265	Uncharacterized protein C10orf38 precursor	R.ALRLPENTSYSDLTAFLTAASSPSEVDSFPYLR.G	3	4.64	0.37	-3.85
IPI00000459	Transmembrane gamma-carboxyglutamic acid protein 1 precursor	R.ANGFFEEIR.Q	2	2.32	0.24	-2.13
IPI00000760	N(G),N(G)-dimethylarginine dimethylaminohydrolase 2	R.EFFVGLSK.W	1	2.13	0.25	-1.89
IPI00000760	N(G),N(G)-dimethylarginine dimethylaminohydrolase 2	R.IVEIGDENATLDGTDVLFTGR.E	2	4.99	0.51	-5.54
IPI00000760	N(G),N(G)-dimethylarginine dimethylaminohydrolase 2	R.IVEIGDENATLDGTDVLFTGR.E	3	2.62	0.20	-5.65
IPI00000760	N(G),N(G)-dimethylarginine dimethylaminohydrolase 2	R.TVVAGSSDAAQK.A	2	3.35	0.37	-2.38
IPI00000775	Isoform 1 of Leucine-rich repeats and immunoglobulin-like domains protein 1 precursor	K.DGGTDFPAAR.E	2	2.77	0.28	-3.70

IPI00000775	Isoform 1 of Leucine-rich repeats and immunoglobulin-like domains protein 1 precursor	K.DGGTDFPAARE.R	2	3.04	0.33	-4.68
IPI00000779	Isoform 1 of ADAM 22 precursor	C.GQAGDASLM*ELEK.R	2	4.32	0.52	-3.23
IPI00000779	Isoform 1 of ADAM 22 precursor	C.GQAGDASLM*ELEKR.K	2	3.26	0.39	-0.16
IPI00000779	Isoform 1 of ADAM 22 precursor	F.ILDVVLNHDLLSSEYIER.H	3	4.05	0.33	-1.53
IPI00000779	Isoform 1 of ADAM 22 precursor	G.QAGDASLM*ELEK.R	2	3.55	0.36	-1.98
IPI00000779	Isoform 1 of ADAM 22 precursor	K.CTLTQDSQCSDGLCCK.K	2	5.15	0.57	-5.19
IPI00000779	Isoform 1 of ADAM 22 precursor	K.FAISENPLITLR.E	2	4.06	0.46	-4.90
IPI00000779	Isoform 1 of ADAM 22 precursor	K.LNIEGTEK.G	2	2.53	0.07	-1.94
IPI00000779	Isoform 1 of ADAM 22 precursor	K.M*DGYSCDGVQGICFGGR.C	2	4.74	0.59	-2.61
IPI00000779	Isoform 1 of ADAM 22 precursor	K.SPSSSTGSIASSR.K	2	3.83	0.45	-2.01
IPI00000779	Isoform 1 of ADAM 22 precursor	K.SRLFEFSLDDLPSEFQQVNITPSK.F	3	4.26	0.35	-3.35
IPI00000779	Isoform 1 of ADAM 22 precursor	K.SVVNM*ADLIYKDQLK.T	3	1.86	0.17	-1.30
IPI00000779	Isoform 1 of ADAM 22 precursor	K.TDLM*AVTLAQSLAHNIGIISDK.R	2	5.35	0.52	-3.26
IPI00000779	Isoform 1 of ADAM 22 precursor	K.TDLM*AVTLAQSLAHNIGIISDK.R	3	4.50	0.53	-4.21
IPI00000779	Isoform 1 of ADAM 22 precursor	R.DVLCGYLLCTNIGNIPR.L	2	4.16	0.46	-3.14
IPI00000779	Isoform 1 of ADAM 22 precursor	R.LFEFSLDDLPSEFQQVNITPSK.F	2	5.06	0.56	-5.31
IPI00000779	Isoform 1 of ADAM 22 precursor	R.LFEFSLDDLPSEFQQVNITPSK.F	3	4.64	0.47	-4.25
IPI00000779	Isoform 1 of ADAM 22 precursor	R.LSVVHTNTYAK.S	1	2.89	0.40	-0.32
IPI00000779	Isoform 1 of ADAM 22 precursor	R.LSVVHTNTYAK.S	2	2.56	0.19	-3.20
IPI00000779	Isoform 1 of ADAM 22 precursor	R.LSVVHTNTYAK.S	3	2.21	0.28	-1.27
IPI00000779	Isoform 1 of ADAM 22 precursor	R.NVEEETKYIELM*IVNDHLM*FKK.H	4	2.90	0.10	-4.63
IPI00000779	Isoform 1 of ADAM 22 precursor	R.SGAAYIGGICSLLK.G	1	3.20	0.36	-2.93
IPI00000779	Isoform 1 of ADAM 22 precursor	R.SGAAYIGGICSLLK.G	2	4.41	0.37	-3.21
IPI00000779	Isoform 1 of ADAM 22 precursor	R.SGGEDESRHDALDTR.V	2	2.68	0.20	-4.42
IPI00000779	Isoform 1 of ADAM 22 precursor	R.SGGEDESRHDALDTR.V	3	2.68	0.34	-2.52
IPI00000779	Isoform 1 of ADAM 22 precursor	R.SGGEDESRHDALDTR.V	4	2.50	0.29	-3.74
IPI00000792	Quinone oxidoreductase	R.KPLLPYTPGSDVAGVIEAVGDNASAFK.K	3	2.97	0.23	-2.24
IPI00000811	Proteasome subunit beta type-6 precursor	R.VTDKLTPIHDR.I	3	2.79	0.21	
IPI00000816	14-3-3 protein epsilon	K.AAFDDAIAELDTLSEESYK.D	3	4.91	0.50	-2.47
IPI00000816	14-3-3 protein epsilon	K.AAFDDAIAELDTLSEESYKDSTLIM*QLLR.D	3	5.76	0.54	-4.57
IPI00000816	14-3-3 protein epsilon	K.AAFDDAIAELDTLSEESYKDSTLIM*QLLR.D	4	4.59	0.26	-3.92
IPI00000816	14-3-3 protein epsilon	K.AAFDDAIAELDTLSEESYKDSTLIMQLLR.D	3	4.26	0.41	
IPI00000816	14-3-3 protein epsilon	K.AASDIAM*TELPPTHPIR.L	2	3.03	0.14	-0.15
IPI00000816	14-3-3 protein epsilon	K.AASDIAM*TELPPTHPIR.L	3	2.92	0.28	-1.03
IPI00000816	14-3-3 protein epsilon	K.EAAENSLVAYK.A	2	2.05	0.11	-4.91
IPI00000816	14-3-3 protein epsilon	K.HLIPAANTGESK.V	2	2.17	0.14	-0.49
IPI00000816	14-3-3 protein epsilon	K.LICCDILDVLDKHLIPAANTGESK.V	4	2.96	0.22	-2.29
IPI00000816	14-3-3 protein epsilon	R.IISSIEQKEENKGGEDKLK.M	3	3.51	0.18	-4.05
IPI00000816	14-3-3 protein epsilon	R.NLLSVAYK.N	1	1.68	0.08	-2.46
IPI00000816	14-3-3 protein epsilon	R.NLLSVAYK.N	2	2.28	0.09	-1.47
IPI00000816	14-3-3 protein epsilon	R.YDEM*VESM*K.K	2	3.00	0.36	-2.18

IPI00000816	14-3-3 protein epsilon	R.YDEM*VESM*KK.V	2	1.44	0.05	-1.17
IPI00000816	14-3-3 protein epsilon	R.YLAEFATGNDRK.E	2	2.84	0.35	-3.90
IPI00000824	Isoform A of NT-3 growth factor receptor precursor	K.LNSQNLYCINADGSQLPLFR.M	2	4.03	0.46	-4.22
IPI00000824	Isoform A of NT-3 growth factor receptor precursor	K.LNSQNLYCINADGSQLPLFR.M	3	4.07	0.29	-5.38
IPI00000824	Isoform A of NT-3 growth factor receptor precursor	R.SLHTLNAVDM*ELYTGLQK.L	3	3.50	0.18	-2.40
IPI00000824	Isoform A of NT-3 growth factor receptor precursor	R.VVSLEEPELR.L	2	2.72	0.12	-3.69
IPI00000824	Isoform A of NT-3 growth factor receptor precursor	R.WM*QLWQEQGEAK.L	2	4.26	0.40	-4.42
IPI00000828	Proenkephalin A precursor	A.EEDDSLANSSDLLK.E	2	4.90	0.39	-1.97
IPI00000828	Proenkephalin A precursor	D.AEEDDSLANSSDLLK.E	2	6.33	0.48	-4.00
IPI00000828	Proenkephalin A precursor	D.AEEDDSLANSSDLLKELLETGDNR.E	2	5.00	0.52	-2.99
IPI00000828	Proenkephalin A precursor	D.AEEDDSLANSSDLLKELLETGDNR.E	3	5.58	0.52	-4.56
IPI00000828	Proenkephalin A precursor	D.AEEDDSLANSSDLLKELLETGDNRER.S	3	5.56	0.49	-4.29
IPI00000828	Proenkephalin A precursor	E.DDSLANSSDLLK.E	2	3.65	0.31	-3.02
IPI00000828	Proenkephalin A precursor	E.EDDSLANSSDLLK.E	2	4.34	0.40	-4.10
IPI00000828	Proenkephalin A precursor	E.LYPM*EPEEEANGSEILAK.R	2	4.65	0.46	-4.75
IPI00000828	Proenkephalin A precursor	K.DAEEDDSLANSSDLLK.E	2	6.38	0.47	-4.28
IPI00000828	Proenkephalin A precursor	K.DAEEDDSLANSSDLLK.E	3	3.48	0.07	0.38
IPI00000828	Proenkephalin A precursor	K.DAEEDDSLANSSDLLKELLETGDNR.E	2	4.56	0.51	-4.41
IPI00000828	Proenkephalin A precursor	K.DAEEDDSLANSSDLLKELLETGDNR.E	3	5.50	0.52	-5.37
IPI00000828	Proenkephalin A precursor	K.DAEEDDSLANSSDLLKELLETGDNRE.R	3	4.03	0.46	-8.02
IPI00000828	Proenkephalin A precursor	K.DAEEDDSLANSSDLLKELLETGDNRER.S	2	2.50	0.23	-3.66
IPI00000828	Proenkephalin A precursor	K.DAEEDDSLANSSDLLKELLETGDNRER.S	3	7.29	0.52	-6.76
IPI00000828	Proenkephalin A precursor	K.DAEEDDSLANSSDLLKELLETGDNRER.S	4	3.57	0.36	-4.50
IPI00000828	Proenkephalin A precursor	K.ELLETGDNR.E	1	1.92	0.09	-3.74
IPI00000828	Proenkephalin A precursor	K.ELLQLSKPELPQDGTST.L	2	3.42	0.40	-2.86
IPI00000828	Proenkephalin A precursor	K.ELLQLSKPELPQDGTSTLR.E	2	4.29	0.46	-4.30
IPI00000828	Proenkephalin A precursor	K.ELLQLSKPELPQDGTSTLR.E	3	4.81	0.44	-3.78
IPI00000828	Proenkephalin A precursor	K.ELLQLSKPELPQDGTSTLRENSKPEESHLL.A	3	4.96	0.48	-3.73
IPI00000828	Proenkephalin A precursor	K.ELLQLSKPELPQDGTSTLRENSKPEESHLLA.K	3	4.89	0.43	-3.81
IPI00000828	Proenkephalin A precursor	K.ELLQLSKPELPQDGTSTLRENSKPEESHLLA.K	4	5.23	0.41	-2.89
IPI00000828	Proenkephalin A precursor	K.IWETCKELLQLSKPELPQDGTSTLR.E	3	5.01	0.49	-2.97
IPI00000828	Proenkephalin A precursor	K.IWETCKELLQLSKPELPQDGTSTLR.E	4	3.38	0.22	-3.91
IPI00000828	Proenkephalin A precursor	K.KDAEEDDSLANSSDLLK.E	2	5.64	0.45	-2.61
IPI00000828	Proenkephalin A precursor	K.KDAEEDDSLANSSDLLKELLETGDNR.E	3	5.78	0.54	-3.53
IPI00000828	Proenkephalin A precursor	K.KDAEEDDSLANSSDLLKELLETGDNR.E	4	3.02	0.14	-2.50
IPI00000828	Proenkephalin A precursor	K.KDAEEDDSLANSSDLLKELLETGDNRER.S	4	5.14	0.44	-4.13
IPI00000828	Proenkephalin A precursor	K.KDAEEDDSLANSSDLLKELLETGDNRER.S	5	3.70	0.24	-2.86

IPI00000828	Proenkephalin A precursor	K.KM*DELYPM*EPEEEANGSEILAK.R	3	3.73	0.34	-1.62
IPI00000828	Proenkephalin A precursor	R.ENSKPEESHLLA.K	2	3.02	0.30	-2.68
IPI00000828	Proenkephalin A precursor	R.FAEALPSDEEGESYSK.E	2	4.23	0.42	-4.07
IPI00000828	Proenkephalin A precursor	R.FAEALPSDEEGESYSKEVPEM*E.K	2	4.49	0.59	-3.18
IPI00000828	Proenkephalin A precursor	R.LVRPADINFLACVM*ECEGK.L	2	3.09	0.39	-5.58
IPI00000828	Proenkephalin A precursor	R.LVRPADINFLACVM*ECEGK.L	3	4.55	0.40	-3.97
IPI00000828	Proenkephalin A precursor	R.LVRPADINFLACVM*ECEGKLPSLK.I	3	5.82	0.55	-5.55
IPI00000828	Proenkephalin A precursor	R.LVRPADINFLACVM*ECEGKLPSLK.I	4	5.27	0.45	-4.46
IPI00000832	Beta-neoendorphin-dynorphin precursor	K.SVGEGPYSELAK.L	2	2.60	0.27	-2.42
IPI00000832	Beta-neoendorphin-dynorphin precursor	K.TQDGPKPINPLICSLQCQAALLPSEEWER.C	3	3.97	0.30	-3.45
IPI00000832	Beta-neoendorphin-dynorphin precursor	R.GLSDGFREGAESELM*R.D	2	1.81	0.11	-3.38
IPI00000871	Prolactin	R.DSHKIDNYLK.L	2	2.49	0.17	-2.60
IPI00000871	Prolactin	R.DSHKIDNYLK.L	3	2.27	0.17	-3.82
IPI00000871	Prolactin	R.LLEGM*ELIVSQVHPETK.E	3	2.99	0.24	-2.67
IPI00000874	Peroxiredoxin-1	K.ATAVM*PDGQFK.D	2	2.20	0.23	-2.76
IPI00000874	Peroxiredoxin-1	K.ATAVM*PDGQFKDISLSDYK.G	2	4.93	0.51	-1.66
IPI00000874	Peroxiredoxin-1	K.ATAVM*PDGQFKDISLSDYK.G	3	2.78	0.43	-2.28
IPI00000874	Peroxiredoxin-1	K.IGHPAPNFK.A	1	2.29	0.22	-4.10
IPI00000874	Peroxiredoxin-1	K.IGHPAPNFK.A	2	1.77	0.10	-2.72
IPI00000874	Peroxiredoxin-1	R.GLFIIDDKGILR.Q	2	3.71	0.33	-2.33
IPI00000874	Peroxiredoxin-1	R.LVQAFQFTDK.H	2	3.24	0.32	-0.52
IPI00000874	Peroxiredoxin-1	R.QITVNDLPVGR.S	1	1.08	0.06	-3.33
IPI00000874	Peroxiredoxin-1	R.QITVNDLPVGR.S	2	2.78	0.29	-3.36
IPI00000874	Peroxiredoxin-1	R.TIAQDYGVLK.A	2	1.97	0.06	-7.57
IPI00000874	Peroxiredoxin-1	R.TIAQDYGVLKADEGISFR.G	3	2.49	0.22	-1.44
IPI00000874	Peroxiredoxin-1	W.KPGSDTIKPDVQK.S	2	3.57	0.32	-2.99
IPI00000877	Hypoxia up-regulated protein 1 precursor	K.AANSLEAFIFETQDK.L	2	4.59	0.46	-2.99
IPI00000877	Hypoxia up-regulated protein 1 precursor	K.AANSLEAFIFETQDK.L	3	2.90	0.11	-3.03
IPI00000877	Hypoxia up-regulated protein 1 precursor	K.AANSLEAFIFETQDKLYQPEYQEVSTEEQREEISGK.L	3	5.32	0.54	-0.11
IPI00000877	Hypoxia up-regulated protein 1 precursor	K.AANSLEAFIFETQDKLYQPEYQEVSTEEQREEISGK.L	4	3.99	0.31	-2.95
IPI00000877	Hypoxia up-regulated protein 1 precursor	K.AEAGPEGVAPAPEGEKK.Q	2	3.98	0.47	-2.89
IPI00000877	Hypoxia up-regulated protein 1 precursor	K.LCQGLFFR.V	2	2.97	0.21	-1.20
IPI00000877	Hypoxia up-regulated protein 1 precursor	K.LPATEKPVLLSK.D	2	3.62	0.51	-3.61
IPI00000877	Hypoxia up-regulated protein 1 precursor	K.LPATEKPVLLSK.D	3	2.09	0.17	-3.90
IPI00000877	Hypoxia up-regulated protein 1 precursor	K.LQDLTLR.D	2	2.33	0.09	-3.01
IPI00000877	Hypoxia up-regulated protein 1 precursor	K.LQDLTLRDLEK.Q	2	3.13	0.17	-1.24
IPI00000877	Hypoxia up-regulated protein 1 precursor	K.LQDLTLRDLEKQER.E	3	2.92	0.06	-2.69
IPI00000877	Hypoxia up-regulated protein 1 precursor	K.LQDLTLRDLEKQEREK.A	3	3.81	0.18	-3.14
IPI00000877	Hypoxia up-regulated protein 1 precursor	K.M*M*ALDREVQYLLNK.A	2	2.59	0.11	-3.06
IPI00000877	Hypoxia up-regulated protein 1 precursor	K.M*M*ALDREVQYLLNK.A	3	3.09	0.16	-4.99
IPI00000877	Hypoxia up-regulated protein 1 precursor	R.LIPEM*DQIFTEVEM*TTLEK.V	3	4.74	0.36	-5.01
IPI00000914	Isoform 1 of Calcitonin precursor	R.LLLAALVQDYVQM*K.A	2	4.72	0.45	-3.61

IPI00000914	Isoform 1 of Calcitonin precursor	R.LLLAALVQDYVQM*K.A	3	5.28	0.48	-4.39
IPI00000914	Isoform 1 of Calcitonin precursor	R.SALESSPADPATLSEDEAR.L	2	5.05	0.57	-4.14
IPI00000959	Isoform 1 of VIP peptides precursor	R.LGDRIPFEGANEPDQVSLKEDIDM*LQNALAENDTPYYDVSR.N	4	3.90	0.26	-3.49
IPI00000977	Mitogen-activated protein kinase kinase kinase 11	R.EWHKTTQM*SAAGTYAWMAPEVIK.A	3	3.33	0.06	
IPI00001120	CDNA FLJ31810 fis, clone NT2RI2009289, weakly similar to CARBOXYPEPTIDASE N 83 KD CHAIN	K.SLEVGDNDLVYISHR.A	2	4.25	0.45	-2.84
IPI00001120	CDNA FLJ31810 fis, clone NT2RI2009289, weakly similar to CARBOXYPEPTIDASE N 83 KD CHAIN	K.SLEVGDNDLVYISHR.A	3	2.69	0.07	-2.25
IPI00001120	CDNA FLJ31810 fis, clone NT2RI2009289, weakly similar to CARBOXYPEPTIDASE N 83 KD CHAIN	R.LQELHIVGAQLR.T	3	3.58	0.26	-2.67
IPI00001399	Adherens junction-associated protein 1	K.AGLAKPPAAAK.S	1	2.26	0.33	-2.07
IPI00001399	Adherens junction-associated protein 1	K.AGLAKPPAAAK.S	2	3.03	0.32	-1.50
IPI00001399	Adherens junction-associated protein 1	K.SSPSLASSSSSSSSAVAGGAPEQQALLR.R	2	5.28	0.56	-2.57
IPI00001399	Adherens junction-associated protein 1	K.SSPSLASSSSSSSSAVAGGAPEQQALLR.R	3	6.02	0.41	-3.20
IPI00001399	Adherens junction-associated protein 1	K.SSPSLASSSSSSSSAVAGGAPEQQALLRR.G	3	4.25	0.43	-1.54
IPI00001399	Adherens junction-associated protein 1	R.DQAAALVPK.A	1	1.81	0.09	-3.59
IPI00001399	Adherens junction-associated protein 1	R.DQAAALVPK.A	2	2.85	0.15	-2.65
IPI00001433	Protocadherin beta 15 precursor	R.GSFVANLANDLGLGVGELAER.G	2	3.40	0.43	-2.53
IPI00001434	Protocadherin beta 14 precursor	R.DLGLGVEELSSR.E	2	3.89	0.41	-3.86
IPI00001477	Isoform 1 of Epithelial discoidin domain-containing receptor 1 precursor	K.DLGPPM*VAR.L	2	1.84	0.11	-1.96
IPI00001477	Isoform 1 of Epithelial discoidin domain-containing receptor 1 precursor	K.DRWGQEVISGNEDPEGVVLK.D	3	4.58	0.32	-2.93
IPI00001477	Isoform 1 of Epithelial discoidin domain-containing receptor 1 precursor	K.EEEYLQVDLQR.L	2	3.90	0.33	-1.17
IPI00001477	Isoform 1 of Epithelial discoidin domain-containing receptor 1 precursor	K.GHFDPAK.C	1	1.61	0.09	-1.96
IPI00001477	Isoform 1 of Epithelial discoidin domain-containing receptor 1 precursor	R.AVSVPLGGR.V	1	1.58	0.23	-2.63
IPI00001477	Isoform 1 of Epithelial discoidin domain-containing receptor 1 precursor	R.AVSVPLGGR.V	2	2.36	0.18	-3.22
IPI00001477	Isoform 1 of Epithelial discoidin domain-containing receptor 1 precursor	R.LESSDGDGAWCPAGSVFPKEEEYLQVDLQR.L	3	5.60	0.52	-5.34
IPI00001477	Isoform 1 of Epithelial discoidin domain-containing receptor 1 precursor	R.VELYGCLWR.D	2	3.29	0.32	-2.06
IPI00001477	Isoform 1 of Epithelial discoidin domain-containing receptor 1 precursor	R.WGQEVISGNEDPEGVVLKDLGPPM*VAR.L	3	5.54	0.39	-2.99
IPI00001477	Isoform 1 of Epithelial discoidin domain-containing receptor 1 precursor	R.YALGM*QDR.T	2	2.34	0.25	-0.97
IPI00001506	Neuropeptide Y precursor	P.SKPDNPGEDAPAEDM*AR.Y	3	3.60	0.29	-2.42
IPI00001506	Neuropeptide Y precursor	R.ESTENVPR.T	2	1.81	0.07	-2.27
IPI00001506	Neuropeptide Y precursor	R.SSPETLISDLLM*R.E	2	4.07	0.39	-4.31

IPI00001506	Neuropeptide Y precursor	R.SSPETLISDLLM*R.E	3	3.93	0.32	-2.24
IPI00001506	Neuropeptide Y precursor	S.PETLISDLLM*R.E	2	3.36	0.35	-2.43
IPI00001568	Vacuolar proton pump subunit D	K.AVELLVELASLQTSFVTLDEAIK.I	2	4.45	0.50	-3.46
IPI00001568	Vacuolar proton pump subunit D	K.AVELLVELASLQTSFVTLDEAIK.I	3	3.41	0.48	-1.99
IPI00001592	Isoform 2 of Transmembrane glycoprotein NMB precursor	K.DVYVVTDQIPVFVTM*FQK.N	2	4.09	0.49	-4.64
IPI00001592	Isoform 2 of Transmembrane glycoprotein NMB precursor	K.DVYVVTDQIPVFVTM*FQK.N	3	4.30	0.47	-4.03
IPI00001592	Isoform 2 of Transmembrane glycoprotein NMB precursor	R.AYVPIAQVK.D	2	2.08	0.18	-1.39
IPI00001593	Lysosomal Pro-X carboxypeptidase precursor	K.AM*LVFAEHR.Y	2	2.73	0.26	-3.39
IPI00001593	Lysosomal Pro-X carboxypeptidase precursor	K.DITDTLVAVTISEGAHHLDLR.T	3	3.43	0.36	-5.47
IPI00001593	Lysosomal Pro-X carboxypeptidase precursor	K.DITDTLVAVTISEGAHHLDLR.T	4	2.48	0.16	-3.06
IPI00001593	Lysosomal Pro-X carboxypeptidase precursor	K.IVTTDFRK.S	2	1.61	0.15	-3.13
IPI00001593	Lysosomal Pro-X carboxypeptidase precursor	K.NALDPM*SVLLAR.S	2	3.00	0.29	-4.28
IPI00001593	Lysosomal Pro-X carboxypeptidase precursor	K.VDHFGFNTVK.T	1	3.09	0.37	-4.27
IPI00001593	Lysosomal Pro-X carboxypeptidase precursor	K.VDHFGFNTVK.T	2	2.63	0.34	-2.83
IPI00001593	Lysosomal Pro-X carboxypeptidase precursor	K.VDHFGFNTVKTFNQR.Y	2	4.57	0.59	-4.82
IPI00001593	Lysosomal Pro-X carboxypeptidase precursor	R.ALGSLHLPTNPTSLPAVAK.N	2	2.82	0.31	-3.87
IPI00001593	Lysosomal Pro-X carboxypeptidase precursor	R.ALGSLHLPTNPTSLPAVAK.N	3	2.93	0.31	-3.35
IPI00001593	Lysosomal Pro-X carboxypeptidase precursor	R.DFYDSAGKQH.-	2	2.49	0.34	-2.29
IPI00001593	Lysosomal Pro-X carboxypeptidase precursor	R.HLNFLTSEQALADFAELIK.H	2	6.98	0.61	-4.90
IPI00001593	Lysosomal Pro-X carboxypeptidase precursor	R.HLNFLTSEQALADFAELIK.H	3	3.18	0.20	-3.86
IPI00001593	Lysosomal Pro-X carboxypeptidase precursor	R.PSWITTM*YGGK.N	2	3.46	0.31	-2.33
IPI00001593	Lysosomal Pro-X carboxypeptidase precursor	R.SWDAINR.L	2	2.73	0.08	-2.38
IPI00001593	Lysosomal Pro-X carboxypeptidase precursor	R.TKNALDPM*SVLLAR.S	2	4.46	0.43	-2.47
IPI00001593	Lysosomal Pro-X carboxypeptidase precursor	R.TKNALDPM*SVLLAR.S	3	4.94	0.28	-2.36
IPI00001593	Lysosomal Pro-X carboxypeptidase precursor	R.YYGESLPFGDNSFKDSR.H	2	4.74	0.51	-1.76
IPI00001593	Lysosomal Pro-X carboxypeptidase precursor	R.YYGESLPFGDNSFKDSR.H	3	3.29	0.36	-2.89
IPI00001610	Insulin-like growth factor IA precursor	A.GPETLCGAELVDALQFVCGDR.G	3	5.12	0.41	-4.59
IPI00001610	Insulin-like growth factor IA precursor	R.GFYFNKPTGYGSSSR.R	2	3.90	0.41	-1.66
IPI00001611	Isoform 1 of Insulin-like growth factor II precursor	A.AYRPSETLCGGELVDTLQFVCGDR.G	3	4.18	0.36	-3.53
IPI00001611	Isoform 1 of Insulin-like growth factor II precursor	A.YRPSETLCGGELVDTLQFVCGDR.G	3	5.05	0.48	-2.46
IPI00001611	Isoform 1 of Insulin-like growth factor II precursor	C.CIAAYRPSETLCGGELVDTLQFVCGDR.G	3	4.21	0.45	-3.76
IPI00001611	Isoform 1 of Insulin-like growth factor II precursor	K.ELEAFREAK.R	2	2.36	0.20	-1.65
IPI00001611	Isoform 1 of Insulin-like growth factor II precursor	K.FFQYDTWK.Q	1	2.22	0.26	-3.71
IPI00001611	Isoform 1 of Insulin-like growth factor II precursor	K.FFQYDTWK.Q	2	2.84	0.24	-2.27
IPI00001611	Isoform 1 of Insulin-like growth factor II precursor	R.GFYFSRPASR.V	2	2.50	0.28	-2.17
IPI00001611	Isoform 1 of Insulin-like growth factor II precursor	R.GIVEECCFR.S	1	2.21	0.24	-3.35
IPI00001611	Isoform 1 of Insulin-like growth factor II precursor	R.GIVEECCFR.S	2	3.29	0.35	-2.94
IPI00001611	Isoform 1 of Insulin-like growth factor II precursor	R.SCDLALLETYCATPAK.S	2	4.76	0.50	-3.80
IPI00001611	Isoform 1 of Insulin-like growth factor II precursor	R.SCDLALLETYCATPAK.S	3	4.43	0.37	-1.63

IPI00001611	Isoform 1 of Insulin-like growth factor II precursor	R.SCDLALLETYCATPAKSE.R	2	5.32	0.50	-3.69
IPI00001611	Isoform 1 of Insulin-like growth factor II precursor	R.SCDLALLETYCATPAKSE.R	3	5.26	0.47	-2.88
IPI00001633	Leucine-rich repeat transmembrane protein FLRT2 precursor	R.AALAQLLK.L	2	2.47	0.15	-2.92
IPI00001633	Leucine-rich repeat transmembrane protein FLRT2 precursor	R.LYLQDNQINHIPLTAFSNLR.K	3	4.05	0.29	-2.13
IPI00001633	Leucine-rich repeat transmembrane protein FLRT2 precursor	R.VLHLQENNIQTISR.A	2	2.60	0.11	0.69
IPI00001662	Opioid-binding protein/cell adhesion molecule precursor	C.SALNDVAAPDVR.K	2	3.56	0.29	-1.02
IPI00001662	Opioid-binding protein/cell adhesion molecule precursor	E.GQGFVSEDEYLEISDIKR.D	2	4.68	0.45	-1.65
IPI00001662	Opioid-binding protein/cell adhesion molecule precursor	K.AM*DNVTVR.Q	2	3.03	0.16	-2.98
IPI00001662	Opioid-binding protein/cell adhesion molecule precursor	K.EGQGFVSEDEYLEISDIKR.D	2	4.48	0.30	-3.76
IPI00001662	Opioid-binding protein/cell adhesion molecule precursor	K.EGQGFVSEDEYLEISDIKR.D	3	2.15	0.18	-2.54
IPI00001662	Opioid-binding protein/cell adhesion molecule precursor	K.GILSCEASAVPM*AEFQWFK.E	2	3.74	0.44	-4.73
IPI00001662	Opioid-binding protein/cell adhesion molecule precursor	K.GILSCEASAVPM*AEFQWFK.E	3	3.30	0.31	-3.44
IPI00001662	Opioid-binding protein/cell adhesion molecule precursor	K.GILSCEASAVPM*AEFQWFKEETR.L	3	4.61	0.50	-2.93
IPI00001662	Opioid-binding protein/cell adhesion molecule precursor	K.ITVNYPPYISK.A	1	2.52	0.26	-2.98
IPI00001662	Opioid-binding protein/cell adhesion molecule precursor	K.ITVNYPPYISK.A	2	3.77	0.29	-3.61
IPI00001662	Opioid-binding protein/cell adhesion molecule precursor	K.NTGVSVGQK.G	1	2.25	0.27	-2.72
IPI00001662	Opioid-binding protein/cell adhesion molecule precursor	K.NTGVSVGQK.G	2	2.55	0.30	-2.41
IPI00001662	Opioid-binding protein/cell adhesion molecule precursor	K.VKITVNYPPYISK.A	2	4.38	0.39	-4.85
IPI00001662	Opioid-binding protein/cell adhesion molecule precursor	K.VKITVNYPPYISK.A	3	1.79	0.16	-2.98
IPI00001662	Opioid-binding protein/cell adhesion molecule precursor	P.VRSGDATFPK.A	1	2.19	0.22	-1.28
IPI00001662	Opioid-binding protein/cell adhesion molecule precursor	R.DQSGEYECSALNDVAAPDVR.K	2	5.21	0.36	
IPI00001662	Opioid-binding protein/cell adhesion molecule precursor	R.DQSGEYECSALNDVAAPDVRK.V	2	4.93	0.50	-2.45

IPI00001662	Opioid-binding protein/cell adhesion molecule precursor	R.DQSGEYECSALNDVAAPDVRK.V	3	3.82	0.37	-3.70
IPI00001662	Opioid-binding protein/cell adhesion molecule precursor	R.LATGLDGM*R.I	2	2.70	0.21	-3.56
IPI00001662	Opioid-binding protein/cell adhesion molecule precursor	R.LATGLDGM*RIENK.G	2	3.03	0.15	-2.65
IPI00001662	Opioid-binding protein/cell adhesion molecule precursor	R.QGESATLR.C	2	1.66	0.15	-3.34
IPI00001662	Opioid-binding protein/cell adhesion molecule precursor	R.SGDATFPK.A	1	2.05	0.16	-3.53
IPI00001662	Opioid-binding protein/cell adhesion molecule precursor	R.SGDATFPK.A	2	2.92	0.23	-4.46
IPI00001662	Opioid-binding protein/cell adhesion molecule precursor	R.STILYAGNDK.W	1	2.48	0.29	-2.46
IPI00001662	Opioid-binding protein/cell adhesion molecule precursor	R.STILYAGNDK.W	2	2.95	0.20	-3.32
IPI00001662	Opioid-binding protein/cell adhesion molecule precursor	R.STILYAGNDKWSIDPR.V	2	3.22	0.34	-1.49
IPI00001662	Opioid-binding protein/cell adhesion molecule precursor	R.STILYAGNDKWSIDPR.V	3	3.41	0.28	-2.55
IPI00001662	Opioid-binding protein/cell adhesion molecule precursor	R.VIILVNTPTQYSIM*IQNVDVYDEGPYTCSVQTDNHPK.T	3	5.71	0.50	-2.87
IPI00001662	Opioid-binding protein/cell adhesion molecule precursor	R.VIILVNTPTQYSIM*IQNVDVYDEGPYTCSVQTDNHPK.T	4	4.75	0.35	-5.38
IPI00001712	Isoform 1 of Catenin alpha-3	R.DQDADNLDRAAGAIRGRAAR.V	2	2.88	0.08	-1.10
IPI00001734	Isoform 1 of Phosphoserine aminotransferase	K.FGTINIVHPK.L	2	2.28	0.25	-3.33
IPI00001734	Isoform 1 of Phosphoserine aminotransferase	K.FGVIFAGAQK.N	2	2.66	0.22	-1.16
IPI00001734	Isoform 1 of Phosphoserine aminotransferase	K.GAVLVCDM*SSNFLSKPVDVSK.F	3	3.83	0.42	-1.18
IPI00001734	Isoform 1 of Phosphoserine aminotransferase	K.GVGISVLEM*SHR.S	2	2.33	0.27	-3.74
IPI00001734	Isoform 1 of Phosphoserine aminotransferase	K.IINNTENLVR.E	2	3.10	0.26	-1.96
IPI00001734	Isoform 1 of Phosphoserine aminotransferase	R.ASLYNAVTIEDVQK.L	2	4.42	0.23	-3.09
IPI00001734	Isoform 1 of Phosphoserine aminotransferase	R.IGNAKGDDALEKR.F	2	3.64	0.26	-1.15
IPI00001755	Glypican-6 precursor	K.GFSLADIPYQEIAGEHLR.I	2	3.68	0.35	-1.23
IPI00001755	Glypican-6 precursor	K.GFSLADIPYQEIAGEHLR.I	3	3.17	0.32	-1.99
IPI00001786	Isoform 2 of Ubiquitin carboxyl-terminal hydrolase 36	K.GNDESAGLDRRGSSSSSPEHSASSDSTK.A	3	2.91	0.17	
IPI00001793	Beta-1,3-N-acetylglucosaminyltransferase radical fringe	R.LPDDCTVGYIVEGLLGAR.L	3	2.69	0.25	-4.54
IPI00001796	Tumor necrosis factor receptor superfamily, member 18 (Fragment)	R.LLLGTGTDAR.C	2	2.17	0.14	-0.32
IPI00001863	Wnt inhibitory factor 1 precursor	R.YEASLIHALRPAGAQLR.Q	3	2.18	0.28	-2.33
IPI00001869	Pappalysin-1 precursor	R.RDDELIK.S	2	2.13	0.07	-7.73

IPI00001872	Isoform 1 of Protocadherin gamma C3 precursor	R.AEFPSGSAPR.E	2	2.12	0.15	-1.17
IPI00001872	Isoform 1 of Protocadherin gamma C3 precursor	R.GTSAGHLVSR.V	2	2.93	0.29	-2.88
IPI00001893	Isoform A of Protocadherin-7 precursor	R.IDREEVNQLR.F	3	3.45	0.05	-2.82
IPI00001893	Isoform A of Protocadherin-7 precursor	R.LDASEGGGGTNPGGR.S	2	4.50	0.46	-3.16
IPI00001893	Isoform A of Protocadherin-7 precursor	R.RLDASEGGGGTNPGGR.S	2	4.55	0.44	-2.54
IPI00001893	Isoform A of Protocadherin-7 precursor	R.RLDASEGGGGTNPGGR.S	3	3.98	0.11	
IPI00001893	Isoform A of Protocadherin-7 precursor	R.SSVFELQVADTPDGEKQPQLIVK.G	3	3.31	0.42	-0.24
IPI00001895	Isoform 1 of Protocadherin-8 precursor	K.VSGDTSFR.L	2	2.05	0.15	-2.20
IPI00001895	Isoform 1 of Protocadherin-8 precursor	R.AQIPVEVSEGAAVGTR.I	2	5.19	0.50	-3.77
IPI00001895	Isoform 1 of Protocadherin-8 precursor	R.DVNDHAPR.F	2	2.08	0.06	-0.87
IPI00001895	Isoform 1 of Protocadherin-8 precursor	R.GPAAPASAGSPER.S	2	3.21	0.41	-1.70
IPI00001895	Isoform 1 of Protocadherin-8 precursor	R.IPLEVPVDEDVGANGLQTVR.L	2	5.72	0.60	-4.19
IPI00001895	Isoform 1 of Protocadherin-8 precursor	R.IPLEVPVDEDVGANGLQTVR.L	3	5.49	0.41	-3.98
IPI00001895	Isoform 1 of Protocadherin-8 precursor	R.VREGDGQLTVGDAGLDR.E	3	2.55	0.12	-1.49
IPI00001895	Isoform 1 of Protocadherin-8 precursor	R.VREGDGQLTVGDAGLDRER.L	3	3.62	0.32	-4.02
IPI00001895	Isoform 1 of Protocadherin-8 precursor	R.YSTFEEDAPGTVIGTLAEDLHM*K.V	2	4.50	0.56	-2.28
IPI00001895	Isoform 1 of Protocadherin-8 precursor	R.YSTFEEDAPGTVIGTLAEDLHM*K.V	3	3.92	0.40	-2.84
IPI00001952	Endonuclease domain-containing 1 protein precursor	D.PNSNLEEAINEAEAITSVNSLGSK.Q	3	4.42	0.46	-2.18
IPI00001952	Endonuclease domain-containing 1 protein precursor	K.DKVAVPEFVWLAACCAVPGGGWAM*GFVK.H	3	3.36	0.21	-3.93
IPI00001952	Endonuclease domain-containing 1 protein precursor	K.ILEVVNQIQDEER.M	2	4.71	0.42	-3.79
IPI00001952	Endonuclease domain-containing 1 protein precursor	K.ILEVVNQIQDEER.M	3	3.68	0.30	-2.89
IPI00001952	Endonuclease domain-containing 1 protein precursor	K.ILEVVNQIQDEERM*VQSQK.S	3	3.34	0.23	-1.17
IPI00001952	Endonuclease domain-containing 1 protein precursor	K.KILEVVNQIQDEER.M	2	4.96	0.41	-2.66
IPI00001952	Endonuclease domain-containing 1 protein precursor	K.KILEVVNQIQDEER.M	3	4.19	0.15	-2.80
IPI00001952	Endonuclease domain-containing 1 protein precursor	K.LLPFNPQLFQNNCGETEQDTEK.M	2	4.78	0.47	-4.35
IPI00001952	Endonuclease domain-containing 1 protein precursor	K.LLPFNPQLFQNNCGETEQDTEK.M	3	3.12	0.27	-5.24
IPI00001952	Endonuclease domain-containing 1 protein precursor	K.QALNTDYLDSDYQR.G	2	4.24	0.55	-2.15
IPI00001952	Endonuclease domain-containing 1 protein precursor	K.VAVPEFVWLAACCAVPGGGWAM*GFVK.H	3	3.57	0.15	-3.30
IPI00001952	Endonuclease domain-containing 1 protein precursor	R.ALTPQCGSGEDLYILTGTVPSDYR.V	2	5.15	0.46	-4.29

IPI00001952	Endonuclease domain-containing 1 protein precursor	R.APRPAPGGAEQR.W	2	2.75	0.19	-2.01
IPI00001952	Endonuclease domain-containing 1 protein precursor	R.APRPAPGGAEQR.W	3	4.31	0.40	-4.49
IPI00001952	Endonuclease domain-containing 1 protein precursor	R.DRIPVYSAFR.A	2	2.88	0.15	-2.77
IPI00001952	Endonuclease domain-containing 1 protein precursor	R.DRIPVYSAFR.A	3	2.36	0.12	-2.97
IPI00001952	Endonuclease domain-containing 1 protein precursor	R.DSDIIEDVM*VK.D	1	3.42	0.31	1.09
IPI00001952	Endonuclease domain-containing 1 protein precursor	R.DSDIIEDVM*VK.D	2	4.05	0.35	-2.72
IPI00001952	Endonuclease domain-containing 1 protein precursor	R.FATLYSTR.D	1	2.11	0.12	-2.92
IPI00001952	Endonuclease domain-containing 1 protein precursor	R.FATLYSTR.D	2	2.99	0.18	-2.69
IPI00001952	Endonuclease domain-containing 1 protein precursor	R.GQLYPFSLSSDVQVATFTLTNSAPM*TQSFQER.W	3	6.65	0.60	-6.01
IPI00001952	Endonuclease domain-containing 1 protein precursor	R.GQLYPFSLSSDVQVATFTLTNSAPM*TQSFQER.W	4	4.66	0.43	-0.95
IPI00001952	Endonuclease domain-containing 1 protein precursor	R.WLVEPQIDDPNSNLEEAINEAEAITSVNSLGSK.Q	3	4.92	0.52	-0.67
IPI00001952	Endonuclease domain-containing 1 protein precursor	R.WYVNLHSLM*DR.A	2	3.01	0.41	-2.95
IPI00001960	Chloride intracellular channel protein 4	K.NSRPEANEALER.G	2	2.63	0.25	-3.38
IPI00002142	Protocadherin-10 precursor	R.FAELVLEKPLDR.E	3	3.47	0.27	-0.13
IPI00002142	Protocadherin-10 precursor	R.FAELVLEKPLDREQQA.V	3	3.63	0.31	-1.93
IPI00002142	Protocadherin-10 precursor	R.FAELVLEKPLDREQQAVHR.Y	3	3.67	0.39	-2.42
IPI00002142	Protocadherin-10 precursor	R.FAELVLEKPLDREQQAVHR.Y	5	2.23	0.13	-2.87
IPI00002142	Protocadherin-10 precursor	R.LTYSIVR.G	2	2.16	0.08	-2.48
IPI00002142	Protocadherin-10 precursor	R.SFDYEQLKDFSFQVEAR.D	3	4.71	0.37	-3.44
IPI00002142	Protocadherin-10 precursor	R.VAAVDADDGENAR.L	2	4.38	0.45	-3.83
IPI00002147	Chitinase-3-like protein 1 precursor	K.AEFIKEAQPGKK.Q	2	3.17	0.24	-3.53
IPI00002147	Chitinase-3-like protein 1 precursor	K.EAGTLAYYEICDFLR.G	2	4.83	0.51	-4.57
IPI00002147	Chitinase-3-like protein 1 precursor	K.EAGTLAYYEICDFLR.G	3	4.99	0.35	-1.98
IPI00002147	Chitinase-3-like protein 1 precursor	K.GNQWVGYDDQESVK.S	2	3.20	0.39	-2.67
IPI00002147	Chitinase-3-like protein 1 precursor	K.LVCYYTSWSQYR.E	2	4.00	0.36	-2.12
IPI00002147	Chitinase-3-like protein 1 precursor	K.LVM*GIPTFGR.S	2	2.73	0.26	-2.38
IPI00002147	Chitinase-3-like protein 1 precursor	K.QLLLSAALSAGK.V	1	2.39	0.16	-3.18
IPI00002147	Chitinase-3-like protein 1 precursor	K.QLLLSAALSAGK.V	2	3.36	0.26	-4.40
IPI00002147	Chitinase-3-like protein 1 precursor	K.TLLSVGGWNFGSQR.F	2	4.45	0.51	-3.22
IPI00002147	Chitinase-3-like protein 1 precursor	K.TLLSVGGWNFGSQR.F	3	2.98	0.34	-1.66

IPI00002147	Chitinase-3-like protein 1 precursor	K.VQYLKDR.Q	2	2.37	0.11	-3.45
IPI00002147	Chitinase-3-like protein 1 precursor	K.VTIDSSYDIAK.I	2	3.47	0.33	-3.34
IPI00002147	Chitinase-3-like protein 1 precursor	R.EGDGSCFPDALDR.F	2	3.93	0.46	-4.42
IPI00002147	Chitinase-3-like protein 1 precursor	R.FPLTNAIK.D	2	2.38	0.09	-2.38
IPI00002147	Chitinase-3-like protein 1 precursor	R.FPLTNAIKDALAAT.-	2	3.54	0.43	-4.44
IPI00002147	Chitinase-3-like protein 1 precursor	R.FSKIASNTQSR.R	2	2.41	0.14	-2.76
IPI00002147	Chitinase-3-like protein 1 precursor	R.FSNTDYAVGYM*LR.L	2	4.75	0.53	-3.31
IPI00002147	Chitinase-3-like protein 1 precursor	R.GQEDASPDRFSNTDYAVGYM*LR.L	2	2.44	0.35	-3.51
IPI00002147	Chitinase-3-like protein 1 precursor	R.GQEDASPDRFSNTDYAVGYM*LR.L	3	5.85	0.53	-4.91
IPI00002147	Chitinase-3-like protein 1 precursor	R.ILGQQVPYATK.G	1	2.53	0.30	-3.67
IPI00002147	Chitinase-3-like protein 1 precursor	R.ILGQQVPYATK.G	2	3.66	0.39	-2.83
IPI00002147	Chitinase-3-like protein 1 precursor	R.LGAPASKLVM*GIPTFGR.S	3	2.50	0.19	-2.76
IPI00002147	Chitinase-3-like protein 1 precursor	R.QLAGAM*VWALDLDDFQGSFCGQDLR.F	3	5.14	0.42	-4.49
IPI00002147	Chitinase-3-like protein 1 precursor	R.SFTLASSETGVGAPISGPGIPGR.F	2	5.68	0.55	-3.78
IPI00002147	Chitinase-3-like protein 1 precursor	R.SFTLASSETGVGAPISGPGIPGR.F	3	4.68	0.49	-2.81
IPI00002147	Chitinase-3-like protein 1 precursor	R.SFTLASSETGVGAPISGPGIPGRFTK.E	2	3.32	0.41	-2.66
IPI00002147	Chitinase-3-like protein 1 precursor	R.SFTLASSETGVGAPISGPGIPGRFTK.E	3	5.88	0.61	-2.67
IPI00002147	Chitinase-3-like protein 1 precursor	R.TFIKSVPPFLR.T	3	2.36	0.25	-3.08
IPI00002147	Chitinase-3-like protein 1 precursor	R.THGFDGLDLAWLYPGR.R	2	4.45	0.51	-2.72
IPI00002147	Chitinase-3-like protein 1 precursor	R.THGFDGLDLAWLYPGR.R	3	2.46	0.15	-2.60
IPI00002147	Chitinase-3-like protein 1 precursor	W.VGYDDQESVK.S	2	3.20	0.42	-2.56
IPI00002191	Putative uncharacterized protein FLJ12684	R.ALFPSLGTYDLEK.A	2	2.73	0.30	-2.89
IPI00002211	Isoform 2 of Semaphorin-6A precursor	K.HANVALFADGK.L	1	2.89	0.41	-0.54
IPI00002211	Isoform 2 of Semaphorin-6A precursor	K.HANVALFADGK.L	2	3.51	0.44	-0.04
IPI00002211	Isoform 2 of Semaphorin-6A precursor	K.HLLDSPDSTDPLGAVSSH.N	2	5.16	0.62	-3.60
IPI00002211	Isoform 2 of Semaphorin-6A precursor	K.M*DTLEPFGDEFSGM*AR.C	2	4.67	0.43	-4.54
IPI00002211	Isoform 2 of Semaphorin-6A precursor	K.SPDSTWTPVPDER.V	2	2.91	0.37	-2.64
IPI00002211	Isoform 2 of Semaphorin-6A precursor	R.DPYCGWIK.E	2	2.75	0.14	-1.77
IPI00002211	Isoform 2 of Semaphorin-6A precursor	R.EIAVEYNTM*GK.V	2	3.34	0.41	-4.24
IPI00002211	Isoform 2 of Semaphorin-6A precursor	R.IM*GM*QLDR.A	2	2.71	0.23	-1.83
IPI00002211	Isoform 2 of Semaphorin-6A precursor	R.LTFEQDIER.G	2	2.67	0.22	-3.91
IPI00002211	Isoform 2 of Semaphorin-6A precursor	R.SLGESPTLR.T	2	2.17	0.15	-2.43
IPI00002211	Isoform 2 of Semaphorin-6A precursor	R.YATSNEFPDDTLNFIK.T	2	4.88	0.46	-7.93
IPI00002236	Lactadherin precursor	K.EVTGIITQGAR.N	2	3.99	0.27	-3.46
IPI00002236	Lactadherin precursor	K.NAVHVNLFETPVEAQYVR.L	3	3.79	0.38	-4.10
IPI00002236	Lactadherin precursor	K.NLFETPILAR.Y	2	2.49	0.12	-2.45
IPI00002236	Lactadherin precursor	K.VAYSLNGHEFDFIHDVNKK.H	3	3.29	0.28	-0.93
IPI00002236	Lactadherin precursor	R.GDVFPSYTCTCLK.G	2	3.35	0.40	-1.42
IPI00002236	Lactadherin precursor	R.LASHEYLK.A	2	2.35	0.12	0.19
IPI00002236	Lactadherin precursor	R.VTFLGLQHWVPELAR.L	3	3.30	0.12	-2.24
IPI00002243	Isoform 1 of Gamma-glutamyltransferase 5 precursor	R.DLLGETLAQLIR.Q	2	3.73	0.30	-4.21

IPI00002280	ProSAAS precursor	A.ADHDVGSELPPEGVLGALLR.V	2	6.02	0.53	-3.61
IPI00002280	ProSAAS precursor	A.ADHDVGSELPPEGVLGALLR.V	3	3.99	0.38	-4.69
IPI00002280	ProSAAS precursor	A.DHDVGSELPPEGVLGALLR.V	2	6.19	0.59	-4.16
IPI00002280	ProSAAS precursor	A.DHDVGSELPPEGVLGALLR.V	3	4.57	0.39	-3.81
IPI00002280	ProSAAS precursor	D.DDPDAPAAQLAR.A	2	3.50	0.45	-1.57
IPI00002280	ProSAAS precursor	D.HDVGSELPPEGVLGALLR.V	2	4.55	0.46	-3.77
IPI00002280	ProSAAS precursor	D.HDVGSELPPEGVLGALLR.V	3	4.39	0.33	-2.70
IPI00002280	ProSAAS precursor	G.SELPPEGVLGALLR.V	2	3.34	0.39	-2.63
IPI00002280	ProSAAS precursor	H.DVGSELPPEGVLGALLR.V	2	3.59	0.23	-2.02
IPI00002280	ProSAAS precursor	K.RLETPAPQVPAR.R	2	3.12	0.31	-2.17
IPI00002280	ProSAAS precursor	L.PPEGVLGALLR.V	1	3.15	0.30	-4.21
IPI00002280	ProSAAS precursor	L.PPEGVLGALLR.V	2	3.72	0.33	-3.29
IPI00002280	ProSAAS precursor	L.SAASPPLAETGAPR.R	2	3.16	0.42	-2.67
IPI00002280	ProSAAS precursor	P.AGPDAEEAGDETPDVDPELLR.Y	2	3.03	0.32	-1.33
IPI00002280	ProSAAS precursor	P.PEGVLGALLR.V	1	2.58	0.13	-2.77
IPI00002280	ProSAAS precursor	R.AADHDVGSELPPEGVLGALL.R	2	4.36	0.47	-4.94
IPI00002280	ProSAAS precursor	R.AADHDVGSELPPEGVLGALLR.V	2	6.30	0.58	-3.82
IPI00002280	ProSAAS precursor	R.AADHDVGSELPPEGVLGALLR.V	3	3.82	0.39	-4.38
IPI00002280	ProSAAS precursor	R.AADHDVGSELPPEGVLGALLRV.K	2	5.52	0.57	-4.15
IPI00002280	ProSAAS precursor	R.ALAHLLEAERQER.A	2	3.24	0.29	-4.00
IPI00002280	ProSAAS precursor	R.ALAHLLEAERQER.A	3	2.78	0.29	
IPI00002280	ProSAAS precursor	R.ARAEAQEAEDQQAR.V	2	4.22	0.48	-5.00
IPI00002280	ProSAAS precursor	R.ARAEAQEAEDQQAR.V	3	3.69	0.28	-3.09
IPI00002280	ProSAAS precursor	R.GEAAGAVQELAR.A	2	3.87	0.25	-3.21
IPI00002280	ProSAAS precursor	R.GLSAASPPLAETGAPR.R	2	3.67	0.29	-3.71
IPI00002280	ProSAAS precursor	R.ILAGSADSEGVAAPR.R	2	4.42	0.38	-4.13
IPI00002280	ProSAAS precursor	R.ILAGSADSEGVAAPR.R	3	3.34	0.15	-3.26
IPI00002280	ProSAAS precursor	R.LETPAPQVPAR.R	2	2.45	0.08	-2.99
IPI00002280	ProSAAS precursor	R.NSDPALGLDDDPDAPAAQLAR.A	2	6.16	0.54	-3.98
IPI00002280	ProSAAS precursor	R.NSDPALGLDDDPDAPAAQLAR.A	3	3.47	0.28	-3.39
IPI00002280	ProSAAS precursor	R.SVPRGEAAGAVQELAR.A	2	3.97	0.43	-2.85
IPI00002280	ProSAAS precursor	R.SVPRGEAAGAVQELAR.A	3	3.90	0.32	-2.27
IPI00002283	Isoform 2 of Patched domain-containing protein 2	R.ETPPLEDLAANQSEDPR.N	3	3.27	0.32	-1.59
IPI00002283	Isoform 2 of Patched domain-containing protein 2	R.ETPPLEDLAANQSEDPRNQR.L	3	2.29	0.14	-1.96
IPI00002307	Isoform 1 of Neuroligin-3 precursor	K.ELVEQDIQPAR.Y	2	3.12	0.19	-1.99
IPI00002307	Isoform 1 of Neuroligin-3 precursor	K.GNYGLLDQIQALR.W	2	3.90	0.15	-2.82
IPI00002307	Isoform 1 of Neuroligin-3 precursor	R.VGVLGFLSTGDQAAK.G	2	4.50	0.26	-2.82
IPI00002307	Isoform 1 of Neuroligin-3 precursor	R.VPLPSEILGPVDQYLGVPYAAPPIGEKR.F	3	5.11	0.55	-4.61
IPI00002307	Isoform 1 of Neuroligin-3 precursor	R.VPLPSEILGPVDQYLGVPYAAPPIGEKR.F	4	3.19	0.14	-4.00
IPI00002320	Leucine-rich repeat transmembrane protein FLRT3 precursor	R.IYLYHNSLDEFPTNLPK.Y	3	3.68	0.20	-3.26

IPI00002320	Leucine-rich repeat transmembrane protein FLRT3 precursor	R.VPPNAFSYLR.Q	2	2.38	0.23	-1.26
IPI00002334	Neuron-specific protein family member 1	K.LSEQETEAAEK.S	2	4.10	0.29	-2.15
IPI00002334	Neuron-specific protein family member 1	K.LSEQETEAAEKSA.-	2	2.83	0.25	-2.88
IPI00002334	Neuron-specific protein family member 1	S.VLSEEKLSEQETEAAEK.S	2	5.67	0.56	-1.56
IPI00002406	Lutheran blood group glycoprotein precursor	R.AGAAGTAEATAR.L	2	3.72	0.42	-1.90
IPI00002406	Lutheran blood group glycoprotein precursor	R.EGDEVTLICSAR.G	2	3.38	0.37	-3.13
IPI00002406	Lutheran blood group glycoprotein precursor	R.EGDTVQLLCR.G	2	2.36	0.15	-2.72
IPI00002406	Lutheran blood group glycoprotein precursor	R.GDGSPSPEYTLFR.L	2	2.31	0.20	-1.57
IPI00002406	Lutheran blood group glycoprotein precursor	R.GRSPPYQLDSQGR.L	2	3.51	0.42	-2.29
IPI00002406	Lutheran blood group glycoprotein precursor	R.LEVPVEM*NPEGYM*TSR.T	2	4.46	0.42	-3.28
IPI00002406	Lutheran blood group glycoprotein precursor	R.LSVPPLVEVM*R.G	2	1.76	0.21	-1.47
IPI00002406	Lutheran blood group glycoprotein precursor	R.VAYLDPLELSEGK.V	2	3.62	0.42	-2.55
IPI00002406	Lutheran blood group glycoprotein precursor	R.VEDYDAADDVQLSK.T	2	5.78	0.47	-1.91
IPI00002412	Palmitoyl-protein thioesterase 1 precursor	K.LQQGYNAM*GFSQGGQFLR.A	2	4.69	0.51	-2.32
IPI00002459	annexin VI isoform 2	K.ALIEILATR.T	2	2.21	0.16	-1.98
IPI00002478	Isoform B of Endothelin-converting enzyme 1	R.QTECM*VEQYSNYSVNGEPVNGRHTLGENIADNGGLK.A	3	2.49	0.21	-3.11
IPI00002491	Isoform 9 of Sorbin and SH3 domain-containing protein 1	R.ETPSSSPASPQETR.Q	2	1.65	0.20	-3.41
IPI00002511	Cyclic AMP-dependent transcription factor ATF-6 alpha	K.EAQDTSDGIIQK.N	2	3.19	0.08	-4.05
IPI00002511	Cyclic AMP-dependent transcription factor ATF-6 alpha	R.NSGSELQVYYASPR.S	2	3.70	0.34	-3.73
IPI00002525	Neudesin precursor	K.ELEALDEVFTK.V	2	3.19	0.26	-0.66
IPI00002525	Neudesin precursor	K.GVVFDVTSGKEFYGR.G	2	4.06	0.49	-1.87
IPI00002525	Neudesin precursor	K.M*SLDPADLTHDTTGLTAK.E	2	3.69	0.44	-3.79
IPI00002525	Neudesin precursor	K.YPIVGYTAR.R	2	2.26	0.09	-1.49
IPI00002525	Neudesin precursor	R.ILNEDGSPNLDFKPEDQPHFDIKDEF.-	4	2.51	0.11	-2.44
IPI00002525	Neudesin precursor	R.LFTEEELAR.Y	2	3.05	0.29	-2.01
IPI00002535	FK506-binding protein 2 precursor	R.KLVIPSELGYGER.G	3	3.74	0.25	-1.44
IPI00002714	Dickkopf-related protein 3 precursor	C.CGDQLCVWGHCTK.M	2	3.07	0.44	2.51
IPI00002714	Dickkopf-related protein 3 precursor	E.CCGDQLCVWGHCTK.M	2	4.06	0.54	-1.91
IPI00002714	Dickkopf-related protein 3 precursor	E.VRQELEDLER.S	2	3.10	0.29	-1.59
IPI00002714	Dickkopf-related protein 3 precursor	F.ASFQYTCQPCR.G	2	3.40	0.39	-2.43
IPI00002714	Dickkopf-related protein 3 precursor	F.PVCTPLPVEGELCHDPASR.L	2	5.11	0.60	-3.23
IPI00002714	Dickkopf-related protein 3 precursor	I.TWELEPDGALDR.C	2	3.49	0.13	-0.96
IPI00002714	Dickkopf-related protein 3 precursor	K.LRSAVEEM*EAEEAAAK.A	2	3.89	0.30	-4.32
IPI00002714	Dickkopf-related protein 3 precursor	L.PVEGELCHDPASR.L	1	2.61	0.17	-1.92
IPI00002714	Dickkopf-related protein 3 precursor	Q.PGLCCAFQR.G	1	2.13	0.19	-2.75
IPI00002714	Dickkopf-related protein 3 precursor	Q.PGLCCAFQR.G	2	3.12	0.29	-2.03
IPI00002714	Dickkopf-related protein 3 precursor	R.DCQPGLCCAFQR.G	1	2.89	0.43	-1.83
IPI00002714	Dickkopf-related protein 3 precursor	R.DCQPGLCCAFQR.G	2	4.30	0.47	-4.38

IPI00002714	Dickkopf-related protein 3 precursor	R.DCQPGLCCAFQR.G	3	4.02	0.40	-1.20
IPI00002714	Dickkopf-related protein 3 precursor	R.DQDGEILLPR.E	1	2.35	0.29	-4.72
IPI00002714	Dickkopf-related protein 3 precursor	R.DQDGEILLPR.E	2	3.73	0.23	-4.50
IPI00002714	Dickkopf-related protein 3 precursor	R.DSECCGDQLCVWGHCTK.M	2	4.97	0.69	-3.04
IPI00002714	Dickkopf-related protein 3 precursor	R.DSECCGDQLCVWGHCTK.M	3	2.99	0.41	-2.24
IPI00002714	Dickkopf-related protein 3 precursor	R.EVEELM*EDTQH.K	2	3.69	0.45	-1.96
IPI00002714	Dickkopf-related protein 3 precursor	R.EVEELM*EDTQHK.L	1	2.58	0.46	0.20
IPI00002714	Dickkopf-related protein 3 precursor	R.EVEELM*EDTQHK.L	2	3.69	0.44	-3.37
IPI00002714	Dickkopf-related protein 3 precursor	R.EVEELM*EDTQHK.L	3	1.90	0.25	1.44
IPI00002714	Dickkopf-related protein 3 precursor	R.EVEELM*EDTQHKLR.S	2	3.61	0.35	-5.56
IPI00002714	Dickkopf-related protein 3 precursor	R.EVEELM*EDTQHKLR.S	3	1.46	0.14	-2.27
IPI00002714	Dickkopf-related protein 3 precursor	R.EVEELMEDTQHK.L	2	3.25	0.27	
IPI00002714	Dickkopf-related protein 3 precursor	R.EVPDEYEVGSFM*EEVR.Q	2	4.31	0.52	-4.24
IPI00002714	Dickkopf-related protein 3 precursor	R.EVPDEYEVGSFM*EEVRQELEDLER.S	2	2.54	0.45	-3.80
IPI00002714	Dickkopf-related protein 3 precursor	R.EVPDEYEVGSFM*EEVRQELEDLER.S	3	3.55	0.42	-5.57
IPI00002714	Dickkopf-related protein 3 precursor	R.EVPDEYEVGSFM*EEVRQELEDLER.S	4	2.96	0.15	-3.88
IPI00002714	Dickkopf-related protein 3 precursor	R.EVPDEYEVGSFMEEVRQELEDLER.S	3	2.25	0.40	-2.69
IPI00002714	Dickkopf-related protein 3 precursor	R.GLLFPVCTPLPVEGELCHD.P	2	4.02	0.52	-4.21
IPI00002714	Dickkopf-related protein 3 precursor	R.GLLFPVCTPLPVEGELCHDPASR.L	2	4.27	0.56	-4.62
IPI00002714	Dickkopf-related protein 3 precursor	R.GLLFPVCTPLPVEGELCHDPASR.L	3	3.00	0.37	-7.60
IPI00002714	Dickkopf-related protein 3 precursor	R.GLLFPVCTPLPVEGELCHDPASR.L	4	3.10	0.34	-2.23
IPI00002714	Dickkopf-related protein 3 precursor	R.LLDLITWELEPDGALDR.C	2	6.04	0.45	-6.15
IPI00002714	Dickkopf-related protein 3 precursor	R.LLDLITWELEPDGALDR.C	3	4.00	0.24	-4.16
IPI00002714	Dickkopf-related protein 3 precursor	R.LLDLITWELEPDGALDRCPCASGLLCQPH.S	3	4.94	0.37	-1.88
IPI00002714	Dickkopf-related protein 3 precursor	R.QELEDLER.S	1	1.37	0.08	-4.10
IPI00002714	Dickkopf-related protein 3 precursor	R.RSHECIIDEDCGPSM*YCQFASFQYTCQPCR.G	4	3.72	0.41	-2.85
IPI00002714	Dickkopf-related protein 3 precursor	R.SAVEEM*EAEEAAAK.A	1	3.27	0.35	-3.16
IPI00002714	Dickkopf-related protein 3 precursor	R.SAVEEM*EAEEAAAK.A	2	5.36	0.51	-4.96
IPI00002714	Dickkopf-related protein 3 precursor	R.SAVEEM*EAEEAAAK.A	3	4.28	0.34	-4.59
IPI00002714	Dickkopf-related protein 3 precursor	R.SAVEEMEAEEAAAK.A	2	3.04	0.26	
IPI00002714	Dickkopf-related protein 3 precursor	R.SHECIIDEDCGPSM*YCQFASFQYTCQPCR.G	3	7.47	0.74	-1.40
IPI00002714	Dickkopf-related protein 3 precursor	R.SHECIIDEDCGPSM*YCQFASFQYTCQPCR.G	4	5.57	0.50	-1.73
IPI00002714	Dickkopf-related protein 3 precursor	R.SLTEEM*ALGEPAAAAAALLGGEEI.-	2	5.48	0.54	-5.15
IPI00002714	Dickkopf-related protein 3 precursor	R.SLTEEM*ALGEPAAAAAALLGGEEI.-	3	2.69	0.27	-2.75
IPI00002714	Dickkopf-related protein 3 precursor	S.APVKPGPALSYPQEEATLNEM*FR.E	3	3.62	0.26	-0.11
IPI00002714	Dickkopf-related protein 3 precursor	S.FM*EEVRQELEDLER.S	3	3.74	0.33	-2.35
IPI00002714	Dickkopf-related protein 3 precursor	V.PDEYEVGSFM*EEVRQELEDLER.S	3	3.77	0.45	-2.56
IPI00002714	Dickkopf-related protein 3 precursor	V.RQELEDLER.S	2	3.13	0.11	-3.94
IPI00002732	EXTL2 protein (Fragment)	K.APDELWNSLGPHPIPVIFK.Q	3	2.79	0.29	-3.22
IPI00002732	EXTL2 protein (Fragment)	K.LLNHYQAVPNLHK.V	2	3.51	0.35	-3.64
IPI00002732	EXTL2 protein (Fragment)	K.LLNHYQAVPNLHK.V	3	2.71	0.15	-2.59
IPI00002732	EXTL2 protein (Fragment)	K.LVNIYDSM*PLR.Y	2	2.34	0.30	-1.02

IPI00002732	EXTL2 protein (Fragment)	K.TSGIFVKPVNM*DNLEK.E	2	3.57	0.31	-3.27
IPI00002732	EXTL2 protein (Fragment)	K.TSGIFVKPVNM*DNLEK.E	3	2.32	0.21	-3.14
IPI00002732	EXTL2 protein (Fragment)	K.VIVVWNNIGEK.A	2	2.89	0.34	-2.17
IPI00002732	EXTL2 protein (Fragment)	K.VIVVWNNIGEKAPDELWNSLGPHPIPVIFK.Q	4	3.45	0.37	-3.14
IPI00002732	EXTL2 protein (Fragment)	K.YLELFQR.Q	2	2.77	0.13	-1.34
IPI00002732	EXTL2 protein (Fragment)	R.QPAAVHALIDDTQNCDDIAM*NFIIAK.H	3	5.31	0.46	-3.60
IPI00002732	EXTL2 protein (Fragment)	R.YSNIM*ISQFGFPYANYK.K	2	5.48	0.54	-4.66
IPI00002732	EXTL2 protein (Fragment)	R.YSNIM*ISQFGFPYANYK.K	3	3.79	0.30	-3.45
IPI00002745	Cathepsin Z precursor	K.M*M*AEIYANGPISCGIM*ATER.L	2	6.47	0.64	-1.28
IPI00002745	Cathepsin Z precursor	K.M*M*AEIYANGPISCGIM*ATER.L	3	5.43	0.54	-2.59
IPI00002745	Cathepsin Z precursor	R.EKM*M*AEIYANGPISCGIM*ATER.L	3	4.39	0.47	-0.58
IPI00002745	Cathepsin Z precursor	R.IVTSTYKDGK.G	1	2.51	0.29	-4.54
IPI00002745	Cathepsin Z precursor	R.IVTSTYKDGK.G	2	3.09	0.31	-2.59
IPI00002745	Cathepsin Z precursor	R.NSWGEPWGER.G	2	2.34	0.15	-2.79
IPI00002745	Cathepsin Z precursor	R.NVDGVNYASITR.N	2	3.47	0.37	-3.03
IPI00002745	Cathepsin Z precursor	R.STYPRPHEYLSPADLPK.S	2	3.90	0.42	-5.08
IPI00002745	Cathepsin Z precursor	R.STYPRPHEYLSPADLPK.S	3	4.52	0.35	-1.28
IPI00002745	Cathepsin Z precursor	R.VGDYGSLSGR.E	1	2.28	0.33	-3.25
IPI00002745	Cathepsin Z precursor	R.VGDYGSLSGR.E	2	3.97	0.41	-4.17
IPI00002745	Cathepsin Z precursor	R.YNLAIEEHCTFGDPIV.-	2	3.92	0.41	-5.04
IPI00002790	Isoform 1 of Protein sel-1 homolog 1 precursor	R.VVAGQIFLDSEESELESSIQEEEDSLK.S	3	6.58	0.55	-2.82
IPI00002816	Cathepsin F precursor	K.DCGPVDTKVPGAGEPK.S	2	3.54	0.33	-1.21
IPI00002816	Cathepsin F precursor	K.FSDLTEEEFR.T	2	1.41	0.09	-2.93
IPI00002816	Cathepsin F precursor	K.GYYYLHR.G	2	2.11	0.21	-1.76
IPI00002816	Cathepsin F precursor	K.KTLLCSFQVLDELGR.H	2	4.14	0.46	-3.15
IPI00002816	Cathepsin F precursor	K.KTLLCSFQVLDELGR.H	3	4.07	0.33	-3.46
IPI00002816	Cathepsin F precursor	K.TLLCSFQVLDELGR.H	2	4.73	0.44	-3.88
IPI00002816	Cathepsin F precursor	K.TLLCSFQVLDELGR.H	3	2.82	0.08	-2.86
IPI00002816	Cathepsin F precursor	R.AASFQAWGPPSPELLAPTR.F	2	3.58	0.38	1.70
IPI00002816	Cathepsin F precursor	R.FALEM*FNR.G	2	2.73	0.27	-2.79
IPI00002816	Cathepsin F precursor	R.GTAQYGVTK.F	2	2.95	0.37	-0.66
IPI00002816	Cathepsin F precursor	R.TIYLNTLLR.K	2	3.09	0.13	-1.29
IPI00002818	Isoform 1 of Kallikrein-11 precursor	K.M*ASPVSITWAVRPLTLSSR.C	3	2.18	0.24	-3.33
IPI00002884	CDNA: FLJ22222 fis, clone HRC01658	R.YFGDKIQNIFSEEDFR.L	3	3.31	0.25	-2.34
IPI00002925	Cocaine- and amphetamine-regulated transcript protein precursor	A.VDDASHEKELIEALQEVLKK.L	3	4.31	0.33	-3.85
IPI00002925	Cocaine- and amphetamine-regulated transcript protein precursor	K.ELIEALQEVLK.K	2	3.85	0.13	-2.18
IPI00002925	Cocaine- and amphetamine-regulated transcript protein precursor	K.YGQVPM*CDAGEQCAVR.K	2	3.83	0.38	-4.90
IPI00002925	Cocaine- and amphetamine-regulated transcript protein precursor	K.YGQVPM*CDAGEQCAVR.K	3	4.06	0.43	-4.70

IPI00002925	Cocaine- and amphetamine-regulated transcript protein precursor	R.ALDIYSAVDDASHEKELIEALQEVLK.K	3	7.10	0.59	-5.50
IPI00002925	Cocaine- and amphetamine-regulated transcript protein precursor	R.ALDIYSAVDDASHEKELIEALQEVLK.K	4	3.56	0.24	-3.23
IPI00002925	Cocaine- and amphetamine-regulated transcript protein precursor	R.GTSCNSFLLK.C	2	2.05	0.08	-1.39
IPI00002925	Cocaine- and amphetamine-regulated transcript protein precursor	S.AVDDASHEKELIEALQEVLK.K	3	5.29	0.42	-2.35
IPI00002966	Heat shock 70 kDa protein 4	K.LFEELGK.Q	2	2.14	0.07	-3.69
IPI00002966	Heat shock 70 kDa protein 4	R.AFSDPFVEAEKSNLAYDIVQLPTGLTGIK.V	3	4.03	0.40	-2.65
IPI00002993	Transcription initiation factor TFIID subunit 9	K.REDDDDDDDDDDDYDNL.-	2	1.12	0.21	-7.27
IPI00003021	Sodium/potassium-transporting ATPase subunit alpha-2 precursor	K.LSLDELGR.K	2	2.60	0.11	-2.68
IPI00003021	Sodium/potassium-transporting ATPase subunit alpha-2 precursor	K.QAADMILLDDNFASIVTGVEEGR.L	2	5.62	0.61	-2.21
IPI00003021	Sodium/potassium-transporting ATPase subunit alpha-2 precursor	K.QAADMILLDDNFASIVTGVEEGR.L	3	4.80	0.48	-1.61
IPI00003021	Sodium/potassium-transporting ATPase subunit alpha-2 precursor	K.VDNSSLTGESEPQTR.S	2	4.55	0.40	-2.59
IPI00003021	Sodium/potassium-transporting ATPase subunit alpha-2 precursor	R.DTAGDASESALLK.C	2	3.87	0.35	-1.81
IPI00003021	Sodium/potassium-transporting ATPase subunit alpha-2 precursor	R.SPEFTHENPLETR.N	3	3.77	0.37	-1.20
IPI00003031	Isoform 2 of Isochorismatase domain-containing protein 2, mitochondrial precursor	R.QSGAFLSTSEGLILQLVGDAVHPQFK.E	3	2.38	0.11	-2.97
IPI00003102	Ciliary neurotrophic factor receptor alpha precursor	K.DNEIGTWSDWSVAAHATPWTEEPR.H	3	2.55	0.21	-7.86
IPI00003102	Ciliary neurotrophic factor receptor alpha precursor	K.EYIIQVAAK.D	2	2.31	0.21	-3.56
IPI00003102	Ciliary neurotrophic factor receptor alpha precursor	R.HSPQEAPHVQYER.L	2	2.75	0.36	-4.81
IPI00003102	Ciliary neurotrophic factor receptor alpha precursor	R.YM*HLFSTIK.Y	2	2.04	0.16	-3.14
IPI00003102	Ciliary neurotrophic factor receptor alpha precursor	R.YRPLILDQWQHVELSDGTAHTITDAYAGK.E	4	3.77	0.25	-2.49
IPI00003102	Ciliary neurotrophic factor receptor alpha precursor	R.YRPLILDQWQHVELSDGTAHTITDAYAGK.E	5	3.21	0.18	-3.45
IPI00003111	Ig kappa chain V-I region AU	R.VTITCQASQDISNYLNWYQQKPGK.A	3	5.06	0.16	
IPI00003176	Serine protease HTRA1 precursor	K.IDHQGKLPVLLLGR.S	2	3.21	0.38	-3.03
IPI00003176	Serine protease HTRA1 precursor	K.IDHQGKLPVLLLGR.S	3	2.97	0.15	
IPI00003176	Serine protease HTRA1 precursor	R.LHRPPVIVLQR.G	2	2.62	0.30	-4.30
IPI00003176	Serine protease HTRA1 precursor	R.LHRPPVIVLQR.G	3	3.32	0.24	-5.55

IPI00003176	Serine protease HTRA1 precursor	R.M*M*SLTSSK.A	2	2.33	0.13	-2.32
IPI00003176	Serine protease HTRA1 precursor	R.RAQAGLCVCASSEPVCGSDANTYANLCQLR.A	3	3.17	0.19	
IPI00003176	Serine protease HTRA1 precursor	R.SSELRPGEFVVAIGSPFSLQNTVTTGIVSTTQR.G	3	6.85	0.58	-3.17
IPI00003269	hypothetical protein LOC345651	K.IIAPPERK.Y	2	1.97	0.05	-3.56
IPI00003269	hypothetical protein LOC345651	K.IKIIAPPER.K	2	2.50	0.12	2.48
IPI00003269	hypothetical protein LOC345651	K.IKIIAPPERK.Y	2	2.55	0.12	-3.24
IPI00003269	hypothetical protein LOC345651	K.YSVWIGGSILASLSTFQQMWISK.Q	2	1.77	0.24	-2.29
IPI00003269	hypothetical protein LOC345651	R.DLTDYLM*K.I	1	2.48	0.24	-3.46
IPI00003269	hypothetical protein LOC345651	R.DLTDYLM*K.I	2	2.37	0.25	-3.00
IPI00003269	hypothetical protein LOC345651	R.HQGVM*VGM*GQK.D	2	2.78	0.19	
IPI00003269	hypothetical protein LOC345651	R.LDLAGRDLTDYLM*K.I	2	2.69	0.21	-4.38
IPI00003269	hypothetical protein LOC345651	R.LDLAGRDLTDYLM*K.I	3	3.36	0.32	-1.97
IPI00003269	hypothetical protein LOC345651	R.SYELPDGQVITIGNER.F	2	4.30	0.39	-4.42
IPI00003348	Guanine nucleotide-binding protein G(I)/G(S)/G(T) subunit beta-2	R.VSCLGVTDDGMAVATGSWDSFLK.I	2	5.50	0.58	-2.87
IPI00003351	Extracellular matrix protein 1 precursor	A.ASEGGFTATGQR.Q	1	2.63	0.39	-3.45
IPI00003351	Extracellular matrix protein 1 precursor	A.ASEGGFTATGQR.Q	2	4.18	0.42	-2.33
IPI00003351	Extracellular matrix protein 1 precursor	A.SEGGFTATGQR.Q	1	1.93	0.19	-1.68
IPI00003351	Extracellular matrix protein 1 precursor	A.SEGGFTATGQR.Q	2	3.60	0.38	-1.33
IPI00003351	Extracellular matrix protein 1 precursor	K.ELPSLQHPNEQK.E	2	2.88	0.29	-6.48
IPI00003351	Extracellular matrix protein 1 precursor	K.EVGPPLPQEAVPLQK.E	2	3.28	0.32	-2.38
IPI00003351	Extracellular matrix protein 1 precursor	K.LLPAQLPAEK.E	1	2.11	0.09	-2.57
IPI00003351	Extracellular matrix protein 1 precursor	K.LLPAQLPAEK.E	2	2.19	0.12	-2.12
IPI00003351	Extracellular matrix protein 1 precursor	K.LLPAQLPAEKEVGPPLPQEAVPLQK.E	2	4.83	0.53	-3.06
IPI00003351	Extracellular matrix protein 1 precursor	K.LLPAQLPAEKEVGPPLPQEAVPLQK.E	3	4.02	0.53	-5.04
IPI00003351	Extracellular matrix protein 1 precursor	K.LLPAQLPAEKEVGPPLPQEAVPLQK.E	4	4.13	0.39	-2.13
IPI00003351	Extracellular matrix protein 1 precursor	K.LVWEEAM*SR.F	2	3.09	0.35	-2.48
IPI00003351	Extracellular matrix protein 1 precursor	L.PAQLPAEKEVGPPLPQEAVPLQK.E	3	6.33	0.55	-2.34
IPI00003351	Extracellular matrix protein 1 precursor	R.ACPSHQPDISSGLELPFPPGVPTLDNIK.N	2	4.25	0.43	-4.35
IPI00003351	Extracellular matrix protein 1 precursor	R.ACPSHQPDISSGLELPFPPGVPTLDNIK.N	3	5.33	0.36	-7.70
IPI00003351	Extracellular matrix protein 1 precursor	R.ACPSHQPDISSGLELPFPPGVPTLDNIK.N	4	3.82	0.31	-2.70
IPI00003351	Extracellular matrix protein 1 precursor	R.APYPNYDRDILTIDIGR.V	3	2.60	0.28	-2.01
IPI00003351	Extracellular matrix protein 1 precursor	R.CCDLPFPEQACCAEEEKLTFINDLCGPR.R	3	3.87	0.47	-4.23
IPI00003351	Extracellular matrix protein 1 precursor	R.CCDLPFPEQACCAEEEKLTFINDLCGPR.R	4	3.28	0.35	-5.73
IPI00003351	Extracellular matrix protein 1 precursor	R.ELLALIQLER.E	1	3.11	0.20	-4.28
IPI00003351	Extracellular matrix protein 1 precursor	R.ELLALIQLER.E	2	4.01	0.27	-5.45
IPI00003351	Extracellular matrix protein 1 precursor	R.FCEAEFSVK.T	1	2.23	0.25	-3.77
IPI00003351	Extracellular matrix protein 1 precursor	R.FCEAEFSVK.T	2	2.94	0.33	-2.10
IPI00003351	Extracellular matrix protein 1 precursor	R.LDGFPPGRPSPDNLNQICLPNR.Q	3	4.89	0.49	-3.46
IPI00003351	Extracellular matrix protein 1 precursor	R.NIWRDPALCCYLSPGDEQVNCFNINYLR.N	3	6.90	0.63	-2.70
IPI00003351	Extracellular matrix protein 1 precursor	R.NLPATDPLQR.E	2	2.29	0.20	-3.06
IPI00003351	Extracellular matrix protein 1 precursor	R.NVALVSGDTENAK.G	1	3.20	0.43	-1.69

IPI00003351	Extracellular matrix protein 1 precursor	R.NVALVSGDTENAK.G	2	4.50	0.47	-1.67
IPI00003351	Extracellular matrix protein 1 precursor	R.QGETLNFLEIGYSR.C	2	4.99	0.47	-5.28
IPI00003351	Extracellular matrix protein 1 precursor	R.QLRPEHFQEVGYAAPPSPPLSR.S	2	2.82	0.33	-2.08
IPI00003351	Extracellular matrix protein 1 precursor	R.QLRPEHFQEVGYAAPPSPPLSR.S	3	3.81	0.24	-1.15
IPI00003351	Extracellular matrix protein 1 precursor	R.RAPYPNYDR.D	2	3.12	0.25	-2.89
IPI00003351	Extracellular matrix protein 1 precursor	R.RAPYPNYDRDILTIDIGR.V	3	3.27	0.19	-3.37
IPI00003351	Extracellular matrix protein 1 precursor	R.SLPM*DHPDSSQHGPPFEGQSQVQPPPSQEATPLQQEK.L	3	5.11	0.44	-3.15
IPI00003351	Extracellular matrix protein 1 precursor	R.SLPM*DHPDSSQHGPPFEGQSQVQPPPSQEATPLQQEK.L	4	4.16	0.41	-4.11
IPI00003353	Neuronal protein 3.1	R.LPKGRLPVPKEVNR.K	3	2.53	0.23	
IPI00003362	HSPA5 protein	K.DAGTIAGLNVM*R.I	2	4.17	0.31	-4.62
IPI00003362	HSPA5 protein	K.DNHLLGTFDLTGIPPAPR.G	3	2.90	0.26	-3.23
IPI00003362	HSPA5 protein	K.ELEEIVQPIISK.L	2	3.62	0.33	-2.70
IPI00003362	HSPA5 protein	K.KKELEEIVQPIISK.L	2	5.20	0.34	-4.19
IPI00003362	HSPA5 protein	K.KKELEEIVQPIISK.L	3	3.50	0.09	-3.21
IPI00003362	HSPA5 protein	K.KKELEEIVQPIISK.L	4	2.83	0.17	-4.51
IPI00003362	HSPA5 protein	K.KSQIFSTASDNQPTVTIK.V	3	3.05	0.25	-3.03
IPI00003362	HSPA5 protein	K.NGRVEIIANDQGNR.I	2	2.21	0.08	-3.02
IPI00003362	HSPA5 protein	K.NKITITNDQNR.L	2	2.86	0.20	-1.66
IPI00003362	HSPA5 protein	K.NQLTSNPENTVFDAK.R	2	3.66	0.42	-1.55
IPI00003362	HSPA5 protein	K.NQLTSNPENTVFDAKR.L	2	3.79	0.49	-1.19
IPI00003362	HSPA5 protein	K.SQIFSTASDNQPTVTIK.V	2	5.17	0.48	-2.61
IPI00003362	HSPA5 protein	K.SQIFSTASDNQPTVTIK.V	3	3.36	0.25	-1.00
IPI00003362	HSPA5 protein	K.TFAPEEISAM*VLTK.M	2	4.24	0.30	-3.32
IPI00003362	HSPA5 protein	K.TFAPEEISAMVLTK.M	2	3.09	0.27	
IPI00003362	HSPA5 protein	K.TKPYIQVDIGGGQTK.T	2	3.55	0.26	-3.33
IPI00003362	HSPA5 protein	K.TKPYIQVDIGGGQTK.T	3	3.67	0.27	-3.05
IPI00003362	HSPA5 protein	K.VLEDSDLK.K	1	1.88	0.12	-4.20
IPI00003362	HSPA5 protein	K.VLEDSDLKKSDIDEIVLVGGSTR.I	3	4.15	0.30	-1.98
IPI00003362	HSPA5 protein	K.VLEDSDLKKSDIDEIVLVGGSTR.I	4	2.26	0.10	-2.09
IPI00003362	HSPA5 protein	K.VTHAVVTVPAYFNDAQR.Q	3	4.25	0.27	-2.82
IPI00003362	HSPA5 protein	K.VYEGERPLTK.D	2	2.85	0.37	-3.30
IPI00003362	HSPA5 protein	K.VYEGERPLTKDNHLLGTFDLTGIPPAPR.G	3	3.77	0.34	-3.89
IPI00003362	HSPA5 protein	R.AKFEELNM*DLFR.S	2	4.01	0.41	-4.50
IPI00003362	HSPA5 protein	R.AKFEELNM*DLFR.S	3	3.89	0.27	-2.29
IPI00003362	HSPA5 protein	R.IDTRNELESYAYSLK.N	2	2.82	0.10	-2.42
IPI00003362	HSPA5 protein	R.IDTRNELESYAYSLK.N	3	4.57	0.30	-2.45
IPI00003362	HSPA5 protein	R.IEIESFFEGEDFSETLTR.A	2	5.49	0.49	-2.33
IPI00003362	HSPA5 protein	R.IINEPTAAAIAYGLDKR.E	2	4.60	0.49	-2.05
IPI00003362	HSPA5 protein	R.IINEPTAAAIAYGLDKR.E	3	2.56	0.07	-2.33
IPI00003362	HSPA5 protein	R.ITPSYVAFTPEGER.L	2	3.52	0.42	-2.76
IPI00003362	HSPA5 protein	R.LIGDAAK.N	1	1.96	0.12	-2.67
IPI00003362	HSPA5 protein	R.M*VNDAEKFAEEDKK.L	3	3.07	0.17	-2.18

IPI00003362	HSPA5 protein	R.M*VNDAEKFAEEDKK.L	4	2.52	0.25	-6.35
IPI00003362	HSPA5 protein	R.NTVVPTKK.S	2	1.99	0.07	-3.94
IPI00003362	HSPA5 protein	R.VM*EHFIK.L	2	2.30	0.06	-0.64
IPI00003362	HSPA5 protein	W.NDPSVQQDIK.F	2	3.04	0.31	-5.02
IPI00003363	Isoform 1 of Protein phosphatase 1 regulatory subunit 1B	R.LSEHSSPEEEASPHQR.A	3	4.94	0.31	-0.14
IPI00003366	Isoform TrkB of BDNF/NT-3 growth factors receptor precursor	K.GNPKPALQWFYNGAILNESK.Y	2	5.05	0.52	-4.03
IPI00003366	Isoform TrkB of BDNF/NT-3 growth factors receptor precursor	K.GNPKPALQWFYNGAILNESK.Y	3	5.18	0.52	-3.92
IPI00003366	Isoform TrkB of BDNF/NT-3 growth factors receptor precursor	K.NSNLQHINFTR.N	2	3.33	0.40	-2.99
IPI00003366	Isoform TrkB of BDNF/NT-3 growth factors receptor precursor	K.NSNLQHINFTR.N	3	1.52	0.14	-4.25
IPI00003366	Isoform TrkB of BDNF/NT-3 growth factors receptor precursor	K.RLEIINEDDVEAYVGLR.N	3	3.59	0.30	-4.64
IPI00003366	Isoform TrkB of BDNF/NT-3 growth factors receptor precursor	K.SITLSCSVAGDPVPNM*YWDVGNLVSK.H	2	4.58	0.54	-3.86
IPI00003366	Isoform TrkB of BDNF/NT-3 growth factors receptor precursor	K.SITLSCSVAGDPVPNM*YWDVGNLVSK.H	3	2.51	0.15	-3.89
IPI00003366	Isoform TrkB of BDNF/NT-3 growth factors receptor precursor	K.SSPDTQDLYCLNESSK.N	2	5.41	0.58	-2.41
IPI00003366	Isoform TrkB of BDNF/NT-3 growth factors receptor precursor	R.ITNISSDDSGK.Q	2	2.84	0.17	-2.53
IPI00003366	Isoform TrkB of BDNF/NT-3 growth factors receptor precursor	R.IWCSDPSPGIVAFPR.L	2	4.73	0.51	-3.95
IPI00003366	Isoform TrkB of BDNF/NT-3 growth factors receptor precursor	R.IWCSDPSPGIVAFPR.L	3	4.95	0.45	-2.66
IPI00003366	Isoform TrkB of BDNF/NT-3 growth factors receptor precursor	R.LEIINEDDVEAYVGLR.N	2	3.30	0.31	-8.16
IPI00003384	Isoform 1 of Cadherin EGF LAG seven-pass G-type receptor 1 precursor	R.DANSVITYQLTGGNTR.N	2	2.88	0.08	
IPI00003391	Teneurin-1	R.ASEASLNSPR.G	2	2.81	0.14	-2.42
IPI00003391	Teneurin-1	R.DYDVVAGR.W	2	2.52	0.25	-5.55
IPI00003392	Transmembrane protein 5	K.SM*GAPFIFIK.N	2	2.50	0.28	-1.38
IPI00003406	Isoform 1 of Drebrin	R.LELLAAYEEVIREESAADWALYTYEDGSDDLK.L	3	3.89	0.43	-2.75
IPI00003441	Isoform 1 of Protein C1orf9 precursor	K.ILAANPEAK.S	2	2.42	0.16	-2.80
IPI00003441	Isoform 1 of Protein C1orf9 precursor	R.EGPINAESLGK.S	2	1.91	0.20	-2.74
IPI00003470	Ig kappa chain V-I region Wes	-.DIQM*TQSPSSVSASVGDR.V	2	5.81	0.37	
IPI00003470	Ig kappa chain V-I region Wes	-.DIQM*TQSPSSVSASVGDR.V	3	4.33	0.36	
IPI00003470	Ig kappa chain V-I region Wes	-.DIQMTQSPSSVSASVGDR.V	2	4.26	0.10	
IPI00003470	Ig kappa chain V-I region Wes	-.DIQMTQSPSSVSASVGDR.V	3	2.97	0.13	

IPI00003527	Ezrin-radixin-moesin-binding phosphoprotein 50	R.AQEAPGQAEPPAAAEVQGAGNENEPR.E	3	2.70	0.20	-2.79
IPI00003527	Ezrin-radixin-moesin-binding phosphoprotein 50	R.SVDPDSPAEASGLR.A	2	3.65	0.35	-2.61
IPI00003590	Isoform 1 of Sulfhydryl oxidase 1 precursor	K.AHFSPSNIILDFPAAGSAAR.R	2	5.58	0.58	-3.66
IPI00003590	Isoform 1 of Sulfhydryl oxidase 1 precursor	K.AHFSPSNIILDFPAAGSAAR.R	3	4.87	0.55	-3.00
IPI00003590	Isoform 1 of Sulfhydryl oxidase 1 precursor	K.FGVTDFPSCYLLFR.N	2	2.89	0.29	-4.31
IPI00003590	Isoform 1 of Sulfhydryl oxidase 1 precursor	K.IPYSFFK.T	2	1.71	0.06	-2.02
IPI00003590	Isoform 1 of Sulfhydryl oxidase 1 precursor	K.TALDDRKEGAVLAK.K	3	2.17	0.15	-2.38
IPI00003590	Isoform 1 of Sulfhydryl oxidase 1 precursor	R.AAPGQEPPEHM*AELQR.N	2	3.67	0.44	-2.77
IPI00003590	Isoform 1 of Sulfhydryl oxidase 1 precursor	R.DTGAALLAESR.A	2	3.84	0.28	-2.50
IPI00003590	Isoform 1 of Sulfhydryl oxidase 1 precursor	R.EVALDLSQHK.G	2	2.86	0.21	-1.60
IPI00003590	Isoform 1 of Sulfhydryl oxidase 1 precursor	R.KFGVTDFPSCYLLFR.N	3	3.63	0.30	-2.59
IPI00003590	Isoform 1 of Sulfhydryl oxidase 1 precursor	R.LAGAPSEDPQFPK.V	2	3.53	0.40	-4.10
IPI00003590	Isoform 1 of Sulfhydryl oxidase 1 precursor	R.LDVPVWDVEATLNFLK.A	2	4.95	0.50	-5.37
IPI00003590	Isoform 1 of Sulfhydryl oxidase 1 precursor	R.LDVPVWDVEATLNFLK.A	3	6.31	0.53	-4.39
IPI00003590	Isoform 1 of Sulfhydryl oxidase 1 precursor	R.NNEEYLALIFEK.G	2	4.20	0.37	-5.06
IPI00003590	Isoform 1 of Sulfhydryl oxidase 1 precursor	R.RVLNTEANVVR.K	2	3.49	0.33	-2.97
IPI00003590	Isoform 1 of Sulfhydryl oxidase 1 precursor	R.RVLNTEANVVR.K	3	2.69	0.18	-2.76
IPI00003590	Isoform 1 of Sulfhydryl oxidase 1 precursor	R.SALYSPSDPLTLLQADTVR.G	3	6.06	0.54	-3.96
IPI00003590	Isoform 1 of Sulfhydryl oxidase 1 precursor	R.SALYSPSDPLTLLQADTVRGAVLGSR.S	3	2.36	0.15	-2.88
IPI00003590	Isoform 1 of Sulfhydryl oxidase 1 precursor	R.SFYTAYLQR.L	1	1.85	0.21	-2.12
IPI00003590	Isoform 1 of Sulfhydryl oxidase 1 precursor	R.SFYTAYLQR.L	2	2.77	0.24	-1.56
IPI00003590	Isoform 1 of Sulfhydryl oxidase 1 precursor	R.VLNTEANVVR.K	1	1.31	0.11	-2.20
IPI00003590	Isoform 1 of Sulfhydryl oxidase 1 precursor	R.VPVLM*ESR.S	2	2.48	0.20	-1.41
IPI00003648	Isoform Delta of Poliovirus receptor-related protein 1 precursor	K.ITQVTWQK.S	2	2.50	0.10	-0.49
IPI00003648	Isoform Delta of Poliovirus receptor-related protein 1 precursor	K.PTNWIEGTQAVLR.A	2	4.28	0.40	-3.74
IPI00003648	Isoform Delta of Poliovirus receptor-related protein 1 precursor	K.QNVAIYNPSM*GVSVLAPYR.E	2	5.43	0.52	-2.71
IPI00003648	Isoform Delta of Poliovirus receptor-related protein 1 precursor	R.LELEDEGVYICEFATFPTGNR.E	2	4.34	0.53	-4.73
IPI00003648	Isoform Delta of Poliovirus receptor-related protein 1 precursor	R.LKGEAEYQEIR.N	2	3.43	0.34	-3.87
IPI00003648	Isoform Delta of Poliovirus receptor-related protein 1 precursor	R.LKGEAEYQEIR.N	3	3.33	0.13	-3.74
IPI00003648	Isoform Delta of Poliovirus receptor-related protein 1 precursor	R.TLFFKGPINYSLAGTYICEATNPIGTR.S	3	3.22	0.26	-2.38
IPI00003648	Isoform Delta of Poliovirus receptor-related protein 1 precursor	R.VEFLRPSFTDGTIR.L	2	2.56	0.20	-3.39
IPI00003648	Isoform Delta of Poliovirus receptor-related protein 1 precursor	R.VEFLRPSFTDGTIR.L	3	3.22	0.39	-2.66
IPI00003799	Isoform 2 of Heme-binding protein 2	K.LNSYIQGK.N	2	2.10	0.07	2.75

IPI00003799	Isoform 2 of Heme-binding protein 2	K.NQEQLLTLASILR.E	2	4.05	0.32	-2.86
IPI00003799	Isoform 2 of Heme-binding protein 2	R.SFDGFSSAQK.N	2	1.92	0.18	-3.80
IPI00003802	Alpha-mannosidase 2	K.DKLTYDSYSPDTFLEM*DLK.Q	3	4.22	0.36	-4.01
IPI00003802	Alpha-mannosidase 2	K.DWVVVDYGTR.L	2	2.82	0.22	-3.01
IPI00003802	Alpha-mannosidase 2	K.FIWSEISYLSK.W	2	2.09	0.13	-2.19
IPI00003802	Alpha-mannosidase 2	K.FLSSSLYTALTEAR.R	2	3.57	0.39	-3.68
IPI00003802	Alpha-mannosidase 2	K.FSSPTLELQGEFSPLQSSLPCDIHLVNLR.T	3	6.08	0.54	-3.60
IPI00003802	Alpha-mannosidase 2	K.GTGLFCSTTQGK.I	2	3.18	0.35	-1.64
IPI00003802	Alpha-mannosidase 2	K.IIGNSAFLLILK.D	2	3.56	0.25	-4.07
IPI00003802	Alpha-mannosidase 2	K.ILESASSNSHLADYVLYK.N	2	4.65	0.51	-3.06
IPI00003802	Alpha-mannosidase 2	K.ILESASSNSHLADYVLYK.N	3	2.84	0.25	-2.39
IPI00003802	Alpha-mannosidase 2	K.INKFLSSSLYTALTEAR.R	3	3.78	0.26	-3.98
IPI00003802	Alpha-mannosidase 2	K.IQFGTLSDFFDALDK.A	2	3.56	0.27	-5.81
IPI00003802	Alpha-mannosidase 2	K.IQFGTLSDFFDALDKADETQR.D	2	5.18	0.51	-5.89
IPI00003802	Alpha-mannosidase 2	K.IQFGTLSDFFDALDKADETQR.D	3	3.75	0.40	-4.14
IPI00003802	Alpha-mannosidase 2	K.ITANLFR.I	2	2.05	0.10	-2.23
IPI00003802	Alpha-mannosidase 2	K.LPLQANVYPM*TTM*AYIQDAK.H	2	4.09	0.35	0.37
IPI00003802	Alpha-mannosidase 2	K.LPLQANVYPM*TTM*AYIQDAK.H	3	4.44	0.38	-6.23
IPI00003802	Alpha-mannosidase 2	K.NKVEDSGIFTIK.N	2	2.89	0.14	-2.23
IPI00003802	Alpha-mannosidase 2	K.NKVEDSGIFTIK.N	3	3.36	0.07	-2.87
IPI00003802	Alpha-mannosidase 2	K.SGAYLFLPDGNAKPYVYTTPPFVR.V	3	4.01	0.41	-3.20
IPI00003802	Alpha-mannosidase 2	K.TFNDYFR.D	2	2.27	0.11	-2.33
IPI00003802	Alpha-mannosidase 2	K.TLEFFWR.Q	2	2.78	0.19	-2.26
IPI00003802	Alpha-mannosidase 2	K.VKIQFGTLSDFFDALDKADETQR.D	3	4.31	0.34	-4.16
IPI00003802	Alpha-mannosidase 2	K.VKIQFGTLSDFFDALDKADETQR.D	4	4.62	0.34	-2.74
IPI00003802	Alpha-mannosidase 2	K.VLLAPLGDDFR.Y	2	2.92	0.31	-1.84
IPI00003802	Alpha-mannosidase 2	R.DKTQYIFNNM*VLK.L	2	3.47	0.19	-1.25
IPI00003802	Alpha-mannosidase 2	R.DSVINLSESVEDGPK.S	2	4.63	0.29	-3.72
IPI00003802	Alpha-mannosidase 2	R.FDQTGLM*K.Q	2	2.48	0.14	-2.27
IPI00003802	Alpha-mannosidase 2	R.FYTDLNGYQIQPR.M	2	3.28	0.25	-7.31
IPI00003802	Alpha-mannosidase 2	R.GLEQGIQDNK.I	2	3.10	0.33	-1.04
IPI00003802	Alpha-mannosidase 2	R.GLEQGIQDNKITANLFR.I	2	3.50	0.43	-2.35
IPI00003802	Alpha-mannosidase 2	R.IYSEVTCFFDHVTHR.V	4	2.84	0.13	-3.01
IPI00003802	Alpha-mannosidase 2	R.LFHSLM*VLEK.I	3	2.74	0.21	-4.57
IPI00003802	Alpha-mannosidase 2	R.LM*QDDNRGLEQGIQDNK.I	3	4.08	0.24	-2.52
IPI00003802	Alpha-mannosidase 2	R.NLGLFQHHDAITGTAK.D	2	3.73	0.54	-2.51
IPI00003802	Alpha-mannosidase 2	R.NLGLFQHHDAITGTAK.D	3	2.38	0.30	-2.00
IPI00003802	Alpha-mannosidase 2	R.SGWAIDPFGHSPTM*AYLLNR.A	3	2.84	0.28	-2.89
IPI00003802	Alpha-mannosidase 2	R.YLVVYNPLEQDR.I	2	3.60	0.34	-4.70
IPI00003807	Lysosomal acid phosphatase precursor	R.LQGGVLLAQIR.K	2	3.35	0.30	-1.62
IPI00003814	Isoform 1 of Dual specificity mitogen-activated protein kinase kinase 6	-.MELGRGAYGVVEKMRHVPSGQIMAVKR.I	3	2.55	0.16	-3.20

IPI00003865	Isoform 1 of Heat shock cognate 71 kDa protein	K.DAGTIAGLNVLR.I	2	3.71	0.22	-2.68
IPI00003865	Isoform 1 of Heat shock cognate 71 kDa protein	K.ITITNDKGR.L	1	2.08	0.20	-3.55
IPI00003865	Isoform 1 of Heat shock cognate 71 kDa protein	K.ITITNDKGR.L	2	2.65	0.24	-2.81
IPI00003865	Isoform 1 of Heat shock cognate 71 kDa protein	K.NSLESYAFNM*K.A	2	3.74	0.46	-2.15
IPI00003865	Isoform 1 of Heat shock cognate 71 kDa protein	K.VQVEYKGETK.S	2	2.40	0.21	-2.65
IPI00003865	Isoform 1 of Heat shock cognate 71 kDa protein	R.LIGDAAK.N	1	1.96	0.12	-2.67
IPI00003865	Isoform 1 of Heat shock cognate 71 kDa protein	R.M*VQEAEKYK.A	2	2.07	0.11	-2.60
IPI00003865	Isoform 1 of Heat shock cognate 71 kDa protein	R.NVLIFDLGGGTFDVSILTIEDGIFEVK.S	3	3.32	0.29	-3.03
IPI00003865	Isoform 1 of Heat shock cognate 71 kDa protein	R.TTPSYVAFTDTER.L	2	2.87	0.41	-0.70
IPI00003907	Isoform 1 of Protocadherin gamma C5 precursor	A.SLANPVLESTPVGTVVGLFNVR.D	2	4.66	0.51	-2.91
IPI00003907	Isoform 1 of Protocadherin gamma C5 precursor	A.SLANPVLESTPVGTVVGLFNVR.D	3	4.62	0.39	-4.00
IPI00003907	Isoform 1 of Protocadherin gamma C5 precursor	L.EDDSDTQQVVVLVR.D	2	3.75	0.30	-3.98
IPI00003907	Isoform 1 of Protocadherin gamma C5 precursor	R.ALLEDDSDTQQVVVLVR.D	2	4.74	0.30	-4.37
IPI00003907	Isoform 1 of Protocadherin gamma C5 precursor	R.ALLEDDSDTQQVVVLVR.D	3	2.77	0.23	-3.26
IPI00003907	Isoform 1 of Protocadherin gamma C5 precursor	R.NLFGLDPSSGAIHVLGPIDFEESR.F	3	3.67	0.29	
IPI00003907	Isoform 1 of Protocadherin gamma C5 precursor	R.VGIPENAPIGTLLLR.L	2	2.62	0.24	-3.96
IPI00003907	Isoform 1 of Protocadherin gamma C5 precursor	R.YSVVEESEPGTLVGNVAQDLGLK.M	2	5.14	0.48	
IPI00003907	Isoform 1 of Protocadherin gamma C5 precursor	R.YSVVEESEPGTLVGNVAQDLGLK.M	3	3.40	0.21	
IPI00003919	Glutaminyl-peptide cyclotransferase precursor	K.M*ASTPHPPGAR.G	2	3.21	0.26	
IPI00003919	Glutaminyl-peptide cyclotransferase precursor	R.LQAIEHELHELGLLK.D	2	3.32	0.28	
IPI00003919	Glutaminyl-peptide cyclotransferase precursor	R.LQAIEHELHELGLLK.D	3	4.98	0.24	
IPI00003919	Glutaminyl-peptide cyclotransferase precursor	R.LQAIEHELHELGLLKDHSLEGR.Y	3	4.98	0.28	
IPI00003919	Glutaminyl-peptide cyclotransferase precursor	R.QIAEGTSISEM*WQNDLQPLLIER.Y	3	3.57	0.16	
IPI00003919	Glutaminyl-peptide cyclotransferase precursor	R.SFSNIISTLNPTAK.R	1	3.24	0.18	
IPI00003919	Glutaminyl-peptide cyclotransferase precursor	R.SFSNIISTLNPTAK.R	2	4.00	0.27	
IPI00003919	Glutaminyl-peptide cyclotransferase precursor	R.SFSNIISTLNPTAKR.H	2	3.11	0.10	
IPI00003919	Glutaminyl-peptide cyclotransferase precursor	R.VFVGATDSAVPCAM*M*LELAR.A	2	3.55	0.32	
IPI00003919	Glutaminyl-peptide cyclotransferase precursor	R.VFVGATDSAVPCAMMLELAR.A	2	5.26	0.34	
IPI00003919	Glutaminyl-peptide cyclotransferase precursor	R.VFVGATDSAVPCAMMLELAR.A	3	3.48	0.10	
IPI00003919	Glutaminyl-peptide cyclotransferase precursor	R.YFQNYSYGGVIQDDHIPFLR.R	2	3.55	0.09	
IPI00003919	Glutaminyl-peptide cyclotransferase precursor	R.YFQNYSYGGVIQDDHIPFLR.R	3	3.50	0.34	
IPI00003919	Glutaminyl-peptide cyclotransferase precursor	R.YFQNYSYGGVIQDDHIPFLRR.G	3	3.85	0.27	
IPI00003921	Isoform 1 of Protein 4.1	C.SFATLALLGSYTIQSELGDYDPELHGVDYVSDFK.L	3	4.31	0.36	-3.15
IPI00003921	Isoform 1 of Protein 4.1	F.ATLALLGSYTIQSELGDYDPELHGVDYVSDFK.L	3	4.53	0.45	-4.74
IPI00003921	Isoform 1 of Protein 4.1	K.AKDLEGVDIILGVCSSGLLVYK.D	3	3.99	0.40	-5.99
IPI00003921	Isoform 1 of Protein 4.1	K.DLEGVDIILGVCSSGLLVYK.D	2	6.05	0.62	-6.47
IPI00003921	Isoform 1 of Protein 4.1	K.DLEGVDIILGVCSSGLLVYK.D	3	5.00	0.60	-5.83
IPI00003921	Isoform 1 of Protein 4.1	K.RVCEHLNLLEEDYFGLAIWDNATSK.T	3	4.52	0.44	-2.96
IPI00003921	Isoform 1 of Protein 4.1	K.RVCEHLNLLEEDYFGLAIWDNATSK.T	4	3.22	0.19	-3.68
IPI00003921	Isoform 1 of Protein 4.1	K.VSLLDDTVYECVVEK.H	2	3.44	0.43	-2.45
IPI00003921	Isoform 1 of Protein 4.1	K.VVVHQETEIADE.-	2	2.29	0.20	-2.82
IPI00003921	Isoform 1 of Protein 4.1	R.LPCSFATLALLGSYTIQSELGDYDPELHGVDYVSDFK.L	3	6.29	0.60	-6.34

IPI00003921	Isoform 1 of Protein 4.1	R.LPCSFATLALLGSYTIQSELGDYDPELHGVDYVSDFK.L	4	5.38	0.48	-4.25
IPI00003921	Isoform 1 of Protein 4.1	R.PTSAPAITQGQVAEGGVLDASAK.K	2	5.13	0.55	-6.65
IPI00003921	Isoform 1 of Protein 4.1	R.SPRPTSAPAITQGQVAEGGVLDASAK.K	3	5.05	0.54	-3.29
IPI00003921	Isoform 1 of Protein 4.1	R.VCEHLNLLEEDYFGLAIWDNATSK.T	2	6.26	0.62	-5.51
IPI00003921	Isoform 1 of Protein 4.1	R.VCEHLNLLEEDYFGLAIWDNATSK.T	3	5.55	0.50	-4.44
IPI00003933	hydroxyacyl glutathione hydrolase isoform 1	R.HVEPGNAAIR.E	2	1.81	0.16	-2.30
IPI00003971	Isoform RTN1-A of Reticulon-1	K.GATPAPQAGEPSPGLGAR.A	2	3.67	0.46	-3.44
IPI00004047	Isoform 1 of Exostosin-2	K.FASVFGTM*PLK.V	2	2.94	0.19	-1.51
IPI00004047	Isoform 1 of Exostosin-2	R.ACLFVPSIDVLNQNTLR.I	2	3.24	0.17	-6.02
IPI00004084	Isoform 2 of Cyclic AMP-dependent transcription factor ATF-6 beta	R.DSVGQLQLYR.H	2	2.70	0.25	-1.09
IPI00004114	Ribonuclease K6 precursor	K.FFIVACDPPQKSDPPYK.L	3	2.35	0.15	-0.18
IPI00004114	Ribonuclease K6 precursor	K.LVPVHLDSIL.-	1	2.31	0.17	-2.25
IPI00004114	Ribonuclease K6 precursor	K.LVPVHLDSIL.-	2	3.12	0.31	-2.10
IPI00004114	Ribonuclease K6 precursor	R.YSAAAQYK.F	1	2.11	0.27	-2.20
IPI00004315	Sialic acid-binding Ig-like lectin 9 precursor	R.EGANTDQDAPVATNNPAR.A	2	4.99	0.53	-3.16
IPI00004315	Sialic acid-binding Ig-like lectin 9 precursor	R.EGANTDQDAPVATNNPAR.A	3	3.56	0.36	-2.17
IPI00004346	C-C chemokine receptor type 10	R.ERSCPASK.R	1	2.04	0.08	
IPI00004367	FXYD domain-containing ion transport regulator 6 precursor	K.EM*DPFHYDYQTLR.I	2	2.74	0.36	-3.34
IPI00004373	Mannose-binding protein C precursor	K.ALQTEM*AR.I	2	2.00	0.12	-1.73
IPI00004409	Discoidin domain-containing receptor 2 precursor	K.QVLDGNSNPYDIFLKDLEPPIVAR.F	3	4.38	0.36	-4.84
IPI00004413	Tumor necrosis factor receptor superfamily member 21 precursor	K.ASNLIGTYR.H	1	2.12	0.19	-2.17
IPI00004413	Tumor necrosis factor receptor superfamily member 21 precursor	K.ASNLIGTYR.H	2	2.75	0.21	-0.83
IPI00004413	Tumor necrosis factor receptor superfamily member 21 precursor	K.AYTDCLSQNLVVIKPGTK.E	2	3.84	0.43	-3.89
IPI00004413	Tumor necrosis factor receptor superfamily member 21 precursor	K.AYTDCLSQNLVVIKPGTK.E	3	2.20	0.13	-1.18
IPI00004413	Tumor necrosis factor receptor superfamily member 21 precursor	K.LPCAALTDR.E	2	2.16	0.11	-0.33
IPI00004413	Tumor necrosis factor receptor superfamily member 21 precursor	R.ATGQVLTCDK.C	2	3.00	0.22	-0.49
IPI00004413	Tumor necrosis factor receptor superfamily member 21 precursor	R.GTFSDVPSSVM*K.C	1	1.83	0.25	-2.90
IPI00004413	Tumor necrosis factor receptor superfamily member 21 precursor	R.GTFSDVPSSVM*K.C	2	3.05	0.16	-3.30
IPI00004413	Tumor necrosis factor receptor superfamily member 21 precursor	R.VCSSCPVGTFTR.H	2	3.66	0.43	-2.69
IPI00004433	Contactin-6 precursor	G.DGLLSRPIFTQEPHDVIFPLDLSK.S	3	4.14	0.37	-3.49
IPI00004433	Contactin-6 precursor	K.ANTIYFASVR.A	1	1.78	0.08	-3.24

IPI00004433	Contactin-6 precursor	K.ANTIYFASVR.A	2	2.62	0.24	-2.97
IPI00004433	Contactin-6 precursor	K.ASVPVVAPVNIHGGGGSR.S	2	2.70	0.40	-3.34
IPI00004433	Contactin-6 precursor	K.ASVPVVAPVNIHGGGGSR.S	3	1.80	0.10	-2.55
IPI00004433	Contactin-6 precursor	K.GQLIFYAPPEWEQK.I	2	4.71	0.48	-4.18
IPI00004433	Contactin-6 precursor	K.LQFAYIEDFETK.T	2	4.28	0.43	-3.29
IPI00004433	Contactin-6 precursor	K.SQAILEIPNFQQEDEGFYECIASNLR.G	3	5.05	0.50	-5.69
IPI00004433	Contactin-6 precursor	K.YQIIYANAELR.V	2	2.92	0.28	-2.68
IPI00004433	Contactin-6 precursor	R.AGPDNNSPIQIFTIQTR.T	2	4.37	0.44	-4.60
IPI00004433	Contactin-6 precursor	R.EGQGVVLLCGPPPHFGDLSYAWTFNDNPLYVQEDNRR.F	4	3.80	0.41	-3.55
IPI00004433	Contactin-6 precursor	R.FPETIQAAK.D	2	2.80	0.22	-2.20
IPI00004433	Contactin-6 precursor	R.FVSQETGNLYIAK.V	2	4.06	0.39	-3.56
IPI00004433	Contactin-6 precursor	R.IFLLEDGSLK.I	2	3.19	0.17	-2.93
IPI00004433	Contactin-6 precursor	R.IGGESVGDLM*IR.N	2	4.01	0.42	-2.97
IPI00004433	Contactin-6 precursor	R.LDGGSLAINSPHTDQDIGM*YQCLATNLLGTILSR.K	3	7.23	0.62	-4.12
IPI00004433	Contactin-6 precursor	R.LDGGSLAINSPHTDQDIGM*YQCLATNLLGTILSR.K	4	3.25	0.09	-3.97
IPI00004433	Contactin-6 precursor	R.LDGSPLPGK.V	2	2.42	0.06	-2.78
IPI00004433	Contactin-6 precursor	R.PIFTQEPHDVIFPLDLSK.S	3	4.11	0.40	-2.32
IPI00004433	Contactin-6 precursor	R.RLDGSPLPGK.V	2	2.45	0.06	-1.50
IPI00004433	Contactin-6 precursor	R.SDAGSYTCIATNQFGTAK.N	2	5.42	0.52	-4.57
IPI00004433	Contactin-6 precursor	R.SVQGPPTPLVQR.T	2	3.38	0.33	-2.65
IPI00004433	Contactin-6 precursor	R.TDGVM*GEYEPK.I	2	3.27	0.22	-3.13
IPI00004433	Contactin-6 precursor	R.TKASVPVVAPVNIHGGGGSR.S	3	3.26	0.40	-3.66
IPI00004433	Contactin-6 precursor	R.TPFSVGWQAVATVPEILNGK.T	2	5.97	0.51	-4.42
IPI00004433	Contactin-6 precursor	R.TPFSVGWQAVATVPEILNGK.T	3	3.21	0.27	-2.39
IPI00004433	Contactin-6 precursor	R.TVSDGGDGSSSEEIR.I	2	3.50	0.45	-3.12
IPI00004433	Contactin-6 precursor	R.TVSDGGDGSSSEEIRIPK.M	2	3.66	0.47	-0.48
IPI00004433	Contactin-6 precursor	R.VLASAPDFSK.S	1	1.38	0.05	-3.77
IPI00004433	Contactin-6 precursor	R.VLASAPDFSK.S	2	2.28	0.13	-1.99
IPI00004433	Contactin-6 precursor	R.VVAGNSIGIGEPSEPSELLR.T	2	4.53	0.45	-2.97
IPI00004433	Contactin-6 precursor	R.VVAGNSIGIGEPSEPSELLR.T	3	3.88	0.24	-2.93
IPI00004440	Receptor-type tyrosine-protein phosphatase-like N precursor	K.AEAPALFSR.T	1	1.55	0.17	-1.77
IPI00004440	Receptor-type tyrosine-protein phosphatase-like N precursor	K.EGLGDRGEKPASPAVQPDAALQR.L	3	3.20	0.24	
IPI00004440	Receptor-type tyrosine-protein phosphatase-like N precursor	K.KTM*EGPVEGR.D	2	2.63	0.16	0.36
IPI00004440	Receptor-type tyrosine-protein phosphatase-like N precursor	K.SELEAQTGLQILQTGVGQR.E	2	4.97	0.42	-2.76
IPI00004440	Receptor-type tyrosine-protein phosphatase-like N precursor	K.SELEAQTGLQILQTGVGQR.E	3	5.39	0.44	-3.06
IPI00004440	Receptor-type tyrosine-protein phosphatase-like N precursor	K.TM*EGPVEGR.D	2	3.08	0.35	-1.09

IPI00004440	Receptor-type tyrosine-protein phosphatase-like N precursor	R.DTAELPAR.T	2	2.23	0.22	-2.42
IPI00004440	Receptor-type tyrosine-protein phosphatase-like N precursor	R.GEKPASPAVQPDAALQR.L	3	3.52	0.23	
IPI00004440	Receptor-type tyrosine-protein phosphatase-like N precursor	R.IRHNEQNLSLADVTQQAGLVK.S	3	3.06	0.19	-3.00
IPI00004440	Receptor-type tyrosine-protein phosphatase-like N precursor	R.LAAVLAGYGVELR.Q	2	4.19	0.38	
IPI00004440	Receptor-type tyrosine-protein phosphatase-like N precursor	R.LPEQGSSSR.A	2	2.51	0.16	-3.26
IPI00004440	Receptor-type tyrosine-protein phosphatase-like N precursor	R.LPQPPVGK.G	2	2.14	0.19	-2.71
IPI00004440	Receptor-type tyrosine-protein phosphatase-like N precursor	R.NPGGVVNVGADIKK.T	2	3.27	0.35	-2.00
IPI00004440	Receptor-type tyrosine-protein phosphatase-like N precursor	R.NPGGVVNVGADIKK.T	3	1.87	0.17	-1.95
IPI00004440	Receptor-type tyrosine-protein phosphatase-like N precursor	R.QLTPEQLSTLLTLLQLLPK.G	2	4.73	0.43	-2.35
IPI00004440	Receptor-type tyrosine-protein phosphatase-like N precursor	R.VSEGSPGM*VSVGPLPK.A	2	3.82	0.48	-4.23
IPI00004457	Membrane copper amine oxidase	R.EALAIVFFGR.Q	2	3.33	0.29	-2.93
IPI00004457	Membrane copper amine oxidase	R.NLVTM*TTAPR.G	2	2.12	0.15	-1.13
IPI00004480	ADAM DEC1 precursor	K.TKHLLGPDYTETLYSPR.G	3	4.10	0.46	-3.26
IPI00004488	Vacuolar proton pump subunit F	K.LIAVIGDEDTVTGFLLGGIGELNK.N	2	5.14	0.44	-3.18
IPI00004488	Vacuolar proton pump subunit F	K.LIAVIGDEDTVTGFLLGGIGELNK.N	3	3.17	0.23	-2.75
IPI00004488	Vacuolar proton pump subunit F	R.DDIGIILINQYIAEMVR.H	2	3.78	0.40	-1.10
IPI00004494	Semaphorin-3E precursor	R.VGYHLEDPLFHLESPR.S	4	3.24	0.23	-2.99
IPI00004494	Semaphorin-3E precursor	R.YYPTGTHAK.R	2	1.67	0.05	-2.37
IPI00004503	lysosomal-associated membrane protein 1	K.AFSVNIFK.V	2	2.81	0.22	-1.31
IPI00004503	lysosomal-associated membrane protein 1	K.TVESITDIR.A	1	2.24	0.21	-3.84
IPI00004503	lysosomal-associated membrane protein 1	K.TVESITDIR.A	2	3.62	0.23	-2.89
IPI00004503	lysosomal-associated membrane protein 1	K.TVESITDIRADIDKK.Y	2	2.91	0.17	-3.44
IPI00004503	lysosomal-associated membrane protein 1	K.TVESITDIRADIDKK.Y	4	2.38	0.15	-1.62
IPI00004503	lysosomal-associated membrane protein 1	R.ALQATVGNSYK.C	1	2.58	0.38	-1.70
IPI00004503	lysosomal-associated membrane protein 1	R.ALQATVGNSYK.C	2	4.13	0.35	-1.53
IPI00004503	lysosomal-associated membrane protein 1	R.FFLQGIQLNTILPDAR.D	2	4.65	0.45	-3.09
IPI00004503	lysosomal-associated membrane protein 1	R.FFLQGIQLNTILPDAR.D	3	3.13	0.19	-2.38
IPI00004503	lysosomal-associated membrane protein 1	R.FFLQGIQLNTILPDARDPAFK.A	2	4.37	0.38	-3.38
IPI00004503	lysosomal-associated membrane protein 1	R.FFLQGIQLNTILPDARDPAFK.A	3	4.21	0.36	-5.07
IPI00004503	lysosomal-associated membrane protein 1	R.FFLQGIQLNTILPDARDPAFK.A	4	2.59	0.15	-1.90
IPI00004533	Kinesin-like protein KIF3B	R.EKDAAEM*LGAK.I	2	1.23	0.06	-1.94
IPI00004534	Phosphoribosylformylglycinamidine synthase	-.MSPVLHFYVRPSGHEGAASGHTRRK.L	3	2.35	0.24	-2.51

IPI00004573	Polymeric immunoglobulin receptor precursor	R.ASVDSGSSEEQGGSSR.A	2	3.79	0.44	-2.65
IPI00004573	Polymeric immunoglobulin receptor precursor	R.ASVDSGSSEEQGGSSRA.L	2	3.29	0.44	-1.25
IPI00004656	Beta-2-microglobulin	C.YVSGFHPSDIEVDLLK.N	2	4.52	0.40	-3.96
IPI00004656	Beta-2-microglobulin	E.KVEHSDLSFSK.D	2	3.79	0.24	-2.38
IPI00004656	Beta-2-microglobulin	F.LNCYVSGFHPSDIEVDLLK.N	2	5.52	0.54	-4.19
IPI00004656	Beta-2-microglobulin	G.FHPSDIEVDLLK.N	3	3.89	0.18	-1.93
IPI00004656	Beta-2-microglobulin	H.PSDIEVDLLK.N	2	3.20	0.26	-0.82
IPI00004656	Beta-2-microglobulin	I.EKVEHSDLSFSK.D	2	3.76	0.38	-3.57
IPI00004656	Beta-2-microglobulin	K.DWSFYLLYYTEFTPTEKDEYACR.V	3	5.95	0.60	-6.73
IPI00004656	Beta-2-microglobulin	K.IQVYSRHPAENGK.S	2	3.34	0.38	-5.05
IPI00004656	Beta-2-microglobulin	K.IQVYSRHPAENGK.S	3	2.14	0.10	-1.96
IPI00004656	Beta-2-microglobulin	K.NGERIEKVEHSDLSFSK.D	2	4.50	0.55	-3.47
IPI00004656	Beta-2-microglobulin	K.NGERIEKVEHSDLSFSK.D	3	3.76	0.34	-3.65
IPI00004656	Beta-2-microglobulin	K.NGERIEKVEHSDLSFSK.D	4	3.36	0.15	-2.28
IPI00004656	Beta-2-microglobulin	K.SNFLNCYVSGFHPSDIEVDLLK.N	2	5.60	0.59	-4.77
IPI00004656	Beta-2-microglobulin	K.SNFLNCYVSGFHPSDIEVDLLK.N	3	4.60	0.48	-7.63
IPI00004656	Beta-2-microglobulin	K.SNFLNCYVSGFHPSDIEVDLLK.N	4	3.98	0.36	-1.94
IPI00004656	Beta-2-microglobulin	K.SNFLNCYVSGFHPSDIEVDLLKNGER.I	3	5.44	0.49	-2.33
IPI00004656	Beta-2-microglobulin	K.SNFLNCYVSGFHPSDIEVDLLKNGER.I	4	3.44	0.15	-4.07
IPI00004656	Beta-2-microglobulin	K.VEHSDLSFSK.D	1	3.58	0.29	-4.20
IPI00004656	Beta-2-microglobulin	K.VEHSDLSFSK.D	2	3.42	0.33	-3.59
IPI00004656	Beta-2-microglobulin	L.NCYVSGFHPSDIEVDLLK.N	2	5.70	0.52	-1.72
IPI00004656	Beta-2-microglobulin	N.CYVSGFHPSDIEVDLLK.N	2	5.68	0.53	-5.77
IPI00004656	Beta-2-microglobulin	N.FLNCYVSGFHPSDIEVDLLK.N	2	5.62	0.56	-3.93
IPI00004656	Beta-2-microglobulin	N.FLNCYVSGFHPSDIEVDLLK.N	3	4.47	0.46	-3.01
IPI00004656	Beta-2-microglobulin	R.IEKVEHSDLSFSK.D	1	4.06	0.38	-4.10
IPI00004656	Beta-2-microglobulin	R.IEKVEHSDLSFSK.D	2	4.85	0.41	-8.48
IPI00004656	Beta-2-microglobulin	R.IEKVEHSDLSFSK.D	3	3.67	0.28	-3.39
IPI00004656	Beta-2-microglobulin	R.TPKIQVYSR.H	2	2.04	0.12	-2.36
IPI00004656	Beta-2-microglobulin	V.SGFHPSDIEVDLLK.N	2	3.84	0.45	-3.12
IPI00004656	Beta-2-microglobulin	Y.VSGFHPSDIEVDLLK.N	2	3.64	0.34	-3.18
IPI00004656	Beta-2-microglobulin	Y.VSGFHPSDIEVDLLK.N	3	4.74	0.38	-2.48
IPI00004669	Polypeptide N-acetylgalactosaminyltransferase 2	K.AQSM*ETLPPGK.V	2	2.06	0.10	-2.58
IPI00004669	Polypeptide N-acetylgalactosaminyltransferase 2	K.EIILVDDYSNDPEDGALLGK.I	2	6.36	0.61	-4.05
IPI00004669	Polypeptide N-acetylgalactosaminyltransferase 2	K.EIILVDDYSNDPEDGALLGK.I	3	4.08	0.42	-1.39
IPI00004669	Polypeptide N-acetylgalactosaminyltransferase 2	K.EIILVDDYSNDPEDGALLGKIEK.V	3	2.84	0.24	-3.69
IPI00004669	Polypeptide N-acetylgalactosaminyltransferase 2	K.FTLNLQQ.-	2	2.04	0.06	-2.82
IPI00004669	Polypeptide N-acetylgalactosaminyltransferase 2	K.GGFDWNLVFK.W	2	3.58	0.30	-2.30
IPI00004669	Polypeptide N-acetylgalactosaminyltransferase 2	K.HM*DLCLTVVDRAPGSLIK.L	3	2.48	0.25	-3.72
IPI00004669	Polypeptide N-acetylgalactosaminyltransferase 2	K.NFYYAAVPSAR.N	2	3.23	0.21	-2.19
IPI00004669	Polypeptide N-acetylgalactosaminyltransferase 2	K.QHPYTFPGGSGTVFAR.N	3	2.71	0.28	-0.80
IPI00004669	Polypeptide N-acetylgalactosaminyltransferase 2	K.WYLENVYPELR.V	2	4.37	0.43	-4.50

IPI00004669	Polypeptide N-acetylgalactosaminyltransferase 2	M.YSGGGSALAGGAGGGAGR.K	2	5.16	0.51	-1.21
IPI00004669	Polypeptide N-acetylgalactosaminyltransferase 2	R.AAEVWM*DEYKNFYYAAVPSAR.N	3	2.48	0.15	-3.47
IPI00004669	Polypeptide N-acetylgalactosaminyltransferase 2	R.NKFNQVESDKLR.M	3	3.62	0.15	-2.34
IPI00004669	Polypeptide N-acetylgalactosaminyltransferase 2	R.QGNPVAPIK.T	2	2.02	0.09	0.33
IPI00004669	Polypeptide N-acetylgalactosaminyltransferase 2	R.SGQDPYAR.N	2	2.50	0.18	-0.71
IPI00004669	Polypeptide N-acetylgalactosaminyltransferase 2	R.TVVSVLKK.S	2	2.26	0.06	-1.73
IPI00004669	Polypeptide N-acetylgalactosaminyltransferase 2	R.VDLPATSVVITFHNEAR.S	3	2.75	0.35	-2.45
IPI00004669	Polypeptide N-acetylgalactosaminyltransferase 2	R.VVSPIIDVINM*DNFQYVGASADLK.G	2	3.88	0.43	-4.57
IPI00004669	Polypeptide N-acetylgalactosaminyltransferase 2	R.VVSPIIDVINM*DNFQYVGASADLK.G	3	5.78	0.51	-4.22
IPI00004669	Polypeptide N-acetylgalactosaminyltransferase 2	R.WPDFNQEAYVGGTM*VR.S	2	4.98	0.53	-3.70
IPI00004669	Polypeptide N-acetylgalactosaminyltransferase 2	Y.SGGGSALAGGAGGGAGR.K	2	3.95	0.29	-2.46
IPI00004798	cDNA FLJ75207	K.YYYVCQYCPAGNWANR.L	2	4.29	0.48	1.57
IPI00004946	chemokine (C-X-C motif) ligand 16	G.HSLAAGPEAGENQK.Q	2	3.43	0.47	-2.91
IPI00004946	chemokine (C-X-C motif) ligand 16	G.NEGSVTGSCYCGK.R	2	3.91	0.52	-1.80
IPI00004946	chemokine (C-X-C motif) ligand 16	H.SLAAGPEAGENQK.Q	2	3.97	0.34	-2.09
IPI00004946	chemokine (C-X-C motif) ligand 16	H.SLAAGPEAGENQKQPEK.N	2	3.77	0.38	-2.09
IPI00004946	chemokine (C-X-C motif) ligand 16	K.RISSDSPPSVQFM*NR.L	3	3.35	0.19	-3.22
IPI00004946	chemokine (C-X-C motif) ligand 16	R.ISSDSPPSVQFM*NR.L	2	3.60	0.37	-3.99
IPI00004946	chemokine (C-X-C motif) ligand 16	S.LAAGPEAGENQK.Q	2	3.46	0.30	-2.04
IPI00004962	Golgi integral membrane protein 4	K.FQSPYEEQLEQQR.L	2	3.69	0.34	-2.46
IPI00004962	Golgi integral membrane protein 4	K.FQSPYEEQLEQQR.L	3	2.94	0.27	-3.00
IPI00004962	Golgi integral membrane protein 4	R.DNQHQDEAEGDPGNRHEPR.E	3	2.60	0.36	-2.73
IPI00004962	Golgi integral membrane protein 4	R.DNQHQDEAEGDPGNRHEPR.E	4	2.60	0.39	-3.04
IPI00004962	Golgi integral membrane protein 4	R.EAANLLEGHAR.A	2	3.12	0.30	-2.34
IPI00004962	Golgi integral membrane protein 4	R.EADPESEADRAAVEDINPA.D	2	4.07	0.40	-2.91
IPI00004962	Golgi integral membrane protein 4	R.LAVQQVEEAQQLR.E	2	4.44	0.31	-3.24
IPI00004962	Golgi integral membrane protein 4	R.LAVQQVEEAQQLREHQEALHQQR.L	3	5.16	0.53	-3.51
IPI00004962	Golgi integral membrane protein 4	R.QAELEEGRPQHQEQLR.Q	3	2.03	0.17	-3.57
IPI00004962	Golgi integral membrane protein 4	R.QQEQQQQQVAR.E	2	2.69	0.27	-3.96
IPI00005038	Ribonuclease UK114	K.APGAIGPYSQAVLVDR.T	2	3.51	0.46	-3.70
IPI00005038	Ribonuclease UK114	K.TTVLLADINDFNTVNEIYK.Q	2	5.85	0.60	-3.92
IPI00005038	Ribonuclease UK114	K.TTVLLADINDFNTVNEIYK.Q	3	5.81	0.53	-3.71
IPI00005107	Niemann-Pick C1 protein precursor	R.QLQTLKDNLQLPLQFLSR.C	3	4.16	0.30	-2.73
IPI00005123	Ephrin-A3 precursor	E.DFEGENPQVPK.L	2	2.96	0.27	-2.66
IPI00005123	Ephrin-A3 precursor	K.INVLEDFEGENPQVPK.L	2	4.47	0.42	-3.19
IPI00005123	Ephrin-A3 precursor	K.INVLEDFEGENPQVPK.L	3	4.30	0.34	-3.33
IPI00005123	Ephrin-A3 precursor	K.INVLEDFEGENPQVPKLEK.S	2	5.10	0.46	-3.59
IPI00005123	Ephrin-A3 precursor	K.INVLEDFEGENPQVPKLEK.S	3	4.78	0.47	-2.64
IPI00005126	Ephrin-B2 precursor	K.FLPGQGLVLYPQIGDKLDIICPK.V	2	4.04	0.44	-2.20
IPI00005126	Ephrin-B2 precursor	K.FLPGQGLVLYPQIGDKLDIICPK.V	3	2.13	0.22	-3.62
IPI00005126	Ephrin-B2 precursor	K.FQEFSPNLWGLEFQK.N	2	5.15	0.41	-3.46
IPI00005126	Ephrin-B2 precursor	K.FQEFSPNLWGLEFQK.N	3	5.53	0.28	-3.83

IPI00005126	Ephrin-B2 precursor	K.TVGQYEYYK.V	1	1.98	0.17	-2.59
IPI00005126	Ephrin-B2 precursor	K.TVGQYEYYK.V	2	3.40	0.40	-1.34
IPI00005126	Ephrin-B2 precursor	K.VGQDASSAGSTR.N	2	3.59	0.35	-2.84
IPI00005126	Ephrin-B2 precursor	K.VYM*VDKDQADR.C	2	3.35	0.39	-2.58
IPI00005126	Ephrin-B2 precursor	K.VYM*VDKDQADR.C	3	2.47	0.24	-2.44
IPI00005128	Isoform 1 of Angiopoietin-2 precursor	R.DAPLEYDDSVQR.L	2	2.86	0.34	-3.61
IPI00005129	Isoform 1 of Secretory carrier-associated membrane protein 1	R.NVPPGLDEYNPFSDSR.T	2	2.06	0.25	-2.35
IPI00005132	Guanine nucleotide-binding protein-like 3-like protein	K.WLDYLRNELPTVAFK.A	3	2.98	0.14	
IPI00005142	Isoform 1 of Basic fibroblast growth factor receptor 1 precursor	A.RPSPTLPEQAQPWGAPVEVESFLVHPGDLLQLR.C	3	6.10	0.58	-3.42
IPI00005142	Isoform 1 of Basic fibroblast growth factor receptor 1 precursor	K.CPSSGTPNPTLR.W	2	2.55	0.06	-0.79
IPI00005142	Isoform 1 of Basic fibroblast growth factor receptor 1 precursor	K.IGPDNLPYVQILK.T	2	3.42	0.31	-4.27
IPI00005142	Isoform 1 of Basic fibroblast growth factor receptor 1 precursor	K.LHAVPAAK.T	1	1.88	0.14	-3.27
IPI00005142	Isoform 1 of Basic fibroblast growth factor receptor 1 precursor	P.SPTLPEQAQPWGAPVEVESFLVHPGDLLQLR.C	3	3.81	0.45	-3.92
IPI00005142	Isoform 1 of Basic fibroblast growth factor receptor 1 precursor	P.WGAPVEVESFLVHPGDLLQLR.C	3	3.75	0.35	-2.88
IPI00005142	Isoform 1 of Basic fibroblast growth factor receptor 1 precursor	R.LRDDVQSINWLR.D	2	3.69	0.27	-2.79
IPI00005142	Isoform 1 of Basic fibroblast growth factor receptor 1 precursor	R.M*PVAPYWTSPEK.M	2	3.40	0.38	-1.81
IPI00005142	Isoform 1 of Basic fibroblast growth factor receptor 1 precursor	W.GAPVEVESFLVHPGDLLQLR.C	2	3.75	0.54	-3.97
IPI00005142	Isoform 1 of Basic fibroblast growth factor receptor 1 precursor	W.GAPVEVESFLVHPGDLLQLR.C	3	3.92	0.49	-3.47
IPI00005153	Isoform Aa of Odorant-binding protein 2a precursor	K.LVGRNPNTNLEALEEFKK.L	3	4.96	0.36	-1.12
IPI00005158	Lon protease homolog, mitochondrial precursor	R.DRMEMINVSGYVAQEKLAIAER.Y	2	1.62	0.06	-2.05
IPI00005159	Actin-related protein 2	R.FEAPEALFQPHLINVEGVGVAELLFNTIQAADIDTR.S	3	4.61	0.43	-3.22
IPI00005159	Actin-related protein 2	R.FEAPEALFQPHLINVEGVGVAELLFNTIQAADIDTR.S	4	3.69	0.20	-3.18
IPI00005222	Ephrin type-B receptor 6 precursor	R.AGLQLNVK.E	2	2.92	0.10	-1.87
IPI00005222	Ephrin type-B receptor 6 precursor	R.ETFTLYYR.Q	2	1.91	0.23	-1.39
IPI00005222	Ephrin type-B receptor 6 precursor	R.LFSYTCPAVLR.S	2	2.91	0.28	-2.87
IPI00005292	Testican-1 precursor	K.DSLGWM*FNK.L	2	2.19	0.15	-2.02
IPI00005292	Testican-1 precursor	K.SLLGAFIPR.C	2	2.89	0.08	-0.57
IPI00005292	Testican-1 precursor	K.VCVTQDYQTALCVSR.K	2	5.17	0.47	-5.46
IPI00005292	Testican-1 precursor	K.YGNELAGSRK.Q	2	1.91	0.23	1.44

IPI00005292	Testican-1 precursor	R.AVTEDDEDEDDDKEDEVGY.I	2	5.17	0.66	-3.84
IPI00005292	Testican-1 precursor	R.AVTEDDEDEDDDKEDEVGYI.W	2	4.85	0.64	-1.95
IPI00005292	Testican-1 precursor	R.CNEEGYYK.A	2	2.13	0.13	-2.28
IPI00005292	Testican-1 precursor	R.FDTSILPICK.D	2	3.18	0.27	-3.82
IPI00005292	Testican-1 precursor	R.NWNPNKPFDQALDPSKDPCLK.V	3	3.28	0.15	-2.58
IPI00005347	Zinc finger Ran-binding domain-containing protein 1	K.QIKNRMKKTDWLFLNACVGVVEGDLAAIEAYKSSGGDIAR.Q	3	2.64	0.18	
IPI00005474	Phospholysine phosphohistidine inorganic pyrophosphate phosphatase	K.ADGYVDNLAEAVDLLLQH.A	2	4.30	0.40	-3.27
IPI00005474	Phospholysine phosphohistidine inorganic pyrophosphate phosphatase	K.ADGYVDNLAEAVDLLLQHA.D	2	4.54	0.48	-3.46
IPI00005474	Phospholysine phosphohistidine inorganic pyrophosphate phosphatase	K.ADGYVDNLAEAVDLLLQHAD.K	2	5.48	0.48	-3.22
IPI00005474	Phospholysine phosphohistidine inorganic pyrophosphate phosphatase	K.SALQAIGVEAHQAVM*IGDDIVGDVGGAQR.C	3	4.05	0.34	-4.96
IPI00005474	Phospholysine phosphohistidine inorganic pyrophosphate phosphatase	K.SALQAIGVEAHQAVM*IGDDIVGDVGGAQR.C	4	2.96	0.15	-2.75
IPI00005474	Phospholysine phosphohistidine inorganic pyrophosphate phosphatase	K.SRAELVGQLQR.L	2	3.28	0.19	-1.90
IPI00005516	Leucine-rich repeat-containing protein 4 precursor	R.GLSEVPQGIPSNTR.Y	2	2.87	0.26	-3.56
IPI00005516	Leucine-rich repeat-containing protein 4 precursor	R.NNPIESIPSYAFNR.V	2	4.73	0.49	-3.34
IPI00005516	Leucine-rich repeat-containing protein 4 precursor	R.NNPIESIPSYAFNR.V	3	2.28	0.13	-1.75
IPI00005516	Leucine-rich repeat-containing protein 4 precursor	R.RGLSEVPQGIPSNTR.Y	2	4.06	0.42	-3.73
IPI00005517	Ephrin-A5 precursor	K.TIGVHDR.V	1	1.53	0.09	-1.61
IPI00005517	Ephrin-A5 precursor	R.VFDVNDKVENSLEPA.D	2	3.17	0.32	-1.17
IPI00005517	Ephrin-A5 precursor	R.YVLYM*VNFDGYSACDHTSK.G	3	2.73	0.22	-3.68
IPI00005531	Isoform 1 of Probable DNA dC->dU-editing enzyme APOBEC-3B	R.DTFYDNFENEPILYGRSYTWLCYEVKIKR.G	3	3.04	0.12	
IPI00005564	Stanniocalcin-1 precursor	K.RNPEAITEVVQLPNHFSNR.Y	3	5.17	0.35	-4.24
IPI00005600	Isoform 1 of Bifunctional heparan sulfate N-deacetylase/N-sulfotransferase 2	R.LFLTDFFR.N	2	3.04	0.21	-1.87
IPI00005605	Isoform 1 of Protein NDRG3	R.THSTSSSLGSGESPFSR.S	2	4.48	0.54	-1.42
IPI00005605	Isoform 1 of Protein NDRG3	R.THSTSSSLGSGESPFSR.S	3	3.33	0.20	-0.03
IPI00005607	Isoform 1 of Deleted in bladder cancer protein 1 precursor	K.LLQSATEAQR.Q	2	3.23	0.19	-1.25
IPI00005614	Isoform Long of Spectrin beta chain, brain 1	R.AQQYYFDAAEAEAWMSEQELYMMSEEK.A	3	4.67	0.54	-0.81
IPI00005652	Isoform 1 of WSC domain-containing protein 2	G.FVGQPAVSGNQANPAAAGGPAEGAELSFLGDM*HLGR.G	3	4.98	0.40	-2.39
IPI00005652	Isoform 1 of WSC domain-containing protein 2	K.TISAYIKM*VDAALKGRNLTGVPDDYYPR.-	3	3.72	0.07	1.94

IPI00005652	Isoform 1 of WSC domain-containing protein 2	L.VFLHSGFVGQPAVSGNQANPAAAGGPAEGAELSFLGDM*HLGR.G	4	4.71	0.38	-2.40
IPI00005652	Isoform 1 of WSC domain-containing protein 2	R.DTGEASSIAR.R	2	3.37	0.31	-2.30
IPI00005668	Aldo-keto reductase family 1 member C2	R.TPALIALR.Y	2	2.87	0.23	-2.11
IPI00005675	NF-kappa-B-repressing factor	R.KMGWTGGGLGKSGEGIR.E	2	1.93	0.06	-5.76
IPI00005690	Matrilin-3 precursor	K.VAIIVTDGRPQDQVNEVAAR.A	3	3.66	0.42	1.51
IPI00005690	Matrilin-3 precursor	R.SVRPLEFTK.V	2	1.88	0.09	-0.25
IPI00005705	Isoform Gamma-1 of Serine/threonine-protein phosphatase PP1-gamma catalytic subunit	K.IKYPENFFLLR.G	3	3.27	0.34	-4.19
IPI00005707	Macrophage mannose receptor 2 precursor	K.CFQVQGQEPQSR.V	2	3.63	0.46	-3.53
IPI00005707	Macrophage mannose receptor 2 precursor	K.QIKQEVEELWIGLNDLK.L	3	2.53	0.06	-3.30
IPI00005707	Macrophage mannose receptor 2 precursor	K.SWVQAQGACQELGAQLLSLASYEEEHFVANM*LNK.I	3	4.34	0.40	-3.36
IPI00005707	Macrophage mannose receptor 2 precursor	R.CLTALPYICK.R	2	2.96	0.23	-2.90
IPI00005707	Macrophage mannose receptor 2 precursor	R.DCSIALPYVCK.K	2	2.46	0.24	-2.09
IPI00005707	Macrophage mannose receptor 2 precursor	R.GTDVREPDDSPQGR.R	2	2.09	0.05	-1.07
IPI00005707	Macrophage mannose receptor 2 precursor	R.GTDVREPDDSPQGR.R	3	3.54	0.31	0.21
IPI00005707	Macrophage mannose receptor 2 precursor	R.TLGDQLSLLLGAR.T	2	4.32	0.39	-3.81
IPI00005707	Macrophage mannose receptor 2 precursor	R.TPLWIGLAGEEGSRR.Y	3	2.34	0.21	-2.83
IPI00005719	Isoform 1 of Ras-related protein Rab-1A	R.M*GPGATAGGAEK.S	2	2.57	0.23	-2.29
IPI00005722	Tyrosine-protein kinase receptor	K.M*FTLNIR.R	2	1.78	0.08	-1.22
IPI00005722	Tyrosine-protein kinase receptor	K.VGEPLWIR.C	2	2.34	0.05	-2.57
IPI00005722	Tyrosine-protein kinase receptor	R.LFTIDLNQTPQTTLPQLFLK.V	3	5.18	0.33	-4.57
IPI00005732	Isoform 1 of Activin receptor type-1B precursor	K.VELVPAGKPFYCLSSEDLR.N	2	2.13	0.17	-4.24
IPI00005732	Isoform 1 of Activin receptor type-1B precursor	K.VELVPAGKPFYCLSSEDLR.N	3	2.67	0.37	-2.27
IPI00005774	Isoform 1 of Low-density lipoprotein receptor-related protein 8 precursor	K.SPSLIFTNR.H	2	2.75	0.18	-0.77
IPI00005774	Isoform 1 of Low-density lipoprotein receptor-related protein 8 precursor	K.TLISSTDFLSHPFGIAVFEDK.V	2	5.61	0.51	-4.08
IPI00005774	Isoform 1 of Low-density lipoprotein receptor-related protein 8 precursor	K.TLISSTDFLSHPFGIAVFEDK.V	3	2.40	0.17	-5.10
IPI00005776	Nucleotide-binding oligomerization domain-containing protein 1	R.CIYETQSQKVGQLAAR.G	2	2.90	0.13	-4.34
IPI00005794	60 kDa protein	F.SIYSPHTGIQEYQDGVPK.I	2	4.02	0.47	-3.01
IPI00005794	60 kDa protein	F.SIYSPHTGIQEYQDGVPK.I	3	4.01	0.32	-2.97
IPI00005794	60 kDa protein	K.AIINLAVYGK.A	1	2.25	0.34	-1.32
IPI00005794	60 kDa protein	K.AIINLAVYGK.A	2	3.51	0.20	-1.44
IPI00005794	60 kDa protein	K.AIQIM*YQNLQQDGLEK.V	2	5.98	0.49	-4.67
IPI00005794	60 kDa protein	K.AIQIM*YQNLQQDGLEK.V	3	4.69	0.37	-5.00
IPI00005794	60 kDa protein	K.AIQIM*YQNLQQDGLEKVHLEPVR.I	4	2.97	0.15	-3.02
IPI00005794	60 kDa protein	K.AIQIMYQNLQQDGLEK.V	2	4.48	0.20	
IPI00005794	60 kDa protein	K.IPTACITVEDAEM*M*SR.M	2	5.34	0.51	-4.42
IPI00005794	60 kDa protein	K.IPTACITVEDAEM*M*SR.M	3	4.32	0.38	-2.88
IPI00005794	60 kDa protein	K.IVVYNQPYINYSR.T	2	3.65	0.36	-2.86

IPI00005794	60 kDa protein	K.TYPDTDSFNTVAEITGSK.Y	2	4.80	0.55	-3.41
IPI00005794	60 kDa protein	K.VGALASLIR.S	2	3.62	0.16	-2.72
IPI00005794	60 kDa protein	K.VHLEPVR.I	1	2.07	0.16	-4.33
IPI00005794	60 kDa protein	K.VHLEPVR.I	2	1.97	0.13	-4.71
IPI00005794	60 kDa protein	R.GEESAVM*LEPR.I	2	3.84	0.39	-2.86
IPI00005794	60 kDa protein	R.GKIVVYNQPYINYSR.T	2	4.53	0.50	-4.12
IPI00005794	60 kDa protein	R.GKIVVYNQPYINYSR.T	3	4.23	0.42	-3.36
IPI00005794	60 kDa protein	R.LALLVDTVGPR.L	1	2.38	0.42	-2.76
IPI00005794	60 kDa protein	R.LALLVDTVGPR.L	2	3.98	0.31	-3.44
IPI00005794	60 kDa protein	R.SVASFSIYSPHTGIQEYQDGVPK.I	2	3.79	0.43	-8.19
IPI00005794	60 kDa protein	R.SVASFSIYSPHTGIQEYQDGVPK.I	3	4.38	0.43	-2.95
IPI00005809	Serum deprivation-response protein	A.SALVEGEIAEEAAEK.A	2	5.32	0.43	-2.76
IPI00005809	Serum deprivation-response protein	K.ERMDRQCAQVKRLENNHAQLLR.R	2	2.15	0.17	
IPI00005809	Serum deprivation-response protein	S.ALVEGEIAEEAAEK.A	2	4.61	0.48	-3.10
IPI00005837	Angiopoietin-related protein 1 precursor	R.DNSLELSQLENK.I	2	2.90	0.20	
IPI00005859	Keratin, type II cytoskeletal 75	R.VSINGCGSSCRSGFGGR.A	2	2.15	0.21	-5.89
IPI00005908	ADAMTS-1 precursor	A.LGRPSEEDEELVVPELER.A	2	5.47	0.48	-2.95
IPI00005908	ADAMTS-1 precursor	A.LGRPSEEDEELVVPELER.A	3	4.29	0.18	-2.77
IPI00005908	ADAMTS-1 precursor	A.LGRPSEEDEELVVPELERAPGHGTTR.L	3	4.94	0.55	-4.52
IPI00005908	ADAMTS-1 precursor	K.SGSETPLPETDLAHCFYSGTVNGDPSSAAALSLCEGVR.G	3	6.17	0.57	-3.01
IPI00005908	ADAMTS-1 precursor	R.GAFYLLGEAYFIQPLPAASER.L	2	5.22	0.57	-5.89
IPI00005908	ADAMTS-1 precursor	R.GAFYLLGEAYFIQPLPAASER.L	3	5.65	0.47	-5.83
IPI00005908	ADAMTS-1 precursor	R.KSGSETPLPETDLAHCFYSGTVNGDPSSAAALSLCEGVR.G	3	6.92	0.47	
IPI00005908	ADAMTS-1 precursor	R.LHAFDQQLDLELRPDSSFLAPGFTLQNVGR.K	3	6.17	0.52	-5.50
IPI00005908	ADAMTS-1 precursor	R.LHAFDQQLDLELRPDSSFLAPGFTLQNVGRK.S	3	6.23	0.61	-5.44
IPI00005908	ADAMTS-1 precursor	R.LHAFDQQLDLELRPDSSFLAPGFTLQNVGRK.S	4	4.20	0.45	-5.46
IPI00005969	F-actin-capping protein subunit alpha-1	K.DVQDSLTVSNEAQTAK.E	2	2.85	0.28	-4.30
IPI00005981	Transgelin-3	K.GASQAGM*TGYGM*PR.Q	2	3.55	0.56	-1.72
IPI00005981	Transgelin-3	K.LVDWIILQCAEDIEHPPPGR.A	3	4.66	0.37	-2.21
IPI00005981	Transgelin-3	K.YDADLENK.L	2	2.20	0.10	-2.70
IPI00005981	Transgelin-3	R.RGFSEEQLR.Q	2	2.08	0.07	-2.02
IPI00005981	Transgelin-3	R.TLM*ALGSVAVTK.D	2	2.80	0.26	-3.54
IPI00006009	Isoform 2 of Pleckstrin homology domain-containing family B member 1	K.TALLEANSTPVR.V	2	2.87	0.31	-3.41
IPI00006009	Isoform 2 of Pleckstrin homology domain-containing family B member 1	R.SRDGLLTVNLR.E	2	2.50	0.22	-2.37
IPI00006034	Cysteine-rich protein 2	K.GVNIGGAGSYIYEKPLAEGPQVTGPIEVPAAR.A	3	6.83	0.53	-1.82
IPI00006034	Cysteine-rich protein 2	K.GVNTGAVGSYIYDRDPEGK.V	3	2.59	0.10	-1.99
IPI00006054	Syntaphilin	K.EDGTGESAGGSPAR.S	3	2.99	0.26	
IPI00006094	Regulating synaptic membrane exocytosis protein 3	R.LGAESQFSDFLDGLGPAQIVGR.Q	2	2.24	0.12	
IPI00006114	Pigment epithelium-derived factor precursor	A.AAVSNFGYDLYR.V	1	2.42	0.40	-3.47

IPI00006114	Pigment epithelium-derived factor precursor	A.AAVSNFGYDLYR.V	2	4.00	0.43	-3.38
IPI00006114	Pigment epithelium-derived factor precursor	A.GFEWNEDGAGTTPSPGLQPAHLTFPLDYHLNQPFIFVLR.D	4	5.08	0.43	-3.07
IPI00006114	Pigment epithelium-derived factor precursor	A.LYYDLISSPDIHGTYKELLDTVTAPQK.N	3	6.11	0.55	-3.17
IPI00006114	Pigment epithelium-derived factor precursor	A.SPPEEGSPDPDSTGALVEEEDPFFKVPVNK.L	3	4.23	0.51	-1.07
IPI00006114	Pigment epithelium-derived factor precursor	D.PDSTGALVEEEDPFFKVPVNK.L	3	4.36	0.44	-1.31
IPI00006114	Pigment epithelium-derived factor precursor	D.PFFKVPVNK.L	2	2.93	0.16	-0.63
IPI00006114	Pigment epithelium-derived factor precursor	D.TDTGALLFIGK.I	2	3.79	0.30	-3.08
IPI00006114	Pigment epithelium-derived factor precursor	E.IPDEISILLLGVAHFK.G	2	3.94	0.45	-3.49
IPI00006114	Pigment epithelium-derived factor precursor	E.IPDEISILLLGVAHFK.G	3	5.26	0.51	-4.02
IPI00006114	Pigment epithelium-derived factor precursor	I.PDEISILLLGVAHFK.G	2	5.29	0.58	-5.35
IPI00006114	Pigment epithelium-derived factor precursor	I.PDEISILLLGVAHFK.G	3	6.15	0.45	-3.73
IPI00006114	Pigment epithelium-derived factor precursor	I.SSPDIHGTYK.E	1	2.38	0.40	-2.23
IPI00006114	Pigment epithelium-derived factor precursor	I.SSPDIHGTYKELLDTVTAPQK.N	2	4.87	0.48	-5.25
IPI00006114	Pigment epithelium-derived factor precursor	K.EIPDEISILLLGVAHFK.G	2	5.69	0.50	-6.04
IPI00006114	Pigment epithelium-derived factor precursor	K.EIPDEISILLLGVAHFK.G	3	1.71	0.21	-4.56
IPI00006114	Pigment epithelium-derived factor precursor	K.EIPDEISILLLGVAHFKG.Q	2	2.90	0.36	-5.58
IPI00006114	Pigment epithelium-derived factor precursor	K.ELLDTVTAPQK.N	1	2.52	0.22	-3.50
IPI00006114	Pigment epithelium-derived factor precursor	K.ELLDTVTAPQK.N	2	3.61	0.32	-3.07
IPI00006114	Pigment epithelium-derived factor precursor	K.IAQLPLTGSM*SIIFFLPLK.V	2	6.12	0.55	-5.63
IPI00006114	Pigment epithelium-derived factor precursor	K.IAQLPLTGSM*SIIFFLPLK.V	3	5.23	0.38	-6.60
IPI00006114	Pigment epithelium-derived factor precursor	K.IAQLPLTGSMSIIFFLPLK.V	2	6.19	0.55	-3.70
IPI00006114	Pigment epithelium-derived factor precursor	K.IAQLPLTGSMSIIFFLPLK.V	3	3.66	0.29	-3.24
IPI00006114	Pigment epithelium-derived factor precursor	K.ITGKPIKLTQVEHR.A	2	3.89	0.33	-4.20
IPI00006114	Pigment epithelium-derived factor precursor	K.ITGKPIKLTQVEHR.A	3	3.00	0.27	-3.43
IPI00006114	Pigment epithelium-derived factor precursor	K.ITGKPIKLTQVEHR.A	4	2.49	0.19	-5.10
IPI00006114	Pigment epithelium-derived factor precursor	K.LAAAVSNFGYDL.Y	1	2.36	0.22	-2.21
IPI00006114	Pigment epithelium-derived factor precursor	K.LAAAVSNFGYDLYR.V	1	3.35	0.57	-2.78
IPI00006114	Pigment epithelium-derived factor precursor	K.LAAAVSNFGYDLYR.V	2	4.82	0.57	-8.47
IPI00006114	Pigment epithelium-derived factor precursor	K.LAAAVSNFGYDLYR.V	3	5.53	0.53	-1.91
IPI00006114	Pigment epithelium-derived factor precursor	K.LKLSYEGEVTK.S	1	2.83	0.31	-3.52
IPI00006114	Pigment epithelium-derived factor precursor	K.LKLSYEGEVTK.S	2	3.96	0.43	-7.04
IPI00006114	Pigment epithelium-derived factor precursor	K.LKLSYEGEVTK.S	3	4.20	0.32	-5.10
IPI00006114	Pigment epithelium-derived factor precursor	K.LQSLFDSPDFSK.I	1	2.94	0.48	-4.30
IPI00006114	Pigment epithelium-derived factor precursor	K.LQSLFDSPDFSK.I	2	4.06	0.42	-5.19
IPI00006114	Pigment epithelium-derived factor precursor	K.LQSLFDSPDFSKITGKPIK.L	3	2.59	0.08	-3.33
IPI00006114	Pigment epithelium-derived factor precursor	K.LQSLFDSPDFSKITGKPIK.L	4	3.59	0.29	-3.05
IPI00006114	Pigment epithelium-derived factor precursor	K.LSYEGEVTK.S	1	2.17	0.12	-4.17
IPI00006114	Pigment epithelium-derived factor precursor	K.LSYEGEVTK.S	2	2.84	0.29	-2.14
IPI00006114	Pigment epithelium-derived factor precursor	K.SLQEM*KLQSLFDSPDFSK.I	2	4.18	0.44	-2.61
IPI00006114	Pigment epithelium-derived factor precursor	K.SLQEM*KLQSLFDSPDFSK.I	3	4.34	0.43	-2.87
IPI00006114	Pigment epithelium-derived factor precursor	K.SSFVAPLEK.S	1	2.41	0.31	-3.80
IPI00006114	Pigment epithelium-derived factor precursor	K.SSFVAPLEK.S	2	2.51	0.14	-1.73

IPI00006114	Pigment epithelium-derived factor precursor	K.TSLEDFYLDEER.T	1	1.71	0.37	-3.08
IPI00006114	Pigment epithelium-derived factor precursor	K.TSLEDFYLDEER.T	2	4.45	0.48	-4.30
IPI00006114	Pigment epithelium-derived factor precursor	K.TSLEDFYLDEERTVR.V	3	2.63	0.22	-2.48
IPI00006114	Pigment epithelium-derived factor precursor	K.TVQAVLTVPK.L	1	1.59	0.13	0.02
IPI00006114	Pigment epithelium-derived factor precursor	K.TVQAVLTVPK.L	2	3.59	0.37	-3.83
IPI00006114	Pigment epithelium-derived factor precursor	K.VTQNLTLIEESLTSEFIHDIDRELK.T	4	3.44	0.12	-4.69
IPI00006114	Pigment epithelium-derived factor precursor	L.LDTVTAPQK.N	1	1.84	0.22	-3.23
IPI00006114	Pigment epithelium-derived factor precursor	L.LSPLSVATALSALSLGAEQR.T	2	4.24	0.34	-2.17
IPI00006114	Pigment epithelium-derived factor precursor	L.LSPLSVATALSALSLGAEQR.T	3	3.61	0.28	-3.74
IPI00006114	Pigment epithelium-derived factor precursor	L.SALSLGAEQR.T	1	1.92	0.24	0.28
IPI00006114	Pigment epithelium-derived factor precursor	L.SVATALSALSLGAEQR.T	2	4.22	0.24	-4.66
IPI00006114	Pigment epithelium-derived factor precursor	L.TGSM*SIIFFLPLK.V	2	3.30	0.30	-4.28
IPI00006114	Pigment epithelium-derived factor precursor	L.YYDLISSPDIHGTYK.E	2	5.08	0.55	-2.87
IPI00006114	Pigment epithelium-derived factor precursor	L.YYDLISSPDIHGTYKELLDTVTAPQK.N	3	4.77	0.49	-1.92
IPI00006114	Pigment epithelium-derived factor precursor	M.KLQSLFDSPDFSK.I	2	4.13	0.44	-3.38
IPI00006114	Pigment epithelium-derived factor precursor	M.SIIFFLPLK.V	2	3.12	0.22	-2.59
IPI00006114	Pigment epithelium-derived factor precursor	M.SPTTNVLLSPLSVATALSALSLGAEQR.T	3	5.04	0.37	-3.34
IPI00006114	Pigment epithelium-derived factor precursor	N.PASPPEEGSPDPDSTGALVEEEDPFFKVPVNK.L	3	4.68	0.58	-3.44
IPI00006114	Pigment epithelium-derived factor precursor	N.VLLSPLSVATALSALSLGAEQR.T	2	6.03	0.60	-3.59
IPI00006114	Pigment epithelium-derived factor precursor	N.VLLSPLSVATALSALSLGAEQR.T	3	5.13	0.53	-4.62
IPI00006114	Pigment epithelium-derived factor precursor	Q.SLFDSPDFSK.I	1	2.35	0.25	-3.34
IPI00006114	Pigment epithelium-derived factor precursor	R.AGFEWNEDGAGTTPSPGLQPAHLTFPLDYHLNQPF.I	3	5.31	0.49	-3.73
IPI00006114	Pigment epithelium-derived factor precursor	R.AGFEWNEDGAGTTPSPGLQPAHLTFPLDYHLNQPFIFVLR.D	3	7.10	0.63	-4.17
IPI00006114	Pigment epithelium-derived factor precursor	R.AGFEWNEDGAGTTPSPGLQPAHLTFPLDYHLNQPFIFVLR.D	4	6.33	0.48	-6.46
IPI00006114	Pigment epithelium-derived factor precursor	R.ALYYDLISSPDIHGTYK.E	2	5.98	0.52	-4.25
IPI00006114	Pigment epithelium-derived factor precursor	R.ALYYDLISSPDIHGTYK.E	3	3.26	0.43	-5.66
IPI00006114	Pigment epithelium-derived factor precursor	R.ALYYDLISSPDIHGTYKELLDTVTAPQK.N	2	3.93	0.57	-4.50
IPI00006114	Pigment epithelium-derived factor precursor	R.ALYYDLISSPDIHGTYKELLDTVTAPQK.N	3	7.04	0.60	-6.20
IPI00006114	Pigment epithelium-derived factor precursor	R.ALYYDLISSPDIHGTYKELLDTVTAPQK.N	4	4.85	0.54	-5.37
IPI00006114	Pigment epithelium-derived factor precursor	R.ALYYDLISSPDIHGTYKELLDTVTAPQKNLK.S	3	5.46	0.50	-3.68
IPI00006114	Pigment epithelium-derived factor precursor	R.ALYYDLISSPDIHGTYKELLDTVTAPQKNLK.S	4	4.40	0.35	-4.28
IPI00006114	Pigment epithelium-derived factor precursor	R.DTDTGALLFIGK.I	1	3.01	0.31	-6.00
IPI00006114	Pigment epithelium-derived factor precursor	R.DTDTGALLFIGK.I	2	4.35	0.42	-4.50
IPI00006114	Pigment epithelium-derived factor precursor	R.DTDTGALLFIGK.I	3	3.45	0.08	-3.45
IPI00006114	Pigment epithelium-derived factor precursor	R.DTDTGALLFIGKILDPR.G	3	3.34	0.33	-4.32
IPI00006114	Pigment epithelium-derived factor precursor	R.DTDTGALLFIGKILDPRGP.-	3	5.01	0.42	-3.68
IPI00006114	Pigment epithelium-derived factor precursor	R.ELKTVQAVLTVPK.L	2	4.10	0.35	-3.59
IPI00006114	Pigment epithelium-derived factor precursor	R.IKSSFVAPLEK.S	1	2.72	0.27	-3.57
IPI00006114	Pigment epithelium-derived factor precursor	R.IKSSFVAPLEK.S	2	3.56	0.32	-3.61
IPI00006114	Pigment epithelium-derived factor precursor	R.IKSSFVAPLEK.S	3	3.60	0.27	-4.48
IPI00006114	Pigment epithelium-derived factor precursor	R.KTSLEDFYLDEER.T	1	3.58	0.50	-2.73
IPI00006114	Pigment epithelium-derived factor precursor	R.KTSLEDFYLDEER.T	2	4.61	0.47	-5.64

IPI00006114	Pigment epithelium-derived factor precursor	R.KTSLEDFYLDEER.T	3	4.17	0.35	-2.91
IPI00006114	Pigment epithelium-derived factor precursor	R.KTSLEDFYLDEERTVR.V	2	3.85	0.38	-3.47
IPI00006114	Pigment epithelium-derived factor precursor	R.LDLQEINNWVQAQM*K.G	2	5.37	0.51	-5.08
IPI00006114	Pigment epithelium-derived factor precursor	R.LDLQEINNWVQAQM*K.G	3	5.22	0.43	-3.90
IPI00006114	Pigment epithelium-derived factor precursor	R.LDLQEINNWVQAQMK.G	2	4.40	0.43	-3.11
IPI00006114	Pigment epithelium-derived factor precursor	R.LDLQEINNWVQAQMK.G	3	2.71	0.15	-3.14
IPI00006114	Pigment epithelium-derived factor precursor	R.SSM*SPTTNVLLSPLSVATALSALSLGAEQR.T	2	5.79	0.61	-4.54
IPI00006114	Pigment epithelium-derived factor precursor	R.SSM*SPTTNVLLSPLSVATALSALSLGAEQR.T	3	5.95	0.53	-7.88
IPI00006114	Pigment epithelium-derived factor precursor	R.SSM*SPTTNVLLSPLSVATALSALSLGAEQR.T	4	5.04	0.48	-4.38
IPI00006114	Pigment epithelium-derived factor precursor	R.SSM*SPTTNVLLSPLSVATALSALSLGAEQRTESIIHR.A	4	5.58	0.43	-3.15
IPI00006114	Pigment epithelium-derived factor precursor	R.STKEIPDEISILLLGVAHFK.G	2	4.59	0.49	-5.94
IPI00006114	Pigment epithelium-derived factor precursor	R.STKEIPDEISILLLGVAHFK.G	3	6.37	0.48	-6.65
IPI00006114	Pigment epithelium-derived factor precursor	R.STKEIPDEISILLLGVAHFK.G	4	4.62	0.39	-4.06
IPI00006114	Pigment epithelium-derived factor precursor	R.TESIIHR.A	2	2.42	0.16	-4.99
IPI00006114	Pigment epithelium-derived factor precursor	R.TVRVPM*M*SDPK.A	3	1.89	0.11	-1.64
IPI00006114	Pigment epithelium-derived factor precursor	R.VLTGNPR.L	1	1.91	0.17	-2.78
IPI00006114	Pigment epithelium-derived factor precursor	R.VRSSM*SPTTNVLLSPLSVATALSALSLGAEQR.T	3	4.14	0.33	-4.14
IPI00006114	Pigment epithelium-derived factor precursor	R.YGLDSDLSCK.I	1	2.77	0.44	-3.90
IPI00006114	Pigment epithelium-derived factor precursor	R.YGLDSDLSCK.I	2	3.26	0.46	-2.41
IPI00006114	Pigment epithelium-derived factor precursor	S.PGLQPAHLTFPLDYHLNQPFIFVLR.D	3	5.56	0.52	-3.26
IPI00006114	Pigment epithelium-derived factor precursor	S.PLSVATALSALSLGAEQR.T	2	3.42	0.39	-2.88
IPI00006114	Pigment epithelium-derived factor precursor	S.PPEEGSPDPDSTGALVEEEDPFFKVPVNK.L	2	3.82	0.42	0.03
IPI00006114	Pigment epithelium-derived factor precursor	S.PPEEGSPDPDSTGALVEEEDPFFKVPVNK.L	3	6.12	0.49	-2.05
IPI00006114	Pigment epithelium-derived factor precursor	T.PSPGLQPAHLTFPLDYHLNQPFIFVLR.D	3	5.45	0.52	-4.01
IPI00006114	Pigment epithelium-derived factor precursor	V.LLSPLSVATALSALSLGAEQR.T	2	5.47	0.52	-2.87
IPI00006114	Pigment epithelium-derived factor precursor	V.QAVLTVPK.L	1	1.90	0.22	0.63
IPI00006114	Pigment epithelium-derived factor precursor	V.SNFGYDLYR.V	1	1.82	0.17	-2.40
IPI00006114	Pigment epithelium-derived factor precursor	W.NEDGAGTTPSPGLQPAHLTFPLDYHLNQPFIFVLR.D	3	3.86	0.33	-5.48
IPI00006114	Pigment epithelium-derived factor precursor	W.NEDGAGTTPSPGLQPAHLTFPLDYHLNQPFIFVLR.D	4	4.77	0.49	-2.62
IPI00006114	Pigment epithelium-derived factor precursor	Y.DLISSPDIHGTYK.E	2	2.94	0.24	-1.36
IPI00006114	Pigment epithelium-derived factor precursor	Y.DLISSPDIHGTYKELLDTVTAPQK.N	3	3.81	0.32	0.47
IPI00006114	Pigment epithelium-derived factor precursor	Y.YDLISSPDIHGTYK.E	2	4.13	0.48	-2.00
IPI00006114	Pigment epithelium-derived factor precursor	Y.YDLISSPDIHGTYKELLDTVTAPQK.N	3	4.59	0.47	-3.99
IPI00006128	Testican-2 precursor	E.DEQWLSSISQYSGK.I	2	4.42	0.52	-4.98
IPI00006128	Testican-2 precursor	K.DSIGWM*FSK.L	2	2.78	0.25	-1.50
IPI00006128	Testican-2 precursor	K.EGETPGNFM*EDEQWLSSISQYSGK.I	2	4.51	0.63	-4.58
IPI00006128	Testican-2 precursor	K.EGETPGNFM*EDEQWLSSISQYSGK.I	3	4.94	0.51	-5.40
IPI00006128	Testican-2 precursor	K.GLKEGETPGNFM*EDEQWLSSISQYSGK.I	2	4.90	0.59	-2.77
IPI00006128	Testican-2 precursor	K.GLKEGETPGNFM*EDEQWLSSISQYSGK.I	3	5.55	0.59	-3.38
IPI00006128	Testican-2 precursor	K.LEQQACLSSK.Q	2	3.47	0.38	-1.43
IPI00006128	Testican-2 precursor	R.FRDEVEDDYIK.S	2	2.83	0.18	-3.40
IPI00006128	Testican-2 precursor	R.FRDEVEDDYIK.S	3	3.33	0.10	-2.59

IPI00006128	Testican-2 precursor	R.FRDEVEDDYIKSWEDNQQG.D	2	5.79	0.55	-3.19
IPI00006130	Uncharacterized calcium-binding protein KIAA0494	K.FSQFLGDPVEK.A	2	2.52	0.17	
IPI00006130	Uncharacterized calcium-binding protein KIAA0494	K.IQSIKKEDSSNSQVSK.L	2	4.77	0.53	-4.00
IPI00006130	Uncharacterized calcium-binding protein KIAA0494	K.IQSIKKEDSSNSQVSK.L	3	3.24	0.22	-3.04
IPI00006130	Uncharacterized calcium-binding protein KIAA0494	K.IQSIKKEDSSNSQVSK.L	4	3.01	0.06	-3.25
IPI00006130	Uncharacterized calcium-binding protein KIAA0494	R.AFDSDGDGRYSFLELR.V	3	3.17	0.30	-1.12
IPI00006146	serum amyloid A2	R.SFFSFLGEAFDGAR.D	2	4.87	0.45	
IPI00006154	Isoform Long of Complement factor H-related protein 2 precursor	K.CLDPCVISQEIM*EK.Y	2	2.83	0.08	
IPI00006154	Isoform Long of Complement factor H-related protein 2 precursor	K.INHGILYDEEK.Y	2	3.57	0.37	-3.89
IPI00006154	Isoform Long of Complement factor H-related protein 2 precursor	K.YKPFSQVPTGEVFYYSCEYNFVSPSK.S	3	4.07	0.27	
IPI00006154	Isoform Long of Complement factor H-related protein 2 precursor	R.LCFFPFVENGHSESSGQTHLEGDTVQIICNTGYR.L	3	5.60	0.33	
IPI00006154	Isoform Long of Complement factor H-related protein 2 precursor	R.LQNNENNISCVER.G	2	3.88	0.44	-0.87
IPI00006154	Isoform Long of Complement factor H-related protein 2 precursor	R.TGDIVEFVCK.S	2	3.16	0.41	-2.96
IPI00006166	Probable G-protein coupled receptor 37 precursor	A.FLAGPSWDLPAAPGRDPAAGR.G	3	3.64	0.22	-1.80
IPI00006166	Probable G-protein coupled receptor 37 precursor	K.TVPGASDLFYWPR.R	2	3.16	0.25	-4.28
IPI00006166	Probable G-protein coupled receptor 37 precursor	L.FLQISEEEEKGPR.G	2	4.36	0.42	-2.47
IPI00006166	Probable G-protein coupled receptor 37 precursor	R.EEQGAAFLAGPSWDLPAAPGRDPAAGR.G	3	4.83	0.55	-4.91
IPI00006166	Probable G-protein coupled receptor 37 precursor	R.GQEPSETLGR.G	1	1.62	0.11	-7.79
IPI00006166	Probable G-protein coupled receptor 37 precursor	R.GQEPSETLGR.G	2	3.21	0.32	-4.67
IPI00006252	Multisynthetase complex auxiliary component p43	N.NDAVLKRLEQKGAEADQIIEYLK.Q	3	3.74	0.12	-0.72
IPI00006444	Isoform 1 of Sodium/potassium/calcium exchanger 2 precursor	A.FSETDTQSTGEASVVSGPR.V	2	6.14	0.61	-2.77
IPI00006444	Isoform 1 of Sodium/potassium/calcium exchanger 2 precursor	F.SETDTQSTGEASVVSGPR.V	2	5.25	0.52	-2.40
IPI00006444	Isoform 1 of Sodium/potassium/calcium exchanger 2 precursor	S.ETDTQSTGEASVVSGPR.V	2	5.09	0.54	-3.14
IPI00006451	Vesicle-fusing ATPase	K.AENSSLNLIGK.A	2	2.84	0.29	-2.57
IPI00006451	Vesicle-fusing ATPase	K.DIEAM*DPSILK.G	2	3.32	0.20	-3.31
IPI00006451	Vesicle-fusing ATPase	K.THPSVVPGSIAFSLPQR.K	3	2.44	0.20	-2.85
IPI00006451	Vesicle-fusing ATPase	R.YTFTLK.T	1	1.77	0.09	-1.40
IPI00006470	Neuron-specific protein family member 2	K.GTKPPSVEDGFQTVPLITPLEVNHLQ.L	3	3.96	0.34	-2.59

IPI00006482	Isoform Long of Sodium/potassium-transporting ATPase subunit alpha-1 precursor	K.EQPLDEELKDAFQNAYLELGGLGER.V	3	4.16	0.36	-3.90
IPI00006482	Isoform Long of Sodium/potassium-transporting ATPase subunit alpha-1 precursor	K.LSLDELHRK.Y	2	2.21	0.11	-3.15
IPI00006482	Isoform Long of Sodium/potassium-transporting ATPase subunit alpha-1 precursor	K.QAADMILLDDNFASIVTGVEEGR.L	2	5.62	0.61	-2.21
IPI00006482	Isoform Long of Sodium/potassium-transporting ATPase subunit alpha-1 precursor	K.QAADMILLDDNFASIVTGVEEGR.L	3	4.80	0.48	-1.61
IPI00006482	Isoform Long of Sodium/potassium-transporting ATPase subunit alpha-1 precursor	K.VDNSSLTGESEPQTR.S	2	4.55	0.40	-2.59
IPI00006482	Isoform Long of Sodium/potassium-transporting ATPase subunit alpha-1 precursor	R.LNIPVSQVNPR.D	2	2.30	0.13	-1.37
IPI00006510	Tubulin beta-1 chain	K.AVLEEDEEVTEEAEMEPEDK.G	2	5.11	0.49	-3.97
IPI00006510	Tubulin beta-1 chain	K.AVLEEDEEVTEEAEMEPEDK.G	3	2.33	0.16	-4.47
IPI00006510	Tubulin beta-1 chain	K.AVLEEDEEVTEEAEMEPEDKGH.-	2	3.72	0.44	-3.30
IPI00006510	Tubulin beta-1 chain	K.AVLEEDEEVTEEAEMEPEDKGH.-	3	2.89	0.37	-4.52
IPI00006524	Uncharacterized protein KIAA0319 precursor	K.TSVDSPVLR.L	2	2.20	0.07	-0.59
IPI00006524	Uncharacterized protein KIAA0319 precursor	R.TYSNAVISPNLETTR.I	2	5.16	0.49	-3.10
IPI00006556	hypothetical protein LOC9865	R.LEELYLGNNLLQALAPGTLAPLRK.L	3	3.44	0.37	-3.15
IPI00006556	hypothetical protein LOC9865	R.LSQLPTALLEPLHSLEALDLSGNELSALHPATFGHLGR.L	5	5.21	0.41	-3.74
IPI00006556	hypothetical protein LOC9865	R.NNALSALSGDIFAASPALYR.L	2	4.46	0.55	-4.06
IPI00006556	hypothetical protein LOC9865	R.NNALSALSGDIFAASPALYR.L	3	3.94	0.37	-3.48
IPI00006601	Secretogranin-1 precursor	A.DASEAHESSSRGEAGAPGEEDIQGPTK.A	3	5.66	0.49	-3.03
IPI00006601	Secretogranin-1 precursor	A.DEPQWSLYPSDSQVSEEVKTR.H	2	3.95	0.47	-4.22
IPI00006601	Secretogranin-1 precursor	A.PGEEDIQGPTK.A	1	3.08	0.28	-3.01
IPI00006601	Secretogranin-1 precursor	A.PGEEDIQGPTKADTEK.W	2	4.13	0.44	-2.22
IPI00006601	Secretogranin-1 precursor	C.IIEVLSNALSK.S	2	4.12	0.31	-3.04
IPI00006601	Secretogranin-1 precursor	D.FYDSEEPVSTHQEAENEKDR.A	3	3.54	0.52	-2.54
IPI00006601	Secretogranin-1 precursor	D.PADASEAHESSSRGEAGAPGEEDIQGPTK.A	4	4.69	0.44	-2.23
IPI00006601	Secretogranin-1 precursor	D.RSSQGGSLPSEEK.G	2	3.71	0.20	-3.71
IPI00006601	Secretogranin-1 precursor	E.AGSQENHPQESK.G	2	3.37	0.33	-2.49
IPI00006601	Secretogranin-1 precursor	E.KSSQESGEEAGSQENHPQESK.G	3	4.56	0.46	-3.09
IPI00006601	Secretogranin-1 precursor	E.LDRNYLNYGEEGAPGK.W	2	4.04	0.37	-2.28
IPI00006601	Secretogranin-1 precursor	E.LDRNYLNYGEEGAPGK.W	3	4.98	0.29	-1.98
IPI00006601	Secretogranin-1 precursor	F.PDFYDSEEPVSTHQEAENEKDR.A	3	3.92	0.52	-2.10
IPI00006601	Secretogranin-1 precursor	G.EAGAPGEEDIQGPTK.A	2	3.04	0.52	-4.03
IPI00006601	Secretogranin-1 precursor	H.GYGEESEEERGLEPGKGR.H	3	4.24	0.42	-3.88
IPI00006601	Secretogranin-1 precursor	K.ADTEKWAEGGGHSR.E	3	3.12	0.13	-1.39
IPI00006601	Secretogranin-1 precursor	K.DKETTENENTKFEV.R	3	4.29	0.44	-1.33
IPI00006601	Secretogranin-1 precursor	K.DKETTENENTKFEVR.L	2	4.76	0.44	-0.61
IPI00006601	Secretogranin-1 precursor	K.DKETTENENTKFEVR.L	3	3.50	0.31	-3.94
IPI00006601	Secretogranin-1 precursor	K.DRADQTVLTEDEKK.E	2	4.17	0.37	-1.99

IPI00006601	Secretogranin-1 precursor	K.DRADQTVLTEDEKK.E	3	3.36	0.23	-0.29
IPI00006601	Secretogranin-1 precursor	K.DRADQTVLTEDEKKELENLAAM*DLELQK.I	3	6.21	0.47	-2.26
IPI00006601	Secretogranin-1 precursor	K.DRADQTVLTEDEKKELENLAAM*DLELQK.I	5	3.86	0.26	-1.42
IPI00006601	Secretogranin-1 precursor	K.DVKDKETTENENTKFEVR.L	2	5.34	0.49	-2.14
IPI00006601	Secretogranin-1 precursor	K.DVKDKETTENENTKFEVR.L	3	3.68	0.43	-2.79
IPI00006601	Secretogranin-1 precursor	K.DVKDKETTENENTKFEVR.L	4	2.92	0.27	-2.28
IPI00006601	Secretogranin-1 precursor	K.ELDRNYLNYGEEGAPGK.W	2	4.52	0.42	-4.21
IPI00006601	Secretogranin-1 precursor	K.ELDRNYLNYGEEGAPGK.W	3	4.55	0.39	-3.02
IPI00006601	Secretogranin-1 precursor	K.ELDRNYLNYGEEGAPGKWQQQGDLQDTKENR.E	4	4.27	0.33	-2.14
IPI00006601	Secretogranin-1 precursor	K.ELENLAAM*DLELQK.I	2	5.38	0.39	-3.97
IPI00006601	Secretogranin-1 precursor	K.ELENLAAM*DLELQK.I	3	5.82	0.30	-2.29
IPI00006601	Secretogranin-1 precursor	K.GERGEDSSEEKHLEEPGETQNAFLNER.K	3	6.04	0.55	-3.49
IPI00006601	Secretogranin-1 precursor	K.GERGEDSSEEKHLEEPGETQNAFLNER.K	4	4.51	0.34	-3.42
IPI00006601	Secretogranin-1 precursor	K.GHPQEESEESNVSM*.A	2	3.93	0.53	-2.50
IPI00006601	Secretogranin-1 precursor	K.GHPQEESEESNVSM*AS.L	2	3.73	0.56	-3.06
IPI00006601	Secretogranin-1 precursor	K.GHPQEESEESNVSM*ASLG.E	2	3.43	0.52	-3.04
IPI00006601	Secretogranin-1 precursor	K.GHPQEESEESNVSM*ASLGE.K	2	4.19	0.58	-3.08
IPI00006601	Secretogranin-1 precursor	K.GYPGVQAPEDLEWER.Y	2	4.62	0.47	-3.48
IPI00006601	Secretogranin-1 precursor	K.HLEEPGETQNAFLNER.K	2	4.61	0.49	-3.31
IPI00006601	Secretogranin-1 precursor	K.HLEEPGETQNAFLNER.K	3	4.36	0.39	-2.63
IPI00006601	Secretogranin-1 precursor	K.HLEEPGETQNAFLNERK.Q	3	3.62	0.45	-0.85
IPI00006601	Secretogranin-1 precursor	K.KEELVAR.S	1	2.17	0.06	-1.71
IPI00006601	Secretogranin-1 precursor	K.KEELVAR.S	2	2.51	0.12	-3.72
IPI00006601	Secretogranin-1 precursor	K.KELENLAAM*DLELQK.I	2	4.81	0.38	-1.86
IPI00006601	Secretogranin-1 precursor	K.KPFSEDVNWGYE.K	2	3.42	0.41	-0.48
IPI00006601	Secretogranin-1 precursor	K.M*AHGYGEESEEER.G	2	3.95	0.54	-3.50
IPI00006601	Secretogranin-1 precursor	K.M*AHGYGEESEEER.G	3	3.67	0.20	-2.04
IPI00006601	Secretogranin-1 precursor	K.M*AHGYGEESEEERGLEPGK.G	2	4.89	0.49	-4.36
IPI00006601	Secretogranin-1 precursor	K.M*AHGYGEESEEERGLEPGK.G	3	3.01	0.31	-4.46
IPI00006601	Secretogranin-1 precursor	K.M*AHGYGEESEEERGLEPGK.G	4	3.12	0.29	-5.79
IPI00006601	Secretogranin-1 precursor	K.M*AHGYGEESEEERGLEPGKGR.H	2	3.72	0.52	-4.71
IPI00006601	Secretogranin-1 precursor	K.M*AHGYGEESEEERGLEPGKGR.H	3	2.30	0.50	-3.60
IPI00006601	Secretogranin-1 precursor	K.M*AHGYGEESEEERGLEPGKGR.H	4	4.30	0.60	-4.82
IPI00006601	Secretogranin-1 precursor	K.QASAIKKEELVA.R	1	2.44	0.25	-5.41
IPI00006601	Secretogranin-1 precursor	K.QASAIKKEELVAR.S	2	1.87	0.30	-4.70
IPI00006601	Secretogranin-1 precursor	K.SAEFPDFYDSEEPVSTH.Q	2	4.47	0.63	-1.20
IPI00006601	Secretogranin-1 precursor	K.SAEFPDFYDSEEPVSTHQEAENEK.D	3	3.27	0.44	-3.91
IPI00006601	Secretogranin-1 precursor	K.SAEFPDFYDSEEPVSTHQEAENEKD.R	3	3.89	0.54	-2.49
IPI00006601	Secretogranin-1 precursor	K.SAEFPDFYDSEEPVSTHQEAENEKDR.A	2	3.47	0.53	-2.64
IPI00006601	Secretogranin-1 precursor	K.SAEFPDFYDSEEPVSTHQEAENEKDR.A	3	5.51	0.59	-3.64
IPI00006601	Secretogranin-1 precursor	K.SAEFPDFYDSEEPVSTHQEAENEKDR.A	4	2.67	0.37	-4.33
IPI00006601	Secretogranin-1 precursor	K.SAEFPDFYDSEEPVSTHQEAENEKDRADQTVLTEDEKK.E	3	5.03	0.53	-0.67

IPI00006601	Secretogranin-1 precursor	K.SAEFPDFYDSEEPVSTHQEAENEKDRADQTVLTEDEKK.E	4	3.43	0.26	-2.21
IPI00006601	Secretogranin-1 precursor	K.SAEFPDFYDSEEPVSTHQEAENEKDRADQTVLTEDEKK.E	6	3.24	0.12	-3.29
IPI00006601	Secretogranin-1 precursor	K.SQREDEEEEEGENYQK.G	3	2.94	0.22	-3.32
IPI00006601	Secretogranin-1 precursor	K.SQREDEEEEEGENYQKGER.G	2	5.86	0.51	-4.78
IPI00006601	Secretogranin-1 precursor	K.SQREDEEEEEGENYQKGER.G	3	5.42	0.48	-4.27
IPI00006601	Secretogranin-1 precursor	K.SSAPPITPECR.Q	2	2.82	0.35	-2.47
IPI00006601	Secretogranin-1 precursor	K.SSQESGEEAGSQENHPQESK.G	2	4.88	0.63	-3.75
IPI00006601	Secretogranin-1 precursor	K.SSQESGEEAGSQENHPQESK.G	3	3.37	0.39	-3.48
IPI00006601	Secretogranin-1 precursor	K.SSQESGEEAGSQENHPQESKGQPR.S	4	2.99	0.28	-4.28
IPI00006601	Secretogranin-1 precursor	K.WQQQGDLQDTK.E	2	3.90	0.36	-2.29
IPI00006601	Secretogranin-1 precursor	L.DRNYLNYGEEGAPGK.W	2	3.57	0.35	-2.41
IPI00006601	Secretogranin-1 precursor	L.LRDPADASEAHESSSR.G	2	3.61	0.34	-4.80
IPI00006601	Secretogranin-1 precursor	L.LRDPADASEAHESSSR.G	3	4.71	0.47	-3.02
IPI00006601	Secretogranin-1 precursor	L.LRDPADASEAHESSSRGEAGAPGEEDIQGPTK.A	4	5.05	0.49	-1.88
IPI00006601	Secretogranin-1 precursor	L.RDPADASEAHESSSR.G	3	4.33	0.48	-2.11
IPI00006601	Secretogranin-1 precursor	N.YLNYGEEGAPGK.W	1	2.81	0.26	-3.26
IPI00006601	Secretogranin-1 precursor	N.YLNYGEEGAPGK.W	2	4.25	0.38	-3.69
IPI00006601	Secretogranin-1 precursor	P.ADASEAHESSSRGEAGAPGEEDIQGPTK.A	3	6.33	0.48	-3.21
IPI00006601	Secretogranin-1 precursor	P.ADASEAHESSSRGEAGAPGEEDIQGPTKADTEK.W	3	7.42	0.57	-3.42
IPI00006601	Secretogranin-1 precursor	P.ADASEAHESSSRGEAGAPGEEDIQGPTKADTEK.W	4	5.75	0.52	-3.42
IPI00006601	Secretogranin-1 precursor	P.SLELDKM*AHGYGEESEEERGLEPGKGR.H	3	3.61	0.41	-1.78
IPI00006601	Secretogranin-1 precursor	Q.ASAIKKEELVAR.S	2	2.95	0.20	-3.93
IPI00006601	Secretogranin-1 precursor	R.ADEPQWSLYPSDSQVSEEVK.T	2	5.11	0.57	-5.19
IPI00006601	Secretogranin-1 precursor	R.ADEPQWSLYPSDSQVSEEVK.T	3	3.42	0.38	-3.67
IPI00006601	Secretogranin-1 precursor	R.ADEPQWSLYPSDSQVSEEVKT.R	2	3.85	0.44	0.40
IPI00006601	Secretogranin-1 precursor	R.ADEPQWSLYPSDSQVSEEVKTR.H	2	4.16	0.53	-2.09
IPI00006601	Secretogranin-1 precursor	R.ADEPQWSLYPSDSQVSEEVKTR.H	3	2.84	0.25	-4.21
IPI00006601	Secretogranin-1 precursor	R.ADQTVLTEDEK.K	2	3.35	0.28	-2.73
IPI00006601	Secretogranin-1 precursor	R.ADQTVLTEDEKK.E	2	3.49	0.27	-0.25
IPI00006601	Secretogranin-1 precursor	R.ADQTVLTEDEKKEL.E	2	3.34	0.35	-3.04
IPI00006601	Secretogranin-1 precursor	R.ADQTVLTEDEKKELEN.L	2	3.91	0.38	-2.70
IPI00006601	Secretogranin-1 precursor	R.ADQTVLTEDEKKELENL.A	2	4.58	0.41	-3.16
IPI00006601	Secretogranin-1 precursor	R.ADQTVLTEDEKKELENLAAM*DLE.L	3	4.27	0.40	-5.05
IPI00006601	Secretogranin-1 precursor	R.ADQTVLTEDEKKELENLAAM*DLELQ.K	3	4.32	0.37	-4.39
IPI00006601	Secretogranin-1 precursor	R.ADQTVLTEDEKKELENLAAM*DLELQK.I	2	5.46	0.51	-2.91
IPI00006601	Secretogranin-1 precursor	R.ADQTVLTEDEKKELENLAAM*DLELQK.I	3	6.32	0.47	-7.12
IPI00006601	Secretogranin-1 precursor	R.ADQTVLTEDEKKELENLAAM*DLELQK.I	4	4.80	0.33	-4.86
IPI00006601	Secretogranin-1 precursor	R.ADQTVLTEDEKKELENLAAM*DLELQK.I	5	4.21	0.19	-2.44
IPI00006601	Secretogranin-1 precursor	R.ADQTVLTEDEKKELENLAAM*DLELQKIAEK.F	4	3.42	0.26	-2.64
IPI00006601	Secretogranin-1 precursor	R.APRPQSEESWDEE.D	2	3.63	0.40	-3.39
IPI00006601	Secretogranin-1 precursor	R.APRPQSEESWDEED.K	2	3.87	0.52	-3.78
IPI00006601	Secretogranin-1 precursor	R.ASEEEPEYGEEIK.G	2	4.35	0.34	-3.92

IPI00006601	Secretogranin-1 precursor	R.ASEEEPEYGEEIKGYPGVQAPEDLEWER.Y	3	5.68	0.38	-7.98
IPI00006601	Secretogranin-1 precursor	R.AYFM*SDTR.E	2	2.24	0.19	-1.20
IPI00006601	Secretogranin-1 precursor	R.AYFM*SDTREE.K	2	3.30	0.45	-3.40
IPI00006601	Secretogranin-1 precursor	R.AYFM*SDTREEK.R	2	2.68	0.18	-3.03
IPI00006601	Secretogranin-1 precursor	R.CIIEVLSNALSK.S	1	3.50	0.40	-2.75
IPI00006601	Secretogranin-1 precursor	R.CIIEVLSNALSK.S	2	5.07	0.45	-5.01
IPI00006601	Secretogranin-1 precursor	R.CIIEVLSNALSK.S	3	2.82	0.12	-2.94
IPI00006601	Secretogranin-1 precursor	R.DPADASEAHESSSR.G	2	4.67	0.62	-3.44
IPI00006601	Secretogranin-1 precursor	R.DPADASEAHESSSR.G	3	2.98	0.46	-1.05
IPI00006601	Secretogranin-1 precursor	R.DPADASEAHESSSRGEAGAPGEEDIQGPTK.A	2	4.49	0.49	-3.41
IPI00006601	Secretogranin-1 precursor	R.DPADASEAHESSSRGEAGAPGEEDIQGPTK.A	3	6.54	0.57	-4.11
IPI00006601	Secretogranin-1 precursor	R.DPADASEAHESSSRGEAGAPGEEDIQGPTK.A	4	4.13	0.37	-2.20
IPI00006601	Secretogranin-1 precursor	R.DPADASEAHESSSRGEAGAPGEEDIQGPTKADTEK.W	3	8.25	0.56	-2.76
IPI00006601	Secretogranin-1 precursor	R.DPADASEAHESSSRGEAGAPGEEDIQGPTKADTEK.W	4	5.38	0.50	-2.35
IPI00006601	Secretogranin-1 precursor	R.DPADASEAHESSSRGEAGAPGEEDIQGPTKADTEK.W	5	2.81	0.40	-2.54
IPI00006601	Secretogranin-1 precursor	R.EKSSQESGEEAGSQENHPQE.S	3	4.50	0.55	-2.46
IPI00006601	Secretogranin-1 precursor	R.EKSSQESGEEAGSQENHPQESK.G	2	5.08	0.58	-4.40
IPI00006601	Secretogranin-1 precursor	R.EKSSQESGEEAGSQENHPQESK.G	3	4.88	0.55	-3.59
IPI00006601	Secretogranin-1 precursor	R.EKSSQESGEEAGSQENHPQESK.G	4	3.75	0.37	-3.80
IPI00006601	Secretogranin-1 precursor	R.EKSSQESGEEAGSQENHPQESKGQPR.S	4	3.43	0.41	-1.62
IPI00006601	Secretogranin-1 precursor	R.ERADEPQWSLYPSDSQVSEEVK.T	2	5.86	0.59	-3.57
IPI00006601	Secretogranin-1 precursor	R.ERADEPQWSLYPSDSQVSEEVK.T	3	6.88	0.56	-3.63
IPI00006601	Secretogranin-1 precursor	R.ERADEPQWSLYPSDSQVSEEVKT.R	2	4.68	0.53	-3.71
IPI00006601	Secretogranin-1 precursor	R.ERADEPQWSLYPSDSQVSEEVKT.R	3	5.58	0.48	-2.50
IPI00006601	Secretogranin-1 precursor	R.FLGEGHHR.V	1	1.70	0.09	-4.26
IPI00006601	Secretogranin-1 precursor	R.FLGEGHHR.V	2	2.40	0.24	-2.38
IPI00006601	Secretogranin-1 precursor	R.FQDKQYSSHHT.A	2	3.79	0.28	-3.80
IPI00006601	Secretogranin-1 precursor	R.FQDKQYSSHHTA.E	2	4.21	0.40	-3.72
IPI00006601	Secretogranin-1 precursor	R.FQDKQYSSHHTAE.K	2	3.99	0.40	-3.94
IPI00006601	Secretogranin-1 precursor	R.GEAGAPGEEDIQGPTK.A	1	3.46	0.37	-4.04
IPI00006601	Secretogranin-1 precursor	R.GEAGAPGEEDIQGPTK.A	2	4.62	0.41	-3.60
IPI00006601	Secretogranin-1 precursor	R.GEAGAPGEEDIQGPTK.A	3	3.49	0.32	-1.94
IPI00006601	Secretogranin-1 precursor	R.GEAGAPGEEDIQGPTKA.D	2	4.12	0.42	-4.69
IPI00006601	Secretogranin-1 precursor	R.GEAGAPGEEDIQGPTKADTE.K	2	3.51	0.48	-4.04
IPI00006601	Secretogranin-1 precursor	R.GEAGAPGEEDIQGPTKADTEK.W	2	4.96	0.52	-3.58
IPI00006601	Secretogranin-1 precursor	R.GEAGAPGEEDIQGPTKADTEK.W	3	1.62	0.20	-2.24
IPI00006601	Secretogranin-1 precursor	R.GEDSSEEKHLEEPGETQNAFLNER.K	2	4.71	0.53	-3.26
IPI00006601	Secretogranin-1 precursor	R.GEDSSEEKHLEEPGETQNAFLNER.K	3	6.62	0.55	-2.34
IPI00006601	Secretogranin-1 precursor	R.GEDSSEEKHLEEPGETQNAFLNER.K	4	3.92	0.41	-2.81
IPI00006601	Secretogranin-1 precursor	R.KDVKDKETTENENTKFEV.R	3	3.97	0.28	-3.07
IPI00006601	Secretogranin-1 precursor	R.KQASAIKKEELV.A	1	3.01	0.26	-5.37
IPI00006601	Secretogranin-1 precursor	R.KQASAIKKEELV.A	2	3.47	0.30	-3.60

IPI00006601	Secretogranin-1 precursor	R.KQASAIKKEELVA.R	2	3.63	0.45	-4.46
IPI00006601	Secretogranin-1 precursor	R.KQASAIKKEELVAR.S	2	5.16	0.39	-4.06
IPI00006601	Secretogranin-1 precursor	R.KQASAIKKEELVAR.S	3	3.22	0.29	-2.77
IPI00006601	Secretogranin-1 precursor	R.LLRDPADASEAHESSSR.G	2	4.54	0.45	-5.12
IPI00006601	Secretogranin-1 precursor	R.LLRDPADASEAHESSSR.G	3	5.11	0.50	-3.98
IPI00006601	Secretogranin-1 precursor	R.LLRDPADASEAHESSSR.G	4	3.74	0.39	-1.77
IPI00006601	Secretogranin-1 precursor	R.LLRDPADASEAHESSSRGEAGAPGEEDIQGPTK.A	3	5.00	0.57	-4.33
IPI00006601	Secretogranin-1 precursor	R.LLRDPADASEAHESSSRGEAGAPGEEDIQGPTK.A	4	5.84	0.48	-3.68
IPI00006601	Secretogranin-1 precursor	R.LLRDPADASEAHESSSRGEAGAPGEEDIQGPTKADTEK.W	4	6.22	0.56	-3.54
IPI00006601	Secretogranin-1 precursor	R.NYLNYGEEGAPGK.W	1	2.88	0.35	-3.25
IPI00006601	Secretogranin-1 precursor	R.NYLNYGEEGAPGK.W	2	4.29	0.47	-3.54
IPI00006601	Secretogranin-1 precursor	R.NYLNYGEEGAPGKWQQQGDLQDTK.E	3	3.57	0.35	-2.19
IPI00006601	Secretogranin-1 precursor	R.NYPSLELDK.M	1	2.22	0.11	-4.24
IPI00006601	Secretogranin-1 precursor	R.NYPSLELDKM*AHGYGEESEEER.G	2	4.87	0.60	-3.45
IPI00006601	Secretogranin-1 precursor	R.NYPSLELDKM*AHGYGEESEEER.G	3	3.39	0.56	-3.13
IPI00006601	Secretogranin-1 precursor	R.NYPSLELDKM*AHGYGEESEEERGLEPGK.G	3	4.52	0.44	-2.88
IPI00006601	Secretogranin-1 precursor	R.NYPSLELDKM*AHGYGEESEEERGLEPGK.G	4	2.93	0.36	-3.01
IPI00006601	Secretogranin-1 precursor	R.NYPSLELDKM*AHGYGEESEEERGLEPGKGR.H	3	3.95	0.43	-0.22
IPI00006601	Secretogranin-1 precursor	R.NYPSLELDKM*AHGYGEESEEERGLEPGKGR.H	4	2.75	0.36	-2.46
IPI00006601	Secretogranin-1 precursor	R.SQEESEEGEEDATSEVDKR.R	3	3.22	0.21	-3.59
IPI00006601	Secretogranin-1 precursor	R.SQEESEEGEEDATSEVDKRR.T	2	4.53	0.48	-2.17
IPI00006601	Secretogranin-1 precursor	R.SQEESEEGEEDATSEVDKRR.T	3	3.29	0.32	-2.40
IPI00006601	Secretogranin-1 precursor	R.SQEESEEGEEDATSEVDKRR.T	4	4.09	0.38	-1.75
IPI00006601	Secretogranin-1 precursor	R.SSQGGSLPSEEK.G	1	2.14	0.09	-3.35
IPI00006601	Secretogranin-1 precursor	R.SSQGGSLPSEEK.G	2	3.96	0.33	-3.27
IPI00006601	Secretogranin-1 precursor	R.SSQGGSLPSEEKGHPQ.E	2	3.81	0.45	-2.91
IPI00006601	Secretogranin-1 precursor	R.SSQGGSLPSEEKGHPQEES.E	2	3.45	0.46	-3.34
IPI00006601	Secretogranin-1 precursor	R.SSQGGSLPSEEKGHPQEESEESN.V	2	3.53	0.43	-4.22
IPI00006601	Secretogranin-1 precursor	R.SSQGGSLPSEEKGHPQEESEESNVSM*A.S	3	3.52	0.42	-3.23
IPI00006601	Secretogranin-1 precursor	R.SSQGGSLPSEEKGHPQEESEESNVSM*ASLG.E	3	3.67	0.42	-1.78
IPI00006601	Secretogranin-1 precursor	R.SSQGGSLPSEEKGHPQEESEESNVSM*ASLGE.K	3	4.32	0.55	-5.24
IPI00006601	Secretogranin-1 precursor	R.SSQGGSLPSEEKGHPQEESEESNVSM*ASLGEK.R	3	4.26	0.39	-2.75
IPI00006601	Secretogranin-1 precursor	R.SSQGGSLPSEEKGHPQEESEESNVSM*ASLGEKR.D	4	3.41	0.16	-3.92
IPI00006601	Secretogranin-1 precursor	R.VAQLDQLLHY.R	2	3.85	0.34	-3.22
IPI00006601	Secretogranin-1 precursor	R.VAQLDQLLHYR.K	2	3.30	0.43	-3.54
IPI00006601	Secretogranin-1 precursor	R.VAQLDQLLHYR.K	3	3.20	0.32	-2.85
IPI00006601	Secretogranin-1 precursor	R.VAQLDQLLHYRK.K	2	3.74	0.39	-2.73
IPI00006601	Secretogranin-1 precursor	S.DSQVSEEVK.T	2	3.03	0.16	-2.29
IPI00006601	Secretogranin-1 precursor	S.DSQVSEEVKTR.H	2	3.23	0.28	-2.37
IPI00006601	Secretogranin-1 precursor	S.EEPVSTHQEAENEKDR.A	2	2.91	0.44	-3.53
IPI00006601	Secretogranin-1 precursor	S.SQESGEEAGSQENHPQESK.G	2	4.50	0.58	-3.88
IPI00006601	Secretogranin-1 precursor	W.SLYPSDSQVSEEVK.T	2	4.04	0.49	-4.37

IPI00006601	Secretogranin-1 precursor	W.SLYPSDSQVSEEVKTR.H	2	4.28	0.50	-3.74
IPI00006601	Secretogranin-1 precursor	Y.DSEEPVSTHQEAENEKDR.A	2	4.35	0.45	-3.30
IPI00006601	Secretogranin-1 precursor	Y.LNYGEEGAPGK.W	2	3.94	0.40	-2.94
IPI00006601	Secretogranin-1 precursor	Y.PSDSQVSEEVK.T	2	4.53	0.43	-2.32
IPI00006601	Secretogranin-1 precursor	Y.PSDSQVSEEVKTR.H	3	3.68	0.35	-0.45
IPI00006608	Isoform APP770 of Amyloid beta A4 protein precursor (Fragment)	A.DRGLTTRPGSGLTNIKTEEISEVK.M	3	3.64	0.29	-3.89
IPI00006608	Isoform APP770 of Amyloid beta A4 protein precursor (Fragment)	A.LEVPTDGNAGLLAEPQIAM*FCGR.L	2	6.63	0.58	-5.73
IPI00006608	Isoform APP770 of Amyloid beta A4 protein precursor (Fragment)	A.LEVPTDGNAGLLAEPQIAM*FCGR.L	3	6.88	0.51	-5.99
IPI00006608	Isoform APP770 of Amyloid beta A4 protein precursor (Fragment)	A.VIQHFQEKVESLEQEAANER.Q	3	5.74	0.45	-4.38
IPI00006608	Isoform APP770 of Amyloid beta A4 protein precursor (Fragment)	E.FVSDALLVPDK.C	2	3.16	0.20	-5.01
IPI00006608	Isoform APP770 of Amyloid beta A4 protein precursor (Fragment)	H.SFGADSVPANTENEVEPVDARPAADR.G	3	3.70	0.37	-4.23
IPI00006608	Isoform APP770 of Amyloid beta A4 protein precursor (Fragment)	K.AVIQHFQEK.V	1	2.34	0.16	-4.69
IPI00006608	Isoform APP770 of Amyloid beta A4 protein precursor (Fragment)	K.AVIQHFQEKVESLEQEAANER.Q	2	6.49	0.58	-5.52
IPI00006608	Isoform APP770 of Amyloid beta A4 protein precursor (Fragment)	K.AVIQHFQEKVESLEQEAANER.Q	3	6.15	0.56	-4.45
IPI00006608	Isoform APP770 of Amyloid beta A4 protein precursor (Fragment)	K.AVIQHFQEKVESLEQEAANER.Q	4	3.75	0.21	-4.67
IPI00006608	Isoform APP770 of Amyloid beta A4 protein precursor (Fragment)	K.CAPFFYGGCGGNR.N	2	3.95	0.42	-2.68
IPI00006608	Isoform APP770 of Amyloid beta A4 protein precursor (Fragment)	K.EGILQYCQEVYPELQITNVVEANQPVTIQNWCK.R	3	4.59	0.56	-3.42
IPI00006608	Isoform APP770 of Amyloid beta A4 protein precursor (Fragment)	K.EGILQYCQEVYPELQITNVVEANQPVTIQNWCKR.G	3	6.48	0.55	-3.71
IPI00006608	Isoform APP770 of Amyloid beta A4 protein precursor (Fragment)	K.EGILQYCQEVYPELQITNVVEANQPVTIQNWCKR.G	4	5.24	0.50	-4.31
IPI00006608	Isoform APP770 of Amyloid beta A4 protein precursor (Fragment)	K.EQNYSDDVLANM*ISEPR.I	2	4.98	0.48	-4.16
IPI00006608	Isoform APP770 of Amyloid beta A4 protein precursor (Fragment)	K.EQNYSDDVLANM*ISEPR.I	3	4.02	0.38	-3.10
IPI00006608	Isoform APP770 of Amyloid beta A4 protein precursor (Fragment)	K.KAVIQHFQEK.V	2	3.27	0.19	-3.14
IPI00006608	Isoform APP770 of Amyloid beta A4 protein precursor (Fragment)	K.LVFFAEDVGSNK.G	2	4.29	0.50	-4.95

IPI00006608	Isoform APP770 of Amyloid beta A4 protein precursor (Fragment)	K.M*DAEFRHDSGY.E	2	3.24	0.47	-2.39
IPI00006608	Isoform APP770 of Amyloid beta A4 protein precursor (Fragment)	K.M*DAEFRHDSGYE.V	2	3.69	0.42	-3.55
IPI00006608	Isoform APP770 of Amyloid beta A4 protein precursor (Fragment)	K.M*DAEFRHDSGYEVHHQ.K	3	4.11	0.49	-2.49
IPI00006608	Isoform APP770 of Amyloid beta A4 protein precursor (Fragment)	K.STNLHDYGM*LLPCGIDKFR.G	3	3.05	0.28	-4.25
IPI00006608	Isoform APP770 of Amyloid beta A4 protein precursor (Fragment)	K.STNLHDYGM*LLPCGIDKFR.G	4	3.92	0.35	-4.44
IPI00006608	Isoform APP770 of Amyloid beta A4 protein precursor (Fragment)	K.TEEISEVKM*.D	1	2.23	0.25	-4.38
IPI00006608	Isoform APP770 of Amyloid beta A4 protein precursor (Fragment)	K.TEEISEVKM*.D	2	3.79	0.39	-2.76
IPI00006608	Isoform APP770 of Amyloid beta A4 protein precursor (Fragment)	K.TEEISEVKM*DAE.F	2	4.13	0.38	-3.05
IPI00006608	Isoform APP770 of Amyloid beta A4 protein precursor (Fragment)	K.TEEISEVKM*DAEFR.H	2	3.94	0.51	-3.48
IPI00006608	Isoform APP770 of Amyloid beta A4 protein precursor (Fragment)	K.TEEISEVKM*DAEFR.H	3	2.65	0.24	-2.05
IPI00006608	Isoform APP770 of Amyloid beta A4 protein precursor (Fragment)	K.THPHFVIPYR.C	1	2.39	0.19	-5.31
IPI00006608	Isoform APP770 of Amyloid beta A4 protein precursor (Fragment)	K.THPHFVIPYR.C	2	2.97	0.38	-4.05
IPI00006608	Isoform APP770 of Amyloid beta A4 protein precursor (Fragment)	K.THPHFVIPYR.C	3	3.46	0.26	-4.52
IPI00006608	Isoform APP770 of Amyloid beta A4 protein precursor (Fragment)	K.TTVELLPVNGEFSLDDLQPWHSFGADSVPANTENEVEPVDAR.P	3	4.70	0.49	-5.12
IPI00006608	Isoform APP770 of Amyloid beta A4 protein precursor (Fragment)	K.VESLEQEAANER.Q	1	2.84	0.36	-1.90
IPI00006608	Isoform APP770 of Amyloid beta A4 protein precursor (Fragment)	K.VESLEQEAANER.Q	2	4.26	0.35	-3.68
IPI00006608	Isoform APP770 of Amyloid beta A4 protein precursor (Fragment)	K.VESLEQEAANER.Q	3	3.99	0.08	-2.10
IPI00006608	Isoform APP770 of Amyloid beta A4 protein precursor (Fragment)	K.WDSDPSGTK.T	1	1.76	0.30	-3.67
IPI00006608	Isoform APP770 of Amyloid beta A4 protein precursor (Fragment)	K.WDSDPSGTK.T	2	2.28	0.07	-1.22
IPI00006608	Isoform APP770 of Amyloid beta A4 protein precursor (Fragment)	K.YLETPGDENEHAHFQK.A	2	4.13	0.52	-4.52
IPI00006608	Isoform APP770 of Amyloid beta A4 protein precursor (Fragment)	K.YLETPGDENEHAHFQK.A	3	2.74	0.27	-3.36

IPI00006608	Isoform APP770 of Amyloid beta A4 protein precursor (Fragment)	K.YLETPGDENEHAHFQK.A	4	2.72	0.27	-3.30
IPI00006608	Isoform APP770 of Amyloid beta A4 protein precursor (Fragment)	L.EVPTDGNAGLLAEPQIAM*FCGR.L	2	4.20	0.42	-5.27
IPI00006608	Isoform APP770 of Amyloid beta A4 protein precursor (Fragment)	L.EVPTDGNAGLLAEPQIAM*FCGR.L	3	4.50	0.45	-4.18
IPI00006608	Isoform APP770 of Amyloid beta A4 protein precursor (Fragment)	L.YNVPAVAEEIQDEVDELLQK.E	2	5.64	0.46	-5.51
IPI00006608	Isoform APP770 of Amyloid beta A4 protein precursor (Fragment)	N.YITALQAVPPRPR.H	2	3.04	0.23	-1.24
IPI00006608	Isoform APP770 of Amyloid beta A4 protein precursor (Fragment)	P.VNGEFSLDDLQPWHSFGADSVPANTENEVEPVDARPAADR.G	4	4.54	0.29	-3.93
IPI00006608	Isoform APP770 of Amyloid beta A4 protein precursor (Fragment)	R.CLVGEFVSDALLVPDK.C	2	5.61	0.47	-8.27
IPI00006608	Isoform APP770 of Amyloid beta A4 protein precursor (Fragment)	R.CLVGEFVSDALLVPDK.C	3	3.54	0.29	-6.01
IPI00006608	Isoform APP770 of Amyloid beta A4 protein precursor (Fragment)	R.CLVGEFVSDALLVPDKCK.F	2	4.76	0.46	-2.83
IPI00006608	Isoform APP770 of Amyloid beta A4 protein precursor (Fragment)	R.CLVGEFVSDALLVPDKCK.F	3	5.06	0.44	-4.86
IPI00006608	Isoform APP770 of Amyloid beta A4 protein precursor (Fragment)	R.EVCSEQAETGPCR.A	2	3.67	0.41	-2.36
IPI00006608	Isoform APP770 of Amyloid beta A4 protein precursor (Fragment)	R.EWEEAER.Q	2	1.41	0.07	-2.94
IPI00006608	Isoform APP770 of Amyloid beta A4 protein precursor (Fragment)	R.GLTTRPGSGLTNIK.T	2	3.60	0.22	-2.71
IPI00006608	Isoform APP770 of Amyloid beta A4 protein precursor (Fragment)	R.GLTTRPGSGLTNIKTEE.I	2	3.43	0.32	-3.10
IPI00006608	Isoform APP770 of Amyloid beta A4 protein precursor (Fragment)	R.GLTTRPGSGLTNIKTEEISE.V	2	3.04	0.39	-2.07
IPI00006608	Isoform APP770 of Amyloid beta A4 protein precursor (Fragment)	R.GLTTRPGSGLTNIKTEEISEV.K	2	3.48	0.45	-4.64
IPI00006608	Isoform APP770 of Amyloid beta A4 protein precursor (Fragment)	R.GLTTRPGSGLTNIKTEEISEVK.M	2	4.94	0.50	-3.58
IPI00006608	Isoform APP770 of Amyloid beta A4 protein precursor (Fragment)	R.GLTTRPGSGLTNIKTEEISEVK.M	3	5.27	0.49	-4.07
IPI00006608	Isoform APP770 of Amyloid beta A4 protein precursor (Fragment)	R.GLTTRPGSGLTNIKTEEISEVK.M	4	2.51	0.26	-2.00
IPI00006608	Isoform APP770 of Amyloid beta A4 protein precursor (Fragment)	R.GLTTRPGSGLTNIKTEEISEVKM*.D	2	4.58	0.42	-4.34
IPI00006608	Isoform APP770 of Amyloid beta A4 protein precursor (Fragment)	R.GLTTRPGSGLTNIKTEEISEVKM*.D	3	5.13	0.45	-4.10

IPI00006608	Isoform APP770 of Amyloid beta A4 protein precursor (Fragment)	R.GLTTRPGSGLTNIKTEEISEVKM*DAE.F	3	3.78	0.38	-4.95
IPI00006608	Isoform APP770 of Amyloid beta A4 protein precursor (Fragment)	R.GLTTRPGSGLTNIKTEEISEVKM*DAEFR.H	4	3.13	0.12	-0.93
IPI00006608	Isoform APP770 of Amyloid beta A4 protein precursor (Fragment)	R.ISYGNDALM*PSLTETK.T	2	5.65	0.48	-3.69
IPI00006608	Isoform APP770 of Amyloid beta A4 protein precursor (Fragment)	R.ISYGNDALM*PSLTETK.T	3	2.38	0.06	-2.09
IPI00006608	Isoform APP770 of Amyloid beta A4 protein precursor (Fragment)	R.LALENYITALQAVPPRPR.H	2	3.07	0.31	-3.36
IPI00006608	Isoform APP770 of Amyloid beta A4 protein precursor (Fragment)	R.LALENYITALQAVPPRPR.H	3	3.55	0.51	-5.03
IPI00006608	Isoform APP770 of Amyloid beta A4 protein precursor (Fragment)	R.LALENYITALQAVPPRPR.H	4	4.45	0.43	-1.40
IPI00006608	Isoform APP770 of Amyloid beta A4 protein precursor (Fragment)	R.LNM*HM*NVQNGK.W	2	3.49	0.35	-3.59
IPI00006608	Isoform APP770 of Amyloid beta A4 protein precursor (Fragment)	R.LNM*HM*NVQNGK.W	3	2.96	0.26	-3.51
IPI00006608	Isoform APP770 of Amyloid beta A4 protein precursor (Fragment)	R.LNM*HM*NVQNGKWDSDPSGTK.T	3	4.53	0.47	-1.49
IPI00006608	Isoform APP770 of Amyloid beta A4 protein precursor (Fragment)	R.LNM*HM*NVQNGKWDSDPSGTK.T	4	3.88	0.34	-0.28
IPI00006608	Isoform APP770 of Amyloid beta A4 protein precursor (Fragment)	R.RLALENYITALQAVPPRPR.H	2	3.34	0.44	-4.43
IPI00006608	Isoform APP770 of Amyloid beta A4 protein precursor (Fragment)	R.RLALENYITALQAVPPRPR.H	3	5.30	0.51	-4.63
IPI00006608	Isoform APP770 of Amyloid beta A4 protein precursor (Fragment)	R.RLALENYITALQAVPPRPR.H	4	3.96	0.43	-4.01
IPI00006608	Isoform APP770 of Amyloid beta A4 protein precursor (Fragment)	R.SQVM*THLR.V	2	2.42	0.09	-2.37
IPI00006608	Isoform APP770 of Amyloid beta A4 protein precursor (Fragment)	R.VEAM*LNDR.R	2	2.96	0.32	-3.35
IPI00006608	Isoform APP770 of Amyloid beta A4 protein precursor (Fragment)	R.WYFDVTEGK.C	2	2.93	0.29	-2.98
IPI00006608	Isoform APP770 of Amyloid beta A4 protein precursor (Fragment)	S.LDDLQPWHSFGADSVPANTENEVEPVDARPAADR.G	3	4.57	0.43	-3.02
IPI00006608	Isoform APP770 of Amyloid beta A4 protein precursor (Fragment)	S.LDDLQPWHSFGADSVPANTENEVEPVDARPAADR.G	4	4.46	0.36	-2.85
IPI00006608	Isoform APP770 of Amyloid beta A4 protein precursor (Fragment)	V.GEFVSDALLVPDK.C	2	3.46	0.31	-3.15
IPI00006608	Isoform APP770 of Amyloid beta A4 protein precursor (Fragment)	V.SDALLVPDK.C	2	2.91	0.32	1.82

IPI00006608	Isoform APP770 of Amyloid beta A4 protein precursor (Fragment)	W.HSFGADSVPANTENEVEPVDARPAADR.G	3	4.50	0.38	-4.47
IPI00006644	Isoform 2 of Plexin-B1 precursor	R.DLTFDGTFEHLYVM*TQSTLLK.V	3	4.86	0.33	-4.66
IPI00006644	Isoform 2 of Plexin-B1 precursor	R.DPYCGWCVLLGR.C	2	3.95	0.47	-3.48
IPI00006644	Isoform 2 of Plexin-B1 precursor	R.FGAVVIAK.T	2	2.63	0.19	-3.32
IPI00006644	Isoform 2 of Plexin-B1 precursor	R.GVGGGIPPITTR.A	2	3.12	0.37	-1.98
IPI00006644	Isoform 2 of Plexin-B1 precursor	R.LSEYSHHFVSAFAR.G	3	3.29	0.29	-3.75
IPI00006644	Isoform 2 of Plexin-B1 precursor	R.VYLGPGSDGHPYSTQSIQQGSAVSR.D	3	5.93	0.42	-3.32
IPI00006644	Isoform 2 of Plexin-B1 precursor	Y.STQSIQQGSAVSR.D	2	3.90	0.43	-2.39
IPI00006657	Protein FAM20B precursor	K.SFGNPSLDER.S	2	2.87	0.23	-0.88
IPI00006657	Protein FAM20B precursor	R.LLDIIDTAVFDYLIGNADR.H	2	5.51	0.52	-2.89
IPI00006657	Protein FAM20B precursor	R.LLDIIDTAVFDYLIGNADR.H	3	5.02	0.40	-2.18
IPI00006657	Protein FAM20B precursor	R.LLSVLATVK.Q	2	2.69	0.23	-1.39
IPI00006657	Protein FAM20B precursor	R.SILAPLYQCCIIR.V	2	3.12	0.36	-2.73
IPI00006662	Apolipoprotein D precursor	K.CPNPPVQENFDVNK.Y	1	2.60	0.19	
IPI00006662	Apolipoprotein D precursor	K.CPNPPVQENFDVNK.Y	2	4.48	0.36	
IPI00006662	Apolipoprotein D precursor	K.CPNPPVQENFDVNK.Y	3	5.21	0.24	
IPI00006662	Apolipoprotein D precursor	K.CPNPPVQENFDVNKYLGR.W	2	5.22	0.46	
IPI00006662	Apolipoprotein D precursor	K.CPNPPVQENFDVNKYLGR.W	3	2.06	0.10	-0.72
IPI00006662	Apolipoprotein D precursor	K.IKVLNQELR.A	1	2.67	0.07	
IPI00006662	Apolipoprotein D precursor	K.IKVLNQELR.A	2	3.09	0.07	
IPI00006662	Apolipoprotein D precursor	K.IPTTFENGR.C	2	2.30	0.15	
IPI00006662	Apolipoprotein D precursor	K.KM*TVTDQVNCPK.L	1	2.10	0.19	
IPI00006662	Apolipoprotein D precursor	K.KM*TVTDQVNCPK.L	2	3.93	0.36	
IPI00006662	Apolipoprotein D precursor	K.KM*TVTDQVNCPK.L	3	3.42	0.09	
IPI00006662	Apolipoprotein D precursor	K.KM*TVTDQVNCPKLS.-	2	3.51	0.20	
IPI00006662	Apolipoprotein D precursor	K.KMTVTDQVNCPK.L	1	3.37	0.22	
IPI00006662	Apolipoprotein D precursor	K.KMTVTDQVNCPK.L	2	3.97	0.32	
IPI00006662	Apolipoprotein D precursor	K.M*TVTDQVNCPK.L	1	2.18	0.28	
IPI00006662	Apolipoprotein D precursor	K.M*TVTDQVNCPK.L	2	4.10	0.45	-4.27
IPI00006662	Apolipoprotein D precursor	K.M*TVTDQVNCPKLS.-	2	4.25	0.43	
IPI00006662	Apolipoprotein D precursor	K.MTVTDQVNCPK.L	2	3.82	0.28	
IPI00006662	Apolipoprotein D precursor	K.MTVTDQVNCPKLS.-	2	4.49	0.35	
IPI00006662	Apolipoprotein D precursor	K.NILTSNNIDVK.K	1	2.75	0.27	-3.04
IPI00006662	Apolipoprotein D precursor	K.NILTSNNIDVK.K	2	2.12	0.09	-1.35
IPI00006662	Apolipoprotein D precursor	K.NILTSNNIDVKK.M	2	2.95	0.24	-3.91
IPI00006662	Apolipoprotein D precursor	K.NILTSNNIDVKK.M	3	2.08	0.23	-4.18
IPI00006662	Apolipoprotein D precursor	N.PPVQENFDVNKYLGR.W	2	5.44	0.40	
IPI00006662	Apolipoprotein D precursor	R.CIQANYSLM*ENGK.I	2	4.06	0.38	
IPI00006662	Apolipoprotein D precursor	R.NPNLPPETVDSLK.N	2	2.92	0.21	-4.77
IPI00006662	Apolipoprotein D precursor	R.NPNLPPETVDSLKNILTSNNIDVK.K	2	2.80	0.12	
IPI00006662	Apolipoprotein D precursor	R.NPNLPPETVDSLKNILTSNNIDVK.K	3	2.36	0.18	-2.49

IPI00006662	Apolipoprotein D precursor	R.NPNLPPETVDSLKNILTSNNIDVKK.M	2	4.77	0.36	
IPI00006662	Apolipoprotein D precursor	R.NPNLPPETVDSLKNILTSNNIDVKK.M	4	2.39	0.10	-3.08
IPI00006662	Apolipoprotein D precursor	R.WYEIEKIPTTFENGR.C	1	4.16	0.36	
IPI00006662	Apolipoprotein D precursor	R.WYEIEKIPTTFENGR.C	2	4.92	0.34	
IPI00006662	Apolipoprotein D precursor	R.WYEIEKIPTTFENGR.C	3	3.16	0.33	-2.65
IPI00006713	Isoform 1 of DnaJ homolog subfamily C member 3	K.ISTLYYQLGDHELSLSEVR.E	3	2.77	0.34	-2.38
IPI00006713	Isoform 1 of DnaJ homolog subfamily C member 3	K.LKNDNTEAFYK.I	2	2.81	0.24	-3.85
IPI00006713	Isoform 1 of DnaJ homolog subfamily C member 3	K.LLAAGQLADALSQFHAAVDGDPDNYIAYYR.R	3	7.18	0.61	-3.71
IPI00006713	Isoform 1 of DnaJ homolog subfamily C member 3	K.M*DFTAAR.L	2	2.30	0.18	-3.61
IPI00006746	Ermin	K.ISEELTDVDSPLPHYR.V	3	3.18	0.16	-2.07
IPI00006803	Carbohydrate sulfotransferase 10	K.FFIVRDPFER.L	2	2.12	0.13	-3.65
IPI00006803	Carbohydrate sulfotransferase 10	K.ILFCQTPK.V	2	2.32	0.06	-1.75
IPI00006803	Carbohydrate sulfotransferase 10	K.LFGYQKPDFLLN.-	2	3.67	0.31	-3.96
IPI00006803	Carbohydrate sulfotransferase 10	R.GIQFEDFVR.Y	1	1.67	0.10	-4.35
IPI00006803	Carbohydrate sulfotransferase 10	R.GIQFEDFVR.Y	2	2.87	0.16	-2.72
IPI00006803	Carbohydrate sulfotransferase 10	R.LSSFSDAEIQKR.L	2	3.04	0.38	-2.73
IPI00006803	Carbohydrate sulfotransferase 10	R.LSSFSDAEIQKR.L	3	1.72	0.19	-2.42
IPI00006900	Something about silencing protein 10	K.TSAAACAVTDLSDDSDFDEKAKLK.Y	2	2.70	0.10	-7.35
IPI00006967	Protocadherin-9 precursor	K.AVTLSILNDNDNFVLDPYSGVIK.S	3	3.57	0.20	-2.85
IPI00006967	Protocadherin-9 precursor	K.AVYDNQYLLETSSLLDYEGTKEFSFK.I	3	3.40	0.36	-3.65
IPI00006967	Protocadherin-9 precursor	K.DLNISHINAATGTSASLVYR.L	3	3.09	0.29	-2.26
IPI00006967	Protocadherin-9 precursor	K.IALITVSDKDTDVNGK.V	2	4.30	0.47	-2.57
IPI00006967	Protocadherin-9 precursor	K.IALITVSDKDTDVNGK.V	3	3.04	0.27	-1.95
IPI00006967	Protocadherin-9 precursor	K.TGVLTASR.V	2	2.53	0.17	-3.25
IPI00006967	Protocadherin-9 precursor	K.VSSSTGEIFTTSNR.I	2	4.09	0.39	-3.17
IPI00006967	Protocadherin-9 precursor	K.VTVLASDGSSTPAR.A	2	3.98	0.46	-4.24
IPI00006967	Protocadherin-9 precursor	K.YTIVSGNNK.G	2	2.41	0.27	1.22
IPI00006967	Protocadherin-9 precursor	R.EELPENVPIGNIPK.D	2	3.31	0.29	-2.76
IPI00006967	Protocadherin-9 precursor	R.SLDREETAIHK.V	3	2.68	0.14	-3.30
IPI00006967	Protocadherin-9 precursor	R.SLDREETAIHKVTVLASDGSSTPAR.A	3	4.67	0.46	-1.80
IPI00006967	Protocadherin-9 precursor	R.YIFGAQVAPATKR.L	2	2.83	0.12	-1.45
IPI00006971	Isoform 1 of Endosialin precursor	R.AACGPSSCYALFPR.R	2	3.82	0.31	-1.95
IPI00006971	Isoform 1 of Endosialin precursor	R.ELGGDLATPR.T	1	2.04	0.20	-3.33
IPI00006971	Isoform 1 of Endosialin precursor	R.LGFRPAEDDPHR.C	2	1.94	0.06	-2.14
IPI00006971	Isoform 1 of Endosialin precursor	R.LGFRPAEDDPHR.C	3	2.84	0.20	-3.35
IPI00006971	Isoform 1 of Endosialin precursor	R.LLWIGLQR.Q	2	2.53	0.11	-1.85
IPI00006971	Isoform 1 of Endosialin precursor	R.TPEEAQRVDSLVGAGPASR.L	2	2.73	0.30	-2.21
IPI00006971	Isoform 1 of Endosialin precursor	R.TPEEAQRVDSLVGAGPASR.L	3	3.83	0.19	-2.98
IPI00006971	Isoform 1 of Endosialin precursor	R.VDSLVGAGPASR.L	2	3.17	0.30	-1.95
IPI00006987	ATP-dependent RNA helicase DDX24	K.RLSGLLKVLDIM*PLTLHACM*HQK.Q	3	3.39	0.10	
IPI00007010	Lysozyme-like protein 6 precursor	K.SYSENLCHVDCQDLLNPNLLAGIHCAK.R	3	3.38	0.06	
IPI00007040	Zinc finger protein 222	K.CGKAFM*HNFQLQKHHR.I	2	1.60	0.06	-5.37

IPI00007047	Protein S100-A8	K.ELDINTDGAVNFQEFLILVIK.M	2	5.67	0.52	-6.15
IPI00007047	Protein S100-A8	K.ELDINTDGAVNFQEFLILVIK.M	3	5.43	0.49	-5.72
IPI00007047	Protein S100-A8	K.GNFHAVYR.D	2	1.83	0.18	-2.08
IPI00007102	Uncharacterized protein C17orf25	K.ILTPLVSLDTPGK.A	2	2.91	0.38	-3.94
IPI00007102	Uncharacterized protein C17orf25	K.IYEKDEEKQR.A	2	1.84	0.18	-0.92
IPI00007102	Uncharacterized protein C17orf25	K.LGNDFM*GITLASSQAVSNAR.K	3	3.75	0.42	-2.58
IPI00007102	Uncharacterized protein C17orf25	K.SLNYWCNLLGM*K.I	2	3.70	0.30	-2.16
IPI00007193	Isoform 2 of Ankyrin repeat domain-containing protein 26	Q.DKMNFDVSNLKDNNEILSQQLFKTESK.L	3	3.55	0.18	-2.15
IPI00007199	Protein Z-dependent protease inhibitor precursor	K.ETSNFGFSLLR.K	2	3.17	0.32	-4.72
IPI00007199	Protein Z-dependent protease inhibitor precursor	K.LFDEINPETK.L	2	3.44	0.22	-3.41
IPI00007199	Protein Z-dependent protease inhibitor precursor	K.LILVDYILFK.G	2	3.11	0.36	-5.07
IPI00007199	Protein Z-dependent protease inhibitor precursor	R.IFSPFADLSELSATGR.N	2	4.76	0.55	-3.26
IPI00007199	Protein Z-dependent protease inhibitor precursor	R.YFDTECVPM*NFR.N	2	1.73	0.14	-3.44
IPI00007221	Plasma serine protease inhibitor precursor	K.AVVEVDESGTR.A	1	1.89	0.17	-2.47
IPI00007221	Plasma serine protease inhibitor precursor	K.AVVEVDESGTR.A	2	3.26	0.45	-2.97
IPI00007221	Plasma serine protease inhibitor precursor	K.FSIEGSYQLEK.V	2	3.23	0.26	-3.65
IPI00007221	Plasma serine protease inhibitor precursor	K.M*QILEGLGLNLQK.S	2	3.47	0.22	
IPI00007221	Plasma serine protease inhibitor precursor	K.M*QQVENGLSEK.T	2	3.00	0.26	-2.07
IPI00007221	Plasma serine protease inhibitor precursor	K.NLDSNAVVIMVNYIFFK.A	2	3.13	0.07	
IPI00007221	Plasma serine protease inhibitor precursor	K.TLYLADTFPTNFR.D	2	3.28	0.28	-3.95
IPI00007221	Plasma serine protease inhibitor precursor	R.AAAATGTIFTFR.S	2	3.86	0.53	-2.70
IPI00007221	Plasma serine protease inhibitor precursor	R.EDQYHYLLDR.N	2	2.69	0.20	
IPI00007221	Plasma serine protease inhibitor precursor	R.GFQQLLQELNQPR.D	2	3.54	0.23	-3.48
IPI00007221	Plasma serine protease inhibitor precursor	R.GFQQLLQELNQPR.D	3	4.16	0.30	-1.37
IPI00007236	Isoform 2 of Neuroligin-1 precursor	K.ELNNEILGPVIQFLGVPYAAPPTGER.R	3	3.16	0.28	-2.88
IPI00007236	Isoform 2 of Neuroligin-1 precursor	K.ELNNEILGPVIQFLGVPYAAPPTGERR.F	3	4.11	0.40	-5.11
IPI00007236	Isoform 2 of Neuroligin-1 precursor	K.ELVDQDIQPAR.Y	2	2.57	0.07	-1.36
IPI00007236	Isoform 2 of Neuroligin-1 precursor	K.FVENIVDSDDGISASDFDFAVSNFVDNLYGYPEGK.D	3	4.56	0.52	-2.11
IPI00007236	Isoform 2 of Neuroligin-1 precursor	K.FVENIVDSDDGISASDFDFAVSNFVDNLYGYPEGKDVLR.E	3	4.98	0.54	-3.34
IPI00007236	Isoform 2 of Neuroligin-1 precursor	K.GNYGLLDLIQALR.W	2	4.12	0.24	-4.60
IPI00007236	Isoform 2 of Neuroligin-1 precursor	K.GNYGLLDLIQALR.W	3	5.31	0.31	-2.89
IPI00007236	Isoform 2 of Neuroligin-1 precursor	K.KPYKELVDQDIQPAR.Y	2	5.15	0.49	-4.43
IPI00007236	Isoform 2 of Neuroligin-1 precursor	K.KPYKELVDQDIQPAR.Y	3	4.83	0.24	-2.99
IPI00007236	Isoform 2 of Neuroligin-1 precursor	R.AIAQSGTALSSWAVSFQPAK.Y	2	5.77	0.49	-3.50
IPI00007236	Isoform 2 of Neuroligin-1 precursor	R.AIAQSGTALSSWAVSFQPAK.Y	3	2.73	0.11	-3.43
IPI00007236	Isoform 2 of Neuroligin-1 precursor	R.LGVLGFLSTGDQAAK.G	2	5.01	0.48	-3.60
IPI00007236	Isoform 2 of Neuroligin-1 precursor	R.WTSENIGFFGGDPLR.I	2	4.52	0.43	-4.08
IPI00007240	Coagulation factor XIII B chain precursor	K.IQTHSTTYR.H	2	1.86	0.13	-2.02
IPI00007240	Coagulation factor XIII B chain precursor	K.VLHGDLIDFVCK.Q	2	2.42	0.16	
IPI00007240	Coagulation factor XIII B chain precursor	R.CFDHHFLEGSR.E	2	2.28	0.15	
IPI00007240	Coagulation factor XIII B chain precursor	R.GDTYPAELYITGSILR.M	2	4.69	0.46	

IPI00007240	Coagulation factor XIII B chain precursor	R.IAQYYYTFK.S	2	2.70	0.14	
IPI00007249	ectonucleotide pyrophosphatase/phosphodiesterase 4	K.KIDDLIGDLVQR.L	2	3.95	0.35	-1.62
IPI00007249	ectonucleotide pyrophosphatase/phosphodiesterase 4	K.KIDDLIGDLVQR.L	3	3.94	0.08	-0.41
IPI00007249	ectonucleotide pyrophosphatase/phosphodiesterase 4	K.LLLVSFDGFR.A	2	3.27	0.23	-4.04
IPI00007249	ectonucleotide pyrophosphatase/phosphodiesterase 4	K.NYEFPHLQNFIK.E	2	3.22	0.32	-4.00
IPI00007249	ectonucleotide pyrophosphatase/phosphodiesterase 4	R.LINLDSCIDHSYYTLIDLSPVAAILPK.I	3	2.66	0.11	2.09
IPI00007257	calsyntenin 1 isoform 2	D.PPLIALDKDAPLR.F	2	3.96	0.32	-4.13
IPI00007257	calsyntenin 1 isoform 2	D.PPLIALDKDAPLR.F	3	3.80	0.38	-2.57
IPI00007257	calsyntenin 1 isoform 2	I.PDGVVSVSPK.E	2	3.32	0.40	-1.49
IPI00007257	calsyntenin 1 isoform 2	K.AM*QHISYLNSR.Q	2	3.52	0.37	-3.84
IPI00007257	calsyntenin 1 isoform 2	K.AM*QHISYLNSR.Q	3	4.15	0.31	-4.11
IPI00007257	calsyntenin 1 isoform 2	K.ATVHIQVNDVNEYAPVFK.E	2	5.28	0.56	-4.37
IPI00007257	calsyntenin 1 isoform 2	K.ATVHIQVNDVNEYAPVFK.E	3	5.17	0.35	-5.40
IPI00007257	calsyntenin 1 isoform 2	K.ATVHIQVNDVNEYAPVFKEK.S	2	5.80	0.50	-5.13
IPI00007257	calsyntenin 1 isoform 2	K.ATVHIQVNDVNEYAPVFKEK.S	3	5.92	0.45	-4.24
IPI00007257	calsyntenin 1 isoform 2	K.ATVHIQVNDVNEYAPVFKEK.S	4	4.80	0.36	-3.41
IPI00007257	calsyntenin 1 isoform 2	K.CFNEATCISVPPVDGYVM*VLQPEEPK.I	3	2.47	0.07	-4.61
IPI00007257	calsyntenin 1 isoform 2	K.CSELNGR.Y	1	1.91	0.16	-1.88
IPI00007257	calsyntenin 1 isoform 2	K.DYSFTIQAYDCGK.G	2	4.68	0.55	-4.60
IPI00007257	calsyntenin 1 isoform 2	K.DYSFTIQAYDCGKGPDGTNVKK.S	3	4.02	0.42	-0.92
IPI00007257	calsyntenin 1 isoform 2	K.DYSFTIQAYDCGKGPDGTNVKK.S	4	2.73	0.35	-1.21
IPI00007257	calsyntenin 1 isoform 2	K.EGLDLQVLEDSGR.G	1	1.82	0.18	-2.66
IPI00007257	calsyntenin 1 isoform 2	K.EGLDLQVLEDSGR.G	2	4.86	0.49	-5.86
IPI00007257	calsyntenin 1 isoform 2	K.EPFTISVWM*R.H	2	2.39	0.35	-4.43
IPI00007257	calsyntenin 1 isoform 2	K.ETILCSSDKTDM*NR.H	2	3.90	0.45	-1.66
IPI00007257	calsyntenin 1 isoform 2	K.ETILCSSDKTDM*NR.H	3	3.03	0.39	-0.98
IPI00007257	calsyntenin 1 isoform 2	K.FKLICSELNGR.Y	3	3.78	0.17	-1.61
IPI00007257	calsyntenin 1 isoform 2	K.GIEVSSSELGM*TFTGVDTM*ASYEEVLHLLR.Y	3	3.83	0.30	-3.11
IPI00007257	calsyntenin 1 isoform 2	K.HKPWLEPTYHGIVTENDNTVLLDPPLIALDKDAPLR.F	4	3.99	0.34	-4.34
IPI00007257	calsyntenin 1 isoform 2	K.IHGQNVPFDAVVVDK.S	2	4.98	0.50	-3.62
IPI00007257	calsyntenin 1 isoform 2	K.IHGQNVPFDAVVVDK.S	3	3.29	0.29	-2.93
IPI00007257	calsyntenin 1 isoform 2	K.IHGQNVPFDAVVVDKSTGEGVIR.S	2	5.01	0.53	-4.89
IPI00007257	calsyntenin 1 isoform 2	K.IHGQNVPFDAVVVDKSTGEGVIR.S	3	5.74	0.52	-5.05
IPI00007257	calsyntenin 1 isoform 2	K.IHGQNVPFDAVVVDKSTGEGVIR.S	4	4.24	0.43	-5.46
IPI00007257	calsyntenin 1 isoform 2	K.ISLSGVHHFAR.A	2	3.16	0.25	-2.13
IPI00007257	calsyntenin 1 isoform 2	K.LICSELNGR.Y	1	2.35	0.29	-2.78
IPI00007257	calsyntenin 1 isoform 2	K.LICSELNGR.Y	2	2.51	0.22	2.09

IPI00007257	calsyntenin 1 isoform 2	K.LNYGKEHQYK.L	1	2.45	0.27	-6.29
IPI00007257	calsyntenin 1 isoform 2	K.LNYGKEHQYK.L	2	3.53	0.36	-4.17
IPI00007257	calsyntenin 1 isoform 2	K.LTVTAYDCGK.K	1	2.13	0.40	-3.69
IPI00007257	calsyntenin 1 isoform 2	K.LTVTAYDCGK.K	2	3.72	0.35	-2.63
IPI00007257	calsyntenin 1 isoform 2	K.LTVTAYDCGKK.R	2	2.44	0.21	
IPI00007257	calsyntenin 1 isoform 2	K.NTEKLNYGK.E	1	2.52	0.17	-2.09
IPI00007257	calsyntenin 1 isoform 2	K.NTEKLNYGK.E	2	2.87	0.30	-1.43
IPI00007257	calsyntenin 1 isoform 2	K.NTEKLNYGKEHQYK.L	2	3.65	0.38	-4.44
IPI00007257	calsyntenin 1 isoform 2	K.NTEKLNYGKEHQYK.L	3	2.92	0.23	-3.48
IPI00007257	calsyntenin 1 isoform 2	K.RATEDVLVK.I	2	2.42	0.09	-0.67
IPI00007257	calsyntenin 1 isoform 2	K.STGEGVIR.S	1	1.48	0.05	-2.72
IPI00007257	calsyntenin 1 isoform 2	K.STGEGVIR.S	2	2.16	0.07	-2.74
IPI00007257	calsyntenin 1 isoform 2	K.SYKATVIEGK.Q	1	2.51	0.07	-1.49
IPI00007257	calsyntenin 1 isoform 2	K.SYKATVIEGK.Q	2	2.66	0.17	-3.09
IPI00007257	calsyntenin 1 isoform 2	K.VEVNVIHTANPM*EHANH.M	2	4.80	0.54	-3.09
IPI00007257	calsyntenin 1 isoform 2	K.VIDCLYTCK.E	1	2.08	0.21	-3.75
IPI00007257	calsyntenin 1 isoform 2	K.VIDCLYTCK.E	2	3.65	0.36	-3.48
IPI00007257	calsyntenin 1 isoform 2	K.VIDCLYTCKEGLDLQVLEDSGR.G	2	4.80	0.45	-4.58
IPI00007257	calsyntenin 1 isoform 2	K.VIDCLYTCKEGLDLQVLEDSGR.G	3	5.50	0.46	-2.92
IPI00007257	calsyntenin 1 isoform 2	M.AAQPQFVHPEHR.S	2	3.19	0.37	-4.60
IPI00007257	calsyntenin 1 isoform 2	P.DGVVSVSPKEPFTISVWM*R.H	2	3.29	0.26	-4.51
IPI00007257	calsyntenin 1 isoform 2	P.DGVVSVSPKEPFTISVWM*R.H	3	3.93	0.41	-1.89
IPI00007257	calsyntenin 1 isoform 2	Q.PQFVHPEHR.S	2	2.92	0.32	-3.59
IPI00007257	calsyntenin 1 isoform 2	R.AASEFESSEGVFLFPELR.I	2	4.71	0.45	-8.90
IPI00007257	calsyntenin 1 isoform 2	R.AASEFESSEGVFLFPELR.I	3	5.03	0.31	-4.67
IPI00007257	calsyntenin 1 isoform 2	R.ATEDVLVK.I	1	2.30	0.10	-2.76
IPI00007257	calsyntenin 1 isoform 2	R.ATEDVLVK.I	2	2.79	0.10	-2.54
IPI00007257	calsyntenin 1 isoform 2	R.FAGEICGFK.I	1	2.01	0.21	-2.18
IPI00007257	calsyntenin 1 isoform 2	R.FAGEICGFK.I	2	2.13	0.19	-1.42
IPI00007257	calsyntenin 1 isoform 2	R.GNLAGLTLR.S	1	1.65	0.11	-1.41
IPI00007257	calsyntenin 1 isoform 2	R.GNLAGLTLR.S	2	3.34	0.37	-2.13
IPI00007257	calsyntenin 1 isoform 2	R.GVQIQAHPSQLVLTLEGEDLGELDK.A	2	5.85	0.47	-2.90
IPI00007257	calsyntenin 1 isoform 2	R.GVQIQAHPSQLVLTLEGEDLGELDK.A	3	6.12	0.40	-5.09
IPI00007257	calsyntenin 1 isoform 2	R.GVQIQAHPSQLVLTLEGEDLGELDK.A	4	5.78	0.39	-2.81
IPI00007257	calsyntenin 1 isoform 2	R.GVQIQAHPSQLVLTLEGEDLGELDKAM*QHISYLNSR.Q	3	4.69	0.55	-3.96
IPI00007257	calsyntenin 1 isoform 2	R.GVQIQAHPSQLVLTLEGEDLGELDKAM*QHISYLNSR.Q	4	8.33	0.54	-6.93
IPI00007257	calsyntenin 1 isoform 2	R.GVQIQAHPSQLVLTLEGEDLGELDKAM*QHISYLNSR.Q	5	4.44	0.43	-5.15
IPI00007257	calsyntenin 1 isoform 2	R.IEYEPGTGALAVFPNIHLETCDEPVASVQATVELETSHIGK.G	4	3.22	0.21	-3.28
IPI00007257	calsyntenin 1 isoform 2	R.IPDGVVSVSPK.E	1	2.03	0.33	-3.87
IPI00007257	calsyntenin 1 isoform 2	R.IPDGVVSVSPK.E	2	3.14	0.38	-2.96
IPI00007257	calsyntenin 1 isoform 2	R.IPDGVVSVSPKEPF.T	2	4.05	0.47	-2.64
IPI00007257	calsyntenin 1 isoform 2	R.IPDGVVSVSPKEPFTISVWM*R.H	3	4.68	0.55	-3.17

IPI00007257	calsyntenin 1 isoform 2	R.LIFLFRQDPSEEKK.Y	2	2.48	0.10	-2.58
IPI00007257	calsyntenin 1 isoform 2	R.LIFLFRQDPSEEKK.Y	3	3.12	0.18	-2.10
IPI00007257	calsyntenin 1 isoform 2	R.LKITSTIK.C	1	2.09	0.21	-4.13
IPI00007257	calsyntenin 1 isoform 2	R.SFVDLSGHNLANPHPF.A	2	4.17	0.48	-3.37
IPI00007257	calsyntenin 1 isoform 2	R.SFVDLSGHNLANPHPF.A	3	4.84	0.50	-2.07
IPI00007257	calsyntenin 1 isoform 2	R.SFVDLSGHNLANPHPFAVVPSTA.T	2	4.47	0.57	-3.64
IPI00007257	calsyntenin 1 isoform 2	R.SFVDLSGHNLANPHPFAVVPSTAT.V	2	4.76	0.52	-4.11
IPI00007257	calsyntenin 1 isoform 2	R.SFVDLSGHNLANPHPFAVVPSTATV.V	2	5.00	0.54	-3.24
IPI00007257	calsyntenin 1 isoform 2	R.SFVDLSGHNLANPHPFAVVPSTATVV.I	2	3.98	0.37	-2.45
IPI00007257	calsyntenin 1 isoform 2	R.SFVDLSGHNLANPHPFAVVPSTATVVI.V	2	4.17	0.38	-2.51
IPI00007257	calsyntenin 1 isoform 2	R.VEAVDADCSPQFSQICSYEIITPDVPFTVDKDGYIK.N	3	5.78	0.61	-3.95
IPI00007257	calsyntenin 1 isoform 2	R.VEAVDADCSPQFSQICSYEIITPDVPFTVDKDGYIK.N	4	4.54	0.37	-3.84
IPI00007257	calsyntenin 1 isoform 2	R.VEAVDADCSPQFSQICSYEIITPDVPFTVDKDGYIKNTEK.L	4	5.79	0.35	-2.78
IPI00007257	calsyntenin 1 isoform 2	R.YISNEFK.V	1	2.32	0.12	-1.62
IPI00007257	calsyntenin 1 isoform 2	V.PFDAVVVDK.S	1	2.67	0.19	-5.98
IPI00007257	calsyntenin 1 isoform 2	V.PFDAVVVDK.S	2	3.54	0.24	-1.24
IPI00007257	calsyntenin 1 isoform 2	W.LEPTYHGIVTENDNTVLLDPPLIALDK.D	3	4.10	0.29	-4.31
IPI00007257	calsyntenin 1 isoform 2	W.LEPTYHGIVTENDNTVLLDPPLIALDKDAPLR.F	3	6.13	0.53	-4.49
IPI00007257	calsyntenin 1 isoform 2	W.LEPTYHGIVTENDNTVLLDPPLIALDKDAPLR.F	4	4.50	0.42	-3.77
IPI00007277	Isoform 1 of Leucine-rich repeat flightless-interacting protein 2	R.SSPGFTNDDTASIVSSDRASRGR.R	3	1.89	0.13	1.48
IPI00007321	Isoform 1 of Acyl-protein thioesterase 1	K.LKTLVNPANVTFKTYEGMMHSSCQQEMMDVK.Q	3	2.95	0.12	
IPI00007402	Importin-7	K.QLQDIATLADQRRAAHESKMIEK.H	3	3.37	0.08	
IPI00007425	desmocollin 1 isoform Dsc1b preproprotein	K.VNLEECLK.S	2	2.36	0.15	-2.14
IPI00007425	desmocollin 1 isoform Dsc1b preproprotein	R.ILEDGSIYTTHDLILSSER.K	3	3.29	0.24	-2.99
IPI00007425	desmocollin 1 isoform Dsc1b preproprotein	R.KSFSIFLSDGQR.R	3	3.05	0.10	-2.84
IPI00007425	desmocollin 1 isoform Dsc1b preproprotein	R.VPSHLQAETLVGK.V	2	3.43	0.33	-2.40
IPI00007512	Glutathione transferase omega-2	K.LFPYDPYERARQKM*LLELFCKVPHLTK.E	4	2.97	0.21	-7.94
IPI00007617	Olfactory receptor 52A1	K.LAAANVQVNK.I	2	2.21	0.10	
IPI00007682	Vacuolar ATP synthase catalytic subunit A	K.EILQEEEDLAEIVQLVGK.A	3	2.35	0.16	-3.02
IPI00007702	Heat shock-related 70 kDa protein 2	K.DIGPNKR.A	2	1.28	0.05	0.61
IPI00007702	Heat shock-related 70 kDa protein 2	K.ITITNDKGR.L	1	2.08	0.20	-3.55
IPI00007702	Heat shock-related 70 kDa protein 2	K.ITITNDKGR.L	2	2.65	0.24	-2.81
IPI00007702	Heat shock-related 70 kDa protein 2	K.NALESYTYNIK.Q	2	3.28	0.35	-2.82
IPI00007702	Heat shock-related 70 kDa protein 2	K.VQVEYKGETK.S	2	2.40	0.21	-2.65
IPI00007702	Heat shock-related 70 kDa protein 2	R.LIGDAAK.N	1	1.96	0.12	-2.67
IPI00007702	Heat shock-related 70 kDa protein 2	R.NQM*AEKDEYEHK.Q	3	3.05	0.19	-1.09
IPI00007702	Heat shock-related 70 kDa protein 2	R.NVLIFDLGGGTFDVSILTIEDGIFEVK.S	3	3.32	0.29	-3.03
IPI00007702	Heat shock-related 70 kDa protein 2	R.TTPSYVAFTDTER.L	2	2.87	0.41	-0.70
IPI00007709	Isoform 1 of ADAM 28 precursor	A.KEPEQQEQFETELK.Y	3	3.60	0.28	-0.36
IPI00007709	Isoform 1 of ADAM 28 precursor	K.IAVLYLK.K	2	2.39	0.21	-3.23
IPI00007709	Isoform 1 of ADAM 28 precursor	K.IAVLYLKK.N	2	2.51	0.16	-1.99

IPI00007709	Isoform 1 of ADAM 28 precursor	R.YFIEPLSPIHR.D	3	3.02	0.31	-2.50
IPI00007750	Tubulin alpha-4A chain	K.AYHEQLSVAEITNACFEPANQMVK.C	3	3.76	0.34	-2.56
IPI00007750	Tubulin alpha-4A chain	K.LSDQCTGLQGFLVFHSFGGGTGSGFTSLLMER.L	3	3.78	0.38	-3.69
IPI00007750	Tubulin alpha-4A chain	R.AFVHWYVGEGMEEGEFSEAR.E	3	4.15	0.38	-2.46
IPI00007750	Tubulin alpha-4A chain	R.AVCMLSNTTAIAEAWAR.L	2	4.25	0.45	-1.86
IPI00007750	Tubulin alpha-4A chain	R.AVCMLSNTTAIAEAWAR.L	3	3.49	0.23	-0.91
IPI00007750	Tubulin alpha-4A chain	R.FDGALNVDLTEFQTNLVPYPR.I	2	5.18	0.51	-1.99
IPI00007750	Tubulin alpha-4A chain	R.FDGALNVDLTEFQTNLVPYPR.I	3	2.65	0.18	-2.07
IPI00007750	Tubulin alpha-4A chain	R.LISQIVSSITASLR.F	2	3.75	0.45	-2.19
IPI00007750	Tubulin alpha-4A chain	R.LISQIVSSITASLR.F	3	2.59	0.17	-0.56
IPI00007752	Tubulin beta-2C chain	K.GHYTEGAELVDSVLDVVR.K	2	2.94	0.28	
IPI00007752	Tubulin beta-2C chain	K.GHYTEGAELVDSVLDVVR.K	3	3.28	0.30	-3.01
IPI00007752	Tubulin beta-2C chain	K.GHYTEGAELVDSVLDVVRK.E	3	3.51	0.45	-3.20
IPI00007752	Tubulin beta-2C chain	K.NM*M*AACDPR.H	2	1.73	0.12	0.11
IPI00007752	Tubulin beta-2C chain	K.VSDTVVEPYNATLSVHQLVENTDETYCIDNEALYDICFR.T	3	4.91	0.43	-2.95
IPI00007752	Tubulin beta-2C chain	K.VSDTVVEPYNATLSVHQLVENTDETYCIDNEALYDICFR.T	4	4.05	0.34	-2.05
IPI00007752	Tubulin beta-2C chain	R.ISEQFTAM*FR.R	2	2.17	0.13	-2.65
IPI00007752	Tubulin beta-2C chain	R.ISEQFTAMFR.R	2	2.87	0.27	-0.81
IPI00007752	Tubulin beta-2C chain	R.LHFFM*PGFAPLTSR.G	3	3.20	0.06	-3.00
IPI00007752	Tubulin beta-2C chain	R.LHFFMPGFAPLTSR.G	3	2.62	0.11	-1.73
IPI00007778	Di-N-acetylchitobiase precursor	K.ATYIQNYR.L	1	1.93	0.16	0.53
IPI00007778	Di-N-acetylchitobiase precursor	K.ATYIQNYR.L	2	2.59	0.28	-0.42
IPI00007778	Di-N-acetylchitobiase precursor	K.ETTDSFHR.E	2	1.92	0.15	-1.84
IPI00007778	Di-N-acetylchitobiase precursor	K.GDVSLKDIIDPAFR.A	2	2.82	0.36	-1.12
IPI00007778	Di-N-acetylchitobiase precursor	K.GDVSLKDIIDPAFR.A	3	3.58	0.07	-1.01
IPI00007778	Di-N-acetylchitobiase precursor	K.M*SINPK.K	1	1.35	0.06	-2.57
IPI00007778	Di-N-acetylchitobiase precursor	K.SYDWSQITTVATFGK.Y	2	5.57	0.55	-2.11
IPI00007778	Di-N-acetylchitobiase precursor	R.HHPDFEVFVFDVGQK.T	2	3.80	0.39	-3.21
IPI00007778	Di-N-acetylchitobiase precursor	R.HHPDFEVFVFDVGQK.T	3	3.98	0.30	-4.07
IPI00007778	Di-N-acetylchitobiase precursor	R.HHPDFEVFVFDVGQK.T	4	3.16	0.18	-3.19
IPI00007778	Di-N-acetylchitobiase precursor	R.VVLKGDVSLKDIIDPAFR.A	3	3.89	0.48	-3.19
IPI00007778	Di-N-acetylchitobiase precursor	R.VVLKGDVSLKDIIDPAFR.A	4	2.63	0.31	-2.28
IPI00007778	Di-N-acetylchitobiase precursor	W.YDNPQSISLK.A	2	2.94	0.26	-0.40
IPI00007797	Fatty acid-binding protein, epidermal	K.ELGVGIALR.K	2	2.35	0.08	-2.18
IPI00007797	Fatty acid-binding protein, epidermal	K.FEETTADGRK.T	2	2.37	0.11	-1.15
IPI00007797	Fatty acid-binding protein, epidermal	K.GFDEYM*K.E	1	1.50	0.08	-2.73
IPI00007797	Fatty acid-binding protein, epidermal	K.LVVECVM*NNVTCTR.I	2	4.11	0.42	-2.93
IPI00007797	Fatty acid-binding protein, epidermal	K.TTQFSCTLGEK.F	2	2.59	0.19	-2.67
IPI00007798	Thyrotropin-releasing hormone-degrading ectoenzyme	K.IIYNALIENELLGFFR.S	2	4.42	0.47	-1.64
IPI00007798	Thyrotropin-releasing hormone-degrading ectoenzyme	K.IIYNALIENELLGFFR.S	3	2.48	0.22	-2.68

IPI00007798	Thyrotropin-releasing hormone-degrading ectoenzyme	K.M*LYQDELFQWLGK.A	2	4.77	0.45	-4.22
IPI00007798	Thyrotropin-releasing hormone-degrading ectoenzyme	K.NYDGVAAASFSR.A	2	3.59	0.48	-2.79
IPI00007798	Thyrotropin-releasing hormone-degrading ectoenzyme	R.AGLIDDAFSLAR.A	2	4.27	0.25	-3.61
IPI00007798	Thyrotropin-releasing hormone-degrading ectoenzyme	R.AGYLPQNIPLEIIR.Y	2	3.49	0.39	-3.31
IPI00007798	Thyrotropin-releasing hormone-degrading ectoenzyme	R.FLGVTQFSPTHAR.K	2	2.64	0.29	-4.23
IPI00007798	Thyrotropin-releasing hormone-degrading ectoenzyme	R.FLTDVLHEVM*LLDGLASSHPVSQEVLQATDIDR.V	4	3.30	0.22	-2.88
IPI00007798	Thyrotropin-releasing hormone-degrading ectoenzyme	R.YVVLHASR.V	2	2.35	0.20	-4.00
IPI00007812	Vacuolar ATP synthase subunit B, brain isoform	K.AVVGEEALTSDDLLYLEFLQK.F	2	5.58	0.51	-2.50
IPI00007812	Vacuolar ATP synthase subunit B, brain isoform	K.AVVGEEALTSDDLLYLEFLQK.F	3	2.78	0.19	-0.94
IPI00007812	Vacuolar ATP synthase subunit B, brain isoform	K.SKDVVDYSEENFAIVFAAMGVNMETAR.F	3	3.11	0.09	-2.16
IPI00007834	Isoform 1 of Ankyrin-2	R.LRCFCM*TDDKVDKTLEQQENFAEVAR.S	3	2.66	0.09	-5.08
IPI00007853	Gamma-interferon-inducible lysosomal thiol reductase precursor	A.SPLQALDFFGNGPPVNYK.T	2	5.86	0.51	-4.66
IPI00007853	Gamma-interferon-inducible lysosomal thiol reductase precursor	A.SPLQALDFFGNGPPVNYK.T	3	4.43	0.40	-1.55
IPI00007853	Gamma-interferon-inducible lysosomal thiol reductase precursor	K.TGNLYLR.G	2	2.62	0.07	-1.92
IPI00007921	Isoform 1 of Neurexin-2-alpha precursor	G.LEFGGGPGQWAR.Y	1	2.25	0.21	-2.15
IPI00007921	Isoform 1 of Neurexin-2-alpha precursor	G.LEFGGGPGQWAR.Y	2	3.51	0.36	-3.09
IPI00007921	Isoform 1 of Neurexin-2-alpha precursor	K.DGDITYCELNAR.F	2	3.78	0.38	-6.71
IPI00007921	Isoform 1 of Neurexin-2-alpha precursor	K.DVVYK.N	1	1.46	0.08	-3.57
IPI00007921	Isoform 1 of Neurexin-2-alpha precursor	K.GKEEFVATFK.G	1	2.39	0.30	-4.24
IPI00007921	Isoform 1 of Neurexin-2-alpha precursor	K.GKEEFVATFK.G	2	2.77	0.35	-2.75
IPI00007921	Isoform 1 of Neurexin-2-alpha precursor	K.GNEFFCYDLSHNPIQSSTDEITLAFR.T	3	5.25	0.53	-1.36
IPI00007921	Isoform 1 of Neurexin-2-alpha precursor	K.GPETLFAGHKLNDNEWHTVRVVR.R	3	2.74	0.07	2.07
IPI00007921	Isoform 1 of Neurexin-2-alpha precursor	K.GSISVNSR.S	2	2.03	0.09	-2.77
IPI00007921	Isoform 1 of Neurexin-2-alpha precursor	K.IGGRDQGRPFQGQVSGLYYNGLK.V	3	4.33	0.40	-2.79
IPI00007921	Isoform 1 of Neurexin-2-alpha precursor	K.IGGRDQGRPFQGQVSGLYYNGLK.V	4	3.18	0.18	-1.26
IPI00007921	Isoform 1 of Neurexin-2-alpha precursor	K.IM*LPNAM*HTEAEDVSLR.F	2	4.27	0.45	-3.86
IPI00007921	Isoform 1 of Neurexin-2-alpha precursor	K.LGERPPALLGSQGLR.G	2	3.23	0.35	-4.01
IPI00007921	Isoform 1 of Neurexin-2-alpha precursor	K.LGERPPALLGSQGLR.G	3	4.48	0.34	-2.69
IPI00007921	Isoform 1 of Neurexin-2-alpha precursor	K.LQGDLSFR.C	1	1.94	0.12	-3.85
IPI00007921	Isoform 1 of Neurexin-2-alpha precursor	K.LQGDLSFR.C	2	2.81	0.15	-2.61
IPI00007921	Isoform 1 of Neurexin-2-alpha precursor	K.NM*FSNLPK.L	2	2.15	0.11	0.24
IPI00007921	Isoform 1 of Neurexin-2-alpha precursor	K.NNDFKLELSR.L	2	2.82	0.13	-2.90

IPI00007921	Isoform 1 of Neurexin-2-alpha precursor	K.SADYVNLSLK.S	1	2.04	0.21	-2.90
IPI00007921	Isoform 1 of Neurexin-2-alpha precursor	K.SADYVNLSLK.S	2	3.46	0.35	-2.53
IPI00007921	Isoform 1 of Neurexin-2-alpha precursor	K.VLALAAESDPNVR.T	2	4.70	0.50	-2.98
IPI00007921	Isoform 1 of Neurexin-2-alpha precursor	K.VLALAAESDPNVR.T	3	3.23	0.22	-2.13
IPI00007921	Isoform 1 of Neurexin-2-alpha precursor	K.VLALAAESDPNVRTEGHLR.L	3	3.47	0.36	-3.22
IPI00007921	Isoform 1 of Neurexin-2-alpha precursor	L.TLNSEVGSLLFSEGGAGR.G	2	3.70	0.36	-3.90
IPI00007921	Isoform 1 of Neurexin-2-alpha precursor	R.AIVADPVTFK.S	2	3.03	0.32	-2.78
IPI00007921	Isoform 1 of Neurexin-2-alpha precursor	R.AYGLM*M*ATTSR.E	2	3.21	0.45	-2.56
IPI00007921	Isoform 1 of Neurexin-2-alpha precursor	R.CEDVAALDPVTFESPEAFVALPR.W	2	6.43	0.55	-5.65
IPI00007921	Isoform 1 of Neurexin-2-alpha precursor	R.CEDVAALDPVTFESPEAFVALPR.W	3	4.18	0.45	-4.21
IPI00007921	Isoform 1 of Neurexin-2-alpha precursor	R.DGFQGCLASVDLNGR.L	2	4.89	0.57	-5.11
IPI00007921	Isoform 1 of Neurexin-2-alpha precursor	R.DGFQGCLASVDLNGRLPDLIADALHR.I	3	4.53	0.44	-3.83
IPI00007921	Isoform 1 of Neurexin-2-alpha precursor	R.DLFIDGR.S	1	2.11	0.13	-1.89
IPI00007921	Isoform 1 of Neurexin-2-alpha precursor	R.DPGNVHTLK.I	2	1.68	0.17	-1.83
IPI00007921	Isoform 1 of Neurexin-2-alpha precursor	R.DQGRPFQGQVSGLYYNGLK.V	2	4.64	0.19	-2.47
IPI00007921	Isoform 1 of Neurexin-2-alpha precursor	R.DQGRPFQGQVSGLYYNGLK.V	3	5.32	0.30	-1.75
IPI00007921	Isoform 1 of Neurexin-2-alpha precursor	R.EATVLSYDGSM*YM*K.I	2	4.74	0.13	-3.25
IPI00007921	Isoform 1 of Neurexin-2-alpha precursor	R.EM*QVASDLFVGGIPPDVR.L	2	4.24	0.40	-4.43
IPI00007921	Isoform 1 of Neurexin-2-alpha precursor	R.EM*QVASDLFVGGIPPDVR.L	3	3.31	0.14	-3.29
IPI00007921	Isoform 1 of Neurexin-2-alpha precursor	R.ESADTLRLELDGGQM*K.L	2	2.41	0.14	-3.70
IPI00007921	Isoform 1 of Neurexin-2-alpha precursor	R.ESADTLRLELDGGQM*K.L	3	2.36	0.09	-2.34
IPI00007921	Isoform 1 of Neurexin-2-alpha precursor	R.FICDCIGTGFLGR.V	2	4.11	0.51	-4.28
IPI00007921	Isoform 1 of Neurexin-2-alpha precursor	R.GATADPLCAPAR.N	2	2.30	0.25	-3.54
IPI00007921	Isoform 1 of Neurexin-2-alpha precursor	R.GLAEAQGAVGVAPFCSR.E	2	5.11	0.45	-3.00
IPI00007921	Isoform 1 of Neurexin-2-alpha precursor	R.KGSISVNSR.S	2	1.64	0.07	-2.32
IPI00007921	Isoform 1 of Neurexin-2-alpha precursor	R.KPAPRPNLR.T	2	1.93	0.06	-2.76
IPI00007921	Isoform 1 of Neurexin-2-alpha precursor	R.LAVGFSTHQR.S	1	1.86	0.11	-2.56
IPI00007921	Isoform 1 of Neurexin-2-alpha precursor	R.LAVGFSTHQR.S	2	3.08	0.24	-2.06
IPI00007921	Isoform 1 of Neurexin-2-alpha precursor	R.LEFHNIETGIM*TER.R	2	3.26	0.34	-1.90
IPI00007921	Isoform 1 of Neurexin-2-alpha precursor	R.LEFHNIETGIM*TER.R	3	4.00	0.31	-1.30
IPI00007921	Isoform 1 of Neurexin-2-alpha precursor	R.LPDLIADALHR.I	2	3.73	0.31	-1.40
IPI00007921	Isoform 1 of Neurexin-2-alpha precursor	R.LPDLIADALHR.I	3	3.84	0.34	-2.68
IPI00007921	Isoform 1 of Neurexin-2-alpha precursor	R.LSALTLSTVK.Y	1	1.55	0.15	-3.39
IPI00007921	Isoform 1 of Neurexin-2-alpha precursor	R.LSALTLSTVK.Y	2	3.42	0.30	-3.00
IPI00007921	Isoform 1 of Neurexin-2-alpha precursor	R.LSALTLSTVKYEPPFR.G	2	4.12	0.41	-3.10
IPI00007921	Isoform 1 of Neurexin-2-alpha precursor	R.LSALTLSTVKYEPPFR.G	3	3.74	0.37	-1.88
IPI00007921	Isoform 1 of Neurexin-2-alpha precursor	R.LSALTLSTVKYEPPFRGLLANLK.L	3	3.18	0.18	-5.31
IPI00007921	Isoform 1 of Neurexin-2-alpha precursor	R.NGLM*LHTGK.S	2	2.94	0.13	
IPI00007921	Isoform 1 of Neurexin-2-alpha precursor	R.NPCANGGLCTVLAPGEVGCDCSHTGFGGK.F	3	5.18	0.50	-3.29
IPI00007921	Isoform 1 of Neurexin-2-alpha precursor	R.NYISNSAQSNGAVVK.E	2	4.83	0.42	-1.90
IPI00007921	Isoform 1 of Neurexin-2-alpha precursor	R.QLTIFNSQAAIK.I	1	1.61	0.18	-2.57
IPI00007921	Isoform 1 of Neurexin-2-alpha precursor	R.QLTIFNSQAAIK.I	2	4.01	0.32	-3.86

IPI00007921	Isoform 1 of Neurexin-2-alpha precursor	R.RTALAVDGEAR.A	2	2.42	0.11	
IPI00007921	Isoform 1 of Neurexin-2-alpha precursor	R.TALAVDGEAR.A	1	2.25	0.26	-3.59
IPI00007921	Isoform 1 of Neurexin-2-alpha precursor	R.TALAVDGEAR.A	2	3.67	0.27	-3.37
IPI00007921	Isoform 1 of Neurexin-2-alpha precursor	R.TGSISLDFR.T	2	2.60	0.24	-1.36
IPI00007921	Isoform 1 of Neurexin-2-alpha precursor	R.TTEPNGLLLFSQGR.R	2	4.03	0.33	-4.20
IPI00007921	Isoform 1 of Neurexin-2-alpha precursor	R.TTEPNGLLLFSQGR.R	3	2.20	0.13	-2.83
IPI00007921	Isoform 1 of Neurexin-2-alpha precursor	R.VDLPLPPEVWTAALR.A	2	3.51	0.41	-5.42
IPI00007921	Isoform 1 of Neurexin-2-alpha precursor	R.VVDEWLLDKGR.Q	3	1.39	0.17	-1.81
IPI00007921	Isoform 1 of Neurexin-2-alpha precursor	R.WAGAASSGELSFSLR.T	2	3.07	0.21	-7.41
IPI00007921	Isoform 1 of Neurexin-2-alpha precursor	R.YPAGNFDNER.L	2	3.12	0.31	-1.90
IPI00007928	Pre-mRNA-processing-splicing factor 8	R.DIILGM*EISAPSQQRQQIAEIEK.Q	3	3.19	0.10	
IPI00007960	Isoform 1 of Periostin precursor	K.DIVTNNGVIHLIDQVLIPDSAK.Q	3	3.13	0.24	-5.34
IPI00007960	Isoform 1 of Periostin precursor	R.AAAITSDILEALGR.D	2	4.16	0.44	-3.09
IPI00007960	Isoform 1 of Periostin precursor	R.VLTQIGTSIQDFIEAEDDLSSFR.A	3	3.41	0.38	-3.42
IPI00008085	Zinc transporter ZIP10 precursor	K.LLTNLGLGER.K	2	2.94	0.28	-1.63
IPI00008085	Zinc transporter ZIP10 precursor	R.GHQDLDPDNEGELR.H	3	3.63	0.25	-0.13
IPI00008087	Follistatin-related protein 5 precursor	K.DKFIYVAQPTLDR.V	3	2.72	0.13	-0.79
IPI00008087	Follistatin-related protein 5 precursor	K.NGIDITPK.L	2	2.35	0.26	-3.65
IPI00008087	Follistatin-related protein 5 precursor	K.TLANILWR.E	2	2.99	0.14	
IPI00008087	Follistatin-related protein 5 precursor	K.VIQPIECEFQR.H	2	2.50	0.28	-2.87
IPI00008087	Follistatin-related protein 5 precursor	K.VVQAVSTDPVPVK.L	2	3.47	0.45	-3.41
IPI00008087	Follistatin-related protein 5 precursor	K.YIM*QENENPNGDDISR.K	2	5.31	0.50	-3.36
IPI00008087	Follistatin-related protein 5 precursor	R.QIQDSGLFGQYLM*TPSKDSLFILDGR.L	3	4.49	0.47	-2.28
IPI00008087	Follistatin-related protein 5 precursor	R.VLIVDVQSQK.V	2	3.02	0.12	-3.26
IPI00008087	Follistatin-related protein 5 precursor	R.YEDTGAYTCIAK.N	2	4.05	0.39	
IPI00008091	Putative DNA helicase INO80 complex homolog 1	K.RDMGHDGIQEEILR.K	3	2.22	0.17	1.77
IPI00008107	Leucine-rich repeat and fibronectin type-III domain-containing protein 2 precursor	K.AIGDPSPLIHWVAPDDR.L	3	2.32	0.21	-1.74
IPI00008107	Leucine-rich repeat and fibronectin type-III domain-containing protein 2 precursor	R.M*VNLHQLSLDHNLLDHIAEGTFADLQK.L	4	3.19	0.11	-4.43
IPI00008107	Leucine-rich repeat and fibronectin type-III domain-containing protein 2 precursor	R.NTISHIQPFSFLDLESLR.S	3	4.48	0.40	-1.33
IPI00008107	Leucine-rich repeat and fibronectin type-III domain-containing protein 2 precursor	R.SLHLDSNRLPSLGEDTLR.G	3	3.84	0.41	-2.94
IPI00008148	Isoform 1 of GDNF family receptor alpha-1 precursor	K.EGLGASSHITTK.S	2	2.46	0.27	-2.42
IPI00008148	Isoform 1 of GDNF family receptor alpha-1 precursor	R.SAYITPCTTSVSNDVCNR.R	2	1.81	0.06	-2.92
IPI00008164	Prolyl endopeptidase	R.VFLDPNILSDDGTVALR.G	2	4.28	0.39	-4.82
IPI00008164	Prolyl endopeptidase	R.YFYFYNTGLQNQR.V	2	4.59	0.48	-2.64
IPI00008202	Headcase protein homolog	R.LDLSELLTHIPRHKLNTFHVRMEDDAQVGQGEDLR.K	6	2.95	0.19	1.39
IPI00008202	Headcase protein homolog	R.WDGSWHQLGTMYTYDILAASPCCQARLNCK.H	3	2.73	0.05	0.01

IPI00008207	Endoplasmic reticulum mannosyl-oligosaccharide 1,2-alpha-mannosidase	K.IPYSDVNIGTGVAHPPR.W	3	3.74	0.40	-2.39
IPI00008207	Endoplasmic reticulum mannosyl-oligosaccharide 1,2-alpha-mannosidase	K.YQDWGWEILQSFSR.F	2	4.71	0.49	-4.77
IPI00008207	Endoplasmic reticulum mannosyl-oligosaccharide 1,2-alpha-mannosidase	K.YQDWGWEILQSFSR.F	3	3.78	0.30	-4.36
IPI00008207	Endoplasmic reticulum mannosyl-oligosaccharide 1,2-alpha-mannosidase	R.DKM*ESFFLGETLK.Y	2	3.26	0.31	-2.81
IPI00008207	Endoplasmic reticulum mannosyl-oligosaccharide 1,2-alpha-mannosidase	R.DKM*ESFFLGETLK.Y	3	3.31	0.25	-0.06
IPI00008207	Endoplasmic reticulum mannosyl-oligosaccharide 1,2-alpha-mannosidase	R.FTRVPSGGYSSINNVQDPQKPEPR.D	3	5.08	0.44	-3.60
IPI00008207	Endoplasmic reticulum mannosyl-oligosaccharide 1,2-alpha-mannosidase	R.TPSKIPYSDVNIGTGVAHPPR.W	4	4.30	0.44	-2.64
IPI00008207	Endoplasmic reticulum mannosyl-oligosaccharide 1,2-alpha-mannosidase	R.VPSGGYSSINNVQDPQKPEPR.D	3	3.17	0.36	-2.07
IPI00008215	NADP-dependent malic enzyme	K.AIVVTDGER.I	2	2.25	0.12	-2.83
IPI00008215	NADP-dependent malic enzyme	K.DM*AAFNERPIIFALSNPTSK.A	3	4.16	0.34	-3.99
IPI00008215	NADP-dependent malic enzyme	K.TATVYPEPQNK.E	2	2.74	0.25	-3.51
IPI00008215	NADP-dependent malic enzyme	R.QITDNIFLTTAEVIAQQVSDK.H	3	4.33	0.42	-4.41
IPI00008215	NADP-dependent malic enzyme	R.QQLNIHGLLPPSFNSQEIQVLR.V	3	3.63	0.23	-1.63
IPI00008223	UV excision repair protein RAD23 homolog B	K.EKIESEKGKDAFPVAGQK.L	3	2.97	0.29	-3.26
IPI00008223	UV excision repair protein RAD23 homolog B	K.IESEKGKDAFPVAGQK.L	3	2.76	0.10	-5.74
IPI00008223	UV excision repair protein RAD23 homolog B	K.TLQQQTFK.I	2	2.49	0.13	-1.19
IPI00008223	UV excision repair protein RAD23 homolog B	R.QIIQQNPSLLPALLQQIGR.E	3	3.72	0.27	-3.68
IPI00008226	73 kDa protein	K.EEDEDYPSEDIEGEDQEDK.E	2	2.47	0.17	
IPI00008274	Adenylyl cyclase-associated protein 1	K.AGAAPYVQAFDSLLAGPVAEYLK.I	3	3.04	0.24	-4.45
IPI00008282	Isoform 1 of Calcium/calmodulin-dependent 3',5'-cyclic nucleotide phosphodiesterase 1A	K.TMSLILHAADISHPAK.S	2	2.34	0.14	
IPI00008290	Isoform 1 of Ephrin type-A receptor 5 precursor	A.SPSNEVNLLDSR.T	1	2.61	0.26	-1.80
IPI00008290	Isoform 1 of Ephrin type-A receptor 5 precursor	A.SPSNEVNLLDSR.T	2	3.84	0.40	-3.11
IPI00008290	Isoform 1 of Ephrin type-A receptor 5 precursor	K.ETFNM*YYFESDDQNGR.N	2	4.73	0.56	-4.14
IPI00008290	Isoform 1 of Ephrin type-A receptor 5 precursor	K.IDTIAADESFTELDLGDR.V	2	6.01	0.59	-4.69
IPI00008290	Isoform 1 of Ephrin type-A receptor 5 precursor	K.NGWEEIGEVDENYAPIHTYQVCK.V	2	4.76	0.58	-0.77
IPI00008290	Isoform 1 of Ephrin type-A receptor 5 precursor	K.NGWEEIGEVDENYAPIHTYQVCK.V	3	4.41	0.37	-2.43
IPI00008290	Isoform 1 of Ephrin type-A receptor 5 precursor	K.VM*EQNQNNWLLTSWISNEGASR.I	2	5.20	0.50	-5.89
IPI00008290	Isoform 1 of Ephrin type-A receptor 5 precursor	R.DVGPLSK.K	1	1.71	0.11	-4.19
IPI00008290	Isoform 1 of Ephrin type-A receptor 5 precursor	R.NIKENQYIK.I	2	2.63	0.16	-3.63
IPI00008290	Isoform 1 of Ephrin type-A receptor 5 precursor	R.TVM*GDLGWIAFPK.N	1	1.40	0.09	-4.50
IPI00008290	Isoform 1 of Ephrin type-A receptor 5 precursor	R.TVM*GDLGWIAFPK.N	2	3.97	0.30	-4.34
IPI00008290	Isoform 1 of Ephrin type-A receptor 5 precursor	R.TVM*GDLGWIAFPK.N	3	3.91	0.16	-2.79

IPI00008303	Isoform 1 of N-acetylglucosamine-1-phosphodiester alpha-N-acetylglucosaminidase precursor	R.VSSSGGLQNAQFGIR.R	2	3.72	0.38	-1.42
IPI00008315	Isoform 1 of Ephrin type-B receptor 1 precursor	R.TVAGYGKFSGKMCFQTLTDDDYK.S	2	1.64	0.12	2.31
IPI00008318	Ephrin type-A receptor 4 precursor	K.ALSTDATCAK.C	1	2.09	0.34	-4.71
IPI00008318	Ephrin type-A receptor 4 precursor	K.ALSTDATCAK.C	2	2.67	0.31	-3.95
IPI00008318	Ephrin type-A receptor 4 precursor	K.CPPHSYSVWEGATSCTCDR.G	3	4.46	0.29	-2.98
IPI00008318	Ephrin type-A receptor 4 precursor	K.ETFNLYYYESDNDKER.F	2	4.56	0.50	-3.62
IPI00008318	Ephrin type-A receptor 4 precursor	K.ETFNLYYYESDNDKER.F	3	2.48	0.15	-2.79
IPI00008318	Ephrin type-A receptor 4 precursor	K.GLNPLTSYVFHVR.A	2	4.09	0.48	-4.50
IPI00008318	Ephrin type-A receptor 4 precursor	K.GLNPLTSYVFHVR.A	3	2.60	0.26	-2.11
IPI00008318	Ephrin type-A receptor 4 precursor	K.IDTIAADESFTQVDIGDR.I	2	6.17	0.50	-7.85
IPI00008318	Ephrin type-A receptor 4 precursor	K.IDTIAADESFTQVDIGDR.I	3	5.61	0.34	-3.67
IPI00008318	Ephrin type-A receptor 4 precursor	K.LNTEIRDVGPLSK.K	2	3.55	0.29	-3.31
IPI00008318	Ephrin type-A receptor 4 precursor	K.LNTEIRDVGPLSKK.G	2	3.84	0.32	-3.33
IPI00008318	Ephrin type-A receptor 4 precursor	K.LNTEIRDVGPLSKK.G	3	3.44	0.33	0.06
IPI00008318	Ephrin type-A receptor 4 precursor	K.M*YCGADGEWLVPIGNCLCNAGHEER.S	3	5.23	0.57	-3.74
IPI00008318	Ephrin type-A receptor 4 precursor	K.YNPNPDQSVSVTVTTNQAAPSSIALVQAK.E	2	4.69	0.60	-4.53
IPI00008318	Ephrin type-A receptor 4 precursor	K.YNPNPDQSVSVTVTTNQAAPSSIALVQAK.E	3	6.35	0.54	-5.08
IPI00008318	Ephrin type-A receptor 4 precursor	K.YYEKDQNER.S	1	1.61	0.18	-3.49
IPI00008318	Ephrin type-A receptor 4 precursor	K.YYEKDQNER.S	2	2.91	0.31	-3.04
IPI00008318	Ephrin type-A receptor 4 precursor	K.YYEKDQNER.S	3	1.78	0.19	-4.84
IPI00008318	Ephrin type-A receptor 4 precursor	L.NPLTSYVFHVR.A	2	3.28	0.46	-2.61
IPI00008318	Ephrin type-A receptor 4 precursor	R.DCNSLPGVM*GTCK.E	2	3.95	0.28	-3.54
IPI00008318	Ephrin type-A receptor 4 precursor	R.DVGPLSK.K	1	1.71	0.11	-4.19
IPI00008318	Ephrin type-A receptor 4 precursor	R.FIRENQFVK.I	1	2.30	0.21	-4.43
IPI00008318	Ephrin type-A receptor 4 precursor	R.FIRENQFVK.I	2	2.20	0.15	-3.41
IPI00008318	Ephrin type-A receptor 4 precursor	R.NLAQFPDTITGADTSSLVEVR.G	2	6.59	0.58	-3.78
IPI00008318	Ephrin type-A receptor 4 precursor	R.NLAQFPDTITGADTSSLVEVR.G	3	4.77	0.41	-3.39
IPI00008318	Ephrin type-A receptor 4 precursor	R.NTDIKGLNPLTSYVFHVR.A	2	4.67	0.53	-4.18
IPI00008318	Ephrin type-A receptor 4 precursor	R.NTDIKGLNPLTSYVFHVR.A	3	4.98	0.49	-3.12
IPI00008318	Ephrin type-A receptor 4 precursor	R.NTDIKGLNPLTSYVFHVR.A	4	4.04	0.38	-3.04
IPI00008318	Ephrin type-A receptor 4 precursor	R.QDISYNVVCK.K	2	2.32	0.08	-3.56
IPI00008318	Ephrin type-A receptor 4 precursor	R.QDISYNVVCKK.C	2	2.18	0.09	-3.43
IPI00008318	Ephrin type-A receptor 4 precursor	R.SVQGELGWIASPLEGGWEEVSIM*DEK.N	2	4.65	0.44	-4.11
IPI00008318	Ephrin type-A receptor 4 precursor	R.SVQGELGWIASPLEGGWEEVSIM*DEK.N	3	4.58	0.55	-3.52
IPI00008318	Ephrin type-A receptor 4 precursor	R.SVQGELGWIASPLEGGWEEVSIM*DEKNTPIR.T	3	4.04	0.30	-4.18
IPI00008318	Ephrin type-A receptor 4 precursor	R.TAAGYGDFSEPLEVTTNTVPSR.I	2	5.24	0.36	-2.46
IPI00008318	Ephrin type-A receptor 4 precursor	R.VYPANEVTLLDSR.S	1	1.91	0.37	-3.94
IPI00008318	Ephrin type-A receptor 4 precursor	R.VYPANEVTLLDSR.S	2	4.23	0.34	-4.00
IPI00008318	Ephrin type-A receptor 4 precursor	R.YSVALAWLEPDRPNGVILEYEVK.Y	3	2.50	0.10	0.42

IPI00008404	Isoform Long of Segment polarity protein dishevelled homolog DVL-1-like	R.IEPGDM*LLQVNDVNFENMSNDDAVRVLR.E	3	2.71	0.05	-1.14
IPI00008422	Isoform 2 of SWI/SNF-related matrix-associated actin-dependent regulator of chromatin subfamily A containing DEAD/H box 1	K.LIESTSTMDGAIAAALLMFGDAGGGPR.K	3	3.34	0.06	
IPI00008433	40S ribosomal protein S5	R.LTNSM*M*MHGRNNGK.K	2	2.19	0.11	2.37
IPI00008438	40S ribosomal protein S10	R.IAIYELLFK.E	2	3.09	0.25	-2.53
IPI00008494	Intercellular adhesion molecule 1 precursor	K.ASVSVTAEDEGTQR.L	2	2.64	0.17	-2.22
IPI00008494	Intercellular adhesion molecule 1 precursor	K.LLGIETPLPK.K	2	1.88	0.14	0.41
IPI00008494	Intercellular adhesion molecule 1 precursor	K.VTLNGVPAQPLGPR.A	2	2.69	0.25	-2.81
IPI00008494	Intercellular adhesion molecule 1 precursor	R.DLEGTYLCR.A	2	2.45	0.42	-2.13
IPI00008497	Ornithine decarboxylase	K.CNDSKAIVKTLAATGTGFDCASK.T	2	1.42	0.05	-7.34
IPI00008504	Carbonic anhydrase 14 precursor	K.LQGTLFSTEEEPSK.L	2	4.58	0.50	-4.08
IPI00008504	Carbonic anhydrase 14 precursor	R.SQISM*EQLEK.L	2	2.93	0.25	-1.94
IPI00008533	Isoform Long of Matrix metalloproteinase-17 precursor	R.AEDLSLGVEWLSR.F	2	2.75	0.28	
IPI00008533	Isoform Long of Matrix metalloproteinase-17 precursor	R.FGYLPPADPTTGQLQTQEELSK.A	2	3.52	0.44	-3.90
IPI00008533	Isoform Long of Matrix metalloproteinase-17 precursor	R.FGYLPPADPTTGQLQTQEELSK.A	3	3.86	0.20	-3.74
IPI00008533	Isoform Long of Matrix metalloproteinase-17 precursor	R.WSDGASYFFR.G	2	3.41	0.36	-3.17
IPI00008556	Isoform 1 of Coagulation factor XI precursor	K.DSVTETLPR.V	2	2.52	0.13	-1.85
IPI00008556	Isoform 1 of Coagulation factor XI precursor	K.TSESGLPSTR.I	2	2.54	0.24	-0.52
IPI00008556	Isoform 1 of Coagulation factor XI precursor	R.GGISGYTLR.L	2	2.21	0.14	-1.57
IPI00008580	Antileukoproteinase precursor	K.CLDPVDTPNPTR.R	2	3.86	0.23	
IPI00008586	Isoform 1 of Chondroitin sulfate proteoglycan 5 precursor	P.AREAGSAVEAEELVK.G	2	3.90	0.17	-3.84
IPI00008586	Isoform 1 of Chondroitin sulfate proteoglycan 5 precursor	R.EAGSAVEAEELVK.G	1	3.37	0.22	-3.88
IPI00008586	Isoform 1 of Chondroitin sulfate proteoglycan 5 precursor	R.EAGSAVEAEELVK.G	2	3.78	0.38	-5.56
IPI00008603	Actin, aortic smooth muscle	K.AGFAGDDAPR.A	1	2.25	0.23	-4.33
IPI00008603	Actin, aortic smooth muscle	K.AGFAGDDAPR.A	2	3.47	0.32	-3.15
IPI00008603	Actin, aortic smooth muscle	K.DSYVGDEAQSK.R	2	3.42	0.43	-3.44
IPI00008603	Actin, aortic smooth muscle	K.DSYVGDEAQSKR.G	2	3.20	0.47	-2.81
IPI00008603	Actin, aortic smooth muscle	K.EITALAPSTM*K.I	1	2.16	0.28	-3.20
IPI00008603	Actin, aortic smooth muscle	K.EITALAPSTM*K.I	2	3.01	0.23	-2.30
IPI00008603	Actin, aortic smooth muscle	K.EITALAPSTMK.I	2	2.64	0.15	-3.46
IPI00008603	Actin, aortic smooth muscle	K.IIAPPERK.Y	2	1.97	0.05	-3.56
IPI00008603	Actin, aortic smooth muscle	K.IKIIAPPER.K	2	2.50	0.12	2.48
IPI00008603	Actin, aortic smooth muscle	K.IKIIAPPERK.Y	2	2.55	0.12	-3.24

IPI00008603	Actin, aortic smooth muscle	K.YSVWIGGSILASLSTFQQMWISK.Q	2	1.77	0.24	-2.29
IPI00008603	Actin, aortic smooth muscle	R.AVFPSIVGRPR.H	2	3.03	0.29	-3.27
IPI00008603	Actin, aortic smooth muscle	R.DLTDYLM*K.I	1	2.48	0.24	-3.46
IPI00008603	Actin, aortic smooth muscle	R.DLTDYLM*K.I	2	2.37	0.25	-3.00
IPI00008603	Actin, aortic smooth muscle	R.GYSFVTTAER.E	2	3.32	0.42	-2.19
IPI00008603	Actin, aortic smooth muscle	R.HQGVM*VGM*GQK.D	2	2.78	0.19	
IPI00008603	Actin, aortic smooth muscle	R.LDLAGRDLTDYLM*K.I	2	2.69	0.21	-4.38
IPI00008603	Actin, aortic smooth muscle	R.LDLAGRDLTDYLM*K.I	3	3.36	0.32	-1.97
IPI00008603	Actin, aortic smooth muscle	R.M*QKEITALAPSTM*K.I	2	3.78	0.31	-4.87
IPI00008603	Actin, aortic smooth muscle	R.SYELPDGQVITIGNER.F	2	4.30	0.39	-4.42
IPI00008603	Actin, aortic smooth muscle	R.VAPEEHPTLLTEAPLNPK.A	3	2.86	0.27	-1.37
IPI00008726	Iron-responsive element-binding protein 2	K.GPYLLGVKAVLAESYEK.I	2	3.11	0.10	
IPI00008726	Iron-responsive element-binding protein 2	R.EFNSYGARR.G	1	2.23	0.09	
IPI00008756	Isoform 1 of Bullous pemphigoid antigen 1, isoforms 1/2/3/4/5/8 (Fragment)	K.DLHSPVAGYWLTASGER.I	3	2.60	0.08	-5.48
IPI00008756	Isoform 1 of Bullous pemphigoid antigen 1, isoforms 1/2/3/4/5/8 (Fragment)	K.FKQSAEEFR.K	2	2.27	0.14	
IPI00008780	Stanniocalcin-2 precursor	G.TDATNPPEGPQDR.S	2	2.90	0.20	-3.89
IPI00008780	Stanniocalcin-2 precursor	K.DLLLHEPYVDLVNLLLTCGEEVK.E	3	3.73	0.21	-3.76
IPI00008780	Stanniocalcin-2 precursor	K.SFIKDALK.C	1	1.96	0.10	-3.39
IPI00008780	Stanniocalcin-2 precursor	K.SFIKDALK.C	2	2.23	0.08	-1.86
IPI00008780	Stanniocalcin-2 precursor	R.EM*VSQLQR.E	2	2.19	0.12	0.80
IPI00008780	Stanniocalcin-2 precursor	R.VIVEM*IHFK.D	2	2.59	0.36	-2.92
IPI00008787	Alpha-N-acetylglucosaminidase precursor	K.NVFQLEQAFVLSK.Q	2	4.72	0.44	-5.03
IPI00008787	Alpha-N-acetylglucosaminidase precursor	K.QLAGLVANYYTPR.W	2	2.60	0.27	-1.28
IPI00008787	Alpha-N-acetylglucosaminidase precursor	R.AAAVSEAEADFYEQNSR.Y	2	5.23	0.54	-2.08
IPI00008787	Alpha-N-acetylglucosaminidase precursor	R.AAAVSEAEADFYEQNSR.Y	3	4.55	0.46	-1.83
IPI00008787	Alpha-N-acetylglucosaminidase precursor	R.AGGVLAYELLPALDEVLASDSR.F	2	5.99	0.52	-4.43
IPI00008787	Alpha-N-acetylglucosaminidase precursor	R.AGGVLAYELLPALDEVLASDSR.F	3	4.55	0.51	-4.79
IPI00008787	Alpha-N-acetylglucosaminidase precursor	R.FLLGSWLEQAR.A	2	3.53	0.33	-3.93
IPI00008787	Alpha-N-acetylglucosaminidase precursor	R.GDTVDLAKK.I	2	2.76	0.16	-2.45
IPI00008787	Alpha-N-acetylglucosaminidase precursor	R.GSTGVAAAAGLHR.Y	2	3.06	0.36	-3.28
IPI00008787	Alpha-N-acetylglucosaminidase precursor	R.KDPVPDLAAWVTSFAAR.R	3	2.78	0.26	-2.60
IPI00008787	Alpha-N-acetylglucosaminidase precursor	R.LFLEALVDSVAQGIPFQQHQFDK.N	3	4.52	0.36	-4.14
IPI00008787	Alpha-N-acetylglucosaminidase precursor	R.LLLTSAPSLATSPAFR.Y	2	4.27	0.53	-4.92
IPI00008787	Alpha-N-acetylglucosaminidase precursor	R.LLLTSAPSLATSPAFRYDLLDLTR.Q	3	2.63	0.23	-3.94
IPI00008787	Alpha-N-acetylglucosaminidase precursor	R.LLVLDLFAESQPVYTR.T	2	4.29	0.41	-4.26
IPI00008787	Alpha-N-acetylglucosaminidase precursor	R.LLVLDLFAESQPVYTR.T	3	2.81	0.21	-2.51
IPI00008787	Alpha-N-acetylglucosaminidase precursor	R.LPRPLPAVPGELTEATPNRYR.Y	3	3.29	0.36	-1.58
IPI00008787	Alpha-N-acetylglucosaminidase precursor	R.QAVQELVSLYYEEAR.S	2	5.20	0.50	-5.36
IPI00008787	Alpha-N-acetylglucosaminidase precursor	R.SFGM*TPVLPAFAGHVPEAVTR.V	2	3.69	0.44	-3.82
IPI00008787	Alpha-N-acetylglucosaminidase precursor	R.SFGM*TPVLPAFAGHVPEAVTR.V	3	3.02	0.37	-3.46

IPI00008787	Alpha-N-acetylglucosaminidase precursor	R.YDLLDLTR.Q	2	2.37	0.16	-1.83
IPI00008787	Alpha-N-acetylglucosaminidase precursor	R.YQLTLWGPEGNILDYANK.Q	2	4.32	0.46	-3.22
IPI00008894	Carboxypeptidase A4 precursor	K.FFGDQVLR.I	2	2.56	0.23	-0.47
IPI00008905	UDP-glucuronosyltransferase 2B15 precursor	K.NGGGFLFPPSYVPVVMSELSDQMIFMERIK.N	4	2.92	0.20	0.56
IPI00008944	Isoform 1 of Neuroendocrine protein 7B2 precursor	D.FSEDQGYPDPPNPCPVGK.T	2	4.38	0.49	-4.88
IPI00008944	Isoform 1 of Neuroendocrine protein 7B2 precursor	F.SEDQGYPDPPNPCPVGK.T	2	3.61	0.46	-4.20
IPI00008944	Isoform 1 of Neuroendocrine protein 7B2 precursor	K.DFSEDQGYPDPPNPCPVGK.T	2	4.45	0.47	
IPI00008944	Isoform 1 of Neuroendocrine protein 7B2 precursor	K.KSVPHFSDEDKDPE.-	2	4.03	0.41	-4.87
IPI00008944	Isoform 1 of Neuroendocrine protein 7B2 precursor	K.KSVPHFSDEDKDPE.-	3	3.68	0.39	-5.14
IPI00008944	Isoform 1 of Neuroendocrine protein 7B2 precursor	K.SVPHFSDEDKDPE.-	1	2.81	0.21	-2.39
IPI00008944	Isoform 1 of Neuroendocrine protein 7B2 precursor	K.SVPHFSDEDKDPE.-	2	3.48	0.41	-4.42
IPI00008944	Isoform 1 of Neuroendocrine protein 7B2 precursor	K.SVPHFSDEDKDPE.-	3	1.76	0.27	-2.11
IPI00008944	Isoform 1 of Neuroendocrine protein 7B2 precursor	K.TADDGCLENTPDTAEFSR.E	2	6.42	0.62	-3.97
IPI00008944	Isoform 1 of Neuroendocrine protein 7B2 precursor	N.LVGPQSIEGGAHEGLQHLGPFGNIPNIVAELTGDNIPK.D	3	5.93	0.50	-3.80
IPI00008944	Isoform 1 of Neuroendocrine protein 7B2 precursor	R.EFQLHQHLFDPEHDYPGLGK.W	2	4.98	0.52	-4.38
IPI00008944	Isoform 1 of Neuroendocrine protein 7B2 precursor	R.EFQLHQHLFDPEHDYPGLGK.W	3	5.07	0.50	-6.23
IPI00008944	Isoform 1 of Neuroendocrine protein 7B2 precursor	R.LDNVVAK.K	1	2.32	0.08	-3.41
IPI00008944	Isoform 1 of Neuroendocrine protein 7B2 precursor	R.LDNVVAKK.S	2	2.32	0.05	-3.22
IPI00008944	Isoform 1 of Neuroendocrine protein 7B2 precursor	R.SVNPYLQGQR.L	1	2.02	0.13	-2.08
IPI00008944	Isoform 1 of Neuroendocrine protein 7B2 precursor	R.SVNPYLQGQR.L	2	3.19	0.31	-2.40
IPI00008944	Isoform 1 of Neuroendocrine protein 7B2 precursor	R.SVNPYLQGQRLDNVVAK.K	2	3.52	0.25	-2.23
IPI00008944	Isoform 1 of Neuroendocrine protein 7B2 precursor	R.SVNPYLQGQRLDNVVAK.K	3	2.63	0.37	-1.06
IPI00008944	Isoform 1 of Neuroendocrine protein 7B2 precursor	R.SVNPYLQGQRLDNVVAKK.S	2	3.69	0.33	-3.68

IPI00008944	Isoform 1 of Neuroendocrine protein 7B2 precursor	R.SVNPYLQGQRLDNVVAKK.S	3	2.39	0.14	-1.79
IPI00008944	Isoform 1 of Neuroendocrine protein 7B2 precursor	R.TPDRVSEADIQR.L	2	2.22	0.05	-1.62
IPI00008944	Isoform 1 of Neuroendocrine protein 7B2 precursor	R.TPDRVSEADIQR.L	3	4.14	0.23	
IPI00008944	Isoform 1 of Neuroendocrine protein 7B2 precursor	R.VSEADIQR.L	1	1.68	0.10	-2.72
IPI00008944	Isoform 1 of Neuroendocrine protein 7B2 precursor	R.VSEADIQR.L	2	2.84	0.17	-3.09
IPI00008944	Isoform 1 of Neuroendocrine protein 7B2 precursor	S.IEGGAHEGLQHLGPFGNIPNIVAELTGDNIPK.D	3	6.82	0.56	-4.41
IPI00008994	Isoform 1 of Protein NDRG2	K.LDPTQTSFLK.M	2	2.53	0.40	-2.52
IPI00008994	Isoform 1 of Protein NDRG2	K.LTGLTSSIPEM*ILGHLFSQEELSGNSELIQK.Y	3	5.78	0.53	-4.50
IPI00008994	Isoform 1 of Protein NDRG2	K.LTGLTSSIPEM*ILGHLFSQEELSGNSELIQK.Y	4	3.82	0.22	-4.28
IPI00008994	Isoform 1 of Protein NDRG2	K.M*ADSGGQPQLTQPGK.L	2	3.94	0.46	-6.01
IPI00008994	Isoform 1 of Protein NDRG2	K.M*ADSGGQPQLTQPGKLTEAFK.Y	3	2.80	0.24	0.79
IPI00008994	Isoform 1 of Protein NDRG2	K.RPAILTYHDVGLNYK.S	2	3.83	0.41	-5.99
IPI00008994	Isoform 1 of Protein NDRG2	Q.THSVETPYGSVTFTVYGTPKPK.R	3	4.75	0.53	-3.32
IPI00008994	Isoform 1 of Protein NDRG2	R.CPVM*LVVGDQAPHEDAVVECNSK.L	3	4.51	0.46	-1.38
IPI00008994	Isoform 1 of Protein NDRG2	R.NIITHAPNLDNIELYWNSYNNRR.D	4	3.00	0.21	-4.33
IPI00008994	Isoform 1 of Protein NDRG2	R.SRTASLTSAASVDGNR.S	3	2.71	0.22	0.65
IPI00008994	Isoform 1 of Protein NDRG2	R.TLSQSSESGTLSSGPPGHT.M	2	4.28	0.58	-4.22
IPI00008994	Isoform 1 of Protein NDRG2	R.TLSQSSESGTLSSGPPGHTM*E.V	2	6.12	0.63	-4.35
IPI00008994	Isoform 1 of Protein NDRG2	R.YALNHPDTVEGLVLINIDPNAK.G	2	5.45	0.49	-3.19
IPI00008994	Isoform 1 of Protein NDRG2	R.YALNHPDTVEGLVLINIDPNAK.G	3	3.63	0.20	-2.85
IPI00008997	WAP four-disulfide core domain protein 1 precursor	K.LYKEYPEGDSK.N	2	2.92	0.25	-2.58
IPI00008997	WAP four-disulfide core domain protein 1 precursor	K.LYKEYPEGDSKNVAEPGRGQQK.H	3	4.51	0.40	-3.28
IPI00008997	WAP four-disulfide core domain protein 1 precursor	K.LYKEYPEGDSKNVAEPGRGQQK.H	4	3.95	0.35	-4.40
IPI00008997	WAP four-disulfide core domain protein 1 precursor	K.SRAEEAGAPGGPR.Q	2	3.54	0.34	-1.86
IPI00008998	Protein tyrosine phosphatase-like protein PTPLAD1	R.VELSDVQNPAISITENVLHFK.A	3	2.84	0.18	-3.29
IPI00009028	Tetranectin precursor	K.CFLAFTQTK.T	1	2.54	0.20	-2.76
IPI00009028	Tetranectin precursor	K.CFLAFTQTK.T	2	3.20	0.37	-2.45
IPI00009028	Tetranectin precursor	K.EQQALQTVCLK.G	1	3.01	0.18	-3.74
IPI00009028	Tetranectin precursor	K.EQQALQTVCLK.G	2	3.32	0.37	-3.62
IPI00009028	Tetranectin precursor	K.EQQALQTVCLKGTK.V	2	2.68	0.25	-2.63
IPI00009028	Tetranectin precursor	K.NWETEITAQPDGGK.T	2	4.46	0.38	-4.28

IPI00009028	Tetranectin precursor	K.NWETEITAQPDGGKTENCAVLSGAANGK.W	2	4.07	0.51	-1.51
IPI00009028	Tetranectin precursor	K.NWETEITAQPDGGKTENCAVLSGAANGK.W	3	6.12	0.48	-2.48
IPI00009028	Tetranectin precursor	K.SRLDTLAQEVALLK.E	2	4.32	0.30	-4.26
IPI00009028	Tetranectin precursor	K.SRLDTLAQEVALLK.E	3	5.44	0.28	-1.98
IPI00009028	Tetranectin precursor	K.SRLDTLAQEVALLKEQQALQTVCLK.G	3	7.37	0.58	-5.46
IPI00009028	Tetranectin precursor	K.SRLDTLAQEVALLKEQQALQTVCLK.G	4	3.04	0.12	-4.68
IPI00009028	Tetranectin precursor	K.TENCAVLSGAANGK.W	2	2.93	0.20	
IPI00009028	Tetranectin precursor	K.TFHEASEDCISR.G	2	4.05	0.45	-4.16
IPI00009028	Tetranectin precursor	K.TFHEASEDCISR.G	3	3.34	0.23	-1.76
IPI00009028	Tetranectin precursor	R.CRDQLPYICQFGIV.-	2	4.68	0.51	-3.54
IPI00009028	Tetranectin precursor	R.DQLPYICQFGIV.-	2	2.16	0.09	-2.08
IPI00009028	Tetranectin precursor	R.GGTLSTPQTGSENDALYEYLR.Q	2	4.96	0.59	-4.27
IPI00009028	Tetranectin precursor	R.GGTLSTPQTGSENDALYEYLR.Q	3	4.25	0.41	-3.25
IPI00009028	Tetranectin precursor	R.IAYKNWETEITAQPDGGKTENCAVLSGAANGK.W	3	6.48	0.53	-3.81
IPI00009028	Tetranectin precursor	R.IAYKNWETEITAQPDGGKTENCAVLSGAANGK.W	4	5.14	0.41	-3.92
IPI00009028	Tetranectin precursor	R.LDTLAQEVALLK.E	1	2.66	0.28	-3.34
IPI00009028	Tetranectin precursor	R.LDTLAQEVALLK.E	2	4.53	0.38	-3.92
IPI00009028	Tetranectin precursor	R.LDTLAQEVALLK.E	3	4.91	0.22	-3.16
IPI00009028	Tetranectin precursor	R.LDTLAQEVALLKEQQALQTVCLK.G	2	5.17	0.55	-3.09
IPI00009028	Tetranectin precursor	R.LDTLAQEVALLKEQQALQTVCLK.G	3	7.33	0.61	-4.02
IPI00009028	Tetranectin precursor	R.LDTLAQEVALLKEQQALQTVCLK.G	4	4.46	0.40	-3.04
IPI00009028	Tetranectin precursor	R.QSVGNEAEIWLGLNDM*AAEGTWVDM*TGAR.I	3	6.01	0.55	-5.00
IPI00009028	Tetranectin precursor	W.ETEITAQPDGGK.T	2	3.01	0.21	-3.99
IPI00009030	Isoform LAMP-2A of Lysosome-associated membrane glycoprotein 2 precursor	K.EQTVSVSGAFQINTFDLR.V	2	2.42	0.11	-3.14
IPI00009030	Isoform LAMP-2A of Lysosome-associated membrane glycoprotein 2 precursor	K.GILTVDELLAIR.I	2	3.28	0.24	-4.69
IPI00009030	Isoform LAMP-2A of Lysosome-associated membrane glycoprotein 2 precursor	K.GILTVDELLAIR.I	3	4.00	0.18	-3.13
IPI00009030	Isoform LAMP-2A of Lysosome-associated membrane glycoprotein 2 precursor	K.YLDFVFAVK.N	1	1.97	0.27	-4.19
IPI00009030	Isoform LAMP-2A of Lysosome-associated membrane glycoprotein 2 precursor	K.YLDFVFAVK.N	2	3.47	0.36	-3.76
IPI00009030	Isoform LAMP-2A of Lysosome-associated membrane glycoprotein 2 precursor	R.IPLNDLFR.C	2	3.02	0.18	-2.46
IPI00009030	Isoform LAMP-2A of Lysosome-associated membrane glycoprotein 2 precursor	R.SHTALLR.L	1	1.99	0.18	-4.42
IPI00009030	Isoform LAMP-2A of Lysosome-associated membrane glycoprotein 2 precursor	R.YETTNKTYK.T	2	2.92	0.23	-3.95
IPI00009054	Isoform BMP1-3 of Bone morphogenetic protein 1 precursor	K.AAAFLGDIALDEEDLR.A	2	5.72	0.46	-4.81

IPI00009054	Isoform BMP1-3 of Bone morphogenetic protein 1 precursor	R.AFQVQQAVDLR.R	2	3.42	0.32	-4.34
IPI00009070	Isoform 1 of HBS1-like protein	K.STLM*GHMLYLLGNINKRTMHKYEQESK.K	3	3.62	0.06	
IPI00009111	Trophoblast glycoprotein precursor	R.AGAFEHLPSLR.Q	2	2.72	0.20	-2.69
IPI00009111	Trophoblast glycoprotein precursor	R.DVLAQLPSLR.H	2	3.14	0.23	-3.21
IPI00009111	Trophoblast glycoprotein precursor	R.NLFLTGNQLAVLPAGAFAR.R	2	5.60	0.53	-5.03
IPI00009111	Trophoblast glycoprotein precursor	R.NLFLTGNQLAVLPAGAFAR.R	3	4.53	0.22	-3.75
IPI00009111	Trophoblast glycoprotein precursor	R.SFEGM*VVAALLAGR.A	2	4.87	0.46	-5.27
IPI00009111	Trophoblast glycoprotein precursor	R.SFEGM*VVAALLAGR.A	3	3.32	0.11	-2.65
IPI00009123	Nucleobindin-2 precursor	K.ADIEEIKSGR.L	2	2.08	0.14	-1.71
IPI00009123	Nucleobindin-2 precursor	K.AKLDSLQDIGM*DHQALLK.Q	3	3.85	0.28	-2.60
IPI00009123	Nucleobindin-2 precursor	K.ELDLVSHHVR.T	2	3.40	0.35	-2.54
IPI00009123	Nucleobindin-2 precursor	K.IEPPDTGLYYDEYLKQVIDVLETDKHFR.E	5	2.37	0.22	-3.84
IPI00009123	Nucleobindin-2 precursor	K.LQKADIEEIKSGR.L	3	2.42	0.08	-1.14
IPI00009123	Nucleobindin-2 precursor	K.QVIDVLETDKHFR.E	2	2.78	0.17	-3.51
IPI00009123	Nucleobindin-2 precursor	K.VQNIHPVESAK.I	2	3.49	0.39	-2.75
IPI00009123	Nucleobindin-2 precursor	K.VQNIHPVESAK.I	3	1.97	0.10	-2.96
IPI00009123	Nucleobindin-2 precursor	R.LVTLEEFLK.A	2	2.74	0.22	-2.62
IPI00009123	Nucleobindin-2 precursor	R.TKLDELKR.Q	2	1.96	0.06	-3.04
IPI00009145	Mannosyl-oligosaccharide 1,2-alpha-mannosidase IB	K.FDGAVEAVAVR.Q	2	2.87	0.28	-3.25
IPI00009145	Mannosyl-oligosaccharide 1,2-alpha-mannosidase IB	K.IKENKPLPPVPIPNLVGIR.G	2	4.79	0.54	-2.82
IPI00009145	Mannosyl-oligosaccharide 1,2-alpha-mannosidase IB	K.IKENKPLPPVPIPNLVGIR.G	3	3.88	0.53	-3.32
IPI00009145	Mannosyl-oligosaccharide 1,2-alpha-mannosidase IB	K.IKENKPLPPVPIPNLVGIR.G	4	2.09	0.18	-1.25
IPI00009145	Mannosyl-oligosaccharide 1,2-alpha-mannosidase IB	K.NPGVFLIHGPDEHR.H	2	3.18	0.33	-3.18
IPI00009145	Mannosyl-oligosaccharide 1,2-alpha-mannosidase IB	K.NPGVFLIHGPDEHR.H	3	2.41	0.35	-1.24
IPI00009145	Mannosyl-oligosaccharide 1,2-alpha-mannosidase IB	R.FDLGLEDVLIPHVDAGK.G	3	4.26	0.45	-2.97
IPI00009145	Mannosyl-oligosaccharide 1,2-alpha-mannosidase IB	R.VNGGFSGVK.D	2	2.12	0.18	-2.98
IPI00009148	Diphosphoinositol polyphosphate phosphohydrolase 1	K.VLQYHKPVQASYFETLR.Q	3	3.60	0.38	-2.32
IPI00009203	Sorting nexin-7	K.FIVKGMVERFNDDFIETRRK.A	3	2.68	0.10	-7.51
IPI00009276	Endothelial protein C receptor precursor	C.SQDASDGLQR.L	2	3.07	0.25	-1.04
IPI00009276	Endothelial protein C receptor precursor	R.EFLEDTCVQYVQK.H	2	5.01	0.44	-1.89
IPI00009276	Endothelial protein C receptor precursor	R.LHM*LQISYFR.D	2	3.02	0.18	-2.15
IPI00009276	Endothelial protein C receptor precursor	R.LHM*LQISYFR.D	3	3.59	0.23	-2.37

IPI00009276	Endothelial protein C receptor precursor	R.TLAFPLTIR.C	2	2.60	0.28	-1.27
IPI00009276	Endothelial protein C receptor precursor	R.TQSGLQSYLLQFHGLVR.L	2	4.71	0.46	-4.03
IPI00009294	Cysteine-rich motor neuron 1 protein precursor	R.LHPSEDSSLDSIASVVVPIII.C	2	3.26	0.34	-4.14
IPI00009335	Brain protein 16	R.ALVNLAADPGLHETLLAADPGLPAR.L	3	3.33	0.12	
IPI00009362	Secretogranin-2 precursor	E.IVEEQYTPQSLATLESVFQELGK.L	2	5.17	0.55	-4.53
IPI00009362	Secretogranin-2 precursor	E.IVEEQYTPQSLATLESVFQELGK.L	3	3.89	0.42	-4.58
IPI00009362	Secretogranin-2 precursor	K.ANNIAYEDVVGGEDWNPVEEK.I	2	6.42	0.56	-4.50
IPI00009362	Secretogranin-2 precursor	K.DQLSDDVSK.V	2	2.55	0.21	-1.08
IPI00009362	Secretogranin-2 precursor	K.EHLNQGSSQETDK.L	2	3.19	0.21	-3.62
IPI00009362	Secretogranin-2 precursor	K.EHLNQGSSQETDK.L	3	2.87	0.13	-2.85
IPI00009362	Secretogranin-2 precursor	K.EHLNQGSSQETDKLAPVS.K	2	4.56	0.54	-3.50
IPI00009362	Secretogranin-2 precursor	K.ENGDESHLPER.D	2	2.44	0.29	-2.12
IPI00009362	Secretogranin-2 precursor	K.ENGDESHLPERDSLSEEDWM*R.I	3	3.09	0.23	
IPI00009362	Secretogranin-2 precursor	K.ENIEKNEQINDEM*.K	2	4.49	0.46	-3.91
IPI00009362	Secretogranin-2 precursor	K.ENKPYALNSEK.N	1	2.17	0.20	-3.79
IPI00009362	Secretogranin-2 precursor	K.ESKDQLSDDVSK.V	2	1.74	0.16	-2.24
IPI00009362	Secretogranin-2 precursor	K.IESQTQEEVR.D	2	3.24	0.34	-2.81
IPI00009362	Secretogranin-2 precursor	K.IESQTQEEVRDSK.E	2	3.65	0.35	-4.51
IPI00009362	Secretogranin-2 precursor	K.IESQTQEEVRDSK.E	3	2.01	0.19	-1.05
IPI00009362	Secretogranin-2 precursor	K.IESQTQEEVRDSKENIEKNEQINDE.M	3	5.18	0.30	-1.94
IPI00009362	Secretogranin-2 precursor	K.IESQTQEEVRDSKENIEKNEQINDEM*.K	3	3.86	0.29	-2.80
IPI00009362	Secretogranin-2 precursor	K.NDDTPNRQYWDEDLLM*K.V	3	3.50	0.41	-1.63
IPI00009362	Secretogranin-2 precursor	K.RLVNAAGSGR.L	2	3.53	0.30	-2.82
IPI00009362	Secretogranin-2 precursor	K.RTNEIVEEQYTPQSLATLESVFQELGK.L	3	4.14	0.34	-3.63
IPI00009362	Secretogranin-2 precursor	K.SGYPKTPGR.A	2	1.40	0.12	-2.70
IPI00009362	Secretogranin-2 precursor	K.TSYFPNPYNQEK.V	2	2.97	0.34	-2.24
IPI00009362	Secretogranin-2 precursor	K.VLEYLNQEK.A	1	2.45	0.12	-3.23
IPI00009362	Secretogranin-2 precursor	K.VLEYLNQEK.A	2	3.18	0.23	-2.40
IPI00009362	Secretogranin-2 precursor	K.VLEYLNQEKAEK.G	2	3.89	0.33	-3.28
IPI00009362	Secretogranin-2 precursor	K.VLEYLNQEKAEK.G	3	1.83	0.14	-1.35
IPI00009362	Secretogranin-2 precursor	L.RQAENEPQSAPK.E	2	2.95	0.20	-0.74
IPI00009362	Secretogranin-2 precursor	N.PVEEKIESQTQEEVR.D	3	3.66	0.32	-0.92
IPI00009362	Secretogranin-2 precursor	P.GQGSSEDDLQEEEQIEQAIK.E	2	6.20	0.35	-4.02
IPI00009362	Secretogranin-2 precursor	P.GQGSSEDDLQEEEQIEQAIKEHLNQGSSQETDK.L	3	6.29	0.58	-3.25
IPI00009362	Secretogranin-2 precursor	P.VGPPKNDDTPNR.Q	2	2.93	0.38	-2.57
IPI00009362	Secretogranin-2 precursor	R.AGTEALPDGLSVEDILNLLGM*ESAANQK.T	3	4.27	0.40	
IPI00009362	Secretogranin-2 precursor	R.ALEYIENLR.Q	2	3.43	0.12	-2.03
IPI00009362	Secretogranin-2 precursor	R.DSKENIEKNEQINDEM*.K	2	5.04	0.50	-2.81
IPI00009362	Secretogranin-2 precursor	R.DSLSEEDWM*R.I	2	2.79	0.26	-3.68
IPI00009362	Secretogranin-2 precursor	R.ELDLPVDLDDISEADLDHPDLFQNR.M	3	2.52	0.25	-3.75
IPI00009362	Secretogranin-2 precursor	R.ERM*DEEQKLYTDDEDDIYK.A	3	4.32	0.46	-3.21
IPI00009362	Secretogranin-2 precursor	R.FPVGPPKNDDTPNR.Q	3	2.84	0.26	

IPI00009362	Secretogranin-2 precursor	R.KESKDQLSDDVSK.V	2	4.51	0.38	-2.68
IPI00009362	Secretogranin-2 precursor	R.KESKDQLSDDVSK.V	3	2.16	0.16	0.21
IPI00009362	Secretogranin-2 precursor	R.LENVQKFPSPEM*IR.A	2	3.30	0.30	-1.94
IPI00009362	Secretogranin-2 precursor	R.LENVQKFPSPEM*IR.A	3	3.54	0.21	-1.97
IPI00009362	Secretogranin-2 precursor	R.LFEKPLDSQSIYQLIEISR.N	3	3.92	0.28	-3.84
IPI00009362	Secretogranin-2 precursor	R.M*LVKYPEIINSNQV.K	2	3.82	0.21	-2.00
IPI00009362	Secretogranin-2 precursor	R.NQLLQKEPDLR.L	2	3.11	0.19	-6.21
IPI00009362	Secretogranin-2 precursor	R.NQLLQKEPDLRLENVQKFPSPEM*IR.A	3	5.52	0.42	
IPI00009362	Secretogranin-2 precursor	R.NQLLQKEPDLRLENVQKFPSPEM*IR.A	4	2.98	0.21	-4.50
IPI00009362	Secretogranin-2 precursor	R.QAENEPQSAPK.E	2	2.13	0.27	-1.72
IPI00009362	Secretogranin-2 precursor	R.QYWDEDLLM*K.V	2	2.50	0.29	-3.18
IPI00009362	Secretogranin-2 precursor	R.TNEIVEEQYTPQSLATLESVFQELGK.L	2	5.77	0.60	-6.41
IPI00009362	Secretogranin-2 precursor	R.TNEIVEEQYTPQSLATLESVFQELGK.L	3	7.35	0.57	-5.97
IPI00009362	Secretogranin-2 precursor	R.TNEIVEEQYTPQSLATLESVFQELGK.L	4	5.13	0.40	-3.41
IPI00009362	Secretogranin-2 precursor	R.TNEIVEEQYTPQSLATLESVFQELGKLTGPNNQ.K	3	4.78	0.50	-4.51
IPI00009362	Secretogranin-2 precursor	R.VPGQGSSEDDLQEEEQIEQAIK.E	2	5.87	0.43	-4.52
IPI00009362	Secretogranin-2 precursor	R.VPGQGSSEDDLQEEEQIEQAIK.E	3	3.42	0.28	-4.79
IPI00009362	Secretogranin-2 precursor	R.VPGQGSSEDDLQEEEQIEQAIKEHLNQGSSQETD.K	3	6.76	0.55	-4.14
IPI00009362	Secretogranin-2 precursor	R.VPGQGSSEDDLQEEEQIEQAIKEHLNQGSSQETDK.L	3	4.35	0.44	-3.36
IPI00009362	Secretogranin-2 precursor	R.VPGQGSSEDDLQEEEQIEQAIKEHLNQGSSQETDK.L	4	6.66	0.52	-3.37
IPI00009362	Secretogranin-2 precursor	R.VPGQGSSEDDLQEEEQIEQAIKEHLNQGSSQETDKLAPVS.K	3	5.29	0.41	-3.56
IPI00009362	Secretogranin-2 precursor	R.VPGQGSSEDDLQEEEQIEQAIKEHLNQGSSQETDKLAPVS.K	4	5.78	0.48	-3.59
IPI00009362	Secretogranin-2 precursor	V.PGQGSSEDDLQEEEQIEQAIK.E	2	5.43	0.51	-5.91
IPI00009362	Secretogranin-2 precursor	V.PGQGSSEDDLQEEEQIEQAIKEHLNQGSSQETDK.L	4	5.37	0.43	-0.54
IPI00009362	Secretogranin-2 precursor	W.NPVEEKIESQTQEEVR.D	3	4.24	0.48	-2.63
IPI00009365	COX16-like protein C14orf112, mitochondrial precursor	K.ISLESEYEK.I	2	2.19	0.18	-1.60
IPI00009377	HSPC212	K.EMEASGAHRDSQKAGER.D	2	2.35	0.16	
IPI00009396	Isoform 1 of Cannabinoid receptor 1	K.FPLTSFR.G	2	1.65	0.12	-1.69
IPI00009396	Isoform 1 of Cannabinoid receptor 1	K.LGYFPQKFPLTSFR.G	3	3.72	0.34	-2.36
IPI00009439	Synaptotagmin-1	K.LQYSLDYDFQNNQLLVGIIQAAELPALDMGGTSDPYVK.V	3	3.92	0.50	-1.46
IPI00009439	Synaptotagmin-1	K.LQYSLDYDFQNNQLLVGIIQAAELPALDMGGTSDPYVK.V	4	2.74	0.15	-1.05
IPI00009439	Synaptotagmin-1	K.TLNPVFNEQFTFK.V	2	2.11	0.26	-1.83
IPI00009471	WD repeat-containing protein 3	R.DVIGFNMAGLDYLKR.E	3	2.70	0.14	-6.62
IPI00009477	Intercellular adhesion molecule 2 precursor	R.QVILTLQPTLVAVGK.S	2	4.75	0.38	-3.79
IPI00009477	Intercellular adhesion molecule 2 precursor	R.VPTVEPLDSLTLFLFR.G	2	4.82	0.53	-5.58
IPI00009477	Intercellular adhesion molecule 2 precursor	R.VPTVEPLDSLTLFLFR.G	3	3.79	0.17	-3.58
IPI00009532	4-aminobutyrate aminotransferase, mitochondrial precursor	K.NLLLAEVINIIK.R	2	4.01	0.29	-4.61
IPI00009771	Lamin-B2	K.EQEMTEM*R.D	2	2.36	0.11	
IPI00009793	Complement C1r-like protein	G.SVLLAQELPQQLTSPGYPEPYGK.G	2	3.91	0.31	-2.64
IPI00009793	Complement C1r-like protein	G.SVLLAQELPQQLTSPGYPEPYGK.G	3	4.60	0.32	-4.29

IPI00009793	Complement C1r-like protein	G.SVLLAQELPQQLTSPGYPEPYGKGQESSTDIK.A	3	3.57	0.35	-3.15
IPI00009793	Complement C1r-like protein	K.APEGFAVR.L	2	2.27	0.18	-2.53
IPI00009793	Complement C1r-like protein	K.GQESSTDIKAPEGFAVR.L	2	3.77	0.36	-2.99
IPI00009793	Complement C1r-like protein	K.GQESSTDIKAPEGFAVR.L	3	2.87	0.29	-0.89
IPI00009793	Complement C1r-like protein	K.LGNFPWQAFTSIHGR.G	3	3.12	0.28	-3.19
IPI00009793	Complement C1r-like protein	K.VLSYVDWIK.G	2	2.86	0.30	-2.35
IPI00009793	Complement C1r-like protein	K.YSRLPVAPR.E	2	2.72	0.21	-2.77
IPI00009793	Complement C1r-like protein	R.GGGALLGDR.W	1	2.35	0.20	-2.80
IPI00009793	Complement C1r-like protein	R.GGGALLGDR.W	2	3.11	0.23	-4.75
IPI00009793	Complement C1r-like protein	R.GSEAINAPGDNPAK.V	2	4.23	0.45	-3.15
IPI00009793	Complement C1r-like protein	R.VVVHPDYR.Q	1	2.24	0.11	-4.13
IPI00009793	Complement C1r-like protein	R.VVVHPDYR.Q	2	2.18	0.22	-1.02
IPI00009802	Isoform V0 of Versican core protein precursor	K.IEVDKNGKDLK.E	2	3.05	0.15	-0.99
IPI00009802	Isoform V0 of Versican core protein precursor	K.IGQDYKGR.V	2	2.06	0.14	-2.37
IPI00009802	Isoform V0 of Versican core protein precursor	K.LLASDAGLYR.C	2	3.43	0.38	-1.16
IPI00009802	Isoform V0 of Versican core protein precursor	R.AATSRYTLNFEAAQK.A	2	3.59	0.19	-2.51
IPI00009802	Isoform V0 of Versican core protein precursor	R.AATSRYTLNFEAAQK.A	3	3.26	0.30	-3.65
IPI00009802	Isoform V0 of Versican core protein precursor	R.AQCGGGLLGVR.T	2	2.59	0.05	-3.12
IPI00009802	Isoform V0 of Versican core protein precursor	R.EIVISER.L	2	1.78	0.07	-4.13
IPI00009802	Isoform V0 of Versican core protein precursor	R.ITEEFLGK.Y	1	2.11	0.06	-3.09
IPI00009802	Isoform V0 of Versican core protein precursor	R.LATVGELQAAWR.N	2	3.69	0.33	-2.47
IPI00009802	Isoform V0 of Versican core protein precursor	R.LGEPNYGAEIR.G	2	3.49	0.27	-2.55
IPI00009802	Isoform V0 of Versican core protein precursor	R.NGFDQCDYGWLSDASVR.H	2	4.84	0.45	-2.06
IPI00009802	Isoform V0 of Versican core protein precursor	R.QEVNPVRQEIESETTSEEQIQEEK.S	3	4.45	0.32	-2.69
IPI00009802	Isoform V0 of Versican core protein precursor	R.SPQETYDVYCYVDHLDGDVFHLTVPSK.F	3	4.09	0.40	0.97
IPI00009802	Isoform V0 of Versican core protein precursor	R.SPQETYDVYCYVDHLDGDVFHLTVPSK.F	4	4.52	0.44	-6.16
IPI00009802	Isoform V0 of Versican core protein precursor	R.VSVPTHPEAVGDASLTVVK.L	2	4.29	0.51	-3.60
IPI00009802	Isoform V0 of Versican core protein precursor	R.VSVPTHPEAVGDASLTVVK.L	3	4.08	0.25	-3.51
IPI00009802	Isoform V0 of Versican core protein precursor	R.YTLNFEAAQK.A	2	3.14	0.29	-3.14
IPI00009826	Carboxypeptidase B precursor	K.YVASYVLEHLY.-	2	3.32	0.36	-2.01
IPI00009826	Carboxypeptidase B precursor	R.SVIGTTFEGR.A	2	3.26	0.35	-1.22
IPI00009826	Carboxypeptidase B precursor	R.YGFLLPESQIR.A	2	2.93	0.19	-2.21
IPI00009865	Keratin, type I cytoskeletal 10	K.ADLEM*QIESLTEELAYLK.K	2	3.96	0.33	
IPI00009865	Keratin, type I cytoskeletal 10	K.ADLEM*QIESLTEELAYLKK.N	3	2.41	0.14	-2.76
IPI00009865	Keratin, type I cytoskeletal 10	K.DAEAWFNEK.S	2	2.70	0.20	
IPI00009865	Keratin, type I cytoskeletal 10	K.GSLGGGFSSGGFSGGSFSR.G	2	5.51	0.61	-3.24
IPI00009865	Keratin, type I cytoskeletal 10	K.QSLEASLAETEGR.Y	2	3.28	0.28	
IPI00009865	Keratin, type I cytoskeletal 10	K.SSSSGSVGESSSKG.P	2	4.27	0.46	-2.60
IPI00009865	Keratin, type I cytoskeletal 10	K.SSSSGSVGESSSKGP.R	2	4.07	0.39	-1.70
IPI00009865	Keratin, type I cytoskeletal 10	K.TIDDLKNQILNLTTDNANILLQIDNAR.L	3	5.71	0.27	
IPI00009865	Keratin, type I cytoskeletal 10	R.GSSGGGCFGGSSGGYGGLGGFGGGSFR.G	2	5.84	0.38	
IPI00009865	Keratin, type I cytoskeletal 10	R.LAADDFR.L	2	2.47	0.23	-3.76

IPI00009865	Keratin, type I cytoskeletal 10	R.LASYLDKVR.A	2	2.65	0.13	-3.49
IPI00009865	Keratin, type I cytoskeletal 10	R.NVSTGDVNVEM*NAAPGVDLTQLLNNM*R.S	2	4.80	0.43	
IPI00009865	Keratin, type I cytoskeletal 10	R.NVSTGDVNVEM*NAAPGVDLTQLLNNM*R.S	3	3.68	0.36	-3.24
IPI00009865	Keratin, type I cytoskeletal 10	R.SGGGGGGGGCGGGGGVSSLR.I	2	3.03	0.36	
IPI00009865	Keratin, type I cytoskeletal 10	R.SQYEQLAEQNRK.D	2	3.41	0.22	-3.30
IPI00009867	Keratin, type II cytoskeletal 5	K.KYEDEINKR.T	2	3.00	0.17	-1.46
IPI00009867	Keratin, type II cytoskeletal 5	R.NLDLDSIIAEVKAQYEEIANR.S	3	3.40	0.16	
IPI00009881	Neuroendocrine secretory protein 55	K.HSTFGQSLTQR.L	2	2.76	0.40	-3.55
IPI00009890	Glia-derived nexin precursor	K.TIDSWMSIM*VPK.R	2	2.85	0.18	
IPI00009890	Glia-derived nexin precursor	R.DM*IDNLLSPDLIDGVLTR.L	2	3.21	0.15	
IPI00009899	Uncharacterized protein C5orf5	R.LVKQMLTRASITPVLGSPSTKR.R	2	2.43	0.20	
IPI00009901	Nuclear transport factor 2	K.ADEDPIM*GFHQM*FLLK.N	2	3.38	0.14	-2.77
IPI00009901	Nuclear transport factor 2	K.IQHSITAQDHQPTPDSCIISM*VVGQLK.A	3	4.13	0.42	-2.87
IPI00009904	Protein disulfide-isomerase A4 precursor	K.IANILKDKDPPIPVAK.I	3	2.52	0.13	-2.69
IPI00009904	Protein disulfide-isomerase A4 precursor	K.IANILKDKDPPIPVAK.I	4	2.89	0.25	-2.37
IPI00009904	Protein disulfide-isomerase A4 precursor	K.KGQAVDYEGSR.T	2	3.66	0.36	-1.27
IPI00009904	Protein disulfide-isomerase A4 precursor	K.M*DATANDVPSDR.Y	2	3.24	0.30	-1.95
IPI00009904	Protein disulfide-isomerase A4 precursor	K.VDATAETDLAK.R	2	3.91	0.26	-2.38
IPI00009904	Protein disulfide-isomerase A4 precursor	K.VSQGQLVVM*QPEK.F	2	3.90	0.32	-3.17
IPI00009904	Protein disulfide-isomerase A4 precursor	K.YALPLVGHR.K	2	2.13	0.16	-1.40
IPI00009904	Protein disulfide-isomerase A4 precursor	K.YGIVDYM*IEQSGPPSKEILTLK.Q	3	3.07	0.26	-1.69
IPI00009904	Protein disulfide-isomerase A4 precursor	R.SHM*M*DVQGSTQDSAIKDFVLK.Y	3	3.14	0.32	-3.04
IPI00009920	Complement component 6 precursor	K.AKDLHLSDVFLK.A	2	4.54	0.52	-2.39
IPI00009920	Complement component 6 precursor	K.AKDLHLSDVFLK.A	3	3.82	0.44	-2.22
IPI00009920	Complement component 6 precursor	K.ALNHLPLEYNSALYSR.I	2	4.00	0.43	-2.71
IPI00009920	Complement component 6 precursor	K.ALNHLPLEYNSALYSR.I	3	4.49	0.27	-4.10
IPI00009920	Complement component 6 precursor	K.ALQEYAAK.F	2	2.14	0.09	-1.55
IPI00009920	Complement component 6 precursor	K.CVCLLPPQCFK.G	2	3.44	0.11	-2.47
IPI00009920	Complement component 6 precursor	K.DLHLSDVFLK.A	2	3.13	0.23	-1.93
IPI00009920	Complement component 6 precursor	K.ENPAVIDFELAPIVDLVR.N	2	6.07	0.53	-5.62
IPI00009920	Complement component 6 precursor	K.ENPAVIDFELAPIVDLVR.N	3	3.69	0.28	-4.53
IPI00009920	Complement component 6 precursor	K.HEGSFIQGAEK.S	2	2.39	0.18	-2.64
IPI00009920	Complement component 6 precursor	K.LSEKHEGSFIQGAEK.S	2	3.88	0.23	-3.16
IPI00009920	Complement component 6 precursor	K.LSEKHEGSFIQGAEK.S	3	4.72	0.45	-2.88
IPI00009920	Complement component 6 precursor	K.RSENINHNSAFK.Q	2	3.57	0.36	-4.85
IPI00009920	Complement component 6 precursor	K.RSENINHNSAFK.Q	3	3.23	0.23	-3.74
IPI00009920	Complement component 6 precursor	K.TFSEWLESVK.E	2	2.86	0.33	-3.24
IPI00009920	Complement component 6 precursor	K.TFSEWLESVKENPAVIDFELAPIVDLVR.N	3	3.72	0.33	-4.33
IPI00009920	Complement component 6 precursor	K.TLNICEVGTIR.C	2	3.64	0.43	-1.33
IPI00009920	Complement component 6 precursor	R.CLPDGTWR.Q	2	1.94	0.10	0.18
IPI00009920	Complement component 6 precursor	R.GEVLDNSFTGGICK.T	2	4.44	0.49	-3.62
IPI00009920	Complement component 6 precursor	R.IFDDFGTHYFTSGSLGGVYDLLYQFSSEELK.N	3	5.65	0.51	-4.39

IPI00009920	Complement component 6 precursor	R.IFDDFGTHYFTSGSLGGVYDLLYQFSSEELKNSGLTEEEAK.H	3	2.81	0.20	-3.49
IPI00009920	Complement component 6 precursor	R.IFDDFGTHYFTSGSLGGVYDLLYQFSSEELKNSGLTEEEAK.H	4	6.39	0.54	-5.35
IPI00009920	Complement component 6 precursor	R.IGESIELTCPK.G	2	3.23	0.32	-3.08
IPI00009920	Complement component 6 precursor	R.KALQEYAAK.F	1	2.42	0.14	-4.59
IPI00009920	Complement component 6 precursor	R.KALQEYAAK.F	2	2.43	0.23	-1.78
IPI00009920	Complement component 6 precursor	R.KYNPIPSVQLM*GNGFHFLAGEPR.G	3	3.93	0.38	-4.22
IPI00009920	Complement component 6 precursor	R.KYNPIPSVQLM*GNGFHFLAGEPR.G	4	3.96	0.24	-3.63
IPI00009920	Complement component 6 precursor	R.QLEWGLER.T	2	1.85	0.13	-0.82
IPI00009920	Complement component 6 precursor	R.SENINHNSAFK.Q	1	2.83	0.19	-0.95
IPI00009920	Complement component 6 precursor	R.SENINHNSAFK.Q	3	1.69	0.22	-0.79
IPI00009920	Complement component 6 precursor	R.SEYGAALAWEK.G	1	2.30	0.12	-2.68
IPI00009920	Complement component 6 precursor	R.SEYGAALAWEK.G	2	3.41	0.32	-2.44
IPI00009920	Complement component 6 precursor	R.TSNPYRVPANLENVGFEVQTAEDDLK.T	3	5.28	0.42	-2.13
IPI00009920	Complement component 6 precursor	R.TSNPYRVPANLENVGFEVQTAEDDLKTDFYK.D	3	6.83	0.50	-3.18
IPI00009920	Complement component 6 precursor	R.TSNPYRVPANLENVGFEVQTAEDDLKTDFYK.D	4	4.59	0.47	-2.71
IPI00009920	Complement component 6 precursor	R.VPANLENVGFEVQTAEDDLKTDFYK.D	3	6.30	0.58	-3.92
IPI00009943	Tumor protein, translationally-controlled 1	K.YIKDYM*K.S	2	2.00	0.07	1.10
IPI00009950	Vesicular integral-membrane protein VIP36 precursor	A.DITDGNSEHLKR.E	2	3.14	0.29	-5.35
IPI00009950	Vesicular integral-membrane protein VIP36 precursor	I.TDGNSEHLKR.E	2	3.08	0.24	-2.65
IPI00009950	Vesicular integral-membrane protein VIP36 precursor	K.DNFHGLAIFLDTYPNDETTER.V	2	6.60	0.57	-8.03
IPI00009950	Vesicular integral-membrane protein VIP36 precursor	K.DNFHGLAIFLDTYPNDETTER.V	3	4.75	0.38	-5.52
IPI00009950	Vesicular integral-membrane protein VIP36 precursor	K.DNVDDPTGNFR.S	2	3.44	0.40	-2.92
IPI00009950	Vesicular integral-membrane protein VIP36 precursor	K.LFQLM*VEHTPDEESIDWTK.I	2	4.70	0.56	-4.23
IPI00009950	Vesicular integral-membrane protein VIP36 precursor	K.LFQLM*VEHTPDEESIDWTK.I	3	5.10	0.45	-3.64
IPI00009950	Vesicular integral-membrane protein VIP36 precursor	K.NCIDITGVR.L	1	2.82	0.20	-3.73
IPI00009950	Vesicular integral-membrane protein VIP36 precursor	K.NCIDITGVR.L	2	3.00	0.17	-1.67
IPI00009950	Vesicular integral-membrane protein VIP36 precursor	K.NLHGDGIALWYTR.D	2	4.00	0.39	-4.76
IPI00009950	Vesicular integral-membrane protein VIP36 precursor	R.DHDTFLAVR.Y	2	2.68	0.29	-1.36
IPI00009950	Vesicular integral-membrane protein VIP36 precursor	R.DRLVPGPVFGSK.D	2	3.05	0.37	-4.13

IPI00009950	Vesicular integral-membrane protein VIP36 precursor	R.DRLVPGPVFGSK.D	3	2.90	0.43	-2.79
IPI00009950	Vesicular integral-membrane protein VIP36 precursor	R.EHSLIKPYQGVGSSSM*PLWDFQGSTM*LTSQYVR.L	3	4.81	0.41	-3.56
IPI00009950	Vesicular integral-membrane protein VIP36 precursor	R.EHSLIKPYQGVGSSSM*PLWDFQGSTM*LTSQYVR.L	4	5.01	0.36	-3.88
IPI00009950	Vesicular integral-membrane protein VIP36 precursor	R.LPTGYYFGASAGTGDLSDNHDIISM*K.L	2	6.25	0.59	-3.89
IPI00009950	Vesicular integral-membrane protein VIP36 precursor	R.LPTGYYFGASAGTGDLSDNHDIISM*K.L	3	6.55	0.61	-5.84
IPI00009950	Vesicular integral-membrane protein VIP36 precursor	R.WTELAGCTADFR.N	2	4.10	0.50	-2.72
IPI00009950	Vesicular integral-membrane protein VIP36 precursor	V.PGPVFGSK.D	1	2.15	0.26	-4.55
IPI00009950	Vesicular integral-membrane protein VIP36 precursor	W.DFQGSTM*LTSQYVR.L	2	4.39	0.47	-2.18
IPI00009997	N-acetyllactosaminide beta-1,3-N-acetylglucosaminyltransferase	A.LASGGVLDASGDYR.V	2	3.61	0.30	-2.17
IPI00009997	N-acetyllactosaminide beta-1,3-N-acetylglucosaminyltransferase	A.SVDNLLHLSGLLER.W	3	3.93	0.45	-1.05
IPI00009997	N-acetyllactosaminide beta-1,3-N-acetylglucosaminyltransferase	D.PREPGEFALLR.S	2	3.14	0.15	-1.42
IPI00009997	N-acetyllactosaminide beta-1,3-N-acetylglucosaminyltransferase	E.PFYVAGGKVPTFDER.F	3	3.56	0.32	0.01
IPI00009997	N-acetyllactosaminide beta-1,3-N-acetylglucosaminyltransferase	E.PGEFALLR.S	2	2.93	0.30	-2.10
IPI00009997	N-acetyllactosaminide beta-1,3-N-acetylglucosaminyltransferase	G.GTALVVPAFEIR.R	2	2.95	0.36	-3.53
IPI00009997	N-acetyllactosaminide beta-1,3-N-acetylglucosaminyltransferase	H.ASVDNLLHLSGLLER.W	2	4.19	0.37	-2.71
IPI00009997	N-acetyllactosaminide beta-1,3-N-acetylglucosaminyltransferase	H.ASVDNLLHLSGLLER.W	3	5.57	0.49	-2.95
IPI00009997	N-acetyllactosaminide beta-1,3-N-acetylglucosaminyltransferase	K.AKYPNSPR.R	2	1.91	0.21	-2.60
IPI00009997	N-acetyllactosaminide beta-1,3-N-acetylglucosaminyltransferase	K.EALKFHPQK.E	1	2.65	0.08	-4.04
IPI00009997	N-acetyllactosaminide beta-1,3-N-acetylglucosaminyltransferase	K.EALKFHPQK.E	2	2.29	0.15	-3.11
IPI00009997	N-acetyllactosaminide beta-1,3-N-acetylglucosaminyltransferase	K.EALKFHPQKEAENQHNK.I	3	3.96	0.29	-3.26
IPI00009997	N-acetyllactosaminide beta-1,3-N-acetylglucosaminyltransferase	K.EEAQLATVLAYALSSHCPDM*R.A	2	4.53	0.48	-3.89

IPI00009997	N-acetyllactosaminide beta-1,3-N-acetylglucosaminyltransferase	K.EEAQLATVLAYALSSHCPDM*R.A	3	2.89	0.37	-3.23
IPI00009997	N-acetyllactosaminide beta-1,3-N-acetylglucosaminyltransferase	K.EEAQLATVLAYALSSHCPDM*R.A	4	3.65	0.17	-3.14
IPI00009997	N-acetyllactosaminide beta-1,3-N-acetylglucosaminyltransferase	K.FHPQKEAENQHNK.I	2	3.53	0.39	-4.36
IPI00009997	N-acetyllactosaminide beta-1,3-N-acetylglucosaminyltransferase	K.FHPQKEAENQHNK.I	4	1.92	0.18	-5.16
IPI00009997	N-acetyllactosaminide beta-1,3-N-acetylglucosaminyltransferase	K.TTM*DPNDVILATH.A	2	3.41	0.40	-2.69
IPI00009997	N-acetyllactosaminide beta-1,3-N-acetylglucosaminyltransferase	K.TTM*DPNDVILATHASVDNLLHLSGLLER.W	2	3.89	0.55	-3.25
IPI00009997	N-acetyllactosaminide beta-1,3-N-acetylglucosaminyltransferase	K.TTM*DPNDVILATHASVDNLLHLSGLLER.W	3	5.40	0.62	-8.09
IPI00009997	N-acetyllactosaminide beta-1,3-N-acetylglucosaminyltransferase	K.TTM*DPNDVILATHASVDNLLHLSGLLER.W	4	4.45	0.52	-5.97
IPI00009997	N-acetyllactosaminide beta-1,3-N-acetylglucosaminyltransferase	K.VPTFDER.F	1	2.00	0.17	-2.82
IPI00009997	N-acetyllactosaminide beta-1,3-N-acetylglucosaminyltransferase	L.LSGLHGQEEQDQYFEFFPPSPR.S	2	4.86	0.44	-3.75
IPI00009997	N-acetyllactosaminide beta-1,3-N-acetylglucosaminyltransferase	L.LSGLHGQEEQDQYFEFFPPSPR.S	3	4.29	0.38	-3.40
IPI00009997	N-acetyllactosaminide beta-1,3-N-acetylglucosaminyltransferase	L.SLLSGLHGQEEQDQYFEFFPPSPR.S	2	5.08	0.57	-1.96
IPI00009997	N-acetyllactosaminide beta-1,3-N-acetylglucosaminyltransferase	L.SLLSGLHGQEEQDQYFEFFPPSPR.S	3	3.99	0.50	-3.43
IPI00009997	N-acetyllactosaminide beta-1,3-N-acetylglucosaminyltransferase	L.YLSLLSGLHGQEEQDQYFEFFPPSPR.S	3	4.17	0.52	-3.45
IPI00009997	N-acetyllactosaminide beta-1,3-N-acetylglucosaminyltransferase	M.DPNDVILATHASVDNLLHLSGLLER.W	3	5.24	0.47	-3.18
IPI00009997	N-acetyllactosaminide beta-1,3-N-acetylglucosaminyltransferase	Q.LLYLSLLSGLHGQEEQDQYFEFFPPSPR.S	3	3.90	0.39	-4.25
IPI00009997	N-acetyllactosaminide beta-1,3-N-acetylglucosaminyltransferase	R.EGANYALVIDVDM*VPSEGLWR.G	2	5.63	0.50	-4.65
IPI00009997	N-acetyllactosaminide beta-1,3-N-acetylglucosaminyltransferase	R.EGANYALVIDVDM*VPSEGLWR.G	3	3.62	0.22	-8.08
IPI00009997	N-acetyllactosaminide beta-1,3-N-acetylglucosaminyltransferase	R.EM*LDQSNQWGGTALVVPAFEIR.R	2	5.70	0.57	-4.16
IPI00009997	N-acetyllactosaminide beta-1,3-N-acetylglucosaminyltransferase	R.EM*LDQSNQWGGTALVVPAFEIR.R	3	5.88	0.48	-5.31
IPI00009997	N-acetyllactosaminide beta-1,3-N-acetylglucosaminyltransferase	R.EM*LDQSNQWGGTALVVPAFEIRR.A	3	3.68	0.45	-5.77

IPI00009997	N-acetyllactosaminide beta-1,3-N-acetylglucosaminyltransferase	R.EPGEFALLR.S	1	1.77	0.28	-2.83
IPI00009997	N-acetyllactosaminide beta-1,3-N-acetylglucosaminyltransferase	R.EPGEFALLR.S	2	2.11	0.18	-3.02
IPI00009997	N-acetyllactosaminide beta-1,3-N-acetylglucosaminyltransferase	R.FRQYGFNR.I	2	2.57	0.07	-3.48
IPI00009997	N-acetyllactosaminide beta-1,3-N-acetylglucosaminyltransferase	R.GLREM*LDQSNQWGGTALVVPAFEIRR.A	4	3.13	0.19	-4.01
IPI00009997	N-acetyllactosaminide beta-1,3-N-acetylglucosaminyltransferase	R.ISQACELHVAGFDFEVLNEGFLVHK.G	3	7.22	0.56	-5.55
IPI00009997	N-acetyllactosaminide beta-1,3-N-acetylglucosaminyltransferase	R.ISQACELHVAGFDFEVLNEGFLVHK.G	4	5.08	0.46	-4.50
IPI00009997	N-acetyllactosaminide beta-1,3-N-acetylglucosaminyltransferase	R.PAYVVPWQDPWEPFYVAGGK.V	2	3.94	0.26	-1.54
IPI00009997	N-acetyllactosaminide beta-1,3-N-acetylglucosaminyltransferase	R.SCQEVFDK.L	1	2.30	0.20	-3.35
IPI00009997	N-acetyllactosaminide beta-1,3-N-acetylglucosaminyltransferase	R.SCQEVFDK.L	2	2.62	0.17	-1.74
IPI00009997	N-acetyllactosaminide beta-1,3-N-acetylglucosaminyltransferase	R.SCQEVFDKLAR.V	2	3.82	0.35	-3.80
IPI00009997	N-acetyllactosaminide beta-1,3-N-acetylglucosaminyltransferase	R.SCQEVFDKLAR.V	3	3.67	0.34	-2.85
IPI00009997	N-acetyllactosaminide beta-1,3-N-acetylglucosaminyltransferase	R.TALASGGVLDASGDYR.V	1	3.93	0.58	-3.61
IPI00009997	N-acetyllactosaminide beta-1,3-N-acetylglucosaminyltransferase	R.TALASGGVLDASGDYR.V	2	5.67	0.57	-8.41
IPI00009997	N-acetyllactosaminide beta-1,3-N-acetylglucosaminyltransferase	R.TALASGGVLDASGDYR.V	3	4.68	0.44	-3.36
IPI00009997	N-acetyllactosaminide beta-1,3-N-acetylglucosaminyltransferase	R.VAM*HLVCPSRYEAAVPDPREPGEFALLR.S	3	2.78	0.25	-3.84
IPI00009997	N-acetyllactosaminide beta-1,3-N-acetylglucosaminyltransferase	R.WEGPLSVSVFAATK.E	2	4.24	0.56	-4.64
IPI00009997	N-acetyllactosaminide beta-1,3-N-acetylglucosaminyltransferase	R.WVNLPEESLLR.P	2	3.22	0.14	-2.20
IPI00009997	N-acetyllactosaminide beta-1,3-N-acetylglucosaminyltransferase	R.WVNLPEESLLRPAYVVPWQDPWEPFYVAGGK.V	3	5.98	0.46	-6.26
IPI00009997	N-acetyllactosaminide beta-1,3-N-acetylglucosaminyltransferase	R.WVNLPEESLLRPAYVVPWQDPWEPFYVAGGK.V	4	5.81	0.50	-5.93
IPI00009997	N-acetyllactosaminide beta-1,3-N-acetylglucosaminyltransferase	R.YEAAVPDPR.E	1	1.53	0.23	-4.42
IPI00009997	N-acetyllactosaminide beta-1,3-N-acetylglucosaminyltransferase	R.YEAAVPDPR.E	2	3.22	0.42	-3.35

IPI00009997	N-acetyllactosaminide beta-1,3-N-acetylglucosaminyltransferase	R.YEAAVPDPREPGEFALLR.S	2	3.18	0.37	-3.93
IPI00009997	N-acetyllactosaminide beta-1,3-N-acetylglucosaminyltransferase	R.YEAAVPDPREPGEFALLR.S	3	2.73	0.38	-3.68
IPI00009997	N-acetyllactosaminide beta-1,3-N-acetylglucosaminyltransferase	W.EPFYVAGGK.V	1	2.34	0.25	-3.05
IPI00009997	N-acetyllactosaminide beta-1,3-N-acetylglucosaminyltransferase	W.GGTALVVPAFEIR.R	2	3.71	0.36	-6.82
IPI00009997	N-acetyllactosaminide beta-1,3-N-acetylglucosaminyltransferase	W.VNLPEESLLRPAYVVPWQDPWEPFYVAGGK.V	3	4.81	0.14	-7.05
IPI00009997	N-acetyllactosaminide beta-1,3-N-acetylglucosaminyltransferase	Y.LSLLSGLHGQEEQDQYFEFFPPSPR.S	2	5.28	0.56	-1.48
IPI00009997	N-acetyllactosaminide beta-1,3-N-acetylglucosaminyltransferase	Y.LSLLSGLHGQEEQDQYFEFFPPSPR.S	3	4.89	0.47	-1.58
IPI00010118	Isoform 1 of Prostate tumor overexpressed gene 1 protein	R.AVRSRSWPASPR.G	2	2.25	0.11	
IPI00010133	Coronin-1A	R.AAPEASGTPSSDAVSR.L	2	4.19	0.46	-3.89
IPI00010148	Brain-specific polypeptide PEP-19	K.KVQEEFDIDM*DAPETER.A	2	5.10	0.52	-2.54
IPI00010148	Brain-specific polypeptide PEP-19	K.KVQEEFDIDM*DAPETER.A	3	3.72	0.33	-2.09
IPI00010148	Brain-specific polypeptide PEP-19	K.VQEEFDIDM*DAPETER.A	2	3.51	0.34	2.00
IPI00010148	Brain-specific polypeptide PEP-19	R.AAVAIQSQFR.K	2	3.48	0.27	-2.88
IPI00010154	Rab GDP dissociation inhibitor alpha	K.FDLGQDVIDFTGHALALYR.T	2	4.85	0.52	-4.24
IPI00010154	Rab GDP dissociation inhibitor alpha	K.FDLGQDVIDFTGHALALYR.T	3	4.02	0.36	-3.60
IPI00010154	Rab GDP dissociation inhibitor alpha	K.FLM*ANGQLVK.M	2	2.34	0.08	-0.96
IPI00010154	Rab GDP dissociation inhibitor alpha	K.FLVFVANFDENDPK.T	2	3.64	0.43	-1.20
IPI00010154	Rab GDP dissociation inhibitor alpha	K.FLVFVANFDENDPKTFEGVDPQTTSM*R.D	3	4.10	0.43	-3.51
IPI00010154	Rab GDP dissociation inhibitor alpha	K.IYKVPSTETEALASNLM*GM*FEK.R	3	4.31	0.31	-1.86
IPI00010154	Rab GDP dissociation inhibitor alpha	K.LYSESLAR.Y	1	1.68	0.11	-2.03
IPI00010154	Rab GDP dissociation inhibitor alpha	K.LYSESLAR.Y	2	2.53	0.20	-3.09
IPI00010154	Rab GDP dissociation inhibitor alpha	K.NTNDANSCQIIIPQNQVNR.K	2	3.37	0.33	-3.67
IPI00010154	Rab GDP dissociation inhibitor alpha	K.QLICDPSYIPDR.V	2	3.11	0.27	-1.90
IPI00010154	Rab GDP dissociation inhibitor alpha	K.SPYLYPLYGLGELPQGFAR.L	2	6.04	0.56	-4.21
IPI00010154	Rab GDP dissociation inhibitor alpha	K.SPYLYPLYGLGELPQGFAR.L	3	4.46	0.33	-4.06
IPI00010154	Rab GDP dissociation inhibitor alpha	K.TFEGVDPQTTSM*R.D	2	2.51	0.19	-0.38
IPI00010154	Rab GDP dissociation inhibitor alpha	K.VLHM*DRNPYYGGESSSITPLEELYKR.F	3	6.68	0.49	-1.27
IPI00010154	Rab GDP dissociation inhibitor alpha	K.VLHM*DRNPYYGGESSSITPLEELYKR.F	4	3.92	0.33	-0.83
IPI00010154	Rab GDP dissociation inhibitor alpha	K.VVEGSFVYKGGK.I	2	2.70	0.34	-2.89
IPI00010154	Rab GDP dissociation inhibitor alpha	R.IKLYSESLAR.Y	1	2.49	0.27	-1.77
IPI00010154	Rab GDP dissociation inhibitor alpha	R.IKLYSESLAR.Y	2	2.49	0.18	-1.31
IPI00010154	Rab GDP dissociation inhibitor alpha	R.KFDLGQDVIDFTGHALALYR.T	2	4.71	0.49	-7.20
IPI00010154	Rab GDP dissociation inhibitor alpha	R.KFDLGQDVIDFTGHALALYR.T	3	7.26	0.56	-6.47
IPI00010154	Rab GDP dissociation inhibitor alpha	R.KFDLGQDVIDFTGHALALYR.T	4	5.53	0.42	-3.92

IPI00010154	Rab GDP dissociation inhibitor alpha	R.KQNDVFGEAEQ.-	2	3.55	0.31	-0.90
IPI00010154	Rab GDP dissociation inhibitor alpha	R.NPYYGGESSSITPLEELYKR.F	3	3.75	0.38	-2.90
IPI00010154	Rab GDP dissociation inhibitor alpha	R.TDDYLDQPCLETVNR.I	2	4.51	0.43	-4.37
IPI00010182	Isoform a 1 of Acyl-CoA-binding protein	K.AYINKVEELK.K	2	2.97	0.23	-2.52
IPI00010182	Isoform a 1 of Acyl-CoA-binding protein	K.AYINKVEELKK.K	2	2.71	0.14	-2.90
IPI00010182	Isoform a 1 of Acyl-CoA-binding protein	K.QATVGDINTERPGM*LDFTGK.A	2	3.04	0.40	-3.06
IPI00010182	Isoform a 1 of Acyl-CoA-binding protein	K.QATVGDINTERPGM*LDFTGK.A	3	2.34	0.11	-3.00
IPI00010193	Isoform 1 of Interferon-alpha/beta receptor beta chain precursor	G.ISYDSPDYTDESCTFK.I	2	3.61	0.45	-6.76
IPI00010207	Ubiquitin-fold modifier 1 precursor	K.FAAEEFKVPAATSAIITNDGIGINPAQTAGNVFLK.H	3	4.89	0.45	-2.51
IPI00010295	Carboxypeptidase N catalytic chain precursor	K.GM*VLDENYNNLANAVISVSGINHDVTSGDHGDYFR.L	4	5.54	0.47	-2.76
IPI00010295	Carboxypeptidase N catalytic chain precursor	K.VQNECPGITR.V	2	2.53	0.18	
IPI00010295	Carboxypeptidase N catalytic chain precursor	R.EALIQFLEQVHQGIK.G	2	4.53	0.38	-2.81
IPI00010295	Carboxypeptidase N catalytic chain precursor	R.EALIQFLEQVHQGIK.G	3	2.47	0.23	-1.82
IPI00010295	Carboxypeptidase N catalytic chain precursor	R.HLYVLEFSDHPGIHEPLEPEVK.Y	3	3.98	0.46	-1.75
IPI00010295	Carboxypeptidase N catalytic chain precursor	R.HLYVLEFSDHPGIHEPLEPEVK.Y	4	3.87	0.39	-1.04
IPI00010295	Carboxypeptidase N catalytic chain precursor	R.IHILPSM*NPDGYEVAAAQGPNKPGYLVGR.N	3	2.98	0.29	-4.11
IPI00010295	Carboxypeptidase N catalytic chain precursor	R.IVQLIQDTR.I	2	3.27	0.09	-1.89
IPI00010295	Carboxypeptidase N catalytic chain precursor	R.NFPDLNTYIYYNEK.Y	2	2.39	0.10	-4.93
IPI00010295	Carboxypeptidase N catalytic chain precursor	R.NNANGVDLNR.N	2	2.96	0.28	-0.81
IPI00010303	Serpin B4	K.FM*FDLFQQFR.K	2	3.04	0.27	-3.22
IPI00010343	Sodium/calcium exchanger 2 precursor	K.AGSDYEYSEGTLVFKPGETQK.E	3	2.84	0.13	-3.78
IPI00010343	Sodium/calcium exchanger 2 precursor	R.LVAPLLATVTILDDDH.A	2	4.59	0.58	-3.90
IPI00010343	Sodium/calcium exchanger 2 precursor	R.LVAPLLATVTILDDDH.A	3	4.15	0.33	-2.47
IPI00010346	Neurolysin, mitochondrial precursor	K.EVRAASTEADKRLSRFDIEMSMRGDIFER.I	3	3.04	0.14	
IPI00010348	Deoxyribonuclease-2-alpha precursor	A.LTCYGDSGQPVDWFVVYK.L	2	4.82	0.55	-3.34
IPI00010348	Deoxyribonuclease-2-alpha precursor	A.LTCYGDSGQPVDWFVVYKLPALR.G	3	3.98	0.39	-2.21
IPI00010348	Deoxyribonuclease-2-alpha precursor	K.QLTYTYPWVYNYQLEGIFAQEFPDLENVVK.G	3	4.88	0.46	-1.91
IPI00010348	Deoxyribonuclease-2-alpha precursor	R.ALINSPEGAVGR.S	2	2.60	0.27	-3.89
IPI00010348	Deoxyribonuclease-2-alpha precursor	R.GGGTLCAQLPALWK.A	2	3.36	0.25	-2.20
IPI00010360	Isoform 1 of Collagen alpha-3(IV) chain precursor	K.CGDPGLPGPDGEPGIPGIGFPGPPGPKGDQGFPGTK.G	3	1.53	0.12	-2.70
IPI00010369	Testis-expressed sequence 15 protein	K.LQDLTLR.D	2	2.33	0.09	-3.01
IPI00010381	VPS10 domain-containing receptor SorCS3 precursor	K.LYDFNLGSVTESSLWR.S	2	4.46	0.55	-4.31
IPI00010381	VPS10 domain-containing receptor SorCS3 precursor	R.AGPELLPQQGGGR.G	1	2.08	0.16	-1.84
IPI00010381	VPS10 domain-containing receptor SorCS3 precursor	R.AGPELLPQQGGGR.G	2	3.87	0.26	-3.06
IPI00010381	VPS10 domain-containing receptor SorCS3 precursor	R.GGEM*QVEAGGTSPAGER.R	2	5.18	0.46	-2.98
IPI00010381	VPS10 domain-containing receptor SorCS3 precursor	R.GGEM*QVEAGGTSPAGER.R	3	2.46	0.13	-2.80

IPI00010381	VPS10 domain-containing receptor SorCS3 precursor	R.GGEM*QVEAGGTSPAGERR.G	3	3.32	0.34	-3.02
IPI00010381	VPS10 domain-containing receptor SorCS3 precursor	R.GIYFTLAM*ENIK.S	2	3.02	0.37	-1.42
IPI00010381	VPS10 domain-containing receptor SorCS3 precursor	R.IQECAETTR.S	2	2.75	0.26	-4.73
IPI00010381	VPS10 domain-containing receptor SorCS3 precursor	R.RAGPELLPQQGGGR.G	2	3.97	0.46	-2.59
IPI00010381	VPS10 domain-containing receptor SorCS3 precursor	R.RAGPELLPQQGGGR.G	3	3.49	0.22	-0.76
IPI00010381	VPS10 domain-containing receptor SorCS3 precursor	R.STDYGTTYEK.L	2	3.12	0.29	-2.90
IPI00010381	VPS10 domain-containing receptor SorCS3 precursor	R.STDYGTTYEKLNDK.V	2	3.71	0.51	0.07
IPI00010381	VPS10 domain-containing receptor SorCS3 precursor	R.VPFVAIR.N	2	1.86	0.12	-2.70
IPI00010402	Putative uncharacterized protein	R.IQYQLVDISQDNALRDEM*R.A	2	2.76	0.39	-2.84
IPI00010402	Putative uncharacterized protein	R.IQYQLVDISQDNALRDEM*R.A	3	2.49	0.10	-1.24
IPI00010402	Putative uncharacterized protein	R.VYSTSVTGSR.E	1	1.89	0.15	-2.82
IPI00010402	Putative uncharacterized protein	R.VYSTSVTGSR.E	2	3.98	0.45	-3.05
IPI00010405	Isoform Long of Tyrosine-protein kinase transmembrane receptor ROR1 precursor	R.DLCRDECEILENVLCQTEYIFAR.S	3	3.54	0.38	-3.97
IPI00010442	Phospholemman precursor	R.TGEPDEEEGTFR.S	2	1.51	0.16	-0.07
IPI00010470	Isoform SNAP-25b of Synaptosomal-associated protein 25	A.LDM*GNEIDTQNR.Q	2	3.42	0.32	-1.32
IPI00010470	Isoform SNAP-25b of Synaptosomal-associated protein 25	L.DM*GNEIDTQNR.Q	2	3.73	0.33	-3.83
IPI00010470	Isoform SNAP-25b of Synaptosomal-associated protein 25	R.RADQLADESLESTRR.M	3	3.48	0.21	-2.21
IPI00010471	Plastin-2	K.AYYHLLEQVAPKGDEEGVPAVVIDM*SGLR.E	4	2.45	0.16	-0.83
IPI00010471	Plastin-2	K.FSLVGIGGQDLNEGNR.T	2	4.65	0.46	-2.91
IPI00010471	Plastin-2	K.IGNFSTDIKDSK.A	3	2.09	0.17	-3.35
IPI00010471	Plastin-2	K.ISTSLPVLDLIDAIQPGSINYDLLK.T	2	3.21	0.41	-3.69
IPI00010471	Plastin-2	K.ISTSLPVLDLIDAIQPGSINYDLLK.T	3	5.65	0.35	-2.05
IPI00010471	Plastin-2	K.ISTSLPVLDLIDAIQPGSINYDLLKTENLNDDEKLNNAK.Y	3	3.87	0.46	-4.10
IPI00010471	Plastin-2	K.ISTSLPVLDLIDAIQPGSINYDLLKTENLNDDEKLNNAK.Y	4	3.53	0.22	-4.31
IPI00010471	Plastin-2	K.M*INLSVPDTIDER.T	2	3.99	0.32	-2.96
IPI00010471	Plastin-2	K.TENLNDDEKLNNAK.Y	2	4.88	0.30	-2.44
IPI00010471	Plastin-2	K.VDTDGNGYISFNELNDLFK.A	2	2.41	0.26	-5.10
IPI00010471	Plastin-2	R.VNHLYSDLSDALVIFQLYEK.I	3	2.58	0.15	-3.35
IPI00010471	Plastin-2	R.VYALPEDLVEVNPK.M	2	3.42	0.33	-3.38
IPI00010575	KIAA1466 protein	-.M*QGDSKFSSQGTGPPYQDLSTK.S	2	2.49	0.10	

IPI00010706	Glutathione synthetase	K.EGIAQTVFLGLNR.S	2	3.53	0.33	-3.31
IPI00010706	Glutathione synthetase	K.QIEINTISASFGGLASR.T	2	4.27	0.55	-4.02
IPI00010706	Glutathione synthetase	K.VQQELSRPGM*LEM*LLPGQPEAVAR.L	3	2.81	0.13	-2.55
IPI00010706	Glutathione synthetase	R.AIENELLAR.N	2	2.33	0.18	-2.39
IPI00010737	Thrombomodulin precursor	R.SSVAADVISLLLNGDGGVGR.R	3	3.50	0.25	-3.06
IPI00010790	Biglycan precursor	K.EISPDTTLLDLQNNDISELRK.D	3	2.95	0.39	-4.80
IPI00010790	Biglycan precursor	K.IQAIELEDLLR.Y	2	3.39	0.14	-3.56
IPI00010790	Biglycan precursor	R.ELHLDNNK.L	1	2.93	0.07	-4.09
IPI00010790	Biglycan precursor	R.LGLGHNQIR.M	2	2.18	0.08	-2.21
IPI00010790	Biglycan precursor	R.VVQCSDLGLK.S	2	3.11	0.37	-2.78
IPI00010796	Protein disulfide-isomerase precursor	K.LKAEGSEIR.L	2	2.26	0.10	0.99
IPI00010796	Protein disulfide-isomerase precursor	K.M*DSTANEVEAVK.V	2	3.79	0.41	-3.88
IPI00010796	Protein disulfide-isomerase precursor	K.QFLQAAEAIDDIPFGITSNSDVFSK.Y	3	4.12	0.41	-4.17
IPI00010796	Protein disulfide-isomerase precursor	K.VDATEESDLAQQYGVR.G	2	4.48	0.43	-4.66
IPI00010796	Protein disulfide-isomerase precursor	K.VDATEESDLAQQYGVR.G	3	2.42	0.12	-2.37
IPI00010796	Protein disulfide-isomerase precursor	K.YKPESEELTAER.I	2	2.68	0.16	-2.87
IPI00010796	Protein disulfide-isomerase precursor	K.YQLDKDGVVLFK.K	3	2.92	0.40	-1.59
IPI00010796	Protein disulfide-isomerase precursor	K.YQLDKDGVVLFKK.F	3	2.67	0.25	0.23
IPI00010796	Protein disulfide-isomerase precursor	R.ILEFFGLK.K	2	2.39	0.20	-1.69
IPI00010796	Protein disulfide-isomerase precursor	R.NNFEGEVTKENLLDFIK.H	3	3.18	0.27	-2.91
IPI00010796	Protein disulfide-isomerase precursor	R.TGPAATTLPDGAAAESLVESSEVAVIGFFK.D	3	4.50	0.49	-8.44
IPI00010808	Interferon-gamma receptor alpha chain precursor	R.VYNVYVR.M	1	1.76	0.11	-0.70
IPI00010810	Electron transfer flavoprotein subunit alpha, mitochondrial precursor	K.TIVAINKDPEAPIFQVADYGIVADLFK.V	3	4.00	0.45	-4.50
IPI00010810	Electron transfer flavoprotein subunit alpha, mitochondrial precursor	R.GTSFDAAATSGGSASSEK.A	2	1.98	0.12	-3.20
IPI00010863	Copper transport protein ATOX1	K.LGGVKYDIDLPNKK.V	3	3.65	0.26	-0.43
IPI00010895	Tubby-related protein 2	K.GEGGTDSDHM*R.H	2	2.07	0.17	
IPI00010896	Chloride intracellular channel protein 1	K.LAALNPESNTAGLDIFAK.F	2	5.14	0.57	-4.16
IPI00010896	Chloride intracellular channel protein 1	K.NSNPALNDNLEK.G	2	3.15	0.43	-1.76
IPI00010896	Chloride intracellular channel protein 1	K.VLDNYLTSPLPEEVDETSAEDEGVSQR.K	3	3.30	0.37	-3.29
IPI00010903	Dopey family member 1	K.IIGPKRLAK.D	2	1.25	0.06	-1.31
IPI00010949	Isoform 1 of Sialate O-acetylesterase precursor	K.KSSDDGFPQIR.W	2	3.27	0.24	-2.55
IPI00010949	Isoform 1 of Sialate O-acetylesterase precursor	K.M*PNTFM*AVAM*DLCDRDSPFGSIHPR.D	4	4.46	0.47	-2.56
IPI00010949	Isoform 1 of Sialate O-acetylesterase precursor	K.QGSIPYDSVTGPSK.H	2	2.94	0.38	-3.66
IPI00010949	Isoform 1 of Sialate O-acetylesterase precursor	K.SSDDGFPQIR.W	2	2.78	0.24	-3.34
IPI00010949	Isoform 1 of Sialate O-acetylesterase precursor	K.YM*SAVCWLFGR.H	2	3.14	0.46	-3.40
IPI00010949	Isoform 1 of Sialate O-acetylesterase precursor	R.DKQTVAYR.L	2	2.30	0.23	-1.98
IPI00010949	Isoform 1 of Sialate O-acetylesterase precursor	R.ELSNTAAYQSVR.I	2	3.91	0.36	-2.76
IPI00010949	Isoform 1 of Sialate O-acetylesterase precursor	R.FASYINNDM*VLQK.E	2	3.75	0.38	-2.61
IPI00010949	Isoform 1 of Sialate O-acetylesterase precursor	R.FFPFGLVQLSSDLSK.K	2	5.45	0.46	-4.32
IPI00010949	Isoform 1 of Sialate O-acetylesterase precursor	R.FFPFGLVQLSSDLSK.K	3	4.01	0.37	-2.35

IPI00010949	Isoform 1 of Sialate O-acetylesterase precursor	R.FFPFGLVQLSSDLSKK.S	2	3.62	0.42	-2.74
IPI00010949	Isoform 1 of Sialate O-acetylesterase precursor	R.YAWTTWPCEYK.Q	2	3.36	0.34	-3.85
IPI00011051	T-cell leukemia homeobox protein 1	K.DRFTGHPYQNRTPPK.K	3	2.58	0.09	-0.74
IPI00011094	Complement C1q tumor necrosis factor-related protein 4 precursor	R.NRDEVQALAFDEQR.R	2	4.05	0.40	-2.58
IPI00011094	Complement C1q tumor necrosis factor-related protein 4 precursor	R.TTPLEGTSEM*AVTFDK.V	2	3.85	0.51	-1.82
IPI00011094	Complement C1q tumor necrosis factor-related protein 4 precursor	R.VPGAYFFSFTAGK.A	2	3.25	0.54	-4.64
IPI00011140	Protein NOV homolog precursor	K.NNEAFLQELELK.T	2	4.39	0.33	-4.56
IPI00011140	Protein NOV homolog precursor	K.TIQAEFQCSPGQIVK.K	2	5.43	0.47	-3.21
IPI00011140	Protein NOV homolog precursor	R.AVLDGCSCCLVCAR.Q	2	3.90	0.43	-2.62
IPI00011140	Protein NOV homolog precursor	R.CPATPPTCAPGVR.A	2	2.31	0.06	-1.70
IPI00011140	Protein NOV homolog precursor	R.CQLDVLLPEPNCPAPR.K	2	4.49	0.50	-3.50
IPI00011140	Protein NOV homolog precursor	R.CQLDVLLPEPNCPAPR.K	3	2.61	0.13	-3.81
IPI00011140	Protein NOV homolog precursor	R.DGQIGCVPR.C	1	2.02	0.21	-4.16
IPI00011140	Protein NOV homolog precursor	R.DGQIGCVPR.C	2	3.15	0.25	-3.12
IPI00011218	Macrophage colony-stimulating factor 1 receptor precursor	G.IPVIEPSVPELVVKPGATVTLR.C	2	4.48	0.56	-4.53
IPI00011218	Macrophage colony-stimulating factor 1 receptor precursor	G.IPVIEPSVPELVVKPGATVTLR.C	3	5.24	0.62	-4.46
IPI00011218	Macrophage colony-stimulating factor 1 receptor precursor	I.PGPPALTLVPAELVR.I	2	4.45	0.49	-2.59
IPI00011218	Macrophage colony-stimulating factor 1 receptor precursor	I.PQQSDFHNNR.Y	2	2.91	0.36	-3.58
IPI00011218	Macrophage colony-stimulating factor 1 receptor precursor	K.FIQSQDYQCSALM*GGR.K	2	5.34	0.56	-4.06
IPI00011218	Macrophage colony-stimulating factor 1 receptor precursor	K.FIQSQDYQCSALM*GGR.K	3	4.49	0.39	-1.07
IPI00011218	Macrophage colony-stimulating factor 1 receptor precursor	K.LAIPQQSDFHNNR.Y	2	3.22	0.44	-3.92
IPI00011218	Macrophage colony-stimulating factor 1 receptor precursor	K.LAIPQQSDFHNNR.Y	3	2.01	0.16	-3.93
IPI00011218	Macrophage colony-stimulating factor 1 receptor precursor	K.LAIPQQSDFHNNRYQK.V	2	3.31	0.38	-2.98
IPI00011218	Macrophage colony-stimulating factor 1 receptor precursor	K.VIPGPPALTLVPAELVR.I	2	2.84	0.51	-3.23
IPI00011218	Macrophage colony-stimulating factor 1 receptor precursor	K.VIPGPPALTLVPAELVR.I	3	3.40	0.33	-2.05
IPI00011218	Macrophage colony-stimulating factor 1 receptor precursor	R.AHNSVGSGSWAFIPISAG.A	2	3.00	0.39	-2.06

IPI00011218	Macrophage colony-stimulating factor 1 receptor precursor	R.AHNSVGSGSWAFIPISAGA.H	2	3.77	0.40	-3.09
IPI00011218	Macrophage colony-stimulating factor 1 receptor precursor	R.AKFIQSQDYQCSALM*GGR.K	2	5.43	0.54	-2.79
IPI00011218	Macrophage colony-stimulating factor 1 receptor precursor	R.ALTFELTLR.Y	2	2.55	0.20	-4.48
IPI00011218	Macrophage colony-stimulating factor 1 receptor precursor	R.CDEAQVLQVWDDPYPEVLSQEPFHK.V	3	5.68	0.36	-3.64
IPI00011218	Macrophage colony-stimulating factor 1 receptor precursor	R.HTFTLSLPR.L	1	2.08	0.08	-3.76
IPI00011218	Macrophage colony-stimulating factor 1 receptor precursor	R.HTFTLSLPR.L	2	2.30	0.28	-3.31
IPI00011218	Macrophage colony-stimulating factor 1 receptor precursor	R.LKVQKVIPGPPALTLVPAELVR.I	3	3.60	0.41	-3.76
IPI00011218	Macrophage colony-stimulating factor 1 receptor precursor	R.LKVQKVIPGPPALTLVPAELVR.I	4	2.46	0.29	-3.34
IPI00011218	Macrophage colony-stimulating factor 1 receptor precursor	R.VVESAYLNLSSEQNLIQEVTVGEGLNLK.V	3	3.24	0.13	-5.25
IPI00011229	Cathepsin D precursor	K.AIGAVPLIQGEYM*IPCEK.V	2	4.27	0.44	-4.05
IPI00011229	Cathepsin D precursor	K.AIGAVPLIQGEYM*IPCEK.V	3	4.47	0.34	-3.05
IPI00011229	Cathepsin D precursor	K.AIGAVPLIQGEYM*IPCEKVSTLPAITLK.L	3	3.89	0.49	-3.09
IPI00011229	Cathepsin D precursor	K.AIGAVPLIQGEYM*IPCEKVSTLPAITLK.L	4	3.68	0.33	-2.04
IPI00011229	Cathepsin D precursor	K.AYWQVHLDQVEVASGLTLCK.E	3	2.83	0.17	-1.81
IPI00011229	Cathepsin D precursor	K.EGCEAIVDTGTSLM*VGPVDEVR.E	3	5.04	0.49	-3.16
IPI00011229	Cathepsin D precursor	K.FDGILGM*AYPR.I	2	3.64	0.41	-3.20
IPI00011229	Cathepsin D precursor	K.GYKLSPEDYTLK.V	2	2.77	0.26	-3.38
IPI00011229	Cathepsin D precursor	K.LGGKGYKLSPEDYTLK.V	2	4.66	0.39	-3.02
IPI00011229	Cathepsin D precursor	K.LLDIACWIHHK.Y	2	3.07	0.25	-3.06
IPI00011229	Cathepsin D precursor	K.LSPEDYTLK.V	1	1.72	0.12	-5.95
IPI00011229	Cathepsin D precursor	K.LSPEDYTLK.V	2	1.60	0.23	-2.77
IPI00011229	Cathepsin D precursor	K.LVDQNIFSFYLSR.D	1	2.62	0.29	-1.43
IPI00011229	Cathepsin D precursor	K.LVDQNIFSFYLSR.D	2	4.88	0.50	-6.03
IPI00011229	Cathepsin D precursor	K.LVDQNIFSFYLSR.D	3	4.76	0.32	-3.40
IPI00011229	Cathepsin D precursor	K.LVDQNIFSFYLSRDPDAQPGGELM*LGGTDSK.Y	2	1.85	0.17	-2.48
IPI00011229	Cathepsin D precursor	K.LVDQNIFSFYLSRDPDAQPGGELM*LGGTDSK.Y	3	6.55	0.58	-4.14
IPI00011229	Cathepsin D precursor	K.LVDQNIFSFYLSRDPDAQPGGELM*LGGTDSK.Y	4	5.17	0.40	-4.15
IPI00011229	Cathepsin D precursor	K.LVDQNIFSFYLSRDPDAQPGGELM*LGGTDSKYYK.G	4	4.10	0.33	-3.00
IPI00011229	Cathepsin D precursor	K.QPGITFIAAK.F	1	1.41	0.14	-2.79
IPI00011229	Cathepsin D precursor	K.QPGITFIAAK.F	2	1.26	0.15	-1.94
IPI00011229	Cathepsin D precursor	K.VSTLPAITLK.L	1	1.63	0.05	-2.34
IPI00011229	Cathepsin D precursor	K.VSTLPAITLK.L	2	2.57	0.21	-2.55
IPI00011229	Cathepsin D precursor	K.YNSDKSSTYVK.N	1	3.33	0.34	-4.12

IPI00011229	Cathepsin D precursor	K.YNSDKSSTYVK.N	2	3.40	0.38	-5.04
IPI00011229	Cathepsin D precursor	K.YNSDKSSTYVK.N	3	1.54	0.15	-2.42
IPI00011229	Cathepsin D precursor	K.YSQAVPAVTEGPIPEVLK.N	2	5.02	0.57	-4.82
IPI00011229	Cathepsin D precursor	R.DNNRVGFAEAAR.L	3	3.75	0.38	-2.45
IPI00011229	Cathepsin D precursor	R.DPDAQPGGELM*LGGTDSK.Y	2	5.39	0.51	-2.93
IPI00011229	Cathepsin D precursor	R.DPDAQPGGELM*LGGTDSK.Y	3	4.78	0.39	-1.60
IPI00011229	Cathepsin D precursor	R.ISVNNVLPVFDNLM*QQK.L	2	4.71	0.49	-6.38
IPI00011229	Cathepsin D precursor	R.ISVNNVLPVFDNLM*QQK.L	3	5.83	0.51	-4.13
IPI00011229	Cathepsin D precursor	R.QVFGEATK.Q	1	1.97	0.08	-2.72
IPI00011229	Cathepsin D precursor	R.QVFGEATK.Q	2	1.85	0.11	-2.50
IPI00011229	Cathepsin D precursor	R.QVFGEATKQPGITFIAAK.F	2	3.89	0.42	-2.88
IPI00011229	Cathepsin D precursor	R.QVFGEATKQPGITFIAAK.F	3	3.55	0.37	-2.60
IPI00011229	Cathepsin D precursor	R.QVFGEATKQPGITFIAAKFDGILGM*AYPR.I	4	3.19	0.15	-4.70
IPI00011229	Cathepsin D precursor	R.RTM*SEVGGSVEDLIAK.G	3	4.27	0.43	-0.64
IPI00011229	Cathepsin D precursor	R.TM*SEVGGSVEDLIAK.G	2	4.91	0.56	-3.55
IPI00011229	Cathepsin D precursor	R.TM*SEVGGSVEDLIAK.G	3	3.92	0.28	-0.88
IPI00011229	Cathepsin D precursor	R.TM*SEVGGSVEDLIAKGPVSK.Y	2	3.77	0.12	-2.84
IPI00011229	Cathepsin D precursor	R.TM*SEVGGSVEDLIAKGPVSK.Y	3	3.16	0.32	-1.53
IPI00011229	Cathepsin D precursor	R.VGFAEAAR.L	1	2.36	0.11	-2.75
IPI00011229	Cathepsin D precursor	R.VGFAEAAR.L	2	2.98	0.19	-3.84
IPI00011229	Cathepsin D precursor	R.VGFAEAARL.-	1	2.08	0.15	-2.80
IPI00011229	Cathepsin D precursor	R.VGFAEAARL.-	2	3.21	0.23	-3.49
IPI00011229	Cathepsin D precursor	R.YYTVFDR.D	1	2.10	0.09	-2.86
IPI00011229	Cathepsin D precursor	R.YYTVFDR.D	2	2.12	0.16	-1.75
IPI00011252	Complement component C8 alpha chain precursor	K.AKM*ESLGITSR.D	2	2.90	0.25	-0.38
IPI00011252	Complement component C8 alpha chain precursor	K.AM*AVEDIISR.V	1	1.25	0.23	-3.71
IPI00011252	Complement component C8 alpha chain precursor	K.M*ESLGITSR.D	2	2.83	0.15	-1.66
IPI00011252	Complement component C8 alpha chain precursor	K.YHFEALADTGISSEFYDNANDLLSK.V	2	6.48	0.68	-2.76
IPI00011252	Complement component C8 alpha chain precursor	K.YHFEALADTGISSEFYDNANDLLSK.V	3	6.62	0.58	-5.36
IPI00011252	Complement component C8 alpha chain precursor	K.YNPVVIDFEM*QPIHEVLR.H	2	5.08	0.53	-3.20
IPI00011252	Complement component C8 alpha chain precursor	K.YNPVVIDFEM*QPIHEVLR.H	3	2.52	0.15	-4.02
IPI00011252	Complement component C8 alpha chain precursor	Q.AQCGQDFQCK.E	2	3.60	0.41	0.77
IPI00011252	Complement component C8 alpha chain precursor	R.AIDEDCSQYEPIPGSQK.A	2	4.48	0.54	-3.65
IPI00011252	Complement component C8 alpha chain precursor	R.AIDEDCSQYEPIPGSQK.A	3	3.50	0.31	-2.39
IPI00011252	Complement component C8 alpha chain precursor	R.ALDQYLM*EFNACR.C	2	4.70	0.46	-2.22
IPI00011252	Complement component C8 alpha chain precursor	R.ECDNPAPQNGGASCPGR.K	2	3.21	0.57	-3.20
IPI00011252	Complement component C8 alpha chain precursor	R.HTSLGPLEAK.R	2	2.18	0.35	-1.35
IPI00011252	Complement component C8 alpha chain precursor	R.HTSLGPLEAKR.Q	3	2.41	0.28	-5.51
IPI00011252	Complement component C8 alpha chain precursor	R.KAM*AVEDIISR.V	2	3.31	0.33	-1.47
IPI00011252	Complement component C8 alpha chain precursor	R.KAM*AVEDIISR.V	3	2.63	0.06	-3.42
IPI00011252	Complement component C8 alpha chain precursor	R.LGSLGAACEQTQTEGAK.A	2	4.39	0.45	0.38
IPI00011252	Complement component C8 alpha chain precursor	R.LYYGDDEKYFR.K	2	3.22	0.39	-2.64

IPI00011252	Complement component C8 alpha chain precursor	R.LYYGDDEKYFR.K	3	1.92	0.24	-1.81
IPI00011252	Complement component C8 alpha chain precursor	R.QAQCGQDFQCK.E	2	3.09	0.49	-1.75
IPI00011252	Complement component C8 alpha chain precursor	R.SLKYNPVVIDFEM*QPIHEVLR.H	3	4.81	0.40	-3.62
IPI00011252	Complement component C8 alpha chain precursor	R.SLKYNPVVIDFEM*QPIHEVLR.H	4	3.24	0.20	-3.60
IPI00011261	Complement component C8 gamma chain precursor	K.YGFCEAADQFHVLDEVR.R	3	4.32	0.50	-2.90
IPI00011261	Complement component C8 gamma chain precursor	K.YGFCEAADQFHVLDEVRR.-	3	3.15	0.43	-3.73
IPI00011261	Complement component C8 gamma chain precursor	K.YGFCEAADQFHVLDEVRR.-	4	3.14	0.21	-2.73
IPI00011261	Complement component C8 gamma chain precursor	R.AEATTLHVAPQGTAM*AVSTFR.K	2	4.26	0.50	-4.11
IPI00011261	Complement component C8 gamma chain precursor	R.AEATTLHVAPQGTAM*AVSTFR.K	3	3.90	0.44	-1.52
IPI00011261	Complement component C8 gamma chain precursor	R.AGQLSVK.L	1	1.75	0.10	-2.57
IPI00011261	Complement component C8 gamma chain precursor	R.FLQEQGHR.A	1	2.31	0.08	-4.46
IPI00011261	Complement component C8 gamma chain precursor	R.FLQEQGHR.A	2	2.91	0.17	0.71
IPI00011261	Complement component C8 gamma chain precursor	R.FLQEQGHRAEATTLHVAPQGTAM*AVSTFR.K	3	5.53	0.48	-4.68
IPI00011261	Complement component C8 gamma chain precursor	R.FLQEQGHRAEATTLHVAPQGTAM*AVSTFR.K	4	4.32	0.38	-3.80
IPI00011261	Complement component C8 gamma chain precursor	R.QLYGDTGVLGR.F	2	2.54	0.35	-2.44
IPI00011261	Complement component C8 gamma chain precursor	R.RPASPISTIQPK.A	1	2.97	0.28	-3.67
IPI00011261	Complement component C8 gamma chain precursor	R.RPASPISTIQPK.A	2	2.76	0.21	-3.91
IPI00011261	Complement component C8 gamma chain precursor	R.RPASPISTIQPK.A	3	3.11	0.08	-2.59
IPI00011261	Complement component C8 gamma chain precursor	R.SLPVSDSVLSGFEQR.V	2	4.97	0.47	-3.52
IPI00011261	Complement component C8 gamma chain precursor	R.VQEAHLTEDQIFYFPK.Y	2	5.16	0.47	-2.86
IPI00011261	Complement component C8 gamma chain precursor	R.VQEAHLTEDQIFYFPK.Y	3	5.68	0.39	-3.34
IPI00011264	Complement factor H-related protein 1 precursor	K.CGPPPPIDNGDITSFPLSVYAPASSVEYQCQNLYQLEGNKR.I	3	5.17	0.49	-3.33
IPI00011264	Complement factor H-related protein 1 precursor	K.INHGILYDEEK.Y	2	3.57	0.37	-3.89
IPI00011264	Complement factor H-related protein 1 precursor	K.YKPFSQVPTGEVFYYSCEYNFVSPSK.S	3	4.07	0.27	
IPI00011264	Complement factor H-related protein 1 precursor	R.EIM*ENYNIALR.W	2	2.73	0.35	-4.22

IPI00011264	Complement factor H-related protein 1 precursor	R.LCFFPFVENGHSESSGQTHLEGDTVQIICNTGYR.L	3	5.60	0.33	
IPI00011264	Complement factor H-related protein 1 precursor	R.LQNNENNISCVER.G	2	3.88	0.44	-0.87
IPI00011264	Complement factor H-related protein 1 precursor	R.NGQWSEPPKCLHPCVISR.E	3	3.60	0.35	
IPI00011264	Complement factor H-related protein 1 precursor	R.QMSKYPSGER.V	2	2.87	0.22	
IPI00011264	Complement factor H-related protein 1 precursor	R.STDTSCVNPPTVQNAYIVSR.Q	2	5.22	0.37	
IPI00011264	Complement factor H-related protein 1 precursor	R.STDTSCVNPPTVQNAYIVSR.Q	3	3.47	0.28	
IPI00011264	Complement factor H-related protein 1 precursor	R.TGESAEFVCK.R	2	2.87	0.29	
IPI00011264	Complement factor H-related protein 1 precursor	R.TGESAEFVCKR.G	2	3.12	0.23	
IPI00011264	Complement factor H-related protein 1 precursor	R.TGESAEFVCKR.G	3	2.46	0.18	
IPI00011264	Complement factor H-related protein 1 precursor	R.TTCWDGKLEYPTCAK.R	2	4.43	0.42	
IPI00011264	Complement factor H-related protein 1 precursor	T.DTSCVNPPTVQNAYIVSR.Q	2	4.29	0.41	-4.43
IPI00011264	Complement factor H-related protein 1 precursor	T.DTSCVNPPTVQNAYIVSR.Q	3	3.75	0.42	-4.38
IPI00011302	CD59 glycoprotein precursor	K.AGLQVYNK.C	1	2.00	0.06	-4.06
IPI00011302	CD59 glycoprotein precursor	K.AGLQVYNK.C	2	3.39	0.23	-2.31
IPI00011302	CD59 glycoprotein precursor	K.FEHCNFNDVTTR.L	2	2.57	0.21	-3.59
IPI00011302	CD59 glycoprotein precursor	R.LRENELTYYCCK.K	2	4.16	0.54	-3.06
IPI00011302	CD59 glycoprotein precursor	R.LRENELTYYCCK.K	3	2.68	0.18	-1.62
IPI00011302	CD59 glycoprotein precursor	S.LQCYNCPNPTADCK.T	2	4.67	0.57	-2.66
IPI00011400	T-lymphoma invasion and metastasis-inducing protein 1	K.NFLVHKKNKKVESATRR.K	2	3.31	0.09	
IPI00011416	Delta(3,5)-Delta(2,4)-dienoyl-CoA isomerase, mitochondrial precursor	K.GDDVARISWYLRDIITRYQETFNVIER.C	3	3.58	0.22	
IPI00011454	Isoform 2 of Neutral alpha-glucosidase AB precursor	K.M*M*DYLQGSGETPQTDVR.W	2	3.86	0.34	
IPI00011454	Isoform 2 of Neutral alpha-glucosidase AB precursor	R.GLLEFEHQR.A	2	2.20	0.20	-2.35
IPI00011454	Isoform 2 of Neutral alpha-glucosidase AB precursor	R.KLVAIVDPHIK.V	3	3.56	0.16	
IPI00011454	Isoform 2 of Neutral alpha-glucosidase AB precursor	R.LDLLEDR.S	2	2.73	0.13	-2.49
IPI00011454	Isoform 2 of Neutral alpha-glucosidase AB precursor	R.LKVTEGGEPYR.L	2	2.41	0.13	-2.00
IPI00011454	Isoform 2 of Neutral alpha-glucosidase AB precursor	R.LSFQHDPETSVLVLR.K	2	2.40	0.14	-3.78
IPI00011454	Isoform 2 of Neutral alpha-glucosidase AB precursor	R.LSFQHDPETSVLVLR.K	3	3.57	0.28	-1.51
IPI00011454	Isoform 2 of Neutral alpha-glucosidase AB precursor	R.QYASLTGTQALPPLFSLGYHQSR.W	3	3.57	0.28	-1.57
IPI00011454	Isoform 2 of Neutral alpha-glucosidase AB precursor	R.SIRPGLSPYR.A	2	2.17	0.05	-2.97
IPI00011454	Isoform 2 of Neutral alpha-glucosidase AB precursor	R.YRVPDVLVADPPIAR.L	3	3.79	0.31	-2.23

IPI00011515	Protein kinase C and casein kinase substrate in neurons protein 1	R.GRLDSGQLGLYPANYVEAI.-	2	3.49	0.42	-4.41
IPI00011518	Isoform A of Beta-secretase 1 precursor	K.KVFEAAVK.S	1	1.72	0.16	-3.63
IPI00011518	Isoform A of Beta-secretase 1 precursor	R.ITILPQQYLRPVEDVATSQDDCYK.F	3	3.81	0.29	-3.72
IPI00011518	Isoform A of Beta-secretase 1 precursor	R.KGVYVPYTQGK.W	2	2.93	0.36	-2.83
IPI00011518	Isoform A of Beta-secretase 1 precursor	R.VEINGQDLK.M	2	2.53	0.07	-2.51
IPI00011564	Syndecan-4 precursor	A.ESIRETEVIDPQDLLEGR.Y	3	4.44	0.12	0.78
IPI00011578	Isoform 1 of Neuroplastin precursor	K.NGVELSATR.K	2	3.02	0.34	-1.46
IPI00011592	Cytoplasmic dynein 1 light intermediate chain 2	K.TGSPGSPGAGGVQSTAK.K	2	2.54	0.38	-2.88
IPI00011605	Cerebellin-1 precursor	K.CLVVCDSNPTSDPTGTALGISVR.S	2	6.64	0.58	-4.59
IPI00011605	Cerebellin-1 precursor	K.CLVVCDSNPTSDPTGTALGISVR.S	3	2.43	0.19	-3.57
IPI00011605	Cerebellin-1 precursor	R.AYLKLER.G	2	2.38	0.10	-3.25
IPI00011605	Cerebellin-1 precursor	R.EAASNGVLIQM*EK.G	2	3.99	0.40	-4.64
IPI00011605	Cerebellin-1 precursor	R.EAASNGVLIQM*EKGDR.A	2	3.96	0.41	-2.57
IPI00011605	Cerebellin-1 precursor	R.EAASNGVLIQM*EKGDR.A	3	2.32	0.27	-1.71
IPI00011605	Cerebellin-1 precursor	R.STFIAPR.K	1	1.69	0.18	-1.09
IPI00011605	Cerebellin-1 precursor	R.TM*IIYFDQVLVNIGNNFDSER.S	2	4.76	0.54	-3.97
IPI00011605	Cerebellin-1 precursor	R.TM*IIYFDQVLVNIGNNFDSER.S	3	6.15	0.49	-2.50
IPI00011643	Isoform 2 of Kunitz-type protease inhibitor 1 precursor	K.AWAGIDLK.V	1	2.07	0.05	-3.15
IPI00011651	Isoform 1 of Receptor-type tyrosine-protein phosphatase gamma precursor	K.ASGDPYWAYSGAYGPEHWVTSSVSCGGR.H	3	5.01	0.63	-2.31
IPI00011651	Isoform 1 of Receptor-type tyrosine-protein phosphatase gamma precursor	K.ATISHVSPDSLYLFR.V	2	4.30	0.44	-3.26
IPI00011651	Isoform 1 of Receptor-type tyrosine-protein phosphatase gamma precursor	K.ATISHVSPDSLYLFR.V	3	5.14	0.36	-3.22
IPI00011651	Isoform 1 of Receptor-type tyrosine-protein phosphatase gamma precursor	K.DDYFVSGAGLPGR.F	2	4.23	0.36	-4.64
IPI00011651	Isoform 1 of Receptor-type tyrosine-protein phosphatase gamma precursor	K.ETFLDPFVLR.D	2	3.18	0.32	-4.52
IPI00011651	Isoform 1 of Receptor-type tyrosine-protein phosphatase gamma precursor	K.ETFLDPFVLRDLLPASLGSYYR.Y	3	3.23	0.25	-3.68
IPI00011651	Isoform 1 of Receptor-type tyrosine-protein phosphatase gamma precursor	K.GVVHHEKETFLDPFVLR.D	2	4.14	0.48	-5.33
IPI00011651	Isoform 1 of Receptor-type tyrosine-protein phosphatase gamma precursor	K.GVVHHEKETFLDPFVLR.D	3	4.55	0.28	-4.17
IPI00011651	Isoform 1 of Receptor-type tyrosine-protein phosphatase gamma precursor	K.TFTKDSDKDLK.A	2	3.32	0.22	-3.96
IPI00011651	Isoform 1 of Receptor-type tyrosine-protein phosphatase gamma precursor	K.TVAILLKDDYFVSGAGLPGR.F	3	4.41	0.36	-4.49
IPI00011651	Isoform 1 of Receptor-type tyrosine-protein phosphatase gamma precursor	R.DLLPASLGSYYR.Y	2	3.78	0.48	-3.31

IPI00011651	Isoform 1 of Receptor-type tyrosine-protein phosphatase gamma precursor	R.DNSALDPIIHGLK.G	2	3.96	0.37	-3.63
IPI00011651	Isoform 1 of Receptor-type tyrosine-protein phosphatase gamma precursor	R.DNSALDPIIHGLK.G	3	2.58	0.23	-1.68
IPI00011651	Isoform 1 of Receptor-type tyrosine-protein phosphatase gamma precursor	R.HQSPIDILDQYAR.V	2	3.40	0.32	-4.73
IPI00011651	Isoform 1 of Receptor-type tyrosine-protein phosphatase gamma precursor	R.IIGAM*AIFFQVSPR.D	2	4.19	0.48	-4.82
IPI00011651	Isoform 1 of Receptor-type tyrosine-protein phosphatase gamma precursor	R.IIGAM*AIFFQVSPR.D	3	3.13	0.20	-1.73
IPI00011651	Isoform 1 of Receptor-type tyrosine-protein phosphatase gamma precursor	R.RFPVEM*QIFFYNPDDFDSFQTAISENR.I	3	5.78	0.47	-2.09
IPI00011651	Isoform 1 of Receptor-type tyrosine-protein phosphatase gamma precursor	R.RFPVEMQIFFYNPDDFDSFQTAISENR.I	3	5.40	0.44	-7.24
IPI00011651	Isoform 1 of Receptor-type tyrosine-protein phosphatase gamma precursor	R.RPVPISYHQLEAFYSIFTTEQQDHVK.S	5	3.38	0.32	-3.49
IPI00011651	Isoform 1 of Receptor-type tyrosine-protein phosphatase gamma precursor	R.YTGSLTTPPCSEIVEWIVFR.R	2	3.53	0.50	-4.68
IPI00011651	Isoform 1 of Receptor-type tyrosine-protein phosphatase gamma precursor	R.YTGSLTTPPCSEIVEWIVFR.R	3	4.64	0.44	-5.19
IPI00011654	Tubulin beta chain	K.GHYTEGAELVDSVLDVVR.K	2	2.94	0.28	
IPI00011654	Tubulin beta chain	K.GHYTEGAELVDSVLDVVR.K	3	3.28	0.30	-3.01
IPI00011654	Tubulin beta chain	K.GHYTEGAELVDSVLDVVRK.E	3	3.51	0.45	-3.20
IPI00011654	Tubulin beta chain	K.MAVTFIGNSTAIQELFK.R	2	5.38	0.44	-2.16
IPI00011654	Tubulin beta chain	K.NM*M*AACDPR.H	2	1.73	0.12	0.11
IPI00011654	Tubulin beta chain	K.VSDTVVEPYNATLSVHQLVENTDETYCIDNEALYDICFR.T	3	4.91	0.43	-2.95
IPI00011654	Tubulin beta chain	K.VSDTVVEPYNATLSVHQLVENTDETYCIDNEALYDICFR.T	4	4.05	0.34	-2.05
IPI00011654	Tubulin beta chain	R.ISEQFTAM*FR.R	2	2.17	0.13	-2.65
IPI00011654	Tubulin beta chain	R.ISEQFTAMFR.R	2	2.87	0.27	-0.81
IPI00011654	Tubulin beta chain	R.LHFFM*PGFAPLTSR.G	3	3.20	0.06	-3.00
IPI00011654	Tubulin beta chain	R.LHFFMPGFAPLTSR.G	3	2.62	0.11	-1.73
IPI00011662	Kunitz-type protease inhibitor 2 precursor	K.CATVTENATGDLATSR.N	2	4.93	0.42	-3.34
IPI00011662	Kunitz-type protease inhibitor 2 precursor	R.NAADSSVPSAPR.R	1	2.05	0.12	-4.38
IPI00011662	Kunitz-type protease inhibitor 2 precursor	R.NAADSSVPSAPR.R	2	3.95	0.43	-3.61
IPI00011662	Kunitz-type protease inhibitor 2 precursor	R.WYFDVER.N	2	1.65	0.05	-1.64
IPI00011730	EMILIN-3 precursor	R.GPLTPPLDEILSK.V	2	1.90	0.19	1.54
IPI00011730	EMILIN-3 precursor	R.LATLAGELSHDSASPGR.S	2	2.39	0.23	-1.03
IPI00011730	EMILIN-3 precursor	R.LGLLAAGLDSLPTEPLRPR.E	3	2.35	0.07	-1.16
IPI00011730	EMILIN-3 precursor	R.RLGLLAAGLDSLPTEPLRPR.E	3	3.99	0.37	-2.13
IPI00011730	EMILIN-3 precursor	R.RLGLLAAGLDSLPTEPLRPR.E	4	2.75	0.24	-0.75
IPI00011730	EMILIN-3 precursor	R.TLAQHTQDIAR.L	2	2.65	0.14	-2.96

IPI00011732	Isoform 1 of GDNF family receptor alpha-2 precursor	R.DFTENPCLR.N	2	2.68	0.21	-2.74
IPI00011732	Isoform 1 of GDNF family receptor alpha-2 precursor	R.LASIFSGTGADPVVSAK.S	2	5.04	0.55	-2.89
IPI00011732	Isoform 1 of GDNF family receptor alpha-2 precursor	R.LASIFSGTGADPVVSAK.S	3	2.37	0.28	-2.69
IPI00011732	Isoform 1 of GDNF family receptor alpha-2 precursor	R.QFFDRVPSEYTYR.M	2	1.37	0.07	-3.50
IPI00011732	Isoform 1 of GDNF family receptor alpha-2 precursor	R.SSYISICNR.E	2	2.99	0.20	-0.60
IPI00011732	Isoform 1 of GDNF family receptor alpha-2 precursor	R.VPSEYTYR.M	2	2.33	0.17	-1.99
IPI00011732	Isoform 1 of GDNF family receptor alpha-2 precursor	S.IFSGTGADPVVSAK.S	2	3.77	0.40	-3.63
IPI00011781	Ankyrin repeat-containing protein C20orf86 precursor	R.TANSEHFEGEKWKHWTSQRAFVALYVASHR.G	3	2.98	0.17	-0.86
IPI00011865	Isoform 2 of Platelet-derived growth factor D precursor	K.IAEFDTVEDLLK.Y	2	1.67	0.16	-1.88
IPI00011865	Isoform 2 of Platelet-derived growth factor D precursor	K.LANVVFFPR.C	2	3.73	0.27	-0.72
IPI00011899	BMP and activin membrane-bound inhibitor homolog precursor	K.SELSACFSR.L	2	2.92	0.28	-0.58
IPI00011899	BMP and activin membrane-bound inhibitor homolog precursor	R.GLHDVLSPPRGEASGQGNR.Y	3	2.48	0.39	-2.71
IPI00011899	BMP and activin membrane-bound inhibitor homolog precursor	R.LLDPQNSNSPLTHGCLDSLASTTDICQAK.Q	3	5.27	0.47	-3.51
IPI00011994	Ectonucleotide pyrophosphatase/phosphodiesterase family member 5 precursor	K.FWEEATPIWITNQR.A	2	4.87	0.51	-5.32
IPI00011994	Ectonucleotide pyrophosphatase/phosphodiesterase family member 5 precursor	K.FWEEATPIWITNQR.A	3	3.70	0.24	-2.84
IPI00011994	Ectonucleotide pyrophosphatase/phosphodiesterase family member 5 precursor	K.LGYLIQM*LK.K	2	3.10	0.19	-2.03
IPI00011994	Ectonucleotide pyrophosphatase/phosphodiesterase family member 5 precursor	K.LGYLIQM*LKK.A	3	1.72	0.13	-3.36
IPI00011994	Ectonucleotide pyrophosphatase/phosphodiesterase family member 5 precursor	K.SFSLDHM*NIYDSK.F	2	3.40	0.30	-3.05

IPI00011994	Ectonucleotide pyrophosphatase/phosphodiesterase family member 5 precursor	K.VLLVSFDGFR.W	2	3.59	0.40	-3.99
IPI00011994	Ectonucleotide pyrophosphatase/phosphodiesterase family member 5 precursor	R.LIELDQYLDKDHYTLIDQSPVAAILPK.E	3	6.09	0.50	-3.15
IPI00011994	Ectonucleotide pyrophosphatase/phosphodiesterase family member 5 precursor	R.LIELDQYLDKDHYTLIDQSPVAAILPK.E	4	3.46	0.40	-4.09
IPI00012007	Adenosylhomocysteinase	K.KLDEAVAEAHLGK.L	3	2.52	0.11	-2.33
IPI00012007	Adenosylhomocysteinase	R.ATDVM*IAGK.V	2	2.47	0.23	0.10
IPI00012009	Isoform 1 of Granulocyte-macrophage colony-stimulating factor receptor alpha chain precursor	K.LSYLDFQYQLDVHRK.N	4	3.19	0.23	-3.26
IPI00012009	Isoform 1 of Granulocyte-macrophage colony-stimulating factor receptor alpha chain precursor	R.EIGIQFFDSLLDTK.K	2	4.78	0.54	-4.24
IPI00012011	Cofilin-1	K.LGGSAVISLEGKPL.-	2	3.17	0.31	-3.48
IPI00012011	Cofilin-1	K.NIILEEGKEILVGDVGQTVDDPYATFVK.M	3	5.14	0.29	-5.93
IPI00012011	Cofilin-1	R.YALYDATYETK.E	2	3.27	0.30	-1.92
IPI00012044	Isoform 1 of Pro-neuregulin-3, membrane-bound isoform precursor	R.DKDLAYCLNDGECFVIETLTGSHK.H	4	3.01	0.18	-4.23
IPI00012048	Nucleoside diphosphate kinase A	R.GDFCIQVGR.N	2	2.05	0.12	-3.65
IPI00012048	Nucleoside diphosphate kinase A	R.GLVGEIIK.R	2	2.03	0.15	-2.61
IPI00012048	Nucleoside diphosphate kinase A	R.VM*LGETNPADSKPGTIR.G	2	4.01	0.37	-2.88
IPI00012048	Nucleoside diphosphate kinase A	R.VM*LGETNPADSKPGTIR.G	3	3.19	0.22	-3.78
IPI00012058	Brain-derived neurotrophic factor precursor	K.VRPNEENNKDADLYTSR.V	3	3.79	0.22	-2.61
IPI00012058	Brain-derived neurotrophic factor precursor	R.GQGGLAYPGVR.T	2	2.82	0.29	0.23
IPI00012058	Brain-derived neurotrophic factor precursor	R.THGTLESVNGPK.A	2	3.45	0.42	-3.54
IPI00012058	Brain-derived neurotrophic factor precursor	T.SLADTFEHVIEELLDEDQK.V	3	4.09	0.25	-2.88
IPI00012075	C-type natriuretic peptide precursor	K.APGGGGANLKGDR.S	2	3.38	0.36	-2.46
IPI00012075	C-type natriuretic peptide precursor	K.KGDKAPGGGGANLKGDR.S	2	3.53	0.41	-3.79
IPI00012075	C-type natriuretic peptide precursor	K.KGDKAPGGGGANLKGDR.S	3	3.77	0.35	-2.64
IPI00012075	C-type natriuretic peptide precursor	K.KGDKAPGGGGANLKGDRS.R	2	4.12	0.49	-4.77
IPI00012075	C-type natriuretic peptide precursor	P.AEELAEPQAAGGGQK.K	2	4.35	0.45	-3.55
IPI00012075	C-type natriuretic peptide precursor	R.TPPAEELAEPQAAGGGQK.K	2	5.95	0.63	-4.19
IPI00012075	C-type natriuretic peptide precursor	R.TPPAEELAEPQAAGGGQK.K	3	3.43	0.23	-3.03
IPI00012102	N-acetylglucosamine-6-sulfatase precursor	K.AFQNVFAPR.N	2	2.79	0.24	-2.98
IPI00012102	N-acetylglucosamine-6-sulfatase precursor	K.IQEPNTFPAILR.S	2	3.26	0.29	-4.10
IPI00012102	N-acetylglucosamine-6-sulfatase precursor	K.YLNEYGAPDAGGLEHVPLGWSYWYALEK.N	3	4.56	0.26	-3.66
IPI00012102	N-acetylglucosamine-6-sulfatase precursor	R.ASILTGK.Y	1	1.64	0.10	-2.08
IPI00012102	N-acetylglucosamine-6-sulfatase precursor	R.LM*M*LQSCSGPTCR.T	2	4.15	0.46	-3.14
IPI00012102	N-acetylglucosamine-6-sulfatase precursor	R.QLYEFDIK.V	2	1.99	0.06	-2.37
IPI00012102	N-acetylglucosamine-6-sulfatase precursor	R.QLYEFDIKVPLLVR.G	2	3.05	0.40	-2.41

IPI00012102	N-acetylglucosamine-6-sulfatase precursor	R.QLYEFDIKVPLLVR.G	3	2.34	0.11	-1.51
IPI00012102	N-acetylglucosamine-6-sulfatase precursor	R.RPNVVLLLTDDQDEVLGGM*TPLKK.T	3	5.07	0.52	-4.89
IPI00012102	N-acetylglucosamine-6-sulfatase precursor	R.SM*CGYQTFFAGK.Y	2	4.06	0.52	-3.92
IPI00012102	N-acetylglucosamine-6-sulfatase precursor	R.TPGVFDPGYR.F	2	2.51	0.22	-1.09
IPI00012102	N-acetylglucosamine-6-sulfatase precursor	R.WQTLLSVDDLVEK.L	2	4.10	0.42	-3.62
IPI00012269	Multimerin-1 precursor	K.FLQSFAR.K	2	2.21	0.11	-2.33
IPI00012269	Multimerin-1 precursor	K.LKEVHEQLLSTEQVSDQK.N	3	3.90	0.33	-3.78
IPI00012269	Multimerin-1 precursor	R.KSNEQATSLNTVGGTGGIGGVGGTGGVGNR.A	3	6.60	0.48	-2.67
IPI00012283	Isoform 1 of Semaphorin-3B precursor	K.TFGTFSSTKDFPDDVIQFAR.N	3	4.14	0.45	-2.88
IPI00012283	Isoform 1 of Semaphorin-3B precursor	K.VFWIPESENPDDDKIYFFFR.E	3	2.85	0.20	-1.98
IPI00012283	Isoform 1 of Semaphorin-3B precursor	R.AFLGPFAHK.E	2	1.64	0.06	-1.83
IPI00012283	Isoform 1 of Semaphorin-3B precursor	R.ETAVEAAPALGR.L	2	3.41	0.37	-2.81
IPI00012283	Isoform 1 of Semaphorin-3B precursor	R.LFVGAENHVASLNLDNISK.R	3	3.67	0.27	-1.55
IPI00012283	Isoform 1 of Semaphorin-3B precursor	R.LVCSVPGVEGDTHFDQLQDVFLLSSR.D	3	3.94	0.34	-3.13
IPI00012283	Isoform 1 of Semaphorin-3B precursor	R.SAVAQIALHR.C	2	2.29	0.13	-2.36
IPI00012303	Selenium-binding protein 1	K.GGLKLNPNFLVDFGKEPLGPALAHELR.Y	4	4.11	0.45	-2.28
IPI00012303	Selenium-binding protein 1	K.GGPVQVLEDEELKSQPEPLVVK.G	3	2.92	0.19	-2.75
IPI00012303	Selenium-binding protein 1	K.LNPNFLVDFGKEPLGPALAHELR.Y	3	4.08	0.47	-4.12
IPI00012303	Selenium-binding protein 1	K.QFYPDLIR.E	2	1.74	0.12	-1.05
IPI00012303	Selenium-binding protein 1	K.RVAGGPQM*IQLSLDGK.R	3	3.95	0.28	-2.55
IPI00012303	Selenium-binding protein 1	R.DGFNPADVEAGLYGSHLYVWDWQR.H	3	3.63	0.45	-3.88
IPI00012303	Selenium-binding protein 1	R.FLHNPDAAQGFVGCALSSTIQR.F	3	2.80	0.05	-3.46
IPI00012303	Selenium-binding protein 1	R.HEIVQTLSLK.D	2	2.29	0.12	-1.26
IPI00012303	Selenium-binding protein 1	R.HEIVQTLSLKDGLIPLEIR.F	3	4.84	0.50	-4.81
IPI00012303	Selenium-binding protein 1	R.IYVVDVGSEPR.A	2	3.90	0.38	-2.51
IPI00012303	Selenium-binding protein 1	R.LTGQLFLGGSIVK.G	2	3.61	0.36	-2.43
IPI00012303	Selenium-binding protein 1	R.NTGTEAPDYLATVDVDPK.S	2	4.98	0.50	-4.38
IPI00012303	Selenium-binding protein 1	R.QYDISDPQRPR.L	2	1.20	0.15	-1.92
IPI00012303	Selenium-binding protein 1	R.TKLVLPSLISSR.I	2	2.75	0.24	-1.82
IPI00012315	Nucleoside diphosphate kinase 3	K.LVQASEELLREHYAELR.E	4	1.84	0.18	-2.14
IPI00012315	Nucleoside diphosphate kinase 3	R.ALIGATNPADAPPGTIR.G	2	3.42	0.25	-3.78
IPI00012386	Cochlin precursor	G.AAPIAITCFTR.G	2	3.33	0.36	-2.56
IPI00012386	Cochlin precursor	K.ADVLCPGGCPLEEFSVYGNIVYASVSSICGAAVHR.G	3	4.13	0.42	-3.31
IPI00012386	Cochlin precursor	R.GVISNSGGPVR.V	1	1.90	0.23	-2.29
IPI00012386	Cochlin precursor	R.GVISNSGGPVR.V	2	3.42	0.21	-1.20
IPI00012386	Cochlin precursor	S.EGAAPIAITCFTR.G	1	3.37	0.40	-3.71
IPI00012386	Cochlin precursor	S.EGAAPIAITCFTR.G	2	4.22	0.47	-3.07
IPI00012391	Isoform Long of Adenomatous polyposis coli protein	K.RSSNDSLNSVSSSDGYGKR.G	2	1.89	0.10	-0.02
IPI00012440	Plasma alpha-L-fucosidase precursor	K.AILGATEVK.L	1	1.74	0.19	-2.75
IPI00012440	Plasma alpha-L-fucosidase precursor	K.AILGATEVK.L	2	2.46	0.15	-1.80
IPI00012440	Plasma alpha-L-fucosidase precursor	K.DNYPPSFKYEDFGPLFTAK.F	2	2.97	0.37	-3.32

IPI00012440	Plasma alpha-L-fucosidase precursor	K.DNYPPSFKYEDFGPLFTAK.F	3	2.43	0.12	-3.03
IPI00012440	Plasma alpha-L-fucosidase precursor	K.FFNANQWADIFQASGAK.Y	2	5.51	0.54	-3.46
IPI00012440	Plasma alpha-L-fucosidase precursor	K.FFNANQWADIFQASGAK.Y	3	4.69	0.35	-3.36
IPI00012440	Plasma alpha-L-fucosidase precursor	K.LVYAIFLK.W	1	1.72	0.07	-2.15
IPI00012440	Plasma alpha-L-fucosidase precursor	K.LVYAIFLK.W	2	2.77	0.27	-1.87
IPI00012440	Plasma alpha-L-fucosidase precursor	K.RDIVKELEVAIR.N	3	3.90	0.20	-2.63
IPI00012440	Plasma alpha-L-fucosidase precursor	K.VNGEAIYETYTWR.S	2	4.37	0.48	-2.97
IPI00012440	Plasma alpha-L-fucosidase precursor	K.YEDFGPLFTAK.F	2	3.92	0.45	-3.18
IPI00012440	Plasma alpha-L-fucosidase precursor	K.YIVLTSK.H	1	2.18	0.21	-2.26
IPI00012440	Plasma alpha-L-fucosidase precursor	K.YIVLTSK.H	2	2.19	0.13	-3.49
IPI00012440	Plasma alpha-L-fucosidase precursor	R.DIVKELEVAIR.N	2	3.51	0.34	-1.98
IPI00012440	Plasma alpha-L-fucosidase precursor	R.EAGISDYLTIEELVK.Q	2	4.29	0.47	-3.79
IPI00012440	Plasma alpha-L-fucosidase precursor	R.FDPTWESLDAR.Q	2	3.19	0.32	-1.51
IPI00012440	Plasma alpha-L-fucosidase precursor	R.QLPAWFDQAK.F	2	2.17	0.17	-2.20
IPI00012441	Isoform 1 of Synaptojanin-1	K.EGEHMLSKAFQSHLKASEHAADIQMVNFDYHQMVKGGKAEK.L	4	3.00	0.11	-5.61
IPI00012503	Isoform Sap-mu-0 of Proactivator polypeptide precursor	D.NGDVCQDCIQM*VTDIQTAVR.T	2	5.50	0.45	-3.98
IPI00012503	Isoform Sap-mu-0 of Proactivator polypeptide precursor	D.NGDVCQDCIQM*VTDIQTAVR.T	3	4.10	0.37	-3.48
IPI00012503	Isoform Sap-mu-0 of Proactivator polypeptide precursor	D.VYCEVCEFLVK.E	1	3.24	0.27	-2.50
IPI00012503	Isoform Sap-mu-0 of Proactivator polypeptide precursor	D.VYCEVCEFLVK.E	2	4.44	0.46	-5.17
IPI00012503	Isoform Sap-mu-0 of Proactivator polypeptide precursor	E.IVDSYLPVILDIIK.G	2	4.12	0.40	-4.24
IPI00012503	Isoform Sap-mu-0 of Proactivator polypeptide precursor	K.DGGFCEVCK.K	2	2.45	0.35	-0.85
IPI00012503	Isoform Sap-mu-0 of Proactivator polypeptide precursor	K.DNGDVCQDCIQM*VTDIQTAVR.T	2	4.67	0.41	
IPI00012503	Isoform Sap-mu-0 of Proactivator polypeptide precursor	K.DNGDVCQDCIQM*VTDIQTAVR.T	3	5.65	0.35	
IPI00012503	Isoform Sap-mu-0 of Proactivator polypeptide precursor	K.DVVTAAGDM*LK.D	2	2.65	0.21	-1.81
IPI00012503	Isoform Sap-mu-0 of Proactivator polypeptide precursor	K.EICALVGFCDEVK.E	2	3.49	0.42	-2.51
IPI00012503	Isoform Sap-mu-0 of Proactivator polypeptide precursor	K.EICALVGFCDEVKEM*PM*QTLVPAK.V	3	2.64	0.26	2.54
IPI00012503	Isoform Sap-mu-0 of Proactivator polypeptide precursor	K.EILDAFDK.M	1	2.32	0.25	-2.23
IPI00012503	Isoform Sap-mu-0 of Proactivator polypeptide precursor	K.EILDAFDK.M	2	2.37	0.07	-1.75

IPI00012503	Isoform Sap-mu-0 of Proactivator polypeptide precursor	K.EIVDSYLPVILDIIK.G	1	3.03	0.41	-3.92
IPI00012503	Isoform Sap-mu-0 of Proactivator polypeptide precursor	K.EIVDSYLPVILDIIK.G	2	5.50	0.49	-5.55
IPI00012503	Isoform Sap-mu-0 of Proactivator polypeptide precursor	K.EIVDSYLPVILDIIK.G	3	5.22	0.45	-4.52
IPI00012503	Isoform Sap-mu-0 of Proactivator polypeptide precursor	K.EIVDSYLPVILDIIKGEM*SRPGEVCSALNLC.E	3	5.00	0.51	-4.08
IPI00012503	Isoform Sap-mu-0 of Proactivator polypeptide precursor	K.EIVDSYLPVILDIIKGEM*SRPGEVCSALNLCES.L	3	4.34	0.34	-4.74
IPI00012503	Isoform Sap-mu-0 of Proactivator polypeptide precursor	K.EM*PM*QTLVPAK.V	2	2.19	0.25	-3.75
IPI00012503	Isoform Sap-mu-0 of Proactivator polypeptide precursor	K.GCSFLPDPYQK.Q	1	2.33	0.25	-2.66
IPI00012503	Isoform Sap-mu-0 of Proactivator polypeptide precursor	K.GCSFLPDPYQK.Q	2	3.28	0.29	-3.98
IPI00012503	Isoform Sap-mu-0 of Proactivator polypeptide precursor	K.HEVPAKSDVYCEVCEFLVK.E	4	3.32	0.22	-4.14
IPI00012503	Isoform Sap-mu-0 of Proactivator polypeptide precursor	K.KLVGYLDR.N	1	2.44	0.26	-3.56
IPI00012503	Isoform Sap-mu-0 of Proactivator polypeptide precursor	K.KLVGYLDR.N	2	3.13	0.24	-1.63
IPI00012503	Isoform Sap-mu-0 of Proactivator polypeptide precursor	K.LVGYLDR.N	1	2.05	0.08	-2.18
IPI00012503	Isoform Sap-mu-0 of Proactivator polypeptide precursor	K.LVGYLDR.N	2	2.62	0.26	-3.76
IPI00012503	Isoform Sap-mu-0 of Proactivator polypeptide precursor	K.LVGYLDRNLEK.N	2	3.01	0.09	-3.01
IPI00012503	Isoform Sap-mu-0 of Proactivator polypeptide precursor	K.NVIPALELVEPIK.K	2	2.47	0.22	-3.54
IPI00012503	Isoform Sap-mu-0 of Proactivator polypeptide precursor	K.NVIPALELVEPIKK.H	2	2.49	0.34	-1.75
IPI00012503	Isoform Sap-mu-0 of Proactivator polypeptide precursor	K.QCDQFVAEYEPVLIEILVEVM*DPSFVCLK.I	3	4.20	0.41	-4.30
IPI00012503	Isoform Sap-mu-0 of Proactivator polypeptide precursor	K.QEILAALEK.G	1	2.83	0.15	-3.05
IPI00012503	Isoform Sap-mu-0 of Proactivator polypeptide precursor	K.QEILAALEK.G	2	2.30	0.13	-2.95
IPI00012503	Isoform Sap-mu-0 of Proactivator polypeptide precursor	K.QLESNKIPELDM*TEVVAPFM*ANIPLLLYPQDGPR.S	3	5.22	0.41	-3.63
IPI00012503	Isoform Sap-mu-0 of Proactivator polypeptide precursor	K.QLESNKIPELDM*TEVVAPFM*ANIPLLLYPQDGPR.S	4	4.73	0.31	-4.23

IPI00012503	Isoform Sap-mu-0 of Proactivator polypeptide precursor	K.SDVYCEVCEFLVK.E	2	4.50	0.50	-3.64
IPI00012503	Isoform Sap-mu-0 of Proactivator polypeptide precursor	K.SDVYCEVCEFLVK.E	3	2.76	0.27	-1.22
IPI00012503	Isoform Sap-mu-0 of Proactivator polypeptide precursor	K.SLPCDICKDVVTAAGDM*LK.D	3	2.72	0.18	-1.71
IPI00012503	Isoform Sap-mu-0 of Proactivator polypeptide precursor	N.GDVCQDCIQM*VTDIQTAVR.T	2	6.29	0.54	-4.29
IPI00012503	Isoform Sap-mu-0 of Proactivator polypeptide precursor	N.GDVCQDCIQM*VTDIQTAVR.T	3	5.48	0.50	-3.55
IPI00012503	Isoform Sap-mu-0 of Proactivator polypeptide precursor	R.LGPGM*ADICK.N	2	3.08	0.18	-1.82
IPI00012503	Isoform Sap-mu-0 of Proactivator polypeptide precursor	R.LPALTVHVTQPK.D	2	3.89	0.50	-4.14
IPI00012503	Isoform Sap-mu-0 of Proactivator polypeptide precursor	R.LPALTVHVTQPK.D	3	2.97	0.32	-3.58
IPI00012503	Isoform Sap-mu-0 of Proactivator polypeptide precursor	S.DVYCEVCEFLVK.E	2	4.32	0.34	-5.01
IPI00012510	EMILIN-2 precursor	K.ATDNEPSQFSEPR.K	2	2.83	0.16	-5.37
IPI00012510	EMILIN-2 precursor	R.TRAPGLSSQHPKPDTTVSGDTETGQSPGVFNTK.E	3	4.39	0.42	-4.03
IPI00012510	EMILIN-2 precursor	R.TVLDLQSSLAGVSENLK.H	2	5.10	0.48	-1.91
IPI00012545	Isoform TGN51 of Trans-Golgi network integral membrane protein 2 precursor	K.DGSNKSGAEEQGPIDGPSK.S	2	4.31	0.47	-2.87
IPI00012545	Isoform TGN51 of Trans-Golgi network integral membrane protein 2 precursor	K.DGSNKSGAEEQGPIDGPSK.S	3	4.19	0.32	-1.39
IPI00012545	Isoform TGN51 of Trans-Golgi network integral membrane protein 2 precursor	K.DGSSKSGAEDQTPKDVPNK.S	3	2.62	0.30	-1.11
IPI00012545	Isoform TGN51 of Trans-Golgi network integral membrane protein 2 precursor	K.DHSKPISNPSDNKELPK.A	3	4.47	0.40	-2.34
IPI00012545	Isoform TGN51 of Trans-Golgi network integral membrane protein 2 precursor	K.DHSKPISNPSDNKELPKADTNQLADK.G	3	3.24	0.29	-3.14
IPI00012545	Isoform TGN51 of Trans-Golgi network integral membrane protein 2 precursor	K.DHSKPISNPSDNKELPKADTNQLADK.G	4	3.23	0.24	-2.91
IPI00012545	Isoform TGN51 of Trans-Golgi network integral membrane protein 2 precursor	K.DSPNKVVPEQPSRK.D	2	1.96	0.13	-3.30
IPI00012545	Isoform TGN51 of Trans-Golgi network integral membrane protein 2 precursor	K.DSPNKVVPEQPSRK.D	3	2.87	0.25	-1.41
IPI00012545	Isoform TGN51 of Trans-Golgi network integral membrane protein 2 precursor	K.DSTGKSGAEAQTPEDSPNR.S	2	3.65	0.52	-2.12
IPI00012545	Isoform TGN51 of Trans-Golgi network integral membrane protein 2 precursor	K.DSTGKSGAEAQTPEDSPNR.S	3	2.81	0.14	-2.25

IPI00012545	Isoform TGN51 of Trans-Golgi network integral membrane protein 2 precursor	K.DVPNKSGADGQTPK.D	2	3.07	0.37	-3.60
IPI00012545	Isoform TGN51 of Trans-Golgi network integral membrane protein 2 precursor	K.M*SGSASSENREGTLSD.S	2	4.25	0.53	-3.63
IPI00012545	Isoform TGN51 of Trans-Golgi network integral membrane protein 2 precursor	K.SGAEAQTPEDSPNR.S	2	4.23	0.45	-3.23
IPI00012545	Isoform TGN51 of Trans-Golgi network integral membrane protein 2 precursor	K.SGAEAQTPEDSPNRSGAEAK.T	2	4.25	0.46	-2.78
IPI00012545	Isoform TGN51 of Trans-Golgi network integral membrane protein 2 precursor	K.SGAEDQTPKDVPNK.S	2	4.18	0.38	-2.34
IPI00012545	Isoform TGN51 of Trans-Golgi network integral membrane protein 2 precursor	K.SGAEDQTPKDVPNK.S	3	3.00	0.27	-3.59
IPI00012545	Isoform TGN51 of Trans-Golgi network integral membrane protein 2 precursor	K.SGAEDQTPKDVPNKSGAEK.Q	2	5.15	0.49	-4.42
IPI00012545	Isoform TGN51 of Trans-Golgi network integral membrane protein 2 precursor	K.SGAEDQTPKDVPNKSGAEK.Q	3	4.36	0.42	-3.34
IPI00012545	Isoform TGN51 of Trans-Golgi network integral membrane protein 2 precursor	K.SGAEDQTPKDVPNKSGAEKQTPK.D	4	3.11	0.12	0.19
IPI00012545	Isoform TGN51 of Trans-Golgi network integral membrane protein 2 precursor	K.SGAEEQGPIDGPSK.S	2	4.78	0.44	-3.19
IPI00012545	Isoform TGN51 of Trans-Golgi network integral membrane protein 2 precursor	K.SGAEEQTSKDSPNKVVPEQPSR.K	4	3.09	0.14	-2.67
IPI00012545	Isoform TGN51 of Trans-Golgi network integral membrane protein 2 precursor	K.SGAEEQTSKDSPNKVVPEQPSRK.D	3	4.54	0.41	-3.63
IPI00012545	Isoform TGN51 of Trans-Golgi network integral membrane protein 2 precursor	K.SGAEEQTSKDSPNKVVPEQPSRK.D	4	3.40	0.26	-2.81
IPI00012545	Isoform TGN51 of Trans-Golgi network integral membrane protein 2 precursor	K.SGSEAQTTKDVPNK.S	2	3.55	0.35	-4.09
IPI00012545	Isoform TGN51 of Trans-Golgi network integral membrane protein 2 precursor	K.SGSEAQTTKDVPNKSGADGQTPK.D	3	4.50	0.38	-3.30
IPI00012545	Isoform TGN51 of Trans-Golgi network integral membrane protein 2 precursor	K.SGSEAQTTKDVPNKSGADGQTPK.D	4	2.86	0.15	-3.06
IPI00012545	Isoform TGN51 of Trans-Golgi network integral membrane protein 2 precursor	K.SHPELQTPK.D	1	2.42	0.11	-3.49
IPI00012545	Isoform TGN51 of Trans-Golgi network integral membrane protein 2 precursor	K.SHPELQTPK.D	2	2.08	0.31	-1.72
IPI00012545	Isoform TGN51 of Trans-Golgi network integral membrane protein 2 precursor	K.SHPEPQTPK.D	1	1.42	0.13	-2.63
IPI00012545	Isoform TGN51 of Trans-Golgi network integral membrane protein 2 precursor	K.SHPEPQTPK.D	2	1.94	0.18	-1.82
IPI00012545	Isoform TGN51 of Trans-Golgi network integral membrane protein 2 precursor	K.SHPEPQTPKDSPSK.S	2	2.96	0.42	-4.33

IPI00012545	Isoform TGN51 of Trans-Golgi network integral membrane protein 2 precursor	K.SSAEAQTPEDTPNK.S	2	4.48	0.37	-4.38
IPI00012545	Isoform TGN51 of Trans-Golgi network integral membrane protein 2 precursor	K.SSAEAQTPEDTPNKSGAEAK.T	2	5.08	0.47	-4.32
IPI00012545	Isoform TGN51 of Trans-Golgi network integral membrane protein 2 precursor	K.SSAEAQTPEDTPNKSGAEAK.T	3	3.63	0.21	-3.41
IPI00012545	Isoform TGN51 of Trans-Golgi network integral membrane protein 2 precursor	K.SSEPTEDVEPK.E	2	2.41	0.24	-2.52
IPI00012545	Isoform TGN51 of Trans-Golgi network integral membrane protein 2 precursor	K.TESGEETDLISPPQEEVK.S	2	4.31	0.37	-3.46
IPI00012545	Isoform TGN51 of Trans-Golgi network integral membrane protein 2 precursor	K.VVPEQPSR.K	2	1.48	0.18	-3.08
IPI00012585	Beta-hexosaminidase beta chain precursor	K.DSAYPEELSR.V	1	1.89	0.08	-3.15
IPI00012585	Beta-hexosaminidase beta chain precursor	K.DVRDM*DDAYDR.L	3	2.76	0.32	-3.69
IPI00012585	Beta-hexosaminidase beta chain precursor	K.EISEVFPDQFIHLGGDEVEFK.C	2	4.15	0.51	-3.57
IPI00012585	Beta-hexosaminidase beta chain precursor	K.EISEVFPDQFIHLGGDEVEFK.C	3	2.59	0.32	-6.07
IPI00012585	Beta-hexosaminidase beta chain precursor	K.EPVAVLK.A	1	1.79	0.06	-1.56
IPI00012585	Beta-hexosaminidase beta chain precursor	K.FNVLHWHIVDDQSFPYQSITFPELSNK.G	3	5.20	0.41	-3.48
IPI00012585	Beta-hexosaminidase beta chain precursor	K.FNVLHWHIVDDQSFPYQSITFPELSNK.G	4	3.62	0.28	-6.03
IPI00012585	Beta-hexosaminidase beta chain precursor	K.GSYSLSHVYTPNDVR.M	2	4.83	0.50	-4.30
IPI00012585	Beta-hexosaminidase beta chain precursor	K.GSYSLSHVYTPNDVR.M	3	3.04	0.22	-3.04
IPI00012585	Beta-hexosaminidase beta chain precursor	K.KLESFYIQK.V	1	3.16	0.10	-2.97
IPI00012585	Beta-hexosaminidase beta chain precursor	K.KLESFYIQK.V	2	3.17	0.18	-2.94
IPI00012585	Beta-hexosaminidase beta chain precursor	K.LAPGTIVEVWK.D	2	2.38	0.34	-2.66
IPI00012585	Beta-hexosaminidase beta chain precursor	K.LAPGTIVEVWKDSAYPEELSR.V	2	4.82	0.55	-3.08
IPI00012585	Beta-hexosaminidase beta chain precursor	K.LAPGTIVEVWKDSAYPEELSR.V	3	3.53	0.28	-4.04
IPI00012585	Beta-hexosaminidase beta chain precursor	K.PGPALWPLPLSVK.M	2	3.62	0.44	-3.45
IPI00012585	Beta-hexosaminidase beta chain precursor	K.TLDAM*AFNK.F	1	1.35	0.14	-3.64
IPI00012585	Beta-hexosaminidase beta chain precursor	K.VLDIIATINK.G	2	2.45	0.23	-1.90
IPI00012585	Beta-hexosaminidase beta chain precursor	K.YYKVEPLDFGGTQK.Q	2	4.00	0.34	-4.12
IPI00012585	Beta-hexosaminidase beta chain precursor	R.DM*DDAYDR.L	2	2.77	0.25	-2.69
IPI00012585	Beta-hexosaminidase beta chain precursor	R.GIAAQPLYAGYCNHENM*.-	2	4.17	0.47	-4.13
IPI00012585	Beta-hexosaminidase beta chain precursor	R.KYYKVEPLDFGGTQK.Q	2	4.41	0.48	-4.26
IPI00012585	Beta-hexosaminidase beta chain precursor	R.M*VIEYAR.L	2	2.33	0.15	-2.88
IPI00012792	Cadherin-5 precursor	K.ELDSTGTPTGK.E	2	2.05	0.30	-2.15
IPI00012792	Cadherin-5 precursor	R.YM*SPPAGNR.A	2	2.27	0.26	-0.04
IPI00012877	Isoform 1 of Interferon-alpha/beta receptor alpha chain precursor	K.LNVYEEIK.L	2	2.61	0.25	-1.56
IPI00012887	Cathepsin L1 precursor	A.TLTFDHSLEAQWTK.W	2	3.54	0.36	-3.06
IPI00012887	Cathepsin L1 precursor	K.AVATVGPISVAIDAGHESFLFYK.E	2	3.79	0.46	-1.01
IPI00012887	Cathepsin L1 precursor	K.GKVFQEPLFYEAPR.S	2	4.38	0.41	-1.95
IPI00012887	Cathepsin L1 precursor	K.GKVFQEPLFYEAPR.S	3	3.68	0.36	-1.93

IPI00012887	Cathepsin L1 precursor	K.GYVTPVK.N	1	1.84	0.17	-0.52
IPI00012887	Cathepsin L1 precursor	K.HSFTM*AM*NAFGDM*TSEEFR.Q	3	3.73	0.37	-3.97
IPI00012887	Cathepsin L1 precursor	K.M*IELHNQEYR.E	2	3.39	0.36	-2.95
IPI00012887	Cathepsin L1 precursor	K.M*IELHNQEYR.E	3	1.81	0.16	-2.99
IPI00012887	Cathepsin L1 precursor	K.M*IELHNQEYREGK.H	3	2.56	0.19	-2.57
IPI00012887	Cathepsin L1 precursor	K.NSWGEEWGM*GGYVK.M	2	4.08	0.41	-3.03
IPI00012887	Cathepsin L1 precursor	K.VFQEPLFYEAPR.S	2	4.17	0.42	-5.64
IPI00012887	Cathepsin L1 precursor	R.EKGYVTPVK.N	1	2.51	0.13	-2.01
IPI00012887	Cathepsin L1 precursor	R.EKGYVTPVK.N	2	1.54	0.32	-1.62
IPI00012887	Cathepsin L1 precursor	R.LYGM*NEEGWR.R	2	3.29	0.43	-1.56
IPI00012887	Cathepsin L1 precursor	R.NHCGIASAASYPTV.-	2	2.18	0.14	-1.86
IPI00012887	Cathepsin L1 precursor	R.QVM*NGFQNR.K	2	2.79	0.30	-1.52
IPI00012887	Cathepsin L1 precursor	W.AFSATGALEGQM*FR.K	2	4.41	0.50	-0.83
IPI00012895	Isoform 1 of Carbonic anhydrase 12 precursor	K.YKGQEAFVPGFNIEELLPER.T	3	2.53	0.11	-0.21
IPI00012948	Proheparin-binding EGF-like growth factor precursor	R.DLQEADLDLLR.V	2	3.88	0.31	-4.05
IPI00012989	Lysosomal alpha-mannosidase precursor	K.ELVDYFLNVATAQGR.Y	2	4.58	0.46	-2.32
IPI00012989	Lysosomal alpha-mannosidase precursor	L.DPANITLEPM*EIR.T	2	3.79	0.27	-3.09
IPI00012989	Lysosomal alpha-mannosidase precursor	R.ASTSLKPPTADLFTGVLPNGYNPPR.N	3	4.74	0.25	-2.61
IPI00012989	Lysosomal alpha-mannosidase precursor	R.ATFDPDTGLLM*EIM*NM*NQQLLLPVR.Q	3	2.86	0.19	-5.40
IPI00012989	Lysosomal alpha-mannosidase precursor	R.DLFSTFTITR.L	2	2.67	0.23	-3.13
IPI00012989	Lysosomal alpha-mannosidase precursor	R.FLEDTFGNDGRPR.V	2	2.16	0.14	-0.66
IPI00012989	Lysosomal alpha-mannosidase precursor	R.HLVLLDTAQAAAAGHR.L	3	4.97	0.51	-2.90
IPI00012989	Lysosomal alpha-mannosidase precursor	R.IYITDGNM*QLTVLTDR.S	2	5.21	0.47	-5.56
IPI00012989	Lysosomal alpha-mannosidase precursor	R.LLKDDGR.G	2	1.80	0.09	-3.36
IPI00012989	Lysosomal alpha-mannosidase precursor	R.LQETTLVANQLR.E	2	4.27	0.35	-3.29
IPI00012989	Lysosomal alpha-mannosidase precursor	R.QHVANDYAR.Q	2	1.39	0.18	-2.70
IPI00012989	Lysosomal alpha-mannosidase precursor	R.QLAAGWGPCEVLLSNALAR.L	3	3.98	0.24	-4.07
IPI00012989	Lysosomal alpha-mannosidase precursor	W.SPALTIENEHIR.A	2	3.29	0.36	-4.80
IPI00013004	Isoform 1 of Pyridoxal kinase	K.VVPLADIITPNQFEAELLSGR.K	3	2.65	0.30	-4.88
IPI00013096	Isoform 1 of Receptor-type tyrosine-protein phosphatase T precursor	K.AVGSLDPSADLSSQR.G	2	3.87	0.39	-3.17
IPI00013096	Isoform 1 of Receptor-type tyrosine-protein phosphatase T precursor	R.DTALM*VTR.V	2	2.60	0.16	-2.92
IPI00013096	Isoform 1 of Receptor-type tyrosine-protein phosphatase T precursor	R.QLTLQWEPFGYAVTR.C	2	2.28	0.10	-2.91
IPI00013096	Isoform 1 of Receptor-type tyrosine-protein phosphatase T precursor	R.SSPGALNVYVK.V	2	2.91	0.14	-2.88
IPI00013096	Isoform 1 of Receptor-type tyrosine-protein phosphatase T precursor	R.VLLTRPGEGGTGPPGPPLTTR.T	3	4.08	0.41	-4.36
IPI00013162	Isoform 1 of OX-2 membrane glycoprotein precursor	K.AVSPENM*VTFSENHGVVIQPAYK.D	3	2.76	0.29	-1.85

IPI00013162	Isoform 1 of OX-2 membrane glycoprotein precursor	K.CSLQNAQEALIVTWQK.K	2	5.72	0.42	-2.93
IPI00013162	Isoform 1 of OX-2 membrane glycoprotein precursor	K.CSLQNAQEALIVTWQK.K	3	4.01	0.10	-2.25
IPI00013162	Isoform 1 of OX-2 membrane glycoprotein precursor	K.NQVGKEVICQVLHLGTVTDFK.Q	3	3.71	0.39	-5.33
IPI00013179	Prostaglandin-H2 D-isomerase precursor	A.APEAQVSVQPNFQQDK.F	1	3.94	0.51	-2.40
IPI00013179	Prostaglandin-H2 D-isomerase precursor	A.APEAQVSVQPNFQQDK.F	2	5.88	0.54	-8.98
IPI00013179	Prostaglandin-H2 D-isomerase precursor	A.APEAQVSVQPNFQQDK.F	3	4.17	0.33	-3.83
IPI00013179	Prostaglandin-H2 D-isomerase precursor	A.APEAQVSVQPNFQQDKFLGR.W	2	5.43	0.56	-5.58
IPI00013179	Prostaglandin-H2 D-isomerase precursor	A.APEAQVSVQPNFQQDKFLGR.W	3	5.89	0.55	-5.06
IPI00013179	Prostaglandin-H2 D-isomerase precursor	A.GSLGSYSYR.S	1	1.93	0.26	-4.54
IPI00013179	Prostaglandin-H2 D-isomerase precursor	A.GSLGSYSYR.S	2	3.05	0.22	1.41
IPI00013179	Prostaglandin-H2 D-isomerase precursor	A.PATDGGLNLTSTFLRK.N	2	3.80	0.43	0.83
IPI00013179	Prostaglandin-H2 D-isomerase precursor	A.PEAQVSVQPNFQQDK.F	2	5.13	0.47	-4.82
IPI00013179	Prostaglandin-H2 D-isomerase precursor	A.PEAQVSVQPNFQQDKFLGR.W	2	5.00	0.53	-5.35
IPI00013179	Prostaglandin-H2 D-isomerase precursor	A.PEAQVSVQPNFQQDKFLGR.W	3	5.20	0.50	-8.48
IPI00013179	Prostaglandin-H2 D-isomerase precursor	A.QGFTEDTIVFLPQTDK.C	2	4.82	0.49	-4.90
IPI00013179	Prostaglandin-H2 D-isomerase precursor	A.QVSVQPNFQQDK.F	2	3.18	0.19	-3.23
IPI00013179	Prostaglandin-H2 D-isomerase precursor	A.QVSVQPNFQQDKFLGR.W	2	3.45	0.39	-2.39
IPI00013179	Prostaglandin-H2 D-isomerase precursor	C.KSVVAPATDGGLNLTSTFLR.K	2	4.77	0.53	-4.07
IPI00013179	Prostaglandin-H2 D-isomerase precursor	D.LQAAPEAQVSVQPNFQQDK.F	2	6.15	0.49	-3.06
IPI00013179	Prostaglandin-H2 D-isomerase precursor	D.LQAAPEAQVSVQPNFQQDK.F	3	3.78	0.28	-2.14
IPI00013179	Prostaglandin-H2 D-isomerase precursor	D.TIVFLPQTDK.C	1	2.01	0.22	-3.27
IPI00013179	Prostaglandin-H2 D-isomerase precursor	D.TIVFLPQTDK.C	2	2.97	0.16	-3.27
IPI00013179	Prostaglandin-H2 D-isomerase precursor	D.YDQYALLYSQGSK.G	2	4.84	0.51	-3.28
IPI00013179	Prostaglandin-H2 D-isomerase precursor	D.YDQYALLYSQGSKGPGEDFR.M	3	4.70	0.54	-0.58
IPI00013179	Prostaglandin-H2 D-isomerase precursor	E.AQVSVQPNFQQDK.F	1	2.38	0.26	-2.84
IPI00013179	Prostaglandin-H2 D-isomerase precursor	E.AQVSVQPNFQQDK.F	2	4.05	0.39	-4.01
IPI00013179	Prostaglandin-H2 D-isomerase precursor	E.AQVSVQPNFQQDKFLGR.W	2	5.22	0.59	-2.77
IPI00013179	Prostaglandin-H2 D-isomerase precursor	E.DTIVFLPQTDK.C	2	3.71	0.22	-3.50
IPI00013179	Prostaglandin-H2 D-isomerase precursor	E.TDYDQYALLYSQGSK.G	2	3.38	0.27	-0.92
IPI00013179	Prostaglandin-H2 D-isomerase precursor	F.TEDTIVFLPQTDK.C	2	4.74	0.45	-4.92
IPI00013179	Prostaglandin-H2 D-isomerase precursor	G.DLQAAPEAQVSVQPNFQQDK.F	2	6.44	0.56	-6.56
IPI00013179	Prostaglandin-H2 D-isomerase precursor	G.DLQAAPEAQVSVQPNFQQDK.F	3	5.35	0.40	-5.17
IPI00013179	Prostaglandin-H2 D-isomerase precursor	G.DLQAAPEAQVSVQPNFQQDKFLGR.W	2	4.02	0.55	-2.36
IPI00013179	Prostaglandin-H2 D-isomerase precursor	G.DLQAAPEAQVSVQPNFQQDKFLGR.W	3	3.62	0.44	-3.31
IPI00013179	Prostaglandin-H2 D-isomerase precursor	G.FTEDTIVFLPQTDK.C	2	3.98	0.37	-4.45
IPI00013179	Prostaglandin-H2 D-isomerase precursor	G.VLGDLQAAPEAQVSVQPNFQQDK.F	2	5.19	0.60	-4.06
IPI00013179	Prostaglandin-H2 D-isomerase precursor	G.VLGDLQAAPEAQVSVQPNFQQDK.F	3	4.54	0.37	-4.34
IPI00013179	Prostaglandin-H2 D-isomerase precursor	G.VLGDLQAAPEAQVSVQPNFQQDKFLGR.W	3	4.59	0.39	-3.03
IPI00013179	Prostaglandin-H2 D-isomerase precursor	K.AQGFTEDTIVFLPQTD.K	2	3.41	0.22	-4.55

IPI00013179	Prostaglandin-H2 D-isomerase precursor	K.AQGFTEDTIVFLPQTDK.C	1	4.58	0.43	-3.12
IPI00013179	Prostaglandin-H2 D-isomerase precursor	K.AQGFTEDTIVFLPQTDK.C	2	5.71	0.52	-8.61
IPI00013179	Prostaglandin-H2 D-isomerase precursor	K.AQGFTEDTIVFLPQTDK.C	3	5.47	0.30	-5.76
IPI00013179	Prostaglandin-H2 D-isomerase precursor	K.AQGFTEDTIVFLPQTDKCM*TEQ.-	2	5.29	0.52	-6.00
IPI00013179	Prostaglandin-H2 D-isomerase precursor	K.AQGFTEDTIVFLPQTDKCM*TEQ.-	3	5.73	0.51	-6.42
IPI00013179	Prostaglandin-H2 D-isomerase precursor	K.AQGFTEDTIVFLPQTDKCMTEQ.-	2	4.19	0.41	-2.53
IPI00013179	Prostaglandin-H2 D-isomerase precursor	K.AQGFTEDTIVFLPQTDKCMTEQ.-	3	4.66	0.34	-7.92
IPI00013179	Prostaglandin-H2 D-isomerase precursor	K.EKFTAF.C	1	1.95	0.17	-2.09
IPI00013179	Prostaglandin-H2 D-isomerase precursor	K.EKFTAFCK.A	1	3.19	0.24	-2.25
IPI00013179	Prostaglandin-H2 D-isomerase precursor	K.EKFTAFCK.A	2	2.01	0.16	-1.77
IPI00013179	Prostaglandin-H2 D-isomerase precursor	K.FTAFCKAQGFTEDTIVFLPQTDK.C	3	3.18	0.26	-1.31
IPI00013179	Prostaglandin-H2 D-isomerase precursor	K.GPGEDFR.M	1	1.89	0.11	-3.93
IPI00013179	Prostaglandin-H2 D-isomerase precursor	K.GPGEDFRM*ATLYSR.T	3	1.95	0.12	-2.79
IPI00013179	Prostaglandin-H2 D-isomerase precursor	K.KAALSM*CK.S	2	1.93	0.13	-3.09
IPI00013179	Prostaglandin-H2 D-isomerase precursor	K.NQCETRTM*LLQPAGSLGSYSYR.S	3	3.46	0.37	-1.35
IPI00013179	Prostaglandin-H2 D-isomerase precursor	K.SVVAPATD.G	1	1.86	0.28	-2.18
IPI00013179	Prostaglandin-H2 D-isomerase precursor	K.SVVAPATDGGLNLTSTFLR.K	2	6.16	0.62	-3.92
IPI00013179	Prostaglandin-H2 D-isomerase precursor	K.SVVAPATDGGLNLTSTFLR.K	3	4.67	0.49	-2.57
IPI00013179	Prostaglandin-H2 D-isomerase precursor	K.SVVAPATDGGLNLTSTFLRK.N	2	4.31	0.46	-2.86
IPI00013179	Prostaglandin-H2 D-isomerase precursor	K.SVVAPATDGGLNLTSTFLRK.N	3	2.53	0.22	-2.92
IPI00013179	Prostaglandin-H2 D-isomerase precursor	L.GDLQAAPEAQVSVQPNFQQDK.F	2	4.91	0.54	-1.06
IPI00013179	Prostaglandin-H2 D-isomerase precursor	L.GDLQAAPEAQVSVQPNFQQDK.F	3	4.12	0.25	-0.83
IPI00013179	Prostaglandin-H2 D-isomerase precursor	L.GDLQAAPEAQVSVQPNFQQDKFLGR.W	3	3.57	0.28	-3.03
IPI00013179	Prostaglandin-H2 D-isomerase precursor	L.LQPAGSLGSYSYR.S	1	2.45	0.28	-2.23
IPI00013179	Prostaglandin-H2 D-isomerase precursor	L.LQPAGSLGSYSYR.S	2	3.68	0.46	-2.97
IPI00013179	Prostaglandin-H2 D-isomerase precursor	L.QPAGSLGSYSYR.S	1	2.00	0.16	-2.13
IPI00013179	Prostaglandin-H2 D-isomerase precursor	M.LLQPAGSLGSYSYR.S	2	4.35	0.52	-4.36
IPI00013179	Prostaglandin-H2 D-isomerase precursor	P.AGSLGSYSYR.S	2	3.21	0.22	-0.53
IPI00013179	Prostaglandin-H2 D-isomerase precursor	P.EAQVSVQPNFQQDK.F	1	2.68	0.30	-2.55
IPI00013179	Prostaglandin-H2 D-isomerase precursor	P.EAQVSVQPNFQQDK.F	2	4.46	0.44	-4.66
IPI00013179	Prostaglandin-H2 D-isomerase precursor	P.EAQVSVQPNFQQDKFLGR.W	2	4.61	0.53	-2.99
IPI00013179	Prostaglandin-H2 D-isomerase precursor	Q.AAPEAQVSVQPNFQQDK.F	2	4.91	0.51	-3.65
IPI00013179	Prostaglandin-H2 D-isomerase precursor	Q.GFTEDTIVFLPQTDK.C	2	4.10	0.42	-3.97
IPI00013179	Prostaglandin-H2 D-isomerase precursor	Q.PAGSLGSYSYR.S	1	3.19	0.40	-8.99
IPI00013179	Prostaglandin-H2 D-isomerase precursor	Q.PAGSLGSYSYR.S	2	3.73	0.45	-2.86
IPI00013179	Prostaglandin-H2 D-isomerase precursor	Q.PNFQQDKFLGR.W	1	3.15	0.33	-3.03
IPI00013179	Prostaglandin-H2 D-isomerase precursor	Q.PNFQQDKFLGR.W	2	4.40	0.45	-3.01
IPI00013179	Prostaglandin-H2 D-isomerase precursor	Q.VSVQPNFQQDKFLGR.W	2	3.94	0.43	-2.79
IPI00013179	Prostaglandin-H2 D-isomerase precursor	R.M*ATLYSR.T	2	2.22	0.28	-6.25
IPI00013179	Prostaglandin-H2 D-isomerase precursor	R.M*ATLYSRTQTPR.A	3	1.85	0.22	-0.13
IPI00013179	Prostaglandin-H2 D-isomerase precursor	R.MATLYSR.T	2	1.66	0.17	-2.17
IPI00013179	Prostaglandin-H2 D-isomerase precursor	R.SPHWGSTYSVSVVETDYDQYALLYSQGSK.G	2	4.84	0.52	

IPI00013179	Prostaglandin-H2 D-isomerase precursor	R.SPHWGSTYSVSVVETDYDQYALLYSQGSK.G	3	6.67	0.59	-7.50
IPI00013179	Prostaglandin-H2 D-isomerase precursor	R.SPHWGSTYSVSVVETDYDQYALLYSQGSKGPGEDFR.M	3	4.86	0.52	-4.84
IPI00013179	Prostaglandin-H2 D-isomerase precursor	R.SPHWGSTYSVSVVETDYDQYALLYSQGSKGPGEDFR.M	4	4.69	0.41	-6.14
IPI00013179	Prostaglandin-H2 D-isomerase precursor	R.TM*LLQPAGSLGS.Y	1	2.69	0.42	-2.91
IPI00013179	Prostaglandin-H2 D-isomerase precursor	R.TM*LLQPAGSLGS.Y	2	3.41	0.30	-1.90
IPI00013179	Prostaglandin-H2 D-isomerase precursor	R.TM*LLQPAGSLGSY.S	1	2.81	0.44	-1.81
IPI00013179	Prostaglandin-H2 D-isomerase precursor	R.TM*LLQPAGSLGSY.S	2	3.63	0.42	-2.18
IPI00013179	Prostaglandin-H2 D-isomerase precursor	R.TM*LLQPAGSLGSYS.Y	1	2.48	0.38	-1.10
IPI00013179	Prostaglandin-H2 D-isomerase precursor	R.TM*LLQPAGSLGSYS.Y	2	3.49	0.39	0.36
IPI00013179	Prostaglandin-H2 D-isomerase precursor	R.TM*LLQPAGSLGSYSY.R	1	3.26	0.50	-1.49
IPI00013179	Prostaglandin-H2 D-isomerase precursor	R.TM*LLQPAGSLGSYSY.R	2	3.65	0.44	0.10
IPI00013179	Prostaglandin-H2 D-isomerase precursor	R.TM*LLQPAGSLGSYSYR.S	2	5.96	0.49	-8.81
IPI00013179	Prostaglandin-H2 D-isomerase precursor	R.TM*LLQPAGSLGSYSYR.S	3	5.50	0.48	-4.26
IPI00013179	Prostaglandin-H2 D-isomerase precursor	R.TMLLQPAGSLGSYSYR.S	2	5.31	0.51	-3.64
IPI00013179	Prostaglandin-H2 D-isomerase precursor	R.TQTPRAELK.E	2	2.22	0.06	-0.48
IPI00013179	Prostaglandin-H2 D-isomerase precursor	R.WFSAGLASNSSWLR.E	1	3.54	0.34	-0.98
IPI00013179	Prostaglandin-H2 D-isomerase precursor	R.WFSAGLASNSSWLR.E	2	5.03	0.48	-7.00
IPI00013179	Prostaglandin-H2 D-isomerase precursor	S.VQPNFQQDKFLGR.W	2	3.04	0.44	-4.87
IPI00013179	Prostaglandin-H2 D-isomerase precursor	S.VSVVETDYDQYALLYSQGSKGPGEDFR.M	3	3.85	0.28	-4.45
IPI00013179	Prostaglandin-H2 D-isomerase precursor	S.VVETDYDQYALLYSQGSK.G	2	5.82	0.58	-5.60
IPI00013179	Prostaglandin-H2 D-isomerase precursor	S.VVETDYDQYALLYSQGSK.G	3	5.33	0.47	-3.26
IPI00013179	Prostaglandin-H2 D-isomerase precursor	S.VVETDYDQYALLYSQGSKGPGEDFR.M	3	4.18	0.55	-3.71
IPI00013179	Prostaglandin-H2 D-isomerase precursor	T.EDTIVFLPQTDK.C	2	3.23	0.22	-4.08
IPI00013179	Prostaglandin-H2 D-isomerase precursor	T.M*LLQPAGSLGSYSYR.S	2	4.71	0.48	-3.20
IPI00013179	Prostaglandin-H2 D-isomerase precursor	V.SVQPNFQQDKFLGR.W	2	4.11	0.43	-5.27
IPI00013179	Prostaglandin-H2 D-isomerase precursor	V.VETDYDQYALLYSQGSK.G	2	5.74	0.55	-3.25
IPI00013179	Prostaglandin-H2 D-isomerase precursor	W.GSTYSVSVVETDYDQYALLYSQGSK.G	2	5.23	0.57	-5.45
IPI00013179	Prostaglandin-H2 D-isomerase precursor	W.GSTYSVSVVETDYDQYALLYSQGSK.G	3	5.57	0.54	-7.45
IPI00013179	Prostaglandin-H2 D-isomerase precursor	W.GSTYSVSVVETDYDQYALLYSQGSKGPGEDFR.M	3	4.49	0.54	-5.28
IPI00013179	Prostaglandin-H2 D-isomerase precursor	W.GSTYSVSVVETDYDQYALLYSQGSKGPGEDFR.M	4	4.81	0.49	-3.13
IPI00013179	Prostaglandin-H2 D-isomerase precursor	Y.ALLYSQGSK.G	1	2.04	0.21	-3.85
IPI00013179	Prostaglandin-H2 D-isomerase precursor	Y.ALLYSQGSKGPGEDFR.M	2	3.53	0.42	-5.38
IPI00013179	Prostaglandin-H2 D-isomerase precursor	Y.DQYALLYSQGSK.G	2	4.01	0.47	-5.26
IPI00013179	Prostaglandin-H2 D-isomerase precursor	Y.DQYALLYSQGSKGPGEDFR.M	2	5.05	0.53	-3.27
IPI00013179	Prostaglandin-H2 D-isomerase precursor	Y.DQYALLYSQGSKGPGEDFR.M	3	3.77	0.43	-2.73
IPI00013179	Prostaglandin-H2 D-isomerase precursor	Y.SQGSKGPGEDFR.M	2	3.22	0.41	-2.42
IPI00013179	Prostaglandin-H2 D-isomerase precursor	Y.SVSVVETDYDQYALLYSQGSK.G	2	6.40	0.61	-5.67
IPI00013179	Prostaglandin-H2 D-isomerase precursor	Y.SVSVVETDYDQYALLYSQGSK.G	3	6.48	0.56	-4.35
IPI00013179	Prostaglandin-H2 D-isomerase precursor	Y.SVSVVETDYDQYALLYSQGSKGPGEDFR.M	3	4.09	0.48	-4.89
IPI00013216	Origin recognition complex subunit 2	K.DQNYVEIMGRDVQESLKNGSATG.G	2	3.72	0.14	0.63
IPI00013219	Integrin-linked protein kinase	R.SVMIDEDMTAR.I	2	2.46	0.26	-3.05
IPI00013272	Isoform 1 of Golgin subfamily A member 4	R.GKYSELVTAYQM*LQREKK.K	3	2.97	0.14	-4.23

IPI00013281	Fukutin-related protein	R.LVAAPVATANPAR.C	2	3.38	0.42	-4.58
IPI00013290	hepatoma-derived growth factor-related protein 2 isoform 1	R.EGPDLDRPGSDRQERER.A	2	1.45	0.09	1.82
IPI00013299	Neuroblastoma, suppression of tumorigenicity 1	K.LALFPDK.S	1	2.35	0.22	-2.79
IPI00013299	Neuroblastoma, suppression of tumorigenicity 1	K.LALFPDK.S	2	2.37	0.11	-3.65
IPI00013302	ADAM 15 precursor	R.ESVHTQTPPEHPLGQR.H	3	3.21	0.32	
IPI00013303	Limbic system-associated membrane protein precursor	K.AANEVSSADVK.Q	1	2.94	0.30	-3.68
IPI00013303	Limbic system-associated membrane protein precursor	K.AANEVSSADVK.Q	2	3.82	0.41	-3.35
IPI00013303	Limbic system-associated membrane protein precursor	K.CEASAVPAPDFEWYR.D	2	4.38	0.52	-3.82
IPI00013303	Limbic system-associated membrane protein precursor	K.CEASAVPAPDFEWYRDDTR.I	2	3.63	0.43	-3.50
IPI00013303	Limbic system-associated membrane protein precursor	K.CEASAVPAPDFEWYRDDTR.I	3	2.78	0.26	-3.53
IPI00013303	Limbic system-associated membrane protein precursor	K.RHSLEYSLR.I	2	2.48	0.17	-3.77
IPI00013303	Limbic system-associated membrane protein precursor	K.TSQVYLIVQVPPK.I	2	4.51	0.43	-7.19
IPI00013303	Limbic system-associated membrane protein precursor	K.TSQVYLIVQVPPK.I	3	4.72	0.29	-2.96
IPI00013303	Limbic system-associated membrane protein precursor	K.VDVYDEGSYTCSVQTQHEPK.T	2	4.77	0.57	-3.10
IPI00013303	Limbic system-associated membrane protein precursor	K.VDVYDEGSYTCSVQTQHEPK.T	3	2.62	0.34	-2.94
IPI00013303	Limbic system-associated membrane protein precursor	K.VTVNYPPTITESK.S	1	2.49	0.35	-3.33
IPI00013303	Limbic system-associated membrane protein precursor	K.VTVNYPPTITESK.S	2	3.54	0.37	-4.12
IPI00013303	Limbic system-associated membrane protein precursor	R.EFEGEEEYLEILGITR.E	2	6.13	0.48	-8.15
IPI00013303	Limbic system-associated membrane protein precursor	R.EFEGEEEYLEILGITR.E	3	4.31	0.39	-4.38
IPI00013303	Limbic system-associated membrane protein precursor	R.GTDNITVR.Q	1	2.09	0.12	-2.30
IPI00013303	Limbic system-associated membrane protein precursor	R.GTDNITVR.Q	2	2.64	0.07	-1.58
IPI00013303	Limbic system-associated membrane protein precursor	R.HLTPTGREFEGEEEYLEILGITR.E	3	4.35	0.42	-4.47
IPI00013303	Limbic system-associated membrane protein precursor	R.HSLEYSLR.I	1	2.70	0.30	-3.76

IPI00013303	Limbic system-associated membrane protein precursor	R.HSLEYSLR.I	2	2.61	0.21	-2.64
IPI00013303	Limbic system-associated membrane protein precursor	R.INSANGLEIK.S	2	3.27	0.25	-1.82
IPI00013303	Limbic system-associated membrane protein precursor	R.IQKVDVYDEGSYTCSVQTQHEPK.T	3	5.16	0.46	-3.81
IPI00013303	Limbic system-associated membrane protein precursor	R.QGDTAILR.C	1	1.33	0.06	-2.18
IPI00013303	Limbic system-associated membrane protein precursor	R.QGDTAILR.C	2	1.71	0.08	-2.67
IPI00013303	Limbic system-associated membrane protein precursor	R.SGIIFAGHDK.W	1	2.25	0.28	-4.50
IPI00013303	Limbic system-associated membrane protein precursor	R.SGIIFAGHDK.W	2	2.70	0.23	-2.85
IPI00013303	Limbic system-associated membrane protein precursor	R.SVDFNRGTDNITVR.Q	3	2.92	0.26	-1.34
IPI00013319	Isoform 2 of 43 kDa receptor-associated protein of the synapse	R.SCPNCRRSSM*KPGFV.-	2	2.55	0.12	
IPI00013455	CLIP1 protein	K.AAQTAEDAMQIMEQM*TK.E	3	2.94	0.18	
IPI00013466	Arsenical pump-driving ATPase	R.SVSEQFKDPEQTTFICVCIAEFLSLYETER.L	3	4.52	0.46	-2.07
IPI00013475	Tubulin beta-2A chain	K.GHYTEGAELVDSVLDVVR.K	2	2.94	0.28	
IPI00013475	Tubulin beta-2A chain	K.GHYTEGAELVDSVLDVVR.K	3	3.28	0.30	-3.01
IPI00013475	Tubulin beta-2A chain	K.GHYTEGAELVDSVLDVVRK.E	3	3.51	0.45	-3.20
IPI00013475	Tubulin beta-2A chain	K.NM*M*AACDPR.H	2	1.73	0.12	0.11
IPI00013475	Tubulin beta-2A chain	K.VSDTVVEPYNATLSVHQLVENTDETYSIDNEALYDICFR.T	3	5.67	0.60	-3.14
IPI00013475	Tubulin beta-2A chain	K.VSDTVVEPYNATLSVHQLVENTDETYSIDNEALYDICFR.T	4	4.05	0.31	-2.09
IPI00013475	Tubulin beta-2A chain	R.ISEQFTAM*FR.R	2	2.17	0.13	-2.65
IPI00013475	Tubulin beta-2A chain	R.ISEQFTAMFR.R	2	2.87	0.27	-0.81
IPI00013475	Tubulin beta-2A chain	R.LHFFM*PGFAPLTSR.G	3	3.20	0.06	-3.00
IPI00013475	Tubulin beta-2A chain	R.LHFFMPGFAPLTSR.G	3	2.62	0.11	-1.73
IPI00013495	Isoform 2 of ATP-binding cassette sub-family F member 1	K.FAALDNEEEDK.E	2	2.63	0.11	
IPI00013508	Alpha-actinin-1	R.ISIEMHGTLEDQLSHLR.Q	3	3.48	0.21	-3.89
IPI00013569	Isoform 1 of Pappalysin-2 precursor	K.ESVHLGPLDTFCDIPLTIK.L	3	3.40	0.16	-2.54
IPI00013569	Isoform 1 of Pappalysin-2 precursor	R.EAETFNSQVGLPILYFSGR.R	2	4.87	0.58	-4.64
IPI00013569	Isoform 1 of Pappalysin-2 precursor	R.LLLRPEVLAEIPR.E	2	2.85	0.31	-2.52
IPI00013569	Isoform 1 of Pappalysin-2 precursor	R.LQHEALNEAFSR.Y	2	3.64	0.39	-4.77
IPI00013569	Isoform 1 of Pappalysin-2 precursor	R.SKESLGEAGIQK.G	2	3.23	0.26	-2.38
IPI00013569	Isoform 1 of Pappalysin-2 precursor	R.SLLLGGDSSEDGHYFR.G	2	4.39	0.53	-2.45
IPI00013569	Isoform 1 of Pappalysin-2 precursor	R.SLLLGGDSSEDGHYFR.G	3	2.33	0.15	-1.46
IPI00013569	Isoform 1 of Pappalysin-2 precursor	R.VGISAVALR.T	2	3.62	0.19	-1.80

IPI00013681	N-terminally extended type 3 canonical transient receptor potential channel	K.CLVVLVVALGLPFLAIGYWIAPCSRLGKILR.S	2	1.06	0.18	-5.96
IPI00013682	Isoform 3 of Ecto-ADP-ribosyltransferase 3 precursor	A.EVLDM*ADNAFDDEYLK.C	2	5.53	0.58	-5.03
IPI00013682	Isoform 3 of Ecto-ADP-ribosyltransferase 3 precursor	K.AFHFYLTR.A	1	2.13	0.16	-3.45
IPI00013682	Isoform 3 of Ecto-ADP-ribosyltransferase 3 precursor	K.AFHFYLTR.A	2	2.47	0.23	-1.82
IPI00013682	Isoform 3 of Ecto-ADP-ribosyltransferase 3 precursor	K.M*AGQSREDYIYGFQFK.A	2	3.28	0.40	-3.30
IPI00013682	Isoform 3 of Ecto-ADP-ribosyltransferase 3 precursor	K.M*AGQSREDYIYGFQFK.A	3	4.00	0.40	-2.11
IPI00013682	Isoform 3 of Ecto-ADP-ribosyltransferase 3 precursor	K.TCSHYECAFLGGLK.T	3	3.22	0.29	-2.56
IPI00013682	Isoform 3 of Ecto-ADP-ribosyltransferase 3 precursor	K.TQIFLPM*NFK.D	2	3.01	0.10	-2.54
IPI00013682	Isoform 3 of Ecto-ADP-ribosyltransferase 3 precursor	K.YVPQLLKEEK.A	2	2.75	0.17	-3.54
IPI00013682	Isoform 3 of Ecto-ADP-ribosyltransferase 3 precursor	R.EDYIYGFQFK.A	2	3.41	0.38	-3.09
IPI00013682	Isoform 3 of Ecto-ADP-ribosyltransferase 3 precursor	R.KTQIFLPM*NFK.D	2	2.51	0.19	-1.27
IPI00013682	Isoform 3 of Ecto-ADP-ribosyltransferase 3 precursor	R.TSQGTSFTFGGLNQAR.F	2	5.05	0.44	-3.13
IPI00013682	Isoform 3 of Ecto-ADP-ribosyltransferase 3 precursor	R.TSQGTSFTFGGLNQAR.F	3	5.11	0.42	-1.77
IPI00013698	Acid ceramidase precursor	K.DAM*WIGFLTR.T	2	2.37	0.23	-3.75
IPI00013698	Acid ceramidase precursor	K.ESLDVYELDAK.Q	2	3.61	0.34	-1.32
IPI00013698	Acid ceramidase precursor	K.GQFETYLR.D	2	1.96	0.08	-3.47
IPI00013698	Acid ceramidase precursor	K.GQFETYLRDCPDPCIGW.-	2	2.66	0.11	
IPI00013698	Acid ceramidase precursor	K.LTVYTTLIDVTK.G	2	4.11	0.45	-6.52
IPI00013698	Acid ceramidase precursor	K.NM*INTFVPSGK.V	2	3.08	0.34	0.29
IPI00013698	Acid ceramidase precursor	K.STYPPSGPTYR.G	2	2.48	0.44	-3.28
IPI00013698	Acid ceramidase precursor	K.VIVNSLK.N	1	1.50	0.14	-2.16
IPI00013698	Acid ceramidase precursor	K.VM*QVVDEKLPGLLGNFPGPFEEEM*K.G	3	4.33	0.22	-5.13
IPI00013698	Acid ceramidase precursor	R.DRKESLDVYELDAK.Q	2	2.49	0.20	-0.43
IPI00013698	Acid ceramidase precursor	R.KSTYPPSGPTYR.G	2	2.10	0.06	-3.56
IPI00013698	Acid ceramidase precursor	R.TSQENISFETM*YDVLSTKPVLNK.L	3	3.15	0.32	-3.81
IPI00013698	Acid ceramidase precursor	R.WYVVQTNYDR.W	2	2.96	0.31	-2.55
IPI00013701	Nociceptin precursor	K.VFPSPLWTPCTK.V	2	2.61	0.27	-3.67
IPI00013701	Nociceptin precursor	R.FGGFTGAR.K	2	2.36	0.21	-2.75
IPI00013701	Nociceptin precursor	R.SLFQEQEEPEPGM*EEAGEM*EQK.Q	2	4.39	0.48	-3.12

IPI00013701	Nociceptin precursor	R.SLFQEQEEPEPGM*EEAGEM*EQK.Q	3	3.03	0.42	-5.87
IPI00013749	Protein kinase C zeta type	R.VIGRGSYAKVLLVRLK.K	2	3.19	0.06	
IPI00013847	Cytochrome b-c1 complex subunit 1, mitochondrial precursor	R.MVLAAAGGVEHQQLLDLAQK.H	3	2.81	0.13	-3.36
IPI00013860	3-hydroxyisobutyrate dehydrogenase, mitochondrial precursor	K.DLGLAQDSATSTK.S	2	3.23	0.33	-2.63
IPI00013897	ADAM 10 precursor	K.AIDTIYQTTDFSGIR.N	2	4.36	0.42	-1.74
IPI00013897	ADAM 10 precursor	K.ENGNYIM*YAR.A	2	1.73	0.10	-1.58
IPI00013897	ADAM 10 precursor	K.SLNTGIITVQNYGSHVPPK.V	3	2.09	0.16	-3.83
IPI00013897	ADAM 10 precursor	R.FEGFIQTR.G	2	2.93	0.20	0.05
IPI00013897	ADAM 10 precursor	R.HYEGLSYNVDSLHQK.H	3	3.25	0.12	
IPI00013897	ADAM 10 precursor	R.TLPFHSVIYHEDDINYPHK.Y	3	2.30	0.30	-3.75
IPI00013933	Isoform DPI of Desmoplakin	K.NMPLQHLLEQIK.E	2	2.71	0.11	-6.93
IPI00013945	Isoform 1 of Uromodulin precursor	R.DSTIQVVENGESSQGR.F	2	4.48	0.43	-0.64
IPI00013945	Isoform 1 of Uromodulin precursor	R.FVGQGGAR.M	1	2.35	0.07	
IPI00013976	Laminin subunit beta-1 precursor	K.AM*DLDQDVLSALAEVEQLSK.M	2	6.91	0.39	-3.49
IPI00013976	Laminin subunit beta-1 precursor	K.AM*DLDQDVLSALAEVEQLSK.M	3	4.79	0.39	-3.34
IPI00013976	Laminin subunit beta-1 precursor	K.DVTEM*M*AQVEVK.L	2	2.65	0.22	-2.50
IPI00013976	Laminin subunit beta-1 precursor	K.EALEEAEKAQVAAEK.A	3	2.32	0.09	-0.77
IPI00013976	Laminin subunit beta-1 precursor	K.ELDSLQTEAESLDNTVKELAEQLEFIK.N	3	5.86	0.53	-3.38
IPI00013976	Laminin subunit beta-1 precursor	K.LHTLGDNLLDSR.M	2	2.42	0.26	-6.09
IPI00013976	Laminin subunit beta-1 precursor	K.M*DKSNEELR.N	2	2.27	0.08	-3.11
IPI00013976	Laminin subunit beta-1 precursor	K.NIGNLFEEAEK.L	2	2.44	0.18	-3.02
IPI00013976	Laminin subunit beta-1 precursor	K.TFRPAAM*LIER.S	3	3.41	0.21	-1.01
IPI00013976	Laminin subunit beta-1 precursor	K.TLDGELDEK.Y	2	2.12	0.16	-2.63
IPI00013976	Laminin subunit beta-1 precursor	K.TLDGELDEKYK.K	2	2.63	0.20	-0.68
IPI00013976	Laminin subunit beta-1 precursor	K.TLLAQANSK.L	1	2.24	0.20	-1.94
IPI00013976	Laminin subunit beta-1 precursor	K.TLLAQANSK.L	2	2.59	0.19	-0.42
IPI00013976	Laminin subunit beta-1 precursor	K.VSEIKDILAQSPAAEPLKNIGNLFEEAEK.L	3	3.14	0.22	-2.21
IPI00013976	Laminin subunit beta-1 precursor	R.ALDPAFKIEDPYSPR.I	3	3.29	0.29	-1.24
IPI00013976	Laminin subunit beta-1 precursor	R.FGYYGDALR.Q	2	3.01	0.33	-1.98
IPI00013976	Laminin subunit beta-1 precursor	R.IPSWTGAGFVR.V	2	3.21	0.36	-3.15
IPI00013976	Laminin subunit beta-1 precursor	R.KAAQNSGEAEYIEK.V	2	4.17	0.31	-3.16
IPI00013976	Laminin subunit beta-1 precursor	R.KVSEIKDILAQSPAAEPLK.N	3	4.11	0.36	-3.96
IPI00013976	Laminin subunit beta-1 precursor	R.KVSEIKDILAQSPAAEPLKNIGNLFEEAEK.L	5	3.34	0.26	-2.58
IPI00013976	Laminin subunit beta-1 precursor	R.LLDELAGK.L	2	2.56	0.06	-2.55
IPI00013976	Laminin subunit beta-1 precursor	R.NFLTQDSADLDSIEAVANEVLK.M	2	6.24	0.46	-6.16
IPI00013976	Laminin subunit beta-1 precursor	R.NFLTQDSADLDSIEAVANEVLK.M	3	5.78	0.42	-4.06
IPI00013976	Laminin subunit beta-1 precursor	R.NVEELKR.K	2	1.91	0.05	-3.03
IPI00013976	Laminin subunit beta-1 precursor	R.SLLKDISQK.V	2	2.13	0.17	-2.23
IPI00013976	Laminin subunit beta-1 precursor	R.VESLSQVEVILQHSAADIAR.A	3	2.87	0.30	-3.83
IPI00013976	Laminin subunit beta-1 precursor	R.YSDIEPSTEGEVIFR.A	2	4.88	0.45	-2.76

IPI00013978	Speckle-type POZ protein	K.NRVEINDVEPEVFKEM*M*CFIYTGK.A	3	3.26	0.09	-4.09
IPI00013991	Isoform 1 of Tropomyosin beta chain	R.LATALQKLEEAEKAADESER.G	3	3.26	0.29	-3.58
IPI00014048	Ribonuclease pancreatic precursor	K.ERHIIVACEGSPYVPVHFDASVEDST.-	3	4.21	0.42	-3.27
IPI00014048	Ribonuclease pancreatic precursor	K.SNSSM*HITDCR.L	2	2.78	0.32	-2.70
IPI00014048	Ribonuclease pancreatic precursor	K.SNSSM*HITDCR.L	3	1.74	0.21	-3.52
IPI00014048	Ribonuclease pancreatic precursor	K.SNSSMHITDCR.L	2	3.47	0.42	
IPI00014048	Ribonuclease pancreatic precursor	R.CKPVNTFVHEPLVDVQNVCFQEK.V	2	4.23	0.54	-2.93
IPI00014048	Ribonuclease pancreatic precursor	R.CKPVNTFVHEPLVDVQNVCFQEK.V	3	5.44	0.51	-4.63
IPI00014048	Ribonuclease pancreatic precursor	R.CKPVNTFVHEPLVDVQNVCFQEK.V	4	4.16	0.26	-3.98
IPI00014048	Ribonuclease pancreatic precursor	R.HIIVACEGSPYVPVH.F	2	4.35	0.62	-2.43
IPI00014048	Ribonuclease pancreatic precursor	R.HIIVACEGSPYVPVH.F	3	3.53	0.30	-1.59
IPI00014048	Ribonuclease pancreatic precursor	R.HIIVACEGSPYVPVHF.D	2	4.36	0.57	-3.41
IPI00014048	Ribonuclease pancreatic precursor	R.HIIVACEGSPYVPVHF.D	3	3.55	0.32	-2.22
IPI00014048	Ribonuclease pancreatic precursor	R.HIIVACEGSPYVPVHFDASVEDST.-	2	4.69	0.53	-4.74
IPI00014048	Ribonuclease pancreatic precursor	R.HIIVACEGSPYVPVHFDASVEDST.-	3	5.04	0.49	-5.15
IPI00014048	Ribonuclease pancreatic precursor	R.QHM*DSDSSPSSSSTYCNQM*M*R.R	2	2.55	0.47	-3.42
IPI00014048	Ribonuclease pancreatic precursor	R.QHM*DSDSSPSSSSTYCNQM*M*R.R	3	5.92	0.61	-5.08
IPI00014048	Ribonuclease pancreatic precursor	R.QHMDSDSSPSSSSTYCNQM*M*R.R	2	4.48	0.37	
IPI00014048	Ribonuclease pancreatic precursor	R.QHMDSDSSPSSSSTYCNQM*M*R.R	3	4.46	0.33	
IPI00014048	Ribonuclease pancreatic precursor	R.QHMDSDSSPSSSSTYCNQM*MR.R	3	3.51	0.09	
IPI00014048	Ribonuclease pancreatic precursor	R.YPNCAYR.T	2	2.35	0.22	-1.45
IPI00014223	Netrin-G1 ligand precursor	K.MTLHPQQIM*IGPRFNR.A	3	2.11	0.14	-7.23
IPI00014223	Netrin-G1 ligand precursor	R.NNPIESIPSYAFNR.V	2	4.73	0.49	-3.34
IPI00014223	Netrin-G1 ligand precursor	R.NNPIESIPSYAFNR.V	3	2.28	0.13	-1.75
IPI00014340	Isoform 1 of Protein phosphatase 1 regulatory subunit 12C	K.VELERATQR.Q	2	2.03	0.10	3.07
IPI00014371	Cadherin-18 precursor	K.VQDINDNAPK.F	2	3.01	0.20	-1.76
IPI00014398	Four and a half LIM domains 1 variant	K.AIVAGDQNVEYK.G	2	3.24	0.40	-1.27
IPI00014439	Dihydropteridine reductase	K.AALDGTPGM*IGYGM*AK.G	2	4.08	0.41	-3.25
IPI00014439	Dihydropteridine reductase	K.EGGLLTLAGAK.A	2	2.36	0.17	-0.63
IPI00014439	Dihydropteridine reductase	K.M*TDSFTEQADQVTAEVGK.L	2	6.45	0.57	-4.80
IPI00014439	Dihydropteridine reductase	K.M*TDSFTEQADQVTAEVGK.L	3	4.62	0.44	-3.87
IPI00014439	Dihydropteridine reductase	K.NRPSSGSLIQVVTTEGR.T	2	4.49	0.44	-3.37
IPI00014439	Dihydropteridine reductase	K.NRPSSGSLIQVVTTEGR.T	3	4.72	0.38	-3.45
IPI00014444	Isoform 1 of Protein SERAC1	R.IGTSTSPPK.S	2	1.94	0.22	
IPI00014516	Isoform 1 of Caldesmon	K.GNVFSSPTAAGTPNK.E	2	1.66	0.06	-1.20
IPI00014537	Isoform 1 of Calumenin precursor	R.QFLMCLSLCTAFALSKPTEK.K	2	2.11	0.07	0.94
IPI00014572	SPARC precursor	K.NVLVTLYER.D	2	3.51	0.26	-2.40
IPI00014572	SPARC precursor	K.NVLVTLYERDEDNNLLTEK.Q	2	5.32	0.45	-3.86
IPI00014572	SPARC precursor	K.NVLVTLYERDEDNNLLTEK.Q	3	4.52	0.51	-4.98
IPI00014572	SPARC precursor	K.NYNM*YIFPVHWQFGQLDQHPIDGYLSHTELAPLR.A	4	4.92	0.39	-2.88
IPI00014572	SPARC precursor	K.QKDIDKDLVI.-	1	1.57	0.12	-1.23

IPI00014572	SPARC precursor	K.RLEAGDHPVELLAR.D	2	4.43	0.41	-1.77
IPI00014572	SPARC precursor	K.RLEAGDHPVELLAR.D	3	3.40	0.33	-0.34
IPI00014572	SPARC precursor	K.TFDSSCHFFATK.C	2	3.71	0.36	
IPI00014572	SPARC precursor	K.VCELDENNTPM*CVCQDPTSCPAPIGEFEK.V	3	5.44	0.39	
IPI00014572	SPARC precursor	K.YIALDEWAGCFGIK.Q	2	4.97	0.51	-3.25
IPI00014572	SPARC precursor	K.YIPPCLDSELTEFPLR.M	2	2.72	0.39	-3.44
IPI00014572	SPARC precursor	K.YIPPCLDSELTEFPLR.M	3	3.83	0.24	-2.04
IPI00014572	SPARC precursor	L.DQHPIDGYLSHTELAPLR.A	3	3.59	0.37	-3.86
IPI00014572	SPARC precursor	L.YERDEDNNLLTEK.Q	2	4.37	0.46	-2.78
IPI00014572	SPARC precursor	R.DEDNNLLTEK.Q	2	3.38	0.30	-2.82
IPI00014572	SPARC precursor	R.FFETCDLDNDKYIALDEWAGCFGIK.Q	3	5.03	0.51	-3.74
IPI00014572	SPARC precursor	R.LEAGDHPVELLAR.D	1	2.86	0.34	-1.31
IPI00014572	SPARC precursor	R.LEAGDHPVELLAR.D	2	3.98	0.42	-2.35
IPI00014572	SPARC precursor	R.LEAGDHPVELLAR.D	3	2.51	0.25	-2.27
IPI00014572	SPARC precursor	Y.ERDEDNNLLTEK.Q	2	4.08	0.32	-2.62
IPI00014572	SPARC precursor	Y.IALDEWAGCFGIK.Q	2	4.28	0.47	-1.77
IPI00014592	Chondroadherin precursor	K.FSDGAFLGVTTLK.H	2	4.75	0.44	-6.02
IPI00014592	Chondroadherin precursor	R.AGAFDDLTELTYLYLDHNK.V	2	3.54	0.39	-2.51
IPI00014592	Chondroadherin precursor	R.AGAFDDLTELTYLYLDHNK.V	3	3.12	0.36	-3.78
IPI00014592	Chondroadherin precursor	R.AGAFDDLTELTYLYLDHNKVTELPR.G	4	3.53	0.41	-2.62
IPI00014592	Chondroadherin precursor	R.LNQLPSNFPFDSLETLALTNNPWK.C	3	4.17	0.34	-4.80
IPI00014592	Chondroadherin precursor	R.NNFPVLAANSFR.A	2	3.19	0.38	-3.26
IPI00014592	Chondroadherin precursor	R.NQLSSYPSAALSK.L	2	3.67	0.42	-2.34
IPI00014592	Chondroadherin precursor	R.VVEELKLSHNPLK.S	2	3.76	0.30	-3.50
IPI00014592	Chondroadherin precursor	R.WLYLSENALSSLQPGALDDVENLAK.F	3	4.16	0.44	-4.63
IPI00014592	Chondroadherin precursor	R.YLETLWLDNTNLEK.F	2	4.92	0.37	-0.62
IPI00014850	Astrocytic phosphoprotein PEA-15	R.RPDLLTM*VVDYR.T	3	3.30	0.33	-2.96
IPI00014899	CDNA FLJ20744 fis, clone HEP06585	R.LPCLHPAGHREASSAPAR.C	3	2.22	0.11	-8.71
IPI00014964	Lymphocyte antigen 6H precursor	K.M*CASSCDFVK.R	2	2.82	0.37	-1.29
IPI00014964	Lymphocyte antigen 6H precursor	K.M*CASSCDFVKR.H	2	3.13	0.26	-2.52
IPI00014964	Lymphocyte antigen 6H precursor	K.QCQPSDTVCASVR.I	2	3.72	0.42	-3.09
IPI00014964	Lymphocyte antigen 6H precursor	K.VDVDCCEK.D	2	2.25	0.23	-1.39
IPI00014964	Lymphocyte antigen 6H precursor	R.HFFSDYLM*GFINSGILK.V	2	4.51	0.41	-3.61
IPI00014964	Lymphocyte antigen 6H precursor	R.HFFSDYLM*GFINSGILK.V	3	4.92	0.34	-4.16
IPI00014964	Lymphocyte antigen 6H precursor	R.ITDPSSSR.K	2	1.98	0.05	-1.90
IPI00014964	Lymphocyte antigen 6H precursor	R.ITDPSSSRK.D	2	2.44	0.10	-3.83
IPI00015047	8D6 antigen (Fragment)	R.APAEVALEIEGPWTLPMEIRGARM*GNLPQPELR.G	4	2.74	0.15	-4.39
IPI00015049	Isoform 2 of Repulsive guidance molecule A precursor	K.CNSEFWSATSGSHAPASDDTPEFCAALR.S	3	6.07	0.59	-2.69
IPI00015049	Isoform 2 of Repulsive guidance molecule A precursor	K.NFQECVDQK.V	1	2.25	0.07	-5.04

IPI00015049	Isoform 2 of Repulsive guidance molecule A precursor	K.NFQECVDQK.V	2	2.68	0.13	-2.18
IPI00015049	Isoform 2 of Repulsive guidance molecule A precursor	K.VSGQHVEIQAK.Y	1	2.97	0.21	-3.51
IPI00015049	Isoform 2 of Repulsive guidance molecule A precursor	K.VSGQHVEIQAK.Y	2	3.70	0.33	-3.01
IPI00015049	Isoform 2 of Repulsive guidance molecule A precursor	K.VYQAEM*DELPAAFVDGSK.N	2	5.32	0.53	-4.71
IPI00015049	Isoform 2 of Repulsive guidance molecule A precursor	K.YIGTTIVVR.Q	2	1.84	0.19	-3.02
IPI00015049	Isoform 2 of Repulsive guidance molecule A precursor	R.SYALCTR.R	1	1.51	0.07	-2.82
IPI00015102	Isoform 1 of CD166 antigen precursor	G.WYTVNSAYGDTIIIPCR.L	2	4.76	0.50	-4.26
IPI00015102	Isoform 1 of CD166 antigen precursor	G.WYTVNSAYGDTIIIPCR.L	3	3.91	0.13	-3.09
IPI00015102	Isoform 1 of CD166 antigen precursor	G.WYTVNSAYGDTIIIPCRLDVPQNLM*FGK.W	3	4.39	0.35	-1.92
IPI00015102	Isoform 1 of CD166 antigen precursor	K.ADIQM*PFTCSVTYYGPSGQK.T	2	5.67	0.55	-4.13
IPI00015102	Isoform 1 of CD166 antigen precursor	K.ADIQM*PFTCSVTYYGPSGQK.T	3	3.62	0.22	-2.05
IPI00015102	Isoform 1 of CD166 antigen precursor	K.ALFLETEQLK.K	1	2.48	0.14	-3.45
IPI00015102	Isoform 1 of CD166 antigen precursor	K.ALFLETEQLK.K	2	3.81	0.19	-3.35
IPI00015102	Isoform 1 of CD166 antigen precursor	K.ALFLETEQLKK.L	2	2.75	0.21	-2.62
IPI00015102	Isoform 1 of CD166 antigen precursor	K.CLGNGNPPPEEFLFYLPGQPEGIR.S	2	4.41	0.51	-3.72
IPI00015102	Isoform 1 of CD166 antigen precursor	K.CLGNGNPPPEEFLFYLPGQPEGIR.S	3	4.51	0.38	-4.76
IPI00015102	Isoform 1 of CD166 antigen precursor	K.EM*DPVTQLYTM*TSTLEYK.T	2	5.29	0.54	-5.29
IPI00015102	Isoform 1 of CD166 antigen precursor	K.EM*DPVTQLYTM*TSTLEYK.T	3	4.36	0.54	-4.75
IPI00015102	Isoform 1 of CD166 antigen precursor	K.KSVQYDDVPEYKDR.L	2	4.91	0.50	-1.51
IPI00015102	Isoform 1 of CD166 antigen precursor	K.KSVQYDDVPEYKDR.L	3	3.58	0.32	-1.60
IPI00015102	Isoform 1 of CD166 antigen precursor	K.SM*IASTAITVHYLDLSLNPSGEVTR.Q	2	4.72	0.52	-2.02
IPI00015102	Isoform 1 of CD166 antigen precursor	K.SM*IASTAITVHYLDLSLNPSGEVTR.Q	3	5.96	0.38	-4.62
IPI00015102	Isoform 1 of CD166 antigen precursor	K.SVQYDDVPEYK.D	2	2.70	0.24	-2.81
IPI00015102	Isoform 1 of CD166 antigen precursor	K.SVQYDDVPEYKDR.L	2	2.14	0.19	-4.07
IPI00015102	Isoform 1 of CD166 antigen precursor	K.SVQYDDVPEYKDR.L	3	2.56	0.17	-1.15
IPI00015102	Isoform 1 of CD166 antigen precursor	K.TIHSEQAVFDIYYPTEQVTIQVLPPK.N	3	4.53	0.38	-4.31
IPI00015102	Isoform 1 of CD166 antigen precursor	K.VFKQPSKPEIVSK.A	3	2.75	0.23	-2.60
IPI00015102	Isoform 1 of CD166 antigen precursor	K.VLHPLEGAVVIIFK.K	2	2.17	0.26	-4.76
IPI00015102	Isoform 1 of CD166 antigen precursor	K.VLHPLEGAVVIIFKK.E	2	3.65	0.47	-3.95
IPI00015102	Isoform 1 of CD166 antigen precursor	K.VLHPLEGAVVIIFKK.E	3	4.39	0.50	-2.97
IPI00015102	Isoform 1 of CD166 antigen precursor	K.VLHPLEGAVVIIFKK.E	4	5.02	0.39	-4.59
IPI00015102	Isoform 1 of CD166 antigen precursor	K.WKYEKPDGSPVFIAFR.S	3	3.56	0.19	-2.80
IPI00015102	Isoform 1 of CD166 antigen precursor	K.YEKPDGSPVFIAFR.S	2	3.78	0.42	-2.87
IPI00015102	Isoform 1 of CD166 antigen precursor	K.YEKPDGSPVFIAFR.S	3	3.28	0.35	-2.21
IPI00015102	Isoform 1 of CD166 antigen precursor	R.ESLTLIVEGKPQIK.M	2	3.15	0.36	-3.48
IPI00015102	Isoform 1 of CD166 antigen precursor	R.ESLTLIVEGKPQIK.M	3	2.17	0.14	-2.06

IPI00015102	Isoform 1 of CD166 antigen precursor	R.FVCM*LVTEDNVFEAPTIVK.V	2	5.56	0.53	-4.82
IPI00015102	Isoform 1 of CD166 antigen precursor	R.FVCM*LVTEDNVFEAPTIVK.V	3	4.48	0.45	-4.08
IPI00015102	Isoform 1 of CD166 antigen precursor	R.LDVPQNLM*FGK.W	1	1.92	0.23	-3.92
IPI00015102	Isoform 1 of CD166 antigen precursor	R.LDVPQNLM*FGK.W	2	3.56	0.44	0.49
IPI00015102	Isoform 1 of CD166 antigen precursor	R.QIGDALPVSCTISASR.N	2	4.02	0.54	-3.18
IPI00015102	Isoform 1 of CD166 antigen precursor	R.SSNTYTLTDVR.R	1	2.14	0.14	-3.19
IPI00015102	Isoform 1 of CD166 antigen precursor	R.SSNTYTLTDVR.R	2	3.44	0.27	-2.42
IPI00015102	Isoform 1 of CD166 antigen precursor	R.SSNTYTLTDVRR.N	2	2.41	0.22	-3.37
IPI00015102	Isoform 1 of CD166 antigen precursor	R.SSNTYTLTDVRR.N	3	1.69	0.25	-3.29
IPI00015102	Isoform 1 of CD166 antigen precursor	R.SSPSFSSLHYQDAGNYVCETALQEVEGLK.K	2	3.90	0.59	-1.97
IPI00015102	Isoform 1 of CD166 antigen precursor	R.SSPSFSSLHYQDAGNYVCETALQEVEGLK.K	3	6.43	0.54	-4.21
IPI00015148	Ras-related protein Rap-1b precursor	K.SALTVQFVQGIFVEK.Y	2	3.76	0.33	-3.93
IPI00015260	Protein kinase C-binding protein NELL2 precursor	G.LGVDPSLQIDVLTELELGESTTGVR.Q	2	6.15	0.61	-4.83
IPI00015260	Protein kinase C-binding protein NELL2 precursor	G.LGVDPSLQIDVLTELELGESTTGVR.Q	3	4.83	0.49	-5.96
IPI00015260	Protein kinase C-binding protein NELL2 precursor	K.AFLFQDTPR.S	1	2.37	0.24	-3.30
IPI00015260	Protein kinase C-binding protein NELL2 precursor	K.AFLFQDTPR.S	2	3.30	0.35	-4.21
IPI00015260	Protein kinase C-binding protein NELL2 precursor	K.ASTATAEQFFQK.L	1	3.25	0.35	-2.48
IPI00015260	Protein kinase C-binding protein NELL2 precursor	K.ASTATAEQFFQK.L	2	4.00	0.37	-2.52
IPI00015260	Protein kinase C-binding protein NELL2 precursor	K.ASTATAEQFFQKLR.N	2	3.30	0.33	-3.76
IPI00015260	Protein kinase C-binding protein NELL2 precursor	K.GYDFCSER.H	1	1.35	0.06	-3.56
IPI00015260	Protein kinase C-binding protein NELL2 precursor	K.HGTECTLCQCK.N	2	3.76	0.55	-2.85
IPI00015260	Protein kinase C-binding protein NELL2 precursor	K.HNGQIWVLENDR.C	2	4.26	0.42	-6.43
IPI00015260	Protein kinase C-binding protein NELL2 precursor	K.IM*ELQDILAK.T	1	3.26	0.22	-3.08
IPI00015260	Protein kinase C-binding protein NELL2 precursor	K.IM*ELQDILAK.T	2	3.92	0.26	-3.98
IPI00015260	Protein kinase C-binding protein NELL2 precursor	K.LVESSGCPALDCPESHQITLSHSCCK.V	3	4.26	0.48	-3.37
IPI00015260	Protein kinase C-binding protein NELL2 precursor	K.NGHICCSVDPQCLQEL.-	2	5.00	0.54	-2.89
IPI00015260	Protein kinase C-binding protein NELL2 precursor	K.NGHICCSVDPQCLQEL.-	3	3.35	0.36	-2.26
IPI00015260	Protein kinase C-binding protein NELL2 precursor	K.SALAYVDGK.C	1	2.76	0.22	-3.72
IPI00015260	Protein kinase C-binding protein NELL2 precursor	K.SALAYVDGK.C	2	3.11	0.21	-3.47
IPI00015260	Protein kinase C-binding protein NELL2 precursor	K.SICQFQGR.T	2	2.67	0.23	-1.55
IPI00015260	Protein kinase C-binding protein NELL2 precursor	K.TCLDEM*NVVR.F	2	3.07	0.15	-2.45
IPI00015260	Protein kinase C-binding protein NELL2 precursor	N.NAHGYFK.G	1	2.06	0.19	-4.87
IPI00015260	Protein kinase C-binding protein NELL2 precursor	Q.IDVLTELELGESTTGVR.Q	3	4.35	0.49	-3.30
IPI00015260	Protein kinase C-binding protein NELL2 precursor	R.ALREDNAYCEDIDECAEGR.H	2	4.81	0.60	-4.36
IPI00015260	Protein kinase C-binding protein NELL2 precursor	R.ALREDNAYCEDIDECAEGR.H	3	5.61	0.45	-3.54
IPI00015260	Protein kinase C-binding protein NELL2 precursor	R.CVTDPCQADTIR.N	2	3.94	0.34	-4.69
IPI00015260	Protein kinase C-binding protein NELL2 precursor	R.CVTDPCQADTIRNDITK.T	2	3.47	0.37	-3.23
IPI00015260	Protein kinase C-binding protein NELL2 precursor	R.CVTDPCQADTIRNDITK.T	3	2.36	0.18	-2.94
IPI00015260	Protein kinase C-binding protein NELL2 precursor	R.ENTM*CVNTPGSFM*CICK.T	2	4.38	0.53	-4.15
IPI00015260	Protein kinase C-binding protein NELL2 precursor	R.FTGSSWIK.H	1	1.16	0.09	-2.86
IPI00015260	Protein kinase C-binding protein NELL2 precursor	R.FTGSSWIK.H	2	2.31	0.14	-1.84
IPI00015260	Protein kinase C-binding protein NELL2 precursor	R.HNCM*ENSICR.N	2	2.32	0.31	-4.30

IPI00015260	Protein kinase C-binding protein NELL2 precursor	R.LDQCYCER.T	2	3.30	0.25	-3.32
IPI00015260	Protein kinase C-binding protein NELL2 precursor	R.LSSQCLHQNGETLYNSGDTWVQNCQQCR.C	3	4.98	0.47	-0.81
IPI00015260	Protein kinase C-binding protein NELL2 precursor	R.M*VCDCENPTVDLFCCPECDPR.L	2	4.09	0.62	-3.05
IPI00015260	Protein kinase C-binding protein NELL2 precursor	R.M*VCDCENPTVDLFCCPECDPR.L	3	5.90	0.53	-3.82
IPI00015260	Protein kinase C-binding protein NELL2 precursor	R.NNAHGYFK.G	1	2.41	0.12	-4.26
IPI00015260	Protein kinase C-binding protein NELL2 precursor	R.NNAHGYFK.G	2	2.24	0.11	-3.64
IPI00015260	Protein kinase C-binding protein NELL2 precursor	R.NTVYSSSGVCVLYECK.D	2	5.60	0.56	-2.87
IPI00015260	Protein kinase C-binding protein NELL2 precursor	R.NTVYSSSGVCVLYECKDQTM*K.L	2	3.79	0.42	-2.05
IPI00015260	Protein kinase C-binding protein NELL2 precursor	R.SIKASTATAEQFFQK.L	2	4.16	0.40	-4.43
IPI00015260	Protein kinase C-binding protein NELL2 precursor	R.SIKASTATAEQFFQK.L	3	3.76	0.11	-3.97
IPI00015260	Protein kinase C-binding protein NELL2 precursor	R.TCPTCNDFHGLVQK.I	2	4.24	0.41	-4.09
IPI00015260	Protein kinase C-binding protein NELL2 precursor	R.TYFEGER.N	1	2.14	0.14	-1.49
IPI00015260	Protein kinase C-binding protein NELL2 precursor	R.TYFEGER.N	2	2.45	0.13	-1.87
IPI00015260	Protein kinase C-binding protein NELL2 precursor	R.VVEKPSTDLPLGTTF.W	2	3.39	0.43	-2.48
IPI00015260	Protein kinase C-binding protein NELL2 precursor	R.VVEKPSTDLPLGTTFWLGQR.N	2	5.25	0.53	-2.85
IPI00015260	Protein kinase C-binding protein NELL2 precursor	R.VVEKPSTDLPLGTTFWLGQR.N	3	4.55	0.43	-4.17
IPI00015260	Protein kinase C-binding protein NELL2 precursor	R.VVEKPSTDLPLGTTFWLGQR.N	4	3.25	0.17	-2.00
IPI00015260	Protein kinase C-binding protein NELL2 precursor	R.YLELESSGHR.N	1	2.27	0.33	-3.69
IPI00015260	Protein kinase C-binding protein NELL2 precursor	R.YLELESSGHR.N	2	2.98	0.30	-2.97
IPI00015260	Protein kinase C-binding protein NELL2 precursor	R.YLELESSGHRNEVR.L	2	3.55	0.32	-4.38
IPI00015260	Protein kinase C-binding protein NELL2 precursor	R.YLELESSGHRNEVR.L	3	2.10	0.26	-2.55
IPI00015260	Protein kinase C-binding protein NELL2 precursor	R.YLELESSGHRNEVR.L	4	2.20	0.12	-3.20
IPI00015285	Ethanolamine-phosphate cytidylyltransferase	R.M*LLVTKAHHSSQEMSSEYREYADSFGK.C	3	2.42	0.05	0.94
IPI00015315	Extracellular matrix protein 2 precursor	K.LYHVPSYLPK.S	3	3.04	0.26	-3.18
IPI00015315	Extracellular matrix protein 2 precursor	K.VNENNLQAIDEESLSDLNQLVTLELEGNNLSEANVNPLAFKPLK.S	3	4.40	0.37	-3.13
IPI00015315	Extracellular matrix protein 2 precursor	K.VNENNLQAIDEESLSDLNQLVTLELEGNNLSEANVNPLAFKPLK.S	4	6.39	0.57	-5.44
IPI00015315	Extracellular matrix protein 2 precursor	R.ELFLDHNDLK.S	1	2.81	0.27	-1.10
IPI00015315	Extracellular matrix protein 2 precursor	R.IAPLAWINQENLESIDLSYNK.L	2	5.23	0.49	-5.22
IPI00015315	Extracellular matrix protein 2 precursor	R.IAPLAWINQENLESIDLSYNK.L	3	6.38	0.48	-5.11
IPI00015315	Extracellular matrix protein 2 precursor	R.IPGYVFGHM*EPGLEYLYLSFNK.L	3	5.07	0.47	-4.51
IPI00015315	Extracellular matrix protein 2 precursor	R.LPSGCSLSYR.T	2	2.62	0.35	0.52
IPI00015315	Extracellular matrix protein 2 precursor	R.NILPEEICNAEEDDDSNLEHLHLENNYIK.I	3	3.34	0.39	-2.41
IPI00015315	Extracellular matrix protein 2 precursor	R.NQGQLYSEGDSR.G	2	4.09	0.41	-2.74
IPI00015315	Extracellular matrix protein 2 precursor	R.SYSSIVLKPQNIK.-	2	4.64	0.45	-3.16
IPI00015315	Extracellular matrix protein 2 precursor	R.SYSSIVLKPQNIK.-	3	1.80	0.24	-1.45
IPI00015315	Extracellular matrix protein 2 precursor	R.VSFYGAYHSLR.E	3	2.44	0.13	-1.52
IPI00015315	Extracellular matrix protein 2 precursor	R.YNKIEENR.I	2	2.86	0.21	-0.39
IPI00015346	Cadherin EGF LAG seven-pass G-type receptor 2 precursor	A.LRGEQPPDLETTVILPESVFR.E	2	4.34	0.53	-0.57
IPI00015346	Cadherin EGF LAG seven-pass G-type receptor 2 precursor	A.LRGEQPPDLETTVILPESVFR.E	3	4.11	0.24	-3.93

IPI00015346	Cadherin EGF LAG seven-pass G-type receptor 2 precursor	L.RGEQPPDLETTVILPESVFR.E	2	3.33	0.35	-2.00
IPI00015346	Cadherin EGF LAG seven-pass G-type receptor 2 precursor	R.DAGTELTGHLVPHHDGLR.V	2	2.59	0.12	
IPI00015346	Cadherin EGF LAG seven-pass G-type receptor 2 precursor	R.DAGTELTGHLVPHHDGLR.V	3	3.07	0.28	-0.50
IPI00015346	Cadherin EGF LAG seven-pass G-type receptor 2 precursor	R.ETPPVVRPAGPGEAQEPEE.L	2	4.19	0.42	-2.42
IPI00015346	Cadherin EGF LAG seven-pass G-type receptor 2 precursor	R.ETPPVVRPAGPGEAQEPEELAR.R	3	2.91	0.19	-1.38
IPI00015346	Cadherin EGF LAG seven-pass G-type receptor 2 precursor	R.LLGIGGHLSPQGK.L	2	3.49	0.43	-3.76
IPI00015346	Cadherin EGF LAG seven-pass G-type receptor 2 precursor	R.SNQFFSLDPVTGAVTTAEELDRETK.S	3	3.73	0.29	-5.34
IPI00015346	Cadherin EGF LAG seven-pass G-type receptor 2 precursor	R.VTAQDHGM*PR.R	2	2.12	0.07	-3.06
IPI00015351	Isoform 1 of UPF0424 protein C1orf128	K.FVESDADEELLFNIPFTGNVK.L	2	4.04	0.31	-3.51
IPI00015351	Isoform 1 of UPF0424 protein C1orf128	K.FVESDADEELLFNIPFTGNVK.L	3	5.32	0.31	-3.52
IPI00015351	Isoform 1 of UPF0424 protein C1orf128	K.NFGADTTK.V	2	1.95	0.09	-2.48
IPI00015351	Isoform 1 of UPF0424 protein C1orf128	K.VFYIGLR.G	2	2.13	0.07	-2.62
IPI00015351	Isoform 1 of UPF0424 protein C1orf128	R.DLTGELEYATK.I	2	3.96	0.40	-3.21
IPI00015351	Isoform 1 of UPF0424 protein C1orf128	R.GLAYGLYLR.I	2	3.45	0.27	-2.91
IPI00015479	UPF0454 protein C12orf49 precursor	R.AAVAFQNLFM*AVEDHFELCLAK.C	3	2.73	0.19	-2.42
IPI00015479	UPF0454 protein C12orf49 precursor	R.TSSQSVQHENTYRDPIAK.Y	2	3.17	0.42	-3.11
IPI00015479	UPF0454 protein C12orf49 precursor	R.TSSQSVQHENTYRDPIAK.Y	3	2.48	0.32	-3.64
IPI00015522	Growth/differentiation factor 5 precursor	K.GQLPGGKAPPKAGSVPSSFLLKKAR.E	3	3.46	0.13	
IPI00015525	Multimerin-2 precursor	K.EAEPLVDIR.V	1	1.87	0.16	-2.77
IPI00015525	Multimerin-2 precursor	K.EAEPLVDIR.V	2	2.49	0.19	-1.75
IPI00015525	Multimerin-2 precursor	R.SLSGTAFGGFLM*FK.T	2	3.93	0.41	-4.44
IPI00015525	Multimerin-2 precursor	R.SLSGTAFGGFLM*FKT.-	2	2.61	0.24	-5.19
IPI00015525	Multimerin-2 precursor	R.TPVCTTGQGSGSTATVFAM*AELQK.G	3	4.13	0.37	-1.99
IPI00015688	Glypican-1 precursor	K.GFSLSDVPQAEISGEHLR.I	2	4.67	0.44	-2.42
IPI00015688	Glypican-1 precursor	K.GFSLSDVPQAEISGEHLR.I	3	2.74	0.28	-1.54
IPI00015688	Glypican-1 precursor	K.M*ALSTASDDR.C	2	3.32	0.36	-1.75
IPI00015688	Glypican-1 precursor	K.VNPQGPGPEEK.R	2	2.35	0.13	-2.25
IPI00015688	Glypican-1 precursor	K.VNPQGPGPEEKR.R	2	2.41	0.21	-3.90
IPI00015688	Glypican-1 precursor	R.AFRDLYSELR.L	2	2.44	0.10	-3.44
IPI00015688	Glypican-1 precursor	R.DVQDFWISLPGTLCSEK.M	2	3.58	0.33	-6.12
IPI00015688	Glypican-1 precursor	R.GANLHLEETLAEFWAR.L	3	4.13	0.17	-4.38
IPI00015688	Glypican-1 precursor	R.GRYLPEVM*GDGLAN.Q	2	3.14	0.34	-1.75
IPI00015688	Glypican-1 precursor	R.GRYLPEVM*GDGLANQINNPEVEVDITKPDM*TIR.Q	4	3.59	0.14	-2.51
IPI00015688	Glypican-1 precursor	R.SFVQGLGVASDVVR.K	2	5.31	0.40	-4.07

IPI00015688	Glypican-1 precursor	R.SFVQGLGVASDVVRK.V	2	4.01	0.39	-3.10
IPI00015688	Glypican-1 precursor	R.SHAELETALR.D	2	3.26	0.37	-1.56
IPI00015688	Glypican-1 precursor	R.SHAELETALRDSSR.V	2	3.35	0.40	-3.06
IPI00015688	Glypican-1 precursor	R.TLQATFPGAFGELYTQNAR.A	2	6.04	0.56	-4.40
IPI00015688	Glypican-1 precursor	R.TLQATFPGAFGELYTQNAR.A	3	4.92	0.44	-4.64
IPI00015688	Glypican-1 precursor	R.TPLTHALPGLSEQEGQK.T	2	4.19	0.52	-2.58
IPI00015688	Glypican-1 precursor	R.TPLTHALPGLSEQEGQK.T	3	3.79	0.22	-1.98
IPI00015688	Glypican-1 precursor	R.VLQAM*LATQLR.S	2	3.67	0.41	-2.61
IPI00015756	Isoform 1 of Receptor-type tyrosine-protein phosphatase kappa precursor	K.GAPISAYQIVVEELHPHRT.K	2	4.45	0.47	-2.81
IPI00015756	Isoform 1 of Receptor-type tyrosine-protein phosphatase kappa precursor	K.GLNPGTLNILVR.V	2	2.60	0.28	-0.59
IPI00015756	Isoform 1 of Receptor-type tyrosine-protein phosphatase kappa precursor	K.IFLNWKEPLDPNGIITQYEISYSSIR.S	3	5.34	0.51	-2.52
IPI00015756	Isoform 1 of Receptor-type tyrosine-protein phosphatase kappa precursor	K.TDQDLYR.C	2	2.02	0.16	-1.79
IPI00015756	Isoform 1 of Receptor-type tyrosine-protein phosphatase kappa precursor	R.GSGVSNFAQLIVR.E	2	3.67	0.24	-3.50
IPI00015756	Isoform 1 of Receptor-type tyrosine-protein phosphatase kappa precursor	R.KESEETIIQTDEDVPGPVPVK.S	3	4.16	0.39	-2.36
IPI00015756	Isoform 1 of Receptor-type tyrosine-protein phosphatase kappa precursor	R.LQEVTKTDQDLYR.C	3	3.49	0.30	-1.33
IPI00015756	Isoform 1 of Receptor-type tyrosine-protein phosphatase kappa precursor	R.NGEDIPVAQTK.N	2	3.54	0.31	-3.84
IPI00015756	Isoform 1 of Receptor-type tyrosine-protein phosphatase kappa precursor	R.VLLTRPGEGGTGLPGPPLITR.T	2	3.98	0.39	-2.63
IPI00015756	Isoform 1 of Receptor-type tyrosine-protein phosphatase kappa precursor	R.VLLTRPGEGGTGLPGPPLITR.T	3	3.07	0.29	-1.78
IPI00015842	Reticulocalbin-1 precursor	K.IVDRIDNDGDGFVTTEELK.T	2	4.70	0.47	-3.03
IPI00015842	Reticulocalbin-1 precursor	K.IVDRIDNDGDGFVTTEELK.T	3	3.49	0.30	-3.86
IPI00015842	Reticulocalbin-1 precursor	K.TFDQLTPDESKER.L	3	2.09	0.11	-0.37
IPI00015842	Reticulocalbin-1 precursor	R.IDNDGDGFVTTEELK.T	2	2.74	0.36	-3.20
IPI00015881	Isoform 1 of Macrophage colony-stimulating factor 1 precursor	K.AFLLVQDIM*EDTM*R.F	2	4.58	0.52	-2.66
IPI00015881	Isoform 1 of Macrophage colony-stimulating factor 1 precursor	P.RFNSVPLTDTGHER.Q	2	3.63	0.29	-4.21
IPI00015881	Isoform 1 of Macrophage colony-stimulating factor 1 precursor	P.RFNSVPLTDTGHER.Q	3	3.51	0.29	-2.76
IPI00015881	Isoform 1 of Macrophage colony-stimulating factor 1 precursor	R.DNTPNAIAIVQLQELSLR.L	3	4.41	0.44	-3.40
IPI00015881	Isoform 1 of Macrophage colony-stimulating factor 1 precursor	R.DPPEPGSPR.I	2	2.18	0.06	-1.23

IPI00015881	Isoform 1 of Macrophage colony-stimulating factor 1 precursor	R.FNSVPLTDTGHER.Q	2	3.88	0.39	-3.79
IPI00015881	Isoform 1 of Macrophage colony-stimulating factor 1 precursor	R.FNSVPLTDTGHER.Q	3	2.09	0.23	-1.32
IPI00015881	Isoform 1 of Macrophage colony-stimulating factor 1 precursor	R.FRDNTPNAIAIVQLQELSLR.L	2	5.49	0.51	-5.09
IPI00015881	Isoform 1 of Macrophage colony-stimulating factor 1 precursor	R.FRDNTPNAIAIVQLQELSLR.L	3	4.57	0.33	-2.01
IPI00015881	Isoform 1 of Macrophage colony-stimulating factor 1 precursor	R.ISSLRPQGLSNPSTLSAQPQLSR.S	3	5.28	0.47	-2.55
IPI00015881	Isoform 1 of Macrophage colony-stimulating factor 1 precursor	R.QSEGSSSPQLQESVFHLLVPSVI.L	2	3.29	0.34	-5.20
IPI00015881	Isoform 1 of Macrophage colony-stimulating factor 1 precursor	R.RSPAEPEGGPASEGAARPLPR.F	3	4.14	0.29	-3.50
IPI00015881	Isoform 1 of Macrophage colony-stimulating factor 1 precursor	R.SHSSGSVLPLGELEGR.R	2	4.58	0.51	-4.05
IPI00015881	Isoform 1 of Macrophage colony-stimulating factor 1 precursor	R.SHSSGSVLPLGELEGR.R	3	3.17	0.30	-3.65
IPI00015881	Isoform 1 of Macrophage colony-stimulating factor 1 precursor	R.SPAEPEGGPASEGAARPLPR.F	2	3.48	0.24	-2.28
IPI00015881	Isoform 1 of Macrophage colony-stimulating factor 1 precursor	R.SPAEPEGGPASEGAARPLPR.F	3	2.47	0.29	-1.62
IPI00015881	Isoform 1 of Macrophage colony-stimulating factor 1 precursor	R.TFYETPLQLLEK.V	2	3.89	0.35	-4.27
IPI00015902	Beta-type platelet-derived growth factor receptor precursor	K.KGDVALPVPYDHQR.G	2	3.67	0.44	-2.02
IPI00015902	Beta-type platelet-derived growth factor receptor precursor	K.TTIGDREVDSDAYYVYR.L	2	3.60	0.36	-3.84
IPI00015902	Beta-type platelet-derived growth factor receptor precursor	K.TTIGDREVDSDAYYVYR.L	3	4.42	0.52	-2.73
IPI00015902	Beta-type platelet-derived growth factor receptor precursor	R.EVDSDAYYVYR.L	2	3.59	0.43	-3.62
IPI00015902	Beta-type platelet-derived growth factor receptor precursor	R.GFSGIFEDR.S	1	2.02	0.18	-3.24
IPI00015902	Beta-type platelet-derived growth factor receptor precursor	R.GFSGIFEDR.S	2	3.03	0.30	-2.28
IPI00015902	Beta-type platelet-derived growth factor receptor precursor	R.LVEPVTDFLLDM*PYHIR.S	3	2.33	0.17	-2.11
IPI00015902	Beta-type platelet-derived growth factor receptor precursor	R.M*SQEPPQEM*AK.A	2	3.33	0.34	-2.27
IPI00015902	Beta-type platelet-derived growth factor receptor precursor	R.TLGDSSAGEIALSTR.N	2	4.52	0.52	-4.27

IPI00015902	Beta-type platelet-derived growth factor receptor precursor	R.VLELSESHPDSGEQTVR.C	2	4.64	0.51	-1.59
IPI00015902	Beta-type platelet-derived growth factor receptor precursor	R.VLELSESHPDSGEQTVR.C	3	2.56	0.08	-1.21
IPI00015902	Beta-type platelet-derived growth factor receptor precursor	R.VTDPQLVVTLHEK.K	2	3.65	0.28	-3.05
IPI00015902	Beta-type platelet-derived growth factor receptor precursor	R.VTDPQLVVTLHEK.K	3	2.14	0.19	-1.48
IPI00015911	Dihydrolipoyl dehydrogenase, mitochondrial precursor	K.ADGGTQVIDTK.N	2	3.46	0.32	-2.87
IPI00015911	Dihydrolipoyl dehydrogenase, mitochondrial precursor	K.ALTGGIAHLFK.Q	2	3.37	0.44	-3.33
IPI00015911	Dihydrolipoyl dehydrogenase, mitochondrial precursor	K.IPNIYAIGDVVAGPM*LAHK.A	3	4.22	0.35	-2.14
IPI00015911	Dihydrolipoyl dehydrogenase, mitochondrial precursor	K.TNADTDGM*VK.I	2	1.98	0.08	-2.57
IPI00015911	Dihydrolipoyl dehydrogenase, mitochondrial precursor	R.VLGAHILGPGAGEM*VNEAALALEYGASCEDIAR.V	3	4.25	0.41	-1.43
IPI00015911	Dihydrolipoyl dehydrogenase, mitochondrial precursor	Y.ADQPIDADVTVIGSGPGGYVAAIK.A	2	5.04	0.54	-3.57
IPI00015911	Dihydrolipoyl dehydrogenase, mitochondrial precursor	Y.ADQPIDADVTVIGSGPGGYVAAIK.A	3	5.47	0.43	-4.50
IPI00015913	5,6-dihydroxyindole-2-carboxylic acid oxidase precursor	R.NTVEGYSDPTGK.Y	2	2.62	0.16	-2.77
IPI00015954	GTP-binding protein SAR1a	R.LGQHVPTLHPTSEELTIAGMTFTTFDLGGHEQAR.R	4	3.81	0.18	-2.35
IPI00015964	Neuromodulin	G.EGDAATEQAAPQAPASSEEK.A	2	4.26	0.54	-2.91
IPI00015964	Neuromodulin	K.ASTDNSPSSKAEDAPAKEEPK.Q	3	3.54	0.32	-3.11
IPI00015964	Neuromodulin	K.ATAQPPTETGESSQAEENIEAVDETKPK.E	3	2.70	0.41	-3.39
IPI00015964	Neuromodulin	K.GEGDAATEQAAPQAPASSEEK.A	2	5.71	0.58	-4.28
IPI00015964	Neuromodulin	K.GEGDAATEQAAPQAPASSEEK.A	3	3.58	0.24	-3.28
IPI00015964	Neuromodulin	K.GEGTTTAEAAPATGSKPDEPGK.A	3	4.47	0.46	-3.80
IPI00015964	Neuromodulin	K.KGEGDAATEQAAPQAPASSEEK.A	3	3.59	0.29	-2.60
IPI00015964	Neuromodulin	K.KGEGTTTAEAAPATGSKPDEPGK.A	3	5.35	0.49	-2.75
IPI00015964	Neuromodulin	K.KGEGTTTAEAAPATGSKPDEPGKAGETPSEEK.K	4	4.91	0.42	-3.61
IPI00015964	Neuromodulin	K.QADVPAAVTAAAATTPAAEDAAAK.A	2	6.05	0.65	-0.39
IPI00015964	Neuromodulin	K.QADVPAAVTAAAATTPAAEDAAAK.A	3	3.57	0.27	-3.47
IPI00015964	Neuromodulin	T.AAAATTPAAEDAAAK.A	2	3.65	0.38	-4.78
IPI00015973	Band 4.1-like protein 2	K.DSSQLGTDATK.E	2	2.94	0.29	-3.14
IPI00015980	Isoform 2 of Multiple PDZ domain protein	R.GPDGLGFSIVGGYGSPHGDLPIYVKTVFAK.G	3	3.67	0.05	
IPI00015983	Sphingosine 1-phosphate receptor Edg-3	R.GRGARASPIQPALDPSRSK.S	3	2.63	0.10	-0.55
IPI00015988	HLA class I histocompatibility antigen, alpha chain G precursor	K.WAAVVVPSGEEQR.Y	2	3.88	0.35	-3.20

IPI00016014	Isoform 1 of Integral membrane protein 2C	K.ISFQPAVAGIK.G	2	2.57	0.21	-2.49
IPI00016014	Isoform 1 of Integral membrane protein 2C	M.VKISFQPAVAGIK.G	2	3.59	0.19	-1.24
IPI00016112	peroxidasin homolog	K.KLESRLSTTECVDAGGESHANNTKWK.K	3	3.07	0.10	-6.49
IPI00016150	Neuroserpin precursor	A.TFPEEAIADLSVNM*YNR.L	2	5.16	0.53	-3.20
IPI00016150	Neuroserpin precursor	A.TFPEEAIADLSVNM*YNR.L	3	3.74	0.42	-2.62
IPI00016150	Neuroserpin precursor	G.ATFPEEAIADLSVNM*YNR.L	2	4.35	0.34	-2.88
IPI00016150	Neuroserpin precursor	G.ATFPEEAIADLSVNM*YNR.L	3	5.03	0.33	-4.64
IPI00016150	Neuroserpin precursor	K.ALGITEIFIK.D	2	3.38	0.36	-2.75
IPI00016150	Neuroserpin precursor	K.ALGITEIFIKDANLTGLSDNKEIFLSK.A	3	3.21	0.07	-2.23
IPI00016150	Neuroserpin precursor	K.AQLVEEWANSVK.K	2	4.10	0.42	-1.34
IPI00016150	Neuroserpin precursor	K.AQLVEEWANSVKK.Q	2	3.79	0.34	-0.69
IPI00016150	Neuroserpin precursor	K.DANLTGLSDNKEIFLSK.A	3	3.61	0.39	-2.09
IPI00016150	Neuroserpin precursor	K.EFSNM*VTAK.E	1	1.79	0.11	-2.70
IPI00016150	Neuroserpin precursor	K.EFSNM*VTAK.E	2	2.12	0.31	-2.24
IPI00016150	Neuroserpin precursor	K.ESQYVM*K.I	1	2.30	0.11	-1.89
IPI00016150	Neuroserpin precursor	K.IANSLFVQNGFHVNEEFLQM*M*K.K	3	4.40	0.47	-3.41
IPI00016150	Neuroserpin precursor	K.KYFNAAVNHVDFSQNVAVANYINK.W	4	3.03	0.06	-4.67
IPI00016150	Neuroserpin precursor	K.NGEEFSFLK.E	1	2.28	0.16	-2.99
IPI00016150	Neuroserpin precursor	K.NGEEFSFLK.E	2	2.93	0.25	-1.80
IPI00016150	Neuroserpin precursor	K.NGEEFSFLKEFSNM*VTAK.E	2	3.51	0.37	-2.45
IPI00016150	Neuroserpin precursor	K.NGEEFSFLKEFSNM*VTAK.E	3	3.62	0.33	-3.17
IPI00016150	Neuroserpin precursor	K.QKVEVYLPR.F	2	1.90	0.35	0.64
IPI00016150	Neuroserpin precursor	K.SQFRPENTR.T	2	1.59	0.10	-2.59
IPI00016150	Neuroserpin precursor	K.VEVYLPR.F	1	1.72	0.19	-1.98
IPI00016150	Neuroserpin precursor	R.ATGEDENILFSPLSIALAM*GM*M*ELGAQGSTQK.E	3	5.35	0.42	-2.79
IPI00016150	Neuroserpin precursor	R.DFDAATYLALINAVYFK.G	2	4.57	0.44	-8.09
IPI00016150	Neuroserpin precursor	R.DFDAATYLALINAVYFK.G	3	4.88	0.40	-2.29
IPI00016150	Neuroserpin precursor	R.FTVEQEIDLKDVLK.A	2	4.77	0.40	-5.43
IPI00016150	Neuroserpin precursor	R.FTVEQEIDLKDVLK.A	3	3.39	0.37	-3.26
IPI00016150	Neuroserpin precursor	R.HSM*GYDSLK.N	2	2.11	0.19	-1.32
IPI00016150	Neuroserpin precursor	R.HSM*GYDSLKNGEEFSFLK.E	3	3.29	0.23	-1.73
IPI00016150	Neuroserpin precursor	R.HSM*GYDSLKNGEEFSFLKEFSNM*VTAK.E	3	2.83	0.12	-3.41
IPI00016150	Neuroserpin precursor	R.HSM*GYDSLKNGEEFSFLKEFSNM*VTAK.E	4	2.63	0.16	-3.20
IPI00016150	Neuroserpin precursor	R.HSM*GYDSLKNGEEFSFLKEFSNM*VTAK.E	5	3.80	0.31	-4.24
IPI00016150	Neuroserpin precursor	R.LRATGEDENILFSPLSIALAM*GM*M*ELGAQGSTQK.E	3	7.30	0.61	-3.65
IPI00016150	Neuroserpin precursor	R.QEVPLATLEPLVK.A	2	3.02	0.34	-2.27
IPI00016150	Neuroserpin precursor	R.TGTILFM*GR.V	2	2.90	0.30	-1.78
IPI00016150	Neuroserpin precursor	R.VM*HPETM*NTSGHDFEEL.-	2	3.77	0.60	-2.11
IPI00016334	Isoform 1 of Cell surface glycoprotein MUC18 precursor	G.VPGEAEQPAPELVEVEVGSTALLK.C	2	4.80	0.52	-3.95
IPI00016334	Isoform 1 of Cell surface glycoprotein MUC18 precursor	G.VPGEAEQPAPELVEVEVGSTALLK.C	3	5.22	0.33	-6.16

IPI00016334	Isoform 1 of Cell surface glycoprotein MUC18 precursor	K.APEEPNIQVNPLGIPVNSKEPEEVATCVGR.N	3	6.07	0.47	-3.53
IPI00016334	Isoform 1 of Cell surface glycoprotein MUC18 precursor	K.AQLVKEDKDAQFYCELNYR.L	2	5.95	0.58	-3.81
IPI00016334	Isoform 1 of Cell surface glycoprotein MUC18 precursor	K.AQLVKEDKDAQFYCELNYR.L	3	5.60	0.47	-3.16
IPI00016334	Isoform 1 of Cell surface glycoprotein MUC18 precursor	K.AQLVKEDKDAQFYCELNYR.L	4	3.66	0.32	-1.51
IPI00016334	Isoform 1 of Cell surface glycoprotein MUC18 precursor	K.DAQFYCELNYR.L	2	3.09	0.27	-2.37
IPI00016334	Isoform 1 of Cell surface glycoprotein MUC18 precursor	K.EDKDAQFYCELNYR.L	2	3.75	0.51	-4.18
IPI00016334	Isoform 1 of Cell surface glycoprotein MUC18 precursor	K.EDKDAQFYCELNYR.L	3	4.32	0.34	-3.16
IPI00016334	Isoform 1 of Cell surface glycoprotein MUC18 precursor	K.EGDRVEIR.C	1	1.31	0.12	-4.10
IPI00016334	Isoform 1 of Cell surface glycoprotein MUC18 precursor	K.EPEEVATCVGR.N	2	2.52	0.37	-3.38
IPI00016334	Isoform 1 of Cell surface glycoprotein MUC18 precursor	K.VWLEVEPVGM*LK.E	2	4.19	0.30	-4.45
IPI00016334	Isoform 1 of Cell surface glycoprotein MUC18 precursor	P.GEAEQPAPELVEVEVGSTALLK.C	2	5.12	0.59	-4.29
IPI00016334	Isoform 1 of Cell surface glycoprotein MUC18 precursor	P.GEAEQPAPELVEVEVGSTALLK.C	3	4.96	0.44	-4.83
IPI00016334	Isoform 1 of Cell surface glycoprotein MUC18 precursor	R.EAEEETTNDNGVLVLEPAR.K	2	4.77	0.40	-3.65
IPI00016334	Isoform 1 of Cell surface glycoprotein MUC18 precursor	R.EAEEETTNDNGVLVLEPAR.K	3	3.75	0.17	-3.63
IPI00016334	Isoform 1 of Cell surface glycoprotein MUC18 precursor	R.EAEEETTNDNGVLVLEPARK.E	2	4.24	0.39	-3.12
IPI00016334	Isoform 1 of Cell surface glycoprotein MUC18 precursor	R.EAEEETTNDNGVLVLEPARK.E	3	3.42	0.43	-1.14
IPI00016334	Isoform 1 of Cell surface glycoprotein MUC18 precursor	R.EETGQVLER.G	1	2.12	0.14	-3.25
IPI00016334	Isoform 1 of Cell surface glycoprotein MUC18 precursor	R.EETGQVLER.G	2	2.38	0.13	-2.56
IPI00016334	Isoform 1 of Cell surface glycoprotein MUC18 precursor	R.EETGQVLERGPVLQLHDLKR.E	3	3.09	0.23	-0.84
IPI00016334	Isoform 1 of Cell surface glycoprotein MUC18 precursor	R.EVTVPVFYPTEK.V	1	2.36	0.28	-3.13
IPI00016334	Isoform 1 of Cell surface glycoprotein MUC18 precursor	R.EVTVPVFYPTEK.V	2	3.29	0.38	-4.62

IPI00016334	Isoform 1 of Cell surface glycoprotein MUC18 precursor	R.GATLALTQVTPQDER.I	2	4.67	0.43	-3.78
IPI00016334	Isoform 1 of Cell surface glycoprotein MUC18 precursor	R.GATLALTQVTPQDER.I	3	3.02	0.18	-2.21
IPI00016334	Isoform 1 of Cell surface glycoprotein MUC18 precursor	R.GPVLQLHDLK.R	2	3.09	0.22	-2.17
IPI00016334	Isoform 1 of Cell surface glycoprotein MUC18 precursor	R.GPVLQLHDLKR.E	2	3.67	0.40	-4.02
IPI00016334	Isoform 1 of Cell surface glycoprotein MUC18 precursor	R.IFLCQGK.R	1	1.89	0.11	-2.11
IPI00016334	Isoform 1 of Cell surface glycoprotein MUC18 precursor	R.IFLCQGK.R	2	1.93	0.13	-1.83
IPI00016334	Isoform 1 of Cell surface glycoprotein MUC18 precursor	R.LSLQDRGATLALTQVTPQDER.I	3	4.32	0.22	-1.77
IPI00016334	Isoform 1 of Cell surface glycoprotein MUC18 precursor	R.QEGSSLTLTCEAESSQDLEFQWLREETGQVLER.G	3	4.89	0.54	-2.86
IPI00016334	Isoform 1 of Cell surface glycoprotein MUC18 precursor	R.QGQGQSEPGEYEQR.L	2	3.89	0.45	-3.73
IPI00016334	Isoform 1 of Cell surface glycoprotein MUC18 precursor	R.QGQGQSEPGEYEQR.L	3	2.79	0.20	-2.15
IPI00016334	Isoform 1 of Cell surface glycoprotein MUC18 precursor	R.TQLVNVAIFGPPWM*AFK.E	2	6.03	0.51	-4.04
IPI00016334	Isoform 1 of Cell surface glycoprotein MUC18 precursor	R.TQLVNVAIFGPPWM*AFK.E	3	3.91	0.27	-2.99
IPI00016334	Isoform 1 of Cell surface glycoprotein MUC18 precursor	R.VHIQSSQTVESSGLYTLQSILK.A	2	6.91	0.63	-4.86
IPI00016334	Isoform 1 of Cell surface glycoprotein MUC18 precursor	R.VHIQSSQTVESSGLYTLQSILK.A	3	5.49	0.49	-6.82
IPI00016334	Isoform 1 of Cell surface glycoprotein MUC18 precursor	R.VLSTLNVLVTPELLETGVECTASNDLGK.N	3	3.30	0.25	-1.67
IPI00016334	Isoform 1 of Cell surface glycoprotein MUC18 precursor	R.VRQGQGQSEPGEYEQR.L	2	5.07	0.48	-4.27
IPI00016334	Isoform 1 of Cell surface glycoprotein MUC18 precursor	R.VRQGQGQSEPGEYEQR.L	3	4.51	0.43	-3.67
IPI00016334	Isoform 1 of Cell surface glycoprotein MUC18 precursor	R.VSPAAPERQEGSSLTLTCEAESSQDLEFQWLREETGQVLER.G	4	4.98	0.43	0.69
IPI00016334	Isoform 1 of Cell surface glycoprotein MUC18 precursor	R.VYKAPEEPNIQVNPLGIPVNSK.E	3	4.74	0.41	-3.07
IPI00016334	Isoform 1 of Cell surface glycoprotein MUC18 precursor	R.VYKAPEEPNIQVNPLGIPVNSKEPEEV.A	3	5.49	0.46	-2.01
IPI00016371	Isoform JM-A of Receptor tyrosine-protein kinase erbB-4 precursor	K.M*EVEENGIK.M	2	2.17	0.09	-2.59

IPI00016371	Isoform JM-A of Receptor tyrosine-protein kinase erbB-4 precursor	R.EVTGYVLVALNQFR.Y	2	3.17	0.27	-3.43
IPI00016422	Netrin receptor DCC precursor	K.AFNNAGEGVPLYESATTR.S	2	5.44	0.52	-3.86
IPI00016422	Netrin receptor DCC precursor	K.DGIHLALGM*DER.K	2	3.11	0.34	-3.32
IPI00016422	Netrin receptor DCC precursor	K.DGIHLALGM*DER.K	3	3.70	0.29	-2.64
IPI00016422	Netrin receptor DCC precursor	K.FTEYSLR.F	2	1.86	0.09	0.73
IPI00016422	Netrin receptor DCC precursor	K.GNIQTFTVFFSR.E	2	3.70	0.39	-2.51
IPI00016422	Netrin receptor DCC precursor	K.GVGPLSDPILFR.T	2	2.33	0.33	-3.68
IPI00016422	Netrin receptor DCC precursor	K.KFTEYSLR.F	2	2.44	0.06	-2.70
IPI00016422	Netrin receptor DCC precursor	K.NGDVVIPSDYFQIVGGSNLR.I	2	4.85	0.51	-2.64
IPI00016422	Netrin receptor DCC precursor	K.NGDVVIPSDYFQIVGGSNLR.I	3	4.17	0.42	-3.00
IPI00016422	Netrin receptor DCC precursor	K.VSWLPPPSGTQNGFITGYK.I	2	3.03	0.34	-3.95
IPI00016422	Netrin receptor DCC precursor	R.DVVPVLVSSR.F	1	2.08	0.21	-2.84
IPI00016422	Netrin receptor DCC precursor	R.DVVPVLVSSR.F	2	2.21	0.16	-2.66
IPI00016422	Netrin receptor DCC precursor	R.FLSEPSDAVTM*R.G	2	3.20	0.41	-3.30
IPI00016422	Netrin receptor DCC precursor	R.FLSQTESVTAFM*GDTVLLK.C	2	5.03	0.48	-2.44
IPI00016422	Netrin receptor DCC precursor	R.FLSQTESVTAFM*GDTVLLK.C	3	2.88	0.16	-2.59
IPI00016422	Netrin receptor DCC precursor	R.GGNVLLDCSAESDRGVPVIK.W	3	3.79	0.27	-2.66
IPI00016422	Netrin receptor DCC precursor	R.GYIIGYGVGSPYAETVR.V	2	5.43	0.52	-4.15
IPI00016422	Netrin receptor DCC precursor	R.ILSDPGLHR.Q	2	2.60	0.15	-3.05
IPI00016422	Netrin receptor DCC precursor	R.QLYFLQRPSNVVAIEGK.D	2	4.24	0.37	-3.67
IPI00016422	Netrin receptor DCC precursor	R.QLYFLQRPSNVVAIEGK.D	3	2.91	0.36	-2.66
IPI00016422	Netrin receptor DCC precursor	R.RGEM*ETLEPNNLWYLFTGLEK.G	3	4.17	0.33	-2.20
IPI00016422	Netrin receptor DCC precursor	R.VVVLPSGALQISR.L	2	3.08	0.38	-2.02
IPI00016422	Netrin receptor DCC precursor	V.VVLPSGALQISRLQPGDIGIYR.C	2	2.94	0.17	1.56
IPI00016467	SLIT and NTRK-like protein 3 precursor	K.AVSLTHLDLR.G	2	2.42	0.22	-2.68
IPI00016467	SLIT and NTRK-like protein 3 precursor	K.KLYLSSNLIQK.I	2	2.72	0.11	-2.56
IPI00016467	SLIT and NTRK-like protein 3 precursor	K.LLFLNNNLLR.T	2	2.74	0.20	-2.38
IPI00016467	SLIT and NTRK-like protein 3 precursor	R.GM*LDHIGR.S	2	1.90	0.24	-2.19
IPI00016467	SLIT and NTRK-like protein 3 precursor	R.VLILNDNLIPM*LPTNLFK.A	2	4.50	0.47	-3.34
IPI00016467	SLIT and NTRK-like protein 3 precursor	R.VLILNDNLIPM*LPTNLFK.A	3	3.32	0.22	-4.09
IPI00016576	Isoform 1 of Grainyhead-like protein 2 homolog	K.AEDFTPVFMAPPVHYPRGDGEEQR.V	3	2.30	0.06	1.13
IPI00016577	CDNA: FLJ22814 fis, clone KAIA3004	K.CWLQGTESSPCHSPCYPLGNLK.G	2	1.75	0.06	-6.76
IPI00016605	Uncharacterized protein C1orf123	K.ATLENITNLRPVGEDFR.W	3	2.64	0.22	-1.59
IPI00016621	Adaptor-related protein complex 2, alpha 2 subunit variant (Fragment)	K.IIGFGSALLEEVDPNPANFVGAGIIHTK.T	3	4.84	0.50	-3.31
IPI00016621	Adaptor-related protein complex 2, alpha 2 subunit variant (Fragment)	K.QLSNPQQEVQNIFK.A	2	3.47	0.36	-4.43
IPI00016621	Adaptor-related protein complex 2, alpha 2 subunit variant (Fragment)	R.YGGTFQNVSVQLPITLNK.F	2	4.78	0.40	-3.81
IPI00016645	Isoform 1 of Ephrin type-A receptor 7 precursor	K.IDTIAADESFTQGDLGER.K	2	5.91	0.62	-4.77
IPI00016645	Isoform 1 of Ephrin type-A receptor 7 precursor	K.IDTIAADESFTQGDLGER.K	3	4.34	0.46	-4.05

IPI00016645	Isoform 1 of Ephrin type-A receptor 7 precursor	R.AFTAAGYGNYSPR.L	2	3.32	0.45	-2.95
IPI00016645	Isoform 1 of Ephrin type-A receptor 7 precursor	R.EIGPLSK.K	1	1.40	0.12	-2.36
IPI00016645	Isoform 1 of Ephrin type-A receptor 7 precursor	R.LDVATLEEATGK.M	2	4.08	0.43	-2.03
IPI00016645	Isoform 1 of Ephrin type-A receptor 7 precursor	R.SVELSWQEPEHPNGVITEYEIK.Y	3	3.22	0.17	-3.38
IPI00016645	Isoform 1 of Ephrin type-A receptor 7 precursor	V.KIDTIAADESFTQGDLGER.K	2	6.39	0.61	-1.98
IPI00016645	Isoform 1 of Ephrin type-A receptor 7 precursor	V.KIDTIAADESFTQGDLGER.K	3	4.38	0.36	-1.48
IPI00016666	Metallothionein-3	D.PETCPCPSGGSCTCADSCK.C	2	4.84	0.71	-4.07
IPI00016666	Metallothionein-3	E.TCPCPSGGSCTCADSCK.C	2	3.24	0.55	-2.13
IPI00016666	Metallothionein-3	K.SCCSCCPAECEK.C	2	4.04	0.50	-3.56
IPI00016679	SLIT and NTRK-like protein 5 precursor	K.FAETDM*R.S	2	1.92	0.16	-2.91
IPI00016679	SLIT and NTRK-like protein 5 precursor	R.DLDEVSKQELCPR.R	2	3.24	0.24	-1.52
IPI00016679	SLIT and NTRK-like protein 5 precursor	R.FVPLTHLDLR.G	2	2.17	0.11	-3.84
IPI00016679	SLIT and NTRK-like protein 5 precursor	R.GIISLSEISPPR.F	2	2.44	0.17	-1.60
IPI00016679	SLIT and NTRK-like protein 5 precursor	R.KIESIAELQPKPYNPK.K	3	2.63	0.30	-3.37
IPI00016679	SLIT and NTRK-like protein 5 precursor	R.RTDFLEATGLDLLHLGNNR.I	3	5.22	0.48	-4.48
IPI00016679	SLIT and NTRK-like protein 5 precursor	R.TDFLEATGLDLLHLGNNR.I	2	3.55	0.49	-3.34
IPI00016679	SLIT and NTRK-like protein 5 precursor	R.TDFLEATGLDLLHLGNNR.I	3	4.57	0.46	-2.88
IPI00016685	Enamelin precursor	R.GDSRKVPNSDGQTQSQNLPK.G	2	1.59	0.20	
IPI00016701	P2Y purinoceptor 14	K.ASNYIFVAIFWIVFLLLIVFYTAITK.K	3	3.33	0.10	
IPI00016862	Isoform Mitochondrial of Glutathione reductase, mitochondrial precursor	K.ADFDNTVAIHPTSSEELVTLR.-	3	4.94	0.46	-4.69
IPI00016862	Isoform Mitochondrial of Glutathione reductase, mitochondrial precursor	K.LGGTCVNVGCVPK.K	2	3.08	0.23	2.70
IPI00016862	Isoform Mitochondrial of Glutathione reductase, mitochondrial precursor	R.GHAAFTSDPKPTIEVSGK.K	2	4.69	0.57	-3.49
IPI00016862	Isoform Mitochondrial of Glutathione reductase, mitochondrial precursor	R.GHAAFTSDPKPTIEVSGK.K	3	2.98	0.29	-2.12
IPI00016862	Isoform Mitochondrial of Glutathione reductase, mitochondrial precursor	R.LNAIYQNNLTK.S	2	3.64	0.33	-2.02
IPI00016862	Isoform Mitochondrial of Glutathione reductase, mitochondrial precursor	R.RAAELGAR.A	2	2.65	0.09	-5.09
IPI00016870	Zona pellucida sperm-binding protein 2 precursor	A.TGATEAEKM*TVSLPGPILLLSDDSSFR.G	3	3.97	0.30	-7.72
IPI00016870	Zona pellucida sperm-binding protein 2 precursor	K.DFM*SFSLPR.V	2	2.84	0.25	-2.33
IPI00016870	Zona pellucida sperm-binding protein 2 precursor	K.LTFISPGQK.V	2	1.83	0.06	-0.98
IPI00016870	Zona pellucida sperm-binding protein 2 precursor	K.M*TVSLPGPILLLSDDSSFR.G	2	4.96	0.53	-2.29
IPI00016870	Zona pellucida sperm-binding protein 2 precursor	K.M*TVSLPGPILLLSDDSSFR.G	3	4.43	0.33	-4.08
IPI00016870	Zona pellucida sperm-binding protein 2 precursor	K.TLTLPEAM*K.E	1	2.01	0.08	
IPI00016870	Zona pellucida sperm-binding protein 2 precursor	R.ATGATEAEKM*TVSLPGPILLLSDDSSFR.G	3	6.38	0.49	-3.16
IPI00016870	Zona pellucida sperm-binding protein 2 precursor	R.GVGSSDLK.A	1	1.53	0.13	-3.97
IPI00016870	Zona pellucida sperm-binding protein 2 precursor	T.VSLPGPILLLSDDSSFR.G	2	4.02	0.52	-4.71
IPI00016915	Insulin-like growth factor-binding protein 7 precursor	E.LLPGDRDNLAIQTR.G	2	3.19	0.19	-2.40

IPI00016915	Insulin-like growth factor-binding protein 7 precursor	K.AGAAAGGPGVSGVCVCK.S	2	4.90	0.54	-3.24
IPI00016915	Insulin-like growth factor-binding protein 7 precursor	K.EDAGEYECHASNSQGQASASAK.I	2	5.34	0.55	-4.02
IPI00016915	Insulin-like growth factor-binding protein 7 precursor	K.EDAGEYECHASNSQGQASASAK.I	3	4.79	0.55	-3.42
IPI00016915	Insulin-like growth factor-binding protein 7 precursor	K.GKAGAAAGGPGVSGVCVCK.S	3	3.94	0.37	-0.47
IPI00016915	Insulin-like growth factor-binding protein 7 precursor	K.GTCEQGPSIVTPPK.D	2	3.30	0.28	-3.60
IPI00016915	Insulin-like growth factor-binding protein 7 precursor	K.ITVVDALHEIPVK.K	1	2.38	0.23	-1.62
IPI00016915	Insulin-like growth factor-binding protein 7 precursor	K.ITVVDALHEIPVK.K	2	3.97	0.44	-3.63
IPI00016915	Insulin-like growth factor-binding protein 7 precursor	K.ITVVDALHEIPVK.K	3	2.67	0.37	-1.34
IPI00016915	Insulin-like growth factor-binding protein 7 precursor	K.ITVVDALHEIPVKK.G	2	3.98	0.39	-3.85
IPI00016915	Insulin-like growth factor-binding protein 7 precursor	K.ITVVDALHEIPVKK.G	3	2.58	0.38	-3.13
IPI00016915	Insulin-like growth factor-binding protein 7 precursor	K.ITVVDALHEIPVKKGEGAEL.-	2	5.09	0.54	-4.10
IPI00016915	Insulin-like growth factor-binding protein 7 precursor	K.ITVVDALHEIPVKKGEGAEL.-	3	3.88	0.36	-4.45
IPI00016915	Insulin-like growth factor-binding protein 7 precursor	K.SRYPVCGSDGTTYPSGCQLR.A	3	3.98	0.26	-5.08
IPI00016915	Insulin-like growth factor-binding protein 7 precursor	L.LPGDRDNLAIQTR.G	2	3.04	0.17	-0.65
IPI00016915	Insulin-like growth factor-binding protein 7 precursor	L.PGDRDNLAIQTR.G	2	3.27	0.19	-0.29
IPI00016915	Insulin-like growth factor-binding protein 7 precursor	R.DACGCCPM*CAR.G	2	3.26	0.40	-3.72
IPI00016915	Insulin-like growth factor-binding protein 7 precursor	R.DNLAIQTR.G	1	2.56	0.11	-3.88
IPI00016915	Insulin-like growth factor-binding protein 7 precursor	R.DNLAIQTR.G	2	3.07	0.17	-3.13
IPI00016915	Insulin-like growth factor-binding protein 7 precursor	R.GEGEPCGGGGAGR.G	2	2.87	0.39	-0.14
IPI00016915	Insulin-like growth factor-binding protein 7 precursor	R.GGPEKHEVTGWVLVSPLSK.E	2	5.92	0.57	-3.21
IPI00016915	Insulin-like growth factor-binding protein 7 precursor	R.GGPEKHEVTGWVLVSPLSK.E	3	4.41	0.43	-2.26

IPI00016915	Insulin-like growth factor-binding protein 7 precursor	R.GGPEKHEVTGWVLVSPLSK.E	4	4.13	0.39	-2.55
IPI00016915	Insulin-like growth factor-binding protein 7 precursor	R.GGPEKHEVTGWVLVSPLSKEDAGEYECHASNSQGQASASAK.I	4	6.66	0.50	-2.22
IPI00016915	Insulin-like growth factor-binding protein 7 precursor	R.GGPEKHEVTGWVLVSPLSKEDAGEYECHASNSQGQASASAK.I	5	5.18	0.35	-2.36
IPI00016915	Insulin-like growth factor-binding protein 7 precursor	R.GYCAPGM*ECVK.S	1	2.25	0.30	-2.43
IPI00016915	Insulin-like growth factor-binding protein 7 precursor	R.GYCAPGM*ECVK.S	2	2.90	0.37	-1.70
IPI00016915	Insulin-like growth factor-binding protein 7 precursor	R.TELLPGDRDNLAIQT.R	2	2.92	0.35	-1.70
IPI00016915	Insulin-like growth factor-binding protein 7 precursor	R.TELLPGDRDNLAIQTR.G	2	3.64	0.40	-2.70
IPI00016915	Insulin-like growth factor-binding protein 7 precursor	R.TELLPGDRDNLAIQTR.G	3	2.73	0.24	-2.41
IPI00016915	Insulin-like growth factor-binding protein 7 precursor	R.YPVCGSDGTTYPSGCQLR.A	2	4.88	0.57	1.05
IPI00016915	Insulin-like growth factor-binding protein 7 precursor	T.VVDALHEIPVK.K	2	2.99	0.39	-1.19
IPI00016915	Insulin-like growth factor-binding protein 7 precursor	V.VDALHEIPVK.K	2	2.90	0.27	-0.57
IPI00016949	Isoform 4 of Electrogenic sodium bicarbonate cotransporter 1	R.DAEASNVLVGEVDFLDTPFIAFVR.L	3	2.98	0.24	-1.89
IPI00017163	Isoform 1 of E3 ubiquitin-protein ligase HECW2	R.KLETKGYGQGPGKLK.L	2	3.46	0.24	
IPI00017163	Isoform 1 of E3 ubiquitin-protein ligase HECW2	R.KLETKGYGQGPGKLK.L	3	3.90	0.18	
IPI00017256	Ras suppressor protein 1	K.NLEVLNFFNNQIEELPTQISSLQK.L	3	4.96	0.38	-6.94
IPI00017257	Cathepsin O precursor	K.DSEYPFK.A	2	2.08	0.12	-2.59
IPI00017257	Cathepsin O precursor	K.GKPLEDLSVQQVIDCSYNNYGCNGGSTLNALNWLNK.M	3	6.51	0.62	-0.66
IPI00017257	Cathepsin O precursor	K.GYSAYDFSDQEDEM*AK.A	2	5.37	0.61	-3.58
IPI00017257	Cathepsin O precursor	K.TGSTPYWIVR.N	2	2.94	0.33	-1.97
IPI00017257	Cathepsin O precursor	R.EREAAAFR.E	2	2.16	0.05	-4.02
IPI00017334	Prohibitin	K.AAELIANSLATAGDGLIELR.K	3	4.84	0.35	-1.79
IPI00017334	Prohibitin	R.AATFGLILDDVSLTHLTFGK.E	2	3.10	0.24	-2.14
IPI00017529	Isoform 1 of Lymphocyte function-associated antigen 3 precursor	K.M*ENDLPQK.I	2	2.64	0.05	-2.57
IPI00017557	Secreted frizzled-related protein 4 precursor	R.GVCISPEAIVTDLPEDVK.W	2	5.63	0.45	-2.74
IPI00017557	Secreted frizzled-related protein 4 precursor	R.M*M*LLENCLVEK.W	2	3.46	0.29	-2.03
IPI00017562	Isoform 2 of Latrophilin-2 precursor	M.IVISQLNPYTLR.F	1	2.56	0.17	-1.54
IPI00017562	Isoform 2 of Latrophilin-2 precursor	M.IVISQLNPYTLR.F	2	3.88	0.38	-4.45
IPI00017562	Isoform 2 of Latrophilin-2 precursor	R.AALPFGLVR.R	2	2.31	0.25	-1.61
IPI00017562	Isoform 2 of Latrophilin-2 precursor	R.SGENAASLANELAK.H	2	3.21	0.21	-1.89

IPI00017567	Isoform Long of Endoglin precursor	K.TQILEWAAERGPITSAAELNDPQSILLR.L	3	4.48	0.34	-3.87
IPI00017567	Isoform Long of Endoglin precursor	R.GEVTYTTSQVSK.G	2	3.33	0.32	-3.63
IPI00017567	Isoform Long of Endoglin precursor	R.SAYSSCGMQVSASMISNEAVVNILSSSSPQRK.K	3	2.53	0.09	2.16
IPI00017567	Isoform Long of Endoglin precursor	R.VLPGHSAGPR.T	2	1.90	0.13	0.79
IPI00017569	Fas apoptotic inhibitory molecule 2	K.APGTEGQQQVHGEK.K	2	3.35	0.36	-4.53
IPI00017569	Fas apoptotic inhibitory molecule 2	K.APGTEGQQQVHGEK.K	3	2.28	0.34	-1.97
IPI00017569	Fas apoptotic inhibitory molecule 2	K.APGTEGQQQVHGEKK.E	2	3.35	0.51	-4.24
IPI00017569	Fas apoptotic inhibitory molecule 2	K.EAPAVPSAPPSYEEATSGEGM*K.A	2	3.66	0.51	-4.34
IPI00017569	Fas apoptotic inhibitory molecule 2	K.EAPAVPSAPPSYEEATSGEGM*K.A	3	2.36	0.30	-1.92
IPI00017569	Fas apoptotic inhibitory molecule 2	K.KEAPAVPSAPPSYEEATSGEGM*K.A	3	3.63	0.35	-1.04
IPI00017569	Fas apoptotic inhibitory molecule 2	K.LSVANKAPGTEGQQQVHGEK.K	2	5.04	0.47	-2.62
IPI00017569	Fas apoptotic inhibitory molecule 2	K.LSVANKAPGTEGQQQVHGEK.K	3	5.28	0.40	-2.20
IPI00017569	Fas apoptotic inhibitory molecule 2	K.LSVANKAPGTEGQQQVHGEKK.E	3	4.72	0.37	-4.35
IPI00017569	Fas apoptotic inhibitory molecule 2	S.VANKAPGTEGQQQVHGEK.K	3	4.05	0.33	-2.24
IPI00017601	Ceruloplasmin precursor	A.YPLSIEPIGVR.F	2	3.60	0.37	-2.72
IPI00017601	Ceruloplasmin precursor	D.PTKDIFTGLIGPM*K.I	3	3.62	0.36	-1.23
IPI00017601	Ceruloplasmin precursor	F.PGTYQTLEM*FPR.T	2	3.96	0.39	-2.65
IPI00017601	Ceruloplasmin precursor	I.ASGLIGPLIICK.K	2	3.28	0.31	-3.14
IPI00017601	Ceruloplasmin precursor	I.ASGLIGPLIICKK.D	2	3.32	0.20	-2.45
IPI00017601	Ceruloplasmin precursor	I.FPGTYQTLEM*FPR.T	2	3.55	0.37	-2.25
IPI00017601	Ceruloplasmin precursor	I.SVDTEHSNIYLQNGPDR.I	2	5.41	0.45	-3.04
IPI00017601	Ceruloplasmin precursor	K.AEEEHLGILGPQLHADVGDK.V	2	4.61	0.48	-1.81
IPI00017601	Ceruloplasmin precursor	K.AEEEHLGILGPQLHADVGDKVK.I	2	5.48	0.48	-4.54
IPI00017601	Ceruloplasmin precursor	K.AEEEHLGILGPQLHADVGDKVK.I	3	5.94	0.52	-4.23
IPI00017601	Ceruloplasmin precursor	K.AEEEHLGILGPQLHADVGDKVK.I	4	4.17	0.28	-4.31
IPI00017601	Ceruloplasmin precursor	K.AEEEHLGILGPQLHADVGDKVK.I	5	3.85	0.21	-3.02
IPI00017601	Ceruloplasmin precursor	K.AETGDKVYVHLK.N	1	2.80	0.38	-4.03
IPI00017601	Ceruloplasmin precursor	K.AETGDKVYVHLK.N	2	3.80	0.46	-3.72
IPI00017601	Ceruloplasmin precursor	K.AETGDKVYVHLK.N	3	3.35	0.35	-3.71
IPI00017601	Ceruloplasmin precursor	K.AGLQAFFQVQECNK.S	2	5.46	0.49	-4.75
IPI00017601	Ceruloplasmin precursor	K.AGLQAFFQVQECNK.S	3	3.44	0.28	-1.40
IPI00017601	Ceruloplasmin precursor	K.ALYLQYTDETFR.T	1	3.44	0.40	-4.06
IPI00017601	Ceruloplasmin precursor	K.ALYLQYTDETFR.T	2	4.57	0.48	-7.99
IPI00017601	Ceruloplasmin precursor	K.DDEEFIESNK.M	2	3.88	0.32	-1.78
IPI00017601	Ceruloplasmin precursor	K.DIASGLIGPLIICK.K	1	3.82	0.42	-3.04
IPI00017601	Ceruloplasmin precursor	K.DIASGLIGPLIICK.K	2	4.25	0.40	-4.58
IPI00017601	Ceruloplasmin precursor	K.DIASGLIGPLIICK.K	3	3.93	0.36	-1.97
IPI00017601	Ceruloplasmin precursor	K.DIASGLIGPLIICKK.D	2	4.86	0.36	-3.58
IPI00017601	Ceruloplasmin precursor	K.DIASGLIGPLIICKK.D	3	1.78	0.12	-0.45
IPI00017601	Ceruloplasmin precursor	K.DIFTGLIGPM*K.I	2	2.90	0.33	-2.25
IPI00017601	Ceruloplasmin precursor	K.DLYSGLIGPLIVCR.R	1	3.90	0.49	-3.87
IPI00017601	Ceruloplasmin precursor	K.DLYSGLIGPLIVCR.R	2	4.30	0.51	-4.67

IPI00017601	Ceruloplasmin precursor	K.DLYSGLIGPLIVCR.R	3	5.48	0.37	-2.73
IPI00017601	Ceruloplasmin precursor	K.DNEDFQESNR.M	2	3.09	0.26	-2.71
IPI00017601	Ceruloplasmin precursor	K.DVDKEFYLFPTVFDENESLLLEDNIR.M	3	6.10	0.56	-6.96
IPI00017601	Ceruloplasmin precursor	K.DVDKEFYLFPTVFDENESLLLEDNIR.M	4	3.24	0.18	-3.45
IPI00017601	Ceruloplasmin precursor	K.EFYLFPTVFDENESLLLEDNIR.M	2	4.53	0.47	-1.74
IPI00017601	Ceruloplasmin precursor	K.EFYLFPTVFDENESLLLEDNIR.M	3	4.99	0.40	-3.88
IPI00017601	Ceruloplasmin precursor	K.ERGPEEEHLGILGPVIWAEVGDTIR.V	2	4.23	0.54	-2.43
IPI00017601	Ceruloplasmin precursor	K.ERGPEEEHLGILGPVIWAEVGDTIR.V	3	7.54	0.56	-7.31
IPI00017601	Ceruloplasmin precursor	K.ERGPEEEHLGILGPVIWAEVGDTIR.V	4	3.79	0.33	-4.10
IPI00017601	Ceruloplasmin precursor	K.EVGPTNADPVCLAK.M	1	2.64	0.39	-1.13
IPI00017601	Ceruloplasmin precursor	K.EVGPTNADPVCLAK.M	2	3.88	0.55	-3.64
IPI00017601	Ceruloplasmin precursor	K.GAYPLSIEPIGVR.F	1	2.54	0.33	-2.91
IPI00017601	Ceruloplasmin precursor	K.GAYPLSIEPIGVR.F	2	4.04	0.35	-3.82
IPI00017601	Ceruloplasmin precursor	K.GAYPLSIEPIGVR.F	3	3.51	0.13	-3.01
IPI00017601	Ceruloplasmin precursor	K.GEFYIGSK.Y	1	2.08	0.19	-1.66
IPI00017601	Ceruloplasmin precursor	K.GEFYIGSK.Y	2	2.73	0.26	-2.70
IPI00017601	Ceruloplasmin precursor	K.HRGVYSSDVFDIFPGTYQTLEM*FPR.T	4	3.83	0.39	-1.14
IPI00017601	Ceruloplasmin precursor	K.HYYIGIIETTWDYASDHGEK.K	2	5.24	0.46	-2.79
IPI00017601	Ceruloplasmin precursor	K.HYYIGIIETTWDYASDHGEK.K	3	6.53	0.58	-5.59
IPI00017601	Ceruloplasmin precursor	K.HYYIGIIETTWDYASDHGEKK.L	3	4.00	0.41	-4.37
IPI00017601	Ceruloplasmin precursor	K.KALYLQYTDETFR.T	2	4.81	0.53	-3.58
IPI00017601	Ceruloplasmin precursor	K.KALYLQYTDETFR.T	3	4.32	0.40	-3.32
IPI00017601	Ceruloplasmin precursor	K.KLISVDTEHSNIYLQNGPDR.I	2	6.63	0.59	-4.83
IPI00017601	Ceruloplasmin precursor	K.KLISVDTEHSNIYLQNGPDR.I	3	6.69	0.54	-5.88
IPI00017601	Ceruloplasmin precursor	K.KLISVDTEHSNIYLQNGPDR.I	4	3.89	0.29	-4.41
IPI00017601	Ceruloplasmin precursor	K.KLISVDTEHSNIYLQNGPDRIGR.L	3	4.94	0.45	-3.75
IPI00017601	Ceruloplasmin precursor	K.KLISVDTEHSNIYLQNGPDRIGR.L	4	2.98	0.28	-2.55
IPI00017601	Ceruloplasmin precursor	K.LISVDTEHSNIYLQNGPDR.I	2	6.02	0.54	-3.18
IPI00017601	Ceruloplasmin precursor	K.LISVDTEHSNIYLQNGPDR.I	3	5.15	0.42	-6.15
IPI00017601	Ceruloplasmin precursor	K.LISVDTEHSNIYLQNGPDRIGR.L	3	3.01	0.28	-1.87
IPI00017601	Ceruloplasmin precursor	K.LVYREYTDASFTNR.K	3	3.35	0.21	-2.72
IPI00017601	Ceruloplasmin precursor	K.LVYREYTDASFTNRK.E	2	3.01	0.30	-4.94
IPI00017601	Ceruloplasmin precursor	K.LVYREYTDASFTNRK.E	3	4.32	0.34	-4.01
IPI00017601	Ceruloplasmin precursor	K.M*YYSAVDPTK.D	1	1.99	0.06	-3.35
IPI00017601	Ceruloplasmin precursor	K.M*YYSAVDPTK.D	2	3.06	0.34	-3.74
IPI00017601	Ceruloplasmin precursor	K.M*YYSAVDPTKDIFTGLIGPM*K.I	2	4.31	0.48	-3.38
IPI00017601	Ceruloplasmin precursor	K.M*YYSAVDPTKDIFTGLIGPM*K.I	3	4.57	0.36	-4.24
IPI00017601	Ceruloplasmin precursor	K.NLASRPYTFHSHGITYYK.E	2	4.30	0.52	-4.05
IPI00017601	Ceruloplasmin precursor	K.NLASRPYTFHSHGITYYK.E	3	4.17	0.38	-3.95
IPI00017601	Ceruloplasmin precursor	K.NM*ATRPYSIHAHGVQTESSTVTPTLPGETLTYVWK.I	4	4.05	0.24	-4.52
IPI00017601	Ceruloplasmin precursor	K.NNEGTYYSPNYNPQSR.S	2	5.00	0.47	-3.76
IPI00017601	Ceruloplasmin precursor	K.NNEGTYYSPNYNPQSR.S	3	3.27	0.17	-3.26

IPI00017601	Ceruloplasmin precursor	K.PVWLGFLGPIIK.A	2	4.19	0.44	-3.44
IPI00017601	Ceruloplasmin precursor	K.TYCSEPEKVDKDNEDFQESNR.M	3	4.93	0.40	
IPI00017601	Ceruloplasmin precursor	K.TYCSEPEKVDKDNEDFQESNR.M	4	2.21	0.11	-1.17
IPI00017601	Ceruloplasmin precursor	K.TYSDHPEK.V	2	2.65	0.32	-2.09
IPI00017601	Ceruloplasmin precursor	K.TYSDHPEKVNKDDEEFIESNK.M	2	5.12	0.53	-4.66
IPI00017601	Ceruloplasmin precursor	K.TYSDHPEKVNKDDEEFIESNK.M	3	6.29	0.57	-3.86
IPI00017601	Ceruloplasmin precursor	K.TYSDHPEKVNKDDEEFIESNK.M	4	2.98	0.36	-3.08
IPI00017601	Ceruloplasmin precursor	K.TYSDHPEKVNKDDEEFIESNK.M	5	2.49	0.13	-2.22
IPI00017601	Ceruloplasmin precursor	K.VDKDNEDFQESNR.M	2	4.40	0.45	-2.97
IPI00017601	Ceruloplasmin precursor	K.VDKDNEDFQESNR.M	3	4.20	0.26	-1.33
IPI00017601	Ceruloplasmin precursor	K.VNKDDEEFIESNK.M	2	4.32	0.33	-5.08
IPI00017601	Ceruloplasmin precursor	K.VNKDDEEFIESNK.M	3	4.50	0.26	-1.35
IPI00017601	Ceruloplasmin precursor	K.VNKDDEEFIESNKM*HAINGR.M	3	3.00	0.27	-5.32
IPI00017601	Ceruloplasmin precursor	K.VNKDDEEFIESNKM*HAINGR.M	4	3.54	0.15	-2.20
IPI00017601	Ceruloplasmin precursor	K.VVYRQYTDSTFRVPVER.K	3	3.20	0.32	-1.44
IPI00017601	Ceruloplasmin precursor	K.WYLFGM*GNEVDVHAAFFHGQALTNK.N	3	3.54	0.36	-4.14
IPI00017601	Ceruloplasmin precursor	K.WYLFGM*GNEVDVHAAFFHGQALTNK.N	4	4.01	0.43	-4.32
IPI00017601	Ceruloplasmin precursor	K.YTVNQCR.R	2	2.78	0.29	-2.16
IPI00017601	Ceruloplasmin precursor	L.GPQLHADVGDKVK.I	2	3.28	0.40	-4.07
IPI00017601	Ceruloplasmin precursor	L.ISVDTEHSNIYLQNGPDR.I	2	4.72	0.45	-1.92
IPI00017601	Ceruloplasmin precursor	L.YSGLIGPLIVCR.R	2	3.20	0.28	-6.01
IPI00017601	Ceruloplasmin precursor	R.ADDKVYPGEQYTYM*LLATEEQSPGEGDGNCVTR.I	3	7.41	0.63	-4.20
IPI00017601	Ceruloplasmin precursor	R.ADDKVYPGEQYTYM*LLATEEQSPGEGDGNCVTR.I	4	5.82	0.61	-2.88
IPI00017601	Ceruloplasmin precursor	R.ADDKVYPGEQYTYMLLATEEQSPGEGDGNCVTR.I	3	7.23	0.58	-5.23
IPI00017601	Ceruloplasmin precursor	R.ADDKVYPGEQYTYMLLATEEQSPGEGDGNCVTR.I	4	5.24	0.40	-4.95
IPI00017601	Ceruloplasmin precursor	R.DTANLFPQTSLTLH.M	2	4.04	0.44	-4.36
IPI00017601	Ceruloplasmin precursor	R.DTANLFPQTSLTLHM*WPDTEGTFNVECLTTDHYTGGM*K.Q	3	4.40	0.53	-3.98
IPI00017601	Ceruloplasmin precursor	R.EYTDASFTNR.K	1	2.35	0.45	-3.89
IPI00017601	Ceruloplasmin precursor	R.EYTDASFTNR.K	2	3.40	0.49	-3.83
IPI00017601	Ceruloplasmin precursor	R.EYTDASFTNRK.E	2	2.58	0.32	-3.42
IPI00017601	Ceruloplasmin precursor	R.FNKNNEGTYYSPNYNPQSR.S	2	5.74	0.38	-4.97
IPI00017601	Ceruloplasmin precursor	R.FNKNNEGTYYSPNYNPQSR.S	3	5.62	0.40	-3.77
IPI00017601	Ceruloplasmin precursor	R.GPEEEHLGILGPVIWAEVGDTIR.V	2	6.42	0.60	-5.04
IPI00017601	Ceruloplasmin precursor	R.GPEEEHLGILGPVIWAEVGDTIR.V	3	6.06	0.57	-8.59
IPI00017601	Ceruloplasmin precursor	R.GPEEEHLGILGPVIWAEVGDTIR.V	4	3.41	0.16	-2.39
IPI00017601	Ceruloplasmin precursor	R.GVYSSDVFDIFPGTYQTLEM*FPR.T	2	5.11	0.59	-5.55
IPI00017601	Ceruloplasmin precursor	R.GVYSSDVFDIFPGTYQTLEM*FPR.T	3	4.75	0.51	-5.54
IPI00017601	Ceruloplasmin precursor	R.GVYSSDVFDIFPGTYQTLEM*FPR.T	4	4.13	0.29	-4.06
IPI00017601	Ceruloplasmin precursor	R.GVYSSDVFDIFPGTYQTLEMFPR.T	2	5.09	0.55	-4.10
IPI00017601	Ceruloplasmin precursor	R.GVYSSDVFDIFPGTYQTLEMFPR.T	3	3.98	0.40	-2.99
IPI00017601	Ceruloplasmin precursor	R.HYYIAAEEIIWNYAPSGIDIFTK.E	3	4.28	0.32	-6.01
IPI00017601	Ceruloplasmin precursor	R.IDTINLFPATLFDAY.M	2	3.27	0.39	-4.19

IPI00017601	Ceruloplasmin precursor	R.IDTINLFPATLFDAYM*VAQNPGEWM*LSCQNLNHLK.A	3	5.70	0.44	-4.83
IPI00017601	Ceruloplasmin precursor	R.IDTINLFPATLFDAYM*VAQNPGEWM*LSCQNLNHLK.A	4	5.65	0.48	-5.83
IPI00017601	Ceruloplasmin precursor	R.IDTINLFPATLFDAYM*VAQNPGEWM*LSCQNLNHLK.A	5	2.84	0.10	-4.92
IPI00017601	Ceruloplasmin precursor	R.IDTINLFPATLFDAYM*VAQNPGEWMLSCQNLNHLK.A	3	5.25	0.15	-3.14
IPI00017601	Ceruloplasmin precursor	R.IDTINLFPATLFDAYM*VAQNPGEWMLSCQNLNHLK.A	4	4.04	0.14	-4.54
IPI00017601	Ceruloplasmin precursor	R.IDTINLFPATLFDAYMVAQNPGEWM*LSCQNLNHLK.A	4	5.40	0.13	-3.59
IPI00017601	Ceruloplasmin precursor	R.IYHSHIDAPK.D	1	2.88	0.25	-5.05
IPI00017601	Ceruloplasmin precursor	R.IYHSHIDAPK.D	2	2.59	0.24	-3.95
IPI00017601	Ceruloplasmin precursor	R.IYHSHIDAPKDIASGLIGPLIICK.K	3	4.19	0.44	-5.22
IPI00017601	Ceruloplasmin precursor	R.KAEEEHLGILGPQLHADVGDK.V	2	5.78	0.48	-3.36
IPI00017601	Ceruloplasmin precursor	R.KAEEEHLGILGPQLHADVGDK.V	3	6.44	0.54	-6.82
IPI00017601	Ceruloplasmin precursor	R.KAEEEHLGILGPQLHADVGDK.V	4	4.17	0.39	-3.48
IPI00017601	Ceruloplasmin precursor	R.KAEEEHLGILGPQLHADVGDKVK.I	2	5.96	0.61	-4.90
IPI00017601	Ceruloplasmin precursor	R.KAEEEHLGILGPQLHADVGDKVK.I	3	7.25	0.57	-6.07
IPI00017601	Ceruloplasmin precursor	R.KAEEEHLGILGPQLHADVGDKVK.I	4	5.47	0.51	-4.56
IPI00017601	Ceruloplasmin precursor	R.KAEEEHLGILGPQLHADVGDKVK.I	5	4.05	0.40	-3.99
IPI00017601	Ceruloplasmin precursor	R.KAEEEHLGILGPQLHADVGDKVK.I	6	2.12	0.22	-3.61
IPI00017601	Ceruloplasmin precursor	R.KLEFALLFLVFDENESWYLDDNIK.T	3	5.27	0.36	
IPI00017601	Ceruloplasmin precursor	R.M*FTTAPDQVDKEDEDFQESNK.M	2	5.02	0.56	-3.37
IPI00017601	Ceruloplasmin precursor	R.M*FTTAPDQVDKEDEDFQESNK.M	3	3.92	0.47	-3.17
IPI00017601	Ceruloplasmin precursor	R.M*YSVNGYTFGSLPGLSM*CAEDR.V	2	4.79	0.61	-1.87
IPI00017601	Ceruloplasmin precursor	R.M*YSVNGYTFGSLPGLSM*CAEDR.V	3	4.87	0.48	-1.64
IPI00017601	Ceruloplasmin precursor	R.M*YSVNGYTFGSLPGLSM*CAEDRVK.W	3	3.31	0.41	-2.51
IPI00017601	Ceruloplasmin precursor	R.PYSIHAHGVQTESSTVTPTLPGETLTYVWK.I	4	4.49	0.31	-2.82
IPI00017601	Ceruloplasmin precursor	R.PYTFHSHGITYYK.E	2	4.18	0.51	-4.08
IPI00017601	Ceruloplasmin precursor	R.QKDVDKEFYLFPTVFDENESLLLEDNIR.M	3	6.14	0.57	-5.17
IPI00017601	Ceruloplasmin precursor	R.QKDVDKEFYLFPTVFDENESLLLEDNIR.M	4	4.68	0.33	-6.05
IPI00017601	Ceruloplasmin precursor	R.QSEDSTFYLGER.T	2	3.54	0.43	-5.44
IPI00017601	Ceruloplasmin precursor	R.QYTDSTFR.V	2	1.55	0.21	-1.78
IPI00017601	Ceruloplasmin precursor	R.QYTDSTFRVPVER.K	3	1.89	0.27	-2.61
IPI00017601	Ceruloplasmin precursor	R.RQSEDSTFYLGER.T	2	4.60	0.44	-3.84
IPI00017601	Ceruloplasmin precursor	R.RQSEDSTFYLGER.T	3	3.33	0.11	-1.79
IPI00017601	Ceruloplasmin precursor	R.SGAGTEDSACIPWAYYSTVDQVK.D	2	5.21	0.51	-5.00
IPI00017601	Ceruloplasmin precursor	R.SGAGTEDSACIPWAYYSTVDQVK.D	3	5.41	0.54	-5.56
IPI00017601	Ceruloplasmin precursor	R.SGAGTEDSACIPWAYYSTVDQVKDLYSGLIGPLIVCR.R	3	6.30	0.61	-3.01
IPI00017601	Ceruloplasmin precursor	R.SGAGTEDSACIPWAYYSTVDQVKDLYSGLIGPLIVCR.R	4	4.30	0.30	-3.75
IPI00017601	Ceruloplasmin precursor	R.SVPPSASHVAPTETFT.Y	2	2.97	0.46	-2.15
IPI00017601	Ceruloplasmin precursor	R.SVPPSASHVAPTETFTYEWTVPK.E	2	3.33	0.38	-5.37
IPI00017601	Ceruloplasmin precursor	R.SVPPSASHVAPTETFTYEWTVPK.E	3	3.41	0.36	-5.94
IPI00017601	Ceruloplasmin precursor	R.TTIEKPVWLGFLGPIIK.A	2	4.91	0.49	-4.57
IPI00017601	Ceruloplasmin precursor	R.TTIEKPVWLGFLGPIIK.A	3	3.81	0.20	-6.26
IPI00017601	Ceruloplasmin precursor	R.TTIEKPVWLGFLGPIIK.A	4	2.59	0.26	-1.40

IPI00017601	Ceruloplasmin precursor	R.VTFHNKGAYPLSIEPIGVR.F	3	4.11	0.42	-3.25
IPI00017601	Ceruloplasmin precursor	S.VPPSASHVAPTETFTYEWTVPK.E	3	3.74	0.40	-2.98
IPI00017601	Ceruloplasmin precursor	V.PPSASHVAPTETFTYEWTVPK.E	2	4.15	0.56	-2.44
IPI00017601	Ceruloplasmin precursor	W.AYYSTVDQVK.D	1	2.39	0.30	-3.51
IPI00017601	Ceruloplasmin precursor	W.AYYSTVDQVK.D	2	3.02	0.35	-3.31
IPI00017601	Ceruloplasmin precursor	W.LGFLGPIIK.A	2	3.37	0.24	-0.93
IPI00017601	Ceruloplasmin precursor	W.PDTEGTFNVECLTTDHYTGGM*K.Q	3	3.52	0.48	-2.13
IPI00017601	Ceruloplasmin precursor	Y.LFPTVFDENESLLLEDNIR.M	2	4.27	0.34	-4.81
IPI00017601	Ceruloplasmin precursor	Y.PLSIEPIGVR.F	1	2.42	0.23	-3.78
IPI00017601	Ceruloplasmin precursor	Y.PLSIEPIGVR.F	2	3.32	0.15	-2.07
IPI00017659	Protein kinase substrate CapZIP	R.VQNEEVGPEHDSQETK.K	3	2.14	0.13	-1.70
IPI00017696	Complement C1s subcomponent precursor	A.EPTM*YGEILSPNYPQAYPSEVEK.S	2	4.61	0.53	-5.58
IPI00017696	Complement C1s subcomponent precursor	A.EPTM*YGEILSPNYPQAYPSEVEK.S	3	5.10	0.46	-4.47
IPI00017696	Complement C1s subcomponent precursor	K.CQPVDCGIPESIENGKVEDPESTLFGSVIR.Y	3	3.94	0.24	
IPI00017696	Complement C1s subcomponent precursor	K.CVPVCGVPR.E	2	2.25	0.19	
IPI00017696	Complement C1s subcomponent precursor	K.CVPVCGVPREPFEEK.Q	2	4.05	0.24	
IPI00017696	Complement C1s subcomponent precursor	K.EDTPNSVWEPAK.A	2	3.33	0.31	-3.34
IPI00017696	Complement C1s subcomponent precursor	K.EVKVEKPTADAEAYVFTPN.M	2	4.04	0.48	-1.83
IPI00017696	Complement C1s subcomponent precursor	K.EVKVEKPTADAEAYVFTPNM*ICAGGEK.G	3	4.71	0.09	
IPI00017696	Complement C1s subcomponent precursor	K.FYAAGLVSWGPQCGTYGLYTR.V	2	4.15	0.51	-3.51
IPI00017696	Complement C1s subcomponent precursor	K.FYAAGLVSWGPQCGTYGLYTR.V	3	4.47	0.16	-2.57
IPI00017696	Complement C1s subcomponent precursor	K.GDSGGAFAVQDPNDK.T	2	4.72	0.50	-4.42
IPI00017696	Complement C1s subcomponent precursor	K.GDSGGAFAVQDPNDKTK.F	2	4.37	0.34	-3.40
IPI00017696	Complement C1s subcomponent precursor	K.GM*DSCKGDSGGAFAVQDPNDK.T	3	3.79	0.37	-2.08
IPI00017696	Complement C1s subcomponent precursor	K.GM*DSCKGDSGGAFAVQDPNDKTK.F	2	3.87	0.43	
IPI00017696	Complement C1s subcomponent precursor	K.GM*DSCKGDSGGAFAVQDPNDKTK.F	3	6.04	0.32	
IPI00017696	Complement C1s subcomponent precursor	K.NYVDWIM*K.T	1	2.11	0.06	-3.24
IPI00017696	Complement C1s subcomponent precursor	K.SDFSNEER.F	2	2.18	0.17	-2.43
IPI00017696	Complement C1s subcomponent precursor	K.SNALDIIFQTDLTGQK.K	2	5.63	0.44	-5.46
IPI00017696	Complement C1s subcomponent precursor	K.SNALDIIFQTDLTGQK.K	3	5.96	0.41	-2.34
IPI00017696	Complement C1s subcomponent precursor	K.SNALDIIFQTDLTGQKK.G	2	4.94	0.49	-5.01
IPI00017696	Complement C1s subcomponent precursor	K.SNALDIIFQTDLTGQKK.G	3	3.45	0.39	-3.81
IPI00017696	Complement C1s subcomponent precursor	K.TM*QENSTPRED.-	2	3.28	0.41	-4.61
IPI00017696	Complement C1s subcomponent precursor	K.VEKPTADAEAYVFTPN.M	2	3.80	0.37	-3.34
IPI00017696	Complement C1s subcomponent precursor	K.VEKPTADAEAYVFTPNM*ICAGGEK.G	2	5.50	0.62	-3.28
IPI00017696	Complement C1s subcomponent precursor	K.VEKPTADAEAYVFTPNM*ICAGGEK.G	3	4.36	0.39	-3.59
IPI00017696	Complement C1s subcomponent precursor	K.VEKPTADAEAYVFTPNM*ICAGGEK.G	4	3.09	0.22	-2.46
IPI00017696	Complement C1s subcomponent precursor	K.VEKPTADAEAYVFTPNMICAGGEK.G	3	4.31	0.23	
IPI00017696	Complement C1s subcomponent precursor	R.CEYQIR.L	2	2.44	0.12	-2.67
IPI00017696	Complement C1s subcomponent precursor	R.DVVQITCLDGFEVVEGR.V	2	5.54	0.49	-6.79
IPI00017696	Complement C1s subcomponent precursor	R.DVVQITCLDGFEVVEGR.V	3	4.71	0.35	-4.17
IPI00017696	Complement C1s subcomponent precursor	R.EDFDVEAADSAGNCLDSLVFVAGDR.Q	2	5.68	0.43	

IPI00017696	Complement C1s subcomponent precursor	R.EDFDVEAADSAGNCLDSLVFVAGDR.Q	3	5.35	0.31	
IPI00017696	Complement C1s subcomponent precursor	R.EPTM*YVGSTSVQTSR.L	2	3.48	0.44	-2.93
IPI00017696	Complement C1s subcomponent precursor	R.EPTM*YVGSTSVQTSR.L	3	3.16	0.33	-2.46
IPI00017696	Complement C1s subcomponent precursor	R.EPTMYVGSTSVQTSR.L	3	3.71	0.06	
IPI00017696	Complement C1s subcomponent precursor	R.IIGGSDADIK.N	1	1.98	0.11	-3.29
IPI00017696	Complement C1s subcomponent precursor	R.IIGGSDADIK.N	2	2.57	0.14	-2.58
IPI00017696	Complement C1s subcomponent precursor	R.QFGPYCGHGFPGPLNIETK.S	2	2.52	0.12	
IPI00017696	Complement C1s subcomponent precursor	R.REDFDVEAADSAGNCLDSLVFVAGDR.Q	3	4.07	0.49	-2.65
IPI00017696	Complement C1s subcomponent precursor	R.SSNNPHSPIVEEFQVPYNK.L	2	4.59	0.46	-4.20
IPI00017696	Complement C1s subcomponent precursor	R.SSNNPHSPIVEEFQVPYNK.L	3	3.54	0.19	-2.91
IPI00017696	Complement C1s subcomponent precursor	R.SSNNPHSPIVEEFQVPYNKLQVIFK.S	3	4.04	0.37	-4.49
IPI00017696	Complement C1s subcomponent precursor	R.SSNNPHSPIVEEFQVPYNKLQVIFK.S	4	5.12	0.37	-4.83
IPI00017696	Complement C1s subcomponent precursor	R.TNFDNDIALVR.L	1	2.41	0.37	-1.61
IPI00017696	Complement C1s subcomponent precursor	R.TNFDNDIALVR.L	2	3.89	0.41	-3.75
IPI00017696	Complement C1s subcomponent precursor	R.VGATSFYSTCQSNGK.W	2	4.75	0.43	-1.38
IPI00017696	Complement C1s subcomponent precursor	R.VGATSFYSTCQSNGK.W	3	4.70	0.44	-0.04
IPI00017696	Complement C1s subcomponent precursor	R.VKNYVDWIM*K.T	2	2.87	0.32	-2.17
IPI00017696	Complement C1s subcomponent precursor	R.VKNYVDWIM*K.T	3	3.01	0.11	-2.95
IPI00017696	Complement C1s subcomponent precursor	W.VNEVLGPELPK.C	2	3.31	0.25	-1.01
IPI00017704	Coactosin-like protein	K.EFVISDRK.E	2	2.42	0.07	-2.26
IPI00017704	Coactosin-like protein	K.EFVISDRKELEEDFIKSELKK.A	3	4.70	0.38	-2.98
IPI00017704	Coactosin-like protein	K.FALITWIGENVSGLQR.A	2	4.79	0.37	-3.82
IPI00017704	Coactosin-like protein	K.FALITWIGENVSGLQR.A	3	5.20	0.44	-3.54
IPI00017704	Coactosin-like protein	R.FTTGDAM*SK.R	2	2.23	0.20	-1.60
IPI00017704	Coactosin-like protein	R.FTTGDAM*SKR.S	2	2.50	0.26	-2.14
IPI00017704	Coactosin-like protein	R.KELEEDFIKSELKK.A	3	4.35	0.32	-3.76
IPI00017704	Coactosin-like protein	R.KELEEDFIKSELKK.A	4	4.01	0.28	-3.95
IPI00017745	Metalloproteinase inhibitor 4 precursor	K.VFIHLCNYIEPWEDLSLVQR.E	3	3.63	0.17	
IPI00017745	Metalloproteinase inhibitor 4 precursor	K.VKDVQYIYTPFDSSLCGVK.L	2	3.79	0.35	
IPI00017745	Metalloproteinase inhibitor 4 precursor	K.VVPASADPADTEK.M	2	1.54	0.22	-3.41
IPI00017841	Isoform 1 of Noelin precursor	K.LTGISDPVTVK.T	1	2.23	0.16	-3.51
IPI00017841	Isoform 1 of Noelin precursor	K.LTGISDPVTVK.T	2	3.27	0.27	-1.41
IPI00017841	Isoform 1 of Noelin precursor	K.M*DELRPLIPVLEEYKADAK.L	4	2.59	0.12	-2.22
IPI00017841	Isoform 1 of Noelin precursor	R.CICTVVAPQQTM*CSR.D	2	4.43	0.49	-2.54
IPI00017841	Isoform 1 of Noelin precursor	W.QVYSSAQDSEGR.C	2	3.42	0.50	-2.96
IPI00017940	LMBR1 domain-containing protein 2	-.M*SGAALGLEIVFVFFLALFLLHR.Y	3	2.18	0.12	0.08
IPI00017964	Small nuclear ribonucleoprotein Sm D3	R.GRGRGM*GRGNIFQKR.R	2	2.07	0.22	
IPI00017968	ADM precursor	K.AGPAQTLIRPQDM*K.G	2	2.63	0.20	-2.70
IPI00017968	ADM precursor	K.AGPAQTLIRPQDM*K.G	3	2.07	0.11	-1.24
IPI00017968	ADM precursor	K.GASRSPEDSSPDAAR.I	2	3.66	0.38	-3.00
IPI00017968	ADM precursor	R.SPEDSSPDAAR.I	2	3.27	0.33	-3.03
IPI00017968	ADM precursor	R.TLVSSKPQAHGAPAPPSGSAPH.F	3	4.60	0.43	-2.74

IPI00017968	ADM precursor	R.TLVSSKPQAHGAPAPPSGSAPHFL.-	3	3.03	0.13	-3.48
IPI00018027	Isoform 1 of Angiogenic factor with G patch and FHA domains 1	K.KDESFVGPTLSKEEKELERRKELKK.I	3	3.71	0.08	
IPI00018098	Isoform 1 of Pre-mRNA-splicing factor 38B	K.TAGQTGMCGGVR.G	2	2.78	0.10	
IPI00018136	Isoform 1 of Vascular cell adhesion protein 1 precursor	K.CSVADVYPFDRLEIDLLK.G	3	3.23	0.34	-4.68
IPI00018136	Isoform 1 of Vascular cell adhesion protein 1 precursor	K.CSVADVYPFDRLEIDLLKGDHLM*K.S	3	2.67	0.06	-4.99
IPI00018136	Isoform 1 of Vascular cell adhesion protein 1 precursor	K.CSVADVYPFDRLEIDLLKGDHLM*K.S	4	3.09	0.32	-3.55
IPI00018136	Isoform 1 of Vascular cell adhesion protein 1 precursor	K.ELQVYISPK.N	1	2.50	0.12	-2.89
IPI00018136	Isoform 1 of Vascular cell adhesion protein 1 precursor	K.EVELIVQEKPFTVEISPGPR.I	3	4.85	0.42	-3.88
IPI00018136	Isoform 1 of Vascular cell adhesion protein 1 precursor	K.GIQVEIYSFPKDPEIHLSGPLEAGKPITVK.C	3	6.53	0.59	-4.11
IPI00018136	Isoform 1 of Vascular cell adhesion protein 1 precursor	K.KAETGDTVLK.S	2	2.48	0.17	-2.60
IPI00018136	Isoform 1 of Vascular cell adhesion protein 1 precursor	K.LHIDDM*EFEPK.Q	2	2.98	0.35	-2.95
IPI00018136	Isoform 1 of Vascular cell adhesion protein 1 precursor	K.LTAFPSESVK.E	2	2.36	0.13	-2.75
IPI00018136	Isoform 1 of Vascular cell adhesion protein 1 precursor	K.NTVISVNPSTK.L	1	2.50	0.35	-2.89
IPI00018136	Isoform 1 of Vascular cell adhesion protein 1 precursor	K.SIDGAYTIR.K	1	1.72	0.16	-2.61
IPI00018136	Isoform 1 of Vascular cell adhesion protein 1 precursor	K.SIDGAYTIRK.A	2	1.81	0.13	-2.97
IPI00018136	Isoform 1 of Vascular cell adhesion protein 1 precursor	K.SLEVTFTPVIEDIGK.V	2	4.80	0.45	-4.85
IPI00018136	Isoform 1 of Vascular cell adhesion protein 1 precursor	K.SQEFLEDADRK.S	2	3.87	0.29	-1.26
IPI00018136	Isoform 1 of Vascular cell adhesion protein 1 precursor	K.VPSVYPLDRLEIELLK.G	2	3.11	0.11	-3.92
IPI00018136	Isoform 1 of Vascular cell adhesion protein 1 precursor	K.VPSVYPLDRLEIELLK.G	3	2.45	0.24	-2.84
IPI00018136	Isoform 1 of Vascular cell adhesion protein 1 precursor	K.VTNEGTTSTLTM*NPVSFGNEHSYLCTATCESR.K	3	5.91	0.63	-3.34
IPI00018136	Isoform 1 of Vascular cell adhesion protein 1 precursor	R.M*EDSGIYVCEGVNLIGK.N	2	5.21	0.58	-4.87
IPI00018136	Isoform 1 of Vascular cell adhesion protein 1 precursor	R.QSTQTLYVNVAPR.D	2	4.28	0.42	-3.01

IPI00018136	Isoform 1 of Vascular cell adhesion protein 1 precursor	R.TQIDSPLNGK.V	2	1.98	0.12	-2.99
IPI00018136	Isoform 1 of Vascular cell adhesion protein 1 precursor	R.TQIDSPLSGK.V	1	2.31	0.32	-2.99
IPI00018136	Isoform 1 of Vascular cell adhesion protein 1 precursor	R.TQIDSPLSGK.V	2	2.20	0.14	-1.26
IPI00018146	14-3-3 protein theta	K.TAFDEAIAELDTLNEDSYKDSTLIM*QLLR.D	3	5.15	0.50	-8.16
IPI00018146	14-3-3 protein theta	R.NLLSVAYK.N	1	1.68	0.08	-2.46
IPI00018146	14-3-3 protein theta	R.NLLSVAYK.N	2	2.28	0.09	-1.47
IPI00018146	14-3-3 protein theta	R.YDDM*ATCM*K.A	2	2.07	0.23	-2.36
IPI00018206	Aspartate aminotransferase, mitochondrial precursor	K.ASAELALGENSEVLK.S	2	3.24	0.19	-3.82
IPI00018206	Aspartate aminotransferase, mitochondrial precursor	K.M*NLGVGAYRDDNGKPYVLPSVR.K	3	3.54	0.37	-2.80
IPI00018206	Aspartate aminotransferase, mitochondrial precursor	K.M*NLGVGAYRDDNGKPYVLPSVR.K	4	3.47	0.25	-3.00
IPI00018206	Aspartate aminotransferase, mitochondrial precursor	R.DAGM*QLQGYR.Y	2	3.46	0.30	-2.82
IPI00018206	Aspartate aminotransferase, mitochondrial precursor	R.IAAAILNTPDLRK.Q	2	1.73	0.12	-2.21
IPI00018206	Aspartate aminotransferase, mitochondrial precursor	R.KAEAQIAAKNLDKEYLPIGGLAEFCK.A	3	3.17	0.16	
IPI00018206	Aspartate aminotransferase, mitochondrial precursor	R.NLFAFFDMAYQGFASGDGDKDAWAVR.H	3	2.54	0.15	-2.44
IPI00018208	Tetratricopeptide repeat protein 33	R.TLQEQQKVAQR.I	2	2.61	0.11	
IPI00018219	Transforming growth factor-beta-induced protein ig-h3 precursor	K.ADHHATNGVVHLIDK.V	3	2.95	0.23	-3.29
IPI00018219	Transforming growth factor-beta-induced protein ig-h3 precursor	K.ADHHATNGVVHLIDK.V	4	2.76	0.19	-3.91
IPI00018219	Transforming growth factor-beta-induced protein ig-h3 precursor	K.AIISNKDILATNGVIHYIDELLIPDSAK.T	3	7.14	0.55	-6.10
IPI00018219	Transforming growth factor-beta-induced protein ig-h3 precursor	K.AIISNKDILATNGVIHYIDELLIPDSAK.T	4	2.98	0.20	-3.97
IPI00018219	Transforming growth factor-beta-induced protein ig-h3 precursor	K.DGTPPIDAHTR.N	1	2.45	0.29	-3.28
IPI00018219	Transforming growth factor-beta-induced protein ig-h3 precursor	K.DGTPPIDAHTR.N	2	2.26	0.24	-3.72
IPI00018219	Transforming growth factor-beta-induced protein ig-h3 precursor	K.DILATNGVIHYIDELLIPDSAK.T	2	5.14	0.44	-3.59
IPI00018219	Transforming growth factor-beta-induced protein ig-h3 precursor	K.DILATNGVIHYIDELLIPDSAK.T	3	3.64	0.34	-4.35

IPI00018219	Transforming growth factor-beta-induced protein ig-h3 precursor	K.GCPAALPLSNLYETLGVVGSTTTQLYTDR.T	3	3.94	0.34	0.07
IPI00018219	Transforming growth factor-beta-induced protein ig-h3 precursor	K.GCPAALPLSNLYETLGVVGSTTTQLYTDRTEK.L	3	3.91	0.38	-5.17
IPI00018219	Transforming growth factor-beta-induced protein ig-h3 precursor	K.GCPAALPLSNLYETLGVVGSTTTQLYTDRTEK.L	4	4.59	0.34	-6.00
IPI00018219	Transforming growth factor-beta-induced protein ig-h3 precursor	K.IPSETLNR.I	2	2.06	0.20	-2.58
IPI00018219	Transforming growth factor-beta-induced protein ig-h3 precursor	K.NNVVSVNKEPVAEPDIM*ATNGVVHVITNVLQPPANRPQER.G	4	6.33	0.49	-4.19
IPI00018219	Transforming growth factor-beta-induced protein ig-h3 precursor	K.NNVVSVNKEPVAEPDIM*ATNGVVHVITNVLQPPANRPQER.G	5	3.51	0.38	-7.11
IPI00018219	Transforming growth factor-beta-induced protein ig-h3 precursor	K.SLQGDKLEVSLK.N	2	3.36	0.25	-2.68
IPI00018219	Transforming growth factor-beta-induced protein ig-h3 precursor	K.SPYQLVLQHSR.L	2	3.89	0.40	-2.70
IPI00018219	Transforming growth factor-beta-induced protein ig-h3 precursor	K.SPYQLVLQHSR.L	3	3.69	0.34	-3.35
IPI00018219	Transforming growth factor-beta-induced protein ig-h3 precursor	K.STVISYECCPGYEKVPGEK.G	2	3.36	0.29	
IPI00018219	Transforming growth factor-beta-induced protein ig-h3 precursor	K.TLFELAAESDVSTAIDLFR.Q	2	4.70	0.48	-6.21
IPI00018219	Transforming growth factor-beta-induced protein ig-h3 precursor	K.VISTITNNIQQIIEIEDTFETLR.A	2	5.88	0.50	-2.97
IPI00018219	Transforming growth factor-beta-induced protein ig-h3 precursor	K.VISTITNNIQQIIEIEDTFETLR.A	3	5.81	0.46	-8.15
IPI00018219	Transforming growth factor-beta-induced protein ig-h3 precursor	K.YHIGDEILVSGGIGALVR.L	3	4.90	0.32	-4.19
IPI00018219	Transforming growth factor-beta-induced protein ig-h3 precursor	K.YLYHGQTLETLGGK.K	2	3.91	0.40	-2.93
IPI00018219	Transforming growth factor-beta-induced protein ig-h3 precursor	K.YLYHGQTLETLGGKK.L	2	4.84	0.40	-3.80
IPI00018219	Transforming growth factor-beta-induced protein ig-h3 precursor	R.AAVAASGLNTM*LEGNGQYTLLAPTNEAFEKIPSETLNR.I	3	6.08	0.54	-4.79
IPI00018219	Transforming growth factor-beta-induced protein ig-h3 precursor	R.AAVAASGLNTM*LEGNGQYTLLAPTNEAFEKIPSETLNR.I	4	4.61	0.36	-4.92
IPI00018219	Transforming growth factor-beta-induced protein ig-h3 precursor	R.EGVYTVFAPTNEAFR.A	2	4.53	0.45	-5.44
IPI00018219	Transforming growth factor-beta-induced protein ig-h3 precursor	R.FSM*LVAAIQSAGLTETLNR.E	2	6.62	0.60	-3.15
IPI00018219	Transforming growth factor-beta-induced protein ig-h3 precursor	R.FSM*LVAAIQSAGLTETLNR.E	3	5.35	0.50	-4.04

IPI00018219	Transforming growth factor-beta-induced protein ig-h3 precursor	R.GDELADSALEIFK.Q	2	4.67	0.42	-3.87
IPI00018219	Transforming growth factor-beta-induced protein ig-h3 precursor	R.ILGDPEALRDLLNNHILK.S	2	3.11	0.30	-4.32
IPI00018219	Transforming growth factor-beta-induced protein ig-h3 precursor	R.ILGDPEALRDLLNNHILK.S	3	3.13	0.36	-3.77
IPI00018219	Transforming growth factor-beta-induced protein ig-h3 precursor	R.LKSLQGDKLEVSLK.N	2	4.63	0.40	-3.60
IPI00018219	Transforming growth factor-beta-induced protein ig-h3 precursor	R.LKSLQGDKLEVSLK.N	3	2.78	0.36	-1.94
IPI00018219	Transforming growth factor-beta-induced protein ig-h3 precursor	R.LLGDAKELANILK.Y	2	3.07	0.14	
IPI00018219	Transforming growth factor-beta-induced protein ig-h3 precursor	R.LTLLAPLNSVFK.D	1	2.70	0.35	-4.18
IPI00018219	Transforming growth factor-beta-induced protein ig-h3 precursor	R.LTLLAPLNSVFK.D	2	3.43	0.25	-4.54
IPI00018219	Transforming growth factor-beta-induced protein ig-h3 precursor	R.LTLLAPLNSVFKDGTPPIDAHTR.N	3	3.39	0.41	-2.14
IPI00018219	Transforming growth factor-beta-induced protein ig-h3 precursor	R.LTLLAPLNSVFKDGTPPIDAHTR.N	4	3.21	0.23	-2.14
IPI00018219	Transforming growth factor-beta-induced protein ig-h3 precursor	R.NHIIKDQLASK.Y	2	2.78	0.23	-3.59
IPI00018219	Transforming growth factor-beta-induced protein ig-h3 precursor	R.QAGLGNHLSGSER.L	1	1.29	0.11	-0.98
IPI00018219	Transforming growth factor-beta-induced protein ig-h3 precursor	R.VLTDELK.H	2	2.30	0.12	-3.92
IPI00018219	Transforming growth factor-beta-induced protein ig-h3 precursor	R.VLTPPM*GTVM*DVLK.G	2	2.50	0.35	-3.04
IPI00018219	Transforming growth factor-beta-induced protein ig-h3 precursor	R.VLTPPM*GTVM*DVLKGDNR.F	2	3.54	0.31	-1.17
IPI00018219	Transforming growth factor-beta-induced protein ig-h3 precursor	R.VLTPPM*GTVM*DVLKGDNR.F	3	2.60	0.12	-2.55
IPI00018219	Transforming growth factor-beta-induced protein ig-h3 precursor	R.YGTLFTM*DR.V	2	3.31	0.27	-2.95
IPI00018236	Ganglioside GM2 activator precursor	K.EGTYSLPK.S	2	1.43	0.14	-2.12
IPI00018236	Ganglioside GM2 activator precursor	K.EVAGLWIK.I	1	2.40	0.17	-2.10
IPI00018236	Ganglioside GM2 activator precursor	K.EVAGLWIK.I	2	2.50	0.19	-2.42
IPI00018236	Ganglioside GM2 activator precursor	K.IAASLKGI.-	1	1.53	0.11	-3.25
IPI00018236	Ganglioside GM2 activator precursor	K.IPCTDYIGSCTFEHFCDVLDM*LIPTGEPCPEPLR.T	3	5.98	0.59	-3.92
IPI00018236	Ganglioside GM2 activator precursor	K.IPCTDYIGSCTFEHFCDVLDM*LIPTGEPCPEPLR.T	4	3.97	0.42	-4.38
IPI00018236	Ganglioside GM2 activator precursor	K.IPCTDYIGSCTFEHFCDVLDM*LIPTGEPCPEPLR.T	5	2.28	0.20	-3.22
IPI00018236	Ganglioside GM2 activator precursor	K.KPSQLSSFSWDNCDEGKDPAVIR.S	4	3.63	0.30	-3.09

IPI00018236	Ganglioside GM2 activator precursor	K.PSQLSSFSWDNCDEGKDPAVIR.S	3	3.87	0.37	-3.19
IPI00018236	Ganglioside GM2 activator precursor	K.SEFVVPDLELPSWLTTGNYR.I	2	5.44	0.59	-5.02
IPI00018236	Ganglioside GM2 activator precursor	K.SEFVVPDLELPSWLTTGNYR.I	3	4.99	0.53	-4.69
IPI00018236	Ganglioside GM2 activator precursor	K.VDLVLEK.E	2	2.69	0.13	-3.46
IPI00018236	Ganglioside GM2 activator precursor	K.VDLVLEKEVAGLWIK.I	2	3.43	0.31	-1.54
IPI00018236	Ganglioside GM2 activator precursor	K.VDLVLEKEVAGLWIK.I	3	4.18	0.32	-0.86
IPI00018236	Ganglioside GM2 activator precursor	R.IESVLSSSGK.R	1	2.36	0.14	-3.73
IPI00018236	Ganglioside GM2 activator precursor	R.IESVLSSSGK.R	2	3.41	0.26	-1.53
IPI00018236	Ganglioside GM2 activator precursor	R.IESVLSSSGKR.L	1	2.60	0.32	-4.05
IPI00018236	Ganglioside GM2 activator precursor	R.IESVLSSSGKR.L	2	3.39	0.24	-5.17
IPI00018236	Ganglioside GM2 activator precursor	R.TYGLPCHCPFK.E	2	2.53	0.36	-0.83
IPI00018246	Isoform 1 of Hexokinase-1	K.FLSQIESDRLALLQVR.A	3	3.11	0.30	-2.75
IPI00018246	Isoform 1 of Hexokinase-1	R.SANLVAATLGAILNR.L	2	4.12	0.15	-5.03
IPI00018246	Isoform 1 of Hexokinase-1	R.SANLVAATLGAILNR.L	3	4.67	0.21	-2.97
IPI00018274	Isoform 1 of Epidermal growth factor receptor precursor	K.LTQLGTFEDHFLSLQR.M	2	4.41	0.48	-2.06
IPI00018274	Isoform 1 of Epidermal growth factor receptor precursor	K.LTQLGTFEDHFLSLQR.M	3	2.99	0.29	-1.87
IPI00018274	Isoform 1 of Epidermal growth factor receptor precursor	K.TIQEVAGYVLIALNTVER.I	2	4.17	0.31	-2.79
IPI00018274	Isoform 1 of Epidermal growth factor receptor precursor	K.TIQEVAGYVLIALNTVER.I	3	4.33	0.23	-2.27
IPI00018274	Isoform 1 of Epidermal growth factor receptor precursor	R.FSNNPALCNVESIQWR.D	2	4.57	0.44	-3.98
IPI00018274	Isoform 1 of Epidermal growth factor receptor precursor	R.GDSFTHTPPLDPQELDILK.T	3	4.14	0.19	-2.11
IPI00018274	Isoform 1 of Epidermal growth factor receptor precursor	R.NLQEILHGAVR.F	2	2.96	0.26	-4.51
IPI00018275	Prion-like protein doppel precursor	R.LVQELCSLK.H	2	2.34	0.22	-0.57
IPI00018305	Insulin-like growth factor-binding protein 3 precursor	A.GASSAGLGPVVR.C	2	3.40	0.39	-1.51
IPI00018305	Insulin-like growth factor-binding protein 3 precursor	K.FLNVLSPR.G	2	2.63	0.15	-1.86
IPI00018305	Insulin-like growth factor-binding protein 3 precursor	K.YGQPLPGYTTK.G	2	2.13	0.15	-2.79
IPI00018305	Insulin-like growth factor-binding protein 3 precursor	R.ALAQCAPPPAVCAELVR.E	3	3.57	0.33	-2.64
IPI00018305	Insulin-like growth factor-binding protein 3 precursor	R.SAGSVESPSVSSTHR.V	2	2.78	0.27	-5.24
IPI00018311	Isoform 2 of Neuroplastin precursor	K.NGVELSATR.K	2	3.02	0.34	-1.46
IPI00018342	Adenylate kinase isoenzyme 1	K.YGYTHLSTGDLLR.S	3	2.35	0.22	-1.01
IPI00018352	Ubiquitin carboxyl-terminal hydrolase isozyme L1	R.VDDKVNFHFILFNNVDGHLYELDGR.M	4	2.80	0.13	-2.50

IPI00018381	Isoform 1 of Tolloid-like protein 1 precursor	K.FCGAEVPEVITSQFNNMRIEFKSDNTVSK.K	3	4.00	0.12	
IPI00018381	Isoform 1 of Tolloid-like protein 1 precursor	K.GKVPLQFSGQNEK.N	2	3.72	0.30	-1.94
IPI00018381	Isoform 1 of Tolloid-like protein 1 precursor	K.GKVPLQFSGQNEK.N	3	3.81	0.24	-3.32
IPI00018381	Isoform 1 of Tolloid-like protein 1 precursor	K.VPLQFSGQNEK.N	2	2.95	0.11	-1.46
IPI00018387	Furin precursor	R.ESPPQQQPPRLPPEVEAGQR.L	3	2.50	0.12	-1.63
IPI00018396	Cerebellin-4 precursor	K.CLVVCDSNPATDSK.G	2	4.26	0.43	-3.66
IPI00018396	Cerebellin-4 precursor	K.CLVVCDSNPATDSKGSSSSPLGISVR.A	3	4.52	0.48	-4.40
IPI00018396	Cerebellin-4 precursor	K.GSSSSPLGISVR.A	1	2.21	0.38	-3.84
IPI00018396	Cerebellin-4 precursor	K.GSSSSPLGISVR.A	2	3.79	0.38	-1.91
IPI00018429	Paired mesoderm homeobox protein 2	R.KNFSVSHLLDLEEVAAAGRLAARPGAR.A	3	3.11	0.17	
IPI00018534	Histone H2B type 1-L	K.AM*GIM*NSFVNDIFER.I	2	4.45	0.47	-5.10
IPI00018534	Histone H2B type 1-L	K.AMGIMNSFVNDIFER.I	2	4.14	0.47	-3.32
IPI00018534	Histone H2B type 1-L	K.AQKKDGKKR.K	2	2.40	0.14	-2.43
IPI00018534	Histone H2B type 1-L	R.LLLPGELAK.H	2	2.25	0.08	-2.29
IPI00018534	Histone H2B type 1-L	R.SRKESYSVYVYKVLK.Q	3	3.27	0.34	-2.25
IPI00018534	Histone H2B type 1-L	R.SRKESYSVYVYKVLK.Q	4	3.14	0.17	-2.98
IPI00018534	Histone H2B type 1-L	R.SRKESYSVYVYKVLKQVHPDTGISSK.A	5	3.64	0.29	-4.59
IPI00018708	Isoform 2 of Centrosomal protein of 63 kDa	K.LENRHLSEM*VMK.L	2	1.50	0.11	-3.64
IPI00018755	High mobility group protein 1-like 10	K.IKGEHPGLSIGDVAK.K	2	3.29	0.41	-3.40
IPI00018769	Thrombospondin-2 precursor	K.DKTHNCHKHAECIYLGHFSDPMYK.C	3	2.79	0.10	0.68
IPI00018769	Thrombospondin-2 precursor	K.DYTAYR.W	1	1.52	0.19	-1.61
IPI00018769	Thrombospondin-2 precursor	K.QVM*ADSGPIYDQTYAGGR.L	2	5.52	0.55	-3.67
IPI00018769	Thrombospondin-2 precursor	R.AYGYSGVSLK.V	2	2.83	0.29	-2.42
IPI00018769	Thrombospondin-2 precursor	R.FDYIPPVNADDLSK.I	2	2.30	0.09	-2.46
IPI00018769	Thrombospondin-2 precursor	R.GNQPVGVGLEAAK.T	2	2.22	0.22	-1.57
IPI00018769	Thrombospondin-2 precursor	R.LCNSPVPQM*GGK.N	2	2.77	0.34	-3.07
IPI00018769	Thrombospondin-2 precursor	R.NFQM*VPLDPK.G	2	3.07	0.28	-2.99
IPI00018769	Thrombospondin-2 precursor	R.YRGNQPVGVGLEAAK.T	2	4.02	0.38	-1.91
IPI00018803	homeobox D12	L.QPPTAKDGPEEQAK.F	1	1.92	0.23	-0.44
IPI00018843	Isoform 1 of D(3) dopamine receptor	K.LSNGRLSTSLKLGPLQPRGVPLR.E	2	1.23	0.17	1.68
IPI00018860	NKG2D ligand 2 precursor	R.DIQLENYTPK.E	2	2.71	0.20	-4.51
IPI00018860	NKG2D ligand 2 precursor	R.DIQLENYTPKEPLTLQAR.M	2	4.52	0.39	-2.18
IPI00018860	NKG2D ligand 2 precursor	R.DIQLENYTPKEPLTLQAR.M	3	4.39	0.27	-4.27
IPI00018860	NKG2D ligand 2 precursor	R.EVVDILTEQLR.D	2	4.32	0.35	-2.57
IPI00018879	Alpha-L-iduronidase precursor	R.ALDYWARPGPFSDPVPYLEVPVPR.G	3	5.21	0.44	-3.35
IPI00018879	Alpha-L-iduronidase precursor	R.KPSTFNLFVFSPDTGAVSGSYR.V	3	3.51	0.26	-2.95
IPI00018909	trefoil factor 3 precursor	R.IPGVPWCFKPLQEAECTF.-	2	3.86	0.38	-6.79
IPI00018914	Tyrosine-protein phosphatase non-receptor type 14	R.YQYYLQVKKDVLEGR.L	3	2.60	0.10	-3.52
IPI00018941	Calcitonin gene-related peptide 2 precursor	K.ASELKQEQETQGSSSAAQ.K	2	5.68	0.58	-7.24
IPI00018941	Calcitonin gene-related peptide 2 precursor	K.ASELKQEQETQGSSSAAQKR.A	4	3.38	0.20	-1.72
IPI00018941	Calcitonin gene-related peptide 2 precursor	R.LLLAALVQDYVQM*K.A	2	4.72	0.45	-3.61

IPI00018941	Calcitonin gene-related peptide 2 precursor	R.LLLAALVQDYVQM*K.A	3	5.28	0.48	-4.39
IPI00018980	Sodium channel subunit beta-1 precursor	R.YENEVLQLEEDERFEGR.V	3	2.61	0.20	-2.91
IPI00019038	Lysozyme C precursor	K.TPGAVNACHLSCSALLQDNIADAVACAK.R	2	4.44	0.50	
IPI00019038	Lysozyme C precursor	K.TPGAVNACHLSCSALLQDNIADAVACAK.R	3	4.56	0.17	
IPI00019038	Lysozyme C precursor	R.ATNYNAGDRSTDYGIFQINSR.Y	2	4.69	0.37	
IPI00019038	Lysozyme C precursor	R.ATNYNAGDRSTDYGIFQINSR.Y	3	2.32	0.24	
IPI00019038	Lysozyme C precursor	R.GISLANWM*CLAK.W	2	3.55	0.38	
IPI00019038	Lysozyme C precursor	R.GISLANWMCLAK.W	2	1.98	0.19	
IPI00019038	Lysozyme C precursor	R.LGM*DGYR.G	2	2.25	0.10	
IPI00019038	Lysozyme C precursor	R.STDYGIFQINSR.Y	2	4.53	0.34	
IPI00019038	Lysozyme C precursor	R.YWCNDGKTPGAVNACHLSCSALLQDNIADAVACAK.R	3	5.61	0.47	
IPI00019146	Isoform 1 of Coxsackievirus and adenovirus receptor precursor	K.FTLSPEDQGPLDIEWLISPADNQK.V	3	2.55	0.11	-3.47
IPI00019157	Chondroitin sulfate proteoglycan 4 precursor	K.DLLFGSIVAVDEPTRPIYR.F	3	2.83	0.15	-2.87
IPI00019157	Chondroitin sulfate proteoglycan 4 precursor	K.SPPSAGYLVM*VSR.G	2	3.15	0.29	-0.75
IPI00019157	Chondroitin sulfate proteoglycan 4 precursor	K.SQVLFSVTR.G	2	2.80	0.11	-2.48
IPI00019157	Chondroitin sulfate proteoglycan 4 precursor	R.APLEVPQALGR.S	2	2.86	0.35	-2.64
IPI00019157	Chondroitin sulfate proteoglycan 4 precursor	R.ASSSAGTDPQLLLYR.V	2	3.80	0.36	-3.96
IPI00019157	Chondroitin sulfate proteoglycan 4 precursor	R.DQLEAAQEAVPPADIVFSVK.S	2	4.10	0.50	-2.06
IPI00019157	Chondroitin sulfate proteoglycan 4 precursor	R.DQPGEPATEFSCR.E	2	2.11	0.05	-2.63
IPI00019157	Chondroitin sulfate proteoglycan 4 precursor	R.DVNERPPQPQASVPLR.L	3	2.50	0.22	-2.12
IPI00019157	Chondroitin sulfate proteoglycan 4 precursor	R.ELEAGSLVYVHR.G	3	1.90	0.14	-1.82
IPI00019157	Chondroitin sulfate proteoglycan 4 precursor	R.FTQADVDSGR.L	2	2.56	0.26	0.48
IPI00019157	Chondroitin sulfate proteoglycan 4 precursor	R.GSLLLGGLDAEASR.H	2	3.86	0.36	-4.07
IPI00019157	Chondroitin sulfate proteoglycan 4 precursor	R.GVLSYLEPR.G	2	2.80	0.12	-0.31
IPI00019157	Chondroitin sulfate proteoglycan 4 precursor	R.LEISVDQYPTHTSNR.G	2	4.12	0.50	-1.97
IPI00019157	Chondroitin sulfate proteoglycan 4 precursor	R.LEISVDQYPTHTSNR.G	3	2.72	0.15	-1.97
IPI00019157	Chondroitin sulfate proteoglycan 4 precursor	R.LSDGEHTSPGHFFR.V	2	3.39	0.20	-3.95
IPI00019157	Chondroitin sulfate proteoglycan 4 precursor	R.LSDGQGFTQDDIQAGR.V	2	3.26	0.37	-2.60
IPI00019157	Chondroitin sulfate proteoglycan 4 precursor	R.QAPLAFQAGGR.R	2	2.08	0.28	-1.92
IPI00019157	Chondroitin sulfate proteoglycan 4 precursor	R.SGDEVHYHVTAGPR.W	2	4.29	0.46	-3.85
IPI00019157	Chondroitin sulfate proteoglycan 4 precursor	R.SGDEVHYHVTAGPR.W	3	2.75	0.25	-1.70
IPI00019157	Chondroitin sulfate proteoglycan 4 precursor	R.STDGDSGSEDLVYTIEQPSNGR.V	2	3.85	0.41	-4.05
IPI00019157	Chondroitin sulfate proteoglycan 4 precursor	R.VAIQPVNDHAPVQTISR.I	2	3.01	0.37	-3.87
IPI00019157	Chondroitin sulfate proteoglycan 4 precursor	R.VSGPYFPTLLGLSLQVLEPPQHGALQKEDGPQAR.T	4	4.02	0.33	-1.81
IPI00019157	Chondroitin sulfate proteoglycan 4 precursor	R.VTGALQFGELQK.Q	2	4.22	0.39	-4.68
IPI00019158	ADAM metallopeptidase domain 8 precursor	K.QVIKPTFAPPVPPVKPGAGAANPGPAEGAVGPK.V	3	2.98	0.17	
IPI00019176	Retinoic acid receptor responder protein 2 precursor	K.LGSEDKVLGR.L	2	3.90	0.19	-2.41
IPI00019176	Retinoic acid receptor responder protein 2 precursor	R.AGEDPHSFYFPGQFAFSK.A	3	3.71	0.40	-3.03

IPI00019176	Retinoic acid receptor responder protein 2 precursor	R.GLQVALEEFHK.H	3	2.07	0.13	-3.18
IPI00019180	Glypican-5 precursor	K.VKGIDPVINQIIDKLK.H	3	3.24	0.30	-3.29
IPI00019180	Glypican-5 precursor	K.VKGIDPVINQIIDKLK.H	4	3.10	0.30	-3.11
IPI00019180	Glypican-5 precursor	R.NAAAFQETLETLIK.Q	2	4.21	0.35	-4.08
IPI00019190	Myocilin precursor	R.IDTVGTDVR.Q	2	2.17	0.21	-3.08
IPI00019190	Myocilin precursor	R.ILKESPSGYLR.S	2	2.24	0.08	-3.57
IPI00019190	Myocilin precursor	R.LRQENENLAR.R	2	3.04	0.17	-1.05
IPI00019190	Myocilin precursor	R.RLESSSQEVAR.L	2	3.02	0.22	-0.99
IPI00019190	Myocilin precursor	R.TAETITGK.Y	2	1.69	0.13	-2.09
IPI00019208	Similar to 60S ribosomal protein L29	K.HNKKGLKKM*QANNAK.A	2	3.83	0.11	
IPI00019209	Semaphorin-3C precursor	K.TSEYFSLSHHPLDYR.I	4	2.68	0.18	-2.84
IPI00019209	Semaphorin-3C precursor	R.ILLM*DEDQDR.I	2	1.71	0.16	-1.24
IPI00019209	Semaphorin-3C precursor	R.VYLTFDELR.E	2	3.03	0.30	-3.78
IPI00019209	Semaphorin-3C precursor	R.YHVLFLGTDR.G	2	2.04	0.10	-2.20
IPI00019242	Matrix metalloproteinase-15 precursor	R.VVVQM*EEVAR.T	2	2.22	0.11	-1.38
IPI00019359	Keratin, type I cytoskeletal 9	K.DIENQYETQITQIEHEVSSSGQEVQSSAK.E	3	4.27	0.25	
IPI00019359	Keratin, type I cytoskeletal 9	K.NYSPYYNTIDDLKDQIVDLTVGNNK.T	3	5.07	0.45	
IPI00019359	Keratin, type I cytoskeletal 9	R.FSSSSGYGGGSSR.V	2	3.35	0.39	-0.91
IPI00019359	Keratin, type I cytoskeletal 9	R.GGSGGSYGGGGSGGGYGGGSGSR.G	2	5.66	0.46	-3.87
IPI00019372	Serglycin precursor	K.GPM*FELLPGESNKIPR.L	3	3.27	0.32	-3.32
IPI00019399	Serum amyloid A-4 protein precursor	K.EALQGVGDMGR.A	2	3.30	0.27	
IPI00019399	Serum amyloid A-4 protein precursor	R.AYWDIM*ISNHQNSNR.Y	2	3.82	0.38	
IPI00019399	Serum amyloid A-4 protein precursor	R.AYWDIMISNHQNSNR.Y	2	4.20	0.41	
IPI00019399	Serum amyloid A-4 protein precursor	R.GNYDAAQRGPGGVWAAK.L	2	3.93	0.10	
IPI00019399	Serum amyloid A-4 protein precursor	R.GNYDAAQRGPGGVWAAK.L	3	2.86	0.20	
IPI00019399	Serum amyloid A-4 protein precursor	R.SFFKEALQGVGDM*GR.A	2	4.23	0.41	
IPI00019399	Serum amyloid A-4 protein precursor	R.VYLQGLIDYYLFGNSSTVLEDSK.S	2	6.04	0.50	
IPI00019399	Serum amyloid A-4 protein precursor	R.VYLQGLIDYYLFGNSSTVLEDSK.S	3	5.93	0.50	
IPI00019399	Serum amyloid A-4 protein precursor	R.VYLQGLIDYYLFGNSSTVLEDSKSNEK.A	3	4.46	0.22	
IPI00019449	Non-secretory ribonuclease precursor	R.DPPQYPVVPVHLDR.I	3	2.89	0.43	-2.19
IPI00019449	Non-secretory ribonuclease precursor	R.RDPPQYPVVPVHLDR.I	2	2.58	0.17	-3.08
IPI00019449	Non-secretory ribonuclease precursor	R.RDPPQYPVVPVHLDR.I	3	4.49	0.31	-3.31
IPI00019449	Non-secretory ribonuclease precursor	R.RDPPQYPVVPVHLDR.I	4	2.33	0.22	-1.97
IPI00019449	Non-secretory ribonuclease precursor	R.YAQTPANM*FYIVACDNR.D	2	4.85	0.53	-1.93
IPI00019485	Isoform 2 of Enoyl-CoA hydratase domain-containing protein 2, mitochondrial precursor	R.GTEVDIASGMAIEGMCYAQNIPTR.D	3	5.13	0.12	
IPI00019501	Ephrin-B3 precursor	K.LYLVGGAQGR.R	2	3.45	0.28	-0.90
IPI00019501	Ephrin-B3 precursor	R.FQAEGGYVLYPQIGDRLDLLCPR.A	3	4.43	0.45	-1.05
IPI00019501	Ephrin-B3 precursor	R.KPVSEM*PM*ER.D	2	2.56	0.32	-1.17
IPI00019501	Ephrin-B3 precursor	R.SHHDYYIIATSDGTR.E	3	3.08	0.06	-3.94
IPI00019502	Myosin-9	K.LQVELDNVTGLLSQSDSK.S	2	5.67	0.57	-4.52

IPI00019502	Myosin-9	K.SMEAEMIQLQEELAAAER.A	2	3.75	0.35	-1.25
IPI00019502	Myosin-9	R.IAEFTTNLTEEEEK.S	2	4.34	0.40	-1.51
IPI00019502	Myosin-9	R.IAQLEEELEEEQGNTELINDR.L	2	5.34	0.49	-2.00
IPI00019502	Myosin-9	R.IAQLEEELEEEQGNTELINDR.L	3	4.82	0.47	-1.79
IPI00019502	Myosin-9	R.LTEMETLQSQLMAEK.L	2	3.43	0.24	-4.94
IPI00019502	Myosin-9	R.NTDQASM*PDNTAAQK.V	2	2.86	0.37	-3.01
IPI00019530	Tyrosine-protein kinase receptor Tie-1 precursor	K.AIVEPEKTTAEFEVPR.L	2	3.31	0.41	-1.56
IPI00019530	Tyrosine-protein kinase receptor Tie-1 precursor	K.AIVEPEKTTAEFEVPR.L	3	3.74	0.33	-2.14
IPI00019533	Chitinase-3-like protein 2 precursor	K.DKSEVM*LYQTINSLK.T	2	3.74	0.37	-1.41
IPI00019533	Chitinase-3-like protein 2 precursor	K.DKSEVM*LYQTINSLK.T	3	4.22	0.33	-1.38
IPI00019533	Chitinase-3-like protein 2 precursor	K.ENTHFTVLIHELAEAFQK.D	3	2.02	0.17	-3.10
IPI00019533	Chitinase-3-like protein 2 precursor	K.GNQWVGYDDVK.S	2	2.83	0.27	-2.72
IPI00019533	Chitinase-3-like protein 2 precursor	K.SCNQGPYPLVQAVK.R	2	4.42	0.43	-2.24
IPI00019533	Chitinase-3-like protein 2 precursor	R.LQDQQVPYAVK.G	2	3.79	0.30	-3.26
IPI00019568	Prothrombin precursor (Fragment)	K.ETWTANVGK.G	1	1.90	0.19	-3.33
IPI00019568	Prothrombin precursor (Fragment)	K.HQDFNSAVQLVENFCR.N	2	5.78	0.62	-5.34
IPI00019568	Prothrombin precursor (Fragment)	K.HQDFNSAVQLVENFCR.N	3	2.88	0.17	-3.59
IPI00019568	Prothrombin precursor (Fragment)	K.KSLEDKTERELLESYIDGR.I	3	3.45	0.38	-3.60
IPI00019568	Prothrombin precursor (Fragment)	K.KSLEDKTERELLESYIDGR.I	4	3.16	0.21	-3.29
IPI00019568	Prothrombin precursor (Fragment)	K.SLEDKTER.E	2	2.45	0.18	-2.11
IPI00019568	Prothrombin precursor (Fragment)	K.SLEDKTERELLESYIDGR.I	2	3.79	0.46	-4.15
IPI00019568	Prothrombin precursor (Fragment)	K.SLEDKTERELLESYIDGR.I	3	3.85	0.40	-4.39
IPI00019568	Prothrombin precursor (Fragment)	K.SPQELLCGASLISDR.W	2	5.15	0.54	-1.87
IPI00019568	Prothrombin precursor (Fragment)	K.SPQELLCGASLISDR.W	3	2.67	0.23	-1.60
IPI00019568	Prothrombin precursor (Fragment)	K.TERELLESYIDGR.I	2	2.93	0.35	-3.21
IPI00019568	Prothrombin precursor (Fragment)	K.TERELLESYIDGR.I	3	3.08	0.20	-2.24
IPI00019568	Prothrombin precursor (Fragment)	K.VIDQFGE.-	1	1.63	0.14	-3.57
IPI00019568	Prothrombin precursor (Fragment)	K.YGFYTHVFR.L	1	2.22	0.22	-3.01
IPI00019568	Prothrombin precursor (Fragment)	K.YGFYTHVFR.L	2	3.68	0.47	-2.52
IPI00019568	Prothrombin precursor (Fragment)	K.YTACETAR.T	2	2.76	0.32	-2.85
IPI00019568	Prothrombin precursor (Fragment)	R.DGKYGFYTHVFR.L	2	3.58	0.49	-2.81
IPI00019568	Prothrombin precursor (Fragment)	R.DGKYGFYTHVFR.L	3	2.98	0.21	-2.65
IPI00019568	Prothrombin precursor (Fragment)	R.DKLAACLEGNCAEGLGTNYR.G	3	4.50	0.49	-2.49
IPI00019568	Prothrombin precursor (Fragment)	R.ELLESYIDGR.I	1	2.72	0.18	-2.39
IPI00019568	Prothrombin precursor (Fragment)	R.ELLESYIDGR.I	2	3.74	0.37	-6.28
IPI00019568	Prothrombin precursor (Fragment)	R.ENLDRDIALM*K.L	1	2.03	0.33	-0.09
IPI00019568	Prothrombin precursor (Fragment)	R.ENLDRDIALM*K.L	2	2.93	0.21	-2.27
IPI00019568	Prothrombin precursor (Fragment)	R.ENLDRDIALM*K.L	3	2.55	0.22	-2.53
IPI00019568	Prothrombin precursor (Fragment)	R.ETAASLLQAGYK.G	1	3.60	0.43	-3.23
IPI00019568	Prothrombin precursor (Fragment)	R.ETAASLLQAGYK.G	2	4.19	0.44	-2.74
IPI00019568	Prothrombin precursor (Fragment)	R.GDACEGDSGGPFVM*K.S	2	4.12	0.51	-4.22
IPI00019568	Prothrombin precursor (Fragment)	R.IVEGSDAEIGM*SPWQVM*LFR.K	2	6.22	0.57	-5.02

IPI00019568	Prothrombin precursor (Fragment)	R.IVEGSDAEIGM*SPWQVM*LFR.K	3	5.78	0.52	-4.56
IPI00019568	Prothrombin precursor (Fragment)	R.KSPQELLCGASLISDR.W	2	5.04	0.54	-2.77
IPI00019568	Prothrombin precursor (Fragment)	R.KSPQELLCGASLISDR.W	3	3.83	0.42	-2.59
IPI00019568	Prothrombin precursor (Fragment)	R.LAVTTHGLPCLAWASAQAK.A	2	5.35	0.59	-3.19
IPI00019568	Prothrombin precursor (Fragment)	R.LAVTTHGLPCLAWASAQAK.A	3	5.17	0.46	-3.86
IPI00019568	Prothrombin precursor (Fragment)	R.NPDSSTTGPWCYTTDPTVR.R	2	5.47	0.62	-4.86
IPI00019568	Prothrombin precursor (Fragment)	R.NPDSSTTGPWCYTTDPTVR.R	3	4.29	0.43	-5.36
IPI00019568	Prothrombin precursor (Fragment)	R.QECSIPVCGQDQVTVAM*TPR.S	2	4.66	0.54	-3.45
IPI00019568	Prothrombin precursor (Fragment)	R.RQECSIPVCGQDQVTVAM*TPR.S	3	5.62	0.50	-3.85
IPI00019568	Prothrombin precursor (Fragment)	R.RQECSIPVCGQDQVTVAMTPR.S	3	3.84	0.26	
IPI00019568	Prothrombin precursor (Fragment)	R.SEGSSVNLSPPLEQCVPDR.G	2	4.04	0.32	-5.53
IPI00019568	Prothrombin precursor (Fragment)	R.SEGSSVNLSPPLEQCVPDR.G	3	3.15	0.18	-3.60
IPI00019568	Prothrombin precursor (Fragment)	R.SEGSSVNLSPPLEQCVPDRGQQYQGR.L	2	2.23	0.29	-1.95
IPI00019568	Prothrombin precursor (Fragment)	R.SEGSSVNLSPPLEQCVPDRGQQYQGR.L	3	3.64	0.37	-3.96
IPI00019568	Prothrombin precursor (Fragment)	R.SEGSSVNLSPPLEQCVPDRGQQYQGR.L	4	3.49	0.24	-1.99
IPI00019568	Prothrombin precursor (Fragment)	R.SGIECQLWR.S	1	2.04	0.13	-3.56
IPI00019568	Prothrombin precursor (Fragment)	R.SGIECQLWR.S	2	3.30	0.22	-2.21
IPI00019568	Prothrombin precursor (Fragment)	R.TATSEYQTFFNPR.T	1	3.04	0.41	-3.35
IPI00019568	Prothrombin precursor (Fragment)	R.TATSEYQTFFNPR.T	2	4.10	0.44	-3.20
IPI00019568	Prothrombin precursor (Fragment)	R.TATSEYQTFFNPR.T	3	2.52	0.39	-1.42
IPI00019576	Coagulation factor X precursor	K.ACIPTGPYPCGK.Q	2	3.01	0.38	-2.95
IPI00019576	Coagulation factor X precursor	K.ETYDFDIAVLR.L	2	3.03	0.32	-3.13
IPI00019576	Coagulation factor X precursor	K.M*LEVPYVDR.N	1	1.66	0.13	-3.40
IPI00019576	Coagulation factor X precursor	K.M*LEVPYVDR.N	2	3.06	0.17	-3.50
IPI00019576	Coagulation factor X precursor	K.TGIVSGFGR.T	1	1.88	0.20	-3.19
IPI00019576	Coagulation factor X precursor	K.TGIVSGFGR.T	2	3.24	0.28	-3.34
IPI00019576	Coagulation factor X precursor	K.YGIYTK.V	1	1.81	0.17	-1.55
IPI00019576	Coagulation factor X precursor	R.DWAESTLM*TQK.T	2	3.52	0.46	-3.45
IPI00019576	Coagulation factor X precursor	R.EQANNILAR.V	2	1.96	0.07	1.07
IPI00019576	Coagulation factor X precursor	R.FTKETYDFDIAVLR.L	3	2.77	0.27	-0.90
IPI00019576	Coagulation factor X precursor	R.GYTLADNGK.A	1	2.15	0.22	-3.59
IPI00019576	Coagulation factor X precursor	R.LKM*LEVPYVDR.N	3	2.45	0.16	-2.32
IPI00019576	Coagulation factor X precursor	R.M*NVAPACLPER.D	2	2.85	0.22	-2.54
IPI00019576	Coagulation factor X precursor	R.NTEQEEGGEAVHEVEVVIK.H	2	4.92	0.44	-4.32
IPI00019576	Coagulation factor X precursor	R.NTEQEEGGEAVHEVEVVIK.H	3	2.83	0.17	-4.27
IPI00019580	Plasminogen precursor	G.EPLDDYVNTQGASLFSVTK.K	2	4.00	0.46	-2.17
IPI00019580	Plasminogen precursor	G.EPLDDYVNTQGASLFSVTKK.Q	2	3.40	0.52	-2.62
IPI00019580	Plasminogen precursor	K.CQSWSSM*TPHR.H	2	2.49	0.16	
IPI00019580	Plasminogen precursor	K.CQSWSSM*TPHR.H	3	3.09	0.19	
IPI00019580	Plasminogen precursor	K.CSGTEASVVAPPPVVLLPDVETPSEEDCM*FGNGK.G	3	5.03	0.34	
IPI00019580	Plasminogen precursor	K.EAQLPVIENK.V	1	2.80	0.28	-2.54
IPI00019580	Plasminogen precursor	K.EAQLPVIENK.V	2	2.77	0.28	-1.61

IPI00019580	Plasminogen precursor	K.EQQCVIM*AENR.K	2	2.37	0.33	-2.94
IPI00019580	Plasminogen precursor	K.EQQCVIM*AENRK.S	2	2.82	0.28	
IPI00019580	Plasminogen precursor	K.KCSGTEASVVAPPPVVLLPDVETPSEEDCM*FGNGK.G	3	4.81	0.26	
IPI00019580	Plasminogen precursor	K.KQLGAGSIEECAAKCEEDEEFTCR.A	3	5.16	0.24	
IPI00019580	Plasminogen precursor	K.LSSPAVITDK.V	1	1.82	0.13	-3.60
IPI00019580	Plasminogen precursor	K.LSSPAVITDK.V	2	2.13	0.18	
IPI00019580	Plasminogen precursor	K.LSSPAVITDKVIPACLPSPNYVVADR.T	3	5.05	0.45	-3.08
IPI00019580	Plasminogen precursor	K.LYDYCDVPQCAAPSFDCGKPQVEPK.K	3	3.16	0.13	
IPI00019580	Plasminogen precursor	K.NLDENYCR.N	2	2.08	0.19	-1.29
IPI00019580	Plasminogen precursor	K.NLDENYCRNPDGK.R	2	3.27	0.24	
IPI00019580	Plasminogen precursor	K.NYCRNPDGDVGGPWCYTTNPR.K	2	4.43	0.23	
IPI00019580	Plasminogen precursor	K.NYCRNPDGDVGGPWCYTTNPR.K	3	5.60	0.46	
IPI00019580	Plasminogen precursor	K.QLGAGSIEECAAK.C	2	3.10	0.45	-2.87
IPI00019580	Plasminogen precursor	K.QLGAGSIEECAAKCEEDEEFTCR.A	2	3.77	0.37	
IPI00019580	Plasminogen precursor	K.QLGAGSIEECAAKCEEDEEFTCR.A	3	3.50	0.46	-1.82
IPI00019580	Plasminogen precursor	K.TPENYPNAGLTM*NYCR.N	2	3.76	0.30	
IPI00019580	Plasminogen precursor	K.VCNRYEFLNGR.V	2	3.73	0.34	
IPI00019580	Plasminogen precursor	K.VCNRYEFLNGR.V	3	3.70	0.12	
IPI00019580	Plasminogen precursor	K.VILGAHQEVNLEPHVQEIEVSR.L	2	4.13	0.20	
IPI00019580	Plasminogen precursor	K.VILGAHQEVNLEPHVQEIEVSR.L	3	5.34	0.40	-2.97
IPI00019580	Plasminogen precursor	K.VILGAHQEVNLEPHVQEIEVSR.L	4	1.85	0.21	-3.29
IPI00019580	Plasminogen precursor	K.VIPACLPSPNYVVADR.T	2	3.58	0.38	
IPI00019580	Plasminogen precursor	K.VIPACLPSPNYVVADRTECFITGWGETQGTFGAGLLK.E	3	3.25	0.40	
IPI00019580	Plasminogen precursor	K.VYLSECK.T	1	1.70	0.20	-2.43
IPI00019580	Plasminogen precursor	R.ATTVTGTPCQDWAAQEPHR.H	2	4.79	0.53	
IPI00019580	Plasminogen precursor	R.CTTPPPSSGPTYQCLK.G	2	3.50	0.35	-3.13
IPI00019580	Plasminogen precursor	R.ELRPWCFTTDPNKR.W	3	2.92	0.12	
IPI00019580	Plasminogen precursor	R.FGM*HFCGGTLISPEWVLTAAHCLEK.S	3	5.95	0.43	
IPI00019580	Plasminogen precursor	R.FSPATHPSEGLEENYCR.N	2	5.41	0.45	
IPI00019580	Plasminogen precursor	R.FSPATHPSEGLEENYCR.N	3	3.11	0.33	
IPI00019580	Plasminogen precursor	R.FVTWIEGVM*R.N	2	3.76	0.27	-3.40
IPI00019580	Plasminogen precursor	R.HSIFTPETNPR.A	1	2.94	0.30	
IPI00019580	Plasminogen precursor	R.HSIFTPETNPR.A	2	3.10	0.31	-2.53
IPI00019580	Plasminogen precursor	R.KLYDYCDVPQCAAPSFDCGKPQVEPK.K	2	3.65	0.39	
IPI00019580	Plasminogen precursor	R.KLYDYCDVPQCAAPSFDCGKPQVEPK.K	3	5.72	0.48	
IPI00019580	Plasminogen precursor	R.M*RDVVLFEKK.V	2	2.49	0.17	
IPI00019580	Plasminogen precursor	R.NPDADKGPWCFTTDPSVR.W	2	3.15	0.34	-4.03
IPI00019580	Plasminogen precursor	R.NPDADKGPWCFTTDPSVR.W	3	3.23	0.42	-2.44
IPI00019580	Plasminogen precursor	R.NPDGDVGGPWCYTTNPR.K	2	4.92	0.59	-4.48
IPI00019580	Plasminogen precursor	R.NPDNDPQGPWCYTTDPEKR.Y	2	2.32	0.15	
IPI00019580	Plasminogen precursor	R.NPDNDPQGPWCYTTDPEKR.Y	3	2.89	0.28	-1.67
IPI00019580	Plasminogen precursor	R.TECFITGWGETQGTFGAGLLK.E	2	6.02	0.50	

IPI00019580	Plasminogen precursor	R.TECFITGWGETQGTFGAGLLK.E	3	3.99	0.22	
IPI00019580	Plasminogen precursor	R.TPENFPCK.N	2	2.73	0.22	-2.19
IPI00019580	Plasminogen precursor	R.TPENFPCKNLDENYCR.N	3	3.52	0.19	
IPI00019580	Plasminogen precursor	R.VQSTELCAGHLAGGTDSCQGDSGGPLVCFEK.D	3	6.09	0.56	-2.99
IPI00019580	Plasminogen precursor	R.VQSTELCAGHLAGGTDSCQGDSGGPLVCFEKDK.Y	3	5.32	0.40	
IPI00019580	Plasminogen precursor	R.YDYCDILECEEECM*HCSGENYDGK.I	3	4.36	0.30	
IPI00019580	Plasminogen precursor	R.YDYCDILECEEECMHCSGENYDGK.I	3	4.12	0.42	
IPI00019580	Plasminogen precursor	R.YEFLNGR.V	2	2.14	0.13	-3.24
IPI00019580	Plasminogen precursor	W.DSQSPHAHGYIPSKFPNK.N	3	3.57	0.24	-2.13
IPI00019581	Coagulation factor XII precursor	K.CFEPQLLR.F	1	1.87	0.16	-2.83
IPI00019581	Coagulation factor XII precursor	K.CFEPQLLR.F	2	2.95	0.22	-2.63
IPI00019581	Coagulation factor XII precursor	K.GRPGPQPWCATTPNFDQDQR.W	2	2.14	0.18	-2.58
IPI00019581	Coagulation factor XII precursor	L.HEAFSPVSYQHDLALLR.L	3	3.68	0.46	-2.89
IPI00019581	Coagulation factor XII precursor	R.LCHCPVGYTGPFCDVDTK.A	3	5.32	0.34	
IPI00019581	Coagulation factor XII precursor	R.LHEAFSPVSYQHDLALLR.L	2	6.32	0.65	-3.56
IPI00019581	Coagulation factor XII precursor	R.LHEAFSPVSYQHDLALLR.L	3	3.78	0.51	-3.51
IPI00019581	Coagulation factor XII precursor	R.LHEAFSPVSYQHDLALLR.L	4	3.53	0.31	-2.90
IPI00019581	Coagulation factor XII precursor	R.LQEDADGSCALLSPYVQPVCLPSGAAR.P	3	5.28	0.34	-4.10
IPI00019581	Coagulation factor XII precursor	R.NKPGVYTDVAYYLAWIR.E	3	3.25	0.22	-3.92
IPI00019581	Coagulation factor XII precursor	R.NPDNDIRPWCFVLNR.D	3	3.45	0.38	-2.36
IPI00019581	Coagulation factor XII precursor	R.PAPEDLTVVLGQER.R	2	3.85	0.38	-2.15
IPI00019581	Coagulation factor XII precursor	R.TEQAAVAR.C	2	2.11	0.12	-3.65
IPI00019581	Coagulation factor XII precursor	R.TTLSGAPCQPWASEATYR.N	2	5.45	0.49	-2.91
IPI00019581	Coagulation factor XII precursor	R.VVGGLVALR.G	2	3.45	0.23	-3.22
IPI00019591	Isoform 1 of Complement factor B precursor (Fragment)	A.PGYDKVKDISEVVTPR.F	2	4.33	0.46	-2.05
IPI00019591	Isoform 1 of Complement factor B precursor (Fragment)	C.PSGFYPYPVQTR.T	2	2.98	0.34	-2.75
IPI00019591	Isoform 1 of Complement factor B precursor (Fragment)	K.ALFVSEEEK.K	1	2.27	0.27	-2.82
IPI00019591	Isoform 1 of Complement factor B precursor (Fragment)	K.ALFVSEEEK.K	2	3.02	0.24	-2.56
IPI00019591	Isoform 1 of Complement factor B precursor (Fragment)	K.ALFVSEEEKK.L	1	2.75	0.12	-3.07
IPI00019591	Isoform 1 of Complement factor B precursor (Fragment)	K.ALFVSEEEKK.L	2	3.20	0.36	-3.06
IPI00019591	Isoform 1 of Complement factor B precursor (Fragment)	K.CLVNLIEK.V	2	3.07	0.13	-1.64
IPI00019591	Isoform 1 of Complement factor B precursor (Fragment)	K.DISEVVTPR.F	1	2.69	0.22	-3.73
IPI00019591	Isoform 1 of Complement factor B precursor (Fragment)	K.DISEVVTPR.F	2	3.42	0.28	-2.70

IPI00019591	Isoform 1 of Complement factor B precursor (Fragment)	K.DNEQHVFK.V	1	2.42	0.13	-3.09
IPI00019591	Isoform 1 of Complement factor B precursor (Fragment)	K.EAGIPEFYDYDVALIK.L	1	1.10	0.36	-2.70
IPI00019591	Isoform 1 of Complement factor B precursor (Fragment)	K.EAGIPEFYDYDVALIK.L	2	4.70	0.51	-6.61
IPI00019591	Isoform 1 of Complement factor B precursor (Fragment)	K.EAGIPEFYDYDVALIK.L	3	3.92	0.38	-4.27
IPI00019591	Isoform 1 of Complement factor B precursor (Fragment)	K.EELLPAQDIK.A	1	2.17	0.15	-2.57
IPI00019591	Isoform 1 of Complement factor B precursor (Fragment)	K.EELLPAQDIK.A	2	2.30	0.09	-3.35
IPI00019591	Isoform 1 of Complement factor B precursor (Fragment)	K.EKLQDEDLGFL.-	1	2.99	0.22	-3.75
IPI00019591	Isoform 1 of Complement factor B precursor (Fragment)	K.EKLQDEDLGFL.-	2	3.86	0.40	-4.54
IPI00019591	Isoform 1 of Complement factor B precursor (Fragment)	K.EVYIKNGDKK.G	2	2.95	0.16	-2.31
IPI00019591	Isoform 1 of Complement factor B precursor (Fragment)	K.ISVIRPSK.G	2	2.51	0.13	-3.46
IPI00019591	Isoform 1 of Complement factor B precursor (Fragment)	K.KCLVNLIEK.V	2	2.26	0.11	-2.98
IPI00019591	Isoform 1 of Complement factor B precursor (Fragment)	K.KDNEQHVFK.V	1	2.03	0.12	-4.48
IPI00019591	Isoform 1 of Complement factor B precursor (Fragment)	K.KDNEQHVFK.V	2	3.04	0.32	-2.96
IPI00019591	Isoform 1 of Complement factor B precursor (Fragment)	K.KEAGIPEFYDYDVALIK.L	2	5.02	0.44	-5.93
IPI00019591	Isoform 1 of Complement factor B precursor (Fragment)	K.KEAGIPEFYDYDVALIK.L	3	4.09	0.34	-3.32
IPI00019591	Isoform 1 of Complement factor B precursor (Fragment)	K.LQDEDLGFL.-	1	1.87	0.18	-4.96
IPI00019591	Isoform 1 of Complement factor B precursor (Fragment)	K.LQDEDLGFL.-	2	2.50	0.21	-2.37
IPI00019591	Isoform 1 of Complement factor B precursor (Fragment)	K.NPREDYLDVYVFGVGPLVNQVNINALASK.K	3	7.19	0.50	-5.23
IPI00019591	Isoform 1 of Complement factor B precursor (Fragment)	K.NPREDYLDVYVFGVGPLVNQVNINALASKK.D	3	3.92	0.29	-1.92
IPI00019591	Isoform 1 of Complement factor B precursor (Fragment)	K.QLNEINYEDHK.L	1	2.07	0.19	-2.47
IPI00019591	Isoform 1 of Complement factor B precursor (Fragment)	K.QLNEINYEDHK.L	2	2.77	0.42	-4.66

IPI00019591	Isoform 1 of Complement factor B precursor (Fragment)	K.QLNEINYEDHKLK.S	2	2.53	0.35	-3.97
IPI00019591	Isoform 1 of Complement factor B precursor (Fragment)	K.RDLEIEVVLFHPNYNINGK.K	3	3.57	0.24	-4.33
IPI00019591	Isoform 1 of Complement factor B precursor (Fragment)	K.VASYGVKPR.Y	1	2.21	0.17	-5.14
IPI00019591	Isoform 1 of Complement factor B precursor (Fragment)	K.VASYGVKPR.Y	2	2.87	0.33	-2.82
IPI00019591	Isoform 1 of Complement factor B precursor (Fragment)	K.VKDISEVVTPR.F	1	2.82	0.25	-3.79
IPI00019591	Isoform 1 of Complement factor B precursor (Fragment)	K.VKDISEVVTPR.F	2	4.19	0.41	-3.41
IPI00019591	Isoform 1 of Complement factor B precursor (Fragment)	K.VKDISEVVTPR.F	3	3.92	0.15	-4.36
IPI00019591	Isoform 1 of Complement factor B precursor (Fragment)	K.VSEADSSNADWVTK.Q	2	5.06	0.51	-2.40
IPI00019591	Isoform 1 of Complement factor B precursor (Fragment)	K.VSVGGEKR.D	2	1.95	0.09	-2.36
IPI00019591	Isoform 1 of Complement factor B precursor (Fragment)	K.YGQTIRPICLPCTEGTTR.A	2	3.71	0.45	-2.92
IPI00019591	Isoform 1 of Complement factor B precursor (Fragment)	K.YGQTIRPICLPCTEGTTR.A	3	3.81	0.36	-4.06
IPI00019591	Isoform 1 of Complement factor B precursor (Fragment)	R.ALRLPPTTTCQQQKEELLPAQDIK.A	3	4.28	0.36	-5.03
IPI00019591	Isoform 1 of Complement factor B precursor (Fragment)	R.DAQYAPGYDK.V	1	2.53	0.29	-3.55
IPI00019591	Isoform 1 of Complement factor B precursor (Fragment)	R.DAQYAPGYDK.V	2	3.31	0.25	-3.99
IPI00019591	Isoform 1 of Complement factor B precursor (Fragment)	R.DAQYAPGYDKVK.D	1	2.24	0.20	0.38
IPI00019591	Isoform 1 of Complement factor B precursor (Fragment)	R.DAQYAPGYDKVK.D	2	3.11	0.30	-3.29
IPI00019591	Isoform 1 of Complement factor B precursor (Fragment)	R.DAQYAPGYDKVK.D	3	1.98	0.20	-3.57
IPI00019591	Isoform 1 of Complement factor B precursor (Fragment)	R.DAQYAPGYDKVKDISEVVTPR.F	2	5.40	0.59	-2.96
IPI00019591	Isoform 1 of Complement factor B precursor (Fragment)	R.DAQYAPGYDKVKDISEVVTPR.F	3	3.77	0.41	-2.75
IPI00019591	Isoform 1 of Complement factor B precursor (Fragment)	R.DAQYAPGYDKVKDISEVVTPR.F	4	3.09	0.23	-1.71
IPI00019591	Isoform 1 of Complement factor B precursor (Fragment)	R.DFHINLFQVLPWLK.E	2	3.25	0.24	-4.14

IPI00019591	Isoform 1 of Complement factor B precursor (Fragment)	R.DFHINLFQVLPWLK.E	3	4.20	0.10	-3.43
IPI00019591	Isoform 1 of Complement factor B precursor (Fragment)	R.DLEIEVVLFHPNYNINGK.K	2	5.07	0.45	-4.16
IPI00019591	Isoform 1 of Complement factor B precursor (Fragment)	R.DLEIEVVLFHPNYNINGK.K	3	3.82	0.38	-4.35
IPI00019591	Isoform 1 of Complement factor B precursor (Fragment)	R.DLEIEVVLFHPNYNINGKK.E	3	4.28	0.42	-4.39
IPI00019591	Isoform 1 of Complement factor B precursor (Fragment)	R.DLEIEVVLFHPNYNINGKK.E	4	3.55	0.25	-1.03
IPI00019591	Isoform 1 of Complement factor B precursor (Fragment)	R.DLLYIGK.D	1	1.98	0.15	-1.85
IPI00019591	Isoform 1 of Complement factor B precursor (Fragment)	R.DLLYIGKDR.K	2	2.89	0.22	-1.61
IPI00019591	Isoform 1 of Complement factor B precursor (Fragment)	R.EDYLDVYVFGVGPLVNQVNINALASK.K	2	5.00	0.53	-1.52
IPI00019591	Isoform 1 of Complement factor B precursor (Fragment)	R.EDYLDVYVFGVGPLVNQVNINALASK.K	3	4.05	0.49	-2.69
IPI00019591	Isoform 1 of Complement factor B precursor (Fragment)	R.EDYLDVYVFGVGPLVNQVNINALASKK.D	3	3.45	0.28	-4.31
IPI00019591	Isoform 1 of Complement factor B precursor (Fragment)	R.FIQVGVISWGVVDVCK.N	2	2.56	0.22	-0.04
IPI00019591	Isoform 1 of Complement factor B precursor (Fragment)	R.FLCTGGVSPYADPNTCR.G	2	5.01	0.49	-5.66
IPI00019591	Isoform 1 of Complement factor B precursor (Fragment)	R.FLCTGGVSPYADPNTCR.G	3	3.59	0.36	-4.42
IPI00019591	Isoform 1 of Complement factor B precursor (Fragment)	R.GDSGGPLIVHK.R	1	2.11	0.22	-4.23
IPI00019591	Isoform 1 of Complement factor B precursor (Fragment)	R.HVIILM*TDGLHNM*GGDPITVIDEIR.D	3	5.75	0.58	-4.90
IPI00019591	Isoform 1 of Complement factor B precursor (Fragment)	R.HVIILM*TDGLHNM*GGDPITVIDEIR.D	4	3.63	0.21	-3.61
IPI00019591	Isoform 1 of Complement factor B precursor (Fragment)	R.KNPREDYLDVYVFGVGPLVNQVNINALASK.K	3	8.07	0.60	-5.11
IPI00019591	Isoform 1 of Complement factor B precursor (Fragment)	R.KNPREDYLDVYVFGVGPLVNQVNINALASK.K	4	5.74	0.46	-4.93
IPI00019591	Isoform 1 of Complement factor B precursor (Fragment)	R.LEDSVTYHCSR.G	2	3.93	0.42	-2.26
IPI00019591	Isoform 1 of Complement factor B precursor (Fragment)	R.LEDSVTYHCSR.G	3	1.94	0.11	-0.75
IPI00019591	Isoform 1 of Complement factor B precursor (Fragment)	R.LLQEGQALEYVCPSGFYPYPVQTR.T	2	4.85	0.55	-5.96

IPI00019591	Isoform 1 of Complement factor B precursor (Fragment)	R.LLQEGQALEYVCPSGFYPYPVQTR.T	3	6.89	0.54	-6.53
IPI00019591	Isoform 1 of Complement factor B precursor (Fragment)	R.LLQEGQALEYVCPSGFYPYPVQTR.T	4	4.22	0.44	-3.67
IPI00019591	Isoform 1 of Complement factor B precursor (Fragment)	R.LPPTTTCQQQK.E	2	3.17	0.44	-3.38
IPI00019591	Isoform 1 of Complement factor B precursor (Fragment)	R.LPPTTTCQQQKEELLPAQDIK.A	3	3.71	0.28	-3.85
IPI00019591	Isoform 1 of Complement factor B precursor (Fragment)	R.PQGSCSLEGVEIK.G	2	4.15	0.33	-3.93
IPI00019591	Isoform 1 of Complement factor B precursor (Fragment)	R.WSGQTAICDNGAGYCSNPGIPIGTR.K	2	5.47	0.59	-1.92
IPI00019591	Isoform 1 of Complement factor B precursor (Fragment)	R.WSGQTAICDNGAGYCSNPGIPIGTR.K	3	5.16	0.50	-2.52
IPI00019591	Isoform 1 of Complement factor B precursor (Fragment)	R.YGLVTYATYPK.I	1	2.08	0.30	-3.70
IPI00019591	Isoform 1 of Complement factor B precursor (Fragment)	R.YGLVTYATYPK.I	2	4.69	0.53	-3.59
IPI00019600	Ubiquitin-conjugating enzyme E2 variant 2	K.INM*NGINNSSGM*VDAR.S	2	4.61	0.45	-5.60
IPI00019600	Ubiquitin-conjugating enzyme E2 variant 2	R.LLEELEEGQK.G	2	2.84	0.19	-3.61
IPI00019600	Ubiquitin-conjugating enzyme E2 variant 2	R.WTGM*IIGPPR.T	2	2.56	0.11	-0.35
IPI00019755	Glutathione transferase omega-1	K.EFTKLEEVLTNKK.T	2	3.76	0.31	-3.50
IPI00019755	Glutathione transferase omega-1	K.GSAPPGPVPEGSIR.I	2	2.13	0.08	-1.06
IPI00019755	Glutathione transferase omega-1	K.VPSLVGSFIR.S	2	2.70	0.31	-0.88
IPI00019771	Fractalkine precursor	K.DAM*QHLDR.Q	2	2.36	0.14	-1.55
IPI00019771	Fractalkine precursor	R.LGVLITPVPDAQAATRR.Q	2	2.57	0.10	-2.98
IPI00019812	Serine/threonine-protein phosphatase 5	K.AFLEENNLDYIIR.S	2	4.51	0.41	-3.21
IPI00019812	Serine/threonine-protein phosphatase 5	K.VLIM*HGGLFSEDGVTLDDIR.K	3	4.09	0.30	-1.54
IPI00019862	butyrophilin, subfamily 2, member A1 isoform 2 precursor	R.ERTEEQM*EEYR.G	2	2.26	0.12	-3.71
IPI00019862	butyrophilin, subfamily 2, member A1 isoform 2 precursor	R.ERTEEQM*EEYR.G	3	2.93	0.17	-2.36
IPI00019862	butyrophilin, subfamily 2, member A1 isoform 2 precursor	R.SQFSPAVFVYK.G	2	3.28	0.42	-2.48
IPI00019862	butyrophilin, subfamily 2, member A1 isoform 2 precursor	R.TEEQM*EEYRGR.I	2	1.55	0.13	-2.85
IPI00019888	Succinate-semialdehyde dehydrogenase, mitochondrial precursor	R.VSMELGGLAPFIVFDSANVDQAVAGAMASK.F	3	2.88	0.26	-2.01
IPI00019901	Isoform 1 of Alpha-adducin	R.NHGLVSVGESVEEAFYYIHNLVVACEIQVR.T	3	4.17	0.33	-3.89
IPI00019901	Isoform 1 of Alpha-adducin	R.NHGLVSVGESVEEAFYYIHNLVVACEIQVR.T	4	4.31	0.35	-3.71
IPI00019904	Isoform 1 of Beta-adducin	K.KLELDGEKETAPEEPGSPAKSAPAS.P	2	3.34	0.09	-4.15
IPI00019904	Isoform 1 of Beta-adducin	K.WGLLPVSHNALLVGDMAYYDFNGEMEQEADR.I	3	6.01	0.52	-3.13

IPI00019906	Isoform 2 of Basigin precursor	A.GTVFTTVEDLGSK.I	2	4.13	0.41	-3.96
IPI00019906	Isoform 2 of Basigin precursor	G.AAGTVFTTVEDLGSK.I	2	4.52	0.49	-3.16
IPI00019906	Isoform 2 of Basigin precursor	K.GGVVLKEDALPGQK.T	2	3.91	0.29	-2.93
IPI00019906	Isoform 2 of Basigin precursor	K.GGVVLKEDALPGQK.T	3	3.58	0.15	-1.75
IPI00019906	Isoform 2 of Basigin precursor	K.SESVPPVTDWAWYK.I	2	3.29	0.43	-3.11
IPI00019906	Isoform 2 of Basigin precursor	K.SSEHINEGETAM*.L	2	3.35	0.45	-3.48
IPI00019906	Isoform 2 of Basigin precursor	R.FFVSSSQGR.S	2	3.02	0.29	-1.65
IPI00019906	Isoform 2 of Basigin precursor	R.SELHIENLNM*EADPGQY.R	2	3.24	0.38	-3.75
IPI00019906	Isoform 2 of Basigin precursor	R.SELHIENLNM*EADPGQYR.C	3	4.52	0.30	-2.64
IPI00019907	Glypican-3 precursor	L.DVDDAPGNSQQATPK.D	2	3.36	0.38	-1.66
IPI00019907	Glypican-3 precursor	Y.DLDVDDAPGNSQQATPK.D	2	4.71	0.43	-2.48
IPI00019943	Afamin precursor	F.HADM*CQSQNEELQR.K	3	4.14	0.19	-0.70
IPI00019943	Afamin precursor	K.AESPEVCFNEESPK.I	2	4.39	0.42	
IPI00019943	Afamin precursor	K.AESPEVCFNEESPKIGN.-	2	3.09	0.30	
IPI00019943	Afamin precursor	K.CCKAESPEVCFNEESPKIGN.-	3	3.17	0.14	
IPI00019943	Afamin precursor	K.CQAYESNRESLLNHFLYEVAR.R	3	3.73	0.13	
IPI00019943	Afamin precursor	K.DGLKYHYLIR.L	3	2.23	0.14	-3.15
IPI00019943	Afamin precursor	K.DLSLREGK.F	2	1.47	0.06	-1.99
IPI00019943	Afamin precursor	K.DM*VEYKDR.C	2	2.67	0.13	-1.82
IPI00019943	Afamin precursor	K.ELISLVEDVSSNYDGCCEGDVVQCIR.D	3	5.77	0.58	-3.12
IPI00019943	Afamin precursor	K.FTFEYSR.R	1	2.03	0.11	-0.75
IPI00019943	Afamin precursor	K.FTFEYSR.R	2	2.40	0.29	-1.50
IPI00019943	Afamin precursor	K.HELTDEELQSLFTNFANVVDK.C	3	5.10	0.45	-3.52
IPI00019943	Afamin precursor	K.HFQNLGKDGLK.Y	2	3.59	0.34	-1.33
IPI00019943	Afamin precursor	K.HFQNLGKDGLK.Y	3	2.46	0.10	-4.04
IPI00019943	Afamin precursor	K.IAPQLSTEELVSLGEK.M	2	4.81	0.53	-7.03
IPI00019943	Afamin precursor	K.IAPQLSTEELVSLGEK.M	3	2.71	0.16	-2.58
IPI00019943	Afamin precursor	K.ICAM*EGLPQK.H	2	3.30	0.35	-3.88
IPI00019943	Afamin precursor	K.KSDVGFLPPFPTLDPEEK.C	2	4.48	0.48	-4.80
IPI00019943	Afamin precursor	K.KSDVGFLPPFPTLDPEEK.C	3	3.70	0.27	-2.51
IPI00019943	Afamin precursor	K.LKHELTDEELQSLFTNFANVVDK.C	2	5.57	0.58	-5.48
IPI00019943	Afamin precursor	K.LKHELTDEELQSLFTNFANVVDK.C	3	6.26	0.56	-6.02
IPI00019943	Afamin precursor	K.LKHELTDEELQSLFTNFANVVDK.C	4	5.66	0.48	-4.38
IPI00019943	Afamin precursor	K.LPNNVLQEK.I	1	3.04	0.16	-3.10
IPI00019943	Afamin precursor	K.LPNNVLQEK.I	2	3.29	0.20	-2.69
IPI00019943	Afamin precursor	K.LVKDM*VEYKDR.C	2	2.78	0.36	-0.22
IPI00019943	Afamin precursor	K.LVKDM*VEYKDR.C	3	2.46	0.20	0.38
IPI00019943	Afamin precursor	K.SCCEEQNKVNCLQTR.A	2	5.43	0.27	
IPI00019943	Afamin precursor	K.SCCEEQNKVNCLQTR.A	3	3.91	0.40	-2.12
IPI00019943	Afamin precursor	K.SDVGFLPPFPTLDPEEK.C	2	4.37	0.38	-4.80
IPI00019943	Afamin precursor	K.TDRFLVNLVK.L	2	2.51	0.05	-4.54
IPI00019943	Afamin precursor	K.TLPECSKLPNNVLQEK.I	2	4.68	0.27	

IPI00019943	Afamin precursor	K.TNFAFR.R	1	1.94	0.09	-0.01
IPI00019943	Afamin precursor	K.VM*NHICSK.Q	2	2.68	0.19	
IPI00019943	Afamin precursor	K.YHYLIR.L	1	1.69	0.06	-4.82
IPI00019943	Afamin precursor	R.AIPVTQYLK.A	1	1.59	0.10	-1.58
IPI00019943	Afamin precursor	R.AIPVTQYLK.A	2	1.17	0.09	-0.96
IPI00019943	Afamin precursor	R.DADPDTFFAK.F	1	2.82	0.36	-3.53
IPI00019943	Afamin precursor	R.DADPDTFFAK.F	2	2.96	0.33	-3.06
IPI00019943	Afamin precursor	R.EGKFTDSENVCQER.D	2	3.61	0.37	
IPI00019943	Afamin precursor	R.ESLLNHFLYEVAR.R	2	3.86	0.43	-3.56
IPI00019943	Afamin precursor	R.ESLLNHFLYEVAR.R	3	2.74	0.23	-1.25
IPI00019943	Afamin precursor	R.FLVNLVK.L	2	2.45	0.06	-3.10
IPI00019943	Afamin precursor	R.IVQIYKDLLR.N	2	2.49	0.11	-1.11
IPI00019943	Afamin precursor	R.IVQIYKDLLR.N	3	2.86	0.34	-3.45
IPI00019943	Afamin precursor	R.KTDRFLVNLVK.L	2	2.78	0.15	-4.37
IPI00019943	Afamin precursor	R.LCFFYNK.K	2	2.53	0.21	-1.41
IPI00019943	Afamin precursor	R.NPFVFAPTLLTVAVHFEEVAK.S	2	4.26	0.52	-2.29
IPI00019943	Afamin precursor	R.NPFVFAPTLLTVAVHFEEVAK.S	3	2.76	0.17	-3.31
IPI00019943	Afamin precursor	R.RHPDLSIPELLR.I	2	3.54	0.37	-5.02
IPI00019943	Afamin precursor	R.TINPAVDHCCK.T	2	3.10	0.29	
IPI00019954	Cystatin-M precursor	K.AQSQLVAGIK.Y	1	1.94	0.13	-1.97
IPI00019954	Cystatin-M precursor	K.AQSQLVAGIK.Y	2	3.47	0.22	-1.82
IPI00019954	Cystatin-M precursor	K.YFLTM*EM*GSTDCR.K	2	2.92	0.51	-4.88
IPI00019954	Cystatin-M precursor	R.DLSPDDPQVQK.A	2	2.50	0.27	-2.60
IPI00019954	Cystatin-M precursor	R.M*VGELRDLSPDDPQVQK.A	2	2.95	0.25	-3.35
IPI00019954	Cystatin-M precursor	R.M*VGELRDLSPDDPQVQK.A	3	3.52	0.30	-1.98
IPI00019954	Cystatin-M precursor	R.VTGDHVDLTTCPLAAGAQQEK.L	3	3.80	0.29	-1.92
IPI00019988	N-sulphoglucosamine sulphohydrolase precursor	R.RSLLFRNAFTSVSSCSPSR.A	2	2.61	0.11	-6.16
IPI00020008	NEDD8 precursor	K.EIEIDIEPTDKVER.I	2	3.19	0.28	-2.91
IPI00020012	Amyloid-like protein 1 precursor	A.ALEGFLAALQADPPQAER.V	2	4.42	0.31	-2.13
IPI00020012	Amyloid-like protein 1 precursor	A.DRQALNEHFQSILQTLEEQVSGER.Q	3	5.03	0.43	-3.54
IPI00020012	Amyloid-like protein 1 precursor	A.PGSAQVAGLCGR.L	1	3.01	0.39	-4.34
IPI00020012	Amyloid-like protein 1 precursor	D.PSGTAVGDPSTR.S	2	3.50	0.41	-2.79
IPI00020012	Amyloid-like protein 1 precursor	E.IQRDELAPAGTGVSR.E	2	3.73	0.34	-1.89
IPI00020012	Amyloid-like protein 1 precursor	F.HSSEIQRDELAPAGTGVSR.E	3	4.16	0.36	-3.34
IPI00020012	Amyloid-like protein 1 precursor	F.LAALQADPPQAER.V	2	3.36	0.26	-2.23
IPI00020012	Amyloid-like protein 1 precursor	G.SLAGGSPGAAEAPGSAQVAGLCGR.L	2	5.88	0.66	-3.29
IPI00020012	Amyloid-like protein 1 precursor	G.SLAGGSPGAAEAPGSAQVAGLCGR.L	3	4.79	0.50	-3.22
IPI00020012	Amyloid-like protein 1 precursor	I.GSLAGGSPGAAEAPGSAQVAGLCGR.L	2	5.34	0.53	-3.16
IPI00020012	Amyloid-like protein 1 precursor	I.GSLAGGSPGAAEAPGSAQVAGLCGR.L	3	4.09	0.45	-2.81
IPI00020012	Amyloid-like protein 1 precursor	K.ADRQALNEHFQSILQTLEEQVSGER.Q	2	4.38	0.53	-3.72
IPI00020012	Amyloid-like protein 1 precursor	K.ADRQALNEHFQSILQTLEEQVSGER.Q	3	7.19	0.57	-5.08
IPI00020012	Amyloid-like protein 1 precursor	K.ADRQALNEHFQSILQTLEEQVSGER.Q	4	2.82	0.19	-4.90

IPI00020012	Amyloid-like protein 1 precursor	K.DDTPM*TLPK.G	2	2.30	0.21	-2.56
IPI00020012	Amyloid-like protein 1 precursor	K.EKM*NPLEQYER.K	2	2.65	0.18	-3.58
IPI00020012	Amyloid-like protein 1 precursor	K.GGLQPPDSKDDTPM*TLPK.G	2	2.93	0.31	-3.00
IPI00020012	Amyloid-like protein 1 precursor	K.GGLQPPDSKDDTPM*TLPK.G	3	3.02	0.24	-1.88
IPI00020012	Amyloid-like protein 1 precursor	K.GSTEQDAASPEKEK.M	2	3.89	0.32	-3.42
IPI00020012	Amyloid-like protein 1 precursor	K.GSTEQDAASPEKEKM*NPLEQYER.K	2	3.06	0.41	-2.67
IPI00020012	Amyloid-like protein 1 precursor	K.GSTEQDAASPEKEKM*NPLEQYER.K	3	3.25	0.26	-2.68
IPI00020012	Amyloid-like protein 1 precursor	K.GSTEQDAASPEKEKM*NPLEQYER.K	4	2.00	0.35	-2.26
IPI00020012	Amyloid-like protein 1 precursor	K.M*NPLEQYER.K	1	1.66	0.24	-2.38
IPI00020012	Amyloid-like protein 1 precursor	K.M*NPLEQYER.K	2	3.13	0.26	-4.45
IPI00020012	Amyloid-like protein 1 precursor	L.APAGTGVSR.E	1	2.16	0.27	-2.49
IPI00020012	Amyloid-like protein 1 precursor	M.NPLEQYER.K	1	2.17	0.19	-3.81
IPI00020012	Amyloid-like protein 1 precursor	P.FHSSEIQR.D	1	2.73	0.13	-5.46
IPI00020012	Amyloid-like protein 1 precursor	P.FHSSEIQRDELAPAGTGVSR.E	2	5.05	0.45	-3.28
IPI00020012	Amyloid-like protein 1 precursor	P.FHSSEIQRDELAPAGTGVSR.E	3	4.77	0.33	-2.63
IPI00020012	Amyloid-like protein 1 precursor	P.KGSTEQDAASPEKEK.M	2	3.11	0.26	-1.94
IPI00020012	Amyloid-like protein 1 precursor	Q.ALNEHFQSILQTLEEQVSGER.Q	2	6.59	0.55	-2.73
IPI00020012	Amyloid-like protein 1 precursor	Q.ALNEHFQSILQTLEEQVSGER.Q	3	3.97	0.33	-2.51
IPI00020012	Amyloid-like protein 1 precursor	R.AALEGFLAALQAD.P	1	2.12	0.17	-4.04
IPI00020012	Amyloid-like protein 1 precursor	R.AALEGFLAALQAD.P	2	3.72	0.26	-4.74
IPI00020012	Amyloid-like protein 1 precursor	R.AALEGFLAALQADPPQAER.V	2	4.92	0.46	-5.73
IPI00020012	Amyloid-like protein 1 precursor	R.AALEGFLAALQADPPQAER.V	3	5.84	0.47	-4.11
IPI00020012	Amyloid-like protein 1 precursor	R.AALEGFLAALQADPPQAER.V	4	3.80	0.16	-1.41
IPI00020012	Amyloid-like protein 1 precursor	R.AKM*DLEER.R	2	2.88	0.18	-3.09
IPI00020012	Amyloid-like protein 1 precursor	R.CLPGEFVSEALLVPEGCR.F	2	3.89	0.42	-4.91
IPI00020012	Amyloid-like protein 1 precursor	R.CLPGEFVSEALLVPEGCR.F	3	4.24	0.29	-4.31
IPI00020012	Amyloid-like protein 1 precursor	R.DELAPAGTGVSR.E	1	2.49	0.25	-3.43
IPI00020012	Amyloid-like protein 1 precursor	R.DELAPAGTGVSR.E	2	3.12	0.28	-3.03
IPI00020012	Amyloid-like protein 1 precursor	R.DELAPAGTGVSRE.A	2	3.43	0.30	-2.87
IPI00020012	Amyloid-like protein 1 precursor	R.EAVSGLLIM*GAGG.G	1	2.42	0.25	-3.74
IPI00020012	Amyloid-like protein 1 precursor	R.EAVSGLLIM*GAGG.G	2	3.04	0.34	-2.60
IPI00020012	Amyloid-like protein 1 precursor	R.EAVSGLLIM*GAGGGS.L	2	3.28	0.35	-2.60
IPI00020012	Amyloid-like protein 1 precursor	R.EWAM*ADNQSK.N	2	2.48	0.28	-1.45
IPI00020012	Amyloid-like protein 1 precursor	R.FQVHTHLQVIEER.V	2	3.96	0.46	-4.47
IPI00020012	Amyloid-like protein 1 precursor	R.FQVHTHLQVIEER.V	3	3.19	0.35	-3.29
IPI00020012	Amyloid-like protein 1 precursor	R.GFPFHSSEIQ.R	1	2.13	0.30	-4.72
IPI00020012	Amyloid-like protein 1 precursor	R.GFPFHSSEIQ.R	2	3.27	0.34	-2.61
IPI00020012	Amyloid-like protein 1 precursor	R.GFPFHSSEIQR.D	2	2.78	0.36	-3.38
IPI00020012	Amyloid-like protein 1 precursor	R.GFPFHSSEIQRDELAPAGTGVSR.E	2	4.10	0.39	-4.71
IPI00020012	Amyloid-like protein 1 precursor	R.GFPFHSSEIQRDELAPAGTGVSR.E	3	3.15	0.25	-4.76
IPI00020012	Amyloid-like protein 1 precursor	R.HQEAQEACSSQGLILHGSGM*LLPCGSDR.F	3	4.78	0.53	-3.14
IPI00020012	Amyloid-like protein 1 precursor	R.HYQHVAAVDPEK.A	2	3.41	0.46	-4.18

IPI00020012	Amyloid-like protein 1 precursor	R.HYQHVAAVDPEKAQQM*R.F	3	3.21	0.30	-3.21
IPI00020012	Amyloid-like protein 1 precursor	R.HYQHVAAVDPEKAQQM*R.F	4	2.93	0.22	-2.06
IPI00020012	Amyloid-like protein 1 precursor	R.LVETHATR.V	1	1.49	0.14	-3.60
IPI00020012	Amyloid-like protein 1 precursor	R.LVETHATR.V	2	2.72	0.29	-3.03
IPI00020012	Amyloid-like protein 1 precursor	R.QALNEHFQSILQTLEEQ.V	2	3.55	0.32	-3.86
IPI00020012	Amyloid-like protein 1 precursor	R.QALNEHFQSILQTLEEQVS.G	2	3.42	0.34	-5.24
IPI00020012	Amyloid-like protein 1 precursor	R.QALNEHFQSILQTLEEQVSGE.R	2	4.82	0.46	-3.56
IPI00020012	Amyloid-like protein 1 precursor	R.QALNEHFQSILQTLEEQVSGER.Q	2	4.56	0.48	-5.71
IPI00020012	Amyloid-like protein 1 precursor	R.QALNEHFQSILQTLEEQVSGER.Q	3	5.58	0.49	-5.68
IPI00020012	Amyloid-like protein 1 precursor	R.QALNEHFQSILQTLEEQVSGER.Q	4	3.66	0.31	-3.53
IPI00020012	Amyloid-like protein 1 precursor	R.QALNEHFQSILQTLEEQVSGERQ.R	3	4.17	0.40	-1.23
IPI00020012	Amyloid-like protein 1 precursor	R.QINEVM*R.E	2	1.89	0.10	-3.21
IPI00020012	Amyloid-like protein 1 precursor	R.QINEVM*REWAM*ADNQSK.N	2	2.54	0.13	
IPI00020012	Amyloid-like protein 1 precursor	R.QINEVM*REWAM*ADNQSK.N	3	2.31	0.23	-3.23
IPI00020012	Amyloid-like protein 1 precursor	R.QM*YPELQIAR.V	2	2.37	0.29	-3.84
IPI00020012	Amyloid-like protein 1 precursor	R.QRLVETHATR.V	2	2.60	0.11	-2.75
IPI00020012	Amyloid-like protein 1 precursor	R.RAALEGFLAALQAD.P	2	2.94	0.23	-1.90
IPI00020012	Amyloid-like protein 1 precursor	R.RAALEGFLAALQADPPQAER.V	3	5.14	0.43	-3.99
IPI00020012	Amyloid-like protein 1 precursor	R.RAALEGFLAALQADPPQAER.V	4	3.24	0.36	-1.37
IPI00020012	Amyloid-like protein 1 precursor	R.VEQATQAIPM*ER.W	2	3.92	0.39	-5.36
IPI00020012	Amyloid-like protein 1 precursor	R.VEQATQAIPM*ER.W	3	2.92	0.10	-3.29
IPI00020012	Amyloid-like protein 1 precursor	R.VIALINDQR.R	2	3.39	0.06	-2.90
IPI00020012	Amyloid-like protein 1 precursor	R.VIALINDQRR.A	2	2.56	0.06	-2.87
IPI00020012	Amyloid-like protein 1 precursor	R.VLEYCR.Q	1	1.32	0.11	-2.36
IPI00020012	Amyloid-like protein 1 precursor	R.WEPDPQR.S	2	1.75	0.09	-2.55
IPI00020012	Amyloid-like protein 1 precursor	R.YLRAEQKEQR.H	3	2.37	0.21	-3.06
IPI00020012	Amyloid-like protein 1 precursor	S.EIQRDELAPAGTGVSR.E	2	4.12	0.38	-1.14
IPI00020012	Amyloid-like protein 1 precursor	S.PGAAEAPGSAQVAGLCGR.L	2	4.07	0.53	-3.66
IPI00020012	Amyloid-like protein 1 precursor	V.EQATQAIPM*ER.W	2	2.93	0.23	-1.75
IPI00020019	Adiponectin precursor	R.NGLYADNDNDSTFTGFLLYHDTN.-	2	5.49	0.56	-2.39
IPI00020058	Isoform 1 of Copper-transporting ATPase 2	K.GGKPLEMAHKIKTVMFDKTGTITHGVPR.V	3	3.24	0.16	
IPI00020091	Alpha-1-acid glycoprotein 2 precursor	K.EQLGEFYEALDCLCIPR.S	2	5.58	0.49	
IPI00020091	Alpha-1-acid glycoprotein 2 precursor	K.EQLGEFYEALDCLCIPR.S	3	5.02	0.22	
IPI00020091	Alpha-1-acid glycoprotein 2 precursor	K.SVQEIQATFFYFTPNKTEDTIFLR.E	3	2.94	0.14	
IPI00020091	Alpha-1-acid glycoprotein 2 precursor	K.TEDTIFLR.E	1	1.64	0.11	
IPI00020091	Alpha-1-acid glycoprotein 2 precursor	K.TLM*FGSYLDDEK.N	2	3.73	0.43	
IPI00020091	Alpha-1-acid glycoprotein 2 precursor	K.TLM*FGSYLDDEKNWGLSFYADKPETTK.E	2	4.80	0.43	
IPI00020091	Alpha-1-acid glycoprotein 2 precursor	K.TLM*FGSYLDDEKNWGLSFYADKPETTK.E	3	5.05	0.49	
IPI00020091	Alpha-1-acid glycoprotein 2 precursor	K.TLMFGSYLDDEK.N	2	3.90	0.36	
IPI00020091	Alpha-1-acid glycoprotein 2 precursor	K.TLMFGSYLDDEKNWGLSFYADKPETTK.E	3	4.48	0.30	
IPI00020091	Alpha-1-acid glycoprotein 2 precursor	K.WFYIASAFR.N	1	2.66	0.25	
IPI00020091	Alpha-1-acid glycoprotein 2 precursor	K.WFYIASAFR.N	2	2.37	0.31	-1.54

IPI00020091	Alpha-1-acid glycoprotein 2 precursor	K.WFYIASAFRNEEYNK.S	2	4.39	0.40	
IPI00020091	Alpha-1-acid glycoprotein 2 precursor	R.EHVAHLLFLR.D	2	3.42	0.31	
IPI00020091	Alpha-1-acid glycoprotein 2 precursor	R.QNQCFYNSSYLNVQR.E	2	4.06	0.28	
IPI00020091	Alpha-1-acid glycoprotein 2 precursor	R.SDVM*YTDWK.K	1	2.83	0.15	
IPI00020091	Alpha-1-acid glycoprotein 2 precursor	R.SDVM*YTDWK.K	2	3.66	0.27	
IPI00020091	Alpha-1-acid glycoprotein 2 precursor	R.SDVM*YTDWKK.D	1	2.97	0.30	
IPI00020091	Alpha-1-acid glycoprotein 2 precursor	R.SDVM*YTDWKK.D	2	3.30	0.27	
IPI00020091	Alpha-1-acid glycoprotein 2 precursor	R.SDVM*YTDWKK.D	3	2.81	0.21	
IPI00020091	Alpha-1-acid glycoprotein 2 precursor	R.SDVMYTDWK.K	2	2.72	0.22	
IPI00020091	Alpha-1-acid glycoprotein 2 precursor	R.SDVMYTDWKK.D	2	3.00	0.35	
IPI00020091	Alpha-1-acid glycoprotein 2 precursor	R.YEGGREHVAHLLFLR.D	2	4.03	0.20	
IPI00020131	Son of sevenless homolog 1	K.GKQLAIKKM*NEIQK.N	2	4.41	0.10	
IPI00020199	Alpha-2,8-sialyltransferase 8B	R.SAVNSLHSK.S	2	2.13	0.05	-2.34
IPI00020201	CMP-N-acetylneuraminate-poly-alpha-2,8-sialyltransferase	K.SSFKPGDVIHYVLDR.R	3	2.88	0.11	-3.76
IPI00020329	Potassium voltage-gated channel subfamily S member 2	R.SLGATLK.Y	1	1.68	0.05	-3.46
IPI00020356	331 kDa protein	R.KTLYKAK.V	2	1.88	0.07	-3.00
IPI00020396	Isoform PACE4A-I of Proprotein convertase subtilisin/kexin type 6 precursor	A.SYDVNGNDYDPSPR.Y	2	3.52	0.52	-3.15
IPI00020396	Isoform PACE4A-I of Proprotein convertase subtilisin/kexin type 6 precursor	L.DGDVTDVVEAK.S	2	3.66	0.31	-1.20
IPI00020396	Isoform PACE4A-I of Proprotein convertase subtilisin/kexin type 6 precursor	T.ILDDGIER.N	2	3.02	0.19	-3.22
IPI00020407	Alpha-1,6-mannosylglycoprotein 6-beta-N-acetylglucosaminyltransferase A	K.TLAVLLDNILQR.I	2	3.21	0.26	-3.71
IPI00020407	Alpha-1,6-mannosylglycoprotein 6-beta-N-acetylglucosaminyltransferase A	R.TQPESSSM*LR.E	2	1.48	0.13	-2.71
IPI00020431	Isoform 1 of TGF-beta receptor type-2 precursor	K.LPYHDFILEDAASPK.C	2	3.66	0.25	-3.59
IPI00020431	Isoform 1 of TGF-beta receptor type-2 precursor	K.LPYHDFILEDAASPK.C	3	2.57	0.06	-1.21
IPI00020470	Isoform 1 of Glycosyltransferase 8 domain-containing protein 1	K.WM*KLNVEEGLYSR.T	2	2.90	0.08	
IPI00020501	Myosin-11	R.NTDQASM*PDNTAAQK.V	2	2.86	0.37	-3.01
IPI00020557	Prolow-density lipoprotein receptor-related protein 1 precursor	K.ADGSGSVVLR.N	2	3.20	0.21	-2.14
IPI00020557	Prolow-density lipoprotein receptor-related protein 1 precursor	K.AKNEPVDRPPVLLIANSQNILATYLSGAQVSTITPTSTR.Q	3	6.68	0.61	-3.71
IPI00020557	Prolow-density lipoprotein receptor-related protein 1 precursor	K.ATALAIM*GDK.L	2	2.49	0.08	-4.49
IPI00020557	Prolow-density lipoprotein receptor-related protein 1 precursor	K.AVTDEEPFLIFANR.Y	2	3.62	0.35	-0.35

IPI00020557	Prolow-density lipoprotein receptor-related protein 1 precursor	K.CLCVEGYAPR.G	2	3.30	0.32	-2.48
IPI00020557	Prolow-density lipoprotein receptor-related protein 1 precursor	K.GIALDPAM*GK.V	1	1.87	0.17	-1.12
IPI00020557	Prolow-density lipoprotein receptor-related protein 1 precursor	K.GIALDPAM*GK.V	2	2.49	0.20	-1.40
IPI00020557	Prolow-density lipoprotein receptor-related protein 1 precursor	K.GPVGLAIDFPESK.L	2	4.47	0.45	-2.54
IPI00020557	Prolow-density lipoprotein receptor-related protein 1 precursor	K.GYLFWTEWGQYPR.I	2	3.83	0.42	-4.60
IPI00020557	Prolow-density lipoprotein receptor-related protein 1 precursor	K.IETAAM*DGTLR.E	2	3.60	0.34	-1.12
IPI00020557	Prolow-density lipoprotein receptor-related protein 1 precursor	K.ITWPNGLTLDYVTER.I	2	2.95	0.34	-3.20
IPI00020557	Prolow-density lipoprotein receptor-related protein 1 precursor	K.IVFPHGITLDLVSR.L	3	3.58	0.45	-1.26
IPI00020557	Prolow-density lipoprotein receptor-related protein 1 precursor	K.KPEHELFLVYGK.G	2	2.66	0.36	-3.62
IPI00020557	Prolow-density lipoprotein receptor-related protein 1 precursor	K.KPEHELFLVYGK.G	3	3.09	0.28	-2.32
IPI00020557	Prolow-density lipoprotein receptor-related protein 1 precursor	K.LSVIGSIR.L	2	2.58	0.13	-2.32
IPI00020557	Prolow-density lipoprotein receptor-related protein 1 precursor	K.LWWADQVSEK.M	2	2.56	0.18	-0.59
IPI00020557	Prolow-density lipoprotein receptor-related protein 1 precursor	K.LYFSDATLDKIER.C	2	3.52	0.38	-2.60
IPI00020557	Prolow-density lipoprotein receptor-related protein 1 precursor	K.NAVVQGLEQPHGLVVHPLR.G	2	5.57	0.50	-1.29
IPI00020557	Prolow-density lipoprotein receptor-related protein 1 precursor	K.NAVVQGLEQPHGLVVHPLR.G	3	3.23	0.43	-1.06
IPI00020557	Prolow-density lipoprotein receptor-related protein 1 precursor	K.NAVVQGLEQPHGLVVHPLR.G	4	2.67	0.19	-1.77
IPI00020557	Prolow-density lipoprotein receptor-related protein 1 precursor	K.NEPVDRPPVLLIANSQNILATYLSGAQVSTITPTSTR.Q	3	6.05	0.50	-3.95
IPI00020557	Prolow-density lipoprotein receptor-related protein 1 precursor	K.NVIALAFDYR.A	2	3.68	0.44	-3.85
IPI00020557	Prolow-density lipoprotein receptor-related protein 1 precursor	K.TLISGM*IDEPHAIVVDPLR.G	3	3.46	0.34	-2.52
IPI00020557	Prolow-density lipoprotein receptor-related protein 1 precursor	K.TVLWPNGLSLDIPAGR.L	2	4.39	0.42	-5.10
IPI00020557	Prolow-density lipoprotein receptor-related protein 1 precursor	K.VFFTDYGQIPK.V	2	4.35	0.45	-3.76

IPI00020557	Prolow-density lipoprotein receptor-related protein 1 precursor	K.VPDEHM*IPIENLM*NPR.A	2	3.67	0.50	-3.73
IPI00020557	Prolow-density lipoprotein receptor-related protein 1 precursor	K.VPDEHM*IPIENLM*NPR.A	3	4.05	0.34	-2.43
IPI00020557	Prolow-density lipoprotein receptor-related protein 1 precursor	K.VYDESIQLDHK.G	2	3.32	0.39	-2.59
IPI00020557	Prolow-density lipoprotein receptor-related protein 1 precursor	K.VYDESIQLDHK.G	3	1.61	0.12	-1.33
IPI00020557	Prolow-density lipoprotein receptor-related protein 1 precursor	R.AALSGANVLTLIEK.D	2	4.56	0.38	-3.48
IPI00020557	Prolow-density lipoprotein receptor-related protein 1 precursor	R.AALSGANVLTLIEKDIR.T	3	2.80	0.37	-1.83
IPI00020557	Prolow-density lipoprotein receptor-related protein 1 precursor	R.AITVHPEK.G	2	1.91	0.18	-0.78
IPI00020557	Prolow-density lipoprotein receptor-related protein 1 precursor	R.AIVVDPLNGWM*YWTDWEEDPKDSR.R	3	3.45	0.46	-3.82
IPI00020557	Prolow-density lipoprotein receptor-related protein 1 precursor	R.CLPGFLGDR.C	2	2.33	0.26	-0.24
IPI00020557	Prolow-density lipoprotein receptor-related protein 1 precursor	R.CNLDGSGLEVIDAM*R.S	2	5.00	0.51	-1.89
IPI00020557	Prolow-density lipoprotein receptor-related protein 1 precursor	R.CTAYFEGSR.C	2	2.49	0.19	-1.35
IPI00020557	Prolow-density lipoprotein receptor-related protein 1 precursor	R.DGILFWTDWDASLPR.I	2	4.44	0.47	-6.77
IPI00020557	Prolow-density lipoprotein receptor-related protein 1 precursor	R.DQITCISK.G	2	2.18	0.07	-2.57
IPI00020557	Prolow-density lipoprotein receptor-related protein 1 precursor	R.DQTWREDVVTNGIGR.V	3	3.91	0.28	-0.91
IPI00020557	Prolow-density lipoprotein receptor-related protein 1 precursor	R.EDVVTNGIGR.V	2	3.14	0.29	-2.45
IPI00020557	Prolow-density lipoprotein receptor-related protein 1 precursor	R.ETLVQDNIQWPTGLAVDYHNER.L	3	3.13	0.12	-4.66
IPI00020557	Prolow-density lipoprotein receptor-related protein 1 precursor	R.ETVITM*SGDDHPR.A	2	3.53	0.41	-0.67
IPI00020557	Prolow-density lipoprotein receptor-related protein 1 precursor	R.GVGGAPPTVTLLR.S	2	3.04	0.38	-1.80
IPI00020557	Prolow-density lipoprotein receptor-related protein 1 precursor	R.IDLETGENR.E	2	3.05	0.22	-3.15
IPI00020557	Prolow-density lipoprotein receptor-related protein 1 precursor	R.IDLHKGDYSVLVPGLR.N	2	3.61	0.43	-4.30
IPI00020557	Prolow-density lipoprotein receptor-related protein 1 precursor	R.IDLHKGDYSVLVPGLR.N	3	3.44	0.21	-3.11

IPI00020557	Prolow-density lipoprotein receptor-related protein 1 precursor	R.IEAASM*SGAGR.R	2	4.05	0.34	-2.31
IPI00020557	Prolow-density lipoprotein receptor-related protein 1 precursor	R.IFFSDIHFGNIQQINDDGSR.R	2	4.84	0.55	-3.63
IPI00020557	Prolow-density lipoprotein receptor-related protein 1 precursor	R.IFFSDIHFGNIQQINDDGSR.R	3	4.13	0.40	-1.86
IPI00020557	Prolow-density lipoprotein receptor-related protein 1 precursor	R.IFFSDIHFGNIQQINDDGSRR.I	3	2.70	0.18	-2.03
IPI00020557	Prolow-density lipoprotein receptor-related protein 1 precursor	R.ITIVENVGSVEGLAYHR.G	3	2.61	0.19	-2.33
IPI00020557	Prolow-density lipoprotein receptor-related protein 1 precursor	R.LDGLCIPLR.W	2	2.24	0.06	-1.59
IPI00020557	Prolow-density lipoprotein receptor-related protein 1 precursor	R.M*YDAQQQQVGTNK.C	2	4.93	0.51	-3.17
IPI00020557	Prolow-density lipoprotein receptor-related protein 1 precursor	R.M*YDAQQQQVGTNK.C	3	2.93	0.27	-5.19
IPI00020557	Prolow-density lipoprotein receptor-related protein 1 precursor	R.NLNAPVQPFEDPEHM*K.N	2	3.84	0.53	-1.99
IPI00020557	Prolow-density lipoprotein receptor-related protein 1 precursor	R.NLNAPVQPFEDPEHM*K.N	3	2.07	0.17	-2.43
IPI00020557	Prolow-density lipoprotein receptor-related protein 1 precursor	R.RITIVENVGSVEGLAYHR.G	3	5.53	0.48	-1.38
IPI00020557	Prolow-density lipoprotein receptor-related protein 1 precursor	R.SERPPIFEIR.M	3	2.92	0.27	-3.91
IPI00020557	Prolow-density lipoprotein receptor-related protein 1 precursor	R.SGFSLGSDGK.S	2	3.06	0.35	-2.49
IPI00020557	Prolow-density lipoprotein receptor-related protein 1 precursor	R.SGSVYRLER.G	2	2.08	0.07	-2.04
IPI00020557	Prolow-density lipoprotein receptor-related protein 1 precursor	R.SLDPFKPFIIFSN.R	2	3.09	0.32	-4.59
IPI00020557	Prolow-density lipoprotein receptor-related protein 1 precursor	R.SLDPFKPFIIFSNR.H	2	3.32	0.42	-3.01
IPI00020557	Prolow-density lipoprotein receptor-related protein 1 precursor	R.SLDPFKPFIIFSNR.H	3	3.39	0.26	-2.17
IPI00020557	Prolow-density lipoprotein receptor-related protein 1 precursor	R.TLLFSGQK.G	1	1.42	0.11	-2.61
IPI00020557	Prolow-density lipoprotein receptor-related protein 1 precursor	R.TNTQPFDLQVYHPSR.Q	2	3.71	0.40	-3.17
IPI00020557	Prolow-density lipoprotein receptor-related protein 1 precursor	R.TNTQPFDLQVYHPSR.Q	3	2.18	0.12	-2.87
IPI00020557	Prolow-density lipoprotein receptor-related protein 1 precursor	R.TTLLAGDIEHPR.A	2	3.57	0.34	-0.85

IPI00020557	Prolow-density lipoprotein receptor-related protein 1 precursor	R.VDKGGALHIYHQR.R	2	3.79	0.31	-4.77
IPI00020557	Prolow-density lipoprotein receptor-related protein 1 precursor	R.VDKGGALHIYHQR.R	3	2.86	0.26	-2.32
IPI00020557	Prolow-density lipoprotein receptor-related protein 1 precursor	R.VYWSDVR.T	2	2.17	0.17	-0.92
IPI00020557	Prolow-density lipoprotein receptor-related protein 1 precursor	R.YDGSGHM*EVLR.G	2	2.43	0.21	-4.27
IPI00020557	Prolow-density lipoprotein receptor-related protein 1 precursor	R.YVVISQGLDKPR.A	2	3.09	0.33	-2.60
IPI00020599	Calreticulin precursor	K.EQFLDGDGWTSR.W	2	3.35	0.31	-3.66
IPI00020599	Calreticulin precursor	K.FVLSSGK.F	1	1.79	0.13	-2.44
IPI00020599	Calreticulin precursor	K.FVLSSGKFYGDEEKDKGLQTSQDAR.F	3	5.07	0.36	-4.05
IPI00020599	Calreticulin precursor	K.FVLSSGKFYGDEEKDKGLQTSQDAR.F	4	3.10	0.24	-4.51
IPI00020599	Calreticulin precursor	K.FYGDEEKDK.G	2	2.24	0.19	-1.99
IPI00020599	Calreticulin precursor	K.FYGDEEKDKGLQTSQDAR.F	2	4.99	0.46	-1.75
IPI00020599	Calreticulin precursor	K.FYGDEEKDKGLQTSQDAR.F	3	3.56	0.33	-2.85
IPI00020599	Calreticulin precursor	K.GKNVLINKDIR.C	2	2.92	0.14	-4.85
IPI00020599	Calreticulin precursor	K.GLQTSQDAR.F	2	2.35	0.06	-1.57
IPI00020599	Calreticulin precursor	K.LFPNSLDQTDM*HGDSEYNIM*FGPDICGPGTK.K	3	4.49	0.50	-3.59
IPI00020599	Calreticulin precursor	K.NVLINKDIR.C	2	2.58	0.15	-1.44
IPI00020599	Calreticulin precursor	K.SDFGKFVLSSGK.F	2	3.46	0.38	-2.60
IPI00020599	Calreticulin precursor	K.SDFGKFVLSSGKFYGDEEKDKGLQTSQDAR.F	4	3.64	0.29	-4.07
IPI00020599	Calreticulin precursor	K.SGTIFDNFLITNDEAYAEEFGNETWGVTK.A	3	6.21	0.57	-3.60
IPI00020599	Calreticulin precursor	K.VHVIFNYK.G	1	2.78	0.26	-4.17
IPI00020599	Calreticulin precursor	R.CKDDEFTHLYTLIVRPDNTYEVK.I	3	4.62	0.39	-2.31
IPI00020599	Calreticulin precursor	R.CKDDEFTHLYTLIVRPDNTYEVK.I	4	3.53	0.25	-3.11
IPI00020599	Calreticulin precursor	R.CKDDEFTHLYTLIVRPDNTYEVK.I	5	2.23	0.12	-3.10
IPI00020599	Calreticulin precursor	R.FYALSASFEPFSNK.G	2	5.01	0.55	-5.23
IPI00020599	Calreticulin precursor	R.QIDNPDYK.G	1	2.31	0.09	-3.18
IPI00020599	Calreticulin precursor	R.QIDNPDYK.G	2	2.02	0.16	-2.52
IPI00020672	Isoform 1 of Dipeptidyl-peptidase 3	R.LEGSDVQLLEYEASAAGLIR.S	3	3.84	0.33	-4.90
IPI00020672	Isoform 1 of Dipeptidyl-peptidase 3	R.LFKEVDGEGKPYYEVR.L	4	2.76	0.23	-2.81
IPI00020672	Isoform 1 of Dipeptidyl-peptidase 3	R.SFSERFPEDGPELEEILTQLATADAR.F	3	4.14	0.32	-4.51
IPI00020672	Isoform 1 of Dipeptidyl-peptidase 3	R.VILGSEAAQQHPEEVR.G	3	2.05	0.14	-1.44
IPI00020672	Isoform 1 of Dipeptidyl-peptidase 3	R.VLLEAGEGLVTITPTTGSDGRPDAR.V	3	3.79	0.44	-2.86
IPI00020692	Isoform 1 of Sodium channel protein type 3 subunit alpha	K.IDSCM*SNNTGIEISKELNYLR.D	2	2.21	0.10	0.28
IPI00020692	Isoform 1 of Sodium channel protein type 3 subunit alpha	R.DGNGTTSGVGTGSSVEK.Y	2	2.32	0.24	
IPI00020747	Sodium channel subunit beta-3 precursor	P.VCVEVPSETEAVQGNPM*K.L	2	5.49	0.50	-4.72
IPI00020747	Sodium channel subunit beta-3 precursor	R.EFEFEAHRPFVK.T	2	3.27	0.38	-1.37

IPI00020747	Sodium channel subunit beta-3 precursor	R.NGHQEVESPFQGR.L	2	3.23	0.42	-4.41
IPI00020884	Plexin-A3 precursor	K.SEYFPTLSSR.K	2	2.11	0.17	-2.33
IPI00020884	Plexin-A3 precursor	R.AHVTGPVEDNAR.C	2	2.92	0.14	-3.78
IPI00020906	Inositol monophosphatase	K.EIQVIPLQRDDED.-	2	2.39	0.14	-2.45
IPI00020906	Inositol monophosphatase	K.LQVSQQEDITK.S	2	3.64	0.33	-1.15
IPI00020906	Inositol monophosphatase	K.SILTDNPTWIIDPIDGTTNFVHR.F	3	3.33	0.19	-6.74
IPI00020906	Inositol monophosphatase	K.SLLVTELGSSR.T	2	2.93	0.34	-2.26
IPI00020906	Inositol monophosphatase	K.SSPVDLVTATDQKVEK.M	2	3.15	0.24	-2.42
IPI00020966	Isoform 1 of Phosphatidylinositol N-acetylglucosaminyltransferase subunit A	C.RGGAGNGHR.A	2	2.91	0.24	-2.59
IPI00020977	Isoform 1 of Connective tissue growth factor precursor	K.DGAPCIFGGTVYR.S	2	3.94	0.47	-4.31
IPI00020977	Isoform 1 of Connective tissue growth factor precursor	R.LPSPDCPFPR.R	2	2.53	0.19	-3.13
IPI00020977	Isoform 1 of Connective tissue growth factor precursor	R.SGESFQSSCK.Y	2	2.40	0.28	-0.66
IPI00020984	Calnexin precursor	K.APVPTGEVYFADSFDR.G	2	4.47	0.56	-3.98
IPI00020984	Calnexin precursor	K.LPGDKGLVLM*SR.A	3	2.44	0.17	-2.18
IPI00020984	Calnexin precursor	R.GTLSGWILSK.A	2	3.27	0.22	-0.63
IPI00020984	Calnexin precursor	R.KIPNPDFFEDLEPFR.M	3	4.71	0.26	-2.38
IPI00020986	Lumican precursor	K.ILGPLSYSK.I	1	1.85	0.07	-1.26
IPI00020986	Lumican precursor	K.ISNIPDEYFK.R	1	2.36	0.20	-3.40
IPI00020986	Lumican precursor	K.ISNIPDEYFK.R	2	3.21	0.14	-2.64
IPI00020986	Lumican precursor	K.ISNIPDEYFKR.F	2	3.13	0.28	-3.06
IPI00020986	Lumican precursor	K.ISNIPDEYFKR.F	3	2.98	0.13	-3.53
IPI00020986	Lumican precursor	K.LKNIPTVNENLENYYLEVNQLEK.F	3	3.52	0.28	-3.99
IPI00020986	Lumican precursor	K.LKNIPTVNENLENYYLEVNQLEKFDIK.S	4	3.74	0.23	-4.27
IPI00020986	Lumican precursor	K.NIPTVNENLENYYLEVNQLEK.F	2	5.64	0.51	-4.32
IPI00020986	Lumican precursor	K.NIPTVNENLENYYLEVNQLEK.F	3	4.39	0.30	-3.14
IPI00020986	Lumican precursor	K.NIPTVNENLENYYLEVNQLEKFDIK.S	2	4.14	0.43	-0.59
IPI00020986	Lumican precursor	K.NIPTVNENLENYYLEVNQLEKFDIK.S	3	2.51	0.09	-3.18
IPI00020986	Lumican precursor	K.RFNALQYLR.L	2	2.52	0.16	-3.65
IPI00020986	Lumican precursor	K.SLEDLQLTHNK.I	1	2.67	0.32	-2.59
IPI00020986	Lumican precursor	K.SLEDLQLTHNK.I	2	3.86	0.37	-2.71
IPI00020986	Lumican precursor	K.SLEDLQLTHNK.I	3	2.05	0.19	-3.01
IPI00020986	Lumican precursor	K.SLEDLQLTHNKITK.L	2	4.05	0.43	-1.12
IPI00020986	Lumican precursor	K.SLEDLQLTHNKITK.L	3	2.07	0.26	-2.07
IPI00020986	Lumican precursor	K.SLEYLDLSFNQIAR.L	2	4.87	0.41	-6.39
IPI00020986	Lumican precursor	K.SLEYLDLSFNQIAR.L	3	5.77	0.44	-3.03
IPI00020986	Lumican precursor	K.SVPM*VPPGIK.Y	1	1.49	0.16	-2.08
IPI00020986	Lumican precursor	K.SVPM*VPPGIK.Y	2	1.54	0.13	-0.53
IPI00020986	Lumican precursor	R.FNALQYLR.L	1	2.47	0.12	-1.86

IPI00020986	Lumican precursor	R.FNALQYLR.L	2	3.27	0.25	-1.08
IPI00020986	Lumican precursor	R.ISETSLPPDM*YECLR.V	2	3.13	0.35	-4.09
IPI00020986	Lumican precursor	R.LKEDAVSAAFK.G	1	2.75	0.40	-4.21
IPI00020986	Lumican precursor	R.LKEDAVSAAFK.G	2	3.68	0.37	-3.15
IPI00020986	Lumican precursor	R.LPSGLPVSLLTLYLDNNK.I	2	5.67	0.59	-5.83
IPI00020986	Lumican precursor	R.LPSGLPVSLLTLYLDNNK.I	3	5.64	0.40	-3.80
IPI00020986	Lumican precursor	R.LPSGLPVSLLTLYLDNNKISNIPDEYFK.R	3	5.74	0.56	-4.08
IPI00020986	Lumican precursor	R.LPSGLPVSLLTLYLDNNKISNIPDEYFK.R	4	3.56	0.31	-3.40
IPI00020986	Lumican precursor	R.LPSGLPVSLLTLYLDNNKISNIPDEYFKR.F	4	4.80	0.47	-3.38
IPI00020986	Lumican precursor	R.NNQIDHIDEK.A	1	2.67	0.25	-1.58
IPI00020986	Lumican precursor	R.NNQIDHIDEK.A	2	3.24	0.27	-2.96
IPI00020987	Prolargin precursor	K.LENLLLLDLQHNR.L	3	2.90	0.27	-1.47
IPI00020987	Prolargin precursor	K.LPGLVFLYM*EK.N	2	3.30	0.31	
IPI00020987	Prolargin precursor	K.NQLEEVPSALPR.N	2	3.83	0.31	-2.18
IPI00020987	Prolargin precursor	R.LSDGVFKPDTFHGLK.N	3	2.76	0.15	-2.36
IPI00020987	Prolargin precursor	R.LSDGVFKPDTFHGLK.N	4	2.93	0.24	-3.36
IPI00020987	Prolargin precursor	R.VLEKLPGLVFLYM*EK.N	3	2.52	0.16	-2.90
IPI00020990	Osteomodulin precursor	K.IDYGVFAK.L	2	2.31	0.24	-3.12
IPI00020990	Osteomodulin precursor	K.LKQAFYIPR.N	2	3.10	0.32	-2.99
IPI00020990	Osteomodulin precursor	K.LM*QLNLCSNR.L	2	3.28	0.32	-2.41
IPI00020990	Osteomodulin precursor	K.LPNLLQLHLEHNNLEEFPFPLPK.S	2	5.17	0.56	-4.17
IPI00020990	Osteomodulin precursor	K.LPNLLQLHLEHNNLEEFPFPLPK.S	3	6.10	0.53	-4.28
IPI00020990	Osteomodulin precursor	K.LPNLLQLHLEHNNLEEFPFPLPK.S	4	4.41	0.40	-5.10
IPI00020990	Osteomodulin precursor	K.LQDIPYNIFNLPNIVELSVGHNK.L	2	4.58	0.44	-2.98
IPI00020990	Osteomodulin precursor	K.LQDIPYNIFNLPNIVELSVGHNK.L	3	3.54	0.30	-4.17
IPI00020990	Osteomodulin precursor	K.LQDIPYNIFNLPNIVELSVGHNK.L	4	4.05	0.30	-4.61
IPI00020990	Osteomodulin precursor	K.QAFYIPR.N	2	1.64	0.19	-1.85
IPI00020990	Osteomodulin precursor	K.SQKIDYGVFAK.L	2	3.52	0.34	-3.26
IPI00020990	Osteomodulin precursor	K.SQKIDYGVFAK.L	3	3.52	0.35	-4.00
IPI00020990	Osteomodulin precursor	R.LLLGYNEISK.L	1	2.92	0.21	-2.72
IPI00020990	Osteomodulin precursor	R.LLLGYNEISK.L	2	2.16	0.15	-2.13
IPI00020990	Osteomodulin precursor	R.STNGQTIQLK.T	2	3.28	0.29	0.26
IPI00020996	Insulin-like growth factor-binding protein complex acid labile chain precursor	K.ALRDFALQNPSAVPR.F	2	4.01	0.31	-2.43
IPI00020996	Insulin-like growth factor-binding protein complex acid labile chain precursor	K.ALRDFALQNPSAVPR.F	3	2.77	0.14	0.47
IPI00020996	Insulin-like growth factor-binding protein complex acid labile chain precursor	K.ANVFVQLPR.L	2	3.02	0.30	-1.86
IPI00020996	Insulin-like growth factor-binding protein complex acid labile chain precursor	K.DLHFLEELQLGHNR.I	3	4.12	0.41	-1.63
IPI00020996	Insulin-like growth factor-binding protein complex acid labile chain precursor	K.DLHFLEELQLGHNR.I	4	3.58	0.25	-2.52

IPI00020996	Insulin-like growth factor-binding protein complex acid labile chain precursor	K.LEYLLLSR.N	1	2.07	0.15	-3.89
IPI00020996	Insulin-like growth factor-binding protein complex acid labile chain precursor	K.LEYLLLSR.N	2	3.36	0.27	-3.01
IPI00020996	Insulin-like growth factor-binding protein complex acid labile chain precursor	R.AFWLDVSHNR.L	3	2.40	0.08	-3.51
IPI00020996	Insulin-like growth factor-binding protein complex acid labile chain precursor	R.DFALQNPSAVPR.F	2	3.59	0.42	-3.85
IPI00020996	Insulin-like growth factor-binding protein complex acid labile chain precursor	R.ELVLAGNR.L	2	2.05	0.08	-3.16
IPI00020996	Insulin-like growth factor-binding protein complex acid labile chain precursor	R.LAELPADALGPLQR.A	2	4.30	0.41	-3.78
IPI00020996	Insulin-like growth factor-binding protein complex acid labile chain precursor	R.LAELPADALGPLQR.A	3	4.12	0.21	-2.73
IPI00020996	Insulin-like growth factor-binding protein complex acid labile chain precursor	R.LAYLQPALFSGLAELR.E	2	5.31	0.31	-4.49
IPI00020996	Insulin-like growth factor-binding protein complex acid labile chain precursor	R.LAYLQPALFSGLAELR.E	3	4.17	0.34	-1.73
IPI00020996	Insulin-like growth factor-binding protein complex acid labile chain precursor	R.LAYLQPALFSGLAELRELDLSR.N	3	2.74	0.29	-4.72
IPI00020996	Insulin-like growth factor-binding protein complex acid labile chain precursor	R.LEALPNSLLAPLGR.L	2	4.46	0.43	-3.96
IPI00020996	Insulin-like growth factor-binding protein complex acid labile chain precursor	R.LFQGLGKLEYLLLSR.N	2	4.67	0.34	-4.13
IPI00020996	Insulin-like growth factor-binding protein complex acid labile chain precursor	R.LFQGLGKLEYLLLSR.N	3	4.13	0.17	-3.54
IPI00020996	Insulin-like growth factor-binding protein complex acid labile chain precursor	R.LSHNAIASLRPR.T	2	2.44	0.16	-4.01
IPI00020996	Insulin-like growth factor-binding protein complex acid labile chain precursor	R.LWLEGNPWDCGCPLK.A	2	4.49	0.40	
IPI00020996	Insulin-like growth factor-binding protein complex acid labile chain precursor	R.NLIAAVAPGAFLGLK.A	2	4.55	0.43	-4.15
IPI00020996	Insulin-like growth factor-binding protein complex acid labile chain precursor	R.NLIAAVAPGAFLGLK.A	3	3.15	0.30	-2.38
IPI00020996	Insulin-like growth factor-binding protein complex acid labile chain precursor	R.NLPEQVFR.G	1	2.21	0.13	-2.86
IPI00020996	Insulin-like growth factor-binding protein complex acid labile chain precursor	R.SFEGLGQLEVLTLDHNQLQEVK.A	2	4.76	0.50	-6.85
IPI00020996	Insulin-like growth factor-binding protein complex acid labile chain precursor	R.SFEGLGQLEVLTLDHNQLQEVK.A	3	3.66	0.49	-3.61
IPI00020996	Insulin-like growth factor-binding protein complex acid labile chain precursor	R.SLALGTFAHTPALASLGLSNNR.L	2	5.49	0.57	-2.93

IPI00020996	Insulin-like growth factor-binding protein complex acid labile chain precursor	R.SLALGTFAHTPALASLGLSNNR.L	3	4.78	0.46	-3.70
IPI00020996	Insulin-like growth factor-binding protein complex acid labile chain precursor	R.TFTPQPPGLER.L	2	2.44	0.24	-2.18
IPI00020996	Insulin-like growth factor-binding protein complex acid labile chain precursor	R.VAGLLEDTFPGLLGLR.V	2	5.38	0.48	-4.53
IPI00020996	Insulin-like growth factor-binding protein complex acid labile chain precursor	R.VAGLLEDTFPGLLGLR.V	3	4.21	0.31	-3.53
IPI00021000	Isoform A of Osteopontin precursor	A.IPVKQADSGSSEEK.Q	2	4.30	0.41	-3.26
IPI00021000	Isoform A of Osteopontin precursor	A.IPVKQADSGSSEEK.Q	3	3.57	0.39	-1.59
IPI00021000	Isoform A of Osteopontin precursor	A.NDESNEHSDVIDSQELSK.V	2	5.40	0.44	-3.67
IPI00021000	Isoform A of Osteopontin precursor	A.NDESNEHSDVIDSQELSK.V	3	3.92	0.45	-2.77
IPI00021000	Isoform A of Osteopontin precursor	A.VSSEETNDFKQETLPSK.S	2	4.68	0.37	-1.91
IPI00021000	Isoform A of Osteopontin precursor	H.SDVIDSQELSK.V	2	2.94	0.30	-1.60
IPI00021000	Isoform A of Osteopontin precursor	I.PVAQDLNAPSDWDSR.G	2	4.56	0.45	-2.57
IPI00021000	Isoform A of Osteopontin precursor	I.PVKQADSGSSEEK.Q	2	3.25	0.38	-1.07
IPI00021000	Isoform A of Osteopontin precursor	K.AIPVAQDLNAPSD.W	2	3.80	0.53	-3.31
IPI00021000	Isoform A of Osteopontin precursor	K.AIPVAQDLNAPSDWDSR.G	2	5.21	0.53	-4.53
IPI00021000	Isoform A of Osteopontin precursor	K.AIPVAQDLNAPSDWDSR.G	3	4.02	0.40	-3.55
IPI00021000	Isoform A of Osteopontin precursor	K.ANDESNEHSDVIDSQELSK.V	2	5.61	0.55	-3.29
IPI00021000	Isoform A of Osteopontin precursor	K.ANDESNEHSDVIDSQELSK.V	3	4.89	0.34	-3.60
IPI00021000	Isoform A of Osteopontin precursor	K.DSYETSQLDDQSAETHSHK.Q	2	5.84	0.65	-2.86
IPI00021000	Isoform A of Osteopontin precursor	K.DSYETSQLDDQSAETHSHK.Q	3	2.98	0.25	-1.85
IPI00021000	Isoform A of Osteopontin precursor	K.DSYETSQLDDQSAETHSHK.Q	4	3.22	0.27	-0.90
IPI00021000	Isoform A of Osteopontin precursor	K.DSYETSQLDDQSAETHSHKQS.R	2	5.31	0.57	-3.55
IPI00021000	Isoform A of Osteopontin precursor	K.DSYETSQLDDQSAETHSHKQSR.L	3	3.78	0.44	-4.42
IPI00021000	Isoform A of Osteopontin precursor	K.FRISHELDSASSE.V	2	3.47	0.35	-1.77
IPI00021000	Isoform A of Osteopontin precursor	K.FRISHELDSASSEVN.-	2	4.27	0.49	-4.28
IPI00021000	Isoform A of Osteopontin precursor	K.FRRPDIQYPDATDEDITSHM*ESEELNGAYK.A	4	4.32	0.48	-2.71
IPI00021000	Isoform A of Osteopontin precursor	K.QLYNKYPDAVATWLNPDPSQK.Q	3	3.40	0.26	-3.78
IPI00021000	Isoform A of Osteopontin precursor	K.QLYNKYPDAVATWLNPDPSQKQN.L	3	4.15	0.31	-2.77
IPI00021000	Isoform A of Osteopontin precursor	K.QNLLAPQNAVSSEETNDFKQETLPSK.S	3	3.06	0.33	-3.92
IPI00021000	Isoform A of Osteopontin precursor	K.RKANDESNEHSDVIDSQELSK.V	3	4.24	0.40	-4.10
IPI00021000	Isoform A of Osteopontin precursor	K.RKANDESNEHSDVIDSQELSK.V	4	3.58	0.47	-2.79
IPI00021000	Isoform A of Osteopontin precursor	K.SKEEDKHLKF.R	2	2.90	0.27	-3.89
IPI00021000	Isoform A of Osteopontin precursor	L.APQNAVSSEETNDFKQETLPSK.S	3	4.14	0.43	-1.96
IPI00021000	Isoform A of Osteopontin precursor	L.DDQSAETHSHK.Q	2	3.41	0.34	-3.47
IPI00021000	Isoform A of Osteopontin precursor	L.LAPQNAVSSEETNDFKQETLPSK.S	2	4.72	0.54	-2.89
IPI00021000	Isoform A of Osteopontin precursor	N.AVSSEETNDFKQETLPSK.S	2	4.70	0.48	-2.49
IPI00021000	Isoform A of Osteopontin precursor	N.AVSSEETNDFKQETLPSK.S	3	3.68	0.28	-1.89
IPI00021000	Isoform A of Osteopontin precursor	N.DESNEHSDVIDSQELSK.V	2	5.48	0.49	-3.53
IPI00021000	Isoform A of Osteopontin precursor	N.EHSDVIDSQELSK.V	2	4.20	0.39	-4.84

IPI00021000	Isoform A of Osteopontin precursor	P.QNAVSSEETNDFKQETLPSK.S	2	4.36	0.41	-1.71
IPI00021000	Isoform A of Osteopontin precursor	P.VAQDLNAPSDWDSR.G	2	4.52	0.49	-2.52
IPI00021000	Isoform A of Osteopontin precursor	R.EFHSHEFHSHED.M	2	2.99	0.46	-4.79
IPI00021000	Isoform A of Osteopontin precursor	R.EFHSHEFHSHEDM*LVVDPK.S	2	2.30	0.41	-3.82
IPI00021000	Isoform A of Osteopontin precursor	R.EFHSHEFHSHEDM*LVVDPK.S	3	2.79	0.21	-4.47
IPI00021000	Isoform A of Osteopontin precursor	R.EFHSHEFHSHEDM*LVVDPK.S	4	2.23	0.11	-2.53
IPI00021000	Isoform A of Osteopontin precursor	R.GDSVVYGLR.S	1	1.96	0.31	-2.54
IPI00021000	Isoform A of Osteopontin precursor	R.GDSVVYGLR.S	2	3.38	0.24	-3.18
IPI00021000	Isoform A of Osteopontin precursor	R.GKDSYETSQLDDQSAETH.S	3	3.98	0.35	-1.82
IPI00021000	Isoform A of Osteopontin precursor	R.GKDSYETSQLDDQSAETHS.H	2	6.10	0.61	-1.71
IPI00021000	Isoform A of Osteopontin precursor	R.GKDSYETSQLDDQSAETHS.H	3	3.58	0.40	-2.88
IPI00021000	Isoform A of Osteopontin precursor	R.GKDSYETSQLDDQSAETHSH.K	3	5.23	0.50	-2.21
IPI00021000	Isoform A of Osteopontin precursor	R.GKDSYETSQLDDQSAETHSHK.Q	2	6.71	0.52	-2.98
IPI00021000	Isoform A of Osteopontin precursor	R.GKDSYETSQLDDQSAETHSHK.Q	3	6.02	0.59	-4.98
IPI00021000	Isoform A of Osteopontin precursor	R.GKDSYETSQLDDQSAETHSHK.Q	4	3.89	0.25	-2.95
IPI00021000	Isoform A of Osteopontin precursor	R.GKDSYETSQLDDQSAETHSHK.Q	5	2.86	0.32	0.25
IPI00021000	Isoform A of Osteopontin precursor	R.GKDSYETSQLDDQSAETHSHKQ.S	2	5.03	0.53	-3.50
IPI00021000	Isoform A of Osteopontin precursor	R.GKDSYETSQLDDQSAETHSHKQ.S	3	5.41	0.55	-3.11
IPI00021000	Isoform A of Osteopontin precursor	R.GKDSYETSQLDDQSAETHSHKQS.R	2	6.09	0.58	-3.46
IPI00021000	Isoform A of Osteopontin precursor	R.GKDSYETSQLDDQSAETHSHKQS.R	3	6.61	0.56	-4.74
IPI00021000	Isoform A of Osteopontin precursor	R.ISHELDSASS.E	1	2.13	0.46	-2.76
IPI00021000	Isoform A of Osteopontin precursor	R.ISHELDSASSE.V	1	2.80	0.42	-4.07
IPI00021000	Isoform A of Osteopontin precursor	R.ISHELDSASSE.V	2	3.11	0.28	-2.99
IPI00021000	Isoform A of Osteopontin precursor	R.ISHELDSASSEVN.-	1	4.12	0.49	-4.10
IPI00021000	Isoform A of Osteopontin precursor	R.ISHELDSASSEVN.-	2	3.69	0.44	-4.48
IPI00021000	Isoform A of Osteopontin precursor	R.KANDESNEHSDVIDSQELSK.V	2	5.74	0.57	-5.59
IPI00021000	Isoform A of Osteopontin precursor	R.KANDESNEHSDVIDSQELSK.V	3	6.60	0.56	-4.99
IPI00021000	Isoform A of Osteopontin precursor	R.RPDIQYPDATDEDITSHM*ESEE.L	3	4.37	0.38	-3.52
IPI00021000	Isoform A of Osteopontin precursor	R.RPDIQYPDATDEDITSHM*ESEELNGAYK.A	3	6.46	0.61	-2.73
IPI00021000	Isoform A of Osteopontin precursor	R.RPDIQYPDATDEDITSHM*ESEELNGAYK.A	4	5.82	0.51	-2.59
IPI00021000	Isoform A of Osteopontin precursor	S.DVIDSQELSK.V	2	3.12	0.30	-2.68
IPI00021000	Isoform A of Osteopontin precursor	T.SQLDDQSAETHSHK.Q	2	3.02	0.30	-2.21
IPI00021000	Isoform A of Osteopontin precursor	V.AQDLNAPSDWDSR.G	2	3.84	0.43	-4.37
IPI00021033	Isoform 1 of Collagen alpha-1(III) chain precursor	K.INTDEIM*TSLK.S	2	2.72	0.18	-2.38
IPI00021033	Isoform 1 of Collagen alpha-1(III) chain precursor	K.LM*GSNEGEFK.A	2	1.96	0.08	-2.67
IPI00021033	Isoform 1 of Collagen alpha-1(III) chain precursor	K.NSIAYM*DQASGNVK.K	2	4.12	0.47	-3.72
IPI00021033	Isoform 1 of Collagen alpha-1(III) chain precursor	K.NSIAYM*DQASGNVKK.A	2	4.02	0.42	-1.83
IPI00021033	Isoform 1 of Collagen alpha-1(III) chain precursor	K.SGEYWVDPNQGCK.L	2	4.07	0.48	-2.33
IPI00021033	Isoform 1 of Collagen alpha-1(III) chain precursor	K.SVNGQIESLISPDGSR.K	2	4.60	0.50	-2.52
IPI00021033	Isoform 1 of Collagen alpha-1(III) chain precursor	K.SVNGQIESLISPDGSR.K	3	3.19	0.14	-2.77
IPI00021033	Isoform 1 of Collagen alpha-1(III) chain precursor	K.VFCNM*ETGETCISANPLNVPR.K	3	2.55	0.26	-2.71
IPI00021033	Isoform 1 of Collagen alpha-1(III) chain precursor	R.GPVGPSGPPGK.D	2	2.17	0.22	-3.11

IPI00021048	Isoform 1 of Myoferlin	K.VFLPKEELYM*PPLVIK.V	2	3.16	0.10	
IPI00021091	Isoform 1 of Leucine-rich glioma-inactivated protein 1 precursor	K.AGFTTIYK.W	2	2.32	0.14	-2.83
IPI00021091	Isoform 1 of Leucine-rich glioma-inactivated protein 1 precursor	R.DTDVEYLEIVR.T	2	4.14	0.41	-3.83
IPI00021119	Carbohydrate sulfotransferase 1	K.IAASEEELKNPSVSLVEER.D	3	4.56	0.44	-1.57
IPI00021119	Carbohydrate sulfotransferase 1	K.TEEIYGFLGIPLDSHVAR.W	3	3.54	0.40	-1.91
IPI00021199	Stathmin-3	K.DTSLEELQK.R	2	2.63	0.13	-2.47
IPI00021199	Stathmin-3	K.RLEAAEER.R	2	2.60	0.09	-0.31
IPI00021263	14-3-3 protein zeta/delta	K.IETELRDICNDVLSLLEK.F	2	3.92	0.28	-3.22
IPI00021263	14-3-3 protein zeta/delta	K.IETELRDICNDVLSLLEK.F	3	5.16	0.41	-2.85
IPI00021263	14-3-3 protein zeta/delta	K.M*KGDYYR.Y	2	2.25	0.17	-2.86
IPI00021263	14-3-3 protein zeta/delta	K.SVTEQGAELSNEER.N	2	4.97	0.37	-3.16
IPI00021263	14-3-3 protein zeta/delta	K.TAFDEAIAELDTLSEESYK.D	2	4.84	0.39	-2.32
IPI00021263	14-3-3 protein zeta/delta	K.TAFDEAIAELDTLSEESYK.D	3	4.35	0.43	-4.28
IPI00021263	14-3-3 protein zeta/delta	K.TAFDEAIAELDTLSEESYKDSTLIM*QLLR.D	3	5.68	0.52	-4.58
IPI00021263	14-3-3 protein zeta/delta	K.TAFDEAIAELDTLSEESYKDSTLIM*QLLR.D	4	4.17	0.31	-4.23
IPI00021263	14-3-3 protein zeta/delta	K.TAFDEAIAELDTLSEESYKDSTLIMQLLR.D	3	7.46	0.62	-4.46
IPI00021263	14-3-3 protein zeta/delta	K.TAFDEAIAELDTLSEESYKDSTLIMQLLR.D	4	4.60	0.24	-3.47
IPI00021263	14-3-3 protein zeta/delta	R.DICNDVLSLLEK.F	2	4.08	0.24	-3.52
IPI00021263	14-3-3 protein zeta/delta	R.DNLTLWTSDTQGDEAEAGEGGEN.-	2	5.93	0.56	-5.64
IPI00021263	14-3-3 protein zeta/delta	R.LGLALNFSVFYYEILNSPEK.A	2	4.16	0.55	-2.27
IPI00021263	14-3-3 protein zeta/delta	R.NLLSVAYK.N	1	1.68	0.08	-2.46
IPI00021263	14-3-3 protein zeta/delta	R.NLLSVAYK.N	2	2.28	0.09	-1.47
IPI00021263	14-3-3 protein zeta/delta	R.VVSSIEQK.T	1	2.24	0.05	-2.39
IPI00021263	14-3-3 protein zeta/delta	R.VVSSIEQK.T	2	3.14	0.16	-2.69
IPI00021263	14-3-3 protein zeta/delta	R.YLAEVAAGDDKK.G	2	3.81	0.31	-2.82
IPI00021263	14-3-3 protein zeta/delta	R.YLAEVAAGDDKK.G	3	2.36	0.16	-3.21
IPI00021263	14-3-3 protein zeta/delta	R.YLAEVAAGDDKKGIVDQSQQAYQEAFEISKK.E	4	5.34	0.45	-2.45
IPI00021274	Ephrin type-A receptor 8 precursor	R.DFLSEASIMGQFDHPNIIRLEGVVTRGR.L	3	4.32	0.07	
IPI00021275	Isoform 1 of Ephrin type-B receptor 2 precursor	K.VDTIAADESFSQVDLGGR.V	2	5.78	0.55	-4.14
IPI00021275	Isoform 1 of Ephrin type-B receptor 2 precursor	K.VDTIAADESFSQVDLGGR.V	3	4.64	0.41	-3.09
IPI00021275	Isoform 1 of Ephrin type-B receptor 2 precursor	R.GSCIANAEEVDVPIKLYCNGDGEWLVPIGR.C	3	3.90	0.42	-4.89
IPI00021275	Isoform 1 of Ephrin type-B receptor 2 precursor	R.QLGLTEPR.I	2	1.50	0.09	-0.94
IPI00021275	Isoform 1 of Ephrin type-B receptor 2 precursor	R.TTSEGATNCVCR.N	2	3.23	0.36	-2.52
IPI00021275	Isoform 1 of Ephrin type-B receptor 2 precursor	R.TYQVCNVFESSQNNWLR.T	2	4.79	0.48	-2.14
IPI00021304	Keratin, type II cytoskeletal 2 epidermal	K.AQYEEIAQR.S	2	2.37	0.11	-2.21
IPI00021304	Keratin, type II cytoskeletal 2 epidermal	K.DVDNAYM*IK.V	2	2.85	0.07	-2.69
IPI00021304	Keratin, type II cytoskeletal 2 epidermal	K.GGSISGGGYGSGGGK.H	2	4.02	0.43	-2.62
IPI00021304	Keratin, type II cytoskeletal 2 epidermal	K.HSSGGGSRGGSSSGGGYGSGGGGSSSVK.G	3	4.66	0.26	
IPI00021304	Keratin, type II cytoskeletal 2 epidermal	K.KYEDEINKR.T	2	3.00	0.17	-1.46
IPI00021304	Keratin, type II cytoskeletal 2 epidermal	K.VDLLNQEIEFLK.V	2	4.27	0.13	

IPI00021304	Keratin, type II cytoskeletal 2 epidermal	R.FLEQQNQVLQTK.W	2	4.27	0.32	-3.17
IPI00021304	Keratin, type II cytoskeletal 2 epidermal	R.GFSSGSAVVSGGSR.R	2	2.72	0.31	-1.48
IPI00021304	Keratin, type II cytoskeletal 2 epidermal	R.GSSSGGGYSSGSSSYGSGGR.Q	2	4.77	0.57	-3.29
IPI00021304	Keratin, type II cytoskeletal 2 epidermal	R.M*SGDLSSNVTVSVTSSTISSNVASK.A	2	4.88	0.41	
IPI00021304	Keratin, type II cytoskeletal 2 epidermal	R.M*SGDLSSNVTVSVTSSTISSNVASK.A	3	4.63	0.40	
IPI00021304	Keratin, type II cytoskeletal 2 epidermal	R.NLDLDSIIAEVKAQYEEIAQR.S	2	3.36	0.22	
IPI00021304	Keratin, type II cytoskeletal 2 epidermal	R.NLDLDSIIAEVKAQYEEIAQR.S	3	5.87	0.48	
IPI00021304	Keratin, type II cytoskeletal 2 epidermal	R.QSGSRGGSGGGGSISGGGYGSGGGSGGR.Y	2	2.66	0.38	
IPI00021304	Keratin, type II cytoskeletal 2 epidermal	R.QSGSRGGSGGGGSISGGGYGSGGGSGGR.Y	3	4.26	0.12	
IPI00021304	Keratin, type II cytoskeletal 2 epidermal	R.SKEEAEALYHSK.Y	2	4.17	0.42	-2.86
IPI00021304	Keratin, type II cytoskeletal 2 epidermal	R.SLVGLGGTK.S	2	2.34	0.20	-1.58
IPI00021304	Keratin, type II cytoskeletal 2 epidermal	R.YGSGGGSKGGSISGGGYGSGGGK.H	3	4.99	0.27	
IPI00021304	Keratin, type II cytoskeletal 2 epidermal	R.YLDGLTAER.T	2	2.31	0.13	-1.87
IPI00021304	Keratin, type II cytoskeletal 2 epidermal	T.SSTISSNVASK.A	1	2.05	0.24	-1.86
IPI00021304	Keratin, type II cytoskeletal 2 epidermal	T.SSTISSNVASK.A	2	3.29	0.32	-0.82
IPI00021327	Isoform 1 of Growth factor receptor-bound protein 2	K.YFLWVVK.F	2	2.58	0.12	-1.48
IPI00021347	Ubiquitin-conjugating enzyme E2 L3	K.NAEEFTKK.Y	2	2.42	0.18	-2.84
IPI00021347	Ubiquitin-conjugating enzyme E2 L3	K.TDQVIQSLIALVNDPQPEHPLR.A	3	3.83	0.45	-0.72
IPI00021347	Ubiquitin-conjugating enzyme E2 L3	R.IEINFPAEYPFKPPKITFK.T	4	2.57	0.19	-3.10
IPI00021363	Histone demethylase JARID1A	K.AAAAKVELVK.E	1	2.07	0.10	
IPI00021364	Properdin precursor	S.DPVLCFTQYEESSGK.C	2	4.77	0.48	-3.91
IPI00021447	Alpha-amylase 2B precursor	K.IAEYM*NHLIDIGVAGFR.L	3	2.98	0.26	-1.59
IPI00021447	Alpha-amylase 2B precursor	K.IYVSDDGK.A	2	1.94	0.07	-1.69
IPI00021447	Alpha-amylase 2B precursor	K.TGSGDIENYNDATQVR.D	2	4.75	0.47	-2.44
IPI00021447	Alpha-amylase 2B precursor	R.LVGLLDLALEKDYVR.S	2	2.97	0.46	-3.28
IPI00021447	Alpha-amylase 2B precursor	R.YQPVSYK.L	1	1.65	0.16	-6.51
IPI00021476	Eukaryotic translation initiation factor 4E-binding protein 3	G.GRDQLPDCYSTTPGGTLYATTPGGTRIIYDR.K	3	3.74	0.19	2.07
IPI00021485	Leucine-rich repeat neuronal protein 1 precursor	K.FLDLNKNPIHK.I	2	3.06	0.31	-2.65
IPI00021485	Leucine-rich repeat neuronal protein 1 precursor	K.IDNPHITYTAR.V	3	2.67	0.25	-2.20
IPI00021485	Leucine-rich repeat neuronal protein 1 precursor	K.TVESLPNLR.E	2	2.44	0.18	0.12
IPI00021485	Leucine-rich repeat neuronal protein 1 precursor	K.VNSNVM*TSNLK.W	2	3.53	0.44	-2.13
IPI00021485	Leucine-rich repeat neuronal protein 1 precursor	R.EATTVDCNDLR.L	2	3.14	0.30	-3.74
IPI00021485	Leucine-rich repeat neuronal protein 1 precursor	R.LKELGINNM*GELVSVDR.Y	3	3.28	0.27	-0.32
IPI00021485	Leucine-rich repeat neuronal protein 1 precursor	R.SVPALESLM*LNNNALNAIYQK.T	3	4.46	0.39	-4.42
IPI00021485	Leucine-rich repeat neuronal protein 1 precursor	R.YALDNLPELTKLEATNNPK.L	3	4.22	0.46	-3.98
IPI00021485	Leucine-rich repeat neuronal protein 1 precursor	R.YTCVAQNVQGADTR.V	2	4.08	0.30	-3.08
IPI00021552	UDP-GalNAc:beta-1,3-N-acetylgalactosaminyltransferase 1	K.FFTGYPLIDNYSYR.G	2	4.77	0.52	-4.58
IPI00021552	UDP-GalNAc:beta-1,3-N-acetylgalactosaminyltransferase 1	K.M*LALSLEDEHLLYGDIIR.Q	2	5.43	0.35	-2.61

IPI00021552	UDP-GalNAc:beta-1,3-N-acetylgalactosaminyltransferase 1	K.M*LALSLEDEHLLYGDIIR.Q	3	4.03	0.37	-3.33
IPI00021552	UDP-GalNAc:beta-1,3-N-acetylgalactosaminyltransferase 1	K.THISYQEYPFK.V	2	3.24	0.45	-1.48
IPI00021552	UDP-GalNAc:beta-1,3-N-acetylgalactosaminyltransferase 1	K.VNIHIPEDTNLFFLYR.I	2	2.93	0.13	-3.23
IPI00021552	UDP-GalNAc:beta-1,3-N-acetylgalactosaminyltransferase 1	K.VNIHIPEDTNLFFLYR.I	3	3.58	0.33	-1.87
IPI00021552	UDP-GalNAc:beta-1,3-N-acetylgalactosaminyltransferase 1	R.VIAAHGFSSK.E	1	2.22	0.22	-3.53
IPI00021552	UDP-GalNAc:beta-1,3-N-acetylgalactosaminyltransferase 1	R.VIAAHGFSSK.E	2	2.66	0.26	-0.32
IPI00021552	UDP-GalNAc:beta-1,3-N-acetylgalactosaminyltransferase 1	R.VNWM*YFYEYEPIYR.Q	2	4.62	0.52	-6.23
IPI00021552	UDP-GalNAc:beta-1,3-N-acetylgalactosaminyltransferase 1	R.VNWM*YFYEYEPIYR.Q	3	3.89	0.44	-4.23
IPI00021552	UDP-GalNAc:beta-1,3-N-acetylgalactosaminyltransferase 1	R.WVTEFCPNAK.Y	2	3.00	0.36	-2.55
IPI00021552	UDP-GalNAc:beta-1,3-N-acetylgalactosaminyltransferase 1	Y.LSLPHYNVIER.V	3	3.54	0.33	-3.69
IPI00021594	Isoform 1 of Glycosylphosphatidylinositol anchor attachment 1 protein	R.TYMSENAM*GSTM*VEEQFAGGDRAR.A	3	2.86	0.08	1.79
IPI00021634	Kinesin light chain 2	R.SSRDMAGGAGPRSESDLEDVGPTAE.W	2	3.22	0.21	-5.82
IPI00021695	Isoform D of Plasma membrane calcium-transporting ATPase 1	K.SM*STVLKNSDGSYR.I	2	2.68	0.17	
IPI00021695	Isoform D of Plasma membrane calcium-transporting ATPase 1	R.M*VTGDNINTAR.A	2	3.18	0.30	0.22
IPI00021727	C4b-binding protein alpha chain precursor	R.KPELVNGR.L	2	2.73	0.06	
IPI00021727	C4b-binding protein alpha chain precursor	R.WTPYQGCEALCCPEPK.L	2	2.81	0.06	
IPI00021733	Bifunctional heparan sulfate N-deacetylase/N-sulfotransferase 4	Q.VTSTEEYPHLKPAR.Y	2	3.63	0.33	-4.30
IPI00021753	Kinesin-like protein KIF13B	K.DRLEESEKLIQEMTVTWEEKLR.K	3	3.02	0.08	2.59
IPI00021766	Isoform 1 of Reticulon-4	K.SEIANAPDGAGSLPCTELPHDLSLK.N	4	2.90	0.14	-4.72
IPI00021770	Isoform 1 of 3-hydroxy-3-methylglutaryl-coenzyme A reductase	K.M*IMSLGLVLVHAHSR.W	2	1.77	0.08	-0.14
IPI00021794	Lysosomal protective protein precursor	K.DTVVVQDLGNIFTR.L	2	4.17	0.42	-3.43
IPI00021794	Lysosomal protective protein precursor	K.GAGHM*VPTDKPLAAFTM*FSR.F	3	4.67	0.47	-1.59
IPI00021794	Lysosomal protective protein precursor	K.YGDSGEQIAGFVK.E	2	4.68	0.47	-3.40
IPI00021794	Lysosomal protective protein precursor	R.SM*NSQYLK.L	2	2.28	0.10	-1.67
IPI00021794	Lysosomal protective protein precursor	R.YEKDTVVVQDLGNIFTR.L	3	4.83	0.33	-3.11
IPI00021812	Neuroblast differentiation-associated protein AHNAK	K.ADVDISGPK.I	2	2.39	0.13	-3.31

IPI00021817	Vitamin K-dependent protein C precursor	K.STTDNDIALLHLAQPATLSQTIVPICLPDSGLAER.E	3	4.27	0.40	-3.13
IPI00021817	Vitamin K-dependent protein C precursor	K.STTDNDIALLHLAQPATLSQTIVPICLPDSGLAER.E	4	4.19	0.37	-2.52
IPI00021817	Vitamin K-dependent protein C precursor	R.DTEDQEDQVDPR.L	2	3.51	0.42	-1.84
IPI00021817	Vitamin K-dependent protein C precursor	R.LGEYDLR.R	2	2.46	0.25	-3.19
IPI00021817	Vitamin K-dependent protein C precursor	R.TFVLNFIK.I	2	2.99	0.25	-3.39
IPI00021831	cAMP-dependent protein kinase type I-alpha regulatory subunit	K.IVVQGEPGDEFFIILEGSAAVLQR.R	3	3.78	0.23	-2.86
IPI00021833	Isoform Long of Platelet-derived growth factor A chain precursor	E.IDSVGSEDSLDTSLR.A	2	4.31	0.49	-4.37
IPI00021833	Isoform Long of Platelet-derived growth factor A chain precursor	I.DSVGSEDSLDTSLR.A	2	3.67	0.42	1.72
IPI00021833	Isoform Long of Platelet-derived growth factor A chain precursor	L.EIDSVGSEDSLDTSLR.A	2	4.25	0.53	-1.88
IPI00021833	Isoform Long of Platelet-derived growth factor A chain precursor	R.LLEIDSVGSEDSLDTSLR.A	2	6.32	0.62	-5.05
IPI00021833	Isoform Long of Platelet-derived growth factor A chain precursor	R.LLEIDSVGSEDSLDTSLR.A	3	4.90	0.47	-4.96
IPI00021834	Isoform Alpha of Tissue factor pathway inhibitor precursor	K.TTLQQEKPDFCFLEEDPGICR.G	3	3.82	0.22	-2.19
IPI00021834	Isoform Alpha of Tissue factor pathway inhibitor precursor	R.FFFNIFTR.Q	2	2.80	0.22	1.16
IPI00021841	Apolipoprotein A-I precursor	K.AKPALEDLR.Q	1	2.31	0.14	
IPI00021841	Apolipoprotein A-I precursor	K.AKPALEDLR.Q	2	2.68	0.29	
IPI00021841	Apolipoprotein A-I precursor	K.AKPALEDLRQGLLPVLESFK.V	2	4.62	0.35	
IPI00021841	Apolipoprotein A-I precursor	K.AKPALEDLRQGLLPVLESFK.V	3	4.00	0.34	
IPI00021841	Apolipoprotein A-I precursor	K.AKPALEDLRQGLLPVLESFKVSFLSALEEYTK.K	3	6.06	0.45	
IPI00021841	Apolipoprotein A-I precursor	K.AKVQPYLDDFQK.K	2	2.89	0.19	
IPI00021841	Apolipoprotein A-I precursor	K.AKVQPYLDDFQKK.W	2	3.74	0.28	
IPI00021841	Apolipoprotein A-I precursor	K.AKVQPYLDDFQKK.W	3	3.66	0.27	
IPI00021841	Apolipoprotein A-I precursor	K.ATEHLSTLSEK.A	1	3.22	0.33	
IPI00021841	Apolipoprotein A-I precursor	K.ATEHLSTLSEK.A	2	2.41	0.37	-3.11
IPI00021841	Apolipoprotein A-I precursor	K.ATEHLSTLSEK.A	3	2.83	0.25	-4.22
IPI00021841	Apolipoprotein A-I precursor	K.ATEHLSTLSEKAKPALEDLR.Q	3	3.51	0.18	
IPI00021841	Apolipoprotein A-I precursor	K.DLATVYVDVLK.D	1	2.54	0.27	
IPI00021841	Apolipoprotein A-I precursor	K.DLATVYVDVLK.D	2	3.38	0.35	-2.60
IPI00021841	Apolipoprotein A-I precursor	K.DLATVYVDVLKDSGR.D	2	4.53	0.49	
IPI00021841	Apolipoprotein A-I precursor	K.DLATVYVDVLKDSGR.D	3	3.68	0.37	
IPI00021841	Apolipoprotein A-I precursor	K.DLATVYVDVLKDSGRDYVSQFEGSALGK.Q	2	4.35	0.35	
IPI00021841	Apolipoprotein A-I precursor	K.DLATVYVDVLKDSGRDYVSQFEGSALGK.Q	3	5.36	0.36	
IPI00021841	Apolipoprotein A-I precursor	K.DSGRDYVSQFEGSALGK.Q	1	4.58	0.32	
IPI00021841	Apolipoprotein A-I precursor	K.DSGRDYVSQFEGSALGK.Q	2	2.84	0.18	-5.98
IPI00021841	Apolipoprotein A-I precursor	K.DSGRDYVSQFEGSALGK.Q	3	5.32	0.31	

IPI00021841	Apolipoprotein A-I precursor	K.ETEGLRQEM*SKDLEEVK.A	2	3.60	0.07	
IPI00021841	Apolipoprotein A-I precursor	K.ETEGLRQEM*SKDLEEVK.A	3	3.70	0.14	
IPI00021841	Apolipoprotein A-I precursor	K.ETEGLRQEM*SKDLEEVKAK.V	3	4.76	0.29	
IPI00021841	Apolipoprotein A-I precursor	K.ETEGLRQEMSKDLEEVKAK.V	3	4.36	0.19	
IPI00021841	Apolipoprotein A-I precursor	K.KWQEEM*ELYR.Q	2	2.78	0.28	
IPI00021841	Apolipoprotein A-I precursor	K.KWQEEM*ELYR.Q	3	4.18	0.14	
IPI00021841	Apolipoprotein A-I precursor	K.KWQEEMELYR.Q	2	2.18	0.18	
IPI00021841	Apolipoprotein A-I precursor	K.LLDNWDSVTSTFSK.L	1	2.70	0.26	
IPI00021841	Apolipoprotein A-I precursor	K.LLDNWDSVTSTFSK.L	2	4.61	0.43	
IPI00021841	Apolipoprotein A-I precursor	K.LREQLGPVTQEFWDNLEK.E	2	6.01	0.49	
IPI00021841	Apolipoprotein A-I precursor	K.LREQLGPVTQEFWDNLEK.E	3	6.33	0.41	
IPI00021841	Apolipoprotein A-I precursor	K.LREQLGPVTQEFWDNLEKETEGLR.Q	2	4.10	0.32	
IPI00021841	Apolipoprotein A-I precursor	K.LREQLGPVTQEFWDNLEKETEGLR.Q	3	6.84	0.42	
IPI00021841	Apolipoprotein A-I precursor	K.LREQLGPVTQEFWDNLEKETEGLRQEM*SK.D	3	5.37	0.43	
IPI00021841	Apolipoprotein A-I precursor	K.LREQLGPVTQEFWDNLEKETEGLRQEMSK.D	3	5.31	0.34	
IPI00021841	Apolipoprotein A-I precursor	K.LSPLGEEM*R.D	2	2.15	0.20	
IPI00021841	Apolipoprotein A-I precursor	K.VEPLRAELQEGAR.Q	1	2.16	0.10	
IPI00021841	Apolipoprotein A-I precursor	K.VEPLRAELQEGAR.Q	2	3.11	0.17	
IPI00021841	Apolipoprotein A-I precursor	K.VQPYLDDFQKK.W	1	2.96	0.09	
IPI00021841	Apolipoprotein A-I precursor	K.VQPYLDDFQKK.W	2	2.72	0.23	
IPI00021841	Apolipoprotein A-I precursor	K.VSFLSALEEYTK.K	1	2.99	0.32	
IPI00021841	Apolipoprotein A-I precursor	K.VSFLSALEEYTK.K	2	4.82	0.42	-4.02
IPI00021841	Apolipoprotein A-I precursor	K.VSFLSALEEYTK.K	3	3.35	0.22	
IPI00021841	Apolipoprotein A-I precursor	K.VSFLSALEEYTKK.L	1	3.00	0.28	
IPI00021841	Apolipoprotein A-I precursor	K.VSFLSALEEYTKK.L	2	4.17	0.41	
IPI00021841	Apolipoprotein A-I precursor	K.VSFLSALEEYTKK.L	3	1.94	0.20	-2.46
IPI00021841	Apolipoprotein A-I precursor	K.WQEEM*ELYR.Q	2	3.38	0.29	
IPI00021841	Apolipoprotein A-I precursor	K.WQEEMELYR.Q	2	3.32	0.17	
IPI00021841	Apolipoprotein A-I precursor	R.AHVDALR.T	1	2.18	0.12	
IPI00021841	Apolipoprotein A-I precursor	R.DYVSQFEGSALGK.Q	1	2.85	0.21	
IPI00021841	Apolipoprotein A-I precursor	R.DYVSQFEGSALGK.Q	2	4.37	0.46	-3.35
IPI00021841	Apolipoprotein A-I precursor	R.EQLGPVTQEFWDNLEK.E	2	4.35	0.50	
IPI00021841	Apolipoprotein A-I precursor	R.EQLGPVTQEFWDNLEKETEGLR.Q	2	3.31	0.40	
IPI00021841	Apolipoprotein A-I precursor	R.EQLGPVTQEFWDNLEKETEGLR.Q	3	2.91	0.34	
IPI00021841	Apolipoprotein A-I precursor	R.EQLGPVTQEFWDNLEKETEGLRQEM*SK.D	3	3.59	0.32	
IPI00021841	Apolipoprotein A-I precursor	R.EQLGPVTQEFWDNLEKETEGLRQEMSK.D	3	3.61	0.25	
IPI00021841	Apolipoprotein A-I precursor	R.EQLGPVTQEFWDNLEKETEGLRQEMSKDLEEVK.A	3	3.36	0.07	
IPI00021841	Apolipoprotein A-I precursor	R.LAARLEALKENGGAR.L	2	3.91	0.35	
IPI00021841	Apolipoprotein A-I precursor	R.LAARLEALKENGGAR.L	3	3.99	0.26	
IPI00021841	Apolipoprotein A-I precursor	R.LAEYHAK.A	2	1.85	0.08	-1.95
IPI00021841	Apolipoprotein A-I precursor	R.LEALKENGGAR.L	1	2.44	0.11	
IPI00021841	Apolipoprotein A-I precursor	R.LEALKENGGAR.L	2	2.81	0.19	-1.86

IPI00021841	Apolipoprotein A-I precursor	R.QEM*SKDLEEVKAK.V	2	2.67	0.26	
IPI00021841	Apolipoprotein A-I precursor	R.QEMSKDLEEVKAK.V	2	4.07	0.29	
IPI00021841	Apolipoprotein A-I precursor	R.QGLLPVLESFK.V	1	2.93	0.28	
IPI00021841	Apolipoprotein A-I precursor	R.QGLLPVLESFK.V	2	2.71	0.26	-4.15
IPI00021841	Apolipoprotein A-I precursor	R.QGLLPVLESFKVSFLSALEEYTK.K	3	3.76	0.15	
IPI00021841	Apolipoprotein A-I precursor	R.QKVEPLRAELQEGAR.Q	2	2.14	0.09	-3.74
IPI00021841	Apolipoprotein A-I precursor	R.THLAPYSDELR.Q	1	2.91	0.41	
IPI00021841	Apolipoprotein A-I precursor	R.THLAPYSDELR.Q	2	2.35	0.28	-3.15
IPI00021841	Apolipoprotein A-I precursor	R.THLAPYSDELR.Q	3	3.35	0.26	
IPI00021841	Apolipoprotein A-I precursor	R.THLAPYSDELRQR.L	2	3.76	0.31	
IPI00021841	Apolipoprotein A-I precursor	R.THLAPYSDELRQR.L	3	3.05	0.32	
IPI00021841	Apolipoprotein A-I precursor	R.VKDLATVYVDVLK.D	1	3.40	0.37	
IPI00021841	Apolipoprotein A-I precursor	R.VKDLATVYVDVLK.D	2	4.55	0.35	
IPI00021841	Apolipoprotein A-I precursor	R.VKDLATVYVDVLK.D	3	4.19	0.28	
IPI00021841	Apolipoprotein A-I precursor	R.VKDLATVYVDVLKDSGR.D	2	5.82	0.54	
IPI00021841	Apolipoprotein A-I precursor	R.VKDLATVYVDVLKDSGR.D	3	5.73	0.49	
IPI00021841	Apolipoprotein A-I precursor	R.VKDLATVYVDVLKDSGR.D	4	2.98	0.21	-2.97
IPI00021841	Apolipoprotein A-I precursor	R.VKDLATVYVDVLKDSGRDYVSQFEGSALGK.Q	2	5.25	0.55	
IPI00021841	Apolipoprotein A-I precursor	R.VKDLATVYVDVLKDSGRDYVSQFEGSALGK.Q	3	6.49	0.46	
IPI00021842	Apolipoprotein E precursor	A.KLEEQAQQIR.L	2	3.87	0.08	-1.82
IPI00021842	Apolipoprotein E precursor	A.KVEQAVETEPEPELR.Q	1	3.62	0.33	
IPI00021842	Apolipoprotein E precursor	A.KVEQAVETEPEPELR.Q	2	5.69	0.50	-3.34
IPI00021842	Apolipoprotein E precursor	A.KVEQAVETEPEPELR.Q	3	3.86	0.25	-2.09
IPI00021842	Apolipoprotein E precursor	K.AYKSELEEQLTPVAEETR.A	2	6.36	0.59	-2.39
IPI00021842	Apolipoprotein E precursor	K.AYKSELEEQLTPVAEETR.A	3	4.53	0.39	-2.67
IPI00021842	Apolipoprotein E precursor	K.AYKSELEEQLTPVAEETRAR.L	3	3.92	0.44	-4.99
IPI00021842	Apolipoprotein E precursor	K.AYKSELEEQLTPVAEETRAR.L	4	4.29	0.32	-3.65
IPI00021842	Apolipoprotein E precursor	K.ELKAYKSELEEQLTPVAEETR.A	3	3.47	0.20	-3.11
IPI00021842	Apolipoprotein E precursor	K.LEEQAQQIR.L	2	3.66	0.10	-5.55
IPI00021842	Apolipoprotein E precursor	K.RLAVYQAGAR.E	2	3.80	0.33	-3.66
IPI00021842	Apolipoprotein E precursor	K.RLAVYQAGAREGAER.G	3	2.76	0.18	
IPI00021842	Apolipoprotein E precursor	K.SELEEQLTPVAEETR.A	1	3.24	0.38	
IPI00021842	Apolipoprotein E precursor	K.SELEEQLTPVAEETR.A	2	5.53	0.51	-3.20
IPI00021842	Apolipoprotein E precursor	K.SELEEQLTPVAEETR.A	3	4.71	0.45	-0.84
IPI00021842	Apolipoprotein E precursor	K.SWFEPLVEDM*QR.Q	2	4.12	0.46	-4.20
IPI00021842	Apolipoprotein E precursor	K.SWFEPLVEDM*QR.Q	3	3.74	0.19	-1.73
IPI00021842	Apolipoprotein E precursor	K.SWFEPLVEDMQR.Q	1	3.25	0.31	
IPI00021842	Apolipoprotein E precursor	K.SWFEPLVEDMQR.Q	2	4.42	0.20	
IPI00021842	Apolipoprotein E precursor	K.SWFEPLVEDMQR.Q	3	4.74	0.25	
IPI00021842	Apolipoprotein E precursor	K.VEQAVETEPEPELR.Q	2	4.52	0.41	-2.95
IPI00021842	Apolipoprotein E precursor	K.VQAAVGTSAAPVPSDNH.-	1	3.22	0.51	-2.94
IPI00021842	Apolipoprotein E precursor	K.VQAAVGTSAAPVPSDNH.-	2	5.91	0.62	-4.69

IPI00021842	Apolipoprotein E precursor	K.VQAAVGTSAAPVPSDNH.-	3	3.45	0.30	-3.10
IPI00021842	Apolipoprotein E precursor	L.GPLVEQGR.V	1	1.86	0.18	-1.33
IPI00021842	Apolipoprotein E precursor	L.SEQVQEELLSSQVTQELR.A	2	3.15	0.21	0.42
IPI00021842	Apolipoprotein E precursor	L.SKELQAAQAR.L	2	3.32	0.27	0.26
IPI00021842	Apolipoprotein E precursor	R.AATVGSLAGQPLQER.A	1	3.34	0.36	-4.10
IPI00021842	Apolipoprotein E precursor	R.AATVGSLAGQPLQER.A	2	4.53	0.47	-3.64
IPI00021842	Apolipoprotein E precursor	R.AATVGSLAGQPLQER.A	3	3.64	0.37	-2.26
IPI00021842	Apolipoprotein E precursor	R.AKLEEQAQQIR.L	1	2.57	0.21	-3.71
IPI00021842	Apolipoprotein E precursor	R.AKLEEQAQQIR.L	2	4.17	0.29	-3.70
IPI00021842	Apolipoprotein E precursor	R.AKLEEQAQQIR.L	3	4.56	0.21	-4.56
IPI00021842	Apolipoprotein E precursor	R.ALM*DETM*K.E	1	1.57	0.09	-3.21
IPI00021842	Apolipoprotein E precursor	R.ALM*DETM*K.E	2	2.64	0.21	-3.35
IPI00021842	Apolipoprotein E precursor	R.ALM*DETM*KELK.A	1	2.24	0.21	-2.46
IPI00021842	Apolipoprotein E precursor	R.ALM*DETM*KELK.A	2	2.76	0.17	-2.82
IPI00021842	Apolipoprotein E precursor	R.ALM*DETMKELK.A	1	2.78	0.25	
IPI00021842	Apolipoprotein E precursor	R.ALM*DETMKELK.A	2	2.82	0.10	
IPI00021842	Apolipoprotein E precursor	R.ALMDETM*KELK.A	1	2.88	0.14	
IPI00021842	Apolipoprotein E precursor	R.ALMDETM*KELK.A	2	2.76	0.22	
IPI00021842	Apolipoprotein E precursor	R.ALMDETMKELK.A	2	2.95	0.28	
IPI00021842	Apolipoprotein E precursor	R.AQAWGER.L	1	1.58	0.17	-2.36
IPI00021842	Apolipoprotein E precursor	R.ARLSKELQAAQAR.L	3	3.76	0.10	
IPI00021842	Apolipoprotein E precursor	R.DADDLQKR.L	2	2.32	0.06	-2.65
IPI00021842	Apolipoprotein E precursor	R.DRLDEVK.E	1	1.71	0.19	-2.95
IPI00021842	Apolipoprotein E precursor	R.DRLDEVKEQVAEVR.A	1	2.52	0.18	
IPI00021842	Apolipoprotein E precursor	R.DRLDEVKEQVAEVR.A	2	4.05	0.28	-4.28
IPI00021842	Apolipoprotein E precursor	R.DRLDEVKEQVAEVR.A	3	4.25	0.31	-2.63
IPI00021842	Apolipoprotein E precursor	R.DRLDEVKEQVAEVR.A	4	3.25	0.26	-4.15
IPI00021842	Apolipoprotein E precursor	R.ERLGPLVEQGR.V	2	3.78	0.24	-3.19
IPI00021842	Apolipoprotein E precursor	R.ERLGPLVEQGR.V	3	2.34	0.08	-4.90
IPI00021842	Apolipoprotein E precursor	R.GEVQAM*LGQSTEELR.V	2	5.19	0.49	-4.19
IPI00021842	Apolipoprotein E precursor	R.GEVQAM*LGQSTEELR.V	3	3.97	0.35	-2.35
IPI00021842	Apolipoprotein E precursor	R.GEVQAM*LGQSTEELRVR.L	2	1.29	0.05	-2.51
IPI00021842	Apolipoprotein E precursor	R.GEVQAM*LGQSTEELRVR.L	3	2.73	0.36	-2.12
IPI00021842	Apolipoprotein E precursor	R.GEVQAMLGQSTEELR.V	1	3.08	0.26	
IPI00021842	Apolipoprotein E precursor	R.GEVQAMLGQSTEELR.V	2	5.46	0.32	
IPI00021842	Apolipoprotein E precursor	R.GEVQAMLGQSTEELR.V	3	3.42	0.10	
IPI00021842	Apolipoprotein E precursor	R.GEVQAMLGQSTEELRVR.L	2	3.44	0.18	
IPI00021842	Apolipoprotein E precursor	R.GEVQAMLGQSTEELRVR.L	3	2.72	0.18	
IPI00021842	Apolipoprotein E precursor	R.GLSAIRER.L	2	2.34	0.13	-2.50
IPI00021842	Apolipoprotein E precursor	R.LAVYQAGAR.E	1	2.22	0.25	-3.45
IPI00021842	Apolipoprotein E precursor	R.LAVYQAGAR.E	2	3.24	0.27	-3.07
IPI00021842	Apolipoprotein E precursor	R.LDEVKEQVAEVR.A	1	3.07	0.17	

IPI00021842	Apolipoprotein E precursor	R.LDEVKEQVAEVR.A	2	4.15	0.42	-2.43
IPI00021842	Apolipoprotein E precursor	R.LDEVKEQVAEVR.A	3	3.90	0.30	-1.09
IPI00021842	Apolipoprotein E precursor	R.LGADM*EDVCGR.L	1	1.21	0.11	-3.35
IPI00021842	Apolipoprotein E precursor	R.LGADM*EDVCGR.L	2	3.84	0.38	-4.75
IPI00021842	Apolipoprotein E precursor	R.LGADMEDVCGR.L	2	3.68	0.42	
IPI00021842	Apolipoprotein E precursor	R.LGPLVEQGR.V	1	2.22	0.25	-3.68
IPI00021842	Apolipoprotein E precursor	R.LGPLVEQGR.V	2	3.37	0.23	-2.15
IPI00021842	Apolipoprotein E precursor	R.LKSWFEPLVEDM*QR.Q	2	4.64	0.45	-3.82
IPI00021842	Apolipoprotein E precursor	R.LKSWFEPLVEDM*QR.Q	3	4.70	0.32	-3.73
IPI00021842	Apolipoprotein E precursor	R.LKSWFEPLVEDMQR.Q	1	3.59	0.36	
IPI00021842	Apolipoprotein E precursor	R.LKSWFEPLVEDMQR.Q	2	4.57	0.41	
IPI00021842	Apolipoprotein E precursor	R.LKSWFEPLVEDMQR.Q	3	5.16	0.29	
IPI00021842	Apolipoprotein E precursor	R.LLRDADDLQK.R	1	1.87	0.05	-3.17
IPI00021842	Apolipoprotein E precursor	R.LLRDADDLQKR.L	1	1.85	0.25	
IPI00021842	Apolipoprotein E precursor	R.LLRDADDLQKR.L	2	3.40	0.32	-4.42
IPI00021842	Apolipoprotein E precursor	R.LLRDADDLQKR.L	3	3.78	0.15	-2.46
IPI00021842	Apolipoprotein E precursor	R.LQAEAFQAR.L	1	2.69	0.12	-0.43
IPI00021842	Apolipoprotein E precursor	R.LQAEAFQAR.L	2	2.95	0.23	-3.17
IPI00021842	Apolipoprotein E precursor	R.LSKELQAAQAR.L	1	2.82	0.35	-3.00
IPI00021842	Apolipoprotein E precursor	R.LSKELQAAQAR.L	2	4.25	0.30	-7.09
IPI00021842	Apolipoprotein E precursor	R.LSKELQAAQAR.L	3	4.21	0.30	-4.81
IPI00021842	Apolipoprotein E precursor	R.LVQYRGEVQAM*LGQSTEELR.V	2	2.09	0.24	
IPI00021842	Apolipoprotein E precursor	R.LVQYRGEVQAM*LGQSTEELR.V	3	4.82	0.44	-6.42
IPI00021842	Apolipoprotein E precursor	R.LVQYRGEVQAM*LGQSTEELRVR.L	4	2.70	0.23	-2.97
IPI00021842	Apolipoprotein E precursor	R.LVQYRGEVQAMLGQSTEELR.V	2	5.52	0.42	
IPI00021842	Apolipoprotein E precursor	R.LVQYRGEVQAMLGQSTEELR.V	3	3.62	0.21	
IPI00021842	Apolipoprotein E precursor	R.QQTEWQSGQR.W	2	2.46	0.30	-2.71
IPI00021842	Apolipoprotein E precursor	R.QWAGLVEK.V	1	1.73	0.11	-2.52
IPI00021842	Apolipoprotein E precursor	R.QWAGLVEK.V	2	1.55	0.08	-1.75
IPI00021842	Apolipoprotein E precursor	R.TRDRLDEVKEQVAEVR.A	2	3.61	0.23	-4.42
IPI00021842	Apolipoprotein E precursor	R.TRDRLDEVKEQVAEVR.A	3	3.53	0.33	-3.67
IPI00021842	Apolipoprotein E precursor	R.TRDRLDEVKEQVAEVR.A	4	3.38	0.28	-2.89
IPI00021842	Apolipoprotein E precursor	R.VRAATVGSLAGQPLQER.A	2	5.02	0.48	-4.02
IPI00021842	Apolipoprotein E precursor	R.VRAATVGSLAGQPLQER.A	3	4.06	0.30	-2.63
IPI00021842	Apolipoprotein E precursor	R.WELALGR.F	1	2.13	0.05	-1.70
IPI00021842	Apolipoprotein E precursor	R.WELALGR.F	2	2.15	0.08	-1.45
IPI00021842	Apolipoprotein E precursor	R.WVQTLSEQVQEELLSSQVTQELR.A	2	6.29	0.58	-4.58
IPI00021842	Apolipoprotein E precursor	R.WVQTLSEQVQEELLSSQVTQELR.A	3	5.94	0.39	-4.90
IPI00021842	Apolipoprotein E precursor	V.GSLAGQPLQER.A	1	2.09	0.20	-2.55
IPI00021842	Apolipoprotein E precursor	W.VQTLSEQVQEELLSSQVTQELR.A	3	4.95	0.31	-5.48
IPI00021854	Apolipoprotein A-II precursor	K.AGTELVNFLSYFVELGTQPA.T	2	5.00	0.42	
IPI00021854	Apolipoprotein A-II precursor	K.AGTELVNFLSYFVELGTQPAT.Q	2	5.83	0.47	

IPI00021854	Apolipoprotein A-II precursor	K.AGTELVNFLSYFVELGTQPATQ.-	2	4.88	0.29	
IPI00021854	Apolipoprotein A-II precursor	K.AGTELVNFLSYFVELGTQPATQ.-	3	5.16	0.42	
IPI00021854	Apolipoprotein A-II precursor	K.EPCVESLVSQYFQTVTDYGK.D	2	5.07	0.49	-3.93
IPI00021854	Apolipoprotein A-II precursor	K.EPCVESLVSQYFQTVTDYGK.D	3	3.88	0.39	
IPI00021854	Apolipoprotein A-II precursor	K.EPCVESLVSQYFQTVTDYGKDLM*EK.V	2	4.20	0.42	
IPI00021854	Apolipoprotein A-II precursor	K.EPCVESLVSQYFQTVTDYGKDLM*EK.V	3	3.32	0.36	
IPI00021854	Apolipoprotein A-II precursor	K.EPCVESLVSQYFQTVTDYGKDLMEK.V	2	3.15	0.36	
IPI00021854	Apolipoprotein A-II precursor	K.EPCVESLVSQYFQTVTDYGKDLMEK.V	3	3.11	0.28	
IPI00021854	Apolipoprotein A-II precursor	K.KAGTELVNFLSYFVELGTQPA.T	2	4.94	0.25	
IPI00021854	Apolipoprotein A-II precursor	K.KAGTELVNFLSYFVELGTQPAT.Q	2	5.20	0.47	
IPI00021854	Apolipoprotein A-II precursor	K.KAGTELVNFLSYFVELGTQPATQ.-	2	5.84	0.48	
IPI00021854	Apolipoprotein A-II precursor	K.KAGTELVNFLSYFVELGTQPATQ.-	3	2.44	0.29	-4.10
IPI00021854	Apolipoprotein A-II precursor	K.SKEQLTPLIK.K	1	2.72	0.22	
IPI00021854	Apolipoprotein A-II precursor	K.SKEQLTPLIK.K	2	2.47	0.20	-1.93
IPI00021854	Apolipoprotein A-II precursor	K.SKEQLTPLIKK.A	2	3.41	0.20	
IPI00021854	Apolipoprotein A-II precursor	K.SKEQLTPLIKK.A	3	3.50	0.21	
IPI00021854	Apolipoprotein A-II precursor	K.SPELQAEAK.S	1	2.63	0.17	
IPI00021854	Apolipoprotein A-II precursor	K.SPELQAEAK.S	2	3.48	0.12	
IPI00021854	Apolipoprotein A-II precursor	K.SYFEKSKEQLTPLIK.K	2	4.06	0.36	
IPI00021854	Apolipoprotein A-II precursor	K.SYFEKSKEQLTPLIK.K	3	2.32	0.16	
IPI00021854	Apolipoprotein A-II precursor	K.SYFEKSKEQLTPLIKK.A	3	2.58	0.37	
IPI00021854	Apolipoprotein A-II precursor	K.VKSPELQAEAK.S	1	2.54	0.23	
IPI00021854	Apolipoprotein A-II precursor	K.VKSPELQAEAK.S	2	2.65	0.15	-3.71
IPI00021854	Apolipoprotein A-II precursor	K.VKSPELQAEAK.S	3	4.01	0.15	
IPI00021854	Apolipoprotein A-II precursor	R.QAKEPCVESLVSQYFQTVTDYGK.D	2	2.75	0.38	
IPI00021854	Apolipoprotein A-II precursor	R.QAKEPCVESLVSQYFQTVTDYGK.D	3	5.98	0.46	
IPI00021854	Apolipoprotein A-II precursor	R.QAKEPCVESLVSQYFQTVTDYGKDLM*EK.V	3	2.66	0.28	
IPI00021855	Apolipoprotein C-I precursor	G.TPDVSSALDKLKEFGNTLEDK.A	2	5.96	0.44	
IPI00021855	Apolipoprotein C-I precursor	K.LKEFGNTLEDKAR.E	2	3.12	0.23	
IPI00021855	Apolipoprotein C-I precursor	K.M*REWFSETFQK.V	2	2.73	0.17	
IPI00021855	Apolipoprotein C-I precursor	R.IKQSELSAK.M	2	2.52	0.07	-2.24
IPI00021856	Apolipoprotein C-II precursor	G.TQQPQQDEM*PSPTFLTQVK.E	2	5.31	0.39	
IPI00021856	Apolipoprotein C-II precursor	K.STAAM*STYTGIFTDQVLSVLKGEE.-	2	4.45	0.38	
IPI00021856	Apolipoprotein C-II precursor	K.STAAMSTYTGIFTDQVLSVLK.G	2	3.53	0.22	
IPI00021856	Apolipoprotein C-II precursor	K.STAAMSTYTGIFTDQVLSVLKGEE.-	2	4.27	0.38	
IPI00021856	Apolipoprotein C-II precursor	K.TAAQNLYEK.T	2	1.70	0.06	0.41
IPI00021857	Apolipoprotein C-III precursor	A.SEAEDASLLSFMQGYM*K.H	2	5.33	0.38	
IPI00021857	Apolipoprotein C-III precursor	K.DALSSVQESQVAQQAR.G	2	4.82	0.35	-2.42
IPI00021857	Apolipoprotein C-III precursor	K.DALSSVQESQVAQQAR.G	3	3.98	0.35	-1.33
IPI00021857	Apolipoprotein C-III precursor	K.TAKDALSSVQESQVAQQAR.G	2	6.55	0.41	
IPI00021857	Apolipoprotein C-III precursor	K.TAKDALSSVQESQVAQQAR.G	3	4.76	0.24	
IPI00021857	Apolipoprotein C-III precursor	R.GWVTDGFSSLK.D	2	3.21	0.34	

IPI00021857	Apolipoprotein C-III precursor	R.GWVTDGFSSLKDYWSTVK.D	2	4.63	0.47	
IPI00021857	Apolipoprotein C-III precursor	R.GWVTDGFSSLKDYWSTVK.D	3	2.43	0.18	
IPI00021885	Isoform 1 of Fibrinogen alpha chain precursor	A.DSGEGDFLAEGGGVR.G	2	5.20	0.44	-6.02
IPI00021885	Isoform 1 of Fibrinogen alpha chain precursor	D.SGEGDFLAEGGGVR.G	2	4.59	0.33	-2.03
IPI00021885	Isoform 1 of Fibrinogen alpha chain precursor	K.ALTDM*PQM*R.M	2	2.32	0.22	1.05
IPI00021885	Isoform 1 of Fibrinogen alpha chain precursor	K.DSHSLTTNIM*EILR.G	3	3.52	0.16	-1.25
IPI00021885	Isoform 1 of Fibrinogen alpha chain precursor	K.ESSSHHPGIAEFPSR.G	2	2.86	0.23	
IPI00021885	Isoform 1 of Fibrinogen alpha chain precursor	K.ESSSHHPGIAEFPSR.G	3	1.85	0.15	-3.72
IPI00021885	Isoform 1 of Fibrinogen alpha chain precursor	K.EVTKEVVTSEDGSDCPEAM*DLGTLSGIGTLDGFR.H	3	6.72	0.54	
IPI00021885	Isoform 1 of Fibrinogen alpha chain precursor	K.EVTKEVVTSEDGSDCPEAMDLGTLSGIGTLDGFR.H	3	5.86	0.49	
IPI00021885	Isoform 1 of Fibrinogen alpha chain precursor	K.EVVTSEDGSDCPEAM*DLGTLSGIGTLDGFR.H	2	4.73	0.39	
IPI00021885	Isoform 1 of Fibrinogen alpha chain precursor	K.EVVTSEDGSDCPEAM*DLGTLSGIGTLDGFR.H	3	6.18	0.52	
IPI00021885	Isoform 1 of Fibrinogen alpha chain precursor	K.GLIDEVNQDFTNR.I	2	3.96	0.41	
IPI00021885	Isoform 1 of Fibrinogen alpha chain precursor	K.LKNSLFEYQK.N	2	2.74	0.27	
IPI00021885	Isoform 1 of Fibrinogen alpha chain precursor	K.LVTSKGDKELR.T	2	3.37	0.28	
IPI00021885	Isoform 1 of Fibrinogen alpha chain precursor	K.M*ADEAGSEADHEGTHSTKR.G	3	2.95	0.13	
IPI00021885	Isoform 1 of Fibrinogen alpha chain precursor	K.NNKDSHSLTTNIM*EILR.G	2	5.01	0.38	
IPI00021885	Isoform 1 of Fibrinogen alpha chain precursor	K.NSLFEYQK.N	1	2.42	0.12	
IPI00021885	Isoform 1 of Fibrinogen alpha chain precursor	K.QFTSSTSYNR.G	2	2.19	0.25	
IPI00021885	Isoform 1 of Fibrinogen alpha chain precursor	K.QFTSSTSYNRGDSTFESK.S	2	4.06	0.34	
IPI00021885	Isoform 1 of Fibrinogen alpha chain precursor	K.TFPGFFSPM*LGEFVSETESR.G	2	4.17	0.40	
IPI00021885	Isoform 1 of Fibrinogen alpha chain precursor	K.TFPGFFSPM*LGEFVSETESR.G	3	4.78	0.39	
IPI00021885	Isoform 1 of Fibrinogen alpha chain precursor	K.TFPGFFSPMLGEFVSETESR.G	2	5.07	0.34	
IPI00021885	Isoform 1 of Fibrinogen alpha chain precursor	K.TVIGPDGHKEVTK.E	2	3.68	0.31	
IPI00021885	Isoform 1 of Fibrinogen alpha chain precursor	K.TVIGPDGHKEVTKEVVTSEDGSDCPEAM*DLGTLSGIGTLDGFR.H	3	4.54	0.31	
IPI00021885	Isoform 1 of Fibrinogen alpha chain precursor	K.VQHIQLLQK.N	1	2.64	0.15	
IPI00021885	Isoform 1 of Fibrinogen alpha chain precursor	K.VQHIQLLQK.N	2	2.48	0.15	
IPI00021885	Isoform 1 of Fibrinogen alpha chain precursor	R.EVDLKDYEDQQK.Q	2	3.98	0.14	
IPI00021885	Isoform 1 of Fibrinogen alpha chain precursor	R.EVDLKDYEDQQKQLEQVIAK.D	2	4.47	0.38	
IPI00021885	Isoform 1 of Fibrinogen alpha chain precursor	R.GDFSSANNR.D	2	2.22	0.13	
IPI00021885	Isoform 1 of Fibrinogen alpha chain precursor	R.GDFSSANNRDNTYNR.V	2	2.37	0.17	
IPI00021885	Isoform 1 of Fibrinogen alpha chain precursor	R.GGSTSYGTGSETESPR.N	2	4.33	0.55	-3.28
IPI00021885	Isoform 1 of Fibrinogen alpha chain precursor	R.GGSTSYGTGSETESPRN.P	2	3.95	0.55	-3.91
IPI00021885	Isoform 1 of Fibrinogen alpha chain precursor	R.GGSTSYGTGSETESPRNPSSAGSWNSGSSGPGSTGNR.N	3	7.35	0.55	
IPI00021885	Isoform 1 of Fibrinogen alpha chain precursor	R.GSESGIFTNTK.E	1	2.46	0.13	
IPI00021885	Isoform 1 of Fibrinogen alpha chain precursor	R.GSESGIFTNTK.E	2	2.82	0.32	-1.59
IPI00021885	Isoform 1 of Fibrinogen alpha chain precursor	R.HPDEAAFFDTASTGK.T	2	2.96	0.28	
IPI00021885	Isoform 1 of Fibrinogen alpha chain precursor	R.HRHPDEAAFFDTASTGK.T	2	4.59	0.39	
IPI00021885	Isoform 1 of Fibrinogen alpha chain precursor	R.M*ELERPGGNEITR.G	3	2.78	0.17	
IPI00021885	Isoform 1 of Fibrinogen alpha chain precursor	R.M*KGLIDEVNQDFTNR.I	2	3.76	0.24	
IPI00021885	Isoform 1 of Fibrinogen alpha chain precursor	R.M*KGLIDEVNQDFTNR.I	3	4.38	0.16	
IPI00021885	Isoform 1 of Fibrinogen alpha chain precursor	R.MKGLIDEVNQDFTNR.I	3	3.99	0.21	

IPI00021885	Isoform 1 of Fibrinogen alpha chain precursor	T.ADSGEGDFLAEGGGVR.G	2	4.92	0.40	-5.07
IPI00021891	Isoform Gamma-B of Fibrinogen gamma chain precursor	K.AIQLTYNPDESSKPNM*IDAATLK.S	2	3.98	0.32	
IPI00021891	Isoform Gamma-B of Fibrinogen gamma chain precursor	K.AIQLTYNPDESSKPNM*IDAATLK.S	3	2.95	0.30	-2.35
IPI00021891	Isoform Gamma-B of Fibrinogen gamma chain precursor	K.AIQLTYNPDESSKPNMIDAATLK.S	2	4.25	0.35	
IPI00021891	Isoform Gamma-B of Fibrinogen gamma chain precursor	K.AIQLTYNPDESSKPNMIDAATLK.S	3	3.87	0.32	
IPI00021891	Isoform Gamma-B of Fibrinogen gamma chain precursor	K.ASTPNGYDNGIIWATWK.T	2	4.75	0.35	
IPI00021891	Isoform Gamma-B of Fibrinogen gamma chain precursor	K.EGFGHLSPTGTTEFWLGNEK.I	2	5.42	0.41	
IPI00021891	Isoform Gamma-B of Fibrinogen gamma chain precursor	K.EGFGHLSPTGTTEFWLGNEK.I	3	4.21	0.31	
IPI00021891	Isoform Gamma-B of Fibrinogen gamma chain precursor	K.IHLISTQSAIPYALR.V	2	5.04	0.40	
IPI00021891	Isoform Gamma-B of Fibrinogen gamma chain precursor	K.QSGLYFIKPLK.A	2	2.36	0.21	
IPI00021891	Isoform Gamma-B of Fibrinogen gamma chain precursor	K.VAQLEAQCQEPCKDTVQIHDITGK.D	2	3.29	0.08	
IPI00021891	Isoform Gamma-B of Fibrinogen gamma chain precursor	K.VAQLEAQCQEPCKDTVQIHDITGK.D	3	5.52	0.44	
IPI00021891	Isoform Gamma-B of Fibrinogen gamma chain precursor	K.VAQLEAQCQEPCKDTVQIHDITGKDCQDIANK.G	3	4.78	0.28	
IPI00021891	Isoform Gamma-B of Fibrinogen gamma chain precursor	K.VGPEADKYR.L	2	2.16	0.21	
IPI00021891	Isoform Gamma-B of Fibrinogen gamma chain precursor	K.YEASILTHDSSIR.Y	2	4.12	0.45	
IPI00021891	Isoform Gamma-B of Fibrinogen gamma chain precursor	K.YEASILTHDSSIR.Y	3	2.47	0.20	
IPI00021891	Isoform Gamma-B of Fibrinogen gamma chain precursor	R.DNCCILDER.F	2	3.10	0.20	
IPI00021891	Isoform Gamma-B of Fibrinogen gamma chain precursor	R.FGSYCPTTCGIADFLSTYQTK.V	2	5.27	0.45	
IPI00021891	Isoform Gamma-B of Fibrinogen gamma chain precursor	R.FGSYCPTTCGIADFLSTYQTK.V	3	4.45	0.33	
IPI00021891	Isoform Gamma-B of Fibrinogen gamma chain precursor	R.KM*LEEIM*KYEASILTHDSSIR.Y	3	3.53	0.38	
IPI00021891	Isoform Gamma-B of Fibrinogen gamma chain precursor	R.LDGSVDFK.K	2	2.45	0.16	
IPI00021891	Isoform Gamma-B of Fibrinogen gamma chain precursor	R.LDGSVDFKK.N	2	2.85	0.29	

IPI00021891	Isoform Gamma-B of Fibrinogen gamma chain precursor	R.LTIGEGQQHHLGGAK.Q	2	3.81	0.38	
IPI00021891	Isoform Gamma-B of Fibrinogen gamma chain precursor	R.LTYAYFAGGDAGDAFDGFDFGDDPSDK.F	2	5.17	0.54	
IPI00021891	Isoform Gamma-B of Fibrinogen gamma chain precursor	R.TSTADYAM*FK.V	1	2.16	0.22	
IPI00021891	Isoform Gamma-B of Fibrinogen gamma chain precursor	R.TSTADYAM*FK.V	2	2.94	0.29	
IPI00021891	Isoform Gamma-B of Fibrinogen gamma chain precursor	R.YLQEIYNSNNQK.I	2	5.32	0.31	
IPI00021900	Tumor necrosis factor ligand superfamily member 12	R.ASLSAQEPAQEELVAEEDQDPSELNPQTEESQDPAPFLNR.L	3	6.34	0.57	-2.85
IPI00021900	Tumor necrosis factor ligand superfamily member 12	R.ASLSAQEPAQEELVAEEDQDPSELNPQTEESQDPAPFLNR.L	4	4.11	0.50	-2.80
IPI00021903	Isoform Alpha of ADAM 23 precursor	K.SVVNLVDSIYK.E	2	2.85	0.26	-1.87
IPI00021903	Isoform Alpha of ADAM 23 precursor	K.SVVNLVDSIYKEQLNTR.V	2	4.26	0.52	-2.36
IPI00021903	Isoform Alpha of ADAM 23 precursor	K.SVVNLVDSIYKEQLNTR.V	3	3.26	0.45	-3.19
IPI00021903	Isoform Alpha of ADAM 23 precursor	K.TLAGQYSK.Q	1	1.58	0.06	-1.84
IPI00021903	Isoform Alpha of ADAM 23 precursor	K.TLAGQYSK.Q	2	1.89	0.08	0.22
IPI00021903	Isoform Alpha of ADAM 23 precursor	R.IGQLQGEIIPTSFYHQGR.V	3	3.46	0.31	-0.96
IPI00021903	Isoform Alpha of ADAM 23 precursor	R.LIYYINQDSESPYHV.L	2	4.52	0.53	-4.91
IPI00021903	Isoform Alpha of ADAM 23 precursor	R.LIYYINQDSESPYHVLDTK.A	2	5.53	0.49	-3.77
IPI00021903	Isoform Alpha of ADAM 23 precursor	R.LIYYINQDSESPYHVLDTK.A	3	3.29	0.47	-1.63
IPI00021903	Isoform Alpha of ADAM 23 precursor	R.SSLSYFGGVCSR.T	2	2.75	0.34	-2.19
IPI00021907	Isoform 1 of Myelin basic protein	R.TAHYGSLPQK.S	2	2.32	0.16	-2.05
IPI00021951	Uncharacterized protein KIAA0247 precursor	R.SVPREQQLPDQGACSSA.G	3	3.98	0.18	2.01
IPI00021983	Isoform 1 of Nicastrin precursor	K.ALADVATVLGR.A	2	3.46	0.34	-1.78
IPI00021983	Isoform 1 of Nicastrin precursor	K.GKFPVQLENVDSFVELGQVALR.T	3	3.34	0.36	-3.11
IPI00021983	Isoform 1 of Nicastrin precursor	K.SGAGVPAVILR.R	2	2.56	0.28	-1.49
IPI00021983	Isoform 1 of Nicastrin precursor	R.LLYGFLIK.A	2	2.14	0.09	-2.23
IPI00021983	Isoform 1 of Nicastrin precursor	R.NQVEDLLATLEK.S	2	2.85	0.41	-3.35
IPI00021985	transmembrane 9 superfamily protein member 4	R.ITEDYYVHLIADNLPVATR.L	3	3.87	0.30	-2.66
IPI00021997	Protein CREG1 precursor	K.IVTPEEYYNVTVQ.-	2	2.44	0.18	-2.49
IPI00021997	Protein CREG1 precursor	R.FVTHVSDWGALATISTLEAVR.G	3	2.47	0.13	-3.99
IPI00022039	Isoform 3 of SLAM family member 5 precursor	K.TSVAYVTPGDSETAPVVTVTHR.N	3	2.67	0.19	-2.33
IPI00022039	Isoform 3 of SLAM family member 5 precursor	R.IHALGPNYNLVISDLR.M	2	3.57	0.43	-4.28
IPI00022039	Isoform 3 of SLAM family member 5 precursor	R.IHALGPNYNLVISDLR.M	3	3.49	0.23	-4.11
IPI00022055	Histone acetyltransferase PCAF	K.TKYVGYIKDYEGATLM*GCELNPR.I	3	3.84	0.14	
IPI00022078	Protein NDRG1	R.SHTSEGAHLDITPNSGAAGNSAGPK.S	4	3.04	0.07	-3.29
IPI00022229	Apolipoprotein B-100 precursor	K.ALVEQGFTVPEIK.T	2	3.20	0.20	
IPI00022229	Apolipoprotein B-100 precursor	K.ALYWVNGQVPDGVSK.V	2	3.67	0.09	
IPI00022229	Apolipoprotein B-100 precursor	K.AQNLYQELLTQEGQASFQGLKDNVFDGLVR.V	3	4.52	0.40	

IPI00022229	Apolipoprotein B-100 precursor	K.ATGVLYDYVNK.Y	2	3.52	0.35	
IPI00022229	Apolipoprotein B-100 precursor	K.AVSMPSFSILGSDVR.V	2	4.17	0.41	
IPI00022229	Apolipoprotein B-100 precursor	K.DKIGVELTGR.T	2	2.38	0.14	
IPI00022229	Apolipoprotein B-100 precursor	K.EVYGFNPEGK.A	2	2.60	0.36	
IPI00022229	Apolipoprotein B-100 precursor	K.FPEVDVLTK.Y	2	2.60	0.23	
IPI00022229	Apolipoprotein B-100 precursor	K.FSVPAGIVIPSFQALTAR.F	2	3.89	0.45	-3.40
IPI00022229	Apolipoprotein B-100 precursor	K.FSVPAGIVIPSFQALTAR.F	3	3.31	0.07	-3.20
IPI00022229	Apolipoprotein B-100 precursor	K.FVTQAEGAK.Q	2	2.86	0.14	
IPI00022229	Apolipoprotein B-100 precursor	K.GFEPTLEALFGK.Q	2	3.66	0.31	
IPI00022229	Apolipoprotein B-100 precursor	K.GFEPTLEALFGKQGFFPDSVNK.A	3	4.28	0.32	
IPI00022229	Apolipoprotein B-100 precursor	K.GISTSAASPAVGTVGM*DM*DEDDDFSK.W	2	3.61	0.30	
IPI00022229	Apolipoprotein B-100 precursor	K.GISTSAASPAVGTVGM*DM*DEDDDFSK.W	3	2.78	0.21	
IPI00022229	Apolipoprotein B-100 precursor	K.GM*TRPLSTLISSSQSCQYTLDAK.R	2	4.30	0.35	
IPI00022229	Apolipoprotein B-100 precursor	K.GM*TRPLSTLISSSQSCQYTLDAK.R	3	4.85	0.24	
IPI00022229	Apolipoprotein B-100 precursor	K.GNVATEISTER.D	2	3.32	0.36	
IPI00022229	Apolipoprotein B-100 precursor	K.HVAEAICK.E	2	2.55	0.14	
IPI00022229	Apolipoprotein B-100 precursor	K.IADFELPTIIVPEQTIEIPSIK.F	2	3.18	0.35	-3.71
IPI00022229	Apolipoprotein B-100 precursor	K.IADFELPTIIVPEQTIEIPSIK.F	3	4.08	0.13	
IPI00022229	Apolipoprotein B-100 precursor	K.IAELSATAQEIIK.S	2	4.29	0.29	
IPI00022229	Apolipoprotein B-100 precursor	K.IEGNLIFDPNNYLPK.E	2	4.19	0.19	
IPI00022229	Apolipoprotein B-100 precursor	K.INNQLTLDSNTK.Y	2	2.20	0.15	
IPI00022229	Apolipoprotein B-100 precursor	K.KLTISEQNIQR.A	2	3.20	0.28	-2.73
IPI00022229	Apolipoprotein B-100 precursor	K.KM*GLAFESTK.S	2	2.07	0.24	
IPI00022229	Apolipoprotein B-100 precursor	K.LDVTTSIGR.R	2	2.77	0.25	
IPI00022229	Apolipoprotein B-100 precursor	K.LKTQFNNNEYSQDLDAYNTK.D	3	3.52	0.29	
IPI00022229	Apolipoprotein B-100 precursor	K.LLSGGNTLHLVSTTK.T	2	4.68	0.37	
IPI00022229	Apolipoprotein B-100 precursor	K.LNDLNSVLVM*PTFHVPFTDLQVPSCK.L	3	4.54	0.36	
IPI00022229	Apolipoprotein B-100 precursor	K.LNDLNSVLVMPTFHVPFTDLQVPSCK.L	3	3.70	0.29	
IPI00022229	Apolipoprotein B-100 precursor	K.LRTSSFALNLPTLPEVKFPEVDVLTK.Y	3	5.08	0.23	
IPI00022229	Apolipoprotein B-100 precursor	K.LTISEQNIQR.A	2	3.19	0.16	-2.19
IPI00022229	Apolipoprotein B-100 precursor	K.NFATSNKM*DM*TFSK.Q	2	3.57	0.34	
IPI00022229	Apolipoprotein B-100 precursor	K.NFVASHIANILNSEELDIQDLKK.L	3	6.94	0.46	
IPI00022229	Apolipoprotein B-100 precursor	K.NKADYVETVLDSTCSSTVQFLEYELNVLGTHK.I	3	5.08	0.40	
IPI00022229	Apolipoprotein B-100 precursor	K.NPNGYSFSIPVK.V	2	3.45	0.39	
IPI00022229	Apolipoprotein B-100 precursor	K.NSEEFAAAM*SR.Y	2	3.87	0.43	
IPI00022229	Apolipoprotein B-100 precursor	K.QTVNLQLQPYSLVTTLNSDLKYNALDLTNNGK.L	3	4.16	0.32	
IPI00022229	Apolipoprotein B-100 precursor	K.QVFLYPEKDEPTYILNIK.R	2	4.81	0.19	
IPI00022229	Apolipoprotein B-100 precursor	K.QVFLYPEKDEPTYILNIKR.G	2	2.82	0.22	
IPI00022229	Apolipoprotein B-100 precursor	K.SHDELPR.T	2	1.65	0.06	-3.18
IPI00022229	Apolipoprotein B-100 precursor	K.SKPTVSSSM*EFK.Y	2	2.33	0.15	
IPI00022229	Apolipoprotein B-100 precursor	K.SPAFTDLHLR.Y	2	3.74	0.34	
IPI00022229	Apolipoprotein B-100 precursor	K.SVGFHLPSR.E	2	2.35	0.28	

IPI00022229	Apolipoprotein B-100 precursor	K.SVSDGIAALDLNAVANK.I	2	5.01	0.35	
IPI00022229	Apolipoprotein B-100 precursor	K.SVSDGIAALDLNAVANK.I	3	4.41	0.31	
IPI00022229	Apolipoprotein B-100 precursor	K.SVSDGIAALDLNAVANKIADFELPTIIVPEQTIEIPSIK.F	3	4.06	0.24	
IPI00022229	Apolipoprotein B-100 precursor	K.SVSLPSLDPASAK.I	2	3.30	0.19	
IPI00022229	Apolipoprotein B-100 precursor	K.TKNSEEFAAAM*SR.Y	2	4.74	0.43	
IPI00022229	Apolipoprotein B-100 precursor	K.TNPTGTQELLDIANYLM*EQIQDDCTGDEDYTYLILR.V	3	3.40	0.31	
IPI00022229	Apolipoprotein B-100 precursor	K.TSQCTLKEVYGFNPEGK.A	2	4.99	0.38	
IPI00022229	Apolipoprotein B-100 precursor	K.VELEVPQLCSFILK.T	2	2.79	0.20	
IPI00022229	Apolipoprotein B-100 precursor	K.VLVDHFGYTK.D	2	2.05	0.17	
IPI00022229	Apolipoprotein B-100 precursor	K.VNWEEEAASGLLTSLKDNVPK.A	3	3.53	0.33	
IPI00022229	Apolipoprotein B-100 precursor	K.VPLLLSEPINIIDALEM*R.D	3	4.43	0.16	
IPI00022229	Apolipoprotein B-100 precursor	K.YDKNQDVHSINLPFFETLQEYFER.N	3	4.57	0.25	
IPI00022229	Apolipoprotein B-100 precursor	K.YGM*VAQVTQTLK.L	2	4.02	0.40	
IPI00022229	Apolipoprotein B-100 precursor	K.YSQPEDSLIPFFEITVPESQLTVSQFTLPK.S	2	3.56	0.22	
IPI00022229	Apolipoprotein B-100 precursor	K.YSQPEDSLIPFFEITVPESQLTVSQFTLPK.S	3	3.32	0.33	-4.95
IPI00022229	Apolipoprotein B-100 precursor	K.YTYNYEAESSSGVPGTADSR.S	2	6.34	0.50	
IPI00022229	Apolipoprotein B-100 precursor	K.YTYNYEAESSSGVPGTADSR.S	3	4.90	0.26	
IPI00022229	Apolipoprotein B-100 precursor	R.AALGKLPQQANDYLNSFNWER.Q	2	2.88	0.13	
IPI00022229	Apolipoprotein B-100 precursor	R.DLKVEDIPLAR.I	2	3.37	0.11	
IPI00022229	Apolipoprotein B-100 precursor	R.EYSGTIASEANTYLNSK.S	2	3.52	0.35	
IPI00022229	Apolipoprotein B-100 precursor	R.HSITNPLAVLCEFISQSIK.S	3	4.53	0.29	
IPI00022229	Apolipoprotein B-100 precursor	R.IGQDGISTSATTNLK.C	2	3.87	0.37	
IPI00022229	Apolipoprotein B-100 precursor	R.IHSGSFQSQVELSNDQEK.A	3	3.49	0.12	
IPI00022229	Apolipoprotein B-100 precursor	R.ILGEELGFASLHDLQLLGK.L	2	4.70	0.36	
IPI00022229	Apolipoprotein B-100 precursor	R.ILGEELGFASLHDLQLLGK.L	3	2.84	0.34	-3.29
IPI00022229	Apolipoprotein B-100 precursor	R.INCKVELEVPQLCSFILK.T	2	5.49	0.33	
IPI00022229	Apolipoprotein B-100 precursor	R.KGNVATEISTER.D	2	3.72	0.33	
IPI00022229	Apolipoprotein B-100 precursor	R.KYTYNYEAESSSGVPGTADSR.S	2	6.26	0.50	
IPI00022229	Apolipoprotein B-100 precursor	R.LELELRPTGEIEQYSVSATYELQR.E	3	4.83	0.20	
IPI00022229	Apolipoprotein B-100 precursor	R.LNTDIAGLASAIDM*STNYNSDSLHFSNVFR.S	3	6.93	0.50	
IPI00022229	Apolipoprotein B-100 precursor	R.LPYTIITTPPLKDFSLWEK.T	3	3.33	0.27	
IPI00022229	Apolipoprotein B-100 precursor	R.M*NFKQELNGNTK.S	2	4.15	0.20	
IPI00022229	Apolipoprotein B-100 precursor	R.NLQNNAEWVYQGAIR.Q	2	5.02	0.41	
IPI00022229	Apolipoprotein B-100 precursor	R.NLQNNAEWVYQGAIR.Q	3	3.90	0.19	
IPI00022229	Apolipoprotein B-100 precursor	R.SEYQADYESLR.F	2	3.10	0.21	
IPI00022229	Apolipoprotein B-100 precursor	R.SPSQADINK.I	2	2.65	0.10	
IPI00022229	Apolipoprotein B-100 precursor	R.TFQIPGYTVPVVNVEVSPFTIEM*SAFGYVFPK.A	3	3.83	0.23	
IPI00022229	Apolipoprotein B-100 precursor	R.TGISPLALIK.G	2	2.82	0.31	-1.33
IPI00022229	Apolipoprotein B-100 precursor	R.TLADLTLLDSPIKVPLLLSEPINIIDALEM*R.D	3	3.76	0.36	-4.01
IPI00022229	Apolipoprotein B-100 precursor	R.TSSFALNLPTLPEVKFPEVDVLTK.Y	2	3.66	0.23	
IPI00022229	Apolipoprotein B-100 precursor	R.TSSFALNLPTLPEVKFPEVDVLTK.Y	3	4.00	0.42	-2.36
IPI00022229	Apolipoprotein B-100 precursor	R.VIGNM*GQTM*EQLTPELK.S	2	3.12	0.20	-4.16

IPI00022229	Apolipoprotein B-100 precursor	R.VPSYTLILPSLELPVLHVPR.N	3	5.05	0.44	-3.83
IPI00022229	Apolipoprotein B-100 precursor	R.YEDGTLSLTSTSDLQSGIIK.N	2	3.20	0.26	
IPI00022277	Coiled-coil domain-containing protein 56	R.FLDELEDEAK.A	2	3.27	0.19	-1.89
IPI00022284	Major prion protein precursor	A.AGAVVGGLGGYM*LGSAM*SRPIIHFGSDYEDR.Y	3	3.71	0.37	-4.31
IPI00022284	Major prion protein precursor	A.VVGGLGGYM*LGSAM*SRPIIHFGSDYEDR.Y	3	4.59	0.46	-3.19
IPI00022284	Major prion protein precursor	G.AAAAGAVVGGLGGYM*LGSAM*SRPIIHFGSDYEDR.Y	3	4.99	0.46	-2.76
IPI00022284	Major prion protein precursor	G.AVVGGLGGYM*LGSAM*SRPIIHFGSDYEDR.Y	3	3.51	0.39	-2.68
IPI00022284	Major prion protein precursor	G.GLGGYM*LGSAM*SRPIIHFGSDYEDR.Y	3	3.95	0.46	-4.65
IPI00022284	Major prion protein precursor	H.M*AGAAAAGAVVGGLGGYM*LGSAM*SRPIIHFGSDYEDR.Y	3	5.61	0.50	-2.85
IPI00022284	Major prion protein precursor	H.M*AGAAAAGAVVGGLGGYM*LGSAM*SRPIIHFGSDYEDR.Y	4	4.41	0.46	-1.05
IPI00022284	Major prion protein precursor	K.GENFTETDVK.M	1	2.36	0.23	-3.68
IPI00022284	Major prion protein precursor	K.GENFTETDVK.M	2	3.67	0.39	-2.95
IPI00022284	Major prion protein precursor	K.HM*AGAAAAGAVVGGLGGYM*LGSAM*SR.P	3	5.35	0.51	-4.63
IPI00022284	Major prion protein precursor	K.QHTVTTTTKGENFTETDVK.M	2	4.11	0.57	-4.84
IPI00022284	Major prion protein precursor	K.QHTVTTTTKGENFTETDVK.M	3	2.31	0.28	-5.18
IPI00022284	Major prion protein precursor	K.QHTVTTTTKGENFTETDVK.M	4	2.51	0.32	-0.50
IPI00022284	Major prion protein precursor	M.SRPIIHFGSDYEDR.Y	2	3.46	0.19	-3.02
IPI00022284	Major prion protein precursor	N.TGGSRYPGQGSPGGNR.Y	3	3.64	0.32	-2.14
IPI00022284	Major prion protein precursor	R.ESQAYYQR.G	1	2.03	0.10	-3.22
IPI00022284	Major prion protein precursor	R.ESQAYYQR.G	2	2.66	0.20	-2.55
IPI00022284	Major prion protein precursor	R.PIIHFGSDYEDR.Y	2	3.37	0.49	-2.22
IPI00022284	Major prion protein precursor	R.PIIHFGSDYEDR.Y	3	4.28	0.53	-2.60
IPI00022284	Major prion protein precursor	R.PIIHFGSDYEDRYYR.E	2	3.88	0.41	-4.50
IPI00022284	Major prion protein precursor	R.PIIHFGSDYEDRYYR.E	3	3.50	0.40	-2.98
IPI00022284	Major prion protein precursor	R.VVEQM*CITQYER.E	2	4.47	0.50	-3.85
IPI00022284	Major prion protein precursor	R.VVEQM*CITQYER.E	3	2.68	0.09	-0.76
IPI00022284	Major prion protein precursor	R.VVEQMCITQYER.E	2	1.99	0.12	-1.80
IPI00022284	Major prion protein precursor	R.YPGQGSPGGNR.Y	2	2.53	0.31	
IPI00022284	Major prion protein precursor	R.YPNQVYYRPM*DEYSNQNNFVHDCVNITIK.Q	3	4.63	0.36	-2.75
IPI00022284	Major prion protein precursor	R.YPNQVYYRPM*DEYSNQNNFVHDCVNITIK.Q	4	3.70	0.38	-2.96
IPI00022284	Major prion protein precursor	S.RPIIHFGSDYEDR.Y	3	4.07	0.31	-2.44
IPI00022295	Platelet factor 4 variant precursor	R.HITSLEVIK.A	2	1.89	0.14	-1.80
IPI00022296	Mast/stem cell growth factor receptor precursor	K.AVPVVSVSK.A	1	2.03	0.34	-2.68
IPI00022296	Mast/stem cell growth factor receptor precursor	K.DVSSSVYSTWK.R	2	3.45	0.39	-0.92
IPI00022296	Mast/stem cell growth factor receptor precursor	K.EDNDTLVR.C	1	1.79	0.10	
IPI00022296	Mast/stem cell growth factor receptor precursor	K.LVVQSSIDSSAFK.H	2	4.37	0.46	-3.77
IPI00022296	Mast/stem cell growth factor receptor precursor	K.TSAYFNFAFK.G	2	2.47	0.26	-1.92
IPI00022296	Mast/stem cell growth factor receptor precursor	R.CPLTDPEVTNYSLK.G	2	3.51	0.34	-3.45
IPI00022296	Mast/stem cell growth factor receptor precursor	R.LVNGM*LQCVAAGFPEPTIDWYFCPGTEQR.C	3	4.20	0.41	-3.11
IPI00022296	Mast/stem cell growth factor receptor precursor	R.QATLTISSAR.V	1	1.85	0.20	-2.47
IPI00022296	Mast/stem cell growth factor receptor precursor	R.QATLTISSAR.V	2	2.98	0.36	-2.26

IPI00022314	Superoxide dismutase [Mn], mitochondrial precursor	K.AIWNVINWENVTER.Y	2	4.15	0.42	-3.17
IPI00022314	Superoxide dismutase [Mn], mitochondrial precursor	K.AIWNVINWENVTER.Y	3	5.02	0.44	-2.93
IPI00022331	Phosphatidylcholine-sterol acyltransferase precursor	K.LAGYLHTLVQNLVNNGYVRDETVR.A	3	2.73	0.08	-4.38
IPI00022331	Phosphatidylcholine-sterol acyltransferase precursor	K.LDKPDVVNWMCYR.K	2	2.33	0.13	
IPI00022331	Phosphatidylcholine-sterol acyltransferase precursor	K.TYSVEYLDSSK.L	1	2.42	0.39	-3.66
IPI00022331	Phosphatidylcholine-sterol acyltransferase precursor	K.TYSVEYLDSSK.L	2	2.34	0.22	-3.66
IPI00022331	Phosphatidylcholine-sterol acyltransferase precursor	R.DLLAGLPAPGVEVYCLYGVGLPTPR.T	2	3.59	0.47	-1.98
IPI00022331	Phosphatidylcholine-sterol acyltransferase precursor	R.DLLAGLPAPGVEVYCLYGVGLPTPR.T	3	2.62	0.16	-1.62
IPI00022331	Phosphatidylcholine-sterol acyltransferase precursor	R.ITTTSPWM*FPSR.M	2	3.29	0.38	-1.77
IPI00022331	Phosphatidylcholine-sterol acyltransferase precursor	R.LEPGQQEEYYR.K	2	2.07	0.28	-2.67
IPI00022331	Phosphatidylcholine-sterol acyltransferase precursor	R.SSGLVSNAPGVQIR.V	1	2.46	0.24	-1.71
IPI00022331	Phosphatidylcholine-sterol acyltransferase precursor	R.SSGLVSNAPGVQIR.V	2	4.25	0.33	-3.22
IPI00022331	Phosphatidylcholine-sterol acyltransferase precursor	R.STELCGLWQGR.Q	1	2.12	0.28	-2.99
IPI00022331	Phosphatidylcholine-sterol acyltransferase precursor	R.STELCGLWQGR.Q	2	3.26	0.26	-3.02
IPI00022331	Phosphatidylcholine-sterol acyltransferase precursor	R.TYIYDHGFPYTDPVGVLYEDGDDTVATR.S	2	4.67	0.63	-3.30
IPI00022331	Phosphatidylcholine-sterol acyltransferase precursor	R.TYIYDHGFPYTDPVGVLYEDGDDTVATR.S	3	7.13	0.49	-5.00
IPI00022331	Phosphatidylcholine-sterol acyltransferase precursor	R.VPGFGK.T	1	1.75	0.19	-2.39
IPI00022333	Brain-specific angiogenesis inhibitor 1 precursor	R.AGPPGPTDDFSVEYLVVGNR.N	2	5.47	0.53	-4.17
IPI00022333	Brain-specific angiogenesis inhibitor 1 precursor	R.DCGGGLQTR.T	2	2.55	0.17	-1.64
IPI00022333	Brain-specific angiogenesis inhibitor 1 precursor	R.LCDPSAPLAFLQASK.Q	2	3.64	0.24	-1.90
IPI00022333	Brain-specific angiogenesis inhibitor 1 precursor	R.TYLGVESFDEVLR.L	2	4.81	0.47	-3.22
IPI00022333	Brain-specific angiogenesis inhibitor 1 precursor	R.TYQFDSFLESTR.T	2	3.84	0.48	-5.07
IPI00022337	Interphotoreceptor retinoid-binding protein precursor	K.AAHIPENAK.D	2	1.60	0.08	-1.58

IPI00022337	Interphotoreceptor retinoid-binding protein precursor	K.ALAILTLR.S	2	2.79	0.11	-2.13
IPI00022337	Interphotoreceptor retinoid-binding protein precursor	K.DRIPGIVPM*QIPSPEVFEELIK.F	3	2.81	0.06	-1.70
IPI00022337	Interphotoreceptor retinoid-binding protein precursor	K.SHEILSISDPQTLASVLTAGVQSSLNDPR.L	3	6.21	0.56	-3.28
IPI00022337	Interphotoreceptor retinoid-binding protein precursor	K.TEVLPGQLGYLR.F	2	2.86	0.36	-2.19
IPI00022337	Interphotoreceptor retinoid-binding protein precursor	R.AKVPTVLQTAGK.L	2	2.47	0.18	-1.63
IPI00022337	Interphotoreceptor retinoid-binding protein precursor	R.FDSFADASVLGVLAPYVLR.Q	2	3.29	0.30	-3.40
IPI00022337	Interphotoreceptor retinoid-binding protein precursor	R.FDSFADASVLGVLAPYVLR.Q	3	3.69	0.29	-2.89
IPI00022337	Interphotoreceptor retinoid-binding protein precursor	R.FNIGGPTSSIPILCSYFFDEGPPVLLDK.I	3	2.69	0.15	-1.72
IPI00022337	Interphotoreceptor retinoid-binding protein precursor	R.GVYLLTSHR.T	2	2.18	0.21	-1.40
IPI00022337	Interphotoreceptor retinoid-binding protein precursor	R.IGESDFFFTVPVSR.S	2	2.38	0.24	-4.52
IPI00022337	Interphotoreceptor retinoid-binding protein precursor	R.SVGASDGSSWEGVGVTPHVVVPAEEALAR.A	3	2.94	0.14	-3.50
IPI00022337	Interphotoreceptor retinoid-binding protein precursor	R.TAVDLESLASQLTADLQEVSGDHR.L	2	4.42	0.51	-5.26
IPI00022337	Interphotoreceptor retinoid-binding protein precursor	R.TAVDLESLASQLTADLQEVSGDHR.L	3	4.55	0.38	-4.62
IPI00022337	Interphotoreceptor retinoid-binding protein precursor	R.TAVDLESLASQLTADLQEVSGDHR.L	4	3.52	0.18	-2.95
IPI00022337	Interphotoreceptor retinoid-binding protein precursor	R.TGGGALDLR.K	2	2.45	0.15	-1.09
IPI00022337	Interphotoreceptor retinoid-binding protein precursor	R.VTSEVALAEILGADLQM*LSGDPHLK.A	3	3.60	0.36	-5.04
IPI00022361	Band 3 anion transport protein	F.SGPLLVFEEAFFSFCETNGLEYIVGR.V	3	3.70	0.30	-2.06
IPI00022361	Band 3 anion transport protein	K.GTVLLDLQETSLAGVAN.Q	2	3.06	0.27	-3.24
IPI00022361	Band 3 anion transport protein	K.GTVLLDLQETSLAGVANQLLDR.F	2	6.19	0.57	-4.63
IPI00022361	Band 3 anion transport protein	K.GTVLLDLQETSLAGVANQLLDR.F	3	5.39	0.48	-4.92
IPI00022361	Band 3 anion transport protein	K.HSHAGELEALGGVKPAVLTR.S	4	3.48	0.34	-0.57
IPI00022361	Band 3 anion transport protein	K.VYVELQELVM*DEK.N	2	4.67	0.37	-4.32
IPI00022361	Band 3 anion transport protein	K.VYVELQELVMDEK.N	2	4.68	0.39	-3.62
IPI00022361	Band 3 anion transport protein	K.VYVELQELVMDEK.N	3	3.28	0.16	-2.77
IPI00022361	Band 3 anion transport protein	R.ADFLEQPVLGFVR.L	2	2.18	0.18	-4.08
IPI00022361	Band 3 anion transport protein	R.FLFVLLGPEAPHIDYTQLGR.A	2	4.84	0.54	-4.91

IPI00022361	Band 3 anion transport protein	R.FLFVLLGPEAPHIDYTQLGR.A	3	4.00	0.39	-4.18
IPI00022361	Band 3 anion transport protein	R.GELLHSLEGFLDCSLVLPPTDAPSEQALLSLVPVQR.E	3	4.37	0.36	-5.38
IPI00022361	Band 3 anion transport protein	R.LQEAAELEAVELPVPIR.F	3	3.56	0.19	-3.04
IPI00022361	Band 3 anion transport protein	R.LQEAAELEAVELPVPIRFLFVLLGPEAPHIDYTQLGR.A	4	2.73	0.15	-2.34
IPI00022361	Band 3 anion transport protein	R.PHLSHLTFWSLLELR.R	2	4.32	0.28	-1.85
IPI00022361	Band 3 anion transport protein	R.PHLSHLTFWSLLELR.R	3	5.00	0.41	-3.88
IPI00022361	Band 3 anion transport protein	R.WVQLEENLGENGAWGR.P	2	5.29	0.51	-5.95
IPI00022367	Isoform 2 of Astrotactin-1 precursor	K.EVAAGQVLK.G	2	2.06	0.14	-3.39
IPI00022367	Isoform 2 of Astrotactin-1 precursor	K.ITLHVPEHLIADGSR.F	3	2.31	0.31	-0.11
IPI00022367	Isoform 2 of Astrotactin-1 precursor	K.ITLHVPEHLIADGSR.F	4	2.93	0.31	-1.97
IPI00022367	Isoform 2 of Astrotactin-1 precursor	K.LNQVAISQALSNALHSLDGATSR.A	3	3.70	0.29	-2.51
IPI00022367	Isoform 2 of Astrotactin-1 precursor	K.SITVSALPFLR.E	2	3.40	0.33	-2.43
IPI00022371	Histidine-rich glycoprotein precursor	A.VSPTDCSAVEPEAEK.A	2	3.23	0.44	-2.90
IPI00022371	Histidine-rich glycoprotein precursor	K.DSPVLIDFFEDTER.Y	2	4.86	0.52	-4.38
IPI00022371	Histidine-rich glycoprotein precursor	K.DSPVLIDFFEDTERYR.K	2	3.40	0.33	
IPI00022371	Histidine-rich glycoprotein precursor	K.DSPVLIDFFEDTERYR.K	3	1.55	0.14	-3.29
IPI00022371	Histidine-rich glycoprotein precursor	K.GEVLPLPEANFPSFPLPHHK.H	2	2.65	0.34	-3.37
IPI00022371	Histidine-rich glycoprotein precursor	K.GEVLPLPEANFPSFPLPHHK.H	4	3.42	0.39	-3.25
IPI00022371	Histidine-rich glycoprotein precursor	K.HPLKPDNQPFPQSVSESCPGK.F	3	4.51	0.32	
IPI00022371	Histidine-rich glycoprotein precursor	K.SGFPQVSM*FFTHTFPK.-	2	4.27	0.41	-4.14
IPI00022371	Histidine-rich glycoprotein precursor	K.SGFPQVSM*FFTHTFPK.-	3	1.58	0.17	-3.68
IPI00022371	Histidine-rich glycoprotein precursor	K.SGFPQVSMFFTHTFPK.-	2	3.19	0.24	
IPI00022371	Histidine-rich glycoprotein precursor	K.YKEENDDFASFR.V	2	4.24	0.44	-2.42
IPI00022371	Histidine-rich glycoprotein precursor	K.YKEENDDFASFR.V	3	3.06	0.19	-2.05
IPI00022371	Histidine-rich glycoprotein precursor	R.ADLFYDVEALDLESPK.N	2	5.30	0.44	-6.33
IPI00022371	Histidine-rich glycoprotein precursor	R.ADLFYDVEALDLESPK.N	3	4.83	0.44	-3.70
IPI00022371	Histidine-rich glycoprotein precursor	R.DGYLFQLLR.I	1	2.21	0.29	-2.40
IPI00022371	Histidine-rich glycoprotein precursor	R.DGYLFQLLR.I	2	3.45	0.15	-3.30
IPI00022371	Histidine-rich glycoprotein precursor	R.GGEGTGYFVDFSVR.N	2	4.41	0.50	-3.01
IPI00022371	Histidine-rich glycoprotein precursor	R.IADAHLDR.V	2	2.33	0.16	-3.40
IPI00022371	Histidine-rich glycoprotein precursor	R.KGEVLPLPEANFPSFPLPHHK.H	3	3.67	0.29	-4.44
IPI00022371	Histidine-rich glycoprotein precursor	R.KYWNDCEPPDSR.R	2	3.48	0.33	
IPI00022371	Histidine-rich glycoprotein precursor	R.RDGYLFQLLR.I	2	2.53	0.16	-2.56
IPI00022371	Histidine-rich glycoprotein precursor	R.RPSEIVIGQCK.V	2	3.66	0.19	
IPI00022371	Histidine-rich glycoprotein precursor	R.RRDGYLFQLLR.I	3	2.77	0.17	-3.29
IPI00022371	Histidine-rich glycoprotein precursor	R.VRGGEGTGYFVDFSVR.N	2	2.27	0.16	-3.77
IPI00022371	Histidine-rich glycoprotein precursor	R.VRGGEGTGYFVDFSVR.N	3	3.91	0.33	-3.64
IPI00022389	Isoform 1 of C-reactive protein precursor	R.GYSIFSYATK.R	2	3.28	0.19	-1.26
IPI00022389	Isoform 1 of C-reactive protein precursor	R.KAFVFPK.E	2	1.72	0.08	-5.01
IPI00022391	Serum amyloid P-component precursor	K.IVLGQEQDSYGGKFDR.S	2	4.34	0.47	-2.25
IPI00022391	Serum amyloid P-component precursor	K.IVLGQEQDSYGGKFDR.S	3	2.07	0.15	-1.34
IPI00022391	Serum amyloid P-component precursor	R.AYSLFSYNTQGR.D	2	3.65	0.43	-2.04

IPI00022391	Serum amyloid P-component precursor	R.DNELLVYK.E	2	2.69	0.20	
IPI00022391	Serum amyloid P-component precursor	R.DNELLVYKER.V	2	2.88	0.27	-1.92
IPI00022391	Serum amyloid P-component precursor	R.GYVIIKPLVWV.-	2	2.79	0.21	-2.37
IPI00022391	Serum amyloid P-component precursor	R.QGYFVEAQPK.I	2	2.85	0.24	-1.19
IPI00022391	Serum amyloid P-component precursor	R.VGEYSLYIGR.H	2	3.39	0.28	
IPI00022392	Complement C1q subcomponent subunit A precursor	K.GHIYQGSEADSVFSGFLIFPSA.-	2	4.07	0.46	-4.11
IPI00022392	Complement C1q subcomponent subunit A precursor	K.KGHIYQGSEADSVFSGFLIFPSA.-	2	3.86	0.50	-5.89
IPI00022392	Complement C1q subcomponent subunit A precursor	K.KGHIYQGSEADSVFSGFLIFPSA.-	3	3.24	0.38	-5.73
IPI00022392	Complement C1q subcomponent subunit A precursor	R.SLGFCDTTNK.G	2	2.28	0.26	-1.24
IPI00022394	Complement C1q subcomponent subunit C precursor	K.FQSVFTVTR.Q	2	3.00	0.18	-1.27
IPI00022394	Complement C1q subcomponent subunit C precursor	K.TNQVNSGGVLLR.L	2	3.46	0.21	-1.97
IPI00022394	Complement C1q subcomponent subunit C precursor	R.FNAVLTNPQGDYDTSTGK.F	2	5.45	0.53	-3.49
IPI00022395	Complement component C9 precursor	A.SSINDAPVLISQK.L	2	3.00	0.27	-2.57
IPI00022395	Complement component C9 precursor	D.RDGNTLTYYR.R	2	3.06	0.23	-2.93
IPI00022395	Complement component C9 precursor	I.EDYINEFSVRK.C	2	3.41	0.18	-2.50
IPI00022395	Complement component C9 precursor	K.ALPTTYEK.G	1	1.90	0.26	-3.16
IPI00022395	Complement component C9 precursor	K.CLCACPFKFEGIACEISK.Q	3	3.47	0.22	-7.59
IPI00022395	Complement component C9 precursor	K.FEGIACEISK.Q	1	2.33	0.28	-4.76
IPI00022395	Complement component C9 precursor	K.FTPTETNKAEQCCEETASSISLHGK.G	3	4.18	0.45	-3.33
IPI00022395	Complement component C9 precursor	K.ISEGLPALEFPNE.K	2	4.07	0.37	-4.01
IPI00022395	Complement component C9 precursor	K.LSPIYNLVPVK.M	2	3.28	0.33	-4.29
IPI00022395	Complement component C9 precursor	K.NFRTEHYEEQIEAFK.S	2	4.05	0.38	-3.25
IPI00022395	Complement component C9 precursor	K.NFRTEHYEEQIEAFK.S	3	3.01	0.24	-4.40
IPI00022395	Complement component C9 precursor	K.QKISEGLPALEFPNE.K	2	3.91	0.36	-2.70
IPI00022395	Complement component C9 precursor	K.TSNFNAAISLK.F	1	2.31	0.21	-3.24
IPI00022395	Complement component C9 precursor	K.TSNFNAAISLK.F	2	3.83	0.38	-3.71
IPI00022395	Complement component C9 precursor	K.YAFELKEK.L	1	2.29	0.17	-3.96
IPI00022395	Complement component C9 precursor	P.WNVASLIYETK.G	2	3.22	0.28	-2.77
IPI00022395	Complement component C9 precursor	R.AIEDYINEFSVR.K	1	2.68	0.34	-3.64
IPI00022395	Complement component C9 precursor	R.AIEDYINEFSVR.K	2	4.61	0.46	-3.63
IPI00022395	Complement component C9 precursor	R.AIEDYINEFSVR.K	3	4.36	0.34	-1.59
IPI00022395	Complement component C9 precursor	R.AIEDYINEFSVRK.C	2	3.78	0.43	-3.25
IPI00022395	Complement component C9 precursor	R.AIEDYINEFSVRK.C	3	1.91	0.16	-1.36
IPI00022395	Complement component C9 precursor	R.CNGDNDCGDFSDEDDCESEPRPPCR.D	3	5.42	0.67	-3.92
IPI00022395	Complement component C9 precursor	R.DGNTLTYYR.R	2	2.80	0.22	-1.62

IPI00022395	Complement component C9 precursor	R.DRDGNTLTYYR.R	2	3.01	0.17	-3.97
IPI00022395	Complement component C9 precursor	R.DRVVEESELAR.T	2	3.69	0.36	-2.89
IPI00022395	Complement component C9 precursor	R.DRVVEESELAR.T	3	3.38	0.10	-2.72
IPI00022395	Complement component C9 precursor	R.DVVLTTTFVDDIK.A	2	4.79	0.44	-4.93
IPI00022395	Complement component C9 precursor	R.GTVIDVTDFVNWASSINDAPVLISQK.L	2	5.51	0.61	-3.84
IPI00022395	Complement component C9 precursor	R.GTVIDVTDFVNWASSINDAPVLISQK.L	3	5.75	0.60	-5.77
IPI00022395	Complement component C9 precursor	R.KGVELKDIKR.C	2	3.00	0.12	-3.71
IPI00022395	Complement component C9 precursor	R.KYAFELK.E	1	2.85	0.08	-2.63
IPI00022395	Complement component C9 precursor	R.KYAFELK.E	2	2.58	0.07	-1.60
IPI00022395	Complement component C9 precursor	R.KYAFELKEK.L	2	2.60	0.14	-3.03
IPI00022395	Complement component C9 precursor	R.NRDVVLTTTFVDDIK.A	2	4.45	0.41	-5.21
IPI00022395	Complement component C9 precursor	R.NRDVVLTTTFVDDIK.A	3	2.64	0.27	-2.39
IPI00022395	Complement component C9 precursor	R.RPWNVASLIYETK.G	2	2.99	0.22	-3.13
IPI00022395	Complement component C9 precursor	R.RPWNVASLIYETK.G	3	2.88	0.10	-0.26
IPI00022395	Complement component C9 precursor	R.SIEVFGQFNGK.R	1	2.85	0.34	-2.10
IPI00022395	Complement component C9 precursor	R.SIEVFGQFNGK.R	2	3.08	0.23	-0.22
IPI00022395	Complement component C9 precursor	R.SIEVFGQFNGKR.C	2	2.96	0.32	-4.01
IPI00022395	Complement component C9 precursor	R.SIEVFGQFNGKR.C	3	2.34	0.19	-3.82
IPI00022395	Complement component C9 precursor	R.TAGYGINILGM*DPLSTPFDNEFYNGLCNR.D	3	4.63	0.38	-3.83
IPI00022395	Complement component C9 precursor	R.TEHYEEQIEAFK.S	1	1.98	0.32	-0.09
IPI00022395	Complement component C9 precursor	R.TEHYEEQIEAFK.S	2	4.07	0.44	-3.72
IPI00022395	Complement component C9 precursor	R.TEHYEEQIEAFK.S	3	3.98	0.23	-2.61
IPI00022395	Complement component C9 precursor	R.TEHYEEQIEAFKSIIQEK.T	3	3.98	0.35	-3.82
IPI00022395	Complement component C9 precursor	R.VVEESELAR.T	1	2.43	0.27	-3.76
IPI00022395	Complement component C9 precursor	R.VVEESELAR.T	2	3.06	0.24	-2.76
IPI00022395	Complement component C9 precursor	W.ASSINDAPVLISQK.L	2	4.05	0.34	-3.01
IPI00022395	Complement component C9 precursor	W.NVASLIYETK.G	1	2.43	0.25	-3.18
IPI00022395	Complement component C9 precursor	W.NVASLIYETK.G	2	3.10	0.27	-2.81
IPI00022417	Leucine-rich alpha-2-glycoprotein precursor	K.ALGHLDLSGNR.L	1	2.81	0.38	-3.54
IPI00022417	Leucine-rich alpha-2-glycoprotein precursor	K.ALGHLDLSGNR.L	2	3.05	0.37	-2.87
IPI00022417	Leucine-rich alpha-2-glycoprotein precursor	K.DLLLPQPDLR.Y	1	2.29	0.07	
IPI00022417	Leucine-rich alpha-2-glycoprotein precursor	K.DLLLPQPDLR.Y	2	2.79	0.23	-3.87
IPI00022417	Leucine-rich alpha-2-glycoprotein precursor	K.ENQLEVLEVSWLHGLK.A	2	4.50	0.40	-0.58
IPI00022417	Leucine-rich alpha-2-glycoprotein precursor	K.ENQLEVLEVSWLHGLK.A	3	2.76	0.21	-0.75
IPI00022417	Leucine-rich alpha-2-glycoprotein precursor	K.GQTLLAVAK.S	2	2.77	0.23	-1.89
IPI00022417	Leucine-rich alpha-2-glycoprotein precursor	K.LQELHLSSNGLESLSPEFLRPVPQLR.V	2	4.23	0.46	
IPI00022417	Leucine-rich alpha-2-glycoprotein precursor	K.LQELHLSSNGLESLSPEFLRPVPQLR.V	3	5.16	0.51	-3.07
IPI00022417	Leucine-rich alpha-2-glycoprotein precursor	K.LQELHLSSNGLESLSPEFLRPVPQLR.V	4	3.81	0.23	-3.94
IPI00022417	Leucine-rich alpha-2-glycoprotein precursor	K.LQVLGKDLLLPQPDLR.Y	2	4.47	0.37	
IPI00022417	Leucine-rich alpha-2-glycoprotein precursor	K.LQVLGKDLLLPQPDLR.Y	3	4.10	0.17	
IPI00022417	Leucine-rich alpha-2-glycoprotein precursor	R.CAGPEAVKGQTLLAVAK.S	2	4.97	0.31	
IPI00022417	Leucine-rich alpha-2-glycoprotein precursor	R.CAGPEAVKGQTLLAVAK.S	3	2.67	0.25	

IPI00022417	Leucine-rich alpha-2-glycoprotein precursor	R.DGFDISGNPWICDQNLSDLYR.W	2	5.26	0.44	
IPI00022417	Leucine-rich alpha-2-glycoprotein precursor	R.DGFDISGNPWICDQNLSDLYR.W	3	4.17	0.27	
IPI00022417	Leucine-rich alpha-2-glycoprotein precursor	R.LHLEGNK.L	1	2.42	0.11	-2.95
IPI00022417	Leucine-rich alpha-2-glycoprotein precursor	R.LHLEGNKLQVLGK.D	2	3.26	0.37	-4.21
IPI00022417	Leucine-rich alpha-2-glycoprotein precursor	R.LHLEGNKLQVLGK.D	3	2.73	0.23	
IPI00022417	Leucine-rich alpha-2-glycoprotein precursor	R.LHLEGNKLQVLGKDLLLPQPDLR.Y	3	5.33	0.40	
IPI00022417	Leucine-rich alpha-2-glycoprotein precursor	R.NALTGLPPGLFQASATLDTLVLK.E	2	3.72	0.47	-5.13
IPI00022417	Leucine-rich alpha-2-glycoprotein precursor	R.NALTGLPPGLFQASATLDTLVLK.E	3	4.98	0.53	-4.25
IPI00022417	Leucine-rich alpha-2-glycoprotein precursor	R.TLDLGENQLETLPPDLLR.G	2	4.93	0.44	-5.65
IPI00022417	Leucine-rich alpha-2-glycoprotein precursor	R.TLDLGENQLETLPPDLLR.G	3	3.07	0.27	-3.98
IPI00022417	Leucine-rich alpha-2-glycoprotein precursor	R.TLDLGENQLETLPPDLLRGPLQLER.L	3	4.49	0.39	
IPI00022417	Leucine-rich alpha-2-glycoprotein precursor	R.VAAGAFQGLR.Q	1	2.02	0.13	-0.93
IPI00022417	Leucine-rich alpha-2-glycoprotein precursor	R.VAAGAFQGLR.Q	2	3.88	0.34	-1.36
IPI00022417	Leucine-rich alpha-2-glycoprotein precursor	R.WLQAQKDK.M	2	2.79	0.06	
IPI00022417	Leucine-rich alpha-2-glycoprotein precursor	R.YLFLNGNK.L	2	2.43	0.17	-1.60
IPI00022420	Plasma retinol-binding protein precursor	D.PNGLPPEAQK.I	2	3.02	0.23	-1.93
IPI00022420	Plasma retinol-binding protein precursor	F.SVDETGQM*SATAK.G	2	4.27	0.49	-1.84
IPI00022420	Plasma retinol-binding protein precursor	K.GNDDHWIVDTDYDTYAVQYSCR.L	2	4.43	0.33	
IPI00022420	Plasma retinol-binding protein precursor	K.GNDDHWIVDTDYDTYAVQYSCR.L	3	4.69	0.58	-5.04
IPI00022420	Plasma retinol-binding protein precursor	K.KDPEGLFLQDNIVAEFSVDETGQM*SATAK.G	3	6.60	0.53	-7.06
IPI00022420	Plasma retinol-binding protein precursor	K.M*KYWGVASFLQK.G	2	3.19	0.31	-4.04
IPI00022420	Plasma retinol-binding protein precursor	K.M*KYWGVASFLQK.G	3	2.92	0.11	-1.05
IPI00022420	Plasma retinol-binding protein precursor	K.YWGVASFLQK.G	1	3.00	0.18	-1.99
IPI00022420	Plasma retinol-binding protein precursor	K.YWGVASFLQK.G	2	3.40	0.27	-2.48
IPI00022420	Plasma retinol-binding protein precursor	R.DPNGLPPEAQK.I	1	2.28	0.35	-3.98
IPI00022420	Plasma retinol-binding protein precursor	R.DPNGLPPEAQK.I	2	3.87	0.41	-5.44
IPI00022420	Plasma retinol-binding protein precursor	R.FSGTWYAM*AK.K	1	2.15	0.37	-3.26
IPI00022420	Plasma retinol-binding protein precursor	R.FSGTWYAM*AK.K	2	3.50	0.42	-3.15
IPI00022420	Plasma retinol-binding protein precursor	R.LIVHNGYCDGR.S	2	2.69	0.39	-3.70
IPI00022420	Plasma retinol-binding protein precursor	R.LIVHNGYCDGR.S	3	2.14	0.10	-4.31
IPI00022420	Plasma retinol-binding protein precursor	R.LLNLDGTCADSYSFVFSR.D	2	5.92	0.63	-8.74
IPI00022420	Plasma retinol-binding protein precursor	R.LLNLDGTCADSYSFVFSR.D	3	4.75	0.51	-5.11
IPI00022420	Plasma retinol-binding protein precursor	R.LLNLDGTCADSYSFVFSRDPNGLPPEAQK.I	3	5.23	0.48	-4.79
IPI00022420	Plasma retinol-binding protein precursor	R.LLNLDGTCADSYSFVFSRDPNGLPPEAQK.I	4	3.31	0.26	-3.07
IPI00022420	Plasma retinol-binding protein precursor	R.LLNNWDVCADM*VGTFTDTEDPAKFK.M	3	4.29	0.54	-4.46
IPI00022420	Plasma retinol-binding protein precursor	R.QEELCLAR.Q	2	1.84	0.13	-2.94
IPI00022420	Plasma retinol-binding protein precursor	R.VKENFDK.A	1	2.41	0.08	-2.55
IPI00022420	Plasma retinol-binding protein precursor	R.VKENFDKAR.F	1	2.90	0.11	-4.25
IPI00022420	Plasma retinol-binding protein precursor	R.VKENFDKAR.F	2	3.40	0.17	-2.51
IPI00022420	Plasma retinol-binding protein precursor	W.DVCADM*VGTFTDTEDPAK.F	2	5.19	0.57	-2.81
IPI00022426	AMBP protein precursor	K.CVLFPYGGCQGNGNK.F	2	3.51	0.26	-3.04
IPI00022426	AMBP protein precursor	K.EDSCQLGYSAGPCM*GM*TSR.Y	2	4.94	0.62	-3.85

IPI00022426	AMBP protein precursor	K.EDSCQLGYSAGPCM*GM*TSR.Y	3	3.01	0.35	-4.20
IPI00022426	AMBP protein precursor	K.FYSEKECR.E	2	1.88	0.19	-2.76
IPI00022426	AMBP protein precursor	K.GVCEETSGAYEKTDTDGK.F	2	5.17	0.46	
IPI00022426	AMBP protein precursor	K.GVCEETSGAYEKTDTDGK.F	3	2.49	0.32	-1.16
IPI00022426	AMBP protein precursor	K.KEDSCQLGYSAGPCM*GM*TSR.Y	3	4.57	0.31	
IPI00022426	AMBP protein precursor	K.TDTDGKFLYHK.S	2	3.12	0.42	-3.11
IPI00022426	AMBP protein precursor	K.WYNLAIGSTCPWLK.K	2	3.40	0.35	
IPI00022426	AMBP protein precursor	K.WYNLAIGSTCPWLKK.I	2	2.97	0.14	
IPI00022426	AMBP protein precursor	R.AFIQLWAFDAVK.G	1	3.08	0.20	-2.71
IPI00022426	AMBP protein precursor	R.AFIQLWAFDAVK.G	2	5.07	0.35	-8.77
IPI00022426	AMBP protein precursor	R.AFIQLWAFDAVK.G	3	3.99	0.27	-2.42
IPI00022426	AMBP protein precursor	R.ETLLQDFR.V	1	2.13	0.18	-0.22
IPI00022426	AMBP protein precursor	R.ETLLQDFR.V	2	2.71	0.15	-1.22
IPI00022426	AMBP protein precursor	R.EYCGVPGDGDEELLR.F	2	3.31	0.51	-4.62
IPI00022426	AMBP protein precursor	R.GECVPGEQEPEPILIPR.V	2	3.64	0.36	
IPI00022426	AMBP protein precursor	R.KGVCEETSGAYEK.T	2	3.79	0.30	
IPI00022426	AMBP protein precursor	R.KGVCEETSGAYEKTDTDGK.F	3	4.44	0.27	
IPI00022426	AMBP protein precursor	R.M*TVSTLVLGEGATEAEISM*TSTR.W	2	5.70	0.58	-5.20
IPI00022426	AMBP protein precursor	R.M*TVSTLVLGEGATEAEISM*TSTR.W	3	4.32	0.48	-3.40
IPI00022426	AMBP protein precursor	R.TVAACNLPIVR.G	1	2.92	0.24	-2.01
IPI00022426	AMBP protein precursor	R.TVAACNLPIVR.G	2	4.38	0.28	-3.20
IPI00022426	AMBP protein precursor	R.VVAQGVGIPEDSIFTM*ADR.G	2	4.66	0.43	
IPI00022426	AMBP protein precursor	R.VVAQGVGIPEDSIFTM*ADR.G	3	4.87	0.40	-3.70
IPI00022426	AMBP protein precursor	R.VVAQGVGIPEDSIFTM*ADRGECVPGEQEPEPILIPR.V	3	4.83	0.36	-2.86
IPI00022426	AMBP protein precursor	R.VVAQGVGIPEDSIFTM*ADRGECVPGEQEPEPILIPR.V	4	4.15	0.45	-3.54
IPI00022426	AMBP protein precursor	R.VVAQGVGIPEDSIFTMADRGECVPGEQEPEPILIPR.V	3	5.14	0.41	
IPI00022429	Alpha-1-acid glycoprotein 1 precursor	K.EQLGEFYEALDCLR.I	1	2.42	0.20	
IPI00022429	Alpha-1-acid glycoprotein 1 precursor	K.EQLGEFYEALDCLR.I	2	4.28	0.45	
IPI00022429	Alpha-1-acid glycoprotein 1 precursor	K.EQLGEFYEALDCLR.I	3	5.31	0.31	
IPI00022429	Alpha-1-acid glycoprotein 1 precursor	K.EQLGEFYEALDCLRIPK.S	2	4.57	0.43	
IPI00022429	Alpha-1-acid glycoprotein 1 precursor	K.NWGLSVYADKPETTKEQLGEFYEALDCLR.I	2	4.33	0.45	
IPI00022429	Alpha-1-acid glycoprotein 1 precursor	K.NWGLSVYADKPETTKEQLGEFYEALDCLR.I	3	6.50	0.49	
IPI00022429	Alpha-1-acid glycoprotein 1 precursor	K.SDVVYTDWK.K	1	2.96	0.30	
IPI00022429	Alpha-1-acid glycoprotein 1 precursor	K.SDVVYTDWK.K	2	3.48	0.38	
IPI00022429	Alpha-1-acid glycoprotein 1 precursor	K.SDVVYTDWKK.D	1	2.79	0.31	
IPI00022429	Alpha-1-acid glycoprotein 1 precursor	K.SDVVYTDWKK.D	2	3.18	0.39	
IPI00022429	Alpha-1-acid glycoprotein 1 precursor	K.SDVVYTDWKK.D	3	2.33	0.20	
IPI00022429	Alpha-1-acid glycoprotein 1 precursor	K.SVQEIQATFFYFTPNKTEDTIFLR.E	3	2.94	0.14	
IPI00022429	Alpha-1-acid glycoprotein 1 precursor	K.TEDTIFLR.E	1	1.64	0.11	
IPI00022429	Alpha-1-acid glycoprotein 1 precursor	K.TYM*LAFDVNDEK.N	2	4.47	0.43	
IPI00022429	Alpha-1-acid glycoprotein 1 precursor	K.TYM*LAFDVNDEKNWGLSVYADKPETTK.E	2	4.53	0.44	
IPI00022429	Alpha-1-acid glycoprotein 1 precursor	K.TYM*LAFDVNDEKNWGLSVYADKPETTK.E	3	5.61	0.47	

IPI00022429	Alpha-1-acid glycoprotein 1 precursor	K.TYMLAFDVNDEK.N	2	3.85	0.37	
IPI00022429	Alpha-1-acid glycoprotein 1 precursor	K.TYMLAFDVNDEKNWGLSVYADKPETTK.E	3	5.32	0.41	
IPI00022429	Alpha-1-acid glycoprotein 1 precursor	K.WFYIASAFR.N	1	2.66	0.25	
IPI00022429	Alpha-1-acid glycoprotein 1 precursor	K.WFYIASAFR.N	2	2.37	0.31	-1.54
IPI00022429	Alpha-1-acid glycoprotein 1 precursor	K.WFYIASAFRNEEYNK.S	2	4.39	0.40	
IPI00022429	Alpha-1-acid glycoprotein 1 precursor	R.IPKSDVVYTDWK.K	2	2.39	0.19	
IPI00022429	Alpha-1-acid glycoprotein 1 precursor	R.IPKSDVVYTDWK.K	3	3.39	0.17	
IPI00022429	Alpha-1-acid glycoprotein 1 precursor	R.IPKSDVVYTDWKK.D	2	3.40	0.31	
IPI00022429	Alpha-1-acid glycoprotein 1 precursor	R.YVGGQEHFAHLLILR.D	2	4.36	0.35	-4.17
IPI00022429	Alpha-1-acid glycoprotein 1 precursor	R.YVGGQEHFAHLLILR.D	3	3.24	0.43	
IPI00022431	Alpha-2-HS-glycoprotein precursor	K.CDSSPDSAEDVRK.V	2	3.29	0.21	
IPI00022431	Alpha-2-HS-glycoprotein precursor	K.CDSSPDSAEDVRK.V	3	3.19	0.32	
IPI00022431	Alpha-2-HS-glycoprotein precursor	K.CNLLAEKQYGFCK.A	2	3.84	0.28	
IPI00022431	Alpha-2-HS-glycoprotein precursor	K.EATEAAKCNLLAEK.Q	2	4.40	0.30	
IPI00022431	Alpha-2-HS-glycoprotein precursor	K.EHAVEGDCDFQLLK.L	1	3.18	0.37	
IPI00022431	Alpha-2-HS-glycoprotein precursor	K.EHAVEGDCDFQLLK.L	2	3.91	0.49	
IPI00022431	Alpha-2-HS-glycoprotein precursor	K.EHAVEGDCDFQLLK.L	3	4.78	0.15	
IPI00022431	Alpha-2-HS-glycoprotein precursor	K.EHAVEGDCDFQLLKLDGK.F	2	5.14	0.49	
IPI00022431	Alpha-2-HS-glycoprotein precursor	K.EHAVEGDCDFQLLKLDGK.F	3	3.33	0.35	
IPI00022431	Alpha-2-HS-glycoprotein precursor	K.HTLNQIDEVK.V	1	2.29	0.22	
IPI00022431	Alpha-2-HS-glycoprotein precursor	K.HTLNQIDEVK.V	2	3.37	0.36	-2.95
IPI00022431	Alpha-2-HS-glycoprotein precursor	K.LDGKFSVVYAK.C	1	2.68	0.27	
IPI00022431	Alpha-2-HS-glycoprotein precursor	K.LDGKFSVVYAK.C	2	3.26	0.31	
IPI00022431	Alpha-2-HS-glycoprotein precursor	K.LDGKFSVVYAK.C	3	2.22	0.27	-3.33
IPI00022431	Alpha-2-HS-glycoprotein precursor	R.AHYDLR.H	1	1.52	0.12	
IPI00022431	Alpha-2-HS-glycoprotein precursor	R.AQLVPLPPSTYVEFTVSGTDCVAK.E	2	4.23	0.36	
IPI00022431	Alpha-2-HS-glycoprotein precursor	R.AQLVPLPPSTYVEFTVSGTDCVAK.E	3	5.43	0.41	
IPI00022431	Alpha-2-HS-glycoprotein precursor	R.HTFM*GVVSLGSPSGEVSHPR.K	2	4.19	0.50	-4.26
IPI00022431	Alpha-2-HS-glycoprotein precursor	R.HTFM*GVVSLGSPSGEVSHPR.K	3	4.55	0.50	-3.15
IPI00022431	Alpha-2-HS-glycoprotein precursor	R.HTFMGVVSLGSPSGEVSHPR.K	2	6.29	0.55	
IPI00022431	Alpha-2-HS-glycoprotein precursor	R.HTFMGVVSLGSPSGEVSHPR.K	3	5.67	0.41	
IPI00022431	Alpha-2-HS-glycoprotein precursor	R.QLKEHAVEGDCDFQLLK.L	2	3.43	0.35	
IPI00022431	Alpha-2-HS-glycoprotein precursor	R.QLKEHAVEGDCDFQLLK.L	3	3.55	0.28	
IPI00022431	Alpha-2-HS-glycoprotein precursor	R.QLKEHAVEGDCDFQLLKLDGK.F	2	3.39	0.28	
IPI00022431	Alpha-2-HS-glycoprotein precursor	R.QPNCDDPETEEAALVAIDYINQNLPWGYK.H	2	3.84	0.32	
IPI00022431	Alpha-2-HS-glycoprotein precursor	R.QPNCDDPETEEAALVAIDYINQNLPWGYK.H	3	5.07	0.40	
IPI00022431	Alpha-2-HS-glycoprotein precursor	R.TVVQPSVGAAAGPVVPPCPGR.I	2	4.79	0.44	-4.36
IPI00022431	Alpha-2-HS-glycoprotein precursor	R.TVVQPSVGAAAGPVVPPCPGR.I	3	5.56	0.47	-4.23
IPI00022431	Alpha-2-HS-glycoprotein precursor	S.APHGPGLIYR.Q	1	2.17	0.34	-4.51
IPI00022431	Alpha-2-HS-glycoprotein precursor	S.APHGPGLIYRQPNCDDPETEEAALVAIDYINQNLPWGYK.H	3	5.78	0.41	
IPI00022432	Transthyretin precursor	A.GPTGTGESKCPLM*VK.V	2	3.90	0.52	-3.04
IPI00022432	Transthyretin precursor	A.LGISPFHEHAEVVFTANDSGPR.R	3	3.78	0.38	-5.70

IPI00022432	Transthyretin precursor	A.LGISPFHEHAEVVFTANDSGPRR.Y	3	3.75	0.41	-5.79
IPI00022432	Transthyretin precursor	G.SPAINVAVHVFR.K	3	3.53	0.34	-3.56
IPI00022432	Transthyretin precursor	G.SPAINVAVHVFRK.A	2	3.00	0.44	-4.06
IPI00022432	Transthyretin precursor	I.NVAVHVFR.K	1	2.12	0.23	-1.87
IPI00022432	Transthyretin precursor	I.SPFHEHAEVVFTANDSGPRR.Y	3	3.53	0.38	-3.79
IPI00022432	Transthyretin precursor	K.AADDTWEPFASGK.T	1	2.72	0.42	-2.54
IPI00022432	Transthyretin precursor	K.AADDTWEPFASGK.T	2	4.67	0.40	-4.24
IPI00022432	Transthyretin precursor	K.ALGISPFHEHAEVVFTANDSGPR.R	2	6.28	0.64	-7.29
IPI00022432	Transthyretin precursor	K.ALGISPFHEHAEVVFTANDSGPR.R	3	6.43	0.51	-8.80
IPI00022432	Transthyretin precursor	K.ALGISPFHEHAEVVFTANDSGPR.R	4	4.57	0.45	-5.79
IPI00022432	Transthyretin precursor	K.ALGISPFHEHAEVVFTANDSGPRR.Y	2	4.14	0.50	-7.01
IPI00022432	Transthyretin precursor	K.ALGISPFHEHAEVVFTANDSGPRR.Y	3	3.77	0.46	-7.71
IPI00022432	Transthyretin precursor	K.ALGISPFHEHAEVVFTANDSGPRR.Y	4	3.12	0.44	-6.57
IPI00022432	Transthyretin precursor	K.ALGISPFHEHAEVVFTANDSGPRR.Y	5	2.79	0.41	-3.32
IPI00022432	Transthyretin precursor	K.TSESGELHGLTTEEEFVEGIYK.V	2	5.39	0.59	-4.06
IPI00022432	Transthyretin precursor	K.TSESGELHGLTTEEEFVEGIYK.V	3	6.55	0.55	-4.91
IPI00022432	Transthyretin precursor	K.TSESGELHGLTTEEEFVEGIYKVEIDTK.S	2	5.07	0.62	-5.36
IPI00022432	Transthyretin precursor	K.TSESGELHGLTTEEEFVEGIYKVEIDTK.S	3	6.80	0.58	-6.45
IPI00022432	Transthyretin precursor	K.TSESGELHGLTTEEEFVEGIYKVEIDTK.S	4	5.82	0.54	-5.79
IPI00022432	Transthyretin precursor	K.TSESGELHGLTTEEEFVEGIYKVEIDTK.S	5	3.13	0.06	-4.21
IPI00022432	Transthyretin precursor	K.VLDAVRGSPAINVAVHVFR.K	2	4.01	0.50	-2.90
IPI00022432	Transthyretin precursor	K.VLDAVRGSPAINVAVHVFR.K	3	6.32	0.53	-4.44
IPI00022432	Transthyretin precursor	K.VLDAVRGSPAINVAVHVFR.K	4	2.65	0.18	-3.13
IPI00022432	Transthyretin precursor	K.VLDAVRGSPAINVAVHVFRK.A	3	3.38	0.32	-4.03
IPI00022432	Transthyretin precursor	K.VLDAVRGSPAINVAVHVFRK.A	5	2.85	0.20	-4.02
IPI00022432	Transthyretin precursor	L.GISPFHEHAEVVFTANDSGPR.R	3	4.01	0.42	-4.76
IPI00022432	Transthyretin precursor	R.GSPAINVAVHVFR.K	1	2.95	0.42	-4.93
IPI00022432	Transthyretin precursor	R.GSPAINVAVHVFR.K	2	4.64	0.54	-8.52
IPI00022432	Transthyretin precursor	R.GSPAINVAVHVFR.K	3	2.19	0.40	-4.77
IPI00022432	Transthyretin precursor	R.GSPAINVAVHVFRK.A	2	4.59	0.51	-5.11
IPI00022432	Transthyretin precursor	R.GSPAINVAVHVFRK.A	3	1.88	0.37	-3.51
IPI00022432	Transthyretin precursor	R.KAADDTWEPFASGK.T	1	3.66	0.33	
IPI00022432	Transthyretin precursor	R.KAADDTWEPFASGK.T	2	4.81	0.58	-3.77
IPI00022432	Transthyretin precursor	R.KAADDTWEPFASGK.T	3	2.75	0.11	-1.73
IPI00022432	Transthyretin precursor	R.RYTIAALLSPYSY.S	2	3.69	0.45	-4.38
IPI00022432	Transthyretin precursor	R.RYTIAALLSPYSYSTTAVVTNPK.E	2	5.70	0.61	-4.58
IPI00022432	Transthyretin precursor	R.RYTIAALLSPYSYSTTAVVTNPK.E	3	4.50	0.49	-4.66
IPI00022432	Transthyretin precursor	R.RYTIAALLSPYSYSTTAVVTNPKE.-	2	6.82	0.61	-6.04
IPI00022432	Transthyretin precursor	R.RYTIAALLSPYSYSTTAVVTNPKE.-	3	5.41	0.53	-6.61
IPI00022432	Transthyretin precursor	R.RYTIAALLSPYSYSTTAVVTNPKE.-	4	4.35	0.40	-4.87
IPI00022432	Transthyretin precursor	R.YTIAALLSPYS.Y	1	2.28	0.24	-2.62
IPI00022432	Transthyretin precursor	R.YTIAALLSPYSYSTTAVVTNPK.E	2	5.32	0.58	-4.46

IPI00022432	Transthyretin precursor	R.YTIAALLSPYSYSTTAVVTNPK.E	3	4.70	0.43	-3.76
IPI00022432	Transthyretin precursor	R.YTIAALLSPYSYSTTAVVTNPKE.-	2	6.85	0.61	-7.96
IPI00022432	Transthyretin precursor	R.YTIAALLSPYSYSTTAVVTNPKE.-	3	5.22	0.42	-6.26
IPI00022432	Transthyretin precursor	S.ESGELHGLTTEEEFVEGIYKVEIDTK.S	3	5.02	0.40	-3.82
IPI00022432	Transthyretin precursor	S.PAINVAVHVFR.K	2	3.76	0.46	-2.67
IPI00022432	Transthyretin precursor	S.PAINVAVHVFR.K	3	4.89	0.50	-4.35
IPI00022432	Transthyretin precursor	S.PAINVAVHVFRK.A	2	3.45	0.44	-3.54
IPI00022432	Transthyretin precursor	S.YSTTAVVTNPK.E	1	2.00	0.21	-4.25
IPI00022432	Transthyretin precursor	T.SESGELHGLTTEEEFVEGIYKVEIDTK.S	3	4.19	0.47	-4.85
IPI00022432	Transthyretin precursor	T.WEPFASGK.T	1	2.02	0.32	-2.86
IPI00022434	Uncharacterized protein ALB	A.DLPSLAADFVESKDVCK.N	1	3.67	0.21	
IPI00022434	Uncharacterized protein ALB	A.DLPSLAADFVESKDVCK.N	2	5.26	0.48	
IPI00022434	Uncharacterized protein ALB	A.FAQYLQQCPFEDHVK.L	2	5.19	0.40	
IPI00022434	Uncharacterized protein ALB	A.KVFDEFKPLVEEPQNLIK.Q	3	6.62	0.37	
IPI00022434	Uncharacterized protein ALB	C.FSALEVDETYVPK.E	2	4.90	0.32	
IPI00022434	Uncharacterized protein ALB	C.FSALEVDETYVPKEFNAETFTFHADICTLSEK.E	3	6.45	0.49	
IPI00022434	Uncharacterized protein ALB	C.FSALEVDETYVPKEFNAETFTFHADICTLSEKER.Q	3	6.06	0.42	
IPI00022434	Uncharacterized protein ALB	C.IAEVENDEM*PADLPSLAADFVESK.D	2	5.54	0.49	
IPI00022434	Uncharacterized protein ALB	C.TVATLRETYGEMADCCAK.Q	2	5.00	0.45	
IPI00022434	Uncharacterized protein ALB	C.VADESAENCDKSLHTLFGDKLCTVATLR.E	3	5.71	0.44	
IPI00022434	Uncharacterized protein ALB	D.LPSLAADFVESKDVCK.N	1	4.13	0.38	
IPI00022434	Uncharacterized protein ALB	D.VFLGM*FLYEYAR.R	2	3.66	0.43	-4.13
IPI00022434	Uncharacterized protein ALB	E.FAEVSKLVTDLTK.V	2	4.99	0.38	
IPI00022434	Uncharacterized protein ALB	E.MPADLPSLAADFVESK.D	1	4.14	0.34	
IPI00022434	Uncharacterized protein ALB	E.PQNLIKQNCELFEQLGEYK.F	2	5.83	0.50	
IPI00022434	Uncharacterized protein ALB	E.PQNLIKQNCELFEQLGEYKFQNALLVR.Y	3	7.04	0.46	
IPI00022434	Uncharacterized protein ALB	E.SAENCDKSLHTLFGDKLCTVATLR.E	3	6.80	0.31	
IPI00022434	Uncharacterized protein ALB	E.TFTFHADICTLSEKER.Q	2	4.95	0.39	
IPI00022434	Uncharacterized protein ALB	E.VENDEM*PADLPSLAADFVESK.D	2	5.73	0.50	
IPI00022434	Uncharacterized protein ALB	F.AQYLQQCPFEDHVKLVNEVTEFAK.T	2	5.08	0.35	
IPI00022434	Uncharacterized protein ALB	H.CIAEVENDEM*PADLPSLAADFVESKDVCK.N	3	5.79	0.36	
IPI00022434	Uncharacterized protein ALB	I.AFAQYLQQCPFEDHVK.L	2	5.05	0.46	
IPI00022434	Uncharacterized protein ALB	K.AACLLPKLDELRDEGK.A	1	3.62	0.15	
IPI00022434	Uncharacterized protein ALB	K.AACLLPKLDELRDEGK.A	2	4.01	0.24	
IPI00022434	Uncharacterized protein ALB	K.AACLLPKLDELRDEGK.A	3	3.95	0.30	
IPI00022434	Uncharacterized protein ALB	K.AACLLPKLDELRDEGKASSAK.Q	2	4.50	0.35	
IPI00022434	Uncharacterized protein ALB	K.AACLLPKLDELRDEGKASSAK.Q	3	4.06	0.27	
IPI00022434	Uncharacterized protein ALB	K.AAFTECCQAADK.A	1	3.41	0.45	
IPI00022434	Uncharacterized protein ALB	K.AAFTECCQAADK.A	2	3.99	0.38	
IPI00022434	Uncharacterized protein ALB	K.AAFTECCQAADKAACLLPK.L	2	5.70	0.50	
IPI00022434	Uncharacterized protein ALB	K.AAFTECCQAADKAACLLPK.L	3	5.42	0.42	
IPI00022434	Uncharacterized protein ALB	K.AAFTECCQAADKAACLLPKLDELR.D	3	5.75	0.39	

IPI00022434	Uncharacterized protein ALB	K.AAFTECCQAADKAACLLPKLDELRDEGK.A	2	4.50	0.37	
IPI00022434	Uncharacterized protein ALB	K.AAFTECCQAADKAACLLPKLDELRDEGK.A	3	5.24	0.37	
IPI00022434	Uncharacterized protein ALB	K.AAFTECCQAADKAACLLPKLDELRDEGKASSAK.Q	3	6.45	0.51	
IPI00022434	Uncharacterized protein ALB	K.AEFAEVSK.L	1	2.70	0.11	
IPI00022434	Uncharacterized protein ALB	K.AEFAEVSK.L	2	2.32	0.06	-2.27
IPI00022434	Uncharacterized protein ALB	K.AEFAEVSKLVTDLTK.V	1	3.71	0.40	
IPI00022434	Uncharacterized protein ALB	K.AEFAEVSKLVTDLTK.V	2	4.59	0.44	-3.50
IPI00022434	Uncharacterized protein ALB	K.AEFAEVSKLVTDLTK.V	3	3.57	0.24	-4.12
IPI00022434	Uncharacterized protein ALB	K.ALVLIAFAQYLQQCPFED.H	2	4.90	0.41	
IPI00022434	Uncharacterized protein ALB	K.ALVLIAFAQYLQQCPFEDHVK.L	2	6.15	0.53	
IPI00022434	Uncharacterized protein ALB	K.ALVLIAFAQYLQQCPFEDHVK.L	3	6.24	0.54	
IPI00022434	Uncharacterized protein ALB	K.ALVLIAFAQYLQQCPFEDHVKLVNEVTEFAK.T	3	6.51	0.50	
IPI00022434	Uncharacterized protein ALB	K.ATKEQLKAVM*DDFAAFVEK.C	2	6.27	0.48	
IPI00022434	Uncharacterized protein ALB	K.ATKEQLKAVM*DDFAAFVEK.C	3	5.05	0.42	
IPI00022434	Uncharacterized protein ALB	K.ATKEQLKAVMDDFAAFVEK.C	2	6.09	0.52	
IPI00022434	Uncharacterized protein ALB	K.ATKEQLKAVMDDFAAFVEK.C	3	4.63	0.36	
IPI00022434	Uncharacterized protein ALB	K.AVM*DDFAAFVEK.C	1	3.46	0.41	
IPI00022434	Uncharacterized protein ALB	K.AVM*DDFAAFVEK.C	2	2.72	0.20	-4.62
IPI00022434	Uncharacterized protein ALB	K.AVM*DDFAAFVEK.C	3	4.27	0.17	
IPI00022434	Uncharacterized protein ALB	K.AVMDDFAAFVEK.C	1	3.38	0.39	
IPI00022434	Uncharacterized protein ALB	K.AVMDDFAAFVEK.C	2	4.72	0.45	
IPI00022434	Uncharacterized protein ALB	K.AVMDDFAAFVEK.C	3	4.80	0.31	
IPI00022434	Uncharacterized protein ALB	K.AVMDDFAAFVEKCCK.A	2	3.36	0.24	
IPI00022434	Uncharacterized protein ALB	K.CASLQKFGER.A	2	3.03	0.18	
IPI00022434	Uncharacterized protein ALB	K.CCAAADPHECYAK.V	1	3.25	0.49	
IPI00022434	Uncharacterized protein ALB	K.CCAAADPHECYAK.V	2	5.15	0.46	
IPI00022434	Uncharacterized protein ALB	K.CCAAADPHECYAKVFDEFKPLVEEPQNLIK.Q	3	5.66	0.38	
IPI00022434	Uncharacterized protein ALB	K.CCTESLVNR.R	1	3.26	0.42	
IPI00022434	Uncharacterized protein ALB	K.CCTESLVNR.R	2	3.78	0.39	
IPI00022434	Uncharacterized protein ALB	K.CCTESLVNRRPCFSALEVDETYVPK.E	3	4.01	0.32	
IPI00022434	Uncharacterized protein ALB	K.DDNPNLPR.L	2	2.71	0.16	
IPI00022434	Uncharacterized protein ALB	K.DLGEENFK.A	1	2.48	0.19	
IPI00022434	Uncharacterized protein ALB	K.DLGEENFK.A	2	2.99	0.11	
IPI00022434	Uncharacterized protein ALB	K.DLGEENFKALVLIAFAQYLQQCPFEDHVK.L	3	5.80	0.53	
IPI00022434	Uncharacterized protein ALB	K.DVCKNYAEAK.D	1	2.80	0.28	
IPI00022434	Uncharacterized protein ALB	K.DVCKNYAEAK.D	2	3.15	0.28	
IPI00022434	Uncharacterized protein ALB	K.DVCKNYAEAK.D	3	2.72	0.23	
IPI00022434	Uncharacterized protein ALB	K.DVCKNYAEAKDVFLGM*FLYEYAR.R	2	5.81	0.52	
IPI00022434	Uncharacterized protein ALB	K.DVCKNYAEAKDVFLGM*FLYEYAR.R	3	6.16	0.51	
IPI00022434	Uncharacterized protein ALB	K.DVCKNYAEAKDVFLGMFLYEYAR.R	2	5.40	0.53	
IPI00022434	Uncharacterized protein ALB	K.DVCKNYAEAKDVFLGMFLYEYAR.R	3	5.99	0.53	
IPI00022434	Uncharacterized protein ALB	K.DVFLGM*FLYEYAR.R	2	3.99	0.47	-5.06

IPI00022434	Uncharacterized protein ALB	K.DVFLGM*FLYEYAR.R	3	5.36	0.36	
IPI00022434	Uncharacterized protein ALB	K.DVFLGMFLYEYAR.R	1	4.45	0.40	
IPI00022434	Uncharacterized protein ALB	K.DVFLGMFLYEYAR.R	2	3.34	0.41	-5.62
IPI00022434	Uncharacterized protein ALB	K.DVFLGMFLYEYAR.R	3	4.99	0.43	
IPI00022434	Uncharacterized protein ALB	K.ECCEKPLLEK.S	1	2.75	0.15	
IPI00022434	Uncharacterized protein ALB	K.ECCEKPLLEK.S	2	2.89	0.28	
IPI00022434	Uncharacterized protein ALB	K.EFNAETFTFHADICTLSEK.E	2	6.58	0.56	
IPI00022434	Uncharacterized protein ALB	K.EFNAETFTFHADICTLSEK.E	3	3.55	0.39	
IPI00022434	Uncharacterized protein ALB	K.EFNAETFTFHADICTLSEKER.Q	2	5.54	0.57	
IPI00022434	Uncharacterized protein ALB	K.EFNAETFTFHADICTLSEKER.Q	3	5.35	0.42	
IPI00022434	Uncharacterized protein ALB	K.EQLKAVM*DDFAAFVEK.C	1	3.76	0.46	
IPI00022434	Uncharacterized protein ALB	K.EQLKAVM*DDFAAFVEK.C	2	5.62	0.47	
IPI00022434	Uncharacterized protein ALB	K.EQLKAVM*DDFAAFVEK.C	3	3.90	0.30	
IPI00022434	Uncharacterized protein ALB	K.EQLKAVMDDFAAFVEK.C	1	5.01	0.49	
IPI00022434	Uncharacterized protein ALB	K.EQLKAVMDDFAAFVEK.C	2	5.14	0.48	
IPI00022434	Uncharacterized protein ALB	K.EQLKAVMDDFAAFVEK.C	3	4.60	0.44	
IPI00022434	Uncharacterized protein ALB	K.FQNALLVR.Y	1	1.76	0.09	-1.15
IPI00022434	Uncharacterized protein ALB	K.FQNALLVR.Y	2	3.03	0.15	-2.03
IPI00022434	Uncharacterized protein ALB	K.HKPKATKEQLK.A	3	2.92	0.14	
IPI00022434	Uncharacterized protein ALB	K.KQTALVELVK.H	1	2.79	0.27	
IPI00022434	Uncharacterized protein ALB	K.KQTALVELVK.H	2	3.26	0.24	
IPI00022434	Uncharacterized protein ALB	K.KVPQVSTPTLVEVSR.N	1	4.15	0.48	
IPI00022434	Uncharacterized protein ALB	K.KVPQVSTPTLVEVSR.N	2	3.59	0.45	-4.27
IPI00022434	Uncharacterized protein ALB	K.KVPQVSTPTLVEVSR.N	3	4.86	0.49	-3.58
IPI00022434	Uncharacterized protein ALB	K.KVPQVSTPTLVEVSRNLGK.V	2	3.37	0.40	
IPI00022434	Uncharacterized protein ALB	K.KVPQVSTPTLVEVSRNLGK.V	3	3.70	0.34	
IPI00022434	Uncharacterized protein ALB	K.KYLYEIAR.R	1	2.61	0.23	
IPI00022434	Uncharacterized protein ALB	K.KYLYEIAR.R	2	3.13	0.29	
IPI00022434	Uncharacterized protein ALB	K.KYLYEIARR.H	2	2.58	0.18	
IPI00022434	Uncharacterized protein ALB	K.LCTVATLR.E	2	1.80	0.09	-1.03
IPI00022434	Uncharacterized protein ALB	K.LCTVATLRETYGEM*ADCCAK.Q	2	3.99	0.43	
IPI00022434	Uncharacterized protein ALB	K.LCTVATLRETYGEM*ADCCAK.Q	3	5.99	0.45	
IPI00022434	Uncharacterized protein ALB	K.LCTVATLRETYGEMADCCAK.Q	2	5.46	0.40	
IPI00022434	Uncharacterized protein ALB	K.LCTVATLRETYGEMADCCAK.Q	3	6.21	0.51	
IPI00022434	Uncharacterized protein ALB	K.LDELRDEGK.A	2	2.16	0.06	-1.65
IPI00022434	Uncharacterized protein ALB	K.LDELRDEGKASSAK.Q	1	2.41	0.10	
IPI00022434	Uncharacterized protein ALB	K.LDELRDEGKASSAK.Q	2	4.30	0.40	
IPI00022434	Uncharacterized protein ALB	K.LDELRDEGKASSAK.Q	3	3.23	0.39	-1.52
IPI00022434	Uncharacterized protein ALB	K.LDELRDEGKASSAKQR.L	3	4.06	0.36	
IPI00022434	Uncharacterized protein ALB	K.LKECCEKPLLEK.S	1	3.16	0.24	
IPI00022434	Uncharacterized protein ALB	K.LKECCEKPLLEK.S	2	4.46	0.29	
IPI00022434	Uncharacterized protein ALB	K.LKECCEKPLLEK.S	3	4.40	0.22	

IPI00022434	Uncharacterized protein ALB	K.LKECCEKPLLEKSHCIAEVENDEM*PADLPSLAADFVESK.D	3	3.46	0.15	
IPI00022434	Uncharacterized protein ALB	K.LKECCEKPLLEKSHCIAEVENDEMPADLPSLAADFVESK.D	3	3.17	0.11	
IPI00022434	Uncharacterized protein ALB	K.LVNEVTEFAK.T	1	2.68	0.32	-4.02
IPI00022434	Uncharacterized protein ALB	K.LVNEVTEFAK.T	2	3.64	0.35	-4.34
IPI00022434	Uncharacterized protein ALB	K.LVNEVTEFAKTCVADESAENCDK.S	3	5.17	0.43	
IPI00022434	Uncharacterized protein ALB	K.LVTDLTK.V	1	2.20	0.11	
IPI00022434	Uncharacterized protein ALB	K.LVTDLTKVHTECCHGDLLECADDR.A	2	4.79	0.46	
IPI00022434	Uncharacterized protein ALB	K.LVTDLTKVHTECCHGDLLECADDR.A	3	7.23	0.53	
IPI00022434	Uncharacterized protein ALB	K.LVTDLTKVHTECCHGDLLECADDRADLAK.Y	2	4.02	0.42	
IPI00022434	Uncharacterized protein ALB	K.LVTDLTKVHTECCHGDLLECADDRADLAK.Y	3	5.08	0.30	
IPI00022434	Uncharacterized protein ALB	K.NYAEAKDVFLGM*FLYEYAR.R	2	5.71	0.54	
IPI00022434	Uncharacterized protein ALB	K.NYAEAKDVFLGM*FLYEYAR.R	3	3.08	0.08	-4.06
IPI00022434	Uncharacterized protein ALB	K.NYAEAKDVFLGMFLYEYAR.R	2	5.95	0.48	
IPI00022434	Uncharacterized protein ALB	K.NYAEAKDVFLGMFLYEYAR.R	3	4.91	0.36	
IPI00022434	Uncharacterized protein ALB	K.QEPERNECFLQHKDDNPNLPR.L	2	4.25	0.36	
IPI00022434	Uncharacterized protein ALB	K.QEPERNECFLQHKDDNPNLPR.L	3	5.72	0.36	
IPI00022434	Uncharacterized protein ALB	K.QNCELFEQLGEYK.F	1	3.85	0.33	
IPI00022434	Uncharacterized protein ALB	K.QNCELFEQLGEYK.F	2	3.52	0.44	-2.05
IPI00022434	Uncharacterized protein ALB	K.QNCELFEQLGEYK.F	3	4.54	0.25	
IPI00022434	Uncharacterized protein ALB	K.QNCELFEQLGEYKFQNALLVR.Y	2	5.42	0.38	
IPI00022434	Uncharacterized protein ALB	K.QNCELFEQLGEYKFQNALLVR.Y	3	6.95	0.48	
IPI00022434	Uncharacterized protein ALB	K.QTALVELVK.H	1	1.96	0.13	
IPI00022434	Uncharacterized protein ALB	K.QTALVELVK.H	2	1.80	0.10	-1.82
IPI00022434	Uncharacterized protein ALB	K.RM*PCAEDYLSVVLNQLCVLHEK.T	2	4.36	0.39	
IPI00022434	Uncharacterized protein ALB	K.RM*PCAEDYLSVVLNQLCVLHEK.T	3	7.25	0.47	
IPI00022434	Uncharacterized protein ALB	K.RM*PCAEDYLSVVLNQLCVLHEKTPVSDR.V	3	4.69	0.39	
IPI00022434	Uncharacterized protein ALB	K.RM*PCAEDYLSVVLNQLCVLHEKTPVSDRVTK.C	3	4.04	0.28	
IPI00022434	Uncharacterized protein ALB	K.RMPCAEDYLSVVLNQLCVLHEK.T	2	5.25	0.45	
IPI00022434	Uncharacterized protein ALB	K.RMPCAEDYLSVVLNQLCVLHEK.T	3	6.52	0.47	
IPI00022434	Uncharacterized protein ALB	K.RMPCAEDYLSVVLNQLCVLHEKTPVSDR.V	3	5.15	0.32	
IPI00022434	Uncharacterized protein ALB	K.SHCIAEVENDEM*PAD.L	2	5.40	0.43	
IPI00022434	Uncharacterized protein ALB	K.SHCIAEVENDEM*PADLPSLAADFVESK.D	2	6.10	0.55	
IPI00022434	Uncharacterized protein ALB	K.SHCIAEVENDEM*PADLPSLAADFVESK.D	3	6.12	0.57	-4.57
IPI00022434	Uncharacterized protein ALB	K.SHCIAEVENDEM*PADLPSLAADFVESKD.V	3	5.94	0.29	
IPI00022434	Uncharacterized protein ALB	K.SHCIAEVENDEM*PADLPSLAADFVESKDVCK.N	2	5.61	0.60	
IPI00022434	Uncharacterized protein ALB	K.SHCIAEVENDEM*PADLPSLAADFVESKDVCK.N	3	7.63	0.56	
IPI00022434	Uncharacterized protein ALB	K.SHCIAEVENDEM*PADLPSLAADFVESKDVCKNYAEAK.D	3	6.65	0.57	
IPI00022434	Uncharacterized protein ALB	K.SHCIAEVENDEMPADLPSLAADFVESK.D	2	6.14	0.54	
IPI00022434	Uncharacterized protein ALB	K.SHCIAEVENDEMPADLPSLAADFVESK.D	3	6.62	0.53	
IPI00022434	Uncharacterized protein ALB	K.SHCIAEVENDEMPADLPSLAADFVESKDVCK.N	2	5.73	0.58	
IPI00022434	Uncharacterized protein ALB	K.SHCIAEVENDEMPADLPSLAADFVESKDVCK.N	3	7.27	0.55	
IPI00022434	Uncharacterized protein ALB	K.SHCIAEVENDEMPADLPSLAADFVESKDVCKNYAEAK.D	3	7.14	0.51	

IPI00022434	Uncharacterized protein ALB	K.SLHTLFGDK.L	1	2.93	0.27	
IPI00022434	Uncharacterized protein ALB	K.SLHTLFGDK.L	2	1.81	0.10	-1.35
IPI00022434	Uncharacterized protein ALB	K.SLHTLFGDKLCTVATLR.E	2	5.67	0.52	
IPI00022434	Uncharacterized protein ALB	K.SLHTLFGDKLCTVATLR.E	3	5.10	0.51	
IPI00022434	Uncharacterized protein ALB	K.SLHTLFGDKLCTVATLRETYGEM*ADCCAK.Q	3	5.26	0.46	
IPI00022434	Uncharacterized protein ALB	K.SLHTLFGDKLCTVATLRETYGEMADCCAK.Q	3	5.27	0.43	
IPI00022434	Uncharacterized protein ALB	K.TCVADESAENCDK.S	1	3.35	0.39	
IPI00022434	Uncharacterized protein ALB	K.TCVADESAENCDK.S	2	4.99	0.38	
IPI00022434	Uncharacterized protein ALB	K.TCVADESAENCDKSLH.T	2	4.98	0.42	
IPI00022434	Uncharacterized protein ALB	K.TCVADESAENCDKSLHTLFGDK.L	2	5.16	0.49	
IPI00022434	Uncharacterized protein ALB	K.TCVADESAENCDKSLHTLFGDK.L	3	5.24	0.45	
IPI00022434	Uncharacterized protein ALB	K.TCVADESAENCDKSLHTLFGDKLCTVATLR.E	2	4.74	0.50	
IPI00022434	Uncharacterized protein ALB	K.TCVADESAENCDKSLHTLFGDKLCTVATLR.E	3	8.62	0.58	
IPI00022434	Uncharacterized protein ALB	K.TCVADESAENCDKSLHTLFGDKLCTVATLRETYGEM*ADCCAK.Q	3	5.06	0.41	
IPI00022434	Uncharacterized protein ALB	K.TCVADESAENCDKSLHTLFGDKLCTVATLRETYGEMADCCAK.Q	3	5.62	0.28	
IPI00022434	Uncharacterized protein ALB	K.TPVSDRVTK.C	2	2.16	0.06	-2.14
IPI00022434	Uncharacterized protein ALB	K.TPVSDRVTKCCTESLVNR.R	3	4.31	0.40	
IPI00022434	Uncharacterized protein ALB	K.TYETTLEK.C	1	1.93	0.10	
IPI00022434	Uncharacterized protein ALB	K.TYETTLEK.C	2	2.62	0.23	
IPI00022434	Uncharacterized protein ALB	K.TYETTLEKCCAAADPHECYAK.V	2	5.25	0.50	
IPI00022434	Uncharacterized protein ALB	K.TYETTLEKCCAAADPHECYAK.V	3	5.13	0.32	
IPI00022434	Uncharacterized protein ALB	K.VFDEFKPLVEEPQNLIK.Q	2	4.55	0.39	-5.33
IPI00022434	Uncharacterized protein ALB	K.VFDEFKPLVEEPQNLIK.Q	3	4.84	0.32	-4.89
IPI00022434	Uncharacterized protein ALB	K.VFDEFKPLVEEPQNLIKQNCELFEQLGEYK.F	3	7.59	0.48	
IPI00022434	Uncharacterized protein ALB	K.VFDEFKPLVEEPQNLIKQNCELFEQLGEYKFQNALLVR.Y	3	6.11	0.54	
IPI00022434	Uncharacterized protein ALB	K.VHTECCHGDLLECADDR.A	2	6.67	0.60	
IPI00022434	Uncharacterized protein ALB	K.VHTECCHGDLLECADDR.A	3	5.51	0.45	
IPI00022434	Uncharacterized protein ALB	K.VHTECCHGDLLECADDRADLAK.Y	2	5.13	0.43	
IPI00022434	Uncharacterized protein ALB	K.VHTECCHGDLLECADDRADLAK.Y	3	6.44	0.50	
IPI00022434	Uncharacterized protein ALB	K.VHTECCHGDLLECADDRADLAKYICENQDSISSK.L	3	4.92	0.43	
IPI00022434	Uncharacterized protein ALB	K.VPQVSTPTLVEVSR.N	1	3.38	0.40	
IPI00022434	Uncharacterized protein ALB	K.VPQVSTPTLVEVSR.N	2	3.92	0.45	
IPI00022434	Uncharacterized protein ALB	K.VPQVSTPTLVEVSR.N	3	3.70	0.29	
IPI00022434	Uncharacterized protein ALB	K.VPQVSTPTLVEVSRNLGK.V	3	2.66	0.20	
IPI00022434	Uncharacterized protein ALB	K.YICENQDSISSK.L	1	3.56	0.39	
IPI00022434	Uncharacterized protein ALB	K.YICENQDSISSK.L	2	1.96	0.16	-2.00
IPI00022434	Uncharacterized protein ALB	K.YICENQDSISSK.L	3	2.98	0.10	
IPI00022434	Uncharacterized protein ALB	K.YICENQDSISSKLK.E	2	4.73	0.43	
IPI00022434	Uncharacterized protein ALB	K.YICENQDSISSKLKECCEKPLLEK.S	3	5.80	0.27	
IPI00022434	Uncharacterized protein ALB	K.YLYEIAR.R	1	2.31	0.18	
IPI00022434	Uncharacterized protein ALB	K.YLYEIAR.R	2	3.11	0.26	
IPI00022434	Uncharacterized protein ALB	L.FEQLGEYKFQNALLVR.Y	2	5.33	0.32	

IPI00022434	Uncharacterized protein ALB	L.IAFAQYLQQCPFEDHVK.L	2	5.51	0.48	
IPI00022434	Uncharacterized protein ALB	L.IKQNCELFEQLGEYK.F	2	4.99	0.28	
IPI00022434	Uncharacterized protein ALB	L.PSLAADFVESKDVCK.N	1	4.05	0.37	
IPI00022434	Uncharacterized protein ALB	L.PSLAADFVESKDVCK.N	2	5.36	0.39	
IPI00022434	Uncharacterized protein ALB	L.VLIAFAQYLQQCPFEDHVK.L	2	5.55	0.45	
IPI00022434	Uncharacterized protein ALB	M.PADLPSLAADFVESK.D	1	4.03	0.52	
IPI00022434	Uncharacterized protein ALB	M.PADLPSLAADFVESK.D	2	5.47	0.47	
IPI00022434	Uncharacterized protein ALB	M.PADLPSLAADFVESKDVCK.N	2	6.02	0.51	
IPI00022434	Uncharacterized protein ALB	M.PCAEDYLSVVLNQLCVLHEK.T	3	6.35	0.43	
IPI00022434	Uncharacterized protein ALB	N.CDKSLHTLFGDKLCTVATLR.E	2	5.54	0.52	
IPI00022434	Uncharacterized protein ALB	P.CAEDYLSVVLNQLCVLHEK.T	2	5.18	0.36	
IPI00022434	Uncharacterized protein ALB	P.CAEDYLSVVLNQLCVLHEK.T	3	6.03	0.45	
IPI00022434	Uncharacterized protein ALB	P.CFSALEVDETYVPK.E	2	5.56	0.47	
IPI00022434	Uncharacterized protein ALB	R.ADLAKYICENQDSISSK.L	2	5.03	0.43	
IPI00022434	Uncharacterized protein ALB	R.AFKAWAVAR.L	1	2.21	0.23	
IPI00022434	Uncharacterized protein ALB	R.AFKAWAVAR.L	2	3.16	0.26	
IPI00022434	Uncharacterized protein ALB	R.DAHKSEVAHR.F	2	2.69	0.26	
IPI00022434	Uncharacterized protein ALB	R.DEGKASSAK.Q	1	2.24	0.15	
IPI00022434	Uncharacterized protein ALB	R.ETYGEM*ADCCAK.Q	1	2.84	0.34	
IPI00022434	Uncharacterized protein ALB	R.ETYGEM*ADCCAK.Q	2	3.10	0.34	-3.76
IPI00022434	Uncharacterized protein ALB	R.ETYGEM*ADCCAKQEPER.N	2	3.90	0.27	
IPI00022434	Uncharacterized protein ALB	R.ETYGEM*ADCCAKQEPERNECFLQHK.D	3	4.59	0.38	
IPI00022434	Uncharacterized protein ALB	R.ETYGEM*ADCCAKQEPERNECFLQHKDDNPNLPR.L	3	4.03	0.39	
IPI00022434	Uncharacterized protein ALB	R.ETYGEMADCCAK.Q	1	3.26	0.45	
IPI00022434	Uncharacterized protein ALB	R.ETYGEMADCCAK.Q	2	4.18	0.47	
IPI00022434	Uncharacterized protein ALB	R.ETYGEMADCCAKQEPER.N	2	3.97	0.34	
IPI00022434	Uncharacterized protein ALB	R.ETYGEMADCCAKQEPERNECFLQHKDDNPNLPR.L	3	3.83	0.29	
IPI00022434	Uncharacterized protein ALB	R.FKDLGEENFK.A	1	3.27	0.31	
IPI00022434	Uncharacterized protein ALB	R.FKDLGEENFK.A	2	3.67	0.24	
IPI00022434	Uncharacterized protein ALB	R.FKDLGEENFK.A	3	2.78	0.18	-3.67
IPI00022434	Uncharacterized protein ALB	R.FKDLGEENFKALVLIAFAQYLQQCPFEDHVK.L	3	7.57	0.58	
IPI00022434	Uncharacterized protein ALB	R.FPKAEFAEVSK.L	1	3.28	0.29	
IPI00022434	Uncharacterized protein ALB	R.FPKAEFAEVSK.L	2	3.86	0.31	
IPI00022434	Uncharacterized protein ALB	R.FPKAEFAEVSK.L	3	4.48	0.30	
IPI00022434	Uncharacterized protein ALB	R.FPKAEFAEVSKLVTDLTK.V	2	6.07	0.48	
IPI00022434	Uncharacterized protein ALB	R.FPKAEFAEVSKLVTDLTK.V	3	5.52	0.39	
IPI00022434	Uncharacterized protein ALB	R.HPDYSVVLLLR.L	1	3.42	0.37	
IPI00022434	Uncharacterized protein ALB	R.HPDYSVVLLLR.L	2	2.94	0.36	
IPI00022434	Uncharacterized protein ALB	R.HPDYSVVLLLR.L	3	4.45	0.12	
IPI00022434	Uncharacterized protein ALB	R.HPYFYAPELLFFAK.R	1	4.65	0.47	
IPI00022434	Uncharacterized protein ALB	R.HPYFYAPELLFFAK.R	2	3.11	0.35	
IPI00022434	Uncharacterized protein ALB	R.HPYFYAPELLFFAK.R	3	3.33	0.28	-2.89

IPI00022434	Uncharacterized protein ALB	R.HPYFYAPELLFFAKR.Y	3	4.41	0.36	
IPI00022434	Uncharacterized protein ALB	R.LAKTYETTLEK.C	1	2.80	0.23	
IPI00022434	Uncharacterized protein ALB	R.LAKTYETTLEK.C	2	3.39	0.31	
IPI00022434	Uncharacterized protein ALB	R.LAKTYETTLEK.C	3	2.39	0.11	1.05
IPI00022434	Uncharacterized protein ALB	R.LAKTYETTLEKCCAAADPHECYAK.V	3	5.45	0.35	
IPI00022434	Uncharacterized protein ALB	R.LITSHLK.A	2	2.28	0.11	
IPI00022434	Uncharacterized protein ALB	R.LSQRFPKAEFAEVSK.L	2	3.95	0.32	
IPI00022434	Uncharacterized protein ALB	R.LSQRFPKAEFAEVSK.L	3	5.85	0.39	
IPI00022434	Uncharacterized protein ALB	R.LSQRFPKAEFAEVSKLVTDLTK.V	3	7.14	0.47	
IPI00022434	Uncharacterized protein ALB	R.LVRPEVDVM*CTAFHDNEETFLK.K	2	4.61	0.42	
IPI00022434	Uncharacterized protein ALB	R.LVRPEVDVM*CTAFHDNEETFLK.K	3	5.81	0.46	
IPI00022434	Uncharacterized protein ALB	R.LVRPEVDVM*CTAFHDNEETFLKK.Y	2	3.75	0.39	
IPI00022434	Uncharacterized protein ALB	R.LVRPEVDVM*CTAFHDNEETFLKK.Y	3	6.57	0.48	
IPI00022434	Uncharacterized protein ALB	R.LVRPEVDVM*CTAFHDNEETFLKKYLYEIAR.R	3	6.13	0.40	
IPI00022434	Uncharacterized protein ALB	R.LVRPEVDVMCTAFHDNEETFLK.K	2	4.67	0.46	
IPI00022434	Uncharacterized protein ALB	R.LVRPEVDVMCTAFHDNEETFLK.K	3	6.49	0.47	
IPI00022434	Uncharacterized protein ALB	R.LVRPEVDVMCTAFHDNEETFLKK.Y	2	4.32	0.39	
IPI00022434	Uncharacterized protein ALB	R.LVRPEVDVMCTAFHDNEETFLKK.Y	3	7.14	0.47	
IPI00022434	Uncharacterized protein ALB	R.M*PCAEDYLSVVLNQLCVLHEK.T	2	5.36	0.46	
IPI00022434	Uncharacterized protein ALB	R.M*PCAEDYLSVVLNQLCVLHEK.T	3	4.25	0.37	-4.98
IPI00022434	Uncharacterized protein ALB	R.M*PCAEDYLSVVLNQLCVLHEKTPVSDR.V	3	5.69	0.42	
IPI00022434	Uncharacterized protein ALB	R.M*PCAEDYLSVVLNQLCVLHEKTPVSDRVTK.C	3	5.84	0.48	
IPI00022434	Uncharacterized protein ALB	R.MPCAEDYLSVVLNQLCVLHEK.T	2	5.15	0.43	
IPI00022434	Uncharacterized protein ALB	R.MPCAEDYLSVVLNQLCVLHEK.T	3	4.21	0.43	-4.61
IPI00022434	Uncharacterized protein ALB	R.MPCAEDYLSVVLNQLCVLHEKTPVSDR.V	3	6.18	0.50	
IPI00022434	Uncharacterized protein ALB	R.MPCAEDYLSVVLNQLCVLHEKTPVSDRVTK.C	3	5.28	0.39	
IPI00022434	Uncharacterized protein ALB	R.NECFLQHK.D	2	2.47	0.14	
IPI00022434	Uncharacterized protein ALB	R.NECFLQHKDDNPNLPR.L	1	3.59	0.32	
IPI00022434	Uncharacterized protein ALB	R.NECFLQHKDDNPNLPR.L	2	4.52	0.36	
IPI00022434	Uncharacterized protein ALB	R.NECFLQHKDDNPNLPR.L	3	4.57	0.35	
IPI00022434	Uncharacterized protein ALB	R.PCFSALEVDETYVPK.E	2	5.95	0.49	
IPI00022434	Uncharacterized protein ALB	R.QIKKQTALVELVK.H	3	2.90	0.25	
IPI00022434	Uncharacterized protein ALB	R.RHPDYSVVLLLR.L	2	3.78	0.40	
IPI00022434	Uncharacterized protein ALB	R.RHPDYSVVLLLR.L	3	5.46	0.33	
IPI00022434	Uncharacterized protein ALB	R.RHPYFYAPELLFFAK.R	2	4.81	0.40	
IPI00022434	Uncharacterized protein ALB	R.RHPYFYAPELLFFAK.R	3	4.26	0.32	-4.29
IPI00022434	Uncharacterized protein ALB	R.RHPYFYAPELLFFAKR.Y	2	3.80	0.38	
IPI00022434	Uncharacterized protein ALB	R.RPCFSALEVDETYVPK.E	1	3.97	0.49	
IPI00022434	Uncharacterized protein ALB	R.RPCFSALEVDETYVPK.E	2	4.52	0.40	
IPI00022434	Uncharacterized protein ALB	R.RPCFSALEVDETYVPK.E	3	3.79	0.26	
IPI00022434	Uncharacterized protein ALB	R.RPCFSALEVDETYVPKEFNAETFTFHADICTLSEK.E	3	6.83	0.48	
IPI00022434	Uncharacterized protein ALB	R.RPCFSALEVDETYVPKEFNAETFTFHADICTLSEKER.Q	3	6.05	0.47	

IPI00022434	Uncharacterized protein ALB	R.VTKCCTESLVNR.R	3	3.44	0.17	
IPI00022434	Uncharacterized protein ALB	R.YKAAFTECCQAADK.A	1	4.06	0.49	
IPI00022434	Uncharacterized protein ALB	R.YKAAFTECCQAADK.A	2	5.28	0.45	
IPI00022434	Uncharacterized protein ALB	R.YKAAFTECCQAADK.A	3	4.94	0.39	
IPI00022434	Uncharacterized protein ALB	R.YKAAFTECCQAADKAACLLPK.L	2	5.51	0.53	
IPI00022434	Uncharacterized protein ALB	R.YKAAFTECCQAADKAACLLPK.L	3	5.35	0.47	
IPI00022434	Uncharacterized protein ALB	R.YKAAFTECCQAADKAACLLPKLDELRDEGK.A	3	6.90	0.49	
IPI00022434	Uncharacterized protein ALB	R.YKAAFTECCQAADKAACLLPKLDELRDEGKASSAK.Q	3	6.75	0.45	
IPI00022434	Uncharacterized protein ALB	R.YTKKVPQVSTPTLVEVSR.N	2	4.95	0.33	
IPI00022434	Uncharacterized protein ALB	R.YTKKVPQVSTPTLVEVSR.N	3	4.84	0.39	
IPI00022434	Uncharacterized protein ALB	S.AENCDKSLHTLFGDKLCTVATLR.E	3	5.73	0.47	
IPI00022434	Uncharacterized protein ALB	V.ADESAENCDKSLHTLFGDKLCTVATLR.E	3	6.13	0.42	
IPI00022434	Uncharacterized protein ALB	V.FDEFKPLVEEPQNLIKQNCELFEQLGEYK.F	3	5.61	0.34	
IPI00022434	Uncharacterized protein ALB	V.LIAFAQYLQQCPFEDHVK.L	2	5.68	0.46	
IPI00022434	Uncharacterized protein ALB	V.MCTAFHDNEETFLKK.Y	2	5.06	0.34	
IPI00022434	Uncharacterized protein ALB	V.PKEFNAETFTFHADICTLSEK.E	2	5.73	0.47	
IPI00022445	Platelet basic protein precursor	K.GKEESLDSDLYAELR.C	2	5.13	0.46	-3.76
IPI00022445	Platelet basic protein precursor	K.GKEESLDSDLYAELR.C	3	3.68	0.25	-2.68
IPI00022445	Platelet basic protein precursor	K.NIQSLEVIGK.G	2	2.88	0.16	-2.40
IPI00022462	Transferrin receptor protein 1	K.VSASPLLYTLIEK.T	2	2.82	0.29	
IPI00022463	Serotransferrin precursor	C.GCSTLNQYFGYSGAFK.C	2	4.92	0.36	
IPI00022463	Serotransferrin precursor	C.PGCGCSTLNQYFGYSGAFK.C	2	5.87	0.52	
IPI00022463	Serotransferrin precursor	I.PIGLLYCDLPEPR.K	2	4.97	0.37	
IPI00022463	Serotransferrin precursor	K.ADRDQYELLCLDNTR.K	1	2.47	0.30	
IPI00022463	Serotransferrin precursor	K.ADRDQYELLCLDNTR.K	2	4.69	0.38	
IPI00022463	Serotransferrin precursor	K.ADRDQYELLCLDNTR.K	3	4.35	0.14	
IPI00022463	Serotransferrin precursor	K.ADRDQYELLCLDNTRKPVDEYK.D	2	3.42	0.05	
IPI00022463	Serotransferrin precursor	K.ADRDQYELLCLDNTRKPVDEYK.D	3	4.63	0.31	
IPI00022463	Serotransferrin precursor	K.ASYLDCIR.A	1	2.22	0.20	
IPI00022463	Serotransferrin precursor	K.ASYLDCIR.A	2	2.90	0.21	
IPI00022463	Serotransferrin precursor	K.AVANFFSGSCAPCADGTDFPQLCQLCPGCGCSTLNQYFGYSGAFK.C	3	5.36	0.47	
IPI00022463	Serotransferrin precursor	K.CDEWSVNSVGK.I	2	3.09	0.18	
IPI00022463	Serotransferrin precursor	K.CDEWSVNSVGKIECVSAETTEDCIAK.I	3	4.79	0.41	
IPI00022463	Serotransferrin precursor	K.CGLVPVLAENYNKSDNCEDTPEAGYFAVAVVK.K	3	5.48	0.43	
IPI00022463	Serotransferrin precursor	K.CGLVPVLAENYNKSDNCEDTPEAGYFAVAVVKK.S	3	4.90	0.46	
IPI00022463	Serotransferrin precursor	K.CLKDGAGDVAFVK.H	1	3.55	0.38	
IPI00022463	Serotransferrin precursor	K.CLKDGAGDVAFVK.H	2	4.53	0.42	
IPI00022463	Serotransferrin precursor	K.CLKDGAGDVAFVK.H	3	5.11	0.36	
IPI00022463	Serotransferrin precursor	K.CSTSSLLEACTFR.R	1	3.44	0.39	
IPI00022463	Serotransferrin precursor	K.CSTSSLLEACTFR.R	2	5.47	0.44	
IPI00022463	Serotransferrin precursor	K.CSTSSLLEACTFR.R	3	3.48	0.17	
IPI00022463	Serotransferrin precursor	K.DCHLAQVPSHTVVAR.S	2	4.42	0.31	

IPI00022463	Serotransferrin precursor	K.DCHLAQVPSHTVVAR.S	3	3.94	0.35	
IPI00022463	Serotransferrin precursor	K.DGAGDVAFVK.H	1	2.34	0.13	
IPI00022463	Serotransferrin precursor	K.DGAGDVAFVK.H	2	3.46	0.26	-3.05
IPI00022463	Serotransferrin precursor	K.DKSKEFQLFSSPHGK.D	2	2.51	0.28	
IPI00022463	Serotransferrin precursor	K.DKSKEFQLFSSPHGKDLLFK.D	2	6.23	0.42	
IPI00022463	Serotransferrin precursor	K.DKSKEFQLFSSPHGKDLLFK.D	3	5.24	0.40	
IPI00022463	Serotransferrin precursor	K.DLLFKDSAHGFLK.V	1	3.62	0.41	
IPI00022463	Serotransferrin precursor	K.DLLFKDSAHGFLK.V	2	4.38	0.40	
IPI00022463	Serotransferrin precursor	K.DLLFKDSAHGFLK.V	3	3.49	0.26	
IPI00022463	Serotransferrin precursor	K.DLLFKDSAHGFLKVPPR.M	2	5.13	0.48	
IPI00022463	Serotransferrin precursor	K.DLLFKDSAHGFLKVPPR.M	3	5.34	0.41	
IPI00022463	Serotransferrin precursor	K.DLLFKDSAHGFLKVPPRM*DAK.M	3	4.19	0.13	
IPI00022463	Serotransferrin precursor	K.DLLFRDDTVCLAK.L	1	3.27	0.33	
IPI00022463	Serotransferrin precursor	K.DLLFRDDTVCLAK.L	2	3.85	0.32	
IPI00022463	Serotransferrin precursor	K.DLLFRDDTVCLAK.L	3	3.82	0.32	
IPI00022463	Serotransferrin precursor	K.DSAHGFLK.V	1	2.44	0.24	
IPI00022463	Serotransferrin precursor	K.DSAHGFLK.V	2	2.40	0.15	
IPI00022463	Serotransferrin precursor	K.DSAHGFLKVPPR.M	1	3.52	0.21	
IPI00022463	Serotransferrin precursor	K.DSAHGFLKVPPR.M	2	3.23	0.23	
IPI00022463	Serotransferrin precursor	K.DSAHGFLKVPPR.M	3	4.65	0.18	
IPI00022463	Serotransferrin precursor	K.DSAHGFLKVPPRM*DAK.M	2	3.69	0.34	
IPI00022463	Serotransferrin precursor	K.DSAHGFLKVPPRM*DAK.M	3	2.58	0.18	
IPI00022463	Serotransferrin precursor	K.DSGFQM*NQLR.G	2	2.65	0.22	-2.60
IPI00022463	Serotransferrin precursor	K.DSGFQMNQLR.G	1	2.50	0.17	
IPI00022463	Serotransferrin precursor	K.DSGFQMNQLR.G	2	3.91	0.24	
IPI00022463	Serotransferrin precursor	K.DSSLCKLCM*GSGLNLCEPNNK.E	3	4.68	0.27	
IPI00022463	Serotransferrin precursor	K.DSSLCKLCM*GSGLNLCEPNNKEGYYGYTGAFR.C	3	5.02	0.40	
IPI00022463	Serotransferrin precursor	K.DSSLCKLCMGSGLNLCEPNNKEGYYGYTGAFR.C	3	3.70	0.21	
IPI00022463	Serotransferrin precursor	K.DYELLCLDGTR.K	1	2.92	0.22	
IPI00022463	Serotransferrin precursor	K.DYELLCLDGTR.K	2	4.14	0.40	
IPI00022463	Serotransferrin precursor	K.DYELLCLDGTRKPVEEYANCHLAR.A	3	5.46	0.41	
IPI00022463	Serotransferrin precursor	K.EDLIWELLNQAQEHFGK.D	2	4.72	0.33	
IPI00022463	Serotransferrin precursor	K.EDLIWELLNQAQEHFGK.D	3	2.14	0.21	
IPI00022463	Serotransferrin precursor	K.EDLIWELLNQAQEHFGKDK.S	2	4.78	0.43	
IPI00022463	Serotransferrin precursor	K.EDLIWELLNQAQEHFGKDK.S	3	2.30	0.17	
IPI00022463	Serotransferrin precursor	K.EDPQTFYYAVAVVK.K	1	3.73	0.16	
IPI00022463	Serotransferrin precursor	K.EDPQTFYYAVAVVK.K	2	4.48	0.45	
IPI00022463	Serotransferrin precursor	K.EDPQTFYYAVAVVK.K	3	5.24	0.33	
IPI00022463	Serotransferrin precursor	K.EDPQTFYYAVAVVKK.D	1	3.33	0.15	
IPI00022463	Serotransferrin precursor	K.EDPQTFYYAVAVVKK.D	2	4.43	0.42	
IPI00022463	Serotransferrin precursor	K.EFQLFSSPHGK.D	1	3.05	0.29	
IPI00022463	Serotransferrin precursor	K.EFQLFSSPHGK.D	2	3.63	0.30	

IPI00022463	Serotransferrin precursor	K.EFQLFSSPHGKDLLFK.D	2	5.05	0.33	
IPI00022463	Serotransferrin precursor	K.EFQLFSSPHGKDLLFKDSAHGFLK.V	2	5.13	0.28	
IPI00022463	Serotransferrin precursor	K.EFQLFSSPHGKDLLFKDSAHGFLK.V	3	6.53	0.38	
IPI00022463	Serotransferrin precursor	K.EGYYGYTGAFR.C	1	2.71	0.37	
IPI00022463	Serotransferrin precursor	K.EGYYGYTGAFR.C	2	3.44	0.40	
IPI00022463	Serotransferrin precursor	K.GDVAFVKHQTVPQNTGGK.N	2	4.66	0.33	
IPI00022463	Serotransferrin precursor	K.GDVAFVKHQTVPQNTGGK.N	3	4.60	0.30	
IPI00022463	Serotransferrin precursor	K.GDVAFVKHQTVPQNTGGKNPDPWAK.N	3	3.41	0.17	
IPI00022463	Serotransferrin precursor	K.HQTVPQNTGGK.N	2	2.73	0.18	
IPI00022463	Serotransferrin precursor	K.HSTIFENLANK.A	1	3.58	0.34	
IPI00022463	Serotransferrin precursor	K.HSTIFENLANK.A	2	3.99	0.39	
IPI00022463	Serotransferrin precursor	K.HSTIFENLANK.A	3	4.47	0.21	
IPI00022463	Serotransferrin precursor	K.HSTIFENLANKADR.D	2	4.56	0.45	
IPI00022463	Serotransferrin precursor	K.HSTIFENLANKADR.D	3	4.65	0.42	
IPI00022463	Serotransferrin precursor	K.HSTIFENLANKADRDQYELLCLDNTR.K	2	3.87	0.38	
IPI00022463	Serotransferrin precursor	K.HSTIFENLANKADRDQYELLCLDNTR.K	3	4.88	0.43	
IPI00022463	Serotransferrin precursor	K.HSTIFENLANKADRDQYELLCLDNTRKPVDEYK.D	3	4.62	0.35	
IPI00022463	Serotransferrin precursor	K.IECVSAETTEDCIAK.I	1	3.64	0.39	
IPI00022463	Serotransferrin precursor	K.IECVSAETTEDCIAK.I	2	5.60	0.44	
IPI00022463	Serotransferrin precursor	K.IECVSAETTEDCIAK.I	3	3.28	0.22	
IPI00022463	Serotransferrin precursor	K.IM*NGEADAM*SLDGGFVYIAGK.C	2	6.64	0.54	
IPI00022463	Serotransferrin precursor	K.IM*NGEADAM*SLDGGFVYIAGK.C	3	6.66	0.46	
IPI00022463	Serotransferrin precursor	K.IM*NGEADAMSLDGGFVYIAGK.C	2	6.50	0.40	
IPI00022463	Serotransferrin precursor	K.IM*NGEADAMSLDGGFVYIAGK.C	3	6.92	0.21	
IPI00022463	Serotransferrin precursor	K.IMNGEADAM*SLDGGFVYIAGK.C	2	5.87	0.30	
IPI00022463	Serotransferrin precursor	K.IMNGEADAMSLDGGFVYIAGK.C	2	5.46	0.48	
IPI00022463	Serotransferrin precursor	K.IMNGEADAMSLDGGFVYIAGK.C	3	3.99	0.12	
IPI00022463	Serotransferrin precursor	K.INHCRFDEFFSEGCAPGSK.K	2	4.50	0.34	
IPI00022463	Serotransferrin precursor	K.INHCRFDEFFSEGCAPGSK.K	3	5.17	0.35	
IPI00022463	Serotransferrin precursor	K.KASYLDCIR.A	1	2.15	0.24	
IPI00022463	Serotransferrin precursor	K.KASYLDCIR.A	2	3.09	0.17	
IPI00022463	Serotransferrin precursor	K.KDSGFQM*NQLR.G	2	3.50	0.34	
IPI00022463	Serotransferrin precursor	K.KDSGFQM*NQLR.G	3	3.68	0.11	
IPI00022463	Serotransferrin precursor	K.KSASDLTWDNLK.G	1	2.83	0.21	
IPI00022463	Serotransferrin precursor	K.KSASDLTWDNLK.G	2	3.34	0.17	
IPI00022463	Serotransferrin precursor	K.LCM*GSGLNLCEPNNK.E	1	3.10	0.38	
IPI00022463	Serotransferrin precursor	K.LCM*GSGLNLCEPNNK.E	2	4.71	0.40	
IPI00022463	Serotransferrin precursor	K.LCM*GSGLNLCEPNNK.E	3	4.47	0.13	
IPI00022463	Serotransferrin precursor	K.LCM*GSGLNLCEPNNKEGYYGYTGAFR.C	2	4.02	0.45	
IPI00022463	Serotransferrin precursor	K.LCM*GSGLNLCEPNNKEGYYGYTGAFR.C	3	6.00	0.50	
IPI00022463	Serotransferrin precursor	K.LCMGSGLNLCEPNNK.E	2	4.18	0.40	
IPI00022463	Serotransferrin precursor	K.LCMGSGLNLCEPNNKEGYYGYTGAFR.C	2	4.13	0.44	

IPI00022463	Serotransferrin precursor	K.LCMGSGLNLCEPNNKEGYYGYTGAFR.C	3	5.65	0.49	
IPI00022463	Serotransferrin precursor	K.LHDRNTYEK.Y	2	2.90	0.22	
IPI00022463	Serotransferrin precursor	K.LHDRNTYEKYLGEEYVK.A	2	4.85	0.42	
IPI00022463	Serotransferrin precursor	K.LHDRNTYEKYLGEEYVK.A	3	5.29	0.27	
IPI00022463	Serotransferrin precursor	K.M*YLGYEYVTAIR.N	2	3.62	0.43	-3.23
IPI00022463	Serotransferrin precursor	K.M*YLGYEYVTAIR.N	3	4.11	0.27	
IPI00022463	Serotransferrin precursor	K.MYLGYEYVTAIR.N	1	2.50	0.33	
IPI00022463	Serotransferrin precursor	K.MYLGYEYVTAIR.N	2	4.77	0.51	
IPI00022463	Serotransferrin precursor	K.MYLGYEYVTAIR.N	3	4.25	0.21	
IPI00022463	Serotransferrin precursor	K.NLNEKDYELLCLDGTR.K	1	4.09	0.45	
IPI00022463	Serotransferrin precursor	K.NLNEKDYELLCLDGTR.K	2	5.45	0.49	
IPI00022463	Serotransferrin precursor	K.NLNEKDYELLCLDGTR.K	3	4.73	0.30	
IPI00022463	Serotransferrin precursor	K.NLNEKDYELLCLDGTRKPVEEYANCHLAR.A	3	5.29	0.45	
IPI00022463	Serotransferrin precursor	K.SASDLTWDNLK.G	1	2.55	0.35	
IPI00022463	Serotransferrin precursor	K.SASDLTWDNLK.G	2	4.05	0.37	
IPI00022463	Serotransferrin precursor	K.SASDLTWDNLKGK.K	1	3.04	0.26	
IPI00022463	Serotransferrin precursor	K.SASDLTWDNLKGK.K	2	4.50	0.32	
IPI00022463	Serotransferrin precursor	K.SDNCEDTPEAGYFAVAVVK.K	2	5.62	0.51	
IPI00022463	Serotransferrin precursor	K.SDNCEDTPEAGYFAVAVVK.K	3	4.02	0.37	
IPI00022463	Serotransferrin precursor	K.SDNCEDTPEAGYFAVAVVKK.S	2	5.16	0.46	
IPI00022463	Serotransferrin precursor	K.SDNCEDTPEAGYFAVAVVKK.S	3	4.23	0.47	
IPI00022463	Serotransferrin precursor	K.SKEFQLFSSPHGK.D	1	2.23	0.41	
IPI00022463	Serotransferrin precursor	K.SKEFQLFSSPHGK.D	2	4.94	0.42	
IPI00022463	Serotransferrin precursor	K.SKEFQLFSSPHGK.D	3	4.19	0.35	
IPI00022463	Serotransferrin precursor	K.SKEFQLFSSPHGKDLLFK.D	2	4.96	0.36	
IPI00022463	Serotransferrin precursor	K.SKEFQLFSSPHGKDLLFK.D	3	4.78	0.22	
IPI00022463	Serotransferrin precursor	K.SKEFQLFSSPHGKDLLFKDSAHGFLK.V	3	7.69	0.50	
IPI00022463	Serotransferrin precursor	K.SVIPSDGPSVACVK.K	1	2.77	0.31	
IPI00022463	Serotransferrin precursor	K.SVIPSDGPSVACVK.K	2	3.28	0.43	
IPI00022463	Serotransferrin precursor	K.SVIPSDGPSVACVKK.A	1	1.71	0.21	
IPI00022463	Serotransferrin precursor	K.SVIPSDGPSVACVKK.A	2	2.84	0.31	
IPI00022463	Serotransferrin precursor	K.YLGEEYVK.A	1	2.18	0.20	
IPI00022463	Serotransferrin precursor	K.YLGEEYVK.A	2	2.96	0.24	
IPI00022463	Serotransferrin precursor	N.SVGKIECVSAETTEDCIAK.I	2	6.35	0.47	
IPI00022463	Serotransferrin precursor	R.AIAANEADAVTLDAGLVYDAYLAPNNLKPVVAEFYGSK.E	3	7.40	0.56	
IPI00022463	Serotransferrin precursor	R.APNHAVVTR.K	1	2.54	0.24	
IPI00022463	Serotransferrin precursor	R.APNHAVVTR.K	2	3.09	0.42	
IPI00022463	Serotransferrin precursor	R.CLVEKGDVAFVK.H	1	3.66	0.38	
IPI00022463	Serotransferrin precursor	R.CLVEKGDVAFVK.H	2	4.45	0.42	
IPI00022463	Serotransferrin precursor	R.CLVEKGDVAFVK.H	3	4.32	0.41	
IPI00022463	Serotransferrin precursor	R.CLVEKGDVAFVKHQTVPQNTGGK.N	3	5.92	0.44	
IPI00022463	Serotransferrin precursor	R.DDTVCLAK.L	1	1.75	0.12	

IPI00022463	Serotransferrin precursor	R.DQYELLCLDNTR.K	2	4.93	0.27	
IPI00022463	Serotransferrin precursor	R.DQYELLCLDNTR.K	3	3.42	0.19	
IPI00022463	Serotransferrin precursor	R.DQYELLCLDNTRKPVDEYKDCHLAQVPSHTVVAR.S	3	5.14	0.42	
IPI00022463	Serotransferrin precursor	R.EGTCPEAPTDECKPVK.W	1	2.79	0.34	
IPI00022463	Serotransferrin precursor	R.EGTCPEAPTDECKPVK.W	2	3.97	0.30	
IPI00022463	Serotransferrin precursor	R.FDEFFSEGCAPGSK.K	1	2.92	0.39	
IPI00022463	Serotransferrin precursor	R.FDEFFSEGCAPGSK.K	2	4.88	0.47	
IPI00022463	Serotransferrin precursor	R.FDEFFSEGCAPGSK.K	3	4.04	0.19	
IPI00022463	Serotransferrin precursor	R.FDEFFSEGCAPGSKK.D	1	3.07	0.36	
IPI00022463	Serotransferrin precursor	R.FDEFFSEGCAPGSKK.D	2	4.43	0.44	
IPI00022463	Serotransferrin precursor	R.FDEFFSEGCAPGSKK.D	3	3.58	0.34	
IPI00022463	Serotransferrin precursor	R.FDEFFSEGCAPGSKKDSSLCK.L	2	4.10	0.28	
IPI00022463	Serotransferrin precursor	R.KCSTSSLLEACTFR.R	1	2.79	0.50	
IPI00022463	Serotransferrin precursor	R.KCSTSSLLEACTFR.R	2	4.86	0.47	
IPI00022463	Serotransferrin precursor	R.KCSTSSLLEACTFR.R	3	3.98	0.19	
IPI00022463	Serotransferrin precursor	R.KPVDEYKDCHLAQVPSHTVVAR.S	3	6.08	0.36	
IPI00022463	Serotransferrin precursor	R.LKCDEWSVNSVGK.I	1	3.40	0.33	
IPI00022463	Serotransferrin precursor	R.LKCDEWSVNSVGK.I	2	4.83	0.34	
IPI00022463	Serotransferrin precursor	R.LKCDEWSVNSVGK.I	3	4.71	0.27	
IPI00022463	Serotransferrin precursor	R.LKCDEWSVNSVGKIECVSAETTEDCIAK.I	2	4.35	0.55	
IPI00022463	Serotransferrin precursor	R.LKCDEWSVNSVGKIECVSAETTEDCIAK.I	3	7.45	0.52	
IPI00022463	Serotransferrin precursor	R.NLREGTCPEAPTDECKPVK.W	2	5.48	0.41	
IPI00022463	Serotransferrin precursor	R.NLREGTCPEAPTDECKPVK.W	3	5.92	0.32	
IPI00022463	Serotransferrin precursor	R.NTYEKYLGEEYVK.A	1	3.75	0.33	
IPI00022463	Serotransferrin precursor	R.NTYEKYLGEEYVK.A	2	4.01	0.32	
IPI00022463	Serotransferrin precursor	R.NTYEKYLGEEYVK.A	3	3.42	0.18	
IPI00022463	Serotransferrin precursor	R.QQQHLFGSNVTDCSGNFCLFR.S	2	3.97	0.41	
IPI00022463	Serotransferrin precursor	R.QQQHLFGSNVTDCSGNFCLFR.S	3	4.10	0.30	
IPI00022463	Serotransferrin precursor	R.SAGWNIPIGLLYCDLPEPR.K	2	5.67	0.45	
IPI00022463	Serotransferrin precursor	R.SAGWNIPIGLLYCDLPEPR.K	3	5.39	0.40	
IPI00022463	Serotransferrin precursor	R.SETKDLLFR.D	1	2.29	0.16	
IPI00022463	Serotransferrin precursor	R.SETKDLLFR.D	2	2.87	0.15	
IPI00022463	Serotransferrin precursor	R.SETKDLLFRDDTVCLAK.L	2	4.77	0.37	
IPI00022463	Serotransferrin precursor	R.SETKDLLFRDDTVCLAK.L	3	4.22	0.24	
IPI00022463	Serotransferrin precursor	R.SM*GGKEDLIWELLNQAQEHFGK.D	2	5.35	0.43	
IPI00022463	Serotransferrin precursor	R.SM*GGKEDLIWELLNQAQEHFGK.D	3	5.80	0.33	
IPI00022463	Serotransferrin precursor	R.SM*GGKEDLIWELLNQAQEHFGKDK.S	2	4.12	0.39	
IPI00022463	Serotransferrin precursor	R.SM*GGKEDLIWELLNQAQEHFGKDK.S	3	5.39	0.32	
IPI00022463	Serotransferrin precursor	R.SMGGKEDLIWELLNQAQEHFGKDK.S	2	4.36	0.25	
IPI00022463	Serotransferrin precursor	R.SMGGKEDLIWELLNQAQEHFGKDK.S	3	6.16	0.40	
IPI00022463	Serotransferrin precursor	R.TAGWNIPM*GLLYNK.I	1	3.32	0.23	
IPI00022463	Serotransferrin precursor	R.TAGWNIPM*GLLYNK.I	2	4.54	0.37	

IPI00022463	Serotransferrin precursor	R.TAGWNIPM*GLLYNK.I	3	3.93	0.34	
IPI00022463	Serotransferrin precursor	R.TAGWNIPMGLLYNK.I	2	4.30	0.31	
IPI00022463	Serotransferrin precursor	R.TAGWNIPMGLLYNK.I	3	3.59	0.20	
IPI00022463	Serotransferrin precursor	R.WCAVSEHEATK.C	1	2.83	0.36	
IPI00022463	Serotransferrin precursor	R.WCAVSEHEATK.C	2	4.09	0.30	
IPI00022463	Serotransferrin precursor	R.WCAVSEHEATK.C	3	2.96	0.18	
IPI00022463	Serotransferrin precursor	R.WCAVSEHEATKCQSFR.D	3	3.47	0.27	
IPI00022463	Serotransferrin precursor	Y.LAPNNLKPVVAEFYGSKEDPQTFYYAVAVVK.K	3	5.74	0.39	
IPI00022488	Hemopexin precursor	A.HGNVAEGETKPDPDVTER.C	3	4.38	0.43	-3.49
IPI00022488	Hemopexin precursor	A.TPLPPTSAHGNVAEGETKPDPDVTER.C	2	3.93	0.48	-3.66
IPI00022488	Hemopexin precursor	D.PVRGEVPPR.Y	2	3.28	0.22	-0.70
IPI00022488	Hemopexin precursor	F.PGIPSPLDAAVECHR.G	2	3.77	0.46	-1.24
IPI00022488	Hemopexin precursor	F.PSPVDAAFR.Q	1	2.74	0.17	-2.68
IPI00022488	Hemopexin precursor	G.NVAEGETKPDPDVTER.C	2	3.82	0.41	-3.71
IPI00022488	Hemopexin precursor	I.PSPLDAAVECHR.G	1	2.40	0.26	-2.78
IPI00022488	Hemopexin precursor	I.PSPLDAAVECHR.G	2	3.29	0.43	-3.75
IPI00022488	Hemopexin precursor	K.ALPQPQNVTSLLGCTH.-	2	2.74	0.37	-3.21
IPI00022488	Hemopexin precursor	K.EVGTPHGIILDSVDAAFICPGSSR.L	2	6.05	0.53	-5.65
IPI00022488	Hemopexin precursor	K.EVGTPHGIILDSVDAAFICPGSSR.L	3	6.16	0.47	-7.12
IPI00022488	Hemopexin precursor	K.GDKVWVYPPEK.K	2	3.04	0.35	
IPI00022488	Hemopexin precursor	K.GDKVWVYPPEK.K	3	2.87	0.06	-3.66
IPI00022488	Hemopexin precursor	K.GDKVWVYPPEKK.E	2	3.61	0.34	-4.41
IPI00022488	Hemopexin precursor	K.GDKVWVYPPEKK.E	3	3.15	0.18	-4.10
IPI00022488	Hemopexin precursor	K.GGYTLVSGYPK.R	1	2.88	0.33	-2.78
IPI00022488	Hemopexin precursor	K.GGYTLVSGYPK.R	2	3.92	0.37	-5.42
IPI00022488	Hemopexin precursor	K.GGYTLVSGYPKR.L	2	2.68	0.27	-2.83
IPI00022488	Hemopexin precursor	K.GGYTLVSGYPKR.L	3	2.15	0.25	-2.07
IPI00022488	Hemopexin precursor	K.GGYTLVSGYPKRLEK.E	2	3.72	0.27	-4.38
IPI00022488	Hemopexin precursor	K.GGYTLVSGYPKRLEK.E	3	1.93	0.24	-4.07
IPI00022488	Hemopexin precursor	K.LLQDEFPGIPSPLDAAVECHR.G	2	5.26	0.36	-3.83
IPI00022488	Hemopexin precursor	K.LLQDEFPGIPSPLDAAVECHR.G	3	3.60	0.20	-3.50
IPI00022488	Hemopexin precursor	K.LLQDEFPGIPSPLDAAVECHRGECQAEGVLFFQGDR.E	3	4.20	0.44	-2.30
IPI00022488	Hemopexin precursor	K.LLQDEFPGIPSPLDAAVECHRGECQAEGVLFFQGDR.E	4	3.78	0.32	-2.32
IPI00022488	Hemopexin precursor	K.LYLVQGTQVYVFLTK.G	2	5.43	0.51	-6.64
IPI00022488	Hemopexin precursor	K.LYLVQGTQVYVFLTK.G	3	5.04	0.50	-7.96
IPI00022488	Hemopexin precursor	K.NFPSPVDAAFR.Q	1	2.62	0.32	-3.24
IPI00022488	Hemopexin precursor	K.NFPSPVDAAFR.Q	2	2.76	0.28	-3.53
IPI00022488	Hemopexin precursor	K.RLEKEVGTPHGIILDSVDAAFICPGSSR.L	3	6.22	0.56	-3.52
IPI00022488	Hemopexin precursor	K.RLEKEVGTPHGIILDSVDAAFICPGSSR.L	5	3.06	0.08	-3.19
IPI00022488	Hemopexin precursor	K.SGAQATWTELPWPHEK.V	2	4.55	0.43	-4.65
IPI00022488	Hemopexin precursor	K.SGAQATWTELPWPHEK.V	3	4.61	0.52	-3.59
IPI00022488	Hemopexin precursor	K.SGAQATWTELPWPHEKVDGALCM*EK.S	2	2.98	0.46	-4.62

IPI00022488	Hemopexin precursor	K.SGAQATWTELPWPHEKVDGALCM*EK.S	3	4.96	0.60	-5.03
IPI00022488	Hemopexin precursor	K.SGAQATWTELPWPHEKVDGALCM*EK.S	4	3.86	0.35	-4.42
IPI00022488	Hemopexin precursor	K.SGAQATWTELPWPHEKVDGALCMEK.S	2	3.55	0.27	
IPI00022488	Hemopexin precursor	K.SGAQATWTELPWPHEKVDGALCMEK.S	3	4.72	0.46	
IPI00022488	Hemopexin precursor	K.SLGPNSCSANGPGLYLIHGPNLYCYSDVEK.L	2	3.62	0.45	-2.60
IPI00022488	Hemopexin precursor	K.SLGPNSCSANGPGLYLIHGPNLYCYSDVEK.L	3	5.13	0.52	-4.17
IPI00022488	Hemopexin precursor	K.SLGPNSCSANGPGLYLIHGPNLYCYSDVEK.L	4	5.04	0.44	-3.37
IPI00022488	Hemopexin precursor	K.SLGPNSCSANGPGLYLIHGPNLYCYSDVEKLNAAK.A	3	4.22	0.39	-2.83
IPI00022488	Hemopexin precursor	K.SLGPNSCSANGPGLYLIHGPNLYCYSDVEKLNAAK.A	4	4.23	0.37	-3.08
IPI00022488	Hemopexin precursor	K.VDGALCM*EK.S	1	1.41	0.15	-4.75
IPI00022488	Hemopexin precursor	K.VDGALCM*EK.S	2	3.15	0.30	-2.54
IPI00022488	Hemopexin precursor	K.VDGALCMEK.S	2	2.11	0.22	
IPI00022488	Hemopexin precursor	L.PPTSAHGNVAEGETKPDPDVTER.C	2	4.36	0.43	-4.25
IPI00022488	Hemopexin precursor	L.PPTSAHGNVAEGETKPDPDVTER.C	3	4.61	0.47	-5.32
IPI00022488	Hemopexin precursor	L.PPTSAHGNVAEGETKPDPDVTER.C	4	5.54	0.47	-1.79
IPI00022488	Hemopexin precursor	N.FPSPVDAAFR.Q	1	2.29	0.35	-3.62
IPI00022488	Hemopexin precursor	N.FPSPVDAAFR.Q	2	3.05	0.34	-2.10
IPI00022488	Hemopexin precursor	N.VAEGETKPDPDVTER.C	2	4.03	0.31	-4.00
IPI00022488	Hemopexin precursor	P.LPPTSAHGNVAEGETKPDPDVTER.C	2	4.30	0.41	-3.37
IPI00022488	Hemopexin precursor	P.LPPTSAHGNVAEGETKPDPDVTER.C	3	4.10	0.50	-3.49
IPI00022488	Hemopexin precursor	P.TSAHGNVAEGETKPDPDVTER.C	2	5.17	0.56	-5.39
IPI00022488	Hemopexin precursor	Q.GHNSVFLIK.G	1	2.33	0.20	-4.62
IPI00022488	Hemopexin precursor	Q.GHNSVFLIKGDK.V	2	3.21	0.45	-3.35
IPI00022488	Hemopexin precursor	R.CSPHLVLSALTSDNHGATYAFSGTHYWR.L	3	6.61	0.51	
IPI00022488	Hemopexin precursor	R.DGWHSWPIAHQWPQGPSAVDAAFSWEEK.L	2	4.43	0.45	
IPI00022488	Hemopexin precursor	R.DGWHSWPIAHQWPQGPSAVDAAFSWEEK.L	3	4.60	0.20	
IPI00022488	Hemopexin precursor	R.DVRDYFM*PCPGR.G	2	2.55	0.31	-3.46
IPI00022488	Hemopexin precursor	R.DVRDYFM*PCPGR.G	3	2.92	0.20	-1.92
IPI00022488	Hemopexin precursor	R.DVRDYFMPCPGR.G	2	2.84	0.09	
IPI00022488	Hemopexin precursor	R.DYFM*PCPGR.G	2	2.93	0.28	-4.51
IPI00022488	Hemopexin precursor	R.DYFMPCPGR.G	2	2.96	0.18	
IPI00022488	Hemopexin precursor	R.EWFWDLATGTM*K.E	1	2.68	0.52	-2.50
IPI00022488	Hemopexin precursor	R.EWFWDLATGTM*K.E	2	3.95	0.49	-3.35
IPI00022488	Hemopexin precursor	R.EWFWDLATGTM*KER.S	3	2.26	0.24	-1.42
IPI00022488	Hemopexin precursor	R.EWFWDLATGTMKER.S	2	3.04	0.42	-2.98
IPI00022488	Hemopexin precursor	R.EWFWDLATGTMKER.S	3	2.04	0.21	-3.19
IPI00022488	Hemopexin precursor	R.FDPVRGEVPPR.Y	2	1.71	0.10	-2.16
IPI00022488	Hemopexin precursor	R.FDPVRGEVPPR.Y	3	1.87	0.29	-4.23
IPI00022488	Hemopexin precursor	R.FDPVRGEVPPRYPR.D	2	1.40	0.27	-2.89
IPI00022488	Hemopexin precursor	R.GECQAEGVLFFQGDR.E	2	3.48	0.11	
IPI00022488	Hemopexin precursor	R.GECQAEGVLFFQGDREWFWDLATGTM*K.E	3	5.87	0.63	-4.16
IPI00022488	Hemopexin precursor	R.GECQAEGVLFFQGDREWFWDLATGTMK.E	3	5.42	0.46	

IPI00022488	Hemopexin precursor	R.GEVPPRYPR.D	2	2.41	0.16	
IPI00022488	Hemopexin precursor	R.LEKEVGTPHGIILDSVDAAFICPGSSR.L	4	3.85	0.24	-1.68
IPI00022488	Hemopexin precursor	R.LWWLDLK.S	1	2.00	0.06	
IPI00022488	Hemopexin precursor	R.LWWLDLK.S	2	2.01	0.05	-0.24
IPI00022488	Hemopexin precursor	R.QGHNSVFLIK.G	1	1.88	0.06	-5.11
IPI00022488	Hemopexin precursor	R.SWPAVGNCSSALR.W	2	3.51	0.39	-3.76
IPI00022488	Hemopexin precursor	R.WKNFPSPVDAAFR.Q	2	2.35	0.28	-3.16
IPI00022488	Hemopexin precursor	R.WKNFPSPVDAAFR.Q	3	4.00	0.39	-3.66
IPI00022488	Hemopexin precursor	R.YYCFQGNQFLR.F	1	3.44	0.40	-2.43
IPI00022488	Hemopexin precursor	R.YYCFQGNQFLR.F	2	3.82	0.45	-5.30
IPI00022488	Hemopexin precursor	R.YYCFQGNQFLR.F	3	4.93	0.36	-2.71
IPI00022488	Hemopexin precursor	S.PLDAAVECHR.G	2	3.28	0.46	-2.55
IPI00022488	Hemopexin precursor	T.SAHGNVAEGETKPDPDVTER.C	3	3.61	0.39	-3.06
IPI00022488	Hemopexin precursor	W.KNFPSPVDAAFR.Q	3	4.28	0.42	-2.86
IPI00022488	Hemopexin precursor	W.PHEKVDGALCM*EK.S	2	3.64	0.33	-3.15
IPI00022488	Hemopexin precursor	W.PQGPSAVDAAFSWEEK.L	2	4.81	0.53	-2.18
IPI00022488	Hemopexin precursor	Y.CYSDVEKLNAAK.A	2	4.02	0.38	0.26
IPI00022488	Hemopexin precursor	Y.YCFQGNQFLR.F	2	3.06	0.25	-1.63
IPI00022542	Rho-associated protein kinase 1	K.QCSMLDVDLK.Q	2	2.65	0.07	
IPI00022606	Isoform 1 of Proline-serine-threonine phosphatase-interacting protein 1	K.TYEQKCR.D	2	2.01	0.20	
IPI00022608	Sortilin-related receptor precursor	K.GGTWEFLQAPAFTGYGEK.I	2	4.13	0.40	
IPI00022608	Sortilin-related receptor precursor	K.IEVANPDGDFR.L	2	3.58	0.14	
IPI00022608	Sortilin-related receptor precursor	K.M*SEDLSLEVCVPDPEFSGK.S	2	2.52	0.24	
IPI00022608	Sortilin-related receptor precursor	K.NLLVNTLYTVR.V	2	2.89	0.22	-0.50
IPI00022608	Sortilin-related receptor precursor	K.SYSPPVPCPVGSTYR.R	2	2.50	0.26	
IPI00022608	Sortilin-related receptor precursor	K.TDLGDSPLAFEHVM*TR.G	3	1.99	0.11	-2.15
IPI00022608	Sortilin-related receptor precursor	K.TNVYISSSAGAR.W	2	3.35	0.36	
IPI00022608	Sortilin-related receptor precursor	R.GFLVVQGDPR.E	2	3.59	0.29	-1.03
IPI00022608	Sortilin-related receptor precursor	R.LEGELVPCPLAEENEFILYAVR.K	2	3.63	0.13	
IPI00022608	Sortilin-related receptor precursor	R.LHGGSAPLPQDR.G	2	2.42	0.10	-0.39
IPI00022608	Sortilin-related receptor precursor	R.NCPTTICDLDTQFR.C	2	2.51	0.22	
IPI00022608	Sortilin-related receptor precursor	R.VVVPYQGPSSDYVVVK.M	2	2.60	0.17	-3.74
IPI00022640	Neurogranin	K.GPGPGGPGGAGVAR.G	2	2.26	0.12	-1.31
IPI00022640	Neurogranin	R.KGPGPGGPGGAGVAR.G	2	3.48	0.46	-4.24
IPI00022640	Neurogranin	R.KGPGPGGPGGAGVAR.G	3	3.41	0.31	-3.02
IPI00022649	Isoform 1 of Solute carrier family 12 member 2	R.AAAAAAAAAAAAAAAGAGAGAK.Q	2	4.79	0.33	-2.74
IPI00022649	Isoform 1 of Solute carrier family 12 member 2	R.DGGGVRDEGPAAAGDGLGRPLGPTPSQSR.F	4	2.39	0.17	-0.13
IPI00022649	Isoform 1 of Solute carrier family 12 member 2	R.VELPGTAVPSVPEDAAPASR.D	2	4.18	0.56	-4.94
IPI00022674	Isoform 1 of Oncostatin-M specific receptor subunit beta precursor	K.GIVLFVSK.V	2	2.24	0.12	-1.25

IPI00022674	Isoform 1 of Oncostatin-M specific receptor subunit beta precursor	K.QIHGEQLDPHVTAFNLNSVPFIR.N	4	2.64	0.11	-4.10
IPI00022674	Isoform 1 of Oncostatin-M specific receptor subunit beta precursor	K.QPSQSYTLFESFSGEK.K	2	2.99	0.11	-4.56
IPI00022774	Transitional endoplasmic reticulum ATPase	K.MDLIDLEDETIDAEVMNSLAVTMDDFR.W	3	3.00	0.32	-2.25
IPI00022774	Transitional endoplasmic reticulum ATPase	R.KKMDLIDLEDETIDAEVMNSLAVTMDDFR.W	3	4.18	0.44	-1.51
IPI00022774	Transitional endoplasmic reticulum ATPase	R.KKMDLIDLEDETIDAEVMNSLAVTMDDFR.W	4	2.92	0.21	-1.62
IPI00022774	Transitional endoplasmic reticulum ATPase	R.QTNPSAMEVEEDDPVPEIRRDHFEEAMR.F	3	2.74	0.18	-6.81
IPI00022792	Microfibril-associated glycoprotein 4 precursor	K.FSTFDRDQDLFVQNCAALSSGAFWFR.S	3	5.78	0.51	-3.18
IPI00022792	Microfibril-associated glycoprotein 4 precursor	K.FSTFDRDQDLFVQNCAALSSGAFWFR.S	4	2.97	0.16	-1.02
IPI00022792	Microfibril-associated glycoprotein 4 precursor	K.GFYYSLK.R	1	1.73	0.17	-2.49
IPI00022792	Microfibril-associated glycoprotein 4 precursor	K.GFYYSLK.R	2	2.32	0.18	-1.24
IPI00022792	Microfibril-associated glycoprotein 4 precursor	K.GFYYSLKR.T	2	2.41	0.26	-0.59
IPI00022792	Microfibril-associated glycoprotein 4 precursor	K.YADFSISPNAVSAEEDGYTLFVAGFEDGGAGDSLSYHSGQK.F	3	6.16	0.61	-1.80
IPI00022792	Microfibril-associated glycoprotein 4 precursor	K.YADFSISPNAVSAEEDGYTLFVAGFEDGGAGDSLSYHSGQK.F	4	4.81	0.44	-3.79
IPI00022792	Microfibril-associated glycoprotein 4 precursor	R.ADGEYWLGLQNM*HLLTLK.Q	2	3.78	0.42	-2.15
IPI00022792	Microfibril-associated glycoprotein 4 precursor	R.ADGEYWLGLQNM*HLLTLK.Q	3	3.26	0.40	-3.58
IPI00022792	Microfibril-associated glycoprotein 4 precursor	R.ADGEYWLGLQNMHLLTLK.Q	3	3.20	0.18	
IPI00022792	Microfibril-associated glycoprotein 4 precursor	W.LGLQNM*HLLTLK.Q	3	3.69	0.34	-0.50
IPI00022810	Dipeptidyl-peptidase 1 precursor	K.KVGTASENVYYVNTAHLK.N	3	3.77	0.44	-2.55
IPI00022810	Dipeptidyl-peptidase 1 precursor	K.KVVVYLQK.L	2	1.84	0.19	-2.47
IPI00022810	Dipeptidyl-peptidase 1 precursor	K.PAPLTAEIQQK.I	2	2.92	0.27	-2.49
IPI00022810	Dipeptidyl-peptidase 1 precursor	K.VGTASENVYYVNTAHLK.N	2	4.09	0.35	-3.94
IPI00022810	Dipeptidyl-peptidase 1 precursor	K.VGTASENVYYVNTAHLK.N	3	2.72	0.37	-4.04
IPI00022810	Dipeptidyl-peptidase 1 precursor	K.VVVYLQK.L	2	2.53	0.14	-2.67
IPI00022810	Dipeptidyl-peptidase 1 precursor	K.YDHNFVK.A	2	2.11	0.19	-3.54
IPI00022810	Dipeptidyl-peptidase 1 precursor	R.NVHGINFVSPVR.N	2	2.74	0.42	-2.36
IPI00022810	Dipeptidyl-peptidase 1 precursor	R.NVHGINFVSPVR.N	3	3.66	0.22	-3.25
IPI00022810	Dipeptidyl-peptidase 1 precursor	W.VFQVGSSGSQR.D	2	3.03	0.30	-1.65
IPI00022822	Isoform 2 of Collagen alpha-1(XVIII) chain precursor	K.LSGVQDGHQDISLLYTEPGAGQTHTAASFR.L	3	5.33	0.48	-3.06
IPI00022822	Isoform 2 of Collagen alpha-1(XVIII) chain precursor	R.AAVPIVNLKDELLFPSWEALFSGSEGPLKPGAR.I	3	5.34	0.54	-3.88
IPI00022822	Isoform 2 of Collagen alpha-1(XVIII) chain precursor	R.AAVPIVNLKDELLFPSWEALFSGSEGPLKPGAR.I	4	4.04	0.38	-3.43
IPI00022822	Isoform 2 of Collagen alpha-1(XVIII) chain precursor	R.AVGLAGTFR.A	2	2.59	0.21	-4.65
IPI00022822	Isoform 2 of Collagen alpha-1(XVIII) chain precursor	R.DFQPVLHLVALNSPLSGGM*R.G	2	5.49	0.56	-2.74
IPI00022822	Isoform 2 of Collagen alpha-1(XVIII) chain precursor	R.DFQPVLHLVALNSPLSGGM*R.G	3	5.04	0.35	-3.92

IPI00022822	Isoform 2 of Collagen alpha-1(XVIII) chain precursor	R.ELLREETGAALKPR.L	2	3.54	0.12	-3.32
IPI00022822	Isoform 2 of Collagen alpha-1(XVIII) chain precursor	R.ELLREETGAALKPR.L	3	4.46	0.32	-1.36
IPI00022822	Isoform 2 of Collagen alpha-1(XVIII) chain precursor	R.GADFQCFQQAR.A	2	3.38	0.35	-3.45
IPI00022822	Isoform 2 of Collagen alpha-1(XVIII) chain precursor	R.GLELEPGAGLFVAQAGGADPDKFQGVIAELK.V	2	2.91	0.16	-2.02
IPI00022822	Isoform 2 of Collagen alpha-1(XVIII) chain precursor	R.GLELEPGAGLFVAQAGGADPDKFQGVIAELK.V	3	2.69	0.30	-3.36
IPI00022822	Isoform 2 of Collagen alpha-1(XVIII) chain precursor	R.GLELEPGAGLFVAQAGGADPDKFQGVIAELK.V	4	3.92	0.40	-3.90
IPI00022822	Isoform 2 of Collagen alpha-1(XVIII) chain precursor	R.IFSFDGKDVLR.H	2	3.06	0.35	-1.78
IPI00022822	Isoform 2 of Collagen alpha-1(XVIII) chain precursor	R.LQDLYSIVR.R	2	3.32	0.28	-2.64
IPI00022822	Isoform 2 of Collagen alpha-1(XVIII) chain precursor	R.LTESYCETWR.T	2	3.31	0.30	-2.34
IPI00022822	Isoform 2 of Collagen alpha-1(XVIII) chain precursor	R.TEAPSATGQASSLLGGR.L	2	5.06	0.53	-3.30
IPI00022822	Isoform 2 of Collagen alpha-1(XVIII) chain precursor	R.YHFPSLFFR.D	3	2.61	0.08	-4.06
IPI00022890	Ig lambda chain V region 4A precursor	R.ALIYSTSNK.H	1	1.66	0.15	
IPI00022890	Ig lambda chain V region 4A precursor	R.FSGSLLGGK.A	2	2.88	0.08	
IPI00022891	ADP/ATP translocase 1	R.M*MMQSGRKGADIM*YTGTVDCWR.K	3	2.43	0.11	2.56
IPI00022891	ADP/ATP translocase 1	R.RMMM*QSGR.K	2	2.50	0.12	
IPI00022891	ADP/ATP translocase 1	R.YFAGNLASGGAAGATSLCFVYPLDFAR.T	2	4.58	0.57	-2.30
IPI00022892	Thy-1 membrane glycoprotein precursor	K.HVLFGTVGVPEHTYR.S	2	4.49	0.57	-4.53
IPI00022892	Thy-1 membrane glycoprotein precursor	K.HVLFGTVGVPEHTYR.S	3	2.71	0.23	-3.07
IPI00022892	Thy-1 membrane glycoprotein precursor	K.KHVLFGTVGVPEHTYR.S	2	5.39	0.50	-5.25
IPI00022892	Thy-1 membrane glycoprotein precursor	K.KHVLFGTVGVPEHTYR.S	3	3.36	0.34	-4.17
IPI00022892	Thy-1 membrane glycoprotein precursor	K.VLYLSAFTSK.D	1	2.34	0.36	-4.97
IPI00022892	Thy-1 membrane glycoprotein precursor	K.VLYLSAFTSK.D	2	3.80	0.35	-3.15
IPI00022892	Thy-1 membrane glycoprotein precursor	K.VTSLTACLVDQSLR.L	2	4.54	0.40	-4.19
IPI00022892	Thy-1 membrane glycoprotein precursor	K.VTSLTACLVDQSLR.L	3	5.23	0.39	-2.98
IPI00022892	Thy-1 membrane glycoprotein precursor	R.HENTSSSPIQYEFSLTR.E	2	3.18	0.48	0.98
IPI00022892	Thy-1 membrane glycoprotein precursor	S.SPIQYEFSLTR.E	2	3.06	0.35	-1.97
IPI00022895	Alpha-1B-glycoprotein precursor	K.HQFLLTGDTQGR.Y	1	3.34	0.38	-2.64
IPI00022895	Alpha-1B-glycoprotein precursor	K.HQFLLTGDTQGR.Y	2	3.66	0.38	-3.33
IPI00022895	Alpha-1B-glycoprotein precursor	K.LLELTGPK.S	1	2.76	0.23	-2.25
IPI00022895	Alpha-1B-glycoprotein precursor	K.LLELTGPK.S	2	2.75	0.25	-3.61
IPI00022895	Alpha-1B-glycoprotein precursor	K.NGVAQEPVHLDSPAIK.H	2	4.38	0.50	-2.87

IPI00022895	Alpha-1B-glycoprotein precursor	K.NGVAQEPVHLDSPAIK.H	3	2.06	0.20	-2.05
IPI00022895	Alpha-1B-glycoprotein precursor	K.NGVAQEPVHLDSPAIKHQFLLTGDTQGR.Y	3	5.53	0.50	-4.18
IPI00022895	Alpha-1B-glycoprotein precursor	K.NGVAQEPVHLDSPAIKHQFLLTGDTQGR.Y	4	2.62	0.25	-3.32
IPI00022895	Alpha-1B-glycoprotein precursor	K.SLPAPWLSM*APVSWITPGLK.T	2	5.56	0.63	-5.35
IPI00022895	Alpha-1B-glycoprotein precursor	K.SLPAPWLSM*APVSWITPGLK.T	3	5.47	0.49	-4.13
IPI00022895	Alpha-1B-glycoprotein precursor	K.SLPAPWLSMAPVSWITPGLK.T	2	4.28	0.51	-2.64
IPI00022895	Alpha-1B-glycoprotein precursor	K.SLPAPWLSMAPVSWITPGLK.T	3	3.21	0.33	-2.80
IPI00022895	Alpha-1B-glycoprotein precursor	K.VTLTCVAPLSGVDFQLR.R	1	3.11	0.18	
IPI00022895	Alpha-1B-glycoprotein precursor	K.VTLTCVAPLSGVDFQLR.R	2	5.40	0.51	-4.43
IPI00022895	Alpha-1B-glycoprotein precursor	K.VTLTCVAPLSGVDFQLR.R	3	5.94	0.52	-3.45
IPI00022895	Alpha-1B-glycoprotein precursor	R.ATWSGAVLAGR.D	1	2.11	0.29	-2.71
IPI00022895	Alpha-1B-glycoprotein precursor	R.ATWSGAVLAGR.D	2	3.94	0.29	-1.75
IPI00022895	Alpha-1B-glycoprotein precursor	R.CEGPIPDVTFELLR.E	1	1.72	0.11	
IPI00022895	Alpha-1B-glycoprotein precursor	R.CEGPIPDVTFELLR.E	2	4.30	0.39	-5.75
IPI00022895	Alpha-1B-glycoprotein precursor	R.CEGPIPDVTFELLR.E	3	2.20	0.13	-1.66
IPI00022895	Alpha-1B-glycoprotein precursor	R.CEGPIPDVTFELLREGETK.A	2	3.31	0.47	-2.45
IPI00022895	Alpha-1B-glycoprotein precursor	R.CEGPIPDVTFELLREGETK.A	3	3.24	0.28	-2.90
IPI00022895	Alpha-1B-glycoprotein precursor	R.CLAPLEGAR.F	1	2.34	0.20	-3.51
IPI00022895	Alpha-1B-glycoprotein precursor	R.CLAPLEGAR.F	2	3.30	0.29	-1.33
IPI00022895	Alpha-1B-glycoprotein precursor	R.FALVREDR.G	2	2.02	0.12	-2.58
IPI00022895	Alpha-1B-glycoprotein precursor	R.GEKELLVPR.S	1	2.08	0.14	-3.40
IPI00022895	Alpha-1B-glycoprotein precursor	R.GEKELLVPR.S	2	1.87	0.05	-2.03
IPI00022895	Alpha-1B-glycoprotein precursor	R.LELHVDGPPPRPQLR.A	2	2.79	0.30	-3.64
IPI00022895	Alpha-1B-glycoprotein precursor	R.LELHVDGPPPRPQLR.A	3	3.22	0.31	-4.75
IPI00022895	Alpha-1B-glycoprotein precursor	R.LELHVDGPPPRPQLR.A	4	2.89	0.36	-4.74
IPI00022895	Alpha-1B-glycoprotein precursor	R.LETPDFQLFK.N	1	2.53	0.11	-3.91
IPI00022895	Alpha-1B-glycoprotein precursor	R.LETPDFQLFK.N	2	2.64	0.28	-4.07
IPI00022895	Alpha-1B-glycoprotein precursor	R.LHDNQNGWSGDSAPVELILSDETLPAPEFSPEPESGR.A	3	6.42	0.57	-3.25
IPI00022895	Alpha-1B-glycoprotein precursor	R.LHDNQNGWSGDSAPVELILSDETLPAPEFSPEPESGR.A	4	4.90	0.39	-3.81
IPI00022895	Alpha-1B-glycoprotein precursor	R.RGEKELLVPR.S	2	3.66	0.27	-4.05
IPI00022895	Alpha-1B-glycoprotein precursor	R.SGLSTGWTQLSK.L	1	2.33	0.30	-5.48
IPI00022895	Alpha-1B-glycoprotein precursor	R.SGLSTGWTQLSK.L	2	3.99	0.34	-2.52
IPI00022895	Alpha-1B-glycoprotein precursor	R.SSTSPDRIFFHLNAVALGDGGHYTCR.Y	3	6.62	0.43	
IPI00022895	Alpha-1B-glycoprotein precursor	R.SWVPHTFESELSDPVELLVAES.-	2	3.90	0.33	-5.98
IPI00022895	Alpha-1B-glycoprotein precursor	R.SWVPHTFESELSDPVELLVAES.-	3	4.52	0.42	-4.08
IPI00022895	Alpha-1B-glycoprotein precursor	R.TDGEGALSEPSATVTIEELAAPPPPVLM*HHGESSQVLHPGNK.V	3	4.24	0.52	-4.31
IPI00022895	Alpha-1B-glycoprotein precursor	R.TDGEGALSEPSATVTIEELAAPPPPVLM*HHGESSQVLHPGNK.V	4	3.62	0.27	-4.07
IPI00022895	Alpha-1B-glycoprotein precursor	R.TDGEGALSEPSATVTIEELAAPPPPVLM*HHGESSQVLHPGNK.V	5	1.98	0.28	-2.72
IPI00022895	Alpha-1B-glycoprotein precursor	R.TDGEGALSEPSATVTIEELAAPPPPVLMHHGESSQVLHPGNK.V	3	5.06	0.41	
IPI00022895	Alpha-1B-glycoprotein precursor	R.TPGAAANLELIFVGPQHAGNYR.C	2	5.63	0.58	-6.05
IPI00022895	Alpha-1B-glycoprotein precursor	R.TPGAAANLELIFVGPQHAGNYR.C	3	6.99	0.62	-5.55
IPI00022895	Alpha-1B-glycoprotein precursor	R.YRLHDNQNGWSGDSAPVELILSDETLPAPEFSPEPESGR.A	3	4.92	0.40	

IPI00022895	Alpha-1B-glycoprotein precursor	W.SGDSAPVELILSDETLPAPEFSPEPESGR.A	3	4.73	0.40	-4.69
IPI00022937	Coagulation factor V	E.KPQSTISGLLGPTLYAEVGDIIK.V	3	4.33	0.43	-3.07
IPI00022937	Coagulation factor V	K.ADKPLSIHPQGIR.Y	2	3.17	0.38	-3.88
IPI00022937	Coagulation factor V	K.DGTDYIEIIPK.E	2	3.85	0.26	-3.90
IPI00022937	Coagulation factor V	K.DSNM*PM*DM*R.E	2	1.91	0.40	-3.64
IPI00022937	Coagulation factor V	K.EDGILGPIIR.A	2	2.52	0.18	-1.62
IPI00022937	Coagulation factor V	K.EFNPLVIVGLSK.D	1	3.26	0.34	-3.86
IPI00022937	Coagulation factor V	K.EFNPLVIVGLSK.D	2	3.89	0.36	-3.76
IPI00022937	Coagulation factor V	K.EKPQSTISGLLGPTLYAEVGDIIK.V	2	4.36	0.42	-2.69
IPI00022937	Coagulation factor V	K.EKPQSTISGLLGPTLYAEVGDIIK.V	3	6.54	0.53	-6.08
IPI00022937	Coagulation factor V	K.EVIITGIQTQGAK.H	2	4.49	0.43	-2.76
IPI00022937	Coagulation factor V	K.FTVNNLAEPQK.A	2	2.92	0.27	-2.52
IPI00022937	Coagulation factor V	K.HTVNPNM*KEDGILGPIIR.A	2	4.06	0.29	
IPI00022937	Coagulation factor V	K.IVYREYEPYFK.K	3	2.11	0.18	-1.71
IPI00022937	Coagulation factor V	K.IVYREYEPYFKK.E	2	2.64	0.07	-3.70
IPI00022937	Coagulation factor V	K.IVYREYEPYFKK.E	3	2.64	0.22	-2.88
IPI00022937	Coagulation factor V	K.KVM*YTQYEDESFTK.H	3	2.65	0.09	0.54
IPI00022937	Coagulation factor V	K.LSEGASYLDHTFPAEK.M	2	4.97	0.44	-2.23
IPI00022937	Coagulation factor V	K.LSEGASYLDHTFPAEK.M	3	2.95	0.22	-2.01
IPI00022937	Coagulation factor V	K.LSEGASYLDHTFPAEKM*DDAVAPGR.E	3	4.62	0.43	-3.37
IPI00022937	Coagulation factor V	K.LSEGASYLDHTFPAEKM*DDAVAPGR.E	4	2.89	0.22	1.98
IPI00022937	Coagulation factor V	K.M*DDAVAPGR.E	2	2.33	0.07	-2.99
IPI00022937	Coagulation factor V	K.M*YEQEWVR.L	2	3.01	0.16	-1.83
IPI00022937	Coagulation factor V	K.NFFNPPIISR.F	2	2.65	0.18	-2.48
IPI00022937	Coagulation factor V	K.NKADKPLSIHPQGIR.Y	4	1.92	0.17	-4.31
IPI00022937	Coagulation factor V	K.QITASSFKK.S	1	2.20	0.07	-4.66
IPI00022937	Coagulation factor V	K.RDPRGEYEEHLGILGPIIR.A	3	3.09	0.27	-2.50
IPI00022937	Coagulation factor V	K.SSM*VDKIFEGNTNTK.G	2	3.25	0.34	-2.02
IPI00022937	Coagulation factor V	K.SSM*VDKIFEGNTNTK.G	3	2.58	0.14	2.92
IPI00022937	Coagulation factor V	K.TFDKQIVLLFAVFDESK.S	3	3.63	0.42	-3.04
IPI00022937	Coagulation factor V	K.WIISSLTPK.H	2	2.61	0.17	-0.71
IPI00022937	Coagulation factor V	K.WNILEFDEPTENDAQCLTRPYYSDVDIM*R.D	3	5.83	0.55	-2.71
IPI00022937	Coagulation factor V	K.YLDSTFTK.R	2	2.63	0.26	-2.12
IPI00022937	Coagulation factor V	R.AADIEQQAVFAVFDENK.S	2	5.60	0.55	-5.68
IPI00022937	Coagulation factor V	R.AADIEQQAVFAVFDENK.S	3	4.83	0.43	-4.21
IPI00022937	Coagulation factor V	R.AEVDDVIQVR.F	2	3.91	0.28	-3.05
IPI00022937	Coagulation factor V	R.AGM*QTPFLIM*DR.D	2	3.53	0.27	-2.22
IPI00022937	Coagulation factor V	R.AVQPGETYTYK.W	1	2.21	0.13	-1.77
IPI00022937	Coagulation factor V	R.AVQPGETYTYK.W	2	2.98	0.41	-2.27
IPI00022937	Coagulation factor V	R.AWAYYSAVNPEKDIHSGLIGPLLICQK.G	4	2.98	0.21	-2.70
IPI00022937	Coagulation factor V	R.DIASGLIGLLLICK.S	2	4.20	0.38	-3.49
IPI00022937	Coagulation factor V	R.DIASGLIGLLLICK.S	3	3.81	0.31	-1.62

IPI00022937	Coagulation factor V	R.ETDIEDSDDIPEDTTYK.K	2	5.42	0.55	-4.58
IPI00022937	Coagulation factor V	R.GEYEEHLGILGPIIR.A	2	3.68	0.42	-0.73
IPI00022937	Coagulation factor V	R.GEYEEHLGILGPIIR.A	3	3.54	0.30	-0.99
IPI00022937	Coagulation factor V	R.KM*HDRLEPEDEESDADYDYQNR.L	4	2.71	0.16	-1.19
IPI00022937	Coagulation factor V	R.KYLDSTFTK.R	2	2.52	0.08	-2.63
IPI00022937	Coagulation factor V	R.LLSLGAGEFK.S	1	1.92	0.16	-2.71
IPI00022937	Coagulation factor V	R.LLSLGAGEFK.S	2	2.17	0.11	-2.75
IPI00022937	Coagulation factor V	R.LNNGGSYNAWSVEK.L	2	4.53	0.48	-1.79
IPI00022937	Coagulation factor V	R.M*PM*GLSTGIISDSQIK.A	2	4.40	0.39	-2.90
IPI00022937	Coagulation factor V	R.M*PM*GLSTGIISDSQIK.A	3	2.94	0.10	-2.19
IPI00022937	Coagulation factor V	R.NVM*YFNGNSDASTIKENQFDPPIVAR.Y	3	4.78	0.49	-4.30
IPI00022937	Coagulation factor V	R.NVMYFNGNSDASTIKENQFDPPIVAR.Y	3	4.20	0.31	
IPI00022937	Coagulation factor V	R.SEAYNTFSER.R	1	2.07	0.25	-0.42
IPI00022937	Coagulation factor V	R.SEAYNTFSER.R	2	3.13	0.34	-2.78
IPI00022937	Coagulation factor V	R.SGPESPGSACR.A	2	2.20	0.09	-2.05
IPI00022937	Coagulation factor V	R.SQHLDNFSNQIGK.H	2	4.35	0.44	-4.02
IPI00022937	Coagulation factor V	R.SQHLDNFSNQIGK.H	3	3.11	0.32	-3.00
IPI00022937	Coagulation factor V	R.SSSPELSEM*LEYDR.S	2	4.31	0.48	-5.08
IPI00022937	Coagulation factor V	R.TFHPLRSEAYNTFSER.R	2	3.28	0.35	-5.62
IPI00022937	Coagulation factor V	R.TFHPLRSEAYNTFSER.R	3	2.26	0.30	-3.67
IPI00022958	PRO0149	R.LPRFQMPNSR.I	2	1.65	0.20	-3.02
IPI00022959	Isoform 1 of Poliovirus receptor-related protein 3 precursor	K.CNADANPPPFK.S	2	2.98	0.21	-1.21
IPI00022959	Isoform 1 of Poliovirus receptor-related protein 3 precursor	K.SSQTVAVHHPQYGFSVQGEYQGR.V	3	5.23	0.48	-3.26
IPI00022959	Isoform 1 of Poliovirus receptor-related protein 3 precursor	K.SSQTVAVHHPQYGFSVQGEYQGR.V	4	2.80	0.20	-2.51
IPI00022959	Isoform 1 of Poliovirus receptor-related protein 3 precursor	K.VTNSLGQR.S	2	2.90	0.16	-3.69
IPI00022977	Creatine kinase B-type	K.DLFDPIIEDR.H	2	1.97	0.14	-4.28
IPI00022977	Creatine kinase B-type	R.GTGGVDTAAVGGVFDVSNADR.L	2	4.15	0.60	-3.61
IPI00022977	Creatine kinase B-type	R.LGFSEVELVQMVVDGVK.L	2	4.83	0.46	-2.45
IPI00022977	Creatine kinase B-type	R.LGFSEVELVQMVVDGVK.L	3	4.92	0.32	-1.24
IPI00022989	Isoform Beta-1 of Retinoic acid receptor beta	K.LQEPLLEALKIYIRK.R	2	2.30	0.13	
IPI00023014	von Willebrand factor precursor	K.LSGEAYGFVAR.I	2	3.76	0.38	-2.00
IPI00023014	von Willebrand factor precursor	K.LSPVYAGK.T	1	1.84	0.08	-1.81
IPI00023014	von Willebrand factor precursor	K.YAGSQVASTSEVLK.Y	2	3.58	0.34	-2.98
IPI00023014	von Willebrand factor precursor	K.YTLFQIFSK.I	2	2.74	0.29	-1.99
IPI00023014	von Willebrand factor precursor	R.DCNTCICR.N	2	2.60	0.24	-2.23
IPI00023014	von Willebrand factor precursor	R.IQHTVTASVR.L	2	1.71	0.06	-1.11
IPI00023014	von Willebrand factor precursor	R.LPGLHNSLVK.L	2	2.03	0.17	-2.30
IPI00023014	von Willebrand factor precursor	R.SFSIIGDFQNGKR.V	2	3.02	0.24	-2.82

IPI00023014	von Willebrand factor precursor	R.SFSIIGDFQNGKR.V	3	2.33	0.22	-1.93
IPI00023014	von Willebrand factor precursor	R.VSM*PYASK.G	2	2.29	0.16	-4.35
IPI00023019	Isoform 1 of Sex hormone-binding globulin precursor	K.QAAGSGHLLALGTPENPSWLSLHLQDQK.V	3	3.29	0.24	-1.22
IPI00023019	Isoform 1 of Sex hormone-binding globulin precursor	K.QAEISASAPTSLR.S	2	3.63	0.27	-2.63
IPI00023019	Isoform 1 of Sex hormone-binding globulin precursor	K.TSSSFEVR.T	2	2.25	0.15	2.60
IPI00023019	Isoform 1 of Sex hormone-binding globulin precursor	K.VVLSSGSGPGLDLPLVLGLPLQLK.L	2	5.25	0.56	-4.44
IPI00023019	Isoform 1 of Sex hormone-binding globulin precursor	K.VVLSSGSGPGLDLPLVLGLPLQLK.L	3	3.81	0.39	-4.42
IPI00023019	Isoform 1 of Sex hormone-binding globulin precursor	R.DSWLDKQAEISASAPTSLR.S	2	5.28	0.43	-3.30
IPI00023019	Isoform 1 of Sex hormone-binding globulin precursor	R.IALGGLLFPASNLR.L	2	4.17	0.41	-3.51
IPI00023019	Isoform 1 of Sex hormone-binding globulin precursor	R.IALGGLLFPASNLR.L	3	3.50	0.30	-2.78
IPI00023019	Isoform 1 of Sex hormone-binding globulin precursor	R.LFLGALPGEDSSTSFCLNGLWAQGQR.L	3	4.05	0.40	-7.59
IPI00023019	Isoform 1 of Sex hormone-binding globulin precursor	R.LRQVSGPLTSK.R	2	3.00	0.28	-3.44
IPI00023019	Isoform 1 of Sex hormone-binding globulin precursor	R.VVLSQGSK.M	1	1.79	0.22	-1.26
IPI00023087	Ubiquitin-conjugating enzyme E2 T	R.AQILGGANTPYEK.G	2	2.61	0.09	
IPI00023152	Isoform 1 of N-acetylated-alpha-linked acidic dipeptidase-like protein	-.MQWTKVLGLGLGAAALLGLGIILGHFAIPKK.A	3	1.88	0.18	-7.78
IPI00023162	UDP-N-acetylglucosamine 2-epimerase/N-acetylmannosamine kinase	R.M*IEQDDFDINTR.L	2	2.60	0.14	1.96
IPI00023184	Isoform 1 of Poly [ADP-ribose] polymerase 3	K.EMFKNTM*ALM*DLDVK.K	3	2.56	0.18	
IPI00023191	Target of myb1	K.AADRLPNLSSPSAEGPPGPPSGPAPR.K	3	4.67	0.37	-1.24
IPI00023191	Target of myb1	R.VLELIPQIANEQLTEELLIVNDNLNNVFLR.H	3	2.36	0.17	-2.20
IPI00023217	Isoform 1 of Ryanodine receptor 2	K.EAATPEEESDTLEK.E	1	3.39	0.08	
IPI00023315	Bone morphogenetic protein 3b precursor	R.YDPFPAGDPEPR.A	2	3.20	0.45	-3.39
IPI00023322	Zinc finger protein ubi-d4	K.TGQPEELVSCSDCGRSGHPSCLQFTPVMMAAVKTYR.W	4	3.03	0.11	-2.77
IPI00023340	Histone acetyltransferase MYST3	R.YSEGDRAVLRGFSESSEEEEEPESPRSSSPPILTKPTLK.R	4	2.40	0.12	-6.68
IPI00023359	Isoform 1 of Malonyl CoA-acyl carrier protein transacylase, mitochondrial precursor	K.KPLVSVYSNVHAHR.Y	3	1.68	0.11	-4.09
IPI00023407	Nck-associated protein 1-like	R.M*LDSVEKLLVETSDLSTFCFHLR.I	3	2.05	0.12	-6.97
IPI00023505	Low affinity immunoglobulin gamma Fc region receptor II-a precursor	K.VTFFQNGK.S	1	2.11	0.25	-3.18

IPI00023505	Low affinity immunoglobulin gamma Fc region receptor II-a precursor	K.VTFFQNGK.S	2	2.14	0.18	-2.48
IPI00023513	Isoform 1 of E3 ubiquitin-protein ligase CHFR	R.SSSCGSGGGGISPK.G	3	2.25	0.19	
IPI00023542	transmembrane emp24 protein transport domain containing 9	K.FSLFAGGM*LR.V	2	3.10	0.33	-1.65
IPI00023576	Leucine-rich repeat transmembrane neuronal protein 2 precursor	K.AIDLTVFETM*PNLK.I	2	2.71	0.33	-1.88
IPI00023576	Leucine-rich repeat transmembrane neuronal protein 2 precursor	K.ILLM*DNNKLNSLDSK.I	2	3.05	0.30	-1.05
IPI00023576	Leucine-rich repeat transmembrane neuronal protein 2 precursor	K.ILLM*DNNKLNSLDSK.I	3	2.86	0.32	-0.19
IPI00023576	Leucine-rich repeat transmembrane neuronal protein 2 precursor	K.INFAHFLR.L	2	2.85	0.10	-1.59
IPI00023576	Leucine-rich repeat transmembrane neuronal protein 2 precursor	R.SLEFLDLSTNR.L	2	3.40	0.32	-3.52
IPI00023598	Tubulin beta-4 chain	K.GHYTEGAELVDAVLDVVR.K	3	2.77	0.06	-1.45
IPI00023598	Tubulin beta-4 chain	K.MAATFIGNSTAIQELFK.R	2	4.57	0.37	-1.97
IPI00023598	Tubulin beta-4 chain	K.NM*M*AACDPR.H	2	1.73	0.12	0.11
IPI00023598	Tubulin beta-4 chain	K.VSDTVVEPYNATLSVHQLVENTDETYCIDNEALYDICFR.T	3	4.91	0.43	-2.95
IPI00023598	Tubulin beta-4 chain	K.VSDTVVEPYNATLSVHQLVENTDETYCIDNEALYDICFR.T	4	4.05	0.34	-2.05
IPI00023598	Tubulin beta-4 chain	R.ISEQFTAM*FR.R	2	2.17	0.13	-2.65
IPI00023598	Tubulin beta-4 chain	R.ISEQFTAMFR.R	2	2.87	0.27	-0.81
IPI00023598	Tubulin beta-4 chain	R.LHFFM*PGFAPLTSR.G	3	3.20	0.06	-3.00
IPI00023598	Tubulin beta-4 chain	R.LHFFMPGFAPLTSR.G	3	2.62	0.11	-1.73
IPI00023643	Sema domain, transmembrane domain (TM), and cytoplasmic domain, (Semaphorin) 6C	R.DLPDDVLTFIK.A	2	2.66	0.24	-3.16
IPI00023643	Sema domain, transmembrane domain (TM), and cytoplasmic domain, (Semaphorin) 6C	R.EVSVEDAR.L	2	1.77	0.09	-2.13
IPI00023643	Sema domain, transmembrane domain (TM), and cytoplasmic domain, (Semaphorin) 6C	R.RIIGLELDTEGHR.L	3	2.81	0.13	-1.79
IPI00023643	Sema domain, transmembrane domain (TM), and cytoplasmic domain, (Semaphorin) 6C	R.SGGPEPILLEEIDAYSPAR.C	2	4.42	0.45	-4.78
IPI00023648	Immunoglobulin superfamily containing leucine-rich repeat protein precursor	K.M*DSNELTFIPR.D	2	3.60	0.28	-1.90
IPI00023648	Immunoglobulin superfamily containing leucine-rich repeat protein precursor	K.TWALTTAVSIPEQDNIACTSPHVLK.G	3	2.76	0.16	-3.55
IPI00023648	Immunoglobulin superfamily containing leucine-rich repeat protein precursor	R.ALPGTPVASSQPR.F	1	2.59	0.33	-2.62
IPI00023648	Immunoglobulin superfamily containing leucine-rich repeat protein precursor	R.ALPGTPVASSQPR.F	2	2.84	0.34	-2.99
IPI00023648	Immunoglobulin superfamily containing leucine-rich repeat protein precursor	R.EVPLLQSLWLAHNEIR.T	2	4.17	0.51	-2.87

IPI00023648	Immunoglobulin superfamily containing leucine-rich repeat protein precursor	R.EVPLLQSLWLAHNEIR.T	3	1.99	0.33	-2.22
IPI00023648	Immunoglobulin superfamily containing leucine-rich repeat protein precursor	R.LPGLPEGAFR.E	1	1.95	0.33	-3.20
IPI00023648	Immunoglobulin superfamily containing leucine-rich repeat protein precursor	R.LPGLPEGAFR.E	2	2.64	0.18	-2.89
IPI00023648	Immunoglobulin superfamily containing leucine-rich repeat protein precursor	R.SLQLNHNR.L	1	1.76	0.08	-5.42
IPI00023648	Immunoglobulin superfamily containing leucine-rich repeat protein precursor	R.TVAAGALASLSHLK.S	2	4.84	0.46	-4.64
IPI00023648	Immunoglobulin superfamily containing leucine-rich repeat protein precursor	R.TVAAGALASLSHLK.S	3	1.90	0.18	-5.17
IPI00023673	Galectin-3-binding protein precursor	K.AVDTWSWGER.A	2	2.98	0.30	
IPI00023673	Galectin-3-binding protein precursor	K.KTLQALEFHTVPFQLLAR.Y	2	4.87	0.51	-3.87
IPI00023673	Galectin-3-binding protein precursor	K.KTLQALEFHTVPFQLLAR.Y	3	3.96	0.30	-3.92
IPI00023673	Galectin-3-binding protein precursor	K.KTLQALEFHTVPFQLLAR.Y	4	3.34	0.17	-3.07
IPI00023673	Galectin-3-binding protein precursor	K.LASAYGAR.Q	1	2.07	0.09	-1.65
IPI00023673	Galectin-3-binding protein precursor	K.LASAYGAR.Q	2	2.48	0.05	-4.31
IPI00023673	Galectin-3-binding protein precursor	K.SGGSDRTIAYENK.A	2	3.20	0.29	-2.58
IPI00023673	Galectin-3-binding protein precursor	K.SGGSDRTIAYENK.A	3	3.62	0.35	-2.24
IPI00023673	Galectin-3-binding protein precursor	K.SQLVYQSR.R	1	1.98	0.07	-1.38
IPI00023673	Galectin-3-binding protein precursor	K.SQLVYQSR.R	2	2.55	0.26	-0.75
IPI00023673	Galectin-3-binding protein precursor	K.TLQALEFHTVPFQLLAR.Y	2	6.11	0.54	-3.53
IPI00023673	Galectin-3-binding protein precursor	K.TLQALEFHTVPFQLLAR.Y	3	4.39	0.40	-3.58
IPI00023673	Galectin-3-binding protein precursor	K.YSSDYFQAPSDYR.Y	2	4.49	0.49	-3.86
IPI00023673	Galectin-3-binding protein precursor	K.YSSDYFQAPSDYRYYPYQSFQTPQHPSFLFQDKR.V	4	5.16	0.52	-5.23
IPI00023673	Galectin-3-binding protein precursor	R.AAFGQGSGPIM*LDEVQCTGTEASLADCK.S	2	5.17	0.62	-3.13
IPI00023673	Galectin-3-binding protein precursor	R.AAFGQGSGPIM*LDEVQCTGTEASLADCK.S	3	6.04	0.62	-4.96
IPI00023673	Galectin-3-binding protein precursor	R.ASHEEVEGLVEK.I	1	2.87	0.34	-3.16
IPI00023673	Galectin-3-binding protein precursor	R.ASHEEVEGLVEK.I	2	3.13	0.33	-3.08
IPI00023673	Galectin-3-binding protein precursor	R.ASHEEVEGLVEK.I	3	3.45	0.21	-2.07
IPI00023673	Galectin-3-binding protein precursor	R.ELSEALGQIFDSQR.G	2	3.95	0.36	-2.71
IPI00023673	Galectin-3-binding protein precursor	R.ELSEALGQIFDSQR.G	3	3.49	0.29	-2.22
IPI00023673	Galectin-3-binding protein precursor	R.GQWGTVCDNLWDLTDASVVCR.A	2	5.84	0.57	-7.94
IPI00023673	Galectin-3-binding protein precursor	R.GQWGTVCDNLWDLTDASVVCR.A	3	4.37	0.33	
IPI00023673	Galectin-3-binding protein precursor	R.IDITLSSVK.C	2	2.69	0.17	-2.41
IPI00023673	Galectin-3-binding protein precursor	R.IYTSPTWSAFVTDSSWSAR.K	2	6.06	0.60	-2.74
IPI00023673	Galectin-3-binding protein precursor	R.IYTSPTWSAFVTDSSWSAR.K	3	2.29	0.17	-3.03
IPI00023673	Galectin-3-binding protein precursor	R.KSQLVYQSR.R	1	2.55	0.23	-4.81
IPI00023673	Galectin-3-binding protein precursor	R.LADGGATNQGR.V	1	1.27	0.14	-2.28
IPI00023673	Galectin-3-binding protein precursor	R.LADGGATNQGR.V	2	3.89	0.47	-2.48
IPI00023673	Galectin-3-binding protein precursor	R.LADGGATNQGRVEIFYR.G	3	3.20	0.15	-2.51

IPI00023673	Galectin-3-binding protein precursor	R.RIDITLSSVK.C	1	2.71	0.31	-3.53
IPI00023673	Galectin-3-binding protein precursor	R.RIDITLSSVK.C	2	3.27	0.29	-3.83
IPI00023673	Galectin-3-binding protein precursor	R.SDLAVPSELALLK.A	1	2.68	0.30	-3.42
IPI00023673	Galectin-3-binding protein precursor	R.SDLAVPSELALLK.A	2	3.21	0.30	-3.94
IPI00023673	Galectin-3-binding protein precursor	R.SDLAVPSELALLKAVDTWSWGER.A	3	2.30	0.12	-3.49
IPI00023673	Galectin-3-binding protein precursor	R.STHTLDLSR.E	1	2.54	0.24	-5.06
IPI00023673	Galectin-3-binding protein precursor	R.STHTLDLSR.E	2	2.69	0.26	-2.61
IPI00023673	Galectin-3-binding protein precursor	R.STHTLDLSRELSEALGQIFDSQR.G	3	3.64	0.33	-5.74
IPI00023673	Galectin-3-binding protein precursor	R.TIAYENK.A	1	1.80	0.17	-3.13
IPI00023673	Galectin-3-binding protein precursor	R.TIAYENK.A	2	2.43	0.22	-5.47
IPI00023673	Galectin-3-binding protein precursor	R.YKGLNLTEDTYKPR.I	3	2.94	0.39	-3.12
IPI00023673	Galectin-3-binding protein precursor	R.YYPYQSFQTPQHPSFLFQDK.R	2	2.85	0.43	-0.83
IPI00023673	Galectin-3-binding protein precursor	R.YYPYQSFQTPQHPSFLFQDK.R	3	2.35	0.16	-2.55
IPI00023673	Galectin-3-binding protein precursor	R.YYPYQSFQTPQHPSFLFQDKR.V	2	4.73	0.47	-3.81
IPI00023673	Galectin-3-binding protein precursor	R.YYPYQSFQTPQHPSFLFQDKR.V	3	2.52	0.32	-4.52
IPI00023673	Galectin-3-binding protein precursor	Y.PYQSFQTPQHPSFLFQDKR.V	3	5.21	0.44	-3.12
IPI00023728	Gamma-glutamyl hydrolase precursor	K.FFNVLTTNTDGK.I	2	2.52	0.21	1.12
IPI00023728	Gamma-glutamyl hydrolase precursor	K.NLDGISHAPNAVK.T	2	3.69	0.34	-3.05
IPI00023728	Gamma-glutamyl hydrolase precursor	K.SINGILFPGGSVDLR.R	2	4.27	0.42	-4.38
IPI00023728	Gamma-glutamyl hydrolase precursor	K.TAFYLAEFFVNEAR.K	2	5.23	0.52	-5.24
IPI00023728	Gamma-glutamyl hydrolase precursor	K.TAFYLAEFFVNEAR.K	3	5.25	0.36	-4.35
IPI00023728	Gamma-glutamyl hydrolase precursor	K.YLESAGAR.V	1	2.25	0.16	-2.74
IPI00023728	Gamma-glutamyl hydrolase precursor	K.YLESAGAR.V	2	2.68	0.16	-2.70
IPI00023728	Gamma-glutamyl hydrolase precursor	R.LDLTEKDYEILFK.S	2	4.29	0.34	-3.55
IPI00023728	Gamma-glutamyl hydrolase precursor	R.LDLTEKDYEILFK.S	3	4.20	0.33	-2.24
IPI00023728	Gamma-glutamyl hydrolase precursor	R.M*FQNFPTELLLSLAVEPLTANFHK.W	3	6.09	0.52	-5.40
IPI00023728	Gamma-glutamyl hydrolase precursor	R.M*FQNFPTELLLSLAVEPLTANFHK.W	4	2.86	0.15	-3.89
IPI00023728	Gamma-glutamyl hydrolase precursor	R.VVPVRLDLTEKDYEILFK.S	2	3.75	0.44	-5.88
IPI00023728	Gamma-glutamyl hydrolase precursor	R.VVPVRLDLTEKDYEILFK.S	3	3.39	0.34	-4.03
IPI00023728	Gamma-glutamyl hydrolase precursor	R.YYIAASYVK.Y	1	2.33	0.28	-3.61
IPI00023728	Gamma-glutamyl hydrolase precursor	R.YYIAASYVK.Y	2	3.10	0.35	-2.25
IPI00023728	Gamma-glutamyl hydrolase precursor	S.LELSRPHGDTAK.K	2	3.27	0.35	-2.89
IPI00023728	Gamma-glutamyl hydrolase precursor	V.PVRLDLTEKDYEILFK.S	3	5.14	0.41	-3.74
IPI00023751	Growth/differentiation factor 8 precursor	K.ALDENGHDLAVTFPGPGEDGLNPFLEVK.V	3	4.27	0.39	-2.41
IPI00023751	Growth/differentiation factor 8 precursor	K.IPAM*VVDR.C	2	2.19	0.16	-3.12
IPI00023751	Growth/differentiation factor 8 precursor	K.M*SPINM*LYFNGK.E	2	2.92	0.40	-2.34
IPI00023751	Growth/differentiation factor 8 precursor	K.YPHTHLVHQANPR.G	2	3.04	0.40	-4.45
IPI00023751	Growth/differentiation factor 8 precursor	R.ELIDQYDVQR.D	2	3.33	0.21	-2.20
IPI00023754	Protein kinase C-binding protein NELL1 precursor	K.AFLFQDIER.E	2	3.04	0.24	-3.41
IPI00023754	Protein kinase C-binding protein NELL1 precursor	R.EIHAAPHVSEK.L	2	2.75	0.34	-4.30
IPI00023780	Isoform 2 of DnaJ homolog subfamily C member 5	K.EINNAHAILTDATKRNIYDK.Y	3	2.91	0.14	
IPI00023807	Semaphorin-4D precursor	K.DHPLM*DDSVTPIDNRPR.L	3	2.93	0.24	-2.54

IPI00023807	Semaphorin-4D precursor	K.YM*QSTTVEQSHTK.W	2	4.18	0.49	-4.39
IPI00023807	Semaphorin-4D precursor	K.YM*QSTTVEQSHTK.W	3	3.59	0.24	-1.27
IPI00023807	Semaphorin-4D precursor	R.FVYAGSNSGVVQAPLAFCGK.H	2	5.93	0.60	-4.09
IPI00023807	Semaphorin-4D precursor	R.TEYAIPWLNEPSFVFADVIR.K	2	4.40	0.53	-3.76
IPI00023807	Semaphorin-4D precursor	R.TEYAIPWLNEPSFVFADVIR.K	3	5.11	0.53	-4.59
IPI00023807	Semaphorin-4D precursor	R.TQALDGTVYDVM*FVSTDR.G	2	5.04	0.59	-4.36
IPI00023814	Isoform 1 of Neogenin precursor	E.PVDTLSVR.G	1	2.00	0.17	-2.29
IPI00023814	Isoform 1 of Neogenin precursor	K.DGTFLNLVSDDR.R	2	3.53	0.39	-2.57
IPI00023814	Isoform 1 of Neogenin precursor	K.DGTFLNLVSDDRR.Q	2	2.38	0.07	-3.19
IPI00023814	Isoform 1 of Neogenin precursor	K.DVTVVSK.E	1	2.35	0.20	-3.44
IPI00023814	Isoform 1 of Neogenin precursor	K.EHNLQVLGLVK.S	2	1.90	0.06	-1.83
IPI00023814	Isoform 1 of Neogenin precursor	K.GM*GPM*SEAVQFR.T	2	3.65	0.40	-0.84
IPI00023814	Isoform 1 of Neogenin precursor	K.GTDKEQDVDVSSHSYTINGLKK.Y	3	4.98	0.43	-4.55
IPI00023814	Isoform 1 of Neogenin precursor	K.GTDKEQDVDVSSHSYTINGLKK.Y	4	3.49	0.25	-1.44
IPI00023814	Isoform 1 of Neogenin precursor	K.HGSGESSAPLRVETQPEVQLPGPAPNLR.A	3	4.99	0.51	-4.82
IPI00023814	Isoform 1 of Neogenin precursor	K.IVKEHNLQVLGLVK.S	2	4.84	0.45	-4.98
IPI00023814	Isoform 1 of Neogenin precursor	K.IVKEHNLQVLGLVK.S	3	3.01	0.25	-3.47
IPI00023814	Isoform 1 of Neogenin precursor	K.KDGTFLNLVSDDRR.Q	3	3.32	0.11	-3.42
IPI00023814	Isoform 1 of Neogenin precursor	K.KYTEYSFR.V	2	2.05	0.15	-2.69
IPI00023814	Isoform 1 of Neogenin precursor	K.LIVAGLPR.F	1	1.93	0.10	-2.83
IPI00023814	Isoform 1 of Neogenin precursor	K.LIVAGLPR.F	2	2.47	0.17	-3.36
IPI00023814	Isoform 1 of Neogenin precursor	K.NEEALDTESSER.L	2	4.07	0.38	-2.80
IPI00023814	Isoform 1 of Neogenin precursor	K.NGDM*VIPSDYFK.I	1	2.89	0.33	-4.65
IPI00023814	Isoform 1 of Neogenin precursor	K.NGDM*VIPSDYFK.I	2	2.44	0.22	-5.15
IPI00023814	Isoform 1 of Neogenin precursor	K.SDEGFYQCIAENDVGNAQAGAQLIILEHAPATTGPLPSAPR.D	4	4.27	0.32	-5.46
IPI00023814	Isoform 1 of Neogenin precursor	K.SDVTETLVSGTQLSQLIEGLDR.G	3	4.76	0.47	-3.83
IPI00023814	Isoform 1 of Neogenin precursor	K.SDVTETLVSGTQLSQLIEGLDRGTEYNFR.V	3	4.62	0.41	-4.51
IPI00023814	Isoform 1 of Neogenin precursor	K.SIM*IHWQPPAPATQNGQITGYK.I	3	4.34	0.41	-2.92
IPI00023814	Isoform 1 of Neogenin precursor	K.VLPDPEVISDLVFLK.Q	2	4.11	0.41	-5.32
IPI00023814	Isoform 1 of Neogenin precursor	K.VLPDPEVISDLVFLK.Q	3	4.37	0.38	-3.99
IPI00023814	Isoform 1 of Neogenin precursor	K.VLPDPEVISDLVFLKQPSPLVR.V	2	4.28	0.54	-4.44
IPI00023814	Isoform 1 of Neogenin precursor	K.VLPDPEVISDLVFLKQPSPLVR.V	3	1.95	0.28	-3.26
IPI00023814	Isoform 1 of Neogenin precursor	K.VLPDPEVISDLVFLKQPSPLVR.V	4	4.57	0.43	-3.23
IPI00023814	Isoform 1 of Neogenin precursor	K.YSDEVELK.V	1	2.00	0.10	-4.14
IPI00023814	Isoform 1 of Neogenin precursor	K.YSDEVELK.V	2	2.96	0.23	-2.37
IPI00023814	Isoform 1 of Neogenin precursor	K.YTEYSFR.V	1	1.72	0.10	-2.71
IPI00023814	Isoform 1 of Neogenin precursor	K.YTEYSFR.V	2	2.08	0.19	-1.50
IPI00023814	Isoform 1 of Neogenin precursor	L.PDPEVISDLVFLKQPSPLVR.V	2	4.01	0.49	-4.21
IPI00023814	Isoform 1 of Neogenin precursor	L.PDPEVISDLVFLKQPSPLVR.V	3	4.36	0.47	-3.63
IPI00023814	Isoform 1 of Neogenin precursor	R.CVVESGGPPK.Y	1	2.35	0.19	-3.81
IPI00023814	Isoform 1 of Neogenin precursor	R.CVVESGGPPK.Y	2	3.18	0.36	-2.28
IPI00023814	Isoform 1 of Neogenin precursor	R.CVVESGGPPKYSDEVELK.V	2	4.17	0.33	-2.22

IPI00023814	Isoform 1 of Neogenin precursor	R.CVVESGGPPKYSDEVELK.V	3	2.34	0.20	-1.99
IPI00023814	Isoform 1 of Neogenin precursor	R.DVVASLVSTR.F	1	2.30	0.35	-3.26
IPI00023814	Isoform 1 of Neogenin precursor	R.DVVASLVSTR.F	2	3.59	0.31	-2.90
IPI00023814	Isoform 1 of Neogenin precursor	R.FTSQPEPSSVYAGNNAILNCEVNADLVPFVR.W	3	4.30	0.39	-4.46
IPI00023814	Isoform 1 of Neogenin precursor	R.GYAIGYGIGSPHAQTIK.V	2	5.45	0.52	-3.34
IPI00023814	Isoform 1 of Neogenin precursor	R.GYAIGYGIGSPHAQTIK.V	3	2.20	0.35	-2.62
IPI00023814	Isoform 1 of Neogenin precursor	R.GYAIGYGIGSPHAQTIKVDYK.Q	3	3.45	0.35	-2.96
IPI00023814	Isoform 1 of Neogenin precursor	R.GYAIGYGIGSPHAQTIKVDYK.Q	4	2.72	0.18	-2.86
IPI00023814	Isoform 1 of Neogenin precursor	R.ITWADNSLPK.H	2	2.60	0.21	-3.87
IPI00023814	Isoform 1 of Neogenin precursor	R.KSDVTETLVSGTQLSQLIEGLDR.G	2	5.11	0.40	-3.36
IPI00023814	Isoform 1 of Neogenin precursor	R.KSDVTETLVSGTQLSQLIEGLDR.G	3	4.52	0.39	-6.21
IPI00023814	Isoform 1 of Neogenin precursor	R.KSDVTETLVSGTQLSQLIEGLDRGTEYNFR.V	3	3.59	0.41	-1.80
IPI00023814	Isoform 1 of Neogenin precursor	R.KSDVTETLVSGTQLSQLIEGLDRGTEYNFR.V	4	4.38	0.36	-3.07
IPI00023814	Isoform 1 of Neogenin precursor	R.LPDLGSDYKPPM*SGSNSP.H	2	4.45	0.52	-3.94
IPI00023814	Isoform 1 of Neogenin precursor	R.LTHQIQELTLDTPYYFK.I	2	6.43	0.57	-3.38
IPI00023814	Isoform 1 of Neogenin precursor	R.LTHQIQELTLDTPYYFK.I	3	3.93	0.30	-3.58
IPI00023814	Isoform 1 of Neogenin precursor	R.QLLPDGSLFISNVVHSK.H	2	3.13	0.43	-3.50
IPI00023814	Isoform 1 of Neogenin precursor	R.QLLPDGSLFISNVVHSK.H	3	2.48	0.25	-2.26
IPI00023814	Isoform 1 of Neogenin precursor	R.QPLLLDDR.V	2	1.71	0.09	-2.79
IPI00023814	Isoform 1 of Neogenin precursor	R.RQLLPDGSLFISNVVHSK.H	3	5.28	0.40	-2.44
IPI00023814	Isoform 1 of Neogenin precursor	R.SGSAPQSPGASIR.T	2	3.35	0.26	-1.66
IPI00023814	Isoform 1 of Neogenin precursor	R.TFTPFYFLVEPVDTLSVR.G	2	6.26	0.52	-5.69
IPI00023814	Isoform 1 of Neogenin precursor	R.TFTPFYFLVEPVDTLSVR.G	3	5.24	0.39	-5.51
IPI00023814	Isoform 1 of Neogenin precursor	R.TLSDVPSAAPQNLSLEVR.N	2	4.52	0.44	-2.96
IPI00023814	Isoform 1 of Neogenin precursor	R.TPASDPHGDNLTYSVFYTK.E	3	3.53	0.39	-1.27
IPI00023814	Isoform 1 of Neogenin precursor	R.VENTSHPGEM*QVTIQNLM*PATVYIFR.V	3	4.03	0.16	-4.96
IPI00023814	Isoform 1 of Neogenin precursor	R.VIGQDVVLPCVASGLPTPTIK.W	2	4.68	0.41	-4.72
IPI00023814	Isoform 1 of Neogenin precursor	R.VIGQDVVLPCVASGLPTPTIK.W	3	5.05	0.42	-4.84
IPI00023814	Isoform 1 of Neogenin precursor	R.VVAYNKHGPGVSTPDVAVR.T	3	3.01	0.45	-3.45
IPI00023814	Isoform 1 of Neogenin precursor	R.YYTIENLDPSSHYVITLK.A	3	3.42	0.33	-1.63
IPI00023824	Fibulin-2 precursor	K.CVDVNECETGVHR.C	3	2.94	0.32	
IPI00023824	Fibulin-2 precursor	R.HCCVSYLQEK.S	2	3.09	0.40	
IPI00023824	Fibulin-2 precursor	R.IGPAPAFTGDTIALNIIK.G	2	3.50	0.41	
IPI00023824	Fibulin-2 precursor	R.IGPAPAFTGDTIALNIIKGNEEGYFGTR.R	3	4.56	0.41	
IPI00023824	Fibulin-2 precursor	R.RPPEPAAAPR.R	2	2.62	0.16	-2.06
IPI00023845	Kallikrein-6 precursor	A.DGDFPDTIQCAYIHLVSR.E	2	4.72	0.47	-2.82
IPI00023845	Kallikrein-6 precursor	A.EEQNKLVHGGPCDK.T	2	3.24	0.40	-4.34
IPI00023845	Kallikrein-6 precursor	F.PDTIQCAYIHLVSR.E	2	3.59	0.39	-2.63
IPI00023845	Kallikrein-6 precursor	H.PDYDAASHDQDIM*LLR.L	2	4.26	0.44	-1.96
IPI00023845	Kallikrein-6 precursor	K.DSCQGDSGGPLVCGDHLR.G	2	5.33	0.58	-3.83
IPI00023845	Kallikrein-6 precursor	K.DSCQGDSGGPLVCGDHLR.G	3	2.92	0.34	-3.76
IPI00023845	Kallikrein-6 precursor	K.EKPGVYTNVCR.Y	2	1.93	0.12	-4.02

IPI00023845	Kallikrein-6 precursor	K.KPNLQVFLGK.H	1	3.22	0.17	-4.37
IPI00023845	Kallikrein-6 precursor	K.KPNLQVFLGK.H	2	3.98	0.23	-4.38
IPI00023845	Kallikrein-6 precursor	K.LSELIQPLPLER.D	2	4.09	0.33	-4.44
IPI00023845	Kallikrein-6 precursor	K.LSELIQPLPLER.D	3	3.25	0.13	-3.01
IPI00023845	Kallikrein-6 precursor	K.LVHGGPCDK.T	2	2.13	0.10	-0.20
IPI00023845	Kallikrein-6 precursor	K.PNLQVFLGK.H	2	2.97	0.21	-0.89
IPI00023845	Kallikrein-6 precursor	K.TADGDFPDTIQCAYIHLVSR.E	2	4.27	0.53	-4.40
IPI00023845	Kallikrein-6 precursor	K.TADGDFPDTIQCAYIHLVSR.E	3	4.33	0.42	-5.07
IPI00023845	Kallikrein-6 precursor	K.TADGDFPDTIQCAYIHLVSREECEHAYPGQITQNM*LCAGDEK.Y	5	4.00	0.29	-2.40
IPI00023845	Kallikrein-6 precursor	K.TSHPYQAALYTSGH.L	2	3.80	0.50	-2.74
IPI00023845	Kallikrein-6 precursor	K.YGKDSCQGDSGGPLVCGDHLR.G	3	3.55	0.24	-5.33
IPI00023845	Kallikrein-6 precursor	Q.RESSQEQSSVVR.A	2	3.79	0.29	-0.78
IPI00023845	Kallikrein-6 precursor	R.AVIHPDYDAASHDQDIM*LLR.L	2	4.75	0.56	-4.57
IPI00023845	Kallikrein-6 precursor	R.AVIHPDYDAASHDQDIM*LLR.L	3	3.86	0.53	-5.53
IPI00023845	Kallikrein-6 precursor	R.AVIHPDYDAASHDQDIM*LLR.L	4	3.80	0.46	-3.90
IPI00023845	Kallikrein-6 precursor	R.EECEHAYPGQITQN.M	2	4.13	0.45	-3.10
IPI00023845	Kallikrein-6 precursor	R.EECEHAYPGQITQNM*LCAGDEK.Y	2	4.63	0.57	-2.55
IPI00023845	Kallikrein-6 precursor	R.EECEHAYPGQITQNM*LCAGDEK.Y	3	6.86	0.55	-3.04
IPI00023845	Kallikrein-6 precursor	R.ESSQEQSSVVR.A	1	1.78	0.08	-2.63
IPI00023845	Kallikrein-6 precursor	R.ESSQEQSSVVR.A	2	3.24	0.36	-3.98
IPI00023845	Kallikrein-6 precursor	R.GLVSWGNIPCGSK.E	1	2.59	0.34	-3.02
IPI00023845	Kallikrein-6 precursor	R.GLVSWGNIPCGSK.E	2	4.10	0.43	-3.27
IPI00023845	Kallikrein-6 precursor	R.QRESSQEQSSVVR.A	1	2.29	0.07	-1.63
IPI00023845	Kallikrein-6 precursor	R.QRESSQEQSSVVR.A	2	1.95	0.25	-3.66
IPI00023845	Kallikrein-6 precursor	R.QRESSQEQSSVVR.A	3	3.83	0.21	-1.86
IPI00023845	Kallikrein-6 precursor	R.YTNWIQK.T	1	2.47	0.18	-2.44
IPI00023845	Kallikrein-6 precursor	R.YTNWIQK.T	2	2.48	0.13	-1.97
IPI00023858	Fc-gamma receptor IIIb	K.DSGSYFCR.G	2	1.98	0.05	-2.95
IPI00023858	Fc-gamma receptor IIIb	K.VTYLQNGK.D	2	2.29	0.12	-0.35
IPI00023858	Fc-gamma receptor IIIb	K.VTYLQNGKDR.K	2	2.41	0.16	-2.52
IPI00023858	Fc-gamma receptor IIIb	S.VLEKDSVTLK.C	2	3.52	0.36	-3.30
IPI00023942	Isoform 2 of Syndecan-3	K.GARPGPGLLDNAIDSGSSAA.Q	2	3.79	0.33	-2.33
IPI00023942	Isoform 2 of Syndecan-3	K.GARPGPGLLDNAIDSGSSAAQLPQK.S	3	3.26	0.26	-1.83
IPI00024012	Frizzled-7 precursor	K.FGFQWPER.L	2	2.14	0.21	-1.54
IPI00024032	TBC1 domain family, member 29	R.VLNDGISLGLTPCLWDM*YLLEG.E	2	3.16	0.21	-0.96
IPI00024034	Cadherin-4 precursor	K.AGFSEDDYTALISQNILEGEK.L	2	5.91	0.62	-3.95
IPI00024034	Cadherin-4 precursor	K.AGFSEDDYTALISQNILEGEK.L	3	5.36	0.36	-2.88
IPI00024034	Cadherin-4 precursor	K.AGFSEDDYTALISQNILEGEKLLQVK.F	3	6.34	0.57	-4.33
IPI00024034	Cadherin-4 precursor	K.GTQYETNSM*DFK.V	2	3.71	0.43	-3.11
IPI00024034	Cadherin-4 precursor	K.VGADGTVFATR.E	1	2.30	0.27	-2.85
IPI00024034	Cadherin-4 precursor	K.VGADGTVFATR.E	2	3.53	0.38	-4.15
IPI00024034	Cadherin-4 precursor	R.IRSDKDNDIPIR.Y	2	2.93	0.16	-3.89

IPI00024034	Cadherin-4 precursor	R.LLVAQTSSPHSGHKPQK.G	2	4.31	0.53	-3.90
IPI00024034	Cadherin-4 precursor	R.LLVAQTSSPHSGHKPQK.G	3	2.47	0.15	-3.50
IPI00024035	Isoform 1 of Cadherin-6 precursor	R.FLYLGPFK.D	2	1.56	0.08	-1.53
IPI00024035	Isoform 1 of Cadherin-6 precursor	R.IVVEDVDEPPVFSK.L	2	3.55	0.33	-3.26
IPI00024035	Isoform 1 of Cadherin-6 precursor	R.TSGFPAKK.R	2	1.79	0.14	-3.98
IPI00024036	Cadherin-8 precursor	K.LVYSILEGQPYFSIEPETAIIK.T	2	4.55	0.46	-4.63
IPI00024036	Cadherin-8 precursor	K.LVYSILEGQPYFSIEPETAIIK.T	3	5.00	0.45	-4.44
IPI00024036	Cadherin-8 precursor	K.VEAANVHIDPR.F	3	1.79	0.16	-3.49
IPI00024036	Cadherin-8 precursor	R.DPDITSSPIR.F	2	3.41	0.30	-3.04
IPI00024036	Cadherin-8 precursor	R.QFNINADDGKITLATPLDR.E	3	4.04	0.31	-3.44
IPI00024046	Cadherin-13 precursor	A.EDLDCTPGFQQK.V	2	3.87	0.35	-5.19
IPI00024046	Cadherin-13 precursor	K.DIQGSLQDIFK.F	1	3.44	0.23	-3.90
IPI00024046	Cadherin-13 precursor	K.DIQGSLQDIFK.F	2	4.03	0.21	-5.23
IPI00024046	Cadherin-13 precursor	K.LRYEVSSPYFK.V	2	3.22	0.35	-2.48
IPI00024046	Cadherin-13 precursor	K.LRYEVSSPYFK.V	3	2.83	0.08	-2.80
IPI00024046	Cadherin-13 precursor	K.TLEGPVPLEVIVIDQNDNRPIFR.E	2	2.97	0.23	-2.11
IPI00024046	Cadherin-13 precursor	K.TLEGPVPLEVIVIDQNDNRPIFR.E	3	2.84	0.32	-3.78
IPI00024046	Cadherin-13 precursor	K.TLEGPVPLEVIVIDQNDNRPIFR.E	4	5.51	0.35	-2.49
IPI00024046	Cadherin-13 precursor	K.TLFVHAR.T	1	1.60	0.08	-3.31
IPI00024046	Cadherin-13 precursor	K.TLFVHAR.T	2	2.24	0.20	-4.75
IPI00024046	Cadherin-13 precursor	K.VNSDGGLVALR.N	1	2.52	0.29	-3.11
IPI00024046	Cadherin-13 precursor	K.VNSDGGLVALR.N	2	4.21	0.30	-2.96
IPI00024046	Cadherin-13 precursor	R.DVGKVVDSDRPER.S	2	2.78	0.23	-1.70
IPI00024046	Cadherin-13 precursor	R.DVGKVVDSDRPER.S	3	2.55	0.16	-1.96
IPI00024046	Cadherin-13 precursor	R.INENTGSVSVTR.T	1	2.15	0.27	-4.66
IPI00024046	Cadherin-13 precursor	R.INENTGSVSVTR.T	2	4.20	0.39	-3.15
IPI00024046	Cadherin-13 precursor	R.M*TAFDADDPATDNALLR.Y	2	5.95	0.58	-3.86
IPI00024046	Cadherin-13 precursor	R.QQTPDKPSPNM*FYIDPEKGDIVTVVSPALLDR.E	3	3.70	0.42	-4.74
IPI00024046	Cadherin-13 precursor	R.QQTPDKPSPNM*FYIDPEKGDIVTVVSPALLDR.E	4	5.12	0.35	-2.58
IPI00024046	Cadherin-13 precursor	R.SIVVSPILIPENQR.Q	2	3.84	0.40	-4.39
IPI00024046	Cadherin-13 precursor	R.TLDREVIAVYQLFVETTDVNGK.T	3	2.98	0.24	-5.01
IPI00024046	Cadherin-13 precursor	R.TLDREVIAVYQLFVETTDVNGK.T	4	4.93	0.46	-2.40
IPI00024046	Cadherin-13 precursor	R.TPHAEDM*AELVIVGGK.D	2	3.98	0.38	-5.29
IPI00024046	Cadherin-13 precursor	R.TPHAEDM*AELVIVGGK.D	3	4.07	0.28	-2.96
IPI00024046	Cadherin-13 precursor	R.TPHAEDM*AELVIVGGKDIQGSLQDIFK.F	3	3.34	0.29	-2.36
IPI00024046	Cadherin-13 precursor	R.TPHAEDM*AELVIVGGKDIQGSLQDIFK.F	4	4.13	0.35	-4.10
IPI00024046	Cadherin-13 precursor	R.YEVSSPYFK.V	1	2.13	0.20	-3.98
IPI00024046	Cadherin-13 precursor	R.YEVSSPYFK.V	2	2.91	0.34	-4.88
IPI00024048	Cadherin-15 precursor	K.TNEGVLSIVK.A	2	2.73	0.21	-1.97
IPI00024048	Cadherin-15 precursor	K.VSVQNEAPLQAAALR.A	2	3.85	0.16	
IPI00024048	Cadherin-15 precursor	R.DPDTEQLQR.L	2	3.37	0.14	-2.61
IPI00024048	Cadherin-15 precursor	R.FSILQQGSPELFSIDELTGEIR.T	2	5.67	0.40	

IPI00024048	Cadherin-15 precursor	R.FSILQQGSPELFSIDELTGEIR.T	3	5.00	0.38	
IPI00024048	Cadherin-15 precursor	R.VLEGAVPGTYVTR.A	2	3.09	0.06	-3.34
IPI00024067	Isoform 1 of Clathrin heavy chain 1	K.WISLNTVALVTDNAVYHWSMEGESQPVK.M	3	2.49	0.14	-2.44
IPI00024067	Isoform 1 of Clathrin heavy chain 1	R.LDNYDAPDIANIAISNELFEEAFAIFR.K	2	2.59	0.49	-2.27
IPI00024067	Isoform 1 of Clathrin heavy chain 1	R.LDNYDAPDIANIAISNELFEEAFAIFR.K	3	4.38	0.44	-0.11
IPI00024094	Rhesus blood group-associated glycoprotein	K.NM*INADFSAATVLISFGAVLGK.T	3	3.24	0.30	-4.69
IPI00024094	Rhesus blood group-associated glycoprotein	K.NMINADFSAATVLISFGAVLGK.T	2	6.51	0.51	-5.76
IPI00024094	Rhesus blood group-associated glycoprotein	K.NMINADFSAATVLISFGAVLGK.T	3	5.32	0.42	-5.16
IPI00024094	Rhesus blood group-associated glycoprotein	M.QAAALGSSIGTAVVGGLMTGLILK.L	2	4.71	0.54	-4.25
IPI00024094	Rhesus blood group-associated glycoprotein	M.QAAALGSSIGTAVVGGLMTGLILK.L	3	4.33	0.42	-4.84
IPI00024094	Rhesus blood group-associated glycoprotein	R.IHDTCGVHNLHGLPGVVGGLAGIVAVAMGASNTSMAM.Q	3	4.63	0.51	-4.67
IPI00024105	Complement C1q tumor necrosis factor-related protein 5 precursor	R.ASLQFDLVK.N	2	3.18	0.17	-1.60
IPI00024105	Complement C1q tumor necrosis factor-related protein 5 precursor	R.VLVNEQGHYDAVTGK.F	2	4.35	0.42	-2.49
IPI00024105	Complement C1q tumor necrosis factor-related protein 5 precursor	R.VPPPSDAPLPFDR.V	2	2.15	0.30	-3.34
IPI00024107	Isoform 1 of Alpha-synuclein	K.AKEGVVAAAEK.T	2	2.05	0.05	2.33
IPI00024129	Peptidyl-prolyl cis-trans isomerase C	K.GYGYK.G	1	1.35	0.11	-2.39
IPI00024129	Peptidyl-prolyl cis-trans isomerase C	K.TVENFVALATGEK.G	2	4.40	0.43	-4.10
IPI00024129	Peptidyl-prolyl cis-trans isomerase C	K.TVENFVALATGEK.G	3	4.03	0.22	-3.39
IPI00024129	Peptidyl-prolyl cis-trans isomerase C	R.IGDKDVGR.I	2	2.67	0.18	-3.83
IPI00024129	Peptidyl-prolyl cis-trans isomerase C	R.VIKDFM*IQGGDITTGDGTGGVSIYGETFPDENFK.L	3	6.32	0.57	-5.92
IPI00024138	Uncharacterized protein ENSP00000374816	R.LLIYGASTR.A	2	3.10	0.20	
IPI00024248	Sodium/iodide cotransporter	K.KPPGFLPTNEDRLFFLGQK.E	4	2.29	0.13	-1.67
IPI00024253	Isoform 1 of Fibroblast growth factor 14	K.FKESVFENYYVIYSSM*LYR.Q	2	2.92	0.06	-6.76
IPI00024272	Integral membrane protein DGCR2/IDD precursor	R.GGDPSHFHAVNVAQPVR.F	3	3.47	0.24	-2.85
IPI00024273	Isoform Long of Very low-density lipoprotein receptor precursor	K.FLFNSDLREPASIAVDPLSGFVYWSDWGEPAK.I	3	5.45	0.55	-3.97
IPI00024273	Isoform Long of Very low-density lipoprotein receptor precursor	K.FTGSELATLVNNLNDAQDIIVYHELVQPSGK.N	3	4.31	0.45	-5.90
IPI00024273	Isoform Long of Very low-density lipoprotein receptor precursor	K.SLEFLAHPLALTIFEDR.V	2	2.96	0.37	-1.31
IPI00024273	Isoform Long of Very low-density lipoprotein receptor precursor	K.SLEFLAHPLALTIFEDR.V	3	3.70	0.34	-2.71
IPI00024284	Basement membrane-specific heparan sulfate proteoglycan core protein precursor	K.AFAHLQVPER.V	2	2.24	0.31	-2.01
IPI00024284	Basement membrane-specific heparan sulfate proteoglycan core protein precursor	K.AGFFGDAM*K.A	2	2.52	0.18	-2.88
IPI00024284	Basement membrane-specific heparan sulfate proteoglycan core protein precursor	K.AGLSSGFIGCVR.E	2	3.74	0.44	-4.16

IPI00024284	Basement membrane-specific heparan sulfate proteoglycan core protein precursor	K.AVTLECVSAGEPR.S	2	3.29	0.26	-3.33
IPI00024284	Basement membrane-specific heparan sulfate proteoglycan core protein precursor	K.DFISLGLQDGHLVFR.Y	2	4.72	0.49	-3.06
IPI00024284	Basement membrane-specific heparan sulfate proteoglycan core protein precursor	K.DFISLGLQDGHLVFR.Y	3	3.56	0.33	-3.71
IPI00024284	Basement membrane-specific heparan sulfate proteoglycan core protein precursor	K.ESDQGAYTCEAM*NAR.G	2	4.32	0.57	-4.12
IPI00024284	Basement membrane-specific heparan sulfate proteoglycan core protein precursor	K.FQGLDLNEELYLGGYPDYGAIPK.A	2	4.36	0.39	-4.30
IPI00024284	Basement membrane-specific heparan sulfate proteoglycan core protein precursor	K.GSVYIGGAPDVATLTGGR.F	2	5.28	0.50	-2.65
IPI00024284	Basement membrane-specific heparan sulfate proteoglycan core protein precursor	K.IPGDQVVSVVFIK.E	2	3.90	0.47	-2.71
IPI00024284	Basement membrane-specific heparan sulfate proteoglycan core protein precursor	K.ITFRPDSADGM*LLYNGQK.R	3	4.02	0.19	-2.35
IPI00024284	Basement membrane-specific heparan sulfate proteoglycan core protein precursor	K.LDGSLPPDSR.L	2	2.21	0.13	-1.87
IPI00024284	Basement membrane-specific heparan sulfate proteoglycan core protein precursor	K.M*ASVGLSDIAM*DTTVTHATSHGR.A	4	4.63	0.45	-3.34
IPI00024284	Basement membrane-specific heparan sulfate proteoglycan core protein precursor	K.NLVLHSARPGAPPPQPLDLQHR.A	3	5.72	0.46	-5.00
IPI00024284	Basement membrane-specific heparan sulfate proteoglycan core protein precursor	K.NLVLHSARPGAPPPQPLDLQHR.A	4	3.07	0.08	-3.71
IPI00024284	Basement membrane-specific heparan sulfate proteoglycan core protein precursor	K.SGPVEDFVSLAM*VGGHLEFR.Y	3	2.96	0.41	-2.84
IPI00024284	Basement membrane-specific heparan sulfate proteoglycan core protein precursor	K.SPAYTLVWTR.L	2	3.73	0.49	-1.90
IPI00024284	Basement membrane-specific heparan sulfate proteoglycan core protein precursor	K.VDSYGGSLR.Y	2	2.14	0.22	-1.96
IPI00024284	Basement membrane-specific heparan sulfate proteoglycan core protein precursor	K.VGGHLRPGIVQSGGVVR.I	2	2.94	0.34	-4.64
IPI00024284	Basement membrane-specific heparan sulfate proteoglycan core protein precursor	K.VGGHLRPGIVQSGGVVR.I	3	3.68	0.23	-3.50
IPI00024284	Basement membrane-specific heparan sulfate proteoglycan core protein precursor	N.DAPGQYGAYFHDDGFLAFPGHVFSR.S	3	5.78	0.52	-4.18
IPI00024284	Basement membrane-specific heparan sulfate proteoglycan core protein precursor	R.AELLVTEAPSKPITVTVEEQR.S	3	5.59	0.53	-2.04
IPI00024284	Basement membrane-specific heparan sulfate proteoglycan core protein precursor	R.AM*DFNGILTIR.N	2	3.32	0.34	-1.98
IPI00024284	Basement membrane-specific heparan sulfate proteoglycan core protein precursor	R.AQLHGASEEPGHFSLTNAASTHTTNEGIFSPTPGELGFSSFHR.L	4	8.60	0.60	-2.67

IPI00024284	Basement membrane-specific heparan sulfate proteoglycan core protein precursor	R.AQLHGASEEPGHFSLTNAASTHTTNEGIFSPTPGELGFSSFHR.L	5	4.79	0.34	-4.77
IPI00024284	Basement membrane-specific heparan sulfate proteoglycan core protein precursor	R.ASYAQQPAESR.V	2	3.95	0.39	-2.09
IPI00024284	Basement membrane-specific heparan sulfate proteoglycan core protein precursor	R.AVLHVHGGGGPR.V	2	2.34	0.28	-3.89
IPI00024284	Basement membrane-specific heparan sulfate proteoglycan core protein precursor	R.CLCLPGFSGPR.C	2	2.47	0.25	-3.15
IPI00024284	Basement membrane-specific heparan sulfate proteoglycan core protein precursor	R.CVASNAYGVAQSVVNLSVHGPPTVSVLPEGPVWVK.V	3	4.60	0.40	-3.17
IPI00024284	Basement membrane-specific heparan sulfate proteoglycan core protein precursor	R.EDGRPVPSGTQQR.H	2	1.78	0.10	-3.02
IPI00024284	Basement membrane-specific heparan sulfate proteoglycan core protein precursor	R.EDGRPVPSGTQQR.H	3	2.09	0.19	-2.49
IPI00024284	Basement membrane-specific heparan sulfate proteoglycan core protein precursor	R.EHLLM*ALADLDELLIR.A	3	3.69	0.27	-3.24
IPI00024284	Basement membrane-specific heparan sulfate proteoglycan core protein precursor	R.EHLLM*ALAGIDTLLIR.A	3	3.12	0.16	-1.50
IPI00024284	Basement membrane-specific heparan sulfate proteoglycan core protein precursor	R.FDAGSGM*ATIR.H	2	3.64	0.35	-2.43
IPI00024284	Basement membrane-specific heparan sulfate proteoglycan core protein precursor	R.FLGDKVTSYGGELR.F	2	4.18	0.41	-2.61
IPI00024284	Basement membrane-specific heparan sulfate proteoglycan core protein precursor	R.FLGDKVTSYGGELR.F	3	3.75	0.36	-2.31
IPI00024284	Basement membrane-specific heparan sulfate proteoglycan core protein precursor	R.FSSGITGCVK.N	2	2.37	0.22	-0.06
IPI00024284	Basement membrane-specific heparan sulfate proteoglycan core protein precursor	R.GHTPTQPGALNQR.Q	2	3.14	0.44	-3.64
IPI00024284	Basement membrane-specific heparan sulfate proteoglycan core protein precursor	R.GHTPTQPGALNQR.Q	3	3.77	0.25	-3.85
IPI00024284	Basement membrane-specific heparan sulfate proteoglycan core protein precursor	R.GM*LEPVQRPDVVLVGAGYR.L	3	4.84	0.51	-2.53
IPI00024284	Basement membrane-specific heparan sulfate proteoglycan core protein precursor	R.GM*VFGIPDGVLELVPQR.G	2	3.76	0.46	-4.76
IPI00024284	Basement membrane-specific heparan sulfate proteoglycan core protein precursor	R.GSIQVDGEELVSGR.S	2	4.28	0.50	-3.39
IPI00024284	Basement membrane-specific heparan sulfate proteoglycan core protein precursor	R.HLISTHFAPGDFQGFALVNPQR.N	3	4.78	0.47	-3.52
IPI00024284	Basement membrane-specific heparan sulfate proteoglycan core protein precursor	R.IAHVELADAGQYR.C	2	3.39	0.35	-2.44
IPI00024284	Basement membrane-specific heparan sulfate proteoglycan core protein precursor	R.IAHVELADAGQYR.C	3	4.36	0.30	-1.10

IPI00024284	Basement membrane-specific heparan sulfate proteoglycan core protein precursor	R.ISSTPAKLEQR.T	2	2.53	0.20	-2.72
IPI00024284	Basement membrane-specific heparan sulfate proteoglycan core protein precursor	R.LDVEFKPLAPDGVLLFSGGK.S	2	5.51	0.53	-4.44
IPI00024284	Basement membrane-specific heparan sulfate proteoglycan core protein precursor	R.LDVEFKPLAPDGVLLFSGGK.S	3	5.30	0.54	-3.86
IPI00024284	Basement membrane-specific heparan sulfate proteoglycan core protein precursor	R.LEGDTLIIPR.V	2	3.00	0.16	-2.33
IPI00024284	Basement membrane-specific heparan sulfate proteoglycan core protein precursor	R.LENNM*LM*LPSVRPQDAGTYVCTATNR.Q	3	3.83	0.37	-3.30
IPI00024284	Basement membrane-specific heparan sulfate proteoglycan core protein precursor	R.LLQVTPADSGEYVCR.V	2	3.57	0.44	-2.95
IPI00024284	Basement membrane-specific heparan sulfate proteoglycan core protein precursor	R.LLSGPYFWSLPSR.F	2	3.88	0.38	-4.33
IPI00024284	Basement membrane-specific heparan sulfate proteoglycan core protein precursor	R.LRSPVISIDPPSSTVQQGQDASFK.C	3	5.31	0.42	-3.84
IPI00024284	Basement membrane-specific heparan sulfate proteoglycan core protein precursor	R.LSGSHSQGVAYPVR.I	2	3.88	0.48	-2.30
IPI00024284	Basement membrane-specific heparan sulfate proteoglycan core protein precursor	R.LSGSHSQGVAYPVR.I	3	3.35	0.13	-2.10
IPI00024284	Basement membrane-specific heparan sulfate proteoglycan core protein precursor	R.LYIFQASPADAGQYVCR.A	2	5.18	0.57	-3.13
IPI00024284	Basement membrane-specific heparan sulfate proteoglycan core protein precursor	R.PGAPPPQPLDLQHR.A	3	4.11	0.39	-1.39
IPI00024284	Basement membrane-specific heparan sulfate proteoglycan core protein precursor	R.QPDFISFGLVGGRPEFR.F	3	2.32	0.33	-1.62
IPI00024284	Basement membrane-specific heparan sulfate proteoglycan core protein precursor	R.RGSIQVDGEELVSGR.S	2	4.64	0.44	-3.60
IPI00024284	Basement membrane-specific heparan sulfate proteoglycan core protein precursor	R.RGSIQVDGEELVSGR.S	3	3.56	0.31	-3.32
IPI00024284	Basement membrane-specific heparan sulfate proteoglycan core protein precursor	R.SIEYSPQLEDAGSR.E	2	3.42	0.45	-4.93
IPI00024284	Basement membrane-specific heparan sulfate proteoglycan core protein precursor	R.SIVPQGGSHSLR.C	2	2.13	0.15	-2.41
IPI00024284	Basement membrane-specific heparan sulfate proteoglycan core protein precursor	R.SLPEVPETIELEVR.T	2	3.41	0.37	-5.17
IPI00024284	Basement membrane-specific heparan sulfate proteoglycan core protein precursor	R.SPGPNVAVNAK.G	1	2.65	0.16	-1.28
IPI00024284	Basement membrane-specific heparan sulfate proteoglycan core protein precursor	R.SPGPNVAVNAK.G	2	3.53	0.43	-1.17
IPI00024284	Basement membrane-specific heparan sulfate proteoglycan core protein precursor	R.SPVISIDPPSSTVQQGQDASFK.C	2	5.51	0.56	-3.46

IPI00024284	Basement membrane-specific heparan sulfate proteoglycan core protein precursor	R.SPVISIDPPSSTVQQGQDASFK.C	3	3.91	0.29	-4.24
IPI00024284	Basement membrane-specific heparan sulfate proteoglycan core protein precursor	R.SQPGSTPLHGQPLVVLQGNNIILEHHVAQEPSPGQPSTFIVPFR.E	4	5.50	0.55	-3.47
IPI00024284	Basement membrane-specific heparan sulfate proteoglycan core protein precursor	R.SQSVRPGADVTFICTAK.S	3	3.52	0.32	-1.83
IPI00024284	Basement membrane-specific heparan sulfate proteoglycan core protein precursor	R.SYEIM*FR.E	2	1.72	0.09	-1.70
IPI00024284	Basement membrane-specific heparan sulfate proteoglycan core protein precursor	R.TCESLGAGGYR.C	2	2.97	0.30	-1.66
IPI00024284	Basement membrane-specific heparan sulfate proteoglycan core protein precursor	R.TPSGLYLGTCER.C	2	3.85	0.41	-2.37
IPI00024284	Basement membrane-specific heparan sulfate proteoglycan core protein precursor	R.VAEGQTLDLK.C	2	3.07	0.23	-2.28
IPI00024284	Basement membrane-specific heparan sulfate proteoglycan core protein precursor	R.VTVTSEGGR.G	2	2.85	0.22	-2.82
IPI00024284	Basement membrane-specific heparan sulfate proteoglycan core protein precursor	R.VVPYFTQTPYSFLPLPTIK.D	2	4.42	0.52	-5.01
IPI00024284	Basement membrane-specific heparan sulfate proteoglycan core protein precursor	R.VVPYFTQTPYSFLPLPTIK.D	3	4.10	0.35	-4.56
IPI00024284	Basement membrane-specific heparan sulfate proteoglycan core protein precursor	R.YELGSGLAVLR.S	1	1.57	0.13	-2.30
IPI00024284	Basement membrane-specific heparan sulfate proteoglycan core protein precursor	R.YELGSGLAVLR.S	2	3.18	0.25	-3.22
IPI00024284	Basement membrane-specific heparan sulfate proteoglycan core protein precursor	R.YQLGSGEAR.L	1	2.01	0.23	-3.10
IPI00024284	Basement membrane-specific heparan sulfate proteoglycan core protein precursor	R.YQLGSGEAR.L	2	3.05	0.24	-4.65
IPI00024307	Ephrin-B1 precursor	K.FQEFSPNYM*GLEFK.K	2	4.36	0.44	-5.43
IPI00024307	Ephrin-B1 precursor	K.NLEPVSWSSLNPK.F	2	2.95	0.14	-3.57
IPI00024346	snRNA-activating protein complex subunit 3	R.EDAAVAR.D	1	1.86	0.13	
IPI00024466	UDP-glucose ceramide glucosyltransferase-like 1 isoform 1	K.ILFLDVLFPLVVDK.F	2	2.15	0.18	-3.88
IPI00024466	UDP-glucose ceramide glucosyltransferase-like 1 isoform 1	R.IIGPLEDSELFNQDDFHLLENIILK.T	3	4.30	0.37	-4.40
IPI00024502	Ubiquilin-4	R.M*YTDIQEPMFSAAREQFGNNPFSSLAGNSDSSSSQPLR.T	3	2.00	0.11	2.96
IPI00024570	Semaphorin-3G precursor	K.DYPDEVLQFAR.A	2	3.72	0.37	-3.88
IPI00024570	Semaphorin-3G precursor	K.M*TAQPGRPFGSTK.D	2	3.51	0.30	-0.21
IPI00024570	Semaphorin-3G precursor	K.M*TAQPGRPFGSTKDYPDEVLQFAR.A	3	4.06	0.42	-4.06
IPI00024570	Semaphorin-3G precursor	K.M*TAQPGRPFGSTKDYPDEVLQFAR.A	4	2.91	0.38	-3.84
IPI00024570	Semaphorin-3G precursor	K.VIALQAGGSAEPEEVVLEELQVFK.V	2	4.69	0.53	-4.62
IPI00024570	Semaphorin-3G precursor	K.VIALQAGGSAEPEEVVLEELQVFK.V	3	4.26	0.28	-4.63

IPI00024570	Semaphorin-3G precursor	K.VPTPITEM*EISVKR.Q	3	2.43	0.31	-1.28
IPI00024570	Semaphorin-3G precursor	R.GEHVLHLEPGSVESGR.G	2	4.83	0.37	-3.97
IPI00024570	Semaphorin-3G precursor	R.IPENSDQDNDKVYFFFSETVPSPDGGSNHVTVSR.V	3	5.85	0.49	-1.58
IPI00024570	Semaphorin-3G precursor	R.IPENSDQDNDKVYFFFSETVPSPDGGSNHVTVSR.V	4	4.92	0.36	-3.25
IPI00024570	Semaphorin-3G precursor	R.LDQAWPDPR.E	2	2.07	0.05	-3.16
IPI00024570	Semaphorin-3G precursor	R.LFLGGLDALYSLR.L	2	4.41	0.39	-3.94
IPI00024570	Semaphorin-3G precursor	R.LFLGGLDALYSLR.L	3	3.22	0.08	-2.68
IPI00024570	Semaphorin-3G precursor	R.LVCSVPGPGGAETHFDQLEDVFLLWPK.A	3	2.64	0.15	-4.01
IPI00024570	Semaphorin-3G precursor	R.SAIFLGPQGSLNLQAM*YLDEYRDR.L	3	2.72	0.18	-3.15
IPI00024570	Semaphorin-3G precursor	R.SDSDQSLLHDPR.F	2	2.81	0.33	0.20
IPI00024572	aspartate beta-hydroxylase isoform e	D.LVDYEEVLAK.A	2	3.39	0.31	-3.12
IPI00024572	aspartate beta-hydroxylase isoform e	K.LGIYDADGDGDFDVDDAK.V	2	6.23	0.56	-5.18
IPI00024572	aspartate beta-hydroxylase isoform e	K.LGIYDADGDGDFDVDDAK.V	3	4.80	0.31	-2.52
IPI00024580	Methylcrotonoyl-CoA carboxylase subunit alpha, mitochondrial precursor	K.IIEEAPAPGIKSEVRK.K	2	1.86	0.18	
IPI00024580	Methylcrotonoyl-CoA carboxylase subunit alpha, mitochondrial precursor	R.NM*TLKDGKNNV.A	1	1.82	0.17	-2.91
IPI00024587	D1 dopamine receptor-interacting protein calcyon	L.AAIGAYPLSR.K	1	2.28	0.29	-3.45
IPI00024587	D1 dopamine receptor-interacting protein calcyon	L.AAIGAYPLSR.K	2	3.02	0.37	-1.73
IPI00024587	D1 dopamine receptor-interacting protein calcyon	R.SILAAIGAYPLSR.K	1	3.34	0.39	-2.80
IPI00024587	D1 dopamine receptor-interacting protein calcyon	R.SILAAIGAYPLSR.K	2	4.49	0.42	-4.51
IPI00024587	D1 dopamine receptor-interacting protein calcyon	R.SILAAIGAYPLSR.K	3	3.49	0.30	-3.82
IPI00024587	D1 dopamine receptor-interacting protein calcyon	S.ILAAIGAYPLSR.K	2	3.45	0.28	-2.24
IPI00024601	Carbonic anhydrase-related protein 10	K.RQSPVNIETSHM*IFDPFLTPLR.I	3	3.77	0.19	-2.66
IPI00024601	Carbonic anhydrase-related protein 10	K.VSDSSNPFLNR.M	2	3.85	0.35	-3.48
IPI00024601	Carbonic anhydrase-related protein 10	K.VSGTM*YNTGR.H	2	2.82	0.26	-2.83
IPI00024601	Carbonic anhydrase-related protein 10	R.LLSQNQPSQIFLSM*SDNFRPVQPLNNR.C	3	5.24	0.46	-2.49
IPI00024601	Carbonic anhydrase-related protein 10	R.LLSQNQPSQIFLSM*SDNFRPVQPLNNR.C	4	4.44	0.34	-1.73
IPI00024601	Carbonic anhydrase-related protein 10	R.QSPVNIETSHM*IFDPFLTPLR.I	2	1.60	0.11	-3.88
IPI00024601	Carbonic anhydrase-related protein 10	R.QSPVNIETSHM*IFDPFLTPLR.I	3	2.96	0.23	-3.20
IPI00024601	Carbonic anhydrase-related protein 10	R.TNINFSLQGK.D	2	3.19	0.36	-2.56
IPI00024621	Isoform 1 of Olfactomedin-like protein 3 precursor	R.AALPYFPR.R	2	1.75	0.15	-1.37
IPI00024621	Isoform 1 of Olfactomedin-like protein 3 precursor	R.EALRTEADTISGR.V	2	2.94	0.27	-3.01
IPI00024621	Isoform 1 of Olfactomedin-like protein 3 precursor	R.EALRTEADTISGR.V	3	2.10	0.18	
IPI00024621	Isoform 1 of Olfactomedin-like protein 3 precursor	R.EVDYLETQNPALPCVEFDEK.V	2	2.85	0.34	
IPI00024621	Isoform 1 of Olfactomedin-like protein 3 precursor	R.EVDYLETQNPALPCVEFDEKVTGGPGTK.G	3	4.59	0.21	
IPI00024621	Isoform 1 of Olfactomedin-like protein 3 precursor	R.IQCSFDASGTLTPER.A	2	4.25	0.40	-2.61
IPI00024621	Isoform 1 of Olfactomedin-like protein 3 precursor	R.LAALEER.L	2	2.38	0.07	-2.74
IPI00024621	Isoform 1 of Olfactomedin-like protein 3 precursor	R.LRDFTLAM*AAR.K	3	3.62	0.09	-3.36
IPI00024621	Isoform 1 of Olfactomedin-like protein 3 precursor	R.RLAALEER.L	2	2.48	0.07	-1.95
IPI00024621	Isoform 1 of Olfactomedin-like protein 3 precursor	R.YGAHASLR.Y	2	1.84	0.06	-3.31
IPI00024662	Chromobox protein homolog 5	R.GLEPEKIIGATDSCGDLMFLMKWK.D	3	2.46	0.17	

IPI00024664	Isoform Long of Ubiquitin carboxyl-terminal hydrolase 5	R.AVDWIFSHIDDLDAEAAMDISEGR.S	3	2.58	0.22	-1.40
IPI00024689	Aquaporin-1	K.VWTSGQVEEYDLDADDINSR.V	2	6.45	0.60	-3.32
IPI00024689	Aquaporin-1	R.DLGGSAPLAIGLSVALGHLLAIDYTGCGINPAR.S	3	5.93	0.59	-6.33
IPI00024704	Uronyl 2-sulfotransferase	K.VLPFPSQVVYNR.V	2	2.32	0.29	-1.71
IPI00024704	Uronyl 2-sulfotransferase	R.FGGDQPVYINIIRDPVNR.F	3	2.92	0.21	-2.22
IPI00024766	Plexin-C1 precursor	R.SLATQELGR.L	2	3.03	0.21	-1.85
IPI00024802	TATA-binding protein-associated factor 172	K.DGM*HHTVTK.H	1	2.14	0.06	
IPI00024818	Isoform 1 of Ubiquitin-specific peptidase-like protein 1	K.SLVTFTNVIPEWHPLNAAHFGPCNNCNSKSQIRK.M	5	3.48	0.21	-4.42
IPI00024825	Isoform A of Proteoglycan-4 precursor	K.DQYYNIDVPSR.T	2	3.28	0.28	-4.14
IPI00024825	Isoform A of Proteoglycan-4 precursor	K.GFGGLTGQIVAALSTAK.Y	2	4.45	0.45	-3.93
IPI00024825	Isoform A of Proteoglycan-4 precursor	K.KAPPPSGASQTIK.S	2	2.10	0.27	-2.93
IPI00024825	Isoform A of Proteoglycan-4 precursor	R.CFESFER.G	2	1.45	0.06	-1.85
IPI00024825	Isoform A of Proteoglycan-4 precursor	R.FTNDIKDAGYPKPIFK.G	3	2.99	0.31	-4.63
IPI00024825	Isoform A of Proteoglycan-4 precursor	R.GLPNVVTSAISLPNIR.K	2	2.72	0.25	
IPI00024825	Isoform A of Proteoglycan-4 precursor	R.VCTAELSCK.G	2	2.17	0.30	-0.58
IPI00024853	Isoform 1 of Periaxin	R.FGLVRAKEGAEEGEKAK.S	2	3.68	0.08	
IPI00024887	Bone morphogenetic protein 6 precursor	R.QQEEQQQQQQLPR.G	2	3.78	0.21	-4.67
IPI00024919	Thioredoxin-dependent peroxide reductase, mitochondrial precursor	K.HLSVNDLPVGR.S	2	2.64	0.36	-4.11
IPI00024919	Thioredoxin-dependent peroxide reductase, mitochondrial precursor	R.DYGVLLEGSGLALR.G	2	5.14	0.45	-4.91
IPI00024920	ATP synthase subunit delta, mitochondrial precursor	K.AQAELVGTADEATR.A	2	3.25	0.37	-4.10
IPI00024929	Adipocyte adhesion molecule precursor	K.VVITYSSR.H	2	1.88	0.38	-1.88
IPI00024929	Adipocyte adhesion molecule precursor	R.IDYNHPGR.V	2	2.65	0.16	-2.85
IPI00024929	Adipocyte adhesion molecule precursor	R.VAFASNFLAGDASLQIEPLKPSDEGR.Y	3	4.46	0.40	-3.27
IPI00024966	Contactin-2 precursor	K.AGDKEAAADRVR.T	2	2.71	0.06	-1.86
IPI00024966	Contactin-2 precursor	K.AGDKEAAADRVR.T	3	2.98	0.30	-1.12
IPI00024966	Contactin-2 precursor	K.AQDAGVYQCLASNPVGTVVSR.E	2	6.30	0.56	-3.96
IPI00024966	Contactin-2 precursor	K.AQDAGVYQCLASNPVGTVVSR.E	3	5.42	0.46	-2.72
IPI00024966	Contactin-2 precursor	K.AVVLWSK.G	2	2.20	0.10	-2.38
IPI00024966	Contactin-2 precursor	K.EATVLVRGPPGPPGGVVVR.D	3	2.70	0.32	-1.53
IPI00024966	Contactin-2 precursor	K.FAQLNLAAEDTR.L	1	2.52	0.23	-2.49
IPI00024966	Contactin-2 precursor	K.FAQLNLAAEDTR.L	2	4.65	0.42	-3.04
IPI00024966	Contactin-2 precursor	K.LSLEDSGM*YQCVAENK.H	2	4.84	0.46	1.12
IPI00024966	Contactin-2 precursor	K.LSLEDSGM*YQCVAENK.H	3	3.09	0.15	-2.51
IPI00024966	Contactin-2 precursor	K.M*LYQNDLHLTPTLHLTGK.N	2	4.82	0.54	-4.02
IPI00024966	Contactin-2 precursor	K.M*LYQNDLHLTPTLHLTGK.N	3	3.92	0.40	-4.00
IPI00024966	Contactin-2 precursor	K.M*LYQNDLHLTPTLHLTGK.N	4	2.80	0.35	-2.48
IPI00024966	Contactin-2 precursor	K.NWIEIPVPEDIGHALVQIR.T	2	4.38	0.46	-4.44

IPI00024966	Contactin-2 precursor	K.NWIEIPVPEDIGHALVQIR.T	3	2.91	0.31	-3.98
IPI00024966	Contactin-2 precursor	K.VISDTEADIGSNLR.W	2	4.95	0.51	-4.74
IPI00024966	Contactin-2 precursor	K.YTCFAENFM*GK.A	2	3.57	0.46	-2.84
IPI00024966	Contactin-2 precursor	K.YTCM*AQTVVDSASK.E	2	5.12	0.57	-2.72
IPI00024966	Contactin-2 precursor	K.YTCM*AQTVVDSASKEATVLVR.G	3	2.62	0.29	0.39
IPI00024966	Contactin-2 precursor	R.ASPPATYR.W	1	1.67	0.18	-0.55
IPI00024966	Contactin-2 precursor	R.DIGDTTIQLSWSR.G	2	4.83	0.50	-5.22
IPI00024966	Contactin-2 precursor	R.EAAPSVAPSGLSGGGGAPGELIVNWTPM*SR.E	2	4.97	0.57	-4.79
IPI00024966	Contactin-2 precursor	R.EAAPSVAPSGLSGGGGAPGELIVNWTPM*SR.E	3	4.23	0.44	-4.30
IPI00024966	Contactin-2 precursor	R.EYQNGDGFGYLLSFR.R	2	4.77	0.47	-3.67
IPI00024966	Contactin-2 precursor	R.EYQNGDGFGYLLSFR.R	3	3.17	0.09	-2.56
IPI00024966	Contactin-2 precursor	R.FGFLQEFSK.E	1	2.84	0.19	-3.55
IPI00024966	Contactin-2 precursor	R.FGFLQEFSK.E	2	3.45	0.26	-2.43
IPI00024966	Contactin-2 precursor	R.FGFLQEFSKEER.D	2	3.35	0.37	-3.54
IPI00024966	Contactin-2 precursor	R.FGFLQEFSKEER.D	3	2.94	0.31	-1.99
IPI00024966	Contactin-2 precursor	R.FGFLQEFSKEERDPVK.A	2	4.18	0.46	-6.56
IPI00024966	Contactin-2 precursor	R.FGFLQEFSKEERDPVK.A	4	2.57	0.28	-2.30
IPI00024966	Contactin-2 precursor	R.GFDNHSPIAK.Y	1	2.27	0.26	-4.59
IPI00024966	Contactin-2 precursor	R.GFDNHSPIAK.Y	2	2.82	0.37	-2.04
IPI00024966	Contactin-2 precursor	R.GGEILIPCQPR.A	1	2.16	0.09	-3.92
IPI00024966	Contactin-2 precursor	R.GGEILIPCQPR.A	2	3.29	0.06	-2.32
IPI00024966	Contactin-2 precursor	R.GPPGPPGGVVVR.D	1	2.59	0.34	-2.49
IPI00024966	Contactin-2 precursor	R.GPPGPPGGVVVR.D	2	2.98	0.20	-0.56
IPI00024966	Contactin-2 precursor	R.HFVSQTTGNLYIAR.T	2	4.79	0.50	-2.61
IPI00024966	Contactin-2 precursor	R.HFVSQTTGNLYIAR.T	3	3.59	0.41	-1.81
IPI00024966	Contactin-2 precursor	R.HQLVGGNLVIM*NPTK.A	2	4.91	0.41	-2.38
IPI00024966	Contactin-2 precursor	R.HQLVGGNLVIM*NPTK.A	3	2.72	0.09	-1.47
IPI00024966	Contactin-2 precursor	R.IIVQAQPEWLK.V	1	2.69	0.22	-2.80
IPI00024966	Contactin-2 precursor	R.IIVQAQPEWLK.V	2	4.18	0.38	-2.92
IPI00024966	Contactin-2 precursor	R.NGEPLASQNR.V	2	2.70	0.34	-1.83
IPI00024966	Contactin-2 precursor	R.NGEPLASQNRVEVLAGDLR.F	2	3.03	0.17	-1.40
IPI00024966	Contactin-2 precursor	R.NGEPLASQNRVEVLAGDLR.F	3	4.03	0.41	-2.47
IPI00024966	Contactin-2 precursor	R.RGDGPESLTALVYSAEEEPR.V	2	5.53	0.54	0.25
IPI00024966	Contactin-2 precursor	R.RGDGPESLTALVYSAEEEPR.V	3	4.86	0.32	-2.24
IPI00024966	Contactin-2 precursor	R.RPPGNISWTFSSSSLSIK.W	3	4.03	0.38	-1.65
IPI00024966	Contactin-2 precursor	R.RTNVKETIGDLTILNAQLR.H	2	5.27	0.40	-3.79
IPI00024966	Contactin-2 precursor	R.RTNVKETIGDLTILNAQLR.H	4	3.26	0.17	-3.02
IPI00024966	Contactin-2 precursor	R.SDEGKYTCFAENFM*GK.A	3	2.30	0.11	-1.50
IPI00024966	Contactin-2 precursor	R.TAGLDTSAR.V	1	2.02	0.24	-2.93
IPI00024966	Contactin-2 precursor	R.TAGLDTSAR.V	2	2.81	0.20	-2.96
IPI00024966	Contactin-2 precursor	R.TNPANIEGNAETAQVLGLTPWM*DYEFR.V	2	5.52	0.64	-2.68
IPI00024966	Contactin-2 precursor	R.TNPANIEGNAETAQVLGLTPWM*DYEFR.V	3	6.13	0.49	-4.37

IPI00024966	Contactin-2 precursor	R.TNVKETIGDLTILNAQLR.H	2	6.36	0.53	-3.51
IPI00024966	Contactin-2 precursor	R.TNVKETIGDLTILNAQLR.H	3	4.64	0.25	-4.08
IPI00024966	Contactin-2 precursor	R.TREAAPSVAPSGLSGGGGAPGELIVNWTPM*SR.E	3	6.49	0.58	-3.16
IPI00024966	Contactin-2 precursor	R.TTGPGGDGIPAEVHIVR.N	3	3.20	0.41	-2.03
IPI00024966	Contactin-2 precursor	R.VEVLAGDLR.F	1	2.30	0.08	-3.45
IPI00024966	Contactin-2 precursor	R.VEVLAGDLR.F	2	3.68	0.26	-1.96
IPI00024966	Contactin-2 precursor	R.VIASNILGTGEPSGPSSK.I	2	6.25	0.53	-3.89
IPI00024966	Contactin-2 precursor	R.VIASNILGTGEPSGPSSK.I	3	3.39	0.27	-3.40
IPI00024966	Contactin-2 precursor	R.VSGLHPNTK.Y	1	2.15	0.26	-5.12
IPI00024966	Contactin-2 precursor	R.VSGLHPNTK.Y	2	2.77	0.28	-3.17
IPI00024966	Contactin-2 precursor	R.VTVTPDGTLIIR.N	1	2.40	0.18	-3.65
IPI00024966	Contactin-2 precursor	R.VTVTPDGTLIIR.N	2	2.97	0.24	-3.10
IPI00024966	Contactin-2 precursor	R.WLLNEFPNFIPTDGR.H	2	4.66	0.49	-6.44
IPI00024966	Contactin-2 precursor	R.WLLNEFPNFIPTDGR.H	3	3.73	0.25	-3.76
IPI00024966	Contactin-2 precursor	T.TGNLYIAR.T	2	3.24	0.18	-0.84
IPI00024966	Contactin-2 precursor	V.SQTTGNLYIAR.T	2	3.32	0.40	-1.55
IPI00024966	Contactin-2 precursor	W.IEIPVPEDIGHALVQIR.T	2	3.16	0.39	-1.88
IPI00024976	Mitochondrial import receptor subunit TOM22 homolog	K.LQM*EQQQQLQQR.Q	2	3.68	0.33	-2.47
IPI00024976	Mitochondrial import receptor subunit TOM22 homolog	P.VVFETEKLQM*EQQQQLQQR.Q	3	4.24	0.30	-2.30
IPI00025019	Proteasome subunit beta type-1 precursor	R.DVYTGDALR.I	2	2.08	0.16	-2.88
IPI00025084	Calpain small subunit 1	R.SM*VAVM*DSDTTGK.L	2	3.93	0.43	-2.70
IPI00025092	Myosin-binding protein C, slow-type	K.IILDGLDADNTVTVIAGNKLRLEIPISGEPPPK.A	3	3.54	0.08	
IPI00025094	CDNA: FLJ22037 fis, clone HEP08868 (Fragment)	K.EHQDRIEELEEELEAERAMRAKIEQNR.K	3	2.70	0.06	-1.04
IPI00025110	Isoform 2 of Mesothelin precursor	K.ALLEVNKGHEM*SPQVATLIDR.F	4	2.92	0.14	-2.96
IPI00025110	Isoform 2 of Mesothelin precursor	K.LDELYPQGYPESVIQHLGYLFLK.M	3	3.29	0.27	-4.26
IPI00025110	Isoform 2 of Mesothelin precursor	K.LLGPHVEGLK.A	2	1.68	0.07	-1.97
IPI00025110	Isoform 2 of Mesothelin precursor	K.LLGPHVEGLKAEER.H	2	3.96	0.41	-1.89
IPI00025110	Isoform 2 of Mesothelin precursor	K.LRTDAVLPLTVAEVQK.L	3	3.90	0.31	-2.03
IPI00025110	Isoform 2 of Mesothelin precursor	R.EIDESLIFYK.K	2	2.16	0.19	-3.17
IPI00025110	Isoform 2 of Mesothelin precursor	R.QLDVLYPK.A	1	2.02	0.06	-3.26
IPI00025110	Isoform 2 of Mesothelin precursor	R.TDAVLPLTVAEVQK.L	2	4.30	0.41	0.13
IPI00025110	Isoform 2 of Mesothelin precursor	R.VNAIPFTYEQLDVLK.H	2	3.36	0.25	-2.92
IPI00025204	CD5 antigen-like precursor	K.GVWGSVCDDNWGEKEDQVVCK.Q	2	4.41	0.27	
IPI00025204	CD5 antigen-like precursor	K.NTCNHDEDTWVECEDPFDLR.L	3	5.01	0.26	
IPI00025204	CD5 antigen-like precursor	R.CSGEEQSLEQCQHR.F	3	2.78	0.38	
IPI00025204	CD5 antigen-like precursor	R.EATLQDCPSGPWGK.N	2	2.63	0.26	
IPI00025204	CD5 antigen-like precursor	R.ELGCGAASGTPSGILYEPPAEKEQK.V	2	4.24	0.36	
IPI00025204	CD5 antigen-like precursor	R.ELGCGAASGTPSGILYEPPAEKEQK.V	3	3.86	0.43	
IPI00025204	CD5 antigen-like precursor	R.LVGGDNLCSGR.L	2	3.00	0.22	
IPI00025252	Protein disulfide-isomerase A3 precursor	A.SDVLELTDDNFESR.I	2	5.03	0.47	-3.75

IPI00025252	Protein disulfide-isomerase A3 precursor	K.AASNLRDNYR.F	2	2.56	0.11	0.31
IPI00025252	Protein disulfide-isomerase A3 precursor	K.DLLIAYYDVDYEK.N	2	4.84	0.46	-3.74
IPI00025252	Protein disulfide-isomerase A3 precursor	K.FEDKTVAYTEQK.M	3	3.31	0.11	-1.13
IPI00025252	Protein disulfide-isomerase A3 precursor	K.FISDKDASIVGFFDDSFSEAHSEFLK.A	3	5.49	0.51	-5.06
IPI00025252	Protein disulfide-isomerase A3 precursor	K.FISDKDASIVGFFDDSFSEAHSEFLK.A	4	3.49	0.21	-2.98
IPI00025252	Protein disulfide-isomerase A3 precursor	K.FVM*QEEFSR.D	2	2.76	0.20	-3.28
IPI00025252	Protein disulfide-isomerase A3 precursor	K.IFRDGEEAGAYDGPR.T	2	2.44	0.08	-2.92
IPI00025252	Protein disulfide-isomerase A3 precursor	K.IFRDGEEAGAYDGPR.T	3	4.17	0.19	-1.94
IPI00025252	Protein disulfide-isomerase A3 precursor	K.LNFAVASR.K	2	2.43	0.10	-2.75
IPI00025252	Protein disulfide-isomerase A3 precursor	K.M*DATANDVPSPYEVR.G	2	5.08	0.41	-4.30
IPI00025252	Protein disulfide-isomerase A3 precursor	K.RLAPEYEAAATR.L	2	3.50	0.41	-2.81
IPI00025252	Protein disulfide-isomerase A3 precursor	K.SEPIPESNDGPVK.V	2	2.34	0.11	-4.02
IPI00025252	Protein disulfide-isomerase A3 precursor	K.TFSHELSDFGLESTAGEIPVVAIR.T	3	3.51	0.33	-3.16
IPI00025252	Protein disulfide-isomerase A3 precursor	K.TVAYTEQK.M	2	2.71	0.15	-2.43
IPI00025252	Protein disulfide-isomerase A3 precursor	K.YGVSGYPTLK.I	1	1.96	0.15	-0.49
IPI00025252	Protein disulfide-isomerase A3 precursor	K.YGVSGYPTLK.I	2	3.24	0.39	-0.85
IPI00025252	Protein disulfide-isomerase A3 precursor	K.YKELGEK.L	1	2.20	0.09	-1.62
IPI00025252	Protein disulfide-isomerase A3 precursor	R.DGEEAGAYDGPR.T	2	3.86	0.41	-1.21
IPI00025252	Protein disulfide-isomerase A3 precursor	R.EATNPPVIQEEKPK.K	2	2.62	0.14	-1.94
IPI00025252	Protein disulfide-isomerase A3 precursor	R.ELSDFISYLQR.E	2	3.85	0.39	-3.50
IPI00025252	Protein disulfide-isomerase A3 precursor	R.FAHTNVESLVNEYDDNGEGIILFRPSHLTNK.F	3	4.84	0.34	-0.85
IPI00025252	Protein disulfide-isomerase A3 precursor	R.FLQDYFDGNLKR.Y	2	3.46	0.39	-4.13
IPI00025252	Protein disulfide-isomerase A3 precursor	R.FLQDYFDGNLKR.Y	3	2.17	0.19	-1.29
IPI00025252	Protein disulfide-isomerase A3 precursor	R.GFPTIYFSPANK.K	2	3.16	0.22	-2.30
IPI00025252	Protein disulfide-isomerase A3 precursor	R.GFPTIYFSPANKK.L	2	2.07	0.11	-4.00
IPI00025252	Protein disulfide-isomerase A3 precursor	R.KTFSHELSDFGLESTAGEIPVVAIR.T	3	4.38	0.40	-4.07
IPI00025252	Protein disulfide-isomerase A3 precursor	R.KTFSHELSDFGLESTAGEIPVVAIR.T	4	3.05	0.17	-3.72
IPI00025252	Protein disulfide-isomerase A3 precursor	R.LAPEYEAAATR.L	2	3.26	0.31	-3.17
IPI00025252	Protein disulfide-isomerase A3 precursor	R.LKGIVPLAK.V	1	2.36	0.09	-2.28
IPI00025252	Protein disulfide-isomerase A3 precursor	R.TADGIVSHLK.K	2	1.84	0.13	-2.04
IPI00025257	Semaphorin-7A precursor	K.AM*LVCSDAATNK.N	2	3.70	0.41	-2.66
IPI00025257	Semaphorin-7A precursor	K.AM*LVCSDAATNKNFNR.L	2	4.22	0.44	-3.73
IPI00025257	Semaphorin-7A precursor	K.AM*LVCSDAATNKNFNR.L	3	2.65	0.27	-2.11
IPI00025257	Semaphorin-7A precursor	K.ATIVHQDQAYDDK.I	2	4.38	0.46	-2.68
IPI00025257	Semaphorin-7A precursor	K.ATIVHQDQAYDDK.I	3	2.81	0.29	-3.19
IPI00025257	Semaphorin-7A precursor	K.ATIVHQDQAYDDKIYYFFR.E	2	5.94	0.48	-5.49
IPI00025257	Semaphorin-7A precursor	K.ATIVHQDQAYDDKIYYFFR.E	3	4.80	0.48	-4.65
IPI00025257	Semaphorin-7A precursor	K.ATIVHQDQAYDDKIYYFFR.E	4	4.76	0.44	-4.54
IPI00025257	Semaphorin-7A precursor	K.CLPDQQPIPTETFQVADR.H	2	3.93	0.42	-4.27
IPI00025257	Semaphorin-7A precursor	K.GYHSSLPNPRPGK.C	2	3.67	0.37	-4.14
IPI00025257	Semaphorin-7A precursor	K.GYHSSLPNPRPGK.C	3	2.73	0.27	-5.17
IPI00025257	Semaphorin-7A precursor	K.LYVSSQWEVSQVPLDLCEVYGGGCHGCLM*SR.D	3	3.45	0.26	-2.68

IPI00025257	Semaphorin-7A precursor	K.TPLFHSK.Y	2	2.53	0.07	-2.40
IPI00025257	Semaphorin-7A precursor	K.VSLAPNSR.Y	2	1.77	0.07	-3.80
IPI00025257	Semaphorin-7A precursor	K.VVEPGEQEHSFAFNIM*EIQPFR.R	2	4.37	0.53	-2.05
IPI00025257	Semaphorin-7A precursor	K.VVEPGEQEHSFAFNIM*EIQPFR.R	3	4.48	0.38	-4.22
IPI00025257	Semaphorin-7A precursor	K.VVEPGEQEHSFAFNIM*EIQPFRR.A	3	2.19	0.20	-2.87
IPI00025257	Semaphorin-7A precursor	K.VVEPGEQEHSFAFNIM*EIQPFRR.A	4	1.89	0.19	-3.16
IPI00025257	Semaphorin-7A precursor	K.VYLFDFPEGK.N	2	3.75	0.39	-3.95
IPI00025257	Semaphorin-7A precursor	R.AAAIQTM*SLDAER.R	2	3.96	0.45	-3.31
IPI00025257	Semaphorin-7A precursor	R.AAAIQTM*SLDAERR.K	3	2.19	0.16	-0.30
IPI00025257	Semaphorin-7A precursor	R.CISIYSSER.S	1	2.08	0.20	-1.87
IPI00025257	Semaphorin-7A precursor	R.CISIYSSER.S	2	3.14	0.23	-1.97
IPI00025257	Semaphorin-7A precursor	R.DCENYITLLER.R	2	4.39	0.40	-4.91
IPI00025257	Semaphorin-7A precursor	R.DPYCGWDQGR.C	2	3.29	0.38	-2.54
IPI00025257	Semaphorin-7A precursor	R.EAQHWQLLPEDGIM*AEH.L	2	3.72	0.53	-3.07
IPI00025257	Semaphorin-7A precursor	R.GDQGGESSLSVSK.W	1	1.88	0.24	-3.81
IPI00025257	Semaphorin-7A precursor	R.GDQGGESSLSVSK.W	2	3.86	0.33	-3.94
IPI00025257	Semaphorin-7A precursor	R.GESELYTSDTVM*QNPQFIK.A	2	5.75	0.57	-4.13
IPI00025257	Semaphorin-7A precursor	R.GESELYTSDTVM*QNPQFIK.A	3	3.94	0.43	-0.29
IPI00025257	Semaphorin-7A precursor	R.GKVYLFDFPEGK.N	2	4.19	0.45	-4.63
IPI00025257	Semaphorin-7A precursor	R.GKVYLFDFPEGK.N	3	4.27	0.29	-3.04
IPI00025257	Semaphorin-7A precursor	R.GYAPFSPDENSLVLFEGDEVYSTIR.K	2	5.82	0.63	-4.06
IPI00025257	Semaphorin-7A precursor	R.GYAPFSPDENSLVLFEGDEVYSTIR.K	3	4.80	0.48	-5.76
IPI00025257	Semaphorin-7A precursor	R.GYAPFSPDENSLVLFEGDEVYSTIRK.Q	3	1.99	0.16	-4.46
IPI00025257	Semaphorin-7A precursor	R.IRGESELYTSDTVM*QNPQFIK.A	2	4.54	0.49	-4.94
IPI00025257	Semaphorin-7A precursor	R.IRGESELYTSDTVM*QNPQFIK.A	3	4.70	0.42	-5.27
IPI00025257	Semaphorin-7A precursor	R.LQDVFLLPDPSGQWR.D	2	4.58	0.39	-3.55
IPI00025257	Semaphorin-7A precursor	R.M*QASHGETFHVLYLTTDR.G	2	5.77	0.53	-2.10
IPI00025257	Semaphorin-7A precursor	R.M*QASHGETFHVLYLTTDR.G	3	5.54	0.51	-3.26
IPI00025257	Semaphorin-7A precursor	R.M*QASHGETFHVLYLTTDR.G	4	3.90	0.22	-0.89
IPI00025257	Semaphorin-7A precursor	R.RAAAIQTM*SLDAER.R	2	2.05	0.20	-4.99
IPI00025257	Semaphorin-7A precursor	R.SEGLLACGTNAR.H	1	2.23	0.29	-0.50
IPI00025257	Semaphorin-7A precursor	R.SEGLLACGTNAR.H	2	4.57	0.48	-2.00
IPI00025257	Semaphorin-7A precursor	R.TVNIGSTK.G	1	2.17	0.13	-1.97
IPI00025257	Semaphorin-7A precursor	R.VEPM*GPLKTPLFHSK.Y	2	2.06	0.17	-3.15
IPI00025257	Semaphorin-7A precursor	R.YYLSCPM*ESR.H	2	3.08	0.40	-0.29
IPI00025276	Isoform XB of Tenascin-X precursor	K.ADSIQGTAR.T	2	2.62	0.12	-2.79
IPI00025276	Isoform XB of Tenascin-X precursor	K.DAQGQPQAVPVAGDENEVTVPGLDPDRK.Y	3	4.36	0.48	-5.18
IPI00025276	Isoform XB of Tenascin-X precursor	K.DRDGQPQVVPVEGSLR.E	2	3.33	0.18	-2.96
IPI00025276	Isoform XB of Tenascin-X precursor	K.DRDGQPQVVR.V	2	2.76	0.15	-1.99
IPI00025276	Isoform XB of Tenascin-X precursor	K.EEPPRPEFLEQPLLGELTVTGVTPDSLR.L	3	4.72	0.49	-4.73
IPI00025276	Isoform XB of Tenascin-X precursor	K.FLLFGIQDGKR.R	3	2.81	0.23	-2.06
IPI00025276	Isoform XB of Tenascin-X precursor	K.FLLYGLLGGKR.L	2	2.62	0.26	-4.41

IPI00025276	Isoform XB of Tenascin-X precursor	K.GFEFSVPFTEM*K.L	2	3.06	0.43	-5.09
IPI00025276	Isoform XB of Tenascin-X precursor	K.LQGLIPGAR.Y	2	2.58	0.17	-0.45
IPI00025276	Isoform XB of Tenascin-X precursor	R.AVAVSGLDPAR.K	2	2.23	0.22	-2.08
IPI00025276	Isoform XB of Tenascin-X precursor	R.AVAVSGLDPARK.Y	2	2.54	0.18	-2.94
IPI00025276	Isoform XB of Tenascin-X precursor	R.AVPVAADQR.T	1	1.32	0.05	-3.11
IPI00025276	Isoform XB of Tenascin-X precursor	R.DAQGQPQAVPVSGDLR.A	2	4.23	0.48	-3.68
IPI00025276	Isoform XB of Tenascin-X precursor	R.DCGEEM*QNGAGASR.T	2	4.24	0.46	-4.16
IPI00025276	Isoform XB of Tenascin-X precursor	R.DRDPNSLLISCAVSYR.G	2	4.69	0.48	-2.05
IPI00025276	Isoform XB of Tenascin-X precursor	R.DRDPNSLLISCAVSYR.G	3	2.96	0.24	-2.36
IPI00025276	Isoform XB of Tenascin-X precursor	R.EVSVPGLDPAHR.Y	2	2.12	0.21	-1.35
IPI00025276	Isoform XB of Tenascin-X precursor	R.FGVPSPSTLEPHPRPLLQR.E	4	1.76	0.16	-0.92
IPI00025276	Isoform XB of Tenascin-X precursor	R.FLLYGLSGR.K	2	2.87	0.20	-0.83
IPI00025276	Isoform XB of Tenascin-X precursor	R.FTLFGIANK.K	2	2.71	0.30	-1.24
IPI00025276	Isoform XB of Tenascin-X precursor	R.GFEESEPLTGFLTTVPDGPTQLR.A	2	4.16	0.54	-5.80
IPI00025276	Isoform XB of Tenascin-X precursor	R.HGPVPVEAR.T	2	2.55	0.25	-3.09
IPI00025276	Isoform XB of Tenascin-X precursor	R.LGPISADSTTAPLEK.E	2	3.54	0.47	-4.15
IPI00025276	Isoform XB of Tenascin-X precursor	R.LGPLSAEGTTGLAPAGQTSEESRPR.L	3	3.72	0.35	-2.24
IPI00025276	Isoform XB of Tenascin-X precursor	R.LGQM*TVR.D	2	2.10	0.14	-2.95
IPI00025276	Isoform XB of Tenascin-X precursor	R.LGVLTVTDTTPDSM*R.L	2	3.86	0.58	-1.48
IPI00025276	Isoform XB of Tenascin-X precursor	R.LNWEAPPGAFDSFLLR.F	2	3.89	0.41	-1.45
IPI00025276	Isoform XB of Tenascin-X precursor	R.LSQLSVTDVTTSSLR.L	2	4.49	0.50	-4.33
IPI00025276	Isoform XB of Tenascin-X precursor	R.LSWTVAQGPFDSFVVQYR.D	2	4.79	0.51	-3.82
IPI00025276	Isoform XB of Tenascin-X precursor	R.SFVVSSLDPDHKYR.F	3	2.12	0.12	-1.20
IPI00025276	Isoform XB of Tenascin-X precursor	R.SGTLYSLTLYGLR.G	2	3.63	0.36	-3.49
IPI00025276	Isoform XB of Tenascin-X precursor	R.TLSPVLESPR.D	2	2.40	0.16	-2.90
IPI00025276	Isoform XB of Tenascin-X precursor	R.TQKLQGLIPGAR.Y	2	1.71	0.20	-2.25
IPI00025276	Isoform XB of Tenascin-X precursor	R.TSTIFLNGNR.E	2	3.20	0.30	-3.04
IPI00025276	Isoform XB of Tenascin-X precursor	R.TVTVEDLEPGKK.Y	2	3.08	0.32	-3.15
IPI00025276	Isoform XB of Tenascin-X precursor	R.VGGKESEVTVGGLEPGHK.Y	4	3.45	0.31	-2.94
IPI00025276	Isoform XB of Tenascin-X precursor	R.VPGHEDGVTISGLEPDHKYK.M	3	4.68	0.44	-0.46
IPI00025276	Isoform XB of Tenascin-X precursor	R.VPGHEDRVTISGLEPDNKYK.M	5	2.96	0.20	-2.77
IPI00025276	Isoform XB of Tenascin-X precursor	R.VRGEESEVTVGGLEPGRK.Y	2	3.83	0.37	-1.22
IPI00025276	Isoform XB of Tenascin-X precursor	R.VRGEESEVTVGGLEPGRK.Y	3	5.13	0.42	-3.01
IPI00025276	Isoform XB of Tenascin-X precursor	R.YEVTVVSVR.G	2	2.60	0.33	-2.79
IPI00025311	Isoform 1 of Breast carcinoma-amplified sequence 1	K.TITPPEPEPTGAPQK.G	2	2.37	0.29	-4.14
IPI00025318	SH3 domain-binding glutamic acid-rich-like protein	R.ENNAVYAFLGLTAPPGSK.E	2	3.86	0.41	-4.94
IPI00025363	Isoform 1 of Glial fibrillary acidic protein	K.ALAAELNQLR.A	2	3.12	0.14	-1.56
IPI00025365	Isoform Long of Endothelin-3 precursor	K.ACLHFCTQTLDVSSNSR.T	3	2.94	0.19	-5.18
IPI00025426	Pregnancy zone protein precursor	K.AGAFCLSEDAGLGISSTASLR.A	2	6.45	0.50	
IPI00025426	Pregnancy zone protein precursor	K.AGAFCLSEDAGLGISSTASLR.A	3	4.78	0.28	
IPI00025426	Pregnancy zone protein precursor	K.ATVLNYLPK.C	2	3.01	0.26	

IPI00025426	Pregnancy zone protein precursor	K.GSFALSFPVESDVAPIAR.M	2	3.50	0.38	
IPI00025426	Pregnancy zone protein precursor	K.M*VSGFIPLKPTVK.M	1	2.26	0.19	
IPI00025426	Pregnancy zone protein precursor	K.M*VSGFIPLKPTVK.M	2	2.92	0.21	-4.20
IPI00025426	Pregnancy zone protein precursor	R.AFQPFFVELTM*PYSVIR.G	2	4.15	0.46	-5.03
IPI00025426	Pregnancy zone protein precursor	R.AFQPFFVELTM*PYSVIR.G	3	4.61	0.30	
IPI00025426	Pregnancy zone protein precursor	R.AFQPFFVELTMPYSVIR.G	2	4.99	0.39	
IPI00025426	Pregnancy zone protein precursor	R.AFQPFFVELTMPYSVIR.G	3	4.07	0.18	
IPI00025426	Pregnancy zone protein precursor	R.NQGNTWLTAFVLK.T	2	3.93	0.37	-1.44
IPI00025426	Pregnancy zone protein precursor	R.SSGSLLNNAIK.G	1	2.43	0.14	
IPI00025426	Pregnancy zone protein precursor	R.SSGSLLNNAIK.G	2	3.25	0.23	-1.71
IPI00025426	Pregnancy zone protein precursor	R.VVVQTESGGR.I	2	2.42	0.32	-0.62
IPI00025426	Pregnancy zone protein precursor	R.YGAATFTR.T	1	2.12	0.22	
IPI00025426	Pregnancy zone protein precursor	R.YGAATFTR.T	2	2.41	0.20	1.23
IPI00025447	Elongation factor 1-alpha	K.IGGIGTVPVGR.V	2	1.68	0.22	-1.61
IPI00025447	Elongation factor 1-alpha	K.STTTGHLIYK.C	2	1.84	0.19	-2.49
IPI00025447	Elongation factor 1-alpha	R.VETGVLKPGM*VVTFAPVNVTTEVK.S	3	5.20	0.40	-2.81
IPI00025465	Mimecan precursor	I.EEIRLEGNPIVLGK.H	2	2.97	0.22	-2.57
IPI00025465	Mimecan precursor	K.DFADIPNLR.R	1	1.63	0.10	-4.22
IPI00025465	Mimecan precursor	K.DFADIPNLR.R	2	3.34	0.26	-2.21
IPI00025465	Mimecan precursor	K.EKETVIIPNEK.S	1	3.53	0.28	-3.43
IPI00025465	Mimecan precursor	K.EKETVIIPNEK.S	2	3.29	0.24	-4.27
IPI00025465	Mimecan precursor	K.ESAYLYAR.F	1	2.10	0.18	-3.22
IPI00025465	Mimecan precursor	K.ESAYLYAR.F	2	1.94	0.25	-2.76
IPI00025465	Mimecan precursor	K.ETVIIPNEK.S	1	2.73	0.18	-4.15
IPI00025465	Mimecan precursor	K.ETVIIPNEK.S	2	2.31	0.12	-2.78
IPI00025465	Mimecan precursor	K.KLNNLTFLYLDHNALESVPLNLPESLR.V	2	4.85	0.63	-5.64
IPI00025465	Mimecan precursor	K.KLNNLTFLYLDHNALESVPLNLPESLR.V	3	6.11	0.55	-5.46
IPI00025465	Mimecan precursor	K.KLNNLTFLYLDHNALESVPLNLPESLR.V	4	2.45	0.18	-4.13
IPI00025465	Mimecan precursor	K.KLTAKDFADIPNLR.R	2	3.27	0.29	-4.08
IPI00025465	Mimecan precursor	K.LNNLTFLYLDHNALESVPLNLPESLR.V	2	5.30	0.55	-4.03
IPI00025465	Mimecan precursor	K.LNNLTFLYLDHNALESVPLNLPESLR.V	3	3.60	0.30	-4.93
IPI00025465	Mimecan precursor	K.LPVLPPK.L	1	1.50	0.06	-1.63
IPI00025465	Mimecan precursor	K.LPVLPPKLTLFNAK.Y	2	2.57	0.27	-3.94
IPI00025465	Mimecan precursor	K.LPVLPPKLTLFNAK.Y	3	4.18	0.40	-3.00
IPI00025465	Mimecan precursor	K.LSLLEELSLAENQLL.K	2	2.91	0.19	-4.04
IPI00025465	Mimecan precursor	K.LSLLEELSLAENQLLK.L	1	2.29	0.44	-2.06
IPI00025465	Mimecan precursor	K.LSLLEELSLAENQLLK.L	2	5.94	0.43	-7.45
IPI00025465	Mimecan precursor	K.LSLLEELSLAENQLLK.L	3	5.28	0.37	-4.37
IPI00025465	Mimecan precursor	K.LSLLEELSLAENQLLKLPVLPPK.L	2	5.59	0.61	-5.18
IPI00025465	Mimecan precursor	K.LSLLEELSLAENQLLKLPVLPPK.L	3	8.11	0.64	-5.43
IPI00025465	Mimecan precursor	K.LSLLEELSLAENQLLKLPVLPPK.L	4	5.91	0.64	-4.20
IPI00025465	Mimecan precursor	K.LTAKDFADIPNLR.R	2	4.11	0.40	-3.33

IPI00025465	Mimecan precursor	K.LTAKDFADIPNLR.R	3	3.58	0.12	-2.23
IPI00025465	Mimecan precursor	K.LTAKDFADIPNLRR.L	2	2.24	0.20	-2.12
IPI00025465	Mimecan precursor	K.LTAKDFADIPNLRR.L	3	1.96	0.19	-0.26
IPI00025465	Mimecan precursor	K.LTAKDFADIPNLRR.L	4	2.70	0.34	-2.35
IPI00025465	Mimecan precursor	K.NIKEKETVIIPNEK.S	2	4.44	0.33	-3.70
IPI00025465	Mimecan precursor	K.NIKEKETVIIPNEK.S	3	4.04	0.17	-3.08
IPI00025465	Mimecan precursor	K.RLPIGSYF.-	1	2.03	0.26	-2.56
IPI00025465	Mimecan precursor	K.RLPIGSYF.-	2	2.19	0.17	-2.40
IPI00025465	Mimecan precursor	L.AENQLLKLPVLPPK.L	2	3.17	0.28	-1.39
IPI00025465	Mimecan precursor	L.EELSLAENQLLKLPVLPPK.L	2	3.43	0.29	-3.56
IPI00025465	Mimecan precursor	L.SLAENQLLKLPVLPPK.L	2	3.26	0.38	-1.52
IPI00025465	Mimecan precursor	L.SLLEELSLAENQLLK.L	2	4.84	0.37	-4.83
IPI00025465	Mimecan precursor	L.SLLEELSLAENQLLK.L	3	4.13	0.25	-2.45
IPI00025465	Mimecan precursor	L.SLLEELSLAENQLLKLPVLPPK.L	3	4.99	0.54	-3.94
IPI00025465	Mimecan precursor	N.IKEKETVIIPNEK.S	2	4.30	0.33	-3.63
IPI00025465	Mimecan precursor	R.DRIEEIRLEGNPIVLGK.H	2	4.06	0.46	-2.41
IPI00025465	Mimecan precursor	R.DRIEEIRLEGNPIVLGK.H	3	3.08	0.36	-1.92
IPI00025465	Mimecan precursor	R.IEEIRLEGNPIVLGK.H	2	3.62	0.36	-3.52
IPI00025465	Mimecan precursor	R.IEEIRLEGNPIVLGK.H	3	4.79	0.35	-2.04
IPI00025465	Mimecan precursor	R.IEEIRLEGNPIVLGKHPNSFICLK.R	3	5.51	0.34	-3.90
IPI00025465	Mimecan precursor	R.IEEIRLEGNPIVLGKHPNSFICLK.R	4	4.32	0.41	-3.15
IPI00025465	Mimecan precursor	R.LDFTGNLIEDIEDGTFSK.L	2	5.45	0.51	-6.34
IPI00025465	Mimecan precursor	R.LDFTGNLIEDIEDGTFSK.L	3	6.29	0.50	-1.73
IPI00025465	Mimecan precursor	R.LEGNPIVLGK.H	1	2.36	0.20	-3.40
IPI00025465	Mimecan precursor	R.LEGNPIVLGK.H	2	3.42	0.19	-3.05
IPI00025465	Mimecan precursor	R.LEGNPIVLGKHPNSFICLK.R	3	3.19	0.38	-3.89
IPI00025465	Mimecan precursor	R.LEGNPIVLGKHPNSFICLK.R	4	2.95	0.28	-2.89
IPI00025465	Mimecan precursor	R.RLDFTGNLIEDIEDGTFSK.L	2	5.99	0.58	-5.46
IPI00025465	Mimecan precursor	R.RLDFTGNLIEDIEDGTFSK.L	3	6.62	0.47	-5.37
IPI00025465	Mimecan precursor	R.RLDFTGNLIEDIEDGTFSKLSLLEELSLAENQLLK.L	3	5.33	0.48	-1.11
IPI00025465	Mimecan precursor	V.PLNLPESLR.V	1	2.74	0.18	-4.12
IPI00025473	Beta-1,4 N-acetylgalactosaminyltransferase 1	R.SYQTNTADTVR.F	2	2.77	0.19	
IPI00025476	Pancreatic alpha-amylase precursor	K.IAEYM*NHLIDIGVAGFR.L	3	2.98	0.26	-1.59
IPI00025476	Pancreatic alpha-amylase precursor	K.IYVSDDGK.A	2	1.94	0.07	-1.69
IPI00025476	Pancreatic alpha-amylase precursor	K.TGSGDIENYNDATQVR.D	2	4.75	0.47	-2.44
IPI00025476	Pancreatic alpha-amylase precursor	R.YQPVSYK.L	1	1.65	0.16	-6.51
IPI00025499	Isoform Tau-F of Microtubule-associated protein tau	R.HLSNVSSTGSIDMVDSPQLATLADEVSASLAK.Q	3	4.95	0.33	-2.12
IPI00025622	AN1-type zinc finger protein 5	R.M*SPM*GTASGSNSPTSDSASVQR.A	3	3.60	0.42	-1.58
IPI00025647	Isoform 1 of F-box only protein 21	R.HPSLAFK.A	1	1.78	0.13	-3.83
IPI00025700	Isoform CD6A of T-cell differentiation antigen CD6 precursor	R.VTCAENR.A	2	2.15	0.05	1.74

IPI00025809	Alpha-1,6-mannosyl-glycoprotein 2-beta-N-acetylglucosaminyltransferase	K.FTVVAISPPRK.N	3	2.88	0.22	-3.45
IPI00025809	Alpha-1,6-mannosyl-glycoprotein 2-beta-N-acetylglucosaminyltransferase	K.NEALAPPLLDAEPAR.G	2	3.08	0.29	-2.25
IPI00025809	Alpha-1,6-mannosyl-glycoprotein 2-beta-N-acetylglucosaminyltransferase	K.VLVPQIPR.I	2	2.02	0.12	-2.00
IPI00025809	Alpha-1,6-mannosyl-glycoprotein 2-beta-N-acetylglucosaminyltransferase	R.SFYGM*ADKVDVK.T	2	2.32	0.12	-2.35
IPI00025812	Carbonic anhydrase-related protein 11 precursor	R.LLSQNPPSQIFQSLSGNSRPLQPLAHR.A	3	3.65	0.40	-4.41
IPI00025812	Carbonic anhydrase-related protein 11 precursor	R.LLSQNPPSQIFQSLSGNSRPLQPLAHR.A	4	4.43	0.40	-1.87
IPI00025812	Carbonic anhydrase-related protein 11 precursor	R.VLYDPFLPPLR.L	2	3.43	0.47	-3.54
IPI00025818	Isoform 1 of Polypeptide N-acetylgalactosaminyltransferase 1	K.ATEEDSQVPSIR.D	2	3.12	0.37	-2.85
IPI00025818	Isoform 1 of Polypeptide N-acetylgalactosaminyltransferase 1	K.GNQLWEYDPVK.L	2	2.66	0.18	-1.86
IPI00025818	Isoform 1 of Polypeptide N-acetylgalactosaminyltransferase 1	K.NFFYIISPGVTK.V	2	4.36	0.34	-2.33
IPI00025818	Isoform 1 of Polypeptide N-acetylgalactosaminyltransferase 1	K.VDYGDISSR.V	1	1.37	0.17	-4.64
IPI00025818	Isoform 1 of Polypeptide N-acetylgalactosaminyltransferase 1	K.VDYGDISSR.V	2	2.97	0.41	-2.93
IPI00025818	Isoform 1 of Polypeptide N-acetylgalactosaminyltransferase 1	R.HYFSLGEIR.N	2	2.38	0.24	-2.43
IPI00025818	Isoform 1 of Polypeptide N-acetylgalactosaminyltransferase 1	R.NVETNQCLDNM*AR.K	2	4.62	0.53	-3.39
IPI00025818	Isoform 1 of Polypeptide N-acetylgalactosaminyltransferase 1	R.TPTM*AGGLFSIDR.D	2	3.02	0.45	-3.90
IPI00025840	Isoform 1 of Ephrin-A1 precursor	K.ITHSPQAHDNPQEKR.L	3	2.42	0.12	-2.86
IPI00025840	Isoform 1 of Ephrin-A1 precursor	R.FTPFTLGK.E	1	1.87	0.29	-2.25
IPI00025840	Isoform 1 of Ephrin-A1 precursor	R.FTPFTLGK.E	2	1.49	0.14	-1.91
IPI00025840	Isoform 1 of Ephrin-A1 precursor	R.FTPFTLGKEFK.E	2	2.02	0.17	-3.13
IPI00025840	Isoform 1 of Ephrin-A1 precursor	R.LAADDPEVR.V	2	3.18	0.16	-2.95
IPI00025846	Isoform 2A of Desmocollin-2 precursor	K.ECFTAANLIHSSDPDFQILEDGSVYTTNTILLSSEKR.S	4	3.35	0.09	-1.47
IPI00025846	Isoform 2A of Desmocollin-2 precursor	K.IFVFLEHQTK.V	3	2.09	0.31	-2.47
IPI00025846	Isoform 2A of Desmocollin-2 precursor	R.GPGVDQEPR.N	2	2.74	0.31	-2.66
IPI00025846	Isoform 2A of Desmocollin-2 precursor	R.LSYQNDPPFGSYVVPITVR.D	2	5.10	0.48	-3.28
IPI00025846	Isoform 2A of Desmocollin-2 precursor	R.SFTILLSNTENQEK.K	2	3.96	0.39	-3.30
IPI00025846	Isoform 2A of Desmocollin-2 precursor	R.SFTILLSNTENQEKKK.I	3	1.88	0.14	-1.92
IPI00025864	Cholinesterase precursor	K.IFFPGVSEFGK.E	2	3.03	0.23	-3.89
IPI00025864	Cholinesterase precursor	K.NIAAFGGNPK.S	1	2.94	0.30	-1.99
IPI00025864	Cholinesterase precursor	K.NIAAFGGNPK.S	2	3.07	0.38	-1.47
IPI00025864	Cholinesterase precursor	K.TQILVGVNKDEGTAFLVYGAPGFSK.D	3	2.47	0.21	-1.67

IPI00025864	Cholinesterase precursor	K.YLTLNTESTR.I	1	2.33	0.18	-3.53
IPI00025864	Cholinesterase precursor	K.YLTLNTESTR.I	2	3.30	0.27	-1.08
IPI00025864	Cholinesterase precursor	R.AILQSGSFNAPWAVTSLYEAR.N	2	5.86	0.61	-1.84
IPI00025864	Cholinesterase precursor	R.AILQSGSFNAPWAVTSLYEAR.N	3	5.08	0.40	-1.58
IPI00025864	Cholinesterase precursor	R.EALGDVVGDYNFICPALEFTK.K	2	5.35	0.61	-4.28
IPI00025864	Cholinesterase precursor	R.EALGDVVGDYNFICPALEFTKK.F	2	3.87	0.54	-4.08
IPI00025869	Alpha-galactosidase A precursor	R.SYTIAVASLGK.G	2	3.43	0.42	-2.05
IPI00025992	Hepcidin precursor	G.SVFPQQTGQLAELQPQDR.A	2	4.31	0.44	-2.89
IPI00025992	Hepcidin precursor	G.SVFPQQTGQLAELQPQDR.A	3	4.94	0.30	-3.09
IPI00026031	Uncharacterized protein C6orf72 precursor	K.DVTEIDILVK.N	2	2.51	0.29	-3.32
IPI00026031	Uncharacterized protein C6orf72 precursor	R.ISCQTLIVK.N	2	2.40	0.13	-2.26
IPI00026050	Ceroid-lipofuscinosis neuronal protein 5	K.IM*HDAIGFR.S	2	1.92	0.14	-2.41
IPI00026050	Ceroid-lipofuscinosis neuronal protein 5	K.ITYEEIPLPIR.N	2	3.85	0.37	-3.47
IPI00026050	Ceroid-lipofuscinosis neuronal protein 5	K.LAEFGAEFK.N	1	2.44	0.26	-2.55
IPI00026050	Ceroid-lipofuscinosis neuronal protein 5	K.LAEFGAEFK.N	2	2.97	0.23	-1.65
IPI00026050	Ceroid-lipofuscinosis neuronal protein 5	K.YGDLLGHLK.I	2	3.28	0.32	-0.77
IPI00026050	Ceroid-lipofuscinosis neuronal protein 5	R.LQAPVWEFK.Y	2	2.84	0.29	-2.94
IPI00026103	ACHE protein	R.EALSDVVGDHNVVCPVAQLAGR.L	3	3.36	0.14	-2.18
IPI00026103	ACHE protein	R.EAPGNVGLLDQR.L	2	3.26	0.40	-3.25
IPI00026103	ACHE protein	R.FSFVPVVDGDFLSDTPEALINAGDFHGLQVLVGVVK.D	3	3.94	0.42	-2.87
IPI00026103	ACHE protein	R.LKTPGGPVSAFLGIPFAEPPM*GPR.R	3	3.18	0.18	-3.31
IPI00026103	ACHE protein	R.VGAFGFLALPGSR.E	2	3.42	0.36	-2.96
IPI00026103	ACHE protein	R.VGVPQVSDLAAEAVVLHYTDWLHPEDPAR.L	3	3.08	0.30	-4.81
IPI00026103	ACHE protein	R.VYAYVFEHR.A	2	2.43	0.30	-4.48
IPI00026104	Isoform Long of Iduronate 2-sulfatase precursor	K.ENGYVTM*SVGK.V	2	3.32	0.39	-3.94
IPI00026104	Isoform Long of Iduronate 2-sulfatase precursor	K.LFPYLDPFDSASQLM*EPGR.Q	2	5.27	0.49	-3.42
IPI00026104	Isoform Long of Iduronate 2-sulfatase precursor	K.LFPYLDPFDSASQLM*EPGR.Q	3	4.59	0.35	-7.20
IPI00026104	Isoform Long of Iduronate 2-sulfatase precursor	K.YSNFDVATHVPLIFYVPGR.T	2	5.18	0.60	-4.07
IPI00026104	Isoform Long of Iduronate 2-sulfatase precursor	K.YSNFDVATHVPLIFYVPGR.T	3	4.24	0.29	-7.05
IPI00026104	Isoform Long of Iduronate 2-sulfatase precursor	R.CPVPSFHVELCR.E	3	3.05	0.33	-1.34
IPI00026104	Isoform Long of Iduronate 2-sulfatase precursor	R.DLEEDPYLPGNPR.E	2	3.66	0.38	-4.22
IPI00026104	Isoform Long of Iduronate 2-sulfatase precursor	R.ELIAYSQYPR.P	2	3.02	0.44	-2.95
IPI00026104	Isoform Long of Iduronate 2-sulfatase precursor	R.FRDLEEDPYLPGNPR.E	2	4.23	0.33	-1.67
IPI00026104	Isoform Long of Iduronate 2-sulfatase precursor	R.FRDLEEDPYLPGNPR.E	3	3.12	0.14	-2.41
IPI00026104	Isoform Long of Iduronate 2-sulfatase precursor	R.GPDGELHANLLCPVDVLDVPEGTLPDKQSTEQAIQLLEK.M	3	6.41	0.56	-4.02
IPI00026104	Isoform Long of Iduronate 2-sulfatase precursor	R.GPDGELHANLLCPVDVLDVPEGTLPDKQSTEQAIQLLEK.M	4	5.48	0.48	-4.85
IPI00026104	Isoform Long of Iduronate 2-sulfatase precursor	R.QSM*DLVELVSLFPTLAGLAGLQVPPR.C	2	3.35	0.37	-1.83
IPI00026104	Isoform Long of Iduronate 2-sulfatase precursor	R.QSM*DLVELVSLFPTLAGLAGLQVPPR.C	3	4.70	0.53	-3.60
IPI00026104	Isoform Long of Iduronate 2-sulfatase precursor	R.SPNIDQLASHSLLFQNAFAQQAV.C	2	4.95	0.51	-4.27
IPI00026104	Isoform Long of Iduronate 2-sulfatase precursor	R.SPNIDQLASHSLLFQNAFAQQAV.C	3	3.64	0.34	-3.56
IPI00026104	Isoform Long of Iduronate 2-sulfatase precursor	R.TASLPEAGEK.L	1	1.43	0.12	-4.50
IPI00026104	Isoform Long of Iduronate 2-sulfatase precursor	R.TASLPEAGEKLFPYLDPFDSASQLM*EPGR.Q	3	6.26	0.52	-5.54

IPI00026104	Isoform Long of Iduronate 2-sulfatase precursor	R.TASLPEAGEKLFPYLDPFDSASQLM*EPGR.Q	4	4.47	0.16	-2.88
IPI00026125	Deoxyribonuclease I-like 1 precursor	R.EPFVAQFSLPSNVLPSLVLVPLHTTPK.A	3	4.08	0.38	-3.13
IPI00026154	Glucosidase 2 subunit beta precursor	E.EAPPPLSPPQPASPAEEDKM*PPYDEQTQAFIDAAQEAR.N	4	5.01	0.39	-3.67
IPI00026154	Glucosidase 2 subunit beta precursor	K.ETM*VTSTTEPSR.C	2	3.52	0.38	-3.41
IPI00026154	Glucosidase 2 subunit beta precursor	K.KLIELQAGKK.S	2	2.43	0.10	-4.06
IPI00026154	Glucosidase 2 subunit beta precursor	K.LIELQAGKK.S	1	2.20	0.06	-4.09
IPI00026154	Glucosidase 2 subunit beta precursor	K.LIELQAGKK.S	2	2.10	0.10	-1.93
IPI00026154	Glucosidase 2 subunit beta precursor	K.SLEDQVEM*LR.T	2	3.94	0.27	-3.33
IPI00026154	Glucosidase 2 subunit beta precursor	K.YEQGTGCWQGPNR.S	2	3.44	0.42	-2.67
IPI00026154	Glucosidase 2 subunit beta precursor	R.ESLQQM*AEVTR.E	2	2.76	0.21	-5.18
IPI00026154	Glucosidase 2 subunit beta precursor	R.NKFEEAER.S	1	2.66	0.14	-1.40
IPI00026154	Glucosidase 2 subunit beta precursor	R.SLKDM*EESIR.N	2	2.87	0.28	-2.89
IPI00026154	Glucosidase 2 subunit beta precursor	R.TVKEEAEKPER.E	2	3.48	0.09	-3.75
IPI00026154	Glucosidase 2 subunit beta precursor	R.TVKEEAEKPER.E	3	3.08	0.09	-3.26
IPI00026154	Glucosidase 2 subunit beta precursor	R.TVKEEAEKPEREAK.E	3	2.31	0.11	-3.79
IPI00026174	Cholecystokinins precursor	A.LTQPVPPADPAGSGLQR.A	2	3.25	0.45	-1.81
IPI00026174	Cholecystokinins precursor	G.ALTQPVPPADPAGSGLQR.A	2	4.34	0.39	-4.00
IPI00026174	Cholecystokinins precursor	L.TQPVPPADPAGSGLQR.A	2	2.95	0.37	-4.03
IPI00026174	Cholecystokinins precursor	R.AHLGALLAR.Y	1	1.90	0.19	-4.44
IPI00026174	Cholecystokinins precursor	R.AHLGALLAR.Y	2	2.36	0.22	-4.14
IPI00026197	Similar to Ig kappa chain V-IV region precursor	G.DIVM*TQSPDSLAVSLGER.A	2	5.76	0.42	
IPI00026197	Similar to Ig kappa chain V-IV region precursor	K.LLIYWASTR.E	2	2.79	0.24	
IPI00026197	Similar to Ig kappa chain V-IV region precursor	K.NYLAWYQQKPGQPPK.L	3	2.60	0.33	
IPI00026197	Similar to Ig kappa chain V-IV region precursor	Y.GDIVM*TQSPDSLAVSLGER.A	2	5.69	0.50	
IPI00026199	Glutathione peroxidase 3 precursor	K.FLVGPDGIPIM*R.W	2	3.45	0.32	-5.02
IPI00026199	Glutathione peroxidase 3 precursor	K.M*DILSYM*R.R	2	3.34	0.20	-1.63
IPI00026199	Glutathione peroxidase 3 precursor	K.NSCPPTSELLGTSDR.L	2	3.01	0.40	-6.52
IPI00026199	Glutathione peroxidase 3 precursor	K.QEPGENSEILPTLK.Y	2	2.34	0.13	-3.72
IPI00026199	Glutathione peroxidase 3 precursor	K.YVRPGGGFVPNFQLFEK.G	2	4.70	0.42	-5.67
IPI00026199	Glutathione peroxidase 3 precursor	K.YVRPGGGFVPNFQLFEK.G	3	3.78	0.41	-3.42
IPI00026199	Glutathione peroxidase 3 precursor	K.YVRPGGGFVPNFQLFEKGDVNGEK.E	3	4.97	0.38	-3.62
IPI00026199	Glutathione peroxidase 3 precursor	K.YVRPGGGFVPNFQLFEKGDVNGEKEQK.F	3	6.06	0.36	-4.28
IPI00026199	Glutathione peroxidase 3 precursor	K.YVRPGGGFVPNFQLFEKGDVNGEKEQK.F	4	4.82	0.45	-3.73
IPI00026199	Glutathione peroxidase 3 precursor	K.YVRPGGGFVPNFQLFEKGDVNGEKEQK.F	5	1.69	0.16	1.80
IPI00026199	Glutathione peroxidase 3 precursor	R.LFWEPM*K.V	2	2.16	0.07	-1.39
IPI00026199	Glutathione peroxidase 3 precursor	R.PGGGFVPNFQLFEK.G	2	4.01	0.39	-3.23
IPI00026199	Glutathione peroxidase 3 precursor	V.PNFQLFEK.G	2	3.12	0.20	-2.12
IPI00026199	Glutathione peroxidase 3 precursor	Y.VRPGGGFVPNFQLFEKGDVNGEKEQK.F	3	5.03	0.21	-1.68
IPI00026216	Puromycin-sensitive aminopeptidase	K.AFFESHPAPSAER.T	2	2.76	0.19	-5.26
IPI00026216	Puromycin-sensitive aminopeptidase	K.AFFESHPAPSAER.T	3	2.32	0.21	-2.00
IPI00026216	Puromycin-sensitive aminopeptidase	K.FALEVAAK.T	2	2.03	0.06	-3.15
IPI00026216	Puromycin-sensitive aminopeptidase	K.ILM*DKPEM*NVVLK.N	2	2.84	0.12	-0.51

IPI00026216	Puromycin-sensitive aminopeptidase	K.LNLGTVGFYR.T	2	3.54	0.26	-2.53
IPI00026216	Puromycin-sensitive aminopeptidase	K.QILSADLR.S	2	1.65	0.05	-2.33
IPI00026216	Puromycin-sensitive aminopeptidase	K.VLTFALSEEVRPQDTVSVIGGVAGGSK.H	3	4.43	0.34	-2.69
IPI00026216	Puromycin-sensitive aminopeptidase	R.AQELDALDNSHPIEVSVGHPSEVDEIFDAISYSK.G	3	7.18	0.63	-4.80
IPI00026216	Puromycin-sensitive aminopeptidase	R.AQELDALDNSHPIEVSVGHPSEVDEIFDAISYSK.G	4	3.87	0.35	-2.04
IPI00026216	Puromycin-sensitive aminopeptidase	R.DAESIHQYLLQR.K	2	2.84	0.28	-1.78
IPI00026216	Puromycin-sensitive aminopeptidase	R.LGLQNDLFSLAR.A	2	3.81	0.35	-3.62
IPI00026216	Puromycin-sensitive aminopeptidase	R.SKYTTPSGEVR.Y	2	2.95	0.40	-3.21
IPI00026216	Puromycin-sensitive aminopeptidase	R.VALSNM*NVIDR.K	2	2.83	0.30	-3.26
IPI00026216	Puromycin-sensitive aminopeptidase	R.VLGATLLPDLIQK.V	2	4.47	0.42	-3.27
IPI00026216	Puromycin-sensitive aminopeptidase	R.YAAVTQFEATDAR.R	2	4.86	0.42	-5.28
IPI00026230	Heterogeneous nuclear ribonucleoprotein H2	R.YVEVFKSNSVEMDWVLK.H	3	2.20	0.17	
IPI00026237	Myelin-associated glycoprotein precursor	K.SLELPFQGAHR.L	2	2.67	0.22	-2.92
IPI00026237	Myelin-associated glycoprotein precursor	R.DTVQCLCVVK.S	2	3.58	0.37	-2.33
IPI00026237	Myelin-associated glycoprotein precursor	R.FDFPDELRPAVVHGVWYFNSPYPK.N	3	2.95	0.13	-3.97
IPI00026237	Myelin-associated glycoprotein precursor	R.FDFPDELRPAVVHGVWYFNSPYPK.N	4	2.64	0.12	-2.91
IPI00026237	Myelin-associated glycoprotein precursor	R.LLGDLGLR.N	2	2.97	0.12	-4.21
IPI00026237	Myelin-associated glycoprotein precursor	R.SGLVLTSILTLR.G	2	3.39	0.26	-3.33
IPI00026240	ADP-ribosyl cyclase 2 precursor	K.DM*GFQYSCINDYRPVK.L	3	2.65	0.17	-1.70
IPI00026240	ADP-ribosyl cyclase 2 precursor	K.GFFADYEIPNLQK.E	2	4.36	0.42	-3.26
IPI00026240	ADP-ribosyl cyclase 2 precursor	R.FM*PLSDVLYGR.V	2	3.16	0.41	-3.58
IPI00026241	Bone marrow stromal antigen 2 precursor	K.LQDASAEVER.L	2	3.36	0.22	-2.70
IPI00026259	N	K.TGHIAAGTSTNGIK.F	2	3.49	0.40	-2.49
IPI00026259	N	R.FLPSYQAVEYM*R.R	2	3.28	0.38	-2.57
IPI00026259	N	R.VGDSPIPGAGAYADDTAGAAAATGNGDILM*R.F	3	4.42	0.46	-3.88
IPI00026262	Isoform 1 of Ras GTPase-activating protein 1	R.ATTLASTLM*EQYMK.A	3	1.98	0.14	-6.13
IPI00026270	Carboxypeptidase M precursor	K.ASLIEYIK.Q	2	2.64	0.18	-1.75
IPI00026270	Carboxypeptidase M precursor	K.GQVFDQNGNPLPNVIVEVQDR.K	3	5.88	0.44	-4.67
IPI00026270	Carboxypeptidase M precursor	K.GQVFDQNGNPLPNVIVEVQDRK.H	3	4.01	0.34	-1.63
IPI00026270	Carboxypeptidase M precursor	K.YVANM*HGDETVGR.E	2	3.73	0.38	-3.54
IPI00026270	Carboxypeptidase M precursor	R.ENYNQYDLNR.N	2	3.11	0.34	-2.70
IPI00026270	Carboxypeptidase M precursor	R.NLWVLVVGR.F	2	2.68	0.15	-1.49
IPI00026270	Carboxypeptidase M precursor	R.SLTPDDDVFQYLAHTYASR.N	2	3.64	0.27	-2.53
IPI00026285	Sia-alpha-2,3-Gal-beta-1,4-GlcNAc-R:alpha 2,8-sialyltransferase	K.YVFSISNNFR.S	2	2.91	0.33	-2.02
IPI00026285	Sia-alpha-2,3-Gal-beta-1,4-GlcNAc-R:alpha 2,8-sialyltransferase	K.YYNNLLTIQDR.N	2	3.35	0.39	-2.72
IPI00026285	Sia-alpha-2,3-Gal-beta-1,4-GlcNAc-R:alpha 2,8-sialyltransferase	R.CNFAPTEAFQR.D	2	3.07	0.35	-2.12
IPI00026299	Isoform Glycophorin C of Glycophorin-C	K.GTEFAESADAALQGDPALQDAGDSSR.K	3	3.44	0.41	-3.14
IPI00026314	Isoform 1 of Gelsolin precursor	D.PDQTDGLGLSYLSSHIANVER.V	3	4.86	0.52	-3.42
IPI00026314	Isoform 1 of Gelsolin precursor	E.PEAM*LQVLGPKPALPAGTEDTAKEDAANRK.L	3	3.89	0.36	-5.07

IPI00026314	Isoform 1 of Gelsolin precursor	E.VQGFESATFLGYFK.S	2	3.97	0.46	-3.75
IPI00026314	Isoform 1 of Gelsolin precursor	K.AALKTASDFITK.M	2	3.47	0.36	-3.46
IPI00026314	Isoform 1 of Gelsolin precursor	K.AALKTASDFITK.M	3	4.03	0.47	-3.31
IPI00026314	Isoform 1 of Gelsolin precursor	K.AGALNSNDAFVLK.T	1	2.57	0.21	-2.97
IPI00026314	Isoform 1 of Gelsolin precursor	K.AGALNSNDAFVLK.T	2	4.49	0.42	-5.57
IPI00026314	Isoform 1 of Gelsolin precursor	K.AGALNSNDAFVLKTPSAAYLWVGTGASEAEK.T	3	6.75	0.53	-3.84
IPI00026314	Isoform 1 of Gelsolin precursor	K.AGALNSNDAFVLKTPSAAYLWVGTGASEAEK.T	4	3.79	0.17	-3.51
IPI00026314	Isoform 1 of Gelsolin precursor	K.AGALNSNDAFVLKTPSAAYLWVGTGASEAEKTGAQELLR.V	3	6.34	0.51	-0.84
IPI00026314	Isoform 1 of Gelsolin precursor	K.AGALNSNDAFVLKTPSAAYLWVGTGASEAEKTGAQELLR.V	4	6.95	0.56	-4.80
IPI00026314	Isoform 1 of Gelsolin precursor	K.AGKEPGLQIWR.V	2	2.53	0.22	-1.08
IPI00026314	Isoform 1 of Gelsolin precursor	K.AGKEPGLQIWR.V	3	3.94	0.25	-3.55
IPI00026314	Isoform 1 of Gelsolin precursor	K.DSQEEEKTEALTSAK.R	2	4.75	0.45	-2.75
IPI00026314	Isoform 1 of Gelsolin precursor	K.DSQEEEKTEALTSAK.R	3	5.95	0.39	-2.84
IPI00026314	Isoform 1 of Gelsolin precursor	K.DSQEEEKTEALTSAKR.Y	2	4.87	0.44	-3.45
IPI00026314	Isoform 1 of Gelsolin precursor	K.DSQEEEKTEALTSAKR.Y	3	4.76	0.31	-3.10
IPI00026314	Isoform 1 of Gelsolin precursor	K.DSQEEEKTEALTSAKR.Y	4	2.79	0.15	-3.55
IPI00026314	Isoform 1 of Gelsolin precursor	K.EPAHLM*SLFGGKPM*IIYK.G	2	2.50	0.28	-3.57
IPI00026314	Isoform 1 of Gelsolin precursor	K.EPGLQIWR.V	1	1.64	0.13	-2.45
IPI00026314	Isoform 1 of Gelsolin precursor	K.EPGLQIWR.V	2	1.79	0.09	-1.40
IPI00026314	Isoform 1 of Gelsolin precursor	K.FDLVPVPTNLYGDFFTGDAYVILK.T	2	5.15	0.57	-6.20
IPI00026314	Isoform 1 of Gelsolin precursor	K.FDLVPVPTNLYGDFFTGDAYVILK.T	3	5.38	0.57	-5.06
IPI00026314	Isoform 1 of Gelsolin precursor	K.GGVASGFKHVVPNEVVVQR.L	2	4.50	0.46	-4.71
IPI00026314	Isoform 1 of Gelsolin precursor	K.GGVASGFKHVVPNEVVVQR.L	3	3.57	0.34	-2.95
IPI00026314	Isoform 1 of Gelsolin precursor	K.HVVPNEVVVQR.L	1	2.72	0.26	-3.48
IPI00026314	Isoform 1 of Gelsolin precursor	K.HVVPNEVVVQR.L	2	2.91	0.34	-3.14
IPI00026314	Isoform 1 of Gelsolin precursor	K.HVVPNEVVVQR.L	3	3.84	0.13	-4.79
IPI00026314	Isoform 1 of Gelsolin precursor	K.KGGVASGFKHVVPNEVVVQR.L	3	3.76	0.34	-3.72
IPI00026314	Isoform 1 of Gelsolin precursor	K.M*DYPKQTQVSVLPEGGETPLFK.Q	3	4.01	0.28	-3.69
IPI00026314	Isoform 1 of Gelsolin precursor	K.NWRDPDQTDGLGLS.Y	2	3.25	0.39	-3.90
IPI00026314	Isoform 1 of Gelsolin precursor	K.NWRDPDQTDGLGLSYLSSH.I	3	4.64	0.55	-2.91
IPI00026314	Isoform 1 of Gelsolin precursor	K.NWRDPDQTDGLGLSYLSSHIANVER.V	2	3.75	0.48	-4.53
IPI00026314	Isoform 1 of Gelsolin precursor	K.NWRDPDQTDGLGLSYLSSHIANVER.V	3	6.89	0.61	-0.08
IPI00026314	Isoform 1 of Gelsolin precursor	K.NWRDPDQTDGLGLSYLSSHIANVER.V	4	5.23	0.53	-3.97
IPI00026314	Isoform 1 of Gelsolin precursor	K.QGFEPPSFVGWFLGWDDDYWSVDPLDR.A	2	2.65	0.53	-2.17
IPI00026314	Isoform 1 of Gelsolin precursor	K.QGFEPPSFVGWFLGWDDDYWSVDPLDR.A	3	4.07	0.41	-5.95
IPI00026314	Isoform 1 of Gelsolin precursor	K.QTQVSVLPEGGETPLFK.Q	2	4.46	0.47	-4.36
IPI00026314	Isoform 1 of Gelsolin precursor	K.QTQVSVLPEGGETPLFK.Q	3	4.08	0.38	-2.49
IPI00026314	Isoform 1 of Gelsolin precursor	K.RYIETDPANR.D	2	2.13	0.13	-3.27
IPI00026314	Isoform 1 of Gelsolin precursor	K.RYIETDPANRDR.R	2	1.94	0.15	-3.83
IPI00026314	Isoform 1 of Gelsolin precursor	K.SEDCFILDHGK.D	2	3.23	0.36	
IPI00026314	Isoform 1 of Gelsolin precursor	K.SEDCFILDHGK.D	3	1.90	0.22	-1.15
IPI00026314	Isoform 1 of Gelsolin precursor	K.SEDCFILDHGKDGK.I	2	3.59	0.41	-3.81

IPI00026314	Isoform 1 of Gelsolin precursor	K.SEDCFILDHGKDGK.I	3	2.48	0.23	-2.04
IPI00026314	Isoform 1 of Gelsolin precursor	K.TASDFITK.M	1	2.33	0.13	-3.24
IPI00026314	Isoform 1 of Gelsolin precursor	K.TASDFITK.M	2	3.01	0.39	-4.23
IPI00026314	Isoform 1 of Gelsolin precursor	K.TASDFITKM*DYPK.Q	2	3.04	0.09	
IPI00026314	Isoform 1 of Gelsolin precursor	K.TEALTSAKR.Y	2	2.80	0.24	-1.36
IPI00026314	Isoform 1 of Gelsolin precursor	K.TGAQELLR.V	1	1.94	0.07	-2.17
IPI00026314	Isoform 1 of Gelsolin precursor	K.TGAQELLR.V	2	3.29	0.18	-2.62
IPI00026314	Isoform 1 of Gelsolin precursor	K.TPSAAYLWVGTGASEAEK.T	2	6.47	0.54	-7.39
IPI00026314	Isoform 1 of Gelsolin precursor	K.TPSAAYLWVGTGASEAEK.T	3	3.99	0.36	-3.40
IPI00026314	Isoform 1 of Gelsolin precursor	K.TPSAAYLWVGTGASEAEKTGAQELLR.V	2	5.13	0.49	-1.46
IPI00026314	Isoform 1 of Gelsolin precursor	K.TPSAAYLWVGTGASEAEKTGAQELLR.V	3	6.36	0.61	-4.13
IPI00026314	Isoform 1 of Gelsolin precursor	K.TPSAAYLWVGTGASEAEKTGAQELLR.V	4	4.17	0.43	-3.10
IPI00026314	Isoform 1 of Gelsolin precursor	K.VPVDPATYGQFYGGDSYIILYNYR.H	2	4.93	0.57	-3.85
IPI00026314	Isoform 1 of Gelsolin precursor	K.VPVDPATYGQFYGGDSYIILYNYR.H	3	5.26	0.50	-5.15
IPI00026314	Isoform 1 of Gelsolin precursor	K.VSNGAGTM*SVSLVADENPFAQGALK.S	2	5.39	0.53	-4.15
IPI00026314	Isoform 1 of Gelsolin precursor	K.VSNGAGTM*SVSLVADENPFAQGALK.S	3	6.19	0.42	-5.56
IPI00026314	Isoform 1 of Gelsolin precursor	K.VSNGAGTM*SVSLVADENPFAQGALKSEDCFILDHGK.D	3	2.97	0.14	-2.06
IPI00026314	Isoform 1 of Gelsolin precursor	K.VSNGAGTM*SVSLVADENPFAQGALKSEDCFILDHGK.D	4	5.34	0.46	-2.67
IPI00026314	Isoform 1 of Gelsolin precursor	K.VSNGAGTM*SVSLVADENPFAQGALKSEDCFILDHGKDGK.I	4	3.41	0.17	-1.20
IPI00026314	Isoform 1 of Gelsolin precursor	L.GLSYLSSHIANVER.V	2	3.70	0.41	-1.87
IPI00026314	Isoform 1 of Gelsolin precursor	Q.GAQSTQDEVAASAILTAQLDEELGGTPVQSR.V	3	5.59	0.52	-4.02
IPI00026314	Isoform 1 of Gelsolin precursor	Q.VLGPKPALPAGTEDTAKEDAANRK.L	3	4.43	0.43	-1.00
IPI00026314	Isoform 1 of Gelsolin precursor	R.AM*AELAA.-	1	1.63	0.08	-2.59
IPI00026314	Isoform 1 of Gelsolin precursor	R.AQPVQVAEGS.E	1	2.17	0.22	-1.54
IPI00026314	Isoform 1 of Gelsolin precursor	R.AQPVQVAEGSEPDGFWEALGGK.A	2	7.39	0.61	-4.26
IPI00026314	Isoform 1 of Gelsolin precursor	R.AQPVQVAEGSEPDGFWEALGGK.A	3	5.93	0.50	-4.09
IPI00026314	Isoform 1 of Gelsolin precursor	R.AQPVQVAEGSEPDGFWEALGGKAAYR.T	3	3.20	0.42	-3.63
IPI00026314	Isoform 1 of Gelsolin precursor	R.ARVHVSEEGTEPEAM*LQVLGPKPALPAGTEDTAKEDAANR.K	4	6.02	0.56	0.06
IPI00026314	Isoform 1 of Gelsolin precursor	R.ARVHVSEEGTEPEAM*LQVLGPKPALPAGTEDTAKEDAANR.K	5	3.51	0.37	-1.37
IPI00026314	Isoform 1 of Gelsolin precursor	R.ARVHVSEEGTEPEAM*LQVLGPKPALPAGTEDTAKEDAANRK.L	4	5.37	0.46	-3.15
IPI00026314	Isoform 1 of Gelsolin precursor	R.ARVHVSEEGTEPEAM*LQVLGPKPALPAGTEDTAKEDAANRK.L	5	2.56	0.21	-3.55
IPI00026314	Isoform 1 of Gelsolin precursor	R.ARVHVSEEGTEPEAM*LQVLGPKPALPAGTEDTAKEDAANRK.L	6	3.26	0.35	-3.09
IPI00026314	Isoform 1 of Gelsolin precursor	R.ATEVPVSWESFNNGDCFILDLGNNIHQWCGSNSNR.Y	3	4.89	0.55	-4.91
IPI00026314	Isoform 1 of Gelsolin precursor	R.ATEVPVSWESFNNGDCFILDLGNNIHQWCGSNSNR.Y	4	5.60	0.49	-2.98
IPI00026314	Isoform 1 of Gelsolin precursor	R.ATEVPVSWESFNNGDCFILDLGNNIHQWCGSNSNRYER.L	4	4.03	0.32	-2.87
IPI00026314	Isoform 1 of Gelsolin precursor	R.AVEVLPKAGALNSNDAFVLK.T	2	4.28	0.57	-2.30
IPI00026314	Isoform 1 of Gelsolin precursor	R.AVEVLPKAGALNSNDAFVLK.T	3	3.88	0.46	-2.45
IPI00026314	Isoform 1 of Gelsolin precursor	R.AVQHREVQGFESATFLGYFK.S	2	4.87	0.53	-4.46
IPI00026314	Isoform 1 of Gelsolin precursor	R.AVQHREVQGFESATFLGYFK.S	3	4.96	0.55	-3.36
IPI00026314	Isoform 1 of Gelsolin precursor	R.AVQHREVQGFESATFLGYFK.S	4	2.83	0.20	-2.54
IPI00026314	Isoform 1 of Gelsolin precursor	R.DPDQTDGLGLSYLSSH.I	2	5.32	0.59	-1.48
IPI00026314	Isoform 1 of Gelsolin precursor	R.DPDQTDGLGLSYLSSHIANVER.V	2	4.98	0.55	-5.04

IPI00026314	Isoform 1 of Gelsolin precursor	R.DPDQTDGLGLSYLSSHIANVER.V	3	4.47	0.54	-3.92
IPI00026314	Isoform 1 of Gelsolin precursor	R.EGGQTAPASTR.L	2	3.48	0.31	-2.71
IPI00026314	Isoform 1 of Gelsolin precursor	R.EGGQTAPASTRLFQVRANSAGATR.A	3	2.14	0.13	-1.91
IPI00026314	Isoform 1 of Gelsolin precursor	R.EVQGFESATFLGYFK.S	1	2.39	0.52	-2.17
IPI00026314	Isoform 1 of Gelsolin precursor	R.EVQGFESATFLGYFK.S	2	5.75	0.57	-7.42
IPI00026314	Isoform 1 of Gelsolin precursor	R.EVQGFESATFLGYFK.S	3	2.84	0.39	-2.90
IPI00026314	Isoform 1 of Gelsolin precursor	R.IEGSNKVPVDPAT.Y	2	3.03	0.31	2.10
IPI00026314	Isoform 1 of Gelsolin precursor	R.IEGSNKVPVDPATYGQF.Y	2	3.34	0.42	-3.47
IPI00026314	Isoform 1 of Gelsolin precursor	R.IEGSNKVPVDPATYGQFYGGDSYIIL.Y	2	3.49	0.45	-2.36
IPI00026314	Isoform 1 of Gelsolin precursor	R.IEGSNKVPVDPATYGQFYGGDSYIIL.Y	3	3.58	0.39	-3.23
IPI00026314	Isoform 1 of Gelsolin precursor	R.IEGSNKVPVDPATYGQFYGGDSYIILYNYR.H	2	3.06	0.51	-3.37
IPI00026314	Isoform 1 of Gelsolin precursor	R.IEGSNKVPVDPATYGQFYGGDSYIILYNYR.H	3	6.13	0.60	-7.65
IPI00026314	Isoform 1 of Gelsolin precursor	R.IEGSNKVPVDPATYGQFYGGDSYIILYNYR.H	4	4.15	0.31	-3.43
IPI00026314	Isoform 1 of Gelsolin precursor	R.LFACSNK.I	1	2.02	0.09	-2.52
IPI00026314	Isoform 1 of Gelsolin precursor	R.LFQVRANSAGATR.A	2	3.10	0.18	0.01
IPI00026314	Isoform 1 of Gelsolin precursor	R.LFQVRANSAGATR.A	3	2.15	0.29	-2.34
IPI00026314	Isoform 1 of Gelsolin precursor	R.PNSM*VVEHPEFLK.A	2	3.19	0.20	-2.39
IPI00026314	Isoform 1 of Gelsolin precursor	R.QGQIIYNWQGAQSTQDEVAASAILTAQLDEELGGTPVQSR.V	3	5.65	0.56	-4.80
IPI00026314	Isoform 1 of Gelsolin precursor	R.QGQIIYNWQGAQSTQDEVAASAILTAQLDEELGGTPVQSR.V	4	5.41	0.44	-6.75
IPI00026314	Isoform 1 of Gelsolin precursor	R.TPITVVK.Q	1	1.94	0.11	-2.51
IPI00026314	Isoform 1 of Gelsolin precursor	R.VEKFDLVPVPTNLYGDFFTGDAYVILK.T	2	4.43	0.54	-4.60
IPI00026314	Isoform 1 of Gelsolin precursor	R.VEKFDLVPVPTNLYGDFFTGDAYVILK.T	3	5.44	0.51	-6.49
IPI00026314	Isoform 1 of Gelsolin precursor	R.VEKFDLVPVPTNLYGDFFTGDAYVILK.T	4	3.77	0.22	-4.08
IPI00026314	Isoform 1 of Gelsolin precursor	R.VHVSEEGTEPEAM*LQVLGPK.P	2	5.43	0.55	-2.72
IPI00026314	Isoform 1 of Gelsolin precursor	R.VHVSEEGTEPEAM*LQVLGPK.P	3	3.83	0.49	-3.16
IPI00026314	Isoform 1 of Gelsolin precursor	R.VHVSEEGTEPEAM*LQVLGPKPALPAGTEDTAK.E	3	5.67	0.50	-0.19
IPI00026314	Isoform 1 of Gelsolin precursor	R.VHVSEEGTEPEAM*LQVLGPKPALPAGTEDTAK.E	4	4.34	0.47	-4.95
IPI00026314	Isoform 1 of Gelsolin precursor	R.VHVSEEGTEPEAM*LQVLGPKPALPAGTEDTAKEDAANR.K	3	5.20	0.58	-3.51
IPI00026314	Isoform 1 of Gelsolin precursor	R.VHVSEEGTEPEAM*LQVLGPKPALPAGTEDTAKEDAANR.K	4	6.02	0.53	-3.73
IPI00026314	Isoform 1 of Gelsolin precursor	R.VHVSEEGTEPEAM*LQVLGPKPALPAGTEDTAKEDAANR.K	5	2.48	0.40	-3.26
IPI00026314	Isoform 1 of Gelsolin precursor	R.VHVSEEGTEPEAM*LQVLGPKPALPAGTEDTAKEDAANRK.L	3	5.54	0.55	-5.03
IPI00026314	Isoform 1 of Gelsolin precursor	R.VHVSEEGTEPEAM*LQVLGPKPALPAGTEDTAKEDAANRK.L	4	5.90	0.51	-4.75
IPI00026314	Isoform 1 of Gelsolin precursor	R.VHVSEEGTEPEAM*LQVLGPKPALPAGTEDTAKEDAANRK.L	5	2.57	0.32	-3.40
IPI00026314	Isoform 1 of Gelsolin precursor	R.VPEARPNSM*VVEHPEFLK.A	2	2.73	0.27	-4.38
IPI00026314	Isoform 1 of Gelsolin precursor	R.VPEARPNSM*VVEHPEFLK.A	3	4.96	0.37	-3.98
IPI00026314	Isoform 1 of Gelsolin precursor	R.VPEARPNSM*VVEHPEFLK.A	4	2.19	0.27	-3.42
IPI00026314	Isoform 1 of Gelsolin precursor	R.VPFDAATLHTSTAM*.A	2	3.96	0.29	-7.65
IPI00026314	Isoform 1 of Gelsolin precursor	R.VPFDAATLHTSTAM*AAQHGM*DDDGTGQK.Q	2	4.55	0.56	-1.26
IPI00026314	Isoform 1 of Gelsolin precursor	R.VPFDAATLHTSTAM*AAQHGM*DDDGTGQK.Q	3	6.06	0.60	-3.47
IPI00026314	Isoform 1 of Gelsolin precursor	R.VPFDAATLHTSTAM*AAQHGM*DDDGTGQK.Q	4	4.32	0.48	-2.78
IPI00026314	Isoform 1 of Gelsolin precursor	R.VPFDAATLHTSTAM*AAQHGM*DDDGTGQKQIWR.I	4	4.15	0.41	-2.59
IPI00026314	Isoform 1 of Gelsolin precursor	R.VVQGKEPAHLM*SLFGGK.P	2	3.16	0.44	-3.59

IPI00026314	Isoform 1 of Gelsolin precursor	R.VVQGKEPAHLM*SLFGGKPM*IIYK.G	3	3.94	0.42	-2.67
IPI00026314	Isoform 1 of Gelsolin precursor	R.YIETDPANR.D	1	2.14	0.26	-3.85
IPI00026314	Isoform 1 of Gelsolin precursor	R.YIETDPANR.D	2	3.04	0.32	-2.90
IPI00026314	Isoform 1 of Gelsolin precursor	R.YIETDPANRDR.R	2	1.93	0.16	-2.57
IPI00026314	Isoform 1 of Gelsolin precursor	R.YIETDPANRDR.R	3	1.77	0.21	-2.74
IPI00026314	Isoform 1 of Gelsolin precursor	R.YIETDPANRDRR.T	3	2.23	0.14	-0.69
IPI00026314	Isoform 1 of Gelsolin precursor	V.GTGASEAEKTGAQELLR.V	3	4.03	0.29	-1.18
IPI00026314	Isoform 1 of Gelsolin precursor	V.PEARPNSM*VVEHPEFLK.A	3	4.19	0.26	-3.03
IPI00026314	Isoform 1 of Gelsolin precursor	V.PFDAATLHTSTAM*AAQHGM*DDDGTGQK.Q	3	4.59	0.51	-0.45
IPI00026314	Isoform 1 of Gelsolin precursor	V.PNEVVVQR.L	2	3.16	0.18	0.81
IPI00026314	Isoform 1 of Gelsolin precursor	V.PVPTNLYGDFFTGDAYVILK.T	2	2.95	0.38	-1.68
IPI00026314	Isoform 1 of Gelsolin precursor	V.VPNEVVVQR.L	1	2.56	0.23	-1.41
IPI00026314	Isoform 1 of Gelsolin precursor	W.QGAQSTQDEVAASAILTAQLDEELGGTPVQSR.V	2	4.11	0.47	-1.00
IPI00026314	Isoform 1 of Gelsolin precursor	W.QGAQSTQDEVAASAILTAQLDEELGGTPVQSR.V	3	5.41	0.53	-7.89
IPI00026314	Isoform 1 of Gelsolin precursor	W.RDPDQTDGLGLSYLSSHIANVER.V	2	3.96	0.43	-4.51
IPI00026314	Isoform 1 of Gelsolin precursor	W.RDPDQTDGLGLSYLSSHIANVER.V	3	6.56	0.54	-7.32
IPI00026314	Isoform 1 of Gelsolin precursor	W.RDPDQTDGLGLSYLSSHIANVER.V	4	4.64	0.49	-3.36
IPI00026314	Isoform 1 of Gelsolin precursor	Y.LSSHIANVER.V	1	2.71	0.26	-4.21
IPI00026314	Isoform 1 of Gelsolin precursor	Y.LSSHIANVER.V	2	3.23	0.18	-1.94
IPI00026358	Gamma-aminobutyric acid receptor-associated protein-like 2	K.AIFLFVDK.T	2	2.75	0.10	-3.12
IPI00026530	Protein ERGIC-53 precursor	K.GHPDLQGQPAEEIFESVGDR.E	3	5.13	0.41	-2.83
IPI00026546	Platelet-activating factor acetylhydrolase IB subunit beta	K.DKEPDVLFVGDSMVQLMQQYEIWR.E	3	4.80	0.22	-2.65
IPI00026546	Platelet-activating factor acetylhydrolase IB subunit beta	R.ELFSPLHALNFGIGGDTTR.H	3	3.19	0.08	-3.43
IPI00026570	Cytochrome c oxidase polypeptide VIIa-liver/heart, mitochondrial precursor	R.ATM*ILTVGGTAYAIYELAVASFPKK.Q	3	2.91	0.11	
IPI00026612	Isoform Beta-1 of Protein phosphatase 1B	S.TAVGVMISPK.H	2	3.22	0.16	0.77
IPI00026665	Glutaminyl-tRNA synthetase	R.MKLVM*EDGKM*DPVAYRVKYTPHHR.T	3	3.15	0.17	
IPI00026800	Scrapie-responsive protein 1 precursor	K.DVFFGPK.I	1	2.18	0.16	-3.45
IPI00026800	Scrapie-responsive protein 1 precursor	K.DVFFGPK.I	2	2.15	0.16	-2.86
IPI00026800	Scrapie-responsive protein 1 precursor	K.GCEMICYCNFSELLCCPK.D	2	3.76	0.30	
IPI00026800	Scrapie-responsive protein 1 precursor	K.GCEMICYCNFSELLCCPK.D	3	3.41	0.13	
IPI00026944	Isoform 1 of Nidogen-1 precursor	C.LSRQELFPFGPGQGDLELEDGDDFVSPALELSGALR.F	3	7.07	0.58	-3.86
IPI00026944	Isoform 1 of Nidogen-1 precursor	K.AFLHVPAK.V	2	2.41	0.09	-2.92
IPI00026944	Isoform 1 of Nidogen-1 precursor	K.ALEGLQYPFAVTSYGK.N	2	4.93	0.55	-4.69
IPI00026944	Isoform 1 of Nidogen-1 precursor	K.ESHPGLFPPTFGAVAPFLADLDTTDGLGK.V	3	5.87	0.56	-4.74
IPI00026944	Isoform 1 of Nidogen-1 precursor	K.ETDAFQPHKQTR.L	2	2.79	0.32	-2.24
IPI00026944	Isoform 1 of Nidogen-1 precursor	K.IETSYM*DGTNR.R	2	2.55	0.23	-2.29
IPI00026944	Isoform 1 of Nidogen-1 precursor	K.M*VYWTDITEPSIGR.A	2	4.28	0.40	-2.60
IPI00026944	Isoform 1 of Nidogen-1 precursor	K.NGFSITGGEFTR.Q	2	2.97	0.28	-4.62

IPI00026944	Isoform 1 of Nidogen-1 precursor	K.VIIGLAFDCVDK.M	2	3.99	0.45	-3.02
IPI00026944	Isoform 1 of Nidogen-1 precursor	K.VYYREDLSPSITQR.A	2	4.43	0.45	-2.56
IPI00026944	Isoform 1 of Nidogen-1 precursor	R.AECLNPSQPSR.R	2	3.02	0.24	-1.38
IPI00026944	Isoform 1 of Nidogen-1 precursor	R.ASLHGGEPTTIIRQDLGSPEGIAVDHLGR.N	4	5.50	0.51	-3.12
IPI00026944	Isoform 1 of Nidogen-1 precursor	R.EDLSPSITQR.A	2	2.89	0.22	-3.61
IPI00026944	Isoform 1 of Nidogen-1 precursor	R.EYTVTEPER.D	2	2.29	0.27	-2.63
IPI00026944	Isoform 1 of Nidogen-1 precursor	R.EYTVTEPERDGASPSR.I	3	2.35	0.12	-1.10
IPI00026944	Isoform 1 of Nidogen-1 precursor	R.FYDRSDIDAVYVTTNGIIATSEPPAK.E	3	3.17	0.33	0.39
IPI00026944	Isoform 1 of Nidogen-1 precursor	R.GFPEISFQPSSAVVVTWESVAPYQGPSRDPDQK.G	3	3.34	0.24	-5.37
IPI00026944	Isoform 1 of Nidogen-1 precursor	R.GFPEISFQPSSAVVVTWESVAPYQGPSRDPDQK.G	4	3.34	0.11	-6.30
IPI00026944	Isoform 1 of Nidogen-1 precursor	R.GGADTYSVPSVLSPR.R	2	2.56	0.13	-1.97
IPI00026944	Isoform 1 of Nidogen-1 precursor	R.GIVTDSVR.G	2	2.14	0.17	-2.13
IPI00026944	Isoform 1 of Nidogen-1 precursor	R.GNLYWTDWNR.D	2	2.70	0.29	-1.23
IPI00026944	Isoform 1 of Nidogen-1 precursor	R.IFVGSSQVPIVFENTDLHSYVVM*NHGR.S	4	2.55	0.11	-3.84
IPI00026944	Isoform 1 of Nidogen-1 precursor	R.KALEGLQYPFAVTSYGK.N	2	5.18	0.53	-4.19
IPI00026944	Isoform 1 of Nidogen-1 precursor	R.QAEVTFVGHPGNLVIK.Q	3	2.13	0.14	-2.55
IPI00026944	Isoform 1 of Nidogen-1 precursor	R.QDLGSPEGIAVDHLGR.N	2	2.67	0.30	-2.05
IPI00026944	Isoform 1 of Nidogen-1 precursor	R.QDLGSPEGIAVDHLGR.N	3	2.23	0.16	-0.35
IPI00026944	Isoform 1 of Nidogen-1 precursor	R.QELFPFGPGQGDLELEDGDDFVSPALELSGALR.F	2	2.13	0.37	-2.51
IPI00026944	Isoform 1 of Nidogen-1 precursor	R.QELFPFGPGQGDLELEDGDDFVSPALELSGALR.F	3	5.66	0.49	-3.14
IPI00026944	Isoform 1 of Nidogen-1 precursor	R.QELFPFGPGQGDLELEDGDDFVSPALELSGALR.F	4	4.02	0.39	-2.68
IPI00026944	Isoform 1 of Nidogen-1 precursor	R.RGGADTYSVPSVLSPR.R	2	3.25	0.32	-3.12
IPI00026944	Isoform 1 of Nidogen-1 precursor	R.RVLFETDLVNPR.G	2	3.12	0.35	-4.36
IPI00026944	Isoform 1 of Nidogen-1 precursor	R.RVLFETDLVNPR.G	3	2.48	0.21	-2.61
IPI00026944	Isoform 1 of Nidogen-1 precursor	R.SDIDAVYVTTNGIIATSEPPAK.E	3	3.28	0.06	0.46
IPI00026944	Isoform 1 of Nidogen-1 precursor	R.SFQLAVETFHQQ.H	2	3.81	0.33	-5.92
IPI00026944	Isoform 1 of Nidogen-1 precursor	R.TQFTCECSIGFR.G	2	3.78	0.43	-3.06
IPI00026944	Isoform 1 of Nidogen-1 precursor	R.VLFETDLVNPR.G	2	2.97	0.39	-3.10
IPI00026944	Isoform 1 of Nidogen-1 precursor	R.VPQIPFGSSVHIEPYTELYHYSTSVITSSSTR.E	4	2.86	0.17	-2.70
IPI00026946	Neuronal pentraxin-2 precursor	H.PIKPGGVLILGQEQDTVGGR.F	3	4.83	0.40	-2.01
IPI00026946	Neuronal pentraxin-2 precursor	K.DRLESLEHQLR.A	2	3.27	0.23	-0.75
IPI00026946	Neuronal pentraxin-2 precursor	K.DRLESLEHQLR.A	3	3.27	0.23	-1.41
IPI00026946	Neuronal pentraxin-2 precursor	K.DTM*GDLPR.D	2	2.20	0.21	-3.30
IPI00026946	Neuronal pentraxin-2 precursor	K.SPDAFKVSLPLR.T	3	4.11	0.30	-3.55
IPI00026946	Neuronal pentraxin-2 precursor	K.TESTLNALLQR.V	2	3.95	0.29	-2.86
IPI00026946	Neuronal pentraxin-2 precursor	K.VAQLPLFVSDGK.W	2	3.27	0.35	-2.73
IPI00026946	Neuronal pentraxin-2 precursor	K.WPVETCEER.L	2	2.71	0.29	-3.79
IPI00026946	Neuronal pentraxin-2 precursor	P.M*QGGAQSPEEELR.A	2	4.17	0.41	-2.82
IPI00026946	Neuronal pentraxin-2 precursor	R.DGM*WEAFQDGEK.L	2	3.78	0.38	-2.79
IPI00026946	Neuronal pentraxin-2 precursor	R.EAIRELTGK.L	1	2.05	0.18	-3.89
IPI00026946	Neuronal pentraxin-2 precursor	R.FDATQAFVGELSQFNIWDR.V	2	5.83	0.51	-4.65
IPI00026946	Neuronal pentraxin-2 precursor	R.GAGATGKDTM*GDLPR.D	2	2.69	0.21	-1.98

IPI00026946	Neuronal pentraxin-2 precursor	R.GNSAFKSPDAFK.V	2	2.84	0.28	-2.16
IPI00026946	Neuronal pentraxin-2 precursor	R.LESLEHQLR.A	2	3.33	0.23	-2.71
IPI00026946	Neuronal pentraxin-2 precursor	R.QKTESTLNALLQR.V	2	2.76	0.42	-4.01
IPI00026946	Neuronal pentraxin-2 precursor	R.SLQTLKDR.L	2	2.08	0.06	-0.51
IPI00026991	Polypeptide N-acetylgalactosaminyltransferase 6	K.SPTFAGGLFSISK.S	2	3.73	0.44	-3.33
IPI00026991	Polypeptide N-acetylgalactosaminyltransferase 6	R.IAEDKTVVVSPDIVTIDLNTFEFAKPVQR.G	4	4.70	0.37	-2.14
IPI00026991	Polypeptide N-acetylgalactosaminyltransferase 6	R.TVYSVLHTTPAILLK.E	2	3.07	0.34	-3.48
IPI00027009	Isoform 1 of Protein kinase C and casein kinase substrate in neurons protein 2	K.QMM*GGFK.E	2	1.57	0.16	
IPI00027038	Isoform 1 of V-set and immunoglobulin domain-containing protein 4 precursor	K.GDVNLPCTYDPLQGYTQVLVK.W	2	4.65	0.54	-4.33
IPI00027038	Isoform 1 of V-set and immunoglobulin domain-containing protein 4 precursor	K.GDVNLPCTYDPLQGYTQVLVK.W	3	2.55	0.16	-5.63
IPI00027038	Isoform 1 of V-set and immunoglobulin domain-containing protein 4 precursor	K.GQVGSEQHSDIVK.F	2	3.65	0.34	-3.62
IPI00027038	Isoform 1 of V-set and immunoglobulin domain-containing protein 4 precursor	K.LSVSKPTVTTGSGYGFTVPQGM*R.I	2	4.46	0.50	-3.82
IPI00027038	Isoform 1 of V-set and immunoglobulin domain-containing protein 4 precursor	K.VPGDVSLQLSTLEM*DDR.S	2	4.17	0.43	-3.85
IPI00027038	Isoform 1 of V-set and immunoglobulin domain-containing protein 4 precursor	R.DKITELR.V	1	2.02	0.14	-2.88
IPI00027038	Isoform 1 of V-set and immunoglobulin domain-containing protein 4 precursor	R.DSSGDHIQQAK.Y	2	2.76	0.28	0.68
IPI00027038	Isoform 1 of V-set and immunoglobulin domain-containing protein 4 precursor	R.GSDPVTIFLR.D	2	3.05	0.32	-3.58
IPI00027038	Isoform 1 of V-set and immunoglobulin domain-containing protein 4 precursor	R.GSPPISYIWYK.Q	2	1.76	0.10	-4.56
IPI00027038	Isoform 1 of V-set and immunoglobulin domain-containing protein 4 precursor	R.SHYTCEVTWQTPDGNQVVR.D	2	4.47	0.50	-1.64
IPI00027038	Isoform 1 of V-set and immunoglobulin domain-containing protein 4 precursor	R.SHYTCEVTWQTPDGNQVVR.D	3	4.14	0.44	-2.10
IPI00027078	Carboxypeptidase D precursor	K.LVGNM*HGDETVSR.Q	2	3.62	0.43	-3.46
IPI00027078	Carboxypeptidase D precursor	K.SQEGDSISVIGR.N	2	3.11	0.32	-2.77
IPI00027078	Carboxypeptidase D precursor	R.EAAAAGLPGLAR.L	2	2.81	0.26	-1.61
IPI00027078	Carboxypeptidase D precursor	R.GFVLDATDGR.G	2	2.55	0.32	-3.03
IPI00027078	Carboxypeptidase D precursor	R.LTAGLGSLIPEGDAGPDAAGPDAAGPLLPGRPQVK.L	3	4.10	0.49	-5.16
IPI00027078	Carboxypeptidase D precursor	R.NKFVLSGNLHGGSVVASYPFDDSPEHK.A	4	3.36	0.27	-4.67
IPI00027087	Isoform 1 of Neural cell adhesion molecule L1 precursor	A.VQGSTAYLLCK.A	2	2.95	0.40	-1.53
IPI00027087	Isoform 1 of Neural cell adhesion molecule L1 precursor	K.AFGAPVPSVQWLDEDGTTVLQDER.F	2	5.67	0.57	-5.12

IPI00027087	Isoform 1 of Neural cell adhesion molecule L1 precursor	K.AFGAPVPSVQWLDEDGTTVLQDER.F	3	4.31	0.43	-4.08
IPI00027087	Isoform 1 of Neural cell adhesion molecule L1 precursor	K.ATNSM*IDR.K	2	2.62	0.39	-2.80
IPI00027087	Isoform 1 of Neural cell adhesion molecule L1 precursor	K.DATQITQGPR.S	2	2.41	0.17	-3.44
IPI00027087	Isoform 1 of Neural cell adhesion molecule L1 precursor	K.ETVKPVEVEEGESVVLPCNPPPSAEPLR.I	3	4.57	0.37	-2.08
IPI00027087	Isoform 1 of Neural cell adhesion molecule L1 precursor	K.LSPYVHYTFR.V	2	2.34	0.24	-3.07
IPI00027087	Isoform 1 of Neural cell adhesion molecule L1 precursor	K.VGEEDDGEYR.C	2	3.10	0.34	-2.42
IPI00027087	Isoform 1 of Neural cell adhesion molecule L1 precursor	K.VKDATQITQGPR.S	2	3.74	0.33	-2.97
IPI00027087	Isoform 1 of Neural cell adhesion molecule L1 precursor	K.YGPGEPSPVSETVVTPEAAPEK.N	2	5.13	0.48	-4.39
IPI00027087	Isoform 1 of Neural cell adhesion molecule L1 precursor	K.YGPGEPSPVSETVVTPEAAPEKNPVDVK.G	3	3.68	0.36	-2.97
IPI00027087	Isoform 1 of Neural cell adhesion molecule L1 precursor	L.IQIPEEYEGHHVM*EPPVITEQSPR.R	3	4.12	0.41	-3.75
IPI00027087	Isoform 1 of Neural cell adhesion molecule L1 precursor	R.AQLLVVGSPGPVPR.L	2	3.58	0.26	-1.96
IPI00027087	Isoform 1 of Neural cell adhesion molecule L1 precursor	R.CLAENSLGSAR.H	1	2.49	0.36	-3.68
IPI00027087	Isoform 1 of Neural cell adhesion molecule L1 precursor	R.CLAENSLGSAR.H	2	3.69	0.32	-1.88
IPI00027087	Isoform 1 of Neural cell adhesion molecule L1 precursor	R.DLQELGDSDKYFIEDGR.L	3	3.28	0.15	-2.63
IPI00027087	Isoform 1 of Neural cell adhesion molecule L1 precursor	R.FQLQATTK.E	1	1.98	0.06	-3.22
IPI00027087	Isoform 1 of Neural cell adhesion molecule L1 precursor	R.GALILSNVQPSDTM*VTQCEAR.N	2	6.30	0.60	-2.89
IPI00027087	Isoform 1 of Neural cell adhesion molecule L1 precursor	R.GALILSNVQPSDTM*VTQCEAR.N	3	3.05	0.33	-3.01
IPI00027087	Isoform 1 of Neural cell adhesion molecule L1 precursor	R.GDGRDLQELGDSDKYFIEDGR.L	3	4.17	0.43	-2.69
IPI00027087	Isoform 1 of Neural cell adhesion molecule L1 precursor	R.GDGRDLQELGDSDKYFIEDGR.L	4	3.29	0.31	-2.39
IPI00027087	Isoform 1 of Neural cell adhesion molecule L1 precursor	R.LVLSDLHLLTQSQVR.V	2	5.14	0.48	-2.16
IPI00027087	Isoform 1 of Neural cell adhesion molecule L1 precursor	R.LVLSDLHLLTQSQVR.V	3	4.97	0.46	-2.70

IPI00027087	Isoform 1 of Neural cell adhesion molecule L1 precursor	R.LVVFPTDDISLK.C	2	3.10	0.35	-1.20
IPI00027087	Isoform 1 of Neural cell adhesion molecule L1 precursor	R.RLVVFPTDDISLK.C	2	4.07	0.24	-4.15
IPI00027087	Isoform 1 of Neural cell adhesion molecule L1 precursor	R.TIIQKEPIDLR.V	2	2.74	0.19	-3.20
IPI00027087	Isoform 1 of Neural cell adhesion molecule L1 precursor	W.LDEDGTTVLQDER.F	2	4.24	0.39	-1.62
IPI00027087	Isoform 1 of Neural cell adhesion molecule L1 precursor	W.SPAEDHNAPIEK.Y	2	3.30	0.36	-3.28
IPI00027166	Metalloproteinase inhibitor 2 precursor	K.AVSEKEVDSGNDIYGNPIK.R	3	3.27	0.16	-1.42
IPI00027166	Metalloproteinase inhibitor 2 precursor	K.AVSEKEVDSGNDIYGNPIKR.I	2	5.53	0.52	-4.58
IPI00027166	Metalloproteinase inhibitor 2 precursor	K.AVSEKEVDSGNDIYGNPIKR.I	3	5.56	0.45	-1.81
IPI00027166	Metalloproteinase inhibitor 2 precursor	K.AVSEKEVDSGNDIYGNPIKR.I	4	2.34	0.15	1.46
IPI00027166	Metalloproteinase inhibitor 2 precursor	K.DIEFIYTAPSSAVCGVSLDVGGK.K	2	2.43	0.18	
IPI00027166	Metalloproteinase inhibitor 2 precursor	K.EVDSGNDIYGNPIKR.I	2	4.12	0.40	-3.25
IPI00027166	Metalloproteinase inhibitor 2 precursor	K.EYLIAGKAEGDGK.M	2	3.63	0.28	-0.10
IPI00027166	Metalloproteinase inhibitor 2 precursor	K.KEYLIAGK.A	2	2.18	0.05	-2.73
IPI00027166	Metalloproteinase inhibitor 2 precursor	K.M*FKGPEKDIEFIYTAPSSAVCGVSLDVGGKK.E	3	5.76	0.51	-4.29
IPI00027166	Metalloproteinase inhibitor 2 precursor	K.M*FKGPEKDIEFIYTAPSSAVCGVSLDVGGKK.E	4	2.82	0.14	-3.24
IPI00027166	Metalloproteinase inhibitor 2 precursor	K.M*HITLCDFIVPWDTLSTTQK.K	3	2.91	0.25	-2.71
IPI00027166	Metalloproteinase inhibitor 2 precursor	R.AKAVSEKEVDSGNDIYGNPIKR.I	2	5.57	0.55	-3.01
IPI00027166	Metalloproteinase inhibitor 2 precursor	R.AKAVSEKEVDSGNDIYGNPIKR.I	3	6.54	0.52	-3.56
IPI00027166	Metalloproteinase inhibitor 2 precursor	R.AKAVSEKEVDSGNDIYGNPIKR.I	4	4.21	0.47	-2.00
IPI00027166	Metalloproteinase inhibitor 2 precursor	R.GAAPPKQEFLDIEDP.-	2	3.11	0.24	-3.42
IPI00027166	Metalloproteinase inhibitor 2 precursor	R.SDGSCAWYR.G	2	2.60	0.32	-1.90
IPI00027166	Metalloproteinase inhibitor 2 precursor	R.YQM*GCECK.I	2	1.96	0.18	
IPI00027174	Isoform 1 of Fibroblast growth factor receptor 3 precursor	K.DGTGLVPSER.V	1	2.17	0.26	-2.54
IPI00027174	Isoform 1 of Fibroblast growth factor receptor 3 precursor	K.DGTGLVPSER.V	2	3.04	0.12	-3.45
IPI00027174	Isoform 1 of Fibroblast growth factor receptor 3 precursor	K.KLLAVPAANTVR.F	3	2.87	0.25	-4.94
IPI00027174	Isoform 1 of Fibroblast growth factor receptor 3 precursor	K.LLAVPAANTVR.F	2	2.39	0.17	-2.73
IPI00027174	Isoform 1 of Fibroblast growth factor receptor 3 precursor	K.VGPDGTPYVTVLK.T	2	3.29	0.35	-1.93
IPI00027174	Isoform 1 of Fibroblast growth factor receptor 3 precursor	R.CPAAGNPTPSISWLK.N	2	4.18	0.40	-3.01
IPI00027192	Procollagen-lysine,2-oxoglutarate 5-dioxygenase 1 precursor	A.KGDAKPEDNLLVLTVATK.E	3	3.99	0.48	-4.10

IPI00027192	Procollagen-lysine,2-oxoglutarate 5-dioxygenase 1 precursor	K.AQVEEFLAQHGSEYQSVK.L	2	5.17	0.45	-2.13
IPI00027192	Procollagen-lysine,2-oxoglutarate 5-dioxygenase 1 precursor	K.AQVEEFLAQHGSEYQSVK.L	3	3.11	0.21	-1.81
IPI00027192	Procollagen-lysine,2-oxoglutarate 5-dioxygenase 1 precursor	K.FLLEYIAPM*TEK.L	2	4.50	0.28	-2.49
IPI00027192	Procollagen-lysine,2-oxoglutarate 5-dioxygenase 1 precursor	K.LQLNYLGNYIPR.F	2	3.74	0.37	-4.33
IPI00027192	Procollagen-lysine,2-oxoglutarate 5-dioxygenase 1 precursor	R.FLGSGGFIGYAPNLSK.L	2	5.23	0.45	-4.26
IPI00027192	Procollagen-lysine,2-oxoglutarate 5-dioxygenase 1 precursor	R.GELQSSDLFHHSK.L	2	3.31	0.39	-3.35
IPI00027192	Procollagen-lysine,2-oxoglutarate 5-dioxygenase 1 precursor	R.IFQNLDGALDEVVLK.F	2	4.98	0.28	-2.44
IPI00027192	Procollagen-lysine,2-oxoglutarate 5-dioxygenase 1 precursor	R.IFQNLDGALDEVVLKFEM*GHVR.A	4	3.68	0.23	-5.06
IPI00027192	Procollagen-lysine,2-oxoglutarate 5-dioxygenase 1 precursor	R.IQGGYENVPTIDIHM*NQIGFER.E	3	3.49	0.23	-0.72
IPI00027192	Procollagen-lysine,2-oxoglutarate 5-dioxygenase 1 precursor	R.LETKYPVVSDGKR.F	3	3.09	0.32	-0.76
IPI00027192	Procollagen-lysine,2-oxoglutarate 5-dioxygenase 1 precursor	R.QQDVFM*FLTNR.H	2	2.56	0.33	-4.94
IPI00027192	Procollagen-lysine,2-oxoglutarate 5-dioxygenase 1 precursor	R.SAQFFNYK.I	2	2.53	0.28	-1.26
IPI00027192	Procollagen-lysine,2-oxoglutarate 5-dioxygenase 1 precursor	R.SEDYVDIVQGR.R	2	3.69	0.43	-2.34
IPI00027192	Procollagen-lysine,2-oxoglutarate 5-dioxygenase 1 precursor	R.VGVWNVPYISNIYLIK.G	2	3.48	0.39	-4.24
IPI00027223	Isocitrate dehydrogenase [NADP] cytoplasmic	K.ISGGSVVEM*QGDEM*TR.I	2	3.80	0.38	-3.01
IPI00027223	Isocitrate dehydrogenase [NADP] cytoplasmic	R.FKDIFQEIYDKQYK.S	3	4.27	0.35	-2.43
IPI00027223	Isocitrate dehydrogenase [NADP] cytoplasmic	R.LIDDM*VAQAM*K.S	2	3.03	0.26	-3.63
IPI00027223	Isocitrate dehydrogenase [NADP] cytoplasmic	R.NILGGTVFR.E	2	1.89	0.05	-1.04
IPI00027230	Endoplasmin precursor	A.DDEVDVDGTVEEDLGK.S	2	5.40	0.47	-3.01
IPI00027230	Endoplasmin precursor	K.AQAYQTGKDISTNYYASQKK.T	3	3.28	0.41	-1.86
IPI00027230	Endoplasmin precursor	K.FAFQAEVNR.M	2	2.81	0.29	-1.56
IPI00027230	Endoplasmin precursor	K.GVVDSDDLPLNVSR.E	2	4.61	0.40	-3.29
IPI00027230	Endoplasmin precursor	K.IYFM*AGSSR.K	2	2.71	0.26	-2.62
IPI00027230	Endoplasmin precursor	K.LGVIEDHSNR.T	2	2.63	0.17	-1.34
IPI00027230	Endoplasmin precursor	K.SILFVPTSAPR.G	2	2.83	0.32	-3.74
IPI00027230	Endoplasmin precursor	K.YSQFINFPIYVWSSK.T	2	4.39	0.43	-3.54
IPI00027230	Endoplasmin precursor	R.FQSSHHPTDITSLDQYVER.M	3	5.83	0.42	-3.85
IPI00027230	Endoplasmin precursor	R.FQSSHHPTDITSLDQYVER.M	4	3.36	0.24	-2.97

IPI00027230	Endoplasmin precursor	R.GLFDEYGSK.K	2	1.71	0.22	-2.18
IPI00027230	Endoplasmin precursor	R.IKEDEDDKTVLDLAVVLFETATLR.S	3	3.60	0.27	-3.72
IPI00027230	Endoplasmin precursor	R.SGYLLPDTK.A	2	2.57	0.10	-1.99
IPI00027230	Endoplasmin precursor	R.VFITDDFHDM*M*PK.Y	2	3.16	0.43	-0.02
IPI00027239	Isoform Alpha of Tumor necrosis factor ligand superfamily member 13 precursor	A.LLTQQTELQSLR.R	2	3.57	0.40	-3.28
IPI00027239	Isoform Alpha of Tumor necrosis factor ligand superfamily member 13 precursor	L.LTQQTELQSLR.R	2	3.68	0.27	-3.29
IPI00027248	Tumor suppressor candidate 2	K.NLIPQGIVK.L	2	2.66	0.12	
IPI00027264	Calretinin	R.LLPVQENFLLK.F	2	2.56	0.15	-3.11
IPI00027310	Isoform 1 of Multiple epidermal growth factor-like domains 8	K.APQTVELPAVAGHTLTAR.R	2	4.79	0.51	-3.63
IPI00027310	Isoform 1 of Multiple epidermal growth factor-like domains 8	K.APQTVELPAVAGHTLTAR.R	3	4.05	0.45	-2.13
IPI00027310	Isoform 1 of Multiple epidermal growth factor-like domains 8	K.CESCLQGYFLLDGK.C	2	5.16	0.59	-3.31
IPI00027310	Isoform 1 of Multiple epidermal growth factor-like domains 8	K.ELQM*SKGEPK.K	2	2.20	0.18	-3.00
IPI00027310	Isoform 1 of Multiple epidermal growth factor-like domains 8	K.LDGGQLVWETLM*DSR.L	2	4.35	0.43	-4.24
IPI00027310	Isoform 1 of Multiple epidermal growth factor-like domains 8	K.LDGGQLVWETLM*DSR.L	3	3.83	0.36	-3.13
IPI00027310	Isoform 1 of Multiple epidermal growth factor-like domains 8	K.WCTNCPEGACIGR.N	2	4.19	0.41	-4.04
IPI00027310	Isoform 1 of Multiple epidermal growth factor-like domains 8	R.CM*EGGLSGPR.D	2	2.84	0.32	-1.37
IPI00027310	Isoform 1 of Multiple epidermal growth factor-like domains 8	R.FLDTGVVQSDR.S	2	4.23	0.29	-3.20
IPI00027310	Isoform 1 of Multiple epidermal growth factor-like domains 8	R.GAM*YLLGGLTAGGVTR.D	2	5.09	0.45	-3.54
IPI00027310	Isoform 1 of Multiple epidermal growth factor-like domains 8	R.GAM*YLLGGLTAGGVTR.D	3	4.67	0.30	-2.11
IPI00027310	Isoform 1 of Multiple epidermal growth factor-like domains 8	R.GDLM*AYK.V	1	1.62	0.07	-2.56
IPI00027310	Isoform 1 of Multiple epidermal growth factor-like domains 8	R.GDLM*AYK.V	2	2.49	0.20	-3.42
IPI00027310	Isoform 1 of Multiple epidermal growth factor-like domains 8	R.GFIYPM*LPGGPGGPGAEDVAVWTR.A	2	4.30	0.56	-4.78
IPI00027310	Isoform 1 of Multiple epidermal growth factor-like domains 8	R.GFIYPM*LPGGPGGPGAEDVAVWTR.A	3	3.90	0.37	-4.52
IPI00027310	Isoform 1 of Multiple epidermal growth factor-like domains 8	R.GPDTENM*EEVGR.W	2	4.07	0.43	-3.69

IPI00027310	Isoform 1 of Multiple epidermal growth factor-like domains 8	R.GPESCSLGCAQATQCALCLR.R	2	5.74	0.64	-3.59
IPI00027310	Isoform 1 of Multiple epidermal growth factor-like domains 8	R.GPESCSLGCAQATQCALCLR.R	3	5.56	0.44	-3.06
IPI00027310	Isoform 1 of Multiple epidermal growth factor-like domains 8	R.LFHASALLGDTM*VVLGGR.S	3	3.90	0.39	-2.59
IPI00027310	Isoform 1 of Multiple epidermal growth factor-like domains 8	R.LGCGGSPCSPM*PR.S	2	3.05	0.36	-2.65
IPI00027310	Isoform 1 of Multiple epidermal growth factor-like domains 8	R.LGHTM*VDGPDATLWM*FGGLGLPQGLLGNLYR.Y	3	4.95	0.32	-2.31
IPI00027310	Isoform 1 of Multiple epidermal growth factor-like domains 8	R.LLALTLPPDPCR.L	2	3.46	0.41	-4.32
IPI00027310	Isoform 1 of Multiple epidermal growth factor-like domains 8	R.LLGDCQACLAFSSPTAPPR.G	3	3.36	0.25	-0.53
IPI00027310	Isoform 1 of Multiple epidermal growth factor-like domains 8	R.LLRGPESCSLGCAQATQCALCLR.R	3	5.42	0.39	-1.68
IPI00027310	Isoform 1 of Multiple epidermal growth factor-like domains 8	R.LSADTASR.F	2	2.52	0.20	-3.22
IPI00027310	Isoform 1 of Multiple epidermal growth factor-like domains 8	R.LYISGGFGGVALGR.L	2	4.43	0.50	-3.67
IPI00027310	Isoform 1 of Multiple epidermal growth factor-like domains 8	R.M*ARGPDTENM*EEVGR.W	3	4.09	0.31	-1.81
IPI00027310	Isoform 1 of Multiple epidermal growth factor-like domains 8	R.QEKAPQTVELPAVAGHTLTAR.R	2	4.24	0.37	-3.87
IPI00027310	Isoform 1 of Multiple epidermal growth factor-like domains 8	R.QEKAPQTVELPAVAGHTLTAR.R	3	3.88	0.38	-2.97
IPI00027310	Isoform 1 of Multiple epidermal growth factor-like domains 8	R.RVGGLLPPGGGAAR.A	2	4.59	0.41	-3.31
IPI00027310	Isoform 1 of Multiple epidermal growth factor-like domains 8	R.SASVGPPM*EESVAHAVAAVGSR.L	2	5.06	0.56	-2.37
IPI00027310	Isoform 1 of Multiple epidermal growth factor-like domains 8	R.SASVGPPM*EESVAHAVAAVGSR.L	3	3.17	0.49	-3.84
IPI00027310	Isoform 1 of Multiple epidermal growth factor-like domains 8	R.SFHAAAYVPAGR.G	1	3.37	0.33	-3.97
IPI00027310	Isoform 1 of Multiple epidermal growth factor-like domains 8	R.SFHAAAYVPAGR.G	2	3.70	0.47	-3.81
IPI00027310	Isoform 1 of Multiple epidermal growth factor-like domains 8	R.SLIAAFCGQR.R	1	1.30	0.09	-2.30
IPI00027310	Isoform 1 of Multiple epidermal growth factor-like domains 8	R.SLIAAFCGQR.R	2	3.69	0.39	-1.86
IPI00027310	Isoform 1 of Multiple epidermal growth factor-like domains 8	R.STTITLTPSAETDVSLVYR.G	2	4.94	0.55	-7.92

IPI00027310	Isoform 1 of Multiple epidermal growth factor-like domains 8	R.STTITLTPSAETDVSLVYR.G	3	2.27	0.18	-3.16
IPI00027310	Isoform 1 of Multiple epidermal growth factor-like domains 8	R.TGVPGGSEISFFFLEPYR.S	2	4.50	0.26	-7.13
IPI00027310	Isoform 1 of Multiple epidermal growth factor-like domains 8	R.TGVPGGSEISFFFLEPYR.S	3	3.97	0.13	-3.40
IPI00027310	Isoform 1 of Multiple epidermal growth factor-like domains 8	R.TLQPGDGEASTPR.C	1	1.97	0.27	-1.84
IPI00027310	Isoform 1 of Multiple epidermal growth factor-like domains 8	R.TLQPGDGEASTPR.C	2	3.56	0.42	-3.28
IPI00027310	Isoform 1 of Multiple epidermal growth factor-like domains 8	R.TLQPGDGEASTPR.C	3	2.65	0.12	-2.26
IPI00027310	Isoform 1 of Multiple epidermal growth factor-like domains 8	R.TWSLLAPSQGAK.R	1	2.36	0.29	-1.83
IPI00027310	Isoform 1 of Multiple epidermal growth factor-like domains 8	R.TWSLLAPSQGAK.R	2	2.99	0.21	-2.12
IPI00027310	Isoform 1 of Multiple epidermal growth factor-like domains 8	R.VGGLLPPGGGAAR.A	2	2.61	0.16	-2.40
IPI00027310	Isoform 1 of Multiple epidermal growth factor-like domains 8	R.WTQM*LAGAEDGGPGPSPR.S	2	4.66	0.51	-6.90
IPI00027310	Isoform 1 of Multiple epidermal growth factor-like domains 8	W.TQM*LAGAEDGGPGPSPR.S	2	4.54	0.50	-3.94
IPI00027310	Isoform 1 of Multiple epidermal growth factor-like domains 8	W.VGEGLGLPVALPAR.W	2	3.04	0.24	-1.28
IPI00027341	Macrophage-capping protein	K.AQVEIVTDGEEPAEM*IQVLGPKPALK.E	3	4.40	0.48	-2.06
IPI00027341	Macrophage-capping protein	K.TSTGAPAAIKK.L	2	2.00	0.27	1.26
IPI00027341	Macrophage-capping protein	K.VSDATGQM*NLTK.V	2	3.90	0.45	-3.66
IPI00027341	Macrophage-capping protein	R.QAALQVAEGFISR.M	2	4.00	0.43	-2.61
IPI00027350	Peroxiredoxin-2	K.ATAVVDGAFK.E	2	2.72	0.29	-1.88
IPI00027350	Peroxiredoxin-2	K.EGGLGPLNIPLLADVTR.R	2	5.21	0.52	-5.12
IPI00027350	Peroxiredoxin-2	K.EGGLGPLNIPLLADVTRR.L	3	2.05	0.12	-2.42
IPI00027350	Peroxiredoxin-2	K.LGCEVLGVSVDSQFTHLAWINTPR.K	3	3.39	0.29	-3.54
IPI00027350	Peroxiredoxin-2	K.TDEGIAYR.G	1	1.38	0.08	-4.31
IPI00027350	Peroxiredoxin-2	K.TDEGIAYR.G	2	2.35	0.20	-2.76
IPI00027350	Peroxiredoxin-2	R.GLFIIDGK.G	2	2.10	0.11	-2.90
IPI00027350	Peroxiredoxin-2	R.IGKPAPDFK.A	2	2.13	0.10	-3.36
IPI00027350	Peroxiredoxin-2	R.KEGGLGPLNIPLLADVTR.R	2	5.45	0.60	-3.67
IPI00027350	Peroxiredoxin-2	R.KEGGLGPLNIPLLADVTR.R	3	3.75	0.39	-2.87
IPI00027350	Peroxiredoxin-2	R.KEGGLGPLNIPLLADVTRR.L	2	2.55	0.15	-4.58
IPI00027350	Peroxiredoxin-2	R.KEGGLGPLNIPLLADVTRR.L	3	4.21	0.46	-4.14
IPI00027350	Peroxiredoxin-2	R.KEGGLGPLNIPLLADVTRR.L	4	3.16	0.21	-2.16
IPI00027350	Peroxiredoxin-2	R.LSEDYGVLK.T	2	3.09	0.22	-1.56

IPI00027350	Peroxiredoxin-2	R.LSEDYGVLKTDEGIAYR.G	2	4.58	0.54	-3.52
IPI00027350	Peroxiredoxin-2	R.LSEDYGVLKTDEGIAYR.G	3	4.43	0.48	-1.64
IPI00027350	Peroxiredoxin-2	R.QITVNDLPVGR.S	1	1.08	0.06	-3.33
IPI00027350	Peroxiredoxin-2	R.QITVNDLPVGR.S	2	2.78	0.29	-3.36
IPI00027350	Peroxiredoxin-2	R.RLSEDYGVLK.T	2	2.13	0.08	-0.49
IPI00027350	Peroxiredoxin-2	R.RLSEDYGVLKTDEGIAYR.G	3	3.26	0.28	-2.09
IPI00027350	Peroxiredoxin-2	R.RLSEDYGVLKTDEGIAYR.G	4	2.10	0.21	-3.85
IPI00027377	aggrecan isoform 2 precursor	K.VSLPNYPAIPSDATLEVQSLR.S	2	3.39	0.36	-4.50
IPI00027377	aggrecan isoform 2 precursor	R.TIEGDFR.W	2	1.87	0.07	-3.62
IPI00027377	aggrecan isoform 2 precursor	R.VRVNSAYQDK.V	2	2.45	0.25	-3.00
IPI00027377	aggrecan isoform 2 precursor	R.YQCTEGFVQR.H	2	2.53	0.23	-1.45
IPI00027377	aggrecan isoform 2 precursor	R.YTLDFDR.A	2	2.30	0.11	-2.79
IPI00027429	Putative uncharacterized protein DKFZp547J2313	K.LTNSQNFDEYM*K.A	2	4.00	0.37	-3.50
IPI00027438	Flotillin-1	K.KAEAFQLYQEAAQLDMLLEK.L	3	4.54	0.36	-3.68
IPI00027444	Leukocyte elastase inhibitor	K.ADLSGM*SGAR.D	2	2.75	0.34	-1.69
IPI00027444	Leukocyte elastase inhibitor	K.FAYGYIEDLK.C	2	2.48	0.19	-1.95
IPI00027457	C1q-related factor precursor	K.FDDVVTNLGNNYDAASGK.F	2	4.75	0.55	-0.92
IPI00027462	Protein S100-A9	K.LGHPDTLNQGEFK.E	3	2.62	0.14	-1.96
IPI00027462	Protein S100-A9	K.QLSFEEFIM*LMAR.L	2	3.39	0.17	-3.25
IPI00027462	Protein S100-A9	K.QLSFEEFIMLMAR.L	2	4.12	0.41	-5.56
IPI00027462	Protein S100-A9	K.QLSFEEFIMLMAR.L	3	3.32	0.06	-1.65
IPI00027462	Protein S100-A9	K.VIEHIMEDLDTNADK.Q	2	4.24	0.39	-3.40
IPI00027462	Protein S100-A9	K.VIEHIMEDLDTNADK.Q	3	3.58	0.23	-3.01
IPI00027462	Protein S100-A9	K.VIEHIMEDLDTNADKQLSFEEFIMLMAR.L	3	7.43	0.49	-4.99
IPI00027462	Protein S100-A9	R.NIETIINTFHQYSVK.L	2	3.11	0.27	-4.84
IPI00027463	Protein S100-A6	K.LQDAEIAR.L	2	2.40	0.14	-3.55
IPI00027463	Protein S100-A6	R.LM*EDLDR.N	2	2.19	0.11	-3.25
IPI00027463	Protein S100-A6	R.LMEDLDRNKDQEVNFQEYVTFLGALALIYNEALKG.-	3	4.25	0.49	-1.26
IPI00027464	Calcineurin subunit B isoform 1	R.VIDIFDTDGNGEVDFKEFIEGVSQFSVK.G	3	4.69	0.46	-3.67
IPI00027466	Carbonic anhydrase 4 precursor	K.ASISGGGLPAPYQAK.Q	2	4.61	0.42	-3.07
IPI00027466	Carbonic anhydrase 4 precursor	K.LYYDKEQTVSM*KDNVRPLQQLGQR.T	3	3.67	0.37	-1.41
IPI00027466	Carbonic anhydrase 4 precursor	K.LYYDKEQTVSM*KDNVRPLQQLGQR.T	4	3.31	0.37	-1.49
IPI00027466	Carbonic anhydrase 4 precursor	K.VVWTVFR.E	2	1.78	0.21	-1.71
IPI00027466	Carbonic anhydrase 4 precursor	K.VVWTVFREPIQLHR.E	2	2.39	0.17	-3.47
IPI00027466	Carbonic anhydrase 4 precursor	R.EQILAFSQK.L	1	2.79	0.16	-3.79
IPI00027466	Carbonic anhydrase 4 precursor	R.EQILAFSQK.L	2	2.25	0.15	-2.27
IPI00027466	Carbonic anhydrase 4 precursor	R.FFFSGYDKK.Q	1	2.16	0.09	-3.67
IPI00027466	Carbonic anhydrase 4 precursor	R.QSPINIVTTK.A	1	1.71	0.08	-3.36
IPI00027466	Carbonic anhydrase 4 precursor	R.QSPINIVTTK.A	2	1.62	0.32	-1.89
IPI00027482	Corticosteroid-binding globulin precursor	K.AVLQLNEEGVDTAGSTGVTLNLTSKPIILR.F	3	5.74	0.36	
IPI00027482	Corticosteroid-binding globulin precursor	K.HLVALSPK.K	1	2.55	0.13	
IPI00027482	Corticosteroid-binding globulin precursor	K.HLVALSPK.K	2	2.67	0.11	

IPI00027482	Corticosteroid-binding globulin precursor	K.HYYESEVLAM*NFQDWATASR.Q	2	2.83	0.10	
IPI00027482	Corticosteroid-binding globulin precursor	K.M*NTVIAALSR.D	2	3.57	0.20	
IPI00027482	Corticosteroid-binding globulin precursor	K.MNTVIAALSR.D	2	3.52	0.17	
IPI00027482	Corticosteroid-binding globulin precursor	R.EENFYVDETTVVK.V	2	2.52	0.20	
IPI00027482	Corticosteroid-binding globulin precursor	R.GLASANVDFAFSLYK.H	2	4.08	0.50	
IPI00027482	Corticosteroid-binding globulin precursor	R.ITQDAQLK.S	1	1.75	0.13	-2.95
IPI00027482	Corticosteroid-binding globulin precursor	R.WSAGLTSSQVDLYIPK.V	2	4.34	0.44	-3.58
IPI00027493	4F2 cell-surface antigen heavy chain	K.ADLLLSTQPGREEGSPLELER.L	2	3.15	0.26	-3.58
IPI00027493	4F2 cell-surface antigen heavy chain	K.ADLLLSTQPGREEGSPLELER.L	3	4.84	0.45	-1.91
IPI00027493	4F2 cell-surface antigen heavy chain	K.GLVLGPIHK.N	2	2.41	0.08	-3.20
IPI00027493	4F2 cell-surface antigen heavy chain	K.GQSEDPGSLLSLFR.R	2	4.01	0.47	-3.09
IPI00027493	4F2 cell-surface antigen heavy chain	K.GRLDYLSSLK.V	2	2.72	0.15	-2.40
IPI00027493	4F2 cell-surface antigen heavy chain	K.NQKDDVAQTDLLQIDPNFGSK.E	3	3.80	0.34	-1.67
IPI00027493	4F2 cell-surface antigen heavy chain	K.NQKDDVAQTDLLQIDPNFGSKEDFDSLLQSAK.K	3	5.53	0.47	-2.58
IPI00027493	4F2 cell-surface antigen heavy chain	K.NQKDDVAQTDLLQIDPNFGSKEDFDSLLQSAK.K	4	4.81	0.38	-2.90
IPI00027493	4F2 cell-surface antigen heavy chain	R.IGDLQAFQGHGAGNLAGLK.G	2	4.52	0.42	-4.10
IPI00027493	4F2 cell-surface antigen heavy chain	R.LKLEPHEGLLLR.F	2	2.79	0.20	-4.95
IPI00027493	4F2 cell-surface antigen heavy chain	R.LKLEPHEGLLLR.F	3	2.58	0.31	-3.34
IPI00027493	4F2 cell-surface antigen heavy chain	R.LLTSFLPAQLLR.L	2	3.96	0.41	-4.66
IPI00027493	4F2 cell-surface antigen heavy chain	R.LLTSFLPAQLLR.L	3	3.92	0.27	-2.82
IPI00027493	4F2 cell-surface antigen heavy chain	R.VILDLTPNYR.G	2	3.30	0.33	-2.75
IPI00027493	4F2 cell-surface antigen heavy chain	R.VILDLTPNYRGENSWFSTQVDTVATK.V	3	5.24	0.51	-1.58
IPI00027497	Glucose-6-phosphate isomerase	K.DVM*PEVNK.V	2	1.91	0.09	-1.53
IPI00027497	Glucose-6-phosphate isomerase	K.HFVALSTNTTK.V	2	2.71	0.32	-2.32
IPI00027497	Glucose-6-phosphate isomerase	K.ILLANFLAQTEALM*R.G	2	4.69	0.45	-4.90
IPI00027497	Glucose-6-phosphate isomerase	K.ILLANFLAQTEALM*R.G	3	5.33	0.51	-2.61
IPI00027497	Glucose-6-phosphate isomerase	K.KIEPELDGSAQVTSHDASTNGLINFIK.Q	3	4.60	0.47	-2.10
IPI00027497	Glucose-6-phosphate isomerase	K.NLVTEDVM*R.M	2	2.41	0.20	-3.14
IPI00027497	Glucose-6-phosphate isomerase	K.STEEARKELQAAGK.S	3	2.47	0.29	-1.33
IPI00027497	Glucose-6-phosphate isomerase	K.TLAQLNPESSLFIIASK.T	2	4.51	0.52	-4.75
IPI00027497	Glucose-6-phosphate isomerase	K.TLAQLNPESSLFIIASK.T	3	4.58	0.50	-2.90
IPI00027497	Glucose-6-phosphate isomerase	K.VFEGNRPTNSIVFTK.L	2	3.35	0.21	-3.50
IPI00027497	Glucose-6-phosphate isomerase	K.VFEGNRPTNSIVFTK.L	3	2.50	0.27	-2.81
IPI00027497	Glucose-6-phosphate isomerase	R.FAAYFQQGDM*ESNGK.Y	2	4.41	0.53	-4.46
IPI00027497	Glucose-6-phosphate isomerase	R.SNTPILVDGKDVM*PEVNK.V	3	2.72	0.15	-2.81
IPI00027507	Complement factor H-related protein 3 precursor	K.CYFPYLENGYNQNYGR.K	2	4.76	0.43	
IPI00027507	Complement factor H-related protein 3 precursor	K.CYFPYLENGYNQNYGR.K	3	5.71	0.28	
IPI00027507	Complement factor H-related protein 3 precursor	R.KCYFPYLENGYNQNYGR.K	2	4.78	0.38	
IPI00027507	Complement factor H-related protein 3 precursor	R.RPYFPVAVGK.Y	1	1.68	0.07	-2.78
IPI00027507	Complement factor H-related protein 3 precursor	R.RPYFPVAVGK.Y	2	3.15	0.20	-3.65
IPI00027547	Dermcidin precursor	K.ENAGEDPGLAR.Q	2	2.99	0.34	-3.84
IPI00027626	T-complex protein 1 subunit zeta	R.AQLGVQAFADALLIIPK.V	2	3.45	0.27	-2.76

IPI00027685	C-C chemokine receptor type 1	R.AFGAQLLPPLYSLVFVIGLVGNILVVLVLVQYKR.L	3	1.05	0.23	-6.44
IPI00027703	Isoform Long of Alpha-mannosidase IIx	K.DGQLEVILDRR.L	2	2.12	0.07	-2.43
IPI00027703	Isoform Long of Alpha-mannosidase IIx	K.DGQLEVILDRR.L	3	2.49	0.21	-3.50
IPI00027703	Isoform Long of Alpha-mannosidase IIx	K.GFDCGLEAK.N	2	1.80	0.08	-1.84
IPI00027703	Isoform Long of Alpha-mannosidase IIx	K.KLPLQANFYPM*PVM*AYIQDAQK.R	3	2.76	0.08	-1.87
IPI00027703	Isoform Long of Alpha-mannosidase IIx	K.KLPLQANFYPM*PVM*AYIQDAQKR.L	3	3.17	0.42	-3.02
IPI00027703	Isoform Long of Alpha-mannosidase IIx	K.TFDKYYTEQTQHILNSM*VSK.L	3	2.90	0.31	-4.24
IPI00027703	Isoform Long of Alpha-mannosidase IIx	K.TFDKYYTEQTQHILNSM*VSK.L	4	3.56	0.37	-2.84
IPI00027703	Isoform Long of Alpha-mannosidase IIx	R.AALLLDQYR.K	1	1.89	0.24	-3.58
IPI00027703	Isoform Long of Alpha-mannosidase IIx	R.AALLLDQYR.K	2	3.56	0.22	-2.22
IPI00027703	Isoform Long of Alpha-mannosidase IIx	R.AALLLDQYRK.K	2	2.27	0.16	-3.35
IPI00027703	Isoform Long of Alpha-mannosidase IIx	R.FSM*VSLLVNSPR.V	2	3.32	0.38	-3.44
IPI00027703	Isoform Long of Alpha-mannosidase IIx	R.FVVLFNPLEQER.F	2	4.74	0.39	-4.94
IPI00027703	Isoform Long of Alpha-mannosidase IIx	R.GAEVLYSLAAAHAR.R	2	3.43	0.32	-3.21
IPI00027703	Isoform Long of Alpha-mannosidase IIx	R.GAEVLYSLAAAHAR.R	3	4.17	0.51	-2.63
IPI00027703	Isoform Long of Alpha-mannosidase IIx	R.GLGQGLKDNKR.T	2	3.55	0.16	-3.38
IPI00027703	Isoform Long of Alpha-mannosidase IIx	R.HEAFPLR.V	2	2.29	0.15	-4.08
IPI00027703	Isoform Long of Alpha-mannosidase IIx	R.IEQLEQLLEENHEIISHIK.D	3	3.65	0.30	-4.24
IPI00027703	Isoform Long of Alpha-mannosidase IIx	R.LSHDALPER.T	1	2.08	0.08	-4.40
IPI00027703	Isoform Long of Alpha-mannosidase IIx	R.LSHDALPER.T	2	2.19	0.16	-2.45
IPI00027703	Isoform Long of Alpha-mannosidase IIx	R.LTLHTAQALGVSSLK.D	2	3.19	0.43	-3.21
IPI00027703	Isoform Long of Alpha-mannosidase IIx	R.PSFFSISPQDCQFALGGR.G	2	5.80	0.56	-3.55
IPI00027703	Isoform Long of Alpha-mannosidase IIx	R.QTWDSDSSTDIFCHM*M*PFYSYDVPHTCGPDPK.I	4	3.52	0.35	-1.96
IPI00027703	Isoform Long of Alpha-mannosidase IIx	R.RSGLAGRYPLSDFTLLTEAR.R	3	3.42	0.40	-3.73
IPI00027703	Isoform Long of Alpha-mannosidase IIx	R.SGLAGRYPLSDFTLLTEAR.R	2	3.56	0.41	-3.26
IPI00027703	Isoform Long of Alpha-mannosidase IIx	R.SGLAGRYPLSDFTLLTEAR.R	3	4.09	0.22	-1.82
IPI00027703	Isoform Long of Alpha-mannosidase IIx	R.SGLAGRYPLSDFTLLTEARR.T	4	2.23	0.26	-2.66
IPI00027703	Isoform Long of Alpha-mannosidase IIx	R.SNVLLVPLGDDFR.Y	2	3.18	0.30	-6.33
IPI00027703	Isoform Long of Alpha-mannosidase IIx	R.SQISVLQNR.I	2	3.04	0.08	-0.46
IPI00027703	Isoform Long of Alpha-mannosidase IIx	R.TLGLFQHHDAITGTAK.E	2	4.54	0.48	-3.17
IPI00027703	Isoform Long of Alpha-mannosidase IIx	R.TLQAEEDTLPSAETALILHR.K	2	5.29	0.50	-2.95
IPI00027703	Isoform Long of Alpha-mannosidase IIx	R.TLQAEEDTLPSAETALILHR.K	3	3.70	0.32	-3.35
IPI00027703	Isoform Long of Alpha-mannosidase IIx	R.TVGSEVQDSHSTSYPSLLSHLTSM*YLNAPALALPVAR.M	3	4.34	0.41	-1.52
IPI00027703	Isoform Long of Alpha-mannosidase IIx	R.TVGSEVQDSHSTSYPSLLSHLTSM*YLNAPALALPVAR.M	4	3.62	0.33	-3.11
IPI00027703	Isoform Long of Alpha-mannosidase IIx	R.TVIQLDSSPR.F	1	2.15	0.17	-2.64
IPI00027703	Isoform Long of Alpha-mannosidase IIx	R.TVIQLDSSPR.F	2	2.52	0.07	-1.46
IPI00027703	Isoform Long of Alpha-mannosidase IIx	R.VIDSGTSDFALSNR.Y	2	4.48	0.49	-4.73
IPI00027703	Isoform Long of Alpha-mannosidase IIx	R.VIDSGTSDFALSNR.Y	3	2.23	0.14	-2.00
IPI00027703	Isoform Long of Alpha-mannosidase IIx	R.VLEAHLR.G	2	2.08	0.15	-1.37
IPI00027703	Isoform Long of Alpha-mannosidase IIx	R.YM*QVWFSGLTGLLK.S	2	4.16	0.34	-8.28
IPI00027703	Isoform Long of Alpha-mannosidase IIx	R.YPLSDFTLLTEAR.R	2	4.02	0.39	-4.06

IPI00027721	Isoform 1 of Alpha-type platelet-derived growth factor receptor precursor	K.FQTIPFNVYALK.A	2	2.58	0.12	-3.26
IPI00027721	Isoform 1 of Alpha-type platelet-derived growth factor receptor precursor	K.GFIEIKPTFSQLEAVNLHEVK.H	3	2.92	0.13	-3.27
IPI00027721	Isoform 1 of Alpha-type platelet-derived growth factor receptor precursor	K.HFVVEVR.A	2	1.79	0.10	-4.09
IPI00027721	Isoform 1 of Alpha-type platelet-derived growth factor receptor precursor	K.LVYTLTVPEATVK.D	2	2.87	0.30	-2.51
IPI00027721	Isoform 1 of Alpha-type platelet-derived growth factor receptor precursor	R.TTDPETPVTLHNSEGVVPASYDSR.Q	3	3.58	0.25	-3.08
IPI00027726	Isoform 1 of Krueppel-like factor 3	K.FFQTPEGLSHGIQM*EPVDLTVNK.R	3	3.36	0.26	2.43
IPI00027744	Isoform 1 of Mineralocorticoid receptor	K.GNPTVNPFPFMDGSYFSFMDDK.D	3	3.13	0.16	
IPI00027780	72 kDa type IV collagenase precursor	A.APSPIIKFPGDVAPK.T	2	3.18	0.45	-2.82
IPI00027780	72 kDa type IV collagenase precursor	C.ATTANYDDDRK.W	2	3.24	0.39	-3.20
IPI00027780	72 kDa type IV collagenase precursor	D.PETVDDAFAR.A	2	3.05	0.41	-1.21
IPI00027780	72 kDa type IV collagenase precursor	K.AVFFAGNEYWIYSASTLER.G	3	3.33	0.08	-3.29
IPI00027780	72 kDa type IV collagenase precursor	K.ESCNLFVLK.D	2	1.83	0.12	-1.56
IPI00027780	72 kDa type IV collagenase precursor	K.FFGLPQTGDLDQNTIETM*R.K	2	5.12	0.63	-5.38
IPI00027780	72 kDa type IV collagenase precursor	K.FFGLPQTGDLDQNTIETM*R.K	3	3.65	0.43	-2.30
IPI00027780	72 kDa type IV collagenase precursor	K.GAYYLKLENQSLK.S	2	4.07	0.28	-4.23
IPI00027780	72 kDa type IV collagenase precursor	K.IDAVYEAPQEEK.A	2	3.97	0.42	-2.24
IPI00027780	72 kDa type IV collagenase precursor	K.LSQDDIK.G	2	2.28	0.06	-2.47
IPI00027780	72 kDa type IV collagenase precursor	K.M*DPGFPK.L	1	1.42	0.08	-2.52
IPI00027780	72 kDa type IV collagenase precursor	K.QDIVFDGIAQIR.G	2	4.37	0.37	-3.27
IPI00027780	72 kDa type IV collagenase precursor	K.TYIFAGDK.F	1	2.21	0.30	-2.06
IPI00027780	72 kDa type IV collagenase precursor	K.TYIFAGDK.F	2	2.23	0.06	-2.43
IPI00027780	72 kDa type IV collagenase precursor	R.AFQVWSDVTPLR.F	2	3.81	0.42	-4.47
IPI00027780	72 kDa type IV collagenase precursor	R.CGNPDVANYNFFPR.K	2	3.85	0.37	-2.13
IPI00027780	72 kDa type IV collagenase precursor	R.DKPM*GPLLVATFWPELPEK.I	3	3.66	0.22	-3.09
IPI00027780	72 kDa type IV collagenase precursor	R.DKPM*GPLLVATFWPELPEKIDAVYEAPQEEK.A	3	6.43	0.53	-4.02
IPI00027780	72 kDa type IV collagenase precursor	R.DKPM*GPLLVATFWPELPEKIDAVYEAPQEEK.A	4	4.23	0.33	-2.76
IPI00027780	72 kDa type IV collagenase precursor	R.GEIFFFKDR.F	2	2.94	0.25	-3.78
IPI00027780	72 kDa type IV collagenase precursor	R.GYPKPLTSLGLPPDVQR.V	2	3.42	0.44	-4.02
IPI00027780	72 kDa type IV collagenase precursor	R.IHDGEADIM*INFGR.W	2	4.22	0.44	-0.97
IPI00027780	72 kDa type IV collagenase precursor	R.IHDGEADIM*INFGR.W	3	4.44	0.32	-0.87
IPI00027780	72 kDa type IV collagenase precursor	R.IIGYTPDLDPETVDDAFAR.A	2	5.67	0.56	-4.48
IPI00027780	72 kDa type IV collagenase precursor	R.IIGYTPDLDPETVDDAFAR.A	3	4.66	0.43	-4.95
IPI00027780	72 kDa type IV collagenase precursor	R.SDGFLWCSTTYNFEK.D	2	4.94	0.47	-3.82
IPI00027780	72 kDa type IV collagenase precursor	R.SDGFLWCSTTYNFEKDGK.Y	3	2.95	0.26	-1.82
IPI00027780	72 kDa type IV collagenase precursor	R.VDAAFNWSK.N	2	2.39	0.30	-2.59
IPI00027782	Stromelysin-1 precursor	R.GEDTSM*NLVQK.Y	2	3.41	0.32	-2.99

IPI00027806	Cysteine-rich secretory protein LCCL domain-containing 1 precursor	K.YM*DEDGEWWIAK.Q	2	4.36	0.50	-3.27
IPI00027827	Extracellular superoxide dismutase [Cu-Zn] precursor	A.CCVVGVCGPGLWER.Q	2	4.69	0.50	-2.22
IPI00027827	Extracellular superoxide dismutase [Cu-Zn] precursor	A.GLAASLAGPHSIVGR.A	2	3.08	0.48	-1.82
IPI00027827	Extracellular superoxide dismutase [Cu-Zn] precursor	A.WTGEDSAEPNSDSAEWIR.D	2	4.67	0.40	-3.49
IPI00027827	Extracellular superoxide dismutase [Cu-Zn] precursor	G.RAVVVHAGEDDLGR.G	2	3.73	0.38	-4.60
IPI00027827	Extracellular superoxide dismutase [Cu-Zn] precursor	G.RAVVVHAGEDDLGR.G	3	3.69	0.36	-2.12
IPI00027827	Extracellular superoxide dismutase [Cu-Zn] precursor	K.VTEIWQEVM*QR.R	2	4.04	0.38	-4.21
IPI00027827	Extracellular superoxide dismutase [Cu-Zn] precursor	K.VTEIWQEVM*QR.R	3	4.53	0.32	-2.20
IPI00027827	Extracellular superoxide dismutase [Cu-Zn] precursor	L.AASLAGPHSIVGR.A	2	3.25	0.43	-3.25
IPI00027827	Extracellular superoxide dismutase [Cu-Zn] precursor	R.AGLAASLAGPHSIVGR.A	1	2.68	0.38	-2.91
IPI00027827	Extracellular superoxide dismutase [Cu-Zn] precursor	R.AGLAASLAGPHSIVGR.A	2	4.66	0.49	-5.29
IPI00027827	Extracellular superoxide dismutase [Cu-Zn] precursor	R.AGLAASLAGPHSIVGR.A	3	3.57	0.46	-3.24
IPI00027827	Extracellular superoxide dismutase [Cu-Zn] precursor	R.AVVVHAGEDDLGR.G	1	3.54	0.41	-2.48
IPI00027827	Extracellular superoxide dismutase [Cu-Zn] precursor	R.AVVVHAGEDDLGR.G	2	4.08	0.57	-2.20
IPI00027827	Extracellular superoxide dismutase [Cu-Zn] precursor	R.AVVVHAGEDDLGR.G	3	3.01	0.38	-2.35
IPI00027827	Extracellular superoxide dismutase [Cu-Zn] precursor	R.AVVVHAGEDDLGRGGNQASVENGNAGR.R	3	4.62	0.55	-3.86
IPI00027827	Extracellular superoxide dismutase [Cu-Zn] precursor	R.AVVVHAGEDDLGRGGNQASVENGNAGR.R	4	3.59	0.27	-3.59
IPI00027827	Extracellular superoxide dismutase [Cu-Zn] precursor	R.AVVVHAGEDDLGRGGNQASVENGNAGRR.L	3	4.41	0.50	-3.85
IPI00027827	Extracellular superoxide dismutase [Cu-Zn] precursor	R.AVVVHAGEDDLGRGGNQASVENGNAGRR.L	4	3.54	0.44	-3.66
IPI00027827	Extracellular superoxide dismutase [Cu-Zn] precursor	R.AVVVHAGEDDLGRGGNQASVENGNAGRR.L	5	2.17	0.18	-0.69
IPI00027827	Extracellular superoxide dismutase [Cu-Zn] precursor	R.DDDGALHAACQVQPSATLDAAQPR.V	2	5.22	0.54	-2.99

IPI00027827	Extracellular superoxide dismutase [Cu-Zn] precursor	R.DDDGALHAACQVQPSATLDAAQPR.V	3	4.55	0.42	-2.19
IPI00027827	Extracellular superoxide dismutase [Cu-Zn] precursor	R.GGNQASVENGNAGRR.L	2	2.20	0.12	-0.63
IPI00027827	Extracellular superoxide dismutase [Cu-Zn] precursor	R.LACCVVGVCGPGLWER.Q	2	4.95	0.42	-1.16
IPI00027827	Extracellular superoxide dismutase [Cu-Zn] precursor	R.LACCVVGVCGPGLWER.Q	3	4.19	0.30	-2.36
IPI00027827	Extracellular superoxide dismutase [Cu-Zn] precursor	R.RDDDGALHAACQVQPSATLDAAQPR.V	3	6.65	0.45	
IPI00027827	Extracellular superoxide dismutase [Cu-Zn] precursor	R.VTGVVLFR.Q	1	2.20	0.24	-3.87
IPI00027827	Extracellular superoxide dismutase [Cu-Zn] precursor	R.VTGVVLFR.Q	2	3.11	0.27	-4.49
IPI00027827	Extracellular superoxide dismutase [Cu-Zn] precursor	R.YRAGLAASLAGPHSIVGR.A	2	3.32	0.31	-4.01
IPI00027827	Extracellular superoxide dismutase [Cu-Zn] precursor	R.YRAGLAASLAGPHSIVGR.A	3	3.01	0.31	-3.60
IPI00027827	Extracellular superoxide dismutase [Cu-Zn] precursor	V.VVHAGEDDLGR.G	2	3.04	0.47	-1.05
IPI00027834	heterogeneous nuclear ribonucleoprotein L isoform a	R.IQHPSNVLHFFNAPLEVTEENFFEICDELGVK.R	3	3.38	0.27	-1.00
IPI00027847	Lipoprotein lipase precursor	R.DFIDIESK.F	1	2.34	0.15	-3.99
IPI00027847	Lipoprotein lipase precursor	R.ITGLDPAGPNFEYAEAPSR.L	2	3.45	0.34	-4.13
IPI00027848	Macrophage mannose receptor 1 precursor	K.DYQYYFSK.E	2	2.27	0.22	-1.82
IPI00027848	Macrophage mannose receptor 1 precursor	K.EGWNFYSNK.C	2	2.39	0.32	-3.29
IPI00027848	Macrophage mannose receptor 1 precursor	K.FAWM*DGSK.V	2	1.82	0.16	-2.51
IPI00027848	Macrophage mannose receptor 1 precursor	K.FEGSESLWNKDPLTSVSYQINSK.S	3	3.49	0.26	-4.04
IPI00027848	Macrophage mannose receptor 1 precursor	K.GDPTM*SWNDINCEHLNNWICQIQK.G	3	3.43	0.35	-4.18
IPI00027848	Macrophage mannose receptor 1 precursor	K.IQM*YFEWSDGTPVTFTK.W	2	5.46	0.63	-3.99
IPI00027848	Macrophage mannose receptor 1 precursor	K.WM*DDTCDSK.R	2	2.15	0.07	-2.12
IPI00027848	Macrophage mannose receptor 1 precursor	K.YFWTGLSDIQTK.G	2	4.26	0.53	-3.78
IPI00027848	Macrophage mannose receptor 1 precursor	R.DALTTCR.K	2	1.96	0.11	-2.86
IPI00027848	Macrophage mannose receptor 1 precursor	R.LITASGSYHK.L	2	2.41	0.11	-1.90
IPI00027848	Macrophage mannose receptor 1 precursor	R.M*GSSLVSIESAAESSFLSYR.V	2	5.84	0.53	-3.81
IPI00027848	Macrophage mannose receptor 1 precursor	R.M*GSSLVSIESAAESSFLSYR.V	3	5.17	0.34	-3.38
IPI00027848	Macrophage mannose receptor 1 precursor	R.NFGDLVSIQSESEKK.F	2	3.73	0.30	-0.93
IPI00027848	Macrophage mannose receptor 1 precursor	R.QFLIYNEDHKR.C	2	2.93	0.28	-3.35
IPI00027848	Macrophage mannose receptor 1 precursor	R.SQGPEIVEVEK.G	2	2.95	0.20	-2.09
IPI00027848	Macrophage mannose receptor 1 precursor	R.TGIAGGLWDVLK.C	2	3.49	0.36	-3.21
IPI00027848	Macrophage mannose receptor 1 precursor	R.YTNWAADEPK.L	2	2.64	0.27	-3.54
IPI00027851	Beta-hexosaminidase alpha chain precursor	K.DFYVVEPLAFEGTPEQK.A	2	4.59	0.50	-4.21

IPI00027851	Beta-hexosaminidase alpha chain precursor	K.DFYVVEPLAFEGTPEQK.A	3	4.33	0.48	-1.59
IPI00027851	Beta-hexosaminidase alpha chain precursor	K.EVIEYAR.L	1	1.82	0.11	-3.23
IPI00027851	Beta-hexosaminidase alpha chain precursor	K.EVIEYAR.L	2	1.96	0.19	-3.16
IPI00027851	Beta-hexosaminidase alpha chain precursor	K.GSYNPVTHIYTAQDVK.E	2	4.46	0.53	-3.24
IPI00027851	Beta-hexosaminidase alpha chain precursor	K.GSYNPVTHIYTAQDVK.E	3	4.15	0.41	-2.77
IPI00027851	Beta-hexosaminidase alpha chain precursor	K.GSYNPVTHIYTAQDVKEVIEYAR.L	3	3.80	0.44	-3.43
IPI00027851	Beta-hexosaminidase alpha chain precursor	K.GSYNPVTHIYTAQDVKEVIEYAR.L	4	3.08	0.13	-3.82
IPI00027851	Beta-hexosaminidase alpha chain precursor	K.GYVVWQEVFDNK.V	2	4.12	0.43	-3.97
IPI00027851	Beta-hexosaminidase alpha chain precursor	K.GYVVWQEVFDNKVK.I	2	4.03	0.36	-3.12
IPI00027851	Beta-hexosaminidase alpha chain precursor	K.GYVVWQEVFDNKVK.I	3	1.67	0.23	-2.19
IPI00027851	Beta-hexosaminidase alpha chain precursor	K.IQPDTIIQVWREDIPVNYM*K.E	2	3.44	0.36	-2.43
IPI00027851	Beta-hexosaminidase alpha chain precursor	K.IQPDTIIQVWREDIPVNYM*K.E	3	1.82	0.11	-3.82
IPI00027851	Beta-hexosaminidase alpha chain precursor	K.LTSDLTFAYER.L	2	3.63	0.24	-4.71
IPI00027851	Beta-hexosaminidase alpha chain precursor	K.QLESFYIQTLLDIVSSYGK.G	2	5.77	0.56	-1.43
IPI00027851	Beta-hexosaminidase alpha chain precursor	K.QLESFYIQTLLDIVSSYGK.G	3	3.81	0.36	-2.38
IPI00027851	Beta-hexosaminidase alpha chain precursor	K.SNPEIQDFM*R.K	1	1.36	0.26	-2.94
IPI00027851	Beta-hexosaminidase alpha chain precursor	K.SNPEIQDFM*R.K	2	3.08	0.45	-3.24
IPI00027851	Beta-hexosaminidase alpha chain precursor	R.ALLSAPWYLNR.I	2	3.39	0.39	-2.39
IPI00027851	Beta-hexosaminidase alpha chain precursor	R.GLETFSQLVWK.S	2	2.88	0.21	-2.38
IPI00027851	Beta-hexosaminidase alpha chain precursor	R.HYLPLSSILDTLDVM*AYNK.L	2	2.60	0.23	-4.22
IPI00027851	Beta-hexosaminidase alpha chain precursor	R.HYLPLSSILDTLDVM*AYNK.L	3	3.68	0.41	-3.76
IPI00027851	Beta-hexosaminidase alpha chain precursor	R.ISYGPDWKDFYVVEPLAFEGTPEQK.A	3	4.47	0.34	-4.38
IPI00027851	Beta-hexosaminidase alpha chain precursor	R.KGSYNPVTHIYTAQDVK.E	2	4.14	0.50	-4.10
IPI00027851	Beta-hexosaminidase alpha chain precursor	R.YVLYPNNFQFQYDVSSAAQPGCSVLDEAFQR.Y	3	4.01	0.29	-5.96
IPI00027875	Synaptotagmin-11	R.NLLVDAAEAGLLSR.D	2	4.91	0.40	-3.28
IPI00027898	Isoform A of Uncharacterized protein C21orf70	K.M*KLRREQWLQKIEAIKLAEQK.H	3	3.51	0.12	
IPI00027972	Isoform 1 of Leukocyte immunoglobulin-like receptor subfamily A member 2 precursor	R.SEHQAQQNQAEFR.M	2	4.15	0.42	-2.74
IPI00027972	Isoform 1 of Leukocyte immunoglobulin-like receptor subfamily A member 2 precursor	R.SEHQAQQNQAEFR.M	3	3.16	0.24	-1.90
IPI00027972	Isoform 1 of Leukocyte immunoglobulin-like receptor subfamily A member 2 precursor	W.AEPGSVIIQGSPVTLR.C	2	4.30	0.51	-3.14
IPI00027984	Putative uncharacterized protein	R.TPSTAWTSAAVK.L	2	2.48	0.19	
IPI00028015	Isoform 2 of Leukocyte-associated immunoglobulin-like receptor 1 precursor	K.WSEQSDYLELLVK.G	2	4.69	0.43	-3.84
IPI00028015	Isoform 2 of Leukocyte-associated immunoglobulin-like receptor 1 precursor	R.FRIDSVSEGNAGPYR.C	2	3.84	0.33	-3.41
IPI00028015	Isoform 2 of Leukocyte-associated immunoglobulin-like receptor 1 precursor	R.FRIDSVSEGNAGPYR.C	3	3.56	0.22	-3.29
IPI00028015	Isoform 2 of Leukocyte-associated immunoglobulin-like receptor 1 precursor	R.IDSVSEGNAGPYR.C	2	3.87	0.42	-3.79
IPI00028053	Gap junction alpha-9 protein	K.RETEGKDSKR.N	1	1.66	0.13	-7.33

IPI00028082	Reversion-inducing cysteine-rich protein with Kazal motifs precursor	K.LGEASDFIVR.Q	2	3.38	0.36	-2.59
IPI00028082	Reversion-inducing cysteine-rich protein with Kazal motifs precursor	R.DVCEQIFSSK.S	2	3.23	0.25	-3.50
IPI00028082	Reversion-inducing cysteine-rich protein with Kazal motifs precursor	R.TDSSPGPSQIK.A	2	2.79	0.18	-3.15
IPI00028193	192 kDa protein	K.DLGRQQADGALPDAQSPELEQQLM*M*EKRNYRK.T	3	3.79	0.07	
IPI00028193	192 kDa protein	R.SLGATLK.Y	1	1.68	0.05	-3.46
IPI00028381	Isoform 1 of Delta-like protein 2 precursor	R.CLVGFVGAR.C	2	2.76	0.17	-1.64
IPI00028383	Uncharacterized protein C16orf24	R.FPLPTWQPVTAVGEGLDR.V	3	3.23	0.08	1.55
IPI00028387	Isoform 1 of Uncharacterized protein C20orf116 precursor	R.AASAGQEPLHNEELAGAGR.V	3	2.31	0.13	0.18
IPI00028387	Isoform 1 of Uncharacterized protein C20orf116 precursor	R.VAQPGPLEPEEPR.A	2	2.52	0.38	-1.57
IPI00028413	Isoform 1 of Inter-alpha-trypsin inhibitor heavy chain H3 precursor	K.EHLVQATPENLQEAR.T	2	5.37	0.50	-2.88
IPI00028413	Isoform 1 of Inter-alpha-trypsin inhibitor heavy chain H3 precursor	K.EHLVQATPENLQEAR.T	3	3.38	0.30	-2.05
IPI00028413	Isoform 1 of Inter-alpha-trypsin inhibitor heavy chain H3 precursor	K.GM*TNINDGLLR.G	2	3.91	0.37	-2.50
IPI00028413	Isoform 1 of Inter-alpha-trypsin inhibitor heavy chain H3 precursor	K.SM*EDKGM*TNINDGLLR.G	2	2.77	0.39	-2.47
IPI00028413	Isoform 1 of Inter-alpha-trypsin inhibitor heavy chain H3 precursor	K.SM*EDKGM*TNINDGLLR.G	3	3.67	0.24	-1.53
IPI00028413	Isoform 1 of Inter-alpha-trypsin inhibitor heavy chain H3 precursor	K.YHFVTPLTSM*VVTKPEDNEDER.A	3	3.43	0.37	-4.97
IPI00028413	Isoform 1 of Inter-alpha-trypsin inhibitor heavy chain H3 precursor	R.DFLGFYVVDSHR.M	3	1.94	0.10	-1.42
IPI00028413	Isoform 1 of Inter-alpha-trypsin inhibitor heavy chain H3 precursor	R.DYIFGNYIER.L	2	2.20	0.27	-2.55
IPI00028413	Isoform 1 of Inter-alpha-trypsin inhibitor heavy chain H3 precursor	R.ESPGNVQIVNGYFVHFFAPQGLPVVPK.N	3	5.12	0.54	-3.69
IPI00028413	Isoform 1 of Inter-alpha-trypsin inhibitor heavy chain H3 precursor	R.ESPGNVQIVNGYFVHFFAPQGLPVVPK.N	4	2.97	0.23	-2.51
IPI00028413	Isoform 1 of Inter-alpha-trypsin inhibitor heavy chain H3 precursor	R.LIQDAVTGLTVNGQITGDKR.G	3	2.85	0.33	-2.42
IPI00028413	Isoform 1 of Inter-alpha-trypsin inhibitor heavy chain H3 precursor	R.LVDEDM*NSFK.A	2	3.58	0.36	-3.31
IPI00028413	Isoform 1 of Inter-alpha-trypsin inhibitor heavy chain H3 precursor	R.LVDEDM*NSFKADVK.G	2	3.95	0.40	-2.45
IPI00028413	Isoform 1 of Inter-alpha-trypsin inhibitor heavy chain H3 precursor	R.SLPEGVANGIEVYSTK.I	2	4.62	0.52	-3.98

IPI00028413	Isoform 1 of Inter-alpha-trypsin inhibitor heavy chain H3 precursor	R.STSIVIM*LTDGDANVGESRPEK.I	3	3.49	0.26	-2.42
IPI00028448	Brain-specific angiogenesis inhibitor 3 precursor	K.KGEEDQKSFFEFLVLNK.V	3	4.45	0.39	-3.51
IPI00028448	Brain-specific angiogenesis inhibitor 3 precursor	K.NAFVFLQYDK.N	1	1.57	0.20	-1.05
IPI00028448	Brain-specific angiogenesis inhibitor 3 precursor	K.NAFVFLQYDK.N	2	3.31	0.38	-3.51
IPI00028448	Brain-specific angiogenesis inhibitor 3 precursor	K.RVPQEQADAAK.F	2	2.88	0.25	-2.72
IPI00028448	Brain-specific angiogenesis inhibitor 3 precursor	K.SFFEFLVLNK.V	2	3.77	0.21	-2.86
IPI00028448	Brain-specific angiogenesis inhibitor 3 precursor	R.RVFPTNFPGLQK.K	2	3.33	0.22	-1.97
IPI00028448	Brain-specific angiogenesis inhibitor 3 precursor	R.TESCGIM*YTK.C	2	2.30	0.32	-3.51
IPI00028448	Brain-specific angiogenesis inhibitor 3 precursor	R.VFPTNFPGLQK.K	2	2.28	0.31	-2.53
IPI00028450	Isoform 1 of Sodium/calcium exchanger 1 precursor	R.VGIIDDDIFEEDENFLVHLSNVK.V	3	3.00	0.19	-4.44
IPI00028481	Ras-related protein Rab-8A	R.NIEEHASADVEK.M	2	2.94	0.25	-3.56
IPI00028520	Isoform 1 of NADH dehydrogenase [ubiquinone] flavoprotein 1, mitochondrial precursor	K.SVCETVLMDFDALVQAQTGLGTAAVIVMDR.S	3	3.43	0.40	-1.58
IPI00028553	Isoform 2 of Multiple inositol polyphosphate phosphatase 1 precursor	C.SLLEPRDPVASSLSPYFGTK.T	3	4.36	0.28	-1.91
IPI00028553	Isoform 2 of Multiple inositol polyphosphate phosphatase 1 precursor	K.TGPEM*QNILK.K	2	2.87	0.17	-0.45
IPI00028553	Isoform 2 of Multiple inositol polyphosphate phosphatase 1 precursor	K.TGPEM*QNILKK.V	3	2.70	0.20	-3.66
IPI00028553	Isoform 2 of Multiple inositol polyphosphate phosphatase 1 precursor	R.CM*DSSAAFLQGLWQHYHPGLPPPDVADM*EFGPPTVNDK.L	4	4.55	0.29	-2.05
IPI00028553	Isoform 2 of Multiple inositol polyphosphate phosphatase 1 precursor	R.DPVASSLSPYFGTK.T	2	4.34	0.38	-5.10
IPI00028553	Isoform 2 of Multiple inositol polyphosphate phosphatase 1 precursor	R.LASLFPALFSR.E	1	2.01	0.21	-3.33
IPI00028553	Isoform 2 of Multiple inositol polyphosphate phosphatase 1 precursor	R.LASLFPALFSR.E	2	2.98	0.24	-2.78
IPI00028561	Kinesin heavy chain isoform 5C	K.SEVKSLVNR.S	2	2.71	0.07	
IPI00028600	Isoform 1 of Kallikrein-7 precursor	K.M*NEYTVHLGSDTLGDRR.A	3	3.02	0.12	-3.40
IPI00028601	Putative metallothionein C20orf127	K.KSCCSCCPM*GCAKCAQGCVCKGACSCCV.-	3	1.64	0.12	2.11
IPI00028614	erythrocyte membrane protein band 4.2 isoform 2	K.AEQYQPLTASVSLQNSLDAPMEDCVISILGR.G	3	4.85	0.46	-2.77
IPI00028614	erythrocyte membrane protein band 4.2 isoform 2	K.EGTLGLTPAVSDLFAAINASCVVWK.C	2	5.27	0.60	-4.43
IPI00028614	erythrocyte membrane protein band 4.2 isoform 2	K.EGTLGLTPAVSDLFAAINASCVVWK.C	3	5.46	0.50	-5.05
IPI00028614	erythrocyte membrane protein band 4.2 isoform 2	L.IYLGTADCIQAESWDFGQFEGDVIDLSLR.L	3	5.00	0.45	-5.32
IPI00028614	erythrocyte membrane protein band 4.2 isoform 2	R.GRPVYDGQAWVLAAVACTVLR.C	2	4.34	0.43	-4.51
IPI00028614	erythrocyte membrane protein band 4.2 isoform 2	R.GRPVYDGQAWVLAAVACTVLR.C	3	2.85	0.23	-5.57
IPI00028614	erythrocyte membrane protein band 4.2 isoform 2	R.LTAMATHSESNLSCFAQEDIAICR.P	3	3.53	0.31	-2.75
IPI00028614	erythrocyte membrane protein band 4.2 isoform 2	R.LTVEVDCNMFQNLTNYK.S	2	5.22	0.46	-4.65
IPI00028614	erythrocyte membrane protein band 4.2 isoform 2	R.MEYLLNQNGLIYLGTADCIQAESWDFGQFEGDVIDLSLR.L	3	5.80	0.60	-4.22
IPI00028614	erythrocyte membrane protein band 4.2 isoform 2	R.MEYLLNQNGLIYLGTADCIQAESWDFGQFEGDVIDLSLR.L	4	3.47	0.19	-1.94

IPI00028614	erythrocyte membrane protein band 4.2 isoform 2	R.PVYDGQAWVLAAVACTVLR.C	2	5.16	0.54	-4.18
IPI00028714	Matrix Gla protein precursor	R.NANTFISPQQR.W	2	3.53	0.34	-2.31
IPI00028786	Isoform 3 of Polycystin-1 precursor	R.IPADATALDVSHNLLR.A	3	3.29	0.35	0.91
IPI00028908	Nidogen-2 precursor	K.EGTSLGEVGGPDLK.G	2	3.11	0.25	-3.10
IPI00028908	Nidogen-2 precursor	K.LANPLHFYEAR.F	2	2.76	0.42	-4.08
IPI00028908	Nidogen-2 precursor	K.VLFYTDLVNPR.A	2	2.89	0.35	-3.02
IPI00028908	Nidogen-2 precursor	R.AGLELGAEPETIVNSGLISPEGLAIDHIR.R	3	4.52	0.38	-4.61
IPI00028908	Nidogen-2 precursor	R.DGVVSVNK.H	2	2.37	0.17	-2.74
IPI00028908	Nidogen-2 precursor	R.EDTSPAVLGLAAR.Y	2	3.88	0.30	-1.20
IPI00028908	Nidogen-2 precursor	R.ETQYVDYDFPTDFPAIAPFLADIDTSHGR.G	3	6.35	0.56	-7.40
IPI00028908	Nidogen-2 precursor	R.FSNLYVGTNGIISTQDFPR.E	2	6.02	0.60	-5.49
IPI00028908	Nidogen-2 precursor	R.FSNLYVGTNGIISTQDFPR.E	3	4.51	0.39	-3.87
IPI00028908	Nidogen-2 precursor	R.FTPTHAFLATWEQVGAYEEVKR.G	3	2.99	0.32	-4.59
IPI00028908	Nidogen-2 precursor	R.GALPSGELNTFQAVLASDGSDSYALFLYPANGLQFLGTRPK.E	3	6.36	0.61	-4.84
IPI00028908	Nidogen-2 precursor	R.GALPSGELNTFQAVLASDGSDSYALFLYPANGLQFLGTRPK.E	4	4.97	0.43	-4.93
IPI00028908	Nidogen-2 precursor	R.GEADDLKSEGPYFSLTSTEQSVK.N	2	5.04	0.52	-1.66
IPI00028908	Nidogen-2 precursor	R.GNLYWTDWNR.D	2	2.70	0.29	-1.23
IPI00028908	Nidogen-2 precursor	R.HAQAQYAYPGAR.F	2	3.03	0.32	-3.61
IPI00028908	Nidogen-2 precursor	R.ILINTDIGLPNGLTFDPFSK.L	2	4.23	0.52	-2.56
IPI00028908	Nidogen-2 precursor	R.KVLFYTDLVNPR.A	2	4.06	0.39	-2.68
IPI00028908	Nidogen-2 precursor	R.KVLFYTDLVNPR.A	3	3.32	0.15	-2.40
IPI00028908	Nidogen-2 precursor	R.VLYREDTSPAVLGLAAR.Y	2	4.20	0.43	-3.26
IPI00028908	Nidogen-2 precursor	R.VLYREDTSPAVLGLAAR.Y	3	3.48	0.40	-2.68
IPI00028908	Nidogen-2 precursor	W.GDQLLQEGDDESSAVVK.L	2	4.81	0.43	-2.72
IPI00028911	Dystroglycan precursor	C.AADEPVTVLTVILDADLTK.M	2	4.36	0.45	-2.63
IPI00028911	Dystroglycan precursor	C.AADEPVTVLTVILDADLTK.M	3	4.62	0.39	-3.10
IPI00028911	Dystroglycan precursor	I.PTDLIASSGDIIK.V	2	4.32	0.33	-2.37
IPI00028911	Dystroglycan precursor	K.GVHYISVSATR.L	1	3.11	0.42	-4.74
IPI00028911	Dystroglycan precursor	K.GVHYISVSATR.L	2	3.64	0.44	-3.29
IPI00028911	Dystroglycan precursor	K.IPSDTFYDHEDTTTDKLK.L	3	2.17	0.17	-2.88
IPI00028911	Dystroglycan precursor	K.LGCSLNQNSVPDIHGVEAPA.R	2	5.39	0.52	-2.49
IPI00028911	Dystroglycan precursor	K.LGCSLNQNSVPDIHGVEAPAR.E	3	4.70	0.45	-2.66
IPI00028911	Dystroglycan precursor	K.LREQQLVGEK.S	2	2.28	0.10	-0.11
IPI00028911	Dystroglycan precursor	K.LVPVVNNR.L	1	2.39	0.18	-1.59
IPI00028911	Dystroglycan precursor	K.LVPVVNNR.L	2	1.48	0.17	-1.74
IPI00028911	Dystroglycan precursor	K.VVENGALLSWK.L	1	1.91	0.09	-3.10
IPI00028911	Dystroglycan precursor	K.VVENGALLSWK.L	2	3.69	0.41	-1.21
IPI00028911	Dystroglycan precursor	R.EGAM*SAQLGYPVVGWHIANK.K	2	5.09	0.50	-2.44
IPI00028911	Dystroglycan precursor	R.EGAM*SAQLGYPVVGWHIANK.K	3	3.37	0.43	-2.80
IPI00028911	Dystroglycan precursor	R.RIAEDDGKPRPAFSNALEPDFK.A	3	2.58	0.12	-1.47
IPI00028911	Dystroglycan precursor	R.RIAEDDGKPRPAFSNALEPDFK.A	4	2.34	0.20	-0.92
IPI00028911	Dystroglycan precursor	R.SFRVTIPTDLIASSGDIIK.V	2	2.80	0.23	-3.21

IPI00028911	Dystroglycan precursor	R.SFRVTIPTDLIASSGDIIK.V	3	2.99	0.12	-0.77
IPI00028911	Dystroglycan precursor	R.SFSEVELHNM*K.L	1	2.61	0.18	-3.26
IPI00028911	Dystroglycan precursor	R.SFSEVELHNM*K.L	2	3.80	0.37	-2.78
IPI00028911	Dystroglycan precursor	R.TASPDPGEVVSSA.C	2	2.96	0.38	-0.97
IPI00028911	Dystroglycan precursor	R.TASPDPGEVVSSACAADEPVTVLTVILDADLTK.M	3	4.86	0.40	-3.83
IPI00028911	Dystroglycan precursor	R.VTIPTDLIASSGDIIK.V	2	5.50	0.57	-4.96
IPI00028911	Dystroglycan precursor	R.VTIPTDLIASSGDIIK.V	3	3.77	0.35	-3.34
IPI00028911	Dystroglycan precursor	W.DSQSHTLEGLPLDTDKGVHYISVSATR.L	3	5.65	0.56	-3.93
IPI00028911	Dystroglycan precursor	W.DSQSHTLEGLPLDTDKGVHYISVSATR.L	4	5.18	0.46	-2.67
IPI00028912	zinc finger protein 161	K.KTPTTVVPLISTIAGDSSR.T	3	3.34	0.11	
IPI00028931	Desmoglein-2 precursor	K.GITEPPFGIFVFNK.D	2	3.19	0.35	-1.09
IPI00028931	Desmoglein-2 precursor	K.VLEGM*VEENQVNVEVTR.I	2	2.67	0.05	
IPI00028931	Desmoglein-2 precursor	R.GNNVEKPLELR.I	3	2.36	0.13	-3.61
IPI00028932	Microtubule-associated serine/threonine-protein kinase 3	K.SRASSSGGSGGGSGGR.V	2	3.12	0.08	
IPI00029012	Eukaryotic translation initiation factor 3 subunit A	K.IDYFERAKRLEEIPLIK.S	2	2.79	0.12	
IPI00029046	Uncharacterized protein KIAA0152 precursor	K.FAEVYFAQSQQK.V	2	4.18	0.41	-2.78
IPI00029046	Uncharacterized protein KIAA0152 precursor	K.FAEVYFAQSQQK.V	3	3.40	0.29	-4.27
IPI00029046	Uncharacterized protein KIAA0152 precursor	K.LSVQGEVSTFTGK.L	2	4.50	0.45	-2.78
IPI00029046	Uncharacterized protein KIAA0152 precursor	K.LYIEFVK.G	2	2.20	0.11	-2.31
IPI00029046	Uncharacterized protein KIAA0152 precursor	K.VCALYIM*AGTVDDVPK.L	2	4.89	0.46	-2.56
IPI00029046	Uncharacterized protein KIAA0152 precursor	R.LNGHVVVK.D	2	2.54	0.25	-5.01
IPI00029046	Uncharacterized protein KIAA0152 precursor	R.SNPEDQILYQTER.Y	2	4.40	0.41	-4.15
IPI00029046	Uncharacterized protein KIAA0152 precursor	R.YNEETFGYEVPIKEEGDYVLVLK.F	3	5.15	0.47	-1.90
IPI00029050	Isoform 1 of Glycosyltransferase-like protein LARGE1	K.SVIQLDLANTKKAMIVPAFETLRYR.L	2	1.91	0.25	
IPI00029050	Isoform 1 of Glycosyltransferase-like protein LARGE1	K.TYSM*EEGTGDSENLR.A	2	4.28	0.43	-5.72
IPI00029061	Selenoprotein P precursor	K.DDFLIYDR.C	2	2.95	0.22	-3.42
IPI00029061	Selenoprotein P precursor	K.LPTDSELAPR.S	1	2.63	0.29	-2.54
IPI00029061	Selenoprotein P precursor	K.LPTDSELAPR.S	2	3.57	0.33	-2.62
IPI00029061	Selenoprotein P precursor	L.PTDSELAPR.S	2	2.96	0.31	-2.25
IPI00029061	Selenoprotein P precursor	M.PASEDLQDLQK.K	2	3.21	0.31	-2.28
IPI00029061	Selenoprotein P precursor	R.DM*PASEDLQDLQK.K	2	4.43	0.42	-2.13
IPI00029061	Selenoprotein P precursor	R.DM*PASEDLQDLQK.K	3	3.80	0.08	-0.70
IPI00029061	Selenoprotein P precursor	R.LVYHLGLPFSFLTFPYVEEAIK.I	3	3.90	0.13	
IPI00029107	Werner syndrome ATP-dependent helicase	K.MTQQVTGELRKLNLSCGTYHAGM*SFSTR.K	4	3.17	0.15	-7.84
IPI00029123	Isoform A of Endothelin B receptor precursor	R.SLAPAEVPKGDR.T	2	2.67	0.27	-2.17
IPI00029131	Neuroendocrine convertase 2 precursor	K.EELEEELDEAVER.S	2	4.76	0.41	-4.42
IPI00029131	Neuroendocrine convertase 2 precursor	R.DM*QHLTVLTSK.R	2	3.11	0.20	1.10
IPI00029131	Neuroendocrine convertase 2 precursor	R.QVAAEHGFGVR.K	2	1.62	0.34	-2.42
IPI00029168	Apolipoprotein	K.CQSWSSM*TPHR.H	2	2.49	0.16	

IPI00029168	Apolipoprotein	K.CQSWSSM*TPHR.H	3	3.09	0.19	
IPI00029168	Apolipoprotein	R.GTYSTTVTGR.T	2	2.73	0.36	-2.13
IPI00029168	Apolipoprotein	R.NPDAVAAPYCYTR.D	2	3.07	0.18	
IPI00029175	Strumpellin	S.SQPGAKRPSNYPESYFQR.V	2	3.33	0.19	0.71
IPI00029193	Hepatocyte growth factor activator precursor	K.DSALSWEYCR.L	2	3.36	0.29	-1.49
IPI00029193	Hepatocyte growth factor activator precursor	K.YIPYTLYSVFNPSDHDLVLIR.L	3	2.34	0.29	-2.78
IPI00029193	Hepatocyte growth factor activator precursor	R.EALVPLVADHK.C	2	2.49	0.07	-3.43
IPI00029193	Hepatocyte growth factor activator precursor	R.LEACESLTR.V	2	2.37	0.21	-2.15
IPI00029193	Hepatocyte growth factor activator precursor	R.TTDVTQTFGIEK.Y	2	4.16	0.43	-4.59
IPI00029193	Hepatocyte growth factor activator precursor	R.VQLSPDLLATLPEPASPGR.Q	2	5.32	0.48	-3.04
IPI00029193	Hepatocyte growth factor activator precursor	R.VQLSPDLLATLPEPASPGR.Q	3	3.17	0.21	-3.19
IPI00029193	Hepatocyte growth factor activator precursor	W.GHLDENVSGYSSSLR.E	2	4.10	0.57	-4.26
IPI00029235	Insulin-like growth factor-binding protein 6 precursor	H.LDSVLQQLQTEVYR.G	2	4.43	0.45	-3.90
IPI00029235	Insulin-like growth factor-binding protein 6 precursor	R.APAVAEENPK.E	1	2.81	0.40	-2.99
IPI00029235	Insulin-like growth factor-binding protein 6 precursor	R.APAVAEENPK.E	2	3.06	0.43	-1.70
IPI00029235	Insulin-like growth factor-binding protein 6 precursor	R.APAVAEENPKESKPQAGTARPQDVN.R	3	4.90	0.33	-3.48
IPI00029235	Insulin-like growth factor-binding protein 6 precursor	R.APAVAEENPKESKPQAGTARPQDVNR.R	4	2.56	0.19	-3.22
IPI00029235	Insulin-like growth factor-binding protein 6 precursor	R.APAVAEENPKESKPQAGTARPQDVNRR.D	4	3.29	0.31	-4.40
IPI00029235	Insulin-like growth factor-binding protein 6 precursor	R.DQQRNPGTSTTPSQPNSAGVQDTEM*GPCR.R	3	4.68	0.51	-3.92
IPI00029235	Insulin-like growth factor-binding protein 6 precursor	R.GAQTLYVPNCDHR.G	2	3.07	0.32	-1.52
IPI00029235	Insulin-like growth factor-binding protein 6 precursor	R.HLDSVLQQLQTEVYR.G	2	5.97	0.56	-4.50
IPI00029235	Insulin-like growth factor-binding protein 6 precursor	R.HLDSVLQQLQTEVYR.G	3	5.47	0.40	-2.81
IPI00029235	Insulin-like growth factor-binding protein 6 precursor	R.NPGTSTTPSQPNSAGVQDTEM*GPCR.R	3	5.12	0.45	-2.07
IPI00029235	Insulin-like growth factor-binding protein 6 precursor	R.RHLDSVLQQLQTEVYR.G	3	4.39	0.31	-3.51
IPI00029236	Insulin-like growth factor-binding protein 5 precursor	K.FVGGAENTAHPR.I	2	2.73	0.33	-2.73
IPI00029236	Insulin-like growth factor-binding protein 5 precursor	R.EHEEPTTSEM*AEETYSPK.I	3	3.37	0.47	-1.24
IPI00029236	Insulin-like growth factor-binding protein 5 precursor	R.IISAPEM*R.Q	2	2.24	0.09	-2.46

IPI00029236	Insulin-like growth factor-binding protein 5 precursor	R.ISELKAEAV.K	1	2.04	0.16	-2.84
IPI00029236	Insulin-like growth factor-binding protein 5 precursor	R.ISELKAEAVKK.D	2	3.58	0.12	-3.72
IPI00029236	Insulin-like growth factor-binding protein 5 precursor	R.ISELKAEAVKK.D	3	2.79	0.12	-4.37
IPI00029260	Monocyte differentiation antigen CD14 precursor	F.PALTSLDLSDNPGLGER.G	2	5.14	0.49	-3.82
IPI00029260	Monocyte differentiation antigen CD14 precursor	H.SLDLSHNSLR.A	1	2.30	0.24	-4.14
IPI00029260	Monocyte differentiation antigen CD14 precursor	K.ELTLEDLK.I	2	2.47	0.15	-3.17
IPI00029260	Monocyte differentiation antigen CD14 precursor	K.FPAIQNLALR.N	2	3.72	0.31	-2.07
IPI00029260	Monocyte differentiation antigen CD14 precursor	K.ITGTM*PPLPLEATGLALSSLR.L	2	4.72	0.56	-6.08
IPI00029260	Monocyte differentiation antigen CD14 precursor	K.ITGTM*PPLPLEATGLALSSLR.L	3	4.46	0.37	-4.27
IPI00029260	Monocyte differentiation antigen CD14 precursor	K.VLSIAQAHSPAFSCEQVR.A	2	4.49	0.43	-1.91
IPI00029260	Monocyte differentiation antigen CD14 precursor	K.VLSIAQAHSPAFSCEQVR.A	3	5.70	0.56	-3.18
IPI00029260	Monocyte differentiation antigen CD14 precursor	R.AFPALTSLDLSDNPGLGER.G	2	6.31	0.55	-4.33
IPI00029260	Monocyte differentiation antigen CD14 precursor	R.AFPALTSLDLSDNPGLGER.G	3	5.70	0.54	-3.77
IPI00029260	Monocyte differentiation antigen CD14 precursor	R.ATVNPSAPR.C	1	1.79	0.08	-2.96
IPI00029260	Monocyte differentiation antigen CD14 precursor	R.ATVNPSAPR.C	2	2.36	0.18	-2.59
IPI00029260	Monocyte differentiation antigen CD14 precursor	R.CM*WSSALNSLNLSFAGLEQVPK.G	2	4.52	0.60	-3.08
IPI00029260	Monocyte differentiation antigen CD14 precursor	R.CM*WSSALNSLNLSFAGLEQVPK.G	3	2.37	0.24	-4.70
IPI00029260	Monocyte differentiation antigen CD14 precursor	R.GLM*AALCPHKFPAIQNLALR.N	3	5.34	0.52	-4.17
IPI00029260	Monocyte differentiation antigen CD14 precursor	R.GLM*AALCPHKFPAIQNLALR.N	4	3.05	0.34	-2.81
IPI00029260	Monocyte differentiation antigen CD14 precursor	R.LKELTLEDLK.I	1	3.04	0.18	-4.21
IPI00029260	Monocyte differentiation antigen CD14 precursor	R.LKELTLEDLK.I	2	3.57	0.22	-3.69
IPI00029260	Monocyte differentiation antigen CD14 precursor	R.LKELTLEDLK.I	3	3.81	0.10	-3.80
IPI00029260	Monocyte differentiation antigen CD14 precursor	R.LKELTLEDLKITGTM*PPLPLEATGLALSSLR.L	3	4.05	0.38	-2.78
IPI00029260	Monocyte differentiation antigen CD14 precursor	R.LKELTLEDLKITGTM*PPLPLEATGLALSSLR.L	4	3.10	0.23	-2.67
IPI00029260	Monocyte differentiation antigen CD14 precursor	R.LTVGAAQVPAQLLVGALR.V	2	5.01	0.49	-4.93
IPI00029260	Monocyte differentiation antigen CD14 precursor	R.LTVGAAQVPAQLLVGALR.V	3	5.05	0.27	-2.48
IPI00029260	Monocyte differentiation antigen CD14 precursor	R.NTGM*ETPTGVCAALAAAGVQPH.S	2	5.73	0.56	-3.29
IPI00029260	Monocyte differentiation antigen CD14 precursor	R.NTGM*ETPTGVCAALAAAGVQPH.S	3	3.87	0.37	-4.66
IPI00029260	Monocyte differentiation antigen CD14 precursor	R.NTGM*ETPTGVCAALAAAGVQPHSLDLSHNSLR.A	3	5.86	0.53	-3.37
IPI00029260	Monocyte differentiation antigen CD14 precursor	R.NTGM*ETPTGVCAALAAAGVQPHSLDLSHNSLR.A	4	7.12	0.58	-1.67
IPI00029260	Monocyte differentiation antigen CD14 precursor	R.RLTVGAAQVPAQLLVGALR.V	2	6.68	0.53	-5.07
IPI00029260	Monocyte differentiation antigen CD14 precursor	R.RLTVGAAQVPAQLLVGALR.V	3	5.07	0.44	-4.49
IPI00029260	Monocyte differentiation antigen CD14 precursor	R.SWLAELQQWLKPGLK.V	2	4.58	0.48	-4.57
IPI00029260	Monocyte differentiation antigen CD14 precursor	R.SWLAELQQWLKPGLK.V	3	2.82	0.25	-4.04
IPI00029260	Monocyte differentiation antigen CD14 precursor	R.VDADADPR.Q	2	2.33	0.08	-3.13
IPI00029260	Monocyte differentiation antigen CD14 precursor	R.VLDLSCNR.L	1	2.03	0.10	-2.46
IPI00029260	Monocyte differentiation antigen CD14 precursor	R.VLDLSCNR.L	2	3.06	0.26	-2.00
IPI00029260	Monocyte differentiation antigen CD14 precursor	V.PAQLLVGALR.V	1	2.73	0.25	-4.35
IPI00029260	Monocyte differentiation antigen CD14 precursor	W.LAELQQWLKPGLK.V	2	3.05	0.21	-4.16

IPI00029273	Isoform 1 of Hepatocyte growth factor receptor precursor	K.AFFM*LDGILSK.Y	2	3.08	0.34	-3.15
IPI00029273	Isoform 1 of Hepatocyte growth factor receptor precursor	K.EVFNILQAAYVSKPGAQLAR.Q	2	4.61	0.51	-2.02
IPI00029273	Isoform 1 of Hepatocyte growth factor receptor precursor	K.EVFNILQAAYVSKPGAQLAR.Q	3	4.04	0.44	-2.81
IPI00029273	Isoform 1 of Hepatocyte growth factor receptor precursor	K.GDLTIANLGTSEGR.F	2	4.23	0.45	-3.54
IPI00029273	Isoform 1 of Hepatocyte growth factor receptor precursor	K.NLNSVSVPR.M	2	2.27	0.14	1.92
IPI00029273	Isoform 1 of Hepatocyte growth factor receptor precursor	K.SFISGGSTITGVGK.N	2	2.60	0.32	-3.02
IPI00029273	Isoform 1 of Hepatocyte growth factor receptor precursor	R.FM*QVVVSR.S	2	2.65	0.20	-1.92
IPI00029273	Isoform 1 of Hepatocyte growth factor receptor precursor	R.TEFTTALQR.V	2	2.55	0.19	-2.31
IPI00029275	Isoform 1 of Melanotransferrin precursor	K.HSTVLENTDGK.T	2	2.19	0.36	-2.82
IPI00029275	Isoform 1 of Melanotransferrin precursor	K.TLPSWGQALLSQDFELLCR.D	3	3.58	0.33	-4.08
IPI00029275	Isoform 1 of Melanotransferrin precursor	R.ADTDGGLIFR.L	2	2.75	0.31	-3.54
IPI00029275	Isoform 1 of Melanotransferrin precursor	R.DSSHAFTLDELR.G	2	2.89	0.12	-2.19
IPI00029275	Isoform 1 of Melanotransferrin precursor	R.IQAEQVDAVTLSGEDIYTAGK.K	2	5.56	0.50	-3.88
IPI00029275	Isoform 1 of Melanotransferrin precursor	R.VPAHAVVVR.A	2	2.64	0.30	-3.49
IPI00029343	Isoform 1 of Contactin-associated protein-like 2 precursor	K.DAGFLSYK.D	2	2.48	0.17	-1.33
IPI00029343	Isoform 1 of Contactin-associated protein-like 2 precursor	K.DAGFLSYKDHLPVSQVVVGDTDR.Q	3	4.64	0.38	-5.05
IPI00029343	Isoform 1 of Contactin-associated protein-like 2 precursor	K.DAGFLSYKDHLPVSQVVVGDTDR.Q	4	2.81	0.12	-2.86
IPI00029343	Isoform 1 of Contactin-associated protein-like 2 precursor	K.DAGFLSYKDHLPVSQVVVGDTDRQGSEAK.L	4	3.93	0.24	-1.13
IPI00029343	Isoform 1 of Contactin-associated protein-like 2 precursor	K.DVIALNFK.T	1	2.21	0.14	-3.51
IPI00029343	Isoform 1 of Contactin-associated protein-like 2 precursor	K.DVIALNFK.T	2	2.75	0.08	-1.28
IPI00029343	Isoform 1 of Contactin-associated protein-like 2 precursor	K.LDHYPSVSYHLPSSSDTLFNSPK.S	3	3.27	0.26	-3.91
IPI00029343	Isoform 1 of Contactin-associated protein-like 2 precursor	K.M*SQIDISSGSGLNDGQWHEVR.F	3	3.88	0.45	-1.39
IPI00029343	Isoform 1 of Contactin-associated protein-like 2 precursor	K.QASLQVDRLPQQIR.K	2	2.86	0.17	-2.85
IPI00029343	Isoform 1 of Contactin-associated protein-like 2 precursor	K.QISAIATQGR.Y	1	2.00	0.21	-2.75

IPI00029343	Isoform 1 of Contactin-associated protein-like 2 precursor	K.TLKDVIALNFK.T	2	3.70	0.42	-2.88
IPI00029343	Isoform 1 of Contactin-associated protein-like 2 precursor	K.TLKDVIALNFK.T	3	2.88	0.11	-3.70
IPI00029343	Isoform 1 of Contactin-associated protein-like 2 precursor	K.VIETGKIDQEIHK.Y	2	2.32	0.15	-3.98
IPI00029343	Isoform 1 of Contactin-associated protein-like 2 precursor	K.YNTPGFTGCLSR.V	2	2.55	0.31	-3.00
IPI00029343	Isoform 1 of Contactin-associated protein-like 2 precursor	R.HELQHPIIAR.Y	2	2.44	0.29	-4.22
IPI00029343	Isoform 1 of Contactin-associated protein-like 2 precursor	R.KDAGFLSYKDHLPVSQVVVGDTDRQGSEAK.L	4	4.18	0.33	-2.13
IPI00029343	Isoform 1 of Contactin-associated protein-like 2 precursor	R.KQISAIATQGR.Y	2	3.06	0.27	-3.19
IPI00029343	Isoform 1 of Contactin-associated protein-like 2 precursor	R.LLNTPDGSPYTWWVGK.A	2	4.48	0.51	-3.50
IPI00029343	Isoform 1 of Contactin-associated protein-like 2 precursor	R.M*LYSDTGR.N	2	2.55	0.29	-3.03
IPI00029343	Isoform 1 of Contactin-associated protein-like 2 precursor	R.M*NGVTLDLEER.A	2	3.16	0.24	-2.95
IPI00029343	Isoform 1 of Contactin-associated protein-like 2 precursor	R.TNSPLQVK.T	2	2.08	0.07	0.02
IPI00029343	Isoform 1 of Contactin-associated protein-like 2 precursor	R.VDNAPDQQNSHPDLAQEEIR.F	3	2.64	0.27	-1.54
IPI00029343	Isoform 1 of Contactin-associated protein-like 2 precursor	R.VQFNQIAPLK.A	1	2.40	0.09	-2.30
IPI00029343	Isoform 1 of Contactin-associated protein-like 2 precursor	R.VQFNQIAPLK.A	2	3.07	0.19	-2.84
IPI00029343	Isoform 1 of Contactin-associated protein-like 2 precursor	R.YNFQAPATNAR.D	2	2.91	0.35	-2.01
IPI00029343	Isoform 1 of Contactin-associated protein-like 2 precursor	R.YNLGGTREPYNIDVDHR.N	4	2.71	0.27	-1.46
IPI00029343	Isoform 1 of Contactin-associated protein-like 2 precursor	R.YSSSDWVTQYR.M	2	3.57	0.39	-2.84
IPI00029372	Uncharacterized protein C4orf15	K.ISSLTSEIMK.L	2	2.78	0.11	
IPI00029533	Integrin beta-8 precursor	K.M*AFFSRDFR.L	2	2.27	0.15	
IPI00029556	Uncharacterized protein C1orf105	K.MVQPRTMKIPDDPK.A	3	4.09	0.14	
IPI00029591	P-selectin glycoprotein ligand 1 precursor	K.ALGPLLARD.R	1	2.06	0.39	-2.74
IPI00029605	N-acetylgalactosamine-6-sulfatase precursor	K.LPLIFHLGR.D	2	1.95	0.09	-2.40
IPI00029605	N-acetylgalactosamine-6-sulfatase precursor	R.AIDGLNLLPTLLQGR.L	2	4.21	0.40	-2.33
IPI00029605	N-acetylgalactosamine-6-sulfatase precursor	R.EIDDSIGK.I	2	1.80	0.17	-2.70
IPI00029605	N-acetylgalactosamine-6-sulfatase precursor	R.LM*DRPIFYYR.G	3	3.03	0.10	-2.48

IPI00029605	N-acetylgalactosamine-6-sulfatase precursor	R.NAYTPQEIVGGIPDSEQLLPELLKK.A	3	3.10	0.33	-1.20
IPI00029605	N-acetylgalactosamine-6-sulfatase precursor	R.NGFYTTNAHAR.N	2	1.28	0.19	-0.74
IPI00029605	N-acetylgalactosamine-6-sulfatase precursor	R.YYEEFPINLK.T	2	3.62	0.26	-2.51
IPI00029606	Isoform B of ADAM 17 precursor	A.PRPPDDPGFGPHQR.L	3	3.72	0.38	-2.31
IPI00029606	Isoform B of ADAM 17 precursor	K.LDSLLSDYDILSLSNIQQHSV.R	2	5.70	0.55	-2.67
IPI00029606	Isoform B of ADAM 17 precursor	K.LDSLLSDYDILSLSNIQQHSV.R	3	5.40	0.50	-3.96
IPI00029606	Isoform B of ADAM 17 precursor	R.LEKLDSLLSDYDILSLSNIQQHSV.R	2	5.06	0.44	-4.02
IPI00029606	Isoform B of ADAM 17 precursor	R.LEKLDSLLSDYDILSLSNIQQHSV.R	3	4.88	0.49	-4.46
IPI00029606	Isoform B of ADAM 17 precursor	R.LEKLDSLLSDYDILSLSNIQQHSVR.K	4	4.71	0.22	-2.52
IPI00029606	Isoform B of ADAM 17 precursor	R.LEKLDSLLSDYDILSLSNIQQHSVRK.R	4	4.20	0.41	-5.51
IPI00029623	Proteasome subunit alpha type-6	K.AINQGGLTSVAVR.G	2	3.09	0.28	-0.88
IPI00029623	Proteasome subunit alpha type-6	R.ILTEAEIDAHLVALAERD.-	3	3.24	0.23	-4.23
IPI00029647	Zymogen granule membrane protein 16 precursor	R.SSSYSGEYGSGGGKR.F	2	2.71	0.31	
IPI00029658	Isoform 1 of EGF-containing fibulin-like extracellular matrix protein 1 precursor	K.CVNHYGGYLCLPK.T	2	3.71	0.27	
IPI00029658	Isoform 1 of EGF-containing fibulin-like extracellular matrix protein 1 precursor	K.CVNHYGGYLCLPK.T	3	4.24	0.20	
IPI00029658	Isoform 1 of EGF-containing fibulin-like extracellular matrix protein 1 precursor	K.DIDECDIVPDACK.G	2	4.41	0.48	
IPI00029658	Isoform 1 of EGF-containing fibulin-like extracellular matrix protein 1 precursor	K.DIDECDIVPDACKGGM*K.C	2	4.48	0.38	
IPI00029658	Isoform 1 of EGF-containing fibulin-like extracellular matrix protein 1 precursor	K.FSCM*CPQGYQVVR.S	2	4.32	0.44	-2.16
IPI00029658	Isoform 1 of EGF-containing fibulin-like extracellular matrix protein 1 precursor	K.FSCMCPQGYQVVR.S	2	3.50	0.37	
IPI00029658	Isoform 1 of EGF-containing fibulin-like extracellular matrix protein 1 precursor	K.RGEQCVDIDECTIPPYCHQR.C	2	4.34	0.20	
IPI00029658	Isoform 1 of EGF-containing fibulin-like extracellular matrix protein 1 precursor	K.RGEQCVDIDECTIPPYCHQR.C	3	4.29	0.15	
IPI00029658	Isoform 1 of EGF-containing fibulin-like extracellular matrix protein 1 precursor	K.SGNENGEFYLR.Q	2	3.04	0.29	
IPI00029658	Isoform 1 of EGF-containing fibulin-like extracellular matrix protein 1 precursor	R.ADQVCINLR.G	1	2.07	0.06	
IPI00029658	Isoform 1 of EGF-containing fibulin-like extracellular matrix protein 1 precursor	R.ADQVCINLR.G	2	2.91	0.20	-1.70
IPI00029658	Isoform 1 of EGF-containing fibulin-like extracellular matrix protein 1 precursor	R.CVCPVSNAM*CR.E	2	3.23	0.34	
IPI00029658	Isoform 1 of EGF-containing fibulin-like extracellular matrix protein 1 precursor	R.CVCPVSNAMCR.E	2	2.68	0.21	
IPI00029658	Isoform 1 of EGF-containing fibulin-like extracellular matrix protein 1 precursor	R.CYPRNPCQDPYILTPENR.C	2	3.97	0.31	

IPI00029658	Isoform 1 of EGF-containing fibulin-like extracellular matrix protein 1 precursor	R.EHIVDLEM*LTVSSIGTFR.T	2	3.16	0.21	
IPI00029658	Isoform 1 of EGF-containing fibulin-like extracellular matrix protein 1 precursor	R.ELPQSIVYK.Y	1	2.75	0.18	
IPI00029658	Isoform 1 of EGF-containing fibulin-like extracellular matrix protein 1 precursor	R.GEQCVDIDECTIPPYCHQR.C	2	4.99	0.33	
IPI00029658	Isoform 1 of EGF-containing fibulin-like extracellular matrix protein 1 precursor	R.GSFACQCPPGYQK.R	2	4.91	0.27	
IPI00029658	Isoform 1 of EGF-containing fibulin-like extracellular matrix protein 1 precursor	R.IKSGNENGEFYLR.Q	2	3.34	0.21	
IPI00029658	Isoform 1 of EGF-containing fibulin-like extracellular matrix protein 1 precursor	R.IQCAAGYEQSEHNVCQDIDECTAGTHNCR.A	2	4.24	0.42	
IPI00029658	Isoform 1 of EGF-containing fibulin-like extracellular matrix protein 1 precursor	R.IQCAAGYEQSEHNVCQDIDECTAGTHNCR.A	3	6.94	0.42	
IPI00029658	Isoform 1 of EGF-containing fibulin-like extracellular matrix protein 1 precursor	R.NPADPQRIPSN.P	2	3.02	0.38	-3.01
IPI00029658	Isoform 1 of EGF-containing fibulin-like extracellular matrix protein 1 precursor	R.NPADPQRIPSNPSHR.I	2	1.69	0.10	-4.08
IPI00029658	Isoform 1 of EGF-containing fibulin-like extracellular matrix protein 1 precursor	R.NPADPQRIPSNPSHR.I	3	3.20	0.38	-3.16
IPI00029658	Isoform 1 of EGF-containing fibulin-like extracellular matrix protein 1 precursor	R.NPADPQRIPSNPSHR.I	4	3.32	0.28	-3.25
IPI00029658	Isoform 1 of EGF-containing fibulin-like extracellular matrix protein 1 precursor	R.NPCQDPYILTPENR.C	2	4.67	0.35	-3.49
IPI00029658	Isoform 1 of EGF-containing fibulin-like extracellular matrix protein 1 precursor	R.QQCKDIDECDIVPDACK.G	3	3.87	0.29	
IPI00029658	Isoform 1 of EGF-containing fibulin-like extracellular matrix protein 1 precursor	R.QTSPVSAM*LVLVK.S	2	3.01	0.32	
IPI00029658	Isoform 1 of EGF-containing fibulin-like extracellular matrix protein 1 precursor	R.QTSPVSAMLVLVK.S	2	2.85	0.26	
IPI00029658	Isoform 1 of EGF-containing fibulin-like extracellular matrix protein 1 precursor	R.SVPSDIFQIQATTIYANTINTFR.I	2	4.42	0.48	-4.20
IPI00029658	Isoform 1 of EGF-containing fibulin-like extracellular matrix protein 1 precursor	R.SVPSDIFQIQATTIYANTINTFR.I	3	4.19	0.34	
IPI00029658	Isoform 1 of EGF-containing fibulin-like extracellular matrix protein 1 precursor	R.TCQDINECETTNECREDEM*CWNYHGGFR.C	3	5.25	0.43	
IPI00029658	Isoform 1 of EGF-containing fibulin-like extracellular matrix protein 1 precursor	R.TSSYLCQYQCVNEPGKFSCM*CPQGYQVVR.S	3	5.20	0.46	
IPI00029693	Isoform A22 of Neuropilin-2 precursor	K.IVLNFNPHFEIEK.H	3	1.63	0.10	-1.30
IPI00029693	Isoform A22 of Neuropilin-2 precursor	K.LHAPLLTR.F	1	1.98	0.14	-4.67
IPI00029693	Isoform A22 of Neuropilin-2 precursor	K.VFQANNDATEVVLNK.L	2	4.40	0.36	-3.02
IPI00029693	Isoform A22 of Neuropilin-2 precursor	K.YDWLDIWDGIPHVGPLIGK.Y	3	4.48	0.39	-4.30

IPI00029693	Isoform A22 of Neuropilin-2 precursor	R.DGDSESADLLGK.H	2	3.94	0.37	-2.23
IPI00029693	Isoform A22 of Neuropilin-2 precursor	R.FLTM*LTAIATQGAISR.E	3	3.27	0.07	-3.60
IPI00029693	Isoform A22 of Neuropilin-2 precursor	R.GGDSITAVEAR.A	2	3.71	0.26	-2.52
IPI00029693	Isoform A22 of Neuropilin-2 precursor	R.IANEQISASSTYSDGR.W	2	4.50	0.52	-4.72
IPI00029693	Isoform A22 of Neuropilin-2 precursor	R.LELFGCR.V	2	2.46	0.16	-1.26
IPI00029693	Isoform A22 of Neuropilin-2 precursor	R.SGEIAIDDIR.I	2	3.54	0.20	-5.71
IPI00029693	Isoform A22 of Neuropilin-2 precursor	R.SPVCM*EFQYQATGGR.G	2	2.65	0.39	-4.13
IPI00029699	Ribonuclease 4 precursor	R.FNTFIHEDIWNIR.S	2	4.03	0.36	
IPI00029699	Ribonuclease 4 precursor	R.FNTFIHEDIWNIR.S	3	3.83	0.23	
IPI00029699	Ribonuclease 4 precursor	R.RVVIACEGNPQVPVHFDG.-	2	3.31	0.31	
IPI00029699	Ribonuclease 4 precursor	R.SICSTTNIQCK.N	2	3.49	0.29	
IPI00029699	Ribonuclease 4 precursor	R.YCNLM*M*QR.R	2	2.28	0.25	
IPI00029700	Isoform Long of Down syndrome cell adhesion molecule precursor	K.VSNDVGADVSK.S	2	3.30	0.28	-2.46
IPI00029700	Isoform Long of Down syndrome cell adhesion molecule precursor	R.VIGYPYYSIK.W	2	2.25	0.18	-2.81
IPI00029722	Kinesin heavy chain isoform 5A	R.LRATAERVKALEGALKEAKEGAMKDKR.R	3	3.06	0.18	
IPI00029723	Follistatin-related protein 1 precursor	K.CLNPSFNPPEK.K	2	2.59	0.22	-1.57
IPI00029723	Follistatin-related protein 1 precursor	K.GAQTQTEEEM*TR.Y	2	4.23	0.41	-3.81
IPI00029723	Follistatin-related protein 1 precursor	K.GAQTQTEEEM*TR.Y	3	3.73	0.27	-3.23
IPI00029723	Follistatin-related protein 1 precursor	K.GAQTQTEEEM*TRYVQELQK.H	3	2.74	0.28	-2.08
IPI00029723	Follistatin-related protein 1 precursor	K.ICANVFCGAGR.E	2	3.90	0.47	-1.88
IPI00029723	Follistatin-related protein 1 precursor	K.LSFQEFLK.C	2	2.99	0.14	-1.57
IPI00029723	Follistatin-related protein 1 precursor	K.NFDNGDSRLDSSEFLK.F	2	4.21	0.40	-4.00
IPI00029723	Follistatin-related protein 1 precursor	R.CVCACGNWVCTAM*TCDGK.N	2	4.70	0.66	-2.56
IPI00029723	Follistatin-related protein 1 precursor	R.DACLTGSK.I	2	1.81	0.14	-4.42
IPI00029723	Follistatin-related protein 1 precursor	R.YVQELQKHQETAEK.T	2	4.48	0.42	-4.98
IPI00029723	Follistatin-related protein 1 precursor	R.YVQELQKHQETAEK.T	3	2.55	0.27	-2.05
IPI00029739	Isoform 1 of Complement factor H precursor	K.AGEQVTYTCATYYK.M	2	4.95	0.53	-2.13
IPI00029739	Isoform 1 of Complement factor H precursor	K.AGEQVTYTCATYYK.M	3	4.26	0.10	
IPI00029739	Isoform 1 of Complement factor H precursor	K.AQTTVTCM*ENGWSPTPR.C	2	4.82	0.42	
IPI00029739	Isoform 1 of Complement factor H precursor	K.AQTTVTCMENGWSPTPR.C	2	2.54	0.17	
IPI00029739	Isoform 1 of Complement factor H precursor	K.AVYTCNEGYQLLGEINYR.E	2	5.34	0.49	-2.57
IPI00029739	Isoform 1 of Complement factor H precursor	K.AVYTCNEGYQLLGEINYR.E	3	3.53	0.09	-2.56
IPI00029739	Isoform 1 of Complement factor H precursor	K.AVYTCNEGYQLLGEINYRECDTDGWTNDIPICEVVK.C	3	5.72	0.44	
IPI00029739	Isoform 1 of Complement factor H precursor	K.CFEGFGIDGPAIAK.C	1	3.29	0.33	
IPI00029739	Isoform 1 of Complement factor H precursor	K.CFEGFGIDGPAIAK.C	2	4.59	0.54	-2.74
IPI00029739	Isoform 1 of Complement factor H precursor	K.CGPPPPIDNGDITSFPLSVYAPASSVEYQCQNLYQLEGNKR.I	3	5.17	0.49	-3.33
IPI00029739	Isoform 1 of Complement factor H precursor	K.CLPVTAPENGK.I	1	2.54	0.15	
IPI00029739	Isoform 1 of Complement factor H precursor	K.CLPVTAPENGK.I	2	3.06	0.24	
IPI00029739	Isoform 1 of Complement factor H precursor	K.CNM*GYEYSER.G	2	2.74	0.30	-3.66
IPI00029739	Isoform 1 of Complement factor H precursor	K.CNM*GYEYSERGDAVCTESGWRPLPSCEEK.S	3	4.54	0.33	

IPI00029739	Isoform 1 of Complement factor H precursor	K.CNMGYEYSERGDAVCTESGWRPLPSCEEK.S	3	5.47	0.44	
IPI00029739	Isoform 1 of Complement factor H precursor	K.CTSTGWIPAPR.C	2	2.94	0.45	-2.15
IPI00029739	Isoform 1 of Complement factor H precursor	K.CVEISCKSPDVINGSPISQK.I	2	6.46	0.45	
IPI00029739	Isoform 1 of Complement factor H precursor	K.CVEISCKSPDVINGSPISQK.I	3	5.11	0.16	
IPI00029739	Isoform 1 of Complement factor H precursor	K.CYFPYLENGYNQNYGR.K	2	4.76	0.43	
IPI00029739	Isoform 1 of Complement factor H precursor	K.CYFPYLENGYNQNYGR.K	3	5.71	0.28	
IPI00029739	Isoform 1 of Complement factor H precursor	K.DGWSAQPTCIK.S	2	2.82	0.13	-2.43
IPI00029739	Isoform 1 of Complement factor H precursor	K.DQYKVGEVLK.F	2	2.59	0.20	-0.68
IPI00029739	Isoform 1 of Complement factor H precursor	K.EFDHNSNIR.Y	2	2.11	0.14	-3.36
IPI00029739	Isoform 1 of Complement factor H precursor	K.EGWIHTVCINGR.W	2	2.50	0.30	
IPI00029739	Isoform 1 of Complement factor H precursor	K.EKTKEEYGHSEVVEYYCNPR.F	2	6.02	0.45	
IPI00029739	Isoform 1 of Complement factor H precursor	K.EKTKEEYGHSEVVEYYCNPR.F	3	5.55	0.32	
IPI00029739	Isoform 1 of Complement factor H precursor	K.EQVQSCGPPPELLNGNVK.E	2	4.88	0.44	-2.16
IPI00029739	Isoform 1 of Complement factor H precursor	K.EQVQSCGPPPELLNGNVK.E	3	4.08	0.39	-3.31
IPI00029739	Isoform 1 of Complement factor H precursor	K.FSCKPGFTIVGPNSVQCYHFGLSPDLPICK.E	3	4.87	0.34	
IPI00029739	Isoform 1 of Complement factor H precursor	K.IDVHLVPDR.K	1	2.03	0.27	-1.65
IPI00029739	Isoform 1 of Complement factor H precursor	K.IDVHLVPDR.K	2	2.49	0.29	-2.14
IPI00029739	Isoform 1 of Complement factor H precursor	K.IIYKENER.F	1	2.62	0.07	
IPI00029739	Isoform 1 of Complement factor H precursor	K.IIYKENER.F	2	2.39	0.18	-3.55
IPI00029739	Isoform 1 of Complement factor H precursor	K.IIYKENERFQYK.C	2	2.83	0.05	
IPI00029739	Isoform 1 of Complement factor H precursor	K.IVSSAM*EPDR.E	2	2.81	0.18	
IPI00029739	Isoform 1 of Complement factor H precursor	K.IVSSAM*EPDREYHFGQAVR.F	2	1.86	0.22	-4.73
IPI00029739	Isoform 1 of Complement factor H precursor	K.IVSSAM*EPDREYHFGQAVR.F	3	3.16	0.38	-3.55
IPI00029739	Isoform 1 of Complement factor H precursor	K.LGYVTADGETSGSITCGK.D	2	5.71	0.50	
IPI00029739	Isoform 1 of Complement factor H precursor	K.LGYVTADGETSGSITCGKDGWSAQPTCIK.S	3	6.54	0.56	
IPI00029739	Isoform 1 of Complement factor H precursor	K.LSYTCEGGFR.I	1	1.81	0.22	
IPI00029739	Isoform 1 of Complement factor H precursor	K.LSYTCEGGFR.I	2	3.13	0.42	-2.74
IPI00029739	Isoform 1 of Complement factor H precursor	K.RPCGHPGDTPFGTFTLTGGNVFEYGVK.A	3	6.37	0.52	
IPI00029739	Isoform 1 of Complement factor H precursor	K.SCDIPVFM*NAR.T	2	2.94	0.15	
IPI00029739	Isoform 1 of Complement factor H precursor	K.SCDIPVFMNAR.T	2	3.35	0.26	
IPI00029739	Isoform 1 of Complement factor H precursor	K.SCDNPYIPNGDYSPLR.I	2	4.05	0.42	-1.90
IPI00029739	Isoform 1 of Complement factor H precursor	K.SIDVACHPGYALPK.A	1	2.38	0.12	
IPI00029739	Isoform 1 of Complement factor H precursor	K.SIDVACHPGYALPK.A	2	3.96	0.28	
IPI00029739	Isoform 1 of Complement factor H precursor	K.SPDVINGSPISQK.I	2	4.04	0.29	-1.68
IPI00029739	Isoform 1 of Complement factor H precursor	K.SPPEISHGVVAHM*SDSYQYGEEVTYK.C	3	6.35	0.48	
IPI00029739	Isoform 1 of Complement factor H precursor	K.SPPEISHGVVAHMSDSYQYGEEVTYK.C	3	4.27	0.28	
IPI00029739	Isoform 1 of Complement factor H precursor	K.SSIDIENGFISESQYTYALK.E	2	6.00	0.46	
IPI00029739	Isoform 1 of Complement factor H precursor	K.SSIDIENGFISESQYTYALKEK.A	3	3.33	0.08	
IPI00029739	Isoform 1 of Complement factor H precursor	K.SSNLIILEEHLK.N	1	3.59	0.31	
IPI00029739	Isoform 1 of Complement factor H precursor	K.SSNLIILEEHLK.N	2	3.81	0.32	-2.64
IPI00029739	Isoform 1 of Complement factor H precursor	K.SSNLIILEEHLK.N	3	3.54	0.14	-2.54
IPI00029739	Isoform 1 of Complement factor H precursor	K.TDCLSLPSFENAIPM*GEK.K	2	3.76	0.22	

IPI00029739	Isoform 1 of Complement factor H precursor	K.TDCLSLPSFENAIPM*GEKK.D	2	2.62	0.20	
IPI00029739	Isoform 1 of Complement factor H precursor	K.TDCLSLPSFENAIPM*GEKK.D	3	3.28	0.31	
IPI00029739	Isoform 1 of Complement factor H precursor	K.TKEEYGHSEVVEYYCNPR.F	2	6.47	0.50	
IPI00029739	Isoform 1 of Complement factor H precursor	K.TKEEYGHSEVVEYYCNPR.F	3	5.89	0.33	
IPI00029739	Isoform 1 of Complement factor H precursor	K.TKEEYGHSEVVEYYCNPR.F	4	3.01	0.33	-3.01
IPI00029739	Isoform 1 of Complement factor H precursor	K.VSVLCQENYLIQEGEEITCK.D	2	4.95	0.33	
IPI00029739	Isoform 1 of Complement factor H precursor	K.VSVLCQENYLIQEGEEITCK.D	3	5.17	0.23	
IPI00029739	Isoform 1 of Complement factor H precursor	K.VSVLCQENYLIQEGEEITCKDGR.W	2	3.41	0.31	
IPI00029739	Isoform 1 of Complement factor H precursor	K.VSVLCQENYLIQEGEEITCKDGR.W	3	3.36	0.24	
IPI00029739	Isoform 1 of Complement factor H precursor	K.WSSPPQCEGLPCK.S	2	3.07	0.29	-2.43
IPI00029739	Isoform 1 of Complement factor H precursor	R.CIRVKTCSKSSIDIENGFISESQYTYALK.E	3	3.64	0.19	
IPI00029739	Isoform 1 of Complement factor H precursor	R.ECDTDGWTNDIPICEVVK.C	2	4.65	0.38	
IPI00029739	Isoform 1 of Complement factor H precursor	R.ECELPKIDVHLVPDR.K	2	3.44	0.30	
IPI00029739	Isoform 1 of Complement factor H precursor	R.ECELPKIDVHLVPDR.K	3	3.47	0.43	
IPI00029739	Isoform 1 of Complement factor H precursor	R.EIM*ENYNIALR.W	2	2.73	0.35	-4.22
IPI00029739	Isoform 1 of Complement factor H precursor	R.FVCNSGYK.I	1	2.08	0.13	
IPI00029739	Isoform 1 of Complement factor H precursor	R.FVCNSGYKIEGDEEM*HCSDDGFWSK.E	3	4.93	0.39	
IPI00029739	Isoform 1 of Complement factor H precursor	R.FVCNSGYKIEGDEEMHCSDDGFWSK.E	3	5.66	0.38	
IPI00029739	Isoform 1 of Complement factor H precursor	R.GDAVCTESGWRPLPSCEEK.S	2	4.59	0.41	
IPI00029739	Isoform 1 of Complement factor H precursor	R.GDAVCTESGWRPLPSCEEK.S	3	3.58	0.25	
IPI00029739	Isoform 1 of Complement factor H precursor	R.KCYFPYLENGYNQNYGR.K	2	4.78	0.38	
IPI00029739	Isoform 1 of Complement factor H precursor	R.KGEWVALNPLR.K	2	3.51	0.23	
IPI00029739	Isoform 1 of Complement factor H precursor	R.KGEWVALNPLR.K	3	4.13	0.10	
IPI00029739	Isoform 1 of Complement factor H precursor	R.NGFYPATR.G	2	2.29	0.11	
IPI00029739	Isoform 1 of Complement factor H precursor	R.NGQWSEPPKCLHPCVISR.E	3	3.60	0.35	
IPI00029739	Isoform 1 of Complement factor H precursor	R.NTEILTGSWSDQTYPEGTQAIYK.C	2	4.68	0.58	-3.39
IPI00029739	Isoform 1 of Complement factor H precursor	R.NTEILTGSWSDQTYPEGTQAIYK.C	3	3.65	0.38	-5.24
IPI00029739	Isoform 1 of Complement factor H precursor	R.QMSKYPSGER.V	2	2.87	0.22	
IPI00029739	Isoform 1 of Complement factor H precursor	R.RNTEILTGSWSDQTYPEGTQAIYK.C	2	5.12	0.41	
IPI00029739	Isoform 1 of Complement factor H precursor	R.RNTEILTGSWSDQTYPEGTQAIYK.C	3	3.88	0.25	
IPI00029739	Isoform 1 of Complement factor H precursor	R.RPYFPVAVGK.Y	1	1.68	0.07	-2.78
IPI00029739	Isoform 1 of Complement factor H precursor	R.RPYFPVAVGK.Y	2	3.15	0.20	-3.65
IPI00029739	Isoform 1 of Complement factor H precursor	R.SITCIHGVWTQLPQCVAIDK.L	2	5.59	0.44	
IPI00029739	Isoform 1 of Complement factor H precursor	R.SITCIHGVWTQLPQCVAIDK.L	3	2.84	0.07	-3.05
IPI00029739	Isoform 1 of Complement factor H precursor	R.SITCIHGVWTQLPQCVAIDKLKK.C	3	4.31	0.16	
IPI00029739	Isoform 1 of Complement factor H precursor	R.SLGNVIM*VCR.K	2	2.91	0.37	0.97
IPI00029739	Isoform 1 of Complement factor H precursor	R.SLGNVIMVCR.K	2	3.70	0.35	
IPI00029739	Isoform 1 of Complement factor H precursor	R.SSQESYAHGTK.L	2	2.43	0.27	-2.59
IPI00029739	Isoform 1 of Complement factor H precursor	R.TGESVEFVCK.R	2	3.27	0.31	
IPI00029739	Isoform 1 of Complement factor H precursor	R.TGESVEFVCKR.G	2	3.18	0.31	
IPI00029739	Isoform 1 of Complement factor H precursor	R.TKNDFTWFK.L	2	2.78	0.25	
IPI00029739	Isoform 1 of Complement factor H precursor	R.TTCWDGKLEYPTCAK.R	2	4.43	0.42	

IPI00029739	Isoform 1 of Complement factor H precursor	R.WQSIPLCVEK.I	1	1.89	0.10	
IPI00029739	Isoform 1 of Complement factor H precursor	R.WQSIPLCVEK.I	2	2.71	0.20	-2.73
IPI00029739	Isoform 1 of Complement factor H precursor	T.DTSCVNPPTVQNAYIVSR.Q	2	4.29	0.41	-4.43
IPI00029739	Isoform 1 of Complement factor H precursor	T.DTSCVNPPTVQNAYIVSR.Q	3	3.75	0.42	-4.38
IPI00029751	Isoform 1 of Contactin-1 precursor	A.HSDGGDGVVSQVK.I	2	3.98	0.52	-2.43
IPI00029751	Isoform 1 of Contactin-1 precursor	A.PSDVGGGGGR.N	1	2.07	0.26	-1.21
IPI00029751	Isoform 1 of Contactin-1 precursor	D.GEYVVEVR.A	2	2.93	0.31	-0.95
IPI00029751	Isoform 1 of Contactin-1 precursor	D.PPIIEGNM*EAAR.A	2	3.27	0.37	-1.55
IPI00029751	Isoform 1 of Contactin-1 precursor	D.PPYHFPDDLSYR.W	2	4.01	0.38	-4.53
IPI00029751	Isoform 1 of Contactin-1 precursor	D.PPYHFPDDLSYR.W	3	4.63	0.43	-1.13
IPI00029751	Isoform 1 of Contactin-1 precursor	G.VSEEDKGFGPIFEEQPINTIYPEESLEGK.V	3	5.30	0.49	-3.02
IPI00029751	Isoform 1 of Contactin-1 precursor	H.SDGGDGVVSQVK.I	2	3.90	0.28	-3.16
IPI00029751	Isoform 1 of Contactin-1 precursor	I.NSAQDAPSEAPTEVGVK.V	2	4.33	0.45	-2.78
IPI00029751	Isoform 1 of Contactin-1 precursor	K.AFNNKGDGPYSLVAVINSAQDAPSEAPTEVGVK.V	2	4.60	0.51	-1.44
IPI00029751	Isoform 1 of Contactin-1 precursor	K.AFNNKGDGPYSLVAVINSAQDAPSEAPTEVGVK.V	3	6.51	0.57	-2.78
IPI00029751	Isoform 1 of Contactin-1 precursor	K.AFNNKGDGPYSLVAVINSAQDAPSEAPTEVGVK.V	4	4.52	0.43	-2.66
IPI00029751	Isoform 1 of Contactin-1 precursor	K.AFNNKGDGPYSLVAVINSAQDAPSEAPTEVGVK.V	5	2.79	0.16	-1.25
IPI00029751	Isoform 1 of Contactin-1 precursor	K.DAGIYYCLASNNYGM*VR.S	2	5.44	0.64	-6.31
IPI00029751	Isoform 1 of Contactin-1 precursor	K.DAGIYYCLASNNYGM*VR.S	3	5.41	0.51	-4.89
IPI00029751	Isoform 1 of Contactin-1 precursor	K.DAKTDPPIIEGNM*EAAR.A	2	3.84	0.41	-3.77
IPI00029751	Isoform 1 of Contactin-1 precursor	K.DAKTDPPIIEGNM*EAAR.A	3	4.72	0.18	-2.84
IPI00029751	Isoform 1 of Contactin-1 precursor	K.ENIHYQR.N	1	2.32	0.17	0.42
IPI00029751	Isoform 1 of Contactin-1 precursor	K.FIPLIPIPER.T	1	2.97	0.20	-3.98
IPI00029751	Isoform 1 of Contactin-1 precursor	K.FIPLIPIPER.T	2	2.40	0.11	-4.48
IPI00029751	Isoform 1 of Contactin-1 precursor	K.GDGPYSLVAVINSAQDAPSEAPTEVGVK.V	2	5.54	0.53	-4.13
IPI00029751	Isoform 1 of Contactin-1 precursor	K.GFGPIFEEQPINTIYPEESLEGK.V	2	4.44	0.51	-6.42
IPI00029751	Isoform 1 of Contactin-1 precursor	K.GM*VLLCDPPYHFPDDLSYR.W	2	4.37	0.58	-3.90
IPI00029751	Isoform 1 of Contactin-1 precursor	K.GM*VLLCDPPYHFPDDLSYR.W	3	3.58	0.24	-3.42
IPI00029751	Isoform 1 of Contactin-1 precursor	K.GMVLLCDPPYHFPDDLSYR.W	2	3.66	0.42	-2.74
IPI00029751	Isoform 1 of Contactin-1 precursor	K.HSIEVPIPR.D	1	2.23	0.35	-2.47
IPI00029751	Isoform 1 of Contactin-1 precursor	K.HSIEVPIPR.D	2	2.55	0.26	-1.72
IPI00029751	Isoform 1 of Contactin-1 precursor	K.HSIEVPIPRDGEYVVEVR.A	2	3.14	0.28	-5.01
IPI00029751	Isoform 1 of Contactin-1 precursor	K.HSIEVPIPRDGEYVVEVR.A	3	2.60	0.25	-2.86
IPI00029751	Isoform 1 of Contactin-1 precursor	K.HSIEVPIPRDGEYVVEVR.A	4	2.66	0.15	-1.69
IPI00029751	Isoform 1 of Contactin-1 precursor	K.IFNIQLEDEGIYECEAENIR.G	2	6.70	0.61	-4.70
IPI00029751	Isoform 1 of Contactin-1 precursor	K.IFNIQLEDEGIYECEAENIR.G	3	7.30	0.53	-5.46
IPI00029751	Isoform 1 of Contactin-1 precursor	K.IFNIQLEDEGIYECEAENIRGK.D	3	4.10	0.30	-3.14
IPI00029751	Isoform 1 of Contactin-1 precursor	K.ILALAPTFEM*NPM*K.K	2	3.94	0.42	-4.24
IPI00029751	Isoform 1 of Contactin-1 precursor	K.ILALAPTFEM*NPM*K.K	3	4.44	0.42	-1.12
IPI00029751	Isoform 1 of Contactin-1 precursor	K.ILALAPTFEM*NPM*KK.K	2	2.34	0.21	-1.95
IPI00029751	Isoform 1 of Contactin-1 precursor	K.ILALAPTFEMNPMK.K	2	2.77	0.17	
IPI00029751	Isoform 1 of Contactin-1 precursor	K.IVESYQIR.Y	1	2.41	0.16	-2.56

IPI00029751	Isoform 1 of Contactin-1 precursor	K.IVESYQIR.Y	2	3.21	0.24	-1.44
IPI00029751	Isoform 1 of Contactin-1 precursor	K.KVTVTNPDTGR.Y	1	2.95	0.17	-1.43
IPI00029751	Isoform 1 of Contactin-1 precursor	K.KVTVTNPDTGR.Y	2	3.06	0.33	-2.74
IPI00029751	Isoform 1 of Contactin-1 precursor	K.NGYAYHKGELR.L	2	3.40	0.42	-2.79
IPI00029751	Isoform 1 of Contactin-1 precursor	K.TDGAAPNVAPSDVGGGGGR.N	2	4.30	0.50	-2.88
IPI00029751	Isoform 1 of Contactin-1 precursor	K.TDGAAPNVAPSDVGGGGGR.N	3	3.88	0.41	-2.35
IPI00029751	Isoform 1 of Contactin-1 precursor	K.TDPPIIEGNM*EAAR.A	2	2.38	0.33	-4.55
IPI00029751	Isoform 1 of Contactin-1 precursor	K.TDPPIIEGNM*EAAR.A	3	2.82	0.33	-2.11
IPI00029751	Isoform 1 of Contactin-1 precursor	K.TILSDDWK.D	1	1.85	0.23	-3.49
IPI00029751	Isoform 1 of Contactin-1 precursor	K.TILSDDWKDAKTDPPIIEGNM*EAAR.A	3	3.73	0.34	-1.65
IPI00029751	Isoform 1 of Contactin-1 precursor	K.VKAFNNKGDGPYSLVAVINSAQDAPSEAPTEVGVK.V	3	5.40	0.40	-3.93
IPI00029751	Isoform 1 of Contactin-1 precursor	K.VKAFNNKGDGPYSLVAVINSAQDAPSEAPTEVGVK.V	4	3.72	0.28	-4.51
IPI00029751	Isoform 1 of Contactin-1 precursor	K.VLEPM*PSTAEISTSGAVLK.I	2	4.92	0.57	-3.39
IPI00029751	Isoform 1 of Contactin-1 precursor	K.VLEPM*PSTAEISTSGAVLK.I	3	4.23	0.41	-2.39
IPI00029751	Isoform 1 of Contactin-1 precursor	K.VLSSSEISVHWEHVLEK.I	2	4.49	0.33	-4.44
IPI00029751	Isoform 1 of Contactin-1 precursor	K.VLSSSEISVHWEHVLEK.I	4	1.95	0.27	-2.25
IPI00029751	Isoform 1 of Contactin-1 precursor	K.VLYRPDGQHDGK.L	2	3.46	0.43	-3.29
IPI00029751	Isoform 1 of Contactin-1 precursor	K.VLYRPDGQHDGK.L	3	2.34	0.26	-3.77
IPI00029751	Isoform 1 of Contactin-1 precursor	K.VLYRPDGQHDGKLYSTHK.H	3	3.41	0.23	-3.38
IPI00029751	Isoform 1 of Contactin-1 precursor	K.VTVTNPDTGR.Y	2	3.47	0.35	-3.99
IPI00029751	Isoform 1 of Contactin-1 precursor	K.YTIQTK.T	1	2.16	0.11	-1.94
IPI00029751	Isoform 1 of Contactin-1 precursor	L.VAVINSAQDAPSEAPTEVGVK.V	2	6.50	0.61	-3.45
IPI00029751	Isoform 1 of Contactin-1 precursor	L.VAVINSAQDAPSEAPTEVGVK.V	3	4.24	0.37	-4.56
IPI00029751	Isoform 1 of Contactin-1 precursor	N.SAQDAPSEAPTEVGVK.V	2	4.14	0.38	-3.96
IPI00029751	Isoform 1 of Contactin-1 precursor	Q.DAPSEAPTEVGVK.V	2	3.07	0.26	-2.63
IPI00029751	Isoform 1 of Contactin-1 precursor	R.AHSDGGDGVVSQVK.I	1	3.37	0.45	-3.57
IPI00029751	Isoform 1 of Contactin-1 precursor	R.AHSDGGDGVVSQVK.I	2	4.26	0.60	-4.41
IPI00029751	Isoform 1 of Contactin-1 precursor	R.AHSDGGDGVVSQVK.I	3	2.35	0.10	-4.34
IPI00029751	Isoform 1 of Contactin-1 precursor	R.ASPFPVYK.W	1	1.96	0.31	-2.80
IPI00029751	Isoform 1 of Contactin-1 precursor	R.ATSVALTWSR.G	1	2.00	0.14	-3.13
IPI00029751	Isoform 1 of Contactin-1 precursor	R.ATSVALTWSR.G	2	3.54	0.30	-3.84
IPI00029751	Isoform 1 of Contactin-1 precursor	R.AVDLIPWM*EYEFR.V	2	3.76	0.48	-3.63
IPI00029751	Isoform 1 of Contactin-1 precursor	R.DGEYVVEVR.A	1	2.60	0.18	-3.49
IPI00029751	Isoform 1 of Contactin-1 precursor	R.DGEYVVEVR.A	2	3.85	0.29	-3.29
IPI00029751	Isoform 1 of Contactin-1 precursor	R.ELTITWAPLSR.E	1	2.58	0.14	-3.87
IPI00029751	Isoform 1 of Contactin-1 precursor	R.ELTITWAPLSR.E	2	3.76	0.37	-4.32
IPI00029751	Isoform 1 of Contactin-1 precursor	R.FVSQTNGNLYIANVEASDK.G	2	5.58	0.47	-3.82
IPI00029751	Isoform 1 of Contactin-1 precursor	R.GEPSIPSNR.I	2	1.72	0.07	-1.71
IPI00029751	Isoform 1 of Contactin-1 precursor	R.GPPGPPGGLR.I	1	2.67	0.21	-1.32
IPI00029751	Isoform 1 of Contactin-1 precursor	R.GPPGPPGGLR.I	2	2.81	0.19	-0.40
IPI00029751	Isoform 1 of Contactin-1 precursor	R.GPPGPPGGLRIEDIR.A	3	1.78	0.22	-2.00
IPI00029751	Isoform 1 of Contactin-1 precursor	R.GSDNHSPISK.Y	1	2.34	0.17	-3.77

IPI00029751	Isoform 1 of Contactin-1 precursor	R.GSDNHSPISK.Y	2	2.75	0.22	-2.61
IPI00029751	Isoform 1 of Contactin-1 precursor	R.GSDNHSPISKYTIQTK.T	2	3.14	0.28	-3.80
IPI00029751	Isoform 1 of Contactin-1 precursor	R.IKTDGAAPNVAPSDVGGGGG.R	2	4.76	0.34	-3.40
IPI00029751	Isoform 1 of Contactin-1 precursor	R.IKTDGAAPNVAPSDVGGGGGR.N	2	5.38	0.54	-3.07
IPI00029751	Isoform 1 of Contactin-1 precursor	R.IKTDGAAPNVAPSDVGGGGGR.N	3	3.38	0.41	-1.32
IPI00029751	Isoform 1 of Contactin-1 precursor	R.KVLEPM*PSTAEISTSGAVLK.I	2	4.27	0.46	-4.10
IPI00029751	Isoform 1 of Contactin-1 precursor	R.KVLEPM*PSTAEISTSGAVLK.I	3	4.35	0.35	-4.11
IPI00029751	Isoform 1 of Contactin-1 precursor	R.LENLLPDTQYFIEVGACNSAGCGPPSDM*IEAFTKK.A	3	4.51	0.41	-5.26
IPI00029751	Isoform 1 of Contactin-1 precursor	R.LENLLPDTQYFIEVGACNSAGCGPPSDM*IEAFTKK.A	4	5.14	0.42	-4.91
IPI00029751	Isoform 1 of Contactin-1 precursor	R.LYDVTFENAGM*YQCIAENTYGAIYANAELK.I	3	6.34	0.54	-4.40
IPI00029751	Isoform 1 of Contactin-1 precursor	R.LYDVTFENAGM*YQCIAENTYGAIYANAELK.I	4	5.31	0.41	-4.23
IPI00029751	Isoform 1 of Contactin-1 precursor	R.M*NNGDVDLTSDR.Y	1	2.01	0.24	-4.44
IPI00029751	Isoform 1 of Contactin-1 precursor	R.M*NNGDVDLTSDR.Y	2	4.16	0.30	-6.15
IPI00029751	Isoform 1 of Contactin-1 precursor	R.M*NNGDVDLTSDRYSM*VGGNLVINNPDK.Q	3	5.64	0.54	-3.35
IPI00029751	Isoform 1 of Contactin-1 precursor	R.M*NNGDVDLTSDRYSM*VGGNLVINNPDKQK.D	3	5.18	0.55	-3.46
IPI00029751	Isoform 1 of Contactin-1 precursor	R.M*NNGDVDLTSDRYSM*VGGNLVINNPDKQK.D	4	5.10	0.47	-0.93
IPI00029751	Isoform 1 of Contactin-1 precursor	R.NDGGIYTCFAENNR.G	2	3.84	0.45	-3.81
IPI00029751	Isoform 1 of Contactin-1 precursor	R.NFM*LDSNGELLIR.N	2	4.95	0.47	-6.88
IPI00029751	Isoform 1 of Contactin-1 precursor	R.NRELTITWAPLSR.E	2	3.63	0.30	-3.94
IPI00029751	Isoform 1 of Contactin-1 precursor	R.NRELTITWAPLSR.E	3	4.14	0.23	-3.17
IPI00029751	Isoform 1 of Contactin-1 precursor	R.RYGHGVSEEDKGFGPIFEEQPINTIYPEESLEGK.V	3	6.66	0.61	-4.84
IPI00029751	Isoform 1 of Contactin-1 precursor	R.RYGHGVSEEDKGFGPIFEEQPINTIYPEESLEGK.V	4	4.51	0.42	-4.21
IPI00029751	Isoform 1 of Contactin-1 precursor	R.RYGHGVSEEDKGFGPIFEEQPINTIYPEESLEGK.V	5	3.74	0.28	-5.48
IPI00029751	Isoform 1 of Contactin-1 precursor	R.STEATLSFGYLDPFPPEERPEVR.V	2	4.04	0.44	-2.76
IPI00029751	Isoform 1 of Contactin-1 precursor	R.STEATLSFGYLDPFPPEERPEVR.V	3	5.04	0.54	-7.39
IPI00029751	Isoform 1 of Contactin-1 precursor	R.STEATLSFGYLDPFPPEERPEVR.V	4	3.77	0.29	-2.07
IPI00029751	Isoform 1 of Contactin-1 precursor	R.TTKPYPADIVVQFK.D	2	4.16	0.50	-3.74
IPI00029751	Isoform 1 of Contactin-1 precursor	R.TTKPYPADIVVQFK.D	3	5.11	0.40	-2.87
IPI00029751	Isoform 1 of Contactin-1 precursor	R.VQVTSQEYSAR.L	1	2.58	0.35	-4.27
IPI00029751	Isoform 1 of Contactin-1 precursor	R.VQVTSQEYSAR.L	2	4.22	0.40	-4.15
IPI00029751	Isoform 1 of Contactin-1 precursor	R.VVATNTLGR.G	1	1.78	0.14	-2.37
IPI00029751	Isoform 1 of Contactin-1 precursor	R.VVATNTLGR.G	2	2.59	0.31	-2.51
IPI00029751	Isoform 1 of Contactin-1 precursor	R.VVATNTLGRGEPSIPSNR.I	2	3.17	0.15	-3.35
IPI00029751	Isoform 1 of Contactin-1 precursor	R.VVATNTLGRGEPSIPSNR.I	3	4.06	0.19	-3.63
IPI00029751	Isoform 1 of Contactin-1 precursor	R.WLLNEFPVFITM*DK.R	2	5.48	0.46	-6.56
IPI00029751	Isoform 1 of Contactin-1 precursor	R.WLLNEFPVFITM*DK.R	3	5.36	0.40	-3.05
IPI00029751	Isoform 1 of Contactin-1 precursor	R.WLLNEFPVFITM*DKR.R	2	4.32	0.33	-5.95
IPI00029751	Isoform 1 of Contactin-1 precursor	R.WLLNEFPVFITM*DKR.R	3	3.64	0.37	-2.34
IPI00029751	Isoform 1 of Contactin-1 precursor	R.WLLNEFPVFITMDK.R	2	4.80	0.39	-4.90
IPI00029751	Isoform 1 of Contactin-1 precursor	R.YGHGVSEEDKGFGPIFEEQPINTIYPEESLEGK.V	3	7.58	0.60	-4.26
IPI00029751	Isoform 1 of Contactin-1 precursor	R.YGHGVSEEDKGFGPIFEEQPINTIYPEESLEGK.V	4	5.02	0.42	-3.43
IPI00029751	Isoform 1 of Contactin-1 precursor	R.YSM*VGGNLVINNPDK.Q	2	5.07	0.49	-3.78

IPI00029751	Isoform 1 of Contactin-1 precursor	R.YSM*VGGNLVINNPDKQK.D	2	5.03	0.50	-3.22
IPI00029751	Isoform 1 of Contactin-1 precursor	R.YSM*VGGNLVINNPDKQK.D	3	3.61	0.37	-2.29
IPI00029751	Isoform 1 of Contactin-1 precursor	R.YTCTAQTIVDNSSASADLVVR.G	2	6.33	0.65	-4.56
IPI00029751	Isoform 1 of Contactin-1 precursor	R.YTCTAQTIVDNSSASADLVVR.G	3	4.88	0.50	-3.22
IPI00029751	Isoform 1 of Contactin-1 precursor	R.YVHKDETM*SPST.A	2	3.01	0.45	-4.66
IPI00029751	Isoform 1 of Contactin-1 precursor	R.YVHKDETM*SPSTAFQVK.V	2	5.22	0.52	-2.74
IPI00029751	Isoform 1 of Contactin-1 precursor	R.YVHKDETM*SPSTAFQVK.V	3	3.25	0.24	-3.50
IPI00029751	Isoform 1 of Contactin-1 precursor	S.DGGDGVVSQVK.I	2	3.84	0.39	-1.88
IPI00029751	Isoform 1 of Contactin-1 precursor	S.EEDKGFGPIFEEQPINTIYPEESLEGK.V	3	4.04	0.28	-2.88
IPI00029751	Isoform 1 of Contactin-1 precursor	V.INSAQDAPSEAPTEVGVK.V	2	5.26	0.47	-6.39
IPI00029751	Isoform 1 of Contactin-1 precursor	W.AAHDKEEAANRVQVTSQEYSAR.L	3	4.08	0.42	-4.18
IPI00029756	Proto-oncogene tyrosine-protein kinase MER precursor	K.INNEEIVSDPIYIEVQGLPHFTK.Q	3	4.37	0.38	-3.25
IPI00029756	Proto-oncogene tyrosine-protein kinase MER precursor	R.GGVGPFSDPVK.I	2	2.82	0.23	-2.13
IPI00029817	Sialidase-1 precursor	R.GTLLAFAEAR.K	2	2.91	0.12	-0.53
IPI00029817	Sialidase-1 precursor	R.NHYTESISVAK.I	2	2.66	0.31	-2.44
IPI00029817	Sialidase-1 precursor	R.QIGSVDTFRIPLITATPR.G	3	2.78	0.18	-1.39
IPI00029819	Neurogenic locus notch homolog protein 3 precursor	R.VASFYCACPM*GK.T	2	3.16	0.40	-3.13
IPI00029863	SERPINF2 protein	K.EQQDSPGNKDFLQSLK.G	2	2.83	0.30	-3.02
IPI00029863	SERPINF2 protein	K.FDPSLTQR.D	2	2.28	0.12	
IPI00029863	SERPINF2 protein	K.GFPIKEDFLEQSEQLFGAKPVSLTGK.Q	3	4.01	0.45	-3.17
IPI00029863	SERPINF2 protein	K.GFPIKEDFLEQSEQLFGAKPVSLTGKQEDDLANINQWVK.E	4	3.27	0.18	-2.19
IPI00029863	SERPINF2 protein	K.HQM*DLVATLSQLGLQELFQAPDLR.G	3	2.66	0.14	-3.78
IPI00029863	SERPINF2 protein	K.LGNQEPGGQTALK.S	1	2.44	0.13	-3.65
IPI00029863	SERPINF2 protein	K.LGNQEPGGQTALK.S	2	3.62	0.20	-3.55
IPI00029863	SERPINF2 protein	K.LGNQEPGGQTALKSPPGVCSR.D	2	5.50	0.48	
IPI00029863	SERPINF2 protein	K.LGNQEPGGQTALKSPPGVCSR.D	3	3.32	0.17	
IPI00029863	SERPINF2 protein	K.QEDDLANINQWVK.E	2	2.91	0.16	
IPI00029863	SERPINF2 protein	K.SPPGVCSRDPTPEQTHR.L	3	3.55	0.20	
IPI00029863	SERPINF2 protein	P.NQEQVSPLTLLK.L	2	3.40	0.24	-2.50
IPI00029863	SERPINF2 protein	Q.DSPGNKDFLQSLK.G	2	2.96	0.17	-0.42
IPI00029863	SERPINF2 protein	Q.LTSGPNQEQVSPLTLLK.L	2	3.38	0.30	-2.28
IPI00029863	SERPINF2 protein	R.DSFHLDEQFTVPVEM*M*QAR.T	2	3.76	0.39	-4.02
IPI00029863	SERPINF2 protein	R.DSFHLDEQFTVPVEM*M*QAR.T	3	3.95	0.28	-3.51
IPI00029863	SERPINF2 protein	R.DSFHLDEQFTVPVEMM*QAR.T	2	4.23	0.10	
IPI00029863	SERPINF2 protein	R.DSFHLDEQFTVPVEMMQAR.T	2	6.02	0.40	
IPI00029863	SERPINF2 protein	R.ELKEQQDSPGNKDFLQSLK.G	2	4.94	0.39	-3.43
IPI00029863	SERPINF2 protein	R.ELKEQQDSPGNKDFLQSLK.G	3	4.20	0.31	
IPI00029863	SERPINF2 protein	R.ELKEQQDSPGNKDFLQSLKGFPR.G	3	3.34	0.18	
IPI00029863	SERPINF2 protein	R.GDKLFGPDLK.L	1	2.54	0.27	-2.73

IPI00029863	SERPINF2 protein	R.GDKLFGPDLK.L	2	2.63	0.19	-2.55
IPI00029863	SERPINF2 protein	R.LCQDLGPGAFR.L	2	3.79	0.36	-7.07
IPI00029863	SERPINF2 protein	R.LQQVLHAGSGPCLPHLLSR.L	2	6.09	0.41	
IPI00029863	SERPINF2 protein	R.LQQVLHAGSGPCLPHLLSR.L	3	4.06	0.31	
IPI00029863	SERPINF2 protein	R.NKFDPSLTQR.D	2	2.34	0.13	-2.02
IPI00029863	SERPINF2 protein	R.QLTSGPNQEQVSPLTLLK.L	2	5.01	0.46	-2.40
IPI00029863	SERPINF2 protein	R.QLTSGPNQEQVSPLTLLK.L	3	3.22	0.19	-1.61
IPI00029863	SERPINF2 protein	R.WFLLEQPEIQVAHFPFK.N	2	4.75	0.37	-2.59
IPI00029863	SERPINF2 protein	R.WFLLEQPEIQVAHFPFK.N	3	2.67	0.18	-2.80
IPI00029928	Elastin	K.LPYGYGPGGVAGAAGK.A	2	3.69	0.26	-2.64
IPI00029928	Elastin	K.YGVGTPAAAAAK.A	2	2.43	0.13	-1.27
IPI00029928	Elastin	R.FPGVGVLPGVPTGAGVKPK.A	3	3.72	0.35	-2.31
IPI00029997	6-phosphogluconolactonase	R.ELPAAVAPAGPASLAR.W	2	3.60	0.39	-2.74
IPI00029997	6-phosphogluconolactonase	R.ILEDQEENPLPAALVQPHTGK.L	3	3.94	0.32	-2.52
IPI00029997	6-phosphogluconolactonase	R.LPIPESQVITINPELPVEEAAEDYAK.K	3	4.61	0.30	-4.31
IPI00029997	6-phosphogluconolactonase	R.TVIFVATGEGK.A	2	2.35	0.19	-2.78
IPI00029997	6-phosphogluconolactonase	R.VTLTLPVLNAAR.T	2	3.69	0.43	-3.30
IPI00030009	Isoform A of Bifunctional 3'-phosphoadenosine 5'-phosphosulfate synthetase 2	K.KDLYEPTHGGKVLSMAPGLTSVEIIPFRVAAYNK.A	3	1.96	0.23	-4.20
IPI00030037	Agouti-signaling protein precursor	K.LRDDRSLRSNSSVNLLDVPSVSIVALNKKSKQIGR.K	3	3.43	0.09	
IPI00030075	Fibroleukin precursor	K.EVQNLKEIVNSLKK.S	2	2.92	0.10	-3.41
IPI00030075	Fibroleukin precursor	K.EVQNLKEIVNSLKK.S	3	1.73	0.15	-2.36
IPI00030075	Fibroleukin precursor	K.LNLVNM*NNIENYVDSK.V	2	4.58	0.51	-3.02
IPI00030075	Fibroleukin precursor	K.LQADDNGDPGR.N	2	3.01	0.16	-2.57
IPI00030075	Fibroleukin precursor	R.ELESEVNKLSSELK.N	2	3.93	0.26	-1.90
IPI00030075	Fibroleukin precursor	R.IEEVFKEVQNLK.E	2	4.89	0.15	-2.60
IPI00030075	Fibroleukin precursor	R.IEEVFKEVQNLK.E	3	3.36	0.14	-1.44
IPI00030075	Fibroleukin precursor	R.IEEVFKEVQNLKEIVNSLKK.S	3	5.10	0.39	-3.29
IPI00030075	Fibroleukin precursor	R.IEEVFKEVQNLKEIVNSLKK.S	4	4.01	0.42	-3.17
IPI00030075	Fibroleukin precursor	R.VRELESEVNKLSSELK.N	3	2.72	0.07	-0.59
IPI00030075	Fibroleukin precursor	R.VRELESEVNKLSSELK.N	4	3.29	0.38	0.03
IPI00030111	Growth/differentiation factor 11 precursor	K.IPGM*VVDR.C	2	2.30	0.24	-3.33
IPI00030111	Growth/differentiation factor 11 precursor	K.M*SPINM*LYFNDK.Q	2	3.54	0.40	-1.82
IPI00030111	Growth/differentiation factor 11 precursor	K.YPHTHLVQQANPR.G	2	3.02	0.41	-3.79
IPI00030205	Ig kappa chain V-III region HAH precursor	G.EIVLTQSPGTLSLSPGER.A	2	6.33	0.22	
IPI00030205	Ig kappa chain V-III region HAH precursor	R.ASQSVSSSYLAWYQQKPGQAPR.L	2	5.52	0.40	
IPI00030205	Ig kappa chain V-III region HAH precursor	R.ASQSVSSSYLAWYQQKPGQAPR.L	3	3.59	0.29	
IPI00030205	Ig kappa chain V-III region HAH precursor	R.ATGIPDRFSGSGSGTDFTLTISR.L	2	4.84	0.38	
IPI00030205	Ig kappa chain V-III region HAH precursor	R.ATGIPDRFSGSGSGTDFTLTISR.L	3	3.97	0.23	
IPI00030205	Ig kappa chain V-III region HAH precursor	R.FSGSGSGTDFTLTISR.L	1	2.55	0.22	
IPI00030205	Ig kappa chain V-III region HAH precursor	R.FSGSGSGTDFTLTISR.L	2	4.49	0.53	
IPI00030205	Ig kappa chain V-III region HAH precursor	R.LLIYGASSR.A	2	3.35	0.21	

IPI00030255	Procollagen-lysine,2-oxoglutarate 5-dioxygenase 3 precursor	K.LSLNLDHK.S	2	2.87	0.21	-2.65
IPI00030255	Procollagen-lysine,2-oxoglutarate 5-dioxygenase 3 precursor	K.LVGPEEALSPGEAR.D	2	3.40	0.36	-3.82
IPI00030255	Procollagen-lysine,2-oxoglutarate 5-dioxygenase 3 precursor	K.LWSNFWGALSPDEYYAR.S	2	3.79	0.19	-3.75
IPI00030255	Procollagen-lysine,2-oxoglutarate 5-dioxygenase 3 precursor	K.QVGYEDQWLQLLR.T	2	2.98	0.19	-6.01
IPI00030255	Procollagen-lysine,2-oxoglutarate 5-dioxygenase 3 precursor	R.AVM*NFVVR.Y	2	2.29	0.15	0.99
IPI00030255	Procollagen-lysine,2-oxoglutarate 5-dioxygenase 3 precursor	R.IFQNLNGALDEVVLK.F	2	4.36	0.20	-3.25
IPI00030255	Procollagen-lysine,2-oxoglutarate 5-dioxygenase 3 precursor	R.IFQNLNGALDEVVLKFDR.N	3	2.61	0.17	-5.63
IPI00030255	Procollagen-lysine,2-oxoglutarate 5-dioxygenase 3 precursor	R.IRNVAYDTLPIVVHGNGPTK.L	3	4.78	0.37	-3.96
IPI00030255	Procollagen-lysine,2-oxoglutarate 5-dioxygenase 3 precursor	R.IRNVAYDTLPIVVHGNGPTK.L	4	2.85	0.10	-3.41
IPI00030255	Procollagen-lysine,2-oxoglutarate 5-dioxygenase 3 precursor	R.LAGGYENVPTVDIHM*K.Q	2	3.84	0.33	-2.25
IPI00030255	Procollagen-lysine,2-oxoglutarate 5-dioxygenase 3 precursor	R.LAGGYENVPTVDIHM*K.Q	3	2.56	0.10	-1.14
IPI00030255	Procollagen-lysine,2-oxoglutarate 5-dioxygenase 3 precursor	R.LLFSAESFCWPEWGLAEQYPEVGTGKR.F	3	3.25	0.20	-1.73
IPI00030255	Procollagen-lysine,2-oxoglutarate 5-dioxygenase 3 precursor	R.LYLDPGLR.E	2	2.21	0.06	-1.99
IPI00030255	Procollagen-lysine,2-oxoglutarate 5-dioxygenase 3 precursor	R.SEDYVELVQR.K	2	2.76	0.13	-3.48
IPI00030255	Procollagen-lysine,2-oxoglutarate 5-dioxygenase 3 precursor	R.TLGLGEEWR.G	2	2.82	0.18	-0.73
IPI00030255	Procollagen-lysine,2-oxoglutarate 5-dioxygenase 3 precursor	R.YDCVISSPR.K	2	3.32	0.35	-2.43
IPI00030319	Forkhead box protein F2	K.SAGGGGAGAGSGGAKKASSGLR.R	2	2.56	0.24	
IPI00030352	Isoform 2 of Growth inhibition and differentiation-related protein 88	K.EKQKESSLSQKEVFK.D	2	1.77	0.19	
IPI00030431	Isoform 1 of Anthrax toxin receptor 1 precursor	R.ASEQIYYENR.Q	2	2.53	0.20	-1.67
IPI00030431	Isoform 1 of Anthrax toxin receptor 1 precursor	R.M*SFIVFSTR.G	2	3.10	0.25	-0.36
IPI00030634	Isoform 1 of Gamma-glutamyltransferase 4 precursor	K.GLHEAHQLYGR.L	3	2.67	0.29	-6.13
IPI00030634	Isoform 1 of Gamma-glutamyltransferase 4 precursor	R.LHPDLQSNLLQVDSEFTEEEIEFLEAR.G	3	5.15	0.43	-3.54

IPI00030706	Activator of 90 kDa heat shock protein ATPase homolog 1	R.VFTTQELVQAFTHAPATLEADR.G	3	2.90	0.22	-5.36
IPI00030739	Apolipoprotein M	K.CVEEFKSLTSCLDSK.A	2	3.58	0.30	
IPI00030739	Apolipoprotein M	K.SLTSCLDSK.A	1	2.24	0.19	
IPI00030739	Apolipoprotein M	K.SLTSCLDSK.A	2	2.49	0.16	
IPI00030739	Apolipoprotein M	K.WIYHLTEGSTDLR.T	2	3.75	0.33	
IPI00030739	Apolipoprotein M	K.WIYHLTEGSTDLR.T	3	3.21	0.22	
IPI00030741	Uncharacterized protein C21orf13	K.GSEEPLQSK.E	2	1.67	0.19	
IPI00030757	Isoform LpNPI of ADAMTS-2 precursor	R.RPPTSPPLGGPQALDTGASLDSLDSLSR.A	3	5.56	0.53	0.34
IPI00030847	Transmembrane 9 superfamily member 3 precursor	K.SISHYHETLGEALQGVELEFSGLDIK.F	3	2.93	0.23	-4.06
IPI00030847	Transmembrane 9 superfamily member 3 precursor	K.SISHYHETLGEALQGVELEFSGLDIK.F	4	3.08	0.15	-3.36
IPI00030847	Transmembrane 9 superfamily member 3 precursor	K.YFSLPFCVGSK.K	2	2.42	0.20	-2.45
IPI00030847	Transmembrane 9 superfamily member 3 precursor	R.DAFVYAIK.N	1	2.09	0.10	-2.10
IPI00030871	Pantetheinase precursor	R.FGQTPVQER.L	2	2.48	0.15	-1.74
IPI00030871	Pantetheinase precursor	R.NLDILEGAITSAADQGAHIIVTPEDAIYGWNFNR.D	3	7.16	0.63	-4.47
IPI00030871	Pantetheinase precursor	R.YQYNTDVVFDSQGK.L	2	4.39	0.49	-1.08
IPI00030876	diaphanous 1 isoform 2	R.VQLNVFDEQGEEDSYDLK.G	2	5.22	0.54	-4.47
IPI00030877	15 kDa selenoprotein isoform 1 precursor	K.LGRFPQVQAFVR.S	3	3.35	0.11	-1.38
IPI00030882	Isoform Flop of Glutamate receptor 2 precursor	R.DKVNDIVDQVITIGK.H	2	4.42	0.50	-1.76
IPI00030882	Isoform Flop of Glutamate receptor 2 precursor	R.DKVNDIVDQVITIGK.H	3	4.44	0.41	-3.82
IPI00030882	Isoform Flop of Glutamate receptor 2 precursor	R.VGM*VQFSTSEFR.L	2	3.57	0.30	-3.74
IPI00030882	Isoform Flop of Glutamate receptor 2 precursor	S.NSIQIGGLFPR.G	2	3.61	0.31	-2.35
IPI00030887	Tyrosine-protein kinase receptor TYRO3 precursor	K.DGAVVQNLDQLYIPVSEQHWIGFLSLK.S	3	6.08	0.43	-7.46
IPI00030887	Tyrosine-protein kinase receptor TYRO3 precursor	K.GLAPASAPQNLHAIR.T	2	3.59	0.37	-3.95
IPI00030887	Tyrosine-protein kinase receptor TYRO3 precursor	K.GLAPASAPQNLHAIR.T	3	2.08	0.27	-2.74
IPI00030887	Tyrosine-protein kinase receptor TYRO3 precursor	R.CANALGPSPYADWVPFQTK.G	2	5.09	0.53	-4.78
IPI00030887	Tyrosine-protein kinase receptor TYRO3 precursor	R.TDSGLILEWEEVIPEAPLEGPLGPYK.L	2	3.56	0.32	-2.50
IPI00030887	Tyrosine-protein kinase receptor TYRO3 precursor	R.TDSGLILEWEEVIPEAPLEGPLGPYK.L	3	5.13	0.44	-4.28
IPI00030887	Tyrosine-protein kinase receptor TYRO3 precursor	W.LTVEGVPFFTVEPK.D	2	3.54	0.30	-0.99
IPI00030887	Tyrosine-protein kinase receptor TYRO3 precursor	W.SQPLVVSSHDR.A	2	3.56	0.34	-0.96

IPI00030919	Mitogen-activated protein kinase kinase 1-interacting protein 1	K.KLPSVEGLHAIVVSDRDGVPVIK.V	3	2.79	0.14	-3.35
IPI00031005	Protein kinase-like protein SgK196	K.IPDISSFLLGHIEGSDM*VR.F	3	2.33	0.06	-1.88
IPI00031008	Isoform 1 of Tenascin precursor	R.TAHISGLPPSTDFIVYLSGLAPSIR.T	3	2.32	0.15	-2.28
IPI00031019	Cystatin-8 precursor	R.SDCRKPLSTNEICAIQENSKLKR.K	3	3.59	0.05	
IPI00031030	Isoform 1 of Amyloid-like protein 2 precursor	A.ANAGTGFAVAEPQIAM*FCGK.L	2	5.32	0.52	-3.16
IPI00031030	Isoform 1 of Amyloid-like protein 2 precursor	A.ANAGTGFAVAEPQIAM*FCGK.L	3	4.62	0.16	-2.12
IPI00031030	Isoform 1 of Amyloid-like protein 2 precursor	A.GYIEALAANAGTGFAVAEPQIAM*FCGK.L	2	3.74	0.40	-6.23
IPI00031030	Isoform 1 of Amyloid-like protein 2 precursor	A.GYIEALAANAGTGFAVAEPQIAM*FCGK.L	3	6.12	0.45	-3.89
IPI00031030	Isoform 1 of Amyloid-like protein 2 precursor	A.LAGYIEALAANAGTGFAVAEPQIAM*FCGK.L	3	6.23	0.44	-5.90
IPI00031030	Isoform 1 of Amyloid-like protein 2 precursor	E.ALAANAGTGFAVAEPQIAM*FCGK.L	2	5.93	0.59	-3.35
IPI00031030	Isoform 1 of Amyloid-like protein 2 precursor	E.ALAANAGTGFAVAEPQIAM*FCGK.L	3	5.30	0.51	-2.92
IPI00031030	Isoform 1 of Amyloid-like protein 2 precursor	K.ALEKEAASEK.Q	2	2.73	0.21	-2.34
IPI00031030	Isoform 1 of Amyloid-like protein 2 precursor	K.ALEKEAASEKQQLVETHLAR.V	3	5.21	0.40	-2.90
IPI00031030	Isoform 1 of Amyloid-like protein 2 precursor	K.ALEKEAASEKQQLVETHLAR.V	4	3.10	0.29	-1.92
IPI00031030	Isoform 1 of Amyloid-like protein 2 precursor	K.ALEKEAASEKQQLVETHLAR.V	5	2.75	0.23	-2.01
IPI00031030	Isoform 1 of Amyloid-like protein 2 precursor	K.AVCSQEAM*TGPCR.A	2	3.91	0.40	-3.29
IPI00031030	Isoform 1 of Amyloid-like protein 2 precursor	K.CLVGEFVSDVLLVPEK.C	2	4.60	0.48	-5.30
IPI00031030	Isoform 1 of Amyloid-like protein 2 precursor	K.CLVGEFVSDVLLVPEK.C	3	3.67	0.27	-3.27
IPI00031030	Isoform 1 of Amyloid-like protein 2 precursor	K.EAASEKQQLVETHLAR.V	3	4.39	0.39	-4.28
IPI00031030	Isoform 1 of Amyloid-like protein 2 precursor	K.EACLTQGM*TLYSYGM*LLPCGVDQFHGTEYVCCPQTK.I	3	5.51	0.55	-3.23
IPI00031030	Isoform 1 of Amyloid-like protein 2 precursor	K.EACLTQGM*TLYSYGM*LLPCGVDQFHGTEYVCCPQTK.I	4	4.11	0.38	-3.55
IPI00031030	Isoform 1 of Amyloid-like protein 2 precursor	K.EEVLQYCQEM*YPELQITNVM*EANQR.V	3	5.55	0.51	-4.59
IPI00031030	Isoform 1 of Amyloid-like protein 2 precursor	K.EM*IFNAER.V	2	2.35	0.15	-3.12
IPI00031030	Isoform 1 of Amyloid-like protein 2 precursor	K.GSGVGEQDGGLIGAEEK.V	2	4.59	0.42	-4.76
IPI00031030	Isoform 1 of Amyloid-like protein 2 precursor	K.GSGVGEQDGGLIGAEEKVINSK.N	2	3.42	0.29	-4.25
IPI00031030	Isoform 1 of Amyloid-like protein 2 precursor	K.GSGVGEQDGGLIGAEEKVINSK.N	3	2.84	0.32	-2.17
IPI00031030	Isoform 1 of Amyloid-like protein 2 precursor	K.IIGSVSK.E	1	1.39	0.08	-1.40
IPI00031030	Isoform 1 of Amyloid-like protein 2 precursor	K.KGSGVGEQDGGLIGAEEK.V	2	5.35	0.34	-3.07
IPI00031030	Isoform 1 of Amyloid-like protein 2 precursor	K.LNM*HVNIQTGK.W	2	3.79	0.39	-2.81
IPI00031030	Isoform 1 of Amyloid-like protein 2 precursor	K.LNM*HVNIQTGKWEPDPTGTK.S	4	3.20	0.18	-2.52
IPI00031030	Isoform 1 of Amyloid-like protein 2 precursor	K.NKVDENM*VIDETL.D	2	3.37	0.32	-4.22
IPI00031030	Isoform 1 of Amyloid-like protein 2 precursor	K.QQLVETHLAR.V	1	1.45	0.13	-4.76
IPI00031030	Isoform 1 of Amyloid-like protein 2 precursor	K.QQLVETHLAR.V	2	1.90	0.09	-3.57
IPI00031030	Isoform 1 of Amyloid-like protein 2 precursor	K.SCFETKEEVLQYCQEM*YPELQITNVM*EANQR.V	3	5.95	0.54	-4.11
IPI00031030	Isoform 1 of Amyloid-like protein 2 precursor	K.SCFETKEEVLQYCQEM*YPELQITNVM*EANQR.V	4	4.62	0.44	-1.75
IPI00031030	Isoform 1 of Amyloid-like protein 2 precursor	K.VPYVAQEIQEEIDELLQEQR.A	2	6.91	0.34	-5.42
IPI00031030	Isoform 1 of Amyloid-like protein 2 precursor	K.VPYVAQEIQEEIDELLQEQR.A	3	5.56	0.26	-4.89
IPI00031030	Isoform 1 of Amyloid-like protein 2 precursor	K.VPYVAQEIQEEIDELLQEQR.A	4	3.41	0.07	-0.97
IPI00031030	Isoform 1 of Amyloid-like protein 2 precursor	L.DVKEM*IFNAER.V	2	3.49	0.36	-2.08
IPI00031030	Isoform 1 of Amyloid-like protein 2 precursor	Q.FTASISETPVDVR.V	2	3.76	0.36	-6.76
IPI00031030	Isoform 1 of Amyloid-like protein 2 precursor	R.ADM*DQFTASISETPVDVR.V	2	5.80	0.65	-6.64

IPI00031030	Isoform 1 of Amyloid-like protein 2 precursor	R.ESVGPLREDFSLSSSA.L	2	3.29	0.43	-6.02
IPI00031030	Isoform 1 of Amyloid-like protein 2 precursor	R.HYQHVLAVDPEK.A	2	3.65	0.36	-4.27
IPI00031030	Isoform 1 of Amyloid-like protein 2 precursor	R.M*ALENYLAALQSDPPRPHR.I	3	3.27	0.34	-3.92
IPI00031030	Isoform 1 of Amyloid-like protein 2 precursor	R.M*ALENYLAALQSDPPRPHR.I	4	4.20	0.39	-2.53
IPI00031030	Isoform 1 of Amyloid-like protein 2 precursor	R.QTLIQHFQAM*VK.A	2	1.79	0.23	-3.65
IPI00031030	Isoform 1 of Amyloid-like protein 2 precursor	R.VEAM*LNDR.R	2	2.96	0.32	-3.35
IPI00031030	Isoform 1 of Amyloid-like protein 2 precursor	R.VGGLEEER.E	1	1.96	0.11	-1.33
IPI00031030	Isoform 1 of Amyloid-like protein 2 precursor	R.VGGLEEER.E	2	2.66	0.22	-2.93
IPI00031030	Isoform 1 of Amyloid-like protein 2 precursor	R.VGGLEEERESVGP.L	2	3.54	0.28	-4.24
IPI00031030	Isoform 1 of Amyloid-like protein 2 precursor	R.VGGLEEERESVGPLR.E	2	3.65	0.31	-2.39
IPI00031030	Isoform 1 of Amyloid-like protein 2 precursor	R.VGGLEEERESVGPLR.E	3	4.11	0.33	-2.36
IPI00031030	Isoform 1 of Amyloid-like protein 2 precursor	R.VGGLEEERESVGPLRED.F	2	3.63	0.34	-1.92
IPI00031030	Isoform 1 of Amyloid-like protein 2 precursor	R.VGGLEEERESVGPLRED.F	3	4.56	0.33	-1.52
IPI00031030	Isoform 1 of Amyloid-like protein 2 precursor	R.VGGLEEERESVGPLREDFS.L	3	3.75	0.28	-2.57
IPI00031030	Isoform 1 of Amyloid-like protein 2 precursor	R.VGGLEEERESVGPLREDFSLSSSA.L	2	3.09	0.34	-2.69
IPI00031030	Isoform 1 of Amyloid-like protein 2 precursor	R.VGGLEEERESVGPLREDFSLSSSA.L	3	3.79	0.42	-5.32
IPI00031030	Isoform 1 of Amyloid-like protein 2 precursor	R.VSIDNWCR.R	2	2.61	0.26	-1.59
IPI00031030	Isoform 1 of Amyloid-like protein 2 precursor	R.VSSEESEEIPPFHPFHPFPALPENEDTQPELYHPM*KK.G	4	3.89	0.21	-4.62
IPI00031030	Isoform 1 of Amyloid-like protein 2 precursor	R.VSSEESEEIPPFHPFHPFPALPENEDTQPELYHPM*KK.G	5	3.46	0.32	-4.58
IPI00031030	Isoform 1 of Amyloid-like protein 2 precursor	R.WYFDLSK.G	1	1.97	0.06	-3.26
IPI00031030	Isoform 1 of Amyloid-like protein 2 precursor	R.WYFDLSK.G	2	2.07	0.20	-2.20
IPI00031086	Insulin-like growth factor-binding protein 1 precursor	R.IPGSPEIR.G	2	2.19	0.08	-1.81
IPI00031121	Carboxypeptidase E precursor	K.AASQPGELKDWFVGR.S	2	3.39	0.36	
IPI00031121	Carboxypeptidase E precursor	K.AASQPGELKDWFVGR.S	3	2.34	0.13	-2.84
IPI00031121	Carboxypeptidase E precursor	K.AVIHWIM*DIPFVLSANLHGGDLVANYPYDETR.S	3	5.11	0.45	-2.31
IPI00031121	Carboxypeptidase E precursor	K.AVIHWIM*DIPFVLSANLHGGDLVANYPYDETR.S	4	3.27	0.23	-2.42
IPI00031121	Carboxypeptidase E precursor	K.DGDYWR.L	2	2.23	0.10	-3.05
IPI00031121	Carboxypeptidase E precursor	K.EGGPNNHLLK.N	1	2.12	0.17	-4.07
IPI00031121	Carboxypeptidase E precursor	K.GNETIVNLIHSTR.I	3	2.71	0.26	-2.32
IPI00031121	Carboxypeptidase E precursor	K.KVAVPYSPAAGVDFELESFSER.K	2	5.05	0.59	-3.14
IPI00031121	Carboxypeptidase E precursor	K.KVAVPYSPAAGVDFELESFSER.K	3	5.11	0.52	-5.67
IPI00031121	Carboxypeptidase E precursor	K.LTASAPGYLAITK.K	1	1.81	0.24	-2.69
IPI00031121	Carboxypeptidase E precursor	K.LTASAPGYLAITK.K	2	4.13	0.37	-2.82
IPI00031121	Carboxypeptidase E precursor	K.LTASAPGYLAITK.K	3	4.43	0.35	-2.74
IPI00031121	Carboxypeptidase E precursor	K.LTASAPGYLAITKK.V	1	1.62	0.09	-4.68
IPI00031121	Carboxypeptidase E precursor	K.LTASAPGYLAITKK.V	2	3.12	0.37	-3.93
IPI00031121	Carboxypeptidase E precursor	K.LTASAPGYLAITKK.V	3	1.89	0.28	-2.15
IPI00031121	Carboxypeptidase E precursor	K.NSLISYLEQIHR.G	2	3.14	0.41	-3.13
IPI00031121	Carboxypeptidase E precursor	K.NSLISYLEQIHR.G	3	2.64	0.19	-2.63
IPI00031121	Carboxypeptidase E precursor	K.TYWEDNKNSLISYLEQIHR.G	4	2.97	0.22	-3.11
IPI00031121	Carboxypeptidase E precursor	K.VAVPYSPAAGVDFELESFSER.K	2	4.96	0.59	-8.60

IPI00031121	Carboxypeptidase E precursor	K.VAVPYSPAAGVDFELESFSER.K	3	3.35	0.35	-4.58
IPI00031121	Carboxypeptidase E precursor	K.YIGNM*HGNEAVGR.E	2	3.66	0.47	-3.91
IPI00031121	Carboxypeptidase E precursor	R.EALVSVWLQCTAISR.I	2	4.83	0.52	-5.51
IPI00031121	Carboxypeptidase E precursor	R.ELLIFLAQYLCNEYQK.G	2	5.53	0.45	-6.58
IPI00031121	Carboxypeptidase E precursor	R.ELLIFLAQYLCNEYQK.G	3	4.73	0.40	-4.25
IPI00031121	Carboxypeptidase E precursor	R.ELLVIELSDNPGVHEPGEPEFK.Y	2	4.96	0.58	-5.66
IPI00031121	Carboxypeptidase E precursor	R.ELLVIELSDNPGVHEPGEPEFK.Y	3	4.76	0.47	-8.88
IPI00031121	Carboxypeptidase E precursor	R.IHIM*PSLNPDGFEK.A	2	3.43	0.35	-3.59
IPI00031121	Carboxypeptidase E precursor	R.IHIM*PSLNPDGFEK.A	3	3.55	0.20	-3.16
IPI00031121	Carboxypeptidase E precursor	R.IVYVNEK.E	2	2.18	0.15	-2.57
IPI00031121	Carboxypeptidase E precursor	R.IVYVNEKEGGPNNHLLK.N	2	5.05	0.41	-3.82
IPI00031121	Carboxypeptidase E precursor	R.IVYVNEKEGGPNNHLLK.N	3	3.20	0.29	-2.99
IPI00031121	Carboxypeptidase E precursor	R.KNDDDSSFVDGTT.N	2	3.07	0.39	-3.14
IPI00031121	Carboxypeptidase E precursor	R.LQQEDGISFEYHR.Y	2	4.26	0.50	-3.94
IPI00031121	Carboxypeptidase E precursor	R.LQQEDGISFEYHR.Y	3	3.57	0.33	-3.05
IPI00031121	Carboxypeptidase E precursor	R.LQQEDGISFEYHRYPELR.E	2	2.74	0.20	-3.87
IPI00031121	Carboxypeptidase E precursor	R.NFPDLDR.I	1	1.60	0.05	-2.79
IPI00031121	Carboxypeptidase E precursor	R.NFPDLDRIVYVNEK.E	2	4.09	0.30	-1.66
IPI00031121	Carboxypeptidase E precursor	R.NFPDLDRIVYVNEK.E	3	1.52	0.12	-2.16
IPI00031121	Carboxypeptidase E precursor	R.NFPDLDRIVYVNEKEGGPNNHLLK.N	3	5.09	0.38	-2.22
IPI00031121	Carboxypeptidase E precursor	R.NFPDLDRIVYVNEKEGGPNNHLLK.N	4	1.49	0.19	-1.47
IPI00031121	Carboxypeptidase E precursor	R.RLQQEDGISFEYHR.Y	2	4.20	0.47	-4.25
IPI00031121	Carboxypeptidase E precursor	R.RLQQEDGISFEYHR.Y	3	4.65	0.38	-3.93
IPI00031121	Carboxypeptidase E precursor	R.RLQQEDGISFEYHRYPELR.E	4	2.94	0.32	-3.28
IPI00031121	Carboxypeptidase E precursor	R.SGSAHEYSSSPDDAIFQSLA.R	2	3.61	0.30	-1.85
IPI00031121	Carboxypeptidase E precursor	R.SGSAHEYSSSPDDAIFQSLAR.A	2	6.29	0.56	-5.15
IPI00031121	Carboxypeptidase E precursor	R.SGSAHEYSSSPDDAIFQSLAR.A	3	4.75	0.42	-3.78
IPI00031121	Carboxypeptidase E precursor	R.SNAQGIDLNR.N	2	4.02	0.30	-2.06
IPI00031121	Carboxypeptidase E precursor	W.EDNKNSLISYLEQIHR.G	2	3.48	0.32	-2.77
IPI00031121	Carboxypeptidase E precursor	W.EDNKNSLISYLEQIHR.G	3	3.99	0.31	-3.16
IPI00031121	Carboxypeptidase E precursor	Y.KLTASAPGYLAITK.K	2	4.39	0.46	-0.75
IPI00031121	Carboxypeptidase E precursor	Y.KLTASAPGYLAITK.K	3	4.15	0.34	-2.41
IPI00031131	Adipocyte plasma membrane-associated protein	R.YSLVLELSDSGAFR.R	2	3.21	0.07	
IPI00031411	Cadherin-related tumor suppressor homolog precursor	R.LPEREKPDR.E	2	3.20	0.07	
IPI00031461	Rab GDP dissociation inhibitor beta	K.DLGTESQIFISR.T	2	4.05	0.35	-3.47
IPI00031461	Rab GDP dissociation inhibitor beta	K.FDLGQDVIDFTGHALALYR.T	2	4.85	0.52	-4.24
IPI00031461	Rab GDP dissociation inhibitor beta	K.FDLGQDVIDFTGHALALYR.T	3	4.02	0.36	-3.60
IPI00031461	Rab GDP dissociation inhibitor beta	K.FLM*ANGQLVK.M	2	2.34	0.08	-0.96
IPI00031461	Rab GDP dissociation inhibitor beta	K.FVSISDLLVPK.D	2	2.57	0.19	-3.21
IPI00031461	Rab GDP dissociation inhibitor beta	K.IYKVPSTEAEALASSLM*GLFEK.R	3	2.77	0.37	-2.15
IPI00031461	Rab GDP dissociation inhibitor beta	K.LYSESLAR.Y	1	1.68	0.11	-2.03

IPI00031461	Rab GDP dissociation inhibitor beta	K.LYSESLAR.Y	2	2.53	0.20	-3.09
IPI00031461	Rab GDP dissociation inhibitor beta	K.NTNDANSCQIIIPQNQVNR.K	2	3.37	0.33	-3.67
IPI00031461	Rab GDP dissociation inhibitor beta	K.SPYLYPLYGLGELPQGFAR.L	2	6.04	0.56	-4.21
IPI00031461	Rab GDP dissociation inhibitor beta	K.SPYLYPLYGLGELPQGFAR.L	3	4.46	0.33	-4.06
IPI00031461	Rab GDP dissociation inhibitor beta	R.IKLYSESLAR.Y	1	2.49	0.27	-1.77
IPI00031461	Rab GDP dissociation inhibitor beta	R.IKLYSESLAR.Y	2	2.49	0.18	-1.31
IPI00031461	Rab GDP dissociation inhibitor beta	R.KFDLGQDVIDFTGHALALYR.T	2	4.71	0.49	-7.20
IPI00031461	Rab GDP dissociation inhibitor beta	R.KFDLGQDVIDFTGHALALYR.T	3	7.26	0.56	-6.47
IPI00031461	Rab GDP dissociation inhibitor beta	R.KFDLGQDVIDFTGHALALYR.T	4	5.53	0.42	-3.92
IPI00031461	Rab GDP dissociation inhibitor beta	R.LSAIYGGTYM*LNKPIEEIIVQNGK.V	3	4.80	0.50	-1.98
IPI00031461	Rab GDP dissociation inhibitor beta	R.TDDYLDQPCYETINR.I	2	4.40	0.45	-6.23
IPI00031461	Rab GDP dissociation inhibitor beta	R.TFEGIDPK.K	2	2.64	0.18	-3.34
IPI00031485	Mitochondrial ribosomal protein 63	R.EQERGHAAVR.R	2	1.72	0.16	
IPI00031506	Potassium/sodium hyperpolarization-activated cyclic nucleotide-gated channel 1	K.STQALHNTNLTR.E	2	3.19	0.25	-1.69
IPI00031506	Potassium/sodium hyperpolarization-activated cyclic nucleotide-gated channel 1	R.ESSSVLNTDPDAEKPR.F	2	3.79	0.33	-2.56
IPI00031506	Potassium/sodium hyperpolarization-activated cyclic nucleotide-gated channel 1	R.GVPPAPPPPAAALPR.E	2	1.74	0.15	-3.42
IPI00031510	Semaphorin-3A precursor	K.DLPDDVITFAR.S	2	2.49	0.34	-2.96
IPI00031510	Semaphorin-3A precursor	K.NPVVYGVFTTSSNIFK.G	2	3.95	0.48	-4.42
IPI00031510	Semaphorin-3A precursor	K.QQQLYIGSTAGVAQLPLHR.C	3	3.08	0.41	-3.12
IPI00031510	Semaphorin-3A precursor	K.TFGGFDSTKDLPDDVITFAR.S	3	4.11	0.34	-3.23
IPI00031510	Semaphorin-3A precursor	R.DPYCAWDGSACSR.Y	2	2.63	0.31	-4.49
IPI00031534	Alpha-N-acetylgalactosaminide alpha-2,6-sialyltransferase 1	K.GYEQDVGTR.T	2	3.02	0.33	-2.09
IPI00031534	Alpha-N-acetylgalactosaminide alpha-2,6-sialyltransferase 1	K.VVTRFPPVPQQQLLLASLPAGSLR.C	3	3.15	0.42	-2.64
IPI00031534	Alpha-N-acetylgalactosaminide alpha-2,6-sialyltransferase 1	R.GKEANQAPPEEQDKVPHTAQR.A	3	4.86	0.39	-4.10
IPI00031534	Alpha-N-acetylgalactosaminide alpha-2,6-sialyltransferase 1	R.GKEANQAPPEEQDKVPHTAQR.A	4	4.18	0.39	-3.69
IPI00031534	Alpha-N-acetylgalactosaminide alpha-2,6-sialyltransferase 1	R.RTTIYAEPVPENNALNTQTQPKAHTTGDR.G	3	3.04	0.17	
IPI00031547	Desmoglein-3 precursor	K.GQYDEEEM*TM*QQAK.R	2	2.79	0.24	-1.21
IPI00031549	Isoform 3A of Desmocollin-3 precursor	K.VILNVPSKLEADKIIGR.V	2	4.12	0.42	-1.57
IPI00031549	Isoform 3A of Desmocollin-3 precursor	K.VILNVPSKLEADKIIGR.V	3	4.00	0.41	-3.85
IPI00031549	Isoform 3A of Desmocollin-3 precursor	R.SFTIWLSDK.R	2	2.55	0.12	-2.26
IPI00031549	Isoform 3A of Desmocollin-3 precursor	R.VLNDGSVVYTAR.A	2	3.24	0.27	-3.38
IPI00031564	Uncharacterized protein C7orf24	K.AIEPNDYTGKVSEEIEDIIK.K	3	3.20	0.12	-6.03
IPI00031564	Uncharacterized protein C7orf24	K.ENGLPLEYQEK.L	2	2.94	0.21	-2.64
IPI00031564	Uncharacterized protein C7orf24	R.LQDFKLDFGNSQGK.T	2	3.56	0.34	-2.81

IPI00031564	Uncharacterized protein C7orf24	R.LQDFKLDFGNSQGK.T	3	3.19	0.37	-1.85
IPI00031564	Uncharacterized protein C7orf24	R.NPSAAFFCVAR.L	2	4.23	0.38	-2.04
IPI00031564	Uncharacterized protein C7orf24	R.SYLM*TNYESAPPSPQYK.K	2	4.29	0.49	-3.33
IPI00031627	DNA-directed RNA polymerase II subunit RPB1	R.AEIQELAM*VPRM*IVTPQSNRPVMGIVQDTLTAVRKFTK.R	4	3.30	0.07	-1.12
IPI00031696	FAST kinase domain-containing protein 3	K.ESHLDTLSR.A	2	2.28	0.06	-0.22
IPI00031708	Fumarylacetoacetase	R.ASSVVVSGTPIR.R	2	2.87	0.18	-1.91
IPI00031708	Fumarylacetoacetase	R.IGVAIGDQILDLSIIK.H	2	4.85	0.45	-4.96
IPI00031708	Fumarylacetoacetase	R.VFLQNLLSVSQAR.L	2	4.21	0.30	-3.01
IPI00031718	Isoform 1 of Ectonucleoside triphosphate diphosphohydrolase 4	K.VSDYISPLLNFAAEHVPR.A	3	2.51	0.18	-3.36
IPI00031718	Isoform 1 of Ectonucleoside triphosphate diphosphohydrolase 4	R.M*GGDYNAAK.F	2	1.76	0.08	-2.94
IPI00031765	Isoform 2 of Protocadherin gamma C4 precursor	R.DVNDHAPR.F	2	2.08	0.06	-0.87
IPI00031769	Esophageal cancer-related gene 4 protein precursor	K.AKEFLGSLK.R	1	2.45	0.14	-2.79
IPI00031769	Esophageal cancer-related gene 4 protein precursor	K.AKEFLGSLK.R	2	2.38	0.21	-1.90
IPI00031769	Esophageal cancer-related gene 4 protein precursor	K.AKEFLGSLKR.Q	2	2.35	0.16	-3.02
IPI00031769	Esophageal cancer-related gene 4 protein precursor	K.EFLGSLK.R	1	2.51	0.10	-2.94
IPI00031769	Esophageal cancer-related gene 4 protein precursor	K.TKVAVDENKAKE.F	2	3.74	0.35	-2.79
IPI00031769	Esophageal cancer-related gene 4 protein precursor	K.VAVDENKAK.E	2	2.43	0.24	-1.17
IPI00031769	Esophageal cancer-related gene 4 protein precursor	K.VAVDENKAKEFLGSLK.R	2	5.01	0.52	-3.51
IPI00031769	Esophageal cancer-related gene 4 protein precursor	K.VAVDENKAKEFLGSLK.R	3	3.10	0.40	-2.72
IPI00031769	Esophageal cancer-related gene 4 protein precursor	R.EAPVPTK.T	1	1.50	0.13	-2.78
IPI00031789	Isoform 1 of Interleukin-1 receptor accessory protein precursor	K.DSCFNSPM*K.L	2	2.74	0.20	-0.86
IPI00031789	Isoform 1 of Interleukin-1 receptor accessory protein precursor	K.FNYSTAHSAGLTLIWYWTR.Q	3	3.46	0.16	
IPI00031789	Isoform 1 of Interleukin-1 receptor accessory protein precursor	K.VAFPLEVVQK.D	1	1.77	0.15	-3.02
IPI00031789	Isoform 1 of Interleukin-1 receptor accessory protein precursor	K.VAFPLEVVQK.D	2	2.83	0.32	-1.67
IPI00031789	Isoform 1 of Interleukin-1 receptor accessory protein precursor	R.DLEEPINFR.L	2	2.35	0.23	-3.86
IPI00031821	Integral membrane protein 2B	K.IFEEEVEFISVPVPEFADSDPANIVHDFNKK.L	4	2.79	0.12	-2.58

IPI00031821	Integral membrane protein 2B	K.VTFNSALAQK.E	2	2.66	0.30	-2.20
IPI00031821	Integral membrane protein 2B	K.YIKDDVILNEPSADAPAA.L	2	4.21	0.45	-6.00
IPI00031821	Integral membrane protein 2B	R.EASNCFAIR.H	1	1.81	0.07	-1.94
IPI00031821	Integral membrane protein 2B	R.EASNCFAIR.H	2	2.37	0.14	-0.97
IPI00031821	Integral membrane protein 2B	R.NLLELLINIK.A	2	3.53	0.16	-2.02
IPI00031907	Isoform 1 of Transmembrane protein 108 precursor	R.GRNPSSTPLGQK.R	2	3.42	0.36	-3.70
IPI00031907	Isoform 1 of Transmembrane protein 108 precursor	R.NPSSTPLGQK.R	2	2.14	0.07	-0.64
IPI00032050	WW domain-binding protein 2	K.AAEAAASAYYNPGNPHN.V	2	3.98	0.56	-5.41
IPI00032050	WW domain-binding protein 2	K.KGTVYLTPYR.V	2	2.46	0.21	-3.68
IPI00032050	WW domain-binding protein 2	R.M*LQVASQASR.G	2	2.69	0.17	-1.16
IPI00032063	Similar to Candidate tumor suppressor protein	K.AIAVDPIAGK.L	2	2.60	0.13	-3.10
IPI00032063	Similar to Candidate tumor suppressor protein	K.IADEYM*IPIENLVNPR.A	2	3.71	0.32	1.48
IPI00032063	Similar to Candidate tumor suppressor protein	K.ILFQNQKEPVGLSIDYVENK.L	3	3.01	0.24	-3.26
IPI00032063	Similar to Candidate tumor suppressor protein	K.LFFTDYGNVAK.V	2	3.38	0.27	-6.65
IPI00032063	Similar to Candidate tumor suppressor protein	K.NELFLFYGK.G	2	2.54	0.25	-1.58
IPI00032063	Similar to Candidate tumor suppressor protein	K.SIHLSDETNLNSPIRPYENPR.Y	4	3.24	0.23	-3.29
IPI00032063	Similar to Candidate tumor suppressor protein	R.IESASM*SGAGR.K	2	3.39	0.49	-1.06
IPI00032063	Similar to Candidate tumor suppressor protein	R.NLYFVDHVGDR.I	2	1.90	0.09	-2.37
IPI00032063	Similar to Candidate tumor suppressor protein	R.QIFVTSK.M	2	1.38	0.09	-2.67
IPI00032063	Similar to Candidate tumor suppressor protein	R.SIAVHPEK.G	2	2.26	0.18	-1.50
IPI00032063	Similar to Candidate tumor suppressor protein	R.TGFNLGSDGR.S	2	3.14	0.31	-0.65
IPI00032063	Similar to Candidate tumor suppressor protein	R.TTLIAGAM*EHPR.A	3	2.41	0.12	-2.46
IPI00032179	Antithrombin III variant	A.EGTQVLELPFKGDDITM*VLILPKPEK.S	3	3.56	0.32	-1.94
IPI00032179	Antithrombin III variant	H.LADSKNDNDNIFLSPLSISTAFAM*TK.L	3	4.76	0.36	-3.54
IPI00032179	Antithrombin III variant	K.ADGESCSASM*M*YQEGK.F	2	4.51	0.57	-4.18
IPI00032179	Antithrombin III variant	K.ADGESCSASM*M*YQEGK.F	3	4.58	0.56	-3.00
IPI00032179	Antithrombin III variant	K.ADGESCSASM*MYQEGK.F	2	4.40	0.07	
IPI00032179	Antithrombin III variant	K.ADGESCSASMM*YQEGK.F	2	4.90	0.08	
IPI00032179	Antithrombin III variant	K.ADGESCSASMMYQEGK.F	2	4.41	0.41	
IPI00032179	Antithrombin III variant	K.AFLEVNEEGSEAAASTAVVIAGR.S	2	6.93	0.56	-7.77
IPI00032179	Antithrombin III variant	K.AFLEVNEEGSEAAASTAVVIAGR.S	3	5.58	0.51	-5.54
IPI00032179	Antithrombin III variant	K.ELFYKADGESCSASM*M*YQEGK.F	2	4.65	0.56	-2.65
IPI00032179	Antithrombin III variant	K.ELFYKADGESCSASMMYQEGK.F	2	5.35	0.41	
IPI00032179	Antithrombin III variant	K.ELFYKADGESCSASMMYQEGK.F	3	4.27	0.23	
IPI00032179	Antithrombin III variant	K.ELTPEVLQEWLDELEEM*M*LVVHM*PR.F	3	2.84	0.13	-4.94
IPI00032179	Antithrombin III variant	K.EQLQDM*GLVDLFSPEK.S	2	4.67	0.44	-4.79
IPI00032179	Antithrombin III variant	K.EQLQDM*GLVDLFSPEK.S	3	3.98	0.33	-4.18
IPI00032179	Antithrombin III variant	K.EQLQDMGLVDLFSPEK.S	2	4.48	0.47	
IPI00032179	Antithrombin III variant	K.GDDITM*VLILPKPEK.S	3	3.34	0.31	-2.67
IPI00032179	Antithrombin III variant	K.LPGIVAEGR.D	2	2.20	0.20	-1.59

IPI00032179	Antithrombin III variant	K.LPGIVAEGRDDLYVSDAFHK.A	3	4.15	0.48	-5.59
IPI00032179	Antithrombin III variant	K.LPGIVAEGRDDLYVSDAFHK.A	4	3.41	0.33	-2.82
IPI00032179	Antithrombin III variant	K.LQPLDFK.E	1	1.41	0.09	-3.27
IPI00032179	Antithrombin III variant	K.LQPLDFKENAEQSR.A	1	3.28	0.29	
IPI00032179	Antithrombin III variant	K.LQPLDFKENAEQSR.A	2	3.88	0.43	-3.29
IPI00032179	Antithrombin III variant	K.LQPLDFKENAEQSR.A	3	1.77	0.19	-3.11
IPI00032179	Antithrombin III variant	K.LVSANRLFGDK.S	2	2.72	0.34	-3.30
IPI00032179	Antithrombin III variant	K.NDNDNIFLSPLSISTAFAM*TK.L	2	6.37	0.59	-4.78
IPI00032179	Antithrombin III variant	K.NDNDNIFLSPLSISTAFAM*TK.L	3	5.71	0.56	-4.04
IPI00032179	Antithrombin III variant	K.NDNDNIFLSPLSISTAFAMTK.L	2	4.89	0.45	
IPI00032179	Antithrombin III variant	K.SKFSPENTR.K	1	2.04	0.13	-4.60
IPI00032179	Antithrombin III variant	K.SKFSPENTR.K	2	2.44	0.08	-2.60
IPI00032179	Antithrombin III variant	K.SKLPGIVAEGR.D	1	2.51	0.33	-2.84
IPI00032179	Antithrombin III variant	K.SKLPGIVAEGR.D	2	3.32	0.38	-1.40
IPI00032179	Antithrombin III variant	K.SKLPGIVAEGRDDLYVSDAFHK.A	2	5.01	0.60	-3.67
IPI00032179	Antithrombin III variant	K.SKLPGIVAEGRDDLYVSDAFHK.A	3	3.73	0.47	-3.50
IPI00032179	Antithrombin III variant	K.SKLPGIVAEGRDDLYVSDAFHK.A	4	5.21	0.54	-4.04
IPI00032179	Antithrombin III variant	K.SKLPGIVAEGRDDLYVSDAFHK.A	5	3.18	0.39	-1.32
IPI00032179	Antithrombin III variant	K.TSDQIHFFFAK.L	1	2.99	0.30	-3.49
IPI00032179	Antithrombin III variant	K.TSDQIHFFFAK.L	2	3.80	0.43	-4.59
IPI00032179	Antithrombin III variant	K.TSDQIHFFFAK.L	3	2.28	0.19	-3.19
IPI00032179	Antithrombin III variant	Q.PLDFKENAEQSR.A	2	4.00	0.45	-3.06
IPI00032179	Antithrombin III variant	R.DDLYVSDAFHK.A	2	2.87	0.27	-3.09
IPI00032179	Antithrombin III variant	R.DDLYVSDAFHK.A	3	2.51	0.18	-3.16
IPI00032179	Antithrombin III variant	R.DIPM*NPM*CIYR.S	2	2.00	0.29	-7.71
IPI00032179	Antithrombin III variant	R.DIPM*NPM*CIYR.S	3	1.82	0.28	-2.61
IPI00032179	Antithrombin III variant	R.EVPLNTIIFM*GR.V	2	3.83	0.41	-4.57
IPI00032179	Antithrombin III variant	R.EVPLNTIIFMGR.V	1	2.93	0.25	-1.66
IPI00032179	Antithrombin III variant	R.EVPLNTIIFMGR.V	2	3.07	0.49	-4.19
IPI00032179	Antithrombin III variant	R.FATTFYQHLADSK.N	2	4.15	0.37	-3.74
IPI00032179	Antithrombin III variant	R.FATTFYQHLADSKNDNDNIFLSPLSISTAF.A	3	3.56	0.30	-3.68
IPI00032179	Antithrombin III variant	R.FATTFYQHLADSKNDNDNIFLSPLSISTAFAM*TK.L	3	6.12	0.58	-5.06
IPI00032179	Antithrombin III variant	R.FATTFYQHLADSKNDNDNIFLSPLSISTAFAM*TK.L	4	4.63	0.43	-5.03
IPI00032179	Antithrombin III variant	R.FRIEDGFSLK.E	2	3.37	0.31	-7.00
IPI00032179	Antithrombin III variant	R.FRIEDGFSLK.E	3	4.32	0.40	-5.24
IPI00032179	Antithrombin III variant	R.FRIEDGFSLKEQLQDM*GLVDLFSPEK.S	3	5.58	0.35	
IPI00032179	Antithrombin III variant	R.FRIEDGFSLKEQLQDMGLVDLFSPEK.S	3	5.13	0.36	
IPI00032179	Antithrombin III variant	R.IEDGFSLK.E	2	2.05	0.19	-2.90
IPI00032179	Antithrombin III variant	R.IEDGFSLKEQLQDM*GLVDLFSPEK.S	3	4.19	0.39	-8.58
IPI00032179	Antithrombin III variant	R.ITDVIPSEAINELTVLVLVNTIYFK.G	2	4.79	0.49	-3.06
IPI00032179	Antithrombin III variant	R.ITDVIPSEAINELTVLVLVNTIYFK.G	3	5.73	0.49	-5.53
IPI00032179	Antithrombin III variant	R.RVAEGTQVLELPFKGDDITM*VLILPKPEK.S	3	6.15	0.55	-5.59

IPI00032179	Antithrombin III variant	R.RVAEGTQVLELPFKGDDITM*VLILPKPEK.S	4	5.65	0.50	-4.99
IPI00032179	Antithrombin III variant	R.RVAEGTQVLELPFKGDDITM*VLILPKPEK.S	5	2.98	0.23	-3.18
IPI00032179	Antithrombin III variant	R.SLNPNRVTFK.A	1	2.17	0.28	-4.99
IPI00032179	Antithrombin III variant	R.SLNPNRVTFK.A	2	2.50	0.28	-3.66
IPI00032179	Antithrombin III variant	R.VAEGTQVLELPFK.G	2	3.46	0.31	-3.45
IPI00032179	Antithrombin III variant	R.VAEGTQVLELPFKGDDITM*VLILPKPEK.S	2	4.06	0.42	-4.30
IPI00032179	Antithrombin III variant	R.VAEGTQVLELPFKGDDITM*VLILPKPEK.S	3	5.97	0.53	-6.53
IPI00032179	Antithrombin III variant	R.VAEGTQVLELPFKGDDITM*VLILPKPEK.S	4	3.75	0.41	-4.81
IPI00032179	Antithrombin III variant	R.VAEGTQVLELPFKGDDITM*VLILPKPEKSLAK.V	5	4.22	0.42	-3.79
IPI00032179	Antithrombin III variant	R.VAEGTQVLELPFKGDDITMVLILPKPEK.S	2	4.70	0.38	
IPI00032179	Antithrombin III variant	R.VAEGTQVLELPFKGDDITMVLILPKPEK.S	3	6.21	0.49	
IPI00032179	Antithrombin III variant	T.SDQIHFFFAK.L	2	3.26	0.24	-2.59
IPI00032179	Antithrombin III variant	V.PLNTIIFM*GR.V	2	3.65	0.36	-3.75
IPI00032187	nischarin	K.ADFNPM*PNRGTHNCRNRNSFK.L	3	2.91	0.18	-5.87
IPI00032220	Angiotensinogen precursor	A.DSQAQLLLSTVVGVFTAPGLHLK.Q	2	4.90	0.50	-3.35
IPI00032220	Angiotensinogen precursor	A.DSQAQLLLSTVVGVFTAPGLHLK.Q	3	5.64	0.49	-5.74
IPI00032220	Angiotensinogen precursor	A.LQDQLVLVAAK.L	2	3.61	0.26	-2.00
IPI00032220	Angiotensinogen precursor	A.NAGKPKDPTFIPAPIQAK.T	2	4.51	0.47	-3.12
IPI00032220	Angiotensinogen precursor	A.NAGKPKDPTFIPAPIQAK.T	3	3.92	0.27	-3.19
IPI00032220	Angiotensinogen precursor	A.VQGLLVAQGR.A	1	1.99	0.25	-3.13
IPI00032220	Angiotensinogen precursor	D.FTELDVAAEKIDR.F	2	4.14	0.45	-2.07
IPI00032220	Angiotensinogen precursor	E.KALQDQLVLVAAK.L	3	3.83	0.18	-2.61
IPI00032220	Angiotensinogen precursor	F.AVYDQSATALHFLGR.V	2	4.50	0.45	-2.40
IPI00032220	Angiotensinogen precursor	F.VQGLALYTPVVLPR.S	2	4.76	0.50	-3.82
IPI00032220	Angiotensinogen precursor	G.LALYTPVVLPR.S	2	3.17	0.32	-4.11
IPI00032220	Angiotensinogen precursor	H.KVLSALQAVQGLLVAQGR.A	3	4.01	0.30	-4.07
IPI00032220	Angiotensinogen precursor	H.YASDLDKVEGLTFQQNSLNWM*K.K	2	5.80	0.53	-3.81
IPI00032220	Angiotensinogen precursor	H.YASDLDKVEGLTFQQNSLNWM*K.K	3	5.78	0.53	-3.47
IPI00032220	Angiotensinogen precursor	K.ALQDQLVLVAAK.L	1	2.94	0.33	-3.57
IPI00032220	Angiotensinogen precursor	K.ALQDQLVLVAAK.L	2	4.36	0.47	-4.68
IPI00032220	Angiotensinogen precursor	K.ALQDQLVLVAAK.L	3	4.20	0.23	-2.32
IPI00032220	Angiotensinogen precursor	K.ALQDQLVLVAAKLDTEDK.L	2	4.53	0.52	-3.00
IPI00032220	Angiotensinogen precursor	K.ALQDQLVLVAAKLDTEDK.L	3	2.25	0.23	-3.58
IPI00032220	Angiotensinogen precursor	K.ALQDQLVLVAAKLDTEDKLR.A	2	2.89	0.48	-1.62
IPI00032220	Angiotensinogen precursor	K.ALQDQLVLVAAKLDTEDKLR.A	3	4.26	0.57	-4.96
IPI00032220	Angiotensinogen precursor	K.ALQDQLVLVAAKLDTEDKLR.A	4	3.05	0.53	-4.03
IPI00032220	Angiotensinogen precursor	K.ANAGKPKDPTFIPAPIQAK.T	2	5.04	0.50	-4.41
IPI00032220	Angiotensinogen precursor	K.ANAGKPKDPTFIPAPIQAK.T	3	5.08	0.45	-3.37
IPI00032220	Angiotensinogen precursor	K.DPTFIPAPIQAK.T	1	3.03	0.38	-4.13
IPI00032220	Angiotensinogen precursor	K.DPTFIPAPIQAK.T	2	3.68	0.36	-4.03
IPI00032220	Angiotensinogen precursor	K.DPTFIPAPIQAK.T	3	2.76	0.10	-2.91
IPI00032220	Angiotensinogen precursor	K.IDRFM*QAVTGWK.T	2	2.77	0.17	-4.58

IPI00032220	Angiotensinogen precursor	K.IDRFM*QAVTGWK.T	3	2.81	0.17	-1.65
IPI00032220	Angiotensinogen precursor	K.LDTEDKLR.A	1	2.07	0.19	-4.05
IPI00032220	Angiotensinogen precursor	K.LDTEDKLR.A	2	3.08	0.17	-1.36
IPI00032220	Angiotensinogen precursor	K.LDTEDKLRAAM*VGM*LANFLGFR.I	4	3.86	0.37	-1.63
IPI00032220	Angiotensinogen precursor	K.PKDPTFIPAPIQAK.T	2	3.88	0.36	-2.31
IPI00032220	Angiotensinogen precursor	K.QPFVQGLALYTPVVLPR.S	2	4.94	0.51	-6.74
IPI00032220	Angiotensinogen precursor	K.QPFVQGLALYTPVVLPR.S	3	5.43	0.51	-7.04
IPI00032220	Angiotensinogen precursor	K.TGCSLM*GASVDSTLAFNTYVHFQGK.M	3	3.23	0.29	-2.08
IPI00032220	Angiotensinogen precursor	K.TSPVDEKALQDQLVLVAAK.L	2	5.72	0.51	-3.50
IPI00032220	Angiotensinogen precursor	K.TSPVDEKALQDQLVLVAAK.L	3	3.29	0.43	-3.19
IPI00032220	Angiotensinogen precursor	K.TSPVDEKALQDQLVLVAAKLDTEDKLR.A	3	4.54	0.45	-3.48
IPI00032220	Angiotensinogen precursor	K.VEGLTFQQNSLNWM*K.K	2	4.50	0.38	-3.42
IPI00032220	Angiotensinogen precursor	K.VLSALQAVQGLLVAQGR.A	1	1.84	0.30	-3.10
IPI00032220	Angiotensinogen precursor	K.VLSALQAVQGLLVAQGR.A	2	5.09	0.51	-7.40
IPI00032220	Angiotensinogen precursor	K.VLSALQAVQGLLVAQGR.A	3	5.52	0.47	-8.83
IPI00032220	Angiotensinogen precursor	L.DAHKVLSALQAVQGLLVAQGR.A	2	6.80	0.68	-4.13
IPI00032220	Angiotensinogen precursor	L.DAHKVLSALQAVQGLLVAQGR.A	3	5.04	0.52	-4.15
IPI00032220	Angiotensinogen precursor	L.DFTELDVAAEKIDR.F	2	4.74	0.49	-2.95
IPI00032220	Angiotensinogen precursor	L.DVAAEKIDR.F	1	2.64	0.22	-2.22
IPI00032220	Angiotensinogen precursor	L.LLSTVVGVFTAPGLHLK.Q	2	4.30	0.50	-4.35
IPI00032220	Angiotensinogen precursor	L.SALQAVQGLLVAQGR.A	2	3.53	0.38	-2.60
IPI00032220	Angiotensinogen precursor	L.STVVGVFTAPGLHLK.Q	2	3.17	0.36	-5.39
IPI00032220	Angiotensinogen precursor	M.PQLVLQGSYDLQDLLAQAELPAILHTELNLQK.L	3	6.35	0.64	-3.32
IPI00032220	Angiotensinogen precursor	N.AGKPKDPTFIPAPIQAK.T	2	3.39	0.38	-2.76
IPI00032220	Angiotensinogen precursor	P.FVQGLALYTPVVLPR.S	2	4.93	0.48	-2.85
IPI00032220	Angiotensinogen precursor	P.FVQGLALYTPVVLPR.S	3	3.96	0.42	-3.16
IPI00032220	Angiotensinogen precursor	Q.AVQGLLVAQGR.A	1	2.37	0.22	-3.35
IPI00032220	Angiotensinogen precursor	Q.DQLVLVAAK.L	1	2.58	0.25	-2.32
IPI00032220	Angiotensinogen precursor	Q.GSYDLQDLLAQAELPAILHTELNLQK.L	3	4.64	0.41	-2.50
IPI00032220	Angiotensinogen precursor	R.AAM*VGM*LANFLGFR.I	2	4.77	0.48	-4.50
IPI00032220	Angiotensinogen precursor	R.AAM*VGM*LANFLGFR.I	3	5.12	0.37	-3.11
IPI00032220	Angiotensinogen precursor	R.AAM*VGMLANFLGFR.I	2	3.35	0.05	-4.02
IPI00032220	Angiotensinogen precursor	R.ADSQAQLLLSTVVGVFTAPGLHLK.Q	2	5.34	0.61	-4.90
IPI00032220	Angiotensinogen precursor	R.ADSQAQLLLSTVVGVFTAPGLHLK.Q	3	7.24	0.59	-5.90
IPI00032220	Angiotensinogen precursor	R.ADSQAQLLLSTVVGVFTAPGLHLK.Q	4	3.84	0.39	-4.11
IPI00032220	Angiotensinogen precursor	R.ADSQAQLLLSTVVGVFTAPGLHLKQPFVQGLALYTPVVLPR.S	3	5.14	0.56	-3.26
IPI00032220	Angiotensinogen precursor	R.ADSQAQLLLSTVVGVFTAPGLHLKQPFVQGLALYTPVVLPR.S	4	5.46	0.60	-3.61
IPI00032220	Angiotensinogen precursor	R.ADSQAQLLLSTVVGVFTAPGLHLKQPFVQGLALYTPVVLPR.S	5	4.04	0.41	-3.51
IPI00032220	Angiotensinogen precursor	R.EPTESTQQLNKPEVLEVTLNRPFLFAVYDQSATALHFLGR.V	4	6.76	0.53	-4.42
IPI00032220	Angiotensinogen precursor	R.FM*QAVTGWK.T	1	2.37	0.21	-3.12
IPI00032220	Angiotensinogen precursor	R.FM*QAVTGWK.T	2	3.01	0.29	-2.60
IPI00032220	Angiotensinogen precursor	R.LDAHKVLSALQAVQGLLVAQGR.A	2	7.59	0.66	-5.25

IPI00032220	Angiotensinogen precursor	R.LDAHKVLSALQAVQGLLVAQGR.A	3	5.05	0.58	-6.81
IPI00032220	Angiotensinogen precursor	R.LDAHKVLSALQAVQGLLVAQGR.A	4	3.88	0.37	-4.51
IPI00032220	Angiotensinogen precursor	R.LQAILGVPWK.D	2	3.65	0.36	-1.98
IPI00032220	Angiotensinogen precursor	R.SLDFTELDVAAEK.I	1	3.41	0.39	-2.08
IPI00032220	Angiotensinogen precursor	R.SLDFTELDVAAEK.I	2	5.25	0.47	-5.58
IPI00032220	Angiotensinogen precursor	R.SLDFTELDVAAEK.I	3	4.79	0.35	-1.65
IPI00032220	Angiotensinogen precursor	R.SLDFTELDVAAEKIDR.F	2	5.41	0.52	-7.26
IPI00032220	Angiotensinogen precursor	R.SLDFTELDVAAEKIDR.F	3	4.46	0.50	-4.90
IPI00032220	Angiotensinogen precursor	R.TIHLTM*PQLVLQGSYDLQDLLAQAELPAILHTELNLQK.L	3	6.56	0.65	-4.37
IPI00032220	Angiotensinogen precursor	R.TIHLTM*PQLVLQGSYDLQDLLAQAELPAILHTELNLQK.L	4	9.25	0.63	-5.57
IPI00032220	Angiotensinogen precursor	R.TIHLTM*PQLVLQGSYDLQDLLAQAELPAILHTELNLQK.L	5	5.70	0.40	-3.76
IPI00032220	Angiotensinogen precursor	R.TIHLTM*PQLVLQGSYDLQDLLAQAELPAILHTELNLQK.L	6	4.51	0.31	-2.42
IPI00032220	Angiotensinogen precursor	R.VANPLSTA.-	1	1.79	0.38	-4.14
IPI00032220	Angiotensinogen precursor	R.VGEVLNSIFFELEADER.E	2	5.99	0.55	-6.94
IPI00032220	Angiotensinogen precursor	R.VGEVLNSIFFELEADER.E	3	5.06	0.40	-4.26
IPI00032220	Angiotensinogen precursor	R.VGEVLNSIFFELEADEREPTESTQQLN.K	3	4.06	0.25	-5.05
IPI00032220	Angiotensinogen precursor	R.VGEVLNSIFFELEADEREPTESTQQLNKPEVLEVTLNR.P	4	4.49	0.22	-4.40
IPI00032220	Angiotensinogen precursor	S.LDFTELDVAAEKIDR.F	2	3.50	0.45	-3.69
IPI00032220	Angiotensinogen precursor	S.PVDEKALQDQLVLVAAK.L	3	4.85	0.51	-2.89
IPI00032220	Angiotensinogen precursor	V.GVFTAPGLHLK.Q	1	1.87	0.22	0.01
IPI00032220	Angiotensinogen precursor	V.LSALQAVQGLLVAQGR.A	2	3.74	0.47	-5.54
IPI00032220	Angiotensinogen precursor	V.LSALQAVQGLLVAQGR.A	3	3.93	0.30	-3.45
IPI00032220	Angiotensinogen precursor	V.VGVFTAPGLHLK.Q	2	2.96	0.34	-2.73
IPI00032227	Isoform 1 of Rabphilin-3A	K.HWYECLKNKDK.K	2	3.39	0.19	
IPI00032227	Isoform 1 of Rabphilin-3A	K.SNDYIGGCQLGISAKGERLKHWYECLK.N	3	3.49	0.19	
IPI00032258	Complement C4-A precursor	A.PFLLQALVR.E	2	3.53	0.25	-4.36
IPI00032258	Complement C4-A precursor	A.PKVVEEQESR.V	2	3.04	0.29	-1.03
IPI00032258	Complement C4-A precursor	D.DPDAPLQPVTPLQLFEGR.R	2	3.34	0.40	-3.65
IPI00032258	Complement C4-A precursor	D.DPDAPLQPVTPLQLFEGR.R	3	4.88	0.46	-2.11
IPI00032258	Complement C4-A precursor	D.HAVDLIQK.G	2	2.94	0.27	-0.14
IPI00032258	Complement C4-A precursor	D.PLDTLGSEGALSPGGVASLLR.L	2	5.16	0.52	-3.43
IPI00032258	Complement C4-A precursor	D.PLDTLGSEGALSPGGVASLLR.L	3	5.06	0.42	-2.98
IPI00032258	Complement C4-A precursor	E.APKVVEEQESR.V	2	3.24	0.37	-3.22
IPI00032258	Complement C4-A precursor	E.APKVVEEQESR.V	3	3.64	0.30	-2.42
IPI00032258	Complement C4-A precursor	F.LSCCQFAESLR.K	2	3.54	0.35	-2.59
IPI00032258	Complement C4-A precursor	K.ADGSYAAWLSR.D	2	3.01	0.24	
IPI00032258	Complement C4-A precursor	K.AEFQDALEK.L	1	2.52	0.13	-4.21
IPI00032258	Complement C4-A precursor	K.AEFQDALEK.L	2	3.23	0.16	-3.14
IPI00032258	Complement C4-A precursor	K.AEFQDALEKLNM*GITDLQGLR.L	2	4.80	0.57	-3.22
IPI00032258	Complement C4-A precursor	K.AEFQDALEKLNM*GITDLQGLR.L	3	6.42	0.49	-5.12
IPI00032258	Complement C4-A precursor	K.AEFQDALEKLNM*GITDLQGLR.L	4	4.59	0.32	-2.23
IPI00032258	Complement C4-A precursor	K.AEFQDALEKLNMGITDLQGLR.L	2	3.83	0.32	

IPI00032258	Complement C4-A precursor	K.AEFQDALEKLNMGITDLQGLR.L	3	5.14	0.35	-4.61
IPI00032258	Complement C4-A precursor	K.AEM*ADQASAWLTR.Q	2	3.96	0.36	-3.82
IPI00032258	Complement C4-A precursor	K.ANSFLGEK.A	1	1.80	0.12	-2.75
IPI00032258	Complement C4-A precursor	K.ANSFLGEK.A	2	2.61	0.23	-3.14
IPI00032258	Complement C4-A precursor	K.DDPDAPLQPVTPLQLFEGR.R	2	4.90	0.57	-4.98
IPI00032258	Complement C4-A precursor	K.DDPDAPLQPVTPLQLFEGR.R	3	3.93	0.32	-3.18
IPI00032258	Complement C4-A precursor	K.DDPDAPLQPVTPLQLFEGRR.N	3	2.03	0.14	-4.63
IPI00032258	Complement C4-A precursor	K.DHAVDLIQK.G	1	3.03	0.30	-2.14
IPI00032258	Complement C4-A precursor	K.DHAVDLIQK.G	2	2.76	0.32	-2.89
IPI00032258	Complement C4-A precursor	K.DVKAAANQM*R.N	2	2.64	0.13	-1.54
IPI00032258	Complement C4-A precursor	K.EGAIHREELVYELNPLDHR.G	2	4.35	0.44	-4.58
IPI00032258	Complement C4-A precursor	K.EGAIHREELVYELNPLDHR.G	3	5.45	0.39	-4.68
IPI00032258	Complement C4-A precursor	K.EGAIHREELVYELNPLDHR.G	4	2.47	0.18	-2.23
IPI00032258	Complement C4-A precursor	K.EGAIHREELVYELNPLDHR.G	5	2.71	0.21	-3.27
IPI00032258	Complement C4-A precursor	K.EGAIHREELVYELNPLDHRG.R	3	4.71	0.30	-2.28
IPI00032258	Complement C4-A precursor	K.EVYM*PSSIFQDDFVIPDISEPGTWK.I	2	4.19	0.53	-3.32
IPI00032258	Complement C4-A precursor	K.EVYM*PSSIFQDDFVIPDISEPGTWK.I	3	2.90	0.17	-4.23
IPI00032258	Complement C4-A precursor	K.FACYYPR.V	1	1.88	0.26	-1.69
IPI00032258	Complement C4-A precursor	K.FACYYPR.V	2	2.69	0.29	-1.19
IPI00032258	Complement C4-A precursor	K.GLCVATPVQLR.V	1	2.09	0.28	-4.05
IPI00032258	Complement C4-A precursor	K.GLCVATPVQLR.V	2	3.26	0.21	-2.73
IPI00032258	Complement C4-A precursor	K.GSVFLRNPSR.N	2	2.67	0.15	-3.14
IPI00032258	Complement C4-A precursor	K.INVKVGGNSK.G	2	2.52	0.17	-2.51
IPI00032258	Complement C4-A precursor	K.ITPGKPYILTVPGHLDEM*QLDIQAR.Y	2	3.47	0.45	-1.39
IPI00032258	Complement C4-A precursor	K.ITPGKPYILTVPGHLDEM*QLDIQAR.Y	3	4.87	0.51	-4.46
IPI00032258	Complement C4-A precursor	K.ITPGKPYILTVPGHLDEM*QLDIQAR.Y	4	3.95	0.28	-4.16
IPI00032258	Complement C4-A precursor	K.ITPGKPYILTVPGHLDEMQLDIQAR.Y	3	4.61	0.41	
IPI00032258	Complement C4-A precursor	K.ITQVLHFTK.D	1	2.24	0.30	-4.38
IPI00032258	Complement C4-A precursor	K.ITQVLHFTK.D	2	3.30	0.38	-3.07
IPI00032258	Complement C4-A precursor	K.ITQVLHFTKDVK.A	2	4.04	0.36	-4.01
IPI00032258	Complement C4-A precursor	K.KEVYM*PSSIFQDDFVIPDISEPGTWK.I	3	4.00	0.29	-1.31
IPI00032258	Complement C4-A precursor	K.KYVLPNFEVK.I	1	3.22	0.21	-1.97
IPI00032258	Complement C4-A precursor	K.KYVLPNFEVK.I	2	3.37	0.31	-2.23
IPI00032258	Complement C4-A precursor	K.KYVLPNFEVK.I	3	3.87	0.27	-2.87
IPI00032258	Complement C4-A precursor	K.LELSVDGAK.Q	1	2.07	0.11	-3.30
IPI00032258	Complement C4-A precursor	K.LELSVDGAK.Q	2	2.62	0.05	-1.34
IPI00032258	Complement C4-A precursor	K.LGQYASPTAK.R	1	2.38	0.30	-3.94
IPI00032258	Complement C4-A precursor	K.LGQYASPTAK.R	2	3.64	0.30	-2.10
IPI00032258	Complement C4-A precursor	K.LGQYASPTAKR.C	2	3.20	0.31	-1.92
IPI00032258	Complement C4-A precursor	K.LHLETDSLALVALGALDTALYAAGSK.S	2	7.37	0.59	-4.74
IPI00032258	Complement C4-A precursor	K.LHLETDSLALVALGALDTALYAAGSK.S	3	5.93	0.57	-5.66
IPI00032258	Complement C4-A precursor	K.LHLETDSLALVALGALDTALYAAGSK.S	4	5.53	0.57	-3.35

IPI00032258	Complement C4-A precursor	K.LNM*GITDLQGLR.L	2	3.92	0.39	-2.16
IPI00032258	Complement C4-A precursor	K.LQETSNWLLSQQQADGSFQDPCPVLDR.S	3	4.75	0.45	-5.13
IPI00032258	Complement C4-A precursor	K.LTSLSDRYVSHFETEGPHVLLYFDSVPTSR.E	3	7.13	0.54	-4.58
IPI00032258	Complement C4-A precursor	K.LTSLSDRYVSHFETEGPHVLLYFDSVPTSR.E	4	4.45	0.37	-4.48
IPI00032258	Complement C4-A precursor	K.LVNGQSHISLSK.A	1	2.64	0.24	-2.75
IPI00032258	Complement C4-A precursor	K.LVNGQSHISLSK.A	2	3.80	0.42	-2.64
IPI00032258	Complement C4-A precursor	K.LVNGQSHISLSKAEFQDALEK.L	3	3.24	0.40	-3.17
IPI00032258	Complement C4-A precursor	K.M*RPSTDTITVM*VENSHGLR.V	2	1.97	0.31	-5.48
IPI00032258	Complement C4-A precursor	K.M*RPSTDTITVM*VENSHGLR.V	3	5.50	0.52	-3.54
IPI00032258	Complement C4-A precursor	K.M*RPSTDTITVM*VENSHGLR.V	4	4.69	0.46	-3.02
IPI00032258	Complement C4-A precursor	K.PVQGVAYVR.F	1	2.57	0.14	-3.21
IPI00032258	Complement C4-A precursor	K.QRVEASISK.A	2	2.72	0.08	-2.72
IPI00032258	Complement C4-A precursor	K.RCCQDGVTR.L	2	2.98	0.12	
IPI00032258	Complement C4-A precursor	K.RHLVPGAPFLLQALVR.E	3	3.46	0.30	-4.10
IPI00032258	Complement C4-A precursor	K.SCGLHQLLR.G	1	2.27	0.15	
IPI00032258	Complement C4-A precursor	K.SCGLHQLLR.G	2	2.91	0.22	
IPI00032258	Complement C4-A precursor	K.SHALQLNNR.Q	1	2.62	0.26	-4.91
IPI00032258	Complement C4-A precursor	K.SHALQLNNR.Q	2	2.98	0.28	-1.91
IPI00032258	Complement C4-A precursor	K.VDFTLSSER.D	1	2.05	0.17	-3.10
IPI00032258	Complement C4-A precursor	K.VDFTLSSER.D	2	3.34	0.38	-4.33
IPI00032258	Complement C4-A precursor	K.VDFTLSSERDFALLSLQVPLK.D	3	3.10	0.18	-3.13
IPI00032258	Complement C4-A precursor	K.VDFTLSSERDFALLSLQVPLKDAK.S	2	3.81	0.45	-2.80
IPI00032258	Complement C4-A precursor	K.VDFTLSSERDFALLSLQVPLKDAK.S	3	5.14	0.47	-7.67
IPI00032258	Complement C4-A precursor	K.VDFTLSSERDFALLSLQVPLKDAK.S	4	3.73	0.47	-4.64
IPI00032258	Complement C4-A precursor	K.VFEAM*NSYDLGCGPGGGDSALQVFQAAGLAFSDGDQWTLSR.K	3	6.49	0.53	
IPI00032258	Complement C4-A precursor	K.VFEAMNSYDLGCGPGGGDSALQVFQAAGLAFSDGDQWTLSR.K	3	5.40	0.36	
IPI00032258	Complement C4-A precursor	K.VGLSGM*AIADVTLLSGFHALR.A	2	3.01	0.31	0.29
IPI00032258	Complement C4-A precursor	K.VGLSGM*AIADVTLLSGFHALR.A	3	6.65	0.56	-4.52
IPI00032258	Complement C4-A precursor	K.VGLSGM*AIADVTLLSGFHALRADLEK.L	4	4.38	0.43	-2.71
IPI00032258	Complement C4-A precursor	K.VGLSGMAIADVTLLSGFHALR.A	3	4.19	0.36	
IPI00032258	Complement C4-A precursor	K.VLQIEKEGAIHR.E	2	4.22	0.27	-4.02
IPI00032258	Complement C4-A precursor	K.VLQIEKEGAIHR.E	3	3.75	0.31	-3.15
IPI00032258	Complement C4-A precursor	K.VLQIEKEGAIHREELVYELNPLDHR.G	3	6.23	0.59	-4.44
IPI00032258	Complement C4-A precursor	K.VLQIEKEGAIHREELVYELNPLDHR.G	4	4.48	0.36	-4.52
IPI00032258	Complement C4-A precursor	K.VLQIEKEGAIHREELVYELNPLDHR.G	5	4.06	0.36	-4.16
IPI00032258	Complement C4-A precursor	K.VLQIEKEGAIHREELVYELNPLDHR.G	6	3.33	0.27	-2.77
IPI00032258	Complement C4-A precursor	K.VLSLAQEQVGGSPEK.L	1	3.34	0.49	-1.95
IPI00032258	Complement C4-A precursor	K.VLSLAQEQVGGSPEK.L	2	5.17	0.51	-4.87
IPI00032258	Complement C4-A precursor	K.VLSLAQEQVGGSPEK.L	3	3.53	0.32	-1.80
IPI00032258	Complement C4-A precursor	K.VLSLAQEQVGGSPEKLQETSNWLLSQQQADGSFQDPCPVLDR.S	3	6.96	0.57	-4.05
IPI00032258	Complement C4-A precursor	K.VLSLAQEQVGGSPEKLQETSNWLLSQQQADGSFQDPCPVLDR.S	4	5.16	0.43	-4.93
IPI00032258	Complement C4-A precursor	K.VLSLAQEQVGGSPEKLQETSNWLLSQQQADGSFQDPCPVLDR.S	5	6.08	0.46	-3.10

IPI00032258	Complement C4-A precursor	K.YVLPNFEVK.I	1	2.71	0.17	-3.38
IPI00032258	Complement C4-A precursor	K.YVLPNFEVK.I	2	2.55	0.19	-1.83
IPI00032258	Complement C4-A precursor	L.GQYASPTAK.R	1	1.90	0.19	-2.03
IPI00032258	Complement C4-A precursor	L.SPGGVASLLR.L	2	4.09	0.30	-2.65
IPI00032258	Complement C4-A precursor	L.VNGQSHISLSK.A	2	3.56	0.35	-0.30
IPI00032258	Complement C4-A precursor	R.AACAQLNDFLQEYGTQGCQV.-	2	3.35	0.53	-2.34
IPI00032258	Complement C4-A precursor	R.ADLEKLTSLSDR.Y	2	3.07	0.34	-2.75
IPI00032258	Complement C4-A precursor	R.ADLEKLTSLSDR.Y	3	2.72	0.32	-0.64
IPI00032258	Complement C4-A precursor	R.ADLEKLTSLSDRYVSHFETEGPHVLLYFDSVPTSR.E	4	2.84	0.13	-3.78
IPI00032258	Complement C4-A precursor	R.ALEILQEEDLIDEDDIPVR.S	2	5.55	0.43	-5.78
IPI00032258	Complement C4-A precursor	R.ALEILQEEDLIDEDDIPVR.S	3	4.88	0.38	-5.46
IPI00032258	Complement C4-A precursor	R.ALEILQEEDLIDEDDIPVRSFFPENWLWR.V	3	4.69	0.52	-3.20
IPI00032258	Complement C4-A precursor	R.AVGSGATFSHYYYM*ILSR.G	2	4.81	0.53	-5.30
IPI00032258	Complement C4-A precursor	R.AVGSGATFSHYYYM*ILSR.G	3	4.04	0.48	-4.94
IPI00032258	Complement C4-A precursor	R.AVGSGATFSHYYYMILSR.G	3	3.29	0.25	
IPI00032258	Complement C4-A precursor	R.CSVFYGAPSK.S	1	2.55	0.31	-3.16
IPI00032258	Complement C4-A precursor	R.CSVFYGAPSK.S	2	3.72	0.39	-2.36
IPI00032258	Complement C4-A precursor	R.DFALLSLQVPLK.D	2	4.18	0.45	-4.86
IPI00032258	Complement C4-A precursor	R.DFALLSLQVPLKDAK.S	2	4.57	0.46	-4.35
IPI00032258	Complement C4-A precursor	R.DFALLSLQVPLKDAK.S	3	3.48	0.43	-3.14
IPI00032258	Complement C4-A precursor	R.DKGQAGLQR.A	2	2.59	0.10	-2.19
IPI00032258	Complement C4-A precursor	R.DSSTWLTAFVLK.V	1	2.40	0.34	-3.95
IPI00032258	Complement C4-A precursor	R.DSSTWLTAFVLK.V	2	4.09	0.39	-6.78
IPI00032258	Complement C4-A precursor	R.EAPKVVEEQESR.V	1	2.24	0.07	
IPI00032258	Complement C4-A precursor	R.EAPKVVEEQESR.V	2	2.88	0.34	-3.16
IPI00032258	Complement C4-A precursor	R.EAPKVVEEQESR.V	3	2.63	0.22	-2.45
IPI00032258	Complement C4-A precursor	R.ECVGFEAVQEVPVGLVQPASATLYDYYNPER.R	2	4.27	0.49	
IPI00032258	Complement C4-A precursor	R.ECVGFEAVQEVPVGLVQPASATLYDYYNPER.R	3	4.22	0.31	-3.37
IPI00032258	Complement C4-A precursor	R.ECVGFEAVQEVPVGLVQPASATLYDYYNPERR.C	3	6.63	0.58	-5.53
IPI00032258	Complement C4-A precursor	R.ECVGFEAVQEVPVGLVQPASATLYDYYNPERR.C	4	4.03	0.46	-3.98
IPI00032258	Complement C4-A precursor	R.ECVGFEAVQEVPVGLVQPASATLYDYYNPERR.C	5	2.74	0.24	-2.59
IPI00032258	Complement C4-A precursor	R.EELVYELNPLDHR.G	2	3.88	0.48	-2.39
IPI00032258	Complement C4-A precursor	R.EFHLHLR.L	1	2.09	0.11	-4.66
IPI00032258	Complement C4-A precursor	R.EM*SGSPASGIPVK.V	1	1.99	0.24	-3.90
IPI00032258	Complement C4-A precursor	R.EM*SGSPASGIPVK.V	2	2.30	0.12	-3.94
IPI00032258	Complement C4-A precursor	R.EPFLSCCQFAESLR.K	2	4.10	0.31	-5.14
IPI00032258	Complement C4-A precursor	R.EPFLSCCQFAESLR.K	3	4.17	0.26	
IPI00032258	Complement C4-A precursor	R.EPFLSCCQFAESLRK.K	2	2.78	0.21	-2.63
IPI00032258	Complement C4-A precursor	R.FGLLDEDGK.K	2	3.33	0.14	-3.04
IPI00032258	Complement C4-A precursor	R.FGLLDEDGKK.T	1	3.16	0.22	-2.26
IPI00032258	Complement C4-A precursor	R.FGLLDEDGKK.T	2	3.16	0.21	-1.47
IPI00032258	Complement C4-A precursor	R.FGLLDEDGKKTFFR.G	2	4.33	0.50	-4.76

IPI00032258	Complement C4-A precursor	R.FGLLDEDGKKTFFR.G	3	4.11	0.31	-4.83
IPI00032258	Complement C4-A precursor	R.FGLLDEDGKKTFFR.G	4	3.11	0.18	-4.04
IPI00032258	Complement C4-A precursor	R.FGLLDEDGKKTFFRGLESQTK.L	3	2.75	0.25	-0.71
IPI00032258	Complement C4-A precursor	R.GCGEQTM*IYLAPTLAASR.Y	2	5.38	0.50	-3.89
IPI00032258	Complement C4-A precursor	R.GCGEQTM*IYLAPTLAASR.Y	3	3.62	0.34	-3.87
IPI00032258	Complement C4-A precursor	R.GCGEQTMIYLAPTLAASR.Y	2	4.91	0.39	
IPI00032258	Complement C4-A precursor	R.GHLFLQTDQPIYNPGQR.V	2	6.47	0.60	-4.40
IPI00032258	Complement C4-A precursor	R.GHLFLQTDQPIYNPGQR.V	3	5.34	0.44	-3.84
IPI00032258	Complement C4-A precursor	R.GLEEELQFSLGSK.I	1	3.08	0.37	-4.48
IPI00032258	Complement C4-A precursor	R.GLEEELQFSLGSK.I	2	4.92	0.52	-7.24
IPI00032258	Complement C4-A precursor	R.GLQDEDGYR.M	1	1.85	0.22	-4.44
IPI00032258	Complement C4-A precursor	R.GLQDEDGYR.M	2	3.43	0.37	-2.87
IPI00032258	Complement C4-A precursor	R.GLQDEDGYRM*K.F	2	2.40	0.12	-4.36
IPI00032258	Complement C4-A precursor	R.GPEVQLVAHSPWLK.D	1	2.62	0.14	
IPI00032258	Complement C4-A precursor	R.GPEVQLVAHSPWLK.D	2	4.57	0.50	-3.48
IPI00032258	Complement C4-A precursor	R.GPEVQLVAHSPWLK.D	3	3.80	0.40	-2.36
IPI00032258	Complement C4-A precursor	R.GPEVQLVAHSPWLKDSLSR.T	3	3.77	0.33	
IPI00032258	Complement C4-A precursor	R.GPEVQLVAHSPWLKDSLSR.T	4	2.32	0.16	-4.40
IPI00032258	Complement C4-A precursor	R.GQIVFM*NR.E	2	2.63	0.24	-1.45
IPI00032258	Complement C4-A precursor	R.GQIVFM*NREPK.R	2	2.49	0.21	
IPI00032258	Complement C4-A precursor	R.GRTLEIPGNSDPNM*IPDGDFNSYVR.V	2	2.68	0.41	-2.81
IPI00032258	Complement C4-A precursor	R.GRTLEIPGNSDPNM*IPDGDFNSYVR.V	3	5.17	0.43	-2.92
IPI00032258	Complement C4-A precursor	R.GSFEFPVGDAVSK.V	1	2.92	0.49	-2.86
IPI00032258	Complement C4-A precursor	R.GSFEFPVGDAVSK.V	2	3.74	0.39	-2.64
IPI00032258	Complement C4-A precursor	R.GSFEFPVGDAVSKVLQIEK.E	2	4.00	0.42	-3.93
IPI00032258	Complement C4-A precursor	R.GSFEFPVGDAVSKVLQIEK.E	3	2.60	0.31	-3.56
IPI00032258	Complement C4-A precursor	R.GSFEFPVGDAVSKVLQIEKEGAIHR.E	3	3.97	0.34	-4.01
IPI00032258	Complement C4-A precursor	R.GSFEFPVGDAVSKVLQIEKEGAIHR.E	4	2.91	0.22	-5.74
IPI00032258	Complement C4-A precursor	R.GSFEFPVGDAVSKVLQIEKEGAIHR.E	5	2.91	0.26	-3.00
IPI00032258	Complement C4-A precursor	R.HLVPGAPFLLQALVR.E	2	3.09	0.39	-5.37
IPI00032258	Complement C4-A precursor	R.HLVPGAPFLLQALVR.E	3	4.01	0.30	-4.25
IPI00032258	Complement C4-A precursor	R.KADGSYAAWLSR.D	2	3.86	0.37	-4.03
IPI00032258	Complement C4-A precursor	R.KADGSYAAWLSR.D	3	3.49	0.11	-3.51
IPI00032258	Complement C4-A precursor	R.KKEVYM*PSSIFQDDFVIPDISEPGTWK.I	3	4.52	0.39	-5.33
IPI00032258	Complement C4-A precursor	R.LLATLCSAEVCQCAEGK.C	2	5.88	0.56	-6.70
IPI00032258	Complement C4-A precursor	R.LLATLCSAEVCQCAEGK.C	3	6.16	0.43	-6.82
IPI00032258	Complement C4-A precursor	R.LLATLCSAEVCQCAEGKCPR.Q	3	2.96	0.16	
IPI00032258	Complement C4-A precursor	R.LLLFSPSVVHLGVPL.S	2	2.99	0.29	-3.96
IPI00032258	Complement C4-A precursor	R.LLLFSPSVVHLGVPLSVGVQLQDVPR.G	2	4.47	0.64	-1.36
IPI00032258	Complement C4-A precursor	R.LLLFSPSVVHLGVPLSVGVQLQDVPR.G	3	6.08	0.58	-4.45
IPI00032258	Complement C4-A precursor	R.LLLFSPSVVHLGVPLSVGVQLQDVPR.G	4	3.95	0.40	-2.47
IPI00032258	Complement C4-A precursor	R.LLLFSPSVVHLGVPLSVGVQLQDVPRGQVVK.G	3	3.59	0.27	

IPI00032258	Complement C4-A precursor	R.LRLEPGKEYLIM*GLDGATYDLEGHPQYLLDSNSWIEEM*PSER.L	4	4.21	0.35	-3.19
IPI00032258	Complement C4-A precursor	R.LTVAAPPSGGPGFLSIER.P	2	4.29	0.44	-4.43
IPI00032258	Complement C4-A precursor	R.LTVAAPPSGGPGFLSIERPDSRPPR.V	2	2.42	0.25	-3.99
IPI00032258	Complement C4-A precursor	R.LTVAAPPSGGPGFLSIERPDSRPPR.V	3	2.34	0.17	-2.13
IPI00032258	Complement C4-A precursor	R.LTVAAPPSGGPGFLSIERPDSRPPR.V	4	2.19	0.18	-3.67
IPI00032258	Complement C4-A precursor	R.M*KFACYYPR.V	2	2.54	0.10	
IPI00032258	Complement C4-A precursor	R.NGESVKLHLETDSLALVALGALDTALYAAGSK.S	3	3.44	0.36	-6.33
IPI00032258	Complement C4-A precursor	R.NGESVKLHLETDSLALVALGALDTALYAAGSK.S	4	4.01	0.25	-3.24
IPI00032258	Complement C4-A precursor	R.NGFKSHALQLNNR.Q	3	3.18	0.31	-2.45
IPI00032258	Complement C4-A precursor	R.PVAFSVVPTAAAAVSLK.V	2	4.50	0.50	-5.05
IPI00032258	Complement C4-A precursor	R.PVAFSVVPTAAAAVSLK.V	3	4.18	0.38	-3.45
IPI00032258	Complement C4-A precursor	R.QGSFQGGFR.S	1	1.45	0.05	-3.46
IPI00032258	Complement C4-A precursor	R.QGSFQGGFR.S	2	2.28	0.34	-2.13
IPI00032258	Complement C4-A precursor	R.RCSVFYGAPSK.S	2	3.45	0.20	
IPI00032258	Complement C4-A precursor	R.RGHLFLQTDQPIYNPGQR.V	2	4.58	0.46	-4.28
IPI00032258	Complement C4-A precursor	R.RGHLFLQTDQPIYNPGQR.V	3	4.79	0.33	-4.04
IPI00032258	Complement C4-A precursor	R.RGHLFLQTDQPIYNPGQR.V	4	2.32	0.16	-3.53
IPI00032258	Complement C4-A precursor	R.SFFPENWLWR.V	1	2.05	0.24	-2.53
IPI00032258	Complement C4-A precursor	R.SFFPENWLWR.V	2	3.48	0.34	-4.33
IPI00032258	Complement C4-A precursor	R.SM*QGGLVGNDETVALTAFVTIALHHGLAVFQDEGAEPLK.Q	3	6.29	0.60	-2.32
IPI00032258	Complement C4-A precursor	R.SM*QGGLVGNDETVALTAFVTIALHHGLAVFQDEGAEPLK.Q	4	6.42	0.50	-8.14
IPI00032258	Complement C4-A precursor	R.SM*QGGLVGNDETVALTAFVTIALHHGLAVFQDEGAEPLKQR.V	3	4.14	0.45	-5.18
IPI00032258	Complement C4-A precursor	R.SM*QGGLVGNDETVALTAFVTIALHHGLAVFQDEGAEPLKQR.V	4	5.33	0.48	-4.07
IPI00032258	Complement C4-A precursor	R.SM*QGGLVGNDETVALTAFVTIALHHGLAVFQDEGAEPLKQR.V	5	4.32	0.39	-3.55
IPI00032258	Complement C4-A precursor	R.STQDTVIALDALSAYWIASHTTEER.G	2	5.04	0.55	-3.85
IPI00032258	Complement C4-A precursor	R.STQDTVIALDALSAYWIASHTTEER.G	3	5.04	0.49	-4.72
IPI00032258	Complement C4-A precursor	R.STQDTVIALDALSAYWIASHTTEERG.L	3	3.66	0.35	-3.92
IPI00032258	Complement C4-A precursor	R.TLEIPGNSDPNM*IPDGDFNSYVR.V	2	4.01	0.46	-5.73
IPI00032258	Complement C4-A precursor	R.TLEIPGNSDPNM*IPDGDFNSYVR.V	3	3.90	0.23	-6.50
IPI00032258	Complement C4-A precursor	R.TTNIQGINLLFSSR.R	1	2.76	0.37	-2.92
IPI00032258	Complement C4-A precursor	R.TTNIQGINLLFSSR.R	2	4.92	0.49	-5.94
IPI00032258	Complement C4-A precursor	R.TTNIQGINLLFSSR.R	3	3.10	0.21	-1.09
IPI00032258	Complement C4-A precursor	R.TTNIQGINLLFSSRR.G	3	1.87	0.16	-3.72
IPI00032258	Complement C4-A precursor	R.TYNVLDM*K.N	1	2.12	0.06	-3.59
IPI00032258	Complement C4-A precursor	R.TYNVLDM*K.N	2	3.22	0.28	-0.99
IPI00032258	Complement C4-A precursor	R.VDVQAGACEGK.L	2	3.75	0.38	-3.30
IPI00032258	Complement C4-A precursor	R.VDVQAGACEGKLELSVDGAK.Q	2	5.46	0.53	-5.36
IPI00032258	Complement C4-A precursor	R.VDVQAGACEGKLELSVDGAK.Q	3	4.72	0.49	-4.50
IPI00032258	Complement C4-A precursor	R.VEASISK.A	1	2.35	0.16	-2.43
IPI00032258	Complement C4-A precursor	R.VEASISKANSFLGEK.A	2	3.61	0.38	-2.51
IPI00032258	Complement C4-A precursor	R.VEASISKANSFLGEK.A	3	2.97	0.21	
IPI00032258	Complement C4-A precursor	R.VEYGFQVK.V	1	2.17	0.15	-3.54

IPI00032258	Complement C4-A precursor	R.VEYGFQVK.V	2	2.70	0.29	-2.50
IPI00032258	Complement C4-A precursor	R.VFALDQK.M	1	2.15	0.11	-3.54
IPI00032258	Complement C4-A precursor	R.VFALDQK.M	2	2.72	0.12	-4.54
IPI00032258	Complement C4-A precursor	R.VFREFHLHLR.L	3	3.52	0.16	-2.35
IPI00032258	Complement C4-A precursor	R.VGDTLNLNLR.A	1	2.45	0.19	-3.63
IPI00032258	Complement C4-A precursor	R.VGDTLNLNLR.A	2	3.88	0.28	-4.23
IPI00032258	Complement C4-A precursor	R.VQQPDCREPFLSCCQFAESLR.K	2	3.08	0.31	
IPI00032258	Complement C4-A precursor	R.VQQPDCREPFLSCCQFAESLR.K	3	5.06	0.53	-2.44
IPI00032258	Complement C4-A precursor	R.VQQPDCREPFLSCCQFAESLRK.K	3	5.12	0.27	
IPI00032258	Complement C4-A precursor	R.VTASDPLDTLGSEGALSPGGVASLLR.L	2	6.71	0.56	-5.11
IPI00032258	Complement C4-A precursor	R.VTASDPLDTLGSEGALSPGGVASLLR.L	3	7.64	0.51	-6.95
IPI00032258	Complement C4-A precursor	R.VTASDPLDTLGSEGALSPGGVASLLRLPR.G	3	4.78	0.50	-3.68
IPI00032258	Complement C4-A precursor	R.VTASDPLDTLGSEGALSPGGVASLLRLPR.G	4	3.56	0.41	-3.75
IPI00032258	Complement C4-A precursor	R.YIYGKPVQGVAY.V	2	3.06	0.41	-3.37
IPI00032258	Complement C4-A precursor	R.YIYGKPVQGVAYVR.F	2	4.19	0.54	-4.05
IPI00032258	Complement C4-A precursor	R.YIYGKPVQGVAYVR.F	3	3.33	0.48	-2.45
IPI00032258	Complement C4-A precursor	R.YLDKTEQWSTLPPETK.D	2	3.68	0.45	-4.38
IPI00032258	Complement C4-A precursor	R.YLDKTEQWSTLPPETK.D	3	3.49	0.28	-2.04
IPI00032258	Complement C4-A precursor	R.YRVFALDQK.M	1	2.09	0.16	-2.31
IPI00032258	Complement C4-A precursor	R.YRVFALDQK.M	2	2.64	0.14	-0.54
IPI00032258	Complement C4-A precursor	R.YVSHFETEGPHVLLYFDSVPTSR.E	2	5.75	0.62	-4.15
IPI00032258	Complement C4-A precursor	R.YVSHFETEGPHVLLYFDSVPTSR.E	3	5.10	0.55	-6.74
IPI00032258	Complement C4-A precursor	S.PGGVASLLR.L	2	3.15	0.15	-3.95
IPI00032258	Complement C4-A precursor	T.PGKPYILTVPGHLDEM*QLDIQAR.Y	3	4.71	0.40	-2.09
IPI00032258	Complement C4-A precursor	V.DFTLSSERDFALLSLQVPLKDAK.S	3	4.21	0.29	-2.73
IPI00032258	Complement C4-A precursor	V.GSGATFSHYYYM*ILSR.G	2	3.83	0.43	-4.82
IPI00032258	Complement C4-A precursor	V.PGAPFLLQALVR.E	1	2.24	0.19	-4.50
IPI00032258	Complement C4-A precursor	W.YFVSSPFSLDLSK.T	2	3.03	0.25	-0.93
IPI00032258	Complement C4-A precursor	Y.ILTVPGHLDEM*QLDIQAR.Y	2	3.61	0.28	-1.12
IPI00032288	MANSC domain-containing protein 1 precursor	K.FGSSDHLEK.L	2	2.76	0.18	-2.16
IPI00032288	MANSC domain-containing protein 1 precursor	K.M*DEASAQLLAYK.E	2	3.88	0.42	-3.09
IPI00032288	MANSC domain-containing protein 1 precursor	K.M*DEASAQLLAYKEK.G	2	3.87	0.39	-2.43
IPI00032288	MANSC domain-containing protein 1 precursor	K.M*DEASAQLLAYKEK.G	3	3.64	0.41	-0.91
IPI00032288	MANSC domain-containing protein 1 precursor	K.SLEDVVIDIQSSLSK.G	2	5.24	0.36	-4.24
IPI00032288	MANSC domain-containing protein 1 precursor	K.SLEDVVIDIQSSLSK.G	3	5.48	0.34	-2.86
IPI00032288	MANSC domain-containing protein 1 precursor	R.IITDFPSLTR.N	2	1.52	0.07	-4.33
IPI00032291	Complement C5 precursor	K.AFTECCVVASQLR.A	2	4.02	0.41	-2.94
IPI00032291	Complement C5 precursor	K.ALLVGEHLNIIVTPK.S	3	3.28	0.36	-1.73
IPI00032291	Complement C5 precursor	K.ALVEGVDQLFTDYQIK.D	2	5.14	0.50	-5.57
IPI00032291	Complement C5 precursor	K.ATLLDIYK.T	2	1.88	0.09	-2.43
IPI00032291	Complement C5 precursor	K.CCYDGACVNNDETCEQR.A	2	6.02	0.65	-4.87
IPI00032291	Complement C5 precursor	K.DGHVILQLNSIPSSDFLCVR.F	3	2.74	0.08	-3.38

IPI00032291	Complement C5 precursor	K.DINYVNPVIK.W	1	3.39	0.28	-2.26
IPI00032291	Complement C5 precursor	K.DINYVNPVIK.W	2	3.27	0.26	-1.09
IPI00032291	Complement C5 precursor	K.DNLQHKDSSVPNTGTAR.M	3	3.82	0.27	-4.21
IPI00032291	Complement C5 precursor	K.DSEITFIKK.V	2	2.16	0.17	-1.12
IPI00032291	Complement C5 precursor	K.DSLDQLVGGVPVTLNAQTIDVNQETSDLDPSK.S	3	2.67	0.14	-4.63
IPI00032291	Complement C5 precursor	K.DSSVPNTGTAR.M	1	1.99	0.11	-1.97
IPI00032291	Complement C5 precursor	K.DSSVPNTGTAR.M	2	2.50	0.13	-1.01
IPI00032291	Complement C5 precursor	K.EFPYRIPLDLVPKTEIKR.I	4	2.42	0.22	-2.30
IPI00032291	Complement C5 precursor	K.ENSQYQPIKLQGTLPVEAR.E	3	3.34	0.28	-2.68
IPI00032291	Complement C5 precursor	K.FQNSAILTIQPK.Q	2	4.71	0.37	-1.77
IPI00032291	Complement C5 precursor	K.FSDASYQSINIPVTQNM*VPSSR.L	2	4.07	0.53	-4.11
IPI00032291	Complement C5 precursor	K.FSDASYQSINIPVTQNM*VPSSR.L	3	3.91	0.46	-3.20
IPI00032291	Complement C5 precursor	K.GGSASTWLTAFALR.V	2	4.62	0.42	-0.74
IPI00032291	Complement C5 precursor	K.GTVYNYR.T	2	2.31	0.29	-1.46
IPI00032291	Complement C5 precursor	K.IDTQDIEASHYR.G	2	3.96	0.47	-1.23
IPI00032291	Complement C5 precursor	K.IDTQDIEASHYR.G	3	2.71	0.15	-1.31
IPI00032291	Complement C5 precursor	K.ITHYNYLILSK.G	2	3.91	0.34	-2.90
IPI00032291	Complement C5 precursor	K.KIEEIAAK.Y	1	2.09	0.08	-3.90
IPI00032291	Complement C5 precursor	K.KIEEIAAK.Y	2	2.74	0.07	-3.30
IPI00032291	Complement C5 precursor	K.LNLVATPLFLKPGIPYPIK.V	3	4.09	0.45	-2.97
IPI00032291	Complement C5 precursor	K.LNLVATPLFLKPGIPYPIKVQVK.D	3	3.68	0.44	-4.26
IPI00032291	Complement C5 precursor	K.LNLVATPLFLKPGIPYPIKVQVK.D	4	3.00	0.40	-4.69
IPI00032291	Complement C5 precursor	K.LQGTLPVEAR.E	2	2.85	0.29	-2.38
IPI00032291	Complement C5 precursor	K.M*SAVEGICTSESPVIDHQGTK.S	3	3.08	0.32	-2.95
IPI00032291	Complement C5 precursor	K.NFKNFEITIK.A	3	2.46	0.07	-1.51
IPI00032291	Complement C5 precursor	K.QCTM*FYSTSNIK.I	2	3.09	0.43	-1.53
IPI00032291	Complement C5 precursor	K.QLPGGQNPVSYVYLEVVSK.H	2	4.11	0.53	-3.94
IPI00032291	Complement C5 precursor	K.QLPGGQNPVSYVYLEVVSK.H	3	3.46	0.38	-3.78
IPI00032291	Complement C5 precursor	K.RM*PITYDNGFLFIHTDKPVYTPDQSVK.V	4	3.94	0.27	-4.46
IPI00032291	Complement C5 precursor	K.SPYIDKITHYNYLILSK.G	2	5.16	0.41	-2.65
IPI00032291	Complement C5 precursor	K.SPYIDKITHYNYLILSK.G	3	4.00	0.34	-3.30
IPI00032291	Complement C5 precursor	K.SPYIDKITHYNYLILSK.G	4	3.64	0.31	0.72
IPI00032291	Complement C5 precursor	K.TDAPDLPEENQAR.E	2	3.94	0.37	-3.97
IPI00032291	Complement C5 precursor	K.TGEAVAEKDSEITFIK.K	2	4.75	0.43	-2.18
IPI00032291	Complement C5 precursor	K.TGEAVAEKDSEITFIK.K	3	3.31	0.35	-2.00
IPI00032291	Complement C5 precursor	K.TGEAVAEKDSEITFIKK.V	2	5.30	0.54	-3.80
IPI00032291	Complement C5 precursor	K.TGEAVAEKDSEITFIKK.V	3	4.59	0.49	-2.56
IPI00032291	Complement C5 precursor	K.TGEAVAEKDSEITFIKK.V	4	3.16	0.25	-3.10
IPI00032291	Complement C5 precursor	K.TLLPVSKPEIR.S	2	2.58	0.21	-2.16
IPI00032291	Complement C5 precursor	K.VFKDVFLEM*NIPYSVVR.G	2	5.44	0.43	-2.79
IPI00032291	Complement C5 precursor	K.VFKDVFLEM*NIPYSVVR.G	3	5.03	0.34	-1.92
IPI00032291	Complement C5 precursor	K.VFKDVFLEM*NIPYSVVRGEQIQLK.G	3	4.21	0.42	-3.36

IPI00032291	Complement C5 precursor	K.VSITSITVENVFVK.Y	2	4.10	0.46	-4.14
IPI00032291	Complement C5 precursor	K.VTCTNAELVK.G	2	2.70	0.26	-1.83
IPI00032291	Complement C5 precursor	K.YKATLLDIYK.T	2	2.56	0.28	-3.43
IPI00032291	Complement C5 precursor	K.YKATLLDIYK.T	3	3.02	0.17	-3.96
IPI00032291	Complement C5 precursor	K.YKATLLDIYKTGEAVAEKDSEITFIK.K	3	5.21	0.59	-3.75
IPI00032291	Complement C5 precursor	K.YKATLLDIYKTGEAVAEKDSEITFIK.K	4	3.11	0.12	-4.11
IPI00032291	Complement C5 precursor	K.YKATLLDIYKTGEAVAEKDSEITFIK.K	5	3.15	0.21	-4.17
IPI00032291	Complement C5 precursor	K.YKATLLDIYKTGEAVAEKDSEITFIKK.V	5	4.14	0.29	-2.39
IPI00032291	Complement C5 precursor	K.YNFSFR.Y	1	1.42	0.16	-2.03
IPI00032291	Complement C5 precursor	K.YNFSFR.Y	2	1.85	0.20	-2.23
IPI00032291	Complement C5 precursor	K.YVLSPYK.L	1	1.99	0.18	-1.91
IPI00032291	Complement C5 precursor	R.EKFSDASYQSINIPVTQNM*VPSSR.L	3	5.27	0.48	-2.26
IPI00032291	Complement C5 precursor	R.ESYSGVTLDPR.G	2	2.42	0.25	-2.33
IPI00032291	Complement C5 precursor	R.ETVLTFIDPEGSEVDM*VEEIDHIGIISFPDFK.I	3	4.05	0.38	-5.95
IPI00032291	Complement C5 precursor	R.ETVLTFIDPEGSEVDM*VEEIDHIGIISFPDFKIPSNPR.Y	4	3.42	0.29	-4.20
IPI00032291	Complement C5 precursor	R.IPLDLVPK.T	2	2.40	0.23	-2.93
IPI00032291	Complement C5 precursor	R.LSM*DIDVSYK.H	2	2.78	0.27	-2.01
IPI00032291	Complement C5 precursor	R.M*PITYDNGFLFIHTDKPVYTPDQSVK.V	3	5.38	0.56	-1.62
IPI00032291	Complement C5 precursor	R.M*VETTAYALLTSLNLK.D	2	4.88	0.43	-3.94
IPI00032291	Complement C5 precursor	R.M*VETTAYALLTSLNLK.D	3	4.00	0.34	-3.37
IPI00032291	Complement C5 precursor	R.M*VETTAYALLTSLNLKDINYVNPVIK.W	3	7.36	0.60	-3.81
IPI00032291	Complement C5 precursor	R.M*VETTAYALLTSLNLKDINYVNPVIK.W	4	4.79	0.31	-3.37
IPI00032291	Complement C5 precursor	R.NADYSYSVWK.G	2	2.68	0.20	-3.19
IPI00032291	Complement C5 precursor	R.SIVSALKR.E	2	2.12	0.17	-2.44
IPI00032291	Complement C5 precursor	R.VVPEGVKR.E	2	1.57	0.07	-1.94
IPI00032291	Complement C5 precursor	R.VYSLNDDLKPAKR.E	2	4.51	0.41	-3.78
IPI00032291	Complement C5 precursor	R.YGGGFYSTQDTINAIEGLTEYSLLVK.Q	2	5.50	0.64	-4.40
IPI00032291	Complement C5 precursor	R.YGGGFYSTQDTINAIEGLTEYSLLVK.Q	3	5.71	0.50	-9.09
IPI00032291	Complement C5 precursor	R.YIYPLDSLTWIEYWPR.D	2	4.47	0.52	-4.43
IPI00032291	Complement C5 precursor	R.YIYPLDSLTWIEYWPR.D	3	3.58	0.21	-4.40
IPI00032292	Metalloproteinase inhibitor 1 precursor	K.GFQALGDAADIR.F	1	2.23	0.15	-2.08
IPI00032292	Metalloproteinase inhibitor 1 precursor	K.GFQALGDAADIR.F	2	4.00	0.33	-2.13
IPI00032292	Metalloproteinase inhibitor 1 precursor	K.LQDGLLHITTCSFVAPWNSLSLAQR.R	2	4.85	0.50	-3.41
IPI00032292	Metalloproteinase inhibitor 1 precursor	K.LQDGLLHITTCSFVAPWNSLSLAQR.R	3	4.86	0.30	-3.51
IPI00032292	Metalloproteinase inhibitor 1 precursor	K.LQSGTHCLWTDQLLQGSEK.G	3	3.19	0.20	
IPI00032292	Metalloproteinase inhibitor 1 precursor	K.M*YKGFQALGDAADIR.F	2	3.71	0.40	-4.34
IPI00032292	Metalloproteinase inhibitor 1 precursor	K.M*YKGFQALGDAADIR.F	3	2.81	0.20	-1.75
IPI00032292	Metalloproteinase inhibitor 1 precursor	K.TYTVGCEECTVFPCLSIPCK.L	2	3.94	0.49	-3.26
IPI00032292	Metalloproteinase inhibitor 1 precursor	R.EPGLCTWQSLR.S	2	2.34	0.29	-2.82
IPI00032292	Metalloproteinase inhibitor 1 precursor	R.FVYTPAM*ESVCGYFHR.S	2	3.62	0.40	
IPI00032292	Metalloproteinase inhibitor 1 precursor	R.SEEFLIAGK.L	2	3.08	0.20	-2.27
IPI00032293	Cystatin-C precursor	A.AGSSPGKPPR.L	1	1.91	0.23	-0.69

IPI00032293	Cystatin-C precursor	A.FCSFQIYAVPWQGTM*TLSK.S	2	5.14	0.55	-4.87
IPI00032293	Cystatin-C precursor	A.FCSFQIYAVPWQGTM*TLSK.S	3	4.55	0.36	-3.55
IPI00032293	Cystatin-C precursor	A.GSSPGKPPR.L	1	1.89	0.17	-0.66
IPI00032293	Cystatin-C precursor	A.GVNYFLDVELGR.T	2	3.69	0.32	-6.69
IPI00032293	Cystatin-C precursor	A.LDFAVGEYNK.A	1	2.41	0.25	-4.01
IPI00032293	Cystatin-C precursor	A.LDFAVGEYNK.A	2	3.31	0.41	-4.70
IPI00032293	Cystatin-C precursor	A.VSPAAGSSPGKPPR.L	1	3.65	0.38	0.34
IPI00032293	Cystatin-C precursor	A.VSPAAGSSPGKPPR.L	2	3.47	0.49	-3.43
IPI00032293	Cystatin-C precursor	C.PFHDQPHLK.R	2	3.13	0.29	-1.17
IPI00032293	Cystatin-C precursor	C.SFQIYAVPWQGTM*TLSK.S	2	5.08	0.42	-4.25
IPI00032293	Cystatin-C precursor	C.SFQIYAVPWQGTMTLSK.S	2	4.65	0.47	-4.21
IPI00032293	Cystatin-C precursor	F.CSFQIYAVPWQGTM*TLSK.S	2	4.32	0.44	-3.71
IPI00032293	Cystatin-C precursor	F.QIYAVPWQGTM*TLSK.S	2	3.01	0.35	-2.77
IPI00032293	Cystatin-C precursor	G.GPM*DASVEEEGVR.R	2	3.85	0.36	-3.93
IPI00032293	Cystatin-C precursor	G.PM*DASVEEEGVR.R	2	4.03	0.37	-2.02
IPI00032293	Cystatin-C precursor	G.VNYFLDVELGR.T	2	3.83	0.41	-4.09
IPI00032293	Cystatin-C precursor	K.AFCSFQIYAVPWQGTM*TLSK.S	2	4.77	0.55	-6.47
IPI00032293	Cystatin-C precursor	K.AFCSFQIYAVPWQGTM*TLSK.S	3	6.22	0.50	-5.58
IPI00032293	Cystatin-C precursor	K.AFCSFQIYAVPWQGTMTLSK.S	2	5.01	0.60	-4.51
IPI00032293	Cystatin-C precursor	K.AFCSFQIYAVPWQGTMTLSK.S	3	4.96	0.46	-3.85
IPI00032293	Cystatin-C precursor	K.QIVAGVNY.F	1	2.30	0.32	-2.75
IPI00032293	Cystatin-C precursor	K.QIVAGVNYFLDVELGR.T	2	5.62	0.64	-7.57
IPI00032293	Cystatin-C precursor	K.QIVAGVNYFLDVELGR.T	3	6.43	0.46	-8.15
IPI00032293	Cystatin-C precursor	K.TQPNLDNCPFHDQPHLK.R	2	4.23	0.52	-4.79
IPI00032293	Cystatin-C precursor	K.TQPNLDNCPFHDQPHLK.R	4	2.73	0.38	-3.64
IPI00032293	Cystatin-C precursor	L.DFAVGEYNK.A	1	2.47	0.32	-4.32
IPI00032293	Cystatin-C precursor	L.VGGPM*DASVEEEGVR.R	2	3.70	0.38	-2.77
IPI00032293	Cystatin-C precursor	N.YFLDVELGR.T	2	3.63	0.35	-2.85
IPI00032293	Cystatin-C precursor	P.AAGSSPGKPPR.L	1	2.12	0.27	-0.32
IPI00032293	Cystatin-C precursor	P.RLVGGPM*DASVEEEGVR.R	3	4.75	0.28	-1.69
IPI00032293	Cystatin-C precursor	R.ALDFAVGEY.N	1	2.00	0.27	-1.22
IPI00032293	Cystatin-C precursor	R.ALDFAVGEYNK.A	1	3.03	0.46	-4.71
IPI00032293	Cystatin-C precursor	R.ALDFAVGEYNK.A	2	4.20	0.47	-5.21
IPI00032293	Cystatin-C precursor	R.KAFCSFQIY.A	1	1.82	0.24	-3.27
IPI00032293	Cystatin-C precursor	R.KAFCSFQIYAVPWQGTM*TLSK.S	2	5.49	0.51	-4.80
IPI00032293	Cystatin-C precursor	R.KAFCSFQIYAVPWQGTM*TLSK.S	3	4.36	0.48	-5.58
IPI00032293	Cystatin-C precursor	R.KQIVAGVNY.F	1	2.81	0.40	-3.05
IPI00032293	Cystatin-C precursor	R.KQIVAGVNYFLDVELGR.T	2	6.50	0.65	-8.05
IPI00032293	Cystatin-C precursor	R.KQIVAGVNYFLDVELGR.T	3	5.84	0.41	-5.89
IPI00032293	Cystatin-C precursor	R.LVGGPM*DASVEEEGVR.R	2	5.57	0.50	-4.98
IPI00032293	Cystatin-C precursor	R.LVGGPM*DASVEEEGVR.R	3	5.41	0.29	-2.79
IPI00032293	Cystatin-C precursor	R.LVGGPM*DASVEEEGVRR.A	2	2.63	0.21	-3.55

IPI00032293	Cystatin-C precursor	R.LVGGPMDASVEEEGVR.R	2	3.57	0.34	
IPI00032293	Cystatin-C precursor	R.RALDFAVGEYNK.A	1	3.42	0.52	-5.06
IPI00032293	Cystatin-C precursor	R.RALDFAVGEYNK.A	2	4.04	0.46	-4.51
IPI00032293	Cystatin-C precursor	R.RALDFAVGEYNK.A	3	3.30	0.28	-4.57
IPI00032293	Cystatin-C precursor	S.FQIYAVPWQGTM*TLSK.S	2	4.89	0.47	-3.95
IPI00032293	Cystatin-C precursor	S.FQIYAVPWQGTM*TLSK.S	3	3.56	0.33	-3.00
IPI00032293	Cystatin-C precursor	S.FQIYAVPWQGTMTLSK.S	2	3.81	0.35	-3.14
IPI00032293	Cystatin-C precursor	S.PAAGSSPGKPPR.L	2	3.53	0.43	-1.70
IPI00032293	Cystatin-C precursor	V.GGPM*DASVEEEGVR.R	2	3.96	0.39	-3.72
IPI00032293	Cystatin-C precursor	V.PWQGTM*TLSK.S	1	2.34	0.26	-5.76
IPI00032293	Cystatin-C precursor	V.PWQGTM*TLSK.S	2	2.98	0.28	-1.94
IPI00032293	Cystatin-C precursor	V.SPAAGSSPGKPPR.L	2	4.12	0.40	-2.31
IPI00032293	Cystatin-C precursor	Y.AVPWQGTM*TLSK.S	1	2.05	0.24	-3.18
IPI00032293	Cystatin-C precursor	Y.AVPWQGTM*TLSK.S	2	2.98	0.42	-3.20
IPI00032311	Lipopolysaccharide-binding protein precursor	K.GLQYAAQEGLLALQSELLR.I	2	5.68	0.39	
IPI00032311	Lipopolysaccharide-binding protein precursor	K.GLQYAAQEGLLALQSELLR.I	3	4.91	0.42	-5.19
IPI00032311	Lipopolysaccharide-binding protein precursor	K.SVSSDLQPYLQTLPVTTEIDSFADIDYSLVEAPR.A	3	5.05	0.33	
IPI00032311	Lipopolysaccharide-binding protein precursor	R.ITDKGLQYAAQEGLLALQSELLR.I	2	4.85	0.46	
IPI00032311	Lipopolysaccharide-binding protein precursor	R.ITDKGLQYAAQEGLLALQSELLR.I	3	3.06	0.20	-2.51
IPI00032311	Lipopolysaccharide-binding protein precursor	R.SPVTLLAAVM*SLPEEHNK.M	3	3.86	0.35	-1.48
IPI00032311	Lipopolysaccharide-binding protein precursor	R.SPVTLLAAVMSLPEEHNK.M	3	4.73	0.36	
IPI00032313	Protein S100-A4	R.ELPSFLGK.R	2	1.42	0.18	-2.81
IPI00032328	Isoform HMW of Kininogen-1 precursor	C.VHPISTQSPDLEPILR.H	3	3.83	0.27	0.40
IPI00032328	Isoform HMW of Kininogen-1 precursor	D.IPTNSPELEETLTHTITK.L	3	4.68	0.54	-3.17
IPI00032328	Isoform HMW of Kininogen-1 precursor	I.PTNSPELEETLTHTITK.L	2	5.22	0.57	-3.52
IPI00032328	Isoform HMW of Kininogen-1 precursor	I.PTNSPELEETLTHTITK.L	3	4.29	0.40	-3.93
IPI00032328	Isoform HMW of Kininogen-1 precursor	K.AATGECTATVGK.R	2	3.81	0.46	-4.72
IPI00032328	Isoform HMW of Kininogen-1 precursor	K.AATGECTATVGKR.S	2	3.43	0.38	-4.18
IPI00032328	Isoform HMW of Kininogen-1 precursor	K.AVDAALKK.Y	2	2.72	0.20	-4.79
IPI00032328	Isoform HMW of Kininogen-1 precursor	K.DFVQPPTK.I	1	1.67	0.06	-2.56
IPI00032328	Isoform HMW of Kininogen-1 precursor	K.DFVQPPTK.I	2	2.48	0.15	-4.19
IPI00032328	Isoform HMW of Kininogen-1 precursor	K.ENFLFLTPDCK.S	1	3.27	0.29	-3.78
IPI00032328	Isoform HMW of Kininogen-1 precursor	K.ENFLFLTPDCK.S	2	4.01	0.31	-3.73
IPI00032328	Isoform HMW of Kininogen-1 precursor	K.FSVATQTCQITPAEGPVVTAQYDCLGCVHPISTQSPDLEPILR.H	3	4.34	0.53	-3.40
IPI00032328	Isoform HMW of Kininogen-1 precursor	K.FSVATQTCQITPAEGPVVTAQYDCLGCVHPISTQSPDLEPILR.H	4	5.64	0.52	-2.18
IPI00032328	Isoform HMW of Kininogen-1 precursor	K.IYPTVNCQPLGM*ISLM*K.R	2	5.10	0.49	-3.81
IPI00032328	Isoform HMW of Kininogen-1 precursor	K.IYPTVNCQPLGM*ISLM*K.R	3	3.71	0.45	-6.75
IPI00032328	Isoform HMW of Kininogen-1 precursor	K.IYPTVNCQPLGMISLM*K.R	2	5.46	0.17	
IPI00032328	Isoform HMW of Kininogen-1 precursor	K.IYPTVNCQPLGMISLMK.R	2	3.48	0.29	
IPI00032328	Isoform HMW of Kininogen-1 precursor	K.KIYPTVNCQPLGM*ISLM*.K	2	4.17	0.47	-2.21
IPI00032328	Isoform HMW of Kininogen-1 precursor	K.KIYPTVNCQPLGM*ISLM*K.R	2	3.85	0.42	-4.40
IPI00032328	Isoform HMW of Kininogen-1 precursor	K.KIYPTVNCQPLGM*ISLM*K.R	3	4.31	0.31	-5.44

IPI00032328	Isoform HMW of Kininogen-1 precursor	K.KLGQSLDCNAEVYVVPWEK.K	2	4.84	0.35	
IPI00032328	Isoform HMW of Kininogen-1 precursor	K.KLGQSLDCNAEVYVVPWEKK.I	3	4.40	0.28	
IPI00032328	Isoform HMW of Kininogen-1 precursor	K.KYFIDFVAR.E	1	2.82	0.27	-3.27
IPI00032328	Isoform HMW of Kininogen-1 precursor	K.KYFIDFVAR.E	2	3.13	0.35	-3.25
IPI00032328	Isoform HMW of Kininogen-1 precursor	K.KYNSQNQSNNQFVLYR.I	2	5.50	0.47	-4.39
IPI00032328	Isoform HMW of Kininogen-1 precursor	K.KYNSQNQSNNQFVLYR.I	3	4.59	0.43	-2.89
IPI00032328	Isoform HMW of Kininogen-1 precursor	K.LDDDLEHQGGHVLDHGHK.H	3	2.68	0.16	-3.43
IPI00032328	Isoform HMW of Kininogen-1 precursor	K.LDDDLEHQGGHVLDHGHKHKHGHGHGK.H	7	2.22	0.10	-3.65
IPI00032328	Isoform HMW of Kininogen-1 precursor	K.LGQSLDCNAEVYVVPWEK.K	2	5.04	0.47	-5.49
IPI00032328	Isoform HMW of Kininogen-1 precursor	K.LGQSLDCNAEVYVVPWEKK.I	3	4.20	0.37	-1.56
IPI00032328	Isoform HMW of Kininogen-1 precursor	K.RPPGFSPFR.S	1	1.59	0.30	-5.39
IPI00032328	Isoform HMW of Kininogen-1 precursor	K.RPPGFSPFR.S	2	2.67	0.22	-3.42
IPI00032328	Isoform HMW of Kininogen-1 precursor	K.SLWNGDTGECTDNAYIDIQLR.I	2	5.24	0.56	-4.45
IPI00032328	Isoform HMW of Kininogen-1 precursor	K.SLWNGDTGECTDNAYIDIQLR.I	3	3.79	0.06	
IPI00032328	Isoform HMW of Kininogen-1 precursor	K.TVGSDTFYSFK.Y	1	2.61	0.30	-1.83
IPI00032328	Isoform HMW of Kininogen-1 precursor	K.TVGSDTFYSFK.Y	2	3.86	0.46	-1.80
IPI00032328	Isoform HMW of Kininogen-1 precursor	K.TVGSDTFYSFKYEIK.E	2	4.11	0.39	-4.98
IPI00032328	Isoform HMW of Kininogen-1 precursor	K.TWQDCEYK.D	2	2.03	0.11	-2.56
IPI00032328	Isoform HMW of Kininogen-1 precursor	K.TWQDCEYKDAAK.A	2	3.78	0.23	
IPI00032328	Isoform HMW of Kininogen-1 precursor	K.YEIKEGDCPVQSGK.T	2	4.78	0.36	
IPI00032328	Isoform HMW of Kininogen-1 precursor	K.YFIDFVAR.E	1	2.65	0.33	-4.15
IPI00032328	Isoform HMW of Kininogen-1 precursor	K.YFIDFVAR.E	2	3.44	0.35	-3.76
IPI00032328	Isoform HMW of Kininogen-1 precursor	K.YNSQNQSNNQFVLYR.I	2	4.61	0.38	-3.21
IPI00032328	Isoform HMW of Kininogen-1 precursor	K.YNSQNQSNNQFVLYR.I	3	5.37	0.47	-3.89
IPI00032328	Isoform HMW of Kininogen-1 precursor	R.DIPTNSPELEETLTHTITK.L	2	4.59	0.51	-4.42
IPI00032328	Isoform HMW of Kininogen-1 precursor	R.IASFSQNCDIYPGKDFVQPPTK.I	2	4.15	0.55	-2.76
IPI00032328	Isoform HMW of Kininogen-1 precursor	R.IASFSQNCDIYPGKDFVQPPTK.I	3	4.84	0.54	-4.44
IPI00032328	Isoform HMW of Kininogen-1 precursor	R.IASFSQNCDIYPGKDFVQPPTK.I	4	3.08	0.17	-3.36
IPI00032328	Isoform HMW of Kininogen-1 precursor	R.KHNLGHGHKHER.D	3	1.96	0.16	-1.52
IPI00032328	Isoform HMW of Kininogen-1 precursor	R.KHNLGHGHKHERDQGHGHQR.G	3	2.84	0.32	-4.15
IPI00032328	Isoform HMW of Kininogen-1 precursor	R.KHNLGHGHKHERDQGHGHQR.G	4	2.87	0.40	-3.92
IPI00032328	Isoform HMW of Kininogen-1 precursor	R.KHNLGHGHKHERDQGHGHQR.G	5	2.32	0.33	-2.83
IPI00032328	Isoform HMW of Kininogen-1 precursor	R.QVVAGLNFR.I	1	1.97	0.18	-1.97
IPI00032328	Isoform HMW of Kininogen-1 precursor	R.QVVAGLNFR.I	2	2.47	0.33	-3.28
IPI00032328	Isoform HMW of Kininogen-1 precursor	R.VQVVAGK.K	1	2.43	0.10	-3.23
IPI00032338	kelch-like 20	R.AM*FTGELAESRQTEVVIR.D	2	1.96	0.12	-4.05
IPI00032405	Endothelin B receptor-like protein 2 precursor	R.VSGGAPLHLGR.H	1	2.20	0.11	-2.23
IPI00032405	Endothelin B receptor-like protein 2 precursor	R.VSGGAPLHLGR.H	2	3.92	0.42	-2.16
IPI00032416	Isoform Long of Protein jagged-2 precursor	R.DLPDSSLIQGAAHAIVAAITQR.G	3	2.35	0.08	-3.65
IPI00032425	Receptor activity-modifying protein 3 precursor	K.AFADM*M*GK.V	2	2.35	0.18	-3.04
IPI00032532	Isoform 2 of Growth arrest-specific protein 6 precursor	K.YGSPYTK.N	1	1.65	0.23	-3.03

IPI00032532	Isoform 2 of Growth arrest-specific protein 6 precursor	R.AVPLSVALVDYHSTK.K	2	4.50	0.50	-2.71
IPI00032532	Isoform 2 of Growth arrest-specific protein 6 precursor	R.CEQVCVNSPGSYTCHCDGR.G	3	3.73	0.26	
IPI00032532	Isoform 2 of Growth arrest-specific protein 6 precursor	R.DGEATLEVDGTR.G	2	2.31	0.26	-2.04
IPI00032532	Isoform 2 of Growth arrest-specific protein 6 precursor	R.DVDECLQGR.C	2	2.81	0.32	-4.21
IPI00032532	Isoform 2 of Growth arrest-specific protein 6 precursor	R.GQSEVSAAQLQER.L	2	4.11	0.34	-2.46
IPI00032532	Isoform 2 of Growth arrest-specific protein 6 precursor	R.GSFYPGSGFAFYSLDYM*R.T	2	4.42	0.57	-3.20
IPI00032532	Isoform 2 of Growth arrest-specific protein 6 precursor	R.LLDLDEAAYK.H	2	3.06	0.24	-1.72
IPI00032532	Isoform 2 of Growth arrest-specific protein 6 precursor	R.M*QCFSVTER.G	2	2.13	0.08	-3.74
IPI00032532	Isoform 2 of Growth arrest-specific protein 6 precursor	R.SPVLTFAGGLPDVPVTSAPVTAFYR.G	2	5.73	0.61	-5.27
IPI00032532	Isoform 2 of Growth arrest-specific protein 6 precursor	R.SPVLTFAGGLPDVPVTSAPVTAFYR.G	3	4.41	0.41	-4.69
IPI00032532	Isoform 2 of Growth arrest-specific protein 6 precursor	R.YLDCINK.Y	2	1.94	0.08	-1.58
IPI00032597	RNA-binding motif protein, X-linked 2	K.SRTAYSGGAEDLERELKKEKPK.H	3	2.90	0.11	
IPI00032826	Hsc70-interacting protein	K.AIDLFTDAIK.L	2	2.46	0.27	-2.99
IPI00032830	Isoform 1 of Oligoribonuclease, mitochondrial precursor (Fragment)	R.ALDDISESIKELQFYR.N	3	3.36	0.29	-1.68
IPI00032904	Beta-synuclein	K.AKEGVVAAAEK.T	2	2.05	0.05	2.33
IPI00033030	Protein ADRM1	R.SQSAAVTPSSTTSSTR.A	2	3.31	0.38	-2.03
IPI00033419	Protein Fem-1 homolog b	R.STPLIIAARNGHAK.V	2	2.63	0.06	
IPI00033466	C-type lectin domain family 11 member A precursor	R.DAVQALQEAQGR.A	2	2.82	0.26	-3.81
IPI00033466	C-type lectin domain family 11 member A precursor	R.DFEAQAAAQAR.C	2	3.84	0.24	
IPI00033560	Isoform Alpha of Receptor-type tyrosine-protein phosphatase R precursor	K.NSIELFVSPINRK.T	2	3.37	0.19	-3.79
IPI00033560	Isoform Alpha of Receptor-type tyrosine-protein phosphatase R precursor	K.SGKPVFIYK.H	1	1.95	0.18	-4.12
IPI00033560	Isoform Alpha of Receptor-type tyrosine-protein phosphatase R precursor	K.SGKPVFIYK.H	2	2.21	0.11	1.52
IPI00033560	Isoform Alpha of Receptor-type tyrosine-protein phosphatase R precursor	K.TGISDALPSEEVLR.S	2	4.06	0.30	-1.58

IPI00033560	Isoform Alpha of Receptor-type tyrosine-protein phosphatase R precursor	R.QGVAAALGLLPQQVHINR.L	2	4.01	0.50	-4.42
IPI00033560	Isoform Alpha of Receptor-type tyrosine-protein phosphatase R precursor	R.QGVAAALGLLPQQVHINR.L	3	3.84	0.37	-4.11
IPI00033600	Isoform 1 of Protein phosphatase 1 regulatory subunit 7	R.AIENIDTLTNLESLFLGK.N	2	5.38	0.52	-5.35
IPI00034006	Tyrosine-protein phosphatase non-receptor type 23	R.M*PMIWLDLK.E	2	2.68	0.07	
IPI00034319	Isoform A of Protein CutA precursor	K.GKIEEDSEVLM*M*IK.T	2	4.88	0.46	-3.22
IPI00034319	Isoform A of Protein CutA precursor	K.GKIEEDSEVLM*M*IK.T	3	3.37	0.23	-2.58
IPI00034319	Isoform A of Protein CutA precursor	K.TQSSLVPALTDFVR.S	1	1.48	0.27	-3.75
IPI00034319	Isoform A of Protein CutA precursor	K.TQSSLVPALTDFVR.S	2	4.24	0.46	-3.86
IPI00034319	Isoform A of Protein CutA precursor	K.TQSSLVPALTDFVR.S	3	2.87	0.15	-2.47
IPI00034319	Isoform A of Protein CutA precursor	R.LAACVNLIPQITSIYEWK.G	2	5.35	0.53	-4.12
IPI00034319	Isoform A of Protein CutA precursor	R.LAACVNLIPQITSIYEWK.G	3	5.21	0.43	-3.56
IPI00034319	Isoform A of Protein CutA precursor	R.SVHPYEVAEVIALPVEQGNFPYLQWVR.Q	3	6.20	0.49	-3.83
IPI00034319	Isoform A of Protein CutA precursor	Y.EVAEVIALPVEQGNFPYLQWVR.Q	2	3.73	0.38	-2.75
IPI00038378	Isoform 1 of Enolase-phosphatase E1	K.LLFGHSTEGDILELVDGHFDTK.I	3	3.89	0.18	-1.30
IPI00040730	protocadherin 21 precursor	K.IDITDAETLSR.S	2	3.98	0.24	-2.90
IPI00040900	Isoform 2 of Heparan sulfate 2-O-sulfotransferase 1	R.HEVREIEQRHTM*DGPRQDATLDEEEDM*VIIYNR.V	5	3.06	0.06	1.82
IPI00043201	Centromere protein J	R.IEEFKKEEM*RKLQK.E	2	2.23	0.21	
IPI00043215	immunoglobulin superfamily, member 1 isoform 1	K.EGAQEPLEQQRPSGYR.A	2	1.74	0.08	-1.68
IPI00043215	immunoglobulin superfamily, member 1 isoform 1	K.EGAQEPLEQQRPSGYR.A	3	2.01	0.13	-1.46
IPI00043215	immunoglobulin superfamily, member 1 isoform 1	K.EGEQEPVQQLGAVGR.E	2	3.99	0.40	-2.67
IPI00043215	immunoglobulin superfamily, member 1 isoform 1	K.TWASPVVTPGAR.V	2	2.79	0.16	-1.33
IPI00043215	immunoglobulin superfamily, member 1 isoform 1	K.WSEPSEPLELVIK.E	2	3.58	0.34	-3.47
IPI00043215	immunoglobulin superfamily, member 1 isoform 1	R.FALLQEGAHVPLQFR.S	2	3.70	0.38	-1.73
IPI00043215	immunoglobulin superfamily, member 1 isoform 1	R.FALLQEGAHVPLQFR.S	3	3.35	0.26	-1.97
IPI00043731	CDNA FLJ30671 fis, clone FCBBF1000687, moderately similar to Mus musculus Rap2 interacting protein 8 (RPIP8) mRNA	R.VACRKVSQNCICSIENMENVSSSRAKGR.A	3	2.37	0.06	-0.19
IPI00043756	Isoform 3 of Zinc transporter ZIP12	K.GLSLISKEDFK.Q	2	2.44	0.25	-3.30
IPI00043756	Isoform 3 of Zinc transporter ZIP12	K.QM*SPGIIQQLLSCSCHLPK.D	3	3.22	0.29	-4.45
IPI00043756	Isoform 3 of Zinc transporter ZIP12	K.SPEDSQAAEM*PIGSM*TASNR.K	2	4.66	0.57	-1.45
IPI00043756	Isoform 3 of Zinc transporter ZIP12	K.SPEDSQAAEM*PIGSM*TASNR.K	3	2.40	0.14	-2.89
IPI00043756	Isoform 3 of Zinc transporter ZIP12	K.SPEDSQAAEM*PIGSM*TASNRK.C	3	3.57	0.41	-4.56
IPI00043756	Isoform 3 of Zinc transporter ZIP12	R.LSELDQLLNTLWTR.S	2	4.65	0.35	-4.63
IPI00043756	Isoform 3 of Zinc transporter ZIP12	R.QLVEIFLQK.G	2	2.65	0.15	-2.23
IPI00043756	Isoform 3 of Zinc transporter ZIP12	R.QYFDTSQSQCM*ETK.T	2	4.23	0.50	-4.64
IPI00043756	Isoform 3 of Zinc transporter ZIP12	Y.FDTSQSQCM*ETK.T	2	4.04	0.46	-3.61
IPI00043810	Isoform 1 of Proline-rich transmembrane protein 1	R.LGAGGLASSAATAQR.G	2	4.64	0.44	-2.02

IPI00043810	Isoform 1 of Proline-rich transmembrane protein 1	R.M*PPDPYLQETR.F	2	2.76	0.29	-3.91
IPI00043978	Isoform 1 of Partitioning-defective 3 homolog B	R.SSDPVPGPPADTQPSASHPGGQSLK.L	3	2.67	0.06	1.09
IPI00044326	Carbohydrate sulfotransferase 14	R.AGDADLQVR.Q	2	3.24	0.12	-2.68
IPI00044369	Isoform 1 of Plexin domain-containing protein 2 precursor	K.EIPVLVTQISSTNHPVK.V	2	4.49	0.47	-1.40
IPI00044369	Isoform 1 of Plexin domain-containing protein 2 precursor	K.EIPVLVTQISSTNHPVK.V	3	1.84	0.28	-3.55
IPI00044369	Isoform 1 of Plexin domain-containing protein 2 precursor	K.VGLSDAFVVVHR.I	2	3.61	0.46	-3.43
IPI00044369	Isoform 1 of Plexin domain-containing protein 2 precursor	K.VGLSDAFVVVHR.I	3	4.64	0.49	-3.11
IPI00044369	Isoform 1 of Plexin domain-containing protein 2 precursor	R.ASVGQDSPEPR.S	2	3.32	0.34	-2.79
IPI00044369	Isoform 1 of Plexin domain-containing protein 2 precursor	R.IIFGYKEIPVLVTQISSTNHPVK.V	3	4.59	0.46	-3.81
IPI00044369	Isoform 1 of Plexin domain-containing protein 2 precursor	R.IYGPSDSASR.D	1	1.42	0.08	-3.74
IPI00044369	Isoform 1 of Plexin domain-containing protein 2 precursor	R.IYGPSDSASR.D	2	3.10	0.23	-4.22
IPI00044369	Isoform 1 of Plexin domain-containing protein 2 precursor	R.M*LTATQYIAPLM*ANFDPSVSR.N	2	4.68	0.48	-4.34
IPI00044369	Isoform 1 of Plexin domain-containing protein 2 precursor	R.TIYEYHRVELQM*SK.I	2	3.01	0.28	-3.13
IPI00044369	Isoform 1 of Plexin domain-containing protein 2 precursor	R.VELQM*SK.I	2	2.31	0.06	-3.44
IPI00044600	VPS10 domain-containing receptor SorCS2 precursor	K.MLTAPFAGPIDHGSLTVQDDYIFFK.A	3	3.13	0.13	
IPI00044600	VPS10 domain-containing receptor SorCS2 precursor	R.LGPHAQLTR.V	1	2.43	0.09	-4.50
IPI00044600	VPS10 domain-containing receptor SorCS2 precursor	R.LGPHAQLTR.V	2	2.72	0.23	-2.66
IPI00044607	Protein phosphatase inhibitor 2-like protein 1	K.KSQKWDEM*NILATYRPADKDYGLMK.I	3	3.38	0.11	
IPI00044707	Hyaluronan and proteoglycan link protein 4 precursor	K.VVDPLAFTDVFVALGPQHR.A	2	5.21	0.59	-3.10
IPI00044707	Hyaluronan and proteoglycan link protein 4 precursor	K.VVDPLAFTDVFVALGPQHR.A	3	3.34	0.46	-4.19
IPI00044707	Hyaluronan and proteoglycan link protein 4 precursor	R.AELQGDGPGDASLVLR.N	2	4.03	0.40	-2.86
IPI00044743	Isoform 1 of Transmembrane protein 132B	K.FSSLPAYLPTNLHISNAEESFFLK.E	3	2.77	0.07	-7.87
IPI00044743	Isoform 1 of Transmembrane protein 132B	K.IAQLQDGR.T	2	2.68	0.17	-0.94
IPI00044743	Isoform 1 of Transmembrane protein 132B	R.IKAAAGVK.I	1	1.79	0.14	
IPI00044743	Isoform 1 of Transmembrane protein 132B	R.TLAGREPGITTVQVLSPLSDSILAEK.T	3	4.71	0.37	-3.18

IPI00044743	Isoform 1 of Transmembrane protein 132B	R.VEPFFIYR.A	2	1.80	0.22	-1.99
IPI00044751	Isoform 1 of M-phase phosphoprotein 1	K.EIVKASSKKSHQIEELEQQIEK.L	3	2.94	0.18	
IPI00044842	Isoform 2 of RAB3A-interacting protein	K.ASAVECGGPK.K	2	2.04	0.13	1.44
IPI00045219	Sorting nexin-18	R.APEPGPAGDGGPGAPAR.Y	2	2.50	0.22	-4.27
IPI00045360	Capicua-like protein/double homeodomain 4 fusion protein	K.VRPPPLKK.T	2	1.84	0.16	
IPI00045511	Isoform 1 of Chloride channel CLIC-like protein 1 precursor	K.IDECEKK.K	2	1.67	0.06	-1.04
IPI00045536	Isoform 3 of Chitinase domain-containing protein 1 precursor	K.FTQISPVWLQLK.R	2	3.96	0.33	-3.05
IPI00045536	Isoform 3 of Chitinase domain-containing protein 1 precursor	K.SQFSDKPVQDR.G	2	3.08	0.34	-3.69
IPI00045839	Isoform 3 of Prolyl 3-hydroxylase 1 precursor	R.SPYNYLQVAYFKINK.L	2	2.95	0.08	
IPI00045841	Isoform 1 of Low-density lipoprotein receptor-related protein 11 precursor	K.AGQDVVLHLPTDGVVLDGR.E	2	4.54	0.53	-2.62
IPI00045841	Isoform 1 of Low-density lipoprotein receptor-related protein 11 precursor	K.AGQDVVLHLPTDGVVLDGR.E	3	5.08	0.45	-2.06
IPI00045841	Isoform 1 of Low-density lipoprotein receptor-related protein 11 precursor	K.FALHSGYSSYSLSR.A	2	3.68	0.44	-2.94
IPI00045841	Isoform 1 of Low-density lipoprotein receptor-related protein 11 precursor	K.FALHSGYSSYSLSR.A	3	4.02	0.14	-1.49
IPI00045841	Isoform 1 of Low-density lipoprotein receptor-related protein 11 precursor	R.AAYSTGGCLHTCSR.Y	2	2.52	0.30	
IPI00045841	Isoform 1 of Low-density lipoprotein receptor-related protein 11 precursor	R.APDGAALATAR.A	2	3.20	0.28	-2.43
IPI00045841	Isoform 1 of Low-density lipoprotein receptor-related protein 11 precursor	R.CSVAVVELPR.R	2	2.78	0.31	-1.52
IPI00045841	Isoform 1 of Low-density lipoprotein receptor-related protein 11 precursor	R.TKDSLAAGASFLR.A	2	3.18	0.31	-3.68
IPI00045841	Isoform 1 of Low-density lipoprotein receptor-related protein 11 precursor	R.TTGPSEDAGGDSLVEK.S	2	4.31	0.37	-2.31
IPI00045928	Sodium/hydrogen exchanger 7	K.SLSCTQEDRAFSTLLVNVSGKFFEYTLKGEISPGK.I	3	3.57	0.07	
IPI00045939	2-aminoethanethiol dioxygenase	R.DCHYYRVLEPVRPKEASSSACDLPR.E	4	2.30	0.13	0.68
IPI00046057	Isoform 2 of Syntaxin-binding protein 1	R.VKEVLLDEDDDLWIALR.H	2	5.66	0.51	-2.73
IPI00056314	Pre-rRNA-processing protein TSR2 homolog	R.EM*ASCITQR.K	2	2.28	0.25	-2.58
IPI00056357	Uncharacterized protein C19orf10 precursor	K.SYLYFTQFK.A	1	2.41	0.32	-3.42
IPI00056357	Uncharacterized protein C19orf10 precursor	K.SYLYFTQFK.A	2	2.99	0.36	-3.24
IPI00056357	Uncharacterized protein C19orf10 precursor	K.TAVAHRPGAFK.A	2	3.24	0.31	-2.88
IPI00056357	Uncharacterized protein C19orf10 precursor	K.TAVAHRPGAFKAELSK.L	3	2.18	0.11	-3.50
IPI00056357	Uncharacterized protein C19orf10 precursor	R.ESDVPLKTEEFEVTK.T	2	3.92	0.50	-4.40
IPI00056357	Uncharacterized protein C19orf10 precursor	R.GAEIEYAM*AYSK.A	2	4.16	0.45	-2.59

IPI00056478	Isoform 1 of Immunoglobulin superfamily member 8 precursor	A.REVLVPEGPLYR.V	2	3.79	0.38	-3.62
IPI00056478	Isoform 1 of Immunoglobulin superfamily member 8 precursor	K.DTQFSYAVFK.S	1	2.84	0.35	-4.47
IPI00056478	Isoform 1 of Immunoglobulin superfamily member 8 precursor	K.DTQFSYAVFK.S	2	3.82	0.36	-3.21
IPI00056478	Isoform 1 of Immunoglobulin superfamily member 8 precursor	K.SRVVAGEVQVQR.L	2	4.09	0.39	-3.26
IPI00056478	Isoform 1 of Immunoglobulin superfamily member 8 precursor	K.SRVVAGEVQVQR.L	3	3.54	0.10	-2.43
IPI00056478	Isoform 1 of Immunoglobulin superfamily member 8 precursor	R.LEAARPGDAGTYR.C	2	2.41	0.19	-1.13
IPI00056478	Isoform 1 of Immunoglobulin superfamily member 8 precursor	R.LQAQDAGIYECHTPSTDTR.Y	2	4.41	0.46	-2.86
IPI00056478	Isoform 1 of Immunoglobulin superfamily member 8 precursor	R.LQAQDAGIYECHTPSTDTR.Y	3	2.75	0.28	-2.44
IPI00056478	Isoform 1 of Immunoglobulin superfamily member 8 precursor	R.LQGDAVVLK.I	1	1.92	0.20	-3.56
IPI00056478	Isoform 1 of Immunoglobulin superfamily member 8 precursor	R.LRLEAARPGDAGTYR.C	2	2.33	0.22	-2.62
IPI00056478	Isoform 1 of Immunoglobulin superfamily member 8 precursor	R.LRLEAARPGDAGTYR.C	3	2.43	0.05	-2.90
IPI00056478	Isoform 1 of Immunoglobulin superfamily member 8 precursor	R.M*TVHEGQELALGCLAR.T	2	4.30	0.47	-2.78
IPI00056478	Isoform 1 of Immunoglobulin superfamily member 8 precursor	R.M*TVHEGQELALGCLAR.T	3	2.91	0.28	-1.13
IPI00056478	Isoform 1 of Immunoglobulin superfamily member 8 precursor	R.SDLAVEAGAPYAER.L	2	4.28	0.48	-3.02
IPI00056478	Isoform 1 of Immunoglobulin superfamily member 8 precursor	R.STLQEVVGIR.S	1	2.05	0.20	-2.97
IPI00056478	Isoform 1 of Immunoglobulin superfamily member 8 precursor	R.STLQEVVGIR.S	2	3.76	0.30	-2.93
IPI00056478	Isoform 1 of Immunoglobulin superfamily member 8 precursor	R.VLPDVLQVSAAPPGPR.G	2	2.04	0.33	-2.84
IPI00056478	Isoform 1 of Immunoglobulin superfamily member 8 precursor	R.VLPDVLQVSAAPPGPR.G	3	3.70	0.24	-2.30
IPI00056478	Isoform 1 of Immunoglobulin superfamily member 8 precursor	R.VVAGEVQVQR.L	2	3.78	0.28	-3.62
IPI00056478	Isoform 1 of Immunoglobulin superfamily member 8 precursor	R.YLGSYSGK.V	1	1.86	0.31	-1.93
IPI00056478	Isoform 1 of Immunoglobulin superfamily member 8 precursor	R.YLGSYSGKVELR.V	2	2.85	0.29	-3.33

IPI00056478	Isoform 1 of Immunoglobulin superfamily member 8 precursor	W.FLYRPEAPDTALGIVSTK.D	3	3.88	0.33	-1.92
IPI00059164	Galactose-3-O-sulfotransferase 3	R.ARPEPVLDNPPPRPIR.V	3	2.85	0.21	-3.36
IPI00059395	Kinesin-like protein KIFC2	R.SPPTRARPPAPLARRSPRGRRISGR.Q	3	3.45	0.17	
IPI00060146	Isoform 1 of Smith-Magenis syndrome chromosome region candidate gene 7 protein	K.RFIDRATSPRDEDDTKADSWK.E	2	2.13	0.09	2.47
IPI00060265	Zinc finger protein 775	R.FSQKPNLTRHLR.N	2	2.09	0.19	
IPI00060308	Isoform 6 of PDZ and LIM domain protein 7	R.ILAHLTGTEFM*QDPDEEHLKKSREK.Y	3	2.34	0.05	-4.65
IPI00060310	Phospholipase D4	K.FWVVDGR.H	1	1.86	0.12	-2.18
IPI00060310	Phospholipase D4	K.TFQTYWVLGVPK.A	2	4.13	0.38	-3.68
IPI00060310	Phospholipase D4	R.FQPFHGLFDGVPTTAYFSASPPALCPQGR.T	3	3.79	0.31	
IPI00060310	Phospholipase D4	R.LLVGCGLNTDPTM*FPYLR.S	2	4.37	0.42	-4.81
IPI00060310	Phospholipase D4	R.LLVGCGLNTDPTM*FPYLR.S	3	3.10	0.31	-4.46
IPI00060310	Phospholipase D4	R.TSTDLQVLAAR.G	2	3.95	0.33	-3.15
IPI00060310	Phospholipase D4	R.YWPVLDNALR.A	2	2.08	0.13	-4.17
IPI00060546	Uncharacterized protein C10orf35	R.LGAAQSPFNDLNR.Q	2	3.94	0.35	-3.26
IPI00060715	BTB/POZ domain-containing protein KCTD12	R.DLQLVLPDYFPER.S	2	3.21	0.34	-5.31
IPI00060715	BTB/POZ domain-containing protein KCTD12	R.LGAPQQPGPGPPPSR.R	2	3.15	0.41	-3.95
IPI00060715	BTB/POZ domain-containing protein KCTD12	R.RLGAPQQPGPGPPPSR.R	3	3.49	0.21	-2.22
IPI00060715	BTB/POZ domain-containing protein KCTD12	R.SGYITIGYR.G	2	2.05	0.17	-1.55
IPI00060715	BTB/POZ domain-containing protein KCTD12	R.SPSGGAAGPLLTPSQSLDGSRR.S	3	3.46	0.34	-2.47
IPI00060715	BTB/POZ domain-containing protein KCTD12	R.YILDYLR.D	2	2.27	0.14	-1.81
IPI00061354	Isoform 2 of Bromodomain adjacent to zinc finger domain protein 2B	R.KLQAQEIAR.Q	1	2.23	0.05	
IPI00061354	Isoform 2 of Bromodomain adjacent to zinc finger domain protein 2B	R.QAAQIKLLRKLQKQEQARVAKEAKKQQAIM*AAEEK.R	3	3.71	0.18	
IPI00061448	13 kDa protein	R.TIAVLLDDILQR.L	2	4.50	0.35	-4.05
IPI00061507	Isoform 3 of Ester hydrolase C11orf54	R.IAEVGGVPYLLPLVNQK.K	2	2.56	0.28	-3.28
IPI00061520	hypothetical protein LOC84752	R.GAGSGGADEVGEGAR.T	2	3.46	0.36	-2.55
IPI00061520	hypothetical protein LOC84752	R.VFLLGVPR.G	2	2.50	0.13	-0.67
IPI00061977	IGHA1 protein	K.GTTVTVSSASPTSPK.V	1	2.33	0.24	
IPI00061977	IGHA1 protein	K.SAVQGPPDR.D	2	2.59	0.20	
IPI00061977	IGHA1 protein	K.SAVQGPPDRDLCGCYSVSSVLSGCAEPWNHGK.T	3	5.00	0.30	
IPI00061977	IGHA1 protein	K.SGNTFRPEVHLLPPPSEELALNELVTLTCLAR.G	2	4.32	0.41	
IPI00061977	IGHA1 protein	K.SGNTFRPEVHLLPPPSEELALNELVTLTCLAR.G	3	6.86	0.60	
IPI00061977	IGHA1 protein	K.TFTCTAAYPESK.T	1	2.27	0.26	
IPI00061977	IGHA1 protein	K.TFTCTAAYPESK.T	2	4.10	0.40	
IPI00061977	IGHA1 protein	K.TFTCTAAYPESKTPLTATLSK.S	2	4.13	0.39	
IPI00061977	IGHA1 protein	K.TFTCTAAYPESKTPLTATLSK.S	3	4.01	0.44	
IPI00061977	IGHA1 protein	K.TPLTATLSK.S	1	2.18	0.20	
IPI00061977	IGHA1 protein	K.TPLTATLSK.S	2	2.50	0.14	
IPI00061977	IGHA1 protein	K.VFPLSLCSTQPDGNVVIACLVQGFFPQEPLSVTWSESGQGVTAR.N	3	3.85	0.24	

IPI00061977	IGHA1 protein	K.YLTWASR.Q	1	1.98	0.18	
IPI00061977	IGHA1 protein	K.YLTWASR.Q	2	1.93	0.24	
IPI00061977	IGHA1 protein	Q.EPSQGTTTFAVTSILR.V	2	3.82	0.43	-5.84
IPI00061977	IGHA1 protein	R.DASGVTFTWTPSSGK.S	1	3.53	0.45	
IPI00061977	IGHA1 protein	R.DASGVTFTWTPSSGK.S	2	5.30	0.49	
IPI00061977	IGHA1 protein	R.EKYLTWASR.Q	1	2.49	0.27	
IPI00061977	IGHA1 protein	R.GFSPKDVLVR.W	2	2.81	0.13	
IPI00061977	IGHA1 protein	R.NFPPSQDASGDLYTTSSQLTLPATQCLAGK.S	2	4.34	0.48	
IPI00061977	IGHA1 protein	R.NFPPSQDASGDLYTTSSQLTLPATQCLAGK.S	3	5.77	0.57	
IPI00061977	IGHA1 protein	R.QEPSQGTTTFAVTSILR.V	2	4.27	0.52	
IPI00061977	IGHA1 protein	R.QEPSQGTTTFAVTSILR.V	3	4.05	0.27	
IPI00061977	IGHA1 protein	R.SDDTAVYYCAR.R	2	3.57	0.35	
IPI00061977	IGHA1 protein	R.SLRSDDTAVYYCAR.R	2	4.54	0.26	
IPI00061977	IGHA1 protein	R.VAAEDWK.K	2	2.23	0.16	
IPI00061977	IGHA1 protein	R.WLQGSQELPR.E	1	3.00	0.19	
IPI00061977	IGHA1 protein	R.WLQGSQELPR.E	2	3.80	0.33	
IPI00061977	IGHA1 protein	R.WLQGSQELPREK.Y	2	2.71	0.15	
IPI00062037	Dynein light chain 2, cytoplasmic	K.YNIEKDIAAYIK.K	2	4.01	0.37	-3.32
IPI00062037	Dynein light chain 2, cytoplasmic	K.YNIEKDIAAYIK.K	3	3.44	0.28	-2.04
IPI00062037	Dynein light chain 2, cytoplasmic	K.YNIEKDIAAYIKK.E	2	4.14	0.33	-3.18
IPI00062037	Dynein light chain 2, cytoplasmic	K.YNIEKDIAAYIKK.E	3	3.85	0.34	-2.24
IPI00062037	Dynein light chain 2, cytoplasmic	R.NFGSYVTHETK.H	2	2.51	0.18	-2.18
IPI00062730	Uncharacterized protein C16orf45	-.M*ELKQSLSTHLEAEKPLR.R	2	2.67	0.10	
IPI00063048	Isoform 2 of Beta-galactoside alpha-2,6-sialyltransferase 2	K.EFFSSQVGR.K	1	2.89	0.26	-2.35
IPI00063048	Isoform 2 of Beta-galactoside alpha-2,6-sialyltransferase 2	K.EFFSSQVGR.K	2	2.11	0.11	-2.19
IPI00063048	Isoform 2 of Beta-galactoside alpha-2,6-sialyltransferase 2	R.AHPAGSFHAGPGDLQK.W	2	4.38	0.57	-4.76
IPI00063048	Isoform 2 of Beta-galactoside alpha-2,6-sialyltransferase 2	R.AHPAGSFHAGPGDLQK.W	3	2.24	0.17	-2.03
IPI00063048	Isoform 2 of Beta-galactoside alpha-2,6-sialyltransferase 2	R.EGAFPAAQVQR.R	1	2.53	0.27	-3.54
IPI00063048	Isoform 2 of Beta-galactoside alpha-2,6-sialyltransferase 2	R.EGAFPAAQVQR.R	2	3.43	0.28	-3.65
IPI00063048	Isoform 2 of Beta-galactoside alpha-2,6-sialyltransferase 2	R.LLPVQGK.Q	1	1.65	0.11	-1.90
IPI00063048	Isoform 2 of Beta-galactoside alpha-2,6-sialyltransferase 2	R.RLLPVQGK.Q	2	2.55	0.19	-5.02
IPI00063827	Isoform 1 of Abhydrolase domain-containing protein 14B	K.TPALIVYGDQDPM*GQTSFEHLK.Q	3	3.29	0.42	-1.54
IPI00064241	Isoform 1 of Zinc finger protein Eos	-.MHTPPALPRRFQGGGR.V	2	2.23	0.06	-6.19

IPI00064296	PRO0633	R.DIM*LYLQRAINTAM*YHIMM*FQLVK.D	3	2.61	0.11	-0.92
IPI00064377	Tumor necrosis factor receptor superfamily member 19L precursor	R.GVEVAAGASSGGETR.Q	2	3.89	0.46	-3.10
IPI00064377	Tumor necrosis factor receptor superfamily member 19L precursor	R.RLEAQVGM*ATR.D	3	3.27	0.21	0.14
IPI00064607	Isoform 1 of Multiple epidermal growth factor-like domains 10 precursor	K.CELPCQDGTYGLNCAER.C	2	5.20	0.49	-4.18
IPI00064607	Isoform 1 of Multiple epidermal growth factor-like domains 10 precursor	K.CTPGWTGLYCTQR.C	2	3.84	0.51	-2.78
IPI00064607	Isoform 1 of Multiple epidermal growth factor-like domains 10 precursor	K.CYHVSGACLCEAGFAGER.C	3	2.49	0.12	-3.38
IPI00064607	Isoform 1 of Multiple epidermal growth factor-like domains 10 precursor	K.NDAVCSPVDGSCTCK.A	2	3.90	0.53	-4.20
IPI00064607	Isoform 1 of Multiple epidermal growth factor-like domains 10 precursor	K.NGASCSPDDGICECAPGFR.G	2	5.48	0.63	-4.65
IPI00064607	Isoform 1 of Multiple epidermal growth factor-like domains 10 precursor	K.NGASCSPDDGICECAPGFR.G	3	4.64	0.51	-3.90
IPI00064607	Isoform 1 of Multiple epidermal growth factor-like domains 10 precursor	R.CDQAGVIIVGNLNSLSR.T	2	5.32	0.49	-3.64
IPI00064607	Isoform 1 of Multiple epidermal growth factor-like domains 10 precursor	R.CPPGYTGAFCEDLCPPGK.H	2	4.55	0.57	-3.08
IPI00064607	Isoform 1 of Multiple epidermal growth factor-like domains 10 precursor	R.CQDECPVGTYGVLCAETCQCVNGGK.C	3	7.55	0.59	-3.21
IPI00064607	Isoform 1 of Multiple epidermal growth factor-like domains 10 precursor	R.LCPEGLYGIK.C	1	1.69	0.11	-1.72
IPI00064607	Isoform 1 of Multiple epidermal growth factor-like domains 10 precursor	R.LCPEGLYGIK.C	2	1.58	0.11	-2.82
IPI00064652	vascular endothelial growth factor A isoform e precursor	K.FM*DVYQR.S	2	1.79	0.12	-1.57
IPI00064652	vascular endothelial growth factor A isoform e precursor	R.GAEESGPPHSPSR.R	2	2.06	0.19	
IPI00064667	Beta-Ala-His dipeptidase precursor	I.PLGAVDDGEHSQNEK.I	2	4.78	0.61	-3.30
IPI00064667	Beta-Ala-His dipeptidase precursor	I.PPVILAELGSDPTK.G	1	3.51	0.37	-1.03
IPI00064667	Beta-Ala-His dipeptidase precursor	I.PPVILAELGSDPTK.G	2	4.27	0.45	-2.47
IPI00064667	Beta-Ala-His dipeptidase precursor	K.AIHLDLEEYR.N	1	2.84	0.32	-3.63
IPI00064667	Beta-Ala-His dipeptidase precursor	K.AIHLDLEEYR.N	2	2.92	0.39	-3.42
IPI00064667	Beta-Ala-His dipeptidase precursor	K.AIHLDLEEYR.N	3	2.87	0.11	-3.83
IPI00064667	Beta-Ala-His dipeptidase precursor	K.EEILM*HLWR.Y	2	2.39	0.20	-2.58
IPI00064667	Beta-Ala-His dipeptidase precursor	K.EWVAIESDSVQPVPR.F	1	1.25	0.21	-2.88
IPI00064667	Beta-Ala-His dipeptidase precursor	K.EWVAIESDSVQPVPR.F	2	5.69	0.52	-5.76
IPI00064667	Beta-Ala-His dipeptidase precursor	K.EWVAIESDSVQPVPR.F	3	4.38	0.27	-2.51
IPI00064667	Beta-Ala-His dipeptidase precursor	K.FIIEGM*EEAGSVALEELVEK.E	2	4.51	0.22	-3.80

IPI00064667	Beta-Ala-His dipeptidase precursor	K.FIIEGM*EEAGSVALEELVEK.E	3	4.44	0.27	-5.24
IPI00064667	Beta-Ala-His dipeptidase precursor	K.FIIEGM*EEAGSVALEELVEKEK.D	3	2.85	0.14	-2.18
IPI00064667	Beta-Ala-His dipeptidase precursor	K.FIIEGM*EEAGSVALEELVEKEKDR.F	3	3.26	0.21	-4.04
IPI00064667	Beta-Ala-His dipeptidase precursor	K.FIIEGM*EEAGSVALEELVEKEKDR.F	4	3.64	0.37	-5.26
IPI00064667	Beta-Ala-His dipeptidase precursor	K.FLFDTKEEILM*HLWR.Y	3	3.06	0.27	-3.07
IPI00064667	Beta-Ala-His dipeptidase precursor	K.GPVLAWINAVSAFR.A	2	5.16	0.52	-5.14
IPI00064667	Beta-Ala-His dipeptidase precursor	K.GPVLAWINAVSAFR.A	3	3.74	0.33	-2.81
IPI00064667	Beta-Ala-His dipeptidase precursor	K.GTVCFYGHLDVQPADR.G	3	3.48	0.29	-1.99
IPI00064667	Beta-Ala-His dipeptidase precursor	K.GTVCFYGHLDVQPADRGDGWLTDPYVLTEVDGK.L	3	5.12	0.41	-4.07
IPI00064667	Beta-Ala-His dipeptidase precursor	K.GTVCFYGHLDVQPADRGDGWLTDPYVLTEVDGK.L	4	5.59	0.50	-5.61
IPI00064667	Beta-Ala-His dipeptidase precursor	K.LFAAFFLEM*AQLH.-	2	5.26	0.60	-7.97
IPI00064667	Beta-Ala-His dipeptidase precursor	K.LFAAFFLEM*AQLH.-	3	5.24	0.49	-4.84
IPI00064667	Beta-Ala-His dipeptidase precursor	K.LFAAFFLEMAQLH.-	2	4.88	0.52	-3.75
IPI00064667	Beta-Ala-His dipeptidase precursor	K.LFAAFFLEMAQLH.-	3	4.41	0.34	-1.95
IPI00064667	Beta-Ala-His dipeptidase precursor	K.M*FQEIVHK.S	1	2.26	0.22	-5.37
IPI00064667	Beta-Ala-His dipeptidase precursor	K.M*FQEIVHK.S	2	2.88	0.28	-4.26
IPI00064667	Beta-Ala-His dipeptidase precursor	K.M*VVSM*TLGLHPWIANIDDTQYLAAK.R	2	3.33	0.42	-1.35
IPI00064667	Beta-Ala-His dipeptidase precursor	K.M*VVSM*TLGLHPWIANIDDTQYLAAK.R	3	5.98	0.57	-5.94
IPI00064667	Beta-Ala-His dipeptidase precursor	K.M*VVSM*TLGLHPWIANIDDTQYLAAKR.A	4	3.78	0.28	-2.89
IPI00064667	Beta-Ala-His dipeptidase precursor	K.M*VVSMTLGLHPWIANIDDTQYLAAK.R	3	5.47	0.19	-2.50
IPI00064667	Beta-Ala-His dipeptidase precursor	K.SVVLIPLGAVDDGEHSQNEK.I	2	5.89	0.61	-5.63
IPI00064667	Beta-Ala-His dipeptidase precursor	K.SVVLIPLGAVDDGEHSQNEK.I	3	2.43	0.18	-2.85
IPI00064667	Beta-Ala-His dipeptidase precursor	K.SVVLIPLGAVDDGEHSQNEKINR.W	2	4.18	0.59	-4.14
IPI00064667	Beta-Ala-His dipeptidase precursor	K.SVVLIPLGAVDDGEHSQNEKINR.W	3	2.55	0.26	-2.22
IPI00064667	Beta-Ala-His dipeptidase precursor	K.SVVLIPLGAVDDGEHSQNEKINR.W	4	2.57	0.29	-2.68
IPI00064667	Beta-Ala-His dipeptidase precursor	K.VFQYIDLHQDEFVQTLK.E	2	6.52	0.56	-5.93
IPI00064667	Beta-Ala-His dipeptidase precursor	K.VFQYIDLHQDEFVQTLK.E	3	5.55	0.51	-4.75
IPI00064667	Beta-Ala-His dipeptidase precursor	K.VFQYIDLHQDEFVQTLKEWVAIESDSVQPVPR.F	3	6.72	0.64	-3.27
IPI00064667	Beta-Ala-His dipeptidase precursor	K.VFQYIDLHQDEFVQTLKEWVAIESDSVQPVPR.F	4	4.45	0.39	-3.48
IPI00064667	Beta-Ala-His dipeptidase precursor	L.IPLGAVDDGEHSQNEK.I	2	5.03	0.52	-2.81
IPI00064667	Beta-Ala-His dipeptidase precursor	L.TDPYVLTEVDGK.L	2	3.37	0.41	-0.63
IPI00064667	Beta-Ala-His dipeptidase precursor	M.GPQQLPDGQSLPIPPVILAELGSDPTK.G	3	3.65	0.35	-3.56
IPI00064667	Beta-Ala-His dipeptidase precursor	P.SLSIHGIEGAFDEPGTK.T	2	4.14	0.44	-2.56
IPI00064667	Beta-Ala-His dipeptidase precursor	P.SLSIHGIEGAFDEPGTK.T	3	4.55	0.33	-2.51
IPI00064667	Beta-Ala-His dipeptidase precursor	P.SPPPALLEK.V	1	2.76	0.11	-2.61
IPI00064667	Beta-Ala-His dipeptidase precursor	Q.YIDLHQDEFVQTLK.E	2	4.96	0.43	-2.72
IPI00064667	Beta-Ala-His dipeptidase precursor	R.ALEQDLPVNIK.F	1	3.09	0.27	-4.33
IPI00064667	Beta-Ala-His dipeptidase precursor	R.ALEQDLPVNIK.F	2	3.96	0.29	-3.30
IPI00064667	Beta-Ala-His dipeptidase precursor	R.DGSTIPIAK.M	1	1.97	0.22	-3.24
IPI00064667	Beta-Ala-His dipeptidase precursor	R.DGSTIPIAK.M	2	2.84	0.25	-4.01
IPI00064667	Beta-Ala-His dipeptidase precursor	R.GATDNKGPVLAWINAVSAFR.A	2	5.09	0.58	-6.27
IPI00064667	Beta-Ala-His dipeptidase precursor	R.GATDNKGPVLAWINAVSAFR.A	3	5.25	0.51	-5.12

IPI00064667	Beta-Ala-His dipeptidase precursor	R.GDGWLTDPYVLTEVDGK.L	2	3.47	0.18	-5.11
IPI00064667	Beta-Ala-His dipeptidase precursor	R.GNSYFM*VEVK.C	1	2.32	0.21	-2.22
IPI00064667	Beta-Ala-His dipeptidase precursor	R.GNSYFM*VEVK.C	2	3.83	0.50	-4.07
IPI00064667	Beta-Ala-His dipeptidase precursor	R.GNSYFMVEVK.C	2	3.63	0.34	-1.25
IPI00064667	Beta-Ala-His dipeptidase precursor	R.HLEDVFSK.R	1	2.35	0.20	-2.28
IPI00064667	Beta-Ala-His dipeptidase precursor	R.HLEDVFSK.R	2	3.38	0.27	-2.92
IPI00064667	Beta-Ala-His dipeptidase precursor	R.HLEDVFSKR.N	1	2.73	0.39	-5.51
IPI00064667	Beta-Ala-His dipeptidase precursor	R.HLEDVFSKR.N	2	3.10	0.29	-1.94
IPI00064667	Beta-Ala-His dipeptidase precursor	R.KPAITYGTR.G	1	2.57	0.26	-5.22
IPI00064667	Beta-Ala-His dipeptidase precursor	R.KPAITYGTR.G	2	2.54	0.29	-2.96
IPI00064667	Beta-Ala-His dipeptidase precursor	R.M*M*AVAADTLQR.L	1	1.08	0.17	-3.11
IPI00064667	Beta-Ala-His dipeptidase precursor	R.M*M*AVAADTLQR.L	2	3.98	0.47	-3.72
IPI00064667	Beta-Ala-His dipeptidase precursor	R.TVFGTEPDM*IR.D	2	3.05	0.30	-3.70
IPI00064667	Beta-Ala-His dipeptidase precursor	R.TVFGTEPDM*IRDGSTIPIAK.M	2	3.39	0.49	-4.10
IPI00064667	Beta-Ala-His dipeptidase precursor	R.TVFGTEPDM*IRDGSTIPIAK.M	3	2.51	0.37	-4.16
IPI00064667	Beta-Ala-His dipeptidase precursor	R.VASVDM*GPQQLPDGQSLPIPPVILAELGSDPTK.G	3	5.24	0.44	-6.13
IPI00064667	Beta-Ala-His dipeptidase precursor	R.WNYIEGTK.L	1	2.25	0.21	-2.65
IPI00064667	Beta-Ala-His dipeptidase precursor	R.WNYIEGTK.L	2	2.43	0.21	-1.87
IPI00064667	Beta-Ala-His dipeptidase precursor	R.YPSLSIHGIEGAFDEPGTK.T	2	5.74	0.57	-3.76
IPI00064667	Beta-Ala-His dipeptidase precursor	R.YPSLSIHGIEGAFDEPGTK.T	3	6.05	0.53	-4.31
IPI00064667	Beta-Ala-His dipeptidase precursor	R.YPSLSIHGIEGAFDEPGTKTVIPGR.V	4	3.65	0.34	-3.86
IPI00064667	Beta-Ala-His dipeptidase precursor	S.SPSPPPALLEK.V	1	2.44	0.31	-3.71
IPI00064667	Beta-Ala-His dipeptidase precursor	V.AIESDSVQPVPR.F	2	2.95	0.17	-3.05
IPI00064667	Beta-Ala-His dipeptidase precursor	V.FGTEPDM*IRDGSTIPIAK.M	2	2.97	0.25	-2.46
IPI00064667	Beta-Ala-His dipeptidase precursor	V.FQYIDLHQDEFVQTLK.E	2	5.44	0.54	-2.54
IPI00064667	Beta-Ala-His dipeptidase precursor	V.LIPLGAVDDGEHSQNEK.I	2	4.52	0.50	-3.40
IPI00064667	Beta-Ala-His dipeptidase precursor	V.VLIPLGAVDDGEHSQNEK.I	2	3.16	0.41	-2.72
IPI00064667	Beta-Ala-His dipeptidase precursor	W.IANIDDTQYLAAK.R	2	4.22	0.44	-3.73
IPI00064667	Beta-Ala-His dipeptidase precursor	W.LTDPYVLTEVDGK.L	2	4.27	0.49	-4.25
IPI00064667	Beta-Ala-His dipeptidase precursor	W.NYIEGTK.L	1	2.08	0.18	-4.41
IPI00064667	Beta-Ala-His dipeptidase precursor	W.VAIESDSVQPVPR.F	2	3.65	0.38	-3.22
IPI00064667	Beta-Ala-His dipeptidase precursor	Y.PSLSIHGIEGAFDEPGTK.T	2	4.25	0.45	-2.29
IPI00064667	Beta-Ala-His dipeptidase precursor	Y.PSLSIHGIEGAFDEPGTK.T	3	5.35	0.45	-1.90
IPI00064935	Alpha-protein kinase 3	K.VRAAGDGEATTPEERESPTVSPRGPRKSLVPGSPGTPGR.E	3	3.82	0.12	
IPI00065276	Isoform 2 of Tether containing UBX domain for GLUT4	K.TVLDDHTQTLFQPQLGDR.V	4	2.90	0.19	-7.48
IPI00065312	N-acetyl-beta-glucosaminyl-glycoprotein 4-beta-N-acetylgalactosaminyltransferase 1	R.DLDM*LFPGGAGR.L	2	3.46	0.34	-4.41
IPI00065312	N-acetyl-beta-glucosaminyl-glycoprotein 4-beta-N-acetylgalactosaminyltransferase 1	R.DTFFLTPR.M	2	2.38	0.32	-1.56
IPI00065931	Isoform 2 of A-kinase anchor protein 13	K.SGQM*FAKEDLKRKKLVRDGSVFLK.N	3	3.91	0.15	
IPI00069058	VGF nerve growth factor inducible precursor	A.APPGRPEAQPPPLSSEHKEPVAGDAVPGPK.D	2	3.86	0.56	-3.46

IPI00069058	VGF nerve growth factor inducible precursor	A.APPGRPEAQPPPLSSEHKEPVAGDAVPGPK.D	3	5.74	0.55	-4.83
IPI00069058	VGF nerve growth factor inducible precursor	A.APPGRPEAQPPPLSSEHKEPVAGDAVPGPKDGSAPEVR.G	4	5.62	0.48	-4.09
IPI00069058	VGF nerve growth factor inducible precursor	A.AVLLQALDRPASPPAPSGSQQGPEEEAAEALLTETVR.S	3	6.61	0.57	-2.86
IPI00069058	VGF nerve growth factor inducible precursor	A.DLASDLLLQYLLQGGAR.Q	2	5.03	0.40	-3.41
IPI00069058	VGF nerve growth factor inducible precursor	A.DLASDLLLQYLLQGGAR.Q	3	5.23	0.33	-1.30
IPI00069058	VGF nerve growth factor inducible precursor	A.LAAVLLQALDRPASPPAPSGSQQGPEEEAAEALLTETVR.S	4	4.75	0.41	-4.82
IPI00069058	VGF nerve growth factor inducible precursor	A.LDRPASPPAPSGSQQGPEEEAAEALLTETVR.S	3	6.57	0.52	-4.81
IPI00069058	VGF nerve growth factor inducible precursor	A.PPGRPEAQPPPLSSEHKEPVAGDAVPGPK.D	3	6.01	0.49	-4.55
IPI00069058	VGF nerve growth factor inducible precursor	A.PPGRPEAQPPPLSSEHKEPVAGDAVPGPK.D	4	4.94	0.40	-4.26
IPI00069058	VGF nerve growth factor inducible precursor	A.PPGRPEAQPPPLSSEHKEPVAGDAVPGPKDGSAPEVR.G	4	5.00	0.43	-4.26
IPI00069058	VGF nerve growth factor inducible precursor	A.SDLLLQYLLQGGAR.Q	2	4.18	0.35	-3.86
IPI00069058	VGF nerve growth factor inducible precursor	A.VLLQALDRPASPPAPSGSQQGPEEEAAEALLTETVR.S	4	4.93	0.37	-4.31
IPI00069058	VGF nerve growth factor inducible precursor	D.PSEELEALASLLQELR.D	2	4.82	0.29	-4.73
IPI00069058	VGF nerve growth factor inducible precursor	D.PSEELEALASLLQELR.D	3	4.35	0.13	-2.64
IPI00069058	VGF nerve growth factor inducible precursor	G.RPEAQPPPLSSEHKEPVAGDAVPGPK.D	3	5.38	0.49	-3.83
IPI00069058	VGF nerve growth factor inducible precursor	G.RPEAQPPPLSSEHKEPVAGDAVPGPK.D	4	6.17	0.57	-4.29
IPI00069058	VGF nerve growth factor inducible precursor	H.SLPAPESPEPAAPPRPQTPENGPEASDPSEELEALASLLQELR.D	3	4.87	0.57	-4.68
IPI00069058	VGF nerve growth factor inducible precursor	H.SLPAPESPEPAAPPRPQTPENGPEASDPSEELEALASLLQELR.D	4	4.84	0.38	-2.95
IPI00069058	VGF nerve growth factor inducible precursor	K.ARRPESALLGGSEAGER.L	2	3.02	0.21	-4.02
IPI00069058	VGF nerve growth factor inducible precursor	K.AYQGVAAPFPK.A	1	2.71	0.37	-2.95
IPI00069058	VGF nerve growth factor inducible precursor	K.AYQGVAAPFPK.A	2	3.94	0.38	-2.88
IPI00069058	VGF nerve growth factor inducible precursor	K.AYQGVAAPFPKA.R	1	2.62	0.34	-1.51
IPI00069058	VGF nerve growth factor inducible precursor	K.AYQGVAAPFPKA.R	2	4.51	0.44	-1.69
IPI00069058	VGF nerve growth factor inducible precursor	K.DGSAPEVR.G	1	1.55	0.19	-2.72
IPI00069058	VGF nerve growth factor inducible precursor	K.DGSAPEVR.G	2	2.38	0.22	-3.92
IPI00069058	VGF nerve growth factor inducible precursor	K.FGEGVSSPK.T	1	1.94	0.11	-3.61
IPI00069058	VGF nerve growth factor inducible precursor	K.FGEGVSSPK.T	2	3.60	0.25	-3.25
IPI00069058	VGF nerve growth factor inducible precursor	K.KNAPPEPVPPPR.A	2	2.65	0.40	-1.10
IPI00069058	VGF nerve growth factor inducible precursor	K.LHLPADDVVS.I	1	1.85	0.28	-1.93
IPI00069058	VGF nerve growth factor inducible precursor	K.LHLPADDVVSIIEEV.E	2	3.09	0.26	-5.65
IPI00069058	VGF nerve growth factor inducible precursor	K.LHLPADDVVSIIEEVE.E	2	3.80	0.34	-5.08
IPI00069058	VGF nerve growth factor inducible precursor	K.LHLPADDVVSIIEEVEE.K	2	4.79	0.44	-8.31
IPI00069058	VGF nerve growth factor inducible precursor	K.LHLPADDVVSIIEEVEE.K	3	3.67	0.31	-1.97
IPI00069058	VGF nerve growth factor inducible precursor	K.LHLPADDVVSIIEEVEEK.R	3	3.95	0.31	-4.91
IPI00069058	VGF nerve growth factor inducible precursor	K.NAPPEPVPPPR.A	1	2.34	0.28	-3.43
IPI00069058	VGF nerve growth factor inducible precursor	K.NAPPEPVPPPR.A	2	2.05	0.20	-3.21
IPI00069058	VGF nerve growth factor inducible precursor	K.RQQETAAAETETR.T	2	3.93	0.33	-2.09
IPI00069058	VGF nerve growth factor inducible precursor	K.THLGEALAPLSK.A	1	3.32	0.34	-3.16
IPI00069058	VGF nerve growth factor inducible precursor	K.THLGEALAPLSK.A	2	3.55	0.48	-2.99
IPI00069058	VGF nerve growth factor inducible precursor	K.THLGEALAPLSK.A	3	3.56	0.08	-4.04
IPI00069058	VGF nerve growth factor inducible precursor	K.THLGEALAPLSKA.Y	2	3.28	0.43	-3.50
IPI00069058	VGF nerve growth factor inducible precursor	L.ADLASDLLLQYLLQGGAR.Q	3	5.32	0.37	-4.14

IPI00069058	VGF nerve growth factor inducible precursor	L.ASDLLLQYLLQGGAR.Q	2	4.86	0.34	-3.64
IPI00069058	VGF nerve growth factor inducible precursor	L.DRPASPPAPSGSQQGPEEEAAEALLTETVR.S	3	5.88	0.49	-4.28
IPI00069058	VGF nerve growth factor inducible precursor	L.LQALDRPASPPAPSGSQQGPEEEAAEALLTETVR.S	3	5.38	0.55	-4.09
IPI00069058	VGF nerve growth factor inducible precursor	L.SKAYQGVAAPFPK.A	2	4.07	0.39	-3.01
IPI00069058	VGF nerve growth factor inducible precursor	N.GPEASDPSEELEALASLLQELR.D	2	5.41	0.53	-5.31
IPI00069058	VGF nerve growth factor inducible precursor	N.GPEASDPSEELEALASLLQELR.D	3	4.09	0.30	-5.25
IPI00069058	VGF nerve growth factor inducible precursor	N.SEPQDEGELFQGVDPR.A	2	4.65	0.35	-5.02
IPI00069058	VGF nerve growth factor inducible precursor	N.SEPQDEGELFQGVDPR.A	3	3.82	0.35	-3.16
IPI00069058	VGF nerve growth factor inducible precursor	P.DSGPLPETHKFGEGVSSPK.T	2	3.82	0.49	-3.92
IPI00069058	VGF nerve growth factor inducible precursor	P.EASDPSEELEALASLLQELR.D	2	4.94	0.43	-2.85
IPI00069058	VGF nerve growth factor inducible precursor	P.ERAPLPPPAPSQFQAR.M	3	4.67	0.33	-2.23
IPI00069058	VGF nerve growth factor inducible precursor	P.ETHKFGEGVSSPK.T	2	3.16	0.28	-3.92
IPI00069058	VGF nerve growth factor inducible precursor	P.GRPEAQPPPLSSEHKEPVAGDAVPGPK.D	2	4.22	0.51	-3.59
IPI00069058	VGF nerve growth factor inducible precursor	P.GRPEAQPPPLSSEHKEPVAGDAVPGPK.D	3	5.49	0.44	-4.07
IPI00069058	VGF nerve growth factor inducible precursor	P.GRPEAQPPPLSSEHKEPVAGDAVPGPK.D	4	6.54	0.53	-3.66
IPI00069058	VGF nerve growth factor inducible precursor	P.GRPEAQPPPLSSEHKEPVAGDAVPGPKDGSAPEVR.G	3	6.80	0.58	0.47
IPI00069058	VGF nerve growth factor inducible precursor	P.GRPEAQPPPLSSEHKEPVAGDAVPGPKDGSAPEVR.G	4	5.08	0.51	-2.47
IPI00069058	VGF nerve growth factor inducible precursor	P.PGRPEAQPPPLSSEHKEPVAGDAVPGPK.D	3	5.89	0.53	-4.37
IPI00069058	VGF nerve growth factor inducible precursor	P.PGRPEAQPPPLSSEHKEPVAGDAVPGPK.D	4	6.32	0.51	-3.60
IPI00069058	VGF nerve growth factor inducible precursor	P.PLSSEHKEPVAGDAVPGPK.D	2	4.44	0.46	-2.70
IPI00069058	VGF nerve growth factor inducible precursor	P.PPLSSEHKEPVAGDAVPGPK.D	2	4.43	0.51	-3.12
IPI00069058	VGF nerve growth factor inducible precursor	P.PPLSSEHKEPVAGDAVPGPK.D	3	4.50	0.29	-2.42
IPI00069058	VGF nerve growth factor inducible precursor	Q.ALDRPASPPAPSGSQQGPEEEAAEALLTETVR.S	3	6.00	0.54	-3.02
IPI00069058	VGF nerve growth factor inducible precursor	Q.ARVPERAPLPPPAPSQFQAR.M	3	4.85	0.36	-4.04
IPI00069058	VGF nerve growth factor inducible precursor	Q.DEGELFQGVDPR.A	2	3.61	0.27	-2.97
IPI00069058	VGF nerve growth factor inducible precursor	Q.TPENGPEASDPSEELEALASLLQELR.D	3	3.81	0.26	-2.64
IPI00069058	VGF nerve growth factor inducible precursor	R.AAPAPTHVR.S	1	1.54	0.15	-3.29
IPI00069058	VGF nerve growth factor inducible precursor	R.AAPAPTHVR.S	2	1.83	0.44	-2.96
IPI00069058	VGF nerve growth factor inducible precursor	R.ALAAVLLQALDR.P	2	3.93	0.40	-3.61
IPI00069058	VGF nerve growth factor inducible precursor	R.ALAAVLLQALDRPASPPAPSGSQQGPEEEAAEALLTET.V	3	3.80	0.40	-2.63
IPI00069058	VGF nerve growth factor inducible precursor	R.ALAAVLLQALDRPASPPAPSGSQQGPEEEAAEALLTETV.R	3	6.07	0.58	-4.30
IPI00069058	VGF nerve growth factor inducible precursor	R.ALAAVLLQALDRPASPPAPSGSQQGPEEEAAEALLTETV.R	4	5.00	0.36	-3.96
IPI00069058	VGF nerve growth factor inducible precursor	R.ALAAVLLQALDRPASPPAPSGSQQGPEEEAAEALLTETVR.S	3	6.50	0.61	-4.77
IPI00069058	VGF nerve growth factor inducible precursor	R.ALAAVLLQALDRPASPPAPSGSQQGPEEEAAEALLTETVR.S	4	5.11	0.53	-6.79
IPI00069058	VGF nerve growth factor inducible precursor	R.ALAAVLLQALDRPASPPAPSGSQQGPEEEAAEALLTETVR.S	5	5.50	0.50	-5.50
IPI00069058	VGF nerve growth factor inducible precursor	R.ALAAVLLQALDRPASPPAPSGSQQGPEEEAAEALLTETVR.S	6	4.34	0.31	-4.41
IPI00069058	VGF nerve growth factor inducible precursor	R.APLPPPAPSQFQAR.M	2	2.48	0.28	-5.81
IPI00069058	VGF nerve growth factor inducible precursor	R.ARQNALLFAEEEDGEAGA.E	2	5.01	0.60	-3.43
IPI00069058	VGF nerve growth factor inducible precursor	R.ARQNALLFAEEEDGEAGAE.D	2	4.24	0.49	1.05
IPI00069058	VGF nerve growth factor inducible precursor	R.ARQNALLFAEEEDGEAGAED.K	2	6.07	0.55	-3.37
IPI00069058	VGF nerve growth factor inducible precursor	R.ASWGEFQAR.V	2	2.95	0.27	-2.89
IPI00069058	VGF nerve growth factor inducible precursor	R.DFSPSSAK.R	1	1.73	0.18	-3.28

IPI00069058	VGF nerve growth factor inducible precursor	R.DFSPSSAKR.Q	2	1.87	0.16	-2.23
IPI00069058	VGF nerve growth factor inducible precursor	R.ESAREEEEAEQERR.G	3	3.46	0.23	-3.27
IPI00069058	VGF nerve growth factor inducible precursor	R.GGEERVGEEDEEAAEAEAEAEEAER.A	3	5.09	0.51	-2.98
IPI00069058	VGF nerve growth factor inducible precursor	R.GGEERVGEEDEEAAEAEAEAEEAERAR.Q	3	5.96	0.46	-1.42
IPI00069058	VGF nerve growth factor inducible precursor	R.GGEERVGEEDEEAAEAEAEAEEAERAR.Q	4	4.99	0.43	-2.80
IPI00069058	VGF nerve growth factor inducible precursor	R.GLQEAAEER.E	1	2.13	0.07	-2.29
IPI00069058	VGF nerve growth factor inducible precursor	R.GLQEAAEER.E	2	2.42	0.07	-0.27
IPI00069058	VGF nerve growth factor inducible precursor	R.GLQEAAEERESAREEEEAEQE.R	2	2.98	0.37	-3.30
IPI00069058	VGF nerve growth factor inducible precursor	R.GLQEAAEERESAREEEEAEQE.R	3	3.62	0.33	0.13
IPI00069058	VGF nerve growth factor inducible precursor	R.GLQEAAEERESAREEEEAEQER.R	3	3.92	0.28	-3.12
IPI00069058	VGF nerve growth factor inducible precursor	R.GLQEAAEERESAREEEEAEQERR.G	3	3.99	0.28	-3.68
IPI00069058	VGF nerve growth factor inducible precursor	R.GLQEAAEERESAREEEEAEQERR.G	4	2.81	0.14	-4.23
IPI00069058	VGF nerve growth factor inducible precursor	R.KKNAPPEPVPPPR.A	2	3.53	0.32	-4.07
IPI00069058	VGF nerve growth factor inducible precursor	R.LADLASDLLLQYLLQGGAR.Q	2	5.69	0.42	-7.37
IPI00069058	VGF nerve growth factor inducible precursor	R.LADLASDLLLQYLLQGGAR.Q	3	5.87	0.41	-4.13
IPI00069058	VGF nerve growth factor inducible precursor	R.LADLASDLLLQYLLQGGARQ.R	2	4.47	0.33	-4.21
IPI00069058	VGF nerve growth factor inducible precursor	R.LADLASDLLLQYLLQGGARQ.R	3	5.00	0.41	-4.42
IPI00069058	VGF nerve growth factor inducible precursor	R.LLQQGLAQVEAG.R	2	3.38	0.25	-3.87
IPI00069058	VGF nerve growth factor inducible precursor	R.LLQQGLAQVEAGR.R	2	3.90	0.42	-3.05
IPI00069058	VGF nerve growth factor inducible precursor	R.LQEQEELENYIEHVLLR.R	3	2.77	0.14	-2.56
IPI00069058	VGF nerve growth factor inducible precursor	R.M*PDSGPLPETH.K	2	3.26	0.47	-2.56
IPI00069058	VGF nerve growth factor inducible precursor	R.M*PDSGPLPETHK.F	1	1.93	0.26	-3.75
IPI00069058	VGF nerve growth factor inducible precursor	R.M*PDSGPLPETHK.F	2	3.60	0.35	-3.05
IPI00069058	VGF nerve growth factor inducible precursor	R.M*PDSGPLPETHK.F	3	2.84	0.16	-3.75
IPI00069058	VGF nerve growth factor inducible precursor	R.M*PDSGPLPETHKFGEGVSSPK.T	2	4.21	0.50	-4.75
IPI00069058	VGF nerve growth factor inducible precursor	R.M*PDSGPLPETHKFGEGVSSPK.T	3	3.17	0.36	-2.85
IPI00069058	VGF nerve growth factor inducible precursor	R.M*PDSGPLPETHKFGEGVSSPK.T	4	2.81	0.23	-2.98
IPI00069058	VGF nerve growth factor inducible precursor	R.NSEPQDEGELFQGVDPR.A	2	4.47	0.43	-4.88
IPI00069058	VGF nerve growth factor inducible precursor	R.NSEPQDEGELFQGVDPR.A	3	4.18	0.42	-4.02
IPI00069058	VGF nerve growth factor inducible precursor	R.QAAAQEERLADLASDLLLQYLLQGGAR.Q	3	3.76	0.12	
IPI00069058	VGF nerve growth factor inducible precursor	R.QNALLFAEEEDGEAGA.E	2	2.91	0.29	-3.80
IPI00069058	VGF nerve growth factor inducible precursor	R.QNALLFAEEEDGEAGAED.K	2	4.73	0.53	-4.03
IPI00069058	VGF nerve growth factor inducible precursor	R.QNALLFAEEEDGEAGAEDKR.S	3	3.35	0.40	-1.72
IPI00069058	VGF nerve growth factor inducible precursor	R.QQETAAAETETR.T	2	3.09	0.47	-3.26
IPI00069058	VGF nerve growth factor inducible precursor	R.RKEAEGTEEGGEEEDDEEM*DPQTIDSLIEL.S	3	4.02	0.38	-4.65
IPI00069058	VGF nerve growth factor inducible precursor	R.RKEAEGTEEGGEEEDDEEM*DPQTIDSLIELSTK.L	3	5.88	0.58	-5.21
IPI00069058	VGF nerve growth factor inducible precursor	R.RKEAEGTEEGGEEEDDEEM*DPQTIDSLIELSTK.L	4	4.87	0.45	-5.08
IPI00069058	VGF nerve growth factor inducible precursor	R.RLQEQEELENYIEHVLLR.R	3	5.85	0.40	-4.66
IPI00069058	VGF nerve growth factor inducible precursor	R.RLQEQEELENYIEHVLLR.R	4	5.49	0.34	-3.42
IPI00069058	VGF nerve growth factor inducible precursor	R.RPESALLGGSEAGE.R	2	4.36	0.50	-1.49
IPI00069058	VGF nerve growth factor inducible precursor	R.RPESALLGGSEAGER.L	2	3.64	0.35	-4.70
IPI00069058	VGF nerve growth factor inducible precursor	R.RPESALLGGSEAGER.L	3	3.92	0.36	-2.91

IPI00069058	VGF nerve growth factor inducible precursor	R.SPQPPPPAPAPARDELP.D	2	3.50	0.44	-3.67
IPI00069058	VGF nerve growth factor inducible precursor	R.SPQPPPPAPAPARDELPD.W	2	3.72	0.50	-4.31
IPI00069058	VGF nerve growth factor inducible precursor	R.SPQPPPPAPAPARDELPDWN.E	2	3.01	0.37	-2.75
IPI00069058	VGF nerve growth factor inducible precursor	R.SPQPPPPAPAPARDELPDWNEVLPPWDREED.E	3	4.19	0.48	-3.69
IPI00069058	VGF nerve growth factor inducible precursor	R.SQTHSLPAPESPEPAAPPRPQTPENGPE.A	3	3.82	0.46	-2.25
IPI00069058	VGF nerve growth factor inducible precursor	R.SQTHSLPAPESPEPAAPPRPQTPENGPEASD.P	3	4.84	0.38	-1.09
IPI00069058	VGF nerve growth factor inducible precursor	R.SQTHSLPAPESPEPAAPPRPQTPENGPEASDPSEE.L	3	5.18	0.50	-2.18
IPI00069058	VGF nerve growth factor inducible precursor	R.SQTHSLPAPESPEPAAPPRPQTPENGPEASDPSEEL.E	3	4.38	0.52	-2.97
IPI00069058	VGF nerve growth factor inducible precursor	R.SQTHSLPAPESPEPAAPPRPQTPENGPEASDPSEELEALAS.L	3	4.06	0.41	-2.38
IPI00069058	VGF nerve growth factor inducible precursor	R.SQTHSLPAPESPEPAAPPRPQTPENGPEASDPSEELEALASL.L	3	4.29	0.44	-2.68
IPI00069058	VGF nerve growth factor inducible precursor	R.SQTHSLPAPESPEPAAPPRPQTPENGPEASDPSEELEALASLLQE.L	3	4.84	0.53	-3.57
IPI00069058	VGF nerve growth factor inducible precursor	R.VGEEDEEAAEAEAEAEEAER.A	2	7.22	0.49	-4.71
IPI00069058	VGF nerve growth factor inducible precursor	R.VGEEDEEAAEAEAEAEEAER.A	3	5.36	0.41	-4.96
IPI00069058	VGF nerve growth factor inducible precursor	R.VGEEDEEAAEAEAEAEEAERAR.Q	2	5.10	0.47	-3.80
IPI00069058	VGF nerve growth factor inducible precursor	R.VGEEDEEAAEAEAEAEEAERAR.Q	3	3.50	0.25	-3.36
IPI00069058	VGF nerve growth factor inducible precursor	R.VNLESPGPER.V	1	1.92	0.26	-2.69
IPI00069058	VGF nerve growth factor inducible precursor	R.VNLESPGPER.V	2	3.05	0.24	-1.99
IPI00069058	VGF nerve growth factor inducible precursor	R.VNLESPGPERVW.R	2	4.06	0.49	-2.74
IPI00069058	VGF nerve growth factor inducible precursor	R.VPERAPLPPPAPSQFQA.R	2	3.21	0.33	-2.34
IPI00069058	VGF nerve growth factor inducible precursor	R.VPERAPLPPPAPSQFQAR.M	2	3.86	0.46	-3.44
IPI00069058	VGF nerve growth factor inducible precursor	R.VPERAPLPPPAPSQFQAR.M	3	4.17	0.36	-3.59
IPI00069058	VGF nerve growth factor inducible precursor	S.EPQDEGELFQGVDPR.A	2	3.48	0.43	-3.86
IPI00071185	Isoform SV1 of PITSLRE serine/threonine-protein kinase CDC2L1	R.REVSAHHRTMREDYSDKVK.A	3	2.32	0.11	-5.88
IPI00071824	Isoform 1 of Cytoskeleton-associated protein 2	R.HTIVDILTMK.S	2	1.50	0.07	-0.17
IPI00072917	alpha 3 type VI collagen isoform 3 precursor	K.ALNLGYALDYAQR.Y	2	4.20	0.44	-4.07
IPI00072917	alpha 3 type VI collagen isoform 3 precursor	K.ALNLGYALDYAQR.Y	3	5.34	0.31	-2.63
IPI00072917	alpha 3 type VI collagen isoform 3 precursor	K.EVYTFASEPNDVFFK.L	2	4.22	0.48	-3.28
IPI00072917	alpha 3 type VI collagen isoform 3 precursor	K.GADQAELEEIAFDSSLVFIPAEFR.A	2	3.69	0.43	-4.11
IPI00072917	alpha 3 type VI collagen isoform 3 precursor	K.GADQAELEEIAFDSSLVFIPAEFR.A	3	3.77	0.34	-0.72
IPI00072917	alpha 3 type VI collagen isoform 3 precursor	K.IASNSATAFR.V	2	2.75	0.34	0.50
IPI00072917	alpha 3 type VI collagen isoform 3 precursor	K.IGDLHPQIVNLLK.S	3	2.85	0.27	-1.55
IPI00072917	alpha 3 type VI collagen isoform 3 precursor	K.KSQPPPPQPAR.S	3	2.54	0.13	-3.74
IPI00072917	alpha 3 type VI collagen isoform 3 precursor	K.LLPYIVGVAQR.H	2	3.30	0.31	-3.80
IPI00072917	alpha 3 type VI collagen isoform 3 precursor	K.LLTPITTLTSEQIQK.L	2	4.23	0.49	-2.09
IPI00072917	alpha 3 type VI collagen isoform 3 precursor	K.LLVLITGGK.S	2	3.34	0.11	-3.10
IPI00072917	alpha 3 type VI collagen isoform 3 precursor	K.LSDAGITPLFLTR.Q	2	3.63	0.30	-3.51
IPI00072917	alpha 3 type VI collagen isoform 3 precursor	K.LVDFLSR.G	1	2.13	0.06	-4.27
IPI00072917	alpha 3 type VI collagen isoform 3 precursor	K.LVDFLSR.G	2	2.20	0.08	-3.42
IPI00072917	alpha 3 type VI collagen isoform 3 precursor	K.LVDKSTELNEEPLM*R.F	2	4.31	0.37	-1.31
IPI00072917	alpha 3 type VI collagen isoform 3 precursor	K.LVDKSTELNEEPLM*R.F	3	4.77	0.20	-1.18
IPI00072917	alpha 3 type VI collagen isoform 3 precursor	K.M*KPLDGSALYTGSALDFVR.N	2	4.47	0.46	-0.88

IPI00072917	alpha 3 type VI collagen isoform 3 precursor	K.M*KPLDGSALYTGSALDFVR.N	3	3.56	0.16	-1.97
IPI00072917	alpha 3 type VI collagen isoform 3 precursor	K.NADPAELEQIVLSPAFILAAESLPK.I	2	5.33	0.45	-2.60
IPI00072917	alpha 3 type VI collagen isoform 3 precursor	K.NADPAELEQIVLSPAFILAAESLPK.I	3	8.08	0.58	-3.61
IPI00072917	alpha 3 type VI collagen isoform 3 precursor	K.QDVVNAVR.Q	2	2.18	0.24	-3.25
IPI00072917	alpha 3 type VI collagen isoform 3 precursor	K.QFGVAPFTIAR.N	2	2.56	0.23	-2.01
IPI00072917	alpha 3 type VI collagen isoform 3 precursor	K.QSGVVPFIFQAK.N	2	3.11	0.24	-3.88
IPI00072917	alpha 3 type VI collagen isoform 3 precursor	K.SDDEVDDPAVELK.Q	2	3.76	0.26	-1.88
IPI00072917	alpha 3 type VI collagen isoform 3 precursor	K.SLDEISQPAQELKR.S	2	3.87	0.30	-1.61
IPI00072917	alpha 3 type VI collagen isoform 3 precursor	K.STELNEEPLM*R.F	2	3.25	0.24	-3.07
IPI00072917	alpha 3 type VI collagen isoform 3 precursor	K.SVEDAQDVSLALTQR.G	2	3.26	0.29	-5.18
IPI00072917	alpha 3 type VI collagen isoform 3 precursor	K.VCAPVLAKPGVISVM*GT.-	2	4.30	0.40	-3.35
IPI00072917	alpha 3 type VI collagen isoform 3 precursor	K.VNIKEVYTFASEPNDVFFK.L	3	3.25	0.24	-3.33
IPI00072917	alpha 3 type VI collagen isoform 3 precursor	K.WYYDPNTK.S	1	1.95	0.06	-3.03
IPI00072917	alpha 3 type VI collagen isoform 3 precursor	K.WYYDPNTK.S	2	1.90	0.17	-1.49
IPI00072917	alpha 3 type VI collagen isoform 3 precursor	K.YIAYLVR.Q	1	1.88	0.15	-2.69
IPI00072917	alpha 3 type VI collagen isoform 3 precursor	K.YIAYLVR.Q	2	2.62	0.16	-2.03
IPI00072917	alpha 3 type VI collagen isoform 3 precursor	R.AAPLQGM*LPGLLAPLR.T	2	3.19	0.39	-4.76
IPI00072917	alpha 3 type VI collagen isoform 3 precursor	R.ALGSAIEYTIENVFESAPNPR.D	2	5.46	0.53	-4.77
IPI00072917	alpha 3 type VI collagen isoform 3 precursor	R.ALGSAIEYTIENVFESAPNPR.D	3	4.09	0.31	-5.37
IPI00072917	alpha 3 type VI collagen isoform 3 precursor	R.ALGSAIEYTIENVFESAPNPR.D	4	3.41	0.44	-2.78
IPI00072917	alpha 3 type VI collagen isoform 3 precursor	R.ALILVGLER.V	2	2.96	0.18	-1.85
IPI00072917	alpha 3 type VI collagen isoform 3 precursor	R.DSFQEVLR.F	2	2.82	0.19	-1.38
IPI00072917	alpha 3 type VI collagen isoform 3 precursor	R.DVVFLIDGSQSAGPEFQYVR.T	2	5.11	0.57	-2.64
IPI00072917	alpha 3 type VI collagen isoform 3 precursor	R.DVVFLIDGSQSAGPEFQYVR.T	3	5.06	0.54	-2.25
IPI00072917	alpha 3 type VI collagen isoform 3 precursor	R.FDEHQSKPEILNLVK.R	3	3.53	0.30	-3.82
IPI00072917	alpha 3 type VI collagen isoform 3 precursor	R.GM*TQLQGTR.A	2	3.08	0.21	-2.16
IPI00072917	alpha 3 type VI collagen isoform 3 precursor	R.IAVAQYSDDVK.V	2	3.47	0.42	-2.18
IPI00072917	alpha 3 type VI collagen isoform 3 precursor	R.IAVAQYSDDVKVESR.F	3	3.26	0.46	-1.68
IPI00072917	alpha 3 type VI collagen isoform 3 precursor	R.IEDGVPQHLVLVLGGK.S	3	2.56	0.30	-1.60
IPI00072917	alpha 3 type VI collagen isoform 3 precursor	R.IEEGVPQFLVLISSGK.S	2	3.45	0.33	-2.76
IPI00072917	alpha 3 type VI collagen isoform 3 precursor	R.ITEGVPQLLIVLTADR.S	2	4.65	0.40	-0.44
IPI00072917	alpha 3 type VI collagen isoform 3 precursor	R.ITEGVPQLLIVLTADR.S	3	2.46	0.27	-2.28
IPI00072917	alpha 3 type VI collagen isoform 3 precursor	R.KVNIKEVYTFASEPNDVFFK.L	2	6.49	0.59	-3.61
IPI00072917	alpha 3 type VI collagen isoform 3 precursor	R.KVNIKEVYTFASEPNDVFFK.L	3	4.91	0.47	-3.73
IPI00072917	alpha 3 type VI collagen isoform 3 precursor	R.LEIGQDLIQVAVAQYADTVRPEFYFNTHPTK.R	4	3.83	0.28	-3.53
IPI00072917	alpha 3 type VI collagen isoform 3 precursor	R.LLPSFVSSENAFYLSPDIR.K	2	4.61	0.45	-4.66
IPI00072917	alpha 3 type VI collagen isoform 3 precursor	R.LLPSFVSSENAFYLSPDIR.K	3	3.92	0.43	-5.46
IPI00072917	alpha 3 type VI collagen isoform 3 precursor	R.LLPSFVSSENAFYLSPDIRK.Q	2	3.61	0.42	-2.13
IPI00072917	alpha 3 type VI collagen isoform 3 precursor	R.LNLLDLDYELAEQLDNIAEK.A	2	6.10	0.49	-4.80
IPI00072917	alpha 3 type VI collagen isoform 3 precursor	R.LNLLDLDYELAEQLDNIAEK.A	3	3.98	0.33	-2.49
IPI00072917	alpha 3 type VI collagen isoform 3 precursor	R.LQPVLQPLPSPGVGGK.R	2	3.96	0.42	-3.58
IPI00072917	alpha 3 type VI collagen isoform 3 precursor	R.LQPVLQPLPSPGVGGKR.D	2	2.81	0.29	-2.00

IPI00072917	alpha 3 type VI collagen isoform 3 precursor	R.NIDRTELQTITNDPR.L	3	2.82	0.23	-1.75
IPI00072917	alpha 3 type VI collagen isoform 3 precursor	R.NILVSSAGSR.I	2	2.78	0.19	-2.24
IPI00072917	alpha 3 type VI collagen isoform 3 precursor	R.NNLFTSSAGYR.A	2	3.14	0.37	-1.42
IPI00072917	alpha 3 type VI collagen isoform 3 precursor	R.QINVGNALEYVSR.N	2	3.71	0.41	-2.68
IPI00072917	alpha 3 type VI collagen isoform 3 precursor	R.QLGTVQQVISER.V	2	3.04	0.35	-2.76
IPI00072917	alpha 3 type VI collagen isoform 3 precursor	R.QLTLLGGPTPNTGAALEFVLR.N	2	4.06	0.48	-3.10
IPI00072917	alpha 3 type VI collagen isoform 3 precursor	R.QLTLLGGPTPNTGAALEFVLR.N	3	5.16	0.46	-3.47
IPI00072917	alpha 3 type VI collagen isoform 3 precursor	R.SASSSTINLM*VSTEPLALTETDICKLPK.D	3	3.72	0.27	-2.09
IPI00072917	alpha 3 type VI collagen isoform 3 precursor	R.SGDDVRNPSVVVK.R	3	1.66	0.11	-0.60
IPI00072917	alpha 3 type VI collagen isoform 3 precursor	R.SGDDVRNPSVVVKR.G	3	2.66	0.27	0.20
IPI00072917	alpha 3 type VI collagen isoform 3 precursor	R.SSDRVDGPASNLK.Q	2	2.87	0.06	-1.47
IPI00072917	alpha 3 type VI collagen isoform 3 precursor	R.SSGIVSLGVGDR.N	2	3.06	0.24	-2.71
IPI00072917	alpha 3 type VI collagen isoform 3 precursor	R.SSIM*AFAIGNK.G	2	3.16	0.36	-0.73
IPI00072917	alpha 3 type VI collagen isoform 3 precursor	R.TRPEFYLNSYM*NKQDVVNAVR.Q	3	4.47	0.51	-3.31
IPI00072917	alpha 3 type VI collagen isoform 3 precursor	R.TRPEFYLNSYM*NKQDVVNAVR.Q	4	3.65	0.28	1.05
IPI00072917	alpha 3 type VI collagen isoform 3 precursor	R.VAVVQYSDR.T	2	3.07	0.23	-1.69
IPI00072917	alpha 3 type VI collagen isoform 3 precursor	R.VNHFVPEAGSR.L	2	2.43	0.23	-0.11
IPI00072917	alpha 3 type VI collagen isoform 3 precursor	R.VVESLDVGQDR.V	2	3.24	0.38	-2.67
IPI00072917	alpha 3 type VI collagen isoform 3 precursor	R.YPPPAVESDAADIVFLIDSSEGVRPDGFAHIR.D	4	4.49	0.40	-3.68
IPI00073763	Semaphorin-4C precursor	K.LLFAGSRSQLVQLPVADCMKYR.S	3	2.95	0.20	
IPI00075013	Complement C1q tumor necrosis factor-related protein 1 precursor	K.SHYAAFSVGR.K	2	2.00	0.26	
IPI00075013	Complement C1q tumor necrosis factor-related protein 1 precursor	K.YRPSQDQGLPASR.C	2	2.21	0.14	-1.38
IPI00075248	Calmodulin	K.EAFSLFDKDGDGTITTK.E	2	4.49	0.40	-1.90
IPI00075248	Calmodulin	K.EAFSLFDKDGDGTITTK.E	3	2.62	0.19	-0.88
IPI00075248	Calmodulin	L.FDKDGDGTITTK.E	2	3.05	0.35	-1.16
IPI00075248	Calmodulin	R.EADIDGDGQVNYEEFVQMMTAK.-	2	4.65	0.59	-2.24
IPI00075248	Calmodulin	R.EADIDGDGQVNYEEFVQMMTAK.-	3	3.15	0.38	-2.41
IPI00075248	Calmodulin	R.VFDKDGNGYISAAELR.H	2	4.15	0.40	0.94
IPI00075248	Calmodulin	R.VFDKDGNGYISAAELR.H	3	2.67	0.18	-0.23
IPI00081836	Histone H2A type 1-H	R.AGLQFPVGR.I	2	2.49	0.10	-2.97
IPI00081836	Histone H2A type 1-H	R.HLQLAIRNDEELNKLLGKVTIAQGGVLPNIQAVLLPK.K	4	3.02	0.27	-2.89
IPI00081836	Histone H2A type 1-H	R.HLQLAIRNDEELNKLLGKVTIAQGGVLPNIQAVLLPK.K	5	2.23	0.19	-3.80
IPI00081836	Histone H2A type 1-H	R.VTIAQGGVLPNIQAVLLPK.K	2	4.34	0.48	-3.79
IPI00090764	Toll-like receptor 1 precursor	K.NFTVSGTRM*VHMLCPSK.I	2	2.49	0.17	
IPI00098902	2-oxoglutarate dehydrogenase E1 component, mitochondrial precursor	R.TVDWALAEYMAFGSLLK.E	3	2.51	0.10	-1.27
IPI00099650	Protein jagged-1 precursor	K.IIDLVSKR.D	2	2.19	0.10	-4.56
IPI00099650	Protein jagged-1 precursor	R.IVLPFSFAWPR.S	2	3.09	0.26	-3.35
IPI00099670	carboxyl ester lipase precursor	K.LGAVYTEGGFVEGVNKK.L	3	2.39	0.08	-1.81
IPI00099670	carboxyl ester lipase precursor	K.TYAYLFSHPSR.M	2	3.48	0.45	-2.37

IPI00099670	carboxyl ester lipase precursor	K.VGCPVGDAAR.M	2	2.20	0.12	0.21
IPI00099670	carboxyl ester lipase precursor	K.VTEEDFYKLVSEFTITK.G	3	3.02	0.15	-3.53
IPI00099838	Isoform 1 of Lysophosphatidic acid phosphatase type 6 precursor	K.NYVEDIPFLSPTFNPQEVFIR.S	3	2.64	0.17	-5.20
IPI00100154	Toll-interacting protein	R.GNKDAAINSLLQM*GEEP.-	2	4.05	0.28	-2.59
IPI00100154	Toll-interacting protein	R.GPVYIGELPQDFLR.I	2	4.12	0.42	-3.24
IPI00100980	EH domain-containing protein 2	R.LVRVHAYIISYLKKEMPSVFGK.E	3	3.55	0.08	
IPI00101608	Isoform 2 of Cysteine-rich with EGF-like domain protein 1 precursor	K.QQEAPDLFQWLCSDSLK.L	2	3.28	0.32	-4.69
IPI00101608	Isoform 2 of Cysteine-rich with EGF-like domain protein 1 precursor	K.QQEAPDLFQWLCSDSLK.L	3	3.55	0.27	-3.55
IPI00101608	Isoform 2 of Cysteine-rich with EGF-like domain protein 1 precursor	R.DEATFPGLYGK.Q	2	2.29	0.26	-2.65
IPI00101608	Isoform 2 of Cysteine-rich with EGF-like domain protein 1 precursor	R.GLVDSFNK.G	1	2.05	0.16	-3.02
IPI00101608	Isoform 2 of Cysteine-rich with EGF-like domain protein 1 precursor	R.TIRDNFGGGNTAWEEENLSKYK.D	3	2.18	0.14	-1.36
IPI00101927	Leucine zipper putative tumor suppressor 2	K.QLQHNYIQMYRRNRQLEQELQQLSLELEAR.E	3	1.64	0.20	1.96
IPI00102435	collagen, type XXI, alpha 1 precursor	K.AIQFALDYLFAK.S	2	3.59	0.42	-4.28
IPI00102435	collagen, type XXI, alpha 1 precursor	K.IAVVLTDGK.S	2	2.91	0.29	-2.97
IPI00102435	collagen, type XXI, alpha 1 precursor	K.IAVVLTDGKSQDDVKDAAQAAR.D	3	3.72	0.46	-3.04
IPI00102435	collagen, type XXI, alpha 1 precursor	K.SQDDVKDAAQAAR.D	2	4.05	0.42	-2.10
IPI00102435	collagen, type XXI, alpha 1 precursor	K.VDLSELTSNVFPEGLPPSYVFVSTQR.F	3	3.99	0.42	-3.64
IPI00102435	collagen, type XXI, alpha 1 precursor	K.YSGKEETVQFDVQK.L	2	4.18	0.32	-2.43
IPI00102435	collagen, type XXI, alpha 1 precursor	K.YSGKEETVQFDVQK.L	3	3.68	0.30	-1.21
IPI00102543	SLIT and NTRK-like protein 1 precursor	K.DFM*LLSNDEICPQLYAR.I	2	4.71	0.49	-1.71
IPI00102543	SLIT and NTRK-like protein 1 precursor	K.KGFTSLQR.F	1	2.14	0.17	-5.16
IPI00102543	SLIT and NTRK-like protein 1 precursor	K.LSNVQELFLR.D	2	4.22	0.28	-2.09
IPI00102543	SLIT and NTRK-like protein 1 precursor	K.LSNVQELFLRDNK.I	3	2.61	0.17	-2.75
IPI00102543	SLIT and NTRK-like protein 1 precursor	K.QTFLGLDDLEYLQADFNLLR.D	2	6.32	0.55	-7.28
IPI00102543	SLIT and NTRK-like protein 1 precursor	K.QTFLGLDDLEYLQADFNLLR.D	3	6.31	0.42	-6.18
IPI00102543	SLIT and NTRK-like protein 1 precursor	K.SHFVDYK.N	1	2.21	0.09	-3.33
IPI00102543	SLIT and NTRK-like protein 1 precursor	R.DIDPGAFQDLNK.L	2	3.11	0.36	-2.07
IPI00102543	SLIT and NTRK-like protein 1 precursor	R.DIDPGAFQDLNKLEVLILNDNLISTLPANVFQYVPITHLDLR.G	3	3.41	0.39	-4.22
IPI00102543	SLIT and NTRK-like protein 1 precursor	R.DIDPGAFQDLNKLEVLILNDNLISTLPANVFQYVPITHLDLR.G	4	5.13	0.48	-4.35
IPI00102543	SLIT and NTRK-like protein 1 precursor	R.FTAPTSQFYHLFLHGNSLTR.L	2	4.97	0.45	-4.70
IPI00102543	SLIT and NTRK-like protein 1 precursor	R.FTAPTSQFYHLFLHGNSLTR.L	3	3.03	0.30	-4.55
IPI00102543	SLIT and NTRK-like protein 1 precursor	R.FTAPTSQFYHLFLHGNSLTR.L	4	2.25	0.25	-3.52
IPI00102543	SLIT and NTRK-like protein 1 precursor	R.ILILNNNLLR.S	2	3.27	0.16	-2.04
IPI00102543	SLIT and NTRK-like protein 1 precursor	R.KQTFLGLDDLEYLQADFNLLR.D	2	5.31	0.49	-1.47
IPI00102543	SLIT and NTRK-like protein 1 precursor	R.KQTFLGLDDLEYLQADFNLLR.D	3	4.29	0.35	-5.40
IPI00102543	SLIT and NTRK-like protein 1 precursor	R.LGSEVLM*SDLKCETPVNFFR.K	3	4.71	0.54	-3.36

IPI00102543	SLIT and NTRK-like protein 1 precursor	R.SLPVDVFAGVSLSK.L	2	3.96	0.35	-3.21
IPI00102543	SLIT and NTRK-like protein 1 precursor	R.VVCEAPTR.L	1	1.38	0.10	-2.32
IPI00102543	SLIT and NTRK-like protein 1 precursor	R.VVCEAPTR.L	2	2.64	0.33	-2.00
IPI00102543	SLIT and NTRK-like protein 1 precursor	R.WLYM*DSNYLDTLSR.E	2	4.76	0.48	-5.77
IPI00102575	ATPase family, AAA domain containing 5	K.AKALHISRSKVTEEIAIPLR.R	3	2.17	0.21	
IPI00102575	ATPase family, AAA domain containing 5	R.KTSIPVKDIKLTQSKAESEASLLNVSTPK.S	2	1.23	0.10	1.59
IPI00102575	ATPase family, AAA domain containing 5	T.TSHANSRDNVTEAAQLNDSIITVSYEEFLKSHK.E	3	3.95	0.17	-1.82
IPI00102678	Isoform 1 of Pecanex-like protein 1	K.SKPLKAEKSM*DSLRSLSTR.S	2	2.26	0.07	-4.09
IPI00102808	Isoform 1 of Dual specificity protein phosphatase 19	K.NARPSICPNSGFM*EQLRTYQEGKESNK.C	3	2.84	0.05	-8.44
IPI00103175	Isoform 1 of Soluble calcium-activated nucleotidase 1	K.AEWLAVKDER.L	2	2.51	0.09	-2.16
IPI00103175	Isoform 1 of Soluble calcium-activated nucleotidase 1	K.AEWLAVKDER.L	3	2.40	0.16	-3.31
IPI00103175	Isoform 1 of Soluble calcium-activated nucleotidase 1	K.AVPWVILSDGDGTVEK.G	2	4.41	0.48	-2.97
IPI00103175	Isoform 1 of Soluble calcium-activated nucleotidase 1	K.GANLLLSASPDFGDIAVSHVGAVVPTHGFSSFK.F	4	3.26	0.18	-2.85
IPI00103175	Isoform 1 of Soluble calcium-activated nucleotidase 1	K.IGSVKYEGIEFI.-	2	3.73	0.38	-3.63
IPI00103175	Isoform 1 of Soluble calcium-activated nucleotidase 1	K.LYSVDDR.T	1	1.65	0.08	-3.47
IPI00103175	Isoform 1 of Soluble calcium-activated nucleotidase 1	K.LYSVDDR.T	2	2.07	0.20	-3.62
IPI00103175	Isoform 1 of Soluble calcium-activated nucleotidase 1	K.YEGIEFI.-	1	1.73	0.19	-2.73
IPI00103175	Isoform 1 of Soluble calcium-activated nucleotidase 1	R.AQEENTWFSYLK.K	2	2.99	0.35	-4.05
IPI00103175	Isoform 1 of Soluble calcium-activated nucleotidase 1	R.GM*ELSDLIVFNGK.L	2	3.58	0.26	-2.95
IPI00103175	Isoform 1 of Soluble calcium-activated nucleotidase 1	R.IAVIADLDTESR.A	2	4.32	0.47	-3.21
IPI00103175	Isoform 1 of Soluble calcium-activated nucleotidase 1	R.KGANLLLSASPDFGDIAVSHVGAVVPTHGFSSFK.F	4	3.21	0.33	-2.84
IPI00103175	Isoform 1 of Soluble calcium-activated nucleotidase 1	R.LYVGGLGK.E	1	2.20	0.15	-3.08
IPI00103175	Isoform 1 of Soluble calcium-activated nucleotidase 1	R.TGVVYQIEGSK.A	1	2.42	0.20	-3.57
IPI00103175	Isoform 1 of Soluble calcium-activated nucleotidase 1	R.TGVVYQIEGSK.A	2	3.70	0.44	-2.81
IPI00103175	Isoform 1 of Soluble calcium-activated nucleotidase 1	R.VASYIM*AFTLDGR.F	2	4.18	0.44	-4.39

IPI00103175	Isoform 1 of Soluble calcium-activated nucleotidase 1	R.VASYIMAFTLDGR.F	2	3.75	0.51	-4.50
IPI00103471	Thioredoxin-like selenoprotein M precursor	K.AFVTQDIPFYHNLVM*K.H	2	3.97	0.38	-3.10
IPI00103471	Thioredoxin-like selenoprotein M precursor	K.AFVTQDIPFYHNLVM*K.H	3	2.61	0.23	-2.30
IPI00103471	Thioredoxin-like selenoprotein M precursor	R.EEINALVQELGFYR.K	2	4.80	0.36	-3.58
IPI00103471	Thioredoxin-like selenoprotein M precursor	R.EEINALVQELGFYRK.A	3	2.62	0.25	-2.62
IPI00103471	Thioredoxin-like selenoprotein M precursor	R.IPLSEM*TREEINALVQELGFYR.K	3	4.64	0.36	-2.12
IPI00103471	Thioredoxin-like selenoprotein M precursor	R.IPLSEM*TREEINALVQELGFYRK.A	4	2.72	0.21	-4.46
IPI00103471	Thioredoxin-like selenoprotein M precursor	R.RYEELER.I	2	1.97	0.06	0.48
IPI00103471	Thioredoxin-like selenoprotein M precursor	R.RYEELERIPLSEM*TR.E	3	2.20	0.30	-0.46
IPI00103471	Thioredoxin-like selenoprotein M precursor	R.RYEELERIPLSEM*TR.E	4	2.36	0.15	-0.68
IPI00103510	Relaxin receptor 2	R.KSIFKIKKKSLSTSIVWIEDSSSLK.L	3	2.99	0.12	
IPI00103552	Mucin-16	R.FPQSVVTTPM*SR.G	2	1.95	0.19	
IPI00103597	Isoform 1 of VPS10 domain-containing receptor SorCS1 precursor	K.SIFDRRCAEEDYRPWQLHSQGEACIMGAK.R	3	1.75	0.21	1.44
IPI00103597	Isoform 1 of VPS10 domain-containing receptor SorCS1 precursor	K.TIAVYEEFR.S	2	2.97	0.37	-2.27
IPI00103597	Isoform 1 of VPS10 domain-containing receptor SorCS1 precursor	K.TILSYLYVCPTNK.R	2	3.79	0.38	-5.24
IPI00103597	Isoform 1 of VPS10 domain-containing receptor SorCS1 precursor	K.VSENPYTSGIIASK.D	2	4.10	0.49	-2.55
IPI00103597	Isoform 1 of VPS10 domain-containing receptor SorCS1 precursor	R.GTGASM*AVAAR.S	2	3.51	0.34	-3.47
IPI00103597	Isoform 1 of VPS10 domain-containing receptor SorCS1 precursor	R.NAFAQM*K.L	1	1.83	0.08	-3.12
IPI00103597	Isoform 1 of VPS10 domain-containing receptor SorCS1 precursor	R.STDDLEQISELLIHTLNQNSVHFELKPGVR.V	3	6.64	0.59	-4.57
IPI00103597	Isoform 1 of VPS10 domain-containing receptor SorCS1 precursor	R.STDDLEQISELLIHTLNQNSVHFELKPGVR.V	4	4.36	0.27	-3.32
IPI00103597	Isoform 1 of VPS10 domain-containing receptor SorCS1 precursor	R.STDDLEQISELLIHTLNQNSVHFELKPGVR.V	5	3.35	0.23	-2.29
IPI00103597	Isoform 1 of VPS10 domain-containing receptor SorCS1 precursor	R.STDYGTTYEK.L	2	3.12	0.29	-2.90
IPI00103597	Isoform 1 of VPS10 domain-containing receptor SorCS1 precursor	R.STDYGTTYEKLNDK.V	2	3.71	0.51	0.07
IPI00103597	Isoform 1 of VPS10 domain-containing receptor SorCS1 precursor	R.WQLIQEGVVPNR.F	2	3.36	0.28	-3.64
IPI00103630	Isoform 2 of Protein phosphatase 1E	R.FNPKFYSFLSAQEPSHK.I	2	2.32	0.13	
IPI00103755	Isoform 2 of Netrin receptor UNC5D precursor	R.ADHNLIIR.Q	2	2.05	0.08	-3.20
IPI00103755	Isoform 2 of Netrin receptor UNC5D precursor	R.KNFEQDPQGR.E	2	2.03	0.21	-1.65
IPI00103853	Isoform 1 of Putative ribosome-binding factor A, mitochondrial precursor	L.YDLNVELSK.V	2	3.03	0.24	-1.51

IPI00103871	Isoform 1 of Roundabout homolog 4 precursor	K.VSGPAAPAQSYTALFR.T	2	4.08	0.46	-4.49
IPI00103871	Isoform 1 of Roundabout homolog 4 precursor	R.ARGPDSNVLLLR.L	2	2.25	0.15	-3.14
IPI00103871	Isoform 1 of Roundabout homolog 4 precursor	R.LSVAVLREDFQIQPR.D	3	4.26	0.41	-3.60
IPI00103871	Isoform 1 of Roundabout homolog 4 precursor	R.VSIQEPQDYTEPVELLAVR.I	2	5.14	0.44	-5.86
IPI00103874	Isoform 1 of Zinc finger FYVE domain-containing protein 1	K.RTHSGGNK.R	2	1.54	0.09	2.76
IPI00103891	Putative uncharacterized protein	R.SANSGSLKEEPLILSTECPLEPATTLR.G	4	2.83	0.15	1.61
IPI00104074	Isoform 1 of Scavenger receptor cysteine-rich type 1 protein M130 precursor	K.AM*SIPM*WVDNVQCPK.G	2	3.40	0.44	-2.42
IPI00104074	Isoform 1 of Scavenger receptor cysteine-rich type 1 protein M130 precursor	K.CGVALSTPGGAR.F	2	2.47	0.17	-1.97
IPI00104074	Isoform 1 of Scavenger receptor cysteine-rich type 1 protein M130 precursor	K.GKINPASLDK.A	2	2.23	0.13	-1.96
IPI00104074	Isoform 1 of Scavenger receptor cysteine-rich type 1 protein M130 precursor	K.INPASLDK.A	1	1.75	0.10	-1.98
IPI00104074	Isoform 1 of Scavenger receptor cysteine-rich type 1 protein M130 precursor	K.SSM*SETTVGVVCR.Q	2	3.67	0.45	-1.94
IPI00104074	Isoform 1 of Scavenger receptor cysteine-rich type 1 protein M130 precursor	K.TSYQVYSK.I	1	1.96	0.10	-1.98
IPI00104074	Isoform 1 of Scavenger receptor cysteine-rich type 1 protein M130 precursor	K.TSYQVYSK.I	2	2.97	0.30	1.06
IPI00104074	Isoform 1 of Scavenger receptor cysteine-rich type 1 protein M130 precursor	R.CAGTVEVEIQR.L	2	3.69	0.31	-3.13
IPI00104074	Isoform 1 of Scavenger receptor cysteine-rich type 1 protein M130 precursor	R.DVGVVCSR.Y	2	2.60	0.36	-3.86
IPI00104074	Isoform 1 of Scavenger receptor cysteine-rich type 1 protein M130 precursor	R.HKEDAGVICSEFM*SLR.L	3	3.41	0.23	-3.49
IPI00104074	Isoform 1 of Scavenger receptor cysteine-rich type 1 protein M130 precursor	R.LASPSEETWITCDNK.I	2	4.62	0.51	-2.69
IPI00104074	Isoform 1 of Scavenger receptor cysteine-rich type 1 protein M130 precursor	R.LQEGPTSCSGR.V	2	2.82	0.32	-1.55
IPI00104074	Isoform 1 of Scavenger receptor cysteine-rich type 1 protein M130 precursor	R.LVGGDIPCSGR.V	2	3.18	0.35	-2.12
IPI00104074	Isoform 1 of Scavenger receptor cysteine-rich type 1 protein M130 precursor	R.VEIWHGGSWGTVCDDSWDLDDAQVVCQQLGCGPALK.A	3	5.90	0.52	-5.52
IPI00104074	Isoform 1 of Scavenger receptor cysteine-rich type 1 protein M130 precursor	R.VEIWHGGSWGTVCDDSWDLDDAQVVCQQLGCGPALK.A	4	4.25	0.37	-4.33
IPI00104074	Isoform 1 of Scavenger receptor cysteine-rich type 1 protein M130 precursor	R.WGTVCDDNFNIDHASVICR.Q	3	3.19	0.44	-2.16
IPI00104907	Isoform 1 of Uncharacterized potential DNA-binding protein C14orf106	K.MTTFNNK.N	2	1.64	0.08	-3.20
IPI00106502	Kelch-like ECH-associated protein 1	R.LLYAVGGFDGTNR.L	2	3.11	0.14	

IPI00106506	Isoform 1 of Evolutionarily conserved signaling intermediate in Toll pathway, mitochondrial precursor	R.LAGSTR.E	1	1.60	0.11	-8.34
IPI00106646	45 kDa calcium-binding protein precursor	K.DLGGFDEDAEPR.R	1	2.55	0.31	-1.91
IPI00106646	45 kDa calcium-binding protein precursor	K.DLGGFDEDAEPR.R	2	4.24	0.39	-2.54
IPI00106646	45 kDa calcium-binding protein precursor	K.DLGGFDEDAEPRR.S	2	2.21	0.14	-2.39
IPI00106646	45 kDa calcium-binding protein precursor	K.GHSEKEVADAIR.L	2	3.48	0.37	
IPI00106646	45 kDa calcium-binding protein precursor	K.LEM*DGHLNR.G	2	3.00	0.16	-2.43
IPI00106646	45 kDa calcium-binding protein precursor	R.ERVANREENEILPPDHLNGVK.L	3	3.66	0.19	-3.68
IPI00106646	45 kDa calcium-binding protein precursor	R.GFHQEVFLGK.D	1	2.33	0.14	-4.14
IPI00106646	45 kDa calcium-binding protein precursor	R.GFHQEVFLGK.D	2	3.43	0.29	-4.93
IPI00106646	45 kDa calcium-binding protein precursor	R.GFHQEVFLGKDLGGFDEDAEPR.R	3	3.26	0.38	-3.55
IPI00106646	45 kDa calcium-binding protein precursor	R.GFHQEVFLGKDLGGFDEDAEPR.R	4	3.10	0.14	-3.63
IPI00106646	45 kDa calcium-binding protein precursor	R.GFHQEVFLGKDLGGFDEDAEPRR.S	3	3.61	0.20	-4.16
IPI00106646	45 kDa calcium-binding protein precursor	R.GFHQEVFLGKDLGGFDEDAEPRR.S	4	3.55	0.22	-3.90
IPI00106646	45 kDa calcium-binding protein precursor	R.VANREENEILPPDHLNGVK.L	3	3.02	0.11	-2.34
IPI00107731	Isoform 6 of Osteoclast associated immunoglobulin-like receptor precursor	R.DVSSELAEFFLEEVTPAQGGSYR.C	2	4.82	0.48	-3.23
IPI00107731	Isoform 6 of Osteoclast associated immunoglobulin-like receptor precursor	R.DVSSELAEFFLEEVTPAQGGSYR.C	3	5.49	0.45	-2.96
IPI00107731	Isoform 6 of Osteoclast associated immunoglobulin-like receptor precursor	R.EGVAAPLQYR.H	2	2.57	0.21	-2.34
IPI00107731	Isoform 6 of Osteoclast associated immunoglobulin-like receptor precursor	R.FGLFKPGEIAPLLFR.D	3	3.00	0.29	-2.27
IPI00107831	Receptor-type tyrosine-protein phosphatase F precursor	K.DFLPVDPATSNGR.I	2	2.71	0.21	-5.46
IPI00107831	Receptor-type tyrosine-protein phosphatase F precursor	K.GSGPLSPSIQSR.T	2	2.96	0.23	-1.81
IPI00107831	Receptor-type tyrosine-protein phosphatase F precursor	K.LIADLQPNTEYSFVLM*NR.G	2	4.72	0.53	-3.51
IPI00107831	Receptor-type tyrosine-protein phosphatase F precursor	K.LIADLQPNTEYSFVLM*NR.G	3	4.40	0.48	-2.30
IPI00107831	Receptor-type tyrosine-protein phosphatase F precursor	K.LSVLEEEQLPPGFPSIDM*GPQLK.V	2	3.51	0.35	-3.62
IPI00107831	Receptor-type tyrosine-protein phosphatase F precursor	K.VM*CVSM*GSTTVR.V	2	1.50	0.20	-1.40
IPI00107831	Receptor-type tyrosine-protein phosphatase F precursor	K.VPEDQTGLSGGVASFVCQATGEPKPR.I	3	5.11	0.56	-5.54
IPI00107831	Receptor-type tyrosine-protein phosphatase F precursor	K.VSSQRFEVIEFDDGAGSVLR.I	3	4.51	0.32	-0.86
IPI00107831	Receptor-type tyrosine-protein phosphatase F precursor	K.VTFDPTSSYTLEDLKPDTLYR.F	3	2.65	0.28	-3.95

IPI00107831	Receptor-type tyrosine-protein phosphatase F precursor	R.AAGTEGPFQEVDGVATTR.Y	2	4.64	0.45	-2.77
IPI00107831	Receptor-type tyrosine-protein phosphatase F precursor	R.AHTDVGPGPESSPVLVR.T	2	4.40	0.45	-1.90
IPI00107831	Receptor-type tyrosine-protein phosphatase F precursor	R.AHTDVGPGPESSPVLVR.T	3	4.45	0.31	-0.88
IPI00107831	Receptor-type tyrosine-protein phosphatase F precursor	R.DEAIYECTATNSLGEINTSAK.L	2	5.43	0.52	-2.58
IPI00107831	Receptor-type tyrosine-protein phosphatase F precursor	R.FEVIEFDDGAGSVLR.I	2	5.17	0.53	-4.04
IPI00107831	Receptor-type tyrosine-protein phosphatase F precursor	R.FTLTGLKPDTTYDIK.V	3	2.91	0.16	-2.43
IPI00107831	Receptor-type tyrosine-protein phosphatase F precursor	R.GFYNRPLSPDLSYQCFVLASLKEPM*DQKR.Y	5	3.09	0.27	-2.96
IPI00107831	Receptor-type tyrosine-protein phosphatase F precursor	R.GPPSEAVR.A	2	1.65	0.12	-3.49
IPI00107831	Receptor-type tyrosine-protein phosphatase F precursor	R.GYQVTYVR.L	2	2.73	0.33	-1.26
IPI00107831	Receptor-type tyrosine-protein phosphatase F precursor	R.IISYTVVFR.D	2	1.94	0.09	-2.44
IPI00107831	Receptor-type tyrosine-protein phosphatase F precursor	R.NVLELSNVVR.S	1	2.98	0.32	-4.23
IPI00107831	Receptor-type tyrosine-protein phosphatase F precursor	R.NVLELSNVVR.S	2	3.57	0.26	-2.44
IPI00107831	Receptor-type tyrosine-protein phosphatase F precursor	R.SDM*GVGVFTPTIEAR.T	2	4.07	0.43	-3.03
IPI00107831	Receptor-type tyrosine-protein phosphatase F precursor	R.TATM*LCAAGGNPDPEISWFKDFLPVDPATSNGR.I	3	4.82	0.52	-3.38
IPI00107831	Receptor-type tyrosine-protein phosphatase F precursor	R.TATM*LCAAGGNPDPEISWFKDFLPVDPATSNGR.I	4	3.24	0.26	-2.71
IPI00107831	Receptor-type tyrosine-protein phosphatase F precursor	R.TGEQAPSSPPR.R	2	2.74	0.23	-2.51
IPI00107831	Receptor-type tyrosine-protein phosphatase F precursor	R.TGEQAPSSPPRR.V	3	2.68	0.27	-4.27
IPI00107831	Receptor-type tyrosine-protein phosphatase F precursor	R.TM*PVEQVFAK.N	2	2.01	0.17	-2.25
IPI00107831	Receptor-type tyrosine-protein phosphatase F precursor	R.VGGSM*LTPR.W	2	3.09	0.22	-3.15
IPI00107831	Receptor-type tyrosine-protein phosphatase F precursor	R.VLAFTAVGDGPPSPTIQVK.T	3	4.83	0.50	-4.50
IPI00107831	Receptor-type tyrosine-protein phosphatase F precursor	R.VLAVNSIGR.G	1	1.87	0.07	-1.34

IPI00107831	Receptor-type tyrosine-protein phosphatase F precursor	R.VLAVNSIGR.G	2	2.99	0.36	-1.27
IPI00107831	Receptor-type tyrosine-protein phosphatase F precursor	R.VLAVNSIGRGPPSEAVR.A	3	3.45	0.38	-2.87
IPI00107831	Receptor-type tyrosine-protein phosphatase F precursor	R.VYYTPDSR.R	2	2.18	0.31	-3.29
IPI00107831	Receptor-type tyrosine-protein phosphatase F precursor	R.WFYIVVVPIDR.V	2	3.76	0.45	-3.33
IPI00107831	Receptor-type tyrosine-protein phosphatase F precursor	R.YSAPANLYVR.E	1	1.79	0.17	-2.65
IPI00107831	Receptor-type tyrosine-protein phosphatase F precursor	R.YSAPANLYVR.E	2	2.87	0.33	-2.22
IPI00107831	Receptor-type tyrosine-protein phosphatase F precursor	R.YSIGGLSPFSEYAFR.V	2	4.41	0.51	-4.96
IPI00107831	Receptor-type tyrosine-protein phosphatase F precursor	S.GALQIESSEESDQGKYECVATNSAGTR.Y	3	6.82	0.64	-2.52
IPI00107886	Semaphorin 6B isoform 2	G.LFPEEPPPLSVAPR.D	2	3.02	0.33	-3.29
IPI00107886	Semaphorin 6B isoform 2	R.DYLNHYPVFVGSGPGR.L	2	3.81	0.31	-1.90
IPI00107886	Semaphorin 6B isoform 2	R.DYLNHYPVFVGSGPGR.L	3	3.01	0.09	-2.53
IPI00107886	Semaphorin 6B isoform 2	R.LTPAEGADDLNIQR.V	2	4.23	0.38	-2.86
IPI00107886	Semaphorin 6B isoform 2	R.SNPSDINVCR.M	2	1.97	0.25	-2.58
IPI00107886	Semaphorin 6B isoform 2	R.VELEPPTSTELR.Y	2	2.59	0.18	-2.87
IPI00141938	H2A histone family, member V isoform 2	R.AGLQFPVGR.I	2	2.49	0.10	-2.97
IPI00143753	Isoform 1 of U2-associated protein SR140	R.LKNPNAPMLPPPK.N	2	2.32	0.14	
IPI00144243	Human immunodeficiency virus type I enhancer-binding protein 2	K.CLELGVSM*TSVDDTETEEAENLEDLHKAAEK.H	4	2.35	0.12	-4.81
IPI00145805	Isoform 1 of TRAF2 and NCK-interacting protein kinase	R.AQRLKFLCER.N	2	2.25	0.06	-0.05
IPI00149044	Isoform 2 of Suppressor of hairy wing homolog 4	R.ACPKCNIHFNLLDPLK.N	2	2.42	0.10	-2.61
IPI00149097	Semaphorin-4A precursor	K.GLQDFDTLLLSGDGNTLYVGAR.E	2	4.34	0.46	-4.84
IPI00149097	Semaphorin-4A precursor	R.ALSFFHQK.G	2	1.57	0.16	-3.09
IPI00149375	Isoform 2 of Uncharacterized protein C11orf56	R.QPLLRHGPVR.E	2	2.22	0.05	-7.29
IPI00150881	Isoform 1 of Coiled-coil domain-containing protein C6orf204	R.M*ILEIQSM*QGKLSK.E	2	3.33	0.12	
IPI00151036	RING finger protein 13	K.DQLKKLPVHKFK.K	3	2.76	0.25	
IPI00151036	RING finger protein 13	K.KIDIPSVFIGESSANSLKDEFTYEK.G	3	4.73	0.25	
IPI00151990	Isoform 1 of Thioredoxin domain-containing protein 15 precursor	R.FSASLAPHFNSLPR.A	3	2.95	0.33	-0.29
IPI00152050	ataxin 2-binding protein 1 isoform 3	R.KIEVNNATARVM*TNK.K	2	1.13	0.09	1.70
IPI00152072	hypothetical protein LOC387758	K.SISYDLDGEESYGK.Y	2	2.40	0.13	
IPI00152145	Protein odd-skipped-related 1	K.LGRGEGPGSPAGGLGALLDVTKLSPEKKPTR.G	3	3.20	0.12	
IPI00152182	Isoform 1 of Kelch domain-containing protein 4	K.EVVAEDGTVVTIK.Q	2	3.05	0.24	

IPI00152216	Isoform 1 of Protein RIC-3 precursor	R.SHLAEAFAK.A	2	2.13	0.15	-1.65
IPI00152326	glutathione S-transferase M1 isoform 2	K.LGLDFPNLPYLIDGAHK.I	3	3.23	0.40	-2.11
IPI00152344	Pyridoxal phosphate phosphatase PHOSPHO2	Q.QGVNYTQIVYIGDGGNDVCPVTFLKNDDVAM*PR.K	3	3.75	0.17	-8.00
IPI00152418	Decay-accelerating factor splicing variant 4	K.CEESFVK.I	1	2.03	0.16	-3.40
IPI00152418	Decay-accelerating factor splicing variant 4	K.GFTM*IGEHSIYCTVNNDEGEWSGPPPECR.G	3	7.28	0.55	-2.41
IPI00152418	Decay-accelerating factor splicing variant 4	K.GFTM*IGEHSIYCTVNNDEGEWSGPPPECR.G	4	3.73	0.40	-2.19
IPI00152418	Decay-accelerating factor splicing variant 4	K.LTCLQNLK.W	2	2.67	0.06	0.33
IPI00152418	Decay-accelerating factor splicing variant 4	K.QPYITQNYFPVGTVVEY.E	2	3.21	0.43	0.13
IPI00152418	Decay-accelerating factor splicing variant 4	K.QPYITQNYFPVGTVVEYECRPGYR.R	3	2.86	0.28	-2.13
IPI00152418	Decay-accelerating factor splicing variant 4	K.WSTAVEFCK.K	2	3.11	0.43	-1.42
IPI00152418	Decay-accelerating factor splicing variant 4	R.DHYGYR.Q	1	1.97	0.22	-5.19
IPI00152418	Decay-accelerating factor splicing variant 4	R.DHYGYR.Q	2	1.55	0.08	-4.98
IPI00152418	Decay-accelerating factor splicing variant 4	R.EIYCPAPPQIDNGIIQGER.D	2	3.99	0.55	-4.78
IPI00152418	Decay-accelerating factor splicing variant 4	R.EPSLSPK.L	1	2.26	0.14	-3.11
IPI00152418	Decay-accelerating factor splicing variant 4	R.LNSASLKQPYITQNYFPVGTVVEYECRPGYR.R	3	4.12	0.36	-5.02
IPI00152418	Decay-accelerating factor splicing variant 4	R.LNSASLKQPYITQNYFPVGTVVEYECRPGYR.R	4	3.44	0.23	-2.86
IPI00152418	Decay-accelerating factor splicing variant 4	R.QSVTYACNK.G	2	1.95	0.26	0.49
IPI00152418	Decay-accelerating factor splicing variant 4	R.REPSLSPK.L	2	2.78	0.20	-3.72
IPI00152418	Decay-accelerating factor splicing variant 4	R.TSFPEDTVITYK.C	2	3.08	0.48	-4.46
IPI00152470	Prokineticin receptor 1	K.SSADLDLKTIGMPATEEVDCIR.L	3	2.85	0.10	0.81
IPI00152524	Isoform 3 of Neuropilin and tolloid-like protein 1 precursor	K.FFADGELESM*GFSAR.Y	2	4.75	0.54	-2.68
IPI00152524	Isoform 3 of Neuropilin and tolloid-like protein 1 precursor	R.DGPFGFSPIIGR.F	2	3.11	0.22	-5.28
IPI00152540	Isoform 1 of CD109 antigen precursor	K.ALSEFAALM*NTER.T	2	2.77	0.27	-2.70
IPI00152540	Isoform 1 of CD109 antigen precursor	K.FLIDTHNR.L	2	2.60	0.13	-3.36
IPI00152540	Isoform 1 of CD109 antigen precursor	K.IPVQLVFK.N	2	2.51	0.08	-1.90
IPI00152540	Isoform 1 of CD109 antigen precursor	K.LSDSWQPR.S	2	2.31	0.19	-1.34
IPI00152540	Isoform 1 of CD109 antigen precursor	K.SNLIQQWLSQQSDLGVISK.T	2	6.11	0.46	-5.73
IPI00152540	Isoform 1 of CD109 antigen precursor	K.SNLIQQWLSQQSDLGVISK.T	3	4.06	0.46	-2.54
IPI00152540	Isoform 1 of CD109 antigen precursor	K.SYSQSILLDLTDNR.L	2	4.41	0.40	-3.29
IPI00152540	Isoform 1 of CD109 antigen precursor	K.TLTLPSLPLNSADEIYELR.V	2	4.37	0.49	-4.91
IPI00152540	Isoform 1 of CD109 antigen precursor	K.TLTLPSLPLNSADEIYELR.V	3	4.78	0.34	-5.02
IPI00152540	Isoform 1 of CD109 antigen precursor	K.VSNTQDASVSIVDYYEPR.R	2	2.65	0.31	1.63
IPI00152540	Isoform 1 of CD109 antigen precursor	K.YTYGKPVK.G	2	2.13	0.10	-2.74
IPI00152540	Isoform 1 of CD109 antigen precursor	R.ADGNQLTLEER.R	2	2.46	0.08	-2.97
IPI00152540	Isoform 1 of CD109 antigen precursor	R.ISVFIQTDK.A	2	3.04	0.32	-1.73
IPI00152540	Isoform 1 of CD109 antigen precursor	R.KYQPNIDVQESIHFLESEFSR.G	3	4.79	0.43	-3.60
IPI00152540	Isoform 1 of CD109 antigen precursor	R.LKELSYM*VVSR.G	2	2.85	0.27	-3.13
IPI00152540	Isoform 1 of CD109 antigen precursor	R.LKELSYM*VVSR.G	3	2.39	0.09	-3.09
IPI00152540	Isoform 1 of CD109 antigen precursor	R.TNIQVTVTGPSSPSPVK.F	2	2.59	0.37	-3.64
IPI00152540	Isoform 1 of CD109 antigen precursor	R.VIHSELQGGNK.S	2	2.59	0.22	0.10

IPI00152540	Isoform 1 of CD109 antigen precursor	R.VQITAIGDVLGPSINGLASLIR.M	3	4.77	0.33	-2.69
IPI00152769	Isoform 1 of Trpc4-associated protein	R.KERLPLYLRLLQRMEHSK.K	3	3.43	0.09	
IPI00152847	WAP, kazal, immunoglobulin, kunitz and NTR domain-containing protein 2 precursor	K.GITLAVVTCR.Y	1	1.74	0.16	-2.22
IPI00152847	WAP, kazal, immunoglobulin, kunitz and NTR domain-containing protein 2 precursor	K.GITLAVVTCR.Y	2	3.95	0.40	-2.19
IPI00152847	WAP, kazal, immunoglobulin, kunitz and NTR domain-containing protein 2 precursor	K.TCDVLKEFLGLH.-	2	3.45	0.39	-1.97
IPI00152847	WAP, kazal, immunoglobulin, kunitz and NTR domain-containing protein 2 precursor	K.TCDVLKEFLGLH.-	3	3.20	0.24	-0.50
IPI00152847	WAP, kazal, immunoglobulin, kunitz and NTR domain-containing protein 2 precursor	R.ADFPLSVVR.G	2	3.05	0.28	-4.23
IPI00152847	WAP, kazal, immunoglobulin, kunitz and NTR domain-containing protein 2 precursor	R.ALVTVDEVLKDEK.M	2	2.91	0.23	-2.92
IPI00152847	WAP, kazal, immunoglobulin, kunitz and NTR domain-containing protein 2 precursor	R.ALVTVDEVLKDEK.M	3	2.24	0.21	-1.35
IPI00152847	WAP, kazal, immunoglobulin, kunitz and NTR domain-containing protein 2 precursor	R.CYM*DAEACSK.G	2	3.50	0.60	-2.75
IPI00152847	WAP, kazal, immunoglobulin, kunitz and NTR domain-containing protein 2 precursor	R.GNVVVTNIAQLVIYNAQLQDAGIYTCTAR.N	3	5.72	0.53	-4.81
IPI00152847	WAP, kazal, immunoglobulin, kunitz and NTR domain-containing protein 2 precursor	R.SDFVILGR.V	2	2.71	0.29	-2.75
IPI00152847	WAP, kazal, immunoglobulin, kunitz and NTR domain-containing protein 2 precursor	R.VSELTEEPDSGR.A	2	3.94	0.34	-3.52
IPI00152849	Isoform 1 of G2/mitotic-specific cyclin-B3	K.RKHATQGTM*SHLKKPLILQTTSGEK.S	4	2.63	0.14	-5.82
IPI00152850	junctional adhesion molecule 3 precursor	K.IQDEQTTYVFFDNK.I	2	4.44	0.37	-3.12
IPI00152850	junctional adhesion molecule 3 precursor	R.DSALYR.C	1	1.63	0.16	-4.17
IPI00152850	junctional adhesion molecule 3 precursor	R.NDVPLPTDSR.A	2	2.02	0.10	-2.50
IPI00152850	junctional adhesion molecule 3 precursor	R.RDSALYR.C	2	2.38	0.14	-4.84
IPI00153049	Isoform 2 of Matrix-remodeling-associated protein 8 precursor	R.AYGPLFLR.D	2	1.78	0.09	-1.46
IPI00153049	Isoform 2 of Matrix-remodeling-associated protein 8 precursor	R.GAPALLTCVNR.G	2	2.87	0.12	-1.94
IPI00153049	Isoform 2 of Matrix-remodeling-associated protein 8 precursor	R.LLDLYSAGEQR.V	2	3.85	0.41	-4.87
IPI00153049	Isoform 2 of Matrix-remodeling-associated protein 8 precursor	R.VAVGADAFER.G	2	2.77	0.27	-3.95
IPI00154528	Isoform 1 of Structural maintenance of chromosomes protein 6	K.FFMKATQLEQM*KEDYSYIMETK.E	3	2.36	0.08	0.38
IPI00154734	seizure related 6 homolog isoform 1	K.EGPWSPESESPM*LR.I	2	1.97	0.11	-3.47
IPI00154734	seizure related 6 homolog isoform 1	K.HFFVELSTDSSGAAAGM*ALR.Y	2	4.92	0.44	-6.67
IPI00154734	seizure related 6 homolog isoform 1	K.HFFVELSTDSSGAAAGM*ALR.Y	3	2.64	0.12	-2.65

IPI00154734	seizure related 6 homolog isoform 1	K.LLNHHPLLEE.F	1	2.53	0.16	-1.99
IPI00154734	seizure related 6 homolog isoform 1	K.LLNHHPLLEEFLQEGLEK.G	3	6.16	0.51	-2.95
IPI00154734	seizure related 6 homolog isoform 1	K.LLNHHPLLEEFLQEGLEK.G	4	3.62	0.19	-0.61
IPI00154734	seizure related 6 homolog isoform 1	K.VSLAEDDDR.L	2	3.17	0.32	-3.34
IPI00154734	seizure related 6 homolog isoform 1	K.VSLAEDDDRLIIR.N	2	3.35	0.24	-2.15
IPI00154734	seizure related 6 homolog isoform 1	K.VSLAEDDDRLIIR.N	3	2.76	0.30	-1.74
IPI00154734	seizure related 6 homolog isoform 1	L.SLEAPTVGK.G	1	1.93	0.18	-2.19
IPI00154734	seizure related 6 homolog isoform 1	R.AASLDGFYNS.R	1	2.20	0.29	-4.01
IPI00154734	seizure related 6 homolog isoform 1	R.AASLDGFYNSR.S	1	2.01	0.33	-3.72
IPI00154734	seizure related 6 homolog isoform 1	R.AASLDGFYNSR.S	2	3.63	0.40	-4.80
IPI00154734	seizure related 6 homolog isoform 1	R.GQDCIWGVHVEEDKR.I	4	2.58	0.17	-2.79
IPI00154734	seizure related 6 homolog isoform 1	R.IGPGDVLTFYDGDDLTAR.V	2	5.82	0.61	-5.42
IPI00154734	seizure related 6 homolog isoform 1	R.IGPGDVLTFYDGDDLTAR.V	3	4.93	0.46	-4.05
IPI00154734	seizure related 6 homolog isoform 1	R.NGDNVEAPPVYDSYEVEYLPIEGLLSSGK.H	2	4.02	0.54	-4.23
IPI00154734	seizure related 6 homolog isoform 1	R.NGDNVEAPPVYDSYEVEYLPIEGLLSSGK.H	3	6.00	0.45	-4.55
IPI00154734	seizure related 6 homolog isoform 1	R.RLISSPK.F	1	1.76	0.15	-4.10
IPI00154734	seizure related 6 homolog isoform 1	R.RPAYGDVTVTSLHPGGSAR.F	2	3.61	0.40	-4.57
IPI00154734	seizure related 6 homolog isoform 1	R.RPAYGDVTVTSLHPGGSAR.F	3	4.78	0.43	-3.74
IPI00154734	seizure related 6 homolog isoform 1	R.VLGQYSGPR.S	1	2.15	0.15	-0.94
IPI00154734	seizure related 6 homolog isoform 1	R.VLGQYSGPR.S	2	2.70	0.33	-2.47
IPI00154734	seizure related 6 homolog isoform 1	R.YEAFQQGHCYEPFVK.Y	2	4.35	0.45	-4.11
IPI00154742	IGL@ protein	K.AAPSVTLFPPSSEELQANK.A	1	4.04	0.50	
IPI00154742	IGL@ protein	K.AAPSVTLFPPSSEELQANK.A	2	3.15	0.34	-1.50
IPI00154742	IGL@ protein	K.AAPSVTLFPPSSEELQANK.A	3	3.32	0.13	
IPI00154742	IGL@ protein	K.ADSSPVKAGVETTTPSK.Q	1	3.85	0.37	
IPI00154742	IGL@ protein	K.ADSSPVKAGVETTTPSK.Q	2	3.50	0.38	
IPI00154742	IGL@ protein	K.ADSSPVKAGVETTTPSK.Q	3	3.46	0.35	
IPI00154742	IGL@ protein	K.ADSSPVKAGVETTTPSKQSNNK.Y	2	5.35	0.42	
IPI00154742	IGL@ protein	K.ADSSPVKAGVETTTPSKQSNNK.Y	3	4.61	0.23	
IPI00154742	IGL@ protein	K.AGVETTTPSK.Q	2	2.62	0.09	-3.23
IPI00154742	IGL@ protein	K.AGVETTTPSKQSNNK.Y	2	4.14	0.32	
IPI00154742	IGL@ protein	K.AGVETTTPSKQSNNKYAASSYLSLTPEQWK.S	3	5.47	0.40	
IPI00154742	IGL@ protein	K.ATLVCLISDFYPGAVTVAWK.A	2	5.07	0.50	
IPI00154742	IGL@ protein	K.ATLVCLISDFYPGAVTVAWK.A	3	3.53	0.31	-3.63
IPI00154742	IGL@ protein	K.ATLVCLISDFYPGAVTVAWKADSSPVK.A	3	3.81	0.20	
IPI00154742	IGL@ protein	K.QSNNKYAASSYLSLTPEQWK.S	2	5.38	0.37	
IPI00154742	IGL@ protein	K.QSNNKYAASSYLSLTPEQWK.S	3	4.26	0.33	
IPI00154742	IGL@ protein	K.YAASSYLSLTPEQWK.S	1	3.49	0.43	
IPI00154742	IGL@ protein	K.YAASSYLSLTPEQWK.S	2	5.52	0.47	
IPI00154742	IGL@ protein	K.YAASSYLSLTPEQWK.S	3	3.44	0.22	
IPI00154742	IGL@ protein	R.FSGSNSGNTATLTISR.V	1	3.22	0.40	
IPI00154742	IGL@ protein	R.FSGSNSGNTATLTISR.V	2	4.32	0.38	

IPI00154742	IGL@ protein	R.ITCGGNNIGSK.S	2	2.81	0.18	
IPI00154742	IGL@ protein	R.SYSCQVTHEGSTVEK.T	1	3.84	0.43	
IPI00154742	IGL@ protein	R.SYSCQVTHEGSTVEK.T	2	3.17	0.41	-4.10
IPI00154742	IGL@ protein	R.SYSCQVTHEGSTVEKTVAPTECS.-	2	5.35	0.45	
IPI00154742	IGL@ protein	R.SYSCQVTHEGSTVEKTVAPTECS.-	3	2.78	0.26	
IPI00154858	Platelet endothelial aggregation receptor 1 precursor	R.FGQDCAETCDCAPDAR.C	1	2.14	0.05	
IPI00155447	MMP28 protein	K.YGYLNEQVPK.A	2	1.98	0.16	-2.82
IPI00155729	Plexin-B3 precursor	K.LGQPVSAVAALQADGHM*IAFLGDTQGQLYK.V	3	4.87	0.49	-2.49
IPI00155729	Plexin-B3 precursor	R.ELPVPIYVTQGEAQR.L	2	3.55	0.39	-2.43
IPI00155729	Plexin-B3 precursor	R.QEQGQVTLSVPR.L	2	2.76	0.20	-1.87
IPI00155729	Plexin-B3 precursor	R.QLAGSQPFSSEGLGR.L	2	3.86	0.48	-2.99
IPI00155729	Plexin-B3 precursor	R.TLLASPFR.Y	2	2.09	0.16	-0.96
IPI00155729	Plexin-B3 precursor	R.TRPQAAPGEAAVLVVFGHAQR.T	3	6.13	0.53	-4.72
IPI00155729	Plexin-B3 precursor	R.TRPQAAPGEAAVLVVFGHAQR.T	4	2.82	0.27	-2.88
IPI00156171	Isoform 1 of Ectonucleotide pyrophosphatase/phosphodiesterase family member 2 precursor	C.PAGFVRPPLIIFSVDGFR.A	3	3.90	0.36	-4.42
IPI00156171	Isoform 1 of Ectonucleotide pyrophosphatase/phosphodiesterase family member 2 precursor	F.DYDYDGLHDTEDKIK.Q	2	3.46	0.44	-1.88
IPI00156171	Isoform 1 of Ectonucleotide pyrophosphatase/phosphodiesterase family member 2 precursor	F.LSNYLTNVDDITLVPGTLGR.I	2	3.89	0.48	-2.95
IPI00156171	Isoform 1 of Ectonucleotide pyrophosphatase/phosphodiesterase family member 2 precursor	I.DKIVGQLM*DGLK.Q	2	2.98	0.32	-1.95
IPI00156171	Isoform 1 of Ectonucleotide pyrophosphatase/phosphodiesterase family member 2 precursor	I.FDYDYDGLHDTEDKIK.Q	2	3.44	0.37	-5.92
IPI00156171	Isoform 1 of Ectonucleotide pyrophosphatase/phosphodiesterase family member 2 precursor	K.AAECPAGFVRPPLIIFSVDGFR.A	2	3.41	0.48	-3.20
IPI00156171	Isoform 1 of Ectonucleotide pyrophosphatase/phosphodiesterase family member 2 precursor	K.AAECPAGFVRPPLIIFSVDGFR.A	3	5.07	0.54	-6.71
IPI00156171	Isoform 1 of Ectonucleotide pyrophosphatase/phosphodiesterase family member 2 precursor	K.AGTFFWSVVIPHER.R	2	3.88	0.50	-4.30
IPI00156171	Isoform 1 of Ectonucleotide pyrophosphatase/phosphodiesterase family member 2 precursor	K.AGTFFWSVVIPHER.R	3	3.64	0.38	-3.43

IPI00156171	Isoform 1 of Ectonucleotide pyrophosphatase/phosphodiesterase family member 2 precursor	K.AGTFFWSVVIPHERR.I	3	2.36	0.24	-5.42
IPI00156171	Isoform 1 of Ectonucleotide pyrophosphatase/phosphodiesterase family member 2 precursor	K.CFFQGDHGFDNK.V	2	3.96	0.46	-2.89
IPI00156171	Isoform 1 of Ectonucleotide pyrophosphatase/phosphodiesterase family member 2 precursor	K.CFFQGDHGFDNKVNSM*QTVFVGYGPTFK.Y	3	3.99	0.37	-3.45
IPI00156171	Isoform 1 of Ectonucleotide pyrophosphatase/phosphodiesterase family member 2 precursor	K.CFFQGDHGFDNKVNSM*QTVFVGYGPTFK.Y	4	3.01	0.15	-4.96
IPI00156171	Isoform 1 of Ectonucleotide pyrophosphatase/phosphodiesterase family member 2 precursor	K.IVGQLM*DGLK.Q	1	1.92	0.16	-4.37
IPI00156171	Isoform 1 of Ectonucleotide pyrophosphatase/phosphodiesterase family member 2 precursor	K.IVGQLM*DGLK.Q	2	3.43	0.36	-3.09
IPI00156171	Isoform 1 of Ectonucleotide pyrophosphatase/phosphodiesterase family member 2 precursor	K.KPDQHFKPYLK.Q	2	3.78	0.19	-4.92
IPI00156171	Isoform 1 of Ectonucleotide pyrophosphatase/phosphodiesterase family member 2 precursor	K.KPDQHFKPYLK.Q	3	2.76	0.20	-4.13
IPI00156171	Isoform 1 of Ectonucleotide pyrophosphatase/phosphodiesterase family member 2 precursor	K.NDKQM*SYGFLFPPYLSSSPEAK.Y	2	5.27	0.57	-4.39
IPI00156171	Isoform 1 of Ectonucleotide pyrophosphatase/phosphodiesterase family member 2 precursor	K.NDKQM*SYGFLFPPYLSSSPEAK.Y	3	4.41	0.46	-3.41
IPI00156171	Isoform 1 of Ectonucleotide pyrophosphatase/phosphodiesterase family member 2 precursor	K.NKLDELNKR.L	2	2.97	0.13	-3.10
IPI00156171	Isoform 1 of Ectonucleotide pyrophosphatase/phosphodiesterase family member 2 precursor	K.QAEVSSVPDHLTSCVRPDVR.V	2	2.45	0.18	-3.52
IPI00156171	Isoform 1 of Ectonucleotide pyrophosphatase/phosphodiesterase family member 2 precursor	K.QGVKAGTFFWSVVIPHER.R	4	3.38	0.24	-2.10
IPI00156171	Isoform 1 of Ectonucleotide pyrophosphatase/phosphodiesterase family member 2 precursor	K.QM*SYGFLFPPYLSSSPEAK.Y	2	4.95	0.54	-4.82

IPI00156171	Isoform 1 of Ectonucleotide pyrophosphatase/phosphodiesterase family member 2 precursor	K.QM*SYGFLFPPYLSSSPEAK.Y	3	3.71	0.15	-4.41
IPI00156171	Isoform 1 of Ectonucleotide pyrophosphatase/phosphodiesterase family member 2 precursor	K.QYVEGSSIPVPTHYYSIITSCLDFTQPADK.C	3	4.55	0.42	-4.45
IPI00156171	Isoform 1 of Ectonucleotide pyrophosphatase/phosphodiesterase family member 2 precursor	K.SYTSCCHDFDELCLK.T	2	4.55	0.46	-0.09
IPI00156171	Isoform 1 of Ectonucleotide pyrophosphatase/phosphodiesterase family member 2 precursor	K.SYTSCCHDFDELCLK.T	3	2.16	0.22	-1.69
IPI00156171	Isoform 1 of Ectonucleotide pyrophosphatase/phosphodiesterase family member 2 precursor	K.TFPNLYTLATGLYPESH.G	2	3.51	0.35	-3.72
IPI00156171	Isoform 1 of Ectonucleotide pyrophosphatase/phosphodiesterase family member 2 precursor	K.TFPNLYTLATGLYPESHGIVGN.S	2	4.19	0.52	-5.02
IPI00156171	Isoform 1 of Ectonucleotide pyrophosphatase/phosphodiesterase family member 2 precursor	K.TFPNLYTLATGLYPESHGIVGNSM*YDPVFDATFHLR.G	3	5.63	0.50	-3.56
IPI00156171	Isoform 1 of Ectonucleotide pyrophosphatase/phosphodiesterase family member 2 precursor	K.TFPNLYTLATGLYPESHGIVGNSM*YDPVFDATFHLR.G	4	2.73	0.42	-4.05
IPI00156171	Isoform 1 of Ectonucleotide pyrophosphatase/phosphodiesterase family member 2 precursor	K.TYLHTYESEI.-	1	2.74	0.38	-4.10
IPI00156171	Isoform 1 of Ectonucleotide pyrophosphatase/phosphodiesterase family member 2 precursor	K.TYLHTYESEI.-	2	3.08	0.36	-2.57
IPI00156171	Isoform 1 of Ectonucleotide pyrophosphatase/phosphodiesterase family member 2 precursor	K.VNSM*QTVFVGYGPTFK.Y	2	4.57	0.51	-4.65
IPI00156171	Isoform 1 of Ectonucleotide pyrophosphatase/phosphodiesterase family member 2 precursor	K.YDAFLVTNM*VPM*YPA.F	2	4.18	0.44	-1.28
IPI00156171	Isoform 1 of Ectonucleotide pyrophosphatase/phosphodiesterase family member 2 precursor	K.YDAFLVTNM*VPM*YPAF.K	2	4.11	0.56	-3.03
IPI00156171	Isoform 1 of Ectonucleotide pyrophosphatase/phosphodiesterase family member 2 precursor	K.YDAFLVTNM*VPM*YPAFK.R	2	5.18	0.54	-5.59

IPI00156171	Isoform 1 of Ectonucleotide pyrophosphatase/phosphodiesterase family member 2 precursor	K.YDAFLVTNM*VPM*YPAFK.R	3	3.31	0.34	-3.66
IPI00156171	Isoform 1 of Ectonucleotide pyrophosphatase/phosphodiesterase family member 2 precursor	K.YDAFLVTNM*VPM*YPAFKR.V	2	3.55	0.49	-4.73
IPI00156171	Isoform 1 of Ectonucleotide pyrophosphatase/phosphodiesterase family member 2 precursor	K.YDAFLVTNM*VPM*YPAFKR.V	3	5.03	0.51	-4.07
IPI00156171	Isoform 1 of Ectonucleotide pyrophosphatase/phosphodiesterase family member 2 precursor	K.YGPFGPEM*TNPLR.E	2	3.34	0.40	-3.44
IPI00156171	Isoform 1 of Ectonucleotide pyrophosphatase/phosphodiesterase family member 2 precursor	K.YGPFGPEM*TNPLREIDK.I	2	3.20	0.23	-3.41
IPI00156171	Isoform 1 of Ectonucleotide pyrophosphatase/phosphodiesterase family member 2 precursor	K.YGPFGPEM*TNPLREIDK.I	3	1.80	0.16	-1.64
IPI00156171	Isoform 1 of Ectonucleotide pyrophosphatase/phosphodiesterase family member 2 precursor	K.YGPFGPEM*TNPLREIDKIVGQLM*DGLK.Q	3	4.89	0.35	-4.93
IPI00156171	Isoform 1 of Ectonucleotide pyrophosphatase/phosphodiesterase family member 2 precursor	K.YGPFGPEM*TNPLREIDKIVGQLM*DGLK.Q	4	2.75	0.25	-4.03
IPI00156171	Isoform 1 of Ectonucleotide pyrophosphatase/phosphodiesterase family member 2 precursor	M.SYGFLFPPYLSSSPEAK.Y	2	3.49	0.41	-5.28
IPI00156171	Isoform 1 of Ectonucleotide pyrophosphatase/phosphodiesterase family member 2 precursor	R.CFELQEAGPPDCR.C	2	5.22	0.56	-3.33
IPI00156171	Isoform 1 of Ectonucleotide pyrophosphatase/phosphodiesterase family member 2 precursor	R.CVNVIFVGDHGM*EDVTCDR.T	2	5.49	0.58	-2.79
IPI00156171	Isoform 1 of Ectonucleotide pyrophosphatase/phosphodiesterase family member 2 precursor	R.CVNVIFVGDHGM*EDVTCDR.T	3	3.67	0.34	-3.25
IPI00156171	Isoform 1 of Ectonucleotide pyrophosphatase/phosphodiesterase family member 2 precursor	R.DIEHLTSLDFFR.K	1	3.49	0.53	-2.14
IPI00156171	Isoform 1 of Ectonucleotide pyrophosphatase/phosphodiesterase family member 2 precursor	R.DIEHLTSLDFFR.K	2	4.04	0.46	-5.32

IPI00156171	Isoform 1 of Ectonucleotide pyrophosphatase/phosphodiesterase family member 2 precursor	R.DIEHLTSLDFFR.K	3	4.67	0.29	-1.15
IPI00156171	Isoform 1 of Ectonucleotide pyrophosphatase/phosphodiesterase family member 2 precursor	R.DIEHLTSLDFFRK.T	2	3.78	0.40	-3.88
IPI00156171	Isoform 1 of Ectonucleotide pyrophosphatase/phosphodiesterase family member 2 precursor	R.DIEHLTSLDFFRK.T	3	3.52	0.40	-1.84
IPI00156171	Isoform 1 of Ectonucleotide pyrophosphatase/phosphodiesterase family member 2 precursor	R.DIEHLTSLDFFRK.T	4	2.09	0.27	-3.04
IPI00156171	Isoform 1 of Ectonucleotide pyrophosphatase/phosphodiesterase family member 2 precursor	R.EIDKIVGQLM*DGLK.Q	2	4.20	0.41	-3.54
IPI00156171	Isoform 1 of Ectonucleotide pyrophosphatase/phosphodiesterase family member 2 precursor	R.EIDKIVGQLM*DGLK.Q	3	2.93	0.31	-2.19
IPI00156171	Isoform 1 of Ectonucleotide pyrophosphatase/phosphodiesterase family member 2 precursor	R.EIDKIVGQLM*DGLKQLK.L	3	2.65	0.17	-3.22
IPI00156171	Isoform 1 of Ectonucleotide pyrophosphatase/phosphodiesterase family member 2 precursor	R.GDCCTNYQVVCK.G	2	3.61	0.41	-2.75
IPI00156171	Isoform 1 of Ectonucleotide pyrophosphatase/phosphodiesterase family member 2 precursor	R.IEDIHLLVER.R	1	2.56	0.26	-2.97
IPI00156171	Isoform 1 of Ectonucleotide pyrophosphatase/phosphodiesterase family member 2 precursor	R.IEDIHLLVER.R	2	3.91	0.27	-1.16
IPI00156171	Isoform 1 of Ectonucleotide pyrophosphatase/phosphodiesterase family member 2 precursor	R.IEDIHLLVER.R	3	3.76	0.32	-2.93
IPI00156171	Isoform 1 of Ectonucleotide pyrophosphatase/phosphodiesterase family member 2 precursor	R.KPLDVYK.K	1	2.48	0.15	-2.51
IPI00156171	Isoform 1 of Ectonucleotide pyrophosphatase/phosphodiesterase family member 2 precursor	R.KPLDVYK.K	2	2.76	0.11	-2.47
IPI00156171	Isoform 1 of Ectonucleotide pyrophosphatase/phosphodiesterase family member 2 precursor	R.KPLDVYKKPSGK.C	2	3.36	0.41	-3.88

IPI00156171	Isoform 1 of Ectonucleotide pyrophosphatase/phosphodiesterase family member 2 precursor	R.NGVNVISGPIFDYDYDGLHDTEDK.I	2	5.15	0.56	-3.76
IPI00156171	Isoform 1 of Ectonucleotide pyrophosphatase/phosphodiesterase family member 2 precursor	R.NGVNVISGPIFDYDYDGLHDTEDK.I	3	3.48	0.25	-5.19
IPI00156171	Isoform 1 of Ectonucleotide pyrophosphatase/phosphodiesterase family member 2 precursor	R.NGVNVISGPIFDYDYDGLHDTEDKIK.Q	2	4.95	0.52	-6.73
IPI00156171	Isoform 1 of Ectonucleotide pyrophosphatase/phosphodiesterase family member 2 precursor	R.NGVNVISGPIFDYDYDGLHDTEDKIK.Q	3	4.82	0.49	-4.02
IPI00156171	Isoform 1 of Ectonucleotide pyrophosphatase/phosphodiesterase family member 2 precursor	R.NGVNVISGPIFDYDYDGLHDTEDKIK.Q	4	4.22	0.33	-4.38
IPI00156171	Isoform 1 of Ectonucleotide pyrophosphatase/phosphodiesterase family member 2 precursor	R.RIEDIHLLVER.R	1	2.06	0.22	-3.60
IPI00156171	Isoform 1 of Ectonucleotide pyrophosphatase/phosphodiesterase family member 2 precursor	R.RIEDIHLLVER.R	2	3.83	0.34	-4.33
IPI00156171	Isoform 1 of Ectonucleotide pyrophosphatase/phosphodiesterase family member 2 precursor	R.RIEDIHLLVER.R	3	2.87	0.25	-2.87
IPI00156171	Isoform 1 of Ectonucleotide pyrophosphatase/phosphodiesterase family member 2 precursor	R.RIEDIHLLVERR.W	2	2.64	0.25	-4.85
IPI00156171	Isoform 1 of Ectonucleotide pyrophosphatase/phosphodiesterase family member 2 precursor	R.SYPEILTLK.T	2	2.48	0.10	-2.75
IPI00156171	Isoform 1 of Ectonucleotide pyrophosphatase/phosphodiesterase family member 2 precursor	R.TEFLSNYLTNVDDITLVPGTLGR.I	2	4.76	0.50	-5.12
IPI00156171	Isoform 1 of Ectonucleotide pyrophosphatase/phosphodiesterase family member 2 precursor	R.TEFLSNYLTNVDDITLVPGTLGR.I	3	4.27	0.42	-3.30
IPI00156171	Isoform 1 of Ectonucleotide pyrophosphatase/phosphodiesterase family member 2 precursor	R.TEFLSNYLTNVDDITLVPGTLGR.I	4	3.41	0.24	-4.35
IPI00156171	Isoform 1 of Ectonucleotide pyrophosphatase/phosphodiesterase family member 2 precursor	R.TNTFRPTM*PEEVTRPNYPGIM*YLQSDFDLGCTCDDKVEPK.N	4	3.52	0.18	-3.53

IPI00156171	Isoform 1 of Ectonucleotide pyrophosphatase/phosphodiesterase family member 2 precursor	R.TNTFRPTM*PEEVTRPNYPGIM*YLQSDFDLGCTCDDKVEPK.N	5	4.96	0.39	-0.26
IPI00156171	Isoform 1 of Ectonucleotide pyrophosphatase/phosphodiesterase family member 2 precursor	R.VRDIEHLTSLDFFR.K	2	4.23	0.45	-5.60
IPI00156171	Isoform 1 of Ectonucleotide pyrophosphatase/phosphodiesterase family member 2 precursor	R.VRDIEHLTSLDFFR.K	3	3.93	0.47	-5.61
IPI00156171	Isoform 1 of Ectonucleotide pyrophosphatase/phosphodiesterase family member 2 precursor	R.VRDIEHLTSLDFFR.K	4	3.05	0.18	-4.53
IPI00156171	Isoform 1 of Ectonucleotide pyrophosphatase/phosphodiesterase family member 2 precursor	R.VRDIEHLTSLDFFRK.T	2	2.77	0.32	-5.23
IPI00156171	Isoform 1 of Ectonucleotide pyrophosphatase/phosphodiesterase family member 2 precursor	R.VRDIEHLTSLDFFRK.T	3	4.98	0.47	-4.38
IPI00156171	Isoform 1 of Ectonucleotide pyrophosphatase/phosphodiesterase family member 2 precursor	R.VRDIEHLTSLDFFRK.T	4	4.25	0.44	-5.05
IPI00156171	Isoform 1 of Ectonucleotide pyrophosphatase/phosphodiesterase family member 2 precursor	R.VSPSFSQNCLAYK.N	1	1.90	0.33	1.67
IPI00156171	Isoform 1 of Ectonucleotide pyrophosphatase/phosphodiesterase family member 2 precursor	R.VSPSFSQNCLAYK.N	2	4.15	0.47	-2.45
IPI00156171	Isoform 1 of Ectonucleotide pyrophosphatase/phosphodiesterase family member 2 precursor	R.VSPSFSQNCLAYKNDK.Q	2	4.72	0.41	-4.90
IPI00156171	Isoform 1 of Ectonucleotide pyrophosphatase/phosphodiesterase family member 2 precursor	R.VWNYFQR.V	1	2.16	0.18	-1.43
IPI00156171	Isoform 1 of Ectonucleotide pyrophosphatase/phosphodiesterase family member 2 precursor	R.VWNYFQR.V	2	2.26	0.08	-0.39
IPI00156171	Isoform 1 of Ectonucleotide pyrophosphatase/phosphodiesterase family member 2 precursor	R.WWGGQPLWITATK.Q	1	2.38	0.42	-2.22
IPI00156171	Isoform 1 of Ectonucleotide pyrophosphatase/phosphodiesterase family member 2 precursor	R.WWGGQPLWITATK.Q	2	4.32	0.46	-3.60

IPI00156171	Isoform 1 of Ectonucleotide pyrophosphatase/phosphodiesterase family member 2 precursor	R.WWGGQPLWITATK.Q	3	2.44	0.11	-1.72
IPI00156171	Isoform 1 of Ectonucleotide pyrophosphatase/phosphodiesterase family member 2 precursor	V.RDIEHLTSLDFFR.K	2	2.98	0.25	-2.44
IPI00156171	Isoform 1 of Ectonucleotide pyrophosphatase/phosphodiesterase family member 2 precursor	Y.GFLFPPYLSSSPEAK.Y	2	3.22	0.38	-2.53
IPI00157414	Ectonucleotide pyrophosphatase/phosphodiesterase family member 6 precursor	K.DALKAVDTVLK.Y	2	2.76	0.10	-1.76
IPI00157414	Ectonucleotide pyrophosphatase/phosphodiesterase family member 6 precursor	K.FVSPLTLVADEGWFITENR.E	2	4.52	0.50	-4.89
IPI00157414	Ectonucleotide pyrophosphatase/phosphodiesterase family member 6 precursor	K.FVSPLTLVADEGWFITENR.E	3	5.14	0.41	-3.88
IPI00157414	Ectonucleotide pyrophosphatase/phosphodiesterase family member 6 precursor	K.HSEIYNK.L	2	2.42	0.11	-3.31
IPI00157414	Ectonucleotide pyrophosphatase/phosphodiesterase family member 6 precursor	K.LLVFLLDGFR.S	2	3.78	0.46	-3.63
IPI00157414	Ectonucleotide pyrophosphatase/phosphodiesterase family member 6 precursor	K.NVPTDINFANAVSDALDSFK.S	2	6.52	0.62	-3.85
IPI00157414	Ectonucleotide pyrophosphatase/phosphodiesterase family member 6 precursor	K.YISLNDLQQVK.D	2	3.35	0.31	-3.30
IPI00157414	Ectonucleotide pyrophosphatase/phosphodiesterase family member 6 precursor	R.ADLAAIYHER.I	2	3.41	0.25	-1.70
IPI00157414	Ectonucleotide pyrophosphatase/phosphodiesterase family member 6 precursor	R.GIFLAFGPDFK.S	2	3.29	0.36	-3.22
IPI00157414	Ectonucleotide pyrophosphatase/phosphodiesterase family member 6 precursor	R.GVKVDYLTPDFPSLSYPNYYTLM*TGR.H	3	4.98	0.48	-3.43
IPI00157414	Ectonucleotide pyrophosphatase/phosphodiesterase family member 6 precursor	R.IDVEGHHYGPASPQRK.D	3	3.25	0.23	-3.72

IPI00157414	Ectonucleotide pyrophosphatase/phosphodiesterase family member 6 precursor	R.SDYISDEALESLPGFK.E	2	3.77	0.30	-5.41
IPI00157414	Ectonucleotide pyrophosphatase/phosphodiesterase family member 6 precursor	R.SDYISDEALESLPGFKEIVSR.G	2	4.27	0.42	-5.21
IPI00157414	Ectonucleotide pyrophosphatase/phosphodiesterase family member 6 precursor	R.SDYISDEALESLPGFKEIVSR.G	3	3.76	0.48	-5.51
IPI00157454	Isoform 1 of Heparan-sulfate 6-O-sulfotransferase 2	K.TGGTTFGR.H	1	1.24	0.17	-0.11
IPI00157454	Isoform 1 of Heparan-sulfate 6-O-sulfotransferase 2	R.ETWLFSR.F	1	1.99	0.19	-2.50
IPI00157454	Isoform 1 of Heparan-sulfate 6-O-sulfotransferase 2	R.KTQYLFEK.T	2	2.11	0.07	2.51
IPI00158992	snRNA-activating protein complex subunit 4	R.SGSQCLSKWKIM*M*GKKQGLRR.R	2	1.70	0.18	
IPI00159049	SET-binding protein	K.ERSSYDSSMSPGMPSPHLK.V	2	2.67	0.22	2.09
IPI00159927	Neurocan core protein precursor	A.M*AVTEM*LGSGQSR.S	2	3.99	0.46	-2.68
IPI00159927	Neurocan core protein precursor	A.VTEM*LGSGQSR.S	2	2.91	0.27	-3.06
IPI00159927	Neurocan core protein precursor	G.EQGTQDITDASER.G	2	3.64	0.52	-4.05
IPI00159927	Neurocan core protein precursor	K.GTVLCGPPPAVENASLIGAR.K	2	5.32	0.49	-4.32
IPI00159927	Neurocan core protein precursor	K.GTVLCGPPPAVENASLIGAR.K	3	5.35	0.43	-1.28
IPI00159927	Neurocan core protein precursor	K.LGSGSVQAALAELVALPCLFTLQPR.P	2	3.86	0.45	-2.16
IPI00159927	Neurocan core protein precursor	K.LGSGSVQAALAELVALPCLFTLQPR.P	3	3.90	0.33	-4.24
IPI00159927	Neurocan core protein precursor	K.YNVHATVR.Y	1	2.52	0.22	-4.90
IPI00159927	Neurocan core protein precursor	K.YNVHATVR.Y	2	2.33	0.23	-3.46
IPI00159927	Neurocan core protein precursor	Q.GTQDITDASER.G	2	3.57	0.28	-3.05
IPI00159927	Neurocan core protein precursor	R.AHHPTSQHGDLETPSSGDEGEILSAEGPPVR.E	3	6.10	0.54	-5.19
IPI00159927	Neurocan core protein precursor	R.APVLELEK.A	1	2.05	0.09	-3.18
IPI00159927	Neurocan core protein precursor	R.ASDSGLYR.C	1	1.48	0.23	-2.48
IPI00159927	Neurocan core protein precursor	R.ASDSGLYR.C	2	3.13	0.19	-2.56
IPI00159927	Neurocan core protein precursor	R.CGGPAPGVR.T	2	2.54	0.18	-2.89
IPI00159927	Neurocan core protein precursor	R.DFQWTDNTGLQFENWR.E	3	5.09	0.37	-3.63
IPI00159927	Neurocan core protein precursor	R.DRYALTFAEAQEACR.L	2	4.51	0.49	-2.89
IPI00159927	Neurocan core protein precursor	R.DRYALTFAEAQEACR.L	3	3.90	0.29	0.54
IPI00159927	Neurocan core protein precursor	R.ELGGEVFYVGPAR.R	1	2.88	0.48	-3.57
IPI00159927	Neurocan core protein precursor	R.ELGGEVFYVGPAR.R	2	4.25	0.45	-4.05
IPI00159927	Neurocan core protein precursor	R.ELGGEVFYVGPARR.L	3	1.80	0.16	-1.44
IPI00159927	Neurocan core protein precursor	R.ENQPDNFFAGGEDCVVM*VAHESGR.W	3	1.84	0.21	-5.09
IPI00159927	Neurocan core protein precursor	R.GIEDEQDLVPLEVTGVVFHYR.S	2	5.30	0.57	-4.03
IPI00159927	Neurocan core protein precursor	R.GIEDEQDLVPLEVTGVVFHYR.S	3	4.65	0.46	-4.65
IPI00159927	Neurocan core protein precursor	R.LSSAIIAAPR.H	1	2.77	0.18	-2.31

IPI00159927	Neurocan core protein precursor	R.LSSAIIAAPR.H	2	3.68	0.18	-1.75
IPI00159927	Neurocan core protein precursor	R.NPQELYDVYCFAR.E	2	4.80	0.51	-4.43
IPI00159927	Neurocan core protein precursor	R.NPQELYDVYCFAR.E	3	2.68	0.11	-1.44
IPI00159927	Neurocan core protein precursor	R.QDLPILVAK.D	2	1.76	0.15	-1.75
IPI00159927	Neurocan core protein precursor	R.QDLPILVAKDNVVR.V	2	2.06	0.21	-2.55
IPI00159927	Neurocan core protein precursor	R.RLTLAGAR.A	2	3.35	0.11	-4.82
IPI00159927	Neurocan core protein precursor	R.RNPQELYDVYCFAR.E	3	3.21	0.23	-3.99
IPI00159927	Neurocan core protein precursor	R.TASGQRQDLPILVAK.D	3	2.15	0.12	-1.54
IPI00159927	Neurocan core protein precursor	R.TGFPSPAER.F	1	2.10	0.11	-2.53
IPI00159927	Neurocan core protein precursor	R.TGFPSPAER.F	2	2.37	0.15	-2.48
IPI00159927	Neurocan core protein precursor	R.VSLPSYPR.R	1	1.47	0.11	-3.24
IPI00159927	Neurocan core protein precursor	R.VSLPSYPR.R	2	2.49	0.23	-2.19
IPI00159927	Neurocan core protein precursor	R.WNDVPCNYNLPYVCK.K	2	4.46	0.53	-4.15
IPI00159927	Neurocan core protein precursor	R.YALTFAEAQEACR.L	2	4.49	0.43	-2.88
IPI00159927	Neurocan core protein precursor	R.YPIQTPR.R	2	2.02	0.09	-2.89
IPI00160369	PRKCA-binding protein	K.VKHRLVENM*SSGTADALGLSR.A	3	3.37	0.17	
IPI00160552	Isoform 1 of Tenascin-R precursor	K.ASGPIDHYR.I	1	1.64	0.08	-3.08
IPI00160552	Isoform 1 of Tenascin-R precursor	K.EQPVVFNHVYNI.N	2	2.98	0.37	-2.45
IPI00160552	Isoform 1 of Tenascin-R precursor	K.GHEFSIPFVEM*K.M	2	3.68	0.46	-3.59
IPI00160552	Isoform 1 of Tenascin-R precursor	K.VATHLSTPQGLQFK.T	2	3.41	0.36	-2.88
IPI00160552	Isoform 1 of Tenascin-R precursor	K.VATHLSTPQGLQFK.T	3	3.35	0.39	-1.74
IPI00160552	Isoform 1 of Tenascin-R precursor	K.VDFILLK.Y	2	3.13	0.11	-3.66
IPI00160552	Isoform 1 of Tenascin-R precursor	K.VVYSTLAGEQYHEVLVPR.G	2	4.36	0.48	-2.20
IPI00160552	Isoform 1 of Tenascin-R precursor	K.VVYSTLAGEQYHEVLVPR.G	3	4.95	0.54	-1.57
IPI00160552	Isoform 1 of Tenascin-R precursor	R.AKVDFILLK.Y	2	2.56	0.20	-1.41
IPI00160552	Isoform 1 of Tenascin-R precursor	R.DGQEAAFASYDR.F	2	4.09	0.52	-2.83
IPI00160552	Isoform 1 of Tenascin-R precursor	R.GRQQSLESTVDAFTGFRPISHL.H	3	4.10	0.37	-1.32
IPI00160552	Isoform 1 of Tenascin-R precursor	R.ITFTPSSGIASEVTVPK.D	2	4.16	0.38	-2.42
IPI00160552	Isoform 1 of Tenascin-R precursor	R.LDSSVVPNTVTEFTITR.L	2	3.95	0.42	-4.66
IPI00160552	Isoform 1 of Tenascin-R precursor	R.LNPATEYEISLNSVR.G	2	5.10	0.51	-3.31
IPI00160552	Isoform 1 of Tenascin-R precursor	R.LNPATEYEISLNSVR.G	3	2.97	0.23	-2.30
IPI00160552	Isoform 1 of Tenascin-R precursor	R.QSALISWQPPR.A	2	2.65	0.23	-4.06
IPI00160552	Isoform 1 of Tenascin-R precursor	R.QSALISWQPPRAEIENYVLTYK.S	3	3.24	0.31	-2.44
IPI00160552	Isoform 1 of Tenascin-R precursor	R.RQNGQTDFFR.K	2	2.25	0.06	-3.28
IPI00160552	Isoform 1 of Tenascin-R precursor	R.SPPTSASVSTVIDGPTQILVR.D	2	4.24	0.35	-1.62
IPI00160552	Isoform 1 of Tenascin-R precursor	R.TSYTLTDLEPGAEYIISVTAER.G	2	4.82	0.55	-5.22
IPI00160552	Isoform 1 of Tenascin-R precursor	R.VGFGNVEDEFWLGLDNIHR.I	2	4.81	0.49	-1.27
IPI00160552	Isoform 1 of Tenascin-R precursor	R.VGFGNVEDEFWLGLDNIHR.I	3	5.58	0.53	-3.95
IPI00160552	Isoform 1 of Tenascin-R precursor	R.VSYRPTQVGR.L	2	2.98	0.11	-0.60
IPI00160552	Isoform 1 of Tenascin-R precursor	R.YEVSVSAVR.G	2	2.34	0.17	-2.90
IPI00161119	Isoform 1 of NF-kappa-B inhibitor beta	R.TPLGSAMLRPNPILAR.L	2	2.41	0.15	
IPI00162329	Isoform 1 of Transmembrane protein 25 precursor	G.ELEPQIDGQTWAER.A	2	3.69	0.49	-3.41

IPI00162329	Isoform 1 of Transmembrane protein 25 precursor	R.HPSLISSDSNNLK.L	2	1.85	0.07	-2.18
IPI00162329	Isoform 1 of Transmembrane protein 25 precursor	R.LAWYLDGQLQEASTSR.L	2	4.71	0.33	-1.71
IPI00162329	Isoform 1 of Transmembrane protein 25 precursor	R.VAGGPGTPR.L	2	2.27	0.32	-2.70
IPI00162547	latrophilin 3 precursor	K.DSLVDVPFPNSYQYIAAVDYNPR.D	2	4.00	0.47	-2.01
IPI00162547	latrophilin 3 precursor	K.DSLVDVPFPNSYQYIAAVDYNPR.D	3	4.00	0.35	-1.19
IPI00162547	latrophilin 3 precursor	K.LPHRVDGTGFVVYDGALFFNKER.T	4	3.06	0.16	-2.36
IPI00162547	latrophilin 3 precursor	K.SGEAIIANANYHDTSPYR.W	2	4.98	0.55	-2.43
IPI00162547	latrophilin 3 precursor	K.SGEAIIANANYHDTSPYR.W	3	2.74	0.31	-1.88
IPI00162547	latrophilin 3 precursor	K.YLEVQYECVPYKVEQK.V	2	4.70	0.41	-1.37
IPI00162547	latrophilin 3 precursor	K.YLEVQYECVPYKVEQK.V	3	2.88	0.11	-1.68
IPI00162547	latrophilin 3 precursor	M.IVISQLNPYTLR.F	1	2.56	0.17	-1.54
IPI00162547	latrophilin 3 precursor	M.IVISQLNPYTLR.F	2	3.88	0.38	-4.45
IPI00162547	latrophilin 3 precursor	R.CPGTDVIM*IESANYGR.T	2	3.68	0.30	-2.71
IPI00162547	latrophilin 3 precursor	R.CPGTDVIM*IESANYGR.T	3	4.20	0.32	-3.93
IPI00162547	latrophilin 3 precursor	R.DNLLYVWNNYHVVK.Y	2	3.62	0.36	-1.84
IPI00162547	latrophilin 3 precursor	R.DNLLYVWNNYHVVK.Y	3	3.03	0.13	0.34
IPI00162547	latrophilin 3 precursor	R.IKSGEAIIANANYHDTSPYR.W	2	5.85	0.62	-2.28
IPI00162547	latrophilin 3 precursor	R.IKSGEAIIANANYHDTSPYR.W	3	3.35	0.33	-2.35
IPI00162547	latrophilin 3 precursor	R.TDDKICDSDPAQM*ENIR.C	3	4.29	0.40	-2.56
IPI00162547	latrophilin 3 precursor	R.TDTLTEYSSKDDFIAGRPTTTYK.L	3	4.73	0.34	-3.96
IPI00162547	latrophilin 3 precursor	R.TDTLTEYSSKDDFIAGRPTTTYKLPHR.V	3	5.59	0.41	-3.99
IPI00162547	latrophilin 3 precursor	R.TDTLTEYSSKDDFIAGRPTTTYKLPHR.V	4	4.57	0.47	-2.70
IPI00162547	latrophilin 3 precursor	R.VDGTGFVVYDGALFFNKER.T	3	3.16	0.27	-2.55
IPI00162735	Isoform 2 of Attractin precursor	K.CFSSDFM*AYDIACDR.W	2	3.79	0.38	
IPI00162735	Isoform 2 of Attractin precursor	K.CTWLIEGQPNR.I	2	2.52	0.25	-1.78
IPI00162735	Isoform 2 of Attractin precursor	K.DSFSNEKFDFR.N	2	2.13	0.15	-1.18
IPI00162735	Isoform 2 of Attractin precursor	K.EQYAVVGHSAHIVTLK.N	2	4.01	0.38	-3.27
IPI00162735	Isoform 2 of Attractin precursor	K.GVKGDECQLCEVENR.Y	2	4.88	0.47	
IPI00162735	Isoform 2 of Attractin precursor	K.GVKGDECQLCEVENR.Y	3	4.39	0.14	
IPI00162735	Isoform 2 of Attractin precursor	K.HCETCISGFYGDPTNGGK.C	2	4.94	0.43	
IPI00162735	Isoform 2 of Attractin precursor	K.ITTAKENYDNAK.L	2	3.94	0.45	-3.21
IPI00162735	Isoform 2 of Attractin precursor	K.LTLTPWVGLR.K	2	2.62	0.22	-2.41
IPI00162735	Isoform 2 of Attractin precursor	K.TNIKEYKDSFSNEK.F	2	4.56	0.37	-3.45
IPI00162735	Isoform 2 of Attractin precursor	K.TNIKEYKDSFSNEK.F	3	3.87	0.39	-2.55
IPI00162735	Isoform 2 of Attractin precursor	K.TNIKEYKDSFSNEKFDFR.N	3	2.66	0.19	-4.84
IPI00162735	Isoform 2 of Attractin precursor	K.TNIKEYKDSFSNEKFDFR.N	4	2.41	0.10	-4.54
IPI00162735	Isoform 2 of Attractin precursor	R.ALYVHGGYK.A	1	2.29	0.32	-3.97
IPI00162735	Isoform 2 of Attractin precursor	R.ALYVHGGYK.A	2	1.94	0.12	-1.44
IPI00162735	Isoform 2 of Attractin precursor	R.CNPGTGQCVCPAGWVGEQCQHCGGR.F	3	3.63	0.31	
IPI00162735	Isoform 2 of Attractin precursor	R.DLDM*FINASK.N	2	3.25	0.39	-3.44
IPI00162735	Isoform 2 of Attractin precursor	R.FRLTGSSGFVTDGPGNYKYK.T	3	3.32	0.22	
IPI00162735	Isoform 2 of Attractin precursor	R.LADDLYR.Y	2	2.64	0.22	-5.11

IPI00162735	Isoform 2 of Attractin precursor	R.LADDLYRYDVDTQM*WTILK.D	3	2.78	0.12	-3.63
IPI00162735	Isoform 2 of Attractin precursor	R.LTGSSGFVTDGPGNYK.Y	2	4.63	0.44	-4.40
IPI00162735	Isoform 2 of Attractin precursor	R.NHNALLASLTTQK.K	2	4.25	0.53	-4.61
IPI00162735	Isoform 2 of Attractin precursor	R.SCALDQNCQWEPR.N	2	3.47	0.28	
IPI00162735	Isoform 2 of Attractin precursor	R.SEAACLAAGPGIR.C	2	3.70	0.16	-3.73
IPI00162735	Isoform 2 of Attractin precursor	R.SVNNVVVR.Y	1	1.64	0.20	-2.12
IPI00162735	Isoform 2 of Attractin precursor	R.SVNNVVVR.Y	2	2.61	0.08	-1.63
IPI00162735	Isoform 2 of Attractin precursor	R.YGHSLALYKDK.I	2	2.82	0.06	-3.70
IPI00162735	Isoform 2 of Attractin precursor	R.YGHSLALYKDK.I	3	2.72	0.09	-4.07
IPI00162735	Isoform 2 of Attractin precursor	R.YQGNPLR.G	2	1.58	0.12	-3.11
IPI00163187	Fascin	K.LINRPIIVFR.G	2	2.34	0.26	-3.61
IPI00163187	Fascin	K.LINRPIIVFR.G	3	2.20	0.29	-4.19
IPI00163187	Fascin	K.VNASASSLKK.K	2	2.56	0.18	-2.16
IPI00163187	Fascin	K.YLTAEAFGFK.V	2	2.96	0.21	-2.88
IPI00163187	Fascin	R.KVTGTLDANR.S	2	2.45	0.30	-0.35
IPI00163187	Fascin	R.LVARPEPATGYTLEFR.S	2	3.01	0.31	-3.34
IPI00163187	Fascin	R.LVARPEPATGYTLEFR.S	3	5.26	0.48	-2.62
IPI00163187	Fascin	R.PADEIAVDRDVPWGVDSLITLAFQDQR.Y	3	4.63	0.42	-6.20
IPI00163187	Fascin	R.YFGGTEDR.L	2	1.72	0.20	-0.77
IPI00163187	Fascin	R.YLAPSGPSGTLK.A	2	2.45	0.12	-0.75
IPI00163187	Fascin	R.YLKGDHAGVLK.A	2	2.29	0.25	-3.44
IPI00163187	Fascin	R.YSVQTADHR.F	2	2.40	0.29	-3.56
IPI00163207	Isoform 1 of N-acetylmuramoyl-L-alanine amidase precursor	A.SLPLLM*DSVIQALAELEQK.V	2	6.11	0.46	-4.29
IPI00163207	Isoform 1 of N-acetylmuramoyl-L-alanine amidase precursor	A.SLPLLM*DSVIQALAELEQK.V	3	5.28	0.35	-3.10
IPI00163207	Isoform 1 of N-acetylmuramoyl-L-alanine amidase precursor	A.SLPLLM*DSVIQALAELEQKVPAAK.T	2	4.82	0.48	-4.58
IPI00163207	Isoform 1 of N-acetylmuramoyl-L-alanine amidase precursor	A.SLPLLM*DSVIQALAELEQKVPAAK.T	4	4.95	0.44	-2.28
IPI00163207	Isoform 1 of N-acetylmuramoyl-L-alanine amidase precursor	A.SLPLLMDSVIQALAELEQK.V	2	4.45	0.41	-2.33
IPI00163207	Isoform 1 of N-acetylmuramoyl-L-alanine amidase precursor	A.SLPLLMDSVIQALAELEQKVPAAK.T	2	5.34	0.38	-4.42
IPI00163207	Isoform 1 of N-acetylmuramoyl-L-alanine amidase precursor	D.GSPDVTTADIGANTPDATK.G	2	4.15	0.51	-2.32
IPI00163207	Isoform 1 of N-acetylmuramoyl-L-alanine amidase precursor	E.TGDTFPDVVAIAPDVR.A	2	2.90	0.16	-4.28
IPI00163207	Isoform 1 of N-acetylmuramoyl-L-alanine amidase precursor	K.ASLLTM*AFLNGALDGVILGDYLSR.T	2	5.56	0.51	-5.10
IPI00163207	Isoform 1 of N-acetylmuramoyl-L-alanine amidase precursor	K.EFTEAFLGCPAIHPR.C	3	2.72	0.38	-2.71

IPI00163207	Isoform 1 of N-acetylmuramoyl-L-alanine amidase precursor	K.EYGVVLAPDGSTVAVEPLLAGLEAGLQGR.R	2	5.16	0.59	-2.92
IPI00163207	Isoform 1 of N-acetylmuramoyl-L-alanine amidase precursor	K.EYGVVLAPDGSTVAVEPLLAGLEAGLQGR.R	3	6.62	0.54	-4.62
IPI00163207	Isoform 1 of N-acetylmuramoyl-L-alanine amidase precursor	K.GCPDVQASLPDAK.A	2	2.72	0.30	-2.48
IPI00163207	Isoform 1 of N-acetylmuramoyl-L-alanine amidase precursor	K.LLQLPLGFLYVHHTYVPAPPCTDFTR.C	3	3.87	0.33	-4.57
IPI00163207	Isoform 1 of N-acetylmuramoyl-L-alanine amidase precursor	K.LLQLPLGFLYVHHTYVPAPPCTDFTR.C	4	2.63	0.10	-4.36
IPI00163207	Isoform 1 of N-acetylmuramoyl-L-alanine amidase precursor	L.PLLM*DSVIQALAELEQKVPAAK.T	3	3.98	0.43	-1.03
IPI00163207	Isoform 1 of N-acetylmuramoyl-L-alanine amidase precursor	M.AFLNGALDGVILGDYLSR.T	2	5.52	0.53	-6.31
IPI00163207	Isoform 1 of N-acetylmuramoyl-L-alanine amidase precursor	M.AFLNGALDGVILGDYLSR.T	3	4.19	0.29	-3.82
IPI00163207	Isoform 1 of N-acetylmuramoyl-L-alanine amidase precursor	R.AGLLRPDYALLGHR.Q	2	3.86	0.46	-2.08
IPI00163207	Isoform 1 of N-acetylmuramoyl-L-alanine amidase precursor	R.AGLLRPDYALLGHR.Q	3	4.59	0.35	-2.91
IPI00163207	Isoform 1 of N-acetylmuramoyl-L-alanine amidase precursor	R.DGSPDVTTADIGANTPDATK.G	2	6.12	0.59	-2.87
IPI00163207	Isoform 1 of N-acetylmuramoyl-L-alanine amidase precursor	R.DGSPDVTTADIGANTPDATK.G	3	3.31	0.35	-1.24
IPI00163207	Isoform 1 of N-acetylmuramoyl-L-alanine amidase precursor	R.DTLPSCAVR.A	2	2.69	0.27	-3.42
IPI00163207	Isoform 1 of N-acetylmuramoyl-L-alanine amidase precursor	R.EGKEYGVVLAPDGSTVAVEPLLAGLEAGLQGR.R	2	3.34	0.52	-3.71
IPI00163207	Isoform 1 of N-acetylmuramoyl-L-alanine amidase precursor	R.EGKEYGVVLAPDGSTVAVEPLLAGLEAGLQGR.R	3	7.36	0.64	-4.83
IPI00163207	Isoform 1 of N-acetylmuramoyl-L-alanine amidase precursor	R.PSLSHLLSQYYGAGVAR.D	2	4.86	0.49	-5.19
IPI00163207	Isoform 1 of N-acetylmuramoyl-L-alanine amidase precursor	R.PSLSHLLSQYYGAGVAR.D	3	5.19	0.39	-4.26
IPI00163207	Isoform 1 of N-acetylmuramoyl-L-alanine amidase precursor	R.QNGAALTSASILAQQVWGTLVLLQR.L	3	4.32	0.19	-3.01
IPI00163207	Isoform 1 of N-acetylmuramoyl-L-alanine amidase precursor	R.QNGAALTSASILAQQVWGTLVLLQR.L	4	2.99	0.13	-0.95
IPI00163207	Isoform 1 of N-acetylmuramoyl-L-alanine amidase precursor	R.RVINLPLDSM*AAPWETGDTFPDVVAIAPDVR.A	3	5.87	0.53	-3.82
IPI00163207	Isoform 1 of N-acetylmuramoyl-L-alanine amidase precursor	R.TDCPGDALFDLLR.T	2	3.97	0.36	-3.05

IPI00163207	Isoform 1 of N-acetylmuramoyl-L-alanine amidase precursor	R.TFTLLDPK.A	2	2.53	0.25	-2.38
IPI00163207	Isoform 1 of N-acetylmuramoyl-L-alanine amidase precursor	R.TPEPRPSLSHLLSQYYGAGVAR.D	2	3.46	0.36	-4.66
IPI00163207	Isoform 1 of N-acetylmuramoyl-L-alanine amidase precursor	R.TPEPRPSLSHLLSQYYGAGVAR.D	3	5.70	0.49	-4.27
IPI00163207	Isoform 1 of N-acetylmuramoyl-L-alanine amidase precursor	R.TPEPRPSLSHLLSQYYGAGVAR.D	4	3.73	0.36	-3.26
IPI00163207	Isoform 1 of N-acetylmuramoyl-L-alanine amidase precursor	S.LPLLM*DSVIQALAELEQKVPAAK.T	3	5.73	0.49	-3.66
IPI00163207	Isoform 1 of N-acetylmuramoyl-L-alanine amidase precursor	W.ETGDTFPDVVAIAPDVR.A	2	3.69	0.41	-2.18
IPI00163207	Isoform 1 of N-acetylmuramoyl-L-alanine amidase precursor	W.ETGDTFPDVVAIAPDVR.A	3	3.98	0.20	-2.12
IPI00163391	Isoform 1 of Putative methyltransferase METT10D	R.RPPPSSVNTGGITEIMAEGGELEFVKR.I	3	3.08	0.13	
IPI00163446	IGHD protein	K.CVVQHTASK.S	2	2.61	0.10	
IPI00163446	IGHD protein	K.NSLYLQM*NSLR.A	2	3.76	0.14	
IPI00163446	IGHD protein	K.NSLYLQMNSLR.A	1	3.55	0.11	
IPI00163446	IGHD protein	K.NSLYLQMNSLR.A	2	4.01	0.17	
IPI00163446	IGHD protein	K.VPTGGVEEGLLER.H	2	4.05	0.41	
IPI00163446	IGHD protein	R.AEDTALYYCAK.H	2	3.60	0.41	
IPI00163446	IGHD protein	R.DNAKNSLYLQM*NSLR.A	2	4.82	0.44	
IPI00163446	IGHD protein	R.DNAKNSLYLQM*NSLR.A	3	4.39	0.37	
IPI00163446	IGHD protein	R.LSCAASGFTFDDYAMHWVR.Q	2	2.32	0.18	
IPI00163446	IGHD protein	R.LSCAASGFTFDDYAMHWVR.Q	3	3.33	0.17	
IPI00163446	IGHD protein	R.SLWNAGTSVTCTLNHPSLPPQR.L	2	3.03	0.14	
IPI00163446	IGHD protein	R.VPAPPSPQPATYTCVVSHEDSR.T	3	4.12	0.39	
IPI00163563	PEBP family protein precursor	K.FPGAVDGATYILVM*VDPDAPSR.A	2	5.45	0.57	-2.76
IPI00163563	PEBP family protein precursor	K.FPGAVDGATYILVM*VDPDAPSR.A	3	5.10	0.41	-4.98
IPI00163563	PEBP family protein precursor	K.FPGAVDGATYILVM*VDPDAPSRAEPR.Q	3	4.10	0.44	-4.59
IPI00163563	PEBP family protein precursor	K.FPGAVDGATYILVM*VDPDAPSRAEPR.Q	4	3.53	0.27	-4.14
IPI00163563	PEBP family protein precursor	K.GKIQGQELSAYQAPSPPAHSGFHR.Y	4	3.25	0.24	-3.02
IPI00163563	PEBP family protein precursor	K.IQGQELSAYQAPSPPAHSGFHR.Y	2	4.90	0.56	-3.16
IPI00163563	PEBP family protein precursor	K.IQGQELSAYQAPSPPAHSGFHR.Y	3	4.32	0.53	-2.69
IPI00163563	PEBP family protein precursor	K.IQGQELSAYQAPSPPAHSGFHR.Y	4	3.47	0.30	-1.77
IPI00163563	PEBP family protein precursor	K.ITSWM*EPIVK.F	2	2.56	0.33	-2.90
IPI00163563	PEBP family protein precursor	K.VISLLPKENK.T	2	2.79	0.19	-2.79
IPI00163563	PEBP family protein precursor	Q.NYQDSPTLQAPR.E	2	4.18	0.49	1.10
IPI00163563	PEBP family protein precursor	R.FHLGEPEASTQFM*TQ.N	2	4.05	0.51	-2.41
IPI00163563	PEBP family protein precursor	R.FHLGEPEASTQFM*TQN.Y	2	3.66	0.46	-2.83
IPI00163563	PEBP family protein precursor	R.FHLGEPEASTQFM*TQNY.Q	2	4.43	0.59	-1.65
IPI00163563	PEBP family protein precursor	R.FHLGEPEASTQFM*TQNYQDSPTLQAPR.E	2	4.39	0.59	-0.29

IPI00163563	PEBP family protein precursor	R.FHLGEPEASTQFM*TQNYQDSPTLQAPR.E	3	6.95	0.60	-6.37
IPI00163563	PEBP family protein precursor	R.FHLGEPEASTQFM*TQNYQDSPTLQAPR.E	4	4.13	0.37	-1.34
IPI00163563	PEBP family protein precursor	R.HWLVTDIK.G	2	1.97	0.15	1.10
IPI00163563	PEBP family protein precursor	R.YQFFVYLQEGK.V	1	3.15	0.29	-2.29
IPI00163563	PEBP family protein precursor	R.YQFFVYLQEGK.V	2	4.55	0.44	-5.10
IPI00163601	Putative uncharacterized protein FLJ10213	-.M*GYFLKLYAYVNSHSLFVWVCDRSYKR.S	3	3.46	0.12	
IPI00163724	Potassium/sodium hyperpolarization-activated cyclic nucleotide-gated channel 3	R.EEIINFTCR.G	2	2.78	0.06	
IPI00163851	Isoform 1 of Eukaryotic translation initiation factor 2-alpha kinase 4	-.M*AGGRGAPGR.G	2	1.90	0.16	
IPI00164066	Isoform 4 of Coiled-coil domain-containing protein 136	K.EQCGDELVAEPADPEEAK.S	2	1.69	0.09	-1.24
IPI00164861	Isoform 3 of Kinesin-like protein KIF13A	R.KRGAIVSEPAIQVRRK.G	2	2.16	0.17	
IPI00164949	Isoform NELF-C of Negative elongation factor C/D	K.AVETVHNLCCNENKGASELVAELSTLYQCIRFPVVAM*GVLK.W	5	3.37	0.13	-7.81
IPI00165009	Isoform 3 of MBT domain-containing protein 1	K.WFDYLR.E	1	1.83	0.08	-2.52
IPI00165044	Isoform 2 of Uncharacterized protein C4orf18	R.AGGPDFLQPSSR.E	2	2.81	0.10	-2.49
IPI00165044	Isoform 2 of Uncharacterized protein C4orf18	R.ASLQHGQAAEKGPHR.S	2	3.66	0.29	-4.38
IPI00165044	Isoform 2 of Uncharacterized protein C4orf18	R.ASLQHGQAAEKGPHR.S	3	3.50	0.24	-2.81
IPI00165044	Isoform 2 of Uncharacterized protein C4orf18	R.EADAVAPGYAQGANLVK.I	2	4.43	0.44	-3.25
IPI00165044	Isoform 2 of Uncharacterized protein C4orf18	R.LLVLEGGAPGAVLR.C	2	3.83	0.43	-2.94
IPI00165044	Isoform 2 of Uncharacterized protein C4orf18	R.LTWGTYQQLLK.Q	2	3.34	0.25	-2.30
IPI00165125	Isoform 1 of Uncharacterized protein C14orf37 precursor	K.EM*LTTNPK.T	2	1.66	0.13	-2.06
IPI00165125	Isoform 1 of Uncharacterized protein C14orf37 precursor	K.LGDNEETQVR.T	2	3.87	0.37	-0.64
IPI00165125	Isoform 1 of Uncharacterized protein C14orf37 precursor	K.TEKFEADTDHR.T	2	3.10	0.39	-3.52
IPI00165229	proprotein convertase subtilisin/kexin type 5 preproprotein	T.ILDDGIER.N	2	3.02	0.19	-3.22
IPI00165438	Muscle type neuropilin 1	K.EGNKPVLFQGNTNPTDVVVAVFPKPLITR.F	3	3.04	0.35	-4.25
IPI00165438	Muscle type neuropilin 1	K.EGNKPVLFQGNTNPTDVVVAVFPKPLITR.F	4	3.51	0.30	-2.50
IPI00165438	Muscle type neuropilin 1	K.FVSDYETHGAGFSIR.Y	2	4.43	0.47	-4.03
IPI00165438	Muscle type neuropilin 1	K.IAPPPVVSSGPFLFIK.F	2	1.36	0.49	-3.69
IPI00165438	Muscle type neuropilin 1	K.IAPPPVVSSGPFLFIK.F	3	4.77	0.46	-1.49
IPI00165438	Muscle type neuropilin 1	K.SFEGNNNYDTPELR.T	2	3.75	0.46	-4.94
IPI00165438	Muscle type neuropilin 1	K.SPGFPEKYPNSLECTYIVFAPK.M	2	2.39	0.31	-1.23
IPI00165438	Muscle type neuropilin 1	K.SPGFPEKYPNSLECTYIVFAPK.M	3	5.47	0.49	-1.99
IPI00165438	Muscle type neuropilin 1	R.EWIQVDLGLLR.F	2	3.02	0.36	-2.23
IPI00165438	Muscle type neuropilin 1	R.FVTAVGTQGAISK.E	2	4.71	0.37	-3.52
IPI00165438	Muscle type neuropilin 1	R.IM*INFNPHFDLEDR.D	3	3.14	0.19	-3.83
IPI00165936	Isoform A of Chloride intracellular channel 6	K.LGTQHPESNSAGNDVFAK.F	3	2.46	0.21	-2.03
IPI00165936	Isoform A of Chloride intracellular channel 6	R.VGDSVDAEGPAGDSVDAEGR.V	2	5.08	0.59	-2.52

IPI00165949	Isoform 2 of Endoplasmic reticulum aminopeptidase 1	K.FELGSSSIAHM*VM*GTTNQFSTR.T	3	3.40	0.34	-1.64
IPI00165949	Isoform 2 of Endoplasmic reticulum aminopeptidase 1	K.GFFSSLKENGSQLR.C	2	3.77	0.44	-2.32
IPI00165949	Isoform 2 of Endoplasmic reticulum aminopeptidase 1	K.GFFSSLKENGSQLR.C	3	2.20	0.23	-1.65
IPI00165949	Isoform 2 of Endoplasmic reticulum aminopeptidase 1	K.IKTQEFPQILTLIGR.N	3	3.45	0.35	-3.39
IPI00165949	Isoform 2 of Endoplasmic reticulum aminopeptidase 1	K.SGIVQYLQK.H	2	3.07	0.11	-0.78
IPI00165949	Isoform 2 of Endoplasmic reticulum aminopeptidase 1	K.SQIEFALCR.T	2	2.93	0.33	-0.89
IPI00165949	Isoform 2 of Endoplasmic reticulum aminopeptidase 1	K.TQEFPQILTLIGR.N	2	4.22	0.46	-3.34
IPI00165949	Isoform 2 of Endoplasmic reticulum aminopeptidase 1	K.VGDYFFGK.C	2	2.98	0.25	-1.91
IPI00165949	Isoform 2 of Endoplasmic reticulum aminopeptidase 1	K.YQFSLSSTEK.S	2	2.62	0.30	-1.75
IPI00165949	Isoform 2 of Endoplasmic reticulum aminopeptidase 1	R.ASLINNAFQLVSIGK.L	2	4.93	0.48	-2.73
IPI00165949	Isoform 2 of Endoplasmic reticulum aminopeptidase 1	R.DM*NEVETQFK.A	2	3.82	0.31	-1.45
IPI00165949	Isoform 2 of Endoplasmic reticulum aminopeptidase 1	R.EM*FDDVSYDK.G	2	2.16	0.17	-3.51
IPI00165949	Isoform 2 of Endoplasmic reticulum aminopeptidase 1	R.ESALLFDAEK.S	2	2.00	0.12	-1.84
IPI00165949	Isoform 2 of Endoplasmic reticulum aminopeptidase 1	R.ILASTQFEPTAAR.M	2	4.89	0.48	-4.07
IPI00165949	Isoform 2 of Endoplasmic reticulum aminopeptidase 1	R.LSEEPLQVLEHPR.Q	3	3.30	0.36	-1.55
IPI00165949	Isoform 2 of Endoplasmic reticulum aminopeptidase 1	R.NPVGYPLAWQFLR.K	2	3.60	0.41	-4.46
IPI00165972	Complement factor D preproprotein	K.VQVLLGAHSLSQPEPSK.R	3	3.12	0.34	-0.80
IPI00165972	Complement factor D preproprotein	K.VQVLLGAHSLSQPEPSKR.L	2	5.48	0.57	-3.27
IPI00165972	Complement factor D preproprotein	K.VQVLLGAHSLSQPEPSKR.L	3	4.70	0.49	-2.78
IPI00165972	Complement factor D preproprotein	R.AVPHPDSQPDTIDHDLLLLQLSEK.A	2	4.89	0.49	-2.70
IPI00165972	Complement factor D preproprotein	R.AVPHPDSQPDTIDHDLLLLQLSEK.A	3	4.16	0.41	-4.24
IPI00165972	Complement factor D preproprotein	R.AVPHPDSQPDTIDHDLLLLQLSEK.A	4	3.36	0.20	-2.42
IPI00165972	Complement factor D preproprotein	R.DSCKGDSGGPLVCGGVLEGVVTSGSR.V	2	5.30	0.58	-3.24
IPI00165972	Complement factor D preproprotein	R.DSCKGDSGGPLVCGGVLEGVVTSGSR.V	3	5.11	0.49	-2.05
IPI00165972	Complement factor D preproprotein	R.KKPGIYTR.V	2	1.96	0.12	-2.36
IPI00165972	Complement factor D preproprotein	R.RPDSLQHVLLPVLDR.A	2	3.65	0.41	-2.29

IPI00165972	Complement factor D preproprotein	R.RPDSLQHVLLPVLDR.A	3	4.56	0.41	-2.58
IPI00165972	Complement factor D preproprotein	R.RPDSLQHVLLPVLDR.A	4	2.96	0.28	-1.50
IPI00165972	Complement factor D preproprotein	R.VDRDVAPGTLCDVAGWGIVNHAGR.R	3	6.32	0.53	-3.12
IPI00165972	Complement factor D preproprotein	R.VDRDVAPGTLCDVAGWGIVNHAGR.R	4	4.32	0.35	-2.26
IPI00165975	Cysteine-rich flanking region, C-terminal domain containing protein	K.HAPGAGGEPDGQAPTSER.K	3	2.86	0.11	-0.73
IPI00165975	Cysteine-rich flanking region, C-terminal domain containing protein	K.HAPGAGGEPDGQAPTSERK.S	3	1.71	0.12	-3.07
IPI00165975	Cysteine-rich flanking region, C-terminal domain containing protein	R.AGLAFVLHCIADGHPTPR.L	3	3.67	0.46	-2.20
IPI00165975	Cysteine-rich flanking region, C-terminal domain containing protein	R.LPALPCAPPSVHLSAEPPLEAPGTPLR.A	3	4.87	0.52	-3.19
IPI00165975	Cysteine-rich flanking region, C-terminal domain containing protein	R.TVEPGALAVLSQLK.N	2	4.54	0.35	-3.69
IPI00165975	Cysteine-rich flanking region, C-terminal domain containing protein	R.TVEPGALAVLSQLK.N	3	4.08	0.26	-2.21
IPI00165975	Cysteine-rich flanking region, C-terminal domain containing protein	R.VAVAATGPPK.H	1	1.92	0.29	-2.65
IPI00166010	Isoform 1 of CCR4-NOT transcription complex subunit 1	R.EVFNCMLRNLFEEYRFFPQYPDK.E	3	3.45	0.06	
IPI00166039	Isoform 1 of Scotin precursor	R.GLSLFPESCPDFCCGTCDDQYCCSDVLKK.F	3	2.54	0.10	-1.89
IPI00166048	Isoform 1 of Cell adhesion molecule 3 precursor	K.AYYTLNVNDPSPVP.S	2	3.17	0.24	-2.71
IPI00166048	Isoform 1 of Cell adhesion molecule 3 precursor	K.CQVKDHEDSSLQWSNPAQQTLYFGEK.R	4	2.67	0.10	-3.09
IPI00166048	Isoform 1 of Cell adhesion molecule 3 precursor	K.DTATLNCQSSGSKPAAR.L	2	3.82	0.32	-2.03
IPI00166048	Isoform 1 of Cell adhesion molecule 3 precursor	K.EGSVPPLK.M	1	1.99	0.17	-3.50
IPI00166048	Isoform 1 of Cell adhesion molecule 3 precursor	K.EGSVPPLK.M	2	1.92	0.08	-3.48
IPI00166048	Isoform 1 of Cell adhesion molecule 3 precursor	K.LLLHCEGR.G	2	1.98	0.05	-2.68
IPI00166048	Isoform 1 of Cell adhesion molecule 3 precursor	K.SDSGTYGCTATSNM*GSYK.A	2	5.02	0.64	-4.11
IPI00166048	Isoform 1 of Cell adhesion molecule 3 precursor	K.SDSGTYGCTATSNM*GSYKA.Y	2	4.90	0.62	-3.61
IPI00166048	Isoform 1 of Cell adhesion molecule 3 precursor	K.SLVTVLGIPQKPIITG.Y	2	3.37	0.34	-4.54
IPI00166048	Isoform 1 of Cell adhesion molecule 3 precursor	K.SLVTVLGIPQKPIITGY.K	2	4.27	0.52	-4.60
IPI00166048	Isoform 1 of Cell adhesion molecule 3 precursor	K.SLVTVLGIPQKPIITGYK.S	2	4.34	0.51	-2.97
IPI00166048	Isoform 1 of Cell adhesion molecule 3 precursor	K.SLVTVLGIPQKPIITGYK.S	3	3.82	0.46	-1.83
IPI00166048	Isoform 1 of Cell adhesion molecule 3 precursor	K.TFTVSSSVTFQVTR.E	2	4.10	0.49	-6.57
IPI00166048	Isoform 1 of Cell adhesion molecule 3 precursor	L.VTVLGIPQKPIITGYK.S	2	3.36	0.48	-2.53
IPI00166048	Isoform 1 of Cell adhesion molecule 3 precursor	R.EDDGASIVCSVNHESLK.G	2	4.36	0.45	-3.36
IPI00166048	Isoform 1 of Cell adhesion molecule 3 precursor	R.EKDTATLNCQSSGSKPAAR.L	3	3.30	0.41	-2.49
IPI00166048	Isoform 1 of Cell adhesion molecule 3 precursor	R.GNPVPQQYLWEK.E	2	3.17	0.47	-3.57
IPI00166048	Isoform 1 of Cell adhesion molecule 3 precursor	R.IEVLYTPTAM*IRPDPPHPR.E	3	3.75	0.45	-5.47
IPI00166048	Isoform 1 of Cell adhesion molecule 3 precursor	R.IEVLYTPTAM*IRPDPPHPR.E	4	3.39	0.41	-3.52
IPI00166048	Isoform 1 of Cell adhesion molecule 3 precursor	R.IQLVTSTPHELSISISNVALADEGEYTCSIFTM*PVR.T	3	4.31	0.30	-1.00
IPI00166048	Isoform 1 of Cell adhesion molecule 3 precursor	R.KGDQELHGEPTR.I	2	3.96	0.41	-3.47

IPI00166048	Isoform 1 of Cell adhesion molecule 3 precursor	R.KGDQELHGEPTR.I	3	3.04	0.25	-3.14
IPI00166048	Isoform 1 of Cell adhesion molecule 3 precursor	R.STSQRIEVLYTPTAM*.I	2	3.17	0.35	-3.24
IPI00166048	Isoform 1 of Cell adhesion molecule 3 precursor	R.STSQRIEVLYTPTAM*IRPDPPHPR.E	4	3.61	0.15	-4.58
IPI00166048	Isoform 1 of Cell adhesion molecule 3 precursor	W.SNPAQQTLYFGEK.R	2	3.38	0.45	-7.03
IPI00166048	Isoform 1 of Cell adhesion molecule 3 precursor	W.SNPAQQTLYFGEKR.A	2	3.74	0.40	-3.39
IPI00166071	B-cell CLL/lymphoma 6 member B protein	R.IHTGEKPYHCDPCGLHFR.H	3	3.22	0.16	
IPI00166075	Leucine-rich repeat LGI family member 3 precursor	R.DTAFCVDSK.A	2	2.91	0.27	-2.44
IPI00166161	Protein SIX6OS1	K.FMQVRLLTPQKQSNSNQWSEKGDK.D	3	1.77	0.18	0.01
IPI00166257	CDNA FLJ37614 fis, clone BRCOC2011769	R.SWQPGPGSLAFTPR.Q	2	2.04	0.17	
IPI00166339	Isoform 1 of Ephrin type-A receptor 10 precursor	K.IDTIAADESFTQGDLGER.K	2	5.91	0.62	-4.77
IPI00166339	Isoform 1 of Ephrin type-A receptor 10 precursor	K.IDTIAADESFTQGDLGER.K	3	4.34	0.46	-4.05
IPI00166339	Isoform 1 of Ephrin type-A receptor 10 precursor	R.DLQYSLSR.S	2	2.50	0.06	0.17
IPI00166339	Isoform 1 of Ephrin type-A receptor 10 precursor	R.GLATFPATAAESAFSTLVEVAGTCVAHSEGEPGSPPR.M	3	2.41	0.15	-3.63
IPI00166339	Isoform 1 of Ephrin type-A receptor 10 precursor	R.GLATFPATAAESAFSTLVEVAGTCVAHSEGEPGSPPR.M	4	3.33	0.29	-4.33
IPI00166339	Isoform 1 of Ephrin type-A receptor 10 precursor	V.KIDTIAADESFTQGDLGER.K	2	6.39	0.61	-1.98
IPI00166339	Isoform 1 of Ephrin type-A receptor 10 precursor	V.KIDTIAADESFTQGDLGER.K	3	4.38	0.36	-1.48
IPI00166392	Immunoglobulin superfamily member 4	E.LDSEDLSDSR.A	2	3.40	0.41	-1.92
IPI00166392	Immunoglobulin superfamily member 4	K.AHSDYM*L.Y	1	1.98	0.22	-3.59
IPI00166392	Immunoglobulin superfamily member 4	K.SDDSVIQLLNPNR.Q	2	3.70	0.29	-3.69
IPI00166392	Immunoglobulin superfamily member 4	N.SAEELDSEDLSDSR.A	2	4.90	0.54	-4.71
IPI00166392	Immunoglobulin superfamily member 4	R.AVDHAVIGGVVA.V	1	2.31	0.40	-0.91
IPI00166392	Immunoglobulin superfamily member 4	R.CEASNIVGK.A	1	2.64	0.28	-2.53
IPI00166392	Immunoglobulin superfamily member 4	R.CEASNIVGK.A	2	2.77	0.28	-1.47
IPI00166392	Immunoglobulin superfamily member 4	R.DFRPLKDSR.F	2	2.10	0.13	-2.82
IPI00166392	Immunoglobulin superfamily member 4	R.EGDALELTCEAIGKPQPVM*VTWVR.V	2	4.29	0.44	-3.67
IPI00166392	Immunoglobulin superfamily member 4	R.EGDALELTCEAIGKPQPVM*VTWVR.V	3	5.86	0.48	-3.55
IPI00166392	Immunoglobulin superfamily member 4	R.NLM*IDIQK.D	1	2.81	0.11	-3.72
IPI00166392	Immunoglobulin superfamily member 4	R.NLM*IDIQK.D	2	2.82	0.05	-1.88
IPI00166392	Immunoglobulin superfamily member 4	R.QTIYFR.D	2	1.24	0.06	-2.35
IPI00166392	Immunoglobulin superfamily member 4	R.VDDEM*PQHAVLSGPNLFINNLNK.T	3	6.32	0.43	-3.17
IPI00166392	Immunoglobulin superfamily member 4	R.YFCQLYTDPPQESYTTITVLVPPR.N	2	4.32	0.57	-3.87
IPI00166392	Immunoglobulin superfamily member 4	R.YFCQLYTDPPQESYTTITVLVPPR.N	3	3.15	0.32	-3.46
IPI00166392	Immunoglobulin superfamily member 4	R.YFCQLYTDPPQESYTTITVLVPPR.N	4	4.48	0.38	-4.83
IPI00166392	Immunoglobulin superfamily member 4	R.YLEVQYKPQVHIQM*TYPLQGLTR.E	2	4.03	0.59	-3.95
IPI00166392	Immunoglobulin superfamily member 4	R.YLEVQYKPQVHIQM*TYPLQGLTR.E	3	5.85	0.50	-6.84
IPI00166392	Immunoglobulin superfamily member 4	R.YLEVQYKPQVHIQM*TYPLQGLTR.E	4	3.41	0.34	-5.00
IPI00166553	Isoform 1 of Protein FAM19A2 precursor	R.AAPSCVDASIVEQK.W	2	3.77	0.41	-3.05
IPI00166613	Isoform 1 of Putative polypeptide N-acetylgalactosaminyltransferase-like protein 1	K.AGEDPYRQHAFNQLESDKLSPDRPI.R	3	5.49	0.34	-3.74
IPI00166613	Isoform 1 of Putative polypeptide N-acetylgalactosaminyltransferase-like protein 1	K.AGEDPYRQHAFNQLESDKLSPDRPI.R	4	5.85	0.43	-2.54

IPI00166613	Isoform 1 of Putative polypeptide N-acetylgalactosaminyltransferase-like protein 1	R.QHAFNQLESDKLSPDRPI.R	2	3.43	0.35	-2.30
IPI00166619	Isoform 2 of Putative transporter SVOPL	K.YRGYMLPLSQVFWLAGSLLIIGLASVIIPTIGWR.W	3	2.43	0.27	-0.25
IPI00166622	similar to CG14446-PA	G.HGVTDNIQR.F	2	2.90	0.39	-1.25
IPI00166622	similar to CG14446-PA	K.DFSLAATSQDEAVVSVPQPR.S	2	2.62	0.26	-0.78
IPI00166622	similar to CG14446-PA	K.KGVNILSAQTR.E	2	3.14	0.30	-3.42
IPI00166622	similar to CG14446-PA	K.LEEPHVATLQDSR.V	2	3.80	0.44	-2.72
IPI00166622	similar to CG14446-PA	K.LEEPHVATLQDSR.V	3	4.12	0.34	-0.53
IPI00166622	similar to CG14446-PA	K.SILAVGVGNVR.V	1	1.78	0.11	-2.93
IPI00166622	similar to CG14446-PA	K.SILAVGVGNVR.V	2	2.97	0.29	-2.41
IPI00166622	similar to CG14446-PA	K.SM*DQPEGTPVELYYTVHPGNER.G	3	2.72	0.14	-2.08
IPI00166622	similar to CG14446-PA	K.STDEDVIKVSER.C	2	3.16	0.29	-1.99
IPI00166622	similar to CG14446-PA	K.TITVLDDK.V	2	1.88	0.10	-2.35
IPI00166622	similar to CG14446-PA	K.VVPLDLM*LTSNFLGPTNK.F	2	3.58	0.25	-3.95
IPI00166622	similar to CG14446-PA	R.AETSFFLK.E	1	1.75	0.11	-2.26
IPI00166622	similar to CG14446-PA	R.AQDSAQLSELR.L	2	4.03	0.28	-2.37
IPI00166622	similar to CG14446-PA	R.EVGM*TTIQVLSPLSDSILAEK.T	2	5.49	0.41	-4.37
IPI00166622	similar to CG14446-PA	R.EVGM*TTIQVLSPLSDSILAEK.T	3	5.46	0.38	-3.85
IPI00166622	similar to CG14446-PA	R.FSSLPPYLPVSYHILR.A	3	2.02	0.13	-2.96
IPI00166622	similar to CG14446-PA	R.KSILAVGVGNVR.V	2	2.90	0.22	-2.84
IPI00166622	similar to CG14446-PA	R.KSILAVGVGNVR.V	3	3.56	0.11	-3.94
IPI00166622	similar to CG14446-PA	R.LAGEGQLQNIPIDFTNFPAHVDLPK.A	3	5.69	0.43	-4.05
IPI00166622	similar to CG14446-PA	R.LPLQIEVSDTELSQIK.G	2	5.30	0.42	-3.76
IPI00166622	similar to CG14446-PA	R.TGQDGHLYGSSPVER.E	3	3.09	0.34	-1.08
IPI00166622	similar to CG14446-PA	R.VDM*TIAEACQK.S	2	3.70	0.35	-1.76
IPI00166622	similar to CG14446-PA	R.VESFFTYK.T	1	1.98	0.25	-3.32
IPI00166622	similar to CG14446-PA	R.VESFFTYK.T	2	2.76	0.28	-1.49
IPI00166622	similar to CG14446-PA	R.WPVVVAEGEGQGPLIR.V	2	4.80	0.38	-4.07
IPI00166622	similar to CG14446-PA	W.GVKQEVGSGGK.H	2	3.17	0.23	-1.05
IPI00166729	alpha-2-glycoprotein 1, zinc	K.AREDIFM*ETLK.D	2	2.98	0.18	
IPI00166729	alpha-2-glycoprotein 1, zinc	K.AYLEEECPATLR.K	2	4.41	0.38	
IPI00166729	alpha-2-glycoprotein 1, zinc	K.CLAYDFYPGK.I	1	2.87	0.26	
IPI00166729	alpha-2-glycoprotein 1, zinc	K.CLAYDFYPGK.I	2	3.57	0.36	
IPI00166729	alpha-2-glycoprotein 1, zinc	K.EIPAWVPFDPAAQITK.Q	2	3.04	0.31	
IPI00166729	alpha-2-glycoprotein 1, zinc	K.HVEDVPAFQALGSLNDLQFFR.Y	2	5.77	0.42	
IPI00166729	alpha-2-glycoprotein 1, zinc	K.HVEDVPAFQALGSLNDLQFFR.Y	3	2.30	0.13	-5.40
IPI00166729	alpha-2-glycoprotein 1, zinc	K.NILDRQDPPSVVVTSHQAPGEK.K	2	4.63	0.39	
IPI00166729	alpha-2-glycoprotein 1, zinc	K.NILDRQDPPSVVVTSHQAPGEK.K	3	6.55	0.43	
IPI00166729	alpha-2-glycoprotein 1, zinc	K.NILDRQDPPSVVVTSHQAPGEKK.K	3	5.42	0.42	
IPI00166729	alpha-2-glycoprotein 1, zinc	K.QKWEAEPVYVQR.A	2	2.35	0.21	
IPI00166729	alpha-2-glycoprotein 1, zinc	K.YYYDGKDYIEFNK.E	1	3.50	0.33	
IPI00166729	alpha-2-glycoprotein 1, zinc	K.YYYDGKDYIEFNK.E	2	4.53	0.32	

IPI00166729	alpha-2-glycoprotein 1, zinc	K.YYYDGKDYIEFNK.E	3	2.68	0.23	
IPI00166729	alpha-2-glycoprotein 1, zinc	K.YYYDGKDYIEFNKEIPAWVPFDPAAQITK.Q	3	5.32	0.31	
IPI00166729	alpha-2-glycoprotein 1, zinc	R.AGEVQEPELR.G	2	3.54	0.09	
IPI00166729	alpha-2-glycoprotein 1, zinc	R.AKAYLEEECPATLR.K	2	4.88	0.41	
IPI00166729	alpha-2-glycoprotein 1, zinc	R.AKAYLEEECPATLRK.Y	2	4.80	0.30	
IPI00166729	alpha-2-glycoprotein 1, zinc	R.AKAYLEEECPATLRK.Y	3	3.76	0.33	
IPI00166729	alpha-2-glycoprotein 1, zinc	R.QDPPSVVVTSHQAPGEK.K	2	1.75	0.18	
IPI00166729	alpha-2-glycoprotein 1, zinc	R.QVEGM*EDWKQDSQLQK.A	2	3.40	0.24	
IPI00166729	alpha-2-glycoprotein 1, zinc	R.QVEGMEDWKQDSQLQK.A	2	3.02	0.31	
IPI00166729	alpha-2-glycoprotein 1, zinc	R.YSLTYIYTGLSK.H	1	3.00	0.40	
IPI00166729	alpha-2-glycoprotein 1, zinc	R.YSLTYIYTGLSK.H	2	4.82	0.43	
IPI00166766	hypothetical protein LOC146556 isoform 2	A.TIADLILSALER.A	2	3.82	0.33	-3.11
IPI00166766	hypothetical protein LOC146556 isoform 2	K.AIQYQQHFSR.R	1	2.66	0.22	-3.36
IPI00166766	hypothetical protein LOC146556 isoform 2	K.AIQYQQHFSR.R	2	3.76	0.41	-2.81
IPI00166766	hypothetical protein LOC146556 isoform 2	K.ATIADLILSALER.A	1	2.12	0.31	-2.10
IPI00166766	hypothetical protein LOC146556 isoform 2	K.ATIADLILSALER.A	2	4.11	0.38	-5.84
IPI00166766	hypothetical protein LOC146556 isoform 2	K.ATIADLILSALER.A	3	5.66	0.25	-4.53
IPI00166766	hypothetical protein LOC146556 isoform 2	K.WAQEPLLQPLSLR.V	2	3.92	0.37	-5.08
IPI00166766	hypothetical protein LOC146556 isoform 2	R.AEAIGYAYPTR.D	1	2.57	0.21	-3.68
IPI00166766	hypothetical protein LOC146556 isoform 2	R.AEAIGYAYPTR.D	2	3.21	0.30	-2.78
IPI00166766	hypothetical protein LOC146556 isoform 2	R.ATVFLEQR.L	1	2.10	0.06	-2.47
IPI00166766	hypothetical protein LOC146556 isoform 2	R.ATVFLEQR.L	2	2.85	0.22	-2.21
IPI00166766	hypothetical protein LOC146556 isoform 2	R.EFQLTLQPGFWK.L	2	3.75	0.30	-4.85
IPI00166766	hypothetical protein LOC146556 isoform 2	R.GCTQGPLQQSQDYINLFCANM*M*DLNR.R	3	5.65	0.48	-4.23
IPI00166766	hypothetical protein LOC146556 isoform 2	R.GCTQGPLQQSQDYINLFCANM*M*DLNRR.A	3	3.13	0.24	-3.67
IPI00166766	hypothetical protein LOC146556 isoform 2	R.LPEINLDGM*VGVR.V	2	4.71	0.41	-4.78
IPI00166766	hypothetical protein LOC146556 isoform 2	R.RAEAIGYAYPTR.D	2	4.43	0.45	-1.79
IPI00166766	hypothetical protein LOC146556 isoform 2	R.VGM*LGEKLEAAIQR.S	3	4.37	0.27	-2.14
IPI00166766	hypothetical protein LOC146556 isoform 2	S.SSLPGLDTAESK.A	1	2.46	0.30	-4.30
IPI00166766	hypothetical protein LOC146556 isoform 2	S.SSLPGLDTAESKATIADLILSALER.A	2	5.02	0.50	-3.96
IPI00166766	hypothetical protein LOC146556 isoform 2	S.SSLPGLDTAESKATIADLILSALER.A	3	3.92	0.27	-5.01
IPI00166766	hypothetical protein LOC146556 isoform 2	T.IADLILSALER.A	2	3.12	0.37	-4.70
IPI00166766	hypothetical protein LOC146556 isoform 2	W.AQEPLLQPLSLR.V	2	3.06	0.32	-2.50
IPI00166776	Protein CREG2 precursor	K.NIVDPEDPR.C	2	2.20	0.33	-2.56
IPI00166807	Isoform 3 of Oxidation resistance protein 1	R.IRDAGNDSASTAPR.S	3	2.22	0.11	-2.37
IPI00166817	Zinc finger protein 561	-.M*AAIYLSR.G	2	2.00	0.07	2.13
IPI00166865	CDGSH iron sulfur domain-containing protein 2	-.M*VLESVAR.I	2	1.78	0.05	3.10
IPI00166892	DPPY splice variant c	K.DVVYK.N	1	1.46	0.08	-3.57
IPI00166892	DPPY splice variant c	K.ILHIDDYELPLQLSLPK.D	3	2.94	0.16	-3.67
IPI00166892	DPPY splice variant c	K.LYASAFSER.Y	2	2.98	0.29	-1.06
IPI00166892	DPPY splice variant c	R.FTGALYPK.G	2	1.95	0.11	-0.09
IPI00166892	DPPY splice variant c	R.KDFVLHDPEAR.W	3	2.84	0.08	-0.63

IPI00166892	DPPY splice variant c	R.LSLEDLFR.K	2	2.19	0.17	-2.40
IPI00166892	DPPY splice variant c	R.QLYSASTEGLLNR.Q	2	2.45	0.18	-3.97
IPI00167006	Uncharacterized protein C13orf26	G.LVPSVLHSYLRNQEHTKK.Q	3	3.57	0.28	-4.36
IPI00167089	Isoform 2 of Activated CDC42 kinase 1	R.EACASDPRLHPVSSR.T	3	3.01	0.15	
IPI00167093	complement factor H-related 1	K.CGPPPPIDNGDITSFPLSVYAPASSVEYQCQNLYQLEGNKR.I	3	5.17	0.49	-3.33
IPI00167093	complement factor H-related 1	K.INHGILYDEEK.Y	2	3.57	0.37	-3.89
IPI00167093	complement factor H-related 1	K.YKPFSQVPTGEVFYYSCEYNFVSPSK.S	3	4.07	0.27	
IPI00167093	complement factor H-related 1	R.EIM*ENYNIALR.W	2	2.73	0.35	-4.22
IPI00167093	complement factor H-related 1	R.LCFFPFVENGHSESSGQTHLEGDTVQIICNTGYR.L	3	5.60	0.33	
IPI00167093	complement factor H-related 1	R.LQNNENNISCVER.G	2	3.88	0.44	-0.87
IPI00167093	complement factor H-related 1	R.NGQWSEPPKCLHPCVISR.E	3	3.60	0.35	
IPI00167093	complement factor H-related 1	R.QMSKYPSGER.V	2	2.87	0.22	
IPI00167093	complement factor H-related 1	R.STDTSCVNPPTVQNAHILSR.Q	2	4.34	0.38	
IPI00167093	complement factor H-related 1	R.STDTSCVNPPTVQNAHILSR.Q	3	4.07	0.31	
IPI00167093	complement factor H-related 1	R.TGESAEFVCK.R	2	2.87	0.29	
IPI00167093	complement factor H-related 1	R.TGESAEFVCKR.G	2	3.12	0.23	
IPI00167093	complement factor H-related 1	R.TGESAEFVCKR.G	3	2.46	0.18	
IPI00167093	complement factor H-related 1	R.TTCWDGKLEYPTCAK.R	2	4.43	0.42	
IPI00167137	Isoform 3 of SLAM family member 7 precursor	K.YGLLHCGNTEKDGKSPLTAHDAR.H	3	2.75	0.06	-1.61
IPI00167154	Uncharacterized protein MAPKBP1	R.AQESVGFLDPAPAANPGPR.R	3	2.43	0.10	-3.69
IPI00167215	Isoform 1 of Hepatocyte cell adhesion molecule precursor	K.DKDSPETEENPAPEPR.S	3	3.68	0.20	-3.16
IPI00167215	Isoform 1 of Hepatocyte cell adhesion molecule precursor	K.DSPETEENPAPEPR.S	2	2.86	0.33	-0.86
IPI00167215	Isoform 1 of Hepatocyte cell adhesion molecule precursor	R.DKPVTVVQSIGTEVIGTLRPDYR.D	3	2.66	0.10	-2.53
IPI00167215	Isoform 1 of Hepatocyte cell adhesion molecule precursor	R.DKPVTVVQSIGTEVIGTLRPDYRDR.I	4	2.49	0.12	-3.71
IPI00167215	Isoform 1 of Hepatocyte cell adhesion molecule precursor	R.LIHGTVGK.S	1	1.83	0.08	-3.97
IPI00167215	Isoform 1 of Hepatocyte cell adhesion molecule precursor	R.LIHGTVGK.S	2	2.18	0.10	-5.40
IPI00167215	Isoform 1 of Hepatocyte cell adhesion molecule precursor	R.SATEPGPPGYSVSPAVPGR.S	2	4.38	0.54	-2.20
IPI00167215	Isoform 1 of Hepatocyte cell adhesion molecule precursor	Y.ILKDKDSPETEENPAPEPR.S	3	4.79	0.44	-2.72
IPI00167254	Isoform 4 of Inactive phospholipase D5	F.SAVDIM*GEDEDGLSEK.N	2	3.98	0.49	-4.35
IPI00167560	PAP-associated domain-containing protein 4	R.GRKRLSDEKNLPLDGK.R	2	2.51	0.11	
IPI00167619	Leucine-rich repeat and transmembrane domain-containing protein 2 precursor	R.LDLSNNFLDR.L	2	3.46	0.33	-3.43
IPI00167638	Isoform 1 of GTP-binding protein 10	K.DRYPRKRFVAGVGANSK.I	3	2.47	0.06	-5.47
IPI00167710	Isoform 1 of Fibulin-7 precursor	K.TISFHYLSLPSNLK.T	2	3.75	0.43	-4.91

IPI00167710	Isoform 1 of Fibulin-7 precursor	K.TPITLFR.M	2	2.02	0.13	-2.42
IPI00167710	Isoform 1 of Fibulin-7 precursor	R.FGIVGGNSR.G	1	1.23	0.21	-0.94
IPI00167710	Isoform 1 of Fibulin-7 precursor	R.M*ATASAPGR.A	2	2.42	0.18	-4.54
IPI00167941	Midasin	R.LNAALATPAKEM*GM*GNVERCR.G	2	2.28	0.13	
IPI00168404	Zinc finger and BTB domain containing 34	R.METTPSKALRSRLQEEGHSDR.G	3	2.61	0.11	-0.10
IPI00168479	Isoform 1 of Apolipoprotein A-I-binding protein precursor	K.GDVREPFHSILSVLK.G	2	2.74	0.19	-5.26
IPI00168479	Isoform 1 of Apolipoprotein A-I-binding protein precursor	K.GDVREPFHSILSVLK.G	3	2.08	0.20	-3.05
IPI00168479	Isoform 1 of Apolipoprotein A-I-binding protein precursor	K.GDVREPFHSILSVLK.G	4	2.57	0.17	-2.92
IPI00168479	Isoform 1 of Apolipoprotein A-I-binding protein precursor	K.GLTVPIASIDIPSGWDVEK.G	2	4.02	0.35	-4.09
IPI00168479	Isoform 1 of Apolipoprotein A-I-binding protein precursor	K.GLTVPIASIDIPSGWDVEKGNAGGIQPDLLISLTAPK.K	3	4.54	0.46	-5.05
IPI00168479	Isoform 1 of Apolipoprotein A-I-binding protein precursor	K.GLTVPIASIDIPSGWDVEKGNAGGIQPDLLISLTAPK.K	4	3.25	0.16	-4.02
IPI00168479	Isoform 1 of Apolipoprotein A-I-binding protein precursor	K.GNAGGIQPDLLISLTAPK.K	2	3.76	0.42	-3.99
IPI00168479	Isoform 1 of Apolipoprotein A-I-binding protein precursor	K.LFGYEPTIYYPK.R	2	3.76	0.35	-3.93
IPI00168479	Isoform 1 of Apolipoprotein A-I-binding protein precursor	K.SATQFTGR.Y	2	2.02	0.39	-1.93
IPI00168479	Isoform 1 of Apolipoprotein A-I-binding protein precursor	R.SPPTVLVICGPGNNGGDGLVCAR.H	2	5.81	0.63	-2.98
IPI00168479	Isoform 1 of Apolipoprotein A-I-binding protein precursor	R.SPPTVLVICGPGNNGGDGLVCAR.H	3	2.84	0.19	-2.95
IPI00168520	Isoform 2 of Matrilin-2 precursor	R.GYTLDPNGK.T	2	1.63	0.27	-2.93
IPI00168520	Isoform 2 of Matrilin-2 precursor	R.M*EALENR.L	2	2.50	0.08	-3.21
IPI00168520	Isoform 2 of Matrilin-2 precursor	R.SVNTHDYAK.V	2	2.45	0.28	-1.49
IPI00168520	Isoform 2 of Matrilin-2 precursor	R.VIM*IVTDGRPQDSVAEVAAK.A	2	2.86	0.37	-2.23
IPI00168626	Isoform 1 of Putative polypeptide N-acetylgalactosaminyltransferase-like protein 4	K.IIERLDHLENVIK.Q	2	3.19	0.19	-3.62
IPI00168626	Isoform 1 of Putative polypeptide N-acetylgalactosaminyltransferase-like protein 4	K.IIERLDHLENVIK.Q	3	3.26	0.24	-2.44
IPI00168626	Isoform 1 of Putative polypeptide N-acetylgalactosaminyltransferase-like protein 4	K.LEEDKGDTLK.I	2	2.54	0.23	-1.12
IPI00168626	Isoform 1 of Putative polypeptide N-acetylgalactosaminyltransferase-like protein 4	K.LTEYVDKVNSQKPGFIK.V	2	4.34	0.44	-3.97
IPI00168626	Isoform 1 of Putative polypeptide N-acetylgalactosaminyltransferase-like protein 4	K.LTEYVDKVNSQKPGFIK.V	3	2.88	0.34	-3.85

IPI00168626	Isoform 1 of Putative polypeptide N-acetylgalactosaminyltransferase-like protein 4	R.LIECSYAK.A	1	1.35	0.09	-2.75
IPI00168626	Isoform 1 of Putative polypeptide N-acetylgalactosaminyltransferase-like protein 4	R.LIECSYAK.A	2	2.25	0.07	-1.57
IPI00168626	Isoform 1 of Putative polypeptide N-acetylgalactosaminyltransferase-like protein 4	R.M*YSDIIAYGVLQNSLK.T	2	6.05	0.51	-3.24
IPI00168626	Isoform 1 of Putative polypeptide N-acetylgalactosaminyltransferase-like protein 4	R.SPALIGCFIVDR.Q	2	3.40	0.37	-4.37
IPI00168626	Isoform 1 of Putative polypeptide N-acetylgalactosaminyltransferase-like protein 4	R.WYLVSVYPEM*R.M	2	3.00	0.23	-4.76
IPI00168806	Isoform 1 of Myeloid/lymphoid or mixed-lineage leukemia protein 3 homolog	R.CTNIYHFTCAIKAQCM*FFK.D	2	1.09	0.07	-5.82
IPI00168847	Isoform 2 of Hyaluronidase-1 precursor	R.ALVQAQHPDWPAPQVEAVAQDQFQGAAR.A	3	3.58	0.17	
IPI00168847	Isoform 2 of Hyaluronidase-1 precursor	R.ALYPSIYM*PAVLEGTGK.S	2	3.50	0.40	
IPI00168847	Isoform 2 of Hyaluronidase-1 precursor	R.AWM*AGTLQLGR.A	2	2.94	0.07	
IPI00168862	Conserved hypothetical protein	-.M*KKKHDGIVYETK.E	2	2.65	0.14	-1.70
IPI00168866	MAM domain containing glycosylphosphatidylinositol anchor 1	K.GQLLEYILTDLR.V	2	4.13	0.34	-3.68
IPI00168866	MAM domain containing glycosylphosphatidylinositol anchor 1	K.GQLLEYILTDLRVPHSYEVR.L	3	4.11	0.38	-6.21
IPI00168866	MAM domain containing glycosylphosphatidylinositol anchor 1	R.FKGQLLPPPPVVPAAAEAPDHAELR.L	3	4.17	0.31	-3.25
IPI00168866	MAM domain containing glycosylphosphatidylinositol anchor 1	R.VDKEAALLPSGLPLEETPDGK.L	3	4.21	0.33	-2.99
IPI00168866	MAM domain containing glycosylphosphatidylinositol anchor 1	R.VDKEAALLPSGLPLEETPDGKLR.L	3	3.46	0.34	-2.77
IPI00168866	MAM domain containing glycosylphosphatidylinositol anchor 1	R.VDVQYLDEPM*LTVHQTVSDVR.G	3	2.86	0.16	-2.97
IPI00168884	Renin receptor precursor	K.DHSPDLYSLELAGLDEIGKR.Y	3	4.05	0.55	-1.85
IPI00168884	Renin receptor precursor	K.DHSPDLYSLELAGLDEIGKR.Y	4	3.23	0.23	-0.83
IPI00168884	Renin receptor precursor	K.FADDM*YSLYGGNAVVELVTVK.S	2	3.58	0.16	
IPI00168884	Renin receptor precursor	K.SFDTSLIR.K	2	2.81	0.18	-2.26
IPI00168884	Renin receptor precursor	R.IPDVAALSMGFSVK.E	2	3.71	0.32	
IPI00168884	Renin receptor precursor	R.LFQENSVLSSLPLNSLSR.N	2	2.84	0.34	-4.32
IPI00168884	Renin receptor precursor	R.NNEVDLLFLSELQVLHDISSLLSR.H	3	3.98	0.21	
IPI00168884	Renin receptor precursor	R.YGEDSEQFRDASK.I	3	2.16	0.13	0.78
IPI00168885	Isoform 1 of Putative ATP-dependent RNA helicase DHX57	K.RYDWQAKSVHAENGK.I	2	2.22	0.27	
IPI00168920	collagen, type XXIV, alpha 1	R.EGIIGPTGR.T	2	1.58	0.16	
IPI00168920	collagen, type XXIV, alpha 1	R.GIPGPHGNPGLPGLPGPKGPK.G	2	1.89	0.17	
IPI00168921	Putative polypeptide N-acetylgalactosaminyltransferase-like protein 3	K.EGFLHLGALGTTTLLPDTR.C	3	3.52	0.31	-2.14

IPI00168921	Putative polypeptide N-acetylgalactosaminyltransferase-like protein 3	R.SGDAFHEIRPR.A	3	2.51	0.20	-4.10
IPI00168921	Putative polypeptide N-acetylgalactosaminyltransferase-like protein 3	R.VILPSIDNIKQDNFEVQR.Y	3	1.96	0.13	-2.58
IPI00169115	Olfactory receptor OR9-8	R.NISFSGCAVQMFFGFAM*GSTECLLLGM*M*AFDR.Y	3	2.91	0.11	
IPI00169259	Small VCP/p97-interacting protein	R.GILDVQSVQEK.R	2	3.39	0.28	-4.46
IPI00169285	Putative phospholipase B-like 2 precursor	K.GLEDSYEGR.V	2	2.84	0.36	-2.06
IPI00169285	Putative phospholipase B-like 2 precursor	R.VLTILEQIPGM*VVVADKTSELYQK.T	3	4.29	0.34	-1.30
IPI00169285	Putative phospholipase B-like 2 precursor	R.VLTILEQIPGMVVVADKTSELYQK.T	3	2.69	0.11	-3.94
IPI00169331	Phosphatidylcholine:ceramide cholinephosphotransferase 2	K.KYSRVQK.I	2	2.70	0.14	
IPI00169383	Phosphoglycerate kinase 1	K.ACANPAAGSVILLENLR.F	2	4.96	0.46	-3.84
IPI00169383	Phosphoglycerate kinase 1	K.AEPAKIEAFR.A	2	2.49	0.10	-3.23
IPI00169383	Phosphoglycerate kinase 1	K.ALESPERPFLAILGGAK.V	3	3.19	0.34	-2.49
IPI00169383	Phosphoglycerate kinase 1	K.ALM*DEVVK.A	2	2.65	0.10	-2.72
IPI00169383	Phosphoglycerate kinase 1	K.DVLFLKDCVGPEVEK.A	3	2.95	0.21	-1.88
IPI00169383	Phosphoglycerate kinase 1	K.ELNYFAK.A	2	1.45	0.20	-2.85
IPI00169383	Phosphoglycerate kinase 1	K.ITLPVDFVTADKFDENAK.T	2	4.54	0.49	-4.00
IPI00169383	Phosphoglycerate kinase 1	K.KYAEAVTR.A	2	1.91	0.16	-2.32
IPI00169383	Phosphoglycerate kinase 1	K.LGDVYVNDAFGTAHR.A	2	4.08	0.49	-3.82
IPI00169383	Phosphoglycerate kinase 1	K.LGDVYVNDAFGTAHR.A	3	3.83	0.44	-1.30
IPI00169383	Phosphoglycerate kinase 1	K.LTLDKLDVK.G	2	2.25	0.10	-0.89
IPI00169383	Phosphoglycerate kinase 1	K.NNQITNNQR.I	2	2.35	0.08	-1.61
IPI00169383	Phosphoglycerate kinase 1	K.QIVWNGPVGVFEWEAFAR.G	2	4.74	0.55	-3.28
IPI00169383	Phosphoglycerate kinase 1	K.QIVWNGPVGVFEWEAFAR.G	3	4.98	0.37	-3.86
IPI00169383	Phosphoglycerate kinase 1	K.SLLGKDVLFLK.D	3	2.06	0.11	-3.04
IPI00169383	Phosphoglycerate kinase 1	K.SVVLM*SHLGRPDGVPM*PDKYSLEPVAVELK.S	4	4.52	0.44	-1.89
IPI00169383	Phosphoglycerate kinase 1	K.VKAEPAKIEAFR.A	2	2.64	0.27	-3.58
IPI00169383	Phosphoglycerate kinase 1	K.VLNNM*EIGTSLFDEEGAK.I	2	4.86	0.45	-3.28
IPI00169383	Phosphoglycerate kinase 1	K.YAEAVTR.A	1	2.06	0.07	-3.57
IPI00169383	Phosphoglycerate kinase 1	K.YAEAVTR.A	2	2.47	0.07	-4.42
IPI00169383	Phosphoglycerate kinase 1	R.VDFNVPM*K.N	2	2.54	0.19	-2.61
IPI00169383	Phosphoglycerate kinase 1	R.VDFNVPM*KNNQITNNQR.I	3	3.01	0.25	-1.70
IPI00169426	Isoform 2 of Cytosolic 5'-nucleotidase 1B	K.LPSSSTSSR.T	1	1.75	0.17	-1.71
IPI00170635	Secreted and transmembrane protein 1 precursor	R.AHGQESAIFNEVAPGYFSR.D	2	5.43	0.62	-4.31
IPI00170635	Secreted and transmembrane protein 1 precursor	R.AHGQESAIFNEVAPGYFSR.D	3	3.97	0.33	-3.85
IPI00170692	Vesicle-associated membrane protein-associated protein A	K.EAKPDELM*DSK.L	2	1.71	0.13	-2.71
IPI00170692	Vesicle-associated membrane protein-associated protein A	K.FKGPFTDVVTTNLK.L	2	4.04	0.37	-3.84
IPI00170766	Isoform 2 of Protein CASC5	K.KTCTTQHQLPK.M	2	2.82	0.06	

IPI00170814	PTK7 protein tyrosine kinase 7 isoform b precursor	R.VFTAGSEER.V	2	2.39	0.21	-1.28
IPI00170814	PTK7 protein tyrosine kinase 7 isoform b precursor	R.VVLAPQDVVVAR.Y	2	3.00	0.36	-3.84
IPI00171196	keratin 13 isoform b	R.LAADDFR.L	2	2.47	0.23	-3.76
IPI00171199	Isoform 2 of Proteasome subunit alpha type-3	K.AVENSSTAIGIR.C	2	2.17	0.08	-1.98
IPI00171199	Isoform 2 of Proteasome subunit alpha type-3	R.VFQVEYAM*K.A	2	2.53	0.13	-2.42
IPI00171230	Isoform 2 of ELKS/RAB6-interacting/CAST family member 1	R.TNSTGGSSGSSVGGGSGK.T	2	2.30	0.11	
IPI00171410	Isoform 1 of Uncharacterized protein C3orf21	K.TNIRELFEEFDSFLPGAIIGIAR.E	3	4.56	0.34	-2.54
IPI00171410	Isoform 1 of Uncharacterized protein C3orf21	R.DHGYSDVFEAYFR.C	2	2.45	0.28	-5.04
IPI00171410	Isoform 1 of Uncharacterized protein C3orf21	R.DHGYSDVFEAYFR.C	3	3.51	0.22	-0.92
IPI00171410	Isoform 1 of Uncharacterized protein C3orf21	R.ELFEEFDSFLPGAIIGIAR.E	2	5.11	0.41	-2.80
IPI00171410	Isoform 1 of Uncharacterized protein C3orf21	R.ELFEEFDSFLPGAIIGIAR.E	3	4.77	0.15	-2.78
IPI00171410	Isoform 1 of Uncharacterized protein C3orf21	R.ETFSSATKR.L	2	1.84	0.14	-2.37
IPI00171410	Isoform 1 of Uncharacterized protein C3orf21	R.LLEPAQVQQLADK.Y	2	3.16	0.26	-3.02
IPI00171410	Isoform 1 of Uncharacterized protein C3orf21	R.LLEPAQVQQLADKYHFR.G	3	2.16	0.23	-2.53
IPI00171410	Isoform 1 of Uncharacterized protein C3orf21	R.VGGPPPEGLPGFNSGVM*LLNLEAM*R.Q	2	2.50	0.08	-4.96
IPI00171411	Golgi phosphoprotein 2	A.LSVSQENPEM*EGPER.D	2	3.47	0.36	-3.23
IPI00171411	Golgi phosphoprotein 2	K.FSYDLSQCINQM*K.E	2	4.00	0.44	-2.78
IPI00171411	Golgi phosphoprotein 2	K.GNVLGNSK.S	1	1.90	0.23	-2.91
IPI00171411	Golgi phosphoprotein 2	K.IQSSHNFQLESVNK.L	2	4.23	0.37	-2.90
IPI00171411	Golgi phosphoprotein 2	K.IQSSHNFQLESVNK.L	3	3.71	0.36	-5.24
IPI00171411	Golgi phosphoprotein 2	K.IQSSHNFQLESVNKLYQDEK.A	3	4.51	0.38	-1.79
IPI00171411	Golgi phosphoprotein 2	K.KNEFQGELEK.Q	2	2.57	0.15	-3.19
IPI00171411	Golgi phosphoprotein 2	K.KNEFQGELEK.Q	3	3.35	0.07	-4.16
IPI00171411	Golgi phosphoprotein 2	K.RQVEKEETNEIQVVNEEPQR.D	3	5.29	0.35	-3.48
IPI00171411	Golgi phosphoprotein 2	K.RQVEKEETNEIQVVNEEPQR.D	4	3.23	0.24	-3.37
IPI00171411	Golgi phosphoprotein 2	K.RQVEKEETNEIQVVNEEPQRDR.L	3	3.68	0.20	-3.13
IPI00171411	Golgi phosphoprotein 2	K.RQVEKEETNEIQVVNEEPQRDR.L	4	3.43	0.18	-2.06
IPI00171411	Golgi phosphoprotein 2	R.DQLVIPDGQEEEQEAAGEGR.N	2	4.47	0.46	-3.91
IPI00171411	Golgi phosphoprotein 2	R.DQLVIPDGQEEEQEAAGEGR.N	3	3.57	0.41	-2.33
IPI00171411	Golgi phosphoprotein 2	R.DRLPQEPGREQVVEDRPVGGR.G	3	3.19	0.17	-3.48
IPI00171411	Golgi phosphoprotein 2	R.DRLPQEPGREQVVEDRPVGGR.G	4	2.60	0.13	-1.96
IPI00171411	Golgi phosphoprotein 2	R.DTINLLDQR.E	1	2.89	0.22	-4.33
IPI00171411	Golgi phosphoprotein 2	R.DTINLLDQR.E	2	3.56	0.20	-2.62
IPI00171411	Golgi phosphoprotein 2	R.DTINLLDQRE.K	2	3.20	0.18	-3.02
IPI00171411	Golgi phosphoprotein 2	R.EQLDKIQSSHNFQLESVNK.L	2	4.60	0.50	-3.38
IPI00171411	Golgi phosphoprotein 2	R.EQLDKIQSSHNFQLESVNK.L	3	3.30	0.26	-3.10
IPI00171411	Golgi phosphoprotein 2	R.EQVVEDRPVGGR.G	2	1.68	0.05	-2.28
IPI00171411	Golgi phosphoprotein 2	R.GFGGAGELGQTPQVQAALSVSQENPEM*EGPER.D	3	6.13	0.49	-4.33
IPI00171411	Golgi phosphoprotein 2	R.KFSYDLSQCINQM*K.E	3	2.65	0.22	-2.67

IPI00171411	Golgi phosphoprotein 2	R.LPQEPGREQVVEDRPVGGR.G	2	2.14	0.08	-4.72
IPI00171411	Golgi phosphoprotein 2	R.LPQEPGREQVVEDRPVGGR.G	3	3.43	0.34	-4.90
IPI00171411	Golgi phosphoprotein 2	R.NIDVFNVEDQKR.D	2	3.42	0.39	-2.74
IPI00171411	Golgi phosphoprotein 2	R.QQLQALSEPQPR.L	2	3.01	0.39	-3.15
IPI00171411	Golgi phosphoprotein 2	R.QVEKEETNEIQVVNEEPQRDR.L	3	2.86	0.22	-3.61
IPI00171412	Isoform 1 of Sulfatase-modifying factor 2 precursor	K.SVLWWLPVEK.A	2	3.48	0.23	-2.80
IPI00171412	Isoform 1 of Sulfatase-modifying factor 2 precursor	R.EATVKPFAIDIFPVTNK.D	3	2.44	0.06	-3.14
IPI00171412	Isoform 1 of Sulfatase-modifying factor 2 precursor	R.MGNTPDSASDNLGFR.C	2	1.97	0.11	-2.20
IPI00171412	Isoform 1 of Sulfatase-modifying factor 2 precursor	W.IDTADGSANHR.A	2	2.96	0.23	-1.33
IPI00171438	Thioredoxin domain-containing protein 5 precursor	K.LFKPGQEAVK.Y	2	2.23	0.11	-1.84
IPI00171438	Thioredoxin domain-containing protein 5 precursor	R.DLESLREYVESQLQR.T	2	3.74	0.25	-3.39
IPI00171438	Thioredoxin domain-containing protein 5 precursor	R.DLESLREYVESQLQR.T	3	4.47	0.29	-2.78
IPI00171438	Thioredoxin domain-containing protein 5 precursor	R.EYVESQLQR.T	2	2.52	0.11	-1.44
IPI00171473	Spondin-1 precursor	K.AQWPAWQPLNVR.A	2	3.38	0.32	-2.13
IPI00171473	Spondin-1 precursor	K.IRPLTSLDHPQSPFYDPEGGSITQVAR.V	3	5.91	0.53	-3.61
IPI00171473	Spondin-1 precursor	K.IRPLTSLDHPQSPFYDPEGGSITQVAR.V	4	3.41	0.21	-3.29
IPI00171473	Spondin-1 precursor	K.SLAELGDCNEDLEQVEK.C	2	4.83	0.44	-5.41
IPI00171473	Spondin-1 precursor	R.AAPSAEFSVDR.T	2	3.39	0.46	-4.07
IPI00171473	Spondin-1 precursor	R.EGYTEFSLR.V	2	2.45	0.24	
IPI00171473	Spondin-1 precursor	R.GFTLIALR.E	2	2.62	0.13	-2.27
IPI00171473	Spondin-1 precursor	R.VTLSAAPPSYFR.G	2	3.11	0.37	-4.42
IPI00171611	Histone H3.2	R.FQSSAVMALQEASEAYLVGLFEDTNLCAIHAK.R	3	3.58	0.38	-1.97
IPI00171647	Isoform 1 of Sialic acid-binding Ig-like lectin 8 precursor	R.SCRKKSARPAAGVGDTGMEDAKAIR.G	2	1.63	0.17	
IPI00171678	Dopamine beta-hydroxylase	R.TPEGLTLLFK.R	2	3.47	0.41	-2.04
IPI00171737	Isoform 2 of Leucine-rich repeat and death domain-containing protein	R.MGLAPKDPALPGSSAPQPPEPAQA.-	3	2.35	0.10	-4.26
IPI00171874	Ras guanyl-releasing protein 3	R.M*TEEFREVASQLGYEK.H	2	2.56	0.17	
IPI00171928	Angiopoietin-related protein 7 precursor	A.AQTVTQTSADAIYDCSSLYQK.N	2	5.91	0.63	-3.92
IPI00171928	Angiopoietin-related protein 7 precursor	A.AQTVTQTSADAIYDCSSLYQK.N	3	5.42	0.41	-2.70
IPI00171928	Angiopoietin-related protein 7 precursor	K.SGLVSFYR.D	2	2.81	0.08	-2.77
IPI00171928	Angiopoietin-related protein 7 precursor	R.LRVEM*EDWEGNLR.Y	3	2.60	0.09	-1.85
IPI00171928	Angiopoietin-related protein 7 precursor	R.VEM*EDWEGNLR.Y	2	2.76	0.27	-3.69
IPI00171928	Angiopoietin-related protein 7 precursor	R.YAEYSHFVLGNELNSYR.L	2	5.88	0.61	-3.27
IPI00171928	Angiopoietin-related protein 7 precursor	R.YAEYSHFVLGNELNSYR.L	3	4.55	0.37	-3.11
IPI00174976	Isoform 1 of MAGUK p55 subfamily member 5	-.MTTSHMNGHVTEESDSEVK.N	3	2.84	0.17	-6.57
IPI00175019	similar to Temporarily Assigned Gene name family member	K.ANLTDMESGSSNAM*NMNVQHEREDKNIQK.M	3	3.96	0.21	

IPI00175654	Probable mast cell antigen 32 homolog precursor	K.SLQITYSLFR.R	2	2.71	0.30	-0.82
IPI00175989	Rho family guanine-nucleotide exchange factor	K.EDLQIYFKYHK.N	3	3.20	0.11	
IPI00175989	Rho family guanine-nucleotide exchange factor	K.KLEEKSQEPLEK.A	2	2.58	0.18	
IPI00176104	Isoform 1 of SLIT and NTRK-like protein 2 precursor	K.LLFLNNNLLR.T	2	2.74	0.20	-2.38
IPI00176104	Isoform 1 of SLIT and NTRK-like protein 2 precursor	K.VLILNDNLLLSLPSNVFR.F	3	3.85	0.25	-3.30
IPI00176193	Isoform 1 of Collagen alpha-1(XIV) chain precursor	K.ASAHAITGPPTELITSEVTAR.S	3	2.77	0.17	-2.92
IPI00176193	Isoform 1 of Collagen alpha-1(XIV) chain precursor	K.LQEIEGPSVSIM*EK.T	2	4.41	0.34	-3.85
IPI00176193	Isoform 1 of Collagen alpha-1(XIV) chain precursor	K.TKETLLDAIK.H	2	2.76	0.13	-2.79
IPI00176193	Isoform 1 of Collagen alpha-1(XIV) chain precursor	K.TLFLGVTNLQAK.H	2	4.11	0.35	-3.78
IPI00176193	Isoform 1 of Collagen alpha-1(XIV) chain precursor	K.TNQLNLQNTATK.A	2	4.25	0.43	-1.99
IPI00176193	Isoform 1 of Collagen alpha-1(XIV) chain precursor	K.VIVVITDGR.S	2	2.77	0.17	-1.79
IPI00176193	Isoform 1 of Collagen alpha-1(XIV) chain precursor	R.DTLFTAESGTR.R	2	3.95	0.44	-3.82
IPI00176193	Isoform 1 of Collagen alpha-1(XIV) chain precursor	R.ILPDTPQEPFALWEILNK.N	2	3.94	0.37	-4.75
IPI00176193	Isoform 1 of Collagen alpha-1(XIV) chain precursor	R.ILPDTPQEPFALWEILNK.N	3	3.82	0.16	-4.42
IPI00176193	Isoform 1 of Collagen alpha-1(XIV) chain precursor	R.ISNVGSNSAR.L	2	2.94	0.32	-0.73
IPI00176193	Isoform 1 of Collagen alpha-1(XIV) chain precursor	R.VVYYPTR.G	2	1.80	0.10	-1.77
IPI00176221	Neuronal growth regulator 1 precursor	C.SAENDVSFPDVR.K	2	3.16	0.23	-2.35
IPI00176221	Neuronal growth regulator 1 precursor	K.GDTAVLR.C	1	1.55	0.09	-1.71
IPI00176221	Neuronal growth regulator 1 precursor	K.KLFNGQQGIIIQNF.S	2	3.46	0.25	-4.00
IPI00176221	Neuronal growth regulator 1 precursor	K.KLFNGQQGIIIQNFSTR.S	2	5.70	0.59	-4.73
IPI00176221	Neuronal growth regulator 1 precursor	K.KLFNGQQGIIIQNFSTR.S	3	4.54	0.29	-4.61
IPI00176221	Neuronal growth regulator 1 precursor	K.LFNGQQGIIIQNFSTR.S	2	5.43	0.46	-3.46
IPI00176221	Neuronal growth regulator 1 precursor	K.LFNGQQGIIIQNFSTR.S	3	5.27	0.35	-3.28
IPI00176221	Neuronal growth regulator 1 precursor	K.VKVVVNFAPTIQEIK.S	3	3.24	0.34	-3.43
IPI00176221	Neuronal growth regulator 1 precursor	K.VVVNFAPTIQEIK.S	1	3.02	0.39	-2.92
IPI00176221	Neuronal growth regulator 1 precursor	K.VVVNFAPTIQEIK.S	2	4.07	0.34	-3.69
IPI00176221	Neuronal growth regulator 1 precursor	N.GQQGIIIQNFSTR.S	2	3.84	0.47	-1.26
IPI00176221	Neuronal growth regulator 1 precursor	P.AGQSVDFPWAAVDNM*M*VR.K	2	4.98	0.60	-2.57
IPI00176221	Neuronal growth regulator 1 precursor	P.AGQSVDFPWAAVDNM*M*VR.K	3	5.94	0.51	-1.98

IPI00176221	Neuronal growth regulator 1 precursor	R.CYLEDGASK.G	1	2.76	0.24	-4.71
IPI00176221	Neuronal growth regulator 1 precursor	R.CYLEDGASK.G	2	3.06	0.41	-2.96
IPI00176221	Neuronal growth regulator 1 precursor	R.DQAGEYECSAENDVSFPDVR.K	2	5.11	0.53	-3.43
IPI00176221	Neuronal growth regulator 1 precursor	R.DQAGEYECSAENDVSFPDVRK.V	2	4.82	0.57	-3.04
IPI00176221	Neuronal growth regulator 1 precursor	R.DQAGEYECSAENDVSFPDVRK.V	3	5.15	0.51	-3.91
IPI00176221	Neuronal growth regulator 1 precursor	R.DYSLQIQNVDVTDDGPYTCSVQTQHTPR.T	3	6.36	0.52	-3.66
IPI00176221	Neuronal growth regulator 1 precursor	R.DYSLQIQNVDVTDDGPYTCSVQTQHTPR.T	4	3.40	0.19	-2.07
IPI00176221	Neuronal growth regulator 1 precursor	R.HISPSAKPFENGQYLDIYGITR.D	2	4.75	0.43	-3.73
IPI00176221	Neuronal growth regulator 1 precursor	R.HISPSAKPFENGQYLDIYGITR.D	3	5.27	0.53	-4.51
IPI00176221	Neuronal growth regulator 1 precursor	R.HISPSAKPFENGQYLDIYGITR.D	4	3.46	0.27	-3.61
IPI00176221	Neuronal growth regulator 1 precursor	R.KGDTAVLR.C	1	2.01	0.05	-1.13
IPI00176221	Neuronal growth regulator 1 precursor	R.KGDTAVLR.C	2	2.93	0.11	-2.46
IPI00176221	Neuronal growth regulator 1 precursor	R.SSIIFAGGDK.W	1	2.49	0.22	-2.75
IPI00176221	Neuronal growth regulator 1 precursor	R.SSIIFAGGDK.W	2	3.11	0.19	-2.11
IPI00176221	Neuronal growth regulator 1 precursor	R.TM*QVHLTVQVPPK.I	2	4.06	0.50	-4.74
IPI00176221	Neuronal growth regulator 1 precursor	R.TM*QVHLTVQVPPK.I	3	4.96	0.49	-4.33
IPI00176221	Neuronal growth regulator 1 precursor	R.VSISTLNKR.D	2	2.45	0.12	-3.07
IPI00176398	Isoform 1 of SLIT and NTRK-like protein 6 precursor	K.VLILNDNAIESLPPNIFR.F	2	4.49	0.43	-3.93
IPI00176398	Isoform 1 of SLIT and NTRK-like protein 6 precursor	R.FVPLTHLDLR.G	2	2.17	0.11	-3.84
IPI00176398	Isoform 1 of SLIT and NTRK-like protein 6 precursor	R.GNQLQTLPYVGFLEHIGR.I	2	2.49	0.41	-2.95
IPI00176424	Neuroligin-2 precursor	K.GNYGLLDQIQALR.W	2	3.90	0.15	-2.82
IPI00176424	Neuroligin-2 precursor	K.TLLALFTDHQWVAPAVATAK.L	3	3.21	0.23	-3.33
IPI00176424	Neuroligin-2 precursor	Q.RGGGGPGGGAPGGPGLGLGSLGEERFPVVNTAYGR.V	3	6.71	0.60	-1.07
IPI00176424	Neuroligin-2 precursor	R.ELNNEILGPVVQFLGVPYATPPLGAR.R	3	4.20	0.41	-2.53
IPI00176424	Neuroligin-2 precursor	R.ELVDQDVQPAR.Y	2	3.12	0.18	-2.14
IPI00176424	Neuroligin-2 precursor	R.LGVLGFLSTGDQAAK.G	2	5.01	0.48	-3.60
IPI00176427	Cell adhesion molecule 4 precursor	K.DDGGIIICEAQNQALPSGHSK.Q	2	4.23	0.51	-5.11
IPI00176427	Cell adhesion molecule 4 precursor	K.ELKGVSSSQENGK.V	2	2.83	0.32	-5.57
IPI00176427	Cell adhesion molecule 4 precursor	K.GVSSSQENGKVWSVASTVR.F	2	4.87	0.49	-3.57
IPI00176427	Cell adhesion molecule 4 precursor	K.GVSSSQENGKVWSVASTVR.F	3	3.83	0.36	-3.41
IPI00176427	Cell adhesion molecule 4 precursor	K.QTQYVLDVQYSPTAR.I	2	4.43	0.46	-5.61
IPI00176427	Cell adhesion molecule 4 precursor	K.VWSVASTVR.F	2	2.88	0.11	-1.70
IPI00176427	Cell adhesion molecule 4 precursor	L.TVLVAPENPVVEVR.E	2	3.53	0.30	-1.89
IPI00176427	Cell adhesion molecule 4 precursor	R.EGDTLVLTCAVTGNPRPNQIR.W	3	4.69	0.54	-2.56
IPI00176427	Cell adhesion molecule 4 precursor	R.EQAVEGGEVELSCLVPR.S	2	5.35	0.53	-4.69
IPI00176427	Cell adhesion molecule 4 precursor	R.EQAVEGGEVELSCLVPR.S	3	3.22	0.21	-2.48
IPI00176427	Cell adhesion molecule 4 precursor	R.FQLEEFSPR.R	1	2.43	0.16	-3.49
IPI00176427	Cell adhesion molecule 4 precursor	R.FQLEEFSPR.R	2	3.53	0.22	-2.28
IPI00176427	Cell adhesion molecule 4 precursor	R.FQLEEFSPRR.V	2	2.50	0.12	-4.42

IPI00176427	Cell adhesion molecule 4 precursor	R.IHASQAVVR.E	2	2.10	0.21	
IPI00176427	Cell adhesion molecule 4 precursor	R.KDDGGIIICEAQNQALPSGHSK.Q	3	6.09	0.45	-3.03
IPI00176427	Cell adhesion molecule 4 precursor	R.KDDGGIIICEAQNQALPSGHSK.Q	4	3.36	0.22	-3.01
IPI00176427	Cell adhesion molecule 4 precursor	R.LHQYDGSIVVIQNPAR.Q	2	5.61	0.57	-4.05
IPI00176427	Cell adhesion molecule 4 precursor	R.LHQYDGSIVVIQNPAR.Q	3	5.29	0.24	-2.76
IPI00176458	protocadherin 1 isoform 2 precursor	A.TRVVYKVPEEQPPNTLIGSLAADYGFPDVGHLYK.L	4	6.01	0.51	-4.20
IPI00176458	protocadherin 1 isoform 2 precursor	K.ANDSDQGANAEIEYTFHQAPEVVR.R	3	3.67	0.41	-4.56
IPI00176458	protocadherin 1 isoform 2 precursor	K.DM*NDNAPTIEIR.G	2	3.71	0.33	-4.70
IPI00176458	protocadherin 1 isoform 2 precursor	K.GLFTISPETGEIQVK.T	2	2.88	0.28	-5.85
IPI00176458	protocadherin 1 isoform 2 precursor	K.LEVGAPYLR.V	2	2.77	0.21	-1.17
IPI00176458	protocadherin 1 isoform 2 precursor	K.TGDIFTTETSIDR.E	2	3.85	0.22	-1.60
IPI00176458	protocadherin 1 isoform 2 precursor	K.TGDIFTTETSIDREGLR.E	2	3.24	0.27	-3.04
IPI00176458	protocadherin 1 isoform 2 precursor	K.TGDIFTTETSIDREGLR.E	3	2.76	0.24	-2.48
IPI00176458	protocadherin 1 isoform 2 precursor	K.VPEEQPPNTLIGSLAADYGFPDVGHLYK.L	3	7.65	0.62	-3.38
IPI00176458	protocadherin 1 isoform 2 precursor	K.YFLQTTTPLDYEK.V	2	2.54	0.30	-5.06
IPI00176458	protocadherin 1 isoform 2 precursor	R.EQQSTYTFQLK.A	2	2.50	0.23	-3.35
IPI00176458	protocadherin 1 isoform 2 precursor	R.GGQEPAGAGSPSPPEDR.N	2	4.30	0.46	-2.92
IPI00176458	protocadherin 1 isoform 2 precursor	R.NTGLITVQGPVDREDLSTLR.F	2	4.03	0.39	-3.89
IPI00176458	protocadherin 1 isoform 2 precursor	R.NTGLITVQGPVDREDLSTLR.F	3	3.62	0.41	-2.87
IPI00176458	protocadherin 1 isoform 2 precursor	R.TLLETLLGHSLDTPLDIDIAGDPEYER.S	3	3.45	0.31	-5.30
IPI00176458	protocadherin 1 isoform 2 precursor	R.VVYKVPEEQPPNTLIGSLAADYGFPDVGHLYK.L	3	5.12	0.57	-2.94
IPI00176458	protocadherin 1 isoform 2 precursor	R.WDSYDLTIK.V	2	2.07	0.24	-1.48
IPI00176581	Isoform 1 of Fanconi anemia group M protein	R.LWQDHPLPTHQVDHSDR.C	3	2.90	0.19	-3.61
IPI00176581	Isoform 1 of Fanconi anemia group M protein	R.STFIAPR.K	1	1.69	0.18	-1.09
IPI00176920	Nephrocystin-4	K.QPAEAVSATEPVTFNPQK.E	2	2.68	0.15	-7.95
IPI00177543	peptidylglycine alpha-amidating monooxygenase isoform a, preproprotein	D.PNNKEGPVLILGR.S	3	3.59	0.20	-0.71
IPI00177543	peptidylglycine alpha-amidating monooxygenase isoform a, preproprotein	G.ADSEHKLETSSGR.V	2	3.50	0.42	-1.26
IPI00177543	peptidylglycine alpha-amidating monooxygenase isoform a, preproprotein	I.PVDEEAFVIDFKPR.A	3	5.08	0.47	-2.28
IPI00177543	peptidylglycine alpha-amidating monooxygenase isoform a, preproprotein	K.AGIEVQEIK.E	1	2.05	0.06	-3.46
IPI00177543	peptidylglycine alpha-amidating monooxygenase isoform a, preproprotein	K.AGIEVQEIK.E	2	2.87	0.11	-1.75
IPI00177543	peptidylglycine alpha-amidating monooxygenase isoform a, preproprotein	K.AGIEVQEIKEAEAVVETK.M	2	4.83	0.43	-4.29
IPI00177543	peptidylglycine alpha-amidating monooxygenase isoform a, preproprotein	K.AGIEVQEIKEAEAVVETK.M	3	4.16	0.37	-4.08
IPI00177543	peptidylglycine alpha-amidating monooxygenase isoform a, preproprotein	K.ANILYAWAR.N	2	3.66	0.33	-0.81

IPI00177543	peptidylglycine alpha-amidating monooxygenase isoform a, preproprotein	K.DGNYWVTDVALHQVFK.L	3	3.05	0.39	-3.20
IPI00177543	peptidylglycine alpha-amidating monooxygenase isoform a, preproprotein	K.EAEAVVETK.M	2	2.24	0.19	-2.00
IPI00177543	peptidylglycine alpha-amidating monooxygenase isoform a, preproprotein	K.EGPVLILGR.S	2	1.99	0.05	-0.91
IPI00177543	peptidylglycine alpha-amidating monooxygenase isoform a, preproprotein	K.ETEYKDKIPLLQQPK.R	2	4.24	0.38	-4.61
IPI00177543	peptidylglycine alpha-amidating monooxygenase isoform a, preproprotein	K.FITQWGEESSGSSPLPGQFTVPHSLALVPLLGQLCVADR.E	3	6.02	0.56	-2.67
IPI00177543	peptidylglycine alpha-amidating monooxygenase isoform a, preproprotein	K.FITQWGEESSGSSPLPGQFTVPHSLALVPLLGQLCVADR.E	4	4.41	0.36	-2.40
IPI00177543	peptidylglycine alpha-amidating monooxygenase isoform a, preproprotein	K.FTLTEKLEHR.S	2	3.00	0.31	-4.14
IPI00177543	peptidylglycine alpha-amidating monooxygenase isoform a, preproprotein	K.FTLTEKLEHR.S	3	2.73	0.27	-4.40
IPI00177543	peptidylglycine alpha-amidating monooxygenase isoform a, preproprotein	K.FVYQQIGLGPIEEDTILVIDPNNAAVLQSSGK.N	2	3.10	0.58	-2.93
IPI00177543	peptidylglycine alpha-amidating monooxygenase isoform a, preproprotein	K.FVYQQIGLGPIEEDTILVIDPNNAAVLQSSGK.N	3	7.50	0.62	-5.69
IPI00177543	peptidylglycine alpha-amidating monooxygenase isoform a, preproprotein	K.FVYQQIGLGPIEEDTILVIDPNNAAVLQSSGK.N	4	4.72	0.40	-3.52
IPI00177543	peptidylglycine alpha-amidating monooxygenase isoform a, preproprotein	K.GSGGLNLGNFFASR.K	2	4.19	0.40	-2.67
IPI00177543	peptidylglycine alpha-amidating monooxygenase isoform a, preproprotein	K.HAVSFM*TCTQNVAPDM*FR.T	2	5.15	0.58	-3.91
IPI00177543	peptidylglycine alpha-amidating monooxygenase isoform a, preproprotein	K.IPLLQQPK.R	2	2.50	0.13	-0.92
IPI00177543	peptidylglycine alpha-amidating monooxygenase isoform a, preproprotein	K.KAGIEVQEIK.E	2	2.77	0.18	-0.24
IPI00177543	peptidylglycine alpha-amidating monooxygenase isoform a, preproprotein	K.LDPNNKEGPVLILGR.S	2	3.26	0.32	-2.86
IPI00177543	peptidylglycine alpha-amidating monooxygenase isoform a, preproprotein	K.LDPNNKEGPVLILGR.S	3	4.10	0.41	-1.88
IPI00177543	peptidylglycine alpha-amidating monooxygenase isoform a, preproprotein	K.LIKEPGSGVPVVL.I	2	3.06	0.40	-3.41
IPI00177543	peptidylglycine alpha-amidating monooxygenase isoform a, preproprotein	K.LIKEPGSGVPVVLI.T	2	3.04	0.36	-2.06
IPI00177543	peptidylglycine alpha-amidating monooxygenase isoform a, preproprotein	K.LIKEPGSGVPVVLIT.T	2	3.58	0.41	-3.08
IPI00177543	peptidylglycine alpha-amidating monooxygenase isoform a, preproprotein	K.LLGEREDVVHVHK.Y	2	2.76	0.29	-2.89

IPI00177543	peptidylglycine alpha-amidating monooxygenase isoform a, preproprotein	K.LLGEREDVVHVHK.Y	3	3.30	0.38	-2.36
IPI00177543	peptidylglycine alpha-amidating monooxygenase isoform a, preproprotein	K.M*ENKPTSSELQ.K	2	3.29	0.17	-1.04
IPI00177543	peptidylglycine alpha-amidating monooxygenase isoform a, preproprotein	K.M*ENKPTSSELQK.M	2	3.52	0.25	-3.90
IPI00177543	peptidylglycine alpha-amidating monooxygenase isoform a, preproprotein	K.M*ENKPTSSELQK.M	3	2.51	0.06	-1.91
IPI00177543	peptidylglycine alpha-amidating monooxygenase isoform a, preproprotein	K.NLFYLPHGLSIDK.D	2	2.86	0.20	-2.77
IPI00177543	peptidylglycine alpha-amidating monooxygenase isoform a, preproprotein	K.NNLVIFHR.G	1	2.60	0.12	-0.99
IPI00177543	peptidylglycine alpha-amidating monooxygenase isoform a, preproprotein	K.NNLVIFHR.G	2	2.82	0.17	0.64
IPI00177543	peptidylglycine alpha-amidating monooxygenase isoform a, preproprotein	K.NYPM*HVFAYR.V	2	2.15	0.16	-0.94
IPI00177543	peptidylglycine alpha-amidating monooxygenase isoform a, preproprotein	K.QSDTYFCM*SM*R.I	2	2.86	0.44	-3.09
IPI00177543	peptidylglycine alpha-amidating monooxygenase isoform a, preproprotein	K.REEEEVLDQGDFYSLLSK.L	2	5.61	0.52	-5.66
IPI00177543	peptidylglycine alpha-amidating monooxygenase isoform a, preproprotein	K.REEEEVLDQGDFYSLLSK.L	3	5.34	0.38	-4.43
IPI00177543	peptidylglycine alpha-amidating monooxygenase isoform a, preproprotein	K.TDTKEFVR.E	1	2.09	0.23	-2.78
IPI00177543	peptidylglycine alpha-amidating monooxygenase isoform a, preproprotein	K.TDTKEFVR.E	2	2.18	0.19	-2.34
IPI00177543	peptidylglycine alpha-amidating monooxygenase isoform a, preproprotein	K.VVSGYR.V	1	1.17	0.08	-2.62
IPI00177543	peptidylglycine alpha-amidating monooxygenase isoform a, preproprotein	K.YNPTEKAESESDLVAEIANVVQ.K	2	4.87	0.46	-0.06
IPI00177543	peptidylglycine alpha-amidating monooxygenase isoform a, preproprotein	L.DPNNKEGPVLILGR.S	3	3.85	0.35	-0.73
IPI00177543	peptidylglycine alpha-amidating monooxygenase isoform a, preproprotein	P.QAFYPVGHPVDVSFGDLLAAR.C	3	3.88	0.39	-3.12
IPI00177543	peptidylglycine alpha-amidating monooxygenase isoform a, preproprotein	R.EDVVHVHK.Y	2	2.20	0.27	-2.42
IPI00177543	peptidylglycine alpha-amidating monooxygenase isoform a, preproprotein	R.EEEEVLDQGDFYSLLSK.L	2	5.52	0.53	-4.70
IPI00177543	peptidylglycine alpha-amidating monooxygenase isoform a, preproprotein	R.EEEEVLDQGDFYSLLSK.L	3	5.40	0.45	-3.25
IPI00177543	peptidylglycine alpha-amidating monooxygenase isoform a, preproprotein	R.EGAEHERGNAILVR.D	2	2.14	0.09	-2.81

IPI00177543	peptidylglycine alpha-amidating monooxygenase isoform a, preproprotein	R.GKGSGGLNLGNFFASR.K	2	5.47	0.55	-4.07
IPI00177543	peptidylglycine alpha-amidating monooxygenase isoform a, preproprotein	R.GKGSGGLNLGNFFASR.K	3	4.18	0.39	-2.26
IPI00177543	peptidylglycine alpha-amidating monooxygenase isoform a, preproprotein	R.IPVDEEAFVIDFKPR.A	2	4.95	0.56	-6.23
IPI00177543	peptidylglycine alpha-amidating monooxygenase isoform a, preproprotein	R.IPVDEEAFVIDFKPR.A	3	4.65	0.48	-4.22
IPI00177543	peptidylglycine alpha-amidating monooxygenase isoform a, preproprotein	R.IVQFSPSGK.F	1	2.27	0.17	-3.21
IPI00177543	peptidylglycine alpha-amidating monooxygenase isoform a, preproprotein	R.IVQFSPSGK.F	2	2.61	0.22	-2.07
IPI00177543	peptidylglycine alpha-amidating monooxygenase isoform a, preproprotein	R.LSTEGSDQEKEDDGSESEEEYSAPLPALAPSSS.-	3	3.29	0.39	-0.99
IPI00177543	peptidylglycine alpha-amidating monooxygenase isoform a, preproprotein	R.M*PGVTPK.Q	1	1.96	0.06	-2.26
IPI00177543	peptidylglycine alpha-amidating monooxygenase isoform a, preproprotein	R.NAPPTRLPK.G	2	2.59	0.14	-2.09
IPI00177543	peptidylglycine alpha-amidating monooxygenase isoform a, preproprotein	R.NGQWTLIGR.Q	1	2.01	0.11	-2.79
IPI00177543	peptidylglycine alpha-amidating monooxygenase isoform a, preproprotein	R.NGQWTLIGR.Q	2	3.26	0.29	-0.53
IPI00177543	peptidylglycine alpha-amidating monooxygenase isoform a, preproprotein	R.QSPQLPQAFYPVGHPVDVSFGDLLAAR.C	2	3.13	0.49	-6.57
IPI00177543	peptidylglycine alpha-amidating monooxygenase isoform a, preproprotein	R.QSPQLPQAFYPVGHPVDVSFGDLLAAR.C	3	4.67	0.54	-4.57
IPI00177543	peptidylglycine alpha-amidating monooxygenase isoform a, preproprotein	R.QSPQLPQAFYPVGHPVDVSFGDLLAAR.C	4	4.72	0.53	-3.46
IPI00177543	peptidylglycine alpha-amidating monooxygenase isoform a, preproprotein	R.SM*QPGSDQNHFCQPTDVAVDPGTGAIYVSDGYCNSR.I	3	6.06	0.62	-4.42
IPI00177543	peptidylglycine alpha-amidating monooxygenase isoform a, preproprotein	R.SM*QPGSDQNHFCQPTDVAVDPGTGAIYVSDGYCNSR.I	4	4.81	0.43	-5.02
IPI00177543	peptidylglycine alpha-amidating monooxygenase isoform a, preproprotein	R.TEATHIGGTSSDEM*CNLYIM*YYM*EAK.H	3	4.79	0.45	-3.46
IPI00177543	peptidylglycine alpha-amidating monooxygenase isoform a, preproprotein	W.PGVYLLPGQVSGVALDPK.N	2	4.54	0.42	-2.14
IPI00177878	Isoform 3 of Transmembrane protein 16D	-.M*EASSSGITNGK.T	2	1.62	0.09	2.62
IPI00178302	Isoform 4 of Semaphorin-6D precursor	A.VSFPEDDEPLNTVDYHYSR.Q	2	4.12	0.51	-2.83
IPI00178302	Isoform 4 of Semaphorin-6D precursor	K.HGLAEAYK.T	1	2.23	0.32	-3.64
IPI00178302	Isoform 4 of Semaphorin-6D precursor	K.HGLAEAYK.T	2	2.78	0.23	-2.78
IPI00178302	Isoform 4 of Semaphorin-6D precursor	K.HGLAEAYKTSIDFPDETLSFIK.S	3	3.43	0.42	-4.69
IPI00178302	Isoform 4 of Semaphorin-6D precursor	K.IRDTLYIAGR.D	2	3.07	0.18	-2.17

IPI00178302	Isoform 4 of Semaphorin-6D precursor	K.LYSATVADFLASDAVIYR.S	2	5.52	0.49	-4.89
IPI00178302	Isoform 4 of Semaphorin-6D precursor	K.LYSATVADFLASDAVIYR.S	3	2.17	0.12	-3.52
IPI00178302	Isoform 4 of Semaphorin-6D precursor	K.TSIDFPDETLSFIK.S	2	3.29	0.29	-4.41
IPI00178302	Isoform 4 of Semaphorin-6D precursor	Q.DDPNTSDFTDPLSGIPK.G	2	4.61	0.55	-1.45
IPI00178302	Isoform 4 of Semaphorin-6D precursor	R.DQVYTVNLNEM*PK.T	2	4.45	0.39	-3.79
IPI00178302	Isoform 4 of Semaphorin-6D precursor	R.DQVYTVNLNEM*PKTEVIPNKK.L	3	3.76	0.39	-2.87
IPI00178302	Isoform 4 of Semaphorin-6D precursor	R.DTLYIAGR.D	2	2.53	0.08	-2.74
IPI00178302	Isoform 4 of Semaphorin-6D precursor	R.EIAVEHNNLGK.A	3	2.79	0.17	-2.90
IPI00178302	Isoform 4 of Semaphorin-6D precursor	R.KFVVQDDPNTSDFTDPLSGIPK.G	3	3.37	0.32	-2.77
IPI00178302	Isoform 4 of Semaphorin-6D precursor	R.LSTLEYDGEEISGLAR.C	2	4.38	0.51	-3.19
IPI00178302	Isoform 4 of Semaphorin-6D precursor	R.QTNVALFADGK.L	1	2.11	0.28	-2.37
IPI00178302	Isoform 4 of Semaphorin-6D precursor	R.QTNVALFADGK.L	2	2.91	0.43	-3.15
IPI00178302	Isoform 4 of Semaphorin-6D precursor	R.SM*GDGSALR.T	2	2.44	0.34	-3.79
IPI00178352	Isoform 1 of Filamin-C	K.AFGPGLEPTGCIVDKPAEFTIDAR.A	3	2.26	0.17	-2.10
IPI00178352	Isoform 1 of Filamin-C	R.TGVEVGKPTHFTVLTK.G	3	2.08	0.13	-1.45
IPI00178352	Isoform 1 of Filamin-C	R.TGVEVGKPTHFTVLTK.G	4	2.95	0.16	-2.66
IPI00178727	Novel protein	K.DICNAM*GSKLTCEKIVKERYENM*MQQQK.L	3	2.65	0.15	1.82
IPI00178727	Novel protein	K.KVASM*M*ESK.D	1	1.32	0.17	-3.20
IPI00178767	Acid sphingomyelinase-like phosphodiesterase 3a precursor	K.GSPVNSLFVAPAVTPVK.S	2	3.41	0.42	-4.76
IPI00178767	Acid sphingomyelinase-like phosphodiesterase 3a precursor	K.KGSPVNSLFVAPAVTPVK.S	2	4.30	0.42	-4.17
IPI00178767	Acid sphingomyelinase-like phosphodiesterase 3a precursor	K.LEYILTQTYDIEDLQPESLYGLAK.Q	3	3.75	0.15	-5.33
IPI00178767	Acid sphingomyelinase-like phosphodiesterase 3a precursor	K.QFTILDSK.Q	2	1.85	0.19	-1.84
IPI00178767	Acid sphingomyelinase-like phosphodiesterase 3a precursor	K.VTTNPNLR.I	2	2.09	0.07	-1.92
IPI00178767	Acid sphingomyelinase-like phosphodiesterase 3a precursor	K.YSDVIAGQFYGHTHR.D	2	4.13	0.54	-4.05
IPI00178767	Acid sphingomyelinase-like phosphodiesterase 3a precursor	K.YSDVIAGQFYGHTHR.D	4	3.58	0.23	-4.26
IPI00178767	Acid sphingomyelinase-like phosphodiesterase 3a precursor	R.DSIM*VLSDK.K	2	3.06	0.11	-0.03
IPI00178894	Zinc finger and BTB domain-containing protein 20	R.SFYSGAM*VSHHETALGLPRDHHMEDPSWITR.I	4	3.13	0.18	1.36
IPI00178926	immunoglobulin J chain	K.CDPTEVELDNQIVTATQSNICDEDSATETCYTYDR.N	3	6.55	0.39	
IPI00178926	immunoglobulin J chain	K.CYTAVVPLVYGGETK.M	2	4.60	0.39	
IPI00178926	immunoglobulin J chain	R.FVYHLSDLCK.K	2	3.11	0.28	
IPI00178926	immunoglobulin J chain	R.SSEDPNEDIVER.N	2	3.03	0.12	
IPI00179473	Isoform 1 of Sequestosome-1	R.LTPVSPESSSTEEK.S	2	2.21	0.31	-5.28
IPI00179589	Myotrophin	R.KPLHYAADCGQLEILEFLLLK.G	3	3.69	0.24	-1.36
IPI00179851	NDT80/PhoG like DNA-binding family protein	K.NSPSLGFHGR.A	2	2.10	0.12	-2.48

IPI00179851	NDT80/PhoG like DNA-binding family protein	R.SGPSQM*ALLPVTNIR.A	2	3.23	0.26	-3.13
IPI00179851	NDT80/PhoG like DNA-binding family protein	R.WPITILSFR.E	2	2.91	0.23	-2.82
IPI00180240	thymosin-like 3	K.ETIEQEKQAGES.-	2	3.61	0.29	-2.72
IPI00180240	thymosin-like 3	K.KTETQEKNPLPSK.E	2	2.99	0.35	-4.88
IPI00180240	thymosin-like 3	K.KTETQEKNPLPSKET.I	3	3.58	0.31	-3.44
IPI00180240	thymosin-like 3	K.KTETQEKNPLPSKETIEQEK.Q	3	2.96	0.08	-4.80
IPI00180240	thymosin-like 3	K.KTETQEKNPLPSKETIEQEKQ.A	3	4.82	0.47	-3.70
IPI00180240	thymosin-like 3	K.KTETQEKNPLPSKETIEQEKQAG.E	3	3.59	0.27	-3.04
IPI00180240	thymosin-like 3	K.KTETQEKNPLPSKETIEQEKQAGES.-	2	3.61	0.48	-4.33
IPI00180240	thymosin-like 3	K.KTETQEKNPLPSKETIEQEKQAGES.-	3	5.43	0.49	-3.86
IPI00180240	thymosin-like 3	K.KTETQEKNPLPSKETIEQEKQAGES.-	4	3.60	0.28	-3.96
IPI00180240	thymosin-like 3	K.LKKTETQEKNPLPSKETIEQEKQAGES.-	4	5.38	0.48	-4.34
IPI00180240	thymosin-like 3	K.NPLPSKETIEQEK.Q	3	2.26	0.22	-1.61
IPI00180240	thymosin-like 3	K.NPLPSKETIEQEKQ.A	2	3.30	0.37	-2.04
IPI00180240	thymosin-like 3	K.NPLPSKETIEQEKQAGES.-	2	3.59	0.39	-3.10
IPI00180240	thymosin-like 3	K.NPLPSKETIEQEKQAGES.-	3	2.94	0.35	-2.16
IPI00180240	thymosin-like 3	K.TETQEKNPLPSK.E	2	3.81	0.38	-3.95
IPI00180240	thymosin-like 3	K.TETQEKNPLPSK.E	3	2.64	0.13	-2.28
IPI00180240	thymosin-like 3	K.TETQEKNPLPSKETIEQ.E	2	3.93	0.33	-3.08
IPI00180240	thymosin-like 3	K.TETQEKNPLPSKETIEQEK.Q	2	5.04	0.43	-3.70
IPI00180240	thymosin-like 3	K.TETQEKNPLPSKETIEQEK.Q	3	4.32	0.40	-4.31
IPI00180240	thymosin-like 3	K.TETQEKNPLPSKETIEQEKQ.A	3	4.09	0.34	-3.58
IPI00180240	thymosin-like 3	K.TETQEKNPLPSKETIEQEKQAGES.-	2	4.30	0.50	-3.66
IPI00180240	thymosin-like 3	K.TETQEKNPLPSKETIEQEKQAGES.-	3	4.55	0.40	-4.15
IPI00180384	dynein, axonemal, heavy chain 7	K.WLIFDGPVDAVWIENMNTVLDDNK.K	3	3.48	0.11	
IPI00180386	Isoform GN-1L of Glycogenin-1	K.VVHFLGR.V	2	1.84	0.17	-3.91
IPI00180426	Isoform 3 of G protein-coupled receptor kinase 4	R.IKKRKGEAM*ALNEKR.I	2	2.07	0.19	
IPI00180707	Isoform 1 of FRAS1-related extracellular matrix protein 2 precursor	K.AFQELGVR.Y	2	2.44	0.06	-2.40
IPI00180707	Isoform 1 of FRAS1-related extracellular matrix protein 2 precursor	R.ALLSPGLAGAAGVPAEEAIVLANR.G	3	4.80	0.46	-4.15
IPI00180707	Isoform 1 of FRAS1-related extracellular matrix protein 2 precursor	R.NLPLVVEELLGTSNALDAR.S	3	4.93	0.50	-3.65
IPI00180707	Isoform 1 of FRAS1-related extracellular matrix protein 2 precursor	R.VGILSGLGALPR.Y	2	3.48	0.26	-2.27
IPI00181079	Meteorin-like protein precursor	R.GSIQQVTHEPERQDSAIHLR.V	3	3.42	0.24	-5.53
IPI00181079	Meteorin-like protein precursor	R.GSIQQVTHEPERQDSAIHLR.V	4	3.09	0.24	-2.50
IPI00181079	Meteorin-like protein precursor	R.SFTDSSGANIYLEK.T	2	4.56	0.31	
IPI00181174	Isoform 1 of Neuroligin-4, X-linked precursor	K.GNYGLLDQIQALR.W	2	3.90	0.15	-2.82
IPI00181174	Isoform 1 of Neuroligin-4, X-linked precursor	R.ILADKVGCNM*LDTTDM*VECLR.N	3	4.22	0.46	-3.11
IPI00181174	Isoform 1 of Neuroligin-4, X-linked precursor	R.LGILGFLSTGDQAAK.G	2	4.85	0.55	-3.56
IPI00181174	Isoform 1 of Neuroligin-4, X-linked precursor	R.TPLPNEILGPVEQYLGVPYASPPTGER.R	3	3.18	0.26	-4.02

IPI00181174	Isoform 1 of Neuroligin-4, X-linked precursor	R.TPLPNEILGPVEQYLGVPYASPPTGERR.F	3	3.62	0.42	-2.09
IPI00181743	Isoform 1 of BAI1-associated protein 3	K.CLGKLQLFQPSFEICPFESELNMDIAAALK.R	3	3.29	0.12	
IPI00181743	Isoform 1 of BAI1-associated protein 3	R.FGRLSVRCHYEAAEQR.L	2	2.95	0.06	
IPI00182138	Isoform 2 of Granulins precursor	K.APAHLSLPDPQALKR.D	3	2.86	0.30	-1.10
IPI00182138	Isoform 2 of Granulins precursor	R.CPDGSTCCELPSGK.Y	2	3.06	0.33	-4.26
IPI00182138	Isoform 2 of Granulins precursor	R.SRCPDGSTCCELPSGK.Y	3	3.61	0.32	
IPI00182194	Teneurin-2	R.DYDVLAGR.W	2	2.77	0.19	-3.00
IPI00182194	Teneurin-2	R.IASIKPVISETPLPVDLYR.Y	2	3.05	0.44	-3.86
IPI00182194	Teneurin-2	R.IASIKPVISETPLPVDLYR.Y	3	2.73	0.13	-2.95
IPI00182438	Isoform 2 of Contactin-5 precursor	K.FIYRDESVPPLTPFEVK.V	3	2.73	0.27	-2.16
IPI00182438	Isoform 2 of Contactin-5 precursor	K.IEVHFPFTVTAAK.G	3	2.51	0.18	-1.13
IPI00182438	Isoform 2 of Contactin-5 precursor	R.AQASSADLM*IR.N	2	2.81	0.25	-0.61
IPI00182438	Isoform 2 of Contactin-5 precursor	R.NDGVM*GEYEPK.I	2	3.10	0.35	-3.94
IPI00182438	Isoform 2 of Contactin-5 precursor	R.SPFSLGWQTVK.T	2	3.74	0.33	-0.70
IPI00182438	Isoform 2 of Contactin-5 precursor	R.VVATNPIGTGDPSTPSR.M	2	4.73	0.47	-3.11
IPI00182944	Isoform 3 of Calcium/calmodulin-dependent protein kinase type II beta chain	K.ICDPGLTSFEPEALGNLVEGMDFHR.F	3	4.55	0.31	
IPI00182944	Isoform 3 of Calcium/calmodulin-dependent protein kinase type II beta chain	K.TTEQLIEAVNNGDFEAYAK.I	2	4.90	0.53	-1.54
IPI00182944	Isoform 3 of Calcium/calmodulin-dependent protein kinase type II beta chain	R.FYFENLLAK.N	2	2.81	0.14	-2.98
IPI00182944	Isoform 3 of Calcium/calmodulin-dependent protein kinase type II beta chain	R.LTQYIDGQGRPR.T	2	2.94	0.17	-3.26
IPI00183002	Isoform 1 of Protein phosphatase 1 regulatory subunit 12A	R.ISEM*EEELKM*LPDLK.A	2	2.20	0.15	
IPI00183206	Isoform 1 of RIM-binding protein 2	R.KITLIKQLAK.S	2	2.50	0.16	
IPI00183321	Isoform 1 of N-acetylgalactosamine 4-sulfate 6-O-sulfotransferase	K.LIINSITTR.I	2	3.08	0.13	-0.75
IPI00183321	Isoform 1 of N-acetylgalactosamine 4-sulfate 6-O-sulfotransferase	K.VFQFLNLGPLSEK.Q	2	4.16	0.44	-6.63
IPI00183321	Isoform 1 of N-acetylgalactosamine 4-sulfate 6-O-sulfotransferase	K.VFQFLNLGPLSEKQEALM*TK.S	2	2.93	0.40	0.90
IPI00183321	Isoform 1 of N-acetylgalactosamine 4-sulfate 6-O-sulfotransferase	R.DRYPVEDYLDLFDLAAHQIHQGLQASSAK.E	4	5.64	0.51	-4.35
IPI00183321	Isoform 1 of N-acetylgalactosamine 4-sulfate 6-O-sulfotransferase	R.LEDHASNVK.Y	2	2.86	0.22	-2.12
IPI00183321	Isoform 1 of N-acetylgalactosamine 4-sulfate 6-O-sulfotransferase	R.STFDALRK.A	2	2.53	0.19	-3.48
IPI00183321	Isoform 1 of N-acetylgalactosamine 4-sulfate 6-O-sulfotransferase	R.YPVEDYLDLFDLAAHQIHQGLQASSAK.E	4	3.59	0.14	-3.29
IPI00183445	Isoform 1 of Latrophilin-1 precursor	K.LM*EQLLDILDAQLQALRPIER.E	3	2.99	0.11	-4.10
IPI00183445	Isoform 1 of Latrophilin-1 precursor	K.SGETVINTANYHDTSPYR.W	2	5.31	0.62	-3.33

IPI00183445	Isoform 1 of Latrophilin-1 precursor	K.VFVCPGTLQK.V	2	2.78	0.28	-2.33
IPI00183445	Isoform 1 of Latrophilin-1 precursor	K.YLEVQYDCVPYK.V	2	4.09	0.36	-3.40
IPI00183445	Isoform 1 of Latrophilin-1 precursor	K.YLEVQYDCVPYKVEQK.V	3	3.49	0.22	-3.08
IPI00183445	Isoform 1 of Latrophilin-1 precursor	R.AGLPFGLM*R.R	2	2.48	0.17	-1.32
IPI00183445	Isoform 1 of Latrophilin-1 precursor	R.CPGSDVIM*VENANYGR.T	2	4.08	0.33	-5.50
IPI00183445	Isoform 1 of Latrophilin-1 precursor	R.EEPVSLTFPNPYQFISSVDYNPR.D	3	4.39	0.46	-4.12
IPI00183445	Isoform 1 of Latrophilin-1 precursor	R.ELACEGYPIELR.C	1	1.78	0.35	-3.29
IPI00183445	Isoform 1 of Latrophilin-1 precursor	R.ELACEGYPIELR.C	2	3.96	0.38	-4.14
IPI00183445	Isoform 1 of Latrophilin-1 precursor	R.IKSGETVINTANYHDTSPYR.W	2	5.95	0.60	-1.83
IPI00183445	Isoform 1 of Latrophilin-1 precursor	R.IKSGETVINTANYHDTSPYR.W	3	4.23	0.45	-0.15
IPI00183445	Isoform 1 of Latrophilin-1 precursor	R.IYVM*PWIPYR.T	2	2.78	0.19	-2.17
IPI00183445	Isoform 1 of Latrophilin-1 precursor	R.LPNRVDGTGFVVYDGAVFYNKER.T	4	3.21	0.20	-3.35
IPI00183445	Isoform 1 of Latrophilin-1 precursor	R.LVVSQLNPYTLR.F	1	2.49	0.20	-2.41
IPI00183445	Isoform 1 of Latrophilin-1 precursor	R.LVVSQLNPYTLR.F	2	4.31	0.40	-3.40
IPI00183445	Isoform 1 of Latrophilin-1 precursor	R.SVYVDDDSEAAGNR.V	2	3.58	0.54	-3.78
IPI00183445	Isoform 1 of Latrophilin-1 precursor	R.SVYVDDDSEAAGNRVDYAFNTNANR.E	3	3.69	0.45	-3.03
IPI00183445	Isoform 1 of Latrophilin-1 precursor	R.TDDKICDADPFQM*ENVQCYLPDAFK.I	3	5.37	0.57	-3.51
IPI00183445	Isoform 1 of Latrophilin-1 precursor	R.TDTLTEYASWEDYVAAR.H	2	5.22	0.58	-4.64
IPI00183445	Isoform 1 of Latrophilin-1 precursor	R.TQCVVVAGSDAFPDPCPGTYK.Y	2	5.27	0.57	-3.64
IPI00183445	Isoform 1 of Latrophilin-1 precursor	R.TQCVVVAGSDAFPDPCPGTYK.Y	3	2.89	0.19	-3.30
IPI00183445	Isoform 1 of Latrophilin-1 precursor	R.VDYAFNTNANREEPVSLTFPNPYQFISSVDYNPR.D	3	4.06	0.43	-1.56
IPI00183487	Xylosyltransferase 1	K.FLVAPLTFSNR.Q	2	3.60	0.32	-2.54
IPI00183487	Xylosyltransferase 1	L.VVWNFSSLDSGAGER.R	2	4.06	0.43	-3.97
IPI00183487	Xylosyltransferase 1	N.FSSLDSGAGER.R	2	3.08	0.24	-3.34
IPI00183487	Xylosyltransferase 1	R.DLPAEPAAAR.G	2	1.87	0.12	-2.42
IPI00183487	Xylosyltransferase 1	R.FQQTARPTFFAR.K	2	2.41	0.22	-0.84
IPI00183487	Xylosyltransferase 1	R.GGAAVGGGEQPPPAPAPR.R	2	5.08	0.58	-4.56
IPI00183487	Xylosyltransferase 1	R.NAYM*EQSFQSLNPVLSLPINPAQVEQAR.R	3	4.88	0.32	-4.33
IPI00183487	Xylosyltransferase 1	R.RGGAAVGGGEQPPPAPAPR.R	2	5.81	0.62	-7.49
IPI00183487	Xylosyltransferase 1	R.RGGAAVGGGEQPPPAPAPR.R	3	4.10	0.39	-3.70
IPI00183487	Xylosyltransferase 1	R.TNDQLVAFLSR.Y	2	3.97	0.34	-3.74
IPI00184019	Isoform 3 of Paired immunoglobulin-like type 2 receptor alpha precursor	G.SGPSYLYGVTQPK.H	2	3.52	0.25	-1.48
IPI00184019	Isoform 3 of Paired immunoglobulin-like type 2 receptor alpha precursor	K.LSITQGQQR.T	2	2.95	0.16	-0.10
IPI00184019	Isoform 3 of Paired immunoglobulin-like type 2 receptor alpha precursor	R.QQWQSIEGTK.L	2	1.78	0.19	-2.17
IPI00184094	UDP-GlcNAc:betaGal beta-1,3-N-acetylglucosaminyltransferase 8	R.AQDDAFVHTPALLAHLR.A	3	3.33	0.34	-2.13
IPI00184094	UDP-GlcNAc:betaGal beta-1,3-N-acetylglucosaminyltransferase 8	R.AQDDAFVHTPALLAHLR.A	4	2.43	0.26	-2.09

IPI00184094	UDP-GlcNAc:betaGal beta-1,3-N-acetylglucosaminyltransferase 8	R.NLLLVRPLGPQASIR.L	3	2.62	0.27	-3.12
IPI00184094	UDP-GlcNAc:betaGal beta-1,3-N-acetylglucosaminyltransferase 8	W.GAAAATEIPDFASYPK.D	2	2.99	0.30	-2.49
IPI00184650	Class B basic helix-loop-helix protein 4	R.SLRLSINARERR.R	3	2.28	0.18	
IPI00184851	Type 2 lactosamine alpha-2,3-sialyltransferase	K.CVVVGNGGVLKNKTLGEK.I	2	2.32	0.11	
IPI00184851	Type 2 lactosamine alpha-2,3-sialyltransferase	K.IASLYGSDKFDLPYGM*R.T	3	3.39	0.30	-1.32
IPI00184851	Type 2 lactosamine alpha-2,3-sialyltransferase	R.ILDPFIIR.T	2	2.64	0.16	-1.72
IPI00184884	Non-structural maintenance of chromosomes element 1 homolog	K.VFDPEKERESGVLKSNK.K	2	2.17	0.10	-7.39
IPI00184997	cDNA FLJ78771, highly similar to Homo sapiens discs, large homolog 7 (Drosophila), mRNA	K.DISTEMIRTKIAHRKSLSQKENR.H	3	3.56	0.09	
IPI00185088	immunoglobulin superfamily, member 11 isoform b	S.LEVSESPGSIQVAR.G	2	4.75	0.39	-1.82
IPI00185146	Importin-9	R.VLTEFTREVTDTQM*PLVAPVILPEMYK.I	3	2.91	0.14	
IPI00185661	Ubiquitin carboxyl-terminal hydrolase 32	K.KMADTSSMDEDFESDYKK.Y	3	2.19	0.18	-2.49
IPI00186004	hypothetical protein LOC57730	R.QRLETEM*QSYRCRLNAARCDHDQSHSSKR.D	3	3.31	0.05	
IPI00186581	amplified in osteosarcoma isoform 2 precursor	K.AGM*ERELENIIQETEK.E	2	1.90	0.06	-3.01
IPI00186621	Orofacial clefting chromosomal breakpoint region 1	K.GNEYGRNYFDPLMDEEINPRQCATEVSR.E	3	3.52	0.05	
IPI00186826	Ephrin receptor	K.VDTVAAEHLTR.K	3	2.07	0.30	-0.84
IPI00186903	Isoform 2 of Apolipoprotein-L1 precursor	K.ILQADQEL.-	2	2.63	0.05	
IPI00186903	Isoform 2 of Apolipoprotein-L1 precursor	K.LNILNNNYK.I	2	2.84	0.09	
IPI00186903	Isoform 2 of Apolipoprotein-L1 precursor	K.VAQELEEKLNILNNNYK.I	2	5.74	0.34	
IPI00186903	Isoform 2 of Apolipoprotein-L1 precursor	R.VTEPISAESGEQVER.V	2	4.23	0.42	
IPI00186903	Isoform 2 of Apolipoprotein-L1 precursor	R.VTEPISAESGEQVERVNEPSILEM*SR.G	3	4.86	0.34	
IPI00186966	Isoform IIA of Myc box-dependent-interacting protein 1	K.VQAQHDYTATDTDELQLK.A	3	3.62	0.40	-2.77
IPI00187143	Isoform 2 of Ras-related protein Rab-4B	R.FAQENELM*FLETSALTGENVEEAFLK.C	3	3.78	0.12	
IPI00215610	55 kDa erythrocyte membrane protein	R.NISANEFLEFGSYQGNMFGTK.F	2	5.25	0.57	-3.18
IPI00215746	Fatty acid-binding protein, adipocyte	K.LVSSENFDDYM*KEVGVGFATR.K	3	3.41	0.31	-1.65
IPI00215767	Isoform Long of Beta-1,4-galactosyltransferase 1	K.FGFSLPYVQYFGGVSALSK.Q	2	3.77	0.45	-1.61
IPI00215767	Isoform Long of Beta-1,4-galactosyltransferase 1	Q.LVGVSTPLQGGSNSAAAIGQSSGELR.T	3	3.58	0.26	-2.09
IPI00215767	Isoform Long of Beta-1,4-galactosyltransferase 1	R.QQLDYGIYVINQAGDTIFNR.A	3	4.72	0.33	-5.82
IPI00215767	Isoform Long of Beta-1,4-galactosyltransferase 1	W.GGEDDDIFNR.I	2	3.47	0.35	-2.24
IPI00215777	Isoform B of Phosphate carrier protein, mitochondrial precursor	K.YYALCGFGGVLSCGLTHTAVVPLDLVK.C	3	2.41	0.10	-2.78
IPI00215894	Isoform LMW of Kininogen-1 precursor	C.VHPISTQSPDLEPILR.H	3	3.83	0.27	0.40
IPI00215894	Isoform LMW of Kininogen-1 precursor	D.IPTNSPELEETLTHTITK.L	3	4.68	0.54	-3.17
IPI00215894	Isoform LMW of Kininogen-1 precursor	I.GEIKEETTSHLR.S	2	3.10	0.30	-0.91
IPI00215894	Isoform LMW of Kininogen-1 precursor	I.GEIKEETTSHLR.S	3	3.87	0.42	-3.02
IPI00215894	Isoform LMW of Kininogen-1 precursor	I.PTNSPELEETLTHTITK.L	2	5.22	0.57	-3.52
IPI00215894	Isoform LMW of Kininogen-1 precursor	I.PTNSPELEETLTHTITK.L	3	4.29	0.40	-3.93

IPI00215894	Isoform LMW of Kininogen-1 precursor	K.AATGECTATVGK.R	2	3.81	0.46	-4.72
IPI00215894	Isoform LMW of Kininogen-1 precursor	K.AATGECTATVGKR.S	2	3.43	0.38	-4.18
IPI00215894	Isoform LMW of Kininogen-1 precursor	K.AGAEPASEREVS.-	1	1.82	0.29	-3.65
IPI00215894	Isoform LMW of Kininogen-1 precursor	K.AGAEPASEREVS.-	2	2.91	0.35	-3.10
IPI00215894	Isoform LMW of Kininogen-1 precursor	K.AVDAALKK.Y	2	2.72	0.20	-4.79
IPI00215894	Isoform LMW of Kininogen-1 precursor	K.DFVQPPTK.I	1	1.67	0.06	-2.56
IPI00215894	Isoform LMW of Kininogen-1 precursor	K.DFVQPPTK.I	2	2.48	0.15	-4.19
IPI00215894	Isoform LMW of Kininogen-1 precursor	K.EETTSHLR.S	1	1.86	0.21	-2.16
IPI00215894	Isoform LMW of Kininogen-1 precursor	K.ENFLFLTPDCK.S	1	3.27	0.29	-3.78
IPI00215894	Isoform LMW of Kininogen-1 precursor	K.ENFLFLTPDCK.S	2	4.01	0.31	-3.73
IPI00215894	Isoform LMW of Kininogen-1 precursor	K.FSVATQTCQITPAEGPVVTAQYDCLGCVHPISTQSPDLEPILR.H	3	4.34	0.53	-3.40
IPI00215894	Isoform LMW of Kininogen-1 precursor	K.FSVATQTCQITPAEGPVVTAQYDCLGCVHPISTQSPDLEPILR.H	4	5.64	0.52	-2.18
IPI00215894	Isoform LMW of Kininogen-1 precursor	K.GRPPKAGAEPASER.E	3	2.65	0.24	-2.15
IPI00215894	Isoform LMW of Kininogen-1 precursor	K.GRPPKAGAEPASEREVS.-	2	2.17	0.35	-5.18
IPI00215894	Isoform LMW of Kininogen-1 precursor	K.IYPTVNCQPLGM*ISLM*K.R	2	5.10	0.49	-3.81
IPI00215894	Isoform LMW of Kininogen-1 precursor	K.IYPTVNCQPLGM*ISLM*K.R	3	3.71	0.45	-6.75
IPI00215894	Isoform LMW of Kininogen-1 precursor	K.IYPTVNCQPLGMISLM*K.R	2	5.46	0.17	
IPI00215894	Isoform LMW of Kininogen-1 precursor	K.IYPTVNCQPLGMISLMK.R	2	3.48	0.29	
IPI00215894	Isoform LMW of Kininogen-1 precursor	K.KIYPTVNCQPLGM*ISLM*.K	2	4.17	0.47	-2.21
IPI00215894	Isoform LMW of Kininogen-1 precursor	K.KIYPTVNCQPLGM*ISLM*K.R	2	3.85	0.42	-4.40
IPI00215894	Isoform LMW of Kininogen-1 precursor	K.KIYPTVNCQPLGM*ISLM*K.R	3	4.31	0.31	-5.44
IPI00215894	Isoform LMW of Kininogen-1 precursor	K.KLGQSLDCNAEVYVVPWEK.K	2	4.84	0.35	
IPI00215894	Isoform LMW of Kininogen-1 precursor	K.KLGQSLDCNAEVYVVPWEKK.I	3	4.40	0.28	
IPI00215894	Isoform LMW of Kininogen-1 precursor	K.KYFIDFVAR.E	1	2.82	0.27	-3.27
IPI00215894	Isoform LMW of Kininogen-1 precursor	K.KYFIDFVAR.E	2	3.13	0.35	-3.25
IPI00215894	Isoform LMW of Kininogen-1 precursor	K.KYNSQNQSNNQFVLYR.I	2	5.50	0.47	-4.39
IPI00215894	Isoform LMW of Kininogen-1 precursor	K.KYNSQNQSNNQFVLYR.I	3	4.59	0.43	-2.89
IPI00215894	Isoform LMW of Kininogen-1 precursor	K.LGQSLDCNAEVYVVPWEK.K	2	5.04	0.47	-5.49
IPI00215894	Isoform LMW of Kininogen-1 precursor	K.LGQSLDCNAEVYVVPWEKK.I	3	4.20	0.37	-1.56
IPI00215894	Isoform LMW of Kininogen-1 precursor	K.RPPGFSPFR.S	1	1.59	0.30	-5.39
IPI00215894	Isoform LMW of Kininogen-1 precursor	K.RPPGFSPFR.S	2	2.67	0.22	-3.42
IPI00215894	Isoform LMW of Kininogen-1 precursor	K.SLWNGDTGECTDNAYIDIQLR.I	2	5.24	0.56	-4.45
IPI00215894	Isoform LMW of Kininogen-1 precursor	K.SLWNGDTGECTDNAYIDIQLR.I	3	3.79	0.06	
IPI00215894	Isoform LMW of Kininogen-1 precursor	K.TVGSDTFYSFK.Y	1	2.61	0.30	-1.83
IPI00215894	Isoform LMW of Kininogen-1 precursor	K.TVGSDTFYSFK.Y	2	3.86	0.46	-1.80
IPI00215894	Isoform LMW of Kininogen-1 precursor	K.TVGSDTFYSFKYEIK.E	2	4.11	0.39	-4.98
IPI00215894	Isoform LMW of Kininogen-1 precursor	K.TWQDCEYK.D	2	2.03	0.11	-2.56
IPI00215894	Isoform LMW of Kininogen-1 precursor	K.TWQDCEYKDAAK.A	2	3.78	0.23	
IPI00215894	Isoform LMW of Kininogen-1 precursor	K.YEIKEGDCPVQSGK.T	2	4.78	0.36	
IPI00215894	Isoform LMW of Kininogen-1 precursor	K.YFIDFVAR.E	1	2.65	0.33	-4.15
IPI00215894	Isoform LMW of Kininogen-1 precursor	K.YFIDFVAR.E	2	3.44	0.35	-3.76
IPI00215894	Isoform LMW of Kininogen-1 precursor	K.YNSQNQSNNQFVLYR.I	2	4.61	0.38	-3.21

IPI00215894	Isoform LMW of Kininogen-1 precursor	K.YNSQNQSNNQFVLYR.I	3	5.37	0.47	-3.89
IPI00215894	Isoform LMW of Kininogen-1 precursor	R.DIPTNSPELEETLTHTITK.L	2	4.59	0.51	-4.42
IPI00215894	Isoform LMW of Kininogen-1 precursor	R.IASFSQNCDIYPGKDFVQPPTK.I	2	4.15	0.55	-2.76
IPI00215894	Isoform LMW of Kininogen-1 precursor	R.IASFSQNCDIYPGKDFVQPPTK.I	3	4.84	0.54	-4.44
IPI00215894	Isoform LMW of Kininogen-1 precursor	R.IASFSQNCDIYPGKDFVQPPTK.I	4	3.08	0.17	-3.36
IPI00215894	Isoform LMW of Kininogen-1 precursor	R.IGEIKEETTSHLR.S	1	3.59	0.38	-1.49
IPI00215894	Isoform LMW of Kininogen-1 precursor	R.IGEIKEETTSHLR.S	2	4.13	0.39	-6.10
IPI00215894	Isoform LMW of Kininogen-1 precursor	R.IGEIKEETTSHLR.S	3	5.01	0.42	-3.12
IPI00215894	Isoform LMW of Kininogen-1 precursor	R.QVVAGLNFR.I	1	1.97	0.18	-1.97
IPI00215894	Isoform LMW of Kininogen-1 precursor	R.QVVAGLNFR.I	2	2.47	0.33	-3.28
IPI00215894	Isoform LMW of Kininogen-1 precursor	R.VQVVAGK.K	1	2.43	0.10	-3.23
IPI00215899	Isoform 2 of Sushi repeat-containing protein SRPX precursor	R.DTADGILTDVILK.G	2	4.45	0.36	-4.61
IPI00215914	ADP-ribosylation factor 1	R.MLAEDELRDAVLLVFANK.Q	2	2.94	0.39	-3.07
IPI00215914	ADP-ribosylation factor 1	R.MLAEDELRDAVLLVFANK.Q	3	4.19	0.33	-1.90
IPI00215979	Bisphosphoglycerate mutase	R.AVGPHQFLGDQEAIQAAIK.K	3	2.63	0.13	-1.87
IPI00215980	Isoform Alpha of Poliovirus receptor-related protein 2 precursor	R.FTLVPSGR.A	2	2.27	0.12	-2.66
IPI00215980	Isoform Alpha of Poliovirus receptor-related protein 2 precursor	R.TDATLSCDVR.S	2	3.24	0.21	-3.61
IPI00215983	Carbonic anhydrase 1	K.ADGLAVIGVLM*K.V	2	3.09	0.34	-3.20
IPI00215983	Carbonic anhydrase 1	K.EIINVGHSFHVNFEDNDNR.S	2	4.28	0.39	-4.05
IPI00215983	Carbonic anhydrase 1	K.EIINVGHSFHVNFEDNDNR.S	3	2.74	0.27	-4.54
IPI00215983	Carbonic anhydrase 1	K.ESISVSSEQLAQFR.S	2	3.33	0.32	-4.54
IPI00215983	Carbonic anhydrase 1	K.GGPFSDSYR.L	2	2.37	0.15	-0.92
IPI00215983	Carbonic anhydrase 1	K.LYPIANGNNQSPVDIK.T	2	4.20	0.33	-4.55
IPI00215983	Carbonic anhydrase 1	K.TSETKHDTSLKPISVSYNPATAK.E	2	5.19	0.50	-3.58
IPI00215983	Carbonic anhydrase 1	K.TSETKHDTSLKPISVSYNPATAK.E	3	4.53	0.46	-3.54
IPI00215983	Carbonic anhydrase 1	K.VLDALQAIK.T	2	3.86	0.26	-1.76
IPI00215983	Carbonic anhydrase 1	K.YSSLAEAASK.A	1	2.57	0.32	-1.98
IPI00215983	Carbonic anhydrase 1	R.SLLSNVEGDNAVPM*QHNNRPTQPLK.G	3	3.99	0.35	-3.39
IPI00215997	CD9 antigen	K.KDVLETFTVK.S	2	2.90	0.18	-2.72
IPI00215997	CD9 antigen	K.TKDEPQRETLK.A	2	3.26	0.13	-3.50
IPI00216049	Isoform 1 of Heterogeneous nuclear ribonucleoprotein K	K.LFQECCPHSTDRVVLIGGKPDR.V	3	2.25	0.19	-6.53
IPI00216106	Isoform 3 of Obg-like ATPase 1	K.IPAFLNVVDIAGLVK.G	2	4.70	0.35	-4.39
IPI00216138	Transgelin	K.IEKKYDEELEER.L	3	4.10	0.36	-2.52
IPI00216138	Transgelin	K.LVNSLYPDGSKPVKVPENPPSM*VFK.Q	3	4.75	0.47	-3.95
IPI00216138	Transgelin	K.QM*EQVAQFLK.A	2	2.64	0.11	-4.04
IPI00216138	Transgelin	R.EFTESQLQEGK.H	2	2.62	0.19	-2.26
IPI00216138	Transgelin	R.GASQAGM*TGYGR.P	2	3.10	0.25	-3.14
IPI00216171	Gamma-enolase	K.ACNCLLLK.V	2	2.34	0.07	-0.79

IPI00216171	Gamma-enolase	K.AVDHINSTIAPALISSGLSVVEQEKLDNLM*LELDGTENK.S	4	6.14	0.49	-5.34
IPI00216171	Gamma-enolase	K.DATNVGDEGGFAPNILENSEALELVK.E	2	5.19	0.52	-1.08
IPI00216171	Gamma-enolase	K.DATNVGDEGGFAPNILENSEALELVK.E	3	3.99	0.40	-2.48
IPI00216171	Gamma-enolase	K.DATNVGDEGGFAPNILENSEALELVKEAIDKAGYTEK.I	4	3.49	0.31	-4.15
IPI00216171	Gamma-enolase	K.DKYGKDATNVGDEGGFAPNILENSEALELVK.E	3	3.69	0.30	-0.69
IPI00216171	Gamma-enolase	K.EAIDKAGYTEK.I	2	1.95	0.17	-4.10
IPI00216171	Gamma-enolase	K.FTANVGIQIVGDDLTVTNPK.R	2	4.76	0.50	-4.69
IPI00216171	Gamma-enolase	K.FTANVGIQIVGDDLTVTNPK.R	3	3.80	0.37	-3.84
IPI00216171	Gamma-enolase	K.IVIGM*DVAASEFYR.D	2	4.12	0.46	-4.55
IPI00216171	Gamma-enolase	K.LAM*QEFM*ILPVGAESFR.D	3	3.67	0.33	-2.83
IPI00216171	Gamma-enolase	K.LAQENGWGVM*VSHR.S	2	2.73	0.33	-1.76
IPI00216171	Gamma-enolase	K.VNQIGSVTEAIQACK.L	2	3.77	0.45	-2.39
IPI00216171	Gamma-enolase	K.YGKDATNVGDEGGFAPNILENSEALELVK.E	3	5.81	0.56	-2.59
IPI00216171	Gamma-enolase	K.YNQLM*R.I	2	2.01	0.11	-2.49
IPI00216171	Gamma-enolase	R.AAVPSGASTGIYEALELR.D	2	5.65	0.33	-8.46
IPI00216171	Gamma-enolase	R.AAVPSGASTGIYEALELR.D	3	4.37	0.39	-3.26
IPI00216171	Gamma-enolase	R.DGKYDLDFK.S	2	2.31	0.19	-1.26
IPI00216171	Gamma-enolase	R.DGKYDLDFKSPTDPSR.Y	3	3.02	0.36	-2.07
IPI00216171	Gamma-enolase	R.FAGHNFR.N	2	1.79	0.05	-3.59
IPI00216171	Gamma-enolase	R.GNPTVEVDLYTAK.G	1	2.42	0.11	-3.08
IPI00216171	Gamma-enolase	R.GNPTVEVDLYTAK.G	2	3.65	0.23	-3.79
IPI00216171	Gamma-enolase	R.HIAQLAGNSDLILPVPAFNVINGGSHAGNK.L	3	4.96	0.38	-6.02
IPI00216171	Gamma-enolase	R.IEEELGDEAR.F	2	3.97	0.12	-1.74
IPI00216171	Gamma-enolase	R.LGAEVYHTLK.G	2	3.51	0.26	-3.28
IPI00216171	Gamma-enolase	R.SGETEDTFIADLVVGLCTGQIK.T	2	5.97	0.60	-5.74
IPI00216171	Gamma-enolase	R.SGETEDTFIADLVVGLCTGQIK.T	3	4.50	0.40	-3.18
IPI00216171	Gamma-enolase	R.YITGDQLGALYQDFVR.D	2	6.01	0.55	-3.48
IPI00216171	Gamma-enolase	R.YITGDQLGALYQDFVR.D	3	5.86	0.28	-2.93
IPI00216171	Gamma-enolase	T.IAPALISSGLSVVEQEKLDNLMLELDGTENK.S	3	5.44	0.45	-2.67
IPI00216288	Isoform 3 of Lethal	R.VGDRAGVTVLKTAGSRCPPQR.H	3	2.48	0.20	
IPI00216298	Thioredoxin	K.CM*PTFQFFK.K	2	2.29	0.22	-1.07
IPI00216298	Thioredoxin	K.EKLEATINELV.-	2	3.52	0.36	-2.62
IPI00216298	Thioredoxin	K.LEATINELV.-	2	2.49	0.06	-2.49
IPI00216298	Thioredoxin	K.M*IKPFFHSLSEK.Y	2	2.70	0.15	-3.33
IPI00216298	Thioredoxin	K.TAFQEALDAAGDK.L	2	4.13	0.31	-3.26
IPI00216298	Thioredoxin	K.VGEFSGANK.E	1	2.29	0.25	-2.85
IPI00216298	Thioredoxin	K.VGEFSGANKEK.L	2	2.71	0.19	-3.61
IPI00216318	Isoform Long of 14-3-3 protein beta/alpha	K.AVTEQGHELSNEER.N	2	4.58	0.48	-2.63
IPI00216318	Isoform Long of 14-3-3 protein beta/alpha	K.AVTEQGHELSNEER.N	3	2.78	0.36	-2.03
IPI00216318	Isoform Long of 14-3-3 protein beta/alpha	K.IEAELQDICNDVLELLDKYLIPNATQPESK.V	3	3.87	0.27	-3.54
IPI00216318	Isoform Long of 14-3-3 protein beta/alpha	K.TAFDEAIAELDTLNEESYKDSTLIM*QLLR.D	3	5.69	0.50	-4.96
IPI00216318	Isoform Long of 14-3-3 protein beta/alpha	K.TAFDEAIAELDTLNEESYKDSTLIM*QLLR.D	4	5.37	0.43	-4.47

IPI00216318	Isoform Long of 14-3-3 protein beta/alpha	K.TAFDEAIAELDTLNEESYKDSTLIMQLLR.D	3	2.32	0.14	-1.58
IPI00216318	Isoform Long of 14-3-3 protein beta/alpha	R.DNLTLWTSENQGDEGDAGEGEN.-	2	3.71	0.56	-5.42
IPI00216318	Isoform Long of 14-3-3 protein beta/alpha	R.EKIEAELQDICNDVLELLDK.Y	3	5.24	0.37	-2.90
IPI00216318	Isoform Long of 14-3-3 protein beta/alpha	R.LGLALNFSVFYYEILNSPEK.A	2	4.16	0.55	-2.27
IPI00216318	Isoform Long of 14-3-3 protein beta/alpha	R.NLLSVAYK.N	1	1.68	0.08	-2.46
IPI00216318	Isoform Long of 14-3-3 protein beta/alpha	R.NLLSVAYK.N	2	2.28	0.09	-1.47
IPI00216318	Isoform Long of 14-3-3 protein beta/alpha	R.YDDM*AAAM*K.A	2	3.02	0.26	-2.31
IPI00216318	Isoform Long of 14-3-3 protein beta/alpha	R.YLSEVASGDNK.Q	2	3.19	0.23	-3.83
IPI00216318	Isoform Long of 14-3-3 protein beta/alpha	R.YLSEVASGDNKQTTVSNSQQAYQEAFEISKK.E	4	3.28	0.11	-2.05
IPI00216319	14-3-3 protein eta	K.AVTELNEPLSNEDRNLLSVAYK.N	3	3.30	0.10	-3.04
IPI00216319	14-3-3 protein eta	K.M*KGDYYR.Y	2	2.25	0.17	-2.86
IPI00216319	14-3-3 protein eta	K.QAFDDAIAELDTLNEDSYKDSTLIM*QLLR.D	3	4.41	0.42	-4.45
IPI00216319	14-3-3 protein eta	R.DNLTLWTSDQQDEEAGEGN.-	2	4.24	0.43	-3.86
IPI00216319	14-3-3 protein eta	R.NLLSVAYK.N	1	1.68	0.08	-2.46
IPI00216319	14-3-3 protein eta	R.NLLSVAYK.N	2	2.28	0.09	-1.47
IPI00216319	14-3-3 protein eta	R.YDDM*ASAM*K.A	2	3.30	0.37	-2.32
IPI00216348	Isoform 2C of Cytoplasmic dynein 1 intermediate chain 2	K.SVSTPSEAGSQDSGDGAVGSR.R	2	4.19	0.50	-2.81
IPI00216348	Isoform 2C of Cytoplasmic dynein 1 intermediate chain 2	R.LAQIREEK.K	2	2.09	0.09	-2.32
IPI00216457	Histone H2A type 2-A	R.AGLQFPVGR.I	2	2.49	0.10	-2.97
IPI00216457	Histone H2A type 2-A	R.HLQLAIRNDEELNKLLGKVTIAQGGVLPNIQAVLLPK.K	4	3.02	0.27	-2.89
IPI00216457	Histone H2A type 2-A	R.HLQLAIRNDEELNKLLGKVTIAQGGVLPNIQAVLLPK.K	5	2.23	0.19	-3.80
IPI00216457	Histone H2A type 2-A	R.VGAGAPVYMAAVLEYLTAEILELAGNAARDNKK.T	4	5.34	0.49	-4.54
IPI00216457	Histone H2A type 2-A	R.VGAGAPVYMAAVLEYLTAEILELAGNAARDNKK.T	5	3.60	0.15	-4.19
IPI00216457	Histone H2A type 2-A	R.VTIAQGGVLPNIQAVLLPK.K	2	4.34	0.48	-3.79
IPI00216461	Acylphosphatase-2	R.M*YTEDEAR.K	2	2.12	0.17	-0.63
IPI00216461	Acylphosphatase-2	R.M*YTEDEARK.I	2	1.88	0.07	-0.83
IPI00216470	Isoform 1 of Phosphatidylinositol-5-phosphate 4-kinase type-2 beta	-.MSSNCTSTTAVAVAPLSASKTKTK.K	3	2.77	0.18	
IPI00216508	Isoform 2 of Sorting nexin-3	K.VAGHPLAQNER.C	2	2.50	0.36	-1.72
IPI00216572	BarH-like homeobox 2	K.WKKMVLKGGQEAPTKPK.G	2	2.71	0.05	-8.59
IPI00216592	Isoform C1 of Heterogeneous nuclear ribonucleoproteins C1/C2	K.GFAFVQYVNER.N	2	1.70	0.06	-2.77
IPI00216602	Isoform 5 of Fibroblast growth factor receptor 2 precursor	K.DAAVISWTK.D	1	2.19	0.18	-1.51
IPI00216602	Isoform 5 of Fibroblast growth factor receptor 2 precursor	K.DAAVISWTK.D	2	3.28	0.24	-0.79
IPI00216602	Isoform 5 of Fibroblast growth factor receptor 2 precursor	R.CLLKDAAVISWTK.D	2	3.61	0.32	-3.62
IPI00216602	Isoform 5 of Fibroblast growth factor receptor 2 precursor	R.CPAGGNPM*PTM*R.W	2	2.89	0.28	-2.76

IPI00216602	Isoform 5 of Fibroblast growth factor receptor 2 precursor	R.DSGLYACTASR.T	1	2.65	0.39	-2.66
IPI00216602	Isoform 5 of Fibroblast growth factor receptor 2 precursor	R.DSGLYACTASR.T	2	3.92	0.46	-1.83
IPI00216602	Isoform 5 of Fibroblast growth factor receptor 2 precursor	R.LHAVPAANTVK.F	1	2.52	0.25	-3.72
IPI00216602	Isoform 5 of Fibroblast growth factor receptor 2 precursor	R.TVLIGEYLQIK.G	1	2.72	0.20	-4.20
IPI00216602	Isoform 5 of Fibroblast growth factor receptor 2 precursor	R.TVLIGEYLQIK.G	2	3.72	0.31	-5.86
IPI00216651	Isoform 1 of Interleukin-28 receptor alpha chain precursor	K.YEVAFWK.E	2	1.13	0.07	2.44
IPI00216683	M-phase inducer phosphatase 3	K.FLGDSANLSILSGGTPKR.C	2	2.16	0.16	
IPI00216691	Profilin-1	K.DSPSVWAAVPGK.T	2	2.53	0.18	-3.33
IPI00216691	Profilin-1	K.STGGAPTFNVTVTK.T	2	3.53	0.35	-2.61
IPI00216691	Profilin-1	K.TDKTLVLLM*GK.E	2	3.28	0.33	-2.72
IPI00216691	Profilin-1	K.TDKTLVLLM*GK.E	3	3.41	0.14	-3.30
IPI00216691	Profilin-1	K.TFVNITPAEVGVLVGK.D	2	2.48	0.17	-3.95
IPI00216691	Profilin-1	K.TFVNITPAEVGVLVGKDR.S	2	4.24	0.36	-2.80
IPI00216691	Profilin-1	K.TLVLLM*GK.E	2	2.88	0.26	-2.56
IPI00216691	Profilin-1	R.DSLLQDGEFSM*DLR.T	3	3.41	0.20	-3.05
IPI00216691	Profilin-1	R.DSLLQDGEFSMDLR.T	2	4.91	0.48	-3.71
IPI00216691	Profilin-1	R.TKSTGGAPTFNVTVTK.T	3	3.05	0.34	1.76
IPI00216694	plastin 3	K.M*INLSVPDTIDER.T	2	3.99	0.32	-2.96
IPI00216694	plastin 3	R.AESM*LQQADK.L	2	2.38	0.06	-2.25
IPI00216697	Isoform Er1 of Ankyrin-1	K.FLLENGANQNVATEDGFTPLAVALQQGHENVVAHLINYGTK.G	4	3.30	0.09	-5.18
IPI00216697	Isoform Er1 of Ankyrin-1	K.LVYANECANFTTNVSAR.F	2	2.94	0.20	-5.01
IPI00216697	Isoform Er1 of Ankyrin-1	K.MAVISEHLGLSWAELAR.E	2	4.56	0.53	-7.68
IPI00216697	Isoform Er1 of Ankyrin-1	K.MAVISEHLGLSWAELAR.E	3	5.74	0.48	-5.66
IPI00216697	Isoform Er1 of Ankyrin-1	K.TGASIDAVTESGLTPLHVASFMGHLPIVK.N	3	4.08	0.40	-3.47
IPI00216697	Isoform Er1 of Ankyrin-1	K.TGASIDAVTESGLTPLHVASFMGHLPIVK.N	4	4.55	0.43	-3.53
IPI00216697	Isoform Er1 of Ankyrin-1	K.TGFTPLHIAAHYENLNVAQLLLNR.G	2	5.27	0.55	-3.28
IPI00216697	Isoform Er1 of Ankyrin-1	K.TGFTPLHIAAHYENLNVAQLLLNR.G	3	7.07	0.54	-4.83
IPI00216697	Isoform Er1 of Ankyrin-1	K.TGFTPLHIAAHYENLNVAQLLLNR.G	4	4.24	0.32	-7.72
IPI00216697	Isoform Er1 of Ankyrin-1	K.VVTDETSFVLVSDK.H	2	4.30	0.45	-1.71
IPI00216697	Isoform Er1 of Ankyrin-1	R.DIEVLEGM*SLFAELSGNLVPVK.K	2	4.13	0.47	-5.42
IPI00216697	Isoform Er1 of Ankyrin-1	R.DIEVLEGM*SLFAELSGNLVPVK.K	3	2.40	0.11	-4.66
IPI00216697	Isoform Er1 of Ankyrin-1	R.DIEVLEGM*SLFAELSGNLVPVKK.A	3	3.27	0.27	-5.55
IPI00216697	Isoform Er1 of Ankyrin-1	R.DIEVLEGMSLFAELSGNLVPVK.K	2	5.21	0.55	-5.79
IPI00216697	Isoform Er1 of Ankyrin-1	R.DIEVLEGMSLFAELSGNLVPVK.K	3	4.75	0.42	-4.79
IPI00216697	Isoform Er1 of Ankyrin-1	R.DIEVLEGMSLFAELSGNLVPVKK.A	2	4.13	0.50	-4.53
IPI00216697	Isoform Er1 of Ankyrin-1	R.DIEVLEGMSLFAELSGNLVPVKK.A	3	4.09	0.40	-6.18

IPI00216697	Isoform Er1 of Ankyrin-1	R.ELVNYGANVNAQSQK.G	2	3.61	0.32	-2.04
IPI00216697	Isoform Er1 of Ankyrin-1	R.IIALGPTGAQFLSPVIVEIPHFASHGR.G	4	4.25	0.38	-2.85
IPI00216697	Isoform Er1 of Ankyrin-1	R.LLCSVIGGTDQAQWEDITGTTK.L	2	6.06	0.61	-5.64
IPI00216697	Isoform Er1 of Ankyrin-1	R.LLCSVIGGTDQAQWEDITGTTK.L	3	2.90	0.26	-3.87
IPI00216697	Isoform Er1 of Ankyrin-1	R.LLLQYDAEIDDITLDHLTPL.H	2	3.77	0.45	-3.48
IPI00216697	Isoform Er1 of Ankyrin-1	R.LLLQYDAEIDDITLDHLTPLHVAAHCGHHR.V	3	3.37	0.31	-4.71
IPI00216697	Isoform Er1 of Ankyrin-1	R.LLLQYDAEIDDITLDHLTPLHVAAHCGHHR.V	5	3.76	0.36	-4.22
IPI00216697	Isoform Er1 of Ankyrin-1	R.LLLQYDAEIDDITLDHLTPLHVAAHCGHHR.V	6	3.88	0.35	-4.48
IPI00216697	Isoform Er1 of Ankyrin-1	R.SLLQYGGSANAESVQGVTPLHLAAQEGHAEM*VALLLSK.Q	4	3.28	0.14	-4.71
IPI00216697	Isoform Er1 of Ankyrin-1	R.SLLQYGGSANAESVQGVTPLHLAAQEGHAEMVALLLSK.Q	3	6.84	0.63	-6.56
IPI00216697	Isoform Er1 of Ankyrin-1	R.SLLQYGGSANAESVQGVTPLHLAAQEGHAEMVALLLSK.Q	4	4.19	0.42	-4.90
IPI00216697	Isoform Er1 of Ankyrin-1	R.SRDIEVLEGMSLFAELSGNLVPVK.K	2	4.47	0.48	-5.20
IPI00216697	Isoform Er1 of Ankyrin-1	R.SRDIEVLEGMSLFAELSGNLVPVK.K	3	2.98	0.31	-4.68
IPI00216697	Isoform Er1 of Ankyrin-1	R.SRDIEVLEGMSLFAELSGNLVPVKK.A	3	5.76	0.48	-7.02
IPI00216697	Isoform Er1 of Ankyrin-1	R.SRDIEVLEGMSLFAELSGNLVPVKK.A	4	4.16	0.31	-3.33
IPI00216697	Isoform Er1 of Ankyrin-1	R.VENPNSLLEQSVALLNLWVIR.E	2	4.37	0.48	-4.65
IPI00216697	Isoform Er1 of Ankyrin-1	R.VENPNSLLEQSVALLNLWVIR.E	3	3.44	0.24	-5.84
IPI00216699	Isoform 2 of Unc-112-related protein 2	R.GEELDEDLFLQLTGGHEAF.-	2	2.75	0.25	-2.99
IPI00216699	Isoform 2 of Unc-112-related protein 2	R.ILEAHQNVAQLSLAEAQLR.F	3	4.22	0.35	-4.02
IPI00216704	Isoform 2 of Spectrin beta chain, erythrocyte	F.VAELASHEGWLENIDAEGK.Q	3	3.63	0.21	-5.56
IPI00216704	Isoform 2 of Spectrin beta chain, erythrocyte	K.DGLNEMWADLLELIDTR.M	2	5.41	0.46	-3.72
IPI00216704	Isoform 2 of Spectrin beta chain, erythrocyte	K.DGLNEMWADLLELIDTR.M	3	3.99	0.22	-2.40
IPI00216704	Isoform 2 of Spectrin beta chain, erythrocyte	K.EQEVSAAWQALLDACAGR.R	2	6.06	0.61	-5.97
IPI00216704	Isoform 2 of Spectrin beta chain, erythrocyte	K.EQEVSAAWQALLDACAGR.R	3	3.46	0.36	-3.61
IPI00216704	Isoform 2 of Spectrin beta chain, erythrocyte	K.FFWEMDEAESWIK.E	2	4.23	0.35	-5.55
IPI00216704	Isoform 2 of Spectrin beta chain, erythrocyte	K.GNLEVLLFTIQSR.M	2	3.04	0.26	-3.75
IPI00216704	Isoform 2 of Spectrin beta chain, erythrocyte	K.GNLEVLLFTIQSR.M	3	2.73	0.27	-2.14
IPI00216704	Isoform 2 of Spectrin beta chain, erythrocyte	K.HQAFVAELASHEGWLENIDAEGK.Q	2	5.79	0.59	-3.51
IPI00216704	Isoform 2 of Spectrin beta chain, erythrocyte	K.HQAFVAELASHEGWLENIDAEGK.Q	3	6.29	0.58	-3.16
IPI00216704	Isoform 2 of Spectrin beta chain, erythrocyte	K.HQAFVAELASHEGWLENIDAEGK.Q	4	2.99	0.23	-3.06
IPI00216704	Isoform 2 of Spectrin beta chain, erythrocyte	K.NFSACLELGESLLQR.Q	2	5.07	0.48	-7.21
IPI00216704	Isoform 2 of Spectrin beta chain, erythrocyte	K.NFSACLELGESLLQR.Q	3	2.67	0.18	-5.33
IPI00216704	Isoform 2 of Spectrin beta chain, erythrocyte	Q.SLQGQEDLLGEVSQLQAFLQDLDDFQAWLSITQK.A	3	7.17	0.63	-2.22
IPI00216704	Isoform 2 of Spectrin beta chain, erythrocyte	R.DANEAQQYYLDADEAEAWIGEQELYVISDEIPKDEEGAIVMLK.R	3	3.88	0.55	-3.48
IPI00216704	Isoform 2 of Spectrin beta chain, erythrocyte	R.DANEAQQYYLDADEAEAWIGEQELYVISDEIPKDEEGAIVMLK.R	4	5.29	0.52	-4.03
IPI00216704	Isoform 2 of Spectrin beta chain, erythrocyte	R.DASVAEAWLIAQEPYLASGDFGHTVDSVEK.L	3	4.63	0.41	-5.30
IPI00216704	Isoform 2 of Spectrin beta chain, erythrocyte	R.DLLSWMESIIR.Q	2	3.79	0.32	-4.22
IPI00216704	Isoform 2 of Spectrin beta chain, erythrocyte	R.DNLELQNFLQNCQELTLWINDK.L	2	5.05	0.54	-4.37
IPI00216704	Isoform 2 of Spectrin beta chain, erythrocyte	R.DNLELQNFLQNCQELTLWINDK.L	3	6.52	0.51	-5.54
IPI00216704	Isoform 2 of Spectrin beta chain, erythrocyte	R.ELHLLGVQVQQFQDVATR.L	3	4.97	0.38	-3.45
IPI00216704	Isoform 2 of Spectrin beta chain, erythrocyte	R.ELYQQVVAQADLR.Q	2	3.98	0.23	-2.66
IPI00216704	Isoform 2 of Spectrin beta chain, erythrocyte	R.GQQLVEAAEIDCQDLEER.L	2	6.01	0.61	-3.65

IPI00216704	Isoform 2 of Spectrin beta chain, erythrocyte	R.KEELGELFAQVPSMGEEGGDADLSIEK.R	3	5.55	0.47	-1.22
IPI00216704	Isoform 2 of Spectrin beta chain, erythrocyte	R.LIDAGHSEAATIAEWK.D	3	2.93	0.20	-2.76
IPI00216704	Isoform 2 of Spectrin beta chain, erythrocyte	R.LQEALDLYTVFGETDACELWM*GEK.E	2	4.54	0.58	-4.99
IPI00216704	Isoform 2 of Spectrin beta chain, erythrocyte	R.LQEALDLYTVFGETDACELWM*GEK.E	3	3.15	0.33	-3.95
IPI00216704	Isoform 2 of Spectrin beta chain, erythrocyte	R.LQEALDLYTVFGETDACELWMGEK.E	2	5.20	0.64	-4.82
IPI00216704	Isoform 2 of Spectrin beta chain, erythrocyte	R.LQEALDLYTVFGETDACELWMGEK.E	3	5.56	0.57	-6.14
IPI00216704	Isoform 2 of Spectrin beta chain, erythrocyte	R.LVAQDNFGYDLAAVEAAK.K	2	5.35	0.49	-4.38
IPI00216704	Isoform 2 of Spectrin beta chain, erythrocyte	R.YFYTGAEILGLIDEK.H	2	5.13	0.46	-5.05
IPI00216704	Isoform 2 of Spectrin beta chain, erythrocyte	R.YFYTGAEILGLIDEK.H	3	2.92	0.19	-3.76
IPI00216728	Neurexin 3-alpha	A.PGLGDFLQLHIEQGK.I	2	3.08	0.35	-4.17
IPI00216728	Neurexin 3-alpha	G.LEFM*GLPNQWAR.Y	2	4.31	0.34	-3.18
IPI00216728	Neurexin 3-alpha	K.AREENVATFR.G	2	3.72	0.23	-2.99
IPI00216728	Neurexin 3-alpha	K.CENVATLDPINFETPEAYISLPK.W	2	5.59	0.47	-5.72
IPI00216728	Neurexin 3-alpha	K.DGAVSLVINLGSGAFEAIVEPVNGK.F	3	3.16	0.21	-6.08
IPI00216728	Neurexin 3-alpha	K.EVVYKNNDIR.L	2	2.17	0.11	-0.62
IPI00216728	Neurexin 3-alpha	K.EVVYKNNDIR.L	3	2.10	0.14	-2.04
IPI00216728	Neurexin 3-alpha	K.GDLYM*AGLAQGM*YSNLPK.L	2	5.40	0.57	-3.13
IPI00216728	Neurexin 3-alpha	K.GDLYM*AGLAQGM*YSNLPK.L	3	3.88	0.45	-2.41
IPI00216728	Neurexin 3-alpha	K.GPETLYAGQK.L	1	2.11	0.26	-1.68
IPI00216728	Neurexin 3-alpha	K.GPETLYAGQK.L	2	2.94	0.21	0.06
IPI00216728	Neurexin 3-alpha	K.GRLFQGQLSGLYYDGLK.V	3	4.55	0.43	-3.36
IPI00216728	Neurexin 3-alpha	K.GYIHYVFDLGNGPNVIK.G	2	4.55	0.44	-1.43
IPI00216728	Neurexin 3-alpha	K.GYIHYVFDLGNGPNVIK.G	3	3.88	0.22	-3.69
IPI00216728	Neurexin 3-alpha	K.IIM*PM*VM*HTEAEDVSFR.F	2	4.07	0.50	-3.30
IPI00216728	Neurexin 3-alpha	K.IIM*PM*VM*HTEAEDVSFR.F	3	2.09	0.32	-2.29
IPI00216728	Neurexin 3-alpha	K.IYGEVVFK.C	1	2.06	0.22	-2.31
IPI00216728	Neurexin 3-alpha	K.IYGEVVFK.C	2	1.55	0.06	-1.54
IPI00216728	Neurexin 3-alpha	K.LM*VNLGKGPETLYAGQK.L	3	3.03	0.26	-1.32
IPI00216728	Neurexin 3-alpha	K.NLDLKGDLYM*AGLAQGM*YSNLPK.L	2	4.64	0.51	-3.72
IPI00216728	Neurexin 3-alpha	K.NLDLKGDLYM*AGLAQGM*YSNLPK.L	3	5.76	0.43	-3.66
IPI00216728	Neurexin 3-alpha	K.SADYVNLALK.D	1	2.35	0.24	-3.84
IPI00216728	Neurexin 3-alpha	K.SADYVNLALK.D	2	3.96	0.37	-1.89
IPI00216728	Neurexin 3-alpha	K.SGGLILYTWPANDRPSTR.S	2	2.76	0.21	-3.81
IPI00216728	Neurexin 3-alpha	K.SGGLILYTWPANDRPSTR.S	3	2.88	0.35	-3.79
IPI00216728	Neurexin 3-alpha	K.TTSPDGFILFNSGDGNDFIAVELVK.G	2	4.16	0.50	-4.19
IPI00216728	Neurexin 3-alpha	K.TTSPDGFILFNSGDGNDFIAVELVK.G	3	3.53	0.34	-4.27
IPI00216728	Neurexin 3-alpha	K.VLNM*AAENNPNIK.I	2	4.53	0.36	-2.56
IPI00216728	Neurexin 3-alpha	K.VLNM*AAENNPNIK.I	3	3.80	0.23	-0.76
IPI00216728	Neurexin 3-alpha	K.VVTQVINGAK.N	1	2.30	0.15	-1.44
IPI00216728	Neurexin 3-alpha	K.VVTQVINGAK.N	2	2.99	0.14	-2.87
IPI00216728	Neurexin 3-alpha	K.YGNSEPR.L	2	1.92	0.25	-1.41
IPI00216728	Neurexin 3-alpha	R.AYGLLVATTSR.D	1	2.21	0.33	-3.49

IPI00216728	Neurexin 3-alpha	R.AYGLLVATTSR.D	2	4.29	0.40	-3.80
IPI00216728	Neurexin 3-alpha	R.DGFQGCLASVDLNGR.L	2	4.89	0.57	-5.11
IPI00216728	Neurexin 3-alpha	R.DGFQGCLASVDLNGRLPDLINDALHR.S	3	5.12	0.47	-2.78
IPI00216728	Neurexin 3-alpha	R.DLFIDGR.S	1	2.11	0.13	-1.89
IPI00216728	Neurexin 3-alpha	R.DNSNTHSLKVDTK.V	2	3.62	0.42	-3.87
IPI00216728	Neurexin 3-alpha	R.DSADTLRLELDGGR.V	3	2.60	0.27	-2.05
IPI00216728	Neurexin 3-alpha	R.DSADTLRLELDGGRVK.L	3	4.11	0.27	-1.81
IPI00216728	Neurexin 3-alpha	R.EASILSYDGSM*YM*K.I	2	4.21	0.13	-1.99
IPI00216728	Neurexin 3-alpha	R.EENVATFR.G	1	2.23	0.18	-3.61
IPI00216728	Neurexin 3-alpha	R.EENVATFR.G	2	2.00	0.23	-3.05
IPI00216728	Neurexin 3-alpha	R.FSM*DCAETAVLSNK.Q	2	4.81	0.52	-4.57
IPI00216728	Neurexin 3-alpha	R.FSM*DCAETAVLSNK.Q	3	2.84	0.28	-3.73
IPI00216728	Neurexin 3-alpha	R.GSEYLCYDLSQNPIQSSSDEITLSFK.T	2	4.47	0.54	-4.80
IPI00216728	Neurexin 3-alpha	R.GVQM*DAEGPCGERPCENGGICFLLDGHPTCDCSTTGYGGK.L	4	4.67	0.45	-2.22
IPI00216728	Neurexin 3-alpha	R.IDSAPGLGDFLQLHIEQGK.I	2	5.03	0.43	-2.90
IPI00216728	Neurexin 3-alpha	R.IDSAPGLGDFLQLHIEQGK.I	3	2.74	0.34	-3.15
IPI00216728	Neurexin 3-alpha	R.LAVGFSTTVK.D	2	2.73	0.28	-1.95
IPI00216728	Neurexin 3-alpha	R.LAVGFSTTVKDGILVR.I	3	2.19	0.19	-2.27
IPI00216728	Neurexin 3-alpha	R.LEFHNIETGIM*TEK.R	2	4.01	0.36	-2.47
IPI00216728	Neurexin 3-alpha	R.LEFHNIETGIM*TEK.R	3	3.67	0.33	-3.05
IPI00216728	Neurexin 3-alpha	R.LEFHNIETGIM*TEKR.Y	2	3.67	0.38	-3.02
IPI00216728	Neurexin 3-alpha	R.LEFHNIETGIM*TEKR.Y	3	3.74	0.43	-2.30
IPI00216728	Neurexin 3-alpha	R.LFQGQLSGLYYDGLK.V	2	5.21	0.39	-6.20
IPI00216728	Neurexin 3-alpha	R.LFQGQLSGLYYDGLK.V	3	4.24	0.36	-3.34
IPI00216728	Neurexin 3-alpha	R.LPDLINDALHR.S	2	3.78	0.36	-2.41
IPI00216728	Neurexin 3-alpha	R.LPDLINDALHR.S	3	4.14	0.33	-2.11
IPI00216728	Neurexin 3-alpha	R.M*GSISFDFR.T	1	1.31	0.22	-3.10
IPI00216728	Neurexin 3-alpha	R.M*GSISFDFR.T	2	3.82	0.38	-2.50
IPI00216728	Neurexin 3-alpha	R.NGLILHTGK.S	1	2.65	0.14	-3.65
IPI00216728	Neurexin 3-alpha	R.NGLILHTGK.S	2	2.77	0.15	-1.89
IPI00216728	Neurexin 3-alpha	R.NIIADPVTFK.T	1	2.97	0.31	-2.13
IPI00216728	Neurexin 3-alpha	R.NIIADPVTFK.T	2	2.61	0.15	-2.52
IPI00216728	Neurexin 3-alpha	R.QLAEM*QNAAGVK.S	1	1.24	0.11	-2.06
IPI00216728	Neurexin 3-alpha	R.QLAEM*QNAAGVK.S	2	2.93	0.37	-3.09
IPI00216728	Neurexin 3-alpha	R.QLTIFNTQAQIAIGGK.D	2	4.62	0.44	-6.95
IPI00216728	Neurexin 3-alpha	R.QLTIFNTQAQIAIGGKDK.G	3	2.53	0.33	-4.21
IPI00216728	Neurexin 3-alpha	R.RTPFTASGESEILDLEGDM*YLGGLPENR.A	3	3.70	0.29	-1.88
IPI00216728	Neurexin 3-alpha	R.SDLSFQFK.T	1	2.63	0.17	-2.25
IPI00216728	Neurexin 3-alpha	R.SDLSFQFK.T	2	2.55	0.09	-2.93
IPI00216728	Neurexin 3-alpha	R.SGTISVNSR.R	1	2.12	0.18	-1.75
IPI00216728	Neurexin 3-alpha	R.SGTISVNSR.R	2	2.13	0.07	-0.95
IPI00216728	Neurexin 3-alpha	R.SKAREENVATFR.G	3	2.06	0.13	-2.77

IPI00216728	Neurexin 3-alpha	R.TPFTASGESEILDLEGDM*YLGGLPENR.A	2	4.48	0.55	-4.54
IPI00216728	Neurexin 3-alpha	R.TPFTASGESEILDLEGDM*YLGGLPENR.A	3	4.09	0.37	-4.17
IPI00216728	Neurexin 3-alpha	R.TPVNDGKYHVVR.F	2	3.84	0.11	-4.34
IPI00216728	Neurexin 3-alpha	R.TTEPNGLILFTHGKPQER.K	2	3.21	0.37	-3.92
IPI00216728	Neurexin 3-alpha	R.TTEPNGLILFTHGKPQER.K	3	2.43	0.09	-0.33
IPI00216728	Neurexin 3-alpha	R.TTEPNGLILFTHGKPQER.K	4	3.21	0.32	-0.66
IPI00216728	Neurexin 3-alpha	W.PVNEHYPTGNTDNER.F	3	3.78	0.46	-1.79
IPI00216774	Cerebellin-2	R.EAASNGVLLLM*ER.E	2	4.00	0.29	-1.19
IPI00216780	Cartilage intermediate layer protein 2 precursor	R.EM*SEAAQAQAR.A	2	2.76	0.19	-3.36
IPI00216882	mannan-binding lectin serine protease 1 isoform 3	K.APEPISTQSHSVLILFHSDNSGENR.G	4	3.04	0.14	-1.53
IPI00216882	mannan-binding lectin serine protease 1 isoform 3	K.DNVEM*DTFQIECLK.D	2	3.06	0.22	
IPI00216882	mannan-binding lectin serine protease 1 isoform 3	K.DQVLVSCDTGYK.V	2	3.81	0.34	-3.52
IPI00216882	mannan-binding lectin serine protease 1 isoform 3	K.SDFSNEER.F	2	2.18	0.17	-2.43
IPI00216882	mannan-binding lectin serine protease 1 isoform 3	R.AAGNECPELQPPVHGK.I	2	2.11	0.25	
IPI00216882	mannan-binding lectin serine protease 1 isoform 3	R.ETTDTEQTPGQEVVLSPGSFM*SITFR.S	3	4.46	0.32	-5.59
IPI00216882	mannan-binding lectin serine protease 1 isoform 3	R.TGVITSPDFPNPYPK.S	2	3.60	0.27	
IPI00216914	Vitelline membrane outer layer protein 1 homolog precursor	R.GLGDDTALNDAR.L	2	3.87	0.35	-2.52
IPI00216921	Isoform 2 of Stathmin-4	R.ERRAQADTVDLNWCVISDMEVIELNK.C	3	2.50	0.08	-3.16
IPI00216963	Isoform 9 of CASP8 and FADD-like apoptosis regulator precursor	R.GPAGGQQPLGGGWASDEECGIQGSEARAVHSSPR.S	3	1.44	0.21	1.08
IPI00216983	Carbonic anhydrase 3	K.EPM*TVSSDQM*AK.L	2	2.30	0.40	-3.65
IPI00216983	Carbonic anhydrase 3	K.GENQSPVELHTK.D	2	2.89	0.24	-2.76
IPI00216983	Carbonic anhydrase 3	K.GKEAPFTK.F	2	2.00	0.06	-3.54
IPI00216983	Carbonic anhydrase 3	K.IGHENGEFQIFLDALDK.I	3	4.07	0.11	-2.49
IPI00216983	Carbonic anhydrase 3	K.IGHENGEFQIFLDALDKIK.T	2	5.26	0.47	-5.18
IPI00216983	Carbonic anhydrase 3	K.IGHENGEFQIFLDALDKIK.T	3	5.31	0.50	-3.86
IPI00216983	Carbonic anhydrase 3	K.IGHENGEFQIFLDALDKIK.T	4	5.76	0.37	-4.07
IPI00216983	Carbonic anhydrase 3	K.YNTFKEALK.Q	2	2.19	0.19	0.87
IPI00216983	Carbonic anhydrase 3	R.VVFDDTYDR.S	2	1.54	0.11	-3.43
IPI00217005	Ankyrin repeat domain-containing protein 18A	K.EAFAGAVKANNSMSKK.L	2	3.09	0.16	
IPI00217005	Ankyrin repeat domain-containing protein 18A	R.NDNQETAAMKPANLKKRK.E	2	2.09	0.11	-7.93
IPI00217012	pleckstrin and Sec7 domain containing	K.VHADPDCRKTPRGKR.G	3	2.11	0.14	-1.22
IPI00217023	MMAA protein	R.EQIPLLEQKVLIGA.L	1	3.64	0.07	
IPI00217146	SLIT and NTRK-like protein 4 precursor	K.AWLENMPYNIYIGEAICETPSDLYGRLLKET.N	3	3.88	0.11	3.01
IPI00217146	SLIT and NTRK-like protein 4 precursor	K.LPYIGVLEHIGR.V	3	2.60	0.15	-3.16
IPI00217146	SLIT and NTRK-like protein 4 precursor	K.LQNIEGGAFLGLSALK.Q	2	5.39	0.53	-3.77
IPI00217146	SLIT and NTRK-like protein 4 precursor	K.LSDGIVVK.E	2	2.16	0.07	-3.00
IPI00217146	SLIT and NTRK-like protein 4 precursor	K.VLILNDNLISFLPDNIFR.F	2	5.70	0.47	-5.12
IPI00217146	SLIT and NTRK-like protein 4 precursor	R.ADTFLGIENLEYLQADYNLIK.Y	2	4.96	0.52	-4.51
IPI00217146	SLIT and NTRK-like protein 4 precursor	R.ADTFLGIENLEYLQADYNLIK.Y	3	4.65	0.42	-5.92
IPI00217146	SLIT and NTRK-like protein 4 precursor	R.FASLTHLDIR.G	2	2.95	0.24	-3.25

IPI00217146	SLIT and NTRK-like protein 4 precursor	R.IQKLPYIGVLEHIGR.V	3	4.24	0.31	-3.61
IPI00217146	SLIT and NTRK-like protein 4 precursor	R.IQKLPYIGVLEHIGR.V	4	4.81	0.33	-3.40
IPI00217236	Tubulin-specific chaperone A	K.M*RAEDGENYDIKK.Q	3	3.07	0.22	-2.12
IPI00217236	Tubulin-specific chaperone A	R.ILENEKDLEEAEEYKEAR.L	4	2.23	0.12	-1.96
IPI00217236	Tubulin-specific chaperone A	R.M*M*IPDCQR.R	2	2.10	0.09	-2.16
IPI00217236	Tubulin-specific chaperone A	R.RLEAAYLDLQR.I	3	3.45	0.11	-4.18
IPI00217258	CCDC100 protein	-.MVSKSDQLLIVVSILEGRHFPK.R	2	1.06	0.18	-6.56
IPI00217264	Isoform 3 of MAP7 domain-containing protein 3	K.EAVGGQAEDHLK.L	3	1.68	0.11	1.00
IPI00217345	Isoform 2 of UDP-GlcNAc:betaGal beta-1,3-N-acetylglucosaminyltransferase 2	K.SLTPHFAR.R	2	1.41	0.18	-3.32
IPI00217345	Isoform 2 of UDP-GlcNAc:betaGal beta-1,3-N-acetylglucosaminyltransferase 2	R.FKDFLLYLR.C	2	3.38	0.27	-4.55
IPI00217345	Isoform 2 of UDP-GlcNAc:betaGal beta-1,3-N-acetylglucosaminyltransferase 2	R.VFLLGQTPPEDNHPDLSDM*LK.F	3	2.93	0.23	-3.55
IPI00217345	Isoform 2 of UDP-GlcNAc:betaGal beta-1,3-N-acetylglucosaminyltransferase 2	R.VTSVVTGFNNLPDR.F	2	4.38	0.45	-2.01
IPI00217376	Isoform 1 of Sodium channel subunit beta-4 precursor	K.ILIEGTVKNEK.S	2	2.77	0.25	-2.86
IPI00217376	Isoform 1 of Sodium channel subunit beta-4 precursor	R.DLEFSDTGK.Y	2	2.40	0.26	-2.36
IPI00217376	Isoform 1 of Sodium channel subunit beta-4 precursor	R.ITLVGSTK.E	2	1.75	0.08	-3.07
IPI00217405	Isoform 1 of E3 ubiquitin-protein ligase UBR1	K.ILTCM*QGMEEIR.R	2	2.53	0.17	2.31
IPI00217435	Signal peptide, CUB and EGF-like domain-containing protein 1 precursor	K.YALHSDGR.T	2	2.32	0.29	-3.08
IPI00217465	Histone H1.2	K.KAKKPAAATVTKK.V	2	2.97	0.29	-2.90
IPI00217465	Histone H1.2	R.KASGPPVSELITK.A	3	3.71	0.18	-3.59
IPI00217465	Histone H1.2	R.SGVSLAALK.K	2	2.29	0.08	-2.33
IPI00217465	Histone H1.2	R.SGVSLAALKK.A	2	3.07	0.16	0.78
IPI00217466	Histone H1.3	K.KVKKPATAAGTKK.V	2	3.00	0.15	-3.00
IPI00217466	Histone H1.3	K.KVKKPATAAGTKK.V	3	3.40	0.22	-2.84
IPI00217466	Histone H1.3	R.KASGPPVSELITK.A	3	3.71	0.18	-3.59
IPI00217466	Histone H1.3	R.SGVSLAALK.K	2	2.29	0.08	-2.33
IPI00217466	Histone H1.3	R.SGVSLAALKK.A	2	3.07	0.16	0.78
IPI00217467	Histone H1.4	K.AKKPAGAAKKPK.K	2	2.72	0.32	-3.80
IPI00217467	Histone H1.4	R.KASGPPVSELITK.A	3	3.71	0.18	-3.59
IPI00217467	Histone H1.4	R.SGVSLAALK.K	2	2.29	0.08	-2.33
IPI00217467	Histone H1.4	R.SGVSLAALKK.A	2	3.07	0.16	0.78
IPI00217493	Myoglobin	H.GATVLTALGGILK.K	2	3.23	0.20	-1.15
IPI00217493	Myoglobin	K.DM*ASNYKELGFQG.-	2	3.77	0.44	-3.76
IPI00217493	Myoglobin	K.GHPETLEKFDK.F	2	2.83	0.29	-3.30
IPI00217493	Myoglobin	K.HGATVLTALGGILK.K	2	4.44	0.50	-1.68

IPI00217493	Myoglobin	K.HGATVLTALGGILK.K	3	3.08	0.14	-0.87
IPI00217493	Myoglobin	K.HPGDFGADAQGAM*NK.A	2	2.84	0.26	-3.03
IPI00217493	Myoglobin	K.HPGDFGADAQGAM*NK.A	3	4.04	0.26	-1.32
IPI00217493	Myoglobin	K.SEDEM*KASEDLKK.H	3	2.68	0.12	-1.86
IPI00217493	Myoglobin	K.VEADIPGHGQEVLIR.L	2	2.53	0.21	-2.78
IPI00217493	Myoglobin	K.VEADIPGHGQEVLIR.L	3	3.49	0.40	-1.31
IPI00217493	Myoglobin	P.GDFGADAQGAM*NK.A	2	3.62	0.42	-4.55
IPI00217537	Isoform 1 of Putative Polycomb group protein ASXL1	K.KKTGVM*LPRVVLTPLK.V	2	2.64	0.11	
IPI00217617	palmitoylated membrane protein 7	K.IIRLVKNREPLGATIKK.D	2	1.49	0.17	-8.73
IPI00217652	Isoform 1 of Glycosyltransferase 8 domain-containing protein 3	R.SLLKPLELELQK.T	3	3.09	0.09	-1.97
IPI00217740	C20orf12 protein	K.SDTPDVNIYYTLDGSKPEFLK.R	2	1.16	0.18	2.21
IPI00217759	Isoform 1 of Alpha-(1,3)-fucosyltransferase 11	K.QPGGITNQFLLDSLK.H	2	3.05	0.39	-3.88
IPI00217759	Isoform 1 of Alpha-(1,3)-fucosyltransferase 11	R.ALLFYGTDFR.A	2	3.20	0.29	-3.57
IPI00217759	Isoform 1 of Alpha-(1,3)-fucosyltransferase 11	R.EEAGDLPVLLWWSPGLFPHFPGDSER.I	3	2.83	0.38	-3.37
IPI00217759	Isoform 1 of Alpha-(1,3)-fucosyltransferase 11	R.LQDTATATTEDPELLAFLSR.Y	2	6.58	0.62	-4.31
IPI00217759	Isoform 1 of Alpha-(1,3)-fucosyltransferase 11	R.LQDTATATTEDPELLAFLSR.Y	3	5.38	0.26	-3.40
IPI00217781	Similar to expressed sequence AI593442	R.TFASPNASGSGNTGAR.G	2	4.28	0.39	-2.26
IPI00217791	Coiled-coil domain-containing protein 105	K.TLASCRDTLNFCFKERLQAVDLM*NQPLDK.V	3	2.48	0.09	1.07
IPI00217831	Ankyrin repeat domain-containing protein 13A	R.TDKAEVVNGYEAKVYTVNNVNVITKIR.T	3	2.93	0.10	-8.33
IPI00217831	Ankyrin repeat domain-containing protein 13A	R.TDKAEVVNGYEAKVYTVNNVNVITKIR.T	4	3.08	0.25	-8.45
IPI00217871	Delta-1-pyrroline-5-carboxylate dehydrogenase, mitochondrial precursor	K.TVIQAEIDAAAELIDFFR.F	3	3.04	0.32	-2.78
IPI00217882	Sortilin precursor	K.ADLGALELWR.T	2	2.44	0.13	-2.07
IPI00217882	Sortilin precursor	K.VVLTAEVSGGSR.G	2	3.06	0.36	
IPI00217882	Sortilin precursor	R.LDAPPPPAAPLPR.W	2	2.37	0.32	-3.56
IPI00217882	Sortilin precursor	R.TEFGM*AIGPENSGK.V	2	4.15	0.46	-3.68
IPI00217948	FRMD4B protein	R.SLDEIAM*DLTETGTQRASKLVTLETKSQ.F	3	3.55	0.24	-3.55
IPI00217963	Keratin, type I cytoskeletal 16	R.LAADDFR.L	2	2.47	0.23	-3.76
IPI00217963	Keratin, type I cytoskeletal 16	R.LASYLDKVR.A	2	2.65	0.13	-3.49
IPI00217966	Isoform 1 of L-lactate dehydrogenase A chain	K.DLADELALVDVIEDK.L	2	4.75	0.48	-3.51
IPI00217966	Isoform 1 of L-lactate dehydrogenase A chain	K.DQLIYNLLKEEQTPQNK.I	2	4.91	0.27	-2.73
IPI00217966	Isoform 1 of L-lactate dehydrogenase A chain	K.DQLIYNLLKEEQTPQNK.I	3	4.59	0.24	-2.30
IPI00217966	Isoform 1 of L-lactate dehydrogenase A chain	K.DYNVTANSK.L	2	2.82	0.33	-3.30
IPI00217966	Isoform 1 of L-lactate dehydrogenase A chain	K.FIIPNVVK.Y	2	2.46	0.09	-1.44
IPI00217966	Isoform 1 of L-lactate dehydrogenase A chain	K.GLYGIKDDVFLSVPCILGQNGISDLVK.V	3	3.30	0.33	-3.40
IPI00217966	Isoform 1 of L-lactate dehydrogenase A chain	K.GYTSWAIGLSVADLAESIMK.N	2	3.13	0.43	-2.46
IPI00217966	Isoform 1 of L-lactate dehydrogenase A chain	K.IVSGKDYNVTANSK.L	2	4.18	0.44	-2.99
IPI00217966	Isoform 1 of L-lactate dehydrogenase A chain	K.IVSGKDYNVTANSK.L	3	2.89	0.33	-1.83
IPI00217966	Isoform 1 of L-lactate dehydrogenase A chain	K.VTLTSEEEAR.L	2	3.49	0.25	-3.36
IPI00217966	Isoform 1 of L-lactate dehydrogenase A chain	N.LVIITAGAR.Q	2	3.11	0.22	-1.00

IPI00217966	Isoform 1 of L-lactate dehydrogenase A chain	R.NVNIFKFIIPNVVK.Y	3	3.16	0.23	-2.65
IPI00217966	Isoform 1 of L-lactate dehydrogenase A chain	R.VIGSGCNLDSAR.F	1	2.49	0.27	-2.12
IPI00217966	Isoform 1 of L-lactate dehydrogenase A chain	R.VIGSGCNLDSAR.F	2	3.57	0.37	-2.86
IPI00217989	Isoform 1 of Protein-associating with the carboxyl-terminal domain of ezrin	K.LGGM*ETVCKVSQATPEFLR.S	2	2.17	0.07	-7.56
IPI00218046	Heparan-sulfate 6-O-sulfotransferase 3	K.NM*AFFGLTEFQR.K	2	3.17	0.34	-6.41
IPI00218046	Heparan-sulfate 6-O-sulfotransferase 3	K.TGGTTFGR.H	1	1.24	0.17	-0.11
IPI00218046	Heparan-sulfate 6-O-sulfotransferase 3	K.TQFLFER.T	2	1.77	0.08	-1.10
IPI00218046	Heparan-sulfate 6-O-sulfotransferase 3	R.AGEAGPPAVPGPAR.R	2	3.17	0.30	-3.71
IPI00218046	Heparan-sulfate 6-O-sulfotransferase 3	R.AGEAGPPAVPGPARR.A	3	2.45	0.23	-2.76
IPI00218046	Heparan-sulfate 6-O-sulfotransferase 3	R.ASNVEINEGAR.Q	2	4.11	0.37	-1.52
IPI00218046	Heparan-sulfate 6-O-sulfotransferase 3	R.EFM*DCTYNLANNR.Q	2	1.94	0.17	-3.21
IPI00218046	Heparan-sulfate 6-O-sulfotransferase 3	R.ETWLFSR.F	1	1.99	0.19	-2.50
IPI00218046	Heparan-sulfate 6-O-sulfotransferase 3	R.FSTGWSCGLHADWTELTNCVPAIM*EK.K	3	2.99	0.33	-4.92
IPI00218046	Heparan-sulfate 6-O-sulfotransferase 3	R.GAAAPEEEDEEPGDPR.E	2	3.20	0.41	-4.02
IPI00218046	Heparan-sulfate 6-O-sulfotransferase 3	R.IEDLNFLDM*QLYEYAK.D	2	5.12	0.50	-4.73
IPI00218046	Heparan-sulfate 6-O-sulfotransferase 3	R.KTQFLFER.T	2	2.83	0.21	-2.44
IPI00218046	Heparan-sulfate 6-O-sulfotransferase 3	R.NFYYITM*LR.D	2	2.91	0.24	-0.96
IPI00218046	Heparan-sulfate 6-O-sulfotransferase 3	R.QRIEDLNFLDM*QLYEYAK.D	2	3.54	0.38	-2.42
IPI00218046	Heparan-sulfate 6-O-sulfotransferase 3	R.QRIEDLNFLDM*QLYEYAK.D	3	3.79	0.29	-1.54
IPI00218075	Protein FAM9B	K.MDKTCSKTK.N	1	2.22	0.06	-3.66
IPI00218130	Glycogen phosphorylase, muscle form	R.LKQEYFVVAATLQDIIR.R	3	3.33	0.27	-2.88
IPI00218131	Protein S100-A12	M.TKLEEHLEGIVNIFHQYSVR.K	3	5.34	0.39	-3.15
IPI00218192	Isoform 2 of Inter-alpha-trypsin inhibitor heavy chain H4 precursor	A.EKNGIDIYSLTVDSR.V	2	4.52	0.51	-2.76
IPI00218192	Isoform 2 of Inter-alpha-trypsin inhibitor heavy chain H4 precursor	K.AEAQAQYSAAVAK.G	1	3.20	0.31	-3.17
IPI00218192	Isoform 2 of Inter-alpha-trypsin inhibitor heavy chain H4 precursor	K.AEAQAQYSAAVAK.G	2	4.88	0.46	-5.13
IPI00218192	Isoform 2 of Inter-alpha-trypsin inhibitor heavy chain H4 precursor	K.AGFSWIEVTFK.N	2	3.83	0.37	-3.40
IPI00218192	Isoform 2 of Inter-alpha-trypsin inhibitor heavy chain H4 precursor	K.EKAEAQAQYSAAVAK.G	2	4.70	0.52	-3.59
IPI00218192	Isoform 2 of Inter-alpha-trypsin inhibitor heavy chain H4 precursor	K.EKAEAQAQYSAAVAK.G	3	2.95	0.20	-1.30
IPI00218192	Isoform 2 of Inter-alpha-trypsin inhibitor heavy chain H4 precursor	K.ETLFSVM*PGLK.M	2	3.05	0.37	-2.95
IPI00218192	Isoform 2 of Inter-alpha-trypsin inhibitor heavy chain H4 precursor	K.GSEM*VVAGK.L	1	2.36	0.16	-2.42
IPI00218192	Isoform 2 of Inter-alpha-trypsin inhibitor heavy chain H4 precursor	K.GSEM*VVAGK.L	2	3.20	0.25	-3.04

IPI00218192	Isoform 2 of Inter-alpha-trypsin inhibitor heavy chain H4 precursor	K.ILDDLSPRDQFNLIVFSTEATQWRPSLVPASAENVNK.A	4	3.61	0.24	-3.03
IPI00218192	Isoform 2 of Inter-alpha-trypsin inhibitor heavy chain H4 precursor	K.ITFELVYEELLK.R	2	4.90	0.43	-6.82
IPI00218192	Isoform 2 of Inter-alpha-trypsin inhibitor heavy chain H4 precursor	K.ITFELVYEELLKR.R	2	3.24	0.31	-3.11
IPI00218192	Isoform 2 of Inter-alpha-trypsin inhibitor heavy chain H4 precursor	K.ITFELVYEELLKR.R	3	2.51	0.18	-4.10
IPI00218192	Isoform 2 of Inter-alpha-trypsin inhibitor heavy chain H4 precursor	K.LALDNGGLAR.R	1	2.35	0.24	-3.22
IPI00218192	Isoform 2 of Inter-alpha-trypsin inhibitor heavy chain H4 precursor	K.LALDNGGLAR.R	2	3.38	0.30	-2.67
IPI00218192	Isoform 2 of Inter-alpha-trypsin inhibitor heavy chain H4 precursor	K.LQDRGPDVLTATVSGK.L	2	4.29	0.52	-2.31
IPI00218192	Isoform 2 of Inter-alpha-trypsin inhibitor heavy chain H4 precursor	K.LQDRGPDVLTATVSGK.L	3	4.37	0.31	-3.49
IPI00218192	Isoform 2 of Inter-alpha-trypsin inhibitor heavy chain H4 precursor	K.NGIDIYSLTVDSR.V	2	4.00	0.40	-2.27
IPI00218192	Isoform 2 of Inter-alpha-trypsin inhibitor heavy chain H4 precursor	K.NPLVWVHASPEHVVVTR.N	3	5.38	0.52	-5.11
IPI00218192	Isoform 2 of Inter-alpha-trypsin inhibitor heavy chain H4 precursor	K.NVVFVIDK.S	1	2.65	0.17	-3.16
IPI00218192	Isoform 2 of Inter-alpha-trypsin inhibitor heavy chain H4 precursor	K.NVVFVIDK.S	2	2.33	0.25	-2.47
IPI00218192	Isoform 2 of Inter-alpha-trypsin inhibitor heavy chain H4 precursor	K.NVVFVIDKSGSM*SGR.K	2	3.23	0.30	-0.83
IPI00218192	Isoform 2 of Inter-alpha-trypsin inhibitor heavy chain H4 precursor	K.NVVFVIDKSGSM*SGR.K	3	2.51	0.33	0.12
IPI00218192	Isoform 2 of Inter-alpha-trypsin inhibitor heavy chain H4 precursor	K.NVVFVIDKSGSM*SGRK.I	3	2.46	0.25	-2.31
IPI00218192	Isoform 2 of Inter-alpha-trypsin inhibitor heavy chain H4 precursor	K.SPEQQETVLDGNLIIR.Y	2	5.01	0.44	-6.49
IPI00218192	Isoform 2 of Inter-alpha-trypsin inhibitor heavy chain H4 precursor	K.SPEQQETVLDGNLIIR.Y	3	4.19	0.20	-4.28
IPI00218192	Isoform 2 of Inter-alpha-trypsin inhibitor heavy chain H4 precursor	K.TGLLLLSDPDKVTIGLLFWDGR.G	3	2.86	0.28	-4.01
IPI00218192	Isoform 2 of Inter-alpha-trypsin inhibitor heavy chain H4 precursor	K.TGLLLLSDPDKVTIGLLFWDGRGEGLR.L	4	3.72	0.15	-1.76
IPI00218192	Isoform 2 of Inter-alpha-trypsin inhibitor heavy chain H4 precursor	K.WKETLFSVM*PGLK.M	2	4.00	0.38	-2.92
IPI00218192	Isoform 2 of Inter-alpha-trypsin inhibitor heavy chain H4 precursor	K.WKETLFSVM*PGLK.M	3	4.12	0.26	-2.35

IPI00218192	Isoform 2 of Inter-alpha-trypsin inhibitor heavy chain H4 precursor	K.YIFHNFM*ER.L	2	2.20	0.19	-0.59
IPI00218192	Isoform 2 of Inter-alpha-trypsin inhibitor heavy chain H4 precursor	R.AISGGSIQIENGYFVHYFAPEGLTTM*PK.N	2	4.26	0.53	-2.44
IPI00218192	Isoform 2 of Inter-alpha-trypsin inhibitor heavy chain H4 precursor	R.AISGGSIQIENGYFVHYFAPEGLTTM*PK.N	3	5.62	0.55	-4.13
IPI00218192	Isoform 2 of Inter-alpha-trypsin inhibitor heavy chain H4 precursor	R.ANTVQEATFQM*ELPK.K	2	5.10	0.41	-4.19
IPI00218192	Isoform 2 of Inter-alpha-trypsin inhibitor heavy chain H4 precursor	R.ANTVQEATFQM*ELPKK.A	2	4.67	0.47	-3.41
IPI00218192	Isoform 2 of Inter-alpha-trypsin inhibitor heavy chain H4 precursor	R.ANTVQEATFQMELPK.K	2	2.99	0.34	
IPI00218192	Isoform 2 of Inter-alpha-trypsin inhibitor heavy chain H4 precursor	R.DQFNLIVFSTEATQWRPSLVPASAENVNK.A	2	2.36	0.40	-1.90
IPI00218192	Isoform 2 of Inter-alpha-trypsin inhibitor heavy chain H4 precursor	R.DQFNLIVFSTEATQWRPSLVPASAENVNK.A	3	6.22	0.54	-3.80
IPI00218192	Isoform 2 of Inter-alpha-trypsin inhibitor heavy chain H4 precursor	R.DQFNLIVFSTEATQWRPSLVPASAENVNK.A	4	6.02	0.46	-3.05
IPI00218192	Isoform 2 of Inter-alpha-trypsin inhibitor heavy chain H4 precursor	R.DTDRFSSHVGGTLGQFYQEVLWGSPAASDDGRR.T	5	3.34	0.21	-2.57
IPI00218192	Isoform 2 of Inter-alpha-trypsin inhibitor heavy chain H4 precursor	R.FAHTVVTSR.V	1	2.67	0.27	-4.67
IPI00218192	Isoform 2 of Inter-alpha-trypsin inhibitor heavy chain H4 precursor	R.FAHTVVTSR.V	2	2.70	0.32	-2.52
IPI00218192	Isoform 2 of Inter-alpha-trypsin inhibitor heavy chain H4 precursor	R.FKPTLSQQQK.S	2	2.83	0.27	-3.54
IPI00218192	Isoform 2 of Inter-alpha-trypsin inhibitor heavy chain H4 precursor	R.FKPTLSQQQK.S	3	3.30	0.14	-5.23
IPI00218192	Isoform 2 of Inter-alpha-trypsin inhibitor heavy chain H4 precursor	R.FSSHVGGTLGQFYQEVLWGSPAASDDGRR.T	3	3.72	0.39	-4.40
IPI00218192	Isoform 2 of Inter-alpha-trypsin inhibitor heavy chain H4 precursor	R.GPDVLTATVSGK.L	1	2.08	0.17	-3.32
IPI00218192	Isoform 2 of Inter-alpha-trypsin inhibitor heavy chain H4 precursor	R.GPDVLTATVSGK.L	2	3.79	0.42	-2.48
IPI00218192	Isoform 2 of Inter-alpha-trypsin inhibitor heavy chain H4 precursor	R.HRQGPVNLLSDPEQGVEVTGQYER.E	3	5.93	0.53	-1.50
IPI00218192	Isoform 2 of Inter-alpha-trypsin inhibitor heavy chain H4 precursor	R.HRQGPVNLLSDPEQGVEVTGQYER.E	4	4.28	0.39	-3.92
IPI00218192	Isoform 2 of Inter-alpha-trypsin inhibitor heavy chain H4 precursor	R.LGVYELLLK.V	2	3.56	0.37	-2.77
IPI00218192	Isoform 2 of Inter-alpha-trypsin inhibitor heavy chain H4 precursor	R.LPEGSVSLIILLTDGDPTVGETNPR.S	3	4.34	0.37	-2.85

IPI00218192	Isoform 2 of Inter-alpha-trypsin inhibitor heavy chain H4 precursor	R.LWAYLTIQQLLEQTVSASDADQQALR.N	3	5.64	0.45	-6.69
IPI00218192	Isoform 2 of Inter-alpha-trypsin inhibitor heavy chain H4 precursor	R.M*NFRPGVLSSR.L	2	1.63	0.05	-3.59
IPI00218192	Isoform 2 of Inter-alpha-trypsin inhibitor heavy chain H4 precursor	R.NM*EQFQVSVSVAPNAK.I	2	4.78	0.45	-2.79
IPI00218192	Isoform 2 of Inter-alpha-trypsin inhibitor heavy chain H4 precursor	R.QGPVNLLSDPEQGVEVTGQYER.E	2	4.85	0.50	-4.06
IPI00218192	Isoform 2 of Inter-alpha-trypsin inhibitor heavy chain H4 precursor	R.QGPVNLLSDPEQGVEVTGQYER.E	3	4.86	0.43	-4.91
IPI00218192	Isoform 2 of Inter-alpha-trypsin inhibitor heavy chain H4 precursor	R.QGPVNLLSDPEQGVEVTGQYEREK.A	3	3.33	0.24	-2.48
IPI00218192	Isoform 2 of Inter-alpha-trypsin inhibitor heavy chain H4 precursor	R.RLDYQEGPPGVEISCWSVEL.-	2	5.37	0.61	-4.23
IPI00218192	Isoform 2 of Inter-alpha-trypsin inhibitor heavy chain H4 precursor	R.RLDYQEGPPGVEISCWSVEL.-	3	4.45	0.40	-3.75
IPI00218192	Isoform 2 of Inter-alpha-trypsin inhibitor heavy chain H4 precursor	R.RLGVYELLLK.V	2	2.32	0.09	-1.65
IPI00218192	Isoform 2 of Inter-alpha-trypsin inhibitor heavy chain H4 precursor	R.RLGVYELLLK.V	3	3.85	0.23	-2.19
IPI00218192	Isoform 2 of Inter-alpha-trypsin inhibitor heavy chain H4 precursor	R.SFAAGIQALGGTNINDAM*LM*AVQLLDSSNQEER.L	3	3.68	0.28	-7.00
IPI00218192	Isoform 2 of Inter-alpha-trypsin inhibitor heavy chain H4 precursor	R.SFAAGIQALGGTNINDAM*LM*AVQLLDSSNQEER.L	4	5.25	0.47	-4.79
IPI00218192	Isoform 2 of Inter-alpha-trypsin inhibitor heavy chain H4 precursor	R.SIQNNVR.E	1	1.62	0.12	-4.71
IPI00218192	Isoform 2 of Inter-alpha-trypsin inhibitor heavy chain H4 precursor	W.VHASPEHVVVTR.N	2	3.07	0.42	-1.67
IPI00218292	Isoform Short of Ubiquitin fusion degradation protein 1 homolog	R.LNITYPM*LFK.L	2	2.33	0.25	-3.68
IPI00218319	Isoform 2 of Tropomyosin alpha-3 chain	K.IQVLQQQADDAEER.A	2	4.75	0.49	-4.03
IPI00218319	Isoform 2 of Tropomyosin alpha-3 chain	R.LATALQKLEEAEKAADESER.G	3	3.26	0.29	-3.58
IPI00218345	Isoform 2 of Tubulin alpha-3C/D chain	K.AYHEQLSVAEITNACFEPANQMVK.C	3	3.76	0.34	-2.56
IPI00218345	Isoform 2 of Tubulin alpha-3C/D chain	R.AVCMLSNTTAIAEAWAR.L	2	4.25	0.45	-1.86
IPI00218345	Isoform 2 of Tubulin alpha-3C/D chain	R.AVCMLSNTTAIAEAWAR.L	3	3.49	0.23	-0.91
IPI00218345	Isoform 2 of Tubulin alpha-3C/D chain	R.FDGALNVDLTEFQTNLVPYPR.I	2	5.18	0.51	-1.99
IPI00218345	Isoform 2 of Tubulin alpha-3C/D chain	R.FDGALNVDLTEFQTNLVPYPR.I	3	2.65	0.18	-2.07
IPI00218345	Isoform 2 of Tubulin alpha-3C/D chain	R.LIGQIVSSITASLR.F	2	3.90	0.47	-2.68
IPI00218345	Isoform 2 of Tubulin alpha-3C/D chain	R.LIGQIVSSITASLR.F	3	2.79	0.15	-0.10
IPI00218398	Matrix metalloproteinase-14 precursor	K.FYGLQVTGK.A	2	1.82	0.17	-2.15
IPI00218407	Fructose-bisphosphate aldolase B	K.VLAAVYK.A	1	2.13	0.18	-2.77
IPI00218413	biotinidase precursor	K.DAQEVHCDEATK.W	2	3.79	0.44	-2.50

IPI00218413	biotinidase precursor	K.EGYLHVCSNGLCCYLLYERPTLSK.E	3	3.64	0.32	-0.21
IPI00218413	biotinidase precursor	K.HVVYPTAWM*NQLPLLAAIEIQK.A	3	3.93	0.40	-6.14
IPI00218413	biotinidase precursor	K.NPVGLIGAENATGETDPSHSK.F	3	3.66	0.43	-3.05
IPI00218413	biotinidase precursor	K.SHLIIAQVAK.N	1	3.17	0.23	-3.88
IPI00218413	biotinidase precursor	K.SHLIIAQVAK.N	2	3.04	0.29	-4.69
IPI00218413	biotinidase precursor	K.SRLSSGLVTAALYGR.L	2	4.59	0.54	-3.34
IPI00218413	biotinidase precursor	K.VDLITFDTPFAGR.F	2	3.81	0.48	-3.62
IPI00218413	biotinidase precursor	R.GDM*FLVANLGTK.E	1	2.77	0.37	-1.96
IPI00218413	biotinidase precursor	R.GDM*FLVANLGTK.E	2	3.19	0.38	-0.30
IPI00218413	biotinidase precursor	R.LSCM*AIR.G	2	2.33	0.14	-2.46
IPI00218413	biotinidase precursor	R.LSSGLVTAALYGR.L	1	2.74	0.42	-2.25
IPI00218413	biotinidase precursor	R.LSSGLVTAALYGR.L	2	4.29	0.44	-4.09
IPI00218413	biotinidase precursor	R.QEALELM*NQNLDIYEQQVM*TAAQK.D	2	4.73	0.53	-3.50
IPI00218413	biotinidase precursor	R.QEALELM*NQNLDIYEQQVM*TAAQK.D	3	6.29	0.47	-4.83
IPI00218413	biotinidase precursor	R.TSIYPFLDFM*PSPQVVR.W	2	5.52	0.59	-5.75
IPI00218413	biotinidase precursor	R.TSIYPFLDFM*PSPQVVR.W	3	5.33	0.42	-1.77
IPI00218414	Carbonic anhydrase 2	K.AVQQPDGLAVLGIFLK.V	2	4.59	0.55	-3.97
IPI00218414	Carbonic anhydrase 2	K.AVQQPDGLAVLGIFLK.V	3	3.41	0.25	-1.98
IPI00218414	Carbonic anhydrase 2	K.VGSAKPGLQK.V	2	2.75	0.05	-1.69
IPI00218414	Carbonic anhydrase 2	K.VVDVLDSIK.T	2	2.60	0.23	-0.53
IPI00218414	Carbonic anhydrase 2	K.YDPSLKPLSVSYDQATSLR.I	2	3.58	0.48	-3.24
IPI00218414	Carbonic anhydrase 2	K.YDPSLKPLSVSYDQATSLR.I	3	4.89	0.48	-0.30
IPI00218465	Phospholipase A-2-activating protein	K.CMMTLQGHTAAVWAVKILPEQGLMLTGSADKTVK.L	3	3.47	0.10	
IPI00218474	Beta-enolase	K.ACNCLLLK.V	2	2.34	0.07	-0.79
IPI00218474	Beta-enolase	K.YNQLM*R.I	2	2.01	0.11	-2.49
IPI00218474	Beta-enolase	R.AAVPSGASTGIYEALELR.D	2	5.65	0.33	-8.46
IPI00218474	Beta-enolase	R.AAVPSGASTGIYEALELR.D	3	4.37	0.39	-3.26
IPI00218474	Beta-enolase	R.SGETEDTFIADLVVGLCTGQIK.T	2	5.97	0.60	-5.74
IPI00218474	Beta-enolase	R.SGETEDTFIADLVVGLCTGQIK.T	3	4.50	0.40	-3.18
IPI00218487	Gap junction alpha-1 protein	K.LAAGHELQPLAIVDQRPSSR.A	3	2.18	0.15	-3.82
IPI00218487	Gap junction alpha-1 protein	K.LAAGHELQPLAIVDQRPSSR.A	4	2.50	0.21	-3.73
IPI00218493	Hypoxanthine-guanine phosphoribosyltransferase	K.FFADLLDYIK.A	2	3.79	0.36	-2.53
IPI00218493	Hypoxanthine-guanine phosphoribosyltransferase	K.NVLIVEDIIDTGK.T	2	3.75	0.42	-2.52
IPI00218493	Hypoxanthine-guanine phosphoribosyltransferase	K.NVLIVEDIIDTGKTM*QTLLSLVRQYNPK.M	3	3.95	0.13	
IPI00218493	Hypoxanthine-guanine phosphoribosyltransferase	K.TM*QTLLSLVR.Q	2	3.51	0.35	-1.41
IPI00218493	Hypoxanthine-guanine phosphoribosyltransferase	K.VIGGDDLSTLTGK.N	2	3.30	0.38	-1.59
IPI00218493	Hypoxanthine-guanine phosphoribosyltransferase	R.SPGVVISDDEPGYDLDLFCIPNHYAEDLER.V	3	5.10	0.48	-4.19
IPI00218493	Hypoxanthine-guanine phosphoribosyltransferase	R.VFIPHGLIM*DR.T	3	1.82	0.22	-3.80
IPI00218539	Isoform B of Collagen alpha-1(XI) chain precursor	K.QLFPGGTFPEDFSILFTVKPK.K	3	3.25	0.32	-4.67
IPI00218539	Isoform B of Collagen alpha-1(XI) chain precursor	R.AIVDTNGITVFGTR.I	2	1.87	0.08	-4.84
IPI00218539	Isoform B of Collagen alpha-1(XI) chain precursor	R.ILDEEVFEGDIQQFLITGDPK.A	2	5.98	0.51	-3.70
IPI00218539	Isoform B of Collagen alpha-1(XI) chain precursor	R.ILDEEVFEGDIQQFLITGDPK.A	3	4.92	0.41	-4.32

IPI00218539	Isoform B of Collagen alpha-1(XI) chain precursor	R.SPVFLFEDHTGKPAPEDYPLFR.T	3	3.98	0.41	-4.64
IPI00218570	Phosphoglycerate mutase 2	K.AM*EAVAAQGK.A	2	2.35	0.13	-1.51
IPI00218570	Phosphoglycerate mutase 2	K.AM*EAVAAQGKA.K	1	2.15	0.23	-2.22
IPI00218570	Phosphoglycerate mutase 2	K.AM*EAVAAQGKA.K	2	3.45	0.34	-1.27
IPI00218570	Phosphoglycerate mutase 2	R.ALPFWNEEIVPQIK.A	2	2.58	0.31	-3.01
IPI00218570	Phosphoglycerate mutase 2	R.KAM*EAVAAQGKA.K	2	3.11	0.29	-0.79
IPI00218570	Phosphoglycerate mutase 2	R.VLIAAHGNSLR.G	1	2.78	0.36	-0.32
IPI00218570	Phosphoglycerate mutase 2	R.VLIAAHGNSLR.G	2	3.18	0.32	-2.03
IPI00218628	Isoform 2 of Integrin alpha-IIb precursor	K.ASVQLLVQDSLNPAVK.S	2	2.73	0.10	-5.37
IPI00218637	Major histocompatibility complex, class II, DQ beta 2	K.DFLVQFKGMCYFTNGTERVRGVARYIYNR.E	3	2.55	0.05	2.84
IPI00218667	Stathmin-2	K.DLSLEEIQK.K	1	2.55	0.10	-1.65
IPI00218667	Stathmin-2	K.DLSLEEIQK.K	2	2.76	0.11	-2.42
IPI00218667	Stathmin-2	K.DLSLEEIQKK.L	2	2.45	0.10	-1.69
IPI00218667	Stathmin-2	K.M*EQIKENR.E	2	1.83	0.05	-2.62
IPI00218725	laminin alpha 2 subunit isoform b precursor	K.ADDKIYFGGLPTLR.N	3	3.16	0.19	-1.21
IPI00218725	laminin alpha 2 subunit isoform b precursor	K.AEECYYDENVAR.R	2	3.97	0.50	-3.82
IPI00218725	laminin alpha 2 subunit isoform b precursor	K.AEQTILPLVDEALQHTTTK.G	2	4.34	0.50	-2.70
IPI00218725	laminin alpha 2 subunit isoform b precursor	K.AEQTILPLVDEALQHTTTK.G	3	2.61	0.20	-3.67
IPI00218725	laminin alpha 2 subunit isoform b precursor	K.FIDFLAIEM*R.K	2	3.26	0.36	-4.57
IPI00218725	laminin alpha 2 subunit isoform b precursor	K.IQDM*SGWYLTDLPGR.I	2	2.11	0.14	-2.62
IPI00218725	laminin alpha 2 subunit isoform b precursor	K.KGSYNNIVVNVK.T	2	2.91	0.14	-2.86
IPI00218725	laminin alpha 2 subunit isoform b precursor	K.LM*FHVDNGAGR.F	3	3.16	0.31	-4.83
IPI00218725	laminin alpha 2 subunit isoform b precursor	K.LPPM*SEELNDKIDDLSQEIKDR.K	4	3.05	0.26	-1.65
IPI00218725	laminin alpha 2 subunit isoform b precursor	K.LVEHVPGQPVR.N	3	3.35	0.19	-4.38
IPI00218725	laminin alpha 2 subunit isoform b precursor	K.M*DGM*GIEM*IDEK.L	2	3.84	0.45	-3.84
IPI00218725	laminin alpha 2 subunit isoform b precursor	K.NLEQEADRLIDK.L	2	3.26	0.09	-1.88
IPI00218725	laminin alpha 2 subunit isoform b precursor	K.TAVADNLLFYLGSAK.F	2	5.60	0.55	-3.10
IPI00218725	laminin alpha 2 subunit isoform b precursor	K.TGFGGVSCDR.C	2	2.96	0.28	-0.71
IPI00218725	laminin alpha 2 subunit isoform b precursor	K.TNAVVKDPSK.N	2	2.29	0.18	-0.46
IPI00218725	laminin alpha 2 subunit isoform b precursor	K.VSVSSGGDCIR.T	2	3.42	0.25	-2.13
IPI00218725	laminin alpha 2 subunit isoform b precursor	K.VTADGEQTGQDAER.T	2	4.44	0.42	-3.56
IPI00218725	laminin alpha 2 subunit isoform b precursor	K.YYGDDPR.V	2	1.80	0.19	-3.38
IPI00218725	laminin alpha 2 subunit isoform b precursor	R.CALGYYGIVK.G	2	2.24	0.17	-2.14
IPI00218725	laminin alpha 2 subunit isoform b precursor	R.EETGFSTYNPQVIIR.G	2	4.15	0.42	1.30
IPI00218725	laminin alpha 2 subunit isoform b precursor	R.INHADFATVQLR.N	2	3.31	0.40	-4.73
IPI00218725	laminin alpha 2 subunit isoform b precursor	R.ISEISM*EVAEQGR.G	2	4.22	0.39	-2.06
IPI00218725	laminin alpha 2 subunit isoform b precursor	R.LADEINSIIDYVEDIQTK.L	3	4.54	0.30	-3.42
IPI00218725	laminin alpha 2 subunit isoform b precursor	R.NSHIAIAFDDTK.V	3	3.11	0.07	-3.64
IPI00218725	laminin alpha 2 subunit isoform b precursor	R.NSHIAIAFDDTKVK.N	2	3.64	0.52	-2.09
IPI00218725	laminin alpha 2 subunit isoform b precursor	R.NSHIAIAFDDTKVK.N	3	3.51	0.20	-1.01
IPI00218725	laminin alpha 2 subunit isoform b precursor	R.QALPHSYYWSAPAPYLGNK.L	3	2.79	0.08	-4.37

IPI00218725	laminin alpha 2 subunit isoform b precursor	R.SLGLICDGCPVGYTGPR.C	2	4.89	0.49	-3.74
IPI00218725	laminin alpha 2 subunit isoform b precursor	R.TFSSSALLM*YLATR.D	2	2.50	0.07	-3.28
IPI00218725	laminin alpha 2 subunit isoform b precursor	R.TPYNILSSPDYVGVTK.G	2	4.74	0.37	-3.93
IPI00218725	laminin alpha 2 subunit isoform b precursor	R.TWVTLKAEQTILPLVDEALQHTTTK.G	4	2.63	0.13	-3.17
IPI00218725	laminin alpha 2 subunit isoform b precursor	R.VAPQQDDLDSPQQISISNAEAR.Q	2	5.64	0.57	-3.65
IPI00218725	laminin alpha 2 subunit isoform b precursor	R.VAPQQDDLDSPQQISISNAEAR.Q	3	4.04	0.32	-3.91
IPI00218730	Rod cGMP-specific 3',5'-cyclic phosphodiesterase subunit alpha	R.KEWKALADEYDAKM*KVQEEKK.Q	3	4.08	0.06	
IPI00218732	Serum paraoxonase/arylesterase 1	K.FDVSSFNPHGISTFTDEDNAM*YLLVVNHPDAK.S	4	2.99	0.15	-0.38
IPI00218732	Serum paraoxonase/arylesterase 1	K.FQEEEKSLLHLK.T	2	3.87	0.21	
IPI00218732	Serum paraoxonase/arylesterase 1	K.FQEEEKSLLHLK.T	3	3.31	0.14	
IPI00218732	Serum paraoxonase/arylesterase 1	K.GIETGSEDM*EILPNGLAFISSGLKYPGIK.S	3	6.56	0.56	
IPI00218732	Serum paraoxonase/arylesterase 1	K.IFFYDSENPPASEVLR.I	2	5.55	0.42	
IPI00218732	Serum paraoxonase/arylesterase 1	K.IFFYDSENPPASEVLR.I	3	4.10	0.17	
IPI00218732	Serum paraoxonase/arylesterase 1	K.ILLM*DLNEEDPTVLELGITGSK.F	2	5.33	0.50	-3.35
IPI00218732	Serum paraoxonase/arylesterase 1	K.ILLM*DLNEEDPTVLELGITGSK.F	3	4.92	0.33	-3.26
IPI00218732	Serum paraoxonase/arylesterase 1	K.SFNPNSPGK.I	1	1.71	0.13	-4.00
IPI00218732	Serum paraoxonase/arylesterase 1	K.SFNPNSPGK.I	2	1.93	0.15	-0.70
IPI00218732	Serum paraoxonase/arylesterase 1	K.SLDFNTLVDNISVDPETGDLWVGCHPNGM*K.I	3	5.89	0.49	-4.62
IPI00218732	Serum paraoxonase/arylesterase 1	K.STVELFKFQEEEK.S	2	3.03	0.16	
IPI00218732	Serum paraoxonase/arylesterase 1	K.STVELFKFQEEEKSLLHLK.T	3	4.17	0.29	
IPI00218732	Serum paraoxonase/arylesterase 1	K.YVYIAELLAHK.I	2	2.99	0.32	
IPI00218732	Serum paraoxonase/arylesterase 1	K.YVYIAELLAHK.I	3	2.79	0.26	
IPI00218732	Serum paraoxonase/arylesterase 1	R.EVQPVELPNCNLVK.G	2	3.01	0.10	
IPI00218732	Serum paraoxonase/arylesterase 1	R.IQNILTEEPK.V	2	3.92	0.25	-3.01
IPI00218732	Serum paraoxonase/arylesterase 1	R.LNALREVQPVELPNCNLVK.G	2	5.48	0.31	
IPI00218732	Serum paraoxonase/arylesterase 1	R.LNALREVQPVELPNCNLVK.G	3	4.89	0.24	
IPI00218733	Superoxide dismutase	A.DDLGKGGNEESTK.T	2	3.42	0.32	-2.49
IPI00218733	Superoxide dismutase	K.ADDLGKGGNEESTK.T	2	3.85	0.41	-4.59
IPI00218733	Superoxide dismutase	K.ADDLGKGGNEESTK.T	3	2.06	0.18	-1.73
IPI00218733	Superoxide dismutase	K.ADDLGKGGNEESTKTGNAGSR.L	2	5.70	0.63	-4.17
IPI00218733	Superoxide dismutase	K.ADDLGKGGNEESTKTGNAGSR.L	3	4.47	0.38	-3.59
IPI00218733	Superoxide dismutase	K.ADDLGKGGNEESTKTGNAGSR.L	4	3.90	0.35	-1.89
IPI00218733	Superoxide dismutase	K.AVCVLKGDGPVQGIINFEQK.E	2	5.26	0.55	-2.59
IPI00218733	Superoxide dismutase	K.AVCVLKGDGPVQGIINFEQK.E	3	5.73	0.50	-4.55
IPI00218733	Superoxide dismutase	K.AVCVLKGDGPVQGIINFEQKES.N	2	5.22	0.57	-3.68
IPI00218733	Superoxide dismutase	K.AVCVLKGDGPVQGIINFEQKES.N	3	4.42	0.52	-4.05
IPI00218733	Superoxide dismutase	K.AVCVLKGDGPVQGIINFEQKESNGPVK.V	3	5.31	0.44	-3.24
IPI00218733	Superoxide dismutase	K.AVCVLKGDGPVQGIINFEQKESNGPVK.V	4	3.62	0.23	-0.13
IPI00218733	Superoxide dismutase	K.AVCVLKGDGPVQGIINFEQKESNGPVKVWGSIK.G	4	3.97	0.13	-4.81
IPI00218733	Superoxide dismutase	K.DGVADVSIEDSVISLSGDHCIIGR.T	3	2.87	0.20	-2.14
IPI00218733	Superoxide dismutase	K.GDGPVQGIINFEQK.E	1	2.18	0.41	-3.95

IPI00218733	Superoxide dismutase	K.GDGPVQGIINFEQK.E	2	4.19	0.44	-5.44
IPI00218733	Superoxide dismutase	K.GDGPVQGIINFEQK.E	3	4.39	0.31	-1.63
IPI00218733	Superoxide dismutase	K.GDGPVQGIINFEQKES.N	2	4.31	0.35	-3.91
IPI00218733	Superoxide dismutase	K.GDGPVQGIINFEQKESNGPVK.V	2	2.99	0.29	2.26
IPI00218733	Superoxide dismutase	K.GDGPVQGIINFEQKESNGPVK.V	3	3.07	0.25	-3.31
IPI00218733	Superoxide dismutase	K.GLTEGLHGFHVHEFGDN.T	2	3.42	0.48	-3.06
IPI00218733	Superoxide dismutase	K.TGNAGSRLACGVIGIAQ.-	2	2.91	0.23	-3.30
IPI00218733	Superoxide dismutase	R.HVGDLGNVTADK.D	2	3.82	0.40	-2.58
IPI00218733	Superoxide dismutase	R.HVGDLGNVTADKDGVADVSIEDSVISLSGDH.C	3	5.00	0.48	-2.39
IPI00218733	Superoxide dismutase	R.HVGDLGNVTADKDGVADVSIEDSVISLSGDHCIIGR.T	3	6.19	0.57	-2.85
IPI00218733	Superoxide dismutase	R.HVGDLGNVTADKDGVADVSIEDSVISLSGDHCIIGR.T	4	6.17	0.54	-6.66
IPI00218733	Superoxide dismutase	R.HVGDLGNVTADKDGVADVSIEDSVISLSGDHCIIGR.T	5	3.28	0.22	-3.31
IPI00218733	Superoxide dismutase	R.LACGVIGIAQ.-	2	2.16	0.10	-2.96
IPI00218733	Superoxide dismutase	R.TLVVHEK.A	1	1.73	0.22	-3.95
IPI00218733	Superoxide dismutase	R.TLVVHEK.A	2	2.23	0.21	-3.52
IPI00218733	Superoxide dismutase	R.TLVVHEKADDLGK.G	2	4.07	0.46	-2.86
IPI00218733	Superoxide dismutase	R.TLVVHEKADDLGK.G	3	1.96	0.19	-2.48
IPI00218733	Superoxide dismutase	R.TLVVHEKADDLGKGGNEESTK.T	2	6.21	0.54	-2.17
IPI00218733	Superoxide dismutase	R.TLVVHEKADDLGKGGNEESTK.T	3	5.59	0.55	-2.26
IPI00218733	Superoxide dismutase	R.TLVVHEKADDLGKGGNEESTK.T	4	2.68	0.25	-2.07
IPI00218733	Superoxide dismutase	R.TLVVHEKADDLGKGGNEESTKTGNAGSR.L	3	5.76	0.47	-4.36
IPI00218733	Superoxide dismutase	R.TLVVHEKADDLGKGGNEESTKTGNAGSR.L	4	4.67	0.43	-3.43
IPI00218795	L-selectin precursor	K.AEIEYLEK.T	2	2.30	0.08	-0.39
IPI00218795	L-selectin precursor	R.SYYWIGIR.K	2	2.69	0.22	-1.50
IPI00218820	Isoform 3 of Tropomyosin beta chain	R.LATALQKLEEAEKAADESER.G	3	3.26	0.29	-3.58
IPI00218834	Low affinity immunoglobulin gamma Fc region receptor III-A precursor	K.AVVFLEPQWYR.V	2	3.48	0.36	-4.12
IPI00218834	Low affinity immunoglobulin gamma Fc region receptor III-A precursor	K.DSGSYFCR.G	2	1.98	0.05	-2.95
IPI00218834	Low affinity immunoglobulin gamma Fc region receptor III-A precursor	K.VTYLQNGK.D	2	2.29	0.12	-0.35
IPI00218834	Low affinity immunoglobulin gamma Fc region receptor III-A precursor	S.VLEKDSVTLK.C	2	3.52	0.36	-3.30
IPI00218874	Isoform B of Osteopontin precursor	A.IPVKQADSGSSEEK.Q	2	4.30	0.41	-3.26
IPI00218874	Isoform B of Osteopontin precursor	A.IPVKQADSGSSEEK.Q	3	3.57	0.39	-1.59
IPI00218874	Isoform B of Osteopontin precursor	A.NDESNEHSDVIDSQELSK.V	2	5.40	0.44	-3.67
IPI00218874	Isoform B of Osteopontin precursor	A.NDESNEHSDVIDSQELSK.V	3	3.92	0.45	-2.77
IPI00218874	Isoform B of Osteopontin precursor	H.SDVIDSQELSK.V	2	2.94	0.30	-1.60
IPI00218874	Isoform B of Osteopontin precursor	I.PVAQDLNAPSDWDSR.G	2	4.56	0.45	-2.57
IPI00218874	Isoform B of Osteopontin precursor	I.PVKQADSGSSEEK.Q	2	3.25	0.38	-1.07
IPI00218874	Isoform B of Osteopontin precursor	K.AIPVAQDLNAPSD.W	2	3.80	0.53	-3.31
IPI00218874	Isoform B of Osteopontin precursor	K.AIPVAQDLNAPSDWDSR.G	2	5.21	0.53	-4.53

IPI00218874	Isoform B of Osteopontin precursor	K.AIPVAQDLNAPSDWDSR.G	3	4.02	0.40	-3.55
IPI00218874	Isoform B of Osteopontin precursor	K.ANDESNEHSDVIDSQELSK.V	2	5.61	0.55	-3.29
IPI00218874	Isoform B of Osteopontin precursor	K.ANDESNEHSDVIDSQELSK.V	3	4.89	0.34	-3.60
IPI00218874	Isoform B of Osteopontin precursor	K.DSYETSQLDDQSAETHSHK.Q	2	5.84	0.65	-2.86
IPI00218874	Isoform B of Osteopontin precursor	K.DSYETSQLDDQSAETHSHK.Q	3	2.98	0.25	-1.85
IPI00218874	Isoform B of Osteopontin precursor	K.DSYETSQLDDQSAETHSHK.Q	4	3.22	0.27	-0.90
IPI00218874	Isoform B of Osteopontin precursor	K.DSYETSQLDDQSAETHSHKQS.R	2	5.31	0.57	-3.55
IPI00218874	Isoform B of Osteopontin precursor	K.DSYETSQLDDQSAETHSHKQSR.L	3	3.78	0.44	-4.42
IPI00218874	Isoform B of Osteopontin precursor	K.FRISHELDSASSE.V	2	3.47	0.35	-1.77
IPI00218874	Isoform B of Osteopontin precursor	K.FRISHELDSASSEVN.-	2	4.27	0.49	-4.28
IPI00218874	Isoform B of Osteopontin precursor	K.FRRPDIQYPDATDEDITSHM*ESEELNGAYK.A	4	4.32	0.48	-2.71
IPI00218874	Isoform B of Osteopontin precursor	K.QLYNKYPDAVATWLNPDPSQK.Q	3	3.40	0.26	-3.78
IPI00218874	Isoform B of Osteopontin precursor	K.QLYNKYPDAVATWLNPDPSQKQN.L	3	4.15	0.31	-2.77
IPI00218874	Isoform B of Osteopontin precursor	K.RKANDESNEHSDVIDSQELSK.V	3	4.24	0.40	-4.10
IPI00218874	Isoform B of Osteopontin precursor	K.RKANDESNEHSDVIDSQELSK.V	4	3.58	0.47	-2.79
IPI00218874	Isoform B of Osteopontin precursor	K.SKEEDKHLKF.R	2	2.90	0.27	-3.89
IPI00218874	Isoform B of Osteopontin precursor	L.DDQSAETHSHK.Q	2	3.41	0.34	-3.47
IPI00218874	Isoform B of Osteopontin precursor	N.DESNEHSDVIDSQELSK.V	2	5.48	0.49	-3.53
IPI00218874	Isoform B of Osteopontin precursor	N.EHSDVIDSQELSK.V	2	4.20	0.39	-4.84
IPI00218874	Isoform B of Osteopontin precursor	P.VAQDLNAPSDWDSR.G	2	4.52	0.49	-2.52
IPI00218874	Isoform B of Osteopontin precursor	R.EFHSHEFHSHED.M	2	2.99	0.46	-4.79
IPI00218874	Isoform B of Osteopontin precursor	R.EFHSHEFHSHEDM*LVVDPK.S	2	2.30	0.41	-3.82
IPI00218874	Isoform B of Osteopontin precursor	R.EFHSHEFHSHEDM*LVVDPK.S	3	2.79	0.21	-4.47
IPI00218874	Isoform B of Osteopontin precursor	R.EFHSHEFHSHEDM*LVVDPK.S	4	2.23	0.11	-2.53
IPI00218874	Isoform B of Osteopontin precursor	R.GDSVVYGLR.S	1	1.96	0.31	-2.54
IPI00218874	Isoform B of Osteopontin precursor	R.GDSVVYGLR.S	2	3.38	0.24	-3.18
IPI00218874	Isoform B of Osteopontin precursor	R.GKDSYETSQLDDQSAETH.S	3	3.98	0.35	-1.82
IPI00218874	Isoform B of Osteopontin precursor	R.GKDSYETSQLDDQSAETHS.H	2	6.10	0.61	-1.71
IPI00218874	Isoform B of Osteopontin precursor	R.GKDSYETSQLDDQSAETHS.H	3	3.58	0.40	-2.88
IPI00218874	Isoform B of Osteopontin precursor	R.GKDSYETSQLDDQSAETHSH.K	3	5.23	0.50	-2.21
IPI00218874	Isoform B of Osteopontin precursor	R.GKDSYETSQLDDQSAETHSHK.Q	2	6.71	0.52	-2.98
IPI00218874	Isoform B of Osteopontin precursor	R.GKDSYETSQLDDQSAETHSHK.Q	3	6.02	0.59	-4.98
IPI00218874	Isoform B of Osteopontin precursor	R.GKDSYETSQLDDQSAETHSHK.Q	4	3.89	0.25	-2.95
IPI00218874	Isoform B of Osteopontin precursor	R.GKDSYETSQLDDQSAETHSHK.Q	5	2.86	0.32	0.25
IPI00218874	Isoform B of Osteopontin precursor	R.GKDSYETSQLDDQSAETHSHKQ.S	2	5.03	0.53	-3.50
IPI00218874	Isoform B of Osteopontin precursor	R.GKDSYETSQLDDQSAETHSHKQ.S	3	5.41	0.55	-3.11
IPI00218874	Isoform B of Osteopontin precursor	R.GKDSYETSQLDDQSAETHSHKQS.R	2	6.09	0.58	-3.46
IPI00218874	Isoform B of Osteopontin precursor	R.GKDSYETSQLDDQSAETHSHKQS.R	3	6.61	0.56	-4.74
IPI00218874	Isoform B of Osteopontin precursor	R.ISHELDSASS.E	1	2.13	0.46	-2.76
IPI00218874	Isoform B of Osteopontin precursor	R.ISHELDSASSE.V	1	2.80	0.42	-4.07
IPI00218874	Isoform B of Osteopontin precursor	R.ISHELDSASSE.V	2	3.11	0.28	-2.99
IPI00218874	Isoform B of Osteopontin precursor	R.ISHELDSASSEVN.-	1	4.12	0.49	-4.10

IPI00218874	Isoform B of Osteopontin precursor	R.ISHELDSASSEVN.-	2	3.69	0.44	-4.48
IPI00218874	Isoform B of Osteopontin precursor	R.KANDESNEHSDVIDSQELSK.V	2	5.74	0.57	-5.59
IPI00218874	Isoform B of Osteopontin precursor	R.KANDESNEHSDVIDSQELSK.V	3	6.60	0.56	-4.99
IPI00218874	Isoform B of Osteopontin precursor	R.RPDIQYPDATDEDITSHM*ESEE.L	3	4.37	0.38	-3.52
IPI00218874	Isoform B of Osteopontin precursor	R.RPDIQYPDATDEDITSHM*ESEELNGAYK.A	3	6.46	0.61	-2.73
IPI00218874	Isoform B of Osteopontin precursor	R.RPDIQYPDATDEDITSHM*ESEELNGAYK.A	4	5.82	0.51	-2.59
IPI00218874	Isoform B of Osteopontin precursor	S.DVIDSQELSK.V	2	3.12	0.30	-2.68
IPI00218874	Isoform B of Osteopontin precursor	T.SQLDDQSAETHSHK.Q	2	3.02	0.30	-2.21
IPI00218874	Isoform B of Osteopontin precursor	V.AQDLNAPSDWDSR.G	2	3.84	0.43	-4.37
IPI00218875	Isoform C of Osteopontin precursor	A.IPVKQADSGSSEEK.Q	2	4.30	0.41	-3.26
IPI00218875	Isoform C of Osteopontin precursor	A.IPVKQADSGSSEEK.Q	3	3.57	0.39	-1.59
IPI00218875	Isoform C of Osteopontin precursor	A.IPVKQADSGSSEEKQNAVSSEETNDFKQETLPSK.S	4	5.26	0.51	-4.49
IPI00218875	Isoform C of Osteopontin precursor	A.NDESNEHSDVIDSQELSK.V	2	5.40	0.44	-3.67
IPI00218875	Isoform C of Osteopontin precursor	A.NDESNEHSDVIDSQELSK.V	3	3.92	0.45	-2.77
IPI00218875	Isoform C of Osteopontin precursor	A.VSSEETNDFKQETLPSK.S	2	4.68	0.37	-1.91
IPI00218875	Isoform C of Osteopontin precursor	H.SDVIDSQELSK.V	2	2.94	0.30	-1.60
IPI00218875	Isoform C of Osteopontin precursor	I.PVAQDLNAPSDWDSR.G	2	4.56	0.45	-2.57
IPI00218875	Isoform C of Osteopontin precursor	I.PVKQADSGSSEEK.Q	2	3.25	0.38	-1.07
IPI00218875	Isoform C of Osteopontin precursor	K.AIPVAQDLNAPSD.W	2	3.80	0.53	-3.31
IPI00218875	Isoform C of Osteopontin precursor	K.AIPVAQDLNAPSDWDSR.G	2	5.21	0.53	-4.53
IPI00218875	Isoform C of Osteopontin precursor	K.AIPVAQDLNAPSDWDSR.G	3	4.02	0.40	-3.55
IPI00218875	Isoform C of Osteopontin precursor	K.ANDESNEHSDVIDSQELSK.V	2	5.61	0.55	-3.29
IPI00218875	Isoform C of Osteopontin precursor	K.ANDESNEHSDVIDSQELSK.V	3	4.89	0.34	-3.60
IPI00218875	Isoform C of Osteopontin precursor	K.DSYETSQLDDQSAETHSHK.Q	2	5.84	0.65	-2.86
IPI00218875	Isoform C of Osteopontin precursor	K.DSYETSQLDDQSAETHSHK.Q	3	2.98	0.25	-1.85
IPI00218875	Isoform C of Osteopontin precursor	K.DSYETSQLDDQSAETHSHK.Q	4	3.22	0.27	-0.90
IPI00218875	Isoform C of Osteopontin precursor	K.DSYETSQLDDQSAETHSHKQS.R	2	5.31	0.57	-3.55
IPI00218875	Isoform C of Osteopontin precursor	K.DSYETSQLDDQSAETHSHKQSR.L	3	3.78	0.44	-4.42
IPI00218875	Isoform C of Osteopontin precursor	K.FRISHELDSASSE.V	2	3.47	0.35	-1.77
IPI00218875	Isoform C of Osteopontin precursor	K.FRISHELDSASSEVN.-	2	4.27	0.49	-4.28
IPI00218875	Isoform C of Osteopontin precursor	K.FRRPDIQYPDATDEDITSHM*ESEELNGAYK.A	4	4.32	0.48	-2.71
IPI00218875	Isoform C of Osteopontin precursor	K.QADSGSSEEKQNAVSSEETNDFKQETLPSK.S	3	4.09	0.38	-4.51
IPI00218875	Isoform C of Osteopontin precursor	K.QADSGSSEEKQNAVSSEETNDFKQETLPSK.S	4	3.36	0.28	-3.02
IPI00218875	Isoform C of Osteopontin precursor	K.RKANDESNEHSDVIDSQELSK.V	3	4.24	0.40	-4.10
IPI00218875	Isoform C of Osteopontin precursor	K.RKANDESNEHSDVIDSQELSK.V	4	3.58	0.47	-2.79
IPI00218875	Isoform C of Osteopontin precursor	K.SKEEDKHLKF.R	2	2.90	0.27	-3.89
IPI00218875	Isoform C of Osteopontin precursor	L.DDQSAETHSHK.Q	2	3.41	0.34	-3.47
IPI00218875	Isoform C of Osteopontin precursor	N.AVSSEETNDFKQETLPSK.S	2	4.70	0.48	-2.49
IPI00218875	Isoform C of Osteopontin precursor	N.AVSSEETNDFKQETLPSK.S	3	3.68	0.28	-1.89
IPI00218875	Isoform C of Osteopontin precursor	N.DESNEHSDVIDSQELSK.V	2	5.48	0.49	-3.53
IPI00218875	Isoform C of Osteopontin precursor	N.EHSDVIDSQELSK.V	2	4.20	0.39	-4.84
IPI00218875	Isoform C of Osteopontin precursor	P.QNAVSSEETNDFKQETLPSK.S	2	4.36	0.41	-1.71

IPI00218875	Isoform C of Osteopontin precursor	P.VAQDLNAPSDWDSR.G	2	4.52	0.49	-2.52
IPI00218875	Isoform C of Osteopontin precursor	R.EFHSHEFHSHED.M	2	2.99	0.46	-4.79
IPI00218875	Isoform C of Osteopontin precursor	R.EFHSHEFHSHEDM*LVVDPK.S	2	2.30	0.41	-3.82
IPI00218875	Isoform C of Osteopontin precursor	R.EFHSHEFHSHEDM*LVVDPK.S	3	2.79	0.21	-4.47
IPI00218875	Isoform C of Osteopontin precursor	R.EFHSHEFHSHEDM*LVVDPK.S	4	2.23	0.11	-2.53
IPI00218875	Isoform C of Osteopontin precursor	R.GDSVVYGLR.S	1	1.96	0.31	-2.54
IPI00218875	Isoform C of Osteopontin precursor	R.GDSVVYGLR.S	2	3.38	0.24	-3.18
IPI00218875	Isoform C of Osteopontin precursor	R.GKDSYETSQLDDQSAETH.S	3	3.98	0.35	-1.82
IPI00218875	Isoform C of Osteopontin precursor	R.GKDSYETSQLDDQSAETHS.H	2	6.10	0.61	-1.71
IPI00218875	Isoform C of Osteopontin precursor	R.GKDSYETSQLDDQSAETHS.H	3	3.58	0.40	-2.88
IPI00218875	Isoform C of Osteopontin precursor	R.GKDSYETSQLDDQSAETHSH.K	3	5.23	0.50	-2.21
IPI00218875	Isoform C of Osteopontin precursor	R.GKDSYETSQLDDQSAETHSHK.Q	2	6.71	0.52	-2.98
IPI00218875	Isoform C of Osteopontin precursor	R.GKDSYETSQLDDQSAETHSHK.Q	3	6.02	0.59	-4.98
IPI00218875	Isoform C of Osteopontin precursor	R.GKDSYETSQLDDQSAETHSHK.Q	4	3.89	0.25	-2.95
IPI00218875	Isoform C of Osteopontin precursor	R.GKDSYETSQLDDQSAETHSHK.Q	5	2.86	0.32	0.25
IPI00218875	Isoform C of Osteopontin precursor	R.GKDSYETSQLDDQSAETHSHKQ.S	2	5.03	0.53	-3.50
IPI00218875	Isoform C of Osteopontin precursor	R.GKDSYETSQLDDQSAETHSHKQ.S	3	5.41	0.55	-3.11
IPI00218875	Isoform C of Osteopontin precursor	R.GKDSYETSQLDDQSAETHSHKQS.R	2	6.09	0.58	-3.46
IPI00218875	Isoform C of Osteopontin precursor	R.GKDSYETSQLDDQSAETHSHKQS.R	3	6.61	0.56	-4.74
IPI00218875	Isoform C of Osteopontin precursor	R.ISHELDSASS.E	1	2.13	0.46	-2.76
IPI00218875	Isoform C of Osteopontin precursor	R.ISHELDSASSE.V	1	2.80	0.42	-4.07
IPI00218875	Isoform C of Osteopontin precursor	R.ISHELDSASSE.V	2	3.11	0.28	-2.99
IPI00218875	Isoform C of Osteopontin precursor	R.ISHELDSASSEVN.-	1	4.12	0.49	-4.10
IPI00218875	Isoform C of Osteopontin precursor	R.ISHELDSASSEVN.-	2	3.69	0.44	-4.48
IPI00218875	Isoform C of Osteopontin precursor	R.KANDESNEHSDVIDSQELSK.V	2	5.74	0.57	-5.59
IPI00218875	Isoform C of Osteopontin precursor	R.KANDESNEHSDVIDSQELSK.V	3	6.60	0.56	-4.99
IPI00218875	Isoform C of Osteopontin precursor	R.RPDIQYPDATDEDITSHM*ESEE.L	3	4.37	0.38	-3.52
IPI00218875	Isoform C of Osteopontin precursor	R.RPDIQYPDATDEDITSHM*ESEELNGAYK.A	3	6.46	0.61	-2.73
IPI00218875	Isoform C of Osteopontin precursor	R.RPDIQYPDATDEDITSHM*ESEELNGAYK.A	4	5.82	0.51	-2.59
IPI00218875	Isoform C of Osteopontin precursor	S.DVIDSQELSK.V	2	3.12	0.30	-2.68
IPI00218875	Isoform C of Osteopontin precursor	T.SQLDDQSAETHSHK.Q	2	3.02	0.30	-2.21
IPI00218875	Isoform C of Osteopontin precursor	V.AQDLNAPSDWDSR.G	2	3.84	0.43	-4.37
IPI00218914	Retinal dehydrogenase 1	R.TIPIDGNFFTYTR.H	2	2.56	0.19	-4.75
IPI00218918	Annexin A1	K.DITSDTSGDFR.N	2	2.94	0.27	-0.97
IPI00218946	Potassium/sodium hyperpolarization-activated cyclic nucleotide-gated channel 2	R.DSASPGAAGGLDPQDSAR.S	2	4.58	0.37	-1.43
IPI00219005	FK506-binding protein 4	K.AEASSGDHPTDTEM*KEEQK.S	3	2.09	0.13	-4.01
IPI00219025	Glutaredoxin-1	K.QGLLEFVDITATNHTNEIQDYLQQLTGAR.T	3	4.36	0.40	-4.93
IPI00219025	Glutaredoxin-1	R.AQEILSQLPIK.Q	2	3.78	0.31	-2.52
IPI00219025	Glutaredoxin-1	R.LKQIGALQ.-	2	3.26	0.10	-1.20
IPI00219025	Glutaredoxin-1	R.RAQEILSQLPIK.Q	2	3.18	0.16	-2.35
IPI00219025	Glutaredoxin-1	R.RAQEILSQLPIK.Q	3	2.52	0.08	-0.34

IPI00219029	Aspartate aminotransferase, cytoplasmic	A.LGDDSPALK.E	2	3.18	0.24	-3.97
IPI00219029	Aspartate aminotransferase, cytoplasmic	K.HIYLLPSGR.I	1	2.09	0.06	-3.38
IPI00219029	Aspartate aminotransferase, cytoplasmic	K.HIYLLPSGR.I	2	2.62	0.35	-3.10
IPI00219029	Aspartate aminotransferase, cytoplasmic	K.IANDNSLNHEYLPILGLAEFR.S	2	5.69	0.60	-3.86
IPI00219029	Aspartate aminotransferase, cytoplasmic	K.IANDNSLNHEYLPILGLAEFR.S	3	5.26	0.38	-4.45
IPI00219029	Aspartate aminotransferase, cytoplasmic	K.LTADFREDPDPRK.V	3	2.39	0.07	-2.01
IPI00219029	Aspartate aminotransferase, cytoplasmic	K.NFGLYNER.V	1	2.16	0.07	-2.31
IPI00219029	Aspartate aminotransferase, cytoplasmic	K.NFGLYNER.V	2	2.46	0.06	-2.51
IPI00219029	Aspartate aminotransferase, cytoplasmic	K.NLDYVATSIHEAVTK.I	2	4.19	0.48	-2.82
IPI00219029	Aspartate aminotransferase, cytoplasmic	K.NLDYVATSIHEAVTK.I	3	2.50	0.27	-3.35
IPI00219029	Aspartate aminotransferase, cytoplasmic	K.NLDYVATSIHEAVTKIQ.-	3	3.04	0.31	-0.97
IPI00219029	Aspartate aminotransferase, cytoplasmic	K.QVEYLVNEK.H	1	2.70	0.18	-3.66
IPI00219029	Aspartate aminotransferase, cytoplasmic	K.QVEYLVNEK.H	2	1.91	0.19	-2.46
IPI00219029	Aspartate aminotransferase, cytoplasmic	K.QVEYLVNEKHIYLLPSGR.I	3	3.20	0.31	-3.38
IPI00219029	Aspartate aminotransferase, cytoplasmic	K.RVGGVQSLGGTGALR.I	2	4.18	0.46	-3.65
IPI00219029	Aspartate aminotransferase, cytoplasmic	K.RVGGVQSLGGTGALR.I	3	2.55	0.09	-1.84
IPI00219029	Aspartate aminotransferase, cytoplasmic	K.TPGTWNHITDQIGM*FSFTGLNPK.Q	3	3.68	0.34	-0.95
IPI00219029	Aspartate aminotransferase, cytoplasmic	K.VNLGVGAYR.T	1	1.70	0.19	-2.44
IPI00219029	Aspartate aminotransferase, cytoplasmic	K.VNLGVGAYR.T	2	3.14	0.33	-1.95
IPI00219029	Aspartate aminotransferase, cytoplasmic	M.APPSVFAEVPQAQPVLVFK.L	2	5.93	0.58	-4.21
IPI00219029	Aspartate aminotransferase, cytoplasmic	M.APPSVFAEVPQAQPVLVFK.L	3	5.05	0.39	-4.33
IPI00219029	Aspartate aminotransferase, cytoplasmic	R.FLFPFFDSAYQGFASGNLER.D	2	5.55	0.65	-5.83
IPI00219029	Aspartate aminotransferase, cytoplasmic	R.FLFPFFDSAYQGFASGNLER.D	3	5.85	0.47	-5.30
IPI00219029	Aspartate aminotransferase, cytoplasmic	R.IGADFLAR.W	2	2.95	0.27	-2.61
IPI00219029	Aspartate aminotransferase, cytoplasmic	R.INVSGLTTK.N	1	1.93	0.18	-3.76
IPI00219029	Aspartate aminotransferase, cytoplasmic	R.INVSGLTTK.N	2	2.85	0.15	-2.90
IPI00219029	Aspartate aminotransferase, cytoplasmic	R.ITWSNPPAQGAR.I	2	3.87	0.36	-1.27
IPI00219029	Aspartate aminotransferase, cytoplasmic	R.IVASTLSNPELFEEWTGNVK.T	2	5.83	0.62	-3.56
IPI00219029	Aspartate aminotransferase, cytoplasmic	R.IVASTLSNPELFEEWTGNVK.T	3	5.63	0.41	-3.69
IPI00219029	Aspartate aminotransferase, cytoplasmic	R.KVNLGVGAYR.T	2	2.68	0.32	-3.83
IPI00219029	Aspartate aminotransferase, cytoplasmic	R.LALGDDSPALK.E	1	2.50	0.24	-3.61
IPI00219029	Aspartate aminotransferase, cytoplasmic	R.LALGDDSPALK.E	2	3.10	0.35	-3.18
IPI00219029	Aspartate aminotransferase, cytoplasmic	R.TDDCHPWVLPVVK.K	3	2.99	0.32	-0.92
IPI00219029	Aspartate aminotransferase, cytoplasmic	R.VGGVQSLGGTGALR.I	1	2.03	0.21	-2.59
IPI00219029	Aspartate aminotransferase, cytoplasmic	R.VGGVQSLGGTGALR.I	2	4.06	0.42	-2.94
IPI00219029	Aspartate aminotransferase, cytoplasmic	R.VGNLTVVGK.E	2	2.83	0.19	-2.29
IPI00219029	Aspartate aminotransferase, cytoplasmic	R.VGNLTVVGKEPESILQ.V	2	4.22	0.36	-1.56
IPI00219029	Aspartate aminotransferase, cytoplasmic	R.VGNLTVVGKEPESILQV.L	2	3.76	0.42	-2.20
IPI00219029	Aspartate aminotransferase, cytoplasmic	R.VGNLTVVGKEPESILQVL.S	2	3.54	0.35	-2.09
IPI00219029	Aspartate aminotransferase, cytoplasmic	R.VGNLTVVGKEPESILQVLSQM*EK.I	2	5.19	0.52	-1.82
IPI00219029	Aspartate aminotransferase, cytoplasmic	R.VGNLTVVGKEPESILQVLSQM*EK.I	3	4.34	0.38	-2.46
IPI00219029	Aspartate aminotransferase, cytoplasmic	R.VGNLTVVGKEPESILQVLSQM*EK.I	4	3.19	0.15	-1.99

IPI00219029	Aspartate aminotransferase, cytoplasmic	R.VGNLTVVGKEPESILQVLSQM*EKIVR.I	4	3.02	0.26	-2.25
IPI00219029	Aspartate aminotransferase, cytoplasmic	W.ENHNAVFSAAGFK.D	2	2.97	0.32	-2.96
IPI00219042	Isoform 3 of Peptidyl-glycine alpha-amidating monooxygenase precursor	D.PNNKEGPVLILGR.S	3	3.59	0.20	-0.71
IPI00219042	Isoform 3 of Peptidyl-glycine alpha-amidating monooxygenase precursor	I.PVDEEAFVIDFKPR.A	3	5.08	0.47	-2.28
IPI00219042	Isoform 3 of Peptidyl-glycine alpha-amidating monooxygenase precursor	K.AGIEVQEIK.E	1	2.05	0.06	-3.46
IPI00219042	Isoform 3 of Peptidyl-glycine alpha-amidating monooxygenase precursor	K.AGIEVQEIK.E	2	2.87	0.11	-1.75
IPI00219042	Isoform 3 of Peptidyl-glycine alpha-amidating monooxygenase precursor	K.AGIEVQEIKDSEHK.L	2	3.31	0.14	-3.38
IPI00219042	Isoform 3 of Peptidyl-glycine alpha-amidating monooxygenase precursor	K.AGIEVQEIKDSEHKLETSSGR.V	3	4.78	0.51	-2.78
IPI00219042	Isoform 3 of Peptidyl-glycine alpha-amidating monooxygenase precursor	K.AGIEVQEIKDSEHKLETSSGR.V	4	2.81	0.19	-1.78
IPI00219042	Isoform 3 of Peptidyl-glycine alpha-amidating monooxygenase precursor	K.ANILYAWAR.N	2	3.66	0.33	-0.81
IPI00219042	Isoform 3 of Peptidyl-glycine alpha-amidating monooxygenase precursor	K.DGNYWVTDVALHQVFK.L	3	3.05	0.39	-3.20
IPI00219042	Isoform 3 of Peptidyl-glycine alpha-amidating monooxygenase precursor	K.EGPVLILGR.S	2	1.99	0.05	-0.91
IPI00219042	Isoform 3 of Peptidyl-glycine alpha-amidating monooxygenase precursor	K.ETEYKDKIPLLQQPK.R	2	4.24	0.38	-4.61
IPI00219042	Isoform 3 of Peptidyl-glycine alpha-amidating monooxygenase precursor	K.FITQWGEESSGSSPLPGQFTVPHSLALVPLLGQLCVADR.E	3	6.02	0.56	-2.67
IPI00219042	Isoform 3 of Peptidyl-glycine alpha-amidating monooxygenase precursor	K.FITQWGEESSGSSPLPGQFTVPHSLALVPLLGQLCVADR.E	4	4.41	0.36	-2.40
IPI00219042	Isoform 3 of Peptidyl-glycine alpha-amidating monooxygenase precursor	K.FTLTEKLEHR.S	2	3.00	0.31	-4.14
IPI00219042	Isoform 3 of Peptidyl-glycine alpha-amidating monooxygenase precursor	K.FTLTEKLEHR.S	3	2.73	0.27	-4.40
IPI00219042	Isoform 3 of Peptidyl-glycine alpha-amidating monooxygenase precursor	K.FVYQQIGLGPIEEDTILVIDPNNAAVLQSSGK.N	2	3.10	0.58	-2.93
IPI00219042	Isoform 3 of Peptidyl-glycine alpha-amidating monooxygenase precursor	K.FVYQQIGLGPIEEDTILVIDPNNAAVLQSSGK.N	3	7.50	0.62	-5.69
IPI00219042	Isoform 3 of Peptidyl-glycine alpha-amidating monooxygenase precursor	K.FVYQQIGLGPIEEDTILVIDPNNAAVLQSSGK.N	4	4.72	0.40	-3.52
IPI00219042	Isoform 3 of Peptidyl-glycine alpha-amidating monooxygenase precursor	K.GSGGLNLGNFFASR.K	2	4.19	0.40	-2.67
IPI00219042	Isoform 3 of Peptidyl-glycine alpha-amidating monooxygenase precursor	K.HAVSFM*TCTQNVAPDM*FR.T	2	5.15	0.58	-3.91

IPI00219042	Isoform 3 of Peptidyl-glycine alpha-amidating monooxygenase precursor	K.IPLLQQPK.R	2	2.50	0.13	-0.92
IPI00219042	Isoform 3 of Peptidyl-glycine alpha-amidating monooxygenase precursor	K.KAGIEVQEIK.E	2	2.77	0.18	-0.24
IPI00219042	Isoform 3 of Peptidyl-glycine alpha-amidating monooxygenase precursor	K.LDPNNKEGPVLILGR.S	2	3.26	0.32	-2.86
IPI00219042	Isoform 3 of Peptidyl-glycine alpha-amidating monooxygenase precursor	K.LDPNNKEGPVLILGR.S	3	4.10	0.41	-1.88
IPI00219042	Isoform 3 of Peptidyl-glycine alpha-amidating monooxygenase precursor	K.LLGEREDVVHVHK.Y	2	2.76	0.29	-2.89
IPI00219042	Isoform 3 of Peptidyl-glycine alpha-amidating monooxygenase precursor	K.LLGEREDVVHVHK.Y	3	3.30	0.38	-2.36
IPI00219042	Isoform 3 of Peptidyl-glycine alpha-amidating monooxygenase precursor	K.NLFYLPHGLSIDK.D	2	2.86	0.20	-2.77
IPI00219042	Isoform 3 of Peptidyl-glycine alpha-amidating monooxygenase precursor	K.NNLVIFHR.G	1	2.60	0.12	-0.99
IPI00219042	Isoform 3 of Peptidyl-glycine alpha-amidating monooxygenase precursor	K.NNLVIFHR.G	2	2.82	0.17	0.64
IPI00219042	Isoform 3 of Peptidyl-glycine alpha-amidating monooxygenase precursor	K.NYPM*HVFAYR.V	2	2.15	0.16	-0.94
IPI00219042	Isoform 3 of Peptidyl-glycine alpha-amidating monooxygenase precursor	K.QSDTYFCM*SM*R.I	2	2.86	0.44	-3.09
IPI00219042	Isoform 3 of Peptidyl-glycine alpha-amidating monooxygenase precursor	K.REEEEVLDQGDFYSLLSK.L	2	5.61	0.52	-5.66
IPI00219042	Isoform 3 of Peptidyl-glycine alpha-amidating monooxygenase precursor	K.REEEEVLDQGDFYSLLSK.L	3	5.34	0.38	-4.43
IPI00219042	Isoform 3 of Peptidyl-glycine alpha-amidating monooxygenase precursor	K.TDTKEFVR.E	1	2.09	0.23	-2.78
IPI00219042	Isoform 3 of Peptidyl-glycine alpha-amidating monooxygenase precursor	K.TDTKEFVR.E	2	2.18	0.19	-2.34
IPI00219042	Isoform 3 of Peptidyl-glycine alpha-amidating monooxygenase precursor	K.VVSGYR.V	1	1.17	0.08	-2.62
IPI00219042	Isoform 3 of Peptidyl-glycine alpha-amidating monooxygenase precursor	K.YNPTEKAESESDLVAEIANVVQ.K	2	4.87	0.46	-0.06
IPI00219042	Isoform 3 of Peptidyl-glycine alpha-amidating monooxygenase precursor	L.DPNNKEGPVLILGR.S	3	3.85	0.35	-0.73
IPI00219042	Isoform 3 of Peptidyl-glycine alpha-amidating monooxygenase precursor	P.QAFYPVGHPVDVSFGDLLAAR.C	3	3.88	0.39	-3.12
IPI00219042	Isoform 3 of Peptidyl-glycine alpha-amidating monooxygenase precursor	R.EDVVHVHK.Y	2	2.20	0.27	-2.42
IPI00219042	Isoform 3 of Peptidyl-glycine alpha-amidating monooxygenase precursor	R.EEEEVLDQGDFYSLLSK.L	2	5.52	0.53	-4.70

IPI00219042	Isoform 3 of Peptidyl-glycine alpha-amidating monooxygenase precursor	R.EEEEVLDQGDFYSLLSK.L	3	5.40	0.45	-3.25
IPI00219042	Isoform 3 of Peptidyl-glycine alpha-amidating monooxygenase precursor	R.EGAEHERGNAILVR.D	2	2.14	0.09	-2.81
IPI00219042	Isoform 3 of Peptidyl-glycine alpha-amidating monooxygenase precursor	R.GKGSGGLNLGNFFASR.K	2	5.47	0.55	-4.07
IPI00219042	Isoform 3 of Peptidyl-glycine alpha-amidating monooxygenase precursor	R.GKGSGGLNLGNFFASR.K	3	4.18	0.39	-2.26
IPI00219042	Isoform 3 of Peptidyl-glycine alpha-amidating monooxygenase precursor	R.IPVDEEAFVIDFKPR.A	2	4.95	0.56	-6.23
IPI00219042	Isoform 3 of Peptidyl-glycine alpha-amidating monooxygenase precursor	R.IPVDEEAFVIDFKPR.A	3	4.65	0.48	-4.22
IPI00219042	Isoform 3 of Peptidyl-glycine alpha-amidating monooxygenase precursor	R.IVQFSPSGK.F	1	2.27	0.17	-3.21
IPI00219042	Isoform 3 of Peptidyl-glycine alpha-amidating monooxygenase precursor	R.IVQFSPSGK.F	2	2.61	0.22	-2.07
IPI00219042	Isoform 3 of Peptidyl-glycine alpha-amidating monooxygenase precursor	R.LSTEGSDQEKEDDGSESEEEYSAPLPALAPSSS.-	3	3.29	0.39	-0.99
IPI00219042	Isoform 3 of Peptidyl-glycine alpha-amidating monooxygenase precursor	R.M*PGVTPK.Q	1	1.96	0.06	-2.26
IPI00219042	Isoform 3 of Peptidyl-glycine alpha-amidating monooxygenase precursor	R.NAPPTRLPK.G	2	2.59	0.14	-2.09
IPI00219042	Isoform 3 of Peptidyl-glycine alpha-amidating monooxygenase precursor	R.NGQWTLIGR.Q	1	2.01	0.11	-2.79
IPI00219042	Isoform 3 of Peptidyl-glycine alpha-amidating monooxygenase precursor	R.NGQWTLIGR.Q	2	3.26	0.29	-0.53
IPI00219042	Isoform 3 of Peptidyl-glycine alpha-amidating monooxygenase precursor	R.QSPQLPQAFYPVGHPVDVSFGDLLAAR.C	2	3.13	0.49	-6.57
IPI00219042	Isoform 3 of Peptidyl-glycine alpha-amidating monooxygenase precursor	R.QSPQLPQAFYPVGHPVDVSFGDLLAAR.C	3	4.67	0.54	-4.57
IPI00219042	Isoform 3 of Peptidyl-glycine alpha-amidating monooxygenase precursor	R.QSPQLPQAFYPVGHPVDVSFGDLLAAR.C	4	4.72	0.53	-3.46
IPI00219042	Isoform 3 of Peptidyl-glycine alpha-amidating monooxygenase precursor	R.SM*QPGSDQNHFCQPTDVAVDPGTGAIYVSDGYCNSR.I	3	6.06	0.62	-4.42
IPI00219042	Isoform 3 of Peptidyl-glycine alpha-amidating monooxygenase precursor	R.SM*QPGSDQNHFCQPTDVAVDPGTGAIYVSDGYCNSR.I	4	4.81	0.43	-5.02
IPI00219042	Isoform 3 of Peptidyl-glycine alpha-amidating monooxygenase precursor	R.TEATHIGGTSSDEM*CNLYIM*YYM*EAK.H	3	4.79	0.45	-3.46
IPI00219042	Isoform 3 of Peptidyl-glycine alpha-amidating monooxygenase precursor	W.PGVYLLPGQVSGVALDPK.N	2	4.54	0.42	-2.14
IPI00219067	Glutathione S-transferase Mu 2	K.ITQSNAILR.Y	2	2.69	0.08	-0.96
IPI00219067	Glutathione S-transferase Mu 2	K.LGLDFPNLPYLIDGTHK.I	3	3.63	0.45	-3.09

IPI00219077	Isoform 1 of Leukotriene A-4 hydrolase	K.DLAAFDKSHDQAVR.T	3	2.21	0.15	-1.97
IPI00219129	Ribosyldihydronicotinamide dehydrogenase	K.NVAVDELSR.Q	2	2.30	0.12	-2.60
IPI00219129	Ribosyldihydronicotinamide dehydrogenase	K.VLIVYAHQEPK.S	3	2.58	0.23	-3.74
IPI00219129	Ribosyldihydronicotinamide dehydrogenase	R.VLCQGFAFDIPGFYDSGLLQGK.L	2	4.48	0.35	0.23
IPI00219131	Isoform 1 of ICOS ligand precursor	R.ALM*SPAGM*LR.G	2	3.15	0.18	-0.18
IPI00219131	Isoform 1 of ICOS ligand precursor	R.AM*VGSDVELSCACPEGSRFDLNDVYVYWQTSESK.T	3	5.44	0.59	-2.89
IPI00219131	Isoform 1 of ICOS ligand precursor	R.DKITENPVSTGEK.N	2	3.55	0.28	-2.63
IPI00219131	Isoform 1 of ICOS ligand precursor	R.FDLNDVYVYWQTSESK.T	2	5.63	0.58	-4.84
IPI00219131	Isoform 1 of ICOS ligand precursor	R.FDLNDVYVYWQTSESK.T	3	4.90	0.39	-4.03
IPI00219131	Isoform 1 of ICOS ligand precursor	R.GLYDVVSVLR.I	1	2.55	0.29	-2.78
IPI00219131	Isoform 1 of ICOS ligand precursor	R.GLYDVVSVLR.I	2	4.02	0.34	-3.00
IPI00219217	L-lactate dehydrogenase B chain	K.EKLIAPVAEEEATVPNNK.I	2	4.43	0.46	-0.79
IPI00219217	L-lactate dehydrogenase B chain	K.GEM*M*DLQHGSLFLQTPK.I	2	2.54	0.19	-1.76
IPI00219217	L-lactate dehydrogenase B chain	K.GM*YGIENEVFLSLPCILNAR.G	2	5.20	0.52	-2.98
IPI00219217	L-lactate dehydrogenase B chain	K.GM*YGIENEVFLSLPCILNAR.G	3	3.30	0.30	-3.76
IPI00219217	L-lactate dehydrogenase B chain	K.ITVVGVGQVGM*ACAISILGK.S	2	5.60	0.54	-2.10
IPI00219217	L-lactate dehydrogenase B chain	K.ITVVGVGQVGMACAISILGK.S	2	3.93	0.45	-2.08
IPI00219217	L-lactate dehydrogenase B chain	K.ITVVGVGQVGMACAISILGK.S	3	4.26	0.52	-1.01
IPI00219217	L-lactate dehydrogenase B chain	K.IVADKDYSVTANSK.I	1	2.82	0.52	-1.85
IPI00219217	L-lactate dehydrogenase B chain	K.IVADKDYSVTANSK.I	2	4.66	0.54	-3.12
IPI00219217	L-lactate dehydrogenase B chain	K.IVADKDYSVTANSK.I	3	3.81	0.47	-2.48
IPI00219217	L-lactate dehydrogenase B chain	K.IVVVTAGVR.Q	2	3.32	0.36	-3.23
IPI00219217	L-lactate dehydrogenase B chain	K.LIAPVAEEEATVPNNK.I	2	3.70	0.41	-4.07
IPI00219217	L-lactate dehydrogenase B chain	K.LKDDEVAQLKK.S	2	3.92	0.29	-4.78
IPI00219217	L-lactate dehydrogenase B chain	K.LKDDEVAQLKK.S	3	4.37	0.25	-4.10
IPI00219217	L-lactate dehydrogenase B chain	K.M*VVESAYEVIK.L	2	3.49	0.30	-3.40
IPI00219217	L-lactate dehydrogenase B chain	K.SLADELALVDVLEDK.L	2	4.95	0.40	-4.43
IPI00219217	L-lactate dehydrogenase B chain	K.SLADELALVDVLEDK.L	3	3.11	0.23	-2.66
IPI00219217	L-lactate dehydrogenase B chain	K.SLADELALVDVLEDKLK.G	2	5.28	0.39	-4.21
IPI00219217	L-lactate dehydrogenase B chain	K.SLADELALVDVLEDKLK.G	3	2.41	0.21	-2.29
IPI00219217	L-lactate dehydrogenase B chain	K.SLADELALVDVLEDKLKGEM*M*DLQHGSLFLQTPK.I	3	5.29	0.40	-2.99
IPI00219217	L-lactate dehydrogenase B chain	K.SLADELALVDVLEDKLKGEM*M*DLQHGSLFLQTPK.I	4	3.90	0.37	-3.55
IPI00219217	L-lactate dehydrogenase B chain	R.NVNVFK.F	1	1.89	0.13	-1.68
IPI00219217	L-lactate dehydrogenase B chain	R.NVNVFKFIIPQIVK.Y	3	3.80	0.32	-3.54
IPI00219217	L-lactate dehydrogenase B chain	R.VIGSGCNLDSAR.F	1	2.49	0.27	-2.12
IPI00219217	L-lactate dehydrogenase B chain	R.VIGSGCNLDSAR.F	2	3.57	0.37	-2.86
IPI00219217	L-lactate dehydrogenase B chain	V.ADKDYSVTANSK.I	2	3.28	0.44	-2.13
IPI00219219	Galectin-1	K.LPDGYEFK.F	2	2.01	0.16	-2.55
IPI00219219	Galectin-1	R.FNAHGDANTIVCNSK.D	2	3.53	0.17	
IPI00219219	Galectin-1	R.LNLEAINYMAADGDFK.I	2	4.61	0.47	-2.83
IPI00219301	Myristoylated alanine-rich C-kinase substrate	K.AEDGATPSPSNETPK.K	2	3.01	0.25	-3.91
IPI00219301	Myristoylated alanine-rich C-kinase substrate	K.GEPAAAAAPEAGASPVEK.E	2	4.11	0.47	-4.06

IPI00219301	Myristoylated alanine-rich C-kinase substrate	K.LSGFSFK.K	1	1.55	0.06	-1.99
IPI00219301	Myristoylated alanine-rich C-kinase substrate	K.LSGFSFKK.N	2	2.34	0.10	-3.98
IPI00219365	Moesin	K.ALTSELANAR.D	2	3.19	0.25	-1.71
IPI00219365	Moesin	K.APDFVFYAPR.L	2	2.34	0.35	-2.52
IPI00219365	Moesin	K.ERQEAEEAKEALLQASR.D	3	4.14	0.40	-3.24
IPI00219365	Moesin	K.IAQDLEM*YGVNYFSIK.N	2	4.85	0.52	-1.79
IPI00219365	Moesin	K.IGFPWSEIR.N	2	2.60	0.06	-0.69
IPI00219365	Moesin	K.KTQEQLALEM*AELTAR.I	2	4.26	0.47	-2.72
IPI00219365	Moesin	K.KTQEQLALEM*AELTAR.I	3	3.40	0.31	-0.38
IPI00219365	Moesin	K.KVTAQDVR.K	2	1.94	0.12	-1.45
IPI00219365	Moesin	K.SGYLAGDK.L	2	2.26	0.12	-2.68
IPI00219365	Moesin	K.TANDM*IHAENM*R.L	2	2.27	0.18	-7.85
IPI00219365	Moesin	K.YGDFNKEVHK.S	2	3.34	0.24	-2.37
IPI00219365	Moesin	R.AKFYPEDVSEELIQDITQR.L	2	4.25	0.51	-2.75
IPI00219365	Moesin	R.AKFYPEDVSEELIQDITQR.L	3	3.64	0.26	-2.87
IPI00219365	Moesin	R.AQSEAEKLAK.E	2	2.23	0.08	-3.42
IPI00219365	Moesin	R.EKEELM*ER.L	2	1.98	0.15	-1.97
IPI00219365	Moesin	R.GM*LREDAVLEYLK.I	3	2.95	0.17	-1.93
IPI00219365	Moesin	R.ISQLEM*AR.Q	2	2.95	0.13	-0.90
IPI00219365	Moesin	R.LKQIEEQTKK.A	2	2.99	0.08	-3.46
IPI00219365	Moesin	R.NISFNDKK.F	1	2.19	0.08	-3.97
IPI00219365	Moesin	R.VLEQHKLNK.D	2	2.18	0.14	-3.56
IPI00219365	Moesin	R.VTTM*DAELEFAIQPNTTGK.Q	2	4.94	0.48	-3.40
IPI00219420	Structural maintenance of chromosomes protein 3	K.LDQDLNEVKAR.V	1	2.85	0.09	-3.10
IPI00219420	Structural maintenance of chromosomes protein 3	R.ELKTKISAM*KEEK.E	2	3.77	0.09	
IPI00219425	Isoform Beta of Poliovirus receptor precursor	G.DVVVQAPTQVPGFLGDSVTLPCYLQVPNM*EVTHVSQLTWAR.H	3	3.87	0.45	-3.21
IPI00219425	Isoform Beta of Poliovirus receptor precursor	G.DVVVQAPTQVPGFLGDSVTLPCYLQVPNM*EVTHVSQLTWAR.H	4	4.58	0.30	-3.11
IPI00219425	Isoform Beta of Poliovirus receptor precursor	K.VQLTGEPVPM*AR.C	2	3.68	0.33	-3.49
IPI00219425	Isoform Beta of Poliovirus receptor precursor	R.VLAKPQNTAEVQK.V	2	3.95	0.37	-3.86
IPI00219425	Isoform Beta of Poliovirus receptor precursor	R.VLAKPQNTAEVQK.V	3	2.19	0.35	-3.83
IPI00219446	Phosphatidylethanolamine-binding protein 1	K.GNDISSGTVLSDYVGSGPPK.G	2	6.48	0.62	-4.73
IPI00219446	Phosphatidylethanolamine-binding protein 1	K.GNDISSGTVLSDYVGSGPPK.G	3	4.80	0.53	-2.42
IPI00219446	Phosphatidylethanolamine-binding protein 1	K.GNDISSGTVLSDYVGSGPPKGTGLHR.Y	2	3.99	0.48	-3.56
IPI00219446	Phosphatidylethanolamine-binding protein 1	K.GNDISSGTVLSDYVGSGPPKGTGLHR.Y	3	4.32	0.49	-2.85
IPI00219446	Phosphatidylethanolamine-binding protein 1	K.GNDISSGTVLSDYVGSGPPKGTGLHR.Y	4	3.18	0.40	-3.27
IPI00219446	Phosphatidylethanolamine-binding protein 1	K.LYEQLSGK.-	1	2.96	0.18	-2.61
IPI00219446	Phosphatidylethanolamine-binding protein 1	K.LYEQLSGK.-	2	2.80	0.21	-2.80
IPI00219446	Phosphatidylethanolamine-binding protein 1	K.LYTLVLTDPDAPSR.K	2	4.37	0.47	-4.27
IPI00219446	Phosphatidylethanolamine-binding protein 1	K.LYTLVLTDPDAPSRK.D	2	3.89	0.44	-2.15
IPI00219446	Phosphatidylethanolamine-binding protein 1	K.LYTLVLTDPDAPSRK.D	3	3.00	0.32	-1.74
IPI00219446	Phosphatidylethanolamine-binding protein 1	K.LYTLVLTDPDAPSRKDPK.Y	2	2.67	0.18	-3.93
IPI00219446	Phosphatidylethanolamine-binding protein 1	K.LYTLVLTDPDAPSRKDPK.Y	3	3.31	0.34	-4.05

IPI00219446	Phosphatidylethanolamine-binding protein 1	K.LYTLVLTDPDAPSRKDPK.Y	4	2.89	0.18	-2.15
IPI00219446	Phosphatidylethanolamine-binding protein 1	K.NRPTSISWDGLDSGK.L	3	3.33	0.27	-3.15
IPI00219446	Phosphatidylethanolamine-binding protein 1	K.VLTPTQVK.N	1	1.66	0.09	-2.66
IPI00219446	Phosphatidylethanolamine-binding protein 1	K.WSGPLSLQEVDEQPQHPLHVTYAGAAVDELGK.V	3	4.81	0.43	-4.41
IPI00219446	Phosphatidylethanolamine-binding protein 1	K.WSGPLSLQEVDEQPQHPLHVTYAGAAVDELGK.V	4	3.84	0.25	-0.77
IPI00219446	Phosphatidylethanolamine-binding protein 1	P.VAGTCYQAEWDDYVPK.L	2	4.66	0.47	-4.08
IPI00219446	Phosphatidylethanolamine-binding protein 1	R.APVAGTCYQAEWDDYVPK.L	2	6.23	0.59	-2.72
IPI00219446	Phosphatidylethanolamine-binding protein 1	R.APVAGTCYQAEWDDYVPK.L	3	3.13	0.28	-2.48
IPI00219446	Phosphatidylethanolamine-binding protein 1	R.YVWLVYEQDRPLK.C	2	3.90	0.41	-2.59
IPI00219446	Phosphatidylethanolamine-binding protein 1	R.YVWLVYEQDRPLK.C	3	2.02	0.15	-2.02
IPI00219446	Phosphatidylethanolamine-binding protein 1	R.YVWLVYEQDRPLKCDEPILSNR.S	3	4.73	0.45	-3.61
IPI00219446	Phosphatidylethanolamine-binding protein 1	R.YVWLVYEQDRPLKCDEPILSNR.S	4	2.36	0.11	-4.61
IPI00219446	Phosphatidylethanolamine-binding protein 1	W.SGPLSLQEVDEQPQHPLHVTYAGAAVDELGK.V	4	5.07	0.51	-1.62
IPI00219465	Transcobalamin-2 precursor	K.AQTPEGHFGNVYSTPLALQFLM*TSPM*PGAELGTACLK.A	3	6.03	0.54	-2.58
IPI00219465	Transcobalamin-2 precursor	K.AQTPEGHFGNVYSTPLALQFLM*TSPM*PGAELGTACLK.A	4	3.93	0.36	-2.99
IPI00219465	Transcobalamin-2 precursor	K.DGETIELR.L	2	2.33	0.07	-3.04
IPI00219465	Transcobalamin-2 precursor	K.TYIDLIFPDCLAPR.V	2	4.10	0.51	-2.03
IPI00219465	Transcobalamin-2 precursor	K.WFLEDEKR.A	2	1.99	0.17	-2.85
IPI00219465	Transcobalamin-2 precursor	R.DPNTPLLQGIADYRPK.D	3	2.72	0.15	-2.60
IPI00219465	Transcobalamin-2 precursor	R.LSLEHLNPSIYVGLR.L	2	3.43	0.32	-1.82
IPI00219465	Transcobalamin-2 precursor	R.LSLEHLNPSIYVGLR.L	3	4.65	0.26	-2.36
IPI00219465	Transcobalamin-2 precursor	R.LSSLQAGTKEDLYLHSLK.L	2	4.21	0.50	-3.35
IPI00219465	Transcobalamin-2 precursor	R.LSSLQAGTKEDLYLHSLK.L	3	2.06	0.19	-1.09
IPI00219465	Transcobalamin-2 precursor	R.VHDSVVDK.L	2	2.51	0.22	-2.75
IPI00219468	Isoform IIa of Profilin-2	K.DREGFFTNGLTLGAK.K	3	2.95	0.23	-1.85
IPI00219468	Isoform IIa of Profilin-2	R.VLVFVM*GK.E	2	2.72	0.20	-2.37
IPI00219525	6-phosphogluconate dehydrogenase, decarboxylating	K.GILFVGSGVSGGEEGAR.Y	2	3.85	0.34	-3.98
IPI00219526	Isoform 1 of Phosphoglucomutase-1	K.ADNFEYSDPVDGSISR.N	2	5.35	0.51	-4.18
IPI00219526	Isoform 1 of Phosphoglucomutase-1	K.DLEALM*FDR.S	2	1.73	0.12	-2.05
IPI00219526	Isoform 1 of Phosphoglucomutase-1	K.FFGNLM*DASK.L	2	2.23	0.21	-1.80
IPI00219526	Isoform 1 of Phosphoglucomutase-1	K.FNISNGGPAPEAITDKIFQISK.T	3	2.96	0.12	-2.38
IPI00219526	Isoform 1 of Phosphoglucomutase-1	K.IALYETPTGWK.F	2	2.51	0.11	-3.48
IPI00219526	Isoform 1 of Phosphoglucomutase-1	K.TIEEYAVCPDLK.V	2	3.88	0.31	-3.51
IPI00219526	Isoform 1 of Phosphoglucomutase-1	K.TQAYQDQKPGTSGLR.K	2	4.42	0.42	-3.22
IPI00219526	Isoform 1 of Phosphoglucomutase-1	K.TQAYQDQKPGTSGLR.K	3	3.27	0.17	-1.73
IPI00219526	Isoform 1 of Phosphoglucomutase-1	K.VFQSSANYAENFIQSIISTVEPAQR.Q	3	3.26	0.30	-6.14
IPI00219526	Isoform 1 of Phosphoglucomutase-1	R.LIFTDGSR.I	2	2.21	0.16	-2.59
IPI00219526	Isoform 1 of Phosphoglucomutase-1	R.LVIGQNGILSTPAVSCIIR.K	2	4.27	0.23	-3.94
IPI00219526	Isoform 1 of Phosphoglucomutase-1	R.LYIDSYEKDVAK.I	2	3.84	0.28	-1.59
IPI00219575	Bleomycin hydrolase	K.HVPEEVLAVLEQEPIILPAWDPM*GALA.E	3	4.23	0.32	-5.60
IPI00219575	Bleomycin hydrolase	K.IGPITPLEFYR.E	2	3.39	0.39	-4.18

IPI00219575	Bleomycin hydrolase	K.TLYNNQPIDFLKK.M	2	2.47	0.18	-2.54
IPI00219622	Proteasome subunit alpha type-2	R.YNEDLELEDAIHTAILTLK.E	2	4.25	0.43	-4.27
IPI00219622	Proteasome subunit alpha type-2	R.YNEDLELEDAIHTAILTLK.E	3	4.05	0.35	-4.93
IPI00219682	Erythrocyte band 7 integral membrane protein	K.DSVTISVDGVVYYR.V	2	2.95	0.34	-5.73
IPI00219682	Erythrocyte band 7 integral membrane protein	K.VIAAEGEMNASR.A	2	3.54	0.41	-3.55
IPI00219684	Fatty acid-binding protein, heart	K.LGVEFDETTADDR.K	2	3.96	0.55	-4.35
IPI00219684	Fatty acid-binding protein, heart	K.SIVTLDGGK.L	2	2.48	0.18	-3.59
IPI00219684	Fatty acid-binding protein, heart	K.SIVTLDGGKLVHLQK.W	2	2.28	0.15	-3.75
IPI00219684	Fatty acid-binding protein, heart	R.QVASM*TKPTTIIEK.N	2	2.70	0.13	-3.66
IPI00219757	Glutathione S-transferase P	K.AFLASPEYVNLPINGNGKQ.-	2	5.07	0.50	-2.80
IPI00219757	Glutathione S-transferase P	K.ALPGQLKPFETLLSQNQGGK.T	2	4.32	0.51	-3.30
IPI00219757	Glutathione S-transferase P	K.ALPGQLKPFETLLSQNQGGK.T	3	4.43	0.29	-3.59
IPI00219757	Glutathione S-transferase P	K.FQDGDLTLYQSNTILR.H	2	5.86	0.55	-3.57
IPI00219757	Glutathione S-transferase P	K.LKAFLASPEYVNLPINGNGKQ.-	3	4.11	0.29	-1.86
IPI00219757	Glutathione S-transferase P	K.YISLIYTNYEAGKDDYVK.A	3	2.34	0.18	-2.00
IPI00219757	Glutathione S-transferase P	M.PPYTVVYFPVR.G	2	3.88	0.45	-3.91
IPI00219757	Glutathione S-transferase P	R.MLLADQGQSWKEEVVTVETWQEGSLK.A	3	4.01	0.36	-2.99
IPI00219757	Glutathione S-transferase P	R.TLGLYGKDQQEAALVDM*VNDGVEDLR.C	3	5.61	0.30	-2.64
IPI00219798	Isoform 1 of Roundabout homolog 1 precursor	G.RRNIAEM*QVLGGYER.G	3	3.65	0.11	-7.53
IPI00219798	Isoform 1 of Roundabout homolog 1 precursor	K.AEASATLTVQEPPHFVVKPR.D	3	2.25	0.12	-4.60
IPI00219798	Isoform 1 of Roundabout homolog 1 precursor	K.ARPFFNEFQGADSEIK.F	2	3.87	0.23	-3.00
IPI00219798	Isoform 1 of Roundabout homolog 1 precursor	K.ARPFFNEFQGADSEIK.F	3	3.19	0.20	-3.47
IPI00219798	Isoform 1 of Roundabout homolog 1 precursor	K.ARPFFNEFQGADSEIKFAK.T	2	4.03	0.36	-1.88
IPI00219798	Isoform 1 of Roundabout homolog 1 precursor	K.ARPFFNEFQGADSEIKFAK.T	3	5.25	0.33	-0.81
IPI00219798	Isoform 1 of Roundabout homolog 1 precursor	K.ARPFFNEFQGADSEIKFAK.T	4	4.01	0.27	-1.25
IPI00219798	Isoform 1 of Roundabout homolog 1 precursor	K.AYLEVTDVIADRPPPVIR.Q	2	3.21	0.35	-4.37
IPI00219798	Isoform 1 of Roundabout homolog 1 precursor	K.AYLEVTDVIADRPPPVIR.Q	3	4.50	0.48	-3.92
IPI00219798	Isoform 1 of Roundabout homolog 1 precursor	K.DGSPLDDKDER.I	2	2.29	0.07	-1.54
IPI00219798	Isoform 1 of Roundabout homolog 1 precursor	K.DGVLVSTQDSR.I	1	1.93	0.24	-4.05
IPI00219798	Isoform 1 of Roundabout homolog 1 precursor	K.DGVLVSTQDSR.I	2	3.62	0.31	-2.25
IPI00219798	Isoform 1 of Roundabout homolog 1 precursor	K.GGERVETDKDDPR.S	2	2.20	0.08	-4.48
IPI00219798	Isoform 1 of Roundabout homolog 1 precursor	K.GLKPNAIYLFLVR.A	2	3.80	0.52	-5.14
IPI00219798	Isoform 1 of Roundabout homolog 1 precursor	K.GLKPNAIYLFLVR.A	3	4.40	0.41	-2.71
IPI00219798	Isoform 1 of Roundabout homolog 1 precursor	K.KDGSPLDDKDER.I	2	2.74	0.22	-3.36
IPI00219798	Isoform 1 of Roundabout homolog 1 precursor	K.LM*ITYTR.K	2	2.11	0.09	-2.15
IPI00219798	Isoform 1 of Roundabout homolog 1 precursor	K.NSVVIPDLRK.G	2	2.80	0.26	-2.81
IPI00219798	Isoform 1 of Roundabout homolog 1 precursor	K.QLENGVLQIR.Y	2	2.80	0.22	-3.43
IPI00219798	Isoform 1 of Roundabout homolog 1 precursor	K.SEPQFIQLDAHGNPVSPEDQVSLAQ.Q	2	3.59	0.40	-4.87
IPI00219798	Isoform 1 of Roundabout homolog 1 precursor	K.SRPDEGVYVCVAR.N	3	2.50	0.07	-2.12
IPI00219798	Isoform 1 of Roundabout homolog 1 precursor	K.TETSAIKGLKPNAIYLFLVR.A	3	3.30	0.26	-2.06
IPI00219798	Isoform 1 of Roundabout homolog 1 precursor	K.TETSAIKGLKPNAIYLFLVR.A	4	3.39	0.27	-1.75
IPI00219798	Isoform 1 of Roundabout homolog 1 precursor	K.TLEEAPSAPPQGVTVSK.N	2	3.31	0.42	-2.99

IPI00219798	Isoform 1 of Roundabout homolog 1 precursor	K.TVDGSTFSVVIPFLVPGIR.Y	2	4.58	0.51	-6.09
IPI00219798	Isoform 1 of Roundabout homolog 1 precursor	K.TVDGSTFSVVIPFLVPGIR.Y	3	4.33	0.29	-3.73
IPI00219798	Isoform 1 of Roundabout homolog 1 precursor	R.AANAYGISDPSQISDPVKTQDVLPTSQGVDHK.Q	3	5.28	0.45	-3.54
IPI00219798	Isoform 1 of Roundabout homolog 1 precursor	R.AANAYGISDPSQISDPVKTQDVLPTSQGVDHK.Q	4	2.99	0.24	-2.69
IPI00219798	Isoform 1 of Roundabout homolog 1 precursor	R.DQVVALGR.T	1	2.87	0.28	-3.37
IPI00219798	Isoform 1 of Roundabout homolog 1 precursor	R.EGSQNLLFSYQPPQSSSR.F	2	4.62	0.49	-3.62
IPI00219798	Isoform 1 of Roundabout homolog 1 precursor	R.ESEVAELTVLERPSFVK.R	2	3.80	0.47	-2.29
IPI00219798	Isoform 1 of Roundabout homolog 1 precursor	R.ESEVAELTVLERPSFVK.R	3	3.02	0.47	-1.56
IPI00219798	Isoform 1 of Roundabout homolog 1 precursor	R.FSVSQTGDLTITNVQR.S	2	4.65	0.41	-5.51
IPI00219798	Isoform 1 of Roundabout homolog 1 precursor	R.IKQLENGVLQIR.Y	2	3.33	0.29	-2.43
IPI00219798	Isoform 1 of Roundabout homolog 1 precursor	R.IKQLENGVLQIR.Y	3	4.54	0.19	-1.57
IPI00219798	Isoform 1 of Roundabout homolog 1 precursor	R.IVEHPSDLIVSK.G	2	3.25	0.38	-2.99
IPI00219798	Isoform 1 of Roundabout homolog 1 precursor	R.KDGVLVSTQDSR.I	2	3.89	0.38	-3.83
IPI00219798	Isoform 1 of Roundabout homolog 1 precursor	R.KDGVLVSTQDSR.I	3	2.08	0.17	-3.44
IPI00219798	Isoform 1 of Roundabout homolog 1 precursor	R.LRQEDFPPR.I	2	1.74	0.08	-5.77
IPI00219798	Isoform 1 of Roundabout homolog 1 precursor	R.M*LLPSGSLFFLR.I	2	3.22	0.24	-3.41
IPI00219798	Isoform 1 of Roundabout homolog 1 precursor	R.QNPSDVM*VAVGEPAVM*ECQPPR.G	3	4.70	0.50	-3.85
IPI00219798	Isoform 1 of Roundabout homolog 1 precursor	R.REGSQNLLFSYQPPQSSSR.F	3	3.68	0.20	-4.19
IPI00219798	Isoform 1 of Roundabout homolog 1 precursor	R.SDVGYYICQTLNVAGSIITK.A	2	5.90	0.53	-3.84
IPI00219798	Isoform 1 of Roundabout homolog 1 precursor	R.SDVGYYICQTLNVAGSIITK.A	3	4.34	0.33	-3.61
IPI00219798	Isoform 1 of Roundabout homolog 1 precursor	R.TVTFQCEATGNPQPAIFWR.R	2	4.44	0.40	-3.68
IPI00219798	Isoform 1 of Roundabout homolog 1 precursor	R.TVTFQCEATGNPQPAIFWR.R	3	4.57	0.34	-3.57
IPI00219798	Isoform 1 of Roundabout homolog 1 precursor	R.YSVEVAASTGAGSGVK.S	2	4.32	0.45	-4.20
IPI00219806	Protein S100-A7	K.GTNYLADVFEK.K	2	3.46	0.29	-4.12
IPI00219806	Protein S100-A7	K.IDFSEFLSLLGDIATDYHK.Q	2	3.17	0.36	-4.69
IPI00219806	Protein S100-A7	K.IDFSEFLSLLGDIATDYHK.Q	3	3.51	0.31	-4.09
IPI00219910	22 kDa protein	K.YVAVM*PPHIGDQPLTGAYTVTLDGR.G	3	3.63	0.32	-1.36
IPI00219910	22 kDa protein	R.LQAVTDDHIR.M	2	2.93	0.25	-2.05
IPI00219910	22 kDa protein	R.NDLSPTTVM*SEGAR.N	2	3.99	0.45	-4.90
IPI00219930	Cellular retinoic acid-binding protein 1	K.VGEGFEEETVDGRK.C	3	2.55	0.26	0.90
IPI00219930	Cellular retinoic acid-binding protein 1	R.QDGDQFYIK.T	2	1.92	0.22	-3.60
IPI00219930	Cellular retinoic acid-binding protein 1	R.SSENFDELLK.A	2	2.68	0.17	-1.72
IPI00219930	Cellular retinoic acid-binding protein 1	R.TTEINFK.V	2	1.51	0.09	-4.83
IPI00220070	6-phosphofructo-2-kinase/fructose-2,6-biphosphatase 4	R.YRYPKGESYEDLVQRLEPVIMELER.Q	2	1.31	0.09	-8.04
IPI00220117	Uncharacterized protein CD99	D.LADGVSGGEGK.G	2	3.18	0.19	-3.10
IPI00220117	Uncharacterized protein CD99	F.SDADLADGVSGGEGK.G	2	4.92	0.46	-4.19
IPI00220117	Uncharacterized protein CD99	G.SFSDADLADGVSGGEGK.G	2	5.21	0.52	-3.06
IPI00220117	Uncharacterized protein CD99	G.SFSDADLADGVSGGEGKGGSDGGGSHRK.E	3	4.14	0.50	-2.76
IPI00220117	Uncharacterized protein CD99	K.EGEEADAPGVIPGIVGA.V	2	3.10	0.45	-3.12
IPI00220117	Uncharacterized protein CD99	K.EGEEADAPGVIPGIVGAVVVA.V	2	2.98	0.28	-3.72
IPI00220117	Uncharacterized protein CD99	K.KPSAGDDFDLGDAVVDGEN.D	2	4.18	0.47	-3.81

IPI00220117	Uncharacterized protein CD99	K.KPSAGDDFDLGDAVVDGENDDPRPPNPPKPM*PN.P	3	3.66	0.23	-2.03
IPI00220117	Uncharacterized protein CD99	K.KPSAGDDFDLGDAVVDGENDDPRPPNPPKPM*PN.P	4	5.00	0.41	-2.32
IPI00220117	Uncharacterized protein CD99	K.KPSAGDDFDLGDAVVDGENDDPRPPNPPKPM*PNPN.P	4	4.79	0.48	-2.27
IPI00220117	Uncharacterized protein CD99	N.HPSSSGSFSDADLADGVSGGEGKGGSDGGGSHR.K	4	4.67	0.39	-2.17
IPI00220117	Uncharacterized protein CD99	N.PNHPSSSGSFSDADLADGVSGGEGK.G	2	5.54	0.56	-2.30
IPI00220117	Uncharacterized protein CD99	N.PNHPSSSGSFSDADLADGVSGGEGK.G	3	5.84	0.55	-2.76
IPI00220117	Uncharacterized protein CD99	N.PNPNHPSSSGSFSDADLADGVSGGEGK.G	3	5.71	0.55	-3.05
IPI00220117	Uncharacterized protein CD99	N.PNPNHPSSSGSFSDADLADGVSGGEGKGGSDGGGSHR.K	5	4.60	0.31	-5.58
IPI00220117	Uncharacterized protein CD99	N.PNPNHPSSSGSFSDADLADGVSGGEGKGGSDGGGSHRK.E	4	5.48	0.45	-4.37
IPI00220117	Uncharacterized protein CD99	P.NHPSSSGSFSDADLADGVSGGEGK.G	3	3.60	0.40	-1.21
IPI00220117	Uncharacterized protein CD99	P.SSSGSFSDADLADGVSGGEGK.G	2	5.59	0.54	-3.98
IPI00220117	Uncharacterized protein CD99	P.SSSGSFSDADLADGVSGGEGKGGSDGGGSHRK.E	3	5.16	0.49	-2.01
IPI00220117	Uncharacterized protein CD99	R.KEGEEADAPGVIPGIVGAVV.V	2	2.92	0.19	-2.94
IPI00220117	Uncharacterized protein CD99	R.KEGEEADAPGVIPGIVGAVVV.A	2	3.88	0.52	-5.17
IPI00220117	Uncharacterized protein CD99	R.KEGEEADAPGVIPGIVGAVVVAVA.G	2	3.41	0.30	-1.25
IPI00220117	Uncharacterized protein CD99	R.KEGEEADAPGVIPGIVGAVVVAVAGA.I	2	3.43	0.37	-3.35
IPI00220117	Uncharacterized protein CD99	S.DADLADGVSGGEGK.G	2	4.64	0.34	-1.74
IPI00220117	Uncharacterized protein CD99	S.FSDADLADGVSGGEGK.G	2	5.43	0.51	-3.95
IPI00220117	Uncharacterized protein CD99	S.FSDADLADGVSGGEGKGGSDGGGSHRK.E	3	4.22	0.47	-0.82
IPI00220117	Uncharacterized protein CD99	S.SGSFSDADLADGVSGGEGK.G	2	5.10	0.52	-2.18
IPI00220117	Uncharacterized protein CD99	S.SSGSFSDADLADGVSGGEGK.G	2	5.18	0.44	-2.31
IPI00220156	Isoform B of Transforming growth factor beta-2 precursor	R.FAGIDGTSTYTSGDQK.T	2	4.79	0.42	-1.81
IPI00220271	Alcohol dehydrogenase	K.GLVQALGLSNFNSR.Q	2	2.08	0.14	-3.84
IPI00220281	Guanine nucleotide-binding protein G(o) subunit alpha 1	K.EIYCHM*TCATDTNNIQVVFDAVTDIIIANNLR.G	3	3.01	0.21	-2.19
IPI00220281	Guanine nucleotide-binding protein G(o) subunit alpha 1	K.EIYCHMTCATDTNNIQVVFDAVTDIIIANNLR.G	3	4.14	0.31	-3.02
IPI00220292	Isoform 1 of Ecto-ADP-ribosyltransferase 3 precursor	A.EVLDM*ADNAFDDEYLK.C	2	5.53	0.58	-5.03
IPI00220292	Isoform 1 of Ecto-ADP-ribosyltransferase 3 precursor	K.AFHFYLTR.A	1	2.13	0.16	-3.45
IPI00220292	Isoform 1 of Ecto-ADP-ribosyltransferase 3 precursor	K.AFHFYLTR.A	2	2.47	0.23	-1.82
IPI00220292	Isoform 1 of Ecto-ADP-ribosyltransferase 3 precursor	K.M*AGQSREDYIYGFQFK.A	2	3.28	0.40	-3.30
IPI00220292	Isoform 1 of Ecto-ADP-ribosyltransferase 3 precursor	K.M*AGQSREDYIYGFQFK.A	3	4.00	0.40	-2.11
IPI00220292	Isoform 1 of Ecto-ADP-ribosyltransferase 3 precursor	K.TCSHYECAFLGGLK.T	3	3.22	0.29	-2.56
IPI00220292	Isoform 1 of Ecto-ADP-ribosyltransferase 3 precursor	K.TQIFLPM*NFK.D	2	3.01	0.10	-2.54

IPI00220292	Isoform 1 of Ecto-ADP-ribosyltransferase 3 precursor	K.YVPQLLKEEK.A	2	2.75	0.17	-3.54
IPI00220292	Isoform 1 of Ecto-ADP-ribosyltransferase 3 precursor	R.EDYIYGFQFK.A	2	3.41	0.38	-3.09
IPI00220292	Isoform 1 of Ecto-ADP-ribosyltransferase 3 precursor	R.KTQIFLPM*NFK.D	2	2.51	0.19	-1.27
IPI00220292	Isoform 1 of Ecto-ADP-ribosyltransferase 3 precursor	R.TSQGTSFTFGGLNQAR.F	2	5.05	0.44	-3.13
IPI00220292	Isoform 1 of Ecto-ADP-ribosyltransferase 3 precursor	R.TSQGTSFTFGGLNQAR.F	3	5.11	0.42	-1.77
IPI00220301	Peroxiredoxin-6	M.PGGLLLGDVAPNFEANTTVGR.I	3	4.12	0.39	-5.46
IPI00220301	Peroxiredoxin-6	R.DFTPVCTTELGR.A	2	3.00	0.27	-3.91
IPI00220301	Peroxiredoxin-6	R.NFDEILR.V	2	2.49	0.07	-2.84
IPI00220327	Keratin, type II cytoskeletal 1	K.DVDGAYM*TK.V	2	2.47	0.30	-2.68
IPI00220327	Keratin, type II cytoskeletal 1	K.KYEDEINKR.T	2	3.00	0.17	-1.46
IPI00220327	Keratin, type II cytoskeletal 1	K.LLEGEESR.I	2	2.26	0.06	-3.07
IPI00220327	Keratin, type II cytoskeletal 1	K.NKLNDLEDALQQAKEDLAR.L	3	4.58	0.27	
IPI00220327	Keratin, type II cytoskeletal 1	K.SLNNQFASFIDK.V	2	3.37	0.36	-7.51
IPI00220327	Keratin, type II cytoskeletal 1	K.SLNNQFASFIDKVR.F	2	3.41	0.28	
IPI00220327	Keratin, type II cytoskeletal 1	K.SLNNQFASFIDKVR.F	3	2.17	0.23	-2.96
IPI00220327	Keratin, type II cytoskeletal 1	K.WELLQQVDTSTR.T	2	4.51	0.27	
IPI00220327	Keratin, type II cytoskeletal 1	K.YEELQITAGR.H	2	3.38	0.18	-2.41
IPI00220327	Keratin, type II cytoskeletal 1	R.FLEQQNQVLQTK.W	2	4.27	0.32	-3.17
IPI00220327	Keratin, type II cytoskeletal 1	R.FSSCGGGGGSFGAGGGFGSR.S	2	3.74	0.42	
IPI00220327	Keratin, type II cytoskeletal 1	R.M*SGECAPNVSVSVSTSHTTISGGGSR.G	3	3.57	0.25	
IPI00220327	Keratin, type II cytoskeletal 1	R.SLDLDSIIAEVK.A	2	4.32	0.40	-2.85
IPI00220327	Keratin, type II cytoskeletal 1	R.THNLEPYFESFINNLR.R	2	3.45	0.24	
IPI00220327	Keratin, type II cytoskeletal 1	R.THNLEPYFESFINNLR.R	3	4.03	0.38	-4.28
IPI00220334	Isoform 3 of Seizure 6-like protein precursor	H.ILGQYLGNSGPQK.L	2	3.14	0.38	-2.17
IPI00220334	Isoform 3 of Seizure 6-like protein precursor	K.ATSAATVQR.A	2	2.58	0.13	-3.65
IPI00220334	Isoform 3 of Seizure 6-like protein precursor	K.IHVGEEK.R	2	2.55	0.13	-2.70
IPI00220334	Isoform 3 of Seizure 6-like protein precursor	K.LYSSTPDLTIQFHSDPAGLIFGK.G	2	5.11	0.57	-2.83
IPI00220334	Isoform 3 of Seizure 6-like protein precursor	K.LYSSTPDLTIQFHSDPAGLIFGK.G	3	2.22	0.11	-2.79
IPI00220334	Isoform 3 of Seizure 6-like protein precursor	K.SALLYDSLQTESVPFEGLLSEGNTIR.I	2	4.87	0.55	-5.04
IPI00220334	Isoform 3 of Seizure 6-like protein precursor	K.SALLYDSLQTESVPFEGLLSEGNTIR.I	3	6.00	0.50	-6.45
IPI00220334	Isoform 3 of Seizure 6-like protein precursor	K.TTSHTELVR.G	1	2.31	0.20	-3.44
IPI00220334	Isoform 3 of Seizure 6-like protein precursor	K.TTSHTELVR.G	2	2.61	0.22	-2.01
IPI00220334	Isoform 3 of Seizure 6-like protein precursor	L.ERDALPEGDASPLGPYLLPSGAPER.G	3	5.02	0.40	-2.86
IPI00220334	Isoform 3 of Seizure 6-like protein precursor	R.AASTFNIR.F	1	1.71	0.11	-0.19
IPI00220334	Isoform 3 of Seizure 6-like protein precursor	R.AASTFNIR.F	2	2.35	0.16	-1.78
IPI00220334	Isoform 3 of Seizure 6-like protein precursor	R.DALPEGDASPLGPYLLPSGAPER.G	3	3.64	0.19	-1.96
IPI00220334	Isoform 3 of Seizure 6-like protein precursor	R.IEFTSDQAR.A	1	1.82	0.05	-3.18

IPI00220334	Isoform 3 of Seizure 6-like protein precursor	R.IEFTSDQAR.A	2	3.16	0.25	-2.59
IPI00220334	Isoform 3 of Seizure 6-like protein precursor	R.LLLHDKDR.M	1	2.48	0.20	-5.14
IPI00220334	Isoform 3 of Seizure 6-like protein precursor	R.LLLHDKDR.M	2	2.83	0.22	-2.19
IPI00220334	Isoform 3 of Seizure 6-like protein precursor	R.SPTNTISVYFR.T	1	2.93	0.35	-3.12
IPI00220334	Isoform 3 of Seizure 6-like protein precursor	R.SPTNTISVYFR.T	2	3.46	0.37	-4.35
IPI00220334	Isoform 3 of Seizure 6-like protein precursor	R.TFQDDGLGTFQLHYQAFM*LSCNFPR.R	3	4.32	0.47	-3.49
IPI00220342	N(G),N(G)-dimethylarginine dimethylaminohydrolase 1	K.EVDM*M*KEALEK.L	2	2.33	0.06	-2.18
IPI00220342	N(G),N(G)-dimethylarginine dimethylaminohydrolase 1	K.IM*QQM*SDHRYDK.L	2	1.29	0.07	-1.09
IPI00220342	N(G),N(G)-dimethylarginine dimethylaminohydrolase 1	K.LQLNIVEM*KDENATLDGGDVLFTGR.E	3	4.65	0.35	-5.07
IPI00220342	N(G),N(G)-dimethylarginine dimethylaminohydrolase 1	K.SFCSM*AGPNLIAIGSSESAQK.A	2	4.19	0.39	-4.72
IPI00220342	N(G),N(G)-dimethylarginine dimethylaminohydrolase 1	K.VDGLLTCCSVLINK.K	2	3.87	0.35	-3.35
IPI00220342	N(G),N(G)-dimethylarginine dimethylaminohydrolase 1	R.ALPESLGQHALR.S	2	2.93	0.37	-3.18
IPI00220342	N(G),N(G)-dimethylarginine dimethylaminohydrolase 1	R.EFFVGLSK.W	1	2.13	0.25	-1.89
IPI00220342	N(G),N(G)-dimethylarginine dimethylaminohydrolase 1	R.EFFVGLSKR.T	2	2.40	0.22	-1.73
IPI00220342	N(G),N(G)-dimethylarginine dimethylaminohydrolase 1	R.GAEILADTFKDYAVSTVPVADGLHLK.S	3	4.17	0.33	-2.08
IPI00220342	N(G),N(G)-dimethylarginine dimethylaminohydrolase 1	R.GAEILADTFKDYAVSTVPVADGLHLK.S	4	4.26	0.28	-2.67
IPI00220342	N(G),N(G)-dimethylarginine dimethylaminohydrolase 1	R.QHQLYVGVLGSK.L	3	3.52	0.22	-4.17
IPI00220342	N(G),N(G)-dimethylarginine dimethylaminohydrolase 1	R.TPEEYPESAK.V	2	2.62	0.25	-2.49
IPI00220361	Calbindin	K.IGIVELAHVLPTEENFLLLFR.C	3	3.36	0.28	-5.29
IPI00220361	Calbindin	K.NKQDLDINNITTYKK.N	2	4.74	0.44	-3.71
IPI00220361	Calbindin	K.NKQDLDINNITTYKK.N	3	3.95	0.39	-3.63
IPI00220361	Calbindin	R.LLPVQENFLLK.F	2	2.56	0.15	-3.11
IPI00220362	10 kDa heat shock protein, mitochondrial	K.GKGGEIQPVSVK.V	2	2.73	0.27	0.67
IPI00220362	10 kDa heat shock protein, mitochondrial	K.VLQATVVAVGSGSK.G	2	4.36	0.49	-3.54
IPI00220362	10 kDa heat shock protein, mitochondrial	K.VVLDDKDYFLFR.D	2	3.48	0.40	-1.83
IPI00220362	10 kDa heat shock protein, mitochondrial	K.VVLDDKDYFLFR.D	3	3.49	0.35	-0.61
IPI00220562	Neuronal pentraxin-1 precursor	A.AETLSQLGQTLQSLK.T	2	4.42	0.35	-3.86
IPI00220562	Neuronal pentraxin-1 precursor	K.ALSGNVIAWAESHIEIYGGATK.W	2	4.74	0.46	-2.64
IPI00220562	Neuronal pentraxin-1 precursor	K.ALSGNVIAWAESHIEIYGGATK.W	3	3.01	0.27	-4.70
IPI00220562	Neuronal pentraxin-1 precursor	K.DNRPGDKFQLTFPLR.T	2	2.45	0.17	-4.00

IPI00220562	Neuronal pentraxin-1 precursor	K.DNRPGDKFQLTFPLR.T	3	3.34	0.34	-4.61
IPI00220562	Neuronal pentraxin-1 precursor	K.ETILSQKETIR.E	2	3.37	0.31	-2.36
IPI00220562	Neuronal pentraxin-1 precursor	K.ETILSQKETIR.E	3	2.44	0.05	-2.09
IPI00220562	Neuronal pentraxin-1 precursor	K.FQLTFPLR.T	2	2.90	0.14	-1.92
IPI00220562	Neuronal pentraxin-1 precursor	K.GQKDNRPGDKFQLTFPLR.T	3	3.94	0.31	-4.72
IPI00220562	Neuronal pentraxin-1 precursor	K.IETALTSLHQR.I	2	3.32	0.32	-3.97
IPI00220562	Neuronal pentraxin-1 precursor	K.IETALTSLHQR.I	3	2.45	0.28	-4.24
IPI00220562	Neuronal pentraxin-1 precursor	K.LPFVINDGK.W	1	2.64	0.12	-2.43
IPI00220562	Neuronal pentraxin-1 precursor	K.LPFVINDGK.W	2	2.91	0.19	-1.86
IPI00220562	Neuronal pentraxin-1 precursor	K.LTPGEVYNLATCSTK.A	2	4.23	0.44	-4.77
IPI00220562	Neuronal pentraxin-1 precursor	K.SLPEM*YAFTVCM*WLK.S	2	2.32	0.28	-4.87
IPI00220562	Neuronal pentraxin-1 precursor	K.TRLENLEQYSR.L	2	3.95	0.29	-3.37
IPI00220562	Neuronal pentraxin-1 precursor	K.VAKLPFVINDGK.W	2	2.50	0.11	-3.50
IPI00220562	Neuronal pentraxin-1 precursor	K.VAKLPFVINDGK.W	3	3.08	0.17	-3.04
IPI00220562	Neuronal pentraxin-1 precursor	K.WTFEACR.Q	2	1.93	0.06	-1.13
IPI00220562	Neuronal pentraxin-1 precursor	R.CESQSTLDPGAGEAR.A	2	5.14	0.51	-3.72
IPI00220562	Neuronal pentraxin-1 precursor	R.KLTPGEVYNLATCSTK.A	3	2.56	0.21	-4.37
IPI00220562	Neuronal pentraxin-1 precursor	R.LENLEQYSR.L	1	2.48	0.15	-3.33
IPI00220562	Neuronal pentraxin-1 precursor	R.LENLEQYSR.L	2	3.59	0.24	-2.65
IPI00220562	Neuronal pentraxin-1 precursor	R.TNYM*YAK.V	1	2.12	0.08	-2.16
IPI00220562	Neuronal pentraxin-1 precursor	R.TNYM*YAK.V	2	2.57	0.26	-1.91
IPI00220562	Neuronal pentraxin-1 precursor	R.TPAAETLSQLGQTLQSLK.T	2	6.07	0.52	-5.78
IPI00220562	Neuronal pentraxin-1 precursor	R.TPAAETLSQLGQTLQSLK.T	3	5.43	0.47	-4.02
IPI00220562	Neuronal pentraxin-1 precursor	R.VKIETALTSLHQR.I	2	4.23	0.42	-4.29
IPI00220562	Neuronal pentraxin-1 precursor	R.VNTLEEGK.G	2	2.39	0.09	-2.31
IPI00220562	Neuronal pentraxin-1 precursor	R.VNTLEEGKGGPR.N	2	3.67	0.41	-3.70
IPI00220562	Neuronal pentraxin-1 precursor	R.VNTLEEGKGGPR.N	3	2.03	0.13	-4.23
IPI00220578	Guanine nucleotide-binding protein G	K.IDFGEAARADDARQLFVLAGSAEEGVMTPELAGVIK.R	3	3.01	0.08	-8.51
IPI00220642	14-3-3 protein gamma	K.AYSEAHEISK.E	2	2.20	0.07	0.74
IPI00220642	14-3-3 protein gamma	K.ELEAVCQDVLSLLDNYLIK.N	2	4.69	0.50	-2.08
IPI00220642	14-3-3 protein gamma	K.ELEAVCQDVLSLLDNYLIK.N	3	2.84	0.10	-3.06
IPI00220642	14-3-3 protein gamma	K.IEKELEAVCQDVLSLLDNYLIK.N	3	3.41	0.25	-4.21
IPI00220642	14-3-3 protein gamma	K.M*KGDYYR.Y	2	2.25	0.17	-2.86
IPI00220642	14-3-3 protein gamma	K.NVTELNEPLSNEER.N	2	2.83	0.37	-2.34
IPI00220642	14-3-3 protein gamma	K.TAFDDAIAELDTLNEDSYKDSTLIM*QLLR.D	3	6.36	0.57	-2.58
IPI00220642	14-3-3 protein gamma	R.NLLSVAYK.N	1	1.68	0.08	-2.46
IPI00220642	14-3-3 protein gamma	R.NLLSVAYK.N	2	2.28	0.09	-1.47
IPI00220642	14-3-3 protein gamma	R.YDDM*AAAM*K.A	2	3.02	0.26	-2.31
IPI00220642	14-3-3 protein gamma	R.YLAEVATGEK.R	2	3.00	0.28	-2.68
IPI00220642	14-3-3 protein gamma	R.YLAEVATGEKR.A	2	2.58	0.21	-2.42
IPI00220644	Isoform M1 of Pyruvate kinase isozymes M1/M2	K.ASDVHEVR.K	2	1.97	0.11	-3.38
IPI00220644	Isoform M1 of Pyruvate kinase isozymes M1/M2	K.CDENILWLDYK.N	2	2.41	0.16	-4.71

IPI00220644	Isoform M1 of Pyruvate kinase isozymes M1/M2	K.CLAAALIVLTESGR.S	2	4.32	0.45	-4.99
IPI00220644	Isoform M1 of Pyruvate kinase isozymes M1/M2	K.CLAAALIVLTESGR.S	3	3.98	0.37	-2.95
IPI00220644	Isoform M1 of Pyruvate kinase isozymes M1/M2	K.FGVEQDVDM*VFASFIR.K	2	5.72	0.57	-7.61
IPI00220644	Isoform M1 of Pyruvate kinase isozymes M1/M2	K.FGVEQDVDM*VFASFIR.K	3	4.92	0.42	-4.92
IPI00220644	Isoform M1 of Pyruvate kinase isozymes M1/M2	K.FGVEQDVDM*VFASFIRK.A	3	3.47	0.36	-3.36
IPI00220644	Isoform M1 of Pyruvate kinase isozymes M1/M2	K.FGVEQDVDMVFASFIR.K	2	5.55	0.59	-5.30
IPI00220644	Isoform M1 of Pyruvate kinase isozymes M1/M2	K.FGVEQDVDMVFASFIR.K	3	3.76	0.30	-3.77
IPI00220644	Isoform M1 of Pyruvate kinase isozymes M1/M2	K.GADFLVTEVENGGSLGSK.K	2	6.03	0.51	-3.13
IPI00220644	Isoform M1 of Pyruvate kinase isozymes M1/M2	K.GADFLVTEVENGGSLGSKK.G	3	2.76	0.15	-2.27
IPI00220644	Isoform M1 of Pyruvate kinase isozymes M1/M2	K.GDYPLEAVR.M	1	1.50	0.30	-3.77
IPI00220644	Isoform M1 of Pyruvate kinase isozymes M1/M2	K.GDYPLEAVR.M	2	2.04	0.06	-1.57
IPI00220644	Isoform M1 of Pyruvate kinase isozymes M1/M2	K.GSGTAEVELK.K	2	3.39	0.26	-1.64
IPI00220644	Isoform M1 of Pyruvate kinase isozymes M1/M2	K.GSGTAEVELKK.G	2	2.95	0.22	-3.24
IPI00220644	Isoform M1 of Pyruvate kinase isozymes M1/M2	K.GVNLPGAAVDLPAVSEKDIQDLK.F	2	4.80	0.53	-2.87
IPI00220644	Isoform M1 of Pyruvate kinase isozymes M1/M2	K.IENHEGVR.R	2	3.04	0.11	-2.98
IPI00220644	Isoform M1 of Pyruvate kinase isozymes M1/M2	K.IISKIENHEGVR.R	2	3.37	0.35	-4.62
IPI00220644	Isoform M1 of Pyruvate kinase isozymes M1/M2	K.ITLDNAYM*EK.C	1	2.11	0.15	-2.12
IPI00220644	Isoform M1 of Pyruvate kinase isozymes M1/M2	K.ITLDNAYM*EK.C	2	3.00	0.32	-2.28
IPI00220644	Isoform M1 of Pyruvate kinase isozymes M1/M2	K.ITLDNAYM*EKCDENILWLDYK.N	3	2.90	0.17	-4.09
IPI00220644	Isoform M1 of Pyruvate kinase isozymes M1/M2	K.IYVDDGLISLQVK.Q	2	4.86	0.41	-3.52
IPI00220644	Isoform M1 of Pyruvate kinase isozymes M1/M2	K.KGVNLPGAAVDLPAVSEK.D	2	4.38	0.51	-3.91
IPI00220644	Isoform M1 of Pyruvate kinase isozymes M1/M2	K.KGVNLPGAAVDLPAVSEK.D	3	3.35	0.26	-2.85
IPI00220644	Isoform M1 of Pyruvate kinase isozymes M1/M2	K.KGVNLPGAAVDLPAVSEKDIQDLK.F	2	5.86	0.60	-5.06
IPI00220644	Isoform M1 of Pyruvate kinase isozymes M1/M2	K.KGVNLPGAAVDLPAVSEKDIQDLK.F	3	4.92	0.47	-4.24
IPI00220644	Isoform M1 of Pyruvate kinase isozymes M1/M2	K.LFEELVR.A	2	2.85	0.12	-2.21
IPI00220644	Isoform M1 of Pyruvate kinase isozymes M1/M2	K.QKGADFLVTEVENGGSLGSK.K	3	4.27	0.37	-2.87
IPI00220644	Isoform M1 of Pyruvate kinase isozymes M1/M2	N.LPGAAVDLPAVSEKDIQDLK.F	3	3.77	0.50	-1.52
IPI00220644	Isoform M1 of Pyruvate kinase isozymes M1/M2	R.AEGSDVANAVLDGADCIM*LSGETAKGDYPLEAVR.M	3	5.48	0.60	-5.27
IPI00220644	Isoform M1 of Pyruvate kinase isozymes M1/M2	R.AEGSDVANAVLDGADCIM*LSGETAKGDYPLEAVR.M	4	5.30	0.49	-2.77
IPI00220644	Isoform M1 of Pyruvate kinase isozymes M1/M2	R.AEGSDVANAVLDGADCIMLSGETAK.G	2	5.40	0.57	-2.91
IPI00220644	Isoform M1 of Pyruvate kinase isozymes M1/M2	R.AEGSDVANAVLDGADCIMLSGETAK.G	3	4.21	0.35	-2.37
IPI00220644	Isoform M1 of Pyruvate kinase isozymes M1/M2	R.AGKPVICATQM*LESM*IK.K	2	3.73	0.50	-3.04
IPI00220644	Isoform M1 of Pyruvate kinase isozymes M1/M2	R.AGKPVICATQM*LESM*IK.K	3	3.42	0.27	-1.80
IPI00220644	Isoform M1 of Pyruvate kinase isozymes M1/M2	R.APIIAVTR.N	1	2.18	0.26	-3.67
IPI00220644	Isoform M1 of Pyruvate kinase isozymes M1/M2	R.APIIAVTR.N	2	2.92	0.31	-4.09
IPI00220644	Isoform M1 of Pyruvate kinase isozymes M1/M2	R.EAEAAM*FHR.K	2	2.75	0.19	-2.76
IPI00220644	Isoform M1 of Pyruvate kinase isozymes M1/M2	R.FDEILEASDGIM*VAR.G	2	4.43	0.53	-4.49
IPI00220644	Isoform M1 of Pyruvate kinase isozymes M1/M2	R.GDLGIEIPAEK.V	2	3.68	0.23	-2.46
IPI00220644	Isoform M1 of Pyruvate kinase isozymes M1/M2	R.GIFPVLCKDPVQEAWAEDVDLR.V	2	5.44	0.55	-3.29
IPI00220644	Isoform M1 of Pyruvate kinase isozymes M1/M2	R.GIFPVLCKDPVQEAWAEDVDLR.V	3	5.87	0.46	-3.71
IPI00220644	Isoform M1 of Pyruvate kinase isozymes M1/M2	R.LDIDSPPITAR.N	1	1.91	0.32	-3.27
IPI00220644	Isoform M1 of Pyruvate kinase isozymes M1/M2	R.LDIDSPPITAR.N	2	3.93	0.34	-3.34

IPI00220644	Isoform M1 of Pyruvate kinase isozymes M1/M2	R.LNFSHGTHEYHAETIK.N	2	4.53	0.48	-5.84
IPI00220644	Isoform M1 of Pyruvate kinase isozymes M1/M2	R.LNFSHGTHEYHAETIK.N	3	3.79	0.38	-3.39
IPI00220644	Isoform M1 of Pyruvate kinase isozymes M1/M2	R.LNFSHGTHEYHAETIK.N	4	2.97	0.34	-3.20
IPI00220644	Isoform M1 of Pyruvate kinase isozymes M1/M2	R.NTGIICTIGPASR.S	1	3.05	0.41	-2.10
IPI00220644	Isoform M1 of Pyruvate kinase isozymes M1/M2	R.NTGIICTIGPASR.S	2	4.66	0.44	-2.25
IPI00220644	Isoform M1 of Pyruvate kinase isozymes M1/M2	R.RFDEILEASDGIM*VAR.G	2	3.07	0.15	-2.80
IPI00220644	Isoform M1 of Pyruvate kinase isozymes M1/M2	R.RFDEILEASDGIM*VAR.G	3	4.87	0.34	-2.15
IPI00220644	Isoform M1 of Pyruvate kinase isozymes M1/M2	R.RFDEILEASDGIMVAR.G	2	3.47	0.35	-3.56
IPI00220644	Isoform M1 of Pyruvate kinase isozymes M1/M2	R.RFDEILEASDGIMVAR.G	3	3.94	0.29	-3.12
IPI00220644	Isoform M1 of Pyruvate kinase isozymes M1/M2	R.SVETLKEM*IK.S	2	2.31	0.25	-1.93
IPI00220644	Isoform M1 of Pyruvate kinase isozymes M1/M2	R.TATESFASDPILYRPVAVALDTK.G	2	4.63	0.56	-4.63
IPI00220644	Isoform M1 of Pyruvate kinase isozymes M1/M2	R.TATESFASDPILYRPVAVALDTK.G	3	3.29	0.35	-4.68
IPI00220644	Isoform M1 of Pyruvate kinase isozymes M1/M2	R.TATESFASDPILYRPVAVALDTKGPE.I	3	5.32	0.55	-1.60
IPI00220644	Isoform M1 of Pyruvate kinase isozymes M1/M2	R.TATESFASDPILYRPVAVALDTKGPEI.R	3	5.90	0.64	-4.97
IPI00220644	Isoform M1 of Pyruvate kinase isozymes M1/M2	R.TATESFASDPILYRPVAVALDTKGPEIR.T	3	6.47	0.62	-5.03
IPI00220644	Isoform M1 of Pyruvate kinase isozymes M1/M2	R.TATESFASDPILYRPVAVALDTKGPEIR.T	4	4.39	0.41	-3.78
IPI00220644	Isoform M1 of Pyruvate kinase isozymes M1/M2	R.VNFAM*NVGK.A	1	2.68	0.27	-3.16
IPI00220644	Isoform M1 of Pyruvate kinase isozymes M1/M2	R.VNFAM*NVGK.A	2	3.09	0.34	-1.97
IPI00220706	Hemoglobin subunit gamma-1	R.FFDSFGNLSSASAIM*GNPK.V	2	5.02	0.53	
IPI00220706	Hemoglobin subunit gamma-1	R.LLVVYPWTQR.F	2	3.24	0.30	-6.61
IPI00220739	Membrane-associated progesterone receptor component 1	G.DQPAASGDSDDDEPPPLPR.L	2	4.30	0.61	-2.36
IPI00220739	Membrane-associated progesterone receptor component 1	G.DQPAASGDSDDDEPPPLPR.L	3	4.27	0.38	-2.28
IPI00220739	Membrane-associated progesterone receptor component 1	K.EGEEPTVYSDEEEPKDESAR.K	3	2.96	0.43	-2.98
IPI00220739	Membrane-associated progesterone receptor component 1	K.FYGPEGPYGVFAGR.D	2	4.43	0.55	-4.51
IPI00220739	Membrane-associated progesterone receptor component 1	K.LLKEGEEPTVYSDEEEPKDESAR.K	3	5.60	0.58	-1.69
IPI00220739	Membrane-associated progesterone receptor component 1	K.LLKEGEEPTVYSDEEEPKDESAR.K	4	3.35	0.26	-2.37
IPI00220739	Membrane-associated progesterone receptor component 1	R.FDGVQDPR.I	2	2.14	0.05	-2.68
IPI00220739	Membrane-associated progesterone receptor component 1	R.GDQPAASGDSDDDEPPPLPR.L	2	3.87	0.51	-2.91
IPI00220739	Membrane-associated progesterone receptor component 1	R.ILM*AINGK.V	2	2.29	0.07	-2.40
IPI00220739	Membrane-associated progesterone receptor component 1	R.KFYGPEGPYGVFAGR.D	2	4.04	0.50	-2.56
IPI00220739	Membrane-associated progesterone receptor component 1	R.KFYGPEGPYGVFAGR.D	3	4.01	0.42	-1.47

IPI00220741	spectrin, alpha, erythrocytic 1	K.ALGVPSSPYTWLTVEVLER.T	2	3.84	0.38	-4.21
IPI00220741	spectrin, alpha, erythrocytic 1	K.DLNTLAEDLLSSGTFNVDQIVK.K	2	5.31	0.54	-3.17
IPI00220741	spectrin, alpha, erythrocytic 1	K.EAIATSVELGEDWER.T	2	2.51	0.33	-2.47
IPI00220741	spectrin, alpha, erythrocytic 1	K.EAYALFQFFQDLDDEESWIEEK.L	2	6.28	0.55	-4.91
IPI00220741	spectrin, alpha, erythrocytic 1	K.EAYALFQFFQDLDDEESWIEEK.L	3	5.64	0.48	-4.54
IPI00220741	spectrin, alpha, erythrocytic 1	K.FQQYVQECADILEWIGDK.E	2	5.90	0.59	-5.03
IPI00220741	spectrin, alpha, erythrocytic 1	K.FQQYVQECADILEWIGDK.E	3	3.28	0.34	-6.31
IPI00220741	spectrin, alpha, erythrocytic 1	K.FQQYVQECADILEWIGDKEAIATSVELGEDWER.T	3	7.26	0.56	-5.60
IPI00220741	spectrin, alpha, erythrocytic 1	K.FQQYVQECADILEWIGDKEAIATSVELGEDWER.T	4	3.84	0.32	-3.23
IPI00220741	spectrin, alpha, erythrocytic 1	K.GYVSLEDYTAFLIDK.E	2	5.43	0.45	-6.15
IPI00220741	spectrin, alpha, erythrocytic 1	K.GYVSLEDYTAFLIDKESENIK.S	3	1.83	0.13	-3.58
IPI00220741	spectrin, alpha, erythrocytic 1	K.GYVSLEDYTAFLIDKESENIKSSDEIENAFQALAEGK.S	4	4.82	0.37	-4.35
IPI00220741	spectrin, alpha, erythrocytic 1	K.HEALENDFAVHETR.V	3	4.48	0.41	-3.32
IPI00220741	spectrin, alpha, erythrocytic 1	K.HEDFEEAFTAQEEK.I	2	4.64	0.49	-1.77
IPI00220741	spectrin, alpha, erythrocytic 1	K.HEDFEEAFTAQEEK.I	3	2.22	0.24	-3.65
IPI00220741	spectrin, alpha, erythrocytic 1	K.ILDQCLELQMFQGNCDQVESWMVAR.E	2	5.34	0.52	-3.34
IPI00220741	spectrin, alpha, erythrocytic 1	K.ILDQCLELQMFQGNCDQVESWMVAR.E	3	5.38	0.60	-6.33
IPI00220741	spectrin, alpha, erythrocytic 1	K.IVDLGDNLEDALILDIK.Y	2	5.60	0.49	-6.17
IPI00220741	spectrin, alpha, erythrocytic 1	K.IVDLGDNLEDALILDIK.Y	3	5.28	0.42	-3.39
IPI00220741	spectrin, alpha, erythrocytic 1	K.KAENTGVELDDVWELQK.K	3	3.36	0.23	-4.90
IPI00220741	spectrin, alpha, erythrocytic 1	K.KFEDFQVELVAK.E	3	3.10	0.21	-2.40
IPI00220741	spectrin, alpha, erythrocytic 1	K.LEESLEYLQFMQNAEEEEAWINEK.N	2	6.03	0.57	-4.81
IPI00220741	spectrin, alpha, erythrocytic 1	K.LEESLEYLQFMQNAEEEEAWINEK.N	3	6.42	0.55	-5.52
IPI00220741	spectrin, alpha, erythrocytic 1	K.LKEAYALFQFFQDLDDEESWIEEK.L	3	3.45	0.37	-4.10
IPI00220741	spectrin, alpha, erythrocytic 1	K.M*EILDNNWTALLELWDER.H	2	4.54	0.34	-4.67
IPI00220741	spectrin, alpha, erythrocytic 1	K.MEILDNNWTALLELWDER.H	2	6.10	0.46	-4.58
IPI00220741	spectrin, alpha, erythrocytic 1	K.MEILDNNWTALLELWDER.H	3	4.88	0.25	-5.06
IPI00220741	spectrin, alpha, erythrocytic 1	K.MVEEGHFAAEDVASR.V	2	4.51	0.47	-3.51
IPI00220741	spectrin, alpha, erythrocytic 1	K.MVEEGHFAAEDVASR.V	3	3.87	0.41	-2.81
IPI00220741	spectrin, alpha, erythrocytic 1	K.NFEM*CQEFEQNASTFLQWILETR.A	3	4.92	0.39	-4.59
IPI00220741	spectrin, alpha, erythrocytic 1	K.NFEMCQEFEQNASTFLQWILETR.A	2	5.49	0.58	-6.25
IPI00220741	spectrin, alpha, erythrocytic 1	K.NFEMCQEFEQNASTFLQWILETR.A	3	6.29	0.54	-5.23
IPI00220741	spectrin, alpha, erythrocytic 1	K.NFEMCQEFEQNASTFLQWILETR.A	4	3.31	0.21	-3.42
IPI00220741	spectrin, alpha, erythrocytic 1	K.TGQEMIEGGHYASDNVTTR.L	3	3.10	0.44	-3.82
IPI00220741	spectrin, alpha, erythrocytic 1	K.YSTIGLAQQWDQLYQLGLR.M	2	5.32	0.54	-5.48
IPI00220741	spectrin, alpha, erythrocytic 1	K.YSTIGLAQQWDQLYQLGLR.M	3	4.77	0.50	-3.85
IPI00220741	spectrin, alpha, erythrocytic 1	N.WISSIGGMVSSQELAEDLTGIEILLER.H	3	4.13	0.31	-4.20
IPI00220741	spectrin, alpha, erythrocytic 1	R.DFEFWLSEAETLLAMK.D	2	5.85	0.52	-6.16
IPI00220741	spectrin, alpha, erythrocytic 1	R.DFEFWLSEAETLLAMK.D	3	6.81	0.50	-3.60
IPI00220741	spectrin, alpha, erythrocytic 1	R.DLQNWISSIGGM*VSSQELAEDLTGIEILLER.H	3	6.68	0.52	-5.44
IPI00220741	spectrin, alpha, erythrocytic 1	R.EKMEILDNNWTALLELWDER.H	3	4.02	0.28	-5.45
IPI00220741	spectrin, alpha, erythrocytic 1	R.FNTSIRDFEFWLSEAETLLAMKDQAR.D	3	2.96	0.37	-3.19

IPI00220741	spectrin, alpha, erythrocytic 1	R.FNTSIRDFEFWLSEAETLLAMKDQAR.D	4	3.89	0.34	-4.19
IPI00220741	spectrin, alpha, erythrocytic 1	R.GDCGDTLAATQSLLMK.H	2	4.78	0.45	-2.59
IPI00220741	spectrin, alpha, erythrocytic 1	R.HLWDLLLELTLEK.G	2	5.25	0.56	-5.25
IPI00220741	spectrin, alpha, erythrocytic 1	R.HLWDLLLELTLEK.G	3	4.86	0.32	-3.75
IPI00220741	spectrin, alpha, erythrocytic 1	R.KGYVSLEDYTAFLIDK.E	2	4.62	0.43	-3.94
IPI00220741	spectrin, alpha, erythrocytic 1	R.KGYVSLEDYTAFLIDK.E	3	4.41	0.33	-5.06
IPI00220741	spectrin, alpha, erythrocytic 1	R.KGYVSLEDYTAFLIDKESENIK.S	3	5.01	0.40	-4.70
IPI00220741	spectrin, alpha, erythrocytic 1	R.KGYVSLEDYTAFLIDKESENIKSSDEIENAFQALAEGK.S	4	4.18	0.31	-3.33
IPI00220741	spectrin, alpha, erythrocytic 1	R.LSEVASLWEELLEATK.Q	2	3.69	0.32	-2.22
IPI00220741	spectrin, alpha, erythrocytic 1	R.QDQVDILTDLAAYFEEIGHPDSK.D	3	4.76	0.44	-4.65
IPI00220741	spectrin, alpha, erythrocytic 1	R.QNDLEANVQFQQYLADLHEAETWIR.E	3	4.48	0.48	-6.73
IPI00220741	spectrin, alpha, erythrocytic 1	R.QYEQCLDFHLFYR.D	3	1.70	0.14	-3.37
IPI00220741	spectrin, alpha, erythrocytic 1	R.RQNDLEANVQFQQYLADLHEAETWIR.E	3	5.50	0.59	-2.82
IPI00220741	spectrin, alpha, erythrocytic 1	R.RQNDLEANVQFQQYLADLHEAETWIR.E	4	3.24	0.05	-3.33
IPI00220741	spectrin, alpha, erythrocytic 1	R.SHLSGYDYVGFTNSYFGN.-	2	4.81	0.54	-4.72
IPI00220741	spectrin, alpha, erythrocytic 1	R.YNEFLLAYEAGDM*LEWIQEK.K	2	4.74	0.52	-5.01
IPI00220741	spectrin, alpha, erythrocytic 1	R.YNEFLLAYEAGDM*LEWIQEK.K	3	2.61	0.11	1.78
IPI00220741	spectrin, alpha, erythrocytic 1	R.YNEFLLAYEAGDMLEWIQEK.K	2	6.57	0.60	-5.72
IPI00220741	spectrin, alpha, erythrocytic 1	R.YNEFLLAYEAGDMLEWIQEK.K	3	5.92	0.51	-4.87
IPI00220741	spectrin, alpha, erythrocytic 1	S.SIGGMVSSQELAEDLTGIEILLER.H	2	5.36	0.54	-3.92
IPI00220741	spectrin, alpha, erythrocytic 1	S.SIGGMVSSQELAEDLTGIEILLER.H	3	5.01	0.38	-4.62
IPI00220741	spectrin, alpha, erythrocytic 1	W.ISSIGGMVSSQELAEDLTGIEILLER.H	3	3.79	0.30	-3.54
IPI00220741	spectrin, alpha, erythrocytic 1	Y.ALFQFFQDLDDEESWIEEK.L	2	5.80	0.54	-4.74
IPI00220741	spectrin, alpha, erythrocytic 1	Y.ALFQFFQDLDDEESWIEEK.L	3	4.93	0.46	-3.62
IPI00220748	Isoform Alpha-7X1A of Integrin alpha-7 precursor	R.ELEPPEQQEPGER.Q	2	2.32	0.19	-4.55
IPI00220748	Isoform Alpha-7X1A of Integrin alpha-7 precursor	R.ELEPPEQQEPGERQEPS.M	2	3.00	0.29	-4.53
IPI00220766	Lactoylglutathione lyase	K.GLAFIQDPDGYWIEILNPNK.M	2	6.08	0.52	-5.23
IPI00220766	Lactoylglutathione lyase	K.SLDFYTR.V	2	2.54	0.21	-1.28
IPI00220766	Lactoylglutathione lyase	R.VLGM*TLIQK.C	2	2.42	0.05	-2.35
IPI00220791	Amphiphysin I variant CT2	K.IGTETTEGAESAQPEAEELEATVPQEK.V	3	3.44	0.34	-2.86
IPI00220827	Thymosin beta-10	K.NTLPTKETIEQE.K	2	3.13	0.28	-1.70
IPI00220827	Thymosin beta-10	K.NTLPTKETIEQEK.R	2	3.68	0.15	-2.09
IPI00220827	Thymosin beta-10	K.NTLPTKETIEQEKR.S	2	3.63	0.32	-4.82
IPI00220827	Thymosin beta-10	K.NTLPTKETIEQEKR.S	3	3.21	0.39	-3.13
IPI00220827	Thymosin beta-10	K.NTLPTKETIEQEKR.S	4	2.36	0.14	-3.78
IPI00220827	Thymosin beta-10	K.TETQEKNTLPTK.E	2	3.23	0.12	-2.69
IPI00220827	Thymosin beta-10	K.TETQEKNTLPTKETIEQEKR.S	3	4.93	0.41	-4.57
IPI00220827	Thymosin beta-10	K.TETQEKNTLPTKETIEQEKR.S	4	3.15	0.29	-3.59
IPI00221006	Isoform 4 of Transcription factor 7-like 2	R.KRDKQPGETNDLSAPK.K	2	2.51	0.06	2.52
IPI00221034	Transcription factor RelB	K.RGM*PDVLGELNSSDPHGIESKRRKK.K	3	4.00	0.11	
IPI00221080	Isoform 2 of Parathyroid hormone-related protein precursor	R.ATSEVSPNSKPSPNTK.N	2	3.92	0.43	-3.54

IPI00221117	Acylphosphatase-1	K.VQGVFFR.K	2	2.00	0.21	-1.44
IPI00221117	Acylphosphatase-1	R.GTVQGQLQGPISK.V	2	2.27	0.21	-2.78
IPI00221178	Isoform 2 of Tumor protein D54	R.TPAVEGLTEAEEEELRAELTKVEEEIVTLR.Q	4	4.95	0.46	-3.02
IPI00221224	Aminopeptidase N	K.DLM*VLNDVYR.V	2	3.34	0.38	-3.21
IPI00221224	Aminopeptidase N	K.DLTALSNM*LPK.G	2	3.55	0.40	-4.34
IPI00221224	Aminopeptidase N	K.DNEETGFGSGTR.A	2	3.47	0.46	-0.32
IPI00221224	Aminopeptidase N	K.EVVLQWFTENSK.-	2	4.44	0.41	-3.75
IPI00221224	Aminopeptidase N	K.LFNDYGGGSFSFSNLIQAVTR.R	2	4.76	0.47	-2.71
IPI00221224	Aminopeptidase N	K.VVATTQM*QAADAR.K	2	4.26	0.46	-2.79
IPI00221224	Aminopeptidase N	N.PASATTLDQSK.A	2	3.71	0.34	0.00
IPI00221224	Aminopeptidase N	R.GVGGSQPPDIDKTELVEPTEYLVVHLK.G	3	4.44	0.39	-4.00
IPI00221224	Aminopeptidase N	R.GVGGSQPPDIDKTELVEPTEYLVVHLK.G	4	3.22	0.32	-3.19
IPI00221224	Aminopeptidase N	R.KVVATTQM*QAADAR.K	2	4.36	0.52	-3.66
IPI00221224	Aminopeptidase N	R.M*LSSFLSEDVFK.Q	2	4.13	0.40	-4.53
IPI00221224	Aminopeptidase N	R.QYM*PWEAALSSLSYFK.L	2	4.23	0.51	-4.80
IPI00221224	Aminopeptidase N	R.RFSTEYELQQLEQFKK.D	3	5.02	0.33	-3.70
IPI00221224	Aminopeptidase N	R.SEYM*EGNVR.K	2	3.31	0.31	-1.40
IPI00221224	Aminopeptidase N	R.VM*AVDALASSHPLSTPASEINTPAQISELFDAISYSK.G	3	7.45	0.58	-2.24
IPI00221224	Aminopeptidase N	R.VM*AVDALASSHPLSTPASEINTPAQISELFDAISYSK.G	4	4.20	0.52	-2.47
IPI00221224	Aminopeptidase N	R.VTLRPYLTPNDR.G	3	2.56	0.11	-2.17
IPI00221235	nucleoporin 160kDa	K.M*AAAGALER.S	1	1.17	0.12	-3.54
IPI00221255	Isoform 2 of Myosin light chain kinase, smooth muscle	R.DLEVVEGSAAR.F	2	3.35	0.37	-3.12
IPI00221332	Uncharacterized protein DNM3	K.ALLQMVQQFAVDFEK.R	2	4.83	0.49	1.93
IPI00233358	islet cell autoantigen 1,69kDa-like isoform 2	K.IIEKYQLRLNGM*KS.-	1	2.16	0.07	
IPI00235647	similar to fibrillarin	R.DLVNVAK.K	1	1.88	0.11	-2.97
IPI00240793	Probable phospholipid-transporting ATPase IF	K.ISPEKPITLAVGDGANDVSMIQEAHVGIGIMGKEGR.Q	3	3.37	0.11	-0.36
IPI00241409	hypothetical protein LOC55747	K.VSNIFDDPLNAFGGQ.-	3	1.61	0.17	-0.45
IPI00241562	reelin isoform a	K.CGILSSGNNLFFNEDGLR.M	2	5.25	0.49	-4.94
IPI00241562	reelin isoform a	K.CGILSSGNNLFFNEDGLR.M	3	3.66	0.19	-0.92
IPI00241562	reelin isoform a	K.CSGSVSQPSVFFPTK.G	2	4.14	0.42	-2.86
IPI00241562	reelin isoform a	K.DFTQAQR.V	1	1.75	0.20	-3.24
IPI00241562	reelin isoform a	K.DFTQAQR.V	2	2.10	0.21	-3.00
IPI00241562	reelin isoform a	K.DNFESAR.V	2	1.82	0.06	-3.42
IPI00241562	reelin isoform a	K.EGVLLDYSTDGGITWTLLHEM*DYQK.Y	3	3.23	0.32	-1.65
IPI00241562	reelin isoform a	K.FLQYWGR.I	2	2.25	0.17	-1.18
IPI00241562	reelin isoform a	K.GAPEEDSAM*VFVSNEVGEHSITTR.D	3	2.97	0.17	-3.43
IPI00241562	reelin isoform a	K.HSCQSDGNSIYFHGNEGSEFNFATTR.D	3	5.85	0.53	
IPI00241562	reelin isoform a	K.ISTKNPDFLKDDFEGQLESDRFLLM*SGGKPSR.K	4	3.81	0.26	-2.25
IPI00241562	reelin isoform a	K.IVVGCEATSCGDLHSVM*LEYTK.D	3	3.23	0.39	-4.02
IPI00241562	reelin isoform a	K.LCTPSM*DTTGYGNLR.F	2	3.41	0.31	
IPI00241562	reelin isoform a	K.LLEHYSYLSYHEPR.I	2	4.22	0.40	

IPI00241562	reelin isoform a	K.LLEHYSYLSYHEPR.I	3	4.76	0.37	
IPI00241562	reelin isoform a	K.LNIGCANQFSSTAPVLLQYSHDAGM*SWFLVK.E	3	5.48	0.49	-1.48
IPI00241562	reelin isoform a	K.M*PVCGSTGDALVFIEK.A	2	4.60	0.43	-2.51
IPI00241562	reelin isoform a	K.RITIQLPDHVSSSATQFR.W	2	4.34	0.43	-3.46
IPI00241562	reelin isoform a	K.RITIQLPDHVSSSATQFR.W	3	4.00	0.47	-3.78
IPI00241562	reelin isoform a	K.RITIQLPDHVSSSATQFR.W	4	3.02	0.28	-4.25
IPI00241562	reelin isoform a	K.RITYPLPESLVGNPVR.F	2	3.45	0.37	-4.50
IPI00241562	reelin isoform a	K.RITYPLPESLVGNPVR.F	3	4.12	0.36	-3.14
IPI00241562	reelin isoform a	K.SDGDRFAVTR.D	2	2.04	0.07	-2.46
IPI00241562	reelin isoform a	K.SGTSLIFKGEGLR.M	2	2.45	0.21	-4.27
IPI00241562	reelin isoform a	K.SLYFNGPGKR.E	2	2.39	0.21	-2.77
IPI00241562	reelin isoform a	K.VPSLVSVVINPELQTPATK.F	2	5.20	0.46	
IPI00241562	reelin isoform a	K.YSDM*QWAIDNFYLGPGCLDNCR.G	2	5.17	0.64	-5.30
IPI00241562	reelin isoform a	K.YSDM*QWAIDNFYLGPGCLDNCR.G	3	4.92	0.31	-5.83
IPI00241562	reelin isoform a	K.YTPHM*DNQVK.L	2	1.99	0.23	
IPI00241562	reelin isoform a	R.APDQPGEGVLLHYSYDNGITWK.L	3	3.28	0.23	
IPI00241562	reelin isoform a	R.APSSQNWLTVNGGK.L	2	3.83	0.48	-2.65
IPI00241562	reelin isoform a	R.DCLPTNVECSR.Y	2	2.43	0.15	-3.28
IPI00241562	reelin isoform a	R.DLTLKPGYVLQFK.L	2	3.20	0.30	-3.49
IPI00241562	reelin isoform a	R.DLTLKPGYVLQFK.L	3	2.12	0.32	-0.99
IPI00241562	reelin isoform a	R.ELDFM*SFLEPQIISIDLPQDAK.T	3	2.58	0.12	-4.15
IPI00241562	reelin isoform a	R.ELIIQPGYM*M*QFK.I	2	4.09	0.36	-4.03
IPI00241562	reelin isoform a	R.ELIIQPGYM*M*QFK.I	3	2.86	0.20	-1.62
IPI00241562	reelin isoform a	R.EVVHFGK.L	1	1.71	0.12	-3.24
IPI00241562	reelin isoform a	R.EVYAVTHDLTPTEGWIM*QFK.I	2	5.09	0.54	-1.92
IPI00241562	reelin isoform a	R.EVYAVTHDLTPTEGWIM*QFK.I	3	4.54	0.40	-2.15
IPI00241562	reelin isoform a	R.EVYAVTHDLTPTEGWIMQFK.I	2	5.42	0.57	-2.04
IPI00241562	reelin isoform a	R.EVYAVTHDLTPTEGWIMQFK.I	3	3.24	0.17	-1.76
IPI00241562	reelin isoform a	R.FSYSDPSIIVLYAK.N	2	4.23	0.37	
IPI00241562	reelin isoform a	R.FYFVM*GGICDPGNSHENDIILYAK.I	3	3.57	0.22	
IPI00241562	reelin isoform a	R.GAEVSFGCGVLASGK.A	2	4.28	0.40	
IPI00241562	reelin isoform a	R.GFGGPYCVPVVPLPSILK.D	2	3.31	0.22	-3.14
IPI00241562	reelin isoform a	R.GFGGPYCVPVVPLPSILKDDFNGNLHPDLWPEVYGAER.G	3	5.12	0.48	-1.94
IPI00241562	reelin isoform a	R.GFGGPYCVPVVPLPSILKDDFNGNLHPDLWPEVYGAER.G	4	4.43	0.43	-4.05
IPI00241562	reelin isoform a	R.GNLNGETIKSGTSLIFKGEGLR.M	3	3.51	0.35	-1.85
IPI00241562	reelin isoform a	R.HDGLDQNDWAIDNVLISGSADQR.T	3	4.06	0.34	-2.95
IPI00241562	reelin isoform a	R.IAFDM*FM*EDK.T	2	3.51	0.42	-2.72
IPI00241562	reelin isoform a	R.IISVELPGDAK.Q	2	3.28	0.27	
IPI00241562	reelin isoform a	R.ILVSDTFNK.W	2	2.96	0.27	-6.40
IPI00241562	reelin isoform a	R.IQGGQVDIDCLSM*DTALIFTENIGKPR.Y	3	2.91	0.24	-1.24
IPI00241562	reelin isoform a	R.ITIQLPDHVSSSATQFR.W	2	4.53	0.49	-2.98
IPI00241562	reelin isoform a	R.ITIQLPDHVSSSATQFR.W	3	5.08	0.53	-3.75

IPI00241562	reelin isoform a	R.ITIVIPR.S	2	1.87	0.10	-3.00
IPI00241562	reelin isoform a	R.ITLPLPPYTR.S	2	2.41	0.24	-1.75
IPI00241562	reelin isoform a	R.ITVYLPLSTISPR.T	2	3.78	0.45	-4.50
IPI00241562	reelin isoform a	R.ITVYLPLSTISPR.T	3	2.39	0.15	-2.78
IPI00241562	reelin isoform a	R.ITYPLPESLVGNPVR.F	2	3.61	0.50	-5.85
IPI00241562	reelin isoform a	R.LSSYHNFYSIR.G	2	3.42	0.29	
IPI00241562	reelin isoform a	R.LSSYHNFYSIR.G	3	2.30	0.20	
IPI00241562	reelin isoform a	R.QAATKPLDLTR.A	2	1.94	0.18	-1.62
IPI00241562	reelin isoform a	R.QAATKPLDLTR.A	3	1.91	0.12	-3.60
IPI00241562	reelin isoform a	R.QAVTQDLDLR.G	1	1.17	0.10	-2.95
IPI00241562	reelin isoform a	R.QAVTQDLDLR.G	2	3.13	0.26	-3.10
IPI00241562	reelin isoform a	R.QVVLEDSLDPVDTGNWLFFPGATVK.H	2	4.88	0.42	
IPI00241562	reelin isoform a	R.QVVLEDSLDPVDTGNWLFFPGATVK.H	3	4.83	0.43	
IPI00241562	reelin isoform a	R.RVIVLLPQK.T	2	2.28	0.11	
IPI00241562	reelin isoform a	R.TVM*LDTFSSAPVPQHER.S	2	2.91	0.29	2.52
IPI00241562	reelin isoform a	R.TVM*LDTFSSAPVPQHER.S	3	2.98	0.26	0.42
IPI00241562	reelin isoform a	R.VIVLLPQK.T	2	3.04	0.23	-2.70
IPI00241562	reelin isoform a	R.VSYNVPLEAR.M	1	2.19	0.23	-3.62
IPI00241562	reelin isoform a	R.VSYNVPLEAR.M	2	2.17	0.10	-1.81
IPI00241562	reelin isoform a	R.VTEANWETIQGGVIGSGCGQLAPYAHGDSLYFNGCQIR.Q	3	5.89	0.52	-1.61
IPI00241562	reelin isoform a	R.WWQPFVISNGIVVSGVER.A	2	5.23	0.49	-4.19
IPI00241562	reelin isoform a	R.WWQPFVISNGIVVSGVER.A	3	5.32	0.32	-3.64
IPI00241562	reelin isoform a	R.YIALEIPLK.A	2	3.10	0.29	-0.55
IPI00241562	reelin isoform a	R.YIALEIPLKAR.S	2	2.08	0.23	-3.12
IPI00242905	Uncharacterized protein ENSP00000344689 (Fragment)	R.KIEVNNATARVM*TNK.K	2	1.13	0.09	1.70
IPI00242905	Uncharacterized protein ENSP00000344689 (Fragment)	R.VMTNKKTGNPYTNGWK.L	2	1.76	0.07	-1.56
IPI00242956	IgGFc-binding protein precursor	D.PHYHSFDGR.K	1	2.52	0.26	-5.29
IPI00242956	IgGFc-binding protein precursor	D.PHYHSFDGR.K	2	3.08	0.36	-4.21
IPI00242956	IgGFc-binding protein precursor	D.PHYTTFDGR.R	1	2.84	0.07	-2.22
IPI00242956	IgGFc-binding protein precursor	D.PHYVTLDGHR.F	1	2.80	0.30	-5.14
IPI00242956	IgGFc-binding protein precursor	K.AGCVAESTAVCR.A	2	3.86	0.45	-2.72
IPI00242956	IgGFc-binding protein precursor	K.AIGYATAADCGR.T	2	3.69	0.46	-3.97
IPI00242956	IgGFc-binding protein precursor	K.AISGLTIDGHAVGAK.L	2	4.69	0.40	-1.31
IPI00242956	IgGFc-binding protein precursor	K.ALASYVAACQAAGVVIEDWR.A	2	5.36	0.47	-4.22
IPI00242956	IgGFc-binding protein precursor	K.ALASYVAACQAAGVVIEDWR.A	3	4.87	0.43	-3.72
IPI00242956	IgGFc-binding protein precursor	K.FYPAGDVLR.V	1	1.92	0.23	-2.57
IPI00242956	IgGFc-binding protein precursor	K.FYPAGDVLR.V	2	2.46	0.26	-2.49
IPI00242956	IgGFc-binding protein precursor	K.GCVLDVCM*GGGDHDILCK.A	2	4.57	0.54	-2.35
IPI00242956	IgGFc-binding protein precursor	K.GCVLDVCM*GGGDHDILCK.A	3	2.46	0.16	-2.36
IPI00242956	IgGFc-binding protein precursor	K.GCVLDVCM*GGGDRDILCK.A	3	2.63	0.27	-2.62

IPI00242956	IgGFc-binding protein precursor	K.LASVSVSR.T	2	2.24	0.17	-3.62
IPI00242956	IgGFc-binding protein precursor	K.LDDGDYLCEDGCQNNCPACTPGQAQHYEGDRLCGM*LTK.L	4	4.91	0.50	-0.45
IPI00242956	IgGFc-binding protein precursor	K.LDGPFAVCHDTLDPRPFLEQCVYDLCVVGGER.L	3	1.82	0.12	-3.65
IPI00242956	IgGFc-binding protein precursor	K.LDGPFAVCHDTLDPRPFLEQCVYDLCVVGGER.L	4	3.99	0.26	-3.05
IPI00242956	IgGFc-binding protein precursor	K.LDPQGAVR.D	2	1.90	0.10	-3.35
IPI00242956	IgGFc-binding protein precursor	K.LDSLVAQQLQSK.N	2	3.50	0.26	-4.29
IPI00242956	IgGFc-binding protein precursor	K.LPVVLANGQIR.A	1	2.28	0.09	-1.70
IPI00242956	IgGFc-binding protein precursor	K.LPVVLANGQIR.A	2	3.78	0.35	-2.61
IPI00242956	IgGFc-binding protein precursor	K.LTYNHGGITGSR.G	1	3.19	0.37	-3.73
IPI00242956	IgGFc-binding protein precursor	K.LTYNHGGITGSR.G	2	3.58	0.52	-3.51
IPI00242956	IgGFc-binding protein precursor	K.LTYNHGGITGSR.G	3	1.83	0.15	-2.76
IPI00242956	IgGFc-binding protein precursor	K.NAAGDLQR.L	2	2.16	0.16	-2.98
IPI00242956	IgGFc-binding protein precursor	K.NTGREEFLTAFLQNYQLAY.S	2	3.70	0.51	-3.20
IPI00242956	IgGFc-binding protein precursor	K.NTGREEFLTAFLQNYQLAYSK.A	2	4.99	0.52	-4.18
IPI00242956	IgGFc-binding protein precursor	K.NTGREEFLTAFLQNYQLAYSK.A	3	3.74	0.32	-4.69
IPI00242956	IgGFc-binding protein precursor	K.VAVIVSNDHAGK.L	1	2.90	0.38	-2.70
IPI00242956	IgGFc-binding protein precursor	K.VAVIVSNDHAGK.L	2	3.44	0.33	-2.47
IPI00242956	IgGFc-binding protein precursor	K.VAVIVSNDHAGK.L	3	2.45	0.09	-2.15
IPI00242956	IgGFc-binding protein precursor	K.VPSSYAEALCGLCGNFNGDPADDLALR.G	3	5.15	0.54	-6.97
IPI00242956	IgGFc-binding protein precursor	K.VRVNGVLTALPVSVADGR.I	2	1.88	0.15	-2.56
IPI00242956	IgGFc-binding protein precursor	K.VRVNGVLTALPVSVADGR.I	3	2.47	0.16	-6.20
IPI00242956	IgGFc-binding protein precursor	K.VTVNGVDM*K.L	1	2.34	0.15	-4.25
IPI00242956	IgGFc-binding protein precursor	K.VTVNGVDM*K.L	2	3.19	0.29	-3.21
IPI00242956	IgGFc-binding protein precursor	K.VTVNGVDM*KLPVVLANGQIR.A	3	3.95	0.38	-2.80
IPI00242956	IgGFc-binding protein precursor	K.YQKEEFCGLLSSPTGPLSSCHK.L	3	5.01	0.21	
IPI00242956	IgGFc-binding protein precursor	N.PAVSYVR.V	1	1.87	0.22	0.95
IPI00242956	IgGFc-binding protein precursor	P.GWDPLCWDECR.G	2	3.31	0.27	-3.30
IPI00242956	IgGFc-binding protein precursor	R.APGWDPLCWDECR.G	2	3.78	0.54	-3.91
IPI00242956	IgGFc-binding protein precursor	R.ASQHGSDVVIETDFGLR.V	2	4.82	0.58	-3.61
IPI00242956	IgGFc-binding protein precursor	R.ASQHGSDVVIETDFGLR.V	3	4.18	0.36	-4.62
IPI00242956	IgGFc-binding protein precursor	R.AYSHSVSLTR.G	1	2.60	0.30	-4.38
IPI00242956	IgGFc-binding protein precursor	R.AYSHSVSLTR.G	2	2.53	0.23	-3.65
IPI00242956	IgGFc-binding protein precursor	R.CLANGGIHYITLDGR.V	2	3.76	0.39	-4.29
IPI00242956	IgGFc-binding protein precursor	R.CLANGGIHYITLDGR.V	3	3.44	0.30	-2.83
IPI00242956	IgGFc-binding protein precursor	R.CPGLQNTIPWYR.V	2	4.01	0.41	-2.47
IPI00242956	IgGFc-binding protein precursor	R.CSCSSSSGLTCQAAGCPPGR.V	2	5.38	0.64	-2.58
IPI00242956	IgGFc-binding protein precursor	R.EYPGQVLVDDVLQYLPFQAADGQVQVFR.Q	3	6.98	0.56	-4.69
IPI00242956	IgGFc-binding protein precursor	R.FAVLQENVAWGNGR.V	2	4.53	0.35	0.42
IPI00242956	IgGFc-binding protein precursor	R.GATTSPGVYELSSR.C	2	3.45	0.40	-4.29
IPI00242956	IgGFc-binding protein precursor	R.GATTSPGVYELSSR.C	3	2.73	0.17	-3.18
IPI00242956	IgGFc-binding protein precursor	R.GEVGFVLVDNQR.S	2	3.08	0.28	2.04
IPI00242956	IgGFc-binding protein precursor	R.GNPAVSYVR.V	1	2.11	0.28	-1.80

IPI00242956	IgGFc-binding protein precursor	R.GNPAVSYVR.V	2	3.20	0.42	-0.25
IPI00242956	IgGFc-binding protein precursor	R.GSQAVSYTR.S	1	2.35	0.35	-0.74
IPI00242956	IgGFc-binding protein precursor	R.GSQAVSYTR.S	2	3.49	0.27	-1.61
IPI00242956	IgGFc-binding protein precursor	R.GSQTVSYTR.A	1	1.78	0.21	-0.51
IPI00242956	IgGFc-binding protein precursor	R.GSQTVSYTR.A	2	2.10	0.18	1.69
IPI00242956	IgGFc-binding protein precursor	R.ISVAQGASK.A	1	1.85	0.21	-2.50
IPI00242956	IgGFc-binding protein precursor	R.ISVAQGASK.A	2	2.63	0.25	-3.51
IPI00242956	IgGFc-binding protein precursor	R.ISVTQGASK.A	1	1.55	0.10	-2.11
IPI00242956	IgGFc-binding protein precursor	R.ISVTQGASK.A	2	2.62	0.12	-2.03
IPI00242956	IgGFc-binding protein precursor	R.KFDFQGTCNYVLATTGCPGVSTQGLTPFTVTTK.N	3	6.10	0.41	
IPI00242956	IgGFc-binding protein precursor	R.LLFDGDAHLLM*SIPSPFR.G	2	4.63	0.52	-4.30
IPI00242956	IgGFc-binding protein precursor	R.LLISSLSESPASVSILSQADNTSK.K	2	5.09	0.59	-4.13
IPI00242956	IgGFc-binding protein precursor	R.LLISSLSESPASVSILSQADNTSK.K	3	3.34	0.24	-4.04
IPI00242956	IgGFc-binding protein precursor	R.LLISSLSESPASVSILSQADNTSKK.V	3	3.86	0.48	-2.96
IPI00242956	IgGFc-binding protein precursor	R.LPVSLSEGR.L	2	2.71	0.25	-1.42
IPI00242956	IgGFc-binding protein precursor	R.LRVPAAYAGSLCGLCGNYNQDPADDLK.A	3	5.84	0.52	-2.50
IPI00242956	IgGFc-binding protein precursor	R.NEVTYDPYLVLIPDVAAYCPAYVVK.S	2	4.40	0.39	-5.34
IPI00242956	IgGFc-binding protein precursor	R.NEVTYDPYLVLIPDVAAYCPAYVVK.S	3	6.03	0.49	-5.86
IPI00242956	IgGFc-binding protein precursor	R.REYPGQVLVDDVLQYLPFQAADGQVQVFR.Q	3	6.84	0.54	-4.66
IPI00242956	IgGFc-binding protein precursor	R.RVSYVGLVTVR.A	2	2.97	0.29	-3.49
IPI00242956	IgGFc-binding protein precursor	R.RVSYVGLVTVR.A	3	3.43	0.23	-4.14
IPI00242956	IgGFc-binding protein precursor	R.SLAAYTAACQAAGVAVKPWR.T	3	3.94	0.42	-4.87
IPI00242956	IgGFc-binding protein precursor	R.SPANCPLSCPANSR.Y	2	3.82	0.39	-2.28
IPI00242956	IgGFc-binding protein precursor	R.SRLPVSLSEGR.L	2	2.61	0.28	-2.79
IPI00242956	IgGFc-binding protein precursor	R.TCQGSCAALSGLTGCTTR.C	2	5.43	0.51	-2.68
IPI00242956	IgGFc-binding protein precursor	R.TPDGSLLVR.Q	2	3.29	0.28	-3.89
IPI00242956	IgGFc-binding protein precursor	R.VAYDLVYYVR.V	1	2.84	0.34	-2.79
IPI00242956	IgGFc-binding protein precursor	R.VAYDLVYYVR.V	2	3.81	0.27	-3.57
IPI00242956	IgGFc-binding protein precursor	R.VDLPAEK.L	1	1.43	0.08	-3.43
IPI00242956	IgGFc-binding protein precursor	R.VDVTLPSSYHGAVCGLCGNM*DR.N	3	2.52	0.21	-2.53
IPI00242956	IgGFc-binding protein precursor	R.VLVENEHRGSQTVSYTR.A	2	3.33	0.33	-4.62
IPI00242956	IgGFc-binding protein precursor	R.VLVENEHRGSQTVSYTR.A	3	3.64	0.47	-3.69
IPI00242956	IgGFc-binding protein precursor	R.VLVENEHRGSQTVSYTR.A	4	2.67	0.10	-2.14
IPI00242956	IgGFc-binding protein precursor	R.VNGVLTALPVSVADGR.I	2	5.00	0.42	-3.17
IPI00242956	IgGFc-binding protein precursor	R.VPAAYAASLCGLCGNYNQDPADDLK.A	3	5.58	0.37	-4.10
IPI00242956	IgGFc-binding protein precursor	R.VPAAYAGSLCGLCGNYNQDPADDLK.A	3	6.63	0.45	-4.08
IPI00242956	IgGFc-binding protein precursor	R.VSYVGLVTVR.A	2	4.07	0.39	-1.13
IPI00242956	IgGFc-binding protein precursor	R.VTAKVPSSYAEALCGLCGNFNGDPADDLALR.G	3	6.93	0.64	-3.07
IPI00242956	IgGFc-binding protein precursor	R.VTLQPYNVAQLQSSVDLSGSK.V	2	5.67	0.60	-4.75
IPI00242956	IgGFc-binding protein precursor	R.VTLQPYNVAQLQSSVDLSGSK.V	3	6.34	0.59	-5.41
IPI00242956	IgGFc-binding protein precursor	R.VTVPGNYYQLM*CGLCGNYNGDPK.D	3	3.45	0.20	-1.87
IPI00242956	IgGFc-binding protein precursor	R.VVTVAALGTNISIHKDEIGK.V	3	2.88	0.27	-3.75

IPI00242956	IgGFc-binding protein precursor	R.VVTVAALGTNISIHKDEIGK.V	4	2.65	0.33	-2.84
IPI00242956	IgGFc-binding protein precursor	R.VVTVAALGTNISIHKDEIGKVR.V	3	3.69	0.47	-4.24
IPI00242956	IgGFc-binding protein precursor	R.VYDLHGSCSYVLAQVCHPKPGDEDFSIVLEK.N	4	4.79	0.35	-3.98
IPI00242956	IgGFc-binding protein precursor	R.YDLAFVVASQATK.L	2	4.46	0.50	-3.69
IPI00242956	IgGFc-binding protein precursor	R.YDLAFVVASQATK.L	3	4.46	0.29	-2.18
IPI00242956	IgGFc-binding protein precursor	R.YYPLGEVFYPGPECER.R	2	5.02	0.61	-3.09
IPI00243221	nardilysin (N-arginine dibasic convertase) isoform a	K.IENLTEEAFNTQVTALIK.L	2	2.80	0.13	
IPI00243338	24 kDa protein	-.M*EKHHVPSDFNVNVK.V	2	2.33	0.12	
IPI00243451	Liver-specific organic anion transporter 3TM12	K.KSHGKDTKVLENERQVM*DEA.N	2	3.49	0.15	-0.52
IPI00243995	Serine/threonine-protein kinase Nek5	K.HIHDRKILHRDIKAQNIFLSK.N	2	3.01	0.07	
IPI00245940	immunoglobulin superfamily 5 like	K.ERSTADTLPDLEEWKSAAGLR.W	3	2.77	0.19	
IPI00246058	PDCD6IP protein	K.NLATAYDNFVELVANLK.E	2	5.25	0.49	-5.28
IPI00246058	PDCD6IP protein	K.NLATAYDNFVELVANLK.E	3	4.08	0.32	-3.86
IPI00246058	PDCD6IP protein	K.STPVNVPISQK.F	2	2.15	0.06	-1.32
IPI00246058	PDCD6IP protein	K.TM*QGSEVVNVLK.S	2	2.44	0.10	-0.41
IPI00246058	PDCD6IP protein	R.EATTLANGVLASLNLPAAIEDVSGDTVPQSILTK.S	3	3.09	0.27	-3.21
IPI00246058	PDCD6IP protein	R.EPTVDISPDTVGTLSLIMLAQAQEVFFLK.A	3	3.49	0.34	-2.49
IPI00246058	PDCD6IP protein	R.LLDEEEATDNDLR.A	2	3.82	0.43	-2.06
IPI00246058	PDCD6IP protein	R.TPSNELYKPLRAEGTNFR.T	3	2.43	0.15	-3.77
IPI00247243	31 kDa protein	A.AEAVSHIQSSGPR.R	2	3.41	0.38	-3.66
IPI00247243	31 kDa protein	A.SPPSGQAVLLR.Q	1	2.57	0.33	-4.39
IPI00247243	31 kDa protein	E.AVSHIQSSGPR.R	2	3.05	0.33	-2.82
IPI00247243	31 kDa protein	K.EPPRELLHELALSVPGAR.S	2	1.68	0.28	-4.15
IPI00247243	31 kDa protein	K.EPPRELLHELALSVPGAR.S	3	3.87	0.42	-5.76
IPI00247243	31 kDa protein	R.EGDDIEM*PCAFR.A	2	3.03	0.42	-1.75
IPI00247243	31 kDa protein	R.ELLHELALSVPGAR.S	2	2.76	0.11	-3.54
IPI00247243	31 kDa protein	R.HGPASAANANNAGAASR.T	2	4.06	0.50	-4.56
IPI00247243	31 kDa protein	R.LQDEGVYECR.V	2	2.99	0.26	-2.04
IPI00247243	31 kDa protein	R.VQGNDISHR.L	2	2.68	0.17	-0.82
IPI00247243	31 kDa protein	R.VSDYSDDDTQEHK.A	2	4.01	0.56	-3.65
IPI00247243	31 kDa protein	R.VSDYSDDDTQEHK.A	3	3.47	0.45	-1.14
IPI00247243	31 kDa protein	R.VSDYSDDDTQEHKAQAM*LR.V	3	3.52	0.49	-3.30
IPI00248596	similar to slit homolog 1	R.SVQYASLSR.F	2	2.65	0.16	-2.19
IPI00249982	Isoform 1 of Death-inducer obliterator 1	K.AAAMAASKK.T	2	2.80	0.14	
IPI00250724	Protein kinase-like domain containing protein	K.AISDIALSFLDM*VNHFDSDFSHR.L	3	3.70	0.32	-1.10
IPI00250724	Protein kinase-like domain containing protein	R.VNNNLQVICDK.I	2	3.28	0.23	-2.31
IPI00251507	Isoform IB of Synapsin-1	K.LWVDTCSEIFGGLDICAVEALHGK.D	3	2.91	0.33	-1.32
IPI00251507	Isoform IB of Synapsin-1	R.QASQAGPVPR.T	2	2.52	0.28	-0.19
IPI00251507	Isoform IB of Synapsin-1	R.QGPPQKPPGPAGPTR.Q	3	2.10	0.12	-2.16
IPI00251507	Isoform IB of Synapsin-1	R.QTSVSGPAPPK.A	2	2.02	0.24	-2.48
IPI00251596	Isoform 1 of Collagen alpha-1(XXIII) chain	R.VAALEEERELLRR.A	3	2.66	0.12	-2.20

IPI00252731	Isoform DPPX-S of Dipeptidyl aminopeptidase-like protein 6	K.IHDPEAK.W	2	1.84	0.15	-3.29
IPI00252731	Isoform DPPX-S of Dipeptidyl aminopeptidase-like protein 6	K.ILAYDEK.G	2	1.93	0.12	-2.70
IPI00252731	Isoform DPPX-S of Dipeptidyl aminopeptidase-like protein 6	K.ILAYDEKGNKIYFLSTEDLPR.R	4	3.15	0.20	-1.82
IPI00252731	Isoform DPPX-S of Dipeptidyl aminopeptidase-like protein 6	K.IPHGDPQSLDPPEVSNAK.L	3	2.53	0.12	-1.46
IPI00252731	Isoform DPPX-S of Dipeptidyl aminopeptidase-like protein 6	K.IYFLSTEDLPR.R	2	3.57	0.27	-3.03
IPI00252731	Isoform DPPX-S of Dipeptidyl aminopeptidase-like protein 6	K.KKVTVEDLFSEDFK.I	2	4.63	0.46	-3.17
IPI00252731	Isoform DPPX-S of Dipeptidyl aminopeptidase-like protein 6	K.KKVTVEDLFSEDFKIHDPEAK.W	5	2.62	0.13	-2.40
IPI00252731	Isoform DPPX-S of Dipeptidyl aminopeptidase-like protein 6	K.LYASAFSER.Y	2	2.98	0.29	-1.06
IPI00252731	Isoform DPPX-S of Dipeptidyl aminopeptidase-like protein 6	K.M*FDLETNEHVKK.A	2	3.65	0.34	-0.80
IPI00252731	Isoform DPPX-S of Dipeptidyl aminopeptidase-like protein 6	K.VTVEDLFSEDFK.I	2	1.97	0.16	-3.14
IPI00252731	Isoform DPPX-S of Dipeptidyl aminopeptidase-like protein 6	K.VTVEDLFSEDFKIHDPEAK.W	3	3.37	0.22	-2.52
IPI00252731	Isoform DPPX-S of Dipeptidyl aminopeptidase-like protein 6	K.VTVEDLFSEDFKIHDPEAK.W	4	2.50	0.25	-0.69
IPI00252731	Isoform DPPX-S of Dipeptidyl aminopeptidase-like protein 6	L.TPAEDNSLSQK.K	2	2.93	0.34	-2.15
IPI00252731	Isoform DPPX-S of Dipeptidyl aminopeptidase-like protein 6	R.LGLLEEKDQM*EAVR.T	3	3.77	0.36	-0.69
IPI00252731	Isoform DPPX-S of Dipeptidyl aminopeptidase-like protein 6	R.SIINFFVECFR.I	2	3.72	0.45	-3.35
IPI00252731	Isoform DPPX-S of Dipeptidyl aminopeptidase-like protein 6	R.TM*LKEQYIDR.T	2	1.99	0.12	-2.69
IPI00252731	Isoform DPPX-S of Dipeptidyl aminopeptidase-like protein 6	R.VSALEEQQFLIIHPTADEK.I	3	4.23	0.45	-3.99
IPI00252731	Isoform DPPX-S of Dipeptidyl aminopeptidase-like protein 6	S.VILLTPAEDNSLSQK.K	2	3.49	0.38	-1.75
IPI00252845	SYT9 protein	-.MLAKVVEGDLAFKGGR.D	2	1.96	0.05	1.80
IPI00253281	Isoform 2 of Epidermal growth factor receptor kinase substrate 8-like protein 1	R.IWGSSQDEAELIR.E	2	1.76	0.09	-6.75
IPI00255145	hypothetical protein	K.QAMSPEDKKRAVKYSM*SHIAQIPVKHDSLK.E	3	4.08	0.10	
IPI00257508	Dihydropyrimidinase-related protein 2	K.AAAFVTSPPLSPDPTTPDFLNSLLSCGDLQVTGSAHCTFNTAQK.A	4	2.42	0.11	-1.61
IPI00257508	Dihydropyrimidinase-related protein 2	R.FQM*PDQGM*TSADDFFQGTK.A	2	2.44	0.12	

IPI00257508	Dihydropyrimidinase-related protein 2	R.IVNDDQSFYADIYMEDGLIK.Q	2	5.84	0.53	-2.08
IPI00257508	Dihydropyrimidinase-related protein 2	R.IVNDDQSFYADIYMEDGLIK.Q	3	3.23	0.23	-1.74
IPI00257508	Dihydropyrimidinase-related protein 2	R.NLHQSGFSLSGAQIDDNIPR.R	3	2.51	0.11	-1.66
IPI00257882	Xaa-Pro dipeptidase	K.VPLALFALNR.Q	2	4.28	0.38	-1.35
IPI00257882	Xaa-Pro dipeptidase	K.YAVDDVQYVDEIASVLTSQKPSVLLTLR.G	3	5.45	0.49	-4.83
IPI00259102	Mammalian ependymin-related protein 1 precursor	K.DGVM*FQIDQATK.Q	2	4.34	0.42	-5.08
IPI00259102	Mammalian ependymin-related protein 1 precursor	R.ALLSYDGLNQR.V	2	3.65	0.36	-3.94
IPI00259102	Mammalian ependymin-related protein 1 precursor	R.LFEYILLYK.D	2	3.54	0.21	-5.11
IPI00259102	Mammalian ependymin-related protein 1 precursor	R.QVM*YQQSSGR.N	2	2.61	0.42	-2.43
IPI00259102	Mammalian ependymin-related protein 1 precursor	R.SYETWIGIYTVK.D	2	4.04	0.33	-7.53
IPI00260755	similar to Rho GTPase activating protein 18	K.CSLPKFTVPKGRLGVTR.I	2	2.60	0.14	
IPI00289058	Ly-6/neurotoxin-like protein 1 precursor	A.LDCHVCAYNGDNCFNPM*R.C	2	4.20	0.63	-1.99
IPI00289058	Ly-6/neurotoxin-like protein 1 precursor	A.LDCHVCAYNGDNCFNPM*R.C	3	3.91	0.44	-1.28
IPI00289058	Ly-6/neurotoxin-like protein 1 precursor	R.CFETVYDGYSK.H	1	3.07	0.44	-3.03
IPI00289058	Ly-6/neurotoxin-like protein 1 precursor	R.CFETVYDGYSK.H	2	4.70	0.54	-3.73
IPI00289058	Ly-6/neurotoxin-like protein 1 precursor	R.CPAM*VAYCM*TTR.T	2	4.22	0.40	-3.81
IPI00289058	Ly-6/neurotoxin-like protein 1 precursor	R.CPAM*VAYCM*TTR.T	3	3.79	0.41	-2.04
IPI00289058	Ly-6/neurotoxin-like protein 1 precursor	R.CPAMVAYCM*TTR.T	2	2.76	0.29	
IPI00289058	Ly-6/neurotoxin-like protein 1 precursor	R.TYYTPTR.M	1	1.72	0.28	-1.29
IPI00289058	Ly-6/neurotoxin-like protein 1 precursor	R.TYYTPTR.M	2	1.72	0.31	-1.70
IPI00289083	Isoform 1 of VWFA and cache domain-containing protein 1 precursor	A.DFSILDEAQVLASQM*R.R	2	4.91	0.43	-3.33
IPI00289083	Isoform 1 of VWFA and cache domain-containing protein 1 precursor	A.DFSILDEAQVLASQM*R.R	3	4.73	0.46	-2.55
IPI00289083	Isoform 1 of VWFA and cache domain-containing protein 1 precursor	K.DAAQVILSAIDEHDKISVLTVADTVR.T	4	4.29	0.32	-3.07
IPI00289083	Isoform 1 of VWFA and cache domain-containing protein 1 precursor	K.GSM*M*VLNQLSNLETTVGR.F	2	5.63	0.54	-1.43
IPI00289083	Isoform 1 of VWFA and cache domain-containing protein 1 precursor	K.GSM*M*VLNQLSNLETTVGR.F	3	4.69	0.37	-2.68
IPI00289083	Isoform 1 of VWFA and cache domain-containing protein 1 precursor	K.ISNGESEVQQLAK.K	2	4.22	0.27	-3.53
IPI00289083	Isoform 1 of VWFA and cache domain-containing protein 1 precursor	K.M*STFVSSVK.S	2	2.58	0.24	-1.80
IPI00289083	Isoform 1 of VWFA and cache domain-containing protein 1 precursor	K.NLNTVPSSK.L	2	2.15	0.15	-0.31
IPI00289083	Isoform 1 of VWFA and cache domain-containing protein 1 precursor	K.SSDSPTQHAVGFQK.A	2	3.55	0.44	-3.48
IPI00289083	Isoform 1 of VWFA and cache domain-containing protein 1 precursor	K.TFLSPATSETK.R	2	2.67	0.23	-0.80
IPI00289083	Isoform 1 of VWFA and cache domain-containing protein 1 precursor	K.TFLSPATSETKR.K	2	2.35	0.09	-2.82

IPI00289083	Isoform 1 of VWFA and cache domain-containing protein 1 precursor	K.WQYFSSEEGIFTVFPAHK.F	3	3.69	0.46	-1.91
IPI00289083	Isoform 1 of VWFA and cache domain-containing protein 1 precursor	R.AFNPGRDLNSVLADNLK.S	3	3.68	0.37	-2.63
IPI00289083	Isoform 1 of VWFA and cache domain-containing protein 1 precursor	R.CFIM*EDR.G	2	1.62	0.07	-0.42
IPI00289083	Isoform 1 of VWFA and cache domain-containing protein 1 precursor	R.IFNSFVYTEK.I	2	3.75	0.30	-2.97
IPI00289083	Isoform 1 of VWFA and cache domain-containing protein 1 precursor	R.LIANPGLK.F	1	1.77	0.05	-3.57
IPI00289083	Isoform 1 of VWFA and cache domain-containing protein 1 precursor	R.LSTTVNSR.A	2	1.89	0.15	-0.79
IPI00289083	Isoform 1 of VWFA and cache domain-containing protein 1 precursor	R.M*VEHYTAYLSDNTR.L	2	4.24	0.50	-3.87
IPI00289083	Isoform 1 of VWFA and cache domain-containing protein 1 precursor	R.M*VEHYTAYLSDNTR.L	3	3.87	0.20	-3.26
IPI00289083	Isoform 1 of VWFA and cache domain-containing protein 1 precursor	R.SRPIYVSTVRPQSK.H	3	3.27	0.14	-2.83
IPI00289083	Isoform 1 of VWFA and cache domain-containing protein 1 precursor	R.YLDVVNR.N	2	2.48	0.22	-2.01
IPI00289204	Reticulon-4 receptor precursor	K.VTTSCPQQGLQAVPVGIPAASQR.I	2	4.91	0.61	-4.35
IPI00289204	Reticulon-4 receptor precursor	K.VTTSCPQQGLQAVPVGIPAASQR.I	3	3.79	0.29	-2.77
IPI00289204	Reticulon-4 receptor precursor	P.CPGACVCYNEPK.V	2	3.70	0.44	-3.60
IPI00289204	Reticulon-4 receptor precursor	R.ATDEEPLGLPK.C	2	3.42	0.43	-3.58
IPI00289204	Reticulon-4 receptor precursor	R.CGLQELGPGLFR.G	2	4.64	0.37	-3.60
IPI00289204	Reticulon-4 receptor precursor	R.GLAALQYLYLQDNALQALPDDTFR.D	2	5.63	0.48	-5.33
IPI00289204	Reticulon-4 receptor precursor	R.GLAALQYLYLQDNALQALPDDTFR.D	3	5.53	0.49	-5.08
IPI00289204	Reticulon-4 receptor precursor	R.GSSSEVPCSLPQR.L	2	2.50	0.22	-1.79
IPI00289204	Reticulon-4 receptor precursor	R.IDAAAFTGLALLEQLDLSDNAQLR.S	2	6.03	0.48	-3.48
IPI00289204	Reticulon-4 receptor precursor	R.IDAAAFTGLALLEQLDLSDNAQLR.S	3	8.08	0.54	-4.86
IPI00289204	Reticulon-4 receptor precursor	R.IDAAAFTGLALLEQLDLSDNAQLR.S	4	5.96	0.39	-2.90
IPI00289204	Reticulon-4 receptor precursor	R.ISHVPAASFR.A	1	2.96	0.26	-4.25
IPI00289204	Reticulon-4 receptor precursor	R.ISHVPAASFR.A	2	2.27	0.19	-2.86
IPI00289204	Reticulon-4 receptor precursor	R.LHTLHLDR.C	2	2.15	0.18	-2.18
IPI00289204	Reticulon-4 receptor precursor	R.LLLHQNR.V	1	2.09	0.07	-4.18
IPI00289204	Reticulon-4 receptor precursor	R.LLLHQNR.V	2	2.29	0.10	-2.96
IPI00289204	Reticulon-4 receptor precursor	R.SVDPATFHGLGR.L	1	1.41	0.22	-2.06
IPI00289204	Reticulon-4 receptor precursor	R.VAHVHPHAFR.D	2	2.63	0.32	-3.23
IPI00289271	Liprin-alpha-2	Q.LNSALPQDIESLTGGLAGSK.G	3	3.54	0.16	-3.21
IPI00289329	Ephrin type-B receptor 3 precursor	K.VDTIAPDESFSR.L	2	3.01	0.31	-2.81
IPI00289334	Isoform 1 of Filamin-B	R.QMQLENVSVALEFLDRESIK.L	3	2.96	0.24	-3.88
IPI00289501	Neurosecretory protein VGF precursor	A.APPGRPEAQPPPLSSEHKEPVAGDAVPGPK.D	2	3.86	0.56	-3.46

IPI00289501	Neurosecretory protein VGF precursor	A.APPGRPEAQPPPLSSEHKEPVAGDAVPGPK.D	3	5.74	0.55	-4.83
IPI00289501	Neurosecretory protein VGF precursor	A.APPGRPEAQPPPLSSEHKEPVAGDAVPGPKDGSAPEVR.G	4	5.62	0.48	-4.09
IPI00289501	Neurosecretory protein VGF precursor	A.AVLLQALDRPASPPAPSGSQQGPEEEAAEALLTETVR.S	3	6.61	0.57	-2.86
IPI00289501	Neurosecretory protein VGF precursor	A.DLASDLLLQYLLQGGAR.Q	2	5.03	0.40	-3.41
IPI00289501	Neurosecretory protein VGF precursor	A.DLASDLLLQYLLQGGAR.Q	3	5.23	0.33	-1.30
IPI00289501	Neurosecretory protein VGF precursor	A.LAAVLLQALDRPASPPAPSGSQQGPEEEAAEALLTETVR.S	4	4.75	0.41	-4.82
IPI00289501	Neurosecretory protein VGF precursor	A.LDRPASPPAPSGSQQGPEEEAAEALLTETVR.S	3	6.57	0.52	-4.81
IPI00289501	Neurosecretory protein VGF precursor	A.PPGRPEAQPPPLSSEHKEPVAGDAVPGPK.D	3	6.01	0.49	-4.55
IPI00289501	Neurosecretory protein VGF precursor	A.PPGRPEAQPPPLSSEHKEPVAGDAVPGPK.D	4	4.94	0.40	-4.26
IPI00289501	Neurosecretory protein VGF precursor	A.PPGRPEAQPPPLSSEHKEPVAGDAVPGPKDGSAPEVR.G	4	5.00	0.43	-4.26
IPI00289501	Neurosecretory protein VGF precursor	A.SDLLLQYLLQGGAR.Q	2	4.18	0.35	-3.86
IPI00289501	Neurosecretory protein VGF precursor	A.VLLQALDRPASPPAPSGSQQGPEEEAAEALLTETVR.S	4	4.93	0.37	-4.31
IPI00289501	Neurosecretory protein VGF precursor	D.PSEELEALASLLQELR.D	2	4.82	0.29	-4.73
IPI00289501	Neurosecretory protein VGF precursor	D.PSEELEALASLLQELR.D	3	4.35	0.13	-2.64
IPI00289501	Neurosecretory protein VGF precursor	G.RPEAQPPPLSSEHKEPVAGDAVPGPK.D	3	5.38	0.49	-3.83
IPI00289501	Neurosecretory protein VGF precursor	G.RPEAQPPPLSSEHKEPVAGDAVPGPK.D	4	6.17	0.57	-4.29
IPI00289501	Neurosecretory protein VGF precursor	K.AYQGVAAPFPK.A	1	2.71	0.37	-2.95
IPI00289501	Neurosecretory protein VGF precursor	K.AYQGVAAPFPK.A	2	3.94	0.38	-2.88
IPI00289501	Neurosecretory protein VGF precursor	K.AYQGVAAPFPKA.R	1	2.62	0.34	-1.51
IPI00289501	Neurosecretory protein VGF precursor	K.AYQGVAAPFPKA.R	2	4.51	0.44	-1.69
IPI00289501	Neurosecretory protein VGF precursor	K.DGSAPEVR.G	1	1.55	0.19	-2.72
IPI00289501	Neurosecretory protein VGF precursor	K.DGSAPEVR.G	2	2.38	0.22	-3.92
IPI00289501	Neurosecretory protein VGF precursor	K.FGEGVSSPK.T	1	1.94	0.11	-3.61
IPI00289501	Neurosecretory protein VGF precursor	K.FGEGVSSPK.T	2	3.60	0.25	-3.25
IPI00289501	Neurosecretory protein VGF precursor	K.LHLPADDVVS.I	1	1.85	0.28	-1.93
IPI00289501	Neurosecretory protein VGF precursor	K.LHLPADDVVSIIEEV.E	2	3.09	0.26	-5.65
IPI00289501	Neurosecretory protein VGF precursor	K.LHLPADDVVSIIEEVE.E	2	3.80	0.34	-5.08
IPI00289501	Neurosecretory protein VGF precursor	K.LHLPADDVVSIIEEVEE.K	2	4.79	0.44	-8.31
IPI00289501	Neurosecretory protein VGF precursor	K.LHLPADDVVSIIEEVEE.K	3	3.67	0.31	-1.97
IPI00289501	Neurosecretory protein VGF precursor	K.LHLPADDVVSIIEEVEEK.R	3	3.95	0.31	-4.91
IPI00289501	Neurosecretory protein VGF precursor	K.RQQETAAAETETR.T	2	3.93	0.33	-2.09
IPI00289501	Neurosecretory protein VGF precursor	K.THLGEALAPLSK.A	1	3.32	0.34	-3.16
IPI00289501	Neurosecretory protein VGF precursor	K.THLGEALAPLSK.A	2	3.55	0.48	-2.99
IPI00289501	Neurosecretory protein VGF precursor	K.THLGEALAPLSK.A	3	3.56	0.08	-4.04
IPI00289501	Neurosecretory protein VGF precursor	K.THLGEALAPLSKA.Y	2	3.28	0.43	-3.50
IPI00289501	Neurosecretory protein VGF precursor	L.ADLASDLLLQYLLQGGAR.Q	3	5.32	0.37	-4.14
IPI00289501	Neurosecretory protein VGF precursor	L.ASDLLLQYLLQGGAR.Q	2	4.86	0.34	-3.64
IPI00289501	Neurosecretory protein VGF precursor	L.DRPASPPAPSGSQQGPEEEAAEALLTETVR.S	3	5.88	0.49	-4.28
IPI00289501	Neurosecretory protein VGF precursor	L.LQALDRPASPPAPSGSQQGPEEEAAEALLTETVR.S	3	5.38	0.55	-4.09
IPI00289501	Neurosecretory protein VGF precursor	L.SKAYQGVAAPFPK.A	2	4.07	0.39	-3.01
IPI00289501	Neurosecretory protein VGF precursor	N.GPEASDPSEELEALASLLQELR.D	2	5.41	0.53	-5.31
IPI00289501	Neurosecretory protein VGF precursor	N.GPEASDPSEELEALASLLQELR.D	3	4.09	0.30	-5.25

IPI00289501	Neurosecretory protein VGF precursor	N.SEPQDEGELFQGVDPR.A	2	4.65	0.35	-5.02
IPI00289501	Neurosecretory protein VGF precursor	N.SEPQDEGELFQGVDPR.A	3	3.82	0.35	-3.16
IPI00289501	Neurosecretory protein VGF precursor	P.DSGPLPETHKFGEGVSSPK.T	2	3.82	0.49	-3.92
IPI00289501	Neurosecretory protein VGF precursor	P.EASDPSEELEALASLLQELR.D	2	4.94	0.43	-2.85
IPI00289501	Neurosecretory protein VGF precursor	P.ERAPLPPPAPSQFQAR.M	3	4.67	0.33	-2.23
IPI00289501	Neurosecretory protein VGF precursor	P.ETHKFGEGVSSPK.T	2	3.16	0.28	-3.92
IPI00289501	Neurosecretory protein VGF precursor	P.GRPEAQPPPLSSEHKEPVAGDAVPGPK.D	2	4.22	0.51	-3.59
IPI00289501	Neurosecretory protein VGF precursor	P.GRPEAQPPPLSSEHKEPVAGDAVPGPK.D	3	5.49	0.44	-4.07
IPI00289501	Neurosecretory protein VGF precursor	P.GRPEAQPPPLSSEHKEPVAGDAVPGPK.D	4	6.54	0.53	-3.66
IPI00289501	Neurosecretory protein VGF precursor	P.GRPEAQPPPLSSEHKEPVAGDAVPGPKDGSAPEVR.G	3	6.80	0.58	0.47
IPI00289501	Neurosecretory protein VGF precursor	P.GRPEAQPPPLSSEHKEPVAGDAVPGPKDGSAPEVR.G	4	5.08	0.51	-2.47
IPI00289501	Neurosecretory protein VGF precursor	P.PGRPEAQPPPLSSEHKEPVAGDAVPGPK.D	3	5.89	0.53	-4.37
IPI00289501	Neurosecretory protein VGF precursor	P.PGRPEAQPPPLSSEHKEPVAGDAVPGPK.D	4	6.32	0.51	-3.60
IPI00289501	Neurosecretory protein VGF precursor	P.PLSSEHKEPVAGDAVPGPK.D	2	4.44	0.46	-2.70
IPI00289501	Neurosecretory protein VGF precursor	P.PPLSSEHKEPVAGDAVPGPK.D	2	4.43	0.51	-3.12
IPI00289501	Neurosecretory protein VGF precursor	P.PPLSSEHKEPVAGDAVPGPK.D	3	4.50	0.29	-2.42
IPI00289501	Neurosecretory protein VGF precursor	Q.ALDRPASPPAPSGSQQGPEEEAAEALLTETVR.S	3	6.00	0.54	-3.02
IPI00289501	Neurosecretory protein VGF precursor	Q.ARVPERAPLPPPAPSQFQAR.M	3	4.85	0.36	-4.04
IPI00289501	Neurosecretory protein VGF precursor	Q.DEGELFQGVDPR.A	2	3.61	0.27	-2.97
IPI00289501	Neurosecretory protein VGF precursor	Q.TPENGPEASDPSEELEALASLLQELR.D	3	3.81	0.26	-2.64
IPI00289501	Neurosecretory protein VGF precursor	R.AAPAPTHVR.S	1	1.54	0.15	-3.29
IPI00289501	Neurosecretory protein VGF precursor	R.AAPAPTHVR.S	2	1.83	0.44	-2.96
IPI00289501	Neurosecretory protein VGF precursor	R.ALAAVLLQALDR.P	2	3.93	0.40	-3.61
IPI00289501	Neurosecretory protein VGF precursor	R.ALAAVLLQALDRPASPPAPSGSQQGPEEEAAEALLTET.V	3	3.80	0.40	-2.63
IPI00289501	Neurosecretory protein VGF precursor	R.ALAAVLLQALDRPASPPAPSGSQQGPEEEAAEALLTETV.R	3	6.07	0.58	-4.30
IPI00289501	Neurosecretory protein VGF precursor	R.ALAAVLLQALDRPASPPAPSGSQQGPEEEAAEALLTETV.R	4	5.00	0.36	-3.96
IPI00289501	Neurosecretory protein VGF precursor	R.ALAAVLLQALDRPASPPAPSGSQQGPEEEAAEALLTETVR.S	3	6.50	0.61	-4.77
IPI00289501	Neurosecretory protein VGF precursor	R.ALAAVLLQALDRPASPPAPSGSQQGPEEEAAEALLTETVR.S	4	5.11	0.53	-6.79
IPI00289501	Neurosecretory protein VGF precursor	R.ALAAVLLQALDRPASPPAPSGSQQGPEEEAAEALLTETVR.S	5	5.50	0.50	-5.50
IPI00289501	Neurosecretory protein VGF precursor	R.ALAAVLLQALDRPASPPAPSGSQQGPEEEAAEALLTETVR.S	6	4.34	0.31	-4.41
IPI00289501	Neurosecretory protein VGF precursor	R.APLPPPAPSQFQAR.M	2	2.48	0.28	-5.81
IPI00289501	Neurosecretory protein VGF precursor	R.ARQNALLFAEEEDGEAGA.E	2	5.01	0.60	-3.43
IPI00289501	Neurosecretory protein VGF precursor	R.ARQNALLFAEEEDGEAGAE.D	2	4.24	0.49	1.05
IPI00289501	Neurosecretory protein VGF precursor	R.ARQNALLFAEEEDGEAGAED.K	2	6.07	0.55	-3.37
IPI00289501	Neurosecretory protein VGF precursor	R.ASWGEFQAR.V	2	2.95	0.27	-2.89
IPI00289501	Neurosecretory protein VGF precursor	R.DFSPSSAK.R	1	1.73	0.18	-3.28
IPI00289501	Neurosecretory protein VGF precursor	R.DFSPSSAKR.Q	2	1.87	0.16	-2.23
IPI00289501	Neurosecretory protein VGF precursor	R.ESAREEEEAEQERR.G	3	3.46	0.23	-3.27
IPI00289501	Neurosecretory protein VGF precursor	R.GLQEAAEER.E	1	2.13	0.07	-2.29
IPI00289501	Neurosecretory protein VGF precursor	R.GLQEAAEER.E	2	2.42	0.07	-0.27
IPI00289501	Neurosecretory protein VGF precursor	R.GLQEAAEERESAREEEEAEQE.R	2	2.98	0.37	-3.30
IPI00289501	Neurosecretory protein VGF precursor	R.GLQEAAEERESAREEEEAEQE.R	3	3.62	0.33	0.13

IPI00289501	Neurosecretory protein VGF precursor	R.GLQEAAAEERESAREEEEAEQER.R	3	3.92	0.28	-3.12
IPI00289501	Neurosecretory protein VGF precursor	R.GLQEAAAEERESAREEEEAEQERR.G	3	3.99	0.28	-3.68
IPI00289501	Neurosecretory protein VGF precursor	R.GLQEAAAEERESAREEEEAEQERR.G	4	2.81	0.14	-4.23
IPI00289501	Neurosecretory protein VGF precursor	R.LADLASDLLLQYLLQGGAR.Q	2	5.69	0.42	-7.37
IPI00289501	Neurosecretory protein VGF precursor	R.LADLASDLLLQYLLQGGAR.Q	3	5.87	0.41	-4.13
IPI00289501	Neurosecretory protein VGF precursor	R.LADLASDLLLQYLLQGGARQ.R	2	4.47	0.33	-4.21
IPI00289501	Neurosecretory protein VGF precursor	R.LADLASDLLLQYLLQGGARQ.R	3	5.00	0.41	-4.42
IPI00289501	Neurosecretory protein VGF precursor	R.LLQQGLAQVEAG.R	2	3.38	0.25	-3.87
IPI00289501	Neurosecretory protein VGF precursor	R.LLQQGLAQVEAGR.R	2	3.90	0.42	-3.05
IPI00289501	Neurosecretory protein VGF precursor	R.LQEQEELENYIEHVLLR.R	3	2.77	0.14	-2.56
IPI00289501	Neurosecretory protein VGF precursor	R.M*PDSGPLPETH.K	2	3.26	0.47	-2.56
IPI00289501	Neurosecretory protein VGF precursor	R.M*PDSGPLPETHK.F	1	1.93	0.26	-3.75
IPI00289501	Neurosecretory protein VGF precursor	R.M*PDSGPLPETHK.F	2	3.60	0.35	-3.05
IPI00289501	Neurosecretory protein VGF precursor	R.M*PDSGPLPETHK.F	3	2.84	0.16	-3.75
IPI00289501	Neurosecretory protein VGF precursor	R.M*PDSGPLPETHKFGEGVSSPK.T	2	4.21	0.50	-4.75
IPI00289501	Neurosecretory protein VGF precursor	R.M*PDSGPLPETHKFGEGVSSPK.T	3	3.17	0.36	-2.85
IPI00289501	Neurosecretory protein VGF precursor	R.M*PDSGPLPETHKFGEGVSSPK.T	4	2.81	0.23	-2.98
IPI00289501	Neurosecretory protein VGF precursor	R.NSEPQDEGELFQGVDPR.A	2	4.47	0.43	-4.88
IPI00289501	Neurosecretory protein VGF precursor	R.NSEPQDEGELFQGVDPR.A	3	4.18	0.42	-4.02
IPI00289501	Neurosecretory protein VGF precursor	R.QAAAQEERLADLASDLLLQYLLQGGAR.Q	3	3.76	0.12	
IPI00289501	Neurosecretory protein VGF precursor	R.QNALLFAEEEDGEAGA.E	2	2.91	0.29	-3.80
IPI00289501	Neurosecretory protein VGF precursor	R.QNALLFAEEEDGEAGAED.K	2	4.73	0.53	-4.03
IPI00289501	Neurosecretory protein VGF precursor	R.QNALLFAEEEDGEAGAEDKR.S	3	3.35	0.40	-1.72
IPI00289501	Neurosecretory protein VGF precursor	R.QQETAAAETETR.T	2	3.09	0.47	-3.26
IPI00289501	Neurosecretory protein VGF precursor	R.RKEAEGTEEGGEEEDDEEM*DPQTIDSLIEL.S	3	4.02	0.38	-4.65
IPI00289501	Neurosecretory protein VGF precursor	R.RKEAEGTEEGGEEEDDEEM*DPQTIDSLIELSTK.L	3	5.88	0.58	-5.21
IPI00289501	Neurosecretory protein VGF precursor	R.RKEAEGTEEGGEEEDDEEM*DPQTIDSLIELSTK.L	4	4.87	0.45	-5.08
IPI00289501	Neurosecretory protein VGF precursor	R.RLQEQEELENYIEHVLLR.R	3	5.85	0.40	-4.66
IPI00289501	Neurosecretory protein VGF precursor	R.RLQEQEELENYIEHVLLR.R	4	5.49	0.34	-3.42
IPI00289501	Neurosecretory protein VGF precursor	R.VNLESPGPER.V	1	1.92	0.26	-2.69
IPI00289501	Neurosecretory protein VGF precursor	R.VNLESPGPER.V	2	3.05	0.24	-1.99
IPI00289501	Neurosecretory protein VGF precursor	R.VNLESPGPERVW.R	2	4.06	0.49	-2.74
IPI00289501	Neurosecretory protein VGF precursor	R.VPERAPLPPPAPSQFQA.R	2	3.21	0.33	-2.34
IPI00289501	Neurosecretory protein VGF precursor	R.VPERAPLPPPAPSQFQAR.M	2	3.86	0.46	-3.44
IPI00289501	Neurosecretory protein VGF precursor	R.VPERAPLPPPAPSQFQAR.M	3	4.17	0.36	-3.59
IPI00289501	Neurosecretory protein VGF precursor	S.EPQDEGELFQGVDPR.A	2	3.48	0.43	-3.86
IPI00289746	Isoform 2 of Serine/threonine-protein kinase PAK 1	R.DVATSPISPTENNTTPPDALTR.N	2	2.96	0.29	-1.24
IPI00289746	Isoform 2 of Serine/threonine-protein kinase PAK 1	R.NTSTM*IGAGSK.D	2	2.21	0.32	-1.30
IPI00289802	Isoform 1 of CUB and sushi domain-containing protein 2	R.GFSAQYQVK.K	2	2.12	0.30	-0.42

IPI00289802	Isoform 1 of CUB and sushi domain-containing protein 2	R.LHFTSDGNHR.Q	2	2.08	0.26	-2.81
IPI00289819	Cation-independent mannose-6-phosphate receptor precursor	K.DGAGNSFDLSSLSR.Y	2	4.53	0.45	-2.17
IPI00289819	Cation-independent mannose-6-phosphate receptor precursor	K.FLHQDIDSGQGIR.N	3	2.80	0.16	-2.35
IPI00289819	Cation-independent mannose-6-phosphate receptor precursor	K.LSGAYLVDDSDPDTSLFINVCR.D	2	5.30	0.57	-6.21
IPI00289819	Cation-independent mannose-6-phosphate receptor precursor	K.LSGAYLVDDSDPDTSLFINVCR.D	3	2.95	0.09	-4.81
IPI00289819	Cation-independent mannose-6-phosphate receptor precursor	K.LSSDVCPTSDK.S	2	2.31	0.17	-2.60
IPI00289819	Cation-independent mannose-6-phosphate receptor precursor	K.LTYENGLLK.M	2	2.06	0.09	-2.26
IPI00289819	Cation-independent mannose-6-phosphate receptor precursor	K.NGAYKVETK.K	2	2.02	0.07	-0.25
IPI00289819	Cation-independent mannose-6-phosphate receptor precursor	K.RYDLSALVR.H	2	2.47	0.14	-2.55
IPI00289819	Cation-independent mannose-6-phosphate receptor precursor	K.VAGLLTQK.L	2	2.52	0.17	-3.44
IPI00289819	Cation-independent mannose-6-phosphate receptor precursor	K.VPIDGPPIDIGR.V	2	2.83	0.36	-2.20
IPI00289819	Cation-independent mannose-6-phosphate receptor precursor	R.ATLITFLCDR.D	2	2.62	0.17	-2.47
IPI00289819	Cation-independent mannose-6-phosphate receptor precursor	R.FVCNDDVYSGPLK.F	2	3.29	0.32	-0.79
IPI00289819	Cation-independent mannose-6-phosphate receptor precursor	R.TVEACPVVR.V	2	2.30	0.07	-0.24
IPI00289819	Cation-independent mannose-6-phosphate receptor precursor	R.TYHSVGDSVLR.S	2	2.19	0.14	0.09
IPI00289819	Cation-independent mannose-6-phosphate receptor precursor	R.YDLSALVR.H	2	2.78	0.15	-3.88
IPI00289819	Cation-independent mannose-6-phosphate receptor precursor	R.YSDNWEAITGTGDPEHYLINVCK.S	3	3.18	0.31	-2.85
IPI00289819	Cation-independent mannose-6-phosphate receptor precursor	R.YVDQVLQLVYK.D	2	3.64	0.34	-2.82
IPI00289831	Isoform PTPS of Receptor-type tyrosine-protein phosphatase S precursor	K.DFLPVDPSASNGR.I	2	2.53	0.31	-3.68
IPI00289831	Isoform PTPS of Receptor-type tyrosine-protein phosphatase S precursor	K.DQIGVSGGVASFVCQATGDPKPR.V	2	4.82	0.57	-3.16
IPI00289831	Isoform PTPS of Receptor-type tyrosine-protein phosphatase S precursor	K.DQIGVSGGVASFVCQATGDPKPR.V	3	3.04	0.19	-4.16

IPI00289831	Isoform PTPS of Receptor-type tyrosine-protein phosphatase S precursor	K.GAVLGRPTLSVQQTPEGSLLAR.W	3	4.28	0.35	-2.53
IPI00289831	Isoform PTPS of Receptor-type tyrosine-protein phosphatase S precursor	K.HNVDDSLLTTVGSLLEDETYTVR.V	3	3.30	0.37	-3.54
IPI00289831	Isoform PTPS of Receptor-type tyrosine-protein phosphatase S precursor	K.IQYNGLTLDVDGR.T	2	4.59	0.47	-1.48
IPI00289831	Isoform PTPS of Receptor-type tyrosine-protein phosphatase S precursor	K.LTVLREDQLPSGFPNIDM*GPQLK.V	2	4.50	0.56	-2.21
IPI00289831	Isoform PTPS of Receptor-type tyrosine-protein phosphatase S precursor	K.LTVLREDQLPSGFPNIDM*GPQLK.V	3	5.47	0.40	-2.59
IPI00289831	Isoform PTPS of Receptor-type tyrosine-protein phosphatase S precursor	K.QYGGFDNR.G	2	1.58	0.14	-2.16
IPI00289831	Isoform PTPS of Receptor-type tyrosine-protein phosphatase S precursor	K.SKSQDGPYQIKEDITTTR.Y	3	3.17	0.32	-1.19
IPI00289831	Isoform PTPS of Receptor-type tyrosine-protein phosphatase S precursor	K.SQDGPYQIK.E	2	2.24	0.06	-1.80
IPI00289831	Isoform PTPS of Receptor-type tyrosine-protein phosphatase S precursor	K.SQDGPYQIKEDITTTR.Y	2	4.53	0.44	-1.56
IPI00289831	Isoform PTPS of Receptor-type tyrosine-protein phosphatase S precursor	K.SQDGPYQIKEDITTTR.Y	3	2.56	0.25	-0.22
IPI00289831	Isoform PTPS of Receptor-type tyrosine-protein phosphatase S precursor	K.TQQGVPGQPM*NLR.A	2	3.45	0.37	-2.98
IPI00289831	Isoform PTPS of Receptor-type tyrosine-protein phosphatase S precursor	K.TSVLLSWEFPDNYNSPTPYK.I	2	3.70	0.47	-4.89
IPI00289831	Isoform PTPS of Receptor-type tyrosine-protein phosphatase S precursor	K.WM*QGAEDLTPEDDM*PVGR.N	2	6.12	0.56	-2.70
IPI00289831	Isoform PTPS of Receptor-type tyrosine-protein phosphatase S precursor	K.WM*QGAEDLTPEDDM*PVGR.N	3	2.35	0.13	-3.49
IPI00289831	Isoform PTPS of Receptor-type tyrosine-protein phosphatase S precursor	L.TPEDDM*PVGR.N	2	3.04	0.39	-2.61
IPI00289831	Isoform PTPS of Receptor-type tyrosine-protein phosphatase S precursor	R.EAGALGPAR.E	2	1.70	0.07	-2.63
IPI00289831	Isoform PTPS of Receptor-type tyrosine-protein phosphatase S precursor	R.ETELPAAAEPGAENALTLQGLKPDTAYDLQVR.A	3	2.82	0.18	-3.94
IPI00289831	Isoform PTPS of Receptor-type tyrosine-protein phosphatase S precursor	R.FETIEFDESAGAVLR.I	2	3.36	0.35	-6.40
IPI00289831	Isoform PTPS of Receptor-type tyrosine-protein phosphatase S precursor	R.FSVLPPTFHPGDQK.Q	2	2.73	0.21	-3.01
IPI00289831	Isoform PTPS of Receptor-type tyrosine-protein phosphatase S precursor	R.FSVLPPTFHPGDQK.Q	3	2.35	0.19	-2.00
IPI00289831	Isoform PTPS of Receptor-type tyrosine-protein phosphatase S precursor	R.GGQFLTPLGSPEDM*DLEELIQDISR.L	2	3.58	0.38	-1.11

IPI00289831	Isoform PTPS of Receptor-type tyrosine-protein phosphatase S precursor	R.GGQFLTPLGSPEDM*DLEELIQDISR.L	3	4.90	0.31	-3.52
IPI00289831	Isoform PTPS of Receptor-type tyrosine-protein phosphatase S precursor	R.GYQVHYVR.M	2	2.74	0.30	-3.75
IPI00289831	Isoform PTPS of Receptor-type tyrosine-protein phosphatase S precursor	R.ITTVAHTEVGPGPESSPVVVR.T	2	5.26	0.54	-1.62
IPI00289831	Isoform PTPS of Receptor-type tyrosine-protein phosphatase S precursor	R.ITTVAHTEVGPGPESSPVVVR.T	3	2.49	0.09	-2.75
IPI00289831	Isoform PTPS of Receptor-type tyrosine-protein phosphatase S precursor	R.ITTVAHTEVGPGPESSPVVVRTDEDVPSAPPR.K	4	3.70	0.38	-1.77
IPI00289831	Isoform PTPS of Receptor-type tyrosine-protein phosphatase S precursor	R.SETFESTPIR.G	2	3.01	0.18	-2.92
IPI00289831	Isoform PTPS of Receptor-type tyrosine-protein phosphatase S precursor	R.SPQGLGAFTPVVR.Q	2	4.15	0.47	-3.04
IPI00289831	Isoform PTPS of Receptor-type tyrosine-protein phosphatase S precursor	R.SRGGLGEEAAEVLSIPEDTPR.G	3	3.73	0.23	-2.10
IPI00289831	Isoform PTPS of Receptor-type tyrosine-protein phosphatase S precursor	R.TATM*LCAASGNPDPEITWFKDFLPVDPSASNGR.I	3	5.86	0.51	-2.98
IPI00289831	Isoform PTPS of Receptor-type tyrosine-protein phosphatase S precursor	R.TDEDVPSAPPR.K	2	3.12	0.36	-2.97
IPI00289831	Isoform PTPS of Receptor-type tyrosine-protein phosphatase S precursor	R.TFDPTTSYVVEDLKPNTEYAFR.L	2	5.76	0.46	-5.49
IPI00289831	Isoform PTPS of Receptor-type tyrosine-protein phosphatase S precursor	R.TFDPTTSYVVEDLKPNTEYAFR.L	3	3.05	0.28	-2.97
IPI00289831	Isoform PTPS of Receptor-type tyrosine-protein phosphatase S precursor	R.TGEQAPASAPR.N	2	3.60	0.38	-2.91
IPI00289831	Isoform PTPS of Receptor-type tyrosine-protein phosphatase S precursor	R.VLAFTSVGDGPLSDPIQVK.T	2	5.71	0.53	-2.41
IPI00289831	Isoform PTPS of Receptor-type tyrosine-protein phosphatase S precursor	R.VLAFTSVGDGPLSDPIQVK.T	3	6.38	0.50	-2.85
IPI00289831	Isoform PTPS of Receptor-type tyrosine-protein phosphatase S precursor	R.YRPLGSEDPEPK.E	3	2.47	0.05	-2.01
IPI00289831	Isoform PTPS of Receptor-type tyrosine-protein phosphatase S precursor	R.YSIGGLSPNSEYEIWVSAVNSIGQGPPSESVVTR.T	3	3.50	0.31	-5.20
IPI00289831	Isoform PTPS of Receptor-type tyrosine-protein phosphatase S precursor	R.YSSPANLYVR.E	1	1.82	0.13	-3.11
IPI00289831	Isoform PTPS of Receptor-type tyrosine-protein phosphatase S precursor	R.YSSPANLYVR.E	2	2.94	0.35	-2.36
IPI00289831	Isoform PTPS of Receptor-type tyrosine-protein phosphatase S precursor	W.DSGNPDPVSYYVIEYK.S	2	2.91	0.33	0.30
IPI00289831	Isoform PTPS of Receptor-type tyrosine-protein phosphatase S precursor	W.M*QGAEDLTPEDDM*PVGR.N	2	5.03	0.57	-4.09
IPI00289837	Coiled-coil domain-containing protein 85A	K.DLEVKQEEVVKENMELK.E	3	3.39	0.06	

IPI00289861	Isoform 1 of Zinc finger CCHC domain-containing protein 11	K.VNIEAVGGEKCALQNSPR.S	2	2.90	0.19	
IPI00289862	Secernin-1	R.AIIESDQEQGR.K	2	3.14	0.33	-4.03
IPI00289862	Secernin-1	R.SIFKPFIFVDDVK.L	3	2.94	0.17	-1.47
IPI00289870	Isoform C of Protocadherin-7 precursor	R.ECDEYGHSDSCWMPVRTSPERK.K	3	2.92	0.15	
IPI00289870	Isoform C of Protocadherin-7 precursor	R.IDREEVNQLR.F	3	3.45	0.05	-2.82
IPI00289870	Isoform C of Protocadherin-7 precursor	R.LDASEGGGGTNPGGR.S	2	4.50	0.46	-3.16
IPI00289870	Isoform C of Protocadherin-7 precursor	R.RLDASEGGGGTNPGGR.S	2	4.55	0.44	-2.54
IPI00289870	Isoform C of Protocadherin-7 precursor	R.RLDASEGGGGTNPGGR.S	3	3.98	0.11	
IPI00289870	Isoform C of Protocadherin-7 precursor	R.SSVFELQVADTPDGEKQPQLIVK.G	3	3.31	0.42	-0.24
IPI00289876	Isoform 1 of Syntaxin-7	K.ITQCSVEIQR.T	2	3.47	0.16	-0.80
IPI00289876	Isoform 1 of Syntaxin-7	K.QQYTNQLAK.E	2	1.98	0.11	0.19
IPI00289876	Isoform 1 of Syntaxin-7	R.TLNQLGTPQDSPELR.Q	2	4.06	0.37	-3.62
IPI00289924	Alpha-2,8-sialyltransferase 8E	K.CAVVGNGGILK.K	2	2.07	0.18	-2.40
IPI00289924	Alpha-2,8-sialyltransferase 8E	K.LKYEVDTSGIYHINQEIFR.M	3	2.97	0.23	-4.27
IPI00289924	Alpha-2,8-sialyltransferase 8E	K.TDVVTVNPSIITER.F	2	3.42	0.29	-2.74
IPI00289924	Alpha-2,8-sialyltransferase 8E	K.YTM*DVGVK.T	2	2.04	0.14	-2.25
IPI00289924	Alpha-2,8-sialyltransferase 8E	K.YVLDDFESPQAVYYFHPQYLVNVSR.Y	3	3.99	0.30	-2.57
IPI00289926	leukocyte immunoglobulin-like receptor, subfamily B, member 4 isoform 2	K.SVTLLCQSR.S	2	3.29	0.30	-0.46
IPI00289926	leukocyte immunoglobulin-like receptor, subfamily B, member 4 isoform 2	R.FSIPSM*TEDYAGR.Y	2	2.54	0.17	-3.04
IPI00289965	Potassium voltage-gated channel subfamily C member 3	R.RAEPCPGLPAAAMGRHGGGGGDSGKIVINVGGVR.H	3	3.12	0.07	3.02
IPI00290078	keratin 4	K.AQYEEIAQR.S	2	2.37	0.11	-2.21
IPI00290085	Cadherin-2 precursor	A.SGEIALCK.T	1	2.32	0.22	-1.99
IPI00290085	Cadherin-2 precursor	A.SGEIALCK.T	2	3.57	0.26	-1.93
IPI00290085	Cadherin-2 precursor	K.DVHEGQPLLNVK.F	1	3.31	0.48	-1.52
IPI00290085	Cadherin-2 precursor	K.DVHEGQPLLNVK.F	2	3.78	0.50	-2.72
IPI00290085	Cadherin-2 precursor	K.DVHEGQPLLNVK.F	3	2.57	0.15	-3.07
IPI00290085	Cadherin-2 precursor	K.ESAEVEEIVFPR.Q	2	3.13	0.26	-2.27
IPI00290085	Cadherin-2 precursor	K.ESAEVEEIVFPR.Q	3	3.47	0.21	-1.07
IPI00290085	Cadherin-2 precursor	K.FLEAGIYEVPIIITDSGNPPK.S	2	4.82	0.56	-3.14
IPI00290085	Cadherin-2 precursor	K.FLEAGIYEVPIIITDSGNPPK.S	3	5.13	0.53	-4.93
IPI00290085	Cadherin-2 precursor	K.FLIYAQDKETQEK.W	2	4.48	0.37	-2.90
IPI00290085	Cadherin-2 precursor	K.FLIYAQDKETQEK.W	3	2.52	0.08	-2.56
IPI00290085	Cadherin-2 precursor	K.IDPVNGQITTIAVLDRESPNVK.N	3	2.74	0.37	-1.69
IPI00290085	Cadherin-2 precursor	K.LSLKPTLTEESVK.E	2	2.88	0.30	-2.96
IPI00290085	Cadherin-2 precursor	K.LSLKPTLTEESVK.E	3	2.92	0.25	-1.34
IPI00290085	Cadherin-2 precursor	K.LSLKPTLTEESVKESAEVEE.I	2	3.13	0.43	-1.93
IPI00290085	Cadherin-2 precursor	K.LSLKPTLTEESVKESAEVEEIVFPR.Q	3	6.77	0.60	-4.53
IPI00290085	Cadherin-2 precursor	K.LSLKPTLTEESVKESAEVEEIVFPR.Q	4	3.06	0.24	-4.38

IPI00290085	Cadherin-2 precursor	K.LSLKPTLTEESVKESAEVEEIVFPR.Q	5	2.40	0.11	-0.52
IPI00290085	Cadherin-2 precursor	K.TGFPEDVYSAVLSK.D	2	5.19	0.56	-4.44
IPI00290085	Cadherin-2 precursor	K.TGFPEDVYSAVLSK.D	3	3.25	0.13	-0.66
IPI00290085	Cadherin-2 precursor	K.TGFPEDVYSAVLSKDVHEGQPLLNVK.F	3	4.87	0.54	-3.54
IPI00290085	Cadherin-2 precursor	K.VDEDGM*VYAVR.S	2	3.66	0.37	-3.43
IPI00290085	Cadherin-2 precursor	K.VQYESSEPADFKVDEDGM*VYAVR.S	2	4.22	0.57	-3.36
IPI00290085	Cadherin-2 precursor	K.VQYESSEPADFKVDEDGM*VYAVR.S	3	3.98	0.50	-3.90
IPI00290085	Cadherin-2 precursor	R.FAIQTDPNSNDGLVTVVKPIDFETNR.M	3	4.38	0.41	-3.12
IPI00290085	Cadherin-2 precursor	R.KVQYESSEPADFKVDEDGM*VYAVR.S	3	5.16	0.49	-4.50
IPI00290085	Cadherin-2 precursor	R.KVQYESSEPADFKVDEDGM*VYAVR.S	4	3.01	0.25	-3.51
IPI00290085	Cadherin-2 precursor	R.M*FVLTVAAENQVPLAK.G	2	5.02	0.54	-2.30
IPI00290085	Cadherin-2 precursor	R.SFPLSSEHAK.F	1	2.37	0.27	-4.27
IPI00290085	Cadherin-2 precursor	R.SFPLSSEHAK.F	2	2.61	0.27	-3.07
IPI00290085	Cadherin-2 precursor	R.YSVTGPGADQPPTGIFIINPISGQLSVTKPLDREQIAR.F	4	5.65	0.37	-4.98
IPI00290094	Splicing factor, arginine/serine-rich 8	K.EGRYTVLAENKSDEK.K	2	1.48	0.10	-8.66
IPI00290283	mannan-binding lectin serine protease 1 isoform 2 precursor	K.APEPISTQSHSVLILFHSDNSGENR.G	4	3.04	0.14	-1.53
IPI00290283	mannan-binding lectin serine protease 1 isoform 2 precursor	K.DNVEM*DTFQIECLK.D	2	3.06	0.22	
IPI00290283	mannan-binding lectin serine protease 1 isoform 2 precursor	K.DQVLVSCDTGYK.V	2	3.81	0.34	-3.52
IPI00290283	mannan-binding lectin serine protease 1 isoform 2 precursor	K.QVYGVYTK.V	1	1.62	0.09	-2.57
IPI00290283	mannan-binding lectin serine protease 1 isoform 2 precursor	K.SDFSNEER.F	2	2.18	0.17	-2.43
IPI00290283	mannan-binding lectin serine protease 1 isoform 2 precursor	K.YSCQEPYYK.M	2	2.54	0.29	
IPI00290283	mannan-binding lectin serine protease 1 isoform 2 precursor	R.AAGNECPELQPPVHGK.I	2	2.11	0.25	
IPI00290283	mannan-binding lectin serine protease 1 isoform 2 precursor	R.APGELEHGLITFSTR.N	3	3.37	0.40	-2.15
IPI00290283	mannan-binding lectin serine protease 1 isoform 2 precursor	R.DTTVIPVSK.E	1	2.34	0.18	-4.86
IPI00290283	mannan-binding lectin serine protease 1 isoform 2 precursor	R.ETTDTEQTPGQEVVLSPGSFM*SITFR.S	3	4.46	0.32	-5.59
IPI00290283	mannan-binding lectin serine protease 1 isoform 2 precursor	R.NAEPGLFPWQALIVVEDTSR.V	2	4.14	0.48	-4.49
IPI00290283	mannan-binding lectin serine protease 1 isoform 2 precursor	R.NAEPGLFPWQALIVVEDTSR.V	3	4.39	0.49	-4.19
IPI00290283	mannan-binding lectin serine protease 1 isoform 2 precursor	R.TGVITSPDFPNPYPK.S	2	3.60	0.27	

IPI00290283	mannan-binding lectin serine protease 1 isoform 2 precursor	R.TLSDVLQYVK.L	2	3.19	0.36	-3.29
IPI00290292	Rhomboid 5 homolog 1	K.DWEKAPEQADLTGGALDRSELERSHLM*LPLER.G	3	3.24	0.09	-6.22
IPI00290308	Tribbles homolog 1	K.DFGDM*HSYVRSR.K	2	2.29	0.08	-1.09
IPI00290315	Chromogranin-A precursor	A.LPVNSPM*NKGDTEVM*K.C	2	3.42	0.48	-3.26
IPI00290315	Chromogranin-A precursor	E.EDSLEAGLPLQVR.G	2	4.39	0.38	-4.33
IPI00290315	Chromogranin-A precursor	H.SGFEDELSEVLENQSSQAELK.E	2	6.15	0.52	-2.66
IPI00290315	Chromogranin-A precursor	H.SGFEDELSEVLENQSSQAELK.E	3	5.34	0.39	-3.60
IPI00290315	Chromogranin-A precursor	K.AEGNNQAPGEEEEEEEEATNTHPPASLPSQK.Y	3	2.95	0.33	-6.28
IPI00290315	Chromogranin-A precursor	K.AEGNNQAPGEEEEEEEEATNTHPPASLPSQK.Y	4	4.72	0.32	-4.44
IPI00290315	Chromogranin-A precursor	K.CIVEVISDTLSKPSPM*PVSQECFETLR.G	3	3.31	0.20	
IPI00290315	Chromogranin-A precursor	K.EAEKSGEATDGARPQALPEPM*QESK.A	2	3.59	0.48	-2.83
IPI00290315	Chromogranin-A precursor	K.EAEKSGEATDGARPQALPEPM*QESK.A	3	3.61	0.43	-3.04
IPI00290315	Chromogranin-A precursor	K.EAVEEPSSKDVM*.E	2	3.22	0.38	-3.22
IPI00290315	Chromogranin-A precursor	K.EAVEEPSSKDVM*E.K	1	2.63	0.40	-2.55
IPI00290315	Chromogranin-A precursor	K.EAVEEPSSKDVM*E.K	2	3.58	0.40	-4.59
IPI00290315	Chromogranin-A precursor	K.EEEEEM*AVVPQGLFR.G	2	3.42	0.31	-3.25
IPI00290315	Chromogranin-A precursor	K.EEEGSANRRPEDQELESLSAIEAELEK.V	3	3.75	0.31	-3.04
IPI00290315	Chromogranin-A precursor	K.EEEGSANRRPEDQELESLSAIEAELEK.V	4	3.22	0.13	-1.67
IPI00290315	Chromogranin-A precursor	K.EEEGSANRRPEDQELESLSAIEAELEKVAHQLQALR.R	5	4.19	0.17	-4.05
IPI00290315	Chromogranin-A precursor	K.ELQDLALQGAK.E	1	3.29	0.19	-3.12
IPI00290315	Chromogranin-A precursor	K.ELQDLALQGAK.E	2	3.54	0.29	-3.30
IPI00290315	Chromogranin-A precursor	K.GEQEHSQQKEEEEEM*AVVPQGLFR.G	2	4.69	0.59	-4.49
IPI00290315	Chromogranin-A precursor	K.GEQEHSQQKEEEEEM*AVVPQGLFR.G	3	5.01	0.51	-4.70
IPI00290315	Chromogranin-A precursor	K.GEQEHSQQKEEEEEM*AVVPQGLFR.G	4	3.13	0.29	-3.81
IPI00290315	Chromogranin-A precursor	K.GLSAEPGWQAK.R	1	2.67	0.25	-2.98
IPI00290315	Chromogranin-A precursor	K.HSGFEDELSEVLENQSSQAELK.E	2	6.60	0.50	-3.03
IPI00290315	Chromogranin-A precursor	K.HSGFEDELSEVLENQSSQAELK.E	3	5.95	0.38	-3.79
IPI00290315	Chromogranin-A precursor	K.HSGFEDELSEVLENQSSQAELKEAVEEPSSK.D	3	6.45	0.56	-5.25
IPI00290315	Chromogranin-A precursor	K.HSGFEDELSEVLENQSSQAELKEAVEEPSSK.D	4	5.25	0.37	-4.93
IPI00290315	Chromogranin-A precursor	K.HSGFEDELSEVLENQSSQAELKEAVEEPSSKDVM*E.K	3	6.01	0.58	-2.78
IPI00290315	Chromogranin-A precursor	K.KEEEGSANRRPEDQELESLSAIEAELEK.V	3	5.27	0.47	-3.84
IPI00290315	Chromogranin-A precursor	K.KEEEGSANRRPEDQELESLSAIEAELEK.V	4	4.53	0.37	-4.60
IPI00290315	Chromogranin-A precursor	K.KEEEGSANRRPEDQELESLSAIEAELEK.V	5	3.67	0.27	-2.51
IPI00290315	Chromogranin-A precursor	K.KEEEGSANRRPEDQELESLSAIEAELEKVAHQ.L	4	4.97	0.41	-5.09
IPI00290315	Chromogranin-A precursor	K.KEEEGSANRRPEDQELESLSAIEAELEKVAHQLQA.L	3	3.61	0.16	-4.82
IPI00290315	Chromogranin-A precursor	K.KEEEGSANRRPEDQELESLSAIEAELEKVAHQLQALR.R	4	3.02	0.13	-4.47
IPI00290315	Chromogranin-A precursor	K.KEEEGSANRRPEDQELESLSAIEAELEKVAHQLQALR.R	5	4.99	0.32	-2.47
IPI00290315	Chromogranin-A precursor	K.KEEEGSANRRPEDQELESLSAIEAELEKVAHQLQALR.R	6	3.57	0.25	-2.47
IPI00290315	Chromogranin-A precursor	K.RLEGQEEEEDNRDSSM*K.L	3	2.39	0.08	-4.05
IPI00290315	Chromogranin-A precursor	K.SGEATDGARPQALPEPM*QESK.A	2	3.51	0.46	-3.53
IPI00290315	Chromogranin-A precursor	K.SGEATDGARPQALPEPM*QESK.A	3	4.60	0.36	-4.64

IPI00290315	Chromogranin-A precursor	K.SGELEQEEER.L	2	3.67	0.24	-3.63
IPI00290315	Chromogranin-A precursor	K.SGELEQEEERLSK.E	2	3.90	0.24	-0.46
IPI00290315	Chromogranin-A precursor	K.SGELEQEEERLSKEWEDS.K	2	4.62	0.44	-4.09
IPI00290315	Chromogranin-A precursor	K.SGELEQEEERLSKEWEDS.K	3	4.71	0.40	-3.58
IPI00290315	Chromogranin-A precursor	K.VAHQLQALR.R	1	2.29	0.26	-5.46
IPI00290315	Chromogranin-A precursor	K.VAHQLQALR.R	2	3.01	0.34	-4.09
IPI00290315	Chromogranin-A precursor	K.YPGPQAEGDSEGLSQGLVDR.E	2	5.85	0.57	-8.17
IPI00290315	Chromogranin-A precursor	K.YPGPQAEGDSEGLSQGLVDR.E	3	4.74	0.37	-3.86
IPI00290315	Chromogranin-A precursor	K.YPGPQAEGDSEGLSQGLVDREK.G	2	5.00	0.51	-2.79
IPI00290315	Chromogranin-A precursor	K.YPGPQAEGDSEGLSQGLVDREK.G	3	6.83	0.54	-6.01
IPI00290315	Chromogranin-A precursor	L.AVDGAGKPGAEEAQDPEGK.G	2	2.93	0.27	-2.92
IPI00290315	Chromogranin-A precursor	L.AVDGAGKPGAEEAQDPEGK.G	3	3.85	0.44	-1.17
IPI00290315	Chromogranin-A precursor	P.GPQAEGDSEGLSQGLVDR.E	2	5.82	0.59	-3.95
IPI00290315	Chromogranin-A precursor	P.GPQAEGDSEGLSQGLVDREK.G	2	4.56	0.58	-2.02
IPI00290315	Chromogranin-A precursor	P.GPQAEGDSEGLSQGLVDREK.G	3	3.87	0.41	-1.39
IPI00290315	Chromogranin-A precursor	R.EDSKEAEKSGEATDGARPQALPEPM*QESK.A	2	4.41	0.45	-4.03
IPI00290315	Chromogranin-A precursor	R.EDSKEAEKSGEATDGARPQALPEPM*QESK.A	3	3.28	0.25	-3.86
IPI00290315	Chromogranin-A precursor	R.EDSKEAEKSGEATDGARPQALPEPM*QESK.A	4	3.64	0.24	-3.53
IPI00290315	Chromogranin-A precursor	R.GGKSGELEQEEER.L	2	4.22	0.35	-3.37
IPI00290315	Chromogranin-A precursor	R.GGKSGELEQEEER.L	3	3.33	0.13	-0.21
IPI00290315	Chromogranin-A precursor	R.GGKSGELEQEEERLSK.E	2	4.79	0.43	-1.88
IPI00290315	Chromogranin-A precursor	R.GGKSGELEQEEERLSK.E	3	3.85	0.21	-3.23
IPI00290315	Chromogranin-A precursor	R.GYPEEKKEEEGSANR.R	2	3.94	0.39	-3.29
IPI00290315	Chromogranin-A precursor	R.GYPEEKKEEEGSANRRPEDQELESLSAIEAELEK.V	3	5.32	0.51	-3.40
IPI00290315	Chromogranin-A precursor	R.GYPEEKKEEEGSANRRPEDQELESLSAIEAELEK.V	4	4.54	0.40	-3.80
IPI00290315	Chromogranin-A precursor	R.GYPEEKKEEEGSANRRPEDQELESLSAIEAELEK.V	5	2.43	0.13	-3.99
IPI00290315	Chromogranin-A precursor	R.GYPEEKKEEEGSANRRPEDQELESLSAIEAELEK.V	6	3.44	0.15	-3.76
IPI00290315	Chromogranin-A precursor	R.LEGQEEEEDNRDSSM*K.L	2	2.84	0.34	-3.05
IPI00290315	Chromogranin-A precursor	R.LEGQEEEEDNRDSSM*K.L	3	2.54	0.33	-2.06
IPI00290315	Chromogranin-A precursor	R.LSKEWEDS.K	1	2.29	0.26	-3.09
IPI00290315	Chromogranin-A precursor	R.RPEDQELESLSAIEAELEK.V	2	6.89	0.48	-4.97
IPI00290315	Chromogranin-A precursor	R.RPEDQELESLSAIEAELEK.V	3	4.87	0.40	-3.99
IPI00290315	Chromogranin-A precursor	R.RPEDQELESLSAIEAELEKVAHQL.Q	3	3.68	0.38	-5.48
IPI00290315	Chromogranin-A precursor	R.RPEDQELESLSAIEAELEKVAHQLQA.L	3	5.98	0.51	-3.32
IPI00290315	Chromogranin-A precursor	R.RPEDQELESLSAIEAELEKVAHQLQALR.R	3	8.25	0.64	-6.06
IPI00290315	Chromogranin-A precursor	R.RPEDQELESLSAIEAELEKVAHQLQALR.R	4	7.46	0.54	-4.93
IPI00290315	Chromogranin-A precursor	R.RPEDQELESLSAIEAELEKVAHQLQALRR.G	5	2.29	0.17	-1.57
IPI00290315	Chromogranin-A precursor	R.SEALAVDGAGKPGAEEAQD.P	2	5.09	0.43	-4.25
IPI00290315	Chromogranin-A precursor	R.SEALAVDGAGKPGAEEAQDPEGK.G	2	5.00	0.41	-4.36
IPI00290315	Chromogranin-A precursor	R.SEALAVDGAGKPGAEEAQDPEGK.G	3	4.88	0.39	-2.83
IPI00290315	Chromogranin-A precursor	R.SEALAVDGAGKPGAEEAQDPEGKGEQEHSQQK.E	4	4.64	0.35	-3.36
IPI00290315	Chromogranin-A precursor	S.GFEDELSEVLENQSSQAELK.E	3	4.56	0.24	-2.93

IPI00290315	Chromogranin-A precursor	V.AHQLQALR.R	1	2.01	0.17	-4.63
IPI00290328	Receptor-type tyrosine-protein phosphatase eta precursor	K.AVSISPTNVILTWK.S	2	3.13	0.25	-4.54
IPI00290328	Receptor-type tyrosine-protein phosphatase eta precursor	K.GDPLGTEGGLDASNTER.S	2	3.76	0.54	-3.31
IPI00290328	Receptor-type tyrosine-protein phosphatase eta precursor	K.TKGDPLGTEGGLDASNTER.S	2	3.83	0.52	-2.59
IPI00290328	Receptor-type tyrosine-protein phosphatase eta precursor	K.TPSSTGPSPVFDIK.A	2	3.07	0.27	-2.95
IPI00290328	Receptor-type tyrosine-protein phosphatase eta precursor	K.YCFEIVPK.G	2	2.64	0.27	-1.06
IPI00290328	Receptor-type tyrosine-protein phosphatase eta precursor	R.AGSPTAPVHDESLVGPVDPSSGQQSR.D	3	4.23	0.43	-2.59
IPI00290328	Receptor-type tyrosine-protein phosphatase eta precursor	R.VLLESIGSHEELTQDSR.L	2	4.40	0.56	-1.66
IPI00290328	Receptor-type tyrosine-protein phosphatase eta precursor	R.VLLESIGSHEELTQDSR.L	3	4.47	0.43	-1.51
IPI00290358	Putative uncharacterized protein gs103	R.QVWGEVPEPSDRSEEPETPAAYR.A	3	2.95	0.29	-2.66
IPI00290744	Fibronectin type-III domain-containing protein C5orf40	R.DGAELDPEANQDAPDAGALQR.G	3	3.84	0.30	0.93
IPI00290826	Transmembrane protein 157 precursor	R.GLAEAAGPR.G	1	2.54	0.16	-3.71
IPI00290826	Transmembrane protein 157 precursor	R.GLAEAAGPR.G	2	2.73	0.20	-3.30
IPI00290854	A-kinase anchor protein 3	K.SCDASLAELGDDKSGDASR.L	3	3.80	0.18	
IPI00290856	Lymphatic vessel endothelial hyaluronic acid receptor 1 precursor	G.SLRAEELSIQVSCR.I	2	3.08	0.30	-3.64
IPI00290856	Lymphatic vessel endothelial hyaluronic acid receptor 1 precursor	K.ANQQLNFTEAK.E	2	3.27	0.30	-1.66
IPI00290856	Lymphatic vessel endothelial hyaluronic acid receptor 1 precursor	K.DQVETALK.A	1	1.97	0.11	-2.80
IPI00290856	Lymphatic vessel endothelial hyaluronic acid receptor 1 precursor	K.DQVETALK.A	2	2.48	0.11	-2.79
IPI00290856	Lymphatic vessel endothelial hyaluronic acid receptor 1 precursor	K.KANQQLNFTEAK.E	2	4.30	0.33	-3.36
IPI00290856	Lymphatic vessel endothelial hyaluronic acid receptor 1 precursor	K.NGVGVLIWK.V	2	3.19	0.27	-1.00
IPI00290856	Lymphatic vessel endothelial hyaluronic acid receptor 1 precursor	R.IM*GITLVSK.K	2	2.66	0.18	-2.68
IPI00290856	Lymphatic vessel endothelial hyaluronic acid receptor 1 precursor	R.IMGITLVSK.K	2	2.64	0.21	-1.76
IPI00290856	Lymphatic vessel endothelial hyaluronic acid receptor 1 precursor	R.LLGLSLAGK.D	1	1.75	0.20	-3.92

IPI00290856	Lymphatic vessel endothelial hyaluronic acid receptor 1 precursor	R.LLGLSLAGK.D	2	2.93	0.20	-3.35
IPI00290856	Lymphatic vessel endothelial hyaluronic acid receptor 1 precursor	R.LLGLSLAGKDQVETALK.A	2	4.97	0.50	-3.70
IPI00290856	Lymphatic vessel endothelial hyaluronic acid receptor 1 precursor	R.LLGLSLAGKDQVETALK.A	3	4.23	0.44	-2.32
IPI00290857	Keratin, type II cytoskeletal 3	K.KYEDEINKR.T	2	3.00	0.17	-1.46
IPI00291005	Malate dehydrogenase, cytoplasmic	K.DLDVAILVGSM*PR.R	2	2.83	0.37	-2.88
IPI00291005	Malate dehydrogenase, cytoplasmic	K.ELTEEKESAFEF.L	2	3.65	0.44	-5.48
IPI00291005	Malate dehydrogenase, cytoplasmic	K.ELTEEKESAFEFL.S	2	3.10	0.36	-4.76
IPI00291005	Malate dehydrogenase, cytoplasmic	K.ELTEEKESAFEFLSSA.-	2	3.21	0.37	-5.84
IPI00291005	Malate dehydrogenase, cytoplasmic	K.ENFSCLTR.L	2	2.12	0.12	-1.56
IPI00291005	Malate dehydrogenase, cytoplasmic	K.EVGVYEALKDDSWLK.G	2	4.52	0.42	-2.74
IPI00291005	Malate dehydrogenase, cytoplasmic	K.FVEGLPINDFSR.E	1	2.29	0.32	-2.57
IPI00291005	Malate dehydrogenase, cytoplasmic	K.FVEGLPINDFSR.E	2	3.43	0.33	-3.83
IPI00291005	Malate dehydrogenase, cytoplasmic	K.GEFVTTVQQR.G	1	2.40	0.35	-4.35
IPI00291005	Malate dehydrogenase, cytoplasmic	K.GEFVTTVQQR.G	2	3.68	0.29	-2.93
IPI00291005	Malate dehydrogenase, cytoplasmic	K.LGVTANDVK.N	1	2.16	0.27	-3.17
IPI00291005	Malate dehydrogenase, cytoplasmic	K.LGVTANDVK.N	2	3.14	0.35	-3.06
IPI00291005	Malate dehydrogenase, cytoplasmic	K.NVIIWGNHSSTQYPDVNHAK.V	3	3.45	0.28	-6.16
IPI00291005	Malate dehydrogenase, cytoplasmic	K.SQGAALDKYAK.K	1	3.25	0.30	-2.79
IPI00291005	Malate dehydrogenase, cytoplasmic	K.SQGAALDKYAK.K	2	3.53	0.40	-1.57
IPI00291005	Malate dehydrogenase, cytoplasmic	K.VIVVGNPANTNCLTASK.S	2	4.40	0.48	-3.18
IPI00291005	Malate dehydrogenase, cytoplasmic	R.EKM*DLTAK.E	2	1.11	0.23	-3.46
IPI00291005	Malate dehydrogenase, cytoplasmic	W.GNHSSTQYPDVNHAK.V	2	4.28	0.53	-4.12
IPI00291006	Malate dehydrogenase, mitochondrial precursor	K.AGAGSATLSM*AYAGAR.F	2	4.71	0.50	-0.98
IPI00291006	Malate dehydrogenase, mitochondrial precursor	K.GYLGPEQLPDCLKGCDVVVIPAGVPR.K	3	4.11	0.30	-0.08
IPI00291006	Malate dehydrogenase, mitochondrial precursor	R.ANTFVAELK.G	2	2.16	0.07	-0.33
IPI00291006	Malate dehydrogenase, mitochondrial precursor	R.FVFSLVDAM*NGK.E	2	3.88	0.43	-3.92
IPI00291006	Malate dehydrogenase, mitochondrial precursor	R.LTLYDIAHTPGVAADLSHIETK.A	3	5.23	0.43	-2.54
IPI00291136	Collagen alpha-1(VI) chain precursor	G.DEGNPGPDGAPGER.G	2	3.03	0.35	-3.29
IPI00291136	Collagen alpha-1(VI) chain precursor	K.CPDYTCPITFSSPADITILLDGSASVGSHNFDTTKR.F	4	5.07	0.46	-2.46
IPI00291136	Collagen alpha-1(VI) chain precursor	K.DVFDFIPGSDQLNVISCQGLAPSQGRPGLSLVK.E	3	4.02	0.36	-2.83
IPI00291136	Collagen alpha-1(VI) chain precursor	K.FEPGQSYAGVVQYSHSQM*QEHVSLR.S	3	1.49	0.14	-4.75
IPI00291136	Collagen alpha-1(VI) chain precursor	K.GDEGEAGDPGDDNNDIAPR.G	2	4.11	0.53	-3.09
IPI00291136	Collagen alpha-1(VI) chain precursor	K.GLEQLLVGGSHLK.E	2	3.71	0.23	-2.33
IPI00291136	Collagen alpha-1(VI) chain precursor	K.GLEQLLVGGSHLKENK.Y	2	4.04	0.36	-4.04
IPI00291136	Collagen alpha-1(VI) chain precursor	K.GLEQLLVGGSHLKENK.Y	3	4.08	0.39	-1.85
IPI00291136	Collagen alpha-1(VI) chain precursor	K.GTYTDCAIK.K	1	1.43	0.17	-3.71
IPI00291136	Collagen alpha-1(VI) chain precursor	K.GTYTDCAIK.K	2	2.13	0.12	-0.18
IPI00291136	Collagen alpha-1(VI) chain precursor	K.GYRGDEGPPGSEGAR.G	2	3.97	0.36	-4.11
IPI00291136	Collagen alpha-1(VI) chain precursor	K.KGLEQLLVGGSHLK.E	2	3.64	0.46	-2.90

IPI00291136	Collagen alpha-1(VI) chain precursor	K.KGLEQLLVGGSHLK.E	3	3.11	0.13	-2.25
IPI00291136	Collagen alpha-1(VI) chain precursor	K.RLLLFSDGNSQGATPAAIEK.A	3	4.07	0.35	-2.57
IPI00291136	Collagen alpha-1(VI) chain precursor	K.SLQWM*AGGTFTGEALQYTR.D	2	6.60	0.60	-5.67
IPI00291136	Collagen alpha-1(VI) chain precursor	K.SLQWM*AGGTFTGEALQYTR.D	3	4.82	0.47	-3.76
IPI00291136	Collagen alpha-1(VI) chain precursor	K.TAEYDVAYGESHLFR.V	2	4.99	0.54	-2.98
IPI00291136	Collagen alpha-1(VI) chain precursor	K.TAEYDVAYGESHLFR.V	3	3.31	0.40	-3.07
IPI00291136	Collagen alpha-1(VI) chain precursor	K.TAEYDVAYGESHLFRVPSYQALLR.G	3	3.89	0.38	-3.80
IPI00291136	Collagen alpha-1(VI) chain precursor	K.TAEYDVAYGESHLFRVPSYQALLR.G	4	2.60	0.29	-3.34
IPI00291136	Collagen alpha-1(VI) chain precursor	K.VFSVAITPDHLEPR.L	2	3.52	0.42	-3.15
IPI00291136	Collagen alpha-1(VI) chain precursor	K.VFSVAITPDHLEPR.L	3	3.09	0.34	-1.70
IPI00291136	Collagen alpha-1(VI) chain precursor	K.YLIVVTDGHPLEGYKEPCGGLEDAVNEAK.H	3	5.38	0.43	-2.69
IPI00291136	Collagen alpha-1(VI) chain precursor	K.YLIVVTDGHPLEGYKEPCGGLEDAVNEAK.H	4	3.57	0.32	-2.84
IPI00291136	Collagen alpha-1(VI) chain precursor	R.AVAFQDCPVDLFFVLDTSESVALR.L	3	4.36	0.31	-4.30
IPI00291136	Collagen alpha-1(VI) chain precursor	R.DAEEAISQTIDTIVDM*IK.N	2	5.37	0.54	-7.38
IPI00291136	Collagen alpha-1(VI) chain precursor	R.DAEEAISQTIDTIVDM*IK.N	3	5.73	0.44	-3.02
IPI00291136	Collagen alpha-1(VI) chain precursor	R.DAEEAISQTIDTIVDMIK.N	2	4.09	0.36	
IPI00291136	Collagen alpha-1(VI) chain precursor	R.DALKSSVDAVK.Y	2	2.96	0.16	1.22
IPI00291136	Collagen alpha-1(VI) chain precursor	R.DELVKFEPGQSYAGVVQYSHSQM*QEHVSLR.S	4	5.09	0.47	-4.23
IPI00291136	Collagen alpha-1(VI) chain precursor	R.DQLLPPSPNNR.I	1	1.75	0.07	-2.33
IPI00291136	Collagen alpha-1(VI) chain precursor	R.DQLLPPSPNNR.I	2	2.61	0.12	-2.74
IPI00291136	Collagen alpha-1(VI) chain precursor	R.DTTPLNVLCSPGIQVVSVGIK.D	2	4.82	0.48	-3.47
IPI00291136	Collagen alpha-1(VI) chain precursor	R.DTTPLNVLCSPGIQVVSVGIK.D	3	3.26	0.33	-3.94
IPI00291136	Collagen alpha-1(VI) chain precursor	R.EGPVGVPGDPGEAGPIGPK.G	2	3.69	0.45	-4.97
IPI00291136	Collagen alpha-1(VI) chain precursor	R.FLTAGR.T	1	1.75	0.15	-3.48
IPI00291136	Collagen alpha-1(VI) chain precursor	R.FLTAGRTDPAHDVR.V	2	2.57	0.11	-1.25
IPI00291136	Collagen alpha-1(VI) chain precursor	R.FYREASSGAAK.K	2	3.82	0.33	-2.44
IPI00291136	Collagen alpha-1(VI) chain precursor	R.GDEGPPGSEGAR.G	2	2.87	0.27	-1.87
IPI00291136	Collagen alpha-1(VI) chain precursor	R.GDPGEAGPQGDQGR.E	2	3.76	0.42	-3.44
IPI00291136	Collagen alpha-1(VI) chain precursor	R.GDPGFEGER.G	2	2.32	0.13	-3.52
IPI00291136	Collagen alpha-1(VI) chain precursor	R.GIDGVDGVK.G	2	2.50	0.24	-2.89
IPI00291136	Collagen alpha-1(VI) chain precursor	R.GPPGLRGDPGFEGER.G	3	2.50	0.15	-1.90
IPI00291136	Collagen alpha-1(VI) chain precursor	R.GVFHQTVSR.K	1	2.47	0.17	-3.17
IPI00291136	Collagen alpha-1(VI) chain precursor	R.GVFHQTVSR.K	2	2.86	0.20	-2.68
IPI00291136	Collagen alpha-1(VI) chain precursor	R.IALVITDGR.S	2	3.32	0.24	-4.15
IPI00291136	Collagen alpha-1(VI) chain precursor	R.LKPYGALVDK.V	2	1.86	0.08	-2.62
IPI00291136	Collagen alpha-1(VI) chain precursor	R.LLLFSDGNSQGATPAAIEK.A	2	6.30	0.54	-4.01
IPI00291136	Collagen alpha-1(VI) chain precursor	R.LLLFSDGNSQGATPAAIEKAVQEAQR.A	3	4.63	0.47	-3.04
IPI00291136	Collagen alpha-1(VI) chain precursor	R.LSIIATDHTYR.R	1	2.84	0.36	-2.75
IPI00291136	Collagen alpha-1(VI) chain precursor	R.LSIIATDHTYR.R	2	3.21	0.32	-3.13
IPI00291136	Collagen alpha-1(VI) chain precursor	R.LSRDELVKFEPGQSYAGVVQY.S	3	3.53	0.38	-3.29
IPI00291136	Collagen alpha-1(VI) chain precursor	R.LSRDELVKFEPGQSYAGVVQYSHSQM*QEHVSLR.S	5	4.33	0.27	-2.31
IPI00291136	Collagen alpha-1(VI) chain precursor	R.M*PGGRDALK.S	2	2.36	0.08	-0.59

IPI00291136	Collagen alpha-1(VI) chain precursor	R.NLVWNAGALHYSDEVEIIQGLTR.M	3	4.01	0.22	-1.88
IPI00291136	Collagen alpha-1(VI) chain precursor	R.NVQELKEAIK.S	2	2.59	0.17	-1.68
IPI00291136	Collagen alpha-1(VI) chain precursor	R.SDTQRDTTPLNVLCSPGIQVVSVGIK.D	3	5.85	0.46	-1.11
IPI00291136	Collagen alpha-1(VI) chain precursor	R.TDPAHDVR.V	2	2.00	0.22	-1.86
IPI00291136	Collagen alpha-1(VI) chain precursor	R.TDPAHDVRVAVVQYSGTGQQRPER.A	4	2.55	0.27	-4.19
IPI00291136	Collagen alpha-1(VI) chain precursor	R.VAVVQYSGTGQQRPER.A	2	3.06	0.23	-3.56
IPI00291136	Collagen alpha-1(VI) chain precursor	R.VAVVQYSGTGQQRPER.A	3	2.55	0.29	-2.38
IPI00291136	Collagen alpha-1(VI) chain precursor	R.VPSYQALLR.G	2	3.03	0.30	-2.28
IPI00291175	Isoform 1 of Vinculin	K.AGEVINQPM*M*M*AAR.Q	2	3.52	0.31	-3.30
IPI00291175	Isoform 1 of Vinculin	K.AIPDLTAPVAAVQAAVSNLVR.V	2	4.04	0.44	-3.91
IPI00291175	Isoform 1 of Vinculin	K.M*SAEINEIIR.V	2	3.11	0.24	-1.96
IPI00291175	Isoform 1 of Vinculin	K.MSAEINEIIR.V	2	3.29	0.20	-2.21
IPI00291175	Isoform 1 of Vinculin	K.RDM*PPAFIKVENACTKLVQAAQMLQSDPYSVPAR.D	4	3.01	0.10	-5.13
IPI00291175	Isoform 1 of Vinculin	R.DPSASPGDAGEQAIR.Q	2	2.58	0.21	-1.26
IPI00291175	Isoform 1 of Vinculin	R.DYLIDGSR.G	2	2.38	0.20	-2.65
IPI00291175	Isoform 1 of Vinculin	R.GILSGTSDLLLTFDEAEVR.K	2	4.54	0.41	-6.51
IPI00291175	Isoform 1 of Vinculin	R.GQGSSPVAM*QK.A	2	2.46	0.22	-2.70
IPI00291262	Clusterin precursor	A.SHTSDSDVPSGVTEVVVK.L	2	5.22	0.58	-3.45
IPI00291262	Clusterin precursor	D.NELQEM*SNQGSK.Y	2	3.97	0.34	-3.14
IPI00291262	Clusterin precursor	D.PITVTVPVEVSR.K	2	3.80	0.44	-2.99
IPI00291262	Clusterin precursor	D.SDPITVTVPVEVSR.K	2	3.36	0.34	-5.82
IPI00291262	Clusterin precursor	F.DSDPITVTVPVEVSR.K	2	4.26	0.47	-4.28
IPI00291262	Clusterin precursor	F.FTREPQDTYHYLPFSLPHR.R	3	4.60	0.33	-3.81
IPI00291262	Clusterin precursor	G.DQTVSDNELQEM*SNQGSK.Y	2	5.96	0.60	-4.66
IPI00291262	Clusterin precursor	G.DQTVSDNELQEM*SNQGSK.Y	3	5.52	0.50	-2.45
IPI00291262	Clusterin precursor	G.DQTVSDNELQEMSNQGSK.Y	2	5.47	0.46	
IPI00291262	Clusterin precursor	G.DQTVSDNELQEMSNQGSK.Y	3	4.38	0.37	-0.03
IPI00291262	Clusterin precursor	H.TSDSDVPSGVTEVVVK.L	2	3.65	0.31	-3.64
IPI00291262	Clusterin precursor	I.DELFQDRFFTR.E	2	2.93	0.26	-2.14
IPI00291262	Clusterin precursor	K.ALQEYR.K	1	1.17	0.13	-1.93
IPI00291262	Clusterin precursor	K.ALQEYRK.K	2	2.16	0.11	-1.25
IPI00291262	Clusterin precursor	K.CREILSVDCSTNNPS.Q	2	4.02	0.34	-4.20
IPI00291262	Clusterin precursor	K.CREILSVDCSTNNPSQAK.L	2	5.84	0.50	-4.15
IPI00291262	Clusterin precursor	K.CREILSVDCSTNNPSQAK.L	3	3.85	0.29	-2.68
IPI00291262	Clusterin precursor	K.EIQNAVNGVK.Q	1	2.61	0.35	-3.54
IPI00291262	Clusterin precursor	K.EIQNAVNGVK.Q	2	2.90	0.25	-3.47
IPI00291262	Clusterin precursor	K.FM*ETVAEK.A	1	2.56	0.23	-3.55
IPI00291262	Clusterin precursor	K.FM*ETVAEK.A	2	2.97	0.22	-3.38
IPI00291262	Clusterin precursor	K.FM*ETVAEKALQEYR.K	2	3.94	0.38	
IPI00291262	Clusterin precursor	K.FM*ETVAEKALQEYR.K	3	3.73	0.42	
IPI00291262	Clusterin precursor	K.FMETVAEK.A	1	2.45	0.15	
IPI00291262	Clusterin precursor	K.FMETVAEK.A	2	2.27	0.19	-1.13

IPI00291262	Clusterin precursor	K.KKKEDALNETR.E	3	3.03	0.26	
IPI00291262	Clusterin precursor	K.LFDSDPITVTVPVEV.S	1	1.95	0.31	-3.48
IPI00291262	Clusterin precursor	K.LFDSDPITVTVPVEV.S	2	3.50	0.28	-3.24
IPI00291262	Clusterin precursor	K.LFDSDPITVTVPVEVSR.K	1	2.60	0.30	
IPI00291262	Clusterin precursor	K.LFDSDPITVTVPVEVSR.K	2	5.98	0.57	-7.61
IPI00291262	Clusterin precursor	K.LFDSDPITVTVPVEVSR.K	3	4.80	0.34	-4.19
IPI00291262	Clusterin precursor	K.LFDSDPITVTVPVEVSRK.N	2	3.77	0.53	-2.96
IPI00291262	Clusterin precursor	K.LFDSDPITVTVPVEVSRK.N	3	3.10	0.38	-4.82
IPI00291262	Clusterin precursor	K.TLIEKTNEER.K	1	2.68	0.15	
IPI00291262	Clusterin precursor	K.TLIEKTNEER.K	2	2.82	0.12	-4.34
IPI00291262	Clusterin precursor	K.TLIEKTNEERK.T	2	3.24	0.22	-4.06
IPI00291262	Clusterin precursor	K.TLLSNLEEAK.K	1	2.26	0.07	-2.19
IPI00291262	Clusterin precursor	K.TLLSNLEEAK.K	2	3.19	0.19	-2.76
IPI00291262	Clusterin precursor	K.TLLSNLEEAKK.K	2	3.40	0.29	-3.83
IPI00291262	Clusterin precursor	K.TLLSNLEEAKKK.K	2	2.94	0.21	-4.41
IPI00291262	Clusterin precursor	K.YVNKEIQNA.V	1	2.07	0.25	-1.37
IPI00291262	Clusterin precursor	K.YVNKEIQNAVNGVK.Q	2	5.33	0.47	-3.97
IPI00291262	Clusterin precursor	K.YVNKEIQNAVNGVK.Q	3	3.70	0.32	-2.12
IPI00291262	Clusterin precursor	K.YVNKEIQNAVNGVKQ.I	2	4.92	0.45	-3.48
IPI00291262	Clusterin precursor	L.FDSDPITVTVPVEVSR.K	2	4.83	0.51	-3.84
IPI00291262	Clusterin precursor	L.FDSDPITVTVPVEVSRK.N	2	3.21	0.43	-3.24
IPI00291262	Clusterin precursor	L.GDQTVSDNELQEM*SNQGSK.Y	2	5.81	0.41	
IPI00291262	Clusterin precursor	L.NEQFNWVSR.L	1	2.58	0.21	-3.01
IPI00291262	Clusterin precursor	L.NEQFNWVSR.L	2	2.97	0.22	-2.94
IPI00291262	Clusterin precursor	Q.LNEQFNWVSR.L	2	3.65	0.23	-2.18
IPI00291262	Clusterin precursor	Q.TVSDNELQEM*SNQGSK.Y	2	4.56	0.51	-4.13
IPI00291262	Clusterin precursor	R.ASSIIDELFQDR.F	1	3.22	0.34	-2.84
IPI00291262	Clusterin precursor	R.ASSIIDELFQDR.F	2	4.22	0.31	-4.42
IPI00291262	Clusterin precursor	R.ASSIIDELFQDR.F	3	3.93	0.20	-1.61
IPI00291262	Clusterin precursor	R.ASSIIDELFQDRF.F	2	4.12	0.39	-4.72
IPI00291262	Clusterin precursor	R.ASSIIDELFQDRFFT.R	2	3.99	0.40	-3.24
IPI00291262	Clusterin precursor	R.ASSIIDELFQDRFFTR.E	2	3.05	0.28	-5.43
IPI00291262	Clusterin precursor	R.ASSIIDELFQDRFFTR.E	3	3.00	0.31	-5.59
IPI00291262	Clusterin precursor	R.ASSIIDELFQDRFFTREPQDT.Y	3	4.37	0.46	-3.20
IPI00291262	Clusterin precursor	R.EILSVDCSTNNPSQAK.L	1	2.36	0.48	-3.04
IPI00291262	Clusterin precursor	R.EILSVDCSTNNPSQAK.L	2	4.55	0.54	-4.10
IPI00291262	Clusterin precursor	R.EILSVDCSTNNPSQAK.L	3	3.22	0.18	
IPI00291262	Clusterin precursor	R.ELDESLQVAER.L	1	2.52	0.38	-3.08
IPI00291262	Clusterin precursor	R.ELDESLQVAER.L	2	3.88	0.43	-4.25
IPI00291262	Clusterin precursor	R.EPQDTYHYLPFSLPHR.R	2	3.20	0.40	-4.31
IPI00291262	Clusterin precursor	R.EPQDTYHYLPFSLPHR.R	3	3.45	0.27	-4.07
IPI00291262	Clusterin precursor	R.FFTREPQDT.Y	1	1.90	0.29	-3.02

IPI00291262	Clusterin precursor	R.FFTREPQDTYHYLPFSLPHR.R	2	4.29	0.44	-3.12
IPI00291262	Clusterin precursor	R.FFTREPQDTYHYLPFSLPHR.R	3	5.19	0.44	-4.07
IPI00291262	Clusterin precursor	R.FFTREPQDTYHYLPFSLPHR.R	4	3.77	0.32	-3.29
IPI00291262	Clusterin precursor	R.FFTREPQDTYHYLPFSLPHR.R	5	2.36	0.12	-2.79
IPI00291262	Clusterin precursor	R.IDSLLENDRQQTHM*LDVM*QDHFSR.A	3	4.36	0.37	-3.80
IPI00291262	Clusterin precursor	R.IDSLLENDRQQTHM*LDVM*QDHFSR.A	4	4.44	0.43	-2.82
IPI00291262	Clusterin precursor	R.KTLLSNLEEAK.K	1	2.37	0.17	-2.61
IPI00291262	Clusterin precursor	R.KTLLSNLEEAK.K	2	3.64	0.31	-1.90
IPI00291262	Clusterin precursor	R.KTLLSNLEEAK.K	3	4.25	0.26	-3.95
IPI00291262	Clusterin precursor	R.KTLLSNLEEAKK.K	2	4.09	0.29	-4.59
IPI00291262	Clusterin precursor	R.KTLLSNLEEAKK.K	3	4.32	0.35	-3.92
IPI00291262	Clusterin precursor	R.KTLLSNLEEAKKK.K	3	3.78	0.19	
IPI00291262	Clusterin precursor	R.M*KDQCDKCREILSVDCSTNNPSQAK.L	3	4.62	0.34	
IPI00291262	Clusterin precursor	R.QQTHM*LDVM*QDHFSR.A	2	2.14	0.42	-1.98
IPI00291262	Clusterin precursor	R.QQTHM*LDVM*QDHFSR.A	3	3.19	0.23	
IPI00291262	Clusterin precursor	R.QQTHMLDVM*QDHFSR.A	2	3.06	0.29	
IPI00291262	Clusterin precursor	R.QQTHMLDVM*QDHFSR.A	3	3.41	0.10	
IPI00291262	Clusterin precursor	R.QQTHMLDVMQDHFSR.A	3	3.79	0.12	
IPI00291262	Clusterin precursor	R.RELDESLQVAER.L	1	2.97	0.26	
IPI00291262	Clusterin precursor	R.RELDESLQVAER.L	2	4.16	0.33	-2.41
IPI00291262	Clusterin precursor	R.RELDESLQVAER.L	3	3.91	0.12	-0.62
IPI00291262	Clusterin precursor	R.SGSGLVGR.Q	1	1.34	0.08	-2.26
IPI00291262	Clusterin precursor	R.VTTVASHTSDSD.V	2	3.11	0.44	-2.27
IPI00291262	Clusterin precursor	R.VTTVASHTSDSDVPSGVTE.V	2	3.18	0.40	-3.13
IPI00291262	Clusterin precursor	R.VTTVASHTSDSDVPSGVTEVVVK.L	2	6.44	0.58	-4.03
IPI00291262	Clusterin precursor	R.VTTVASHTSDSDVPSGVTEVVVK.L	3	4.01	0.25	-4.66
IPI00291262	Clusterin precursor	R.VTTVASHTSDSDVPSGVTEVVVKLFDSDPITVTVPVEVSR.K	3	7.10	0.54	
IPI00291262	Clusterin precursor	S.DNELQEM*SNQGSK.Y	2	3.96	0.42	-4.42
IPI00291262	Clusterin precursor	S.DSDVPSGVTEVVVK.L	2	3.07	0.40	-3.12
IPI00291262	Clusterin precursor	T.SDSDVPSGVTEVVVK.L	2	4.18	0.41	-3.87
IPI00291262	Clusterin precursor	T.SSLLEQLNEQFNWVSR.L	2	4.95	0.50	-3.50
IPI00291262	Clusterin precursor	T.TVASHTSDSDVPSGVTEVVVK.L	2	6.76	0.56	-4.30
IPI00291262	Clusterin precursor	T.VSDNELQEM*SNQGSK.Y	2	4.98	0.51	-3.44
IPI00291262	Clusterin precursor	T.VSDNELQEM*SNQGSK.Y	3	4.84	0.45	-2.78
IPI00291262	Clusterin precursor	V.ASHTSDSDVPSGVTEVVVK.L	2	6.03	0.57	-4.02
IPI00291262	Clusterin precursor	V.ASHTSDSDVPSGVTEVVVK.L	3	3.63	0.18	-1.69
IPI00291262	Clusterin precursor	V.NKEIQNAVNGVK.Q	2	3.36	0.14	-3.12
IPI00291262	Clusterin precursor	V.PSGVTEVVVK.L	1	2.33	0.26	-2.08
IPI00291262	Clusterin precursor	V.PSGVTEVVVK.L	2	3.56	0.29	-1.36
IPI00291262	Clusterin precursor	V.SDNELQEM*SNQGSK.Y	2	4.05	0.51	-1.76
IPI00291262	Clusterin precursor	Y.VNKEIQNAVNGVK.Q	2	3.14	0.17	-3.32
IPI00291395	fibronectin leucine rich transmembrane protein 1	K.LYLQDNAISHIPYNTLAK.M	2	3.99	0.44	-3.76

IPI00291395	fibronectin leucine rich transmembrane protein 1	R.GLFDDLGNLAQLLLR.N	3	3.13	0.21	-2.79
IPI00291395	fibronectin leucine rich transmembrane protein 1	R.GLTSIPADIPDDATTLYLQNNQINNAGIPQDLK.T	3	4.17	0.34	-3.61
IPI00291395	fibronectin leucine rich transmembrane protein 1	R.NHLSSIPSGLPHTLEELR.L	3	3.52	0.41	-2.23
IPI00291463	Radical S-adenosyl methionine domain-containing protein 2	R.FNVEEDMTEQIK.A	2	2.24	0.08	-1.90
IPI00291488	Isoform 1 of WAP four-disulfide core domain protein 2 precursor	K.CCSAGCATFCSLPNDKEGSCPQVNINFPQLGLCR.D	3	5.77	0.60	-1.24
IPI00291488	Isoform 1 of WAP four-disulfide core domain protein 2 precursor	K.CCSAGCATFCSLPNDKEGSCPQVNINFPQLGLCR.D	4	4.91	0.49	-2.04
IPI00291488	Isoform 1 of WAP four-disulfide core domain protein 2 precursor	R.DQCQVDSQCPGQM*K.C	2	4.41	0.58	-4.12
IPI00291488	Isoform 1 of WAP four-disulfide core domain protein 2 precursor	R.DQCQVDSQCPGQM*K.C	3	3.00	0.32	-2.75
IPI00291807	C3 and PZP-like, alpha-2-macroglobulin domain containing 8	A.AQPQAPGYLIAAPSVFR.A	2	2.97	0.40	-3.60
IPI00291807	C3 and PZP-like, alpha-2-macroglobulin domain containing 8	K.M*GEPVASAHTAK.I	2	2.14	0.24	-3.54
IPI00291807	C3 and PZP-like, alpha-2-macroglobulin domain containing 8	K.VPDSITSWVGEAVALSTSQGLGIAEPSLLK.T	3	3.08	0.29	-2.33
IPI00291807	C3 and PZP-like, alpha-2-macroglobulin domain containing 8	R.AAPALEKPIR.L	2	2.34	0.34	-3.56
IPI00291807	C3 and PZP-like, alpha-2-macroglobulin domain containing 8	R.DM*IPADVPEHFR.G	3	2.85	0.23	-1.26
IPI00291807	C3 and PZP-like, alpha-2-macroglobulin domain containing 8	R.VLFYFDEIPSR.C	2	3.41	0.34	-3.78
IPI00291866	Plasma protease C1 inhibitor precursor	A.TSSSSQDPESLQDR.G	2	3.86	0.48	-1.99
IPI00291866	Plasma protease C1 inhibitor precursor	D.PDLQVSAMQHQTVLELTETGVEAAAASAISVAR.T	3	4.03	0.38	-2.89
IPI00291866	Plasma protease C1 inhibitor precursor	F.SIASLLTQVLLGAGENTK.T	2	4.85	0.43	-4.85
IPI00291866	Plasma protease C1 inhibitor precursor	F.SIASLLTQVLLGAGENTK.T	3	5.09	0.43	-3.85
IPI00291866	Plasma protease C1 inhibitor precursor	H.QTVLELTETGVEAAAASAISVAR.T	3	4.28	0.31	-4.74
IPI00291866	Plasma protease C1 inhibitor precursor	K.AIM*EKLEM*SK.F	1	2.10	0.10	-2.87
IPI00291866	Plasma protease C1 inhibitor precursor	K.AIM*EKLEM*SK.F	2	3.23	0.22	-4.39
IPI00291866	Plasma protease C1 inhibitor precursor	K.AIM*EKLEM*SKFQPTLLTLPR.I	3	4.27	0.44	-3.99
IPI00291866	Plasma protease C1 inhibitor precursor	K.DFTCVHQALK.G	1	2.73	0.25	-4.06
IPI00291866	Plasma protease C1 inhibitor precursor	K.DFTCVHQALK.G	2	3.08	0.36	-2.55
IPI00291866	Plasma protease C1 inhibitor precursor	K.FPVFM*GR.V	2	2.15	0.17	-2.81
IPI00291866	Plasma protease C1 inhibitor precursor	K.FQPTLLTLPR.I	1	2.40	0.27	-3.35
IPI00291866	Plasma protease C1 inhibitor precursor	K.FQPTLLTLPR.I	2	2.91	0.24	-4.25
IPI00291866	Plasma protease C1 inhibitor precursor	K.GVTSVSQIFHSPDLAIR.D	2	4.56	0.38	-4.05
IPI00291866	Plasma protease C1 inhibitor precursor	K.GVTSVSQIFHSPDLAIR.D	3	3.80	0.39	-4.02
IPI00291866	Plasma protease C1 inhibitor precursor	K.HRLEDM*EQALSPSVFK.A	2	3.84	0.47	-3.54
IPI00291866	Plasma protease C1 inhibitor precursor	K.HRLEDM*EQALSPSVFK.A	3	2.16	0.11	-1.29

IPI00291866	Plasma protease C1 inhibitor precursor	K.KVETNM*AFSPFSIASLLTQVLLGAGENTK.T	2	4.23	0.54	-1.94
IPI00291866	Plasma protease C1 inhibitor precursor	K.KVETNM*AFSPFSIASLLTQVLLGAGENTK.T	3	4.79	0.31	-2.82
IPI00291866	Plasma protease C1 inhibitor precursor	K.KYPVAHFIDQTLK.A	2	4.46	0.41	-4.53
IPI00291866	Plasma protease C1 inhibitor precursor	K.KYPVAHFIDQTLK.A	3	3.50	0.33	-3.49
IPI00291866	Plasma protease C1 inhibitor precursor	K.LEM*SKFQPTLLTLPR.I	2	3.46	0.41	-2.30
IPI00291866	Plasma protease C1 inhibitor precursor	K.LEM*SKFQPTLLTLPR.I	3	2.73	0.23	-1.81
IPI00291866	Plasma protease C1 inhibitor precursor	K.LYHAFSAM*K.K	1	2.67	0.26	-3.69
IPI00291866	Plasma protease C1 inhibitor precursor	K.LYHAFSAM*K.K	2	2.27	0.17	-2.94
IPI00291866	Plasma protease C1 inhibitor precursor	K.NSVIKVPM*M*NSK.K	2	3.12	0.30	-3.68
IPI00291866	Plasma protease C1 inhibitor precursor	K.TNLESILSYPK.D	1	1.87	0.23	-2.91
IPI00291866	Plasma protease C1 inhibitor precursor	K.TNLESILSYPK.D	2	4.22	0.39	-3.40
IPI00291866	Plasma protease C1 inhibitor precursor	K.TNLESILSYPKDFTCVHQALK.G	2	3.84	0.39	-6.99
IPI00291866	Plasma protease C1 inhibitor precursor	K.TNLESILSYPKDFTCVHQALK.G	3	4.30	0.24	
IPI00291866	Plasma protease C1 inhibitor precursor	K.TNLESILSYPKDFTCVHQALK.G	4	2.81	0.31	-3.89
IPI00291866	Plasma protease C1 inhibitor precursor	K.TRM*EPFHFK.N	3	2.30	0.11	-4.21
IPI00291866	Plasma protease C1 inhibitor precursor	K.TTFDPK.K	1	1.57	0.11	-3.31
IPI00291866	Plasma protease C1 inhibitor precursor	K.TTFDPKK.T	1	1.95	0.15	-3.82
IPI00291866	Plasma protease C1 inhibitor precursor	K.TTFDPKK.T	2	2.29	0.20	-4.82
IPI00291866	Plasma protease C1 inhibitor precursor	K.VATTVISK.M	1	1.47	0.18	-2.06
IPI00291866	Plasma protease C1 inhibitor precursor	K.VATTVISK.M	2	1.99	0.08	-1.83
IPI00291866	Plasma protease C1 inhibitor precursor	K.VPM*M*NSK.K	2	1.87	0.08	-2.66
IPI00291866	Plasma protease C1 inhibitor precursor	K.VTTSQDM*LSIM*EK.L	2	4.20	0.40	-4.38
IPI00291866	Plasma protease C1 inhibitor precursor	K.YPVAHFIDQTLK.A	2	4.13	0.31	-3.91
IPI00291866	Plasma protease C1 inhibitor precursor	K.YPVAHFIDQTLK.A	3	4.27	0.38	-2.59
IPI00291866	Plasma protease C1 inhibitor precursor	K.YPVAHFIDQTLKAK.V	2	4.65	0.50	-3.21
IPI00291866	Plasma protease C1 inhibitor precursor	Q.PTLLTLPR.I	2	3.29	0.10	-1.97
IPI00291866	Plasma protease C1 inhibitor precursor	R.IKVTTSQDM*LSIM*EK.L	2	5.09	0.56	-4.09
IPI00291866	Plasma protease C1 inhibitor precursor	R.IKVTTSQDM*LSIM*EK.L	3	4.23	0.39	-4.29
IPI00291866	Plasma protease C1 inhibitor precursor	R.LEDM*EQALSPSVFK.A	1	1.82	0.41	-1.96
IPI00291866	Plasma protease C1 inhibitor precursor	R.LEDM*EQALSPSVFK.A	2	4.92	0.47	-4.91
IPI00291866	Plasma protease C1 inhibitor precursor	R.LEDM*EQALSPSVFK.A	3	3.86	0.14	-0.91
IPI00291866	Plasma protease C1 inhibitor precursor	R.LLDSLPSDTR.L	1	1.87	0.16	-3.69
IPI00291866	Plasma protease C1 inhibitor precursor	R.LLDSLPSDTR.L	2	3.84	0.29	-3.49
IPI00291866	Plasma protease C1 inhibitor precursor	R.LLDSLPSDTRLVLLNAIYLSAK.W	2	2.94	0.46	-4.43
IPI00291866	Plasma protease C1 inhibitor precursor	R.LLDSLPSDTRLVLLNAIYLSAK.W	3	4.34	0.54	-4.41
IPI00291866	Plasma protease C1 inhibitor precursor	R.LVLLNAIYLSAK.W	1	2.77	0.26	-2.02
IPI00291866	Plasma protease C1 inhibitor precursor	R.LVLLNAIYLSAK.W	2	5.03	0.55	-5.62
IPI00291866	Plasma protease C1 inhibitor precursor	R.M*EPFHFK.N	2	1.90	0.06	-2.30
IPI00291866	Plasma protease C1 inhibitor precursor	R.TLYSSSPR.V	1	1.97	0.18	-3.82
IPI00291866	Plasma protease C1 inhibitor precursor	R.TLYSSSPR.V	2	2.42	0.31	-3.66
IPI00291866	Plasma protease C1 inhibitor precursor	V.SQIFHSPDLAIR.D	2	3.68	0.35	-1.58
IPI00291866	Plasma protease C1 inhibitor precursor	V.TSVSQIFHSPDLAIR.D	2	3.95	0.39	-0.43

IPI00291922	Proteasome subunit alpha type-5	R.LFQVEYAIEAIK.L	2	4.17	0.27	-4.46
IPI00291939	Structural maintenance of chromosomes protein 1A	K.NQHLAKKSEVNDKNHEM*EEIR.K	3	2.92	0.13	
IPI00291987	Insulin-like growth factor-binding protein-like 1 precursor	K.SPEGTQALEELPGDHVNIAVQVR.G	3	4.63	0.35	-2.29
IPI00292071	Secretogranin-3 precursor	E.DNFEELQYFPNFYALLK.S	2	4.60	0.52	-4.55
IPI00292071	Secretogranin-3 precursor	E.LSAERPLNEQIAEAEEDKIKK.T	3	5.71	0.44	-3.17
IPI00292071	Secretogranin-3 precursor	G.KTEAYLEAIRK.N	2	3.41	0.32	-3.85
IPI00292071	Secretogranin-3 precursor	I.DDYDSTK.S	1	2.20	0.24	-1.01
IPI00292071	Secretogranin-3 precursor	I.TESQAHTLEDEVAEVLQK.L	2	5.45	0.53	-0.16
IPI00292071	Secretogranin-3 precursor	K.AITEKEKIEK.E	2	3.73	0.23	-3.17
IPI00292071	Secretogranin-3 precursor	K.AITEKEKIEKE.R	2	3.07	0.17	-3.21
IPI00292071	Secretogranin-3 precursor	K.AITEKEKIEKER.Q	2	3.95	0.19	-4.50
IPI00292071	Secretogranin-3 precursor	K.DDNSNPGGKTDEPKGKTEAYLEAIRK.N	3	5.15	0.49	-4.23
IPI00292071	Secretogranin-3 precursor	K.DSTKDDNSNPGGKTDEPK.G	2	3.57	0.35	-3.17
IPI00292071	Secretogranin-3 precursor	K.DSTKDDNSNPGGKTDEPK.G	3	2.44	0.16	-1.45
IPI00292071	Secretogranin-3 precursor	K.EAKEKETLITIM*K.T	2	3.19	0.36	-1.32
IPI00292071	Secretogranin-3 precursor	K.EANNYEEDPNKPTSWTENQAGK.I	2	3.62	0.44	-3.13
IPI00292071	Secretogranin-3 precursor	K.EANNYEEDPNKPTSWTENQAGK.I	3	4.16	0.46	-1.98
IPI00292071	Secretogranin-3 precursor	K.EKETLITIM*K.T	1	2.53	0.24	-4.08
IPI00292071	Secretogranin-3 precursor	K.EKETLITIM*K.T	2	3.22	0.32	-4.47
IPI00292071	Secretogranin-3 precursor	K.ETLITIM*K.T	1	2.59	0.10	-3.82
IPI00292071	Secretogranin-3 precursor	K.ETLITIM*K.T	2	1.85	0.07	-1.88
IPI00292071	Secretogranin-3 precursor	K.EYGSLK.D	1	1.95	0.07	-3.85
IPI00292071	Secretogranin-3 precursor	K.EYGSLKDSTK.D	1	2.72	0.36	-4.34
IPI00292071	Secretogranin-3 precursor	K.EYGSLKDSTK.D	2	2.29	0.29	-2.21
IPI00292071	Secretogranin-3 precursor	K.EYGSLKDSTKDDNSNPGGK.T	2	4.31	0.46	-6.45
IPI00292071	Secretogranin-3 precursor	K.EYGSLKDSTKDDNSNPGGK.T	3	2.44	0.32	-3.87
IPI00292071	Secretogranin-3 precursor	K.EYGSLKDSTKDDNSNPGGKTDEPK.G	3	5.59	0.42	-4.00
IPI00292071	Secretogranin-3 precursor	K.EYGSLKDSTKDDNSNPGGKTDEPK.G	4	2.57	0.25	-2.77
IPI00292071	Secretogranin-3 precursor	K.EYGSLKDSTKDDNSNPGGKTDEPKGK.T	3	4.48	0.42	-1.74
IPI00292071	Secretogranin-3 precursor	K.FQDDPDGLHQL.D	1	2.56	0.38	-2.80
IPI00292071	Secretogranin-3 precursor	K.FQDDPDGLHQL.D	2	3.22	0.24	-2.98
IPI00292071	Secretogranin-3 precursor	K.FQDDPDGLHQLDGTP.L	2	2.96	0.34	-3.89
IPI00292071	Secretogranin-3 precursor	K.FQDDPDGLHQLDGTPLTAEDIVH.K	3	5.17	0.40	-2.91
IPI00292071	Secretogranin-3 precursor	K.FQDDPDGLHQLDGTPLTAEDIVHK.I	2	5.34	0.58	-3.87
IPI00292071	Secretogranin-3 precursor	K.FQDDPDGLHQLDGTPLTAEDIVHK.I	3	5.26	0.46	-2.91
IPI00292071	Secretogranin-3 precursor	K.FQDDPDGLHQLDGTPLTAEDIVHK.I	4	4.51	0.46	-3.74
IPI00292071	Secretogranin-3 precursor	K.FQDDPDGLHQLDGTPLTAEDIVHKI.A	3	3.95	0.26	-2.80
IPI00292071	Secretogranin-3 precursor	K.GENDETVSNTLTLTNGLER.R	2	5.70	0.50	-3.91
IPI00292071	Secretogranin-3 precursor	K.GENDETVSNTLTLTNGLER.R	3	4.60	0.34	-3.58
IPI00292071	Secretogranin-3 precursor	K.GENDETVSNTLTLTNGLERR.T	3	2.69	0.19	-2.58

IPI00292071	Secretogranin-3 precursor	K.GILDKEEAEAIKR.I	2	3.75	0.33	-3.13
IPI00292071	Secretogranin-3 precursor	K.GILDKEEAEAIKR.I	3	4.52	0.40	-3.00
IPI00292071	Secretogranin-3 precursor	K.GKTEAYLEAIR.K	2	3.64	0.30	0.89
IPI00292071	Secretogranin-3 precursor	K.GKTEAYLEAIRK.N	2	3.22	0.15	-3.31
IPI00292071	Secretogranin-3 precursor	K.GNKEDYDLSK.M	1	3.09	0.30	-4.30
IPI00292071	Secretogranin-3 precursor	K.GNKEDYDLSK.M	2	3.16	0.34	-4.07
IPI00292071	Secretogranin-3 precursor	K.IPEKVTPM*AAIQDGLAK.G	3	4.27	0.34	-3.15
IPI00292071	Secretogranin-3 precursor	K.KGNKEDYDLSK.M	1	2.69	0.30	-5.42
IPI00292071	Secretogranin-3 precursor	K.KGNKEDYDLSK.M	2	3.65	0.32	-3.19
IPI00292071	Secretogranin-3 precursor	K.KGNKEDYDLSK.M	3	2.09	0.12	-4.57
IPI00292071	Secretogranin-3 precursor	K.LIDDYDSTK.S	1	2.22	0.37	-4.29
IPI00292071	Secretogranin-3 precursor	K.LIDDYDSTK.S	2	3.17	0.32	-3.99
IPI00292071	Secretogranin-3 precursor	K.LIDDYDSTKSGLDHK.F	2	3.68	0.39	-2.05
IPI00292071	Secretogranin-3 precursor	K.LIDDYDSTKSGLDHK.F	4	2.70	0.29	-2.34
IPI00292071	Secretogranin-3 precursor	K.LLNLGLITESQAHTLEDEVAEVLQK.L	2	5.69	0.58	-3.89
IPI00292071	Secretogranin-3 precursor	K.LLNLGLITESQAHTLEDEVAEVLQK.L	3	7.54	0.61	-7.09
IPI00292071	Secretogranin-3 precursor	K.LLNLGLITESQAHTLEDEVAEVLQK.L	4	5.16	0.44	-5.38
IPI00292071	Secretogranin-3 precursor	K.LNVEDVDSTK.N	1	2.79	0.37	-2.06
IPI00292071	Secretogranin-3 precursor	K.LNVEDVDSTK.N	2	3.21	0.29	-2.86
IPI00292071	Secretogranin-3 precursor	K.LNVEDVDSTKN.R	2	3.67	0.43	-4.92
IPI00292071	Secretogranin-3 precursor	K.LNVEDVDSTKNR.K	2	3.76	0.40	-1.35
IPI00292071	Secretogranin-3 precursor	K.LNVEDVDSTKNR.K	3	2.59	0.18	-2.01
IPI00292071	Secretogranin-3 precursor	K.M*EKEYGSLK.D	1	1.74	0.09	-2.21
IPI00292071	Secretogranin-3 precursor	K.M*EKEYGSLK.D	2	2.69	0.09	
IPI00292071	Secretogranin-3 precursor	K.M*EKEYGSLKDSTK.D	2	3.44	0.31	-1.33
IPI00292071	Secretogranin-3 precursor	K.M*EKEYGSLKDSTK.D	3	2.70	0.29	0.73
IPI00292071	Secretogranin-3 precursor	K.M*EKEYGSLKDSTKDDNSNPGGK.T	3	3.77	0.35	-0.54
IPI00292071	Secretogranin-3 precursor	K.M*EKEYGSLKDSTKDDNSNPGGKTDEPK.G	3	4.89	0.47	-2.52
IPI00292071	Secretogranin-3 precursor	K.M*EKEYGSLKDSTKDDNSNPGGKTDEPK.G	4	4.26	0.47	-1.49
IPI00292071	Secretogranin-3 precursor	K.M*RDFINKQADAYVEK.G	3	3.20	0.08	-2.34
IPI00292071	Secretogranin-3 precursor	K.NATDNISK.L	2	2.03	0.14	-2.73
IPI00292071	Secretogranin-3 precursor	K.QADAYVEK.G	1	2.15	0.21	-4.03
IPI00292071	Secretogranin-3 precursor	K.QADAYVEK.G	2	1.55	0.17	-3.51
IPI00292071	Secretogranin-3 precursor	K.QADAYVEKGILDKEEAEAIKR.I	4	3.56	0.22	-2.58
IPI00292071	Secretogranin-3 precursor	K.SGLDHKFQDDPDGLHQL.D	2	3.74	0.49	-2.94
IPI00292071	Secretogranin-3 precursor	K.SGLDHKFQDDPDGLHQLDGTPLTAEDIVHK.I	3	5.67	0.57	-3.51
IPI00292071	Secretogranin-3 precursor	K.SGLDHKFQDDPDGLHQLDGTPLTAEDIVHK.I	4	3.63	0.29	-2.72
IPI00292071	Secretogranin-3 precursor	K.SGLDHKFQDDPDGLHQLDGTPLTAEDIVHK.I	5	3.61	0.28	-3.94
IPI00292071	Secretogranin-3 precursor	K.SHEETDSTKEEAAK.M	2	3.68	0.53	-4.70
IPI00292071	Secretogranin-3 precursor	K.SHEETDSTKEEAAK.M	3	3.01	0.40	-2.51
IPI00292071	Secretogranin-3 precursor	K.SIDSEKEAKEKETLITIM*K.T	2	5.13	0.55	-1.15
IPI00292071	Secretogranin-3 precursor	K.SIDSEKEAKEKETLITIM*K.T	3	4.25	0.55	-1.23

IPI00292071	Secretogranin-3 precursor	K.SIDSEKEAKEKETLITIM*K.T	4	2.91	0.40	-0.33
IPI00292071	Secretogranin-3 precursor	K.TDEPKGKTEAYLEAIRK.N	2	3.16	0.24	-4.45
IPI00292071	Secretogranin-3 precursor	K.TDEPKGKTEAYLEAIRK.N	3	3.37	0.36	-3.46
IPI00292071	Secretogranin-3 precursor	K.TEAYLEAIR.K	2	3.57	0.28	-1.25
IPI00292071	Secretogranin-3 precursor	K.TEAYLEAIRK.N	2	3.20	0.30	-3.54
IPI00292071	Secretogranin-3 precursor	K.TYPPENKPGQSNYSFVDNLNLLK.A	3	1.99	0.13	-2.30
IPI00292071	Secretogranin-3 precursor	K.TYSEDNFEELQYFPNFYALLK.S	2	5.20	0.51	-4.60
IPI00292071	Secretogranin-3 precursor	K.TYSEDNFEELQYFPNFYALLK.S	3	5.42	0.38	-5.36
IPI00292071	Secretogranin-3 precursor	K.VTPM*AAIQDGLAK.G	2	3.34	0.32	-3.22
IPI00292071	Secretogranin-3 precursor	K.VTPM*AAIQDGLAKGENDETVSNTLTLTNGLE.R	3	5.34	0.50	-6.13
IPI00292071	Secretogranin-3 precursor	K.VTPM*AAIQDGLAKGENDETVSNTLTLTNGLER.R	3	5.51	0.45	-5.35
IPI00292071	Secretogranin-3 precursor	K.VTPM*AAIQDGLAKGENDETVSNTLTLTNGLER.R	4	3.77	0.26	-5.54
IPI00292071	Secretogranin-3 precursor	K.YGTISPEEGVSYLENLDEM*IALQTK.N	2	4.35	0.52	-5.48
IPI00292071	Secretogranin-3 precursor	K.YGTISPEEGVSYLENLDEM*IALQTK.N	3	5.74	0.47	-5.96
IPI00292071	Secretogranin-3 precursor	K.YGTISPEEGVSYLENLDEM*IALQTK.N	4	5.66	0.43	-3.16
IPI00292071	Secretogranin-3 precursor	L.DGTPLTAEDIVHK.I	2	2.97	0.23	-3.31
IPI00292071	Secretogranin-3 precursor	L.GLITESQAHTLEDEVAEVLQK.L	2	5.32	0.56	-3.78
IPI00292071	Secretogranin-3 precursor	L.GLITESQAHTLEDEVAEVLQK.L	3	4.47	0.34	-4.03
IPI00292071	Secretogranin-3 precursor	L.ITESQAHTLEDEVAEVLQK.L	2	4.38	0.48	-1.67
IPI00292071	Secretogranin-3 precursor	L.LNLGLITESQAHTLEDEVAEVLQK.L	3	6.33	0.53	-3.92
IPI00292071	Secretogranin-3 precursor	L.NLGLITESQAHTLEDEVAEVLQK.L	2	5.16	0.51	-3.41
IPI00292071	Secretogranin-3 precursor	L.SAERPLNEQIAEAEEDKIKK.T	3	5.10	0.37	-1.81
IPI00292071	Secretogranin-3 precursor	N.LGLITESQAHTLEDEVAEVLQK.L	2	4.85	0.48	-1.99
IPI00292071	Secretogranin-3 precursor	N.LGLITESQAHTLEDEVAEVLQK.L	3	3.63	0.32	-3.96
IPI00292071	Secretogranin-3 precursor	R.AVFDKIVSK.L	1	2.32	0.12	-2.41
IPI00292071	Secretogranin-3 precursor	R.AVFDKIVSK.L	2	2.72	0.26	-2.78
IPI00292071	Secretogranin-3 precursor	R.DFINKQADAYVEK.G	2	3.71	0.34	-2.60
IPI00292071	Secretogranin-3 precursor	R.ELSAERPLNEQIAEAEED.K	2	5.05	0.44	-3.90
IPI00292071	Secretogranin-3 precursor	R.ELSAERPLNEQIAEAEEDK.I	2	4.46	0.36	-5.00
IPI00292071	Secretogranin-3 precursor	R.ELSAERPLNEQIAEAEEDK.I	3	3.58	0.27	-3.69
IPI00292071	Secretogranin-3 precursor	R.ELSAERPLNEQIAEAEEDKI.K	2	5.16	0.35	-3.76
IPI00292071	Secretogranin-3 precursor	R.ELSAERPLNEQIAEAEEDKI.K	3	5.32	0.33	-2.61
IPI00292071	Secretogranin-3 precursor	R.ELSAERPLNEQIAEAEEDKIK.K	2	4.38	0.28	-2.36
IPI00292071	Secretogranin-3 precursor	R.ELSAERPLNEQIAEAEEDKIK.K	3	5.80	0.32	-1.07
IPI00292071	Secretogranin-3 precursor	R.ELSAERPLNEQIAEAEEDKIKK.T	2	4.49	0.37	-4.98
IPI00292071	Secretogranin-3 precursor	R.ELSAERPLNEQIAEAEEDKIKK.T	3	6.56	0.52	-4.30
IPI00292071	Secretogranin-3 precursor	R.ELSAERPLNEQIAEAEEDKIKK.T	5	2.83	0.25	-3.45
IPI00292071	Secretogranin-3 precursor	R.IYEENDR.A	1	1.57	0.06	-2.65
IPI00292071	Secretogranin-3 precursor	R.IYEENDR.A	2	2.31	0.06	-0.60
IPI00292071	Secretogranin-3 precursor	R.IYEENDRAVFDK.I	2	3.77	0.41	-4.53
IPI00292071	Secretogranin-3 precursor	R.IYEENDRAVFDKIVSK.L	2	3.56	0.22	-5.32
IPI00292071	Secretogranin-3 precursor	R.IYEENDRAVFDKIVSK.L	3	3.44	0.28	-5.37

IPI00292071	Secretogranin-3 precursor	R.KLIDDYDSTK.S	1	3.21	0.40	-3.53
IPI00292071	Secretogranin-3 precursor	R.KLIDDYDSTK.S	2	3.48	0.34	-3.28
IPI00292071	Secretogranin-3 precursor	R.KLIDDYDSTKSGLDHK.F	3	2.04	0.18	-1.13
IPI00292071	Secretogranin-3 precursor	R.KLIDDYDSTKSGLDHK.F	4	3.80	0.33	-2.45
IPI00292071	Secretogranin-3 precursor	R.KLIDDYDSTKSGLDHKFQ.D	3	4.41	0.37	-2.29
IPI00292071	Secretogranin-3 precursor	R.SSPLDNKLNVEDVDSTK.N	2	5.03	0.51	-5.13
IPI00292071	Secretogranin-3 precursor	R.SSPLDNKLNVEDVDSTK.N	3	3.48	0.43	-3.91
IPI00292071	Secretogranin-3 precursor	R.SSPLDNKLNVEDVDSTKN.R	2	4.79	0.52	-3.04
IPI00292071	Secretogranin-3 precursor	R.SSPLDNKLNVEDVDSTKNR.K	2	4.65	0.49	-3.49
IPI00292071	Secretogranin-3 precursor	R.SSPLDNKLNVEDVDSTKNR.K	3	4.13	0.56	-3.00
IPI00292071	Secretogranin-3 precursor	R.SSPLDNKLNVEDVDSTKNRK.L	3	3.96	0.46	-2.27
IPI00292071	Secretogranin-3 precursor	R.TKTYSEDNFEELQYFPN.F	2	5.24	0.49	-3.22
IPI00292071	Secretogranin-3 precursor	R.TKTYSEDNFEELQYFPNF.Y	2	4.35	0.45	-2.87
IPI00292071	Secretogranin-3 precursor	R.TKTYSEDNFEELQYFPNFY.A	2	3.42	0.25	-1.97
IPI00292071	Secretogranin-3 precursor	R.TKTYSEDNFEELQYFPNFYALLK.S	2	4.90	0.49	-3.89
IPI00292071	Secretogranin-3 precursor	R.TKTYSEDNFEELQYFPNFYALLK.S	3	5.67	0.42	-4.49
IPI00292071	Secretogranin-3 precursor	R.TKTYSEDNFEELQYFPNFYALLK.S	4	3.38	0.27	-2.97
IPI00292071	Secretogranin-3 precursor	S.AERPLNEQIAEAEEDKIKK.T	3	6.00	0.44	-2.42
IPI00292071	Secretogranin-3 precursor	T.YSEDNFEELQYFPNFYALLK.S	2	4.73	0.51	-4.50
IPI00292071	Secretogranin-3 precursor	Y.SEDNFEELQYFPNFYALLK.S	2	5.00	0.51	-3.31
IPI00292150	Latent-transforming growth factor beta-binding protein 2 precursor	K.AISM*LQGLCYR.S	2	2.74	0.35	0.60
IPI00292150	Latent-transforming growth factor beta-binding protein 2 precursor	L.FREQDAPVAGLQPVER.A	3	3.65	0.26	-1.18
IPI00292150	Latent-transforming growth factor beta-binding protein 2 precursor	R.EQDAPVAGLQPVER.A	2	4.12	0.47	-2.73
IPI00292150	Latent-transforming growth factor beta-binding protein 2 precursor	R.GAGGQSM*SEAPTGDHAPAPTR.M	2	4.12	0.49	0.76
IPI00292150	Latent-transforming growth factor beta-binding protein 2 precursor	R.GAGGQSM*SEAPTGDHAPAPTR.M	3	3.59	0.25	-1.70
IPI00292150	Latent-transforming growth factor beta-binding protein 2 precursor	R.SGEPPRPLPPAAPRPR.G	3	3.68	0.46	-2.93
IPI00292150	Latent-transforming growth factor beta-binding protein 2 precursor	R.SLGPGTCTLPLAQR.I	2	3.59	0.31	-2.95
IPI00292150	Latent-transforming growth factor beta-binding protein 2 precursor	R.SSAAGEGTLAR.A	2	2.79	0.16	-1.96
IPI00292150	Latent-transforming growth factor beta-binding protein 2 precursor	R.SSEVYAQLCNVAR.I	2	4.36	0.48	-3.22
IPI00292150	Latent-transforming growth factor beta-binding protein 2 precursor	R.STPLGQQQPAPR.T	2	3.11	0.41	-3.08
IPI00292218	Hepatocyte growth factor-like protein precursor	K.CEIAGWGETKGTGNDTVLNVALLNVISNQECNIK.H	3	1.90	0.10	2.99
IPI00292218	Hepatocyte growth factor-like protein precursor	R.QEATTVSCFR.G	2	2.68	0.32	-2.57

IPI00292218	Hepatocyte growth factor-like protein precursor	R.SPLNDFQVLR.G	2	3.19	0.28	-1.99
IPI00292218	Hepatocyte growth factor-like protein precursor	R.VALICLPPEWYVVPPGTK.C	2	2.87	0.22	-2.06
IPI00292218	Hepatocyte growth factor-like protein precursor	R.VALICLPPEWYVVPPGTK.C	3	2.77	0.06	-0.07
IPI00292218	Hepatocyte growth factor-like protein precursor	R.VSVFVDWIHK.V	3	1.54	0.15	-3.30
IPI00292300	contactin associated protein-like 5	K.LM*STLKDVISLK.F	3	3.19	0.20	-1.80
IPI00292300	contactin associated protein-like 5	K.SDVADFDGR.S	2	2.16	0.21	-3.04
IPI00292300	contactin associated protein-like 5	K.VTENLGLDSEVAK.A	2	4.20	0.35	-5.29
IPI00292300	contactin associated protein-like 5	R.LLNTPDGTPFTWWIGR.S	2	4.18	0.48	-4.45
IPI00292300	contactin associated protein-like 5	R.NLKETSLQVDNLPR.S	2	3.90	0.48	-3.46
IPI00292300	contactin associated protein-like 5	R.NLKETSLQVDNLPR.S	3	3.35	0.07	-0.38
IPI00292304	Uncharacterized protein C9orf4	R.ARGDTGADEAVPR.H	2	3.52	0.40	-3.05
IPI00292304	Uncharacterized protein C9orf4	R.DEEGVFENNR.V	2	2.86	0.27	-2.67
IPI00292304	Uncharacterized protein C9orf4	R.GDTGADEAVPR.H	2	3.23	0.23	-3.05
IPI00292304	Uncharacterized protein C9orf4	R.HDIDSPPASER.V	2	3.06	0.30	-3.31
IPI00292304	Uncharacterized protein C9orf4	R.HDSSYGTFAGEFYDLR.Y	3	2.39	0.08	-1.30
IPI00292304	Uncharacterized protein C9orf4	R.NPARDEEGVFENNR.V	2	3.62	0.35	-3.89
IPI00292304	Uncharacterized protein C9orf4	R.RPRQGGGGAGGSAAARAR.A	2	2.92	0.07	
IPI00292304	Uncharacterized protein C9orf4	R.YGKPGCNAETCDYFLSYR.M	3	3.43	0.17	-0.69
IPI00292393	Sodium channel protein type 4 subunit alpha	K.QASYMYRHSHDGSGDDAPEKEGLLANTM*SK.M	3	2.49	0.06	0.63
IPI00292496	Beta-tubulin 4Q	K.NM*M*AACDPR.H	2	1.73	0.12	0.11
IPI00292496	Beta-tubulin 4Q	R.LHFFM*PGFAPLTSR.G	3	3.20	0.06	-3.00
IPI00292496	Beta-tubulin 4Q	R.LHFFMPGFAPLTSR.G	3	2.62	0.11	-1.73
IPI00292530	Inter-alpha-trypsin inhibitor heavy chain H1 precursor	K.AAISGENAGLVR.A	1	2.74	0.41	-2.52
IPI00292530	Inter-alpha-trypsin inhibitor heavy chain H1 precursor	K.AAISGENAGLVR.A	2	3.81	0.42	-2.05
IPI00292530	Inter-alpha-trypsin inhibitor heavy chain H1 precursor	K.ADVQAHGEGQEFSITCLVDEEEM*KK.L	3	5.06	0.45	-4.17
IPI00292530	Inter-alpha-trypsin inhibitor heavy chain H1 precursor	K.ADVQAHGEGQEFSITCLVDEEEM*KK.L	4	3.88	0.29	-3.14
IPI00292530	Inter-alpha-trypsin inhibitor heavy chain H1 precursor	K.ELAAQTIK.K	1	1.85	0.13	-2.44
IPI00292530	Inter-alpha-trypsin inhibitor heavy chain H1 precursor	K.ELAAQTIK.K	2	1.98	0.06	-2.97
IPI00292530	Inter-alpha-trypsin inhibitor heavy chain H1 precursor	K.ELAAQTIKK.S	1	2.02	0.10	-3.94
IPI00292530	Inter-alpha-trypsin inhibitor heavy chain H1 precursor	K.ELAAQTIKK.S	2	2.04	0.15	-1.84
IPI00292530	Inter-alpha-trypsin inhibitor heavy chain H1 precursor	K.GSLVQASEANLQAAQDFVR.G	2	6.74	0.63	-4.19
IPI00292530	Inter-alpha-trypsin inhibitor heavy chain H1 precursor	K.GSLVQASEANLQAAQDFVR.G	3	6.29	0.46	-3.52

IPI00292530	Inter-alpha-trypsin inhibitor heavy chain H1 precursor	K.ILGDM*QPGDYFDLVLFGTR.V	2	5.55	0.50	-5.28
IPI00292530	Inter-alpha-trypsin inhibitor heavy chain H1 precursor	K.ILGDM*QPGDYFDLVLFGTR.V	3	3.84	0.31	-4.18
IPI00292530	Inter-alpha-trypsin inhibitor heavy chain H1 precursor	K.LDAQASFLPK.E	2	3.04	0.25	-3.46
IPI00292530	Inter-alpha-trypsin inhibitor heavy chain H1 precursor	K.QLVHHFEIDVDIFEPQGISK.L	2	2.96	0.32	-4.33
IPI00292530	Inter-alpha-trypsin inhibitor heavy chain H1 precursor	K.QLVHHFEIDVDIFEPQGISK.L	3	4.16	0.26	-5.40
IPI00292530	Inter-alpha-trypsin inhibitor heavy chain H1 precursor	K.QYYEGSEIVVAGR.I	1	1.03	0.05	-2.84
IPI00292530	Inter-alpha-trypsin inhibitor heavy chain H1 precursor	K.QYYEGSEIVVAGR.I	2	3.71	0.43	-3.33
IPI00292530	Inter-alpha-trypsin inhibitor heavy chain H1 precursor	K.TAFISDFAVTADGNAFIGDIK.D	2	4.24	0.46	-5.82
IPI00292530	Inter-alpha-trypsin inhibitor heavy chain H1 precursor	K.TAFISDFAVTADGNAFIGDIKDK.V	3	3.16	0.24	-3.02
IPI00292530	Inter-alpha-trypsin inhibitor heavy chain H1 precursor	K.VTFQLTYEEVLKR.N	3	2.82	0.25	-2.31
IPI00292530	Inter-alpha-trypsin inhibitor heavy chain H1 precursor	K.VTYDVSR.D	1	2.04	0.12	-2.91
IPI00292530	Inter-alpha-trypsin inhibitor heavy chain H1 precursor	K.VTYDVSR.D	2	2.43	0.15	-4.13
IPI00292530	Inter-alpha-trypsin inhibitor heavy chain H1 precursor	K.VTYDVSRDK.I	2	2.41	0.16	-2.82
IPI00292530	Inter-alpha-trypsin inhibitor heavy chain H1 precursor	Q.M*SLDYGFVTPLTSM*SIR.G	2	5.14	0.54	-2.23
IPI00292530	Inter-alpha-trypsin inhibitor heavy chain H1 precursor	R.EVAFDLEIPK.T	1	2.84	0.19	-2.85
IPI00292530	Inter-alpha-trypsin inhibitor heavy chain H1 precursor	R.EVAFDLEIPK.T	2	2.96	0.28	-2.50
IPI00292530	Inter-alpha-trypsin inhibitor heavy chain H1 precursor	R.FAHYVVTSQVVNTANEAR.E	2	6.75	0.55	-4.28
IPI00292530	Inter-alpha-trypsin inhibitor heavy chain H1 precursor	R.FAHYVVTSQVVNTANEAR.E	3	5.32	0.30	-6.35
IPI00292530	Inter-alpha-trypsin inhibitor heavy chain H1 precursor	R.GFSLDEATNLNGGLLR.G	2	5.26	0.50	-5.41
IPI00292530	Inter-alpha-trypsin inhibitor heavy chain H1 precursor	R.GHM*LENHVER.L	2	2.69	0.26	-3.36
IPI00292530	Inter-alpha-trypsin inhibitor heavy chain H1 precursor	R.GM*ADQDGLKPTIDKPSEDSPPLEM*LGPR.R	3	5.23	0.49	-2.98

IPI00292530	Inter-alpha-trypsin inhibitor heavy chain H1 precursor	R.GM*ADQDGLKPTIDKPSEDSPPLEM*LGPR.R	4	4.37	0.50	-2.33
IPI00292530	Inter-alpha-trypsin inhibitor heavy chain H1 precursor	R.IADNKQSSFK.A	1	2.61	0.25	-4.28
IPI00292530	Inter-alpha-trypsin inhibitor heavy chain H1 precursor	R.IADNKQSSFK.A	2	3.32	0.33	-2.92
IPI00292530	Inter-alpha-trypsin inhibitor heavy chain H1 precursor	R.IYEDHDATQQLQGFYSQVAK.P	2	4.97	0.57	-2.07
IPI00292530	Inter-alpha-trypsin inhibitor heavy chain H1 precursor	R.IYEDHDATQQLQGFYSQVAK.P	3	4.77	0.38	-2.00
IPI00292530	Inter-alpha-trypsin inhibitor heavy chain H1 precursor	R.KAAISGENAGLVR.A	1	2.78	0.35	-2.94
IPI00292530	Inter-alpha-trypsin inhibitor heavy chain H1 precursor	R.KAAISGENAGLVR.A	2	3.52	0.38	-3.71
IPI00292530	Inter-alpha-trypsin inhibitor heavy chain H1 precursor	R.LWAYLTIQELLAK.R	2	3.18	0.22	-3.86
IPI00292530	Inter-alpha-trypsin inhibitor heavy chain H1 precursor	R.NHM*QYEIVIK.V	1	1.85	0.06	-2.78
IPI00292530	Inter-alpha-trypsin inhibitor heavy chain H1 precursor	R.NHM*QYEIVIK.V	2	2.46	0.27	-2.80
IPI00292530	Inter-alpha-trypsin inhibitor heavy chain H1 precursor	R.NHM*QYEIVIK.V	3	3.85	0.20	-1.38
IPI00292530	Inter-alpha-trypsin inhibitor heavy chain H1 precursor	R.QAVDTAVDGVFIR.S	2	4.41	0.46	-2.76
IPI00292530	Inter-alpha-trypsin inhibitor heavy chain H1 precursor	R.QAVDTAVDGVFIR.S	3	3.69	0.29	-1.55
IPI00292530	Inter-alpha-trypsin inhibitor heavy chain H1 precursor	R.TM*EQFTIHLTVNPQSK.V	2	3.79	0.38	-3.65
IPI00292530	Inter-alpha-trypsin inhibitor heavy chain H1 precursor	R.TM*EQFTIHLTVNPQSK.V	3	3.17	0.33	-4.69
IPI00292550	Isoform 1 of Polypeptide N-acetylgalactosaminyltransferase 13	K.ATPYTFPGGTGHVINK.N	2	3.15	0.25	-3.67
IPI00292550	Isoform 1 of Polypeptide N-acetylgalactosaminyltransferase 13	K.AVLIPKDDQEK.M	2	2.38	0.12	-1.76
IPI00292550	Isoform 1 of Polypeptide N-acetylgalactosaminyltransferase 13	K.VDYGDVSVRK.T	2	2.92	0.41	-1.24
IPI00292550	Isoform 1 of Polypeptide N-acetylgalactosaminyltransferase 13	R.GNQLWEYDAER.L	2	3.42	0.36	-2.54
IPI00292550	Isoform 1 of Polypeptide N-acetylgalactosaminyltransferase 13	R.RYYSLGEIR.N	2	2.54	0.05	-2.09
IPI00292550	Isoform 1 of Polypeptide N-acetylgalactosaminyltransferase 13	R.SPHYLLSEVILVDDASERDFLK.L	4	3.22	0.28	-3.08

IPI00292550	Isoform 1 of Polypeptide N-acetylgalactosaminyltransferase 13	R.TDDLCLDVSR.L	2	3.38	0.33	-2.49
IPI00292550	Isoform 1 of Polypeptide N-acetylgalactosaminyltransferase 13	R.TPTM*AGGLFSIDR.D	2	3.02	0.45	-3.90
IPI00292550	Isoform 1 of Polypeptide N-acetylgalactosaminyltransferase 13	R.TVYSVINR.S	2	1.79	0.07	-2.20
IPI00292550	Isoform 1 of Polypeptide N-acetylgalactosaminyltransferase 13	R.YYSLGEIR.N	2	2.01	0.08	-1.87
IPI00292657	NADP-dependent leukotriene B4 12-hydroxydehydrogenase	K.EGDTM*M*GQQVAK.V	2	2.27	0.25	-3.66
IPI00292657	NADP-dependent leukotriene B4 12-hydroxydehydrogenase	R.LKEGDTM*M*GQQVAK.V	3	2.48	0.28	-1.26
IPI00292732	fibromodulin precursor	K.IPPVNTNLENLYLQGNR.I	2	4.97	0.43	-3.81
IPI00292732	fibromodulin precursor	K.IPPVNTNLENLYLQGNR.I	3	3.70	0.28	-3.34
IPI00292732	fibromodulin precursor	K.YLPFVPSR.M	2	1.64	0.17	-1.90
IPI00292732	fibromodulin precursor	R.DCPQECDCPPNFPTAM*YCDNR.N	3	5.34	0.40	
IPI00292732	fibromodulin precursor	R.KVPDGLPSALEQLYM*EHNNVYTVPDSYFR.G	3	3.14	0.28	-1.55
IPI00292732	fibromodulin precursor	R.LDGNEIKR.S	2	2.85	0.21	-2.63
IPI00292732	fibromodulin precursor	R.SAM*PADAPLCLR.L	2	2.25	0.13	-2.74
IPI00292732	fibromodulin precursor	R.SLILLDLSYNHLR.K	2	3.41	0.24	
IPI00292732	fibromodulin precursor	T.NLENLYLQGNR.I	2	3.28	0.35	-1.61
IPI00292791	Contactin-3 precursor	G.ELLLQGPVFIKEPSNSIFPVGSEDKK.I	3	4.48	0.43	-3.58
IPI00292791	Contactin-3 precursor	K.ATTDGGDGTSSEQIRIPR.I	3	1.85	0.20	-1.48
IPI00292791	Contactin-3 precursor	K.HGLVYSSAELK.V	2	3.34	0.42	-0.44
IPI00292791	Contactin-3 precursor	K.IEVQFPETLPAAK.G	2	4.02	0.36	-2.25
IPI00292791	Contactin-3 precursor	K.IGGGEPSLPSEK.V	2	2.51	0.25	-3.63
IPI00292791	Contactin-3 precursor	K.LECFALGNPIPQINWR.R	2	4.27	0.34	-4.73
IPI00292791	Contactin-3 precursor	K.LNGGNLVVINPNR.N	2	2.87	0.32	-1.93
IPI00292791	Contactin-3 precursor	K.LQFAYLENFK.T	2	4.04	0.37	-2.26
IPI00292791	Contactin-3 precursor	K.NGAALVLEER.T	2	3.94	0.29	-1.52
IPI00292791	Contactin-3 precursor	K.VLLNWEQVK.A	2	2.99	0.30	-1.45
IPI00292791	Contactin-3 precursor	R.ITSM*DAR.G	2	1.98	0.18	-2.94
IPI00292791	Contactin-3 precursor	R.SDGLPFSSK.I	2	2.64	0.07	-2.33
IPI00292791	Contactin-3 precursor	R.SDGVM*GEYEPK.I	2	3.16	0.38	-4.66
IPI00292791	Contactin-3 precursor	R.VLGSPTPLVLR.S	2	2.32	0.24	-2.12
IPI00292946	Thyroxine-binding globulin precursor	K.AQWANPFDPSKTEDSSSFLIDK.T	2	4.13	0.46	-3.11
IPI00292946	Thyroxine-binding globulin precursor	K.AQWANPFDPSKTEDSSSFLIDK.T	3	2.64	0.29	-4.87
IPI00292946	Thyroxine-binding globulin precursor	K.AVLHIGEK.G	2	2.54	0.22	-2.68
IPI00292946	Thyroxine-binding globulin precursor	K.EGQM*ESVEAAM*SSK.T	2	4.33	0.53	-4.07
IPI00292946	Thyroxine-binding globulin precursor	K.EGQM*ESVEAAM*SSK.T	3	4.60	0.36	-2.05
IPI00292946	Thyroxine-binding globulin precursor	K.FSISATYDLGATLLK.M	2	4.94	0.45	-6.35
IPI00292946	Thyroxine-binding globulin precursor	K.GTEAAAVPEVELSDQPENTFLHPIIQIDR.S	2	3.11	0.41	-3.97

IPI00292946	Thyroxine-binding globulin precursor	K.GTEAAAVPEVELSDQPENTFLHPIIQIDR.S	3	4.04	0.49	-3.74
IPI00292946	Thyroxine-binding globulin precursor	K.M*GIQHAYSENADFSGLTEDNGLK.L	2	5.52	0.56	-1.10
IPI00292946	Thyroxine-binding globulin precursor	K.M*GIQHAYSENADFSGLTEDNGLK.L	3	5.96	0.43	-1.96
IPI00292946	Thyroxine-binding globulin precursor	K.M*GIQHAYSENADFSGLTEDNGLKLSNAAHK.A	4	4.92	0.46	-3.62
IPI00292946	Thyroxine-binding globulin precursor	K.M*SSINADFAFNLYR.R	2	4.90	0.55	-4.59
IPI00292946	Thyroxine-binding globulin precursor	K.NALALFVLPK.E	1	2.18	0.09	-4.04
IPI00292946	Thyroxine-binding globulin precursor	K.NALALFVLPK.E	2	4.40	0.43	-2.81
IPI00292946	Thyroxine-binding globulin precursor	K.NALALFVLPKEGQM*ESVEAAM*SSK.T	3	4.70	0.49	-0.98
IPI00292946	Thyroxine-binding globulin precursor	K.QEINSHVEM*QTK.G	2	1.95	0.24	-4.14
IPI00292946	Thyroxine-binding globulin precursor	K.TEDSSSFLIDK.T	2	4.17	0.51	-2.03
IPI00292946	Thyroxine-binding globulin precursor	R.LLQKGWVDLFVPK.F	3	2.91	0.28	-1.15
IPI00292946	Thyroxine-binding globulin precursor	R.SFM*LLILER.S	2	3.54	0.30	-3.48
IPI00292946	Thyroxine-binding globulin precursor	R.SFMLLILER.S	2	3.45	0.21	-1.70
IPI00292950	Serpin peptidase inhibitor, clade D (Heparin cofactor), member 1	F.TVDRPFLFLIYEHR.T	3	3.94	0.18	-2.73
IPI00292950	Serpin peptidase inhibitor, clade D (Heparin cofactor), member 1	G.GSKGPLDQLEK.G	2	3.02	0.28	-2.82
IPI00292950	Serpin peptidase inhibitor, clade D (Heparin cofactor), member 1	K.DALENIDPATQM*M*ILNCIYFK.G	3	3.00	0.10	
IPI00292950	Serpin peptidase inhibitor, clade D (Heparin cofactor), member 1	K.FKLEKNYNLVESLK.L	2	4.26	0.27	-4.34
IPI00292950	Serpin peptidase inhibitor, clade D (Heparin cofactor), member 1	K.GLIKDALENIDPATQM*M*ILNCIYFK.G	3	5.16	0.47	-4.51
IPI00292950	Serpin peptidase inhibitor, clade D (Heparin cofactor), member 1	K.GPLDQLEK.G	1	2.14	0.13	-2.54
IPI00292950	Serpin peptidase inhibitor, clade D (Heparin cofactor), member 1	K.GPLDQLEK.G	2	2.89	0.12	-2.84
IPI00292950	Serpin peptidase inhibitor, clade D (Heparin cofactor), member 1	K.HQGTITVNEEGTQATTVTTVGFM*PLSTQVR.F	3	4.25	0.31	-3.03
IPI00292950	Serpin peptidase inhibitor, clade D (Heparin cofactor), member 1	K.NGNM*AGISDQR.I	2	3.89	0.12	-2.52
IPI00292950	Serpin peptidase inhibitor, clade D (Heparin cofactor), member 1	K.NYNLVESLK.L	1	3.17	0.17	-3.68
IPI00292950	Serpin peptidase inhibitor, clade D (Heparin cofactor), member 1	K.NYNLVESLK.L	2	3.04	0.26	-2.94
IPI00292950	Serpin peptidase inhibitor, clade D (Heparin cofactor), member 1	K.QFPILLDFK.T	1	2.22	0.17	-3.25
IPI00292950	Serpin peptidase inhibitor, clade D (Heparin cofactor), member 1	K.QFPILLDFK.T	2	2.45	0.28	-3.27
IPI00292950	Serpin peptidase inhibitor, clade D (Heparin cofactor), member 1	K.QFPILLDFKTK.V	2	2.86	0.11	-2.99

IPI00292950	Serpin peptidase inhibitor, clade D (Heparin cofactor), member 1	K.TLEAQLTPR.V	1	2.13	0.24	-3.31
IPI00292950	Serpin peptidase inhibitor, clade D (Heparin cofactor), member 1	K.TLEAQLTPR.V	2	3.44	0.32	-3.05
IPI00292950	Serpin peptidase inhibitor, clade D (Heparin cofactor), member 1	K.VREYYFAEAQIADFSDPAFISK.T	2	4.62	0.49	-5.11
IPI00292950	Serpin peptidase inhibitor, clade D (Heparin cofactor), member 1	K.VREYYFAEAQIADFSDPAFISK.T	3	3.69	0.34	-6.18
IPI00292950	Serpin peptidase inhibitor, clade D (Heparin cofactor), member 1	K.YEITTIHNLFR.K	2	3.51	0.49	-3.80
IPI00292950	Serpin peptidase inhibitor, clade D (Heparin cofactor), member 1	K.YEITTIHNLFR.K	3	2.96	0.38	-3.35
IPI00292950	Serpin peptidase inhibitor, clade D (Heparin cofactor), member 1	R.EVVKVSM*M*QTK.G	2	2.80	0.31	-2.58
IPI00292950	Serpin peptidase inhibitor, clade D (Heparin cofactor), member 1	R.EYYFAEAQIADFSDPAFISK.T	2	6.26	0.61	-4.43
IPI00292950	Serpin peptidase inhibitor, clade D (Heparin cofactor), member 1	R.EYYFAEAQIADFSDPAFISK.T	3	4.91	0.51	-4.21
IPI00292950	Serpin peptidase inhibitor, clade D (Heparin cofactor), member 1	R.FTVDRPFLFLIYEHR.T	2	2.49	0.18	-5.40
IPI00292950	Serpin peptidase inhibitor, clade D (Heparin cofactor), member 1	R.FTVDRPFLFLIYEHR.T	3	5.76	0.40	-3.94
IPI00292950	Serpin peptidase inhibitor, clade D (Heparin cofactor), member 1	R.FTVDRPFLFLIYEHR.T	4	3.61	0.28	-2.55
IPI00292950	Serpin peptidase inhibitor, clade D (Heparin cofactor), member 1	R.IQRLNILNAK.F	2	2.76	0.17	-2.15
IPI00292950	Serpin peptidase inhibitor, clade D (Heparin cofactor), member 1	R.M*LFDKNGNM*AGISDQR.I	2	4.15	0.49	-2.94
IPI00292950	Serpin peptidase inhibitor, clade D (Heparin cofactor), member 1	R.M*LFDKNGNM*AGISDQR.I	3	4.48	0.44	-4.28
IPI00292950	Serpin peptidase inhibitor, clade D (Heparin cofactor), member 1	R.MLFDKNGNM*AGISDQR.I	2	3.51	0.15	
IPI00292950	Serpin peptidase inhibitor, clade D (Heparin cofactor), member 1	R.NFGYTLR.S	1	2.23	0.18	-2.45
IPI00292950	Serpin peptidase inhibitor, clade D (Heparin cofactor), member 1	R.NFGYTLR.S	2	2.59	0.09	-2.17
IPI00292950	Serpin peptidase inhibitor, clade D (Heparin cofactor), member 1	R.SVNDLYIQK.Q	1	2.17	0.14	-3.93
IPI00292950	Serpin peptidase inhibitor, clade D (Heparin cofactor), member 1	R.SVNDLYIQK.Q	2	3.61	0.22	-3.42
IPI00292950	Serpin peptidase inhibitor, clade D (Heparin cofactor), member 1	R.SVNDLYIQKQFPILLDFK.T	2	3.70	0.30	-2.27

IPI00292950	Serpin peptidase inhibitor, clade D (Heparin cofactor), member 1	R.SVNDLYIQKQFPILLDFK.T	3	4.18	0.38	-3.83
IPI00292950	Serpin peptidase inhibitor, clade D (Heparin cofactor), member 1	R.SVNDLYIQKQFPILLDFKTK.V	2	4.66	0.55	-5.45
IPI00292950	Serpin peptidase inhibitor, clade D (Heparin cofactor), member 1	R.SVNDLYIQKQFPILLDFKTK.V	3	5.23	0.45	-3.62
IPI00292950	Serpin peptidase inhibitor, clade D (Heparin cofactor), member 1	R.TSCLLFM*GR.V	2	3.15	0.36	-2.78
IPI00292950	Serpin peptidase inhibitor, clade D (Heparin cofactor), member 1	R.TSCLLFMGR.V	2	2.66	0.34	
IPI00292950	Serpin peptidase inhibitor, clade D (Heparin cofactor), member 1	Y.FAEAQIADFSDPAFISK.T	2	5.24	0.49	-3.25
IPI00292950	Serpin peptidase inhibitor, clade D (Heparin cofactor), member 1	Y.FAEAQIADFSDPAFISK.T	3	4.47	0.39	-2.47
IPI00293088	Lysosomal alpha-glucosidase precursor	A.VPTQCDVPPNSR.F	2	3.58	0.46	-1.98
IPI00293088	Lysosomal alpha-glucosidase precursor	K.AITQEQCEAR.G	2	2.83	0.28	-2.03
IPI00293088	Lysosomal alpha-glucosidase precursor	L.DVM*M*ETENR.L	2	3.18	0.28	-2.86
IPI00293088	Lysosomal alpha-glucosidase precursor	R.AGYIIPLQGPGLTTTESR.Q	2	3.03	0.18	-1.87
IPI00293088	Lysosomal alpha-glucosidase precursor	R.APSPLYSVEFSEEPFGVIVHR.Q	3	4.69	0.42	-2.89
IPI00293088	Lysosomal alpha-glucosidase precursor	R.GAYTQVIFLAR.N	2	3.53	0.25	-2.50
IPI00293088	Lysosomal alpha-glucosidase precursor	R.LDVM*M*ETENR.L	2	3.36	0.40	-3.22
IPI00293088	Lysosomal alpha-glucosidase precursor	R.NHNSLLSLPQEPYSFSEPAQQAM*R.K	3	5.32	0.53	-4.98
IPI00293088	Lysosomal alpha-glucosidase precursor	R.VTSEGAGLQLQK.V	2	3.96	0.33	-2.37
IPI00293095	Isoform 1 of Coiled-coil domain-containing protein 83	R.DLMSSSDESTILHLSHENSIEDLQYVK.I	3	3.20	0.12	
IPI00293128	Exostosin-1	K.ASISTENFRPNFDVSIPLFSK.D	3	2.87	0.19	-2.34
IPI00293128	Exostosin-1	K.IAESYQNILAAIEGSR.F	3	3.19	0.21	-3.89
IPI00293128	Exostosin-1	R.LDPVLFKDQVSILR.K	3	2.87	0.25	-1.28
IPI00293128	Exostosin-1	R.LLLQIPSTIR.S	2	2.68	0.16	-2.56
IPI00293276	Macrophage migration inhibitory factor	K.LLCGLLAER.L	1	2.01	0.14	-2.18
IPI00293276	Macrophage migration inhibitory factor	K.LLCGLLAER.L	2	3.27	0.23	-1.65
IPI00293276	Macrophage migration inhibitory factor	M.PM*FIVNTNVPR.A	2	3.28	0.38	-2.71
IPI00293303	Legumain precursor	K.ASSPVPLPPVTHLDLTPSPDVPLTIM*K.R	3	4.55	0.46	-4.11
IPI00293303	Legumain precursor	K.DYTGEDVTPQNFLAVLR.G	2	5.26	0.48	-5.29
IPI00293303	Legumain precursor	K.DYTGEDVTPQNFLAVLRGDAEAVK.G	3	3.02	0.38	-3.31
IPI00293303	Legumain precursor	K.IVSLLAASEAEVEQLLSER.A	2	5.62	0.45	-2.53
IPI00293303	Legumain precursor	K.IVSLLAASEAEVEQLLSER.A	3	3.26	0.36	-3.33
IPI00293303	Legumain precursor	K.LM*NTNDLEESR.Q	2	3.05	0.10	-3.02
IPI00293303	Legumain precursor	K.VM*QFQGM*K.R	2	2.27	0.26	-0.30
IPI00293303	Legumain precursor	R.KASSPVPLPPVTHLDLTPSPDVPLTIM*K.R	3	5.13	0.44	-4.02
IPI00293303	Legumain precursor	R.KIVSLLAASEAEVEQLLSER.A	3	2.99	0.23	-4.96
IPI00293361	Isoform 2 of Small G protein signaling modulator 2	K.M*AALFTKVGK.T	2	1.62	0.08	-2.47

IPI00293396	adaptor-related protein complex 1, gamma 1 subunit isoform a	R.TQAEEREMIQK.E	2	1.24	0.05	-7.43
IPI00293460	ATP-binding cassette sub-family A member 1	R.SVVLTSHSMEECEALCTRM*AIMVNGR.F	3	2.91	0.07	-4.39
IPI00293460	ATP-binding cassette sub-family A member 1	S.VVLTSHSMEECEALCTRMAIMVNGR.F	3	3.75	0.22	1.10
IPI00293464	DNA damage-binding protein 1	K.LVFSNVNLK.E	2	2.32	0.21	-1.76
IPI00293464	DNA damage-binding protein 1	K.VTLGTQPTVLR.T	2	2.80	0.23	-1.70
IPI00293464	DNA damage-binding protein 1	R.ILKLPSFELLHK.E	3	3.91	0.31	-3.65
IPI00293530	C3a anaphylatoxin chemotactic receptor	K.IPSGFPIEDHETSPLDNSDAFLSTHLK.L	3	5.99	0.58	-3.00
IPI00293530	C3a anaphylatoxin chemotactic receptor	K.IPSGFPIEDHETSPLDNSDAFLSTHLK.L	4	4.55	0.26	-2.44
IPI00293539	Isoform 2 of Cadherin-11 precursor	K.GKEGQVLQR.S	2	3.14	0.21	-1.81
IPI00293539	Isoform 2 of Cadherin-11 precursor	K.YILSGEGAGTIFVIDDK.S	2	2.93	0.29	
IPI00293539	Isoform 2 of Cadherin-11 precursor	R.VLDVNDNAPK.F	2	2.66	0.16	-1.85
IPI00293679	Isoform 1 of Potassium voltage-gated channel subfamily KQT member 4	K.SLQTRVDQIVGRGPGDR.K	3	3.42	0.06	
IPI00293723	Neurexophilin-4 precursor	R.AGAAGALPAQR.T	1	2.72	0.32	-4.31
IPI00293723	Neurexophilin-4 precursor	R.AGAAGALPAQR.T	2	3.03	0.23	-1.46
IPI00293723	Neurexophilin-4 precursor	R.AGAAGALPAQRT.K	1	2.01	0.17	-1.96
IPI00293723	Neurexophilin-4 precursor	R.AGAAGALPAQRT.K	2	3.12	0.28	-0.92
IPI00293723	Neurexophilin-4 precursor	R.SSDGLGVGR.A	1	1.61	0.10	-1.89
IPI00293723	Neurexophilin-4 precursor	R.SSDGLGVGR.A	2	3.38	0.15	-2.35
IPI00293748	Isoform 1 of Multiple inositol polyphosphate phosphatase 1 precursor	C.SLLEPRDPVASSLSPYFGTK.T	3	4.36	0.28	-1.91
IPI00293748	Isoform 1 of Multiple inositol polyphosphate phosphatase 1 precursor	K.DKEPLTAYNYKK.Q	2	3.05	0.23	-1.55
IPI00293748	Isoform 1 of Multiple inositol polyphosphate phosphatase 1 precursor	K.TGPEM*QNILK.K	2	2.87	0.17	-0.45
IPI00293748	Isoform 1 of Multiple inositol polyphosphate phosphatase 1 precursor	K.TGPEM*QNILKK.V	3	2.70	0.20	-3.66
IPI00293748	Isoform 1 of Multiple inositol polyphosphate phosphatase 1 precursor	R.CM*DSSAAFLQGLWQHYHPGLPPPDVADM*EFGPPTVNDK.L	4	4.55	0.29	-2.05
IPI00293748	Isoform 1 of Multiple inositol polyphosphate phosphatase 1 precursor	R.DPVASSLSPYFGTK.T	2	4.34	0.38	-5.10
IPI00293748	Isoform 1 of Multiple inositol polyphosphate phosphatase 1 precursor	R.GYGYTINSR.S	2	2.85	0.33	-0.64
IPI00293748	Isoform 1 of Multiple inositol polyphosphate phosphatase 1 precursor	R.LASLFPALFSR.E	1	2.01	0.21	-3.33
IPI00293748	Isoform 1 of Multiple inositol polyphosphate phosphatase 1 precursor	R.LASLFPALFSR.E	2	2.98	0.24	-2.78
IPI00293748	Isoform 1 of Multiple inositol polyphosphate phosphatase 1 precursor	R.SSCTLFQDIFQHLDK.A	3	2.69	0.29	-1.53
IPI00293748	Isoform 1 of Multiple inositol polyphosphate phosphatase 1 precursor	R.SSCTLFQDIFQHLDKAVEQK.Q	3	3.46	0.28	-4.92

IPI00293748	Isoform 1 of Multiple inositol polyphosphate phosphatase 1 precursor	R.SSCTLFQDIFQHLDKAVEQK.Q	4	2.86	0.13	-4.76
IPI00293748	Isoform 1 of Multiple inositol polyphosphate phosphatase 1 precursor	R.VQM*LLNEK.V	2	2.90	0.09	-0.79
IPI00293757	Isoform 1 of Netrin receptor UNC5C precursor	K.TFEQEPLGK.E	2	2.88	0.33	-1.81
IPI00293757	Isoform 1 of Netrin receptor UNC5C precursor	R.EVSIEISR.Q	1	2.21	0.15	-2.82
IPI00293757	Isoform 1 of Netrin receptor UNC5C precursor	R.EVSIEISR.Q	2	1.64	0.11	-2.52
IPI00293757	Isoform 1 of Netrin receptor UNC5C precursor	R.KTFEQEPLGK.E	2	3.31	0.36	-2.25
IPI00293836	Isoform 3 of Cell adhesion molecule 2 precursor	K.ALRDNRIELVR.A	2	2.11	0.06	-2.53
IPI00293836	Isoform 3 of Cell adhesion molecule 2 precursor	K.AYLTVLGVPEKPQISGFSSPVM*EGDLM*QLTCK.T	3	4.26	0.48	-3.53
IPI00293836	Isoform 3 of Cell adhesion molecule 2 precursor	K.AYLTVLGVPEKPQISGFSSPVM*EGDLM*QLTCK.T	4	4.50	0.37	-1.96
IPI00293836	Isoform 3 of Cell adhesion molecule 2 precursor	K.DGGELPDPDR.M	2	3.13	0.31	-3.81
IPI00293836	Isoform 3 of Cell adhesion molecule 2 precursor	K.DGGELPDPDRM*VVSGR.E	3	1.88	0.20	-3.43
IPI00293836	Isoform 3 of Cell adhesion molecule 2 precursor	K.IIPSTPFPQEGQPLILTCESK.G	2	3.84	0.41	-5.56
IPI00293836	Isoform 3 of Cell adhesion molecule 2 precursor	K.IIPSTPFPQEGQPLILTCESK.G	3	3.75	0.37	-5.43
IPI00293836	Isoform 3 of Cell adhesion molecule 2 precursor	K.TFTVSSTLDFR.V	2	3.54	0.49	-4.60
IPI00293836	Isoform 3 of Cell adhesion molecule 2 precursor	K.TSGSKPAADIR.W	1	1.61	0.20	-3.66
IPI00293836	Isoform 3 of Cell adhesion molecule 2 precursor	K.TSGSKPAADIR.W	2	3.16	0.22	-1.65
IPI00293836	Isoform 3 of Cell adhesion molecule 2 precursor	K.YLKEEDANR.K	2	3.09	0.20	-2.65
IPI00293836	Isoform 3 of Cell adhesion molecule 2 precursor	K.YLKEEDANRK.T	2	2.71	0.19	-3.23
IPI00293836	Isoform 3 of Cell adhesion molecule 2 precursor	K.YLKEEDANRK.T	3	3.41	0.25	-3.72
IPI00293836	Isoform 3 of Cell adhesion molecule 2 precursor	R.ELNILFLNK.T	2	2.70	0.16	-3.82
IPI00293836	Isoform 3 of Cell adhesion molecule 2 precursor	R.KTFTVSSTLDFR.V	3	1.97	0.20	-3.60
IPI00293836	Isoform 3 of Cell adhesion molecule 2 precursor	R.SDDGVAVICR.V	1	2.56	0.23	-3.65
IPI00293836	Isoform 3 of Cell adhesion molecule 2 precursor	R.SDDGVAVICR.V	2	3.55	0.32	-3.24
IPI00293836	Isoform 3 of Cell adhesion molecule 2 precursor	R.VDHESLNATPQVAM*QVLEIHYTPSVK.I	3	4.89	0.49	-3.72
IPI00293849	Receptor-type tyrosine-protein phosphatase mu precursor	K.AVSSFDPEIDLSNQSGR.V	2	5.07	0.45	-3.84
IPI00293849	Receptor-type tyrosine-protein phosphatase mu precursor	K.GFGPPATNQFTTK.I	2	4.06	0.43	-1.39
IPI00293849	Receptor-type tyrosine-protein phosphatase mu precursor	K.SNSPPGLLNVYVK.V	2	3.26	0.43	-2.52
IPI00293925	Isoform 1 of Ficolin-3 precursor	K.YGIDWASGR.G	2	2.73	0.21	-2.12
IPI00293925	Isoform 1 of Ficolin-3 precursor	R.ALPVFCDM*DTEGGGWLVFQR.R	2	4.27	0.50	-8.17
IPI00293925	Isoform 1 of Ficolin-3 precursor	R.ALPVFCDM*DTEGGGWLVFQR.R	3	4.12	0.44	-5.16
IPI00293925	Isoform 1 of Ficolin-3 precursor	R.LLGEVDHYQLALGK.F	2	3.79	0.38	-1.02
IPI00293925	Isoform 1 of Ficolin-3 precursor	R.TFAHYATFR.L	2	2.69	0.25	-3.71
IPI00293971	Sodium/potassium-transporting ATPase subunit beta-2	R.INAANIATDDER.D	2	3.82	0.43	-3.15
IPI00293971	Sodium/potassium-transporting ATPase subunit beta-2	R.INAANIATDDERD.K	2	3.65	0.19	-2.46

IPI00293971	Sodium/potassium-transporting ATPase subunit beta-2	R.INAANIATDDERDKFAGR.V	2	3.89	0.32	-3.80
IPI00293971	Sodium/potassium-transporting ATPase subunit beta-2	R.INAANIATDDERDKFAGR.V	3	2.98	0.19	-2.38
IPI00293971	Sodium/potassium-transporting ATPase subunit beta-2	R.INAANIATDDERDKFAGR.V	4	2.56	0.32	-1.49
IPI00294004	Vitamin K-dependent protein S precursor	K.ASFTCTCKPGWQGEK.C	2	3.83	0.33	
IPI00294004	Vitamin K-dependent protein S precursor	K.DCKDVDECSLKPSICGTAVCK.N	2	4.96	0.32	
IPI00294004	Vitamin K-dependent protein S precursor	K.DCKDVDECSLKPSICGTAVCK.N	3	3.74	0.19	
IPI00294004	Vitamin K-dependent protein S precursor	K.DVDECSLKPSICGTAVCK.N	2	3.58	0.46	
IPI00294004	Vitamin K-dependent protein S precursor	K.EAVM*DINKPGPLFKPENGLLETK.V	2	3.34	0.15	
IPI00294004	Vitamin K-dependent protein S precursor	K.EAVM*DINKPGPLFKPENGLLETK.V	3	3.55	0.36	-3.93
IPI00294004	Vitamin K-dependent protein S precursor	K.EAVM*DINKPGPLFKPENGLLETK.V	4	2.74	0.33	-1.67
IPI00294004	Vitamin K-dependent protein S precursor	K.HCLVTVEK.G	2	2.86	0.16	
IPI00294004	Vitamin K-dependent protein S precursor	K.IEVQLKNEHTSK.I	2	3.18	0.27	-2.84
IPI00294004	Vitamin K-dependent protein S precursor	K.ITTGGDVINNGLWNM*VSVEELEHSISIK.I	3	3.08	0.12	-3.21
IPI00294004	Vitamin K-dependent protein S precursor	K.SQDILLSVENTVIYR.I	2	2.25	0.10	-3.34
IPI00294004	Vitamin K-dependent protein S precursor	K.VYFAGFPR.K	1	1.88	0.20	-1.52
IPI00294004	Vitamin K-dependent protein S precursor	K.VYFAGFPR.K	2	3.10	0.20	-1.52
IPI00294004	Vitamin K-dependent protein S precursor	R.FSAEFDFR.T	2	3.05	0.15	-3.27
IPI00294004	Vitamin K-dependent protein S precursor	R.IQALSLCSDQQSHLEFR.V	2	5.35	0.47	
IPI00294004	Vitamin K-dependent protein S precursor	R.IQALSLCSDQQSHLEFR.V	3	2.77	0.23	-2.18
IPI00294004	Vitamin K-dependent protein S precursor	R.KVESELIKPINPR.L	2	4.29	0.37	-4.11
IPI00294004	Vitamin K-dependent protein S precursor	R.KVESELIKPINPR.L	3	2.91	0.23	-2.23
IPI00294004	Vitamin K-dependent protein S precursor	R.NNLELSTPLK.I	2	2.82	0.15	-1.85
IPI00294004	Vitamin K-dependent protein S precursor	R.NNLELSTPLKIETISHEDLQR.Q	2	3.95	0.30	
IPI00294004	Vitamin K-dependent protein S precursor	R.NNLELSTPLKIETISHEDLQR.Q	3	4.42	0.41	-3.77
IPI00294004	Vitamin K-dependent protein S precursor	R.QLAVLDK.A	1	1.66	0.09	-2.85
IPI00294004	Vitamin K-dependent protein S precursor	R.QSTNAYPDLR.S	2	2.54	0.15	-1.46
IPI00294004	Vitamin K-dependent protein S precursor	R.SCVNAIPDQCSPLPCNEDGYM*SCKDGK.A	3	3.72	0.39	-2.21
IPI00294004	Vitamin K-dependent protein S precursor	R.SFQTGLFTAAR.Q	1	2.04	0.35	-3.06
IPI00294004	Vitamin K-dependent protein S precursor	R.SFQTGLFTAAR.Q	2	4.28	0.43	-2.15
IPI00294004	Vitamin K-dependent protein S precursor	R.VNRNNLELSTPLK.I	2	2.82	0.22	0.24
IPI00294004	Vitamin K-dependent protein S precursor	R.VNRNNLELSTPLKIETISHEDLQR.Q	3	6.52	0.51	-4.23
IPI00294004	Vitamin K-dependent protein S precursor	R.VNRNNLELSTPLKIETISHEDLQR.Q	4	4.83	0.46	-2.16
IPI00294193	Isoform 1 of Inter-alpha-trypsin inhibitor heavy chain H4 precursor	A.EKNGIDIYSLTVDSR.V	2	4.52	0.51	-2.76
IPI00294193	Isoform 1 of Inter-alpha-trypsin inhibitor heavy chain H4 precursor	K.AEAQAQYSAAVAK.G	1	3.20	0.31	-3.17
IPI00294193	Isoform 1 of Inter-alpha-trypsin inhibitor heavy chain H4 precursor	K.AEAQAQYSAAVAK.G	2	4.88	0.46	-5.13

IPI00294193	Isoform 1 of Inter-alpha-trypsin inhibitor heavy chain H4 precursor	K.AGFSWIEVTFK.N	2	3.83	0.37	-3.40
IPI00294193	Isoform 1 of Inter-alpha-trypsin inhibitor heavy chain H4 precursor	K.EKAEAQAQYSAAVAK.G	2	4.70	0.52	-3.59
IPI00294193	Isoform 1 of Inter-alpha-trypsin inhibitor heavy chain H4 precursor	K.EKAEAQAQYSAAVAK.G	3	2.95	0.20	-1.30
IPI00294193	Isoform 1 of Inter-alpha-trypsin inhibitor heavy chain H4 precursor	K.ETLFSVM*PGLK.M	2	3.05	0.37	-2.95
IPI00294193	Isoform 1 of Inter-alpha-trypsin inhibitor heavy chain H4 precursor	K.GSEM*VVAGK.L	1	2.36	0.16	-2.42
IPI00294193	Isoform 1 of Inter-alpha-trypsin inhibitor heavy chain H4 precursor	K.GSEM*VVAGK.L	2	3.20	0.25	-3.04
IPI00294193	Isoform 1 of Inter-alpha-trypsin inhibitor heavy chain H4 precursor	K.ILDDLSPRDQFNLIVFSTEATQWRPSLVPASAENVNK.A	4	3.61	0.24	-3.03
IPI00294193	Isoform 1 of Inter-alpha-trypsin inhibitor heavy chain H4 precursor	K.ITFELVYEELLK.R	2	4.90	0.43	-6.82
IPI00294193	Isoform 1 of Inter-alpha-trypsin inhibitor heavy chain H4 precursor	K.ITFELVYEELLKR.R	2	3.24	0.31	-3.11
IPI00294193	Isoform 1 of Inter-alpha-trypsin inhibitor heavy chain H4 precursor	K.ITFELVYEELLKR.R	3	2.51	0.18	-4.10
IPI00294193	Isoform 1 of Inter-alpha-trypsin inhibitor heavy chain H4 precursor	K.LALDNGGLAR.R	1	2.35	0.24	-3.22
IPI00294193	Isoform 1 of Inter-alpha-trypsin inhibitor heavy chain H4 precursor	K.LALDNGGLAR.R	2	3.38	0.30	-2.67
IPI00294193	Isoform 1 of Inter-alpha-trypsin inhibitor heavy chain H4 precursor	K.LQDRGPDVLTATVSGK.L	2	4.29	0.52	-2.31
IPI00294193	Isoform 1 of Inter-alpha-trypsin inhibitor heavy chain H4 precursor	K.LQDRGPDVLTATVSGK.L	3	4.37	0.31	-3.49
IPI00294193	Isoform 1 of Inter-alpha-trypsin inhibitor heavy chain H4 precursor	K.NGIDIYSLTVDSR.V	2	4.00	0.40	-2.27
IPI00294193	Isoform 1 of Inter-alpha-trypsin inhibitor heavy chain H4 precursor	K.NPLVWVHASPEHVVVTR.N	3	5.38	0.52	-5.11
IPI00294193	Isoform 1 of Inter-alpha-trypsin inhibitor heavy chain H4 precursor	K.NVVFVIDK.S	1	2.65	0.17	-3.16
IPI00294193	Isoform 1 of Inter-alpha-trypsin inhibitor heavy chain H4 precursor	K.NVVFVIDK.S	2	2.33	0.25	-2.47
IPI00294193	Isoform 1 of Inter-alpha-trypsin inhibitor heavy chain H4 precursor	K.NVVFVIDKSGSM*SGR.K	2	3.23	0.30	-0.83
IPI00294193	Isoform 1 of Inter-alpha-trypsin inhibitor heavy chain H4 precursor	K.NVVFVIDKSGSM*SGR.K	3	2.51	0.33	0.12
IPI00294193	Isoform 1 of Inter-alpha-trypsin inhibitor heavy chain H4 precursor	K.NVVFVIDKSGSM*SGRK.I	3	2.46	0.25	-2.31

IPI00294193	Isoform 1 of Inter-alpha-trypsin inhibitor heavy chain H4 precursor	K.SPEQQETVLDGNLIIR.Y	2	5.01	0.44	-6.49
IPI00294193	Isoform 1 of Inter-alpha-trypsin inhibitor heavy chain H4 precursor	K.SPEQQETVLDGNLIIR.Y	3	4.19	0.20	-4.28
IPI00294193	Isoform 1 of Inter-alpha-trypsin inhibitor heavy chain H4 precursor	K.TGLLLLSDPDKVTIGLLFWDGR.G	3	2.86	0.28	-4.01
IPI00294193	Isoform 1 of Inter-alpha-trypsin inhibitor heavy chain H4 precursor	K.TGLLLLSDPDKVTIGLLFWDGRGEGLR.L	4	3.72	0.15	-1.76
IPI00294193	Isoform 1 of Inter-alpha-trypsin inhibitor heavy chain H4 precursor	K.WKETLFSVM*PGLK.M	2	4.00	0.38	-2.92
IPI00294193	Isoform 1 of Inter-alpha-trypsin inhibitor heavy chain H4 precursor	K.WKETLFSVM*PGLK.M	3	4.12	0.26	-2.35
IPI00294193	Isoform 1 of Inter-alpha-trypsin inhibitor heavy chain H4 precursor	K.YIFHNFM*ER.L	2	2.20	0.19	-0.59
IPI00294193	Isoform 1 of Inter-alpha-trypsin inhibitor heavy chain H4 precursor	R.AISGGSIQIENGYFVHYFAPEGLTTM*PK.N	2	4.26	0.53	-2.44
IPI00294193	Isoform 1 of Inter-alpha-trypsin inhibitor heavy chain H4 precursor	R.AISGGSIQIENGYFVHYFAPEGLTTM*PK.N	3	5.62	0.55	-4.13
IPI00294193	Isoform 1 of Inter-alpha-trypsin inhibitor heavy chain H4 precursor	R.ANTVQEATFQM*ELPK.K	2	5.10	0.41	-4.19
IPI00294193	Isoform 1 of Inter-alpha-trypsin inhibitor heavy chain H4 precursor	R.ANTVQEATFQM*ELPKK.A	2	4.67	0.47	-3.41
IPI00294193	Isoform 1 of Inter-alpha-trypsin inhibitor heavy chain H4 precursor	R.ANTVQEATFQMELPK.K	2	2.99	0.34	
IPI00294193	Isoform 1 of Inter-alpha-trypsin inhibitor heavy chain H4 precursor	R.DQFNLIVFSTEATQWRPSLVPASAENVNK.A	2	2.36	0.40	-1.90
IPI00294193	Isoform 1 of Inter-alpha-trypsin inhibitor heavy chain H4 precursor	R.DQFNLIVFSTEATQWRPSLVPASAENVNK.A	3	6.22	0.54	-3.80
IPI00294193	Isoform 1 of Inter-alpha-trypsin inhibitor heavy chain H4 precursor	R.DQFNLIVFSTEATQWRPSLVPASAENVNK.A	4	6.02	0.46	-3.05
IPI00294193	Isoform 1 of Inter-alpha-trypsin inhibitor heavy chain H4 precursor	R.DTDRFSSHVGGTLGQFYQEVLWGSPAASDDGRR.T	5	3.34	0.21	-2.57
IPI00294193	Isoform 1 of Inter-alpha-trypsin inhibitor heavy chain H4 precursor	R.FAHTVVTSR.V	1	2.67	0.27	-4.67
IPI00294193	Isoform 1 of Inter-alpha-trypsin inhibitor heavy chain H4 precursor	R.FAHTVVTSR.V	2	2.70	0.32	-2.52
IPI00294193	Isoform 1 of Inter-alpha-trypsin inhibitor heavy chain H4 precursor	R.FKPTLSQQQK.S	2	2.83	0.27	-3.54
IPI00294193	Isoform 1 of Inter-alpha-trypsin inhibitor heavy chain H4 precursor	R.FKPTLSQQQK.S	3	3.30	0.14	-5.23
IPI00294193	Isoform 1 of Inter-alpha-trypsin inhibitor heavy chain H4 precursor	R.FSSHVGGTLGQFYQEVLWGSPAASDDGRR.T	3	3.72	0.39	-4.40

IPI00294193	Isoform 1 of Inter-alpha-trypsin inhibitor heavy chain H4 precursor	R.GPDVLTATVSGK.L	1	2.08	0.17	-3.32
IPI00294193	Isoform 1 of Inter-alpha-trypsin inhibitor heavy chain H4 precursor	R.GPDVLTATVSGK.L	2	3.79	0.42	-2.48
IPI00294193	Isoform 1 of Inter-alpha-trypsin inhibitor heavy chain H4 precursor	R.HRQGPVNLLSDPEQGVEVTGQYER.E	3	5.93	0.53	-1.50
IPI00294193	Isoform 1 of Inter-alpha-trypsin inhibitor heavy chain H4 precursor	R.HRQGPVNLLSDPEQGVEVTGQYER.E	4	4.28	0.39	-3.92
IPI00294193	Isoform 1 of Inter-alpha-trypsin inhibitor heavy chain H4 precursor	R.LGVYELLLK.V	2	3.56	0.37	-2.77
IPI00294193	Isoform 1 of Inter-alpha-trypsin inhibitor heavy chain H4 precursor	R.LPEGSVSLIILLTDGDPTVGETNPR.S	3	4.34	0.37	-2.85
IPI00294193	Isoform 1 of Inter-alpha-trypsin inhibitor heavy chain H4 precursor	R.LWAYLTIQQLLEQTVSASDADQQALR.N	3	5.64	0.45	-6.69
IPI00294193	Isoform 1 of Inter-alpha-trypsin inhibitor heavy chain H4 precursor	R.M*NFRPGVLSSR.L	2	1.63	0.05	-3.59
IPI00294193	Isoform 1 of Inter-alpha-trypsin inhibitor heavy chain H4 precursor	R.NM*EQFQVSVSVAPNAK.I	2	4.78	0.45	-2.79
IPI00294193	Isoform 1 of Inter-alpha-trypsin inhibitor heavy chain H4 precursor	R.QGPVNLLSDPEQGVEVTGQYER.E	2	4.85	0.50	-4.06
IPI00294193	Isoform 1 of Inter-alpha-trypsin inhibitor heavy chain H4 precursor	R.QGPVNLLSDPEQGVEVTGQYER.E	3	4.86	0.43	-4.91
IPI00294193	Isoform 1 of Inter-alpha-trypsin inhibitor heavy chain H4 precursor	R.QGPVNLLSDPEQGVEVTGQYEREK.A	3	3.33	0.24	-2.48
IPI00294193	Isoform 1 of Inter-alpha-trypsin inhibitor heavy chain H4 precursor	R.RLDYQEGPPGVEISCWSVEL.-	2	5.37	0.61	-4.23
IPI00294193	Isoform 1 of Inter-alpha-trypsin inhibitor heavy chain H4 precursor	R.RLDYQEGPPGVEISCWSVEL.-	3	4.45	0.40	-3.75
IPI00294193	Isoform 1 of Inter-alpha-trypsin inhibitor heavy chain H4 precursor	R.RLGVYELLLK.V	2	2.32	0.09	-1.65
IPI00294193	Isoform 1 of Inter-alpha-trypsin inhibitor heavy chain H4 precursor	R.RLGVYELLLK.V	3	3.85	0.23	-2.19
IPI00294193	Isoform 1 of Inter-alpha-trypsin inhibitor heavy chain H4 precursor	R.SFAAGIQALGGTNINDAM*LM*AVQLLDSSNQEER.L	3	3.68	0.28	-7.00
IPI00294193	Isoform 1 of Inter-alpha-trypsin inhibitor heavy chain H4 precursor	R.SFAAGIQALGGTNINDAM*LM*AVQLLDSSNQEER.L	4	5.25	0.47	-4.79
IPI00294193	Isoform 1 of Inter-alpha-trypsin inhibitor heavy chain H4 precursor	R.SIQNNVR.E	1	1.62	0.12	-4.71
IPI00294193	Isoform 1 of Inter-alpha-trypsin inhibitor heavy chain H4 precursor	W.VHASPEHVVVTR.N	2	3.07	0.42	-1.67
IPI00294210	DNA-binding protein inhibitor ID-2	K.LKELVPSIPQNK.K	3	3.39	0.06	
IPI00294215	Uncharacterized protein KIAA0232	E.SSGIETLVEELCSRLK.D	2	2.93	0.21	-9.00

IPI00294395	Complement component C8 beta chain precursor	K.CQHEM*DQYWGIGSLASGINLFTNSFEGPVLDHR.Y	4	3.76	0.21	-2.07
IPI00294395	Complement component C8 beta chain precursor	K.EYESYSDFERNVTEKM*ASKSGFSFGFK.I	4	2.22	0.20	0.55
IPI00294395	Complement component C8 beta chain precursor	K.IPGIFELGISSQSDR.G	2	4.72	0.57	-4.43
IPI00294395	Complement component C8 beta chain precursor	K.IPGIFELGISSQSDR.G	3	3.30	0.23	-2.90
IPI00294395	Complement component C8 beta chain precursor	K.QALEEFQK.E	1	1.51	0.06	-3.73
IPI00294395	Complement component C8 beta chain precursor	K.RLPLEYSYGEYR.D	2	2.68	0.30	-3.66
IPI00294395	Complement component C8 beta chain precursor	K.RLPLEYSYGEYRDLFR.D	2	3.30	0.36	-2.91
IPI00294395	Complement component C8 beta chain precursor	K.RLPLEYSYGEYRDLFR.D	3	2.27	0.13	-2.76
IPI00294395	Complement component C8 beta chain precursor	K.SGFSFGFK.I	2	2.25	0.22	-2.52
IPI00294395	Complement component C8 beta chain precursor	K.VEPLYELVTATDFAYSSTVR.Q	2	5.33	0.55	-6.71
IPI00294395	Complement component C8 beta chain precursor	K.VEPLYELVTATDFAYSSTVR.Q	3	3.79	0.35	-5.51
IPI00294395	Complement component C8 beta chain precursor	K.VKVEPLYELVTATDFAYSSTVR.Q	2	5.47	0.53	-2.52
IPI00294395	Complement component C8 beta chain precursor	K.VKVEPLYELVTATDFAYSSTVR.Q	3	4.51	0.48	-6.32
IPI00294395	Complement component C8 beta chain precursor	R.CEGFVCAQTGR.C	2	4.33	0.45	-1.48
IPI00294395	Complement component C8 beta chain precursor	R.DFGTHYITEAVLGGIYEYTLVM*NK.E	3	3.67	0.31	-4.54
IPI00294395	Complement component C8 beta chain precursor	R.DTM*VEDLVVLVR.G	2	3.81	0.31	-3.48
IPI00294395	Complement component C8 beta chain precursor	R.DTM*VEDLVVLVR.G	3	3.63	0.13	-3.11
IPI00294395	Complement component C8 beta chain precursor	R.FRKPYNVESYTPQTQGK.Y	2	3.99	0.37	-3.29
IPI00294395	Complement component C8 beta chain precursor	R.FRKPYNVESYTPQTQGK.Y	3	5.17	0.42	-2.03
IPI00294395	Complement component C8 beta chain precursor	R.GDYTLNNVHACAK.N	2	3.57	0.40	2.11
IPI00294395	Complement component C8 beta chain precursor	R.GGASEHITTLAYQELPTADLM*QEWGDAVQYNPAIIK.V	3	6.60	0.55	-4.23
IPI00294395	Complement component C8 beta chain precursor	R.GGASEHITTLAYQELPTADLM*QEWGDAVQYNPAIIK.V	4	4.44	0.24	-5.03
IPI00294395	Complement component C8 beta chain precursor	R.GILNEIKDR.N	2	2.64	0.10	-3.99
IPI00294395	Complement component C8 beta chain precursor	R.KPYNVESYTPQTQGK.Y	2	4.21	0.48	-6.51
IPI00294395	Complement component C8 beta chain precursor	R.KPYNVESYTPQTQGK.Y	3	2.87	0.15	-2.89
IPI00294395	Complement component C8 beta chain precursor	R.KPYNVESYTPQTQGKYEFILK.E	3	4.34	0.42	-2.84
IPI00294395	Complement component C8 beta chain precursor	R.LLCNGDNDCGDQSDEANCRR.I	3	3.84	0.27	
IPI00294395	Complement component C8 beta chain precursor	R.LPLEYSYGEYR.D	2	3.43	0.45	-3.13
IPI00294395	Complement component C8 beta chain precursor	R.LPLEYSYGEYRDLFR.D	2	2.74	0.22	-2.32
IPI00294395	Complement component C8 beta chain precursor	R.LPLEYSYGEYRDLFR.D	3	2.17	0.20	-1.19
IPI00294395	Complement component C8 beta chain precursor	R.SDLEVAHYK.L	1	2.17	0.31	-3.17
IPI00294395	Complement component C8 beta chain precursor	R.SDLEVAHYK.L	2	2.88	0.30	-2.22
IPI00294395	Complement component C8 beta chain precursor	R.SLM*LHYEFLQR.V	2	3.40	0.34	-4.39
IPI00294395	Complement component C8 beta chain precursor	R.SLM*LHYEFLQR.V	3	3.21	0.28	-1.69
IPI00294519	Isoform 1 of Telomerase protein component 1	K.KANTPETQTPGTDPSTCRESDASM*DSDASM*DSEPTPHLK.T	3	2.32	0.06	-1.88
IPI00294615	Fibulin-5 precursor	R.CM*CPAENPGCR.D	2	2.82	0.31	
IPI00294615	Fibulin-5 precursor	R.DM*DVVSGR.S	2	2.54	0.14	-3.37
IPI00294615	Fibulin-5 precursor	R.SVPADIFQM*QATTR.Y	2	2.36	0.14	
IPI00294615	Fibulin-5 precursor	R.TIPEACRGDM*M*CVNQNGGYLCIPR.T	3	4.45	0.23	
IPI00294615	Fibulin-5 precursor	R.YPGAYYIFQIK.S	2	3.30	0.16	
IPI00294619	Protein TFG	G.LTDDQVSGPPSAPAEDR.S	2	4.75	0.53	-3.50
IPI00294619	Protein TFG	L.TDDQVSGPPSAPAEDR.S	2	3.92	0.48	-3.25

IPI00294705	Papilin	K.GENFYYK.H	2	1.85	0.06	-1.31
IPI00294705	Papilin	R.GAEGDLAPER.L	2	2.36	0.19	-2.20
IPI00294705	Papilin	R.GPTSEPLVIELISQEPNPGVHYEYHLPLR.R	4	4.64	0.28	-4.52
IPI00294705	Papilin	R.GYNQILIVPM*GATSILIDEAAASR.N	2	3.05	0.25	-3.38
IPI00294705	Papilin	R.GYNQILIVPM*GATSILIDEAAASR.N	3	4.02	0.35	-5.72
IPI00294705	Papilin	R.ISLAGVEPSLVQAALGQLVR.L	2	5.48	0.51	-4.19
IPI00294705	Papilin	R.ISLAGVEPSLVQAALGQLVR.L	3	4.19	0.30	-3.62
IPI00294705	Papilin	R.LRLDQNQPR.V	2	3.16	0.12	-2.11
IPI00294705	Papilin	R.VHQSPDGTLLIYNLR.A	3	3.33	0.16	-3.04
IPI00294705	Papilin	R.VVDASPGQR.I	2	2.35	0.15	-2.63
IPI00294713	Isoform 1 of Mannan-binding lectin serine protease 2 precursor	K.WPEPVFGR.L	2	1.71	0.10	-0.69
IPI00294713	Isoform 1 of Mannan-binding lectin serine protease 2 precursor	R.AGYVLHR.N	2	2.48	0.25	-3.40
IPI00294834	Aspartyl/asparaginyl beta-hydroxylase	K.LGIYDADGDGDFDVDDAK.V	2	6.23	0.56	-5.18
IPI00294834	Aspartyl/asparaginyl beta-hydroxylase	K.LGIYDADGDGDFDVDDAK.V	3	4.80	0.31	-2.52
IPI00294834	Aspartyl/asparaginyl beta-hydroxylase	K.LGIYDADGDGDFDVDDAKVLLGLK.E	2	5.19	0.61	-2.10
IPI00294834	Aspartyl/asparaginyl beta-hydroxylase	K.LGIYDADGDGDFDVDDAKVLLGLK.E	3	4.70	0.47	-2.14
IPI00294834	Aspartyl/asparaginyl beta-hydroxylase	K.VHYGFILK.A	2	1.92	0.22	-2.68
IPI00294834	Aspartyl/asparaginyl beta-hydroxylase	R.GAIETYQEVASLPDVPADLLK.L	2	4.46	0.33	-5.30
IPI00294834	Aspartyl/asparaginyl beta-hydroxylase	R.SLYNVNGLK.A	2	2.50	0.14	-1.18
IPI00294910	Protein PARM-1 precursor	R.VIM*QEVEHALSSGSIAAI.T	2	3.33	0.30	-4.87
IPI00295098	Signal recognition particle receptor subunit beta	K.NTPSFLIACNKQDIAM*AK.S	3	2.31	0.07	-5.86
IPI00295172	Ninjurin-1	K.YDLNNPDK.H	2	2.40	0.14	
IPI00295386	Carbonyl reductase [NADPH] 1	K.ILLNACCPGWVR.T	2	3.70	0.40	-2.63
IPI00295386	Carbonyl reductase [NADPH] 1	K.TNFFGTR.D	2	1.82	0.07	-2.34
IPI00295386	Carbonyl reductase [NADPH] 1	R.FHQLDIDDLQSIR.A	3	4.09	0.31	-1.34
IPI00295386	Carbonyl reductase [NADPH] 1	R.GQAAVQQLQAEGLSPR.F	2	4.48	0.47	-1.89
IPI00295386	Carbonyl reductase [NADPH] 1	R.VVNVSSIM*SVR.A	2	4.24	0.40	-1.52
IPI00295399	Cadherin-10 precursor	K.ATDADTGKNAEVEYR.I	3	3.78	0.45	-0.77
IPI00295399	Cadherin-10 precursor	K.LHSDQDKGDGSLK.Y	2	2.89	0.32	-2.72
IPI00295399	Cadherin-10 precursor	R.DPDSISSPIR.F	2	3.30	0.31	-2.31
IPI00295399	Cadherin-10 precursor	R.TLRPVEPESEFVIK.I	3	3.00	0.18	-2.31
IPI00295399	Cadherin-10 precursor	R.TPVPQQR.I	2	2.14	0.05	-3.06
IPI00295399	Cadherin-10 precursor	R.VIYSILQGQPYFSVEPETGIIR.T	3	5.38	0.24	-2.54
IPI00295414	Collagen alpha-1(XV) chain precursor	R.AAGLLSTYR.A	1	1.44	0.15	-3.14
IPI00295414	Collagen alpha-1(XV) chain precursor	R.AAGLLSTYR.A	2	2.73	0.20	-1.01
IPI00295414	Collagen alpha-1(XV) chain precursor	R.AFLSSHLQDLSTIVR.K	2	4.83	0.50	-3.34
IPI00295414	Collagen alpha-1(XV) chain precursor	R.AFLSSHLQDLSTIVR.K	3	3.43	0.27	-2.53
IPI00295414	Collagen alpha-1(XV) chain precursor	R.GGVLFAITDAFQK.V	2	3.21	0.32	-3.84
IPI00295414	Collagen alpha-1(XV) chain precursor	R.LVDNYCEAWR.T	2	2.09	0.12	-0.60
IPI00295414	Collagen alpha-1(XV) chain precursor	R.TADTAVTGLASPLSTGK.I	2	4.37	0.50	-2.52

IPI00295414	Collagen alpha-1(XV) chain precursor	R.TADTAVTGLASPLSTGK.I	3	3.17	0.09	-1.28
IPI00295414	Collagen alpha-1(XV) chain precursor	R.YSLPIVNLK.G	1	2.13	0.24	-3.16
IPI00295414	Collagen alpha-1(XV) chain precursor	R.YSLPIVNLK.G	2	1.91	0.22	-0.26
IPI00295469	Copine-6	K.YSVLLVLTDGVVSDMAETR.T	2	2.53	0.16	-2.35
IPI00295502	Isoform 1 of Protein Wiz	K.ALAKMM*GGAGPGSSLEAR.S	2	2.48	0.10	
IPI00295503	Isoform 2 of Probable ATP-dependent RNA helicase DDX58	K.RAKIFCARQNCSHDWGIHVK.Y	3	2.31	0.07	-3.50
IPI00295542	Nucleobindin-1 precursor	K.AKM*DAEQDPNVQVDHLNLLK.Q	3	2.91	0.22	-3.05
IPI00295542	Nucleobindin-1 precursor	K.APAAHPEGQLK.F	1	2.46	0.30	-3.83
IPI00295542	Nucleobindin-1 precursor	K.APAAHPEGQLK.F	2	3.30	0.43	-2.76
IPI00295542	Nucleobindin-1 precursor	K.EVWEELDGLDPNRFNPK.T	3	2.22	0.14	-2.77
IPI00295542	Nucleobindin-1 precursor	K.FHPDTDDVPVPAPAGDQK.E	2	5.11	0.58	-2.80
IPI00295542	Nucleobindin-1 precursor	K.FHPDTDDVPVPAPAGDQK.E	3	3.43	0.24	-1.70
IPI00295542	Nucleobindin-1 precursor	K.FHPDTDDVPVPAPAGDQKE.V	2	4.94	0.56	-2.98
IPI00295542	Nucleobindin-1 precursor	K.FHPDTDDVPVPAPAGDQKEVDTSEK.K	3	4.92	0.56	-1.56
IPI00295542	Nucleobindin-1 precursor	K.FHPDTDDVPVPAPAGDQKEVDTSEKK.L	3	4.40	0.42	-4.68
IPI00295542	Nucleobindin-1 precursor	K.FHPDTDDVPVPAPAGDQKEVDTSEKK.L	4	2.39	0.13	-3.51
IPI00295542	Nucleobindin-1 precursor	K.LLERLPEVEVPQHL.-	2	3.00	0.34	-2.06
IPI00295542	Nucleobindin-1 precursor	K.LLERLPEVEVPQHL.-	3	3.41	0.19	-2.12
IPI00295542	Nucleobindin-1 precursor	K.LQAANAEDIKSGK.L	2	4.23	0.39	-2.50
IPI00295542	Nucleobindin-1 precursor	K.LQAANAEDIKSGK.L	3	2.16	0.25	-2.69
IPI00295542	Nucleobindin-1 precursor	K.M*DAEQDPNVQVDHLNLLK.Q	3	4.04	0.32	-1.32
IPI00295542	Nucleobindin-1 precursor	K.NVDTNQDRLVTLEEFLASTQR.K	3	2.79	0.23	-1.52
IPI00295542	Nucleobindin-1 precursor	K.QFEHLDPQNQHTFEAR.D	3	2.20	0.20	-3.19
IPI00295542	Nucleobindin-1 precursor	K.TFFILHDINSDGVLDEQELEALFTK.E	3	5.85	0.50	-4.25
IPI00295542	Nucleobindin-1 precursor	K.TFFILHDINSDGVLDEQELEALFTKELEK.V	3	4.07	0.38	-3.54
IPI00295542	Nucleobindin-1 precursor	K.TFFILHDINSDGVLDEQELEALFTKELEK.V	4	3.45	0.09	-1.45
IPI00295542	Nucleobindin-1 precursor	K.VNVPGSQAQLK.E	2	2.73	0.33	-1.84
IPI00295542	Nucleobindin-1 precursor	K.VYDPKNEEDDM*REM*EEERLR.M	4	2.38	0.21	-2.66
IPI00295542	Nucleobindin-1 precursor	R.DLAQYDAAHHEEFKR.Y	3	2.64	0.40	-2.81
IPI00295542	Nucleobindin-1 precursor	R.DLELLIQTATR.D	2	3.86	0.36	-3.85
IPI00295542	Nucleobindin-1 precursor	R.EKLQAANAEDIKSGK.L	2	4.16	0.43	-1.97
IPI00295542	Nucleobindin-1 precursor	R.EKLQAANAEDIKSGK.L	3	2.26	0.20	
IPI00295542	Nucleobindin-1 precursor	R.ELDFVSHHVR.T	2	2.96	0.33	-3.39
IPI00295542	Nucleobindin-1 precursor	R.ELQQAVLHM*EQR.K	2	2.99	0.21	
IPI00295542	Nucleobindin-1 precursor	R.ELQQAVLHM*EQR.K	3	1.89	0.13	-1.72
IPI00295542	Nucleobindin-1 precursor	R.LPEVEVPQHL.-	2	2.94	0.26	-2.44
IPI00295542	Nucleobindin-1 precursor	R.LSQETEALGR.S	1	1.89	0.29	-3.13
IPI00295542	Nucleobindin-1 precursor	R.LSQETEALGR.S	2	4.09	0.32	-2.49
IPI00295542	Nucleobindin-1 precursor	R.LVTLEEFLASTQR.K	2	3.71	0.32	-4.76
IPI00295542	Nucleobindin-1 precursor	R.LVTLEEFLASTQR.K	3	3.61	0.34	-2.86
IPI00295542	Nucleobindin-1 precursor	R.RYLESLGEEQRK.E	3	4.16	0.16	

IPI00295542	Nucleobindin-1 precursor	R.TKLDELKR.Q	2	1.96	0.06	-3.04
IPI00295542	Nucleobindin-1 precursor	R.YLESLGEEQR.K	2	2.88	0.18	-3.36
IPI00295542	Nucleobindin-1 precursor	R.YLESLGEEQRK.E	2	2.72	0.21	-4.53
IPI00295542	Nucleobindin-1 precursor	R.YLQEVIDVLETDGHFR.E	2	4.78	0.38	-3.56
IPI00295542	Nucleobindin-1 precursor	R.YLQEVIDVLETDGHFR.E	3	3.70	0.35	-4.50
IPI00295577	Receptor-type tyrosine-protein phosphatase beta precursor	R.DPVYSR.H	1	1.17	0.09	-2.62
IPI00295618	Isoform Long of Platelet endothelial cell adhesion molecule precursor	R.DQNFVILEFPVEEQDR.V	2	4.50	0.46	-3.23
IPI00295741	Cathepsin B precursor	A.FGAVEAISDR.I	2	3.84	0.36	-1.22
IPI00295741	Cathepsin B precursor	K.DIM*AEIYK.N	2	3.18	0.18	-2.23
IPI00295741	Cathepsin B precursor	K.EIRDQGSCGSCWAFGAVEAISDR.I	3	4.53	0.34	-1.75
IPI00295741	Cathepsin B precursor	K.HYGYNSYSVSNSEKDIM*AEIYK.N	3	3.80	0.55	-1.92
IPI00295741	Cathepsin B precursor	K.HYGYNSYSVSNSEKDIM*AEIYK.N	4	3.59	0.33	-0.92
IPI00295741	Cathepsin B precursor	K.ICEPGYSPTYK.Q	2	2.97	0.28	-2.63
IPI00295741	Cathepsin B precursor	K.LPASFDAR.E	2	2.27	0.14	-3.07
IPI00295741	Cathepsin B precursor	K.NGPVEGAFSVYSDFLLYK.S	2	5.80	0.59	-8.14
IPI00295741	Cathepsin B precursor	K.NGPVEGAFSVYSDFLLYK.S	3	4.59	0.45	-2.88
IPI00295741	Cathepsin B precursor	K.QDKHYGYNSYSVSNSEK.D	3	2.33	0.31	-3.49
IPI00295741	Cathepsin B precursor	K.SGVYQHVTGEM*M*GGHAIR.I	3	3.42	0.37	
IPI00295741	Cathepsin B precursor	R.DQGSCGSCWAFGAVEAISDR.I	2	5.56	0.58	-4.20
IPI00295741	Cathepsin B precursor	R.DQGSCGSCWAFGAVEAISDR.I	3	5.10	0.48	-4.77
IPI00295741	Cathepsin B precursor	R.EQWPQCPTIK.E	2	2.58	0.21	-3.15
IPI00295741	Cathepsin B precursor	R.PSFHPLSDELVNYVNKR.N	3	4.66	0.42	-1.84
IPI00295741	Cathepsin B precursor	R.SRPSFHPLSDELVNYVNK.R	3	4.37	0.35	-3.97
IPI00295741	Cathepsin B precursor	R.TDQYWEKI.-	2	2.68	0.29	-2.91
IPI00295741	Cathepsin B precursor	R.VM*FTEDLKLPASFDAR.E	2	2.74	0.37	-2.61
IPI00295741	Cathepsin B precursor	R.VM*FTEDLKLPASFDAR.E	3	4.05	0.31	-2.61
IPI00295741	Cathepsin B precursor	W.AFGAVEAISDR.I	2	3.90	0.38	-2.86
IPI00295767	Noelin-2 precursor	K.LTGVSNPITVR.A	2	3.41	0.31	-2.10
IPI00295767	Noelin-2 precursor	R.LDPHTLEVM*R.S	2	2.03	0.25	-0.96
IPI00295832	Oligodendrocyte-myelin glycoprotein precursor	C.ICPLQCICTER.H	2	3.75	0.31	-2.71
IPI00295832	Oligodendrocyte-myelin glycoprotein precursor	I.SNNRLESLPAHLPR.S	2	3.43	0.22	-3.52
IPI00295832	Oligodendrocyte-myelin glycoprotein precursor	K.DTTFTSTDK.A	2	1.92	0.14	-2.54
IPI00295832	Oligodendrocyte-myelin glycoprotein precursor	K.ETTFGATLSK.D	2	2.24	0.27	-4.00
IPI00295832	Oligodendrocyte-myelin glycoprotein precursor	K.FTFIPDQSFDQLFQLQEIT.L	2	3.61	0.45	-4.33
IPI00295832	Oligodendrocyte-myelin glycoprotein precursor	K.FTFIPDQSFDQLFQLQEITLYNNR.W	2	4.70	0.48	-4.92
IPI00295832	Oligodendrocyte-myelin glycoprotein precursor	K.FTFIPDQSFDQLFQLQEITLYNNR.W	3	6.36	0.42	-5.30
IPI00295832	Oligodendrocyte-myelin glycoprotein precursor	K.FTFIPDQSFDQLFQLQEITLYNNR.W	4	2.76	0.19	-3.19
IPI00295832	Oligodendrocyte-myelin glycoprotein precursor	K.SDTAYQWNLK.Y	2	2.66	0.24	-1.92
IPI00295832	Oligodendrocyte-myelin glycoprotein precursor	K.YLDVSK.N	1	2.10	0.06	-3.44
IPI00295832	Oligodendrocyte-myelin glycoprotein precursor	R.LESLPAHLPR.S	2	3.35	0.27	-2.91

IPI00295832	Oligodendrocyte-myelin glycoprotein precursor	R.SLEVLNLSSNK.L	1	2.99	0.31	-2.53
IPI00295832	Oligodendrocyte-myelin glycoprotein precursor	R.SLEVLNLSSNK.L	2	4.23	0.44	-2.75
IPI00295832	Oligodendrocyte-myelin glycoprotein precursor	R.SLEVLNLSSNKLWTVPTNM*PSK.L	3	2.64	0.07	-2.92
IPI00295832	Oligodendrocyte-myelin glycoprotein precursor	R.TKETTFGATLSK.D	2	3.86	0.44	-2.58
IPI00295832	Oligodendrocyte-myelin glycoprotein precursor	R.TKETTFGATLSK.D	3	3.54	0.24	-3.81
IPI00295832	Oligodendrocyte-myelin glycoprotein precursor	R.TLDISNNR.L	1	2.02	0.12	-2.93
IPI00295832	Oligodendrocyte-myelin glycoprotein precursor	R.TLDISNNR.L	2	2.36	0.07	-1.42
IPI00295832	Oligodendrocyte-myelin glycoprotein precursor	R.TLDISNNRLESLPAHLPR.S	2	3.09	0.28	-5.10
IPI00295832	Oligodendrocyte-myelin glycoprotein precursor	R.TLDISNNRLESLPAHLPR.S	3	5.00	0.44	-4.01
IPI00295832	Oligodendrocyte-myelin glycoprotein precursor	R.TLDISNNRLESLPAHLPR.S	4	1.79	0.21	-1.73
IPI00296058	EGF-containing fibulin-like extracellular matrix protein 2 precursor	K.IGPECVDIDECR.Y	2	3.62	0.47	
IPI00296058	EGF-containing fibulin-like extracellular matrix protein 2 precursor	R.DGFSCSDIDECSYSSYLCQYR.C	2	4.06	0.27	
IPI00296058	EGF-containing fibulin-like extracellular matrix protein 2 precursor	R.SCVDVNECDM*GAPCEQR.C	2	5.39	0.40	
IPI00296058	EGF-containing fibulin-like extracellular matrix protein 2 precursor	R.SCVDVNECDM*GAPCEQR.C	3	3.23	0.24	
IPI00296058	EGF-containing fibulin-like extracellular matrix protein 2 precursor	R.SVPADVFQIQATSVYPGAYNAFQIR.A	2	4.07	0.35	
IPI00296099	Thrombospondin-1 precursor	K.GGVNDNFQGVLQNVR.F	2	4.44	0.41	-3.30
IPI00296099	Thrombospondin-1 precursor	R.AQGYSGLSVK.V	2	1.72	0.19	0.61
IPI00296099	Thrombospondin-1 precursor	R.DNCQYVYNVDQR.D	2	3.53	0.32	-3.93
IPI00296099	Thrombospondin-1 precursor	R.FVFGTTPEDILR.N	2	2.67	0.33	-3.61
IPI00296099	Thrombospondin-1 precursor	R.GTLLALER.K	2	2.88	0.17	-3.97
IPI00296099	Thrombospondin-1 precursor	R.IEDANLIPPVPDDKFQDLVDAVR.A	3	2.55	0.19	-0.95
IPI00296099	Thrombospondin-1 precursor	R.TIVTTLQDSIR.K	2	3.55	0.42	-2.21
IPI00296141	Dipeptidyl-peptidase 2 precursor	K.DLFLQGAYDTVR.W	2	4.07	0.33	-3.65
IPI00296141	Dipeptidyl-peptidase 2 precursor	K.DLTQLFM*FAR.N	2	2.94	0.32	-4.15
IPI00296141	Dipeptidyl-peptidase 2 precursor	K.SLPFGAQSTQR.G	2	3.24	0.24	-1.59
IPI00296141	Dipeptidyl-peptidase 2 precursor	R.AASNIIFSNGNLDPWAGGGIR.R	2	6.08	0.45	-4.08
IPI00296141	Dipeptidyl-peptidase 2 precursor	R.AASNIIFSNGNLDPWAGGGIR.R	3	5.29	0.29	-3.60
IPI00296141	Dipeptidyl-peptidase 2 precursor	R.APDPGFQER.F	2	2.63	0.25	-2.23
IPI00296141	Dipeptidyl-peptidase 2 precursor	R.ASHPEDPASVVEAR.K	2	2.83	0.34	-2.32
IPI00296141	Dipeptidyl-peptidase 2 precursor	R.DVTADFEGQSPK.C	1	2.28	0.17	-1.68
IPI00296141	Dipeptidyl-peptidase 2 precursor	R.DVTADFEGQSPK.C	2	4.10	0.40	-2.90
IPI00296141	Dipeptidyl-peptidase 2 precursor	R.GALLVFAEHR.Y	2	3.41	0.32	-3.09
IPI00296141	Dipeptidyl-peptidase 2 precursor	R.GHTELLTVEQALADFAELLR.A	2	5.77	0.59	-4.22
IPI00296141	Dipeptidyl-peptidase 2 precursor	R.GHTELLTVEQALADFAELLR.A	3	3.87	0.39	-6.03
IPI00296141	Dipeptidyl-peptidase 2 precursor	R.GHTELLTVEQALADFAELLR.A	4	5.07	0.24	-3.38
IPI00296141	Dipeptidyl-peptidase 2 precursor	R.KLEATIIGEWVK.A	2	3.82	0.27	-3.95
IPI00296141	Dipeptidyl-peptidase 2 precursor	R.LDHFNFER.F	1	1.76	0.22	-3.98

IPI00296141	Dipeptidyl-peptidase 2 precursor	R.LDHFNFER.F	2	2.15	0.07	-2.45
IPI00296141	Dipeptidyl-peptidase 2 precursor	R.NAFTVLAM*M*DYPYPTDFLGPLPANPVK.V	2	4.31	0.46	-4.11
IPI00296141	Dipeptidyl-peptidase 2 precursor	R.NAFTVLAM*M*DYPYPTDFLGPLPANPVK.V	3	5.86	0.58	-4.55
IPI00296141	Dipeptidyl-peptidase 2 precursor	R.NAFTVLAM*M*DYPYPTDFLGPLPANPVK.V	4	4.82	0.42	-3.82
IPI00296141	Dipeptidyl-peptidase 2 precursor	R.QIKDLFLQGAYDTVR.W	2	3.96	0.42	-2.77
IPI00296141	Dipeptidyl-peptidase 2 precursor	R.WEFGTCQPLSDEKDLTQLFM*FAR.N	2	4.38	0.49	-3.22
IPI00296141	Dipeptidyl-peptidase 2 precursor	R.WEFGTCQPLSDEKDLTQLFM*FAR.N	3	4.79	0.51	-3.59
IPI00296165	Complement C1r subcomponent precursor	K.EFM*SQGNK.M	1	1.82	0.22	-4.86
IPI00296165	Complement C1r subcomponent precursor	K.FLEPFDIDDHQQVHCPYDQLQIYANGK.N	3	5.43	0.36	
IPI00296165	Complement C1r subcomponent precursor	K.GFLAYYQAVDLDECASR.S	2	6.23	0.50	
IPI00296165	Complement C1r subcomponent precursor	K.LFGEVTSPLFPKPYPNNFETTTVITVPTGYR.V	3	3.97	0.41	-3.97
IPI00296165	Complement C1r subcomponent precursor	K.LVFQQFDLEPSEGCFYDYVK.I	2	2.69	0.31	-6.30
IPI00296165	Complement C1r subcomponent precursor	K.M*GNFPWQVFTNIHGR.G	2	2.58	0.18	
IPI00296165	Complement C1r subcomponent precursor	K.M*GNFPWQVFTNIHGR.G	3	4.01	0.31	
IPI00296165	Complement C1r subcomponent precursor	K.NRM*DVFSQNM*FCAGHPSLK.Q	3	3.66	0.35	
IPI00296165	Complement C1r subcomponent precursor	K.QDACQGDSGGVFAVR.D	2	4.44	0.46	-4.75
IPI00296165	Complement C1r subcomponent precursor	K.QDACQGDSGGVFAVRDPNTDR.W	3	2.85	0.20	-2.22
IPI00296165	Complement C1r subcomponent precursor	K.QRPPDLDTSSNAVDLLFFTDESGDSR.G	2	2.49	0.46	-2.90
IPI00296165	Complement C1r subcomponent precursor	K.QRPPDLDTSSNAVDLLFFTDESGDSR.G	3	4.75	0.45	-4.92
IPI00296165	Complement C1r subcomponent precursor	K.TLDEFTIIQNLQPQYQFR.D	2	5.00	0.50	-5.07
IPI00296165	Complement C1r subcomponent precursor	K.TLDEFTIIQNLQPQYQFR.D	3	4.95	0.42	-4.87
IPI00296165	Complement C1r subcomponent precursor	K.VLNYVDWIK.K	2	3.29	0.35	-1.82
IPI00296165	Complement C1r subcomponent precursor	K.VLNYVDWIKK.E	2	3.33	0.25	-3.18
IPI00296165	Complement C1r subcomponent precursor	K.VLNYVDWIKK.E	3	2.23	0.21	
IPI00296165	Complement C1r subcomponent precursor	R.CLPVCGKPVNPVEQR.Q	2	4.03	0.42	
IPI00296165	Complement C1r subcomponent precursor	R.DYFIATCK.Q	2	2.96	0.35	-1.52
IPI00296165	Complement C1r subcomponent precursor	R.ESEQGVYTCTAQGIWK.N	2	4.48	0.41	-2.75
IPI00296165	Complement C1r subcomponent precursor	R.ESEQGVYTCTAQGIWK.N	3	3.31	0.31	
IPI00296165	Complement C1r subcomponent precursor	R.FVRLPVANPQACENWLR.G	3	5.29	0.30	
IPI00296165	Complement C1r subcomponent precursor	R.GGGALLGDR.W	1	2.35	0.20	-2.80
IPI00296165	Complement C1r subcomponent precursor	R.GGGALLGDR.W	2	3.11	0.23	-4.75
IPI00296165	Complement C1r subcomponent precursor	R.GGGALLGDRWILTAAHTLYPK.E	4	3.36	0.30	-1.08
IPI00296165	Complement C1r subcomponent precursor	R.GYGFYTK.V	1	1.62	0.26	-0.61
IPI00296165	Complement C1r subcomponent precursor	R.IQYYCHEPYYK.M	2	2.66	0.30	
IPI00296165	Complement C1r subcomponent precursor	R.LPVANPQACENWLR.G	2	4.80	0.53	-3.70
IPI00296165	Complement C1r subcomponent precursor	R.M*DVFSQNM*FCAGHPSLK.Q	3	2.67	0.24	
IPI00296165	Complement C1r subcomponent precursor	R.VKLVFQQFDLEPSEGCFYDYVK.I	2	4.67	0.24	
IPI00296165	Complement C1r subcomponent precursor	R.VKLVFQQFDLEPSEGCFYDYVK.I	3	3.81	0.28	-2.37
IPI00296165	Complement C1r subcomponent precursor	R.VSVHPDYR.Q	1	2.00	0.17	-2.54
IPI00296165	Complement C1r subcomponent precursor	R.VSVHPDYR.Q	2	2.46	0.18	-1.87
IPI00296165	Complement C1r subcomponent precursor	R.YTTEIIK.C	2	2.53	0.22	-2.60
IPI00296165	Complement C1r subcomponent precursor	R.YTTTM*GVNTYK.A	2	3.28	0.29	

IPI00296176	Coagulation factor IX precursor	K.FTIYNNM*FCAGFHEGGR.D	3	2.67	0.26	-2.57
IPI00296176	Coagulation factor IX precursor	K.ITVVAGEHNIEETEHTEQKR.N	3	3.82	0.41	-2.59
IPI00296176	Coagulation factor IX precursor	K.NCELDVTCNIK.N	2	3.41	0.28	-3.68
IPI00296176	Coagulation factor IX precursor	K.VDAFCGGSIVNEK.W	2	4.19	0.39	-5.22
IPI00296176	Coagulation factor IX precursor	K.WIVTAAHCVETGVK.I	2	3.45	0.31	-2.97
IPI00296176	Coagulation factor IX precursor	K.YGIYTK.V	1	1.81	0.17	-1.55
IPI00296176	Coagulation factor IX precursor	K.YNHDIALLELDEPLVLNSYVTPICIADKEYTNIFLK.F	4	3.65	0.22	-3.56
IPI00296176	Coagulation factor IX precursor	R.SALVLQYLR.V	2	3.25	0.23	-2.49
IPI00296176	Coagulation factor IX precursor	R.SALVLQYLRVPLVDR.A	3	3.19	0.32	-1.84
IPI00296176	Coagulation factor IX precursor	R.VVGGEDAKPGQFPWQVVLNGK.V	3	4.03	0.41	-3.99
IPI00296197	Nucleotide exchange factor SIL1 precursor	K.LGGLQVLR.T	2	3.03	0.08	-2.14
IPI00296197	Nucleotide exchange factor SIL1 precursor	K.LLVILATEQPLTAK.K	2	3.14	0.39	-3.78
IPI00296197	Nucleotide exchange factor SIL1 precursor	K.VQVEAIEGGALQK.L	2	4.41	0.41	-2.65
IPI00296197	Nucleotide exchange factor SIL1 precursor	L.DINTNTYTSQDLK.S	2	3.67	0.39	-3.70
IPI00296197	Nucleotide exchange factor SIL1 precursor	R.VVTLLYDLVTEK.M	2	2.54	0.43	-2.75
IPI00296219	Glutaminase liver isoform, mitochondrial precursor	K.ENLESMV.-	1	1.90	0.07	-3.67
IPI00296259	Transmembrane emp24 domain-containing protein 4 precursor	G.LYFHIGETEKR.C	2	3.02	0.28	-4.30
IPI00296337	Isoform 1 of DNA-dependent protein kinase catalytic subunit	R.QFINLMLPM*K.E	2	2.93	0.07	
IPI00296374	Zinc finger protein-like 1	R.AAADSDPNLDPLM*NPHIR.V	2	3.33	0.41	-1.94
IPI00296374	Zinc finger protein-like 1	R.AAADSDPNLDPLM*NPHIR.V	3	2.30	0.26	-1.72
IPI00296441	Adenosine deaminase	R.LGHGYHTLEDQALYNR.L	3	3.72	0.25	-3.97
IPI00296441	Adenosine deaminase	R.TVHAGEVGSAEVVKEAVDILKTER.L	5	2.34	0.13	-0.22
IPI00296461	Isoform 1 of Sphingomyelin phosphodiesterase precursor	R.IGGFYALSPYPGLR.L	2	3.29	0.31	-4.53
IPI00296534	Isoform D of Fibulin-1 precursor	A.DVLLEACCADGHR.M	2	3.03	0.48	-2.30
IPI00296534	Isoform D of Fibulin-1 precursor	K.DCSLPYATESK.E	1	2.15	0.06	
IPI00296534	Isoform D of Fibulin-1 precursor	K.DCSLPYATESK.E	2	2.86	0.21	-3.65
IPI00296534	Isoform D of Fibulin-1 precursor	K.DCSLPYATESKECR.M	2	3.72	0.37	
IPI00296534	Isoform D of Fibulin-1 precursor	K.DIDECESGIHNCLPDFICQNTLGSFR.C	3	5.39	0.30	
IPI00296534	Isoform D of Fibulin-1 precursor	K.IIEVEEEQEDPYLNDR.C	2	6.01	0.38	
IPI00296534	Isoform D of Fibulin-1 precursor	K.IIEVEEEQEDPYLNDR.C	3	2.83	0.19	
IPI00296534	Isoform D of Fibulin-1 precursor	K.RCCHCCLLGRAAQAQGQSCEYSLMVGYQCGQVFR.A	3	3.61	0.15	
IPI00296534	Isoform D of Fibulin-1 precursor	K.SCRPNDVTCVFDPVHTISHTVISLPTFR.E	3	5.80	0.45	
IPI00296534	Isoform D of Fibulin-1 precursor	K.SQETGDLDVGGLQETDK.I	2	5.28	0.51	-3.36
IPI00296534	Isoform D of Fibulin-1 precursor	K.SQETGDLDVGGLQETDKIIEVEEEQEDPYLNDR.C	3	6.07	0.52	-2.24
IPI00296534	Isoform D of Fibulin-1 precursor	K.TGYYFDGISR.M	1	2.29	0.26	-3.34
IPI00296534	Isoform D of Fibulin-1 precursor	K.TGYYFDGISR.M	2	3.41	0.35	-3.13
IPI00296534	Isoform D of Fibulin-1 precursor	R.AAQAQGQSCEYSLM*VGYQCGQVFR.A	2	5.48	0.44	
IPI00296534	Isoform D of Fibulin-1 precursor	R.AAQAQGQSCEYSLM*VGYQCGQVFR.A	3	5.89	0.39	
IPI00296534	Isoform D of Fibulin-1 precursor	R.AAQAQGQSCEYSLMVGYQCGQVFR.A	2	5.48	0.44	

IPI00296534	Isoform D of Fibulin-1 precursor	R.AAQAQGQSCEYSLMVGYQCGQVFR.A	3	5.87	0.37	
IPI00296534	Isoform D of Fibulin-1 precursor	R.AITPPHPASQANIIFDITEGNLR.D	2	5.11	0.45	
IPI00296534	Isoform D of Fibulin-1 precursor	R.AITPPHPASQANIIFDITEGNLR.D	3	3.46	0.16	-5.11
IPI00296534	Isoform D of Fibulin-1 precursor	R.CATPHGDNASLEATFVKR.C	3	3.87	0.29	
IPI00296534	Isoform D of Fibulin-1 precursor	R.CLAFECPENYR.R	2	2.79	0.23	-2.83
IPI00296534	Isoform D of Fibulin-1 precursor	R.CLAFECPENYRR.S	2	2.32	0.10	
IPI00296534	Isoform D of Fibulin-1 precursor	R.CVDVDECAPPAEPCGK.G	2	4.39	0.32	
IPI00296534	Isoform D of Fibulin-1 precursor	R.DSFDIIKR.Y	2	1.85	0.09	-0.73
IPI00296534	Isoform D of Fibulin-1 precursor	R.DSSCGTGYELTEDNSCK.D	2	4.61	0.47	
IPI00296534	Isoform D of Fibulin-1 precursor	R.EFTRPEEIIFLR.A	2	3.37	0.15	
IPI00296534	Isoform D of Fibulin-1 precursor	R.GYHLNEEGTR.C	1	3.38	0.28	-1.40
IPI00296534	Isoform D of Fibulin-1 precursor	R.GYHLNEEGTR.C	2	2.95	0.34	-2.36
IPI00296534	Isoform D of Fibulin-1 precursor	R.GYHLNEEGTRCVDVDECAPPAEPCGK.G	2	4.55	0.36	
IPI00296534	Isoform D of Fibulin-1 precursor	R.GYHLNEEGTRCVDVDECAPPAEPCGK.G	3	6.29	0.38	
IPI00296534	Isoform D of Fibulin-1 precursor	R.GYQLSDVDGVTCEDIDECALPTGGHICSYR.C	2	4.38	0.40	
IPI00296534	Isoform D of Fibulin-1 precursor	R.GYQLSDVDGVTCEDIDECALPTGGHICSYR.C	3	6.52	0.46	
IPI00296534	Isoform D of Fibulin-1 precursor	R.M*CVDVNECQR.Y	2	3.56	0.47	-2.81
IPI00296534	Isoform D of Fibulin-1 precursor	R.M*VQEQCCHSQLEELHCATGISLANEQDR.C	3	6.38	0.45	
IPI00296534	Isoform D of Fibulin-1 precursor	R.M*VQEQCCHSQLEELHCATGISLANEQDR.C	4	3.36	0.16	-3.38
IPI00296534	Isoform D of Fibulin-1 precursor	R.MVQEQCCHSQLEELHCATGISLANEQDR.C	2	4.88	0.39	
IPI00296534	Isoform D of Fibulin-1 precursor	R.MVQEQCCHSQLEELHCATGISLANEQDR.C	3	7.00	0.32	
IPI00296534	Isoform D of Fibulin-1 precursor	R.RGYQLSDVDGVTCEDIDECALPTGGHICSYR.C	2	4.38	0.31	
IPI00296534	Isoform D of Fibulin-1 precursor	R.RGYQLSDVDGVTCEDIDECALPTGGHICSYR.C	3	6.75	0.43	
IPI00296534	Isoform D of Fibulin-1 precursor	R.SAATLQQEKTDTVR.C	2	4.41	0.31	
IPI00296534	Isoform D of Fibulin-1 precursor	R.SAATLQQEKTDTVR.C	3	2.62	0.26	
IPI00296534	Isoform D of Fibulin-1 precursor	R.YM*DGM*TVGVVR.Q	2	2.32	0.32	-3.44
IPI00296534	Isoform D of Fibulin-1 precursor	R.YMDGM*TVGVVR.Q	2	2.89	0.27	
IPI00296537	Isoform C of Fibulin-1 precursor	A.DVLLEACCADGHR.M	2	3.03	0.48	-2.30
IPI00296537	Isoform C of Fibulin-1 precursor	K.DCSLPYATESK.E	1	2.15	0.06	
IPI00296537	Isoform C of Fibulin-1 precursor	K.DCSLPYATESK.E	2	2.86	0.21	-3.65
IPI00296537	Isoform C of Fibulin-1 precursor	K.DCSLPYATESKECR.M	2	3.72	0.37	
IPI00296537	Isoform C of Fibulin-1 precursor	K.DIDECESGIHNCLPDFICQNTLGSFR.C	3	5.39	0.30	
IPI00296537	Isoform C of Fibulin-1 precursor	K.IIEVEEEQEDPYLNDR.C	2	6.01	0.38	
IPI00296537	Isoform C of Fibulin-1 precursor	K.IIEVEEEQEDPYLNDR.C	3	2.83	0.19	
IPI00296537	Isoform C of Fibulin-1 precursor	K.RCCHCCLLGRAAQAQGQSCEYSLMVGYQCGQVFR.A	3	3.61	0.15	
IPI00296537	Isoform C of Fibulin-1 precursor	K.SQETGDLDVGGLQETDK.I	2	5.28	0.51	-3.36
IPI00296537	Isoform C of Fibulin-1 precursor	K.SQETGDLDVGGLQETDKIIEVEEEQEDPYLNDR.C	3	6.07	0.52	-2.24
IPI00296537	Isoform C of Fibulin-1 precursor	K.TGYYFDGISR.M	1	2.29	0.26	-3.34
IPI00296537	Isoform C of Fibulin-1 precursor	K.TGYYFDGISR.M	2	3.41	0.35	-3.13
IPI00296537	Isoform C of Fibulin-1 precursor	K.VSPHSGVVALTKPVPEPR.D	2	5.23	0.60	-4.07
IPI00296537	Isoform C of Fibulin-1 precursor	K.VSPHSGVVALTKPVPEPR.D	3	2.50	0.21	-3.27
IPI00296537	Isoform C of Fibulin-1 precursor	K.VSPHSGVVALTKPVPEPR.D	4	4.64	0.45	-3.50

IPI00296537	Isoform C of Fibulin-1 precursor	R.AAQAQGQSCEYSLM*VGYQCGQVFR.A	2	5.48	0.44	
IPI00296537	Isoform C of Fibulin-1 precursor	R.AAQAQGQSCEYSLM*VGYQCGQVFR.A	3	5.89	0.39	
IPI00296537	Isoform C of Fibulin-1 precursor	R.AAQAQGQSCEYSLMVGYQCGQVFR.A	2	5.48	0.44	
IPI00296537	Isoform C of Fibulin-1 precursor	R.AAQAQGQSCEYSLMVGYQCGQVFR.A	3	5.87	0.37	
IPI00296537	Isoform C of Fibulin-1 precursor	R.CATPHGDNASLEATFVKR.C	3	3.87	0.29	
IPI00296537	Isoform C of Fibulin-1 precursor	R.CLAFECPENYR.R	2	2.79	0.23	-2.83
IPI00296537	Isoform C of Fibulin-1 precursor	R.CLAFECPENYRR.S	2	2.32	0.10	
IPI00296537	Isoform C of Fibulin-1 precursor	R.CVDVDECAPPAEPCGK.G	2	4.39	0.32	
IPI00296537	Isoform C of Fibulin-1 precursor	R.DSSCGTGYELTEDNSCK.D	2	4.61	0.47	
IPI00296537	Isoform C of Fibulin-1 precursor	R.ECSKLPLR.I	1	2.31	0.16	
IPI00296537	Isoform C of Fibulin-1 precursor	R.GYHLNEEGTR.C	1	3.38	0.28	-1.40
IPI00296537	Isoform C of Fibulin-1 precursor	R.GYHLNEEGTR.C	2	2.95	0.34	-2.36
IPI00296537	Isoform C of Fibulin-1 precursor	R.GYHLNEEGTRCVDVDECAPPAEPCGK.G	2	4.55	0.36	
IPI00296537	Isoform C of Fibulin-1 precursor	R.GYHLNEEGTRCVDVDECAPPAEPCGK.G	3	6.29	0.38	
IPI00296537	Isoform C of Fibulin-1 precursor	R.GYQLSDVDGVTCEDIDECALPTGGHICSYR.C	2	4.38	0.40	
IPI00296537	Isoform C of Fibulin-1 precursor	R.GYQLSDVDGVTCEDIDECALPTGGHICSYR.C	3	6.52	0.46	
IPI00296537	Isoform C of Fibulin-1 precursor	R.HGTVSSFVAK.L	1	2.05	0.26	
IPI00296537	Isoform C of Fibulin-1 precursor	R.HGTVSSFVAK.L	2	2.92	0.37	-3.17
IPI00296537	Isoform C of Fibulin-1 precursor	R.ITYYHLSFPTNIQAPAVVFR.M	2	5.75	0.59	-3.09
IPI00296537	Isoform C of Fibulin-1 precursor	R.ITYYHLSFPTNIQAPAVVFR.M	3	5.75	0.40	-3.84
IPI00296537	Isoform C of Fibulin-1 precursor	R.KVSPHSGVVALTKPVPEPR.D	3	2.82	0.23	-4.14
IPI00296537	Isoform C of Fibulin-1 precursor	R.LPCHENR.E	2	2.42	0.14	
IPI00296537	Isoform C of Fibulin-1 precursor	R.M*CVDVNECQR.Y	2	3.56	0.47	-2.81
IPI00296537	Isoform C of Fibulin-1 precursor	R.M*GPSSAVPGDSM*QLAITGGNEEGFFTTR.K	2	4.94	0.47	
IPI00296537	Isoform C of Fibulin-1 precursor	R.M*GPSSAVPGDSM*QLAITGGNEEGFFTTR.K	3	3.80	0.35	-4.59
IPI00296537	Isoform C of Fibulin-1 precursor	R.M*GPSSAVPGDSM*QLAITGGNEEGFFTTRK.V	3	2.44	0.26	-2.33
IPI00296537	Isoform C of Fibulin-1 precursor	R.M*GPSSAVPGDSMQLAITGGNEEGFFTTR.K	3	3.68	0.22	
IPI00296537	Isoform C of Fibulin-1 precursor	R.M*VQEQCCHSQLEELHCATGISLANEQDR.C	3	6.38	0.45	
IPI00296537	Isoform C of Fibulin-1 precursor	R.M*VQEQCCHSQLEELHCATGISLANEQDR.C	4	3.36	0.16	-3.38
IPI00296537	Isoform C of Fibulin-1 precursor	R.MVQEQCCHSQLEELHCATGISLANEQDR.C	2	4.88	0.39	
IPI00296537	Isoform C of Fibulin-1 precursor	R.MVQEQCCHSQLEELHCATGISLANEQDR.C	3	7.00	0.32	
IPI00296537	Isoform C of Fibulin-1 precursor	R.RGYQLSDVDGVTCEDIDECALPTGGHICSYR.C	2	4.38	0.31	
IPI00296537	Isoform C of Fibulin-1 precursor	R.RGYQLSDVDGVTCEDIDECALPTGGHICSYR.C	3	6.75	0.43	
IPI00296558	Carboxypeptidase-like protein X2 precursor	K.DGDYWR.L	2	2.23	0.10	-3.05
IPI00296558	Carboxypeptidase-like protein X2 precursor	K.ITDFQLHASTVKR.Y	2	3.36	0.29	-2.88
IPI00296558	Carboxypeptidase-like protein X2 precursor	K.TQEHTPTPDDHVFR.W	3	2.69	0.31	-2.66
IPI00296558	Carboxypeptidase-like protein X2 precursor	R.FTGVITQGR.N	2	2.81	0.15	-0.74
IPI00296558	Carboxypeptidase-like protein X2 precursor	R.RPQEPRPPK.R	2	2.31	0.13	-2.75
IPI00296608	Complement component C7 precursor	K.AASGTQNNVLR.G	1	2.18	0.18	-1.02
IPI00296608	Complement component C7 precursor	K.AASGTQNNVLR.G	2	3.74	0.31	-5.59
IPI00296608	Complement component C7 precursor	K.AASGTQNNVLRGEPFIR.G	2	3.19	0.21	-3.56
IPI00296608	Complement component C7 precursor	K.AASGTQNNVLRGEPFIR.G	3	2.53	0.44	-4.66

IPI00296608	Complement component C7 precursor	K.DGFVQDEGTM*FPVGK.N	2	4.36	0.52	-3.33
IPI00296608	Complement component C7 precursor	K.DGFVQDEGTM*FPVGK.N	3	2.96	0.29	-1.88
IPI00296608	Complement component C7 precursor	K.ELSHLPSLYDYSAYR.R	2	2.54	0.21	-2.06
IPI00296608	Complement component C7 precursor	K.ELSHLPSLYDYSAYR.R	3	4.18	0.35	-1.99
IPI00296608	Complement component C7 precursor	K.ENPLTQAVPK.C	2	1.92	0.05	-2.35
IPI00296608	Complement component C7 precursor	K.EQTM*SECEAGALR.C	2	3.25	0.38	-5.48
IPI00296608	Complement component C7 precursor	K.EVPCASVK.K	1	1.89	0.15	-1.72
IPI00296608	Complement component C7 precursor	K.IACVLPVLM*DGIQSHPQKPFYTVGEK.V	3	2.98	0.30	-4.45
IPI00296608	Complement component C7 precursor	K.IACVLPVLM*DGIQSHPQKPFYTVGEK.V	4	2.96	0.32	-3.29
IPI00296608	Complement component C7 precursor	K.LKQNDFNSVEEK.K	2	4.33	0.37	-3.81
IPI00296608	Complement component C7 precursor	K.LKQNDFNSVEEK.K	3	3.30	0.27	-3.30
IPI00296608	Complement component C7 precursor	K.LKQNDFNSVEEKK.C	2	4.22	0.31	-3.75
IPI00296608	Complement component C7 precursor	K.LKQNDFNSVEEKK.C	3	3.53	0.25	-1.85
IPI00296608	Complement component C7 precursor	K.LTPLYELVK.E	2	1.86	0.22	-1.62
IPI00296608	Complement component C7 precursor	K.M*PYECGPSLDVCAQDER.S	2	5.27	0.54	-2.50
IPI00296608	Complement component C7 precursor	K.M*PYECGPSLDVCAQDER.S	3	4.34	0.32	-2.69
IPI00296608	Complement component C7 precursor	K.NVVYTCNEGYSLIGNPVAR.C	2	5.74	0.54	-4.57
IPI00296608	Complement component C7 precursor	K.NVVYTCNEGYSLIGNPVAR.C	3	5.01	0.45	-3.06
IPI00296608	Complement component C7 precursor	K.QKLTPLYELVK.E	3	3.04	0.18	-3.88
IPI00296608	Complement component C7 precursor	K.QNDFNSVEEK.K	2	2.11	0.29	-3.39
IPI00296608	Complement component C7 precursor	K.SSGWHFVVK.F	2	2.34	0.15	-2.00
IPI00296608	Complement component C7 precursor	K.VFSGDGKDFYR.L	1	2.26	0.33	-2.68
IPI00296608	Complement component C7 precursor	K.VFSGDGKDFYR.L	2	3.64	0.45	-2.30
IPI00296608	Complement component C7 precursor	K.VTVSCSGGM*SLEGPSAFLCGSSLK.W	2	5.41	0.54	-5.39
IPI00296608	Complement component C7 precursor	K.VTVSCSGGM*SLEGPSAFLCGSSLK.W	3	5.95	0.46	-4.87
IPI00296608	Complement component C7 precursor	R.CFSGQCISK.S	2	1.97	0.14	-0.94
IPI00296608	Complement component C7 precursor	R.DSCTLPASAEK.A	1	2.30	0.36	-1.46
IPI00296608	Complement component C7 precursor	R.DSCTLPASAEK.A	2	3.68	0.34	-3.27
IPI00296608	Complement component C7 precursor	R.GCPTEEGCGER.F	2	3.12	0.38	-2.50
IPI00296608	Complement component C7 precursor	R.GGGAGFISGLSYLELDNPAGNK.R	2	6.20	0.54	-6.93
IPI00296608	Complement component C7 precursor	R.GGGAGFISGLSYLELDNPAGNK.R	3	5.16	0.41	-3.50
IPI00296608	Complement component C7 precursor	R.GGGAGFISGLSYLELDNPAGNKR.R	2	4.54	0.48	-4.97
IPI00296608	Complement component C7 precursor	R.GGGAGFISGLSYLELDNPAGNKR.R	3	3.47	0.37	0.26
IPI00296608	Complement component C7 precursor	R.GGGAGFISGLSYLELDNPAGNKRR.Y	3	3.41	0.37	-3.30
IPI00296608	Complement component C7 precursor	R.GGGAGFISGLSYLELDNPAGNKRR.Y	4	2.84	0.13	-1.09
IPI00296608	Complement component C7 precursor	R.GQSISVTSIRPCAAET.Q	2	3.00	0.37	-1.11
IPI00296608	Complement component C7 precursor	R.GQSISVTSIRPCAAETQ.-	2	5.34	0.50	-3.58
IPI00296608	Complement component C7 precursor	R.GQSISVTSIRPCAAETQ.-	3	2.63	0.19	-3.24
IPI00296608	Complement component C7 precursor	R.ILPLTVCK.M	1	2.32	0.24	-3.41
IPI00296608	Complement component C7 precursor	R.ILPLTVCK.M	2	2.07	0.29	-2.84
IPI00296608	Complement component C7 precursor	R.KVFSGDGK.D	2	1.70	0.06	-1.82
IPI00296608	Complement component C7 precursor	R.KVFSGDGKDFYR.L	2	3.90	0.35	-4.59

IPI00296608	Complement component C7 precursor	R.KVFSGDGKDFYR.L	3	2.21	0.18	-2.98
IPI00296608	Complement component C7 precursor	R.LLEPHCFPLSLVPTEFCPSPPALK.D	3	5.03	0.40	-3.97
IPI00296608	Complement component C7 precursor	R.LLEPHCFPLSLVPTEFCPSPPALKDGFVQDEGTM*FPVGK.N	4	5.43	0.45	-5.23
IPI00296608	Complement component C7 precursor	R.LSGNVLSYTFQVK.I	1	3.15	0.37	-0.52
IPI00296608	Complement component C7 precursor	R.LSGNVLSYTFQVK.I	2	4.78	0.42	-4.23
IPI00296608	Complement component C7 precursor	R.RLIDQYGTHYLQSGSLGGEYR.V	2	5.26	0.57	-2.74
IPI00296608	Complement component C7 precursor	R.RLIDQYGTHYLQSGSLGGEYR.V	3	6.12	0.56	-2.65
IPI00296608	Complement component C7 precursor	R.RLIDQYGTHYLQSGSLGGEYR.V	4	2.40	0.16	-1.65
IPI00296608	Complement component C7 precursor	R.SCVGETTESTQCEDEELEHLR.L	2	4.73	0.61	-2.75
IPI00296608	Complement component C7 precursor	R.SCVGETTESTQCEDEELEHLR.L	3	4.01	0.52	-1.75
IPI00296608	Complement component C7 precursor	R.SVAVYGQYGGQPCVGNAFETQSCEPTR.G	2	5.20	0.57	-4.11
IPI00296608	Complement component C7 precursor	R.SVAVYGQYGGQPCVGNAFETQSCEPTR.G	3	5.95	0.59	-5.90
IPI00296608	Complement component C7 precursor	R.SYTSHTNEIHKGK.S	2	3.74	0.44	-4.78
IPI00296608	Complement component C7 precursor	R.SYTSHTNEIHKGK.S	3	1.74	0.25	-2.87
IPI00296608	Complement component C7 precursor	R.VLFYVDSEK.L	1	2.39	0.27	-2.05
IPI00296608	Complement component C7 precursor	R.VLFYVDSEK.L	2	2.95	0.31	-4.11
IPI00296608	Complement component C7 precursor	R.VLFYVDSEKLK.Q	2	3.85	0.42	-3.38
IPI00296608	Complement component C7 precursor	R.VLFYVDSEKLK.Q	3	2.16	0.26	-2.40
IPI00296608	Complement component C7 precursor	R.VLFYVDSEKLKQNDFNSVEEK.K	3	3.19	0.23	-2.57
IPI00296608	Complement component C7 precursor	R.VLFYVDSEKLKQNDFNSVEEKK.C	3	4.39	0.37	-3.51
IPI00296608	Complement component C7 precursor	R.VLFYVDSEKLKQNDFNSVEEKK.C	4	3.38	0.40	-2.92
IPI00296608	Complement component C7 precursor	R.YSAWAESVTNLPQVIK.Q	2	4.84	0.38	-4.22
IPI00296608	Complement component C7 precursor	W.AESVTNLPQVIK.Q	2	4.26	0.29	-2.77
IPI00296727	Kinesin-like protein KIF2B	K.VYGTFFEIYGGKVYDLLNWKKK.L	3	2.98	0.14	
IPI00296777	SPARC-like protein 1 precursor	A.LLM*EPTDDGNTTPR.N	2	4.34	0.37	-4.33
IPI00296777	SPARC-like protein 1 precursor	A.PGVSSFTDSNQQESITKR.E	2	4.65	0.46	-3.68
IPI00296777	SPARC-like protein 1 precursor	A.PGVSSFTDSNQQESITKR.E	3	3.71	0.50	-2.20
IPI00296777	SPARC-like protein 1 precursor	E.KVHENENIGTTEPGEHQEAK.K	3	4.58	0.34	-3.90
IPI00296777	SPARC-like protein 1 precursor	F.TDSNQQESITKR.E	2	2.96	0.28	-2.36
IPI00296777	SPARC-like protein 1 precursor	G.LEAISNHKETEEK.T	2	3.73	0.22	-2.86
IPI00296777	SPARC-like protein 1 precursor	H.SASDDYFIPSQAFLEAER.A	2	4.06	0.42	-1.56
IPI00296777	SPARC-like protein 1 precursor	I.PSQAFLEAER.A	2	3.40	0.28	-1.31
IPI00296777	SPARC-like protein 1 precursor	I.PTCTDFEVIQFPLR.M	2	4.92	0.52	-3.32
IPI00296777	SPARC-like protein 1 precursor	K.ETAVSTEDDSHHK.A	2	2.22	0.26	-2.85
IPI00296777	SPARC-like protein 1 precursor	K.ETAVSTEDDSHHKAEK.S	2	4.09	0.46	-1.33
IPI00296777	SPARC-like protein 1 precursor	K.ETAVSTEDDSHHKAEK.S	3	1.74	0.15	-0.16
IPI00296777	SPARC-like protein 1 precursor	K.GHQLQLDYFGACK.S	2	4.44	0.43	-4.28
IPI00296777	SPARC-like protein 1 precursor	K.HIQETEWQSQEGK.T	2	4.39	0.49	-3.70
IPI00296777	SPARC-like protein 1 precursor	K.HIQETEWQSQEGK.T	3	3.50	0.28	-3.32
IPI00296777	SPARC-like protein 1 precursor	K.HSQGLRDQGNQEQD.P	2	3.71	0.52	-0.56
IPI00296777	SPARC-like protein 1 precursor	K.HSQGLRDQGNQEQDPNISNGEEEEEKEPGEVGTHNDNQER.K	4	5.92	0.55	-4.64
IPI00296777	SPARC-like protein 1 precursor	K.HSQGLRDQGNQEQDPNISNGEEEEEKEPGEVGTHNDNQER.K	5	4.93	0.46	-4.04

IPI00296777	SPARC-like protein 1 precursor	K.HSQGLRDQGNQEQDPNISNGEEEEEKEPGEVGTHNDNQER.K	6	2.21	0.11	-4.17
IPI00296777	SPARC-like protein 1 precursor	K.KAENSSNEEETSSEGNM*R.V	2	4.62	0.64	-3.71
IPI00296777	SPARC-like protein 1 precursor	K.KAENSSNEEETSSEGNM*R.V	3	2.90	0.34	-3.07
IPI00296777	SPARC-like protein 1 precursor	K.KLSENTDFLAPGVS.S	1	2.43	0.33	2.11
IPI00296777	SPARC-like protein 1 precursor	K.KLSENTDFLAPGVSSFT.D	2	2.94	0.26	-2.10
IPI00296777	SPARC-like protein 1 precursor	K.KLSENTDFLAPGVSSFTDSN.Q	2	5.22	0.62	-6.77
IPI00296777	SPARC-like protein 1 precursor	K.KLSENTDFLAPGVSSFTDSNQQES.I	2	5.00	0.54	-5.01
IPI00296777	SPARC-like protein 1 precursor	K.KLSENTDFLAPGVSSFTDSNQQESIT.K	2	4.74	0.53	-3.86
IPI00296777	SPARC-like protein 1 precursor	K.KLSENTDFLAPGVSSFTDSNQQESIT.K	3	4.43	0.38	-3.92
IPI00296777	SPARC-like protein 1 precursor	K.KLSENTDFLAPGVSSFTDSNQQESITK.R	2	5.04	0.52	-1.31
IPI00296777	SPARC-like protein 1 precursor	K.KLSENTDFLAPGVSSFTDSNQQESITK.R	3	6.67	0.46	-4.21
IPI00296777	SPARC-like protein 1 precursor	K.KLSENTDFLAPGVSSFTDSNQQESITKR.E	2	4.36	0.52	-2.93
IPI00296777	SPARC-like protein 1 precursor	K.KLSENTDFLAPGVSSFTDSNQQESITKR.E	3	7.09	0.60	-4.47
IPI00296777	SPARC-like protein 1 precursor	K.KLSENTDFLAPGVSSFTDSNQQESITKR.E	4	3.08	0.40	-3.43
IPI00296777	SPARC-like protein 1 precursor	K.KLSENTDFLAPGVSSFTDSNQQESITKREENQEQPR.N	3	4.18	0.39	-3.57
IPI00296777	SPARC-like protein 1 precursor	K.KLSENTDFLAPGVSSFTDSNQQESITKREENQEQPR.N	4	5.13	0.38	-3.68
IPI00296777	SPARC-like protein 1 precursor	K.KLSENTDFLAPGVSSFTDSNQQESITKREENQEQPR.N	5	2.35	0.19	-3.22
IPI00296777	SPARC-like protein 1 precursor	K.LSENTDFLAPGVSSFTDSNQQESITK.R	2	5.45	0.54	-3.63
IPI00296777	SPARC-like protein 1 precursor	K.LSENTDFLAPGVSSFTDSNQQESITK.R	3	4.59	0.48	-4.27
IPI00296777	SPARC-like protein 1 precursor	K.LSENTDFLAPGVSSFTDSNQQESITKR.E	2	4.02	0.38	-2.22
IPI00296777	SPARC-like protein 1 precursor	K.LSENTDFLAPGVSSFTDSNQQESITKR.E	3	5.40	0.49	-5.15
IPI00296777	SPARC-like protein 1 precursor	K.LSENTDFLAPGVSSFTDSNQQESITKR.E	4	2.74	0.12	-1.38
IPI00296777	SPARC-like protein 1 precursor	K.LSENTDFLAPGVSSFTDSNQQESITKREENQEQPR.N	3	3.70	0.28	-1.99
IPI00296777	SPARC-like protein 1 precursor	K.M*QEDEFDQGNQEQEDNSNAEM*EEENASNVNK.H	3	7.00	0.71	-2.42
IPI00296777	SPARC-like protein 1 precursor	K.NILM*QLYEANSEHAGYLNEK.Q	2	5.89	0.50	-4.52
IPI00296777	SPARC-like protein 1 precursor	K.NILM*QLYEANSEHAGYLNEK.Q	3	3.68	0.37	-4.02
IPI00296777	SPARC-like protein 1 precursor	K.NYHM*YVYPVHWQFSELDQHPM*DR.V	3	3.79	0.38	-4.12
IPI00296777	SPARC-like protein 1 precursor	K.NYHM*YVYPVHWQFSELDQHPM*DR.V	4	3.60	0.42	-3.92
IPI00296777	SPARC-like protein 1 precursor	K.NYHM*YVYPVHWQFSELDQHPM*DR.V	5	2.39	0.20	-3.05
IPI00296777	SPARC-like protein 1 precursor	K.QEEDNTQSDDILEESDQPTQVSK.M	2	4.30	0.52	-0.91
IPI00296777	SPARC-like protein 1 precursor	K.QEEDNTQSDDILEESDQPTQVSK.M	3	4.70	0.47	-2.08
IPI00296777	SPARC-like protein 1 precursor	K.RLLAGDHPIDLLLR.D	2	3.68	0.35	-3.29
IPI00296777	SPARC-like protein 1 precursor	K.RLLAGDHPIDLLLR.D	3	3.20	0.24	-2.30
IPI00296777	SPARC-like protein 1 precursor	K.RLLAGDHPIDLLLR.D	4	2.89	0.25	-4.52
IPI00296777	SPARC-like protein 1 precursor	K.SIPTCTDFEVIQFPLR.M	2	4.58	0.38	-4.04
IPI00296777	SPARC-like protein 1 precursor	K.SIPTCTDFEVIQFPLR.M	3	4.30	0.25	-4.76
IPI00296777	SPARC-like protein 1 precursor	K.SKEESHEQSAEQGK.S	2	2.96	0.39	-1.61
IPI00296777	SPARC-like protein 1 precursor	K.SKEESHEQSAEQGKSSSQELGLK.D	3	6.12	0.46	-4.54
IPI00296777	SPARC-like protein 1 precursor	K.SKEESHEQSAEQGKSSSQELGLK.D	4	4.36	0.50	-2.87
IPI00296777	SPARC-like protein 1 precursor	K.SSSQELGLK.D	1	1.78	0.09	-1.65
IPI00296777	SPARC-like protein 1 precursor	K.SSSQELGLK.D	2	2.60	0.23	-1.02
IPI00296777	SPARC-like protein 1 precursor	K.SSSQELGLKDQEDS.D	2	3.29	0.23	-3.34

IPI00296777	SPARC-like protein 1 precursor	K.SSSQELGLKDQEDSDGDLS.V	2	4.12	0.46	-3.67
IPI00296777	SPARC-like protein 1 precursor	K.TGLEAISNHK.E	1	2.45	0.29	-3.92
IPI00296777	SPARC-like protein 1 precursor	K.TGLEAISNHK.E	2	3.20	0.26	-5.45
IPI00296777	SPARC-like protein 1 precursor	K.TGLEAISNHKET.E	2	3.11	0.40	-2.00
IPI00296777	SPARC-like protein 1 precursor	K.TGLEAISNHKETEE.K	2	3.13	0.11	1.69
IPI00296777	SPARC-like protein 1 precursor	K.TGLEAISNHKETEEK.T	2	4.72	0.49	-4.51
IPI00296777	SPARC-like protein 1 precursor	K.TGLEAISNHKETEEK.T	3	2.98	0.30	-3.57
IPI00296777	SPARC-like protein 1 precursor	K.TGLEAISNHKETEEK.T	4	3.16	0.33	-5.06
IPI00296777	SPARC-like protein 1 precursor	K.TGLEAISNHKETEEKT.V	2	4.26	0.49	-4.62
IPI00296777	SPARC-like protein 1 precursor	K.TGLEAISNHKETEEKT.V	3	3.50	0.44	-2.11
IPI00296777	SPARC-like protein 1 precursor	K.TGLEAISNHKETEEKTVSEA.L	2	4.40	0.43	-5.25
IPI00296777	SPARC-like protein 1 precursor	K.TGLEAISNHKETEEKTVSEA.L	3	4.78	0.47	-3.08
IPI00296777	SPARC-like protein 1 precursor	K.TGLEAISNHKETEEKTVSEALLM*EPTDDGNTTPR.N	3	6.57	0.57	-3.74
IPI00296777	SPARC-like protein 1 precursor	K.TVSEALLM*EPTDDGNTTPR.N	2	5.77	0.52	-5.10
IPI00296777	SPARC-like protein 1 precursor	K.TVSEALLM*EPTDDGNTTPR.N	3	4.57	0.42	-4.14
IPI00296777	SPARC-like protein 1 precursor	K.VHENENIGTTEPGEHQ.E	2	4.00	0.47	-3.92
IPI00296777	SPARC-like protein 1 precursor	K.VHENENIGTTEPGEHQE.A	2	4.87	0.59	-4.73
IPI00296777	SPARC-like protein 1 precursor	K.VHENENIGTTEPGEHQE.A	3	4.18	0.33	-2.75
IPI00296777	SPARC-like protein 1 precursor	K.VHENENIGTTEPGEHQEA.K	3	4.63	0.36	-2.62
IPI00296777	SPARC-like protein 1 precursor	K.VHENENIGTTEPGEHQEAK.K	2	6.56	0.60	-3.69
IPI00296777	SPARC-like protein 1 precursor	K.VHENENIGTTEPGEHQEAK.K	3	4.38	0.45	-3.40
IPI00296777	SPARC-like protein 1 precursor	K.VHENENIGTTEPGEHQEAK.K	4	2.53	0.23	-2.93
IPI00296777	SPARC-like protein 1 precursor	K.VHENENIGTTEPGEHQEAKK.A	3	3.68	0.32	-3.27
IPI00296777	SPARC-like protein 1 precursor	L.LAGDHPIDLLLR.D	2	3.10	0.39	-2.70
IPI00296777	SPARC-like protein 1 precursor	L.LM*EPTDDGNTTPR.N	2	4.38	0.42	-3.65
IPI00296777	SPARC-like protein 1 precursor	L.M*EPTDDGNTTPR.N	2	3.65	0.37	-3.93
IPI00296777	SPARC-like protein 1 precursor	L.SENTDFLAPGVSSFTDSNQQESITKR.E	3	4.27	0.42	-2.70
IPI00296777	SPARC-like protein 1 precursor	R.AEDEENEKETAVSTEDDSHHK.A	3	4.19	0.49	-3.21
IPI00296777	SPARC-like protein 1 precursor	R.AEDEENEKETAVSTEDDSHHKAEK.S	3	5.77	0.54	-4.90
IPI00296777	SPARC-like protein 1 precursor	R.AEDEENEKETAVSTEDDSHHKAEK.S	4	2.60	0.15	-2.68
IPI00296777	SPARC-like protein 1 precursor	R.AEDEENEKETAVSTEDDSHHKAEK.S	5	2.37	0.15	-3.04
IPI00296777	SPARC-like protein 1 precursor	R.AQSIAYHL.K	1	1.97	0.28	-2.37
IPI00296777	SPARC-like protein 1 precursor	R.AQSIAYHLK.I	1	2.10	0.08	-3.85
IPI00296777	SPARC-like protein 1 precursor	R.AQSIAYHLK.I	2	3.03	0.32	-2.51
IPI00296777	SPARC-like protein 1 precursor	R.DQGNQEQDPNISNGEEEEEKEPGEVGTHNDN.Q	3	4.11	0.47	-3.21
IPI00296777	SPARC-like protein 1 precursor	R.DQGNQEQDPNISNGEEEEEKEPGEVGTHNDNQER.K	3	5.61	0.62	-3.24
IPI00296777	SPARC-like protein 1 precursor	R.DQGNQEQDPNISNGEEEEEKEPGEVGTHNDNQER.K	4	5.03	0.56	-4.37
IPI00296777	SPARC-like protein 1 precursor	R.DQGNQEQDPNISNGEEEEEKEPGEVGTHNDNQERK.T	4	4.28	0.46	-1.26
IPI00296777	SPARC-like protein 1 precursor	R.DQGNQEQDPNISNGEEEEEKEPGEVGTHNDNQERK.T	5	2.77	0.31	-0.98
IPI00296777	SPARC-like protein 1 precursor	R.EHANSKQEEDNTQSDDILEESDQPTQVSK.M	3	6.52	0.55	-3.32
IPI00296777	SPARC-like protein 1 precursor	R.EHANSKQEEDNTQSDDILEESDQPTQVSK.M	4	4.41	0.39	-2.71
IPI00296777	SPARC-like protein 1 precursor	R.EKVHENENIGTTEPGEHQE.A	3	4.23	0.33	-6.64

IPI00296777	SPARC-like protein 1 precursor	R.EKVHENENIGTTEPGEHQEA.K	2	5.49	0.56	-4.36
IPI00296777	SPARC-like protein 1 precursor	R.EKVHENENIGTTEPGEHQEA.K	3	5.37	0.45	-3.41
IPI00296777	SPARC-like protein 1 precursor	R.EKVHENENIGTTEPGEHQEAK.K	2	4.68	0.50	-3.32
IPI00296777	SPARC-like protein 1 precursor	R.EKVHENENIGTTEPGEHQEAK.K	3	4.85	0.44	-3.97
IPI00296777	SPARC-like protein 1 precursor	R.EKVHENENIGTTEPGEHQEAK.K	4	2.97	0.32	-3.13
IPI00296777	SPARC-like protein 1 precursor	R.FFEECDPNKDK.H	2	2.55	0.23	
IPI00296777	SPARC-like protein 1 precursor	R.FFEECDPNKDKHITLK.E	4	2.59	0.17	0.67
IPI00296777	SPARC-like protein 1 precursor	R.HSASDDYFIPSQA.F	1	2.67	0.45	-2.95
IPI00296777	SPARC-like protein 1 precursor	R.HSASDDYFIPSQAFLE.A	2	3.68	0.49	-4.11
IPI00296777	SPARC-like protein 1 precursor	R.HSASDDYFIPSQAFLEAER.A	2	6.39	0.60	-7.58
IPI00296777	SPARC-like protein 1 precursor	R.HSASDDYFIPSQAFLEAER.A	3	4.70	0.34	-3.48
IPI00296777	SPARC-like protein 1 precursor	R.KTELPREHANSKQEEDNTQSDDILEESDQPTQVSK.M	5	3.17	0.34	-3.00
IPI00296777	SPARC-like protein 1 precursor	R.LLAGDHPIDLLLR.D	1	2.65	0.42	-1.58
IPI00296777	SPARC-like protein 1 precursor	R.LLAGDHPIDLLLR.D	2	4.50	0.43	-3.28
IPI00296777	SPARC-like protein 1 precursor	R.NHGVDDDGDDDGDDGGTDGPR.H	2	5.16	0.68	-4.11
IPI00296777	SPARC-like protein 1 precursor	R.NHGVDDDGDDDGDDGGTDGPR.H	3	6.20	0.74	-4.50
IPI00296777	SPARC-like protein 1 precursor	R.VHAVDSCM*SFQCK.R	2	4.39	0.56	-4.79
IPI00296777	SPARC-like protein 1 precursor	R.VHAVDSCM*SFQCK.R	3	2.72	0.16	-3.56
IPI00296777	SPARC-like protein 1 precursor	R.VLTHSELAPLR.A	1	3.11	0.20	-3.38
IPI00296777	SPARC-like protein 1 precursor	R.VLTHSELAPLR.A	2	3.28	0.30	-3.27
IPI00296777	SPARC-like protein 1 precursor	R.VLTHSELAPLR.A	3	3.74	0.16	-3.98
IPI00296777	SPARC-like protein 1 precursor	S.DDILEESDQPTQVSK.M	2	3.63	0.40	-2.32
IPI00296777	SPARC-like protein 1 precursor	S.FTDSNQQESITK.R	2	3.98	0.35	-3.36
IPI00296777	SPARC-like protein 1 precursor	S.FTDSNQQESITKR.E	2	4.14	0.37	-1.37
IPI00296777	SPARC-like protein 1 precursor	T.GLEAISNHKETEEK.T	2	4.15	0.42	-3.40
IPI00296866	interphotoreceptor matrix proteoglycan 2	K.SAVSFLLPEESTDLSLATK.K	2	3.73	0.41	-4.78
IPI00296913	ADP-sugar pyrophosphatase	R.VYSYALALK.H	2	2.56	0.24	-1.61
IPI00296922	Laminin subunit beta-2 precursor	K.AM*DYDLLLR.L	2	2.57	0.14	-2.89
IPI00296922	Laminin subunit beta-2 precursor	K.LGIVQGIVGAR.N	2	3.74	0.22	-1.35
IPI00296922	Laminin subunit beta-2 precursor	K.TFRPAAM*LVER.S	3	3.79	0.17	-0.95
IPI00296922	Laminin subunit beta-2 precursor	R.AEQLRDEAR.D	2	2.26	0.05	-1.49
IPI00296922	Laminin subunit beta-2 precursor	R.AGNSLAASTAEETAGSAQGR.A	2	4.92	0.58	-0.94
IPI00296922	Laminin subunit beta-2 precursor	R.AGNSLAASTAEETAGSAQGR.A	3	3.49	0.40	-1.65
IPI00296922	Laminin subunit beta-2 precursor	R.ALAEGGSILSR.V	2	2.59	0.18	-2.18
IPI00296922	Laminin subunit beta-2 precursor	R.AQGIAQGAIR.G	2	2.96	0.26	0.45
IPI00296922	Laminin subunit beta-2 precursor	R.DLLQAAQDKLQR.L	2	3.16	0.34	-2.11
IPI00296922	Laminin subunit beta-2 precursor	R.DTEQTLYQVQER.M	2	3.75	0.31	-2.38
IPI00296922	Laminin subunit beta-2 precursor	R.EIGEATEHLTQLEADLTDVQDENFNANHALSGLER.D	3	7.27	0.61	-4.26
IPI00296922	Laminin subunit beta-2 precursor	R.EIGEATEHLTQLEADLTDVQDENFNANHALSGLER.D	4	4.88	0.32	-3.18
IPI00296922	Laminin subunit beta-2 precursor	R.GQVEQANQELQELIQSVK.D	2	5.91	0.44	-3.89
IPI00296922	Laminin subunit beta-2 precursor	R.GQVEQANQELQELIQSVKDFLNQEGADPDSIEM*VATR.V	4	4.19	0.20	-4.40
IPI00296922	Laminin subunit beta-2 precursor	R.GQVLDVVER.L	2	2.57	0.15	-2.55

IPI00296922	Laminin subunit beta-2 precursor	R.HTQAELQR.A	2	1.88	0.21	-1.78
IPI00296922	Laminin subunit beta-2 precursor	R.IQGTLQPHAR.Y	2	2.43	0.26	-1.88
IPI00296922	Laminin subunit beta-2 precursor	R.IQNVVTSFAPQRR.A	2	1.92	0.11	-2.83
IPI00296922	Laminin subunit beta-2 precursor	R.LQEGQTLEFLVASVPK.A	2	4.30	0.47	-3.60
IPI00296922	Laminin subunit beta-2 precursor	R.TGGSAQPETPYSGPGLLIDSLVLLPR.V	2	4.45	0.54	-6.55
IPI00296922	Laminin subunit beta-2 precursor	R.TGGSAQPETPYSGPGLLIDSLVLLPR.V	3	3.28	0.19	-4.07
IPI00296922	Laminin subunit beta-2 precursor	R.VLELSIPASAEQIQHLAGAIAER.V	3	2.49	0.26	-3.98
IPI00296922	Laminin subunit beta-2 precursor	R.YFSYDCGADFPGVPLAPPR.H	2	3.37	0.32	-2.89
IPI00296922	Laminin subunit beta-2 precursor	R.YLIFPNPVCLEPGISYK.L	2	4.03	0.42	-4.08
IPI00296922	Laminin subunit beta-2 precursor	R.YSEIEPSTEGEVIYR.V	2	3.67	0.31	-0.71
IPI00296992	AXL receptor tyrosine kinase isoform 1	K.TSSFSCEAHNAK.G	2	2.83	0.18	
IPI00296992	AXL receptor tyrosine kinase isoform 1	R.APLQGTLLGYR.L	1	3.36	0.43	-2.16
IPI00296992	AXL receptor tyrosine kinase isoform 1	R.APLQGTLLGYR.L	2	3.62	0.39	-2.13
IPI00296992	AXL receptor tyrosine kinase isoform 1	R.LAYQGQDTPEVLM*DIGLR.Q	2	5.42	0.51	-4.33
IPI00296992	AXL receptor tyrosine kinase isoform 1	R.LAYQGQDTPEVLM*DIGLR.Q	3	4.02	0.33	-2.65
IPI00296992	AXL receptor tyrosine kinase isoform 1	R.TATITVLPQQPR.N	1	2.35	0.38	-3.04
IPI00296992	AXL receptor tyrosine kinase isoform 1	R.TATITVLPQQPR.N	2	3.43	0.39	-3.17
IPI00297040	Serine protease inhibitor Kazal-type 6 precursor	K.ISLKHPGK.C	1	1.61	0.13	-4.91
IPI00297124	Isoform 1 of Interleukin-6 receptor subunit beta precursor	A.YTDEGGKDGPEFTFTTPK.F	2	4.71	0.51	-3.26
IPI00297124	Isoform 1 of Interleukin-6 receptor subunit beta precursor	A.YTDEGGKDGPEFTFTTPK.F	3	3.62	0.41	-2.76
IPI00297124	Isoform 1 of Interleukin-6 receptor subunit beta precursor	K.CYLITVTPVYADGPGSPESIK.A	2	5.61	0.54	-2.95
IPI00297124	Isoform 1 of Interleukin-6 receptor subunit beta precursor	K.DNM*LWVEWTTPR.E	2	3.27	0.39	-4.46
IPI00297124	Isoform 1 of Interleukin-6 receptor subunit beta precursor	K.IDPSHTQGYR.T	2	2.27	0.41	-3.49
IPI00297124	Isoform 1 of Interleukin-6 receptor subunit beta precursor	K.ILDYEVTLTR.W	1	2.39	0.20	-4.07
IPI00297124	Isoform 1 of Interleukin-6 receptor subunit beta precursor	K.ILDYEVTLTR.W	2	3.94	0.40	-4.32
IPI00297124	Isoform 1 of Interleukin-6 receptor subunit beta precursor	K.LTWTNPSIK.S	2	2.07	0.07	-2.13
IPI00297124	Isoform 1 of Interleukin-6 receptor subunit beta precursor	K.NEAVLEWDQLPVDVQNGFIR.N	3	4.32	0.40	-2.33
IPI00297124	Isoform 1 of Interleukin-6 receptor subunit beta precursor	K.SDAAVLTIPACDFQATHPVM*DLK.A	2	2.90	0.23	-6.53
IPI00297124	Isoform 1 of Interleukin-6 receptor subunit beta precursor	K.SDAAVLTIPACDFQATHPVM*DLK.A	3	2.65	0.11	-3.96
IPI00297124	Isoform 1 of Interleukin-6 receptor subunit beta precursor	K.TNHFTIPK.E	1	2.03	0.14	-3.95

IPI00297124	Isoform 1 of Interleukin-6 receptor subunit beta precursor	K.VKPNPPHNLSVINSEELSSILK.L	2	4.23	0.51	-3.51
IPI00297124	Isoform 1 of Interleukin-6 receptor subunit beta precursor	K.VKPNPPHNLSVINSEELSSILK.L	3	6.49	0.52	-5.04
IPI00297124	Isoform 1 of Interleukin-6 receptor subunit beta precursor	K.VKPNPPHNLSVINSEELSSILK.L	4	3.42	0.25	-2.47
IPI00297124	Isoform 1 of Interleukin-6 receptor subunit beta precursor	K.VTSDHINFDPVYK.V	2	3.15	0.31	-5.07
IPI00297124	Isoform 1 of Interleukin-6 receptor subunit beta precursor	K.VTSDHINFDPVYK.V	3	2.52	0.19	1.07
IPI00297124	Isoform 1 of Interleukin-6 receptor subunit beta precursor	K.YNIQYR.T	1	1.83	0.14	-1.01
IPI00297124	Isoform 1 of Interleukin-6 receptor subunit beta precursor	K.YNIQYR.T	2	2.20	0.13	-1.81
IPI00297124	Isoform 1 of Interleukin-6 receptor subunit beta precursor	R.M*AAYTDEGGKDGPEFTFTTPK.F	2	4.59	0.55	-3.12
IPI00297124	Isoform 1 of Interleukin-6 receptor subunit beta precursor	R.M*AAYTDEGGKDGPEFTFTTPK.F	3	3.93	0.52	-2.83
IPI00297124	Isoform 1 of Interleukin-6 receptor subunit beta precursor	R.SSFTVQDLKPFTEYVFR.I	2	4.67	0.41	-4.00
IPI00297124	Isoform 1 of Interleukin-6 receptor subunit beta precursor	R.SSFTVQDLKPFTEYVFR.I	3	4.82	0.42	-4.35
IPI00297124	Isoform 1 of Interleukin-6 receptor subunit beta precursor	R.YLATLTVR.N	2	2.31	0.14	-1.36
IPI00297180	Cadherin-9 precursor	K.DNTAGIM*TR.K	2	1.99	0.22	-2.46
IPI00297180	Cadherin-9 precursor	K.LHTDQDKGDGNLK.Y	2	3.62	0.38	-0.63
IPI00297181	Cadherin-7 precursor	K.IQDINDNEPK.F	2	3.38	0.25	-3.72
IPI00297181	Cadherin-7 precursor	K.RLDREEQAYYTLR.A	3	2.54	0.10	-2.44
IPI00297181	Cadherin-7 precursor	R.VVYSILQGQPYFSVEPK.T	2	3.45	0.15	
IPI00297188	Brain-specific angiogenesis inhibitor 2 precursor	K.NFVQLCLSAEPSEAPR.L	2	5.38	0.46	-4.53
IPI00297188	Brain-specific angiogenesis inhibitor 2 precursor	L.APAALAFR.F	1	2.10	0.23	-2.33
IPI00297188	Brain-specific angiogenesis inhibitor 2 precursor	R.LLAPAALAFR.F	1	1.92	0.27	-2.56
IPI00297188	Brain-specific angiogenesis inhibitor 2 precursor	R.LLAPAALAFR.F	2	3.60	0.24	-1.89
IPI00297188	Brain-specific angiogenesis inhibitor 2 precursor	R.YGEEPEEEPKVK.T	2	3.43	0.24	-2.02
IPI00297208	similar to Myosin-10	L.EVLRLEEFIQQNK.T	1	4.33	0.12	
IPI00297224	Sushi domain-containing protein 5	K.GSGEQQIM*R.A	2	2.85	0.16	-3.13
IPI00297224	Sushi domain-containing protein 5	R.ELM*EDSRTEADEDRGQGDSSEEAPKQD.R	3	3.51	0.41	-2.68
IPI00297224	Sushi domain-containing protein 5	R.ELM*EDSRTEADEDRGQGDSSEEAPKQDR.L	4	4.40	0.43	-4.24
IPI00297224	Sushi domain-containing protein 5	R.GAHLASADELR.R	2	2.19	0.19	-2.25
IPI00297224	Sushi domain-containing protein 5	R.GAHLASADELRR.V	3	2.86	0.32	-4.60
IPI00297224	Sushi domain-containing protein 5	R.LVSISVGR.E	1	1.30	0.09	-2.93
IPI00297224	Sushi domain-containing protein 5	R.TEADEDRGQGDSSEEAPKQDR.L	3	4.55	0.44	-2.73

IPI00297224	Sushi domain-containing protein 5	R.TGLEM*GDELLYVCAPGHIM*GHR.E	3	3.10	0.37	-2.72
IPI00297224	Sushi domain-containing protein 5	R.TGLEM*GDELLYVCAPGHIM*GHR.E	4	3.02	0.23	-2.83
IPI00297251	Isoform 2 of Probable E3 ubiquitin-protein ligase MGRN1	F.ITEEVDESSSPQQGTR.A	2	4.42	0.31	-2.76
IPI00297252	Isoform 1 of Extracellular sulfatase Sulf-2 precursor	K.LHIDHEIETLQNK.I	3	3.92	0.28	-1.30
IPI00297252	Isoform 1 of Extracellular sulfatase Sulf-2 precursor	R.SVAIEVDGR.V	1	1.68	0.07	-3.26
IPI00297252	Isoform 1 of Extracellular sulfatase Sulf-2 precursor	R.SVAIEVDGR.V	2	2.82	0.26	-3.10
IPI00297252	Isoform 1 of Extracellular sulfatase Sulf-2 precursor	R.VYHVGLGDAAQPR.N	1	3.36	0.34	-3.01
IPI00297252	Isoform 1 of Extracellular sulfatase Sulf-2 precursor	R.VYHVGLGDAAQPR.N	2	3.57	0.33	-3.78
IPI00297252	Isoform 1 of Extracellular sulfatase Sulf-2 precursor	R.VYHVGLGDAAQPR.N	3	4.60	0.26	-3.76
IPI00297263	Isoform 1 of Protein HEG homolog 1 precursor	K.EGVM*VQTSGK.S	2	2.38	0.42	-4.35
IPI00297263	Isoform 1 of Protein HEG homolog 1 precursor	K.SGTASEM*GTER.A	2	1.88	0.07	-2.42
IPI00297263	Isoform 1 of Protein HEG homolog 1 precursor	K.TM*HVATVFTDGGPR.T	3	2.74	0.06	-2.46
IPI00297263	Isoform 1 of Protein HEG homolog 1 precursor	R.ALSLAPLAGAGLELQLER.R	2	4.12	0.44	-3.47
IPI00297263	Isoform 1 of Protein HEG homolog 1 precursor	R.RALSLAPLAGAGLELQLER.R	3	2.78	0.18	-1.41
IPI00297263	Isoform 1 of Protein HEG homolog 1 precursor	R.SLTVSLGPVSK.T	2	3.19	0.26	-2.13
IPI00297263	Isoform 1 of Protein HEG homolog 1 precursor	R.SYSESSSTSSSESLNSSAPR.G	2	5.48	0.53	-3.33
IPI00297263	Isoform 1 of Protein HEG homolog 1 precursor	R.TSSDHTDHTYLSSTFTK.G	2	4.01	0.53	-3.46
IPI00297263	Isoform 1 of Protein HEG homolog 1 precursor	R.TSSDHTDHTYLSSTFTKGER.A	3	4.63	0.45	-3.16
IPI00297263	Isoform 1 of Protein HEG homolog 1 precursor	R.VTGNPGDEEFIEPSTENEFGLTSLR.W	2	4.76	0.43	-5.98
IPI00297263	Isoform 1 of Protein HEG homolog 1 precursor	R.VTGNPGDEEFIEPSTENEFGLTSLR.W	3	4.96	0.46	-4.31
IPI00297277	Isoform 1 of RING finger protein 150 precursor	R.GEVVM*ASSAHDR.L	2	3.32	0.45	-2.65
IPI00297277	Isoform 1 of RING finger protein 150 precursor	R.GEVVM*ASSAHDR.L	3	2.03	0.18	-3.71
IPI00297284	Insulin-like growth factor-binding protein 2 precursor	A.PPAAVAAVAGGAR.M	2	4.57	0.50	-2.21
IPI00297284	Insulin-like growth factor-binding protein 2 precursor	F.YNEQQEAR.G	2	3.26	0.23	-1.03
IPI00297284	Insulin-like growth factor-binding protein 2 precursor	K.GGKHHLGLEEPK.K	2	2.30	0.30	-3.38
IPI00297284	Insulin-like growth factor-binding protein 2 precursor	K.HHLGLEEPK.K	2	2.53	0.19	-3.22
IPI00297284	Insulin-like growth factor-binding protein 2 precursor	K.LIQGAPTIR.G	1	2.26	0.19	-2.71
IPI00297284	Insulin-like growth factor-binding protein 2 precursor	K.LIQGAPTIRGDPECHLFYNEQQEAR.G	3	4.29	0.36	-3.06

IPI00297284	Insulin-like growth factor-binding protein 2 precursor	K.SGM*KELAVFR.E	1	1.32	0.10	-1.81
IPI00297284	Insulin-like growth factor-binding protein 2 precursor	K.SGM*KELAVFR.E	2	2.87	0.15	0.06
IPI00297284	Insulin-like growth factor-binding protein 2 precursor	R.DAEYGASPEQVADNGDDHSEGGLVENHVDSTM*NM*LGGGGSAGR.K	3	4.83	0.56	-2.67
IPI00297284	Insulin-like growth factor-binding protein 2 precursor	R.DAEYGASPEQVADNGDDHSEGGLVENHVDSTM*NM*LGGGGSAGR.K	4	5.22	0.57	-2.18
IPI00297284	Insulin-like growth factor-binding protein 2 precursor	R.EPGCGCCSVCAR.L	2	2.29	0.46	-2.57
IPI00297284	Insulin-like growth factor-binding protein 2 precursor	R.GDPECHLFYNEQQEAR.G	3	2.97	0.29	-2.26
IPI00297284	Insulin-like growth factor-binding protein 2 precursor	R.GECWCVNPNTGK.L	2	4.09	0.44	-3.54
IPI00297284	Insulin-like growth factor-binding protein 2 precursor	R.LAACGPPPVAPPAAVAAVAGGAR.M	2	5.61	0.58	-3.17
IPI00297284	Insulin-like growth factor-binding protein 2 precursor	R.LAACGPPPVAPPAAVAAVAGGAR.M	3	3.90	0.35	-4.80
IPI00297284	Insulin-like growth factor-binding protein 2 precursor	R.LEGEACGVYTPR.C	2	3.99	0.44	-3.49
IPI00297284	Insulin-like growth factor-binding protein 2 precursor	R.RDAEYGASPEQVADNGDDHSEGGLVENHVDSTM*NM*LGGGGSAGR.K	5	3.80	0.39	-1.07
IPI00297284	Insulin-like growth factor-binding protein 2 precursor	R.TPCQQELDQVLER.I	2	4.06	0.24	
IPI00297288	Cdc42 GTPase-activating protein	R.ELPNPLLTYELYEK.F	2	2.65	0.11	0.08
IPI00297444	Isoform 1 of CD177 antigen precursor	R.GGGIFSNLR.V	2	2.44	0.19	-0.97
IPI00297487	Cathepsin H precursor	A.AELSVNSLEK.F	1	2.04	0.17	-4.07
IPI00297487	Cathepsin H precursor	K.GIM*GEDTYPYQGK.D	2	3.84	0.49	-4.01
IPI00297487	Cathepsin H precursor	K.GNFVSPVK.N	1	2.17	0.28	0.24
IPI00297487	Cathepsin H precursor	K.GNFVSPVK.N	2	2.12	0.13	-1.19
IPI00297487	Cathepsin H precursor	K.M*ALNQFSDM*SFAEIK.H	2	5.11	0.38	-5.67
IPI00297487	Cathepsin H precursor	K.M*LSLAEQQLVDCAQDFNNHGCQGGLPSQAFEYILYNK.G	3	6.85	0.55	-3.39
IPI00297487	Cathepsin H precursor	K.M*LSLAEQQLVDCAQDFNNHGCQGGLPSQAFEYILYNK.G	4	6.55	0.50	-4.34
IPI00297487	Cathepsin H precursor	K.NQGACGSCWTFSTTGALESAIAIATGK.M	3	4.92	0.46	-5.18
IPI00297487	Cathepsin H precursor	K.TPDKVNHAVLAVGYGEK.N	2	5.98	0.55	-3.41
IPI00297487	Cathepsin H precursor	K.TPDKVNHAVLAVGYGEK.N	3	4.17	0.42	-1.88
IPI00297487	Cathepsin H precursor	K.TPDKVNHAVLAVGYGEK.N	4	2.88	0.18	-0.16
IPI00297487	Cathepsin H precursor	K.TYSTEEYHHR.L	2	2.58	0.43	-3.87
IPI00297487	Cathepsin H precursor	R.KTYSTEEYHHR.L	2	3.69	0.48	-6.24
IPI00297550	Coagulation factor XIII A chain precursor	K.KPLNTEGVM*K.S	2	3.00	0.16	
IPI00297646	Collagen alpha-1(I) chain precursor	K.ALLLQGSNEIEIR.A	2	3.64	0.34	-5.13
IPI00297646	Collagen alpha-1(I) chain precursor	K.NSVAYM*DQQTGNLK.K	2	3.93	0.38	-2.90

IPI00297646	Collagen alpha-1(I) chain precursor	K.NSVAYM*DQQTGNLKK.A	2	4.30	0.45	-2.94
IPI00297646	Collagen alpha-1(I) chain precursor	K.NSVAYM*DQQTGNLKK.A	3	2.14	0.22	-1.85
IPI00297646	Collagen alpha-1(I) chain precursor	K.SGDRGETGPAGPAGPVGPVG.A	2	4.71	0.60	-2.08
IPI00297646	Collagen alpha-1(I) chain precursor	K.SGDRGETGPAGPAGPVGPVGAR.G	2	5.19	0.55	-3.20
IPI00297646	Collagen alpha-1(I) chain precursor	K.SGDRGETGPAGPAGPVGPVGAR.G	3	4.57	0.41	-2.17
IPI00297646	Collagen alpha-1(I) chain precursor	K.SGEYWIDPNQGCNLDAIK.V	2	5.27	0.51	-1.94
IPI00297646	Collagen alpha-1(I) chain precursor	K.SLSQQIENIR.S	1	2.13	0.13	-2.93
IPI00297646	Collagen alpha-1(I) chain precursor	K.SLSQQIENIR.S	2	3.79	0.17	-3.98
IPI00297646	Collagen alpha-1(I) chain precursor	K.STGGISVPGPM*GPSGPR.G	2	2.23	0.16	-3.81
IPI00297646	Collagen alpha-1(I) chain precursor	K.TSRLPIIDVAPLDVGAPDQEFGFDVGPVCFL.-	3	4.84	0.49	-4.15
IPI00297646	Collagen alpha-1(I) chain precursor	K.VFCNM*ETGETCVYPTQPSVAQK.N	2	3.95	0.40	-3.24
IPI00297646	Collagen alpha-1(I) chain precursor	K.VFCNM*ETGETCVYPTQPSVAQK.N	3	4.63	0.49	-2.58
IPI00297646	Collagen alpha-1(I) chain precursor	K.VLCDDVICDETK.N	2	4.33	0.47	-1.94
IPI00297646	Collagen alpha-1(I) chain precursor	R.DLEVDTTLK.S	1	2.15	0.21	-2.68
IPI00297646	Collagen alpha-1(I) chain precursor	R.DLEVDTTLK.S	2	2.59	0.09	-2.57
IPI00297646	Collagen alpha-1(I) chain precursor	R.DRDLEVDTTLK.S	2	3.28	0.21	-2.15
IPI00297646	Collagen alpha-1(I) chain precursor	R.GETGPAGPAGPVGPVGAR.G	2	4.24	0.41	-5.56
IPI00297646	Collagen alpha-1(I) chain precursor	R.GFSGLDGAKGDAGPAGPK.G	3	2.21	0.21	-1.90
IPI00297646	Collagen alpha-1(I) chain precursor	R.GPAGPQGPRGDKGETGEQGDR.G	3	4.04	0.30	-3.04
IPI00297646	Collagen alpha-1(I) chain precursor	R.GQAGVM*GFPGPK.G	2	2.29	0.13	
IPI00297646	Collagen alpha-1(I) chain precursor	R.ICVCDNGK.V	1	2.14	0.25	-2.33
IPI00297646	Collagen alpha-1(I) chain precursor	R.ICVCDNGK.V	2	2.37	0.19	-1.22
IPI00297646	Collagen alpha-1(I) chain precursor	R.YYRADDANVVR.D	3	3.40	0.26	-3.52
IPI00297655	Neurogenic locus notch homolog protein 2 precursor	R.DSQGELM*VYPYYGEK.S	2	3.49	0.25	
IPI00297655	Neurogenic locus notch homolog protein 2 precursor	R.DTYECTCQVGFTGK.E	2	4.07	0.38	-3.47
IPI00297655	Neurogenic locus notch homolog protein 2 precursor	R.SLPGEQEQEVAGSK.V	2	3.28	0.40	-4.45
IPI00297714	Gamma-synuclein	K.TVEEAENIAVTSGVVR.K	2	4.21	0.36	-1.98
IPI00297714	Gamma-synuclein	K.TVEEAENIAVTSGVVR.K	3	4.16	0.27	-2.94
IPI00297779	T-complex protein 1 subunit beta	R.MLPTIIADNAGYDSADLVAQLR.A	3	2.91	0.15	-2.16
IPI00297779	T-complex protein 1 subunit beta	R.QDLMNIAGTTLSSK.L	2	2.67	0.11	
IPI00298237	Isoform 1 of Tripeptidyl-peptidase 1 precursor	K.WLLAAGAQK.C	1	2.45	0.14	-2.16
IPI00298237	Isoform 1 of Tripeptidyl-peptidase 1 precursor	K.WLLAAGAQK.C	2	2.53	0.17	-2.22
IPI00298237	Isoform 1 of Tripeptidyl-peptidase 1 precursor	K.YLTLENVADLVR.P	2	4.09	0.45	-3.92
IPI00298237	Isoform 1 of Tripeptidyl-peptidase 1 precursor	K.YLTLENVADLVRPSPLTLHTVQK.W	2	4.39	0.58	-4.30
IPI00298237	Isoform 1 of Tripeptidyl-peptidase 1 precursor	K.YLTLENVADLVRPSPLTLHTVQK.W	3	5.35	0.37	-4.75
IPI00298237	Isoform 1 of Tripeptidyl-peptidase 1 precursor	K.YLTLENVADLVRPSPLTLHTVQK.W	4	3.39	0.29	-3.58
IPI00298237	Isoform 1 of Tripeptidyl-peptidase 1 precursor	R.ADPEEELSLTFALR.Q	2	3.96	0.39	-6.58
IPI00298237	Isoform 1 of Tripeptidyl-peptidase 1 precursor	R.AYPDVAALSDGYWVVSNR.V	2	4.82	0.50	-1.31
IPI00298237	Isoform 1 of Tripeptidyl-peptidase 1 precursor	R.FPPTSSLR.Q	2	2.16	0.08	0.19

IPI00298237	Isoform 1 of Tripeptidyl-peptidase 1 precursor	R.GCHESCLDEEVEGQGFCSGPGWDPVTGWGTPNFPALLK.T	3	5.20	0.54	-1.83
IPI00298237	Isoform 1 of Tripeptidyl-peptidase 1 precursor	R.GCHESCLDEEVEGQGFCSGPGWDPVTGWGTPNFPALLK.T	4	3.80	0.33	-1.60
IPI00298237	Isoform 1 of Tripeptidyl-peptidase 1 precursor	R.ILSGRPPLGFLNPR.L	2	2.82	0.16	-2.60
IPI00298237	Isoform 1 of Tripeptidyl-peptidase 1 precursor	R.ILSGRPPLGFLNPR.L	3	2.21	0.35	-2.86
IPI00298237	Isoform 1 of Tripeptidyl-peptidase 1 precursor	R.LFGGNFAHQASVAR.V	2	4.65	0.41	-3.11
IPI00298237	Isoform 1 of Tripeptidyl-peptidase 1 precursor	R.LSELVQAVSDPSSPQYGK.Y	2	5.46	0.58	-4.11
IPI00298237	Isoform 1 of Tripeptidyl-peptidase 1 precursor	R.LSELVQAVSDPSSPQYGK.Y	3	5.64	0.53	-2.56
IPI00298237	Isoform 1 of Tripeptidyl-peptidase 1 precursor	R.LYQQHGAGLFDVTR.G	2	4.21	0.46	-3.33
IPI00298237	Isoform 1 of Tripeptidyl-peptidase 1 precursor	R.LYQQHGAGLFDVTR.G	3	3.80	0.34	-3.07
IPI00298237	Isoform 1 of Tripeptidyl-peptidase 1 precursor	R.PSYQEEAVTK.F	2	3.30	0.41	-4.52
IPI00298237	Isoform 1 of Tripeptidyl-peptidase 1 precursor	R.QAELLLPGAEFHHYVGGPTETHVVR.S	3	3.17	0.29	-3.76
IPI00298237	Isoform 1 of Tripeptidyl-peptidase 1 precursor	R.QAELLLPGAEFHHYVGGPTETHVVR.S	4	4.12	0.33	0.67
IPI00298237	Isoform 1 of Tripeptidyl-peptidase 1 precursor	R.QRPEPQVTGTVGLHLGVTPSVIR.K	3	5.89	0.47	-2.88
IPI00298237	Isoform 1 of Tripeptidyl-peptidase 1 precursor	R.QRPEPQVTGTVGLHLGVTPSVIR.K	4	3.83	0.41	-2.86
IPI00298237	Isoform 1 of Tripeptidyl-peptidase 1 precursor	R.TLPPGWVSLGR.A	1	1.67	0.09	-3.22
IPI00298237	Isoform 1 of Tripeptidyl-peptidase 1 precursor	R.TLPPGWVSLGR.A	2	1.45	0.16	-1.59
IPI00298237	Isoform 1 of Tripeptidyl-peptidase 1 precursor	R.VNTELM*K.A	2	2.47	0.22	-3.14
IPI00298237	Isoform 1 of Tripeptidyl-peptidase 1 precursor	R.VPIPWVSGTSASTPVFGGILSLINEHR.I	3	4.79	0.54	-4.11
IPI00298258	UNC13B protein	K.GLIWDTM*VGTVWIALK.T	2	2.29	0.15	
IPI00298281	Laminin subunit gamma-1 precursor	K.AFDITYVR.L	1	1.96	0.23	-3.37
IPI00298281	Laminin subunit gamma-1 precursor	K.AFDITYVR.L	2	2.19	0.23	-2.11
IPI00298281	Laminin subunit gamma-1 precursor	K.DVDQNLM*DR.L	2	2.90	0.32	-5.19
IPI00298281	Laminin subunit gamma-1 precursor	K.GRDTLQEANDILNNLKDFDRR.V	4	3.23	0.27	-0.91
IPI00298281	Laminin subunit gamma-1 precursor	K.KGRDTLQEANDILNNLKDFDR.R	4	3.88	0.28	-3.45
IPI00298281	Laminin subunit gamma-1 precursor	K.LKDYEDLREDM*R.G	3	1.86	0.17	-2.35
IPI00298281	Laminin subunit gamma-1 precursor	K.QLQEAEKELKR.K	2	2.22	0.26	-3.08
IPI00298281	Laminin subunit gamma-1 precursor	K.SYYYAISDFAVGGR.C	2	3.95	0.49	-3.51
IPI00298281	Laminin subunit gamma-1 precursor	R.ATAESASECLPCDCNGR.S	2	4.81	0.59	-2.89
IPI00298281	Laminin subunit gamma-1 precursor	R.DTLQEANDILNNLKDFDRR.V	3	2.89	0.05	-3.41
IPI00298281	Laminin subunit gamma-1 precursor	R.EAQQALGSAAADATEAK.N	2	5.74	0.57	-2.48
IPI00298281	Laminin subunit gamma-1 precursor	R.EAQQALGSAAADATEAK.N	3	4.05	0.43	-1.64
IPI00298281	Laminin subunit gamma-1 precursor	R.KVSDLENEAK.K	2	2.61	0.09	1.83
IPI00298281	Laminin subunit gamma-1 precursor	R.LSAEDLVLEGAGLR.V	2	4.19	0.36	-3.07
IPI00298281	Laminin subunit gamma-1 precursor	R.LSAEDLVLEGAGLR.V	3	3.81	0.13	-1.32
IPI00298281	Laminin subunit gamma-1 precursor	R.NTIEETGNLAEQAR.A	2	4.69	0.38	-4.14
IPI00298281	Laminin subunit gamma-1 precursor	R.SAGYLDDVTLASAR.P	2	4.81	0.44	-4.92
IPI00298281	Laminin subunit gamma-1 precursor	R.VKLQELESLIANLGTGDEM*VTDQAFEDR.L	3	6.60	0.47	-3.53
IPI00298281	Laminin subunit gamma-1 precursor	R.VSVPLIAQGNSYPSETTVK.Y	2	4.30	0.56	-4.47
IPI00298281	Laminin subunit gamma-1 precursor	R.YFIAPAK.F	2	1.72	0.10	-3.72
IPI00298285	Isoform 1 of Receptor tyrosine-protein kinase erbB-3 precursor	R.IYISANR.Q	2	2.09	0.06	-0.96
IPI00298337	cDNA FLJ77671	R.VDSPTMVRGENQVSPCQGRR.C	2	1.69	0.11	-2.53

IPI00298388	Isoform 1 of Phosphoinositide-3-kinase-interacting protein 1 precursor	E.VQVFAPANALPAR.S	2	3.16	0.32	-0.90
IPI00298388	Isoform 1 of Phosphoinositide-3-kinase-interacting protein 1 precursor	G.SGGCFWDNGHLYREDQTSPAPGLR.C	3	5.01	0.51	-3.33
IPI00298388	Isoform 1 of Phosphoinositide-3-kinase-interacting protein 1 precursor	P.ARSEAAAVQPVIGISQR.V	2	5.92	0.45	-3.85
IPI00298388	Isoform 1 of Phosphoinositide-3-kinase-interacting protein 1 precursor	R.EDQTSPAPGLR.C	1	2.06	0.26	-5.36
IPI00298388	Isoform 1 of Phosphoinositide-3-kinase-interacting protein 1 precursor	R.EDQTSPAPGLR.C	2	3.45	0.31	-3.41
IPI00298388	Isoform 1 of Phosphoinositide-3-kinase-interacting protein 1 precursor	R.GPWCYVSGEAGVPEK.R	2	2.93	0.30	-2.54
IPI00298388	Isoform 1 of Phosphoinositide-3-kinase-interacting protein 1 precursor	R.NPDEDPRGPWCYVSGEAGVPEK.R	2	2.27	0.19	-3.22
IPI00298388	Isoform 1 of Phosphoinositide-3-kinase-interacting protein 1 precursor	R.NPDEDPRGPWCYVSGEAGVPEK.R	3	3.39	0.40	-3.23
IPI00298388	Isoform 1 of Phosphoinositide-3-kinase-interacting protein 1 precursor	R.NPDEDPRGPWCYVSGEAGVPEKRPCEDLR.C	4	3.00	0.32	-3.76
IPI00298388	Isoform 1 of Phosphoinositide-3-kinase-interacting protein 1 precursor	R.SEAAAVQPVIGI.S	2	3.07	0.28	-1.43
IPI00298388	Isoform 1 of Phosphoinositide-3-kinase-interacting protein 1 precursor	R.SEAAAVQPVIGISQ.R	2	3.42	0.49	-3.67
IPI00298388	Isoform 1 of Phosphoinositide-3-kinase-interacting protein 1 precursor	R.SEAAAVQPVIGISQR.V	2	4.84	0.48	-3.12
IPI00298388	Isoform 1 of Phosphoinositide-3-kinase-interacting protein 1 precursor	R.SEAAAVQPVIGISQR.V	3	4.67	0.32	-2.11
IPI00298388	Isoform 1 of Phosphoinositide-3-kinase-interacting protein 1 precursor	W.DNGHLYREDQTSPAPGLR.C	2	4.20	0.41	-6.02
IPI00298388	Isoform 1 of Phosphoinositide-3-kinase-interacting protein 1 precursor	W.DNGHLYREDQTSPAPGLR.C	3	4.90	0.50	-2.77
IPI00298476	Isoform 1 of Gremlin-1 precursor	K.AQHNDSEQTQSPQQPGSR.N	3	3.08	0.26	-2.75
IPI00298476	Isoform 1 of Gremlin-1 precursor	K.GSQGAIPPPDKAQHNDSEQTQSPQQPGSR.N	4	2.70	0.16	-5.04
IPI00298497	Fibrinogen beta chain precursor	K.AHYGGFTVQNEANKYQISVNK.Y	2	6.10	0.36	
IPI00298497	Fibrinogen beta chain precursor	K.AHYGGFTVQNEANKYQISVNK.Y	3	4.79	0.55	
IPI00298497	Fibrinogen beta chain precursor	K.DNENVVNEYSSELEK.H	2	4.92	0.34	
IPI00298497	Fibrinogen beta chain precursor	K.DNENVVNEYSSELEKHQLYIDETVNSNIPTNLR.V	3	5.97	0.43	
IPI00298497	Fibrinogen beta chain precursor	K.EDGGGWWYNR.C	2	3.04	0.31	
IPI00298497	Fibrinogen beta chain precursor	K.GGETSEM*YLIQPDSSVKPYR.V	3	4.32	0.35	
IPI00298497	Fibrinogen beta chain precursor	K.HGTDDGVVWM*NWK.G	2	3.78	0.42	
IPI00298497	Fibrinogen beta chain precursor	K.HQLYIDETVNSNIPTNLR.V	2	5.48	0.47	
IPI00298497	Fibrinogen beta chain precursor	K.IQKLESDVSAQM*EYCR.T	2	5.23	0.46	
IPI00298497	Fibrinogen beta chain precursor	K.IQKLESDVSAQM*EYCR.T	3	3.40	0.18	

IPI00298497	Fibrinogen beta chain precursor	K.LESDVSAQM*EYCR.T	2	4.50	0.49	
IPI00298497	Fibrinogen beta chain precursor	K.LESDVSAQMEYCR.T	2	4.69	0.42	
IPI00298497	Fibrinogen beta chain precursor	K.NYCGLPGEYWLGNDK.I	2	3.79	0.28	
IPI00298497	Fibrinogen beta chain precursor	K.NYCGLPGEYWLGNDKISQLTR.M	2	3.89	0.43	
IPI00298497	Fibrinogen beta chain precursor	K.QCSKEDGGGWWYNR.C	2	2.45	0.27	
IPI00298497	Fibrinogen beta chain precursor	K.QGFGNVATNTDGK.N	2	3.62	0.37	
IPI00298497	Fibrinogen beta chain precursor	K.QGFGNVATNTDGKNYCGLPGEYWLGNDK.I	3	3.86	0.31	
IPI00298497	Fibrinogen beta chain precursor	K.QVKDNENVVNEYSSELEKHQLYIDETVNSNIPTNLR.V	3	4.37	0.30	
IPI00298497	Fibrinogen beta chain precursor	K.REEAPSLRPAPPPISGGGYR.A	3	4.00	0.36	
IPI00298497	Fibrinogen beta chain precursor	K.YQISVNK.Y	1	2.25	0.12	
IPI00298497	Fibrinogen beta chain precursor	R.EEAPSLRPAPPPISGGGYR.A	3	2.67	0.24	
IPI00298497	Fibrinogen beta chain precursor	R.KAPDAGGCLHADPDLGVLCPTGCQLQEALLQQERPIR.N	3	4.59	0.41	
IPI00298497	Fibrinogen beta chain precursor	R.KGGETSEM*YLIQPDSSVKPYR.V	3	5.48	0.27	
IPI00298497	Fibrinogen beta chain precursor	R.KWDPYKQGFGNVATNTDGK.N	3	3.33	0.18	
IPI00298497	Fibrinogen beta chain precursor	R.M*GPTELLIEMEDWK.G	2	4.40	0.37	
IPI00298497	Fibrinogen beta chain precursor	R.NSVDELNNNVEAVSQTSSSSFQYM*YLLK.D	3	5.98	0.43	
IPI00298497	Fibrinogen beta chain precursor	R.NSVDELNNNVEAVSQTSSSSFQYMYLLK.D	3	5.43	0.42	
IPI00298497	Fibrinogen beta chain precursor	R.QDGSVDFGR.K	2	2.09	0.21	
IPI00298497	Fibrinogen beta chain precursor	R.SKIQKLESDVSAQM*EYCR.T	2	5.95	0.42	
IPI00298497	Fibrinogen beta chain precursor	R.SKIQKLESDVSAQM*EYCR.T	3	5.12	0.32	
IPI00298497	Fibrinogen beta chain precursor	R.TPCTVSCNIPVVSGKECEEIIR.K	2	4.91	0.50	
IPI00298497	Fibrinogen beta chain precursor	R.TPCTVSCNIPVVSGKECEEIIR.K	3	4.48	0.33	
IPI00298497	Fibrinogen beta chain precursor	R.TPCTVSCNIPVVSGKECEEIIRK.G	3	4.01	0.42	
IPI00298497	Fibrinogen beta chain precursor	R.VYCDM*NTENGGWTVIQNR.Q	2	5.45	0.35	
IPI00298497	Fibrinogen beta chain precursor	R.VYCDM*NTENGGWTVIQNR.Q	3	4.45	0.22	
IPI00298497	Fibrinogen beta chain precursor	R.VYCDMNTENGGWTVIQNR.Q	2	5.15	0.37	
IPI00298547	Protein DJ-1	K.APLVLKD.-	1	1.66	0.10	-1.11
IPI00298547	Protein DJ-1	K.DGLILTSR.G	2	2.67	0.15	-3.45
IPI00298547	Protein DJ-1	K.EGPYDVVVLPGGNLGAQNLSESAAVK.E	3	3.59	0.30	-5.28
IPI00298547	Protein DJ-1	K.EGPYDVVVLPGGNLGAQNLSESAAVKEILK.E	3	3.33	0.12	-3.78
IPI00298547	Protein DJ-1	K.EILKEQENR.K	2	2.35	0.07	-2.97
IPI00298547	Protein DJ-1	K.GAEEM*ETVIPVDVM*R.R	2	3.68	0.39	-4.68
IPI00298547	Protein DJ-1	K.GAEEM*ETVIPVDVM*R.R	3	2.59	0.12	-2.71
IPI00298547	Protein DJ-1	K.VTTHPLAK.D	1	2.10	0.11	-3.07
IPI00298547	Protein DJ-1	R.DVVICPDASLEDAKKEGPYDVVVLPGGNLGAQNLSESAAVK.E	4	4.92	0.30	-4.29
IPI00298547	Protein DJ-1	R.GPGTSFEFALAIVEALNGK.E	2	6.23	0.58	-5.35
IPI00298547	Protein DJ-1	R.GPGTSFEFALAIVEALNGK.E	3	3.59	0.32	-3.22
IPI00298547	Protein DJ-1	R.GPGTSFEFALAIVEALNGKEVAAQVK.A	3	1.99	0.12	-0.07
IPI00298650	ADAMTS-8 precursor	R.GLSGSFLLDGEEFTIQPQGAGGSLAQPHR.L	3	5.40	0.49	-0.36
IPI00298650	ADAMTS-8 precursor	R.KVSGSLTPTNYGYNDIVTIPAGATNIDVK.Q	3	3.59	0.27	-3.35
IPI00298650	ADAMTS-8 precursor	R.LQSFRPLPEPLTVQLLTVPGEVFPPK.V	3	3.05	0.39	-4.00

IPI00298702	solute carrier family 39 (zinc transporter), member 6 isoform 1	R.NVKDSVSASEVTSTVYNTVSEGTHFLETIETPRPGK.L	4	3.23	0.26	-4.56
IPI00298738	DNA-directed RNA polymerase, mitochondrial precursor	R.GRTYPCPPHFNHLGSDVAR.A	4	2.24	0.14	-5.19
IPI00298793	Beta-mannosidase precursor	K.GGEAVCLYEEPVSELLRR.C	3	2.36	0.13	-2.99
IPI00298793	Beta-mannosidase precursor	K.GSPGLSFYFK.I	2	2.12	0.27	-1.61
IPI00298793	Beta-mannosidase precursor	K.LPQSTDPLR.T	2	2.98	0.24	-3.31
IPI00298793	Beta-mannosidase precursor	K.LQTQQTYSIELQPGKR.I	2	4.27	0.42	-4.63
IPI00298793	Beta-mannosidase precursor	K.QM*LYQAGLHFK.L	3	2.03	0.14	-4.12
IPI00298793	Beta-mannosidase precursor	K.VNLILEGVDTVSK.I	2	5.20	0.43	-4.46
IPI00298793	Beta-mannosidase precursor	R.DVNSIELR.F	2	3.03	0.17	-2.03
IPI00298793	Beta-mannosidase precursor	R.FASEYGYQSWPSFSTLEK.V	2	3.78	0.35	-4.77
IPI00298793	Beta-mannosidase precursor	R.FNDLNYR.W	1	2.02	0.10	-2.08
IPI00298793	Beta-mannosidase precursor	R.FNDLNYR.W	2	2.70	0.14	-2.25
IPI00298793	Beta-mannosidase precursor	R.FQSAVLYAAQQSK.A	2	4.49	0.40	-3.56
IPI00298793	Beta-mannosidase precursor	R.IEAYNICHLNYFTFSPIYDK.S	3	3.94	0.22	-0.57
IPI00298793	Beta-mannosidase precursor	R.TILFYPWEPTSK.N	2	3.43	0.34	-3.82
IPI00298793	Beta-mannosidase precursor	R.TILFYPWEPTSKNELEQSFHVTSLTDIY.-	3	2.49	0.05	-2.61
IPI00298793	Beta-mannosidase precursor	R.TVELIEEPIKGSPGLSFYFK.I	3	3.96	0.45	-6.07
IPI00298793	Beta-mannosidase precursor	R.YSFDITNVVR.D	2	3.77	0.39	-3.61
IPI00298793	Beta-mannosidase precursor	R.YSFDITNVVRDVNSIELR.F	3	4.02	0.41	-1.95
IPI00298828	Beta-2-glycoprotein 1 precursor	A.GRTCPKPDDLPFSTVVPLK.T	3	4.40	0.31	-3.97
IPI00298828	Beta-2-glycoprotein 1 precursor	C.PFAGILENGAVR.Y	2	3.95	0.33	-3.27
IPI00298828	Beta-2-glycoprotein 1 precursor	C.PFPSRPDNGFVNYPAKPTLYYK.D	3	3.88	0.17	-3.79
IPI00298828	Beta-2-glycoprotein 1 precursor	K.ATFGCHDGYSLDGPEEIECTK.L	2	4.88	0.54	-4.05
IPI00298828	Beta-2-glycoprotein 1 precursor	K.ATFGCHDGYSLDGPEEIECTK.L	3	5.17	0.40	-4.43
IPI00298828	Beta-2-glycoprotein 1 precursor	K.ATVVYQGER.V	1	2.18	0.24	-3.28
IPI00298828	Beta-2-glycoprotein 1 precursor	K.ATVVYQGER.V	2	3.16	0.38	-2.13
IPI00298828	Beta-2-glycoprotein 1 precursor	K.CPFPSRPDNGFVNYPAKPTLYYK.D	3	5.39	0.43	-3.92
IPI00298828	Beta-2-glycoprotein 1 precursor	K.CPFPSRPDNGFVNYPAKPTLYYKDK.A	3	5.74	0.41	
IPI00298828	Beta-2-glycoprotein 1 precursor	K.CSYTEDAQCIDGTIEVPK.C	2	6.29	0.57	-4.66
IPI00298828	Beta-2-glycoprotein 1 precursor	K.CSYTEDAQCIDGTIEVPK.C	3	5.66	0.48	-4.68
IPI00298828	Beta-2-glycoprotein 1 precursor	K.CSYTEDAQCIDGTIEVPKCFK.E	3	2.59	0.24	
IPI00298828	Beta-2-glycoprotein 1 precursor	K.CTEEGKWSPELPVCAPIICPPPSIPTFATLR.V	2	2.07	0.45	-3.51
IPI00298828	Beta-2-glycoprotein 1 precursor	K.CTEEGKWSPELPVCAPIICPPPSIPTFATLR.V	3	6.36	0.57	-4.90
IPI00298828	Beta-2-glycoprotein 1 precursor	K.CTEEGKWSPELPVCAPIICPPPSIPTFATLR.V	4	3.24	0.31	-3.99
IPI00298828	Beta-2-glycoprotein 1 precursor	K.DKATFGCHDGYSLDGPEEIECTK.L	3	5.82	0.36	
IPI00298828	Beta-2-glycoprotein 1 precursor	K.FICPLTGLWPINTLK.C	2	4.98	0.49	-5.50
IPI00298828	Beta-2-glycoprotein 1 precursor	K.FICPLTGLWPINTLK.C	3	3.69	0.21	-2.90
IPI00298828	Beta-2-glycoprotein 1 precursor	K.KATVVYQGER.V	1	2.74	0.31	-4.39
IPI00298828	Beta-2-glycoprotein 1 precursor	K.KATVVYQGER.V	2	3.46	0.37	-3.65
IPI00298828	Beta-2-glycoprotein 1 precursor	K.KCSYTEDAQCIDGTIEVPK.C	2	5.04	0.56	-3.78

IPI00298828	Beta-2-glycoprotein 1 precursor	K.KCSYTEDAQCIDGTIEVPK.C	3	3.63	0.31	-2.72
IPI00298828	Beta-2-glycoprotein 1 precursor	K.NGM*LHGDKVSFFCK.N	2	3.74	0.42	
IPI00298828	Beta-2-glycoprotein 1 precursor	K.NGM*LHGDKVSFFCK.N	3	2.68	0.25	
IPI00298828	Beta-2-glycoprotein 1 precursor	K.TFYEPGEEIT.Y	1	2.27	0.23	-2.13
IPI00298828	Beta-2-glycoprotein 1 precursor	K.TFYEPGEEITYSCKPGYVSR.G	2	5.11	0.55	-2.76
IPI00298828	Beta-2-glycoprotein 1 precursor	K.TFYEPGEEITYSCKPGYVSR.G	3	2.31	0.20	
IPI00298828	Beta-2-glycoprotein 1 precursor	K.WSPELPVCAPIICPPPSIPTFATLR.V	2	3.89	0.56	-4.70
IPI00298828	Beta-2-glycoprotein 1 precursor	K.WSPELPVCAPIICPPPSIPTFATLR.V	3	4.91	0.51	-5.35
IPI00298828	Beta-2-glycoprotein 1 precursor	P.FPSRPDNGFVNYPAKPTLYYKDK.A	3	4.96	0.45	-1.09
IPI00298828	Beta-2-glycoprotein 1 precursor	R.KFICPLTGLWPINTLK.C	2	4.19	0.45	-4.37
IPI00298828	Beta-2-glycoprotein 1 precursor	R.KFICPLTGLWPINTLK.C	3	3.01	0.23	-5.46
IPI00298828	Beta-2-glycoprotein 1 precursor	R.TCPKPDDLPFSTVVPLK.T	2	3.68	0.24	
IPI00298828	Beta-2-glycoprotein 1 precursor	R.TCPKPDDLPFSTVVPLK.T	3	3.20	0.26	-4.30
IPI00298828	Beta-2-glycoprotein 1 precursor	R.TCPKPDDLPFSTVVPLKTFYEPGEEITYSCKPGYVSR.G	4	3.57	0.15	-4.63
IPI00298828	Beta-2-glycoprotein 1 precursor	R.VCPFAGILENGAVR.Y	1	3.21	0.32	-1.79
IPI00298828	Beta-2-glycoprotein 1 precursor	R.VCPFAGILENGAVR.Y	2	4.60	0.38	-6.17
IPI00298828	Beta-2-glycoprotein 1 precursor	R.VCPFAGILENGAVR.Y	3	4.91	0.32	-2.26
IPI00298828	Beta-2-glycoprotein 1 precursor	R.YTTFEYPNTISFSCNTGFYLNGADSAK.C	2	4.10	0.53	-3.00
IPI00298828	Beta-2-glycoprotein 1 precursor	R.YTTFEYPNTISFSCNTGFYLNGADSAK.C	3	4.81	0.53	-5.56
IPI00298828	Beta-2-glycoprotein 1 precursor	W.SPELPVCAPIICPPPSIPTFATLR.V	2	4.23	0.56	-2.35
IPI00298971	Vitronectin precursor	I.YISGM*APRPSLAK.K	2	3.04	0.33	-1.10
IPI00298971	Vitronectin precursor	K.AVRPGYPK.L	2	2.36	0.09	-3.84
IPI00298971	Vitronectin precursor	K.CQCDELCSYYQSCCTDYTAECKPQVTR.G	3	5.55	0.69	-3.02
IPI00298971	Vitronectin precursor	K.LIRDVWGIEGPIDAAFTR.I	2	4.08	0.53	-4.12
IPI00298971	Vitronectin precursor	K.LIRDVWGIEGPIDAAFTR.I	3	5.12	0.38	-2.96
IPI00298971	Vitronectin precursor	R.CTEGFNVDK.K	2	2.29	0.20	-0.60
IPI00298971	Vitronectin precursor	R.CTEGFNVDKK.C	2	2.56	0.10	-0.73
IPI00298971	Vitronectin precursor	R.CTEGFNVDKK.C	3	3.44	0.10	
IPI00298971	Vitronectin precursor	R.DVWGIEGPIDAAFTR.I	1	1.28	0.26	-5.04
IPI00298971	Vitronectin precursor	R.DVWGIEGPIDAAFTR.I	2	4.74	0.47	-5.58
IPI00298971	Vitronectin precursor	R.DVWGIEGPIDAAFTR.I	3	4.35	0.41	-2.28
IPI00298971	Vitronectin precursor	R.DWHGVPGQVDAAM*AGR.I	2	3.81	0.46	-3.30
IPI00298971	Vitronectin precursor	R.DWHGVPGQVDAAM*AGR.I	3	5.40	0.38	-2.26
IPI00298971	Vitronectin precursor	R.DWHGVPGQVDAAMAGR.I	3	4.19	0.25	
IPI00298971	Vitronectin precursor	R.FEDGVLDPDYPR.N	1	2.44	0.33	-4.04
IPI00298971	Vitronectin precursor	R.FEDGVLDPDYPR.N	2	4.65	0.35	-5.06
IPI00298971	Vitronectin precursor	R.GQYCYELDEK.A	1	2.65	0.14	-3.25
IPI00298971	Vitronectin precursor	R.GQYCYELDEK.A	2	3.37	0.46	-3.37
IPI00298971	Vitronectin precursor	R.IYISGM*APR.P	2	3.13	0.23	-0.45
IPI00298971	Vitronectin precursor	R.IYISGM*APRPSLAK.K	2	4.02	0.38	-3.22
IPI00298971	Vitronectin precursor	R.M*DWLVPATCEPIQSVFFFSGDK.Y	2	4.04	0.47	1.48
IPI00298971	Vitronectin precursor	R.M*DWLVPATCEPIQSVFFFSGDKYYR.V	3	2.38	0.31	-2.99

IPI00298971	Vitronectin precursor	R.RVDTVDPPYPR.S	1	1.72	0.25	-4.60
IPI00298971	Vitronectin precursor	R.RVDTVDPPYPR.S	3	2.64	0.20	-3.59
IPI00298971	Vitronectin precursor	R.SIAQYWLGCPAPGH.L	2	3.53	0.45	-4.32
IPI00298971	Vitronectin precursor	R.SIAQYWLGCPAPGHL.-	2	4.55	0.51	-4.58
IPI00298971	Vitronectin precursor	R.SIAQYWLGCPAPGHL.-	3	4.47	0.29	-3.92
IPI00298971	Vitronectin precursor	R.TSAGTRQPQFISR.D	2	3.52	0.25	-4.19
IPI00298971	Vitronectin precursor	R.TSAGTRQPQFISR.D	3	2.49	0.34	-2.51
IPI00298971	Vitronectin precursor	R.VDTVDPPYPR.S	2	2.70	0.18	-1.96
IPI00298971	Vitronectin precursor	W.GIEGPIDAAFTR.I	1	2.13	0.34	-3.82
IPI00298971	Vitronectin precursor	W.GIEGPIDAAFTR.I	2	3.69	0.44	-4.27
IPI00298994	Talin-1	D.PEDPTVIAENELLGAAAAIEAAAK.K	3	4.56	0.44	-3.32
IPI00298994	Talin-1	K.AAAFEEQENETVVVK.E	2	5.17	0.36	-3.21
IPI00298994	Talin-1	K.AIAVTVQEMVTK.S	2	3.20	0.44	-3.61
IPI00298994	Talin-1	K.AVASAAAALVLK.A	2	3.33	0.32	-2.86
IPI00298994	Talin-1	K.EADESLNFEEQILEAAK.S	2	5.35	0.43	-4.24
IPI00298994	Talin-1	K.LAQAAQSSVATITR.L	2	4.54	0.45	-4.51
IPI00298994	Talin-1	K.PAAVAAENEEIGSHIK.H	2	4.23	0.52	-3.38
IPI00298994	Talin-1	K.PAAVAAENEEIGSHIK.H	3	5.26	0.54	-2.17
IPI00298994	Talin-1	K.VLVQNAAGSQEK.L	2	2.83	0.06	-3.82
IPI00298994	Talin-1	R.FGQDFSTFLEAGVEMAGQAPSQEDR.A	2	4.44	0.47	-3.76
IPI00298994	Talin-1	R.FGQDFSTFLEAGVEMAGQAPSQEDR.A	3	4.33	0.36	-4.20
IPI00298994	Talin-1	R.GVAALTSDPAVQAIVLDTASDVLDKASSLIEEAKK.A	4	3.02	0.11	-3.47
IPI00299024	Brain acid soluble protein 1	A.EGAATEEEGTPKESEPQAAAEPAEAK.E	3	5.40	0.44	-4.42
IPI00299024	Brain acid soluble protein 1	E.GAATEEEGTPKESEPQAAAEPAEAK.E	3	4.88	0.48	-1.57
IPI00299024	Brain acid soluble protein 1	K.AAEAAAAPAESAAPAAGEEPSKEEGEPK.K	2	4.56	0.48	-2.84
IPI00299024	Brain acid soluble protein 1	K.AEGAATEEEGTPKESEPQAAAEPAEAK.E	3	5.31	0.52	-3.94
IPI00299024	Brain acid soluble protein 1	K.AEPPKAPEQEQAAPGPAAGGEAPK.A	2	3.95	0.47	-3.06
IPI00299024	Brain acid soluble protein 1	K.AEPPKAPEQEQAAPGPAAGGEAPK.A	3	4.29	0.36	-3.15
IPI00299024	Brain acid soluble protein 1	K.AEPPKAPEQEQAAPGPAAGGEAPK.A	4	2.82	0.21	-1.95
IPI00299024	Brain acid soluble protein 1	K.AEPPKAPEQEQAAPGPAAGGEAPKA.A	3	4.79	0.47	-2.36
IPI00299024	Brain acid soluble protein 1	K.AQGPAASAEEPKP.V	2	3.37	0.44	-1.85
IPI00299024	Brain acid soluble protein 1	K.AQGPAASAEEPKPVEAPAANSDQTVTVKE.-	3	6.64	0.54	-3.62
IPI00299024	Brain acid soluble protein 1	K.DKKAEGAATEEEGTPKESEPQAAAEPAEAK.E	4	3.88	0.35	-3.80
IPI00299024	Brain acid soluble protein 1	K.EEAPKAEPEKTEGAAEAK.A	3	2.93	0.18	-3.31
IPI00299024	Brain acid soluble protein 1	K.ESEPQAAAEPAEAK.E	2	3.56	0.32	-3.84
IPI00299024	Brain acid soluble protein 1	K.ETPAATEAPSSTPK.A	1	1.85	0.15	-1.33
IPI00299024	Brain acid soluble protein 1	K.ETPAATEAPSSTPK.A	2	3.63	0.48	-3.76
IPI00299024	Brain acid soluble protein 1	K.ETPAATEAPSSTPKA.Q	2	3.59	0.54	-4.17
IPI00299024	Brain acid soluble protein 1	K.KTEAPAAPAAQETK.S	2	4.66	0.38	-4.22
IPI00299024	Brain acid soluble protein 1	K.KTEAPAAPAAQETK.S	3	3.09	0.24	-3.02
IPI00299024	Brain acid soluble protein 1	K.SDGAPASDSKPGSSEAAPSSK.E	2	4.54	0.52	-4.68
IPI00299024	Brain acid soluble protein 1	K.SDGAPASDSKPGSSEAAPSSK.E	3	3.59	0.31	-3.62

IPI00299024	Brain acid soluble protein 1	K.SDGAPASDSKPGSSEAAPSSKETPAATEAPSSTPK.A	4	3.37	0.28	-2.14
IPI00299024	Brain acid soluble protein 1	K.TEAPAAPAAQETK.S	2	3.22	0.42	-3.41
IPI00299059	Isoform 2 of Neural cell adhesion molecule L1-like protein precursor	A.IEIPSSVQQVPTIIK.Q	1	2.58	0.32	-3.22
IPI00299059	Isoform 2 of Neural cell adhesion molecule L1-like protein precursor	A.IEIPSSVQQVPTIIK.Q	2	4.57	0.41	-3.82
IPI00299059	Isoform 2 of Neural cell adhesion molecule L1-like protein precursor	A.IEIPSSVQQVPTIIK.Q	3	5.36	0.35	-3.07
IPI00299059	Isoform 2 of Neural cell adhesion molecule L1-like protein precursor	C.TASNFLGTATHDFHVIVEEPPR.W	3	4.78	0.49	-6.62
IPI00299059	Isoform 2 of Neural cell adhesion molecule L1-like protein precursor	F.AGDVVFPR.E	2	3.30	0.23	-1.47
IPI00299059	Isoform 2 of Neural cell adhesion molecule L1-like protein precursor	I.ENVSYQDKGNYR.C	2	3.08	0.35	-3.51
IPI00299059	Isoform 2 of Neural cell adhesion molecule L1-like protein precursor	I.PSSVQQVPTIIK.Q	2	3.78	0.29	-2.47
IPI00299059	Isoform 2 of Neural cell adhesion molecule L1-like protein precursor	K.DGEAFEINGTEDGR.I	2	5.08	0.51	-3.06
IPI00299059	Isoform 2 of Neural cell adhesion molecule L1-like protein precursor	K.DGENYATVVGYSAFLHCEFFASPEAVVSWQK.V	3	5.81	0.53	-3.43
IPI00299059	Isoform 2 of Neural cell adhesion molecule L1-like protein precursor	K.DGNPFYFTDHR.I	1	2.37	0.22	-1.95
IPI00299059	Isoform 2 of Neural cell adhesion molecule L1-like protein precursor	K.DGNPFYFTDHR.I	2	3.28	0.40	-3.15
IPI00299059	Isoform 2 of Neural cell adhesion molecule L1-like protein precursor	K.DGNPFYFTDHR.I	3	1.71	0.11	-0.62
IPI00299059	Isoform 2 of Neural cell adhesion molecule L1-like protein precursor	K.DSRNDYCCFAAFPR.L	2	3.30	0.29	-1.24
IPI00299059	Isoform 2 of Neural cell adhesion molecule L1-like protein precursor	K.DSRNDYCCFAAFPR.L	3	3.52	0.43	-2.05
IPI00299059	Isoform 2 of Neural cell adhesion molecule L1-like protein precursor	K.EKIDPLEVEEGD.P	2	3.66	0.30	-2.92
IPI00299059	Isoform 2 of Neural cell adhesion molecule L1-like protein precursor	K.EKIDPLEVEEGDPIVLPCNPPK.G	2	4.85	0.42	-3.47
IPI00299059	Isoform 2 of Neural cell adhesion molecule L1-like protein precursor	K.EKIDPLEVEEGDPIVLPCNPPK.G	3	5.69	0.45	-3.96
IPI00299059	Isoform 2 of Neural cell adhesion molecule L1-like protein precursor	K.EKIDPLEVEEGDPIVLPCNPPK.G	4	3.40	0.26	-2.07
IPI00299059	Isoform 2 of Neural cell adhesion molecule L1-like protein precursor	K.EM*IIKWEPLK.S	2	3.10	0.21	-2.37
IPI00299059	Isoform 2 of Neural cell adhesion molecule L1-like protein precursor	K.EM*IIKWEPLK.S	3	2.93	0.18	-1.91

IPI00299059	Isoform 2 of Neural cell adhesion molecule L1-like protein precursor	K.EM*IIKWEPLKSM*EQNGPGLEYR.V	4	2.71	0.12	-2.75
IPI00299059	Isoform 2 of Neural cell adhesion molecule L1-like protein precursor	K.GAGPESEPYIFQTPEGVPEQPTFLK.V	2	5.03	0.56	-5.30
IPI00299059	Isoform 2 of Neural cell adhesion molecule L1-like protein precursor	K.GAGPESEPYIFQTPEGVPEQPTFLK.V	3	5.61	0.46	-5.25
IPI00299059	Isoform 2 of Neural cell adhesion molecule L1-like protein precursor	K.GDLYFANVEEK.D	1	2.42	0.39	-3.98
IPI00299059	Isoform 2 of Neural cell adhesion molecule L1-like protein precursor	K.GDLYFANVEEK.D	2	3.87	0.37	-4.03
IPI00299059	Isoform 2 of Neural cell adhesion molecule L1-like protein precursor	K.GDLYFANVEEKDS.R	2	3.05	0.29	-0.73
IPI00299059	Isoform 2 of Neural cell adhesion molecule L1-like protein precursor	K.GDLYFANVEEKDSR.N	2	4.06	0.44	-2.35
IPI00299059	Isoform 2 of Neural cell adhesion molecule L1-like protein precursor	K.GDLYFANVEEKDSR.N	3	3.21	0.37	-1.02
IPI00299059	Isoform 2 of Neural cell adhesion molecule L1-like protein precursor	K.GDLYFANVEEKDSRNDYCCFAAFPR.L	3	3.20	0.20	-0.61
IPI00299059	Isoform 2 of Neural cell adhesion molecule L1-like protein precursor	K.GEILLLECFAEGLPTPQVDWNK.I	2	5.02	0.51	-6.79
IPI00299059	Isoform 2 of Neural cell adhesion molecule L1-like protein precursor	K.GEILLLECFAEGLPTPQVDWNK.I	3	3.49	0.25	-5.04
IPI00299059	Isoform 2 of Neural cell adhesion molecule L1-like protein precursor	K.GLPPLHIYWM*NIELEHIEQDER.V	3	3.83	0.39	-3.33
IPI00299059	Isoform 2 of Neural cell adhesion molecule L1-like protein precursor	K.GNPEPTFSWTK.D	1	2.14	0.28	-3.00
IPI00299059	Isoform 2 of Neural cell adhesion molecule L1-like protein precursor	K.GNPEPTFSWTK.D	2	2.14	0.32	-3.82
IPI00299059	Isoform 2 of Neural cell adhesion molecule L1-like protein precursor	K.GYQINWWK.T	2	2.65	0.25	-1.55
IPI00299059	Isoform 2 of Neural cell adhesion molecule L1-like protein precursor	K.IDPLEVEEGDPIVLPCNPPK.G	2	5.17	0.46	-5.35
IPI00299059	Isoform 2 of Neural cell adhesion molecule L1-like protein precursor	K.IDPLEVEEGDPIVLPCNPPK.G	3	5.09	0.37	-5.38
IPI00299059	Isoform 2 of Neural cell adhesion molecule L1-like protein precursor	K.IENVSYQDK.G	1	2.64	0.27	-3.25
IPI00299059	Isoform 2 of Neural cell adhesion molecule L1-like protein precursor	K.IENVSYQDK.G	2	3.04	0.20	-6.51
IPI00299059	Isoform 2 of Neural cell adhesion molecule L1-like protein precursor	K.IENVSYQDKGNYR.C	2	4.39	0.52	-3.17
IPI00299059	Isoform 2 of Neural cell adhesion molecule L1-like protein precursor	K.IENVSYQDKGNYR.C	3	2.29	0.16	-2.12

IPI00299059	Isoform 2 of Neural cell adhesion molecule L1-like protein precursor	K.IGGDLPK.G	1	2.06	0.12	-2.35
IPI00299059	Isoform 2 of Neural cell adhesion molecule L1-like protein precursor	K.ISGVNLTQK.T	1	2.19	0.11	-3.21
IPI00299059	Isoform 2 of Neural cell adhesion molecule L1-like protein precursor	K.ISGVNLTQK.T	2	3.03	0.30	-2.32
IPI00299059	Isoform 2 of Neural cell adhesion molecule L1-like protein precursor	K.KPQSAVYSTGSNGILLCEAEGEPQPTIK.W	3	3.36	0.37	-4.13
IPI00299059	Isoform 2 of Neural cell adhesion molecule L1-like protein precursor	K.KTTVILPLAPFVR.Y	1	3.41	0.42	-3.83
IPI00299059	Isoform 2 of Neural cell adhesion molecule L1-like protein precursor	K.KTTVILPLAPFVR.Y	2	3.92	0.36	-5.09
IPI00299059	Isoform 2 of Neural cell adhesion molecule L1-like protein precursor	K.KTTVILPLAPFVR.Y	3	3.50	0.30	-3.12
IPI00299059	Isoform 2 of Neural cell adhesion molecule L1-like protein precursor	K.LGIAM*SEEIEFIVPSVPK.F	2	3.11	0.24	-3.26
IPI00299059	Isoform 2 of Neural cell adhesion molecule L1-like protein precursor	K.LGIAM*SEEIEFIVPSVPKFPK.E	2	4.50	0.47	-3.09
IPI00299059	Isoform 2 of Neural cell adhesion molecule L1-like protein precursor	K.LGIAM*SEEIEFIVPSVPKFPK.E	3	5.36	0.47	-7.75
IPI00299059	Isoform 2 of Neural cell adhesion molecule L1-like protein precursor	K.LLLPPTESGSESSITILK.G	2	2.74	0.47	-6.14
IPI00299059	Isoform 2 of Neural cell adhesion molecule L1-like protein precursor	K.LLLPPTESGSESSITILK.G	3	5.35	0.54	-4.00
IPI00299059	Isoform 2 of Neural cell adhesion molecule L1-like protein precursor	K.LLLPPTESGSESSITILKGEILLLECFAEGLPTPQVDWNK.I	4	3.33	0.19	-1.73
IPI00299059	Isoform 2 of Neural cell adhesion molecule L1-like protein precursor	K.SM*EQNGPGLEYR.V	2	3.98	0.45	-3.35
IPI00299059	Isoform 2 of Neural cell adhesion molecule L1-like protein precursor	K.TAVTANLDIR.N	1	2.24	0.19	-3.68
IPI00299059	Isoform 2 of Neural cell adhesion molecule L1-like protein precursor	K.TAVTANLDIR.N	2	3.58	0.33	-4.21
IPI00299059	Isoform 2 of Neural cell adhesion molecule L1-like protein precursor	K.THPVEVFEPGAEH.I	2	3.54	0.47	-3.64
IPI00299059	Isoform 2 of Neural cell adhesion molecule L1-like protein precursor	K.THPVEVFEPGAEH.I	3	3.54	0.29	-2.49
IPI00299059	Isoform 2 of Neural cell adhesion molecule L1-like protein precursor	K.THPVEVFEPGAEHIV.R	2	4.08	0.52	-2.90
IPI00299059	Isoform 2 of Neural cell adhesion molecule L1-like protein precursor	K.THPVEVFEPGAEHIVR.L	2	4.64	0.49	-3.61
IPI00299059	Isoform 2 of Neural cell adhesion molecule L1-like protein precursor	K.THPVEVFEPGAEHIVR.L	3	3.70	0.47	-3.09

IPI00299059	Isoform 2 of Neural cell adhesion molecule L1-like protein precursor	K.THPVEVFEPGAEHIVR.L	4	3.08	0.24	-2.81
IPI00299059	Isoform 2 of Neural cell adhesion molecule L1-like protein precursor	K.TKSLLDGR.T	1	1.99	0.11	-1.32
IPI00299059	Isoform 2 of Neural cell adhesion molecule L1-like protein precursor	K.TLKIENVSYQDK.G	1	3.05	0.34	-3.45
IPI00299059	Isoform 2 of Neural cell adhesion molecule L1-like protein precursor	K.TLKIENVSYQDK.G	2	4.25	0.40	-3.79
IPI00299059	Isoform 2 of Neural cell adhesion molecule L1-like protein precursor	K.TLKIENVSYQDK.G	3	2.60	0.27	-1.28
IPI00299059	Isoform 2 of Neural cell adhesion molecule L1-like protein precursor	K.TLKIENVSYQDKGNYR.C	2	5.15	0.54	-4.31
IPI00299059	Isoform 2 of Neural cell adhesion molecule L1-like protein precursor	K.TLKIENVSYQDKGNYR.C	3	3.80	0.45	-3.32
IPI00299059	Isoform 2 of Neural cell adhesion molecule L1-like protein precursor	K.TTVILPLAPFVR.Y	1	2.70	0.19	-3.73
IPI00299059	Isoform 2 of Neural cell adhesion molecule L1-like protein precursor	K.TTVILPLAPFVR.Y	2	3.66	0.29	-4.32
IPI00299059	Isoform 2 of Neural cell adhesion molecule L1-like protein precursor	K.TTVILPLAPFVR.Y	3	3.41	0.22	-3.67
IPI00299059	Isoform 2 of Neural cell adhesion molecule L1-like protein precursor	K.VDKDTATLSWGLPK.K	3	2.45	0.13	-0.29
IPI00299059	Isoform 2 of Neural cell adhesion molecule L1-like protein precursor	K.VEEVKPLEGR.R	1	2.61	0.21	-3.78
IPI00299059	Isoform 2 of Neural cell adhesion molecule L1-like protein precursor	K.VEEVKPLEGR.R	2	3.27	0.20	-1.98
IPI00299059	Isoform 2 of Neural cell adhesion molecule L1-like protein precursor	K.VEEVKPLEGRR.Y	2	2.49	0.32	-2.29
IPI00299059	Isoform 2 of Neural cell adhesion molecule L1-like protein precursor	K.VIKVDKDTATLSWGLPK.K	2	4.67	0.46	-3.28
IPI00299059	Isoform 2 of Neural cell adhesion molecule L1-like protein precursor	K.VIKVDKDTATLSWGLPK.K	3	4.80	0.49	-3.48
IPI00299059	Isoform 2 of Neural cell adhesion molecule L1-like protein precursor	K.VIKVDKDTATLSWGLPK.K	4	3.33	0.10	-2.07
IPI00299059	Isoform 2 of Neural cell adhesion molecule L1-like protein precursor	K.VQAINQLGSGPD.P	2	3.00	0.39	-2.45
IPI00299059	Isoform 2 of Neural cell adhesion molecule L1-like protein precursor	K.VQVAFPFDEYFQIECEAK.G	2	5.71	0.57	-7.38
IPI00299059	Isoform 2 of Neural cell adhesion molecule L1-like protein precursor	K.VQVAFPFDEYFQIECEAK.G	3	5.20	0.43	-4.39
IPI00299059	Isoform 2 of Neural cell adhesion molecule L1-like protein precursor	K.VTWSTVPK.D	2	2.36	0.17	-1.30

IPI00299059	Isoform 2 of Neural cell adhesion molecule L1-like protein precursor	L.PPTESGSESSITILK.G	2	4.83	0.46	-3.42
IPI00299059	Isoform 2 of Neural cell adhesion molecule L1-like protein precursor	N.GSPVDNHPFAGDVVFPR.E	2	4.00	0.48	-1.80
IPI00299059	Isoform 2 of Neural cell adhesion molecule L1-like protein precursor	Q.PSQPSDHHETPPAAPDRNPQNIR.V	3	3.87	0.44	-2.83
IPI00299059	Isoform 2 of Neural cell adhesion molecule L1-like protein precursor	R.ACTSQGCGKPITEESSTLGEGSK.G	2	5.38	0.46	
IPI00299059	Isoform 2 of Neural cell adhesion molecule L1-like protein precursor	R.ACTSQGCGKPITEESSTLGEGSK.G	3	4.41	0.44	-1.32
IPI00299059	Isoform 2 of Neural cell adhesion molecule L1-like protein precursor	R.CTASNFLGTATHDFHVIVEEPPR.W	3	4.88	0.49	-5.84
IPI00299059	Isoform 2 of Neural cell adhesion molecule L1-like protein precursor	R.CTASNFLGTATHDFHVIVEEPPR.W	4	4.80	0.40	-4.19
IPI00299059	Isoform 2 of Neural cell adhesion molecule L1-like protein precursor	R.CTASNFLGTATHDFHVIVEEPPRWTK.K	3	4.30	0.41	-1.49
IPI00299059	Isoform 2 of Neural cell adhesion molecule L1-like protein precursor	R.CTASNFLGTATHDFHVIVEEPPRWTK.K	4	3.24	0.24	-2.29
IPI00299059	Isoform 2 of Neural cell adhesion molecule L1-like protein precursor	R.IPNEGHISHFQGK.Y	2	4.57	0.44	-4.18
IPI00299059	Isoform 2 of Neural cell adhesion molecule L1-like protein precursor	R.IPNEGHISHFQGK.Y	3	3.62	0.21	-3.49
IPI00299059	Isoform 2 of Neural cell adhesion molecule L1-like protein precursor	R.NDYCCFAAFPR.L	1	1.86	0.45	-2.32
IPI00299059	Isoform 2 of Neural cell adhesion molecule L1-like protein precursor	R.NDYCCFAAFPR.L	2	3.63	0.44	-3.18
IPI00299059	Isoform 2 of Neural cell adhesion molecule L1-like protein precursor	R.NSGM*VPSLDAFSEFHLTVLAYNSK.G	3	3.09	0.40	-2.77
IPI00299059	Isoform 2 of Neural cell adhesion molecule L1-like protein precursor	R.SQPSQPSDHHETPPAAPDR.N	3	3.06	0.34	-3.26
IPI00299059	Isoform 2 of Neural cell adhesion molecule L1-like protein precursor	R.SQPSQPSDHHETPPAAPDR.N	4	2.47	0.20	-3.57
IPI00299059	Isoform 2 of Neural cell adhesion molecule L1-like protein precursor	R.SQPSQPSDHHETPPAAPDRNPQNIR.V	2	3.61	0.48	-2.87
IPI00299059	Isoform 2 of Neural cell adhesion molecule L1-like protein precursor	R.SQPSQPSDHHETPPAAPDRNPQNIR.V	3	3.29	0.45	-3.63
IPI00299059	Isoform 2 of Neural cell adhesion molecule L1-like protein precursor	R.SQPSQPSDHHETPPAAPDRNPQNIR.V	5	2.08	0.32	-2.76
IPI00299059	Isoform 2 of Neural cell adhesion molecule L1-like protein precursor	R.THPKEVNILR.F	2	1.91	0.40	-4.07
IPI00299059	Isoform 2 of Neural cell adhesion molecule L1-like protein precursor	R.TTEEDAGSYSCWVENAIGK.T	2	6.92	0.63	-3.41

IPI00299059	Isoform 2 of Neural cell adhesion molecule L1-like protein precursor	R.TTEEDAGSYSCWVENAIGK.T	3	3.68	0.41	-3.53
IPI00299059	Isoform 2 of Neural cell adhesion molecule L1-like protein precursor	R.VIAVNEVGR.S	1	2.60	0.24	-2.26
IPI00299059	Isoform 2 of Neural cell adhesion molecule L1-like protein precursor	R.VIAVNEVGR.S	2	3.46	0.25	-3.16
IPI00299059	Isoform 2 of Neural cell adhesion molecule L1-like protein precursor	R.VM*TPAVYAPYDVK.V	1	2.75	0.38	-3.49
IPI00299059	Isoform 2 of Neural cell adhesion molecule L1-like protein precursor	R.VM*TPAVYAPYDVK.V	2	4.20	0.44	-6.05
IPI00299059	Isoform 2 of Neural cell adhesion molecule L1-like protein precursor	R.VM*TPAVYAPYDVK.V	3	3.42	0.22	-1.87
IPI00299059	Isoform 2 of Neural cell adhesion molecule L1-like protein precursor	R.VNGSPVDNHPF.A	1	1.84	0.27	-2.89
IPI00299059	Isoform 2 of Neural cell adhesion molecule L1-like protein precursor	R.VNGSPVDNHPFAGDVVFPR.E	2	5.26	0.62	-3.33
IPI00299059	Isoform 2 of Neural cell adhesion molecule L1-like protein precursor	R.VNGSPVDNHPFAGDVVFPR.E	3	2.83	0.34	-5.15
IPI00299059	Isoform 2 of Neural cell adhesion molecule L1-like protein precursor	R.VYM*SQK.G	1	1.63	0.10	-3.36
IPI00299059	Isoform 2 of Neural cell adhesion molecule L1-like protein precursor	R.VYM*SQKGDLYFANVEEK.D	2	5.00	0.51	-3.21
IPI00299059	Isoform 2 of Neural cell adhesion molecule L1-like protein precursor	R.VYM*SQKGDLYFANVEEK.D	3	3.11	0.28	-2.30
IPI00299059	Isoform 2 of Neural cell adhesion molecule L1-like protein precursor	R.VYM*SQKGDLYFANVEEKDSR.N	4	3.10	0.18	-3.05
IPI00299059	Isoform 2 of Neural cell adhesion molecule L1-like protein precursor	S.PVDNHPFAGDVVFPR.E	2	3.62	0.44	-6.18
IPI00299059	Isoform 2 of Neural cell adhesion molecule L1-like protein precursor	S.PVDNHPFAGDVVFPR.E	3	3.60	0.45	-0.46
IPI00299059	Isoform 2 of Neural cell adhesion molecule L1-like protein precursor	V.NGSPVDNHPFAGDVVFPR.E	2	4.03	0.53	0.67
IPI00299059	Isoform 2 of Neural cell adhesion molecule L1-like protein precursor	Y.FANVEEKDSR.N	2	3.07	0.25	-2.09
IPI00299076	Receptor-binding cancer antigen expressed on SiSo cells (Fragment)	-.M*AITQFR.L	2	1.63	0.07	-8.18
IPI00299083	Junctional adhesion molecule B precursor	K.LDTGEYSCEAR.N	2	3.60	0.41	-3.89
IPI00299083	Junctional adhesion molecule B precursor	R.AEM*IDFNIR.I	2	3.41	0.18	-1.70
IPI00299086	Syntenin-1	K.DSTGHVGFIFK.N	2	2.41	0.26	-2.98
IPI00299086	Syntenin-1	K.SIDNGIFVQLVQANSPASLVGLR.F	3	5.41	0.40	-0.98
IPI00299086	Syntenin-1	K.VDKVIQAQTAFSANPANPAILSEASAPIPHDGNLYPR.L	4	3.72	0.10	-2.05
IPI00299086	Syntenin-1	K.VIQAQTAFSANPANPAILSEASAPIPHDGNLYPR.L	3	4.94	0.45	-3.44

IPI00299086	Syntenin-1	K.VIQAQTAFSANPANPAILSEASAPIPHDGNLYPR.L	4	4.04	0.34	-3.24
IPI00299086	Syntenin-1	R.LKSIDNGIFVQLVQANSPASLVGLR.F	3	3.55	0.27	-0.89
IPI00299086	Syntenin-1	R.PSSINYM*VAPVTGNDVGIR.R	2	4.77	0.58	-1.72
IPI00299116	Podocalyxin-like protein 1 precursor	K.ATFNPAQDK.C	2	2.31	0.11	-0.69
IPI00299116	Podocalyxin-like protein 1 precursor	K.CEDLETQTQSEK.Q	2	3.44	0.28	-4.30
IPI00299145	Keratin, type II cytoskeletal 6C	K.AQYEEIAQR.S	2	2.37	0.11	-2.21
IPI00299145	Keratin, type II cytoskeletal 6C	K.KYEDEINKR.T	2	3.00	0.17	-1.46
IPI00299145	Keratin, type II cytoskeletal 6C	R.NLDLDSIIAEVKAQYEEIAQR.S	2	3.36	0.22	
IPI00299145	Keratin, type II cytoskeletal 6C	R.NLDLDSIIAEVKAQYEEIAQR.S	3	5.87	0.48	
IPI00299147	Small ubiquitin-related modifier 3 precursor	K.VAGQDGSVVQFK.I	2	3.15	0.28	-1.78
IPI00299150	Cathepsin S precursor	K.GPVSVGVDAR.H	2	3.11	0.32	-1.99
IPI00299150	Cathepsin S precursor	K.YTELPYGR.E	2	2.30	0.12	-1.31
IPI00299150	Cathepsin S precursor	K.YTELPYGREDVLK.E	2	3.18	0.21	-2.02
IPI00299263	ADP-ribosylation factor GTPase-activating protein 3	K.MNISGKKNVDSDRLGM*GFGNCR.S	2	1.86	0.09	-1.59
IPI00299299	Stress 70 protein chaperone microsome-associated 60 kDa protein precursor	K.IFTAEELEAEIGRYPFK.V	2	4.00	0.40	-4.16
IPI00299299	Stress 70 protein chaperone microsome-associated 60 kDa protein precursor	K.ILVPIQQVLK.E	2	1.99	0.19	-2.83
IPI00299299	Stress 70 protein chaperone microsome-associated 60 kDa protein precursor	K.LFDTLNEDLFQK.I	2	4.44	0.35	-2.12
IPI00299299	Stress 70 protein chaperone microsome-associated 60 kDa protein precursor	K.LGGQDFNQR.L	2	3.52	0.21	-1.88
IPI00299299	Stress 70 protein chaperone microsome-associated 60 kDa protein precursor	K.LKEM*AEAYLGM*PVANAVISVPAEFDLK.Q	3	3.97	0.25	-3.17
IPI00299299	Stress 70 protein chaperone microsome-associated 60 kDa protein precursor	K.QIYQTYGFVPSR.K	2	3.34	0.39	-2.97
IPI00299299	Stress 70 protein chaperone microsome-associated 60 kDa protein precursor	R.KLFDTLNEDLFQK.I	2	4.68	0.43	-2.97
IPI00299299	Stress 70 protein chaperone microsome-associated 60 kDa protein precursor	R.KLFDTLNEDLFQK.I	3	4.75	0.21	-1.22
IPI00299299	Stress 70 protein chaperone microsome-associated 60 kDa protein precursor	R.LLQYLYK.Q	2	2.72	0.07	-0.50
IPI00299299	Stress 70 protein chaperone microsome-associated 60 kDa protein precursor	R.QAVEM*VK.L	2	1.33	0.20	-3.71
IPI00299299	Stress 70 protein chaperone microsome-associated 60 kDa protein precursor	R.VINEPTAAAM*AYGLHK.A	2	4.73	0.54	-3.36
IPI00299399	Protein S100-B	K.AM*VALIDVFHQYSGR.E	2	3.98	0.42	-2.66
IPI00299399	Protein S100-B	K.AM*VALIDVFHQYSGR.E	3	3.79	0.38	-2.64
IPI00299399	Protein S100-B	K.ELINNELSHFLEEIKEQEVVDK.V	3	3.91	0.25	-2.16
IPI00299399	Protein S100-B	K.SELKELINNELSHFLEEIKEQEVVDK.V	4	3.13	0.17	-4.15
IPI00299435	apolipoprotein F precursor	R.SGVQQLIQYYQDQK.D	2	5.06	0.27	

IPI00299485	Complement component C1q receptor precursor	R.LLDDLVTCASR.N	2	3.54	0.32	-4.57
IPI00299503	Isoform 1 of Phosphatidylinositol-glycan-specific phospholipase D precursor	K.ETTLGDM*TGK.C	2	2.19	0.30	-2.52
IPI00299503	Isoform 1 of Phosphatidylinositol-glycan-specific phospholipase D precursor	K.VAFLTVTLHQGGATR.M	2	2.87	0.25	
IPI00299503	Isoform 1 of Phosphatidylinositol-glycan-specific phospholipase D precursor	R.ELLLEHQDAYQAGIVFPDCFYPSICK.G	3	4.31	0.09	
IPI00299503	Isoform 1 of Phosphatidylinositol-glycan-specific phospholipase D precursor	R.FGSSLITVR.S	2	3.36	0.25	-2.00
IPI00299503	Isoform 1 of Phosphatidylinositol-glycan-specific phospholipase D precursor	R.IADVTSGLIGGEDGR.V	2	4.48	0.51	-1.80
IPI00299503	Isoform 1 of Phosphatidylinositol-glycan-specific phospholipase D precursor	R.ILEGFQPSGR.F	2	2.87	0.22	-2.09
IPI00299503	Isoform 1 of Phosphatidylinositol-glycan-specific phospholipase D precursor	R.TM*FIGGSQLSQK.H	2	3.13	0.33	-2.29
IPI00299547	Neutrophil gelatinase-associated lipocalin precursor	K.VPLQQNFQDNQFQGK.W	2	4.65	0.48	-3.53
IPI00299547	Neutrophil gelatinase-associated lipocalin precursor	K.VPLQQNFQDNQFQGK.W	3	2.97	0.20	2.27
IPI00299547	Neutrophil gelatinase-associated lipocalin precursor	R.TKELTSELKENFIR.F	3	2.73	0.08	-3.37
IPI00299571	Isoform 2 of Protein disulfide-isomerase A6 precursor	K.GSFSEQGINEFLR.E	2	3.65	0.33	-3.08
IPI00299571	Isoform 2 of Protein disulfide-isomerase A6 precursor	K.IFQKGESPVDYDGGR.T	3	4.12	0.44	-2.48
IPI00299571	Isoform 2 of Protein disulfide-isomerase A6 precursor	K.LAAVDATVNQVLASR.Y	2	4.85	0.46	-0.99
IPI00299571	Isoform 2 of Protein disulfide-isomerase A6 precursor	K.NRPEDYQGGR.T	2	2.59	0.15	-2.35
IPI00299571	Isoform 2 of Protein disulfide-isomerase A6 precursor	K.VGAVDADKHHSLGGQYGVQGFPTIK.I	3	4.21	0.43	-4.03
IPI00299571	Isoform 2 of Protein disulfide-isomerase A6 precursor	R.ALDLFSDNAPPPELLEIINEDIAKR.T	3	3.57	0.32	-3.67
IPI00299571	Isoform 2 of Protein disulfide-isomerase A6 precursor	R.TGEAIVDAALSALR.Q	2	4.17	0.31	-2.99
IPI00299571	Isoform 2 of Protein disulfide-isomerase A6 precursor	R.TGEAIVDAALSALR.Q	3	4.83	0.21	-2.61
IPI00299571	Isoform 2 of Protein disulfide-isomerase A6 precursor	R.TRSDIVSR.A	2	2.90	0.09	-1.45
IPI00299571	Isoform 2 of Protein disulfide-isomerase A6 precursor	W.AAAASEVKEQTK.G	2	3.27	0.32	-2.31
IPI00299627	Dual oxidase 2 precursor	R.SSPIIIQLLSDRCLQVLNR.H	2	2.73	0.13	

IPI00299652	Isoform Long of ADAM 11 precursor	K.LNVEGTER.G	1	1.63	0.10	-1.80
IPI00299652	Isoform Long of ADAM 11 precursor	K.SVVNLADVIYK.E	2	3.57	0.31	-3.85
IPI00299652	Isoform Long of ADAM 11 precursor	K.SVVNLADVIYKEQLNTR.I	2	4.94	0.54	-4.17
IPI00299652	Isoform Long of ADAM 11 precursor	K.SVVNLADVIYKEQLNTR.I	3	4.08	0.47	-2.41
IPI00299652	Isoform Long of ADAM 11 precursor	R.EGLPEPSDATHLFSGR.T	2	3.51	0.38	-3.74
IPI00299652	Isoform Long of ADAM 11 precursor	R.EGLPEPSDATHLFSGR.T	3	1.90	0.16	-1.61
IPI00299652	Isoform Long of ADAM 11 precursor	R.LGDLVGDISSVTFYHQGK.E	2	4.50	0.54	-3.32
IPI00299652	Isoform Long of ADAM 11 precursor	R.LGDLVGDISSVTFYHQGK.E	3	2.98	0.41	-3.13
IPI00299652	Isoform Long of ADAM 11 precursor	R.QSVVLTSNFAK.S	2	2.90	0.35	1.02
IPI00299679	Isoform B of Ral guanine nucleotide dissociation stimulator-like 1	R.LSLLFLGSDM*ITSPTPTK.E	3	1.77	0.19	1.05
IPI00299699	Neural proliferation differentiation and control protein 1 precursor	L.EDEIDFLAQELAR.K	2	4.03	0.27	-5.49
IPI00299699	Neural proliferation differentiation and control protein 1 precursor	R.KESGHSTPPLPK.D	2	1.70	0.07	-4.25
IPI00299699	Neural proliferation differentiation and control protein 1 precursor	R.LEDEIDFLAQELAR.K	2	5.68	0.37	-4.91
IPI00299699	Neural proliferation differentiation and control protein 1 precursor	R.LEDEIDFLAQELAR.K	3	5.41	0.29	-3.32
IPI00299699	Neural proliferation differentiation and control protein 1 precursor	R.LEDEIDFLAQELARK.E	3	2.50	0.05	-2.36
IPI00299724	Isoform 1 of Signal regulatory protein beta-1 precursor	K.EGHFPR.V	1	2.01	0.16	-4.20
IPI00299724	Isoform 1 of Signal regulatory protein beta-1 precursor	K.GSPDDVEFK.S	1	2.24	0.25	-4.42
IPI00299724	Isoform 1 of Signal regulatory protein beta-1 precursor	K.GSPDDVEFK.S	2	1.94	0.32	-2.31
IPI00299724	Isoform 1 of Signal regulatory protein beta-1 precursor	K.SGAGTELSVR.A	1	1.96	0.38	-2.71
IPI00299724	Isoform 1 of Signal regulatory protein beta-1 precursor	K.SGAGTELSVR.A	2	3.47	0.31	-2.61
IPI00299724	Isoform 1 of Signal regulatory protein beta-1 precursor	R.KGSPDDVEFK.S	1	2.78	0.32	-3.92
IPI00299724	Isoform 1 of Signal regulatory protein beta-1 precursor	R.KGSPDDVEFK.S	2	3.05	0.34	-3.33
IPI00299738	Procollagen C-endopeptidase enhancer 1 precursor	K.AQGTLTTPNWPESDYPPGISCSWHIIAPPDQVIALTFEK.F	3	5.98	0.63	-5.12
IPI00299738	Procollagen C-endopeptidase enhancer 1 precursor	K.GVSYLLM*GQVEENRGPVLPPESFVVLHRPNQDQILTNLSK.R	4	4.74	0.24	-3.15
IPI00299738	Procollagen C-endopeptidase enhancer 1 precursor	K.GVSYLLM*GQVEENRGPVLPPESFVVLHRPNQDQILTNLSK.R	5	3.90	0.29	-3.58

IPI00299738	Procollagen C-endopeptidase enhancer 1 precursor	K.SM*VREPGEGLAVTVSLIGAYK.T	3	3.13	0.24	-2.90
IPI00299738	Procollagen C-endopeptidase enhancer 1 precursor	K.TGGLDLPSPPTGASLK.F	2	3.62	0.31	-2.75
IPI00299738	Procollagen C-endopeptidase enhancer 1 precursor	R.ATSGTEHQFCGGR.L	2	3.82	0.30	
IPI00299738	Procollagen C-endopeptidase enhancer 1 precursor	R.EPGEGLAVTVSLIGAYK.T	2	2.72	0.37	
IPI00299738	Procollagen C-endopeptidase enhancer 1 precursor	R.FCGTFRPAPLVAPGNQVTLR.M	2	3.63	0.43	-3.44
IPI00299738	Procollagen C-endopeptidase enhancer 1 precursor	R.FCGTFRPAPLVAPGNQVTLR.M	3	4.19	0.23	-3.05
IPI00299738	Procollagen C-endopeptidase enhancer 1 precursor	R.GFLLWYSGR.A	1	1.89	0.11	-0.69
IPI00299738	Procollagen C-endopeptidase enhancer 1 precursor	R.GFLLWYSGR.A	2	3.46	0.24	-1.48
IPI00299738	Procollagen C-endopeptidase enhancer 1 precursor	R.VFDLELHPACR.Y	2	2.98	0.28	
IPI00299738	Procollagen C-endopeptidase enhancer 1 precursor	R.YDALEVFAGSGTSGQR.L	2	5.84	0.55	-3.78
IPI00299738	Procollagen C-endopeptidase enhancer 1 precursor	R.YDALEVFAGSGTSGQR.L	3	3.74	0.43	-2.24
IPI00299738	Procollagen C-endopeptidase enhancer 1 precursor	R.YDSVSVFNGAVSDDSR.R	2	4.87	0.51	-4.76
IPI00299738	Procollagen C-endopeptidase enhancer 1 precursor	R.YDSVSVFNGAVSDDSRR.L	2	2.86	0.18	-3.73
IPI00299758	Carbohydrate sulfotransferase 12	K.LYEADFVLFGYPKPENLLRD.-	3	4.14	0.49	-3.56
IPI00299758	Carbohydrate sulfotransferase 12	R.ELTADSDVDEFLDKFLSAGVK.Q	3	2.18	0.25	-4.38
IPI00299758	Carbohydrate sulfotransferase 12	R.KETEQPPAPGSM*EESVR.G	3	3.16	0.24	-1.99
IPI00299778	Serum paraoxonase/lactonase 3	K.YPGM*PNFAPDEPGKIFLM*DLNEQNPR.A	3	3.81	0.32	
IPI00300020	Excitatory amino acid transporter 2	R.VHEDIEM*TK.T	2	2.54	0.20	-2.18
IPI00300052	Keratin type II cuticular Hb4	K.LLEGEESR.I	2	2.26	0.06	-3.07
IPI00300207	Isoform 1 of Uncharacterized protein FLJ44066	R.SLGATLK.Y	1	1.68	0.05	-3.46
IPI00300241	Leucine-rich repeat-containing protein 4B precursor	K.DIPNLTALVRLEELELSGNR.L	3	2.17	0.13	-3.08
IPI00300241	Leucine-rich repeat-containing protein 4B precursor	K.IEVGAFNGLPSLNTLELFDNR.L	2	5.02	0.57	-4.94
IPI00300241	Leucine-rich repeat-containing protein 4B precursor	K.IEVGAFNGLPSLNTLELFDNR.L	3	5.81	0.43	-5.72
IPI00300241	Leucine-rich repeat-containing protein 4B precursor	K.IEVGAFNGLPSLNTLELFDNRLTTVPTQAFEYLSK.L	3	6.21	0.51	-3.73

IPI00300241	Leucine-rich repeat-containing protein 4B precursor	K.IEVGAFNGLPSLNTLELFDNRLTTVPTQAFEYLSK.L	4	2.65	0.14	-4.50
IPI00300241	Leucine-rich repeat-containing protein 4B precursor	K.LWLM*HAQVATIER.N	3	2.84	0.28	-3.65
IPI00300241	Leucine-rich repeat-containing protein 4B precursor	K.SLEELNLSHNNLM*SLPHDLFTPLHR.L	3	4.53	0.49	-4.93
IPI00300241	Leucine-rich repeat-containing protein 4B precursor	K.SLEELNLSHNNLM*SLPHDLFTPLHR.L	4	3.95	0.34	-4.76
IPI00300241	Leucine-rich repeat-containing protein 4B precursor	K.SLEELNLSHNNLM*SLPHDLFTPLHR.L	5	3.41	0.35	-4.55
IPI00300241	Leucine-rich repeat-containing protein 4B precursor	R.DLAEVPASIPVNTR.Y	2	4.37	0.51	-4.99
IPI00300241	Leucine-rich repeat-containing protein 4B precursor	R.DLAEVPASIPVNTR.Y	3	2.40	0.26	-2.91
IPI00300241	Leucine-rich repeat-containing protein 4B precursor	R.HLEILQLSK.N	1	2.59	0.16	-3.45
IPI00300241	Leucine-rich repeat-containing protein 4B precursor	R.HLEILQLSK.N	2	2.26	0.26	-1.77
IPI00300241	Leucine-rich repeat-containing protein 4B precursor	R.KIEVGAFNGLPSLNTLELFDNR.L	2	5.36	0.60	-1.84
IPI00300241	Leucine-rich repeat-containing protein 4B precursor	R.KIEVGAFNGLPSLNTLELFDNR.L	3	5.57	0.47	-1.27
IPI00300241	Leucine-rich repeat-containing protein 4B precursor	R.KIEVGAFNGLPSLNTLELFDNRLTTVPTQAFEYLSK.L	4	3.50	0.26	-4.55
IPI00300241	Leucine-rich repeat-containing protein 4B precursor	R.LDLGELKRLEYISEAAFEGLVNLR.Y	3	4.14	0.31	-2.86
IPI00300241	Leucine-rich repeat-containing protein 4B precursor	R.LDLIRPGSFQGLTSLR.K	2	2.82	0.30	-3.79
IPI00300241	Leucine-rich repeat-containing protein 4B precursor	R.LDLIRPGSFQGLTSLR.K	3	4.07	0.39	-2.00
IPI00300241	Leucine-rich repeat-containing protein 4B precursor	R.LDLIRPGSFQGLTSLRK.L	2	1.98	0.24	-1.92
IPI00300241	Leucine-rich repeat-containing protein 4B precursor	R.LDLIRPGSFQGLTSLRK.L	3	4.09	0.25	-2.78
IPI00300241	Leucine-rich repeat-containing protein 4B precursor	R.LEELELSGNRLDLIRPGSFQGLTSLR.K	3	3.28	0.29	-3.78
IPI00300241	Leucine-rich repeat-containing protein 4B precursor	R.LEELELSGNRLDLIRPGSFQGLTSLR.K	4	4.09	0.36	-3.61
IPI00300241	Leucine-rich repeat-containing protein 4B precursor	R.LEELELSGNRLDLIRPGSFQGLTSLRK.L	4	4.29	0.21	-4.99
IPI00300241	Leucine-rich repeat-containing protein 4B precursor	R.LEYISEAAFEGLVNLR.Y	2	2.68	0.17	0.57

IPI00300241	Leucine-rich repeat-containing protein 4B precursor	R.LTTVPTQAFEYLSK.L	2	3.44	0.35	-3.50
IPI00300241	Leucine-rich repeat-containing protein 4B precursor	R.NAFDDLK.S	1	2.24	0.10	-3.77
IPI00300241	Leucine-rich repeat-containing protein 4B precursor	R.NAFDDLKSLEELNLSHNNLM*SLPHDLFTPLHR.L	3	5.05	0.51	-4.61
IPI00300241	Leucine-rich repeat-containing protein 4B precursor	R.NAFDDLKSLEELNLSHNNLM*SLPHDLFTPLHR.L	5	3.65	0.33	-3.80
IPI00300241	Leucine-rich repeat-containing protein 4B precursor	R.NAFDDLKSLEELNLSHNNLM*SLPHDLFTPLHR.L	6	2.64	0.24	-4.04
IPI00300241	Leucine-rich repeat-containing protein 4B precursor	R.NNPIESIPSYAFNR.V	2	4.73	0.49	-3.34
IPI00300241	Leucine-rich repeat-containing protein 4B precursor	R.NNPIESIPSYAFNR.V	3	2.28	0.13	-1.75
IPI00300241	Leucine-rich repeat-containing protein 4B precursor	R.PGSFQGLTSLR.K	2	3.28	0.35	-1.50
IPI00300241	Leucine-rich repeat-containing protein 4B precursor	R.RDLAEVPASIPVNTR.Y	2	4.26	0.38	-2.51
IPI00300241	Leucine-rich repeat-containing protein 4B precursor	R.RDLAEVPASIPVNTR.Y	3	3.38	0.31	-1.71
IPI00300241	Leucine-rich repeat-containing protein 4B precursor	R.TDTFKHLR.H	2	2.05	0.07	-2.15
IPI00300241	Leucine-rich repeat-containing protein 4B precursor	R.YIGELDQSHFTCYAP.V	2	2.92	0.33	-3.27
IPI00300241	Leucine-rich repeat-containing protein 4B precursor	R.YLNLGM*CNLKDIPNLTALVR.L	3	3.86	0.27	-1.93
IPI00300241	Leucine-rich repeat-containing protein 4B precursor	R.YLNLQENGIQVIR.T	2	4.09	0.29	-2.55
IPI00300241	Leucine-rich repeat-containing protein 4B precursor	R.YLNLQENGIQVIR.T	3	4.83	0.32	-1.12
IPI00300244	zinc finger, CW type with PWWP domain 1	K.RIFAPPAQKSYSLLPCSPNSPK.E	3	2.58	0.18	
IPI00300407	Syndecan-2 precursor	R.KM*DPAEEDTNVYTEK.H	2	4.03	0.40	-4.36
IPI00300407	Syndecan-2 precursor	R.KM*DPAEEDTNVYTEK.H	3	3.67	0.28	-2.30
IPI00300623	Pro-MCH precursor	K.GFQKEDTAEK.S	2	2.92	0.21	-1.87
IPI00300623	Pro-MCH precursor	R.NLDDDM*VFNTFR.L	2	4.03	0.49	-2.21
IPI00300725	Keratin, type II cytoskeletal 6A	K.AQYEEIAQR.S	2	2.37	0.11	-2.21
IPI00300725	Keratin, type II cytoskeletal 6A	K.KYEDEINKR.T	2	3.00	0.17	-1.46
IPI00300725	Keratin, type II cytoskeletal 6A	R.NLDLDSIIAEVKAQYEEIAQR.S	2	3.36	0.22	
IPI00300725	Keratin, type II cytoskeletal 6A	R.NLDLDSIIAEVKAQYEEIAQR.S	3	5.87	0.48	
IPI00300838	Carbohydrate sulfotransferase 8	F.ISLQDPTELAPQQVPGIK.F	2	4.82	0.39	-3.64
IPI00300838	Carbohydrate sulfotransferase 8	F.ISLQDPTELAPQQVPGIK.F	3	4.15	0.34	-3.63
IPI00300838	Carbohydrate sulfotransferase 8	I.SLQDPTELAPQQVPGIK.F	2	4.48	0.39	-4.53

IPI00300838	Carbohydrate sulfotransferase 8	R.DLSSGAPR.G	2	2.26	0.19	-3.97
IPI00300838	Carbohydrate sulfotransferase 8	R.NLPAPDQPQPPLQR.G	2	3.74	0.39	-3.96
IPI00300990	Isoform 1 of Uncharacterized protein C1orf77	R.GGMSLR.G	1	1.29	0.06	2.52
IPI00301058	Vasodilator-stimulated phosphoprotein	R.VKQELLEEVKKELQKVKEEIIEAFVQELRKRGSP.-	3	2.98	0.16	
IPI00301098	Uncharacterized protein C1orf187 precursor	K.CFDDCM*CVEGLR.C	2	3.40	0.49	-3.77
IPI00301143	Isoform 1 of Peptidase inhibitor 16 precursor	K.LQGVEETNIELLVCNYEPPGNVK.G	2	4.79	0.49	-1.97
IPI00301143	Isoform 1 of Peptidase inhibitor 16 precursor	K.LQGVEETNIELLVCNYEPPGNVK.G	3	2.62	0.15	-4.73
IPI00301143	Isoform 1 of Peptidase inhibitor 16 precursor	K.SADKVTDKTKVPSR.S	3	2.61	0.08	-2.63
IPI00301143	Isoform 1 of Peptidase inhibitor 16 precursor	R.AQVSPTASDM*LHM*R.W	2	3.65	0.50	-3.82
IPI00301143	Isoform 1 of Peptidase inhibitor 16 precursor	R.AQVSPTASDM*LHM*R.W	3	1.84	0.15	-7.17
IPI00301143	Isoform 1 of Peptidase inhibitor 16 precursor	R.LM*VELHNLYR.A	2	3.19	0.36	-3.71
IPI00301143	Isoform 1 of Peptidase inhibitor 16 precursor	R.LM*VELHNLYR.A	3	3.37	0.27	-3.62
IPI00301143	Isoform 1 of Peptidase inhibitor 16 precursor	R.SPENSLDPK.M	2	2.36	0.15	-1.15
IPI00301143	Isoform 1 of Peptidase inhibitor 16 precursor	R.WDEELAAFAK.A	1	2.41	0.35	-3.23
IPI00301143	Isoform 1 of Peptidase inhibitor 16 precursor	R.WDEELAAFAK.A	2	4.05	0.43	-2.66
IPI00301180	Isoform 2 of Solute carrier family 12 member 5	K.NKGPSPVSSEGIKD.F	2	3.23	0.35	-3.44
IPI00301180	Isoform 2 of Solute carrier family 12 member 5	R.EIQSITDESR.G	2	3.27	0.29	-2.75
IPI00301255	Immunoglobulin superfamily member 21 precursor	G.YLTVNIEPLPPVVAGDAVTLK.C	2	5.73	0.41	-4.14
IPI00301255	Immunoglobulin superfamily member 21 precursor	K.HPALSM*PM*QAEVTLVAPK.G	3	2.41	0.09	-2.61
IPI00301255	Immunoglobulin superfamily member 21 precursor	K.SLSLLDAENR.G	1	2.42	0.26	-3.56
IPI00301255	Immunoglobulin superfamily member 21 precursor	K.SLSLLDAENR.G	2	3.50	0.21	-1.84
IPI00301255	Immunoglobulin superfamily member 21 precursor	R.LIVFENPNIPR.G	2	3.26	0.42	-3.10
IPI00301255	Immunoglobulin superfamily member 21 precursor	R.LLDGSAEFDGK.E	2	2.33	0.35	-2.44
IPI00301255	Immunoglobulin superfamily member 21 precursor	R.TPSSDGTVEVR.A	2	3.16	0.27	-1.48
IPI00301288	polydom	Y.DDFLDTVQETATSIGNAK.S	2	5.64	0.51	-2.72
IPI00301288	polydom	Y.DDFLDTVQETATSIGNAK.S	3	3.62	0.36	-1.41
IPI00301294	Protein FAM134A	R.DLGEGEEGELAPPEDLLGRPQALSR.Q	3	2.80	0.32	-2.49
IPI00301364	Isoform 1 of S-phase kinase-associated protein 1A	K.TM*LEDLGM*DDEGDDDPVPLPNVNAAILKK.V	3	4.93	0.44	-3.41
IPI00301364	Isoform 1 of S-phase kinase-associated protein 1A	K.TVANM*IK.G	1	1.33	0.11	-2.41
IPI00301364	Isoform 1 of S-phase kinase-associated protein 1A	K.VDQGTLFELILAANYLDIK.G	2	4.99	0.51	-3.00
IPI00301364	Isoform 1 of S-phase kinase-associated protein 1A	K.VDQGTLFELILAANYLDIK.G	3	3.95	0.24	-2.72
IPI00301395	Probable serine carboxypeptidase CPVL precursor	K.FLSLPEVR.Q	1	1.33	0.09	-3.29
IPI00301395	Probable serine carboxypeptidase CPVL precursor	K.FLSLPEVR.Q	2	2.30	0.09	-1.01
IPI00301395	Probable serine carboxypeptidase CPVL precursor	K.GRELSLVGPFPGLNM*K.S	2	3.88	0.50	-2.82

IPI00301395	Probable serine carboxypeptidase CPVL precursor	K.IFKSDSEVAGYIR.Q	2	4.16	0.39	0.02
IPI00301395	Probable serine carboxypeptidase CPVL precursor	K.INLNGIAIGDGYSDPESIIGGYAEFLYQIGLLDEK.Q	3	3.26	0.36	-3.12
IPI00301395	Probable serine carboxypeptidase CPVL precursor	K.NNDFYVTGESYAGK.Y	2	3.77	0.39	-4.49
IPI00301395	Probable serine carboxypeptidase CPVL precursor	K.QNWFEAFEILDK.L	2	3.69	0.34	-4.22
IPI00301395	Probable serine carboxypeptidase CPVL precursor	K.SDSEVAGYIR.Q	2	2.08	0.11	-2.48
IPI00301395	Probable serine carboxypeptidase CPVL precursor	R.CTEPEDQLYYVK.F	2	4.05	0.38	-3.87
IPI00301395	Probable serine carboxypeptidase CPVL precursor	R.ELSLVGPFPGLNM*K.S	2	2.86	0.32	-2.77
IPI00301395	Probable serine carboxypeptidase CPVL precursor	R.GGGHILPYDQPLR.A	2	3.01	0.35	-3.79
IPI00301395	Probable serine carboxypeptidase CPVL precursor	R.GGGHILPYDQPLR.A	3	3.66	0.15	-3.36
IPI00301395	Probable serine carboxypeptidase CPVL precursor	R.KQNWFEAFEILDK.L	3	4.49	0.23	-3.21
IPI00301395	Probable serine carboxypeptidase CPVL precursor	R.SVSM*PPKGDSGQPLFLTPYIEAGK.I	3	5.02	0.40	-2.93
IPI00301459	1-O-acylceramide synthase precursor	K.ICFGDGDGTVNLK.S	2	3.90	0.46	-3.64
IPI00301459	1-O-acylceramide synthase precursor	K.TFSLEFLDPSK.S	2	3.69	0.27	-4.00
IPI00301459	1-O-acylceramide synthase precursor	P.AGRHPPVVLVPGDLGNQLEAK.L	2	4.28	0.49	-3.84
IPI00301459	1-O-acylceramide synthase precursor	R.AFVSLGAPWGGVAK.T	2	3.72	0.10	-3.28
IPI00301459	1-O-acylceramide synthase precursor	R.APNENGPYFLALR.E	2	3.76	0.40	-3.29
IPI00301459	1-O-acylceramide synthase precursor	R.ATQFPDGVDVR.V	2	2.47	0.23	-3.35
IPI00301459	1-O-acylceramide synthase precursor	R.HPPVVLVPGDLGNQLEAK.L	3	3.04	0.31	-2.33
IPI00301459	1-O-acylceramide synthase precursor	R.VLASGDNNRIPVIGPLK.I	2	2.94	0.24	-2.99
IPI00301459	1-O-acylceramide synthase precursor	R.VLASGDNNRIPVIGPLK.I	3	2.77	0.06	-2.34
IPI00301459	1-O-acylceramide synthase precursor	R.VPGFGK.T	1	1.75	0.19	-2.39
IPI00301465	14-3-3-associated AKT substrate	K.YNQPFEDTPVVQMATLTYETPQGLR.I	4	3.11	0.09	-1.10
IPI00301579	Epididymal secretory protein E1 precursor	D.KTYSYLNKLPVKSEYPSIK.L	3	4.04	0.33	-3.64
IPI00301579	Epididymal secretory protein E1 precursor	K.AVVHGILM*GVPVPFPIPEPDGCK.S	2	3.85	0.45	-1.85
IPI00301579	Epididymal secretory protein E1 precursor	K.AVVHGILM*GVPVPFPIPEPDGCK.S	3	4.26	0.35	-6.97
IPI00301579	Epididymal secretory protein E1 precursor	K.DCGSVDGVIK.E	1	2.20	0.23	-4.15
IPI00301579	Epididymal secretory protein E1 precursor	K.DCGSVDGVIK.E	2	3.13	0.28	-2.78
IPI00301579	Epididymal secretory protein E1 precursor	K.DCGSVDGVIKEVNVSPCPTQPCQLSK.G	2	3.93	0.41	-2.58
IPI00301579	Epididymal secretory protein E1 precursor	K.DCGSVDGVIKEVNVSPCPTQPCQLSK.G	3	5.50	0.43	-4.11
IPI00301579	Epididymal secretory protein E1 precursor	K.DCGSVDGVIKEVNVSPCPTQPCQLSK.G	4	4.37	0.37	-2.66
IPI00301579	Epididymal secretory protein E1 precursor	K.DKTYSYLNK.L	1	2.63	0.21	-4.42

IPI00301579	Epididymal secretory protein E1 precursor	K.DKTYSYLNK.L	2	2.65	0.13	-3.47
IPI00301579	Epididymal secretory protein E1 precursor	K.DKTYSYLNKLPVK.S	2	4.66	0.35	-3.78
IPI00301579	Epididymal secretory protein E1 precursor	K.DKTYSYLNKLPVK.S	3	3.78	0.39	-2.52
IPI00301579	Epididymal secretory protein E1 precursor	K.DKTYSYLNKLPVKSEYPSIK.L	2	5.65	0.50	-4.30
IPI00301579	Epididymal secretory protein E1 precursor	K.DKTYSYLNKLPVKSEYPSIK.L	3	5.19	0.45	-4.79
IPI00301579	Epididymal secretory protein E1 precursor	K.DKTYSYLNKLPVKSEYPSIK.L	4	2.80	0.17	-3.01
IPI00301579	Epididymal secretory protein E1 precursor	K.EVNVSPCPTQPCQLSK.G	2	3.92	0.45	-3.11
IPI00301579	Epididymal secretory protein E1 precursor	K.EVNVSPCPTQPCQLSK.G	3	3.33	0.34	-1.91
IPI00301579	Epididymal secretory protein E1 precursor	K.LPVKSEYPSIK.L	1	2.75	0.34	-4.25
IPI00301579	Epididymal secretory protein E1 precursor	K.LPVKSEYPSIK.L	2	3.84	0.40	-4.32
IPI00301579	Epididymal secretory protein E1 precursor	K.LPVKSEYPSIK.L	3	2.85	0.31	-4.38
IPI00301579	Epididymal secretory protein E1 precursor	K.LVVEWQLQDDK.N	2	4.09	0.32	-3.05
IPI00301579	Epididymal secretory protein E1 precursor	K.LVVEWQLQDDKNQSLFCWEIPVQIVSHL.-	3	3.07	0.20	-4.66
IPI00301579	Epididymal secretory protein E1 precursor	K.NQSLFCWEIPVQIVSH.L	2	4.00	0.38	-3.76
IPI00301579	Epididymal secretory protein E1 precursor	K.NQSLFCWEIPVQIVSHL.-	2	3.55	0.36	-4.15
IPI00301579	Epididymal secretory protein E1 precursor	K.SEYPSIK.L	1	2.01	0.14	-2.76
IPI00301579	Epididymal secretory protein E1 precursor	K.SEYPSIK.L	2	2.24	0.15	-3.02
IPI00301579	Epididymal secretory protein E1 precursor	K.SGINCPIQK.D	2	2.93	0.07	0.12
IPI00301579	Epididymal secretory protein E1 precursor	K.TYSYLNK.L	1	1.78	0.18	-2.31
IPI00301579	Epididymal secretory protein E1 precursor	K.TYSYLNK.L	2	2.35	0.20	-1.13
IPI00301579	Epididymal secretory protein E1 precursor	K.TYSYLNKLPVK.S	1	2.75	0.17	-2.88
IPI00301579	Epididymal secretory protein E1 precursor	K.TYSYLNKLPVK.S	2	3.24	0.32	-3.90
IPI00301579	Epididymal secretory protein E1 precursor	K.TYSYLNKLPVK.S	3	1.55	0.22	-3.22
IPI00301579	Epididymal secretory protein E1 precursor	K.TYSYLNKLPVKSEYPSIK.L	2	4.46	0.44	-4.67
IPI00301579	Epididymal secretory protein E1 precursor	K.TYSYLNKLPVKSEYPSIK.L	3	3.88	0.43	-3.11
IPI00301579	Epididymal secretory protein E1 precursor	K.TYSYLNKLPVKSEYPSIK.L	4	2.83	0.21	-2.83
IPI00301631	Isoform 1 of Torsin-3A precursor	K.EETLDEIAQM*MVYVPK.E	2	2.61	0.13	
IPI00301812	Isoform 1 of SPARC-related modular calcium-binding protein 1 precursor	R.QSALEEAQQNPR.E	2	3.76	0.37	-3.25
IPI00301812	Isoform 1 of SPARC-related modular calcium-binding protein 1 precursor	R.SYESM*CEYQR.A	2	3.26	0.47	-1.67
IPI00301865	Isoform 1 of Transmembrane protein 132A precursor	A.PLDPVYLPAALELLDAPEHFR.V	2	3.28	0.43	-4.50
IPI00301865	Isoform 1 of Transmembrane protein 132A precursor	C.GQAPLDPVYLPAALELLDAPEHFR.V	2	4.72	0.56	-2.77
IPI00301865	Isoform 1 of Transmembrane protein 132A precursor	D.PVYLPAALELLDAPEHFR.V	2	4.53	0.45	-4.81
IPI00301865	Isoform 1 of Transmembrane protein 132A precursor	D.PVYLPAALELLDAPEHFR.V	3	4.71	0.41	-2.66
IPI00301865	Isoform 1 of Transmembrane protein 132A precursor	K.AEELVNTAPLTGVPQHVPVR.L	2	4.17	0.44	1.87

IPI00301865	Isoform 1 of Transmembrane protein 132A precursor	K.AEELVNTAPLTGVPQHVPVR.L	3	3.90	0.38	-1.92
IPI00301865	Isoform 1 of Transmembrane protein 132A precursor	K.M*VWEILVSER.D	2	2.53	0.20	-2.78
IPI00301865	Isoform 1 of Transmembrane protein 132A precursor	Q.APLDPVYLPAALELLDAPEHFR.V	3	4.85	0.55	-4.47
IPI00301865	Isoform 1 of Transmembrane protein 132A precursor	R.ASYPPFATQQVVPPR.V	2	2.78	0.22	-0.74
IPI00301865	Isoform 1 of Transmembrane protein 132A precursor	R.ASYPPFATQQVVPPR.V	3	3.96	0.28	-1.65
IPI00301865	Isoform 1 of Transmembrane protein 132A precursor	R.AVSVEAAVTPAEPYAR.V	2	3.32	0.45	-3.43
IPI00301865	Isoform 1 of Transmembrane protein 132A precursor	R.FLAPFAAHPLDGGR.R	2	3.62	0.41	-3.65
IPI00301865	Isoform 1 of Transmembrane protein 132A precursor	R.FLAPFAAHPLDGGR.R	3	2.52	0.23	-1.10
IPI00301865	Isoform 1 of Transmembrane protein 132A precursor	R.FLAPFAAHPLDGGRR.L	4	2.19	0.17	-1.42
IPI00301865	Isoform 1 of Transmembrane protein 132A precursor	R.IELTDTTLEQVR.G	2	4.32	0.38	-1.24
IPI00301865	Isoform 1 of Transmembrane protein 132A precursor	R.LTVWAPLLPLR.I	2	3.47	0.29	-3.39
IPI00301865	Isoform 1 of Transmembrane protein 132A precursor	R.QVAGSVGGNTGVR.G	2	3.29	0.33	-1.83
IPI00301865	Isoform 1 of Transmembrane protein 132A precursor	R.SETFLLLQPWPR.A	2	3.57	0.33	-4.77
IPI00301865	Isoform 1 of Transmembrane protein 132A precursor	R.SPLSDSILGEQALAVTDDKVSVLELR.V	2	5.16	0.56	-2.96
IPI00301865	Isoform 1 of Transmembrane protein 132A precursor	R.SPLSDSILGEQALAVTDDKVSVLELR.V	3	6.44	0.55	-4.87
IPI00301865	Isoform 1 of Transmembrane protein 132A precursor	R.SPLSDSILGEQALAVTDDKVSVLELR.V	4	5.10	0.36	-4.54
IPI00301865	Isoform 1 of Transmembrane protein 132A precursor	R.VPDM*PVRPGQLFSATLLLR.H	3	3.55	0.47	-2.28
IPI00301865	Isoform 1 of Transmembrane protein 132A precursor	R.VPGPAEGPAEPAAEASDEAER.R	2	5.56	0.59	-3.59
IPI00301865	Isoform 1 of Transmembrane protein 132A precursor	R.VPGPAEGPAEPAAEASDEAER.R	3	4.14	0.23	-3.52
IPI00301865	Isoform 1 of Transmembrane protein 132A precursor	R.VPGPAEGPAEPAAEASDEAERR.A	3	3.60	0.54	-2.54
IPI00301865	Isoform 1 of Transmembrane protein 132A precursor	R.VQPVM*GISLTLSR.G	2	3.85	0.45	-3.80

IPI00301865	Isoform 1 of Transmembrane protein 132A precursor	R.VVVGREPGVTSIEVR.S	2	3.40	0.32	-2.59
IPI00301865	Isoform 1 of Transmembrane protein 132A precursor	R.VVVGREPGVTSIEVR.S	3	3.42	0.44	-3.06
IPI00301923	Isoform 1 of Cell division protein kinase 9	K.LELVKGQKRKVK.D	2	2.33	0.15	
IPI00301961	Neuroendocrine convertase 1 precursor	K.LNIPYENFYEALEK.L	2	4.15	0.44	-4.66
IPI00301961	Neuroendocrine convertase 1 precursor	P.EAASAIAEELGYDLLGQIGSLENHYLFK.H	3	4.53	0.46	-4.68
IPI00301961	Neuroendocrine convertase 1 precursor	R.DDRLLQALVDILNEEN.-	2	4.14	0.39	-2.22
IPI00301961	Neuroendocrine convertase 1 precursor	R.DELEEGAPSQAM*LR.L	2	4.21	0.41	-3.40
IPI00301961	Neuroendocrine convertase 1 precursor	R.DELEEGAPSQAM*LR.L	3	2.38	0.13	-0.49
IPI00301961	Neuroendocrine convertase 1 precursor	R.LLQALVDILNEEN.-	2	3.94	0.35	
IPI00301961	Neuroendocrine convertase 1 precursor	R.RDELEEGAPSQAM*LR.L	2	2.80	0.22	-3.02
IPI00301961	Neuroendocrine convertase 1 precursor	R.RDELEEGAPSQAM*LR.L	3	3.31	0.18	-1.25
IPI00302133	Transient receptor potential cation channel subfamily V member 5	K.LPRCLWPRSGICGCEFGLGDRWFLRVENHNDQNPLR.V	3	1.51	0.25	-5.92
IPI00302181	Isoform 1 of Voltage-dependent calcium channel subunit alpha-2/delta-3 precursor	K.GILLGVVGTDVPVKELLK.T	2	3.77	0.29	-2.80
IPI00302181	Isoform 1 of Voltage-dependent calcium channel subunit alpha-2/delta-3 precursor	K.GILLGVVGTDVPVKELLK.T	3	2.97	0.39	-3.73
IPI00302181	Isoform 1 of Voltage-dependent calcium channel subunit alpha-2/delta-3 precursor	K.LWASAFGGEIK.S	2	3.41	0.31	-2.39
IPI00302181	Isoform 1 of Voltage-dependent calcium channel subunit alpha-2/delta-3 precursor	K.VFVDNFDRDPSLIWQYFGSAK.G	2	3.78	0.49	-3.46
IPI00302181	Isoform 1 of Voltage-dependent calcium channel subunit alpha-2/delta-3 precursor	K.VFVDNFDRDPSLIWQYFGSAK.G	3	4.48	0.30	-4.26
IPI00302181	Isoform 1 of Voltage-dependent calcium channel subunit alpha-2/delta-3 precursor	K.YSGSQLLQK.K	2	3.10	0.26	-0.47
IPI00302181	Isoform 1 of Voltage-dependent calcium channel subunit alpha-2/delta-3 precursor	R.EHLDKLFAK.G	2	1.96	0.13	-2.84
IPI00302181	Isoform 1 of Voltage-dependent calcium channel subunit alpha-2/delta-3 precursor	R.HLSQLEAIK.L	2	1.68	0.08	-2.33
IPI00302181	Isoform 1 of Voltage-dependent calcium channel subunit alpha-2/delta-3 precursor	R.IFTYLIGR.E	2	2.96	0.22	-1.51
IPI00302181	Isoform 1 of Voltage-dependent calcium channel subunit alpha-2/delta-3 precursor	R.INLFVGAEQLTNQDFLK.A	2	5.72	0.53	-4.17
IPI00302181	Isoform 1 of Voltage-dependent calcium channel subunit alpha-2/delta-3 precursor	R.INLFVGAEQLTNQDFLK.A	3	4.61	0.33	-2.69
IPI00302181	Isoform 1 of Voltage-dependent calcium channel subunit alpha-2/delta-3 precursor	R.KSPVVAAVGIQM*K.L	2	3.53	0.49	-2.60
IPI00302181	Isoform 1 of Voltage-dependent calcium channel subunit alpha-2/delta-3 precursor	R.SKGILLGVVGTDVPVKELLK.T	3	3.46	0.35	-4.80

IPI00302181	Isoform 1 of Voltage-dependent calcium channel subunit alpha-2/delta-3 precursor	R.VLVM*TNDYYYTDIK.G	2	4.26	0.42	-3.63
IPI00302592	filamin A, alpha isoform 1	K.AGVAPLQVK.V	2	2.41	0.07	-3.26
IPI00302592	filamin A, alpha isoform 1	K.DAGEGGLSLAIEGPSK.A	2	3.80	0.32	-3.73
IPI00302592	filamin A, alpha isoform 1	K.FNEEHIPDSPFVVPVASPSGDAR.R	3	3.28	0.23	-4.37
IPI00302592	filamin A, alpha isoform 1	K.GLVEPVDVVDNADGTQTVNYVPSR.E	3	2.70	0.15	-4.14
IPI00302592	filamin A, alpha isoform 1	K.VTAQGPGLEPSGNIANK.T	2	3.60	0.47	-1.49
IPI00302592	filamin A, alpha isoform 1	R.AGQSAAGAAPGGGVDTR.D	2	4.08	0.41	-3.25
IPI00302592	filamin A, alpha isoform 1	R.EATTEFSVDAR.A	2	2.73	0.38	-2.88
IPI00302592	filamin A, alpha isoform 1	R.QMQLENVSVALEFLDRESIK.L	3	2.96	0.24	-3.88
IPI00302641	Protocadherin Fat 2 precursor	K.ALDREQASSYSLK.L	2	2.88	0.10	
IPI00302641	Protocadherin Fat 2 precursor	K.ATDSGQPPLSASVR.L	2	2.69	0.12	
IPI00302641	Protocadherin Fat 2 precursor	K.AVAAQDPVIYSLVR.G	2	3.97	0.30	
IPI00302641	Protocadherin Fat 2 precursor	K.EM*EHSVGVQM*R.S	2	2.31	0.39	-3.46
IPI00302641	Protocadherin Fat 2 precursor	K.FSEPLYTFSAPEDLPEGSEIGIVK.A	2	3.20	0.25	
IPI00302641	Protocadherin Fat 2 precursor	K.FSEPLYTFSAPEDLPEGSEIGIVK.A	3	3.60	0.21	-3.56
IPI00302641	Protocadherin Fat 2 precursor	K.IIAAQGLPR.G	2	3.14	0.21	-1.97
IPI00302641	Protocadherin Fat 2 precursor	K.IILTDENDNPPQFK.A	2	2.77	0.32	-2.45
IPI00302641	Protocadherin Fat 2 precursor	K.LDQANHAPHTLTVK.A	3	2.75	0.34	-2.62
IPI00302641	Protocadherin Fat 2 precursor	K.SLIYTIHGSQDPGSASLFQLDPSSGVLVTVGK.L	3	4.40	0.20	
IPI00302641	Protocadherin Fat 2 precursor	K.SNPLFQSPYYK.V	2	3.27	0.30	-2.47
IPI00302641	Protocadherin Fat 2 precursor	K.TGNADEAVTIHPVTGSISVLNPAFLGLSR.K	3	5.83	0.47	-2.55
IPI00302641	Protocadherin Fat 2 precursor	K.TLDADISEQNR.Q	2	3.11	0.33	-3.20
IPI00302641	Protocadherin Fat 2 precursor	K.TPVAVVFAR.D	2	3.25	0.27	-1.74
IPI00302641	Protocadherin Fat 2 precursor	K.VLQLILSDPDSPENGPPYSFR.I	2	3.60	0.24	
IPI00302641	Protocadherin Fat 2 precursor	K.VLQLILSDPDSPENGPPYSFR.I	3	3.80	0.46	-3.11
IPI00302641	Protocadherin Fat 2 precursor	R.DVSYQIVEDGSDVSK.F	2	5.04	0.36	
IPI00302641	Protocadherin Fat 2 precursor	R.FSGQSYVR.Y	1	2.18	0.23	-3.34
IPI00302641	Protocadherin Fat 2 precursor	R.FSGQSYVR.Y	2	2.49	0.21	-1.32
IPI00302641	Protocadherin Fat 2 precursor	R.FTQLHYEASVPDTIAPGTELLQVR.A	3	4.01	0.29	-2.43
IPI00302641	Protocadherin Fat 2 precursor	R.GTTPESNKDGVFSLDPDTGVIK.V	3	3.55	0.10	-3.34
IPI00302641	Protocadherin Fat 2 precursor	R.HFSVGAPDGK.I	2	2.99	0.33	-1.61
IPI00302641	Protocadherin Fat 2 precursor	R.LAATDPDAGFNGK.L	2	3.22	0.37	-3.40
IPI00302641	Protocadherin Fat 2 precursor	R.LEKPLQVRPQAPLELTVR.A	4	3.17	0.24	-2.57
IPI00302641	Protocadherin Fat 2 precursor	R.LIYNIVEEEPLM*LFTTDFK.T	2	6.20	0.42	
IPI00302641	Protocadherin Fat 2 precursor	R.LIYNIVEEEPLM*LFTTDFK.T	3	4.94	0.46	-5.76
IPI00302641	Protocadherin Fat 2 precursor	R.LKVPEDLPPGTVLTFLDASDPDLGPAGEVR.Y	3	4.48	0.42	-4.58
IPI00302641	Protocadherin Fat 2 precursor	R.LSILTPR.H	2	2.09	0.06	-3.84
IPI00302641	Protocadherin Fat 2 precursor	R.LSPVSPGPVYR.L	2	2.21	0.19	-0.61
IPI00302641	Protocadherin Fat 2 precursor	R.SCQADITLHVEDVNDNAPR.F	2	3.05	0.20	
IPI00302641	Protocadherin Fat 2 precursor	R.SGSGPYFYSQIR.G	2	3.27	0.36	-3.10
IPI00302641	Protocadherin Fat 2 precursor	R.SNEFSLVSVK.D	2	2.05	0.18	-2.04

IPI00302641	Protocadherin Fat 2 precursor	R.VGASDGVFR.A	2	2.90	0.13	-2.46
IPI00302641	Protocadherin Fat 2 precursor	R.VHVTEQSHYAPSALPLEIFITVGEDEFQGGM*VGK.I	4	2.95	0.11	-3.39
IPI00302641	Protocadherin Fat 2 precursor	R.VQAIDQDKGK.S	2	2.23	0.08	-1.04
IPI00302641	Protocadherin Fat 2 precursor	R.VQLSEFSPPGSR.V	2	2.63	0.09	
IPI00302641	Protocadherin Fat 2 precursor	R.VVVGILDVNDNPPIFSHK.L	3	2.57	0.34	-2.95
IPI00302641	Protocadherin Fat 2 precursor	R.VVVHILDQNDLKPLFSPPSYR.V	3	3.11	0.13	
IPI00302840	Sodium/potassium-transporting ATPase subunit alpha-3	K.GFAFDCDDVNFTTDNLCFVGLMSMIDPPR.A	3	2.87	0.32	-2.44
IPI00302840	Sodium/potassium-transporting ATPase subunit alpha-3	K.MQVNAEEVVVGDLVEIK.G	2	4.74	0.41	-2.53
IPI00302840	Sodium/potassium-transporting ATPase subunit alpha-3	K.MQVNAEEVVVGDLVEIK.G	3	3.31	0.06	-1.26
IPI00302840	Sodium/potassium-transporting ATPase subunit alpha-3	K.QAADMILLDDNFASIVTGVEEGR.L	2	5.62	0.61	-2.21
IPI00302840	Sodium/potassium-transporting ATPase subunit alpha-3	K.QAADMILLDDNFASIVTGVEEGR.L	3	4.80	0.48	-1.61
IPI00302840	Sodium/potassium-transporting ATPase subunit alpha-3	K.VDNSSLTGESEPQTR.S	2	4.55	0.40	-2.59
IPI00302840	Sodium/potassium-transporting ATPase subunit alpha-3	R.LNIPVSQVNPR.D	2	2.30	0.13	-1.37
IPI00302850	Small nuclear ribonucleoprotein Sm D1	R.YFILPDSLPLDTLLVDVEPK.V	2	3.17	0.26	
IPI00302944	Isoform 4 of Collagen alpha-1(XII) chain precursor	K.ALALGALQNIR.Y	2	3.44	0.23	-1.05
IPI00302944	Isoform 4 of Collagen alpha-1(XII) chain precursor	K.GGNTLTGM*ALNFIR.Q	2	3.18	0.33	-4.96
IPI00302944	Isoform 4 of Collagen alpha-1(XII) chain precursor	K.VLVVVTDGR.S	2	2.80	0.22	-2.40
IPI00302944	Isoform 4 of Collagen alpha-1(XII) chain precursor	R.VFGETTNSLSVAWDHADGPVQQYR.I	3	2.53	0.18	-0.12
IPI00302962	Amphiphysin I variant CT4 (Fragment)	K.IGTETTEGAESAQPEAEELEATVPQEK.V	3	3.44	0.34	-2.86
IPI00303071	Cat eye syndrome critical region protein 1 precursor	K.FVETHPEFIGIK.I	2	3.36	0.32	-5.75
IPI00303071	Cat eye syndrome critical region protein 1 precursor	K.IIYSDHR.S	2	1.82	0.11	-3.59
IPI00303071	Cat eye syndrome critical region protein 1 precursor	K.TYQEVAQK.F	2	1.85	0.08	-0.84
IPI00303071	Cat eye syndrome critical region protein 1 precursor	K.WILLEDYR.K	2	3.63	0.24	-2.79
IPI00303071	Cat eye syndrome critical region protein 1 precursor	R.FAHPTPRPSEK.C	2	1.91	0.07	-3.63
IPI00303071	Cat eye syndrome critical region protein 1 precursor	R.IGHGFALSK.H	1	2.15	0.26	-3.61
IPI00303071	Cat eye syndrome critical region protein 1 precursor	R.IGHGFALSK.H	2	2.70	0.18	-3.10
IPI00303071	Cat eye syndrome critical region protein 1 precursor	R.LVLNTKEELANER.L	3	3.46	0.32	-3.21

IPI00303071	Cat eye syndrome critical region protein 1 precursor	R.SKDVAVIAESIR.M	2	3.89	0.40	-3.58
IPI00303071	Cat eye syndrome critical region protein 1 precursor	R.SKDVAVIAESIR.M	3	3.54	0.05	-3.09
IPI00303071	Cat eye syndrome critical region protein 1 precursor	R.SQVFNILR.M	2	1.93	0.09	-0.43
IPI00303161	Endothelial cell-selective adhesion molecule precursor	K.DSGPYSCSVNVQDK.Q	2	4.03	0.40	-4.51
IPI00303161	Endothelial cell-selective adhesion molecule precursor	R.QLPSFQTFFAPALDVIR.G	2	5.72	0.52	-4.59
IPI00303161	Endothelial cell-selective adhesion molecule precursor	R.QLPSFQTFFAPALDVIR.G	3	4.31	0.39	-3.63
IPI00303210	Isoform 2 of Ectonucleotide pyrophosphatase/phosphodiesterase family member 2 precursor	C.PAGFVRPPLIIFSVDGFR.A	3	3.90	0.36	-4.42
IPI00303210	Isoform 2 of Ectonucleotide pyrophosphatase/phosphodiesterase family member 2 precursor	F.DYDYDGLHDTEDKIK.Q	2	3.46	0.44	-1.88
IPI00303210	Isoform 2 of Ectonucleotide pyrophosphatase/phosphodiesterase family member 2 precursor	F.LSNYLTNVDDITLVPGTLGR.I	2	3.89	0.48	-2.95
IPI00303210	Isoform 2 of Ectonucleotide pyrophosphatase/phosphodiesterase family member 2 precursor	I.DKIVGQLM*DGLK.Q	2	2.98	0.32	-1.95
IPI00303210	Isoform 2 of Ectonucleotide pyrophosphatase/phosphodiesterase family member 2 precursor	I.FDYDYDGLHDTEDKIK.Q	2	3.44	0.37	-5.92
IPI00303210	Isoform 2 of Ectonucleotide pyrophosphatase/phosphodiesterase family member 2 precursor	K.AAECPAGFVRPPLIIFSVDGFR.A	2	3.41	0.48	-3.20
IPI00303210	Isoform 2 of Ectonucleotide pyrophosphatase/phosphodiesterase family member 2 precursor	K.AAECPAGFVRPPLIIFSVDGFR.A	3	5.07	0.54	-6.71
IPI00303210	Isoform 2 of Ectonucleotide pyrophosphatase/phosphodiesterase family member 2 precursor	K.AGTFFWSVVIPHER.R	2	3.88	0.50	-4.30
IPI00303210	Isoform 2 of Ectonucleotide pyrophosphatase/phosphodiesterase family member 2 precursor	K.AGTFFWSVVIPHER.R	3	3.64	0.38	-3.43
IPI00303210	Isoform 2 of Ectonucleotide pyrophosphatase/phosphodiesterase family member 2 precursor	K.AGTFFWSVVIPHERR.I	3	2.36	0.24	-5.42

IPI00303210	Isoform 2 of Ectonucleotide pyrophosphatase/phosphodiesterase family member 2 precursor	K.CFFQGDHGFDNK.V	2	3.96	0.46	-2.89
IPI00303210	Isoform 2 of Ectonucleotide pyrophosphatase/phosphodiesterase family member 2 precursor	K.CFFQGDHGFDNKVNSM*QTVFVGYGPTFK.Y	3	3.99	0.37	-3.45
IPI00303210	Isoform 2 of Ectonucleotide pyrophosphatase/phosphodiesterase family member 2 precursor	K.CFFQGDHGFDNKVNSM*QTVFVGYGPTFK.Y	4	3.01	0.15	-4.96
IPI00303210	Isoform 2 of Ectonucleotide pyrophosphatase/phosphodiesterase family member 2 precursor	K.IVGQLM*DGLK.Q	1	1.92	0.16	-4.37
IPI00303210	Isoform 2 of Ectonucleotide pyrophosphatase/phosphodiesterase family member 2 precursor	K.IVGQLM*DGLK.Q	2	3.43	0.36	-3.09
IPI00303210	Isoform 2 of Ectonucleotide pyrophosphatase/phosphodiesterase family member 2 precursor	K.KPDQHFKPYLK.Q	2	3.78	0.19	-4.92
IPI00303210	Isoform 2 of Ectonucleotide pyrophosphatase/phosphodiesterase family member 2 precursor	K.KPDQHFKPYLK.Q	3	2.76	0.20	-4.13
IPI00303210	Isoform 2 of Ectonucleotide pyrophosphatase/phosphodiesterase family member 2 precursor	K.NDKQM*SYGFLFPPYLSSSPEAK.Y	2	5.27	0.57	-4.39
IPI00303210	Isoform 2 of Ectonucleotide pyrophosphatase/phosphodiesterase family member 2 precursor	K.NDKQM*SYGFLFPPYLSSSPEAK.Y	3	4.41	0.46	-3.41
IPI00303210	Isoform 2 of Ectonucleotide pyrophosphatase/phosphodiesterase family member 2 precursor	K.NKLDELNKR.L	2	2.97	0.13	-3.10
IPI00303210	Isoform 2 of Ectonucleotide pyrophosphatase/phosphodiesterase family member 2 precursor	K.QAEVSSVPDHLTSCVRPDVR.V	2	2.45	0.18	-3.52
IPI00303210	Isoform 2 of Ectonucleotide pyrophosphatase/phosphodiesterase family member 2 precursor	K.QGVKAGTFFWSVVIPHER.R	4	3.38	0.24	-2.10
IPI00303210	Isoform 2 of Ectonucleotide pyrophosphatase/phosphodiesterase family member 2 precursor	K.QM*SYGFLFPPYLSSSPEAK.Y	2	4.95	0.54	-4.82
IPI00303210	Isoform 2 of Ectonucleotide pyrophosphatase/phosphodiesterase family member 2 precursor	K.QM*SYGFLFPPYLSSSPEAK.Y	3	3.71	0.15	-4.41

IPI00303210	Isoform 2 of Ectonucleotide pyrophosphatase/phosphodiesterase family member 2 precursor	K.QYVEGSSIPVPTHYYSIITSCLDFTQPADK.C	3	4.55	0.42	-4.45
IPI00303210	Isoform 2 of Ectonucleotide pyrophosphatase/phosphodiesterase family member 2 precursor	K.SYTSCCHDFDELCLK.T	2	4.55	0.46	-0.09
IPI00303210	Isoform 2 of Ectonucleotide pyrophosphatase/phosphodiesterase family member 2 precursor	K.SYTSCCHDFDELCLK.T	3	2.16	0.22	-1.69
IPI00303210	Isoform 2 of Ectonucleotide pyrophosphatase/phosphodiesterase family member 2 precursor	K.TFPNLYTLATGLYPESH.G	2	3.51	0.35	-3.72
IPI00303210	Isoform 2 of Ectonucleotide pyrophosphatase/phosphodiesterase family member 2 precursor	K.TFPNLYTLATGLYPESHGIVGN.S	2	4.19	0.52	-5.02
IPI00303210	Isoform 2 of Ectonucleotide pyrophosphatase/phosphodiesterase family member 2 precursor	K.TFPNLYTLATGLYPESHGIVGNSM*YDPVFDATFHLR.G	3	5.63	0.50	-3.56
IPI00303210	Isoform 2 of Ectonucleotide pyrophosphatase/phosphodiesterase family member 2 precursor	K.TFPNLYTLATGLYPESHGIVGNSM*YDPVFDATFHLR.G	4	2.73	0.42	-4.05
IPI00303210	Isoform 2 of Ectonucleotide pyrophosphatase/phosphodiesterase family member 2 precursor	K.TYLHTYESEI.-	1	2.74	0.38	-4.10
IPI00303210	Isoform 2 of Ectonucleotide pyrophosphatase/phosphodiesterase family member 2 precursor	K.TYLHTYESEI.-	2	3.08	0.36	-2.57
IPI00303210	Isoform 2 of Ectonucleotide pyrophosphatase/phosphodiesterase family member 2 precursor	K.VNSM*QTVFVGYGPTFK.Y	2	4.57	0.51	-4.65
IPI00303210	Isoform 2 of Ectonucleotide pyrophosphatase/phosphodiesterase family member 2 precursor	K.YDAFLVTNM*VPM*YPA.F	2	4.18	0.44	-1.28
IPI00303210	Isoform 2 of Ectonucleotide pyrophosphatase/phosphodiesterase family member 2 precursor	K.YDAFLVTNM*VPM*YPAF.K	2	4.11	0.56	-3.03
IPI00303210	Isoform 2 of Ectonucleotide pyrophosphatase/phosphodiesterase family member 2 precursor	K.YDAFLVTNM*VPM*YPAFK.R	2	5.18	0.54	-5.59
IPI00303210	Isoform 2 of Ectonucleotide pyrophosphatase/phosphodiesterase family member 2 precursor	K.YDAFLVTNM*VPM*YPAFK.R	3	3.31	0.34	-3.66

IPI00303210	Isoform 2 of Ectonucleotide pyrophosphatase/phosphodiesterase family member 2 precursor	K.YDAFLVTNM*VPM*YPAFKR.V	2	3.55	0.49	-4.73
IPI00303210	Isoform 2 of Ectonucleotide pyrophosphatase/phosphodiesterase family member 2 precursor	K.YDAFLVTNM*VPM*YPAFKR.V	3	5.03	0.51	-4.07
IPI00303210	Isoform 2 of Ectonucleotide pyrophosphatase/phosphodiesterase family member 2 precursor	M.SYGFLFPPYLSSSPEAK.Y	2	3.49	0.41	-5.28
IPI00303210	Isoform 2 of Ectonucleotide pyrophosphatase/phosphodiesterase family member 2 precursor	R.CFELQEAGPPDCR.C	2	5.22	0.56	-3.33
IPI00303210	Isoform 2 of Ectonucleotide pyrophosphatase/phosphodiesterase family member 2 precursor	R.CVNVIFVGDHGM*EDVTCDR.T	2	5.49	0.58	-2.79
IPI00303210	Isoform 2 of Ectonucleotide pyrophosphatase/phosphodiesterase family member 2 precursor	R.CVNVIFVGDHGM*EDVTCDR.T	3	3.67	0.34	-3.25
IPI00303210	Isoform 2 of Ectonucleotide pyrophosphatase/phosphodiesterase family member 2 precursor	R.DIEHLTSLDFFR.K	1	3.49	0.53	-2.14
IPI00303210	Isoform 2 of Ectonucleotide pyrophosphatase/phosphodiesterase family member 2 precursor	R.DIEHLTSLDFFR.K	2	4.04	0.46	-5.32
IPI00303210	Isoform 2 of Ectonucleotide pyrophosphatase/phosphodiesterase family member 2 precursor	R.DIEHLTSLDFFR.K	3	4.67	0.29	-1.15
IPI00303210	Isoform 2 of Ectonucleotide pyrophosphatase/phosphodiesterase family member 2 precursor	R.DIEHLTSLDFFRK.T	2	3.78	0.40	-3.88
IPI00303210	Isoform 2 of Ectonucleotide pyrophosphatase/phosphodiesterase family member 2 precursor	R.DIEHLTSLDFFRK.T	3	3.52	0.40	-1.84
IPI00303210	Isoform 2 of Ectonucleotide pyrophosphatase/phosphodiesterase family member 2 precursor	R.DIEHLTSLDFFRK.T	4	2.09	0.27	-3.04
IPI00303210	Isoform 2 of Ectonucleotide pyrophosphatase/phosphodiesterase family member 2 precursor	R.EIDKIVGQLM*DGLK.Q	2	4.20	0.41	-3.54
IPI00303210	Isoform 2 of Ectonucleotide pyrophosphatase/phosphodiesterase family member 2 precursor	R.EIDKIVGQLM*DGLK.Q	3	2.93	0.31	-2.19

IPI00303210	Isoform 2 of Ectonucleotide pyrophosphatase/phosphodiesterase family member 2 precursor	R.EIDKIVGQLM*DGLKQLK.L	3	2.65	0.17	-3.22
IPI00303210	Isoform 2 of Ectonucleotide pyrophosphatase/phosphodiesterase family member 2 precursor	R.GDCCTNYQVVCK.G	2	3.61	0.41	-2.75
IPI00303210	Isoform 2 of Ectonucleotide pyrophosphatase/phosphodiesterase family member 2 precursor	R.IEDIHLLVER.R	1	2.56	0.26	-2.97
IPI00303210	Isoform 2 of Ectonucleotide pyrophosphatase/phosphodiesterase family member 2 precursor	R.IEDIHLLVER.R	2	3.91	0.27	-1.16
IPI00303210	Isoform 2 of Ectonucleotide pyrophosphatase/phosphodiesterase family member 2 precursor	R.IEDIHLLVER.R	3	3.76	0.32	-2.93
IPI00303210	Isoform 2 of Ectonucleotide pyrophosphatase/phosphodiesterase family member 2 precursor	R.KPLDVYK.K	1	2.48	0.15	-2.51
IPI00303210	Isoform 2 of Ectonucleotide pyrophosphatase/phosphodiesterase family member 2 precursor	R.KPLDVYK.K	2	2.76	0.11	-2.47
IPI00303210	Isoform 2 of Ectonucleotide pyrophosphatase/phosphodiesterase family member 2 precursor	R.KPLDVYKKPSGK.C	2	3.36	0.41	-3.88
IPI00303210	Isoform 2 of Ectonucleotide pyrophosphatase/phosphodiesterase family member 2 precursor	R.NGVNVISGPIFDYDYDGLHDTEDK.I	2	5.15	0.56	-3.76
IPI00303210	Isoform 2 of Ectonucleotide pyrophosphatase/phosphodiesterase family member 2 precursor	R.NGVNVISGPIFDYDYDGLHDTEDK.I	3	3.48	0.25	-5.19
IPI00303210	Isoform 2 of Ectonucleotide pyrophosphatase/phosphodiesterase family member 2 precursor	R.NGVNVISGPIFDYDYDGLHDTEDKIK.Q	2	4.95	0.52	-6.73
IPI00303210	Isoform 2 of Ectonucleotide pyrophosphatase/phosphodiesterase family member 2 precursor	R.NGVNVISGPIFDYDYDGLHDTEDKIK.Q	3	4.82	0.49	-4.02
IPI00303210	Isoform 2 of Ectonucleotide pyrophosphatase/phosphodiesterase family member 2 precursor	R.NGVNVISGPIFDYDYDGLHDTEDKIK.Q	4	4.22	0.33	-4.38
IPI00303210	Isoform 2 of Ectonucleotide pyrophosphatase/phosphodiesterase family member 2 precursor	R.RIEDIHLLVER.R	1	2.06	0.22	-3.60

IPI00303210	Isoform 2 of Ectonucleotide pyrophosphatase/phosphodiesterase family member 2 precursor	R.RIEDIHLLVER.R	2	3.83	0.34	-4.33
IPI00303210	Isoform 2 of Ectonucleotide pyrophosphatase/phosphodiesterase family member 2 precursor	R.RIEDIHLLVER.R	3	2.87	0.25	-2.87
IPI00303210	Isoform 2 of Ectonucleotide pyrophosphatase/phosphodiesterase family member 2 precursor	R.RIEDIHLLVERR.W	2	2.64	0.25	-4.85
IPI00303210	Isoform 2 of Ectonucleotide pyrophosphatase/phosphodiesterase family member 2 precursor	R.SYPEILTLK.T	2	2.48	0.10	-2.75
IPI00303210	Isoform 2 of Ectonucleotide pyrophosphatase/phosphodiesterase family member 2 precursor	R.TEFLSNYLTNVDDITLVPGTLGR.I	2	4.76	0.50	-5.12
IPI00303210	Isoform 2 of Ectonucleotide pyrophosphatase/phosphodiesterase family member 2 precursor	R.TEFLSNYLTNVDDITLVPGTLGR.I	3	4.27	0.42	-3.30
IPI00303210	Isoform 2 of Ectonucleotide pyrophosphatase/phosphodiesterase family member 2 precursor	R.TEFLSNYLTNVDDITLVPGTLGR.I	4	3.41	0.24	-4.35
IPI00303210	Isoform 2 of Ectonucleotide pyrophosphatase/phosphodiesterase family member 2 precursor	R.TNTFRPTM*PEEVTRPNYPGIM*YLQSDFDLGCTCDDKVEPK.N	4	3.52	0.18	-3.53
IPI00303210	Isoform 2 of Ectonucleotide pyrophosphatase/phosphodiesterase family member 2 precursor	R.TNTFRPTM*PEEVTRPNYPGIM*YLQSDFDLGCTCDDKVEPK.N	5	4.96	0.39	-0.26
IPI00303210	Isoform 2 of Ectonucleotide pyrophosphatase/phosphodiesterase family member 2 precursor	R.VRDIEHLTSLDFFR.K	2	4.23	0.45	-5.60
IPI00303210	Isoform 2 of Ectonucleotide pyrophosphatase/phosphodiesterase family member 2 precursor	R.VRDIEHLTSLDFFR.K	3	3.93	0.47	-5.61
IPI00303210	Isoform 2 of Ectonucleotide pyrophosphatase/phosphodiesterase family member 2 precursor	R.VRDIEHLTSLDFFR.K	4	3.05	0.18	-4.53
IPI00303210	Isoform 2 of Ectonucleotide pyrophosphatase/phosphodiesterase family member 2 precursor	R.VRDIEHLTSLDFFRK.T	2	2.77	0.32	-5.23
IPI00303210	Isoform 2 of Ectonucleotide pyrophosphatase/phosphodiesterase family member 2 precursor	R.VRDIEHLTSLDFFRK.T	3	4.98	0.47	-4.38

IPI00303210	Isoform 2 of Ectonucleotide pyrophosphatase/phosphodiesterase family member 2 precursor	R.VRDIEHLTSLDFFRK.T	4	4.25	0.44	-5.05
IPI00303210	Isoform 2 of Ectonucleotide pyrophosphatase/phosphodiesterase family member 2 precursor	R.VSPSFSQNCLAYK.N	1	1.90	0.33	1.67
IPI00303210	Isoform 2 of Ectonucleotide pyrophosphatase/phosphodiesterase family member 2 precursor	R.VSPSFSQNCLAYK.N	2	4.15	0.47	-2.45
IPI00303210	Isoform 2 of Ectonucleotide pyrophosphatase/phosphodiesterase family member 2 precursor	R.VSPSFSQNCLAYKNDK.Q	2	4.72	0.41	-4.90
IPI00303210	Isoform 2 of Ectonucleotide pyrophosphatase/phosphodiesterase family member 2 precursor	R.VWNYFQR.V	1	2.16	0.18	-1.43
IPI00303210	Isoform 2 of Ectonucleotide pyrophosphatase/phosphodiesterase family member 2 precursor	R.VWNYFQR.V	2	2.26	0.08	-0.39
IPI00303210	Isoform 2 of Ectonucleotide pyrophosphatase/phosphodiesterase family member 2 precursor	R.WWGGQPLWITATK.Q	1	2.38	0.42	-2.22
IPI00303210	Isoform 2 of Ectonucleotide pyrophosphatase/phosphodiesterase family member 2 precursor	R.WWGGQPLWITATK.Q	2	4.32	0.46	-3.60
IPI00303210	Isoform 2 of Ectonucleotide pyrophosphatase/phosphodiesterase family member 2 precursor	R.WWGGQPLWITATK.Q	3	2.44	0.11	-1.72
IPI00303210	Isoform 2 of Ectonucleotide pyrophosphatase/phosphodiesterase family member 2 precursor	V.RDIEHLTSLDFFR.K	2	2.98	0.25	-2.44
IPI00303210	Isoform 2 of Ectonucleotide pyrophosphatase/phosphodiesterase family member 2 precursor	Y.GFLFPPYLSSSPEAK.Y	2	3.22	0.38	-2.53
IPI00303318	Protein FAM49B	K.DAEGILEDLQSYR.G	2	3.66	0.40	-4.56
IPI00303476	ATP synthase subunit beta, mitochondrial precursor	K.SLQDIIAILGMDELSEEDKLTVSR.A	2	4.74	0.52	-1.94
IPI00303476	ATP synthase subunit beta, mitochondrial precursor	K.SLQDIIAILGMDELSEEDKLTVSR.A	3	4.59	0.48	-3.83
IPI00303476	ATP synthase subunit beta, mitochondrial precursor	K.VLDSGAPIKIPVGPETLGR.I	3	4.19	0.35	-1.93
IPI00303476	ATP synthase subunit beta, mitochondrial precursor	R.VALTGLTVAEYFRDQEGQDVLLFIDNIFR.F	3	3.11	0.32	-2.56

IPI00303882	Isoform B of Mannose-6-phosphate receptor-binding protein 1	K.VSGAQEM*VSSAK.D	2	3.31	0.25	-5.09
IPI00303894	Protein FAM3A precursor	K.LFSELGSR.N	2	2.65	0.05	-1.85
IPI00303894	Protein FAM3A precursor	K.SPFEQHVK.N	2	2.66	0.26	0.09
IPI00303894	Protein FAM3A precursor	R.DSWVFVGAK.G	2	2.25	0.14	-1.11
IPI00303894	Protein FAM3A precursor	R.KLFSELGSR.N	2	2.14	0.15	-2.07
IPI00303894	Protein FAM3A precursor	R.VVSGAANVIGPK.I	2	2.77	0.39	-0.81
IPI00303894	Protein FAM3A precursor	S.ILLGGPGSGFPR.I	2	3.11	0.16	-2.03
IPI00303963	Complement C2 precursor (Fragment)	K.ALHQVFEHM*LDVSK.L	2	3.97	0.50	-2.79
IPI00303963	Complement C2 precursor (Fragment)	K.ALHQVFEHM*LDVSK.L	3	2.70	0.39	-1.85
IPI00303963	Complement C2 precursor (Fragment)	K.ALHQVFEHM*LDVSK.L	4	3.20	0.24	-2.05
IPI00303963	Complement C2 precursor (Fragment)	K.AVISPGFDVFAK.K	1	3.10	0.30	-4.10
IPI00303963	Complement C2 precursor (Fragment)	K.AVISPGFDVFAK.K	2	3.11	0.42	-3.93
IPI00303963	Complement C2 precursor (Fragment)	K.AVISPGFDVFAKK.N	2	2.67	0.21	-2.74
IPI00303963	Complement C2 precursor (Fragment)	K.AVISPGFDVFAKK.N	3	2.17	0.18	-1.80
IPI00303963	Complement C2 precursor (Fragment)	K.ESASLM*VDR.I	2	2.21	0.20	-3.64
IPI00303963	Complement C2 precursor (Fragment)	K.KNQGILEFYGDDIALLK.L	2	5.38	0.46	-3.44
IPI00303963	Complement C2 precursor (Fragment)	K.KNQGILEFYGDDIALLK.L	3	4.47	0.41	-4.49
IPI00303963	Complement C2 precursor (Fragment)	K.NQGILEFYGDDIALLK.L	2	6.22	0.47	-7.46
IPI00303963	Complement C2 precursor (Fragment)	K.RNDYLDIYAIGVGK.L	2	4.41	0.50	-3.67
IPI00303963	Complement C2 precursor (Fragment)	K.RNDYLDIYAIGVGK.L	3	4.36	0.37	-1.52
IPI00303963	Complement C2 precursor (Fragment)	K.TAVDHIR.E	2	2.37	0.29	-4.32
IPI00303963	Complement C2 precursor (Fragment)	K.TAVDHIREILNINQK.R	2	3.66	0.23	-2.60
IPI00303963	Complement C2 precursor (Fragment)	K.VLM*SVLNDNSR.D	2	3.77	0.36	-3.22
IPI00303963	Complement C2 precursor (Fragment)	K.VPPPRDFHINLFR.M	2	2.34	0.14	-3.80
IPI00303963	Complement C2 precursor (Fragment)	K.VPPPRDFHINLFR.M	3	1.99	0.10	-3.31
IPI00303963	Complement C2 precursor (Fragment)	R.CPAPVSFENGIYTPR.L	2	3.92	0.52	-2.12
IPI00303963	Complement C2 precursor (Fragment)	R.CSSNLVLTGSSER.E	2	4.94	0.45	-1.54
IPI00303963	Complement C2 precursor (Fragment)	R.DFHINLFR.M	1	2.52	0.19	-3.02
IPI00303963	Complement C2 precursor (Fragment)	R.DFHINLFR.M	2	2.33	0.09	-1.26
IPI00303963	Complement C2 precursor (Fragment)	R.DHENELLNK.Q	1	3.02	0.30	-3.38
IPI00303963	Complement C2 precursor (Fragment)	R.DHENELLNK.Q	2	3.19	0.34	-1.76
IPI00303963	Complement C2 precursor (Fragment)	R.DM*TEVISSLENANYK.D	2	5.61	0.43	-3.40
IPI00303963	Complement C2 precursor (Fragment)	R.ECQGNGVWSGTEPICR.Q	2	3.24	0.36	-0.06
IPI00303963	Complement C2 precursor (Fragment)	R.ELNELGSK.K	2	1.72	0.14	-4.00
IPI00303963	Complement C2 precursor (Fragment)	R.ELNELGSKK.D	1	2.42	0.16	-4.39
IPI00303963	Complement C2 precursor (Fragment)	R.ELNELGSKKDGER.H	2	2.32	0.16	-2.97
IPI00303963	Complement C2 precursor (Fragment)	R.EVVTDQFLCSGTQEDESPCKGESGGAVFLER.R	3	5.26	0.54	-6.75
IPI00303963	Complement C2 precursor (Fragment)	R.EVVTDQFLCSGTQEDESPCKGESGGAVFLER.R	4	4.56	0.31	-3.29
IPI00303963	Complement C2 precursor (Fragment)	R.GALISDQWVLTAAHCFR.D	2	3.93	0.49	-2.59
IPI00303963	Complement C2 precursor (Fragment)	R.HAFILQDTK.A	1	3.11	0.25	-1.00
IPI00303963	Complement C2 precursor (Fragment)	R.HAFILQDTK.A	2	3.15	0.41	-1.58

IPI00303963	Complement C2 precursor (Fragment)	R.HAIILLTDGK.S	1	2.91	0.26	-2.89
IPI00303963	Complement C2 precursor (Fragment)	R.HAIILLTDGK.S	2	3.19	0.35	-2.07
IPI00303963	Complement C2 precursor (Fragment)	R.LLGM*ETM*AWQEIR.H	2	3.20	0.27	-2.91
IPI00303963	Complement C2 precursor (Fragment)	R.QHLGDVLNFLPL.-	2	2.84	0.31	-3.82
IPI00303963	Complement C2 precursor (Fragment)	R.QPYSYDFPEDVAPALGTSFSHM*LGATNPTQK.T	3	4.46	0.44	-5.10
IPI00303963	Complement C2 precursor (Fragment)	R.QPYSYDFPEDVAPALGTSFSHM*LGATNPTQK.T	4	2.94	0.19	-2.02
IPI00304273	Apolipoprotein A-IV precursor	A.EVSADQVATVM*WDYFSQLSNNAK.E	2	5.18	0.63	-4.09
IPI00304273	Apolipoprotein A-IV precursor	A.EVSADQVATVM*WDYFSQLSNNAK.E	3	6.70	0.61	-4.05
IPI00304273	Apolipoprotein A-IV precursor	A.EVSADQVATVMWDYFSQLSNNAK.E	2	5.28	0.57	-2.32
IPI00304273	Apolipoprotein A-IV precursor	A.EVSADQVATVMWDYFSQLSNNAK.E	3	4.17	0.28	-0.76
IPI00304273	Apolipoprotein A-IV precursor	A.KIDQNVEELKGR.L	2	3.77	0.24	-2.82
IPI00304273	Apolipoprotein A-IV precursor	D.YFSQLSNNAK.E	2	3.18	0.33	-1.38
IPI00304273	Apolipoprotein A-IV precursor	K.AKIDQNVEELK.G	2	2.95	0.11	-3.37
IPI00304273	Apolipoprotein A-IV precursor	K.AKIDQNVEELKGR.L	2	4.21	0.40	-4.06
IPI00304273	Apolipoprotein A-IV precursor	K.AKIDQNVEELKGR.L	3	4.25	0.43	-3.20
IPI00304273	Apolipoprotein A-IV precursor	K.ALVQQM*EQLR.Q	2	3.54	0.24	-3.15
IPI00304273	Apolipoprotein A-IV precursor	K.ALVQQMEQLR.Q	2	3.06	0.09	-1.29
IPI00304273	Apolipoprotein A-IV precursor	K.DLRDKVNSFFSTFK.E	2	4.07	0.35	-3.79
IPI00304273	Apolipoprotein A-IV precursor	K.DLRDKVNSFFSTFK.E	3	3.71	0.28	-3.81
IPI00304273	Apolipoprotein A-IV precursor	K.DLRDKVNSFFSTFK.E	4	2.40	0.11	-2.99
IPI00304273	Apolipoprotein A-IV precursor	K.DLRDKVNSFFSTFKEK.E	2	3.99	0.44	-3.65
IPI00304273	Apolipoprotein A-IV precursor	K.DSEKLKEEIGKELEELR.A	2	5.33	0.34	-3.95
IPI00304273	Apolipoprotein A-IV precursor	K.DSEKLKEEIGKELEELR.A	3	4.80	0.39	-2.49
IPI00304273	Apolipoprotein A-IV precursor	K.DSEKLKEEIGKELEELR.A	4	4.01	0.41	-2.73
IPI00304273	Apolipoprotein A-IV precursor	K.EAVEHLQK.S	1	2.15	0.12	-4.16
IPI00304273	Apolipoprotein A-IV precursor	K.EAVEHLQK.S	2	2.53	0.24	-2.28
IPI00304273	Apolipoprotein A-IV precursor	K.EKESQDKTLSLPELEQQQEQQQEQQQEQVQM*LAPLES.-	3	5.05	0.47	-1.76
IPI00304273	Apolipoprotein A-IV precursor	K.EKESQDKTLSLPELEQQQEQQQEQQQEQVQM*LAPLES.-	4	3.18	0.18	-4.46
IPI00304273	Apolipoprotein A-IV precursor	K.ESQDKTLSLPELEQQQEQQQEQQQEQVQM*LAPLES.-	3	5.13	0.45	-4.02
IPI00304273	Apolipoprotein A-IV precursor	K.IDQNVEELK.G	1	2.68	0.15	-4.80
IPI00304273	Apolipoprotein A-IV precursor	K.IDQNVEELK.G	2	3.15	0.18	-2.93
IPI00304273	Apolipoprotein A-IV precursor	K.IDQNVEELKGR.L	2	3.91	0.26	-3.45
IPI00304273	Apolipoprotein A-IV precursor	K.IDQNVEELKGR.L	3	3.30	0.19	-3.94
IPI00304273	Apolipoprotein A-IV precursor	K.IDQTVEELR.R	2	2.80	0.15	-1.87
IPI00304273	Apolipoprotein A-IV precursor	K.IDQTVEELRR.S	2	2.46	0.10	-2.07
IPI00304273	Apolipoprotein A-IV precursor	K.IGDNLRELQQR.L	3	1.90	0.10	-1.75
IPI00304273	Apolipoprotein A-IV precursor	K.KLVPFATELHER.L	2	3.25	0.34	-4.46
IPI00304273	Apolipoprotein A-IV precursor	K.KLVPFATELHER.L	3	4.22	0.40	-3.89
IPI00304273	Apolipoprotein A-IV precursor	K.LGEVNTYAGDLQK.K	1	3.35	0.42	-2.75
IPI00304273	Apolipoprotein A-IV precursor	K.LGEVNTYAGDLQK.K	2	4.45	0.46	-3.68
IPI00304273	Apolipoprotein A-IV precursor	K.LGEVNTYAGDLQKK.L	2	3.34	0.30	-2.41
IPI00304273	Apolipoprotein A-IV precursor	K.LGEVNTYAGDLQKK.L	3	2.66	0.19	-0.32

IPI00304273	Apolipoprotein A-IV precursor	K.LGPHAGDVEGHLSFLEK.D	2	5.19	0.45	-6.99
IPI00304273	Apolipoprotein A-IV precursor	K.LGPHAGDVEGHLSFLEK.D	3	5.05	0.46	-6.78
IPI00304273	Apolipoprotein A-IV precursor	K.LGPHAGDVEGHLSFLEK.D	4	4.24	0.47	-5.57
IPI00304273	Apolipoprotein A-IV precursor	K.LGPHAGDVEGHLSFLEKDLR.D	2	5.88	0.54	-4.39
IPI00304273	Apolipoprotein A-IV precursor	K.LGPHAGDVEGHLSFLEKDLR.D	3	4.00	0.41	-3.61
IPI00304273	Apolipoprotein A-IV precursor	K.LGPHAGDVEGHLSFLEKDLR.D	4	3.42	0.33	-2.28
IPI00304273	Apolipoprotein A-IV precursor	K.LKEEIGKELEELR.A	2	4.46	0.37	-3.55
IPI00304273	Apolipoprotein A-IV precursor	K.LKEEIGKELEELR.A	3	4.80	0.44	-2.42
IPI00304273	Apolipoprotein A-IV precursor	K.LNHQLEGLTFQM*K.K	2	4.02	0.32	-4.33
IPI00304273	Apolipoprotein A-IV precursor	K.LNHQLEGLTFQM*K.K	3	3.90	0.31	-3.13
IPI00304273	Apolipoprotein A-IV precursor	K.LVPFATELHER.L	2	2.83	0.49	-3.94
IPI00304273	Apolipoprotein A-IV precursor	K.NAEELKAR.I	1	2.00	0.06	-4.41
IPI00304273	Apolipoprotein A-IV precursor	K.NAEELKAR.I	2	2.85	0.12	-2.95
IPI00304273	Apolipoprotein A-IV precursor	K.SELTQQLNALFQDK.L	1	3.86	0.43	-4.16
IPI00304273	Apolipoprotein A-IV precursor	K.SELTQQLNALFQDK.L	2	5.38	0.38	-5.02
IPI00304273	Apolipoprotein A-IV precursor	K.SELTQQLNALFQDK.L	3	4.62	0.45	-3.25
IPI00304273	Apolipoprotein A-IV precursor	K.SELTQQLNALFQDKLGEVNTYAGDLQK.K	2	5.07	0.59	-2.50
IPI00304273	Apolipoprotein A-IV precursor	K.SELTQQLNALFQDKLGEVNTYAGDLQK.K	3	6.61	0.63	-5.14
IPI00304273	Apolipoprotein A-IV precursor	K.SELTQQLNALFQDKLGEVNTYAGDLQK.K	4	5.12	0.44	-3.50
IPI00304273	Apolipoprotein A-IV precursor	K.SLAELGGHLDQQVEEFR.R	2	5.67	0.51	-8.42
IPI00304273	Apolipoprotein A-IV precursor	K.SLAELGGHLDQQVEEFR.R	3	5.92	0.50	-4.30
IPI00304273	Apolipoprotein A-IV precursor	K.SLAELGGHLDQQVEEFRR.R	2	4.43	0.35	-5.16
IPI00304273	Apolipoprotein A-IV precursor	K.SLAELGGHLDQQVEEFRR.R	3	4.55	0.41	-5.23
IPI00304273	Apolipoprotein A-IV precursor	K.SLAELGGHLDQQVEEFRR.R	4	1.74	0.17	-2.65
IPI00304273	Apolipoprotein A-IV precursor	K.VKIDQTVEELRR.S	2	2.91	0.15	-3.70
IPI00304273	Apolipoprotein A-IV precursor	K.VKIDQTVEELRR.S	3	2.36	0.14	-1.79
IPI00304273	Apolipoprotein A-IV precursor	K.VNSFFSTFK.E	1	1.91	0.15	-3.24
IPI00304273	Apolipoprotein A-IV precursor	K.VNSFFSTFK.E	2	2.75	0.39	-1.57
IPI00304273	Apolipoprotein A-IV precursor	L.AELGGHLDQQVEEFR.R	2	3.91	0.26	0.99
IPI00304273	Apolipoprotein A-IV precursor	L.APYAQDTQEKLNHQLEGLTFQM*K.K	3	3.92	0.47	-4.42
IPI00304273	Apolipoprotein A-IV precursor	L.LPHANEVSQK.I	1	2.12	0.30	-3.98
IPI00304273	Apolipoprotein A-IV precursor	L.RTQVNTQAEQLR.R	2	2.91	0.18	-4.18
IPI00304273	Apolipoprotein A-IV precursor	R.DKVNSFFSTFK.E	1	3.20	0.37	-1.59
IPI00304273	Apolipoprotein A-IV precursor	R.DKVNSFFSTFK.E	2	3.46	0.33	-2.96
IPI00304273	Apolipoprotein A-IV precursor	R.DKVNSFFSTFK.E	3	2.62	0.09	-2.89
IPI00304273	Apolipoprotein A-IV precursor	R.DKVNSFFSTFKEK.E	2	4.68	0.34	-2.14
IPI00304273	Apolipoprotein A-IV precursor	R.DKVNSFFSTFKEK.E	3	3.48	0.44	-1.26
IPI00304273	Apolipoprotein A-IV precursor	R.DKVNSFFSTFKEKESQDK.T	3	2.45	0.14	-3.94
IPI00304273	Apolipoprotein A-IV precursor	R.ENADSLQASLRPHADELK.A	2	3.23	0.24	-4.63
IPI00304273	Apolipoprotein A-IV precursor	R.ENADSLQASLRPHADELK.A	3	2.62	0.21	-2.95
IPI00304273	Apolipoprotein A-IV precursor	R.ENADSLQASLRPHADELKAK.I	3	3.53	0.38	-2.09
IPI00304273	Apolipoprotein A-IV precursor	R.GNLRGNTEGLQK.S	2	2.86	0.21	-2.92

IPI00304273	Apolipoprotein A-IV precursor	R.GNLRGNTEGLQK.S	3	2.80	0.42	-3.25
IPI00304273	Apolipoprotein A-IV precursor	R.ISASAEELR.Q	1	2.28	0.06	-1.84
IPI00304273	Apolipoprotein A-IV precursor	R.ISASAEELR.Q	2	3.74	0.30	-1.75
IPI00304273	Apolipoprotein A-IV precursor	R.ISASAEELRQR.L	2	2.66	0.07	-3.51
IPI00304273	Apolipoprotein A-IV precursor	R.LAKDSEKLKEEIGKELEELR.A	3	4.01	0.43	-4.47
IPI00304273	Apolipoprotein A-IV precursor	R.LAKDSEKLKEEIGKELEELR.A	4	4.38	0.37	-4.67
IPI00304273	Apolipoprotein A-IV precursor	R.LAPLAEDVR.G	1	1.83	0.07	-3.95
IPI00304273	Apolipoprotein A-IV precursor	R.LAPLAEDVR.G	2	2.44	0.14	-2.78
IPI00304273	Apolipoprotein A-IV precursor	R.LEPYADQLR.T	1	2.24	0.08	-3.56
IPI00304273	Apolipoprotein A-IV precursor	R.LEPYADQLR.T	2	2.14	0.18	-3.19
IPI00304273	Apolipoprotein A-IV precursor	R.LLPHANEVSQK.I	1	2.80	0.18	-3.97
IPI00304273	Apolipoprotein A-IV precursor	R.LLPHANEVSQK.I	2	2.44	0.35	-3.52
IPI00304273	Apolipoprotein A-IV precursor	R.LTPYADEFK.V	1	1.83	0.25	-3.56
IPI00304273	Apolipoprotein A-IV precursor	R.LTPYADEFK.V	2	1.98	0.31	-2.48
IPI00304273	Apolipoprotein A-IV precursor	R.LTPYADEFKVK.I	2	2.06	0.33	-1.89
IPI00304273	Apolipoprotein A-IV precursor	R.QKLGPHAGDVEGHLSFLEK.D	2	2.19	0.30	-2.46
IPI00304273	Apolipoprotein A-IV precursor	R.QLTPYAQR.M	2	1.57	0.09	-2.18
IPI00304273	Apolipoprotein A-IV precursor	R.RQLTPYAQR.M	2	2.87	0.11	-2.48
IPI00304273	Apolipoprotein A-IV precursor	R.RVEPYGENFNK.A	1	2.62	0.36	-3.03
IPI00304273	Apolipoprotein A-IV precursor	R.RVEPYGENFNK.A	2	3.74	0.28	-4.12
IPI00304273	Apolipoprotein A-IV precursor	R.RVEPYGENFNK.A	3	3.47	0.34	-2.74
IPI00304273	Apolipoprotein A-IV precursor	R.SLAPYAQDTQEK.L	1	2.78	0.35	-3.17
IPI00304273	Apolipoprotein A-IV precursor	R.SLAPYAQDTQEK.L	2	3.51	0.45	-3.51
IPI00304273	Apolipoprotein A-IV precursor	R.SLAPYAQDTQEKLNHQLEGLTFQM*K.K	2	3.15	0.31	-3.76
IPI00304273	Apolipoprotein A-IV precursor	R.SLAPYAQDTQEKLNHQLEGLTFQM*K.K	3	4.29	0.47	-3.95
IPI00304273	Apolipoprotein A-IV precursor	R.TQVNTQAEQLR.R	1	2.64	0.15	-2.85
IPI00304273	Apolipoprotein A-IV precursor	R.TQVNTQAEQLR.R	2	3.97	0.30	-2.53
IPI00304273	Apolipoprotein A-IV precursor	R.TQVNTQAEQLRR.Q	2	3.01	0.19	-3.58
IPI00304273	Apolipoprotein A-IV precursor	R.VEPYGENFNK.A	1	2.20	0.18	-3.90
IPI00304273	Apolipoprotein A-IV precursor	R.VEPYGENFNK.A	2	2.19	0.24	-3.50
IPI00304273	Apolipoprotein A-IV precursor	R.VLRENADSLQASLRPHADELK.A	2	3.44	0.24	-4.31
IPI00304273	Apolipoprotein A-IV precursor	R.VLRENADSLQASLRPHADELK.A	3	3.64	0.25	-4.09
IPI00304273	Apolipoprotein A-IV precursor	R.VLRENADSLQASLRPHADELK.A	4	3.43	0.31	-1.88
IPI00304273	Apolipoprotein A-IV precursor	R.VLRENADSLQASLRPHADELK.A	5	3.77	0.35	-3.36
IPI00304273	Apolipoprotein A-IV precursor	V.LRENADSLQASLRPHADELK.A	3	3.62	0.21	-3.34
IPI00304273	Apolipoprotein A-IV precursor	V.PFATELHER.L	2	2.90	0.18	-2.61
IPI00304273	Apolipoprotein A-IV precursor	W.DYFSQLSNNAK.E	2	3.37	0.28	-3.10
IPI00304331	Galactosylgalactosylxylosylprotein 3-beta-glucuronosyltransferase 3	K.M*KQEEQLQR.Q	2	2.76	0.10	-3.27
IPI00304331	Galactosylgalactosylxylosylprotein 3-beta-glucuronosyltransferase 3	R.FEGPQVQDGR.V	2	2.93	0.38	-3.04

IPI00304331	Galactosylgalactosylxylosylprotein 3-beta-glucuronosyltransferase 3	R.GHLESSLLSHLVDPK.D	2	3.23	0.32	-3.65
IPI00304331	Galactosylgalactosylxylosylprotein 3-beta-glucuronosyltransferase 3	R.ISQLQAELR.R	2	3.07	0.07	-1.60
IPI00304331	Galactosylgalactosylxylosylprotein 3-beta-glucuronosyltransferase 3	R.LSQTLSLVPR.L	2	3.54	0.20	-1.36
IPI00304527	Protein FAM83B	K.KPSDSLSVASSSR.E	2	2.20	0.06	-3.72
IPI00304577	Isoform A of AP-2 complex subunit alpha-1	K.ANHPM*DAEVTK.A	3	2.58	0.14	-2.65
IPI00304577	Isoform A of AP-2 complex subunit alpha-1	K.LLGFGSALLDNVDPNPENFVGAGIIQTK.A	3	5.05	0.47	-4.26
IPI00304577	Isoform A of AP-2 complex subunit alpha-1	K.NAILFETISLIIHYDSEPNLLVR.A	3	2.70	0.24	-1.38
IPI00304596	Non-POU domain-containing octamer-binding protein	R.NLPQYVSNELLEEAFSVFGQVER.A	3	3.26	0.32	-4.82
IPI00304789	27 kDa protein	R.SQGKGIFLFRRLKDIVDWR.K	3	3.68	0.12	
IPI00304840	Isoform 2C2 of Collagen alpha-2(VI) chain precursor	K.NFVINVVNR.L	2	2.83	0.29	-2.23
IPI00304840	Isoform 2C2 of Collagen alpha-2(VI) chain precursor	K.NLQGISSFR.R	2	2.62	0.23	-1.27
IPI00304840	Isoform 2C2 of Collagen alpha-2(VI) chain precursor	R.AAVFHEK.D	2	2.00	0.07	-3.47
IPI00304840	Isoform 2C2 of Collagen alpha-2(VI) chain precursor	R.KQNVVPTVLALGSDVDM*DVLTTLSLGDR.A	3	3.49	0.29	-4.79
IPI00304840	Isoform 2C2 of Collagen alpha-2(VI) chain precursor	R.LGEQNFHK.A	2	1.88	0.12	-3.69
IPI00304840	Isoform 2C2 of Collagen alpha-2(VI) chain precursor	R.RDDDPLNAR.V	2	2.57	0.10	0.40
IPI00304865	transforming growth factor, beta receptor III	A.TAGPEPGALCELSPVSASHPVQALM*ESFTVLSGCASR.G	3	4.86	0.58	-4.02
IPI00304865	transforming growth factor, beta receptor III	K.NFLSLNYLAEYLQPK.A	2	4.47	0.44	-5.35
IPI00304865	transforming growth factor, beta receptor III	K.SIRDDIPSTQGNLVK.W	2	4.04	0.14	-3.00
IPI00304865	transforming growth factor, beta receptor III	R.GTTGLPQEVHVLNLR.T	2	3.70	0.45	-4.06
IPI00304865	transforming growth factor, beta receptor III	R.ILLDPGALPALQNPPIR.G	3	2.99	0.34	-2.70
IPI00304865	transforming growth factor, beta receptor III	R.KEYGAVTSFTELK.I	2	3.98	0.31	-5.35
IPI00304865	transforming growth factor, beta receptor III	R.TAGQGPGQLQR.E	2	3.54	0.33	-1.37
IPI00304925	Heat shock 70 kDa protein 1	K.ATAGDTHLGGEDFDNR.L	3	2.66	0.36	-1.77
IPI00304925	Heat shock 70 kDa protein 1	K.DNNLLGR.F	1	2.13	0.11	-3.90
IPI00304925	Heat shock 70 kDa protein 1	K.ITITNDKGR.L	1	2.08	0.20	-3.55
IPI00304925	Heat shock 70 kDa protein 1	K.ITITNDKGR.L	2	2.65	0.24	-2.81
IPI00304925	Heat shock 70 kDa protein 1	K.LLQDFFNGR.D	2	2.31	0.36	-3.60
IPI00304925	Heat shock 70 kDa protein 1	K.NALESYAFNM*K.S	2	3.63	0.36	-3.06
IPI00304925	Heat shock 70 kDa protein 1	K.VQVSYKGETK.A	2	2.85	0.32	-2.71
IPI00304925	Heat shock 70 kDa protein 1	K.YKAEDEVQR.E	2	2.82	0.27	-0.92
IPI00304925	Heat shock 70 kDa protein 1	K.YKAEDEVQRER.V	2	2.74	0.21	-3.20
IPI00304925	Heat shock 70 kDa protein 1	R.AM*TKDNNLLGR.F	3	2.60	0.09	-4.52

IPI00304925	Heat shock 70 kDa protein 1	R.LIGDAAK.N	1	1.96	0.12	-2.67
IPI00304925	Heat shock 70 kDa protein 1	R.LSKEEIER.M	2	2.41	0.10	-3.04
IPI00304925	Heat shock 70 kDa protein 1	R.M*VQEAEKYK.A	2	2.07	0.11	-2.60
IPI00304925	Heat shock 70 kDa protein 1	R.TTPSYVAFTDTER.L	2	2.87	0.41	-0.70
IPI00304962	Collagen alpha-2(I) chain precursor	K.AVILQGSNDVELVAEGNSR.F	2	4.81	0.49	-1.46
IPI00304962	Collagen alpha-2(I) chain precursor	K.AVILQGSNDVELVAEGNSR.F	3	4.58	0.23	-1.95
IPI00304962	Collagen alpha-2(I) chain precursor	K.DYEVDATLK.S	2	1.93	0.11	-1.06
IPI00304962	Collagen alpha-2(I) chain precursor	K.EM*ATQLAFM*R.L	2	2.96	0.37	-2.94
IPI00304962	Collagen alpha-2(I) chain precursor	K.GVGLGPGPM*GLM*GPR.G	2	3.15	0.45	-3.39
IPI00304962	Collagen alpha-2(I) chain precursor	K.HGNRGETGPSGPVGPAGAVGPR.G	3	4.54	0.45	-3.35
IPI00304962	Collagen alpha-2(I) chain precursor	K.NSIAYM*DEETGNLKK.A	2	3.93	0.33	-2.55
IPI00304962	Collagen alpha-2(I) chain precursor	K.SLNNQIETLLTPEGSR.K	2	3.72	0.43	-5.07
IPI00304962	Collagen alpha-2(I) chain precursor	R.AQPENIPAK.N	1	1.75	0.17	-1.87
IPI00304962	Collagen alpha-2(I) chain precursor	R.GPAGPSGPAGKDGR.T	2	3.24	0.37	-3.52
IPI00304962	Collagen alpha-2(I) chain precursor	R.GPSGPQGIR.G	2	2.56	0.16	-2.37
IPI00305380	Insulin-like growth factor-binding protein 4 precursor	K.GELDCHQLADSFRE.-	3	3.11	0.28	-0.41
IPI00305380	Insulin-like growth factor-binding protein 4 precursor	K.LPGGLEPK.G	2	2.32	0.13	-3.06
IPI00305380	Insulin-like growth factor-binding protein 4 precursor	K.TGVKLPGGLEPK.G	1	2.16	0.17	-3.79
IPI00305380	Insulin-like growth factor-binding protein 4 precursor	K.TGVKLPGGLEPK.G	2	2.19	0.10	-2.81
IPI00305380	Insulin-like growth factor-binding protein 4 precursor	R.EDARPVPQGSCQSELHR.A	3	3.34	0.27	-2.56
IPI00305461	Inter-alpha-trypsin inhibitor heavy chain H2 precursor	A.DAPPQDPSCCSGALYYGSK.V	3	3.74	0.42	-1.99
IPI00305461	Inter-alpha-trypsin inhibitor heavy chain H2 precursor	K.AGELEVFNGYFVHFFAPDNLDPIPK.N	2	4.26	0.47	-4.23
IPI00305461	Inter-alpha-trypsin inhibitor heavy chain H2 precursor	K.AGELEVFNGYFVHFFAPDNLDPIPK.N	3	6.25	0.55	-7.74
IPI00305461	Inter-alpha-trypsin inhibitor heavy chain H2 precursor	K.AGELEVFNGYFVHFFAPDNLDPIPK.N	4	3.50	0.27	-3.93
IPI00305461	Inter-alpha-trypsin inhibitor heavy chain H2 precursor	K.AHVSFKPTVAQQR.I	2	3.66	0.47	-4.16
IPI00305461	Inter-alpha-trypsin inhibitor heavy chain H2 precursor	K.AHVSFKPTVAQQR.I	3	3.03	0.40	-1.98
IPI00305461	Inter-alpha-trypsin inhibitor heavy chain H2 precursor	K.DKHADPDFTR.K	1	2.36	0.32	-4.15
IPI00305461	Inter-alpha-trypsin inhibitor heavy chain H2 precursor	K.DKHADPDFTR.K	2	2.86	0.42	-3.48

IPI00305461	Inter-alpha-trypsin inhibitor heavy chain H2 precursor	K.DKHADPDFTR.K	3	2.45	0.21	-1.84
IPI00305461	Inter-alpha-trypsin inhibitor heavy chain H2 precursor	K.FDPAKLDQIESVITATSANTQLVLETLAQM*DDLQDFLSK.D	3	6.79	0.63	-4.75
IPI00305461	Inter-alpha-trypsin inhibitor heavy chain H2 precursor	K.FDPAKLDQIESVITATSANTQLVLETLAQM*DDLQDFLSK.D	4	6.84	0.49	-4.28
IPI00305461	Inter-alpha-trypsin inhibitor heavy chain H2 precursor	K.FDPAKLDQIESVITATSANTQLVLETLAQM*DDLQDFLSKDK.H	3	3.02	0.11	-3.63
IPI00305461	Inter-alpha-trypsin inhibitor heavy chain H2 precursor	K.FDPAKLDQIESVITATSANTQLVLETLAQM*DDLQDFLSKDK.H	4	4.43	0.32	-4.46
IPI00305461	Inter-alpha-trypsin inhibitor heavy chain H2 precursor	K.FQLVAENR.R	2	2.42	0.08	-1.60
IPI00305461	Inter-alpha-trypsin inhibitor heavy chain H2 precursor	K.FYNQVSTPLLR.N	1	2.60	0.32	-2.95
IPI00305461	Inter-alpha-trypsin inhibitor heavy chain H2 precursor	K.FYNQVSTPLLR.N	2	4.04	0.37	-2.93
IPI00305461	Inter-alpha-trypsin inhibitor heavy chain H2 precursor	K.HADPDFTR.K	2	2.42	0.13	-2.87
IPI00305461	Inter-alpha-trypsin inhibitor heavy chain H2 precursor	K.HLEVDVWVIEPQGLR.F	2	4.88	0.56	-3.21
IPI00305461	Inter-alpha-trypsin inhibitor heavy chain H2 precursor	K.HLEVDVWVIEPQGLR.F	3	3.22	0.13	-2.51
IPI00305461	Inter-alpha-trypsin inhibitor heavy chain H2 precursor	K.IQPSGGTNINEALLR.A	2	3.76	0.50	-2.84
IPI00305461	Inter-alpha-trypsin inhibitor heavy chain H2 precursor	K.KFYNQVSTPLLR.N	2	3.59	0.34	-3.56
IPI00305461	Inter-alpha-trypsin inhibitor heavy chain H2 precursor	K.LGSYEHR.I	2	2.40	0.07	-1.55
IPI00305461	Inter-alpha-trypsin inhibitor heavy chain H2 precursor	K.NVKENIQDNISLFSLGM*GFDVDYDFLKR.L	4	3.20	0.13	-3.14
IPI00305461	Inter-alpha-trypsin inhibitor heavy chain H2 precursor	K.RLSNENHGIAQR.I	2	3.91	0.36	-3.89
IPI00305461	Inter-alpha-trypsin inhibitor heavy chain H2 precursor	K.TAGLVR.S	1	1.70	0.10	-1.87
IPI00305461	Inter-alpha-trypsin inhibitor heavy chain H2 precursor	K.TILDDLRAEDHFSVIDFNQNIR.T	2	3.31	0.41	-2.04
IPI00305461	Inter-alpha-trypsin inhibitor heavy chain H2 precursor	K.TILDDLRAEDHFSVIDFNQNIR.T	3	5.78	0.51	-4.14
IPI00305461	Inter-alpha-trypsin inhibitor heavy chain H2 precursor	K.TILDDLRAEDHFSVIDFNQNIR.T	4	3.40	0.17	-2.66
IPI00305461	Inter-alpha-trypsin inhibitor heavy chain H2 precursor	K.VQFELHYQEVK.W	2	3.95	0.11	-3.37

IPI00305461	Inter-alpha-trypsin inhibitor heavy chain H2 precursor	K.VQSTITSR.M	1	1.83	0.09	-3.66
IPI00305461	Inter-alpha-trypsin inhibitor heavy chain H2 precursor	K.VQSTITSR.M	2	2.54	0.17	-3.47
IPI00305461	Inter-alpha-trypsin inhibitor heavy chain H2 precursor	K.VVNNSPQPQNVVFDVQIPK.G	2	5.60	0.55	-4.53
IPI00305461	Inter-alpha-trypsin inhibitor heavy chain H2 precursor	K.VVNNSPQPQNVVFDVQIPK.G	3	5.95	0.51	-2.88
IPI00305461	Inter-alpha-trypsin inhibitor heavy chain H2 precursor	L.SNENHGIAQR.I	2	3.78	0.39	-2.46
IPI00305461	Inter-alpha-trypsin inhibitor heavy chain H2 precursor	R.AEDHFSVIDFNQNIR.T	2	4.45	0.44	-2.31
IPI00305461	Inter-alpha-trypsin inhibitor heavy chain H2 precursor	R.AEDHFSVIDFNQNIR.T	3	4.52	0.17	-2.49
IPI00305461	Inter-alpha-trypsin inhibitor heavy chain H2 precursor	R.AIFILNEANNLGLLDPNSVSLIILVSDGDPTVGELK.L	3	2.73	0.36	-2.54
IPI00305461	Inter-alpha-trypsin inhibitor heavy chain H2 precursor	R.ETAVDGELVVLYDVK.R	2	4.98	0.55	-5.54
IPI00305461	Inter-alpha-trypsin inhibitor heavy chain H2 precursor	R.FLHVPDTFEGHFDGVPVISK.G	2	4.96	0.48	-4.08
IPI00305461	Inter-alpha-trypsin inhibitor heavy chain H2 precursor	R.FLHVPDTFEGHFDGVPVISK.G	3	3.96	0.44	-4.04
IPI00305461	Inter-alpha-trypsin inhibitor heavy chain H2 precursor	R.FLHVPDTFEGHFDGVPVISK.G	4	3.74	0.37	-3.36
IPI00305461	Inter-alpha-trypsin inhibitor heavy chain H2 precursor	R.IYGNQDTSSQLK.K	2	3.62	0.37	-4.56
IPI00305461	Inter-alpha-trypsin inhibitor heavy chain H2 precursor	R.IYGNQDTSSQLKK.F	2	4.54	0.23	-3.47
IPI00305461	Inter-alpha-trypsin inhibitor heavy chain H2 precursor	R.IYGNQDTSSQLKK.F	3	1.80	0.19	-2.90
IPI00305461	Inter-alpha-trypsin inhibitor heavy chain H2 precursor	R.IYLQPGR.L	1	2.09	0.11	-3.44
IPI00305461	Inter-alpha-trypsin inhibitor heavy chain H2 precursor	R.IYLQPGR.L	2	2.19	0.09	-3.37
IPI00305461	Inter-alpha-trypsin inhibitor heavy chain H2 precursor	R.LSNENHGIAQR.I	1	2.90	0.30	-3.56
IPI00305461	Inter-alpha-trypsin inhibitor heavy chain H2 precursor	R.LSNENHGIAQR.I	2	4.17	0.39	-3.65
IPI00305461	Inter-alpha-trypsin inhibitor heavy chain H2 precursor	R.LSNENHGIAQR.I	3	2.02	0.20	-5.27
IPI00305461	Inter-alpha-trypsin inhibitor heavy chain H2 precursor	R.M*ATTM*IQSK.V	1	1.36	0.09	-3.34

IPI00305461	Inter-alpha-trypsin inhibitor heavy chain H2 precursor	R.M*ATTM*IQSK.V	2	3.15	0.40	-1.73
IPI00305461	Inter-alpha-trypsin inhibitor heavy chain H2 precursor	R.M*ATTMIQSK.V	2	2.53	0.25	
IPI00305461	Inter-alpha-trypsin inhibitor heavy chain H2 precursor	R.M*LADAPPQDPSCCSGALYYGSK.V	2	4.39	0.62	-4.01
IPI00305461	Inter-alpha-trypsin inhibitor heavy chain H2 precursor	R.M*LADAPPQDPSCCSGALYYGSK.V	3	3.62	0.38	-3.80
IPI00305461	Inter-alpha-trypsin inhibitor heavy chain H2 precursor	R.MATTM*IQSK.V	2	2.81	0.26	
IPI00305461	Inter-alpha-trypsin inhibitor heavy chain H2 precursor	R.NVQFNYPHTSVTDVTQNNFHNYFGGSEIVVAGK.F	3	5.11	0.39	-3.16
IPI00305461	Inter-alpha-trypsin inhibitor heavy chain H2 precursor	R.NVQFNYPHTSVTDVTQNNFHNYFGGSEIVVAGK.F	4	4.76	0.45	-3.96
IPI00305461	Inter-alpha-trypsin inhibitor heavy chain H2 precursor	R.SILQM*SLDHHIVTPLTSLVIENEAGDER.M	3	7.09	0.54	-3.28
IPI00305461	Inter-alpha-trypsin inhibitor heavy chain H2 precursor	R.SILQM*SLDHHIVTPLTSLVIENEAGDER.M	4	4.24	0.40	-3.57
IPI00305461	Inter-alpha-trypsin inhibitor heavy chain H2 precursor	R.SLAPTAAAKR.R	2	2.42	0.12	-2.96
IPI00305461	Inter-alpha-trypsin inhibitor heavy chain H2 precursor	R.SSALDM*ENFR.T	2	3.07	0.25	-3.88
IPI00305461	Inter-alpha-trypsin inhibitor heavy chain H2 precursor	R.SSALDM*ENFRTEVNVLPGAK.V	3	3.58	0.45	-2.52
IPI00305461	Inter-alpha-trypsin inhibitor heavy chain H2 precursor	R.TEVNVLPGAK.V	1	2.45	0.22	-4.24
IPI00305461	Inter-alpha-trypsin inhibitor heavy chain H2 precursor	R.TEVNVLPGAK.V	2	3.14	0.34	-2.40
IPI00305461	Inter-alpha-trypsin inhibitor heavy chain H2 precursor	W.AYLTINQLLAER.S	2	3.75	0.39	-5.25
IPI00305461	Inter-alpha-trypsin inhibitor heavy chain H2 precursor	W.RNDLISATK.T	2	3.00	0.24	-1.11
IPI00305833	Smu-1 suppressor of mec-8 and unc-52 protein homolog	R.AAIAQALAGEVSVVPPSR.L	2	2.26	0.12	
IPI00305833	Smu-1 suppressor of mec-8 and unc-52 protein homolog	R.GEWIYCVGEDFVLYCFSTVTGK.L	3	2.98	0.15	-8.66
IPI00305975	Spondin-2 precursor	K.NQYVSNGLR.D	2	2.26	0.17	-1.31
IPI00305975	Spondin-2 precursor	K.YSITFTGK.W	1	1.46	0.23	-2.59
IPI00306046	Isoform 1 of EGF-like repeat and discoidin I-like domain-containing protein 3 precursor	K.DKVFQGNFDNDTHRK.N	3	3.17	0.22	-1.95
IPI00306322	Collagen alpha-2(IV) chain precursor	K.IAVQPGTVGPQGR.R	2	3.47	0.33	-2.25
IPI00306322	Collagen alpha-2(IV) chain precursor	K.YSFWLTTIPEQSFQGSPSADTLK.A	2	3.40	0.40	-4.27

IPI00306322	Collagen alpha-2(IV) chain precursor	R.GDPGPPGPPPVILPGMK.D	2	3.59	0.08	
IPI00306322	Collagen alpha-2(IV) chain precursor	R.GLDGYQGPDGPR.G	2	2.57	0.31	-2.99
IPI00306322	Collagen alpha-2(IV) chain precursor	R.GVSGFPGADGIPGHPGQGGPR.G	3	1.84	0.12	-1.50
IPI00306322	Collagen alpha-2(IV) chain precursor	R.RGPPGAPGEM*GPQGPPGEPGFR.G	3	3.38	0.29	-4.54
IPI00306332	60S ribosomal protein L24	-.MKVELCSFSGYKIYPGHGRR.Y	3	2.53	0.16	0.23
IPI00306339	secreted phosphoprotein 1 isoform b	A.IPVKQADSGSSEEK.Q	2	4.30	0.41	-3.26
IPI00306339	secreted phosphoprotein 1 isoform b	A.IPVKQADSGSSEEK.Q	3	3.57	0.39	-1.59
IPI00306339	secreted phosphoprotein 1 isoform b	A.NDESNEHSDVIDSQELSK.V	2	5.40	0.44	-3.67
IPI00306339	secreted phosphoprotein 1 isoform b	A.NDESNEHSDVIDSQELSK.V	3	3.92	0.45	-2.77
IPI00306339	secreted phosphoprotein 1 isoform b	H.SDVIDSQELSK.V	2	2.94	0.30	-1.60
IPI00306339	secreted phosphoprotein 1 isoform b	I.PVAQDLNAPSDWDSR.G	2	4.56	0.45	-2.57
IPI00306339	secreted phosphoprotein 1 isoform b	I.PVKQADSGSSEEK.Q	2	3.25	0.38	-1.07
IPI00306339	secreted phosphoprotein 1 isoform b	K.AIPVAQDLNAPSD.W	2	3.80	0.53	-3.31
IPI00306339	secreted phosphoprotein 1 isoform b	K.AIPVAQDLNAPSDWDSR.G	2	5.21	0.53	-4.53
IPI00306339	secreted phosphoprotein 1 isoform b	K.AIPVAQDLNAPSDWDSR.G	3	4.02	0.40	-3.55
IPI00306339	secreted phosphoprotein 1 isoform b	K.ANDESNEHSDVIDSQELSK.V	2	5.61	0.55	-3.29
IPI00306339	secreted phosphoprotein 1 isoform b	K.ANDESNEHSDVIDSQELSK.V	3	4.89	0.34	-3.60
IPI00306339	secreted phosphoprotein 1 isoform b	K.DSYETSQLDDQSAETHSHK.Q	2	5.84	0.65	-2.86
IPI00306339	secreted phosphoprotein 1 isoform b	K.DSYETSQLDDQSAETHSHK.Q	3	2.98	0.25	-1.85
IPI00306339	secreted phosphoprotein 1 isoform b	K.DSYETSQLDDQSAETHSHK.Q	4	3.22	0.27	-0.90
IPI00306339	secreted phosphoprotein 1 isoform b	K.DSYETSQLDDQSAETHSHKQS.R	2	5.31	0.57	-3.55
IPI00306339	secreted phosphoprotein 1 isoform b	K.DSYETSQLDDQSAETHSHKQSR.L	3	3.78	0.44	-4.42
IPI00306339	secreted phosphoprotein 1 isoform b	K.FRISHELDSASSE.V	2	3.47	0.35	-1.77
IPI00306339	secreted phosphoprotein 1 isoform b	K.FRISHELDSASSEVN.-	2	4.27	0.49	-4.28
IPI00306339	secreted phosphoprotein 1 isoform b	K.FRRPDIQYPDATDEDITSHM*ESEELNGAYK.A	4	4.32	0.48	-2.71
IPI00306339	secreted phosphoprotein 1 isoform b	K.QLYNKYPDAVATWLNPDPSQK.Q	3	3.40	0.26	-3.78
IPI00306339	secreted phosphoprotein 1 isoform b	K.QLYNKYPDAVATWLNPDPSQKQN.L	3	4.15	0.31	-2.77
IPI00306339	secreted phosphoprotein 1 isoform b	K.QNLLAPQTLPSK.S	1	2.22	0.32	-2.40
IPI00306339	secreted phosphoprotein 1 isoform b	K.QNLLAPQTLPSK.S	2	3.32	0.29	-4.06
IPI00306339	secreted phosphoprotein 1 isoform b	K.RKANDESNEHSDVIDSQELSK.V	3	4.24	0.40	-4.10
IPI00306339	secreted phosphoprotein 1 isoform b	K.RKANDESNEHSDVIDSQELSK.V	4	3.58	0.47	-2.79
IPI00306339	secreted phosphoprotein 1 isoform b	K.SKEEDKHLKF.R	2	2.90	0.27	-3.89
IPI00306339	secreted phosphoprotein 1 isoform b	L.DDQSAETHSHK.Q	2	3.41	0.34	-3.47
IPI00306339	secreted phosphoprotein 1 isoform b	N.DESNEHSDVIDSQELSK.V	2	5.48	0.49	-3.53
IPI00306339	secreted phosphoprotein 1 isoform b	N.EHSDVIDSQELSK.V	2	4.20	0.39	-4.84
IPI00306339	secreted phosphoprotein 1 isoform b	P.VAQDLNAPSDWDSR.G	2	4.52	0.49	-2.52
IPI00306339	secreted phosphoprotein 1 isoform b	R.EFHSHEFHSHED.M	2	2.99	0.46	-4.79
IPI00306339	secreted phosphoprotein 1 isoform b	R.EFHSHEFHSHEDM*LVVDPK.S	2	2.30	0.41	-3.82
IPI00306339	secreted phosphoprotein 1 isoform b	R.EFHSHEFHSHEDM*LVVDPK.S	3	2.79	0.21	-4.47
IPI00306339	secreted phosphoprotein 1 isoform b	R.EFHSHEFHSHEDM*LVVDPK.S	4	2.23	0.11	-2.53
IPI00306339	secreted phosphoprotein 1 isoform b	R.GDSVVYGLR.S	1	1.96	0.31	-2.54
IPI00306339	secreted phosphoprotein 1 isoform b	R.GDSVVYGLR.S	2	3.38	0.24	-3.18

IPI00306339	secreted phosphoprotein 1 isoform b	R.GKDSYETSQLDDQSAETH.S	3	3.98	0.35	-1.82
IPI00306339	secreted phosphoprotein 1 isoform b	R.GKDSYETSQLDDQSAETHS.H	2	6.10	0.61	-1.71
IPI00306339	secreted phosphoprotein 1 isoform b	R.GKDSYETSQLDDQSAETHS.H	3	3.58	0.40	-2.88
IPI00306339	secreted phosphoprotein 1 isoform b	R.GKDSYETSQLDDQSAETHSH.K	3	5.23	0.50	-2.21
IPI00306339	secreted phosphoprotein 1 isoform b	R.GKDSYETSQLDDQSAETHSHK.Q	2	6.71	0.52	-2.98
IPI00306339	secreted phosphoprotein 1 isoform b	R.GKDSYETSQLDDQSAETHSHK.Q	3	6.02	0.59	-4.98
IPI00306339	secreted phosphoprotein 1 isoform b	R.GKDSYETSQLDDQSAETHSHK.Q	4	3.89	0.25	-2.95
IPI00306339	secreted phosphoprotein 1 isoform b	R.GKDSYETSQLDDQSAETHSHK.Q	5	2.86	0.32	0.25
IPI00306339	secreted phosphoprotein 1 isoform b	R.GKDSYETSQLDDQSAETHSHKQ.S	2	5.03	0.53	-3.50
IPI00306339	secreted phosphoprotein 1 isoform b	R.GKDSYETSQLDDQSAETHSHKQ.S	3	5.41	0.55	-3.11
IPI00306339	secreted phosphoprotein 1 isoform b	R.GKDSYETSQLDDQSAETHSHKQS.R	2	6.09	0.58	-3.46
IPI00306339	secreted phosphoprotein 1 isoform b	R.GKDSYETSQLDDQSAETHSHKQS.R	3	6.61	0.56	-4.74
IPI00306339	secreted phosphoprotein 1 isoform b	R.ISHELDSASS.E	1	2.13	0.46	-2.76
IPI00306339	secreted phosphoprotein 1 isoform b	R.ISHELDSASSE.V	1	2.80	0.42	-4.07
IPI00306339	secreted phosphoprotein 1 isoform b	R.ISHELDSASSE.V	2	3.11	0.28	-2.99
IPI00306339	secreted phosphoprotein 1 isoform b	R.ISHELDSASSEVN.-	1	4.12	0.49	-4.10
IPI00306339	secreted phosphoprotein 1 isoform b	R.ISHELDSASSEVN.-	2	3.69	0.44	-4.48
IPI00306339	secreted phosphoprotein 1 isoform b	R.KANDESNEHSDVIDSQELSK.V	2	5.74	0.57	-5.59
IPI00306339	secreted phosphoprotein 1 isoform b	R.KANDESNEHSDVIDSQELSK.V	3	6.60	0.56	-4.99
IPI00306339	secreted phosphoprotein 1 isoform b	R.RPDIQYPDATDEDITSHM*ESEE.L	3	4.37	0.38	-3.52
IPI00306339	secreted phosphoprotein 1 isoform b	R.RPDIQYPDATDEDITSHM*ESEELNGAYK.A	3	6.46	0.61	-2.73
IPI00306339	secreted phosphoprotein 1 isoform b	R.RPDIQYPDATDEDITSHM*ESEELNGAYK.A	4	5.82	0.51	-2.59
IPI00306339	secreted phosphoprotein 1 isoform b	S.DVIDSQELSK.V	2	3.12	0.30	-2.68
IPI00306339	secreted phosphoprotein 1 isoform b	T.SQLDDQSAETHSHK.Q	2	3.02	0.30	-2.21
IPI00306339	secreted phosphoprotein 1 isoform b	V.AQDLNAPSDWDSR.G	2	3.84	0.43	-4.37
IPI00306339	secreted phosphoprotein 1 isoform b	W.LNPDPSQKQNLLAPQTLPSK.S	3	4.49	0.36	-2.36
IPI00306413	Tubulin polymerization-promoting protein family member 3	K.ALEELATKR.F	2	2.32	0.12	-2.54
IPI00306549	CDNA FLJ11065 fis, clone PLACE1004868, weakly similar to MALE STERILITY PROTEIN 2	R.NIHYLFNTALFLIAWRLLIAR.S	2	1.01	0.08	-7.09
IPI00306576	Arylsulfatase B precursor	R.DGEEVATGYK.N	2	2.69	0.31	-2.94
IPI00306576	Arylsulfatase B precursor	R.GVGFVASPLLK.Q	2	3.25	0.27	-2.48
IPI00306710	Isoform 1 of Chordin precursor	K.GFYGSEAQGVVK.D	2	3.13	0.39	-1.83
IPI00306710	Isoform 1 of Chordin precursor	K.GFYGSEAQGVVKDLEPELLR.H	3	3.04	0.26	-2.10
IPI00306710	Isoform 1 of Chordin precursor	R.CVLCACEAPQWGR.R	2	2.71	0.17	
IPI00306710	Isoform 1 of Chordin precursor	R.LQILHQGQLLR.E	3	2.57	0.17	-3.18
IPI00306710	Isoform 1 of Chordin precursor	R.QLPGHCCQTCPQER.S	3	2.54	0.22	
IPI00306844	Corticotropin-releasing factor-binding protein precursor	K.VFDGWILK.G	2	2.64	0.13	-1.45
IPI00306844	Corticotropin-releasing factor-binding protein precursor	R.SSQNVAM*IFFR.V	2	2.52	0.27	-2.81
IPI00306850	EGF-like-domain, multiple 3	R.GYELDTDQR.T	2	2.53	0.30	-3.90

IPI00306853	Carbohydrate sulfotransferase 3	R.SVSLLEER.G	2	2.45	0.13	-2.39
IPI00306884	CDNA FLJ11867 fis, clone HEMBA1006976, weakly similar to H.sapiens Gal-beta(1-3/1-4)GlcNAc alpha-2.3-sialyltransferase	K.NIQRLNNAPVAGYEGDVGSKTTM*R.L	5	2.26	0.12	-7.74
IPI00306959	Keratin, type II cytoskeletal 7	K.LLEGEESR.I	2	2.26	0.06	-3.07
IPI00307276	ADAMTS-4 precursor	K.APLGSPSPRPR.R	2	3.08	0.21	-2.39
IPI00307276	ADAMTS-4 precursor	R.GAELHLQPLEGGTPNSAGGPGAHILR.R	2	5.44	0.55	-3.92
IPI00307276	ADAMTS-4 precursor	R.GAELHLQPLEGGTPNSAGGPGAHILR.R	3	6.84	0.56	-4.76
IPI00307276	ADAMTS-4 precursor	R.GAELHLQPLEGGTPNSAGGPGAHILR.R	4	3.99	0.24	-3.86
IPI00307276	ADAMTS-4 precursor	R.GAELHLQPLEGGTPNSAGGPGAHILRR.K	3	2.74	0.19	-4.20
IPI00307591	Zinc finger protein 609	K.MEGLLNGSSDPHQSR.L	2	2.31	0.10	
IPI00307592	ATP-binding cassette, sub-family A, member 2 isoform a	L.SAGPGTSGSHLDR.S	2	3.29	0.43	-3.39
IPI00307592	ATP-binding cassette, sub-family A, member 2 isoform a	R.DAVCSGQAAAR.A	2	3.05	0.25	-2.28
IPI00307592	ATP-binding cassette, sub-family A, member 2 isoform a	R.DAVCSGQAAARA.R	2	3.72	0.41	-3.22
IPI00307592	ATP-binding cassette, sub-family A, member 2 isoform a	R.FSGLSAELR.N	2	2.39	0.19	-1.24
IPI00307592	ATP-binding cassette, sub-family A, member 2 isoform a	R.ILTVPESQK.G	1	2.19	0.07	-1.84
IPI00307592	ATP-binding cassette, sub-family A, member 2 isoform a	R.ILTVPESQK.G	2	2.34	0.08	-1.18
IPI00307611	Isoform 1 of Microtubule-associated serine/threonine-protein kinase 4	K.QTLSPKHPKPSTVK.D	2	2.70	0.06	
IPI00307612	Cadherin-20 precursor	K.IQDINDNEPK.F	2	3.38	0.25	-3.72
IPI00307702	H53_GS1 (Fragment)	R.EMDAAGFDFSLPCTQKLTQNGTR.S	3	2.96	0.10	1.18
IPI00307729	ADAMTS-3 precursor	R.YREYELVTPVSTNLEGR.Y	3	2.59	0.09	-3.65
IPI00328113	Fibrillin-1 precursor	G.ADANLEAGNVK.E	2	3.34	0.34	-1.45
IPI00328113	Fibrillin-1 precursor	K.GYILQEDGR.S	2	2.78	0.19	-2.41
IPI00328113	Fibrillin-1 precursor	K.ILCPGGEGFRPNPITVILEDIDECQELPGLCQGGK.C	3	4.31	0.44	-3.71
IPI00328113	Fibrillin-1 precursor	R.AGYQSTLTR.T	2	3.20	0.26	0.06
IPI00328113	Fibrillin-1 precursor	R.ILELLPALTTLTNHNR.Y	3	2.92	0.15	-1.66
IPI00328243	Phospholipase D3	K.ALLNVVDNAR.S	1	2.16	0.20	-4.20
IPI00328243	Phospholipase D3	K.ALLNVVDNAR.S	2	3.71	0.30	-2.64
IPI00328243	Phospholipase D3	K.LFVVPADEAQAR.I	2	3.44	0.28	-3.38
IPI00328243	Phospholipase D3	K.LTHGVLHTK.F	2	2.18	0.11	-2.26
IPI00328243	Phospholipase D3	R.IAVSKPSGPQPQADLQALLQSGAQVR.M	3	5.35	0.40	-2.75
IPI00328243	Phospholipase D3	R.IAVSKPSGPQPQADLQALLQSGAQVR.M	4	3.08	0.13	-2.78
IPI00328243	Phospholipase D3	R.SQLEAIFLR.D	2	3.67	0.28	-2.14
IPI00328257	Isoform A of AP-1 complex subunit beta-1	K.DIPNENEAQFQIR.D	2	3.75	0.30	-4.40
IPI00328257	Isoform A of AP-1 complex subunit beta-1	K.LQSSNIFTVAK.R	2	2.10	0.12	-3.05

IPI00328257	Isoform A of AP-1 complex subunit beta-1	K.M*EPLNNLQVAVK.N	2	3.13	0.30	-3.02
IPI00328257	Isoform A of AP-1 complex subunit beta-1	K.RNVEGQDM*LYQSLK.L	3	2.64	0.29	-1.14
IPI00328257	Isoform A of AP-1 complex subunit beta-1	R.NSFGLAPAAPLQVHAPLSPNQTVEISLPLSTVGSVM*K.M	3	4.93	0.52	-4.57
IPI00328257	Isoform A of AP-1 complex subunit beta-1	R.NVEGQDM*LYQSLK.L	2	3.99	0.46	-3.71
IPI00328260	Protein FAN	K.VWSGVPAEM*PGTK.R	2	2.12	0.19	
IPI00328270	Neuronal PAS domain-containing protein 2	R.DQFNVLIKELSSMLPGNTRK.M	3	3.76	0.06	
IPI00328298	Isoform 2 of Structural maintenance of chromosomes protein 4	R.AQKIRSKKLSVLIHNSDEHK.D	3	2.30	0.14	-3.01
IPI00328361	Seryl-tRNA synthetase, mitochondrial precursor	R.RSFTTEKRNRNLLYEYAR.E	3	3.56	0.13	
IPI00328391	N-acetylgalactosaminyltransferase 7	K.AKPLVLGPEFK.Q	1	2.52	0.29	-5.07
IPI00328391	N-acetylgalactosaminyltransferase 7	K.AKPLVLGPEFK.Q	3	1.88	0.12	-6.10
IPI00328391	N-acetylgalactosaminyltransferase 7	K.EPEPPGVVGGPGEK.A	2	2.80	0.36	-3.35
IPI00328391	N-acetylgalactosaminyltransferase 7	K.NVDWGEIR.G	2	2.99	0.18	-1.91
IPI00328391	N-acetylgalactosaminyltransferase 7	K.RVPLTPQEK.R	2	2.45	0.16	-2.30
IPI00328391	N-acetylgalactosaminyltransferase 7	K.WFM*EEIAYDITSHYPLPPK.N	3	4.06	0.43	-2.75
IPI00328391	N-acetylgalactosaminyltransferase 7	R.DVNDPM*PNR.G	2	2.52	0.14	-2.95
IPI00328391	N-acetylgalactosaminyltransferase 7	R.FTHIPSGK.C	1	1.71	0.13	-4.35
IPI00328391	N-acetylgalactosaminyltransferase 7	R.GFETAYCIDSM*GK.T	2	4.10	0.54	-4.49
IPI00328391	N-acetylgalactosaminyltransferase 7	R.INEANQLM*QYDQCLTK.G	2	5.70	0.54	-3.10
IPI00328391	N-acetylgalactosaminyltransferase 7	R.KYLAEIVLIDDFSNKEHLK.E	4	3.14	0.08	-3.37
IPI00328391	N-acetylgalactosaminyltransferase 7	R.M*GGNQLFR.I	2	3.00	0.21	-1.83
IPI00328391	N-acetylgalactosaminyltransferase 7	R.SEVLHQVFISNCDSSK.T	3	3.56	0.33	-2.22
IPI00328391	N-acetylgalactosaminyltransferase 7	R.SPAM*AGGLFAIER.E	2	3.67	0.36	-2.34
IPI00328431	Isoform 1 of Netrin receptor UNC5B precursor	K.CNGEWVSQNDHVTQEGLDEATGLR.V	3	4.72	0.45	-2.66
IPI00328431	Isoform 1 of Netrin receptor UNC5B precursor	K.NEDVIDPTQDTNFLLTIDHNLIIR.Q	3	2.95	0.14	-6.43
IPI00328488	Isoform 1 of Epididymis-specific alpha-mannosidase precursor	K.QGPISDNYLFTPGK.A	2	3.51	0.37	-3.91
IPI00328488	Isoform 1 of Epididymis-specific alpha-mannosidase precursor	R.AYAANVYTSVVEELAR.G	2	3.76	0.51	-3.64
IPI00328488	Isoform 1 of Epididymis-specific alpha-mannosidase precursor	R.DM*YATHLASGM*LGVR.K	2	2.95	0.48	-2.55
IPI00328488	Isoform 1 of Epididymis-specific alpha-mannosidase precursor	R.FIAVEQEFFR.L	2	3.80	0.40	-3.44
IPI00328488	Isoform 1 of Epididymis-specific alpha-mannosidase precursor	R.IEQEYQAGPLELNR.E	2	4.31	0.19	-3.64
IPI00328488	Isoform 1 of Epididymis-specific alpha-mannosidase precursor	R.RASALLYAGESM*FTR.Y	3	2.71	0.18	-0.90
IPI00328488	Isoform 1 of Epididymis-specific alpha-mannosidase precursor	R.SALALQHRPVVLFGDLAGTAPK.L	3	5.93	0.48	-1.28
IPI00328488	Isoform 1 of Epididymis-specific alpha-mannosidase precursor	R.TFFIHFQQQ.-	2	2.76	0.33	-1.63
IPI00328520	Isoform 2 of Proline-rich transmembrane protein 2	K.AGLAPETTETPAGASETAQATDLSLSPGGESK.A	2	4.64	0.55	-4.11

IPI00328520	Isoform 2 of Proline-rich transmembrane protein 2	K.AGLAPETTETPAGASETAQATDLSLSPGGESK.A	3	3.61	0.46	-2.78
IPI00328520	Isoform 2 of Proline-rich transmembrane protein 2	L.AGVPDQPEAPQPGPNTTAAPVDSGPK.A	2	3.97	0.47	-2.84
IPI00328522	KTEL motif-containing protein 1	R.KNTKLVDAEYTK.N	2	2.62	0.12	
IPI00328550	Thrombospondin-4 precursor	K.DVDIDSYPDEELPCSAR.N	2	3.13	0.40	
IPI00328550	Thrombospondin-4 precursor	R.AFAGPSQKPETIELR.T	2	2.50	0.20	
IPI00328550	Thrombospondin-4 precursor	R.AFAGPSQKPETIELR.T	3	2.16	0.16	-1.91
IPI00328550	Thrombospondin-4 precursor	R.AVAEPGIQLK.A	1	2.29	0.16	-3.43
IPI00328550	Thrombospondin-4 precursor	R.AVAEPGIQLK.A	2	2.38	0.20	-2.73
IPI00328550	Thrombospondin-4 precursor	R.KPQDFLEELK.L	2	2.64	0.15	-1.99
IPI00328550	Thrombospondin-4 precursor	R.KPQDFLEELKLVVR.G	3	3.53	0.19	-1.04
IPI00328550	Thrombospondin-4 precursor	R.KPQDFLEELKLVVR.G	4	2.59	0.12	-0.87
IPI00328550	Thrombospondin-4 precursor	R.LNPGALLPVLTDPALNDLYVISTFK.L	3	4.38	0.57	-4.04
IPI00328550	Thrombospondin-4 precursor	R.QFLGQM*TQLNQLLGEVK.D	2	3.94	0.31	-4.87
IPI00328587	Enolase	K.KLNVTEQEK.I	2	2.25	0.12	-1.08
IPI00328587	Enolase	K.LM*IEM*DGTENK.S	2	2.73	0.23	-3.68
IPI00328609	Kallistatin precursor	K.ATLDVDEAGTEAAAATSFAIK.F	2	6.29	0.58	-4.18
IPI00328609	Kallistatin precursor	K.ATLDVDEAGTEAAAATSFAIKFFSAQTNR.H	3	4.53	0.52	-4.11
IPI00328609	Kallistatin precursor	K.DFYVDENTTVR.V	2	3.50	0.38	-2.64
IPI00328609	Kallistatin precursor	K.FFSAQTNR.H	1	1.91	0.09	0.90
IPI00328609	Kallistatin precursor	K.FFSAQTNR.H	2	2.82	0.16	2.52
IPI00328609	Kallistatin precursor	K.FSISGSYVLDQILPR.L	2	4.75	0.40	-4.62
IPI00328609	Kallistatin precursor	K.FSISGSYVLDQILPR.L	3	5.05	0.33	-3.37
IPI00328609	Kallistatin precursor	K.GDATVFFILPNQGK.M	2	3.77	0.28	-3.71
IPI00328609	Kallistatin precursor	K.IAPANADFAFR.F	1	2.49	0.28	-3.31
IPI00328609	Kallistatin precursor	K.IAPANADFAFR.F	2	3.00	0.45	-3.37
IPI00328609	Kallistatin precursor	K.IVDLVSELKK.D	2	2.95	0.30	-2.96
IPI00328609	Kallistatin precursor	K.LFHTNFYDTVGTIQLINDHVK.K	2	6.05	0.49	-4.09
IPI00328609	Kallistatin precursor	K.LFHTNFYDTVGTIQLINDHVK.K	3	4.83	0.49	-5.97
IPI00328609	Kallistatin precursor	K.LFHTNFYDTVGTIQLINDHVK.K	4	3.61	0.34	-4.23
IPI00328609	Kallistatin precursor	K.M*REIEEVLTPEM*LM*R.W	2	1.54	0.10	-3.39
IPI00328609	Kallistatin precursor	K.VVDPTKP.-	1	1.59	0.06	-3.66
IPI00328609	Kallistatin precursor	K.WADLSGITK.Q	1	1.79	0.05	-3.32
IPI00328609	Kallistatin precursor	K.WADLSGITK.Q	2	3.00	0.24	-2.00
IPI00328609	Kallistatin precursor	R.EIEEVLTPEM*LM*R.W	2	4.09	0.42	-4.53
IPI00328609	Kallistatin precursor	R.FYYLIASETPGK.N	2	4.15	0.42	-5.15
IPI00328609	Kallistatin precursor	R.GKIVDLVSELKK.D	2	3.69	0.33	-4.05
IPI00328609	Kallistatin precursor	R.GKIVDLVSELKK.D	3	4.62	0.38	-3.68
IPI00328609	Kallistatin precursor	R.LGFTDLFSK.W	1	2.38	0.15	-4.02
IPI00328609	Kallistatin precursor	R.LGFTDLFSK.W	2	3.20	0.34	-2.33
IPI00328609	Kallistatin precursor	R.M*DYKGDATVFFILPNQGK.M	2	4.58	0.51	-3.78
IPI00328609	Kallistatin precursor	R.M*DYKGDATVFFILPNQGK.M	3	4.60	0.35	-2.87
IPI00328609	Kallistatin precursor	R.TTPKDFYVDENTTVR.V	3	3.45	0.32	-2.91

IPI00328609	Kallistatin precursor	R.VGSALFLSHNLK.F	2	3.33	0.26	-2.32
IPI00328609	Kallistatin precursor	R.VGSALFLSHNLK.F	3	2.17	0.32	-4.25
IPI00328680	Multiple coagulation factor deficiency protein 2 precursor	A.EEPAASFSQPGSM*GLDK.N	2	4.76	0.54	-4.26
IPI00328680	Multiple coagulation factor deficiency protein 2 precursor	K.NTVHDQEHIM*EHLEGVINKPEAEM*SPQELQLHYFK.M	4	4.85	0.35	-2.83
IPI00328680	Multiple coagulation factor deficiency protein 2 precursor	K.NTVHDQEHIM*EHLEGVINKPEAEM*SPQELQLHYFK.M	5	3.80	0.31	-3.76
IPI00328680	Multiple coagulation factor deficiency protein 2 precursor	K.NTVHDQEHIM*EHLEGVINKPEAEM*SPQELQLHYFK.M	6	3.53	0.28	-1.12
IPI00328703	Out at first protein homolog precursor	K.ASEQAELPR.C	2	2.48	0.13	0.92
IPI00328703	Out at first protein homolog precursor	K.GQSQFQALCFVTQLQHNEIIPSEAM*AK.L	3	4.38	0.41	-0.36
IPI00328703	Out at first protein homolog precursor	R.FWLEQGVDSSVFEALPK.A	2	5.39	0.30	
IPI00328709	Gremlin-2 precursor	K.NRPAGAIPSPYK.D	2	2.17	0.19	-4.01
IPI00328745	Reticulon-4 receptor-like 1 precursor	K.LHALYLYK.C	2	2.14	0.20	-1.68
IPI00328745	Reticulon-4 receptor-like 1 precursor	K.LWSLGPGTFR.G	2	2.96	0.30	-2.31
IPI00328745	Reticulon-4 receptor-like 1 precursor	K.SHTLTTTDR.A	2	1.72	0.15	0.55
IPI00328745	Reticulon-4 receptor-like 1 precursor	R.GSSSAVPCVSPGLR.H	2	1.92	0.18	-1.70
IPI00328745	Reticulon-4 receptor-like 1 precursor	R.TLAPETFQGLVK.L	2	3.26	0.23	-3.28
IPI00328746	Reticulon-4 receptor-like 2 precursor	H.LYGVAEAGAPPADPSTLYR.D	2	4.74	0.46	-4.79
IPI00328746	Reticulon-4 receptor-like 2 precursor	L.YGVAEAGAPPADPSTLYR.D	2	3.48	0.38	-4.83
IPI00328746	Reticulon-4 receptor-like 2 precursor	R.CQLSSLPGNIFR.G	2	3.67	0.39	-2.33
IPI00328746	Reticulon-4 receptor-like 2 precursor	R.DLPAEDSR.G	1	1.54	0.09	-4.30
IPI00328746	Reticulon-4 receptor-like 2 precursor	R.DLPAEDSR.G	2	1.66	0.18	-2.66
IPI00328746	Reticulon-4 receptor-like 2 precursor	R.HLQALEELDLGDNR.H	3	2.69	0.10	-1.78
IPI00328746	Reticulon-4 receptor-like 2 precursor	R.LFLQNNLIR.T	2	3.22	0.12	-2.63
IPI00328746	Reticulon-4 receptor-like 2 precursor	R.LLLHGNR.L	2	1.74	0.13	-3.57
IPI00328746	Reticulon-4 receptor-like 2 precursor	R.LLTEHVFR.G	2	2.49	0.28	-3.60
IPI00328746	Reticulon-4 receptor-like 2 precursor	R.LQSLHLYR.C	1	2.15	0.18	-3.76
IPI00328746	Reticulon-4 receptor-like 2 precursor	R.LQSLHLYR.C	2	2.33	0.06	-1.75
IPI00328746	Reticulon-4 receptor-like 2 precursor	R.SLEPDTFQGLER.L	2	3.53	0.37	-4.37
IPI00328746	Reticulon-4 receptor-like 2 precursor	R.VSSSDVTCATPPER.Q	2	3.09	0.40	-4.69
IPI00328746	Reticulon-4 receptor-like 2 precursor	R.VSSSDVTCATPPER.Q	3	2.19	0.21	-3.79
IPI00328826	Uncharacterized protein CADPS2	K.TYDTVHRRLTVEEATASVSEGGGLQGITM*K.D	3	2.59	0.14	-8.06
IPI00328826	Uncharacterized protein CADPS2	R.KCLEKAALINYTR.L	2	2.06	0.20	
IPI00328829	inter-alpha trypsin inhibitor heavy chain precursor 5 isoform 1	D.PHFVVDFPLSR.L	1	2.99	0.28	-3.59
IPI00328829	inter-alpha trypsin inhibitor heavy chain precursor 5 isoform 1	D.PHFVVDFPLSR.L	2	3.87	0.36	-4.64
IPI00328829	inter-alpha trypsin inhibitor heavy chain precursor 5 isoform 1	D.PHFVVDFPLSR.L	3	3.75	0.30	-2.54

IPI00328829	inter-alpha trypsin inhibitor heavy chain precursor 5 isoform 1	K.AAFFLSYEELLQR.R	2	4.19	0.32	-6.66
IPI00328829	inter-alpha trypsin inhibitor heavy chain precursor 5 isoform 1	K.AAFFLSYEELLQR.R	3	3.76	0.22	-2.30
IPI00328829	inter-alpha trypsin inhibitor heavy chain precursor 5 isoform 1	K.DHLISVTPDSIR.D	2	2.95	0.28	-1.94
IPI00328829	inter-alpha trypsin inhibitor heavy chain precursor 5 isoform 1	K.DVTGSPRPGGDGEGDTNHIER.L	3	3.98	0.44	-0.97
IPI00328829	inter-alpha trypsin inhibitor heavy chain precursor 5 isoform 1	K.ELLSSWLQSDDEPEKER.L	2	4.77	0.44	-2.35
IPI00328829	inter-alpha trypsin inhibitor heavy chain precursor 5 isoform 1	K.IYNGEEQIDCWFAR.N	2	4.57	0.46	-3.46
IPI00328829	inter-alpha trypsin inhibitor heavy chain precursor 5 isoform 1	K.LSLENCGLTR.R	2	2.49	0.16	-1.27
IPI00328829	inter-alpha trypsin inhibitor heavy chain precursor 5 isoform 1	K.TDVPVRPQK.A	2	2.42	0.09	-3.18
IPI00328829	inter-alpha trypsin inhibitor heavy chain precursor 5 isoform 1	K.TSVDGDPHFVVDFPLSR.L	3	3.17	0.40	-2.41
IPI00328829	inter-alpha trypsin inhibitor heavy chain precursor 5 isoform 1	K.VYIHHM*SPTGGTDINGALQR.A	3	4.03	0.43	-4.42
IPI00328829	inter-alpha trypsin inhibitor heavy chain precursor 5 isoform 1	K.VYQGEITER.E	2	3.40	0.22	-0.73
IPI00328829	inter-alpha trypsin inhibitor heavy chain precursor 5 isoform 1	K.YVAHSGIGDR.S	1	2.32	0.28	-5.40
IPI00328829	inter-alpha trypsin inhibitor heavy chain precursor 5 isoform 1	K.YVAHSGIGDR.S	2	2.55	0.27	-0.58
IPI00328829	inter-alpha trypsin inhibitor heavy chain precursor 5 isoform 1	R.AQALAVSYR.F	1	2.16	0.16	-3.43
IPI00328829	inter-alpha trypsin inhibitor heavy chain precursor 5 isoform 1	R.AQALAVSYR.F	2	2.77	0.17	-1.76
IPI00328829	inter-alpha trypsin inhibitor heavy chain precursor 5 isoform 1	R.ASAVIPSKDK.A	2	2.72	0.23	-2.59
IPI00328829	inter-alpha trypsin inhibitor heavy chain precursor 5 isoform 1	R.EQSIGDIQVLNGYFVHYFAPK.D	2	3.69	0.42	-2.36
IPI00328829	inter-alpha trypsin inhibitor heavy chain precursor 5 isoform 1	R.FLTPFTSM*K.L	1	1.68	0.19	-2.21
IPI00328829	inter-alpha trypsin inhibitor heavy chain precursor 5 isoform 1	R.FSIIGFSNR.I	2	3.29	0.29	-1.69
IPI00328829	inter-alpha trypsin inhibitor heavy chain precursor 5 isoform 1	R.IAQNGILGDFIIR.Y	2	4.08	0.40	-3.97
IPI00328829	inter-alpha trypsin inhibitor heavy chain precursor 5 isoform 1	R.IAQNGILGDFIIR.Y	3	4.27	0.31	-2.79

IPI00328829	inter-alpha trypsin inhibitor heavy chain precursor 5 isoform 1	R.IDYPPSSVVQATK.T	2	3.31	0.37	-3.34
IPI00328829	inter-alpha trypsin inhibitor heavy chain precursor 5 isoform 1	R.KIYNGEEQIDCWFAR.N	2	3.89	0.50	-4.04
IPI00328829	inter-alpha trypsin inhibitor heavy chain precursor 5 isoform 1	R.LTVCFNIDGQPGDILR.L	2	4.70	0.43	-3.00
IPI00328829	inter-alpha trypsin inhibitor heavy chain precursor 5 isoform 1	R.LWSYLTTK.E	1	1.63	0.28	-3.38
IPI00328829	inter-alpha trypsin inhibitor heavy chain precursor 5 isoform 1	R.LWSYLTTK.E	2	2.33	0.10	-2.26
IPI00328829	inter-alpha trypsin inhibitor heavy chain precursor 5 isoform 1	R.M*DGLEEAHGM*SAAM*GPEPVVQSVR.G	3	6.47	0.57	-2.52
IPI00328829	inter-alpha trypsin inhibitor heavy chain precursor 5 isoform 1	R.RVHEEEDAGSQLIGFYDEIR.T	3	3.06	0.29	-4.25
IPI00328829	inter-alpha trypsin inhibitor heavy chain precursor 5 isoform 1	R.RVHEEEDAGSQLIGFYDEIR.T	4	3.92	0.27	-3.97
IPI00328829	inter-alpha trypsin inhibitor heavy chain precursor 5 isoform 1	R.SVSLIVFLTDGKPTVGETHTLK.I	3	3.66	0.49	-4.68
IPI00328829	inter-alpha trypsin inhibitor heavy chain precursor 5 isoform 1	R.SYLEITPSR.V	1	1.93	0.11	-3.19
IPI00328829	inter-alpha trypsin inhibitor heavy chain precursor 5 isoform 1	R.SYLEITPSR.V	2	2.90	0.18	-1.63
IPI00328829	inter-alpha trypsin inhibitor heavy chain precursor 5 isoform 1	R.TITILINKPER.S	2	2.88	0.05	-3.69
IPI00328829	inter-alpha trypsin inhibitor heavy chain precursor 5 isoform 1	R.TPLLSDIR.I	1	2.10	0.14	-2.76
IPI00328829	inter-alpha trypsin inhibitor heavy chain precursor 5 isoform 1	R.TPLLSDIR.I	2	2.74	0.26	-1.05
IPI00328829	inter-alpha trypsin inhibitor heavy chain precursor 5 isoform 1	R.TPLLSDIRIDYPPSSVVQATK.T	3	3.97	0.39	-1.34
IPI00328829	inter-alpha trypsin inhibitor heavy chain precursor 5 isoform 1	R.VHEEEDAGSQLIGFYDEIR.T	2	5.62	0.56	-2.43
IPI00328829	inter-alpha trypsin inhibitor heavy chain precursor 5 isoform 1	R.VHEEEDAGSQLIGFYDEIR.T	3	3.71	0.29	-2.09
IPI00328829	inter-alpha trypsin inhibitor heavy chain precursor 5 isoform 1	R.YDVNREQSIGDIQVLNGYFVHYFAPK.D	4	3.88	0.25	-2.76
IPI00329332	Syntaxin-12	K.ETNELLKELGSLPLPLSTSEQR.Q	2	3.99	0.48	-1.46
IPI00329332	Syntaxin-12	K.ETNELLKELGSLPLPLSTSEQR.Q	3	3.49	0.19	-3.33
IPI00329332	Syntaxin-12	R.DFSSIIQTCSGNIQR.I	2	4.96	0.43	-3.29
IPI00329352	Nodal modulator 1 precursor	K.FLLFSSLVTK.E	2	2.80	0.27	-3.53
IPI00329352	Nodal modulator 1 precursor	K.GGDINFVFTGFSVNGK.V	2	4.41	0.55	-4.71
IPI00329352	Nodal modulator 1 precursor	K.GQPLGPAGVQVSLR.N	2	2.62	0.36	-3.56

IPI00329352	Nodal modulator 1 precursor	K.VNAM*TFTFDNVLPGK.Y	2	2.50	0.29	-2.47
IPI00329352	Nodal modulator 1 precursor	K.VVLSSQDKDK.S	2	2.64	0.28	-2.45
IPI00329352	Nodal modulator 1 precursor	K.YQTDCAPNNGYFM*IPLYDKGDFILK.I	3	3.03	0.28	-3.41
IPI00329352	Nodal modulator 1 precursor	R.EDGSFSFYSLPSGGYTVIPFYR.G	2	3.75	0.41	-2.64
IPI00329352	Nodal modulator 1 precursor	V.LGQAASDNSGPEDAKR.Q	2	3.92	0.46	-2.22
IPI00329482	Isoform 1 of Laminin subunit alpha-4 precursor	K.DVEIPLDSKPVSSWPAYFSIVK.I	3	2.85	0.17	-3.59
IPI00329482	Isoform 1 of Laminin subunit alpha-4 precursor	K.IQINNAENTM*K.S	2	2.53	0.10	-2.84
IPI00329482	Isoform 1 of Laminin subunit alpha-4 precursor	K.LSDLQEALDQALNHVR.D	3	4.11	0.32	-3.13
IPI00329482	Isoform 1 of Laminin subunit alpha-4 precursor	K.SLLSDVEELVEKENQASR.K	3	3.49	0.40	-2.54
IPI00329482	Isoform 1 of Laminin subunit alpha-4 precursor	R.IRELIAQTR.S	2	2.92	0.21	-1.55
IPI00329482	Isoform 1 of Laminin subunit alpha-4 precursor	R.TLFPVVLEQLDDYNAK.L	2	5.06	0.49	-3.56
IPI00329538	Prostasin precursor	K.LGAHQLDSYSEDAK.V	3	3.34	0.26	-0.42
IPI00329538	Prostasin precursor	K.VSTLKDIIPHPSYLQEGSQGDIALLQLSRPITFSR.Y	4	3.61	0.28	-3.82
IPI00329593	Isoform 2 of ADP-dependent glucokinase	R.FFSDKETFHDIAQVASEFPGAQHYVGGNAALIGQK.F	4	5.10	0.43	-5.51
IPI00329605	DNA mismatch repair protein Msh3	R.M*GAADNIYKGR.S	2	2.08	0.05	-1.35
IPI00329685	Putative uncharacterized protein DKFZp686G12235	K.AQLDAAVTFGPSQVAR.G	2	3.47	0.29	-0.49
IPI00329685	Putative uncharacterized protein DKFZp686G12235	R.GGLPLEEVTVAEVLAAR.G	3	2.96	0.25	-2.38
IPI00329685	Putative uncharacterized protein DKFZp686G12235	R.GYLTGM*AGK.W	2	1.86	0.05	-1.89
IPI00329685	Putative uncharacterized protein DKFZp686G12235	R.QSLFFYPSYPDEVR.G	2	4.70	0.46	-3.58
IPI00329685	Putative uncharacterized protein DKFZp686G12235	R.YM*AFAHDLM*ADAQR.Q	3	2.91	0.28	
IPI00329688	Protein YIPF3	R.DIPAM*LPAAR.L	1	1.72	0.14	-4.06
IPI00329688	Protein YIPF3	R.DIPAM*LPAAR.L	2	2.19	0.29	-2.86
IPI00329775	Isoform 1 of Carboxypeptidase B2 precursor	A.FQSGQVLAALPR.T	1	3.25	0.30	-3.66
IPI00329775	Isoform 1 of Carboxypeptidase B2 precursor	A.FQSGQVLAALPR.T	2	3.62	0.37	-3.46
IPI00329775	Isoform 1 of Carboxypeptidase B2 precursor	K.AVASFLRR.N	2	1.81	0.16	-3.53
IPI00329775	Isoform 1 of Carboxypeptidase B2 precursor	K.AYISM*HSYSQHIVFPYSY.T	2	3.52	0.48	-4.22
IPI00329775	Isoform 1 of Carboxypeptidase B2 precursor	K.AYISM*HSYSQHIVFPYSYTR.S	3	4.72	0.43	-3.35
IPI00329775	Isoform 1 of Carboxypeptidase B2 precursor	K.AYISM*HSYSQHIVFPYSYTR.S	4	3.12	0.31	-3.57
IPI00329775	Isoform 1 of Carboxypeptidase B2 precursor	K.IHIGSSFEK.Y	1	2.54	0.35	-3.77
IPI00329775	Isoform 1 of Carboxypeptidase B2 precursor	K.IHIGSSFEKYPLYVLK.V	2	5.09	0.59	-3.98
IPI00329775	Isoform 1 of Carboxypeptidase B2 precursor	K.IHIGSSFEKYPLYVLK.V	3	2.59	0.16	-3.01
IPI00329775	Isoform 1 of Carboxypeptidase B2 precursor	K.NAIWIDCGIHAR.E	3	2.96	0.18	-2.63
IPI00329775	Isoform 1 of Carboxypeptidase B2 precursor	K.SKDHEELSLVASEAVR.A	3	2.94	0.23	-3.99
IPI00329775	Isoform 1 of Carboxypeptidase B2 precursor	K.SKDHEELSLVASEAVR.A	4	2.65	0.18	-4.15
IPI00329775	Isoform 1 of Carboxypeptidase B2 precursor	K.YPLYVLK.V	2	1.91	0.07	-2.34
IPI00329775	Isoform 1 of Carboxypeptidase B2 precursor	K.YSFTIELR.D	2	2.29	0.22	-1.28
IPI00329775	Isoform 1 of Carboxypeptidase B2 precursor	R.DTGTYGFLLPER.Y	2	3.88	0.34	-4.39

IPI00329775	Isoform 1 of Carboxypeptidase B2 precursor	R.EAFAAVSK.I	1	2.04	0.13	-2.97
IPI00329775	Isoform 1 of Carboxypeptidase B2 precursor	R.HPDM*LTK.I	2	1.60	0.12	-2.88
IPI00329775	Isoform 1 of Carboxypeptidase B2 precursor	R.LVDFYVM*PVVNVDGYDYSWK.K	2	3.98	0.44	-4.00
IPI00329775	Isoform 1 of Carboxypeptidase B2 precursor	R.YTHGHGSETLYLAPGGGDDWIYDLGIK.Y	3	4.58	0.35	-3.71
IPI00329775	Isoform 1 of Carboxypeptidase B2 precursor	R.YTHGHGSETLYLAPGGGDDWIYDLGIK.Y	4	3.26	0.16	-3.95
IPI00329791	cDNA FLJ78679, highly similar to Homo sapiens DEAD (Asp-Glu-Ala-Asp) box polypeptide 46 (DDX46), mRNA	K.EKTDGGESSKEKKK.D	2	3.11	0.09	
IPI00329801	Annexin A5	K.GLGTDEESILTLLTSR.S	3	2.49	0.21	-3.18
IPI00329801	Annexin A5	K.NFATSLYSM*IK.G	2	4.02	0.46	-1.11
IPI00329801	Annexin A5	K.VLTEIIASR.T	2	2.95	0.27	-3.84
IPI00329801	Annexin A5	R.DLLDDLKSELTGKFEK.L	3	3.11	0.29	-2.04
IPI00329801	Annexin A5	R.SEIDLFNIR.K	2	2.43	0.22	-2.63
IPI00333126	Leucine-rich repeat-containing protein 56	R.HPESQQEGAVAPWGPRR.V	2	2.35	0.13	
IPI00333140	Delta and Notch-like epidermal growth factor-related receptor precursor	A.NPVPAAPLSAPGPCAAQPCR.N	2	4.50	0.55	-3.55
IPI00333140	Delta and Notch-like epidermal growth factor-related receptor precursor	A.NPVPAAPLSAPGPCAAQPCR.N	3	3.88	0.41	-2.71
IPI00333140	Delta and Notch-like epidermal growth factor-related receptor precursor	K.IDYCILDPCR.N	1	2.22	0.26	0.25
IPI00333140	Delta and Notch-like epidermal growth factor-related receptor precursor	K.IDYCILDPCR.N	2	3.45	0.42	-2.43
IPI00333140	Delta and Notch-like epidermal growth factor-related receptor precursor	K.VTATGFQQCSLIDGR.S	2	4.51	0.44	-3.54
IPI00333140	Delta and Notch-like epidermal growth factor-related receptor precursor	N.PVPAAPLSAPGPCAAQPCR.N	2	3.69	0.49	-3.58
IPI00333140	Delta and Notch-like epidermal growth factor-related receptor precursor	R.DLVNGYECVCLAEYK.G	2	5.19	0.57	-4.27
IPI00333140	Delta and Notch-like epidermal growth factor-related receptor precursor	R.SQATVTLPTWQPK.T	2	3.59	0.28	-2.57
IPI00333140	Delta and Notch-like epidermal growth factor-related receptor precursor	R.SVGTSYK.C	1	1.36	0.14	-2.80
IPI00333197	Isoform 2 of GRIP and coiled-coil domain-containing protein 2	K.KENIKMKQEVEDSVTKM*GDAHK.E	3	2.55	0.06	-8.00
IPI00333410	Isoform 1 of Ubiquitin-conjugating enzyme E2 Q1	K.ELRDIYRSQSFK.G	2	1.92	0.18	
IPI00333776	Isoform 1 of Neuronal cell adhesion molecule precursor	E.PFSHYTLNVR.V	2	3.34	0.37	-2.69
IPI00333776	Isoform 1 of Neuronal cell adhesion molecule precursor	H.HQTEVSGTQTTAQLK.L	2	4.78	0.55	-3.18
IPI00333776	Isoform 1 of Neuronal cell adhesion molecule precursor	K.AAPYWITAPQNLVLSPGEDGTLICR.A	2	5.33	0.54	-5.00

IPI00333776	Isoform 1 of Neuronal cell adhesion molecule precursor	K.AAPYWITAPQNLVLSPGEDGTLICR.A	3	6.05	0.45	-4.91
IPI00333776	Isoform 1 of Neuronal cell adhesion molecule precursor	K.AAPYWITAPQNLVLSPGEDGTLICR.A	4	4.08	0.32	-4.52
IPI00333776	Isoform 1 of Neuronal cell adhesion molecule precursor	K.AETYEGVYQCTAR.N	2	4.85	0.53	-3.95
IPI00333776	Isoform 1 of Neuronal cell adhesion molecule precursor	K.ASEPDKNPTAVEGLGSEPDNLVITWKPLNGFESNGPGLQYK.V	3	6.19	0.51	-1.26
IPI00333776	Isoform 1 of Neuronal cell adhesion molecule precursor	K.ASEPDKNPTAVEGLGSEPDNLVITWKPLNGFESNGPGLQYK.V	4	5.14	0.41	-3.83
IPI00333776	Isoform 1 of Neuronal cell adhesion molecule precursor	K.DNRELPSDER.F	2	2.26	0.08	-1.16
IPI00333776	Isoform 1 of Neuronal cell adhesion molecule precursor	K.DSTGTYTCVAR.N	1	2.43	0.41	-2.36
IPI00333776	Isoform 1 of Neuronal cell adhesion molecule precursor	K.DSTGTYTCVAR.N	2	4.21	0.47	-4.76
IPI00333776	Isoform 1 of Neuronal cell adhesion molecule precursor	K.EDGM*LPK.N	1	1.38	0.11	-1.82
IPI00333776	Isoform 1 of Neuronal cell adhesion molecule precursor	K.EELRGNVLSLECIAEGLPTPIIYWAK.E	3	4.42	0.34	-5.09
IPI00333776	Isoform 1 of Neuronal cell adhesion molecule precursor	K.EKLEPITLQSGQSLVLPCRPPIGLPPPIIFWM*DNSFQR.L	3	4.40	0.55	-0.96
IPI00333776	Isoform 1 of Neuronal cell adhesion molecule precursor	K.EKLEPITLQSGQSLVLPCRPPIGLPPPIIFWM*DNSFQR.L	4	6.08	0.49	-4.94
IPI00333776	Isoform 1 of Neuronal cell adhesion molecule precursor	K.EKLEPITLQSGQSLVLPCRPPIGLPPPIIFWM*DNSFQR.L	5	3.82	0.17	-0.45
IPI00333776	Isoform 1 of Neuronal cell adhesion molecule precursor	K.FIIEYEDAM*HKPGLWHHQTEVSGTQTTAQLK.L	3	6.10	0.54	-5.04
IPI00333776	Isoform 1 of Neuronal cell adhesion molecule precursor	K.FIIEYEDAM*HKPGLWHHQTEVSGTQTTAQLK.L	4	5.77	0.46	-4.49
IPI00333776	Isoform 1 of Neuronal cell adhesion molecule precursor	K.FYFYAQTSAGSGSQITEEAVTTVDEAGILPPDVGAGK.V	3	2.99	0.23	-2.15
IPI00333776	Isoform 1 of Neuronal cell adhesion molecule precursor	K.FYFYAQTSAGSGSQITEEAVTTVDEAGILPPDVGAGK.V	4	3.11	0.13	-3.74
IPI00333776	Isoform 1 of Neuronal cell adhesion molecule precursor	K.GEGPASPDRVFNTPEGVPSAPSSLK.I	2	3.70	0.41	-3.01
IPI00333776	Isoform 1 of Neuronal cell adhesion molecule precursor	K.GEGPASPDRVFNTPEGVPSAPSSLK.I	3	4.79	0.43	-2.81
IPI00333776	Isoform 1 of Neuronal cell adhesion molecule precursor	K.IDGDTIIFSNVQER.S	2	5.23	0.46	-3.56
IPI00333776	Isoform 1 of Neuronal cell adhesion molecule precursor	K.IDGDTIIFSNVQER.S	3	5.17	0.30	-0.47

IPI00333776	Isoform 1 of Neuronal cell adhesion molecule precursor	K.ILTFQGSK.T	2	2.22	0.13	-2.25
IPI00333776	Isoform 1 of Neuronal cell adhesion molecule precursor	K.IVNPTLDSLTLEWDPPSHPNGILTEYTLK.Y	2	4.84	0.53	-3.73
IPI00333776	Isoform 1 of Neuronal cell adhesion molecule precursor	K.IVNPTLDSLTLEWDPPSHPNGILTEYTLK.Y	3	5.21	0.51	-4.58
IPI00333776	Isoform 1 of Neuronal cell adhesion molecule precursor	K.IVNPTLDSLTLEWDPPSHPNGILTEYTLK.Y	4	4.06	0.30	-3.90
IPI00333776	Isoform 1 of Neuronal cell adhesion molecule precursor	K.KILTFQGSK.T	1	2.83	0.24	-4.92
IPI00333776	Isoform 1 of Neuronal cell adhesion molecule precursor	K.LLEDLVQPPTITQQSPK.D	2	4.51	0.43	-4.96
IPI00333776	Isoform 1 of Neuronal cell adhesion molecule precursor	K.LLEDLVQPPTITQQSPK.D	3	4.30	0.37	-3.08
IPI00333776	Isoform 1 of Neuronal cell adhesion molecule precursor	K.LLEDLVQPPTITQQSPKDYIIDPR.E	2	2.14	0.21	-3.78
IPI00333776	Isoform 1 of Neuronal cell adhesion molecule precursor	K.LLEDLVQPPTITQQSPKDYIIDPR.E	4	3.68	0.21	-4.15
IPI00333776	Isoform 1 of Neuronal cell adhesion molecule precursor	K.LSPYVNYSFR.V	1	2.03	0.14	-2.76
IPI00333776	Isoform 1 of Neuronal cell adhesion molecule precursor	K.LSPYVNYSFR.V	2	3.02	0.41	-3.03
IPI00333776	Isoform 1 of Neuronal cell adhesion molecule precursor	K.NEVHLEIK.D	1	2.25	0.16	-4.23
IPI00333776	Isoform 1 of Neuronal cell adhesion molecule precursor	K.NEVHLEIK.D	2	3.03	0.17	-1.47
IPI00333776	Isoform 1 of Neuronal cell adhesion molecule precursor	K.NEVHLEIKDPTWIVK.Q	3	3.65	0.30	-3.36
IPI00333776	Isoform 1 of Neuronal cell adhesion molecule precursor	K.NLNFSTR.Y	2	2.17	0.14	-2.56
IPI00333776	Isoform 1 of Neuronal cell adhesion molecule precursor	K.PLNGFESNGPGLQYK.V	3	3.53	0.23	-2.06
IPI00333776	Isoform 1 of Neuronal cell adhesion molecule precursor	K.QPEYAVVQR.G	1	2.25	0.34	-3.31
IPI00333776	Isoform 1 of Neuronal cell adhesion molecule precursor	K.QPEYAVVQR.G	2	1.99	0.24	-2.06
IPI00333776	Isoform 1 of Neuronal cell adhesion molecule precursor	K.SLPSEASEQYLTK.A	1	2.36	0.33	-2.91
IPI00333776	Isoform 1 of Neuronal cell adhesion molecule precursor	K.SLPSEASEQYLTK.A	2	4.46	0.39	-6.04
IPI00333776	Isoform 1 of Neuronal cell adhesion molecule precursor	K.SLPSEASEQYLTK.A	3	3.29	0.34	-2.28

IPI00333776	Isoform 1 of Neuronal cell adhesion molecule precursor	K.SVQLSWTPGDDNNSPITK.F	2	5.19	0.57	-4.24
IPI00333776	Isoform 1 of Neuronal cell adhesion molecule precursor	K.THGM*LPGLEPFSHYTLNVR.V	2	4.97	0.56	-5.68
IPI00333776	Isoform 1 of Neuronal cell adhesion molecule precursor	K.THGM*LPGLEPFSHYTLNVR.V	3	4.27	0.47	-3.70
IPI00333776	Isoform 1 of Neuronal cell adhesion molecule precursor	K.TLQIIHVSEADSGNYQCIAK.N	2	5.96	0.62	-3.27
IPI00333776	Isoform 1 of Neuronal cell adhesion molecule precursor	K.TLQIIHVSEADSGNYQCIAK.N	3	5.97	0.45	-4.53
IPI00333776	Isoform 1 of Neuronal cell adhesion molecule precursor	K.VQALNDM*GFAPEPAVVM*GHSGEDLPM*VAPGNVR.V	3	5.08	0.55	-4.04
IPI00333776	Isoform 1 of Neuronal cell adhesion molecule precursor	K.VQALNDM*GFAPEPAVVM*GHSGEDLPM*VAPGNVR.V	4	3.53	0.27	-4.19
IPI00333776	Isoform 1 of Neuronal cell adhesion molecule precursor	K.YIVSGTPTFVPYLIK.V	1	3.11	0.41	-1.34
IPI00333776	Isoform 1 of Neuronal cell adhesion molecule precursor	K.YIVSGTPTFVPYLIK.V	2	5.14	0.51	-5.26
IPI00333776	Isoform 1 of Neuronal cell adhesion molecule precursor	K.YIVSGTPTFVPYLIK.V	3	3.67	0.26	-3.52
IPI00333776	Isoform 1 of Neuronal cell adhesion molecule precursor	L.PSEASEQYLTK.A	2	3.75	0.44	-2.53
IPI00333776	Isoform 1 of Neuronal cell adhesion molecule precursor	L.TNGVPIEIAPDDPSR.K	2	4.14	0.45	-4.34
IPI00333776	Isoform 1 of Neuronal cell adhesion molecule precursor	P.GLEPFSHYTLNVR.V	2	3.39	0.30	-2.09
IPI00333776	Isoform 1 of Neuronal cell adhesion molecule precursor	Q.PPTITQQSPKDYIIDPR.E	2	3.53	0.47	-4.33
IPI00333776	Isoform 1 of Neuronal cell adhesion molecule precursor	R.EDYICYAR.F	2	1.83	0.08	-2.72
IPI00333776	Isoform 1 of Neuronal cell adhesion molecule precursor	R.ENIVIQCEAK.G	1	2.94	0.17	-3.05
IPI00333776	Isoform 1 of Neuronal cell adhesion molecule precursor	R.ENIVIQCEAK.G	2	3.30	0.22	-2.91
IPI00333776	Isoform 1 of Neuronal cell adhesion molecule precursor	R.ERPPTFLTPEGNASNK.E	2	3.21	0.21	-3.57
IPI00333776	Isoform 1 of Neuronal cell adhesion molecule precursor	R.ERPPTFLTPEGNASNKEELR.G	2	4.57	0.36	-5.17
IPI00333776	Isoform 1 of Neuronal cell adhesion molecule precursor	R.ERPPTFLTPEGNASNKEELR.G	3	5.10	0.47	-3.44
IPI00333776	Isoform 1 of Neuronal cell adhesion molecule precursor	R.ERPPTFLTPEGNASNKEELRGNVLSLECIAEGLPTPIIYWAK.E	4	4.38	0.32	-4.01

IPI00333776	Isoform 1 of Neuronal cell adhesion molecule precursor	R.GAAVSNNIVVRPSR.S	2	2.63	0.14	-4.64
IPI00333776	Isoform 1 of Neuronal cell adhesion molecule precursor	R.GAAVSNNIVVRPSR.S	3	3.00	0.35	-3.92
IPI00333776	Isoform 1 of Neuronal cell adhesion molecule precursor	R.GHLQGYR.I	1	1.87	0.08	-5.06
IPI00333776	Isoform 1 of Neuronal cell adhesion molecule precursor	R.GNVLSLECIAEGLPTPIIYWAK.E	2	5.21	0.54	-5.18
IPI00333776	Isoform 1 of Neuronal cell adhesion molecule precursor	R.GNVLSLECIAEGLPTPIIYWAK.E	3	5.02	0.39	-5.31
IPI00333776	Isoform 1 of Neuronal cell adhesion molecule precursor	R.GNVLSLECIAEGLPTPIIYWAKEDGM*LPK.N	3	3.52	0.34	-4.35
IPI00333776	Isoform 1 of Neuronal cell adhesion molecule precursor	R.GSM*VSFECK.V	1	2.54	0.21	-3.09
IPI00333776	Isoform 1 of Neuronal cell adhesion molecule precursor	R.GSM*VSFECK.V	2	2.97	0.26	-1.60
IPI00333776	Isoform 1 of Neuronal cell adhesion molecule precursor	R.ILTPANTLYQVIANR.P	2	4.08	0.48	-3.49
IPI00333776	Isoform 1 of Neuronal cell adhesion molecule precursor	R.ISWLTNGVPIEIAPDDPSR.K	2	4.97	0.37	-3.38
IPI00333776	Isoform 1 of Neuronal cell adhesion molecule precursor	R.ISWLTNGVPIEIAPDDPSR.K	3	3.57	0.06	-3.74
IPI00333776	Isoform 1 of Neuronal cell adhesion molecule precursor	R.ISWLTNGVPIEIAPDDPSRK.I	2	3.68	0.36	-1.53
IPI00333776	Isoform 1 of Neuronal cell adhesion molecule precursor	R.ISWLTNGVPIEIAPDDPSRK.I	3	5.40	0.40	-2.54
IPI00333776	Isoform 1 of Neuronal cell adhesion molecule precursor	R.KIDGDTIIFSNVQER.S	2	5.34	0.51	-5.43
IPI00333776	Isoform 1 of Neuronal cell adhesion molecule precursor	R.KIDGDTIIFSNVQER.S	3	6.06	0.36	-4.35
IPI00333776	Isoform 1 of Neuronal cell adhesion molecule precursor	R.TVYKNFEK.T	1	1.90	0.08	-3.71
IPI00333776	Isoform 1 of Neuronal cell adhesion molecule precursor	R.TVYKNFEK.T	2	2.31	0.15	-1.93
IPI00333776	Isoform 1 of Neuronal cell adhesion molecule precursor	R.VFNTPEGVPSAPSSLK.I	2	4.62	0.44	-5.50
IPI00333776	Isoform 1 of Neuronal cell adhesion molecule precursor	R.VFNTPEGVPSAPSSLK.I	3	2.99	0.11	-2.34
IPI00333776	Isoform 1 of Neuronal cell adhesion molecule precursor	R.VKAAPYWITAPQNLVLSPGEDGTLICR.A	3	5.92	0.48	-2.80
IPI00333776	Isoform 1 of Neuronal cell adhesion molecule precursor	R.VKAAPYWITAPQNLVLSPGEDGTLICR.A	4	2.50	0.11	-3.10

IPI00333776	Isoform 1 of Neuronal cell adhesion molecule precursor	R.VM*AVNSIGK.S	1	2.43	0.18	-3.09
IPI00333776	Isoform 1 of Neuronal cell adhesion molecule precursor	R.VM*AVNSIGK.S	2	2.77	0.20	-2.73
IPI00333776	Isoform 1 of Neuronal cell adhesion molecule precursor	R.VSQGLNGDLYFSNVLPEDTR.E	2	5.50	0.51	-3.83
IPI00333776	Isoform 1 of Neuronal cell adhesion molecule precursor	R.VSQGLNGDLYFSNVLPEDTR.E	3	4.38	0.24	-2.49
IPI00333776	Isoform 1 of Neuronal cell adhesion molecule precursor	R.VSQGLNGDLYFSNVLPEDTREDYICYAR.F	2	1.61	0.09	-3.71
IPI00333776	Isoform 1 of Neuronal cell adhesion molecule precursor	R.VSQGLNGDLYFSNVLPEDTREDYICYAR.F	3	4.89	0.46	-6.46
IPI00333776	Isoform 1 of Neuronal cell adhesion molecule precursor	R.VSQGLNGDLYFSNVLPEDTREDYICYAR.F	4	3.87	0.29	-5.93
IPI00333776	Isoform 1 of Neuronal cell adhesion molecule precursor	R.VVNGKGEGPASPDR.V	2	3.98	0.47	-4.17
IPI00333776	Isoform 1 of Neuronal cell adhesion molecule precursor	R.VVNGKGEGPASPDR.V	3	3.11	0.41	-3.22
IPI00333776	Isoform 1 of Neuronal cell adhesion molecule precursor	R.VVNGKGEGPASPDRVFNTPEGVPSAPSSLK.I	2	3.50	0.52	-3.25
IPI00333776	Isoform 1 of Neuronal cell adhesion molecule precursor	R.VVNGKGEGPASPDRVFNTPEGVPSAPSSLK.I	3	4.69	0.46	-4.11
IPI00333776	Isoform 1 of Neuronal cell adhesion molecule precursor	S.PYVNYSFR.V	2	3.17	0.25	-2.62
IPI00333776	Isoform 1 of Neuronal cell adhesion molecule precursor	T.PGDDNNSPITK.F	2	3.60	0.43	-2.53
IPI00333776	Isoform 1 of Neuronal cell adhesion molecule precursor	W.HHQTEVSGTQTTAQLK.L	2	4.57	0.48	-4.41
IPI00333776	Isoform 1 of Neuronal cell adhesion molecule precursor	W.HHQTEVSGTQTTAQLK.L	3	4.01	0.31	-3.60
IPI00333776	Isoform 1 of Neuronal cell adhesion molecule precursor	W.ITAPQNLVLSPGEDGTLICR.A	2	4.37	0.47	-5.49
IPI00333776	Isoform 1 of Neuronal cell adhesion molecule precursor	W.ITAPQNLVLSPGEDGTLICR.A	3	4.47	0.40	-4.79
IPI00333776	Isoform 1 of Neuronal cell adhesion molecule precursor	W.LTNGVPIEIAPDDPSR.K	2	4.35	0.48	-3.90
IPI00333776	Isoform 1 of Neuronal cell adhesion molecule precursor	W.TPGDDNNSPITK.F	2	3.21	0.43	-0.47
IPI00334238	neuronal pentraxin receptor	A.ELEHGSSAYSPPDAFK.I	2	3.38	0.37	-1.76
IPI00334238	neuronal pentraxin receptor	A.LHGAGGSAGPPALPGAPAASAHPLPPGPLFSR.F	3	6.32	0.57	-2.87
IPI00334238	neuronal pentraxin receptor	A.LPGGADNASVASGAAASPGPQR.S	2	5.27	0.59	-3.33
IPI00334238	neuronal pentraxin receptor	A.LPGGADNASVASGAAASPGPQR.S	3	4.54	0.44	-3.36

IPI00334238	neuronal pentraxin receptor	C.IIASVPLAASPAR.A	1	2.40	0.33	-3.01
IPI00334238	neuronal pentraxin receptor	C.IIASVPLAASPAR.A	2	3.67	0.53	-3.49
IPI00334238	neuronal pentraxin receptor	C.IIASVPLAASPAR.A	3	3.70	0.30	-2.62
IPI00334238	neuronal pentraxin receptor	D.SPALILELEDAVR.A	2	4.78	0.42	-2.30
IPI00334238	neuronal pentraxin receptor	G.DAAGAAPGEREELLLLQSTAEQLR.Q	2	4.21	0.44	-4.35
IPI00334238	neuronal pentraxin receptor	G.DAAGAAPGEREELLLLQSTAEQLR.Q	3	4.65	0.31	-4.69
IPI00334238	neuronal pentraxin receptor	G.RVAELEHGSSAYSPPDAFK.I	3	3.90	0.43	-0.53
IPI00334238	neuronal pentraxin receptor	H.PIKPHGILILGQEQDTLGGR.F	2	4.65	0.45	-2.32
IPI00334238	neuronal pentraxin receptor	H.PIKPHGILILGQEQDTLGGR.F	3	6.40	0.38	-2.49
IPI00334238	neuronal pentraxin receptor	I.IASVPLAASPAR.A	2	3.66	0.46	-2.72
IPI00334238	neuronal pentraxin receptor	K.AAFDVCK.G	1	1.99	0.17	-2.43
IPI00334238	neuronal pentraxin receptor	K.AAFDVCK.G	2	1.88	0.08	-3.05
IPI00334238	neuronal pentraxin receptor	K.AAFDVCKG.R	1	2.14	0.29	-1.78
IPI00334238	neuronal pentraxin receptor	K.AAFDVCKG.R	2	3.06	0.30	-0.67
IPI00334238	neuronal pentraxin receptor	K.ALPELYAFTACM*WLR.S	2	5.20	0.53	-4.48
IPI00334238	neuronal pentraxin receptor	K.ALPELYAFTACM*WLR.S	3	3.23	0.42	-2.83
IPI00334238	neuronal pentraxin receptor	K.ALPELYAFTACMWLR.S	2	4.55	0.49	-5.35
IPI00334238	neuronal pentraxin receptor	K.ELDVLQGR.V	1	1.82	0.08	-2.79
IPI00334238	neuronal pentraxin receptor	K.ELDVLQGR.V	2	2.40	0.10	-2.82
IPI00334238	neuronal pentraxin receptor	K.LVEAFGGATK.A	2	3.56	0.31	-3.08
IPI00334238	neuronal pentraxin receptor	K.M*DQLEGQLLAQVLALEK.E	2	6.32	0.42	-3.33
IPI00334238	neuronal pentraxin receptor	K.M*DQLEGQLLAQVLALEK.E	3	5.12	0.29	-3.19
IPI00334238	neuronal pentraxin receptor	K.M*DQLEGQLLAQVLALEKER.V	2	3.79	0.45	-1.88
IPI00334238	neuronal pentraxin receptor	K.M*DQLEGQLLAQVLALEKER.V	3	4.38	0.41	-3.37
IPI00334238	neuronal pentraxin receptor	K.MDQLEGQLLAQVLALEK.E	2	5.38	0.24	
IPI00334238	neuronal pentraxin receptor	K.MDQLEGQLLAQVLALEK.E	3	4.32	0.33	
IPI00334238	neuronal pentraxin receptor	K.MDQLEGQLLAQVLALEKER.V	3	4.24	0.21	
IPI00334238	neuronal pentraxin receptor	K.VAQLPLSLK.D	1	2.50	0.19	-3.33
IPI00334238	neuronal pentraxin receptor	K.VAQLPLSLK.D	2	2.95	0.23	-1.69
IPI00334238	neuronal pentraxin receptor	P.GGADNASVASGAAASPGPQR.S	2	5.42	0.54	-5.13
IPI00334238	neuronal pentraxin receptor	Q.EVEKELDVLQGR.V	2	3.47	0.31	-2.89
IPI00334238	neuronal pentraxin receptor	R.ADQDTIRELTGK.L	2	2.86	0.15	-1.48
IPI00334238	neuronal pentraxin receptor	R.ALPGGADNASVASGAAASPGPQR.S	2	4.99	0.51	-3.37
IPI00334238	neuronal pentraxin receptor	R.ALPGGADNASVASGAAASPGPQR.S	3	2.30	0.16	-3.34
IPI00334238	neuronal pentraxin receptor	R.DGLWSAYQDGELQGSGENLAAWHPIKPHGILILGQEQDTLGGR.F	3	4.48	0.34	
IPI00334238	neuronal pentraxin receptor	R.DGLWSAYQDGELQGSGENLAAWHPIKPHGILILGQEQDTLGGR.F	5	2.94	0.10	-4.42
IPI00334238	neuronal pentraxin receptor	R.FLCTPLAAACPSGAQQGDAAGAAPGEREELLLLQSTAEQLR.Q	3	5.92	0.53	-4.02
IPI00334238	neuronal pentraxin receptor	R.FLCTPLAAACPSGAQQGDAAGAAPGEREELLLLQSTAEQLR.Q	4	4.98	0.42	-3.50
IPI00334238	neuronal pentraxin receptor	R.IDRLEQELPAR.V	2	2.56	0.21	-2.29
IPI00334238	neuronal pentraxin receptor	R.IRADQDTIRELTGK.L	2	3.19	0.32	-1.52
IPI00334238	neuronal pentraxin receptor	R.IRADQDTIRELTGK.L	3	3.86	0.36	-2.19
IPI00334238	neuronal pentraxin receptor	R.KALPELYAFTACM*WLR.S	2	4.50	0.46	-3.92

IPI00334238	neuronal pentraxin receptor	R.KALPELYAFTACM*WLR.S	3	3.99	0.38	-2.76
IPI00334238	neuronal pentraxin receptor	R.QEVEKELDVLQGR.V	2	3.61	0.30	0.29
IPI00334238	neuronal pentraxin receptor	R.QRQEVEKELDVLQGR.V	2	3.31	0.37	-3.69
IPI00334238	neuronal pentraxin receptor	R.QRQEVEKELDVLQGR.V	3	3.51	0.41	-2.81
IPI00334238	neuronal pentraxin receptor	R.QRQEVEKELDVLQGR.V	4	3.10	0.11	-2.73
IPI00334238	neuronal pentraxin receptor	R.QTALQQEAR.I	1	1.84	0.11	-1.85
IPI00334238	neuronal pentraxin receptor	R.QTALQQEAR.I	2	2.60	0.16	-1.41
IPI00334238	neuronal pentraxin receptor	R.RDTM*ADGPWDSPALILELEDAVR.A	3	3.24	0.11	-2.64
IPI00334238	neuronal pentraxin receptor	R.SLSALHGAGGSAGPPALPGAPAA.S	2	4.35	0.54	-3.03
IPI00334238	neuronal pentraxin receptor	R.SLSALHGAGGSAGPPALPGAPAASAHPLPPGPLFSR.F	3	6.90	0.63	-4.39
IPI00334238	neuronal pentraxin receptor	R.SLSALHGAGGSAGPPALPGAPAASAHPLPPGPLFSR.F	4	3.83	0.31	-3.52
IPI00334238	neuronal pentraxin receptor	R.SSGTGQGTPFSYSVPGQANEIVLLEAGHEPM*ELLINDK.V	3	5.02	0.50	-4.89
IPI00334238	neuronal pentraxin receptor	R.SSGTGQGTPFSYSVPGQANEIVLLEAGHEPM*ELLINDK.V	4	2.66	0.18	-3.97
IPI00334238	neuronal pentraxin receptor	R.SSGTGQGTPFSYSVPGQANEIVLLEAGHEPMELLINDK.V	3	4.67	0.31	
IPI00334238	neuronal pentraxin receptor	R.VAELEHGSSAYSPPDAFK.I	2	5.38	0.49	-3.77
IPI00334238	neuronal pentraxin receptor	R.VAELEHGSSAYSPPDAFK.I	3	4.57	0.34	-3.40
IPI00334238	neuronal pentraxin receptor	R.VAELEHGSSAYSPPDAFKISIPIR.N	3	6.67	0.50	-3.26
IPI00334238	neuronal pentraxin receptor	R.VAELEHGSSAYSPPDAFKISIPIR.N	4	5.05	0.41	-2.62
IPI00334238	neuronal pentraxin receptor	V.AELEHGSSAYSPPDAFK.I	2	4.78	0.53	-2.72
IPI00334238	neuronal pentraxin receptor	W.DSPALILELEDAVR.A	2	4.83	0.34	-3.40
IPI00334282	Protein FAM3C precursor	A.SGAANVVGPK.I	1	2.19	0.32	-2.27
IPI00334282	Protein FAM3C precursor	D.ASLGNLFAR.S	2	2.97	0.26	-0.22
IPI00334282	Protein FAM3C precursor	E.IKM*DASLGNLFAR.S	3	3.98	0.21	-1.32
IPI00334282	Protein FAM3C precursor	I.KM*DASLGNLFAR.S	2	3.14	0.35	-4.99
IPI00334282	Protein FAM3C precursor	I.KM*DASLGNLFAR.S	3	3.66	0.18	-3.36
IPI00334282	Protein FAM3C precursor	K.AIQDGTIVLM*GTYDDGATK.L	2	6.86	0.56	-5.01
IPI00334282	Protein FAM3C precursor	K.AIQDGTIVLM*GTYDDGATK.L	3	5.92	0.48	-4.19
IPI00334282	Protein FAM3C precursor	K.AIQDGTIVLM*GTYDDGATKLNDEAR.R	2	5.56	0.54	-8.24
IPI00334282	Protein FAM3C precursor	K.AIQDGTIVLM*GTYDDGATKLNDEAR.R	3	5.78	0.53	-4.68
IPI00334282	Protein FAM3C precursor	K.AIQDGTIVLM*GTYDDGATKLNDEAR.R	4	4.03	0.46	-3.25
IPI00334282	Protein FAM3C precursor	K.AIQDGTIVLM*GTYDDGATKLNDEARR.L	3	4.90	0.41	-6.12
IPI00334282	Protein FAM3C precursor	K.AIQDGTIVLM*GTYDDGATKLNDEARR.L	4	3.38	0.38	-3.38
IPI00334282	Protein FAM3C precursor	K.AIQDGTIVLMGTYDDGATKLNDEAR.R	3	4.43	0.47	-2.17
IPI00334282	Protein FAM3C precursor	K.DTNKYEGWPEVVEM*EGCIPQKQD.-	3	3.08	0.37	-2.98
IPI00334282	Protein FAM3C precursor	K.ICLEDNVLM*SGVK.N	1	1.61	0.16	-3.95
IPI00334282	Protein FAM3C precursor	K.ICLEDNVLM*SGVK.N	2	3.72	0.36	-4.27
IPI00334282	Protein FAM3C precursor	K.M*ASGAANVVGPK.I	1	2.90	0.45	-4.92
IPI00334282	Protein FAM3C precursor	K.M*ASGAANVVGPK.I	2	4.24	0.42	-3.44
IPI00334282	Protein FAM3C precursor	K.M*DASLGNLFAR.S	2	4.15	0.47	-2.74
IPI00334282	Protein FAM3C precursor	K.SPFEQHIK.N	1	2.16	0.09	-5.05
IPI00334282	Protein FAM3C precursor	K.SPFEQHIK.N	2	3.12	0.18	-2.06
IPI00334282	Protein FAM3C precursor	K.TGEVLDTK.Y	1	2.54	0.19	-4.32

IPI00334282	Protein FAM3C precursor	K.TGEVLDTK.Y	2	3.04	0.30	-3.83
IPI00334282	Protein FAM3C precursor	K.TGEVLDTKYFDM*WGGDVAPFIEFLK.A	3	4.80	0.41	-4.60
IPI00334282	Protein FAM3C precursor	K.TKSPFEQHIK.N	3	2.99	0.16	-5.13
IPI00334282	Protein FAM3C precursor	K.YFDM*WGGDVAPFIEFLK.A	2	5.63	0.53	-5.55
IPI00334282	Protein FAM3C precursor	K.YFDM*WGGDVAPFIEFLK.A	3	3.12	0.18	-4.72
IPI00334282	Protein FAM3C precursor	L.IADLGSTSITNLGFR.D	2	4.19	0.51	-3.25
IPI00334282	Protein FAM3C precursor	M.ASGAANVVGPK.I	1	2.04	0.23	-2.61
IPI00334282	Protein FAM3C precursor	M.ASGAANVVGPK.I	2	2.92	0.30	0.07
IPI00334282	Protein FAM3C precursor	M.DASLGNLFAR.S	2	3.65	0.22	-2.95
IPI00334282	Protein FAM3C precursor	R.DNWVFCGGK.G	1	2.62	0.35	-5.17
IPI00334282	Protein FAM3C precursor	R.DNWVFCGGK.G	2	3.06	0.40	-3.46
IPI00334282	Protein FAM3C precursor	R.GINVALANGK.T	1	2.90	0.30	-1.79
IPI00334282	Protein FAM3C precursor	R.GINVALANGK.T	2	3.81	0.43	-2.85
IPI00334282	Protein FAM3C precursor	R.LIADLGSTSITNLGFR.D	2	5.97	0.56	-6.52
IPI00334282	Protein FAM3C precursor	R.LIADLGSTSITNLGFR.D	3	5.52	0.47	-3.84
IPI00334282	Protein FAM3C precursor	R.RLIADLGSTSITNLGFR.D	2	5.39	0.51	-7.05
IPI00334282	Protein FAM3C precursor	R.RLIADLGSTSITNLGFR.D	3	4.20	0.43	-6.92
IPI00334282	Protein FAM3C precursor	R.SALDTAAR.S	2	2.54	0.19	-3.36
IPI00334282	Protein FAM3C precursor	S.GAANVVGPK.I	1	1.96	0.23	-1.93
IPI00334282	Protein FAM3C precursor	W.GGDVAPFIEFLK.A	1	2.49	0.29	-1.46
IPI00334282	Protein FAM3C precursor	W.GGDVAPFIEFLK.A	2	3.07	0.33	-4.01
IPI00334667	Isoform 2 of Receptor-type tyrosine-protein phosphatase N2 precursor	A.LGESGEQADGPK.A	2	2.93	0.30	-2.44
IPI00334667	Isoform 2 of Receptor-type tyrosine-protein phosphatase N2 precursor	K.ATLRGDSFPDDGVQDDDDRLYQEVHR.L	4	3.06	0.30	-3.35
IPI00334667	Isoform 2 of Receptor-type tyrosine-protein phosphatase N2 precursor	K.DLLGQQPHSEPGA.A	2	2.97	0.27	-2.97
IPI00334667	Isoform 2 of Receptor-type tyrosine-protein phosphatase N2 precursor	K.DLLGQQPHSEPGAAAFGE.L	2	3.89	0.49	-3.49
IPI00334667	Isoform 2 of Receptor-type tyrosine-protein phosphatase N2 precursor	K.DLLGQQPHSEPGAAAFGELQNQM*PGPSK.E	3	4.23	0.43	-2.51
IPI00334667	Isoform 2 of Receptor-type tyrosine-protein phosphatase N2 precursor	K.KSEHPESSLSSEEETAGVENVK.S	2	6.69	0.68	-3.46
IPI00334667	Isoform 2 of Receptor-type tyrosine-protein phosphatase N2 precursor	K.KSEHPESSLSSEEETAGVENVK.S	3	4.15	0.35	-4.02
IPI00334667	Isoform 2 of Receptor-type tyrosine-protein phosphatase N2 precursor	K.LSGTGFTWQDDYTQYVM*DQELADLPK.T	3	3.24	0.12	-4.84
IPI00334667	Isoform 2 of Receptor-type tyrosine-protein phosphatase N2 precursor	K.SEHPESSLSSEEETAGVENVK.S	2	6.53	0.66	-3.40
IPI00334667	Isoform 2 of Receptor-type tyrosine-protein phosphatase N2 precursor	K.SEHPESSLSSEEETAGVENVK.S	3	2.98	0.22	-1.58

IPI00334667	Isoform 2 of Receptor-type tyrosine-protein phosphatase N2 precursor	R.AALGESGEQADGPK.A	2	4.40	0.56	-2.34
IPI00334667	Isoform 2 of Receptor-type tyrosine-protein phosphatase N2 precursor	R.GDSFPDDGVQDDDDRLYQEVHR.L	3	2.02	0.17	-1.55
IPI00334667	Isoform 2 of Receptor-type tyrosine-protein phosphatase N2 precursor	R.GYIVTDREVLGPAVTFK.V	3	3.89	0.17	-2.32
IPI00334667	Isoform 2 of Receptor-type tyrosine-protein phosphatase N2 precursor	R.HLPFLEALSQAPASDVLAR.T	3	3.27	0.29	-2.91
IPI00334667	Isoform 2 of Receptor-type tyrosine-protein phosphatase N2 precursor	R.LLPGALPFARPLDM*ER.K	2	3.16	0.07	
IPI00334667	Isoform 2 of Receptor-type tyrosine-protein phosphatase N2 precursor	R.LSATLGGLLQDHGS.R	2	3.06	0.34	-4.45
IPI00334667	Isoform 2 of Receptor-type tyrosine-protein phosphatase N2 precursor	R.LSATLGGLLQDHGSR.L	2	3.53	0.39	0.62
IPI00334667	Isoform 2 of Receptor-type tyrosine-protein phosphatase N2 precursor	R.RPEASSPARPSK.H	2	1.92	0.08	-3.50
IPI00334667	Isoform 2 of Receptor-type tyrosine-protein phosphatase N2 precursor	R.RPEASSPARPSK.H	3	2.62	0.23	-3.02
IPI00334667	Isoform 2 of Receptor-type tyrosine-protein phosphatase N2 precursor	R.TLGQLQPDELSPK.V	2	3.88	0.45	-5.95
IPI00334667	Isoform 2 of Receptor-type tyrosine-protein phosphatase N2 precursor	R.YEVSPVALQR.L	1	2.04	0.32	-3.89
IPI00334667	Isoform 2 of Receptor-type tyrosine-protein phosphatase N2 precursor	R.YEVSPVALQR.L	2	2.53	0.31	-1.88
IPI00334667	Isoform 2 of Receptor-type tyrosine-protein phosphatase N2 precursor	S.EHPESSLSSEEETAGVENVK.S	2	4.76	0.57	-3.43
IPI00335168	Isoform Non-muscle of Myosin light polypeptide 6	R.ALGQNPTNAEVLK.V	2	3.27	0.28	-3.09
IPI00335437	Ankyrin repeat and zinc finger domain-containing protein 1	R.DLLAGPSWAKALEEAGTILLRAPRSGR.S	3	2.97	0.10	
IPI00335541	Isoform 1 of Protein timeless homolog	R.GNRHSRFGGSYIVQGLK.S	3	2.88	0.16	
IPI00335589	RNA methyltransferase-like protein 1	K.ELPVDKLKGVSLIKVK.F	2	2.41	0.15	
IPI00335946	Family with sequence similarity 120B	R.VKENFDK.A	1	2.41	0.08	-2.55
IPI00337351	MAM domain-containing glycosylphosphatidylinositol anchor protein 2 precursor	K.AENGLGSPAIK.S	2	2.22	0.12	-1.30
IPI00337351	MAM domain-containing glycosylphosphatidylinositol anchor protein 2 precursor	R.ADKEVAM*PDGSM*QM*ESYDGTLR.I	3	4.77	0.46	-7.56
IPI00337351	MAM domain-containing glycosylphosphatidylinositol anchor protein 2 precursor	R.CSFLVTGK.A	2	2.27	0.24	-1.67

IPI00337351	MAM domain-containing glycosylphosphatidylinositol anchor protein 2 precursor	R.GQEVLLQGSDKGVEIYEPFFTQGETK.I	3	4.74	0.28	-4.53
IPI00337351	MAM domain-containing glycosylphosphatidylinositol anchor protein 2 precursor	R.TGQFDSQEYTEYAVK.S	2	4.27	0.52	-2.60
IPI00337351	MAM domain-containing glycosylphosphatidylinositol anchor protein 2 precursor	R.VLTYEWR.L	2	2.13	0.12	-2.07
IPI00337385	Isoform 1 of Pre-mRNA-processing factor 40 homolog A	K.TGKDSGNWDTSGSELSEGELEKRRR.T	3	2.33	0.07	-2.52
IPI00337548	Cell growth regulator with EF hand domain protein 1	K.DGVTRPDSEVQHQLLPNPFQPGQEQLGLLQSYLK.G	3	6.32	0.45	
IPI00337548	Cell growth regulator with EF hand domain protein 1	K.DGVTRPDSEVQHQLLPNPFQPGQEQLGLLQSYLK.G	4	3.01	0.14	-2.18
IPI00337548	Cell growth regulator with EF hand domain protein 1	K.NTQNDFEVHIVQVENDEI.-	2	4.66	0.30	
IPI00337548	Cell growth regulator with EF hand domain protein 1	K.SPLRQETQEAPGPR.E	2	3.49	0.27	-4.08
IPI00337548	Cell growth regulator with EF hand domain protein 1	K.SPLRQETQEAPGPR.E	3	3.81	0.35	-2.33
IPI00337548	Cell growth regulator with EF hand domain protein 1	K.SPLRQETQEAPGPREEAK.G	2	2.81	0.25	-1.59
IPI00337548	Cell growth regulator with EF hand domain protein 1	K.SPLRQETQEAPGPREEAK.G	3	2.93	0.08	-0.45
IPI00337548	Cell growth regulator with EF hand domain protein 1	K.SPLRQETQEAPGPREEAK.G	4	3.63	0.20	-0.76
IPI00337548	Cell growth regulator with EF hand domain protein 1	K.VLETQDLNGDGLM*TPAELINFPGVALR.H	3	5.66	0.49	
IPI00337548	Cell growth regulator with EF hand domain protein 1	L.RHVEPGEPLAPSPQEPQAVGR.Q	3	4.21	0.37	-5.45
IPI00337548	Cell growth regulator with EF hand domain protein 1	R.ENGEEAKELPGETLESK.N	2	4.37	0.48	-2.49
IPI00337548	Cell growth regulator with EF hand domain protein 1	R.ENGEEAKELPGETLESK.N	3	3.70	0.33	-1.07
IPI00337548	Cell growth regulator with EF hand domain protein 1	R.ESLDPVQEPGGQAEADGDVPGPR.G	2	5.68	0.52	-3.63
IPI00337548	Cell growth regulator with EF hand domain protein 1	R.ESLDPVQEPGGQAEADGDVPGPR.G	3	2.97	0.40	-2.85
IPI00337548	Cell growth regulator with EF hand domain protein 1	R.GEAEGQAEAKGDAPGPR.G	3	3.73	0.39	-1.77

IPI00337548	Cell growth regulator with EF hand domain protein 1	R.HVEPGEPLAPSPQEPQAVGR.Q	2	4.97	0.58	-2.15
IPI00337548	Cell growth regulator with EF hand domain protein 1	R.HVEPGEPLAPSPQEPQAVGR.Q	3	2.86	0.16	-2.77
IPI00337548	Cell growth regulator with EF hand domain protein 1	R.RESLDPVQEPGGQAEADGDVPGPR.G	3	5.29	0.49	-3.64
IPI00337612	Discoidin, CUB and LCCL domain-containing protein 1 precursor	R.GFLLTYASSDHPDLITCLER.A	3	3.82	0.38	-1.21
IPI00337612	Discoidin, CUB and LCCL domain-containing protein 1 precursor	R.TTGSTQSNFNFYVK.S	2	3.89	0.46	-2.90
IPI00373823	Cytochrome P450 26C1	K.GSMGWPFFGETLHWLVQGSR.F	2	2.43	0.07	-3.24
IPI00373872	polycystin 1-like 2 isoform a	R.EALQLMVSSESFIFDNVTISLTWLLSPYIGNLS.C	3	3.82	0.19	2.99
IPI00374039	Conserved hypothetical protein	R.MQDTM*VQELALAKK.Q	2	2.77	0.07	
IPI00374065	similar to melanoma inhibitory activity 3 isoform 1	K.FGSTADALVSDDETTR.L	2	4.03	0.48	-3.28
IPI00374065	similar to melanoma inhibitory activity 3 isoform 1	K.SAYDDTENDLKGAAIHISK.G	3	2.53	0.24	-2.94
IPI00374065	similar to melanoma inhibitory activity 3 isoform 1	K.TLQDM*EKNPELSK.E	2	2.97	0.26	0.36
IPI00374065	similar to melanoma inhibitory activity 3 isoform 1	R.FSSPDEIDLPRELEDEVPILGR.N	3	3.69	0.40	-3.71
IPI00374065	similar to melanoma inhibitory activity 3 isoform 1	R.QFDVNLQVPDR.A	2	2.04	0.17	-2.95
IPI00374065	similar to melanoma inhibitory activity 3 isoform 1	R.SDFSDSIK.I	2	2.38	0.11	-2.95
IPI00374129	NLR family, pyrin domain containing 3 isoform b	K.YVRSRFQCIEDRNAR.L	2	1.37	0.07	-4.06
IPI00374301	hypothetical protein	R.SLSSPNCFGPQPGGPEMR.R	2	2.00	0.06	-5.34
IPI00374563	Agrin precursor	K.ALQSNHFELSLR.T	2	3.47	0.39	-3.50
IPI00374563	Agrin precursor	K.ALQSNHFELSLR.T	3	2.61	0.23	-3.73
IPI00374563	Agrin precursor	K.AYGTGFVGCLR.D	1	1.69	0.30	-2.77
IPI00374563	Agrin precursor	K.AYGTGFVGCLR.D	2	3.27	0.31	-2.86
IPI00374563	Agrin precursor	K.SAGDVDTLAFDGR.T	1	2.66	0.44	-1.72
IPI00374563	Agrin precursor	K.SAGDVDTLAFDGR.T	2	4.36	0.55	-3.87
IPI00374563	Agrin precursor	K.SELFGETAR.S	1	1.79	0.11	-3.48
IPI00374563	Agrin precursor	K.SELFGETAR.S	2	2.70	0.20	-2.18
IPI00374563	Agrin precursor	K.VLGAPVPAFEGR.S	2	2.57	0.23	-2.30
IPI00374563	Agrin precursor	R.AIVDVHFDPTTAFR.A	2	3.46	0.40	-3.17
IPI00374563	Agrin precursor	R.ALEPQGLLLYNGNAR.G	2	2.48	0.38	-3.91
IPI00374563	Agrin precursor	R.DVVVGR.H	1	2.12	0.11	-3.17
IPI00374563	Agrin precursor	R.EAACLQQTQIEEAR.A	2	3.86	0.31	-2.83
IPI00374563	Agrin precursor	R.FGALCEAETGR.C	2	2.75	0.29	-2.54
IPI00374563	Agrin precursor	R.GKDFLALALLDGR.V	3	3.17	0.28	-2.11
IPI00374563	Agrin precursor	R.GM*LCGFGAVCEPNAEGPGR.A	2	5.62	0.57	-2.78
IPI00374563	Agrin precursor	R.GPSGLLLYNGQK.T	2	3.78	0.41	-1.35
IPI00374563	Agrin precursor	R.LELGIGPGAATR.G	2	1.79	0.08	-2.51
IPI00374563	Agrin precursor	R.LLDVNNQR.L	2	2.42	0.11	-2.07
IPI00374563	Agrin precursor	R.RPLQEHVR.F	2	2.88	0.18	-3.10
IPI00374563	Agrin precursor	R.SFLAFPTLR.A	2	2.76	0.28	-0.74

IPI00374563	Agrin precursor	R.SIESTLDDLFR.N	2	3.97	0.39	-2.88
IPI00374563	Agrin precursor	R.STVPVNTNR.W	2	2.31	0.19	0.20
IPI00374563	Agrin precursor	R.TFVEYLNAVTESEK.A	2	5.38	0.52	-4.64
IPI00374563	Agrin precursor	R.VLGESPVPHTVLNLKEPLYVGGAPDFSK.L	3	4.83	0.55	-1.91
IPI00374563	Agrin precursor	R.VLGESPVPHTVLNLKEPLYVGGAPDFSK.L	4	3.47	0.30	-2.77
IPI00374563	Agrin precursor	W.LGGLPELPVGPALPK.A	2	2.95	0.25	-1.65
IPI00374590	cancer susceptibility candidate 4 isoform a	K.IQSNDGKELDINNQVVPK.N	3	2.38	0.10	-2.97
IPI00374590	cancer susceptibility candidate 4 isoform a	K.VAENVADKNEEPSSNHIPHGK.E	3	3.95	0.34	-3.40
IPI00374590	cancer susceptibility candidate 4 isoform a	R.FFDENESPVDPQHGSK.L	2	4.86	0.56	-3.13
IPI00374590	cancer susceptibility candidate 4 isoform a	R.FFDENESPVDPQHGSK.L	3	3.12	0.35	-1.97
IPI00374670	hypothetical protein isoform 2	R.DGVSLGAVSSTEEASRCR.R	2	2.12	0.06	-0.37
IPI00374732	similar to peptidylprolyl isomerase A isoform 1	K.GSRFHRIIPGFM*CQGGDFTR.H	2	2.33	0.15	0.45
IPI00374732	similar to peptidylprolyl isomerase A isoform 1	K.HTGPGILSM*ANAGPNTNGSQFFICTAK.S	3	5.75	0.44	-3.52
IPI00374732	similar to peptidylprolyl isomerase A isoform 1	K.KITIADCGQLE.-	2	2.86	0.28	-3.75
IPI00374862	Isoform 1 of Kelch-like protein 5	K.QLHPSNCLGIRSFADAQGCTDLHKVAHNYTMEHFMEVIR.N	3	1.36	0.16	2.58
IPI00374914	hypothetical protein	K.TACSLDAR.E	2	2.65	0.21	-3.13
IPI00374914	hypothetical protein	R.ALCACWPAGH.-	1	1.82	0.30	-2.39
IPI00374914	hypothetical protein	R.ALCACWPAGH.-	2	3.25	0.46	-2.56
IPI00374914	hypothetical protein	R.TETLLLQAER.R	1	2.27	0.22	-3.23
IPI00374914	hypothetical protein	R.TETLLLQAER.R	2	3.13	0.28	-3.77
IPI00375174	Ankyrin repeat and sterile alpha motif domain-containing protein 1B	K.DYSFEDLCHTISDHYLDNLSK.I	2	1.59	0.11	-3.43
IPI00375205	Isoform 1 of Polypeptide N-acetylgalactosaminyltransferase 10	K.IAWDLPK.F	2	2.30	0.10	-2.15
IPI00375205	Isoform 1 of Polypeptide N-acetylgalactosaminyltransferase 10	K.RIPIPPELQK.A	2	2.66	0.18	-2.39
IPI00375205	Isoform 1 of Polypeptide N-acetylgalactosaminyltransferase 10	K.TFFLGDGQK.L	1	2.07	0.21	-3.22
IPI00375205	Isoform 1 of Polypeptide N-acetylgalactosaminyltransferase 10	K.TFFLGDGQK.L	2	2.27	0.09	-2.79
IPI00375205	Isoform 1 of Polypeptide N-acetylgalactosaminyltransferase 10	K.TIVCPM*IDVIDHDDFR.Y	2	3.43	0.43	-3.37
IPI00375205	Isoform 1 of Polypeptide N-acetylgalactosaminyltransferase 10	K.TIVCPM*IDVIDHDDFR.Y	3	1.72	0.11	-2.52
IPI00375205	Isoform 1 of Polypeptide N-acetylgalactosaminyltransferase 10	R.ERQPDGTPGGSGAAVAPAAGQGSHSR.Q	3	4.37	0.30	-5.28
IPI00375205	Isoform 1 of Polypeptide N-acetylgalactosaminyltransferase 10	R.M*LGASVATGDVITFLDSHCEANVNWLPPLLDR.I	3	3.04	0.22	-2.98
IPI00375205	Isoform 1 of Polypeptide N-acetylgalactosaminyltransferase 10	R.VGNGEQGRPYPM*TDAER.V	3	2.90	0.18	-2.88
IPI00375205	Isoform 1 of Polypeptide N-acetylgalactosaminyltransferase 10	R.YETQAGDAM*R.G	2	3.28	0.34	-2.09

IPI00375364	Isoform 3 of Chitotriosidase-1 precursor	K.LILGM*PTYGR.S	2	2.58	0.20	-2.80
IPI00375364	Isoform 3 of Chitotriosidase-1 precursor	K.VTGHNSPLYK.R	2	2.41	0.18	-3.22
IPI00375364	Isoform 3 of Chitotriosidase-1 precursor	R.FTTLVQDLANAFQQEAQTSGK.E	2	6.00	0.52	-2.93
IPI00375364	Isoform 3 of Chitotriosidase-1 precursor	R.FTTLVQDLANAFQQEAQTSGK.E	3	5.13	0.34	-3.81
IPI00375364	Isoform 3 of Chitotriosidase-1 precursor	R.FTTLVQDLANAFQQEAQTSGKER.L	3	3.68	0.38	-3.68
IPI00375364	Isoform 3 of Chitotriosidase-1 precursor	R.VGAPATGSGTPGPFTK.E	2	4.13	0.52	-3.52
IPI00375547	Protein tyrosine phosphatase receptor type D	K.GPGPYSPSVQFR.T	2	3.15	0.32	-1.00
IPI00375547	Protein tyrosine phosphatase receptor type D	K.GYYIIIVPLKK.S	2	3.43	0.43	-4.48
IPI00375547	Protein tyrosine phosphatase receptor type D	K.GYYIIIVPLKK.S	3	2.87	0.30	-3.75
IPI00375547	Protein tyrosine phosphatase receptor type D	K.HNVADSQITTIGNLVPQK.T	2	3.88	0.50	-3.32
IPI00375547	Protein tyrosine phosphatase receptor type D	K.HNVADSQITTIGNLVPQKTYSVK.V	3	6.15	0.49	-1.87
IPI00375547	Protein tyrosine phosphatase receptor type D	K.ILYDDGKM*VEEVDGR.A	3	3.65	0.34	-2.62
IPI00375547	Protein tyrosine phosphatase receptor type D	K.KVSNQRFEVIEFDDGSGSVLR.I	3	4.66	0.47	-1.18
IPI00375547	Protein tyrosine phosphatase receptor type D	K.LIVNLKPEK.S	2	2.45	0.18	-2.57
IPI00375547	Protein tyrosine phosphatase receptor type D	K.LVSTTGAVPGKPR.L	2	3.18	0.41	-2.61
IPI00375547	Protein tyrosine phosphatase receptor type D	K.M*VEEVDGR.A	2	2.33	0.13	-1.84
IPI00375547	Protein tyrosine phosphatase receptor type D	K.NSEELYKEIDGVATTR.Y	3	3.38	0.38	-2.06
IPI00375547	Protein tyrosine phosphatase receptor type D	K.SYSFVLTNR.G	1	1.61	0.12	-2.28
IPI00375547	Protein tyrosine phosphatase receptor type D	K.SYSFVLTNR.G	2	3.47	0.26	-1.82
IPI00375547	Protein tyrosine phosphatase receptor type D	K.TNLDGM*ITVQLPEVPANENIK.G	2	4.72	0.49	-5.07
IPI00375547	Protein tyrosine phosphatase receptor type D	K.VSNQRFEVIEFDDGSGSVLR.I	3	4.46	0.34	-3.71
IPI00375547	Protein tyrosine phosphatase receptor type D	K.WM*LGAEDLTPEDDM*PIGR.N	2	3.53	0.42	-2.68
IPI00375547	Protein tyrosine phosphatase receptor type D	K.YTAVDGEDDKPHEILGIPSDTTK.Y	3	3.65	0.20	-0.96
IPI00375547	Protein tyrosine phosphatase receptor type D	P.VLTQTSEQAPSSAPR.D	2	4.27	0.35	-2.52
IPI00375547	Protein tyrosine phosphatase receptor type D	R.EVELKPYIAAHFDVLPTEFTLGDDKHYGGFTNK.Q	3	4.11	0.32	-5.08
IPI00375547	Protein tyrosine phosphatase receptor type D	R.EVELKPYIAAHFDVLPTEFTLGDDKHYGGFTNK.Q	4	4.71	0.48	-3.02
IPI00375547	Protein tyrosine phosphatase receptor type D	R.EVELKPYIAAHFDVLPTEFTLGDDKHYGGFTNK.Q	5	3.66	0.24	-3.70
IPI00375547	Protein tyrosine phosphatase receptor type D	R.GALQIEQSEESDQGKYECVATNSAGTR.Y	3	6.59	0.59	-2.64
IPI00375547	Protein tyrosine phosphatase receptor type D	R.GFPTIDM*GPQLK.V	2	2.74	0.26	-3.67
IPI00375547	Protein tyrosine phosphatase receptor type D	R.GPPSEPVLTQTSEQAPSSAPR.D	2	4.98	0.60	-2.90
IPI00375547	Protein tyrosine phosphatase receptor type D	R.GYQVHYVR.M	2	2.74	0.30	-3.75
IPI00375547	Protein tyrosine phosphatase receptor type D	R.ITIEPGTSYR.L	2	2.44	0.24	-1.91
IPI00375547	Protein tyrosine phosphatase receptor type D	R.LQGLKPNSLYYFR.L	2	2.32	0.24	-3.75
IPI00375547	Protein tyrosine phosphatase receptor type D	R.LTVLREDQIPR.G	3	2.42	0.26	-3.08
IPI00375547	Protein tyrosine phosphatase receptor type D	R.NVLELNDVR.Q	1	2.93	0.22	-2.75
IPI00375547	Protein tyrosine phosphatase receptor type D	R.NVLELNDVR.Q	2	3.13	0.26	-2.16
IPI00375547	Protein tyrosine phosphatase receptor type D	R.SESIGGTPIR.G	2	2.66	0.15	-1.49
IPI00375547	Protein tyrosine phosphatase receptor type D	R.SPQGLGASTAEISAR.T	2	4.61	0.47	-3.72
IPI00375547	Protein tyrosine phosphatase receptor type D	R.TATM*LCAASGNPDPEITWFKDFLPVDTSNNNGR.I	3	5.81	0.53	-1.15
IPI00375547	Protein tyrosine phosphatase receptor type D	R.TATM*LCAASGNPDPEITWFKDFLPVDTSNNNGR.I	4	3.94	0.38	-2.09
IPI00375547	Protein tyrosine phosphatase receptor type D	R.TNEDVPSGPPR.K	2	3.03	0.37	-2.60
IPI00375547	Protein tyrosine phosphatase receptor type D	R.TPVDQTGVSGGVASFICQATGDPRPK.I	2	3.76	0.54	-1.52

IPI00375547	Protein tyrosine phosphatase receptor type D	R.TPVDQTGVSGGVASFICQATGDPRPK.I	3	3.93	0.48	-2.52
IPI00375547	Protein tyrosine phosphatase receptor type D	R.TPVDQTGVSGGVASFICQATGDPRPK.I	4	2.50	0.15	-1.89
IPI00375547	Protein tyrosine phosphatase receptor type D	R.VVAVNNIGR.G	2	3.13	0.25	-2.06
IPI00375547	Protein tyrosine phosphatase receptor type D	R.VVAVNNIGRGPPSEPVLTQTSEQAPSSAPR.D	3	5.98	0.60	-2.36
IPI00375547	Protein tyrosine phosphatase receptor type D	R.YSAPANLYVR.E	1	1.79	0.17	-2.65
IPI00375547	Protein tyrosine phosphatase receptor type D	R.YSAPANLYVR.E	2	2.87	0.33	-2.22
IPI00375547	Protein tyrosine phosphatase receptor type D	R.YSVAGLSPYSDYEFR.V	2	3.48	0.46	-2.49
IPI00375746	Isoform 1 of Guanylate-binding protein 6	R.KCFVFDRPTNDKDLLANIEKVSEK.Q	3	3.40	0.14	-4.81
IPI00375803	Isoform 1 of GON-4-like protein	R.DPLREQKDLAFAQAYLTR.V	3	2.45	0.16	
IPI00375879	Uncharacterized protein KIAA1467	K.ETPATSAVTSDQK.S	2	3.99	0.44	-4.97
IPI00375879	Uncharacterized protein KIAA1467	R.IKFVEAPYEI.-	2	3.22	0.23	-2.18
IPI00375879	Uncharacterized protein KIAA1467	R.TGNPVGRPVK.Y	2	2.41	0.20	-2.66
IPI00375881	Polycystic kidney disease 1-like protein 3	K.WRFFTGKRNILDTSIILISFILLGLDM*K.S	3	2.96	0.23	
IPI00376087	putative binding protein 7a5	K.VISKEQVM*FMSDSVFTTR.N	2	2.81	0.09	
IPI00376131	Similar to Leucine rich repeat neuronal 6C	R.CLNPGDLAALPALEELDLSENAIAHVEPGAFANLPR.L	3	3.97	0.33	-3.43
IPI00376131	Similar to Leucine rich repeat neuronal 6C	R.CLNPGDLAALPALEELDLSENAIAHVEPGAFANLPR.L	4	3.72	0.25	-3.88
IPI00376131	Similar to Leucine rich repeat neuronal 6C	R.LTAVPDGIPAETR.L	2	2.80	0.28	-2.87
IPI00376131	Similar to Leucine rich repeat neuronal 6C	R.RAFAGLLALEELTLER.C	3	2.96	0.14	-3.76
IPI00376237	Isoform 2 of Transcription factor LBX2	K.TASRAENNSQACRPQR.R	2	2.22	0.06	-3.26
IPI00376383	Centrosomal protein 110kDa	K.EELEKVTRLTQLEQSALQAELEKER.Q	3	2.44	0.17	-7.77
IPI00376394	Sulfhydryl oxidase 2 precursor	K.LFHTLTVEASTHPDALVGTGFEDDPQAVLQTM*R.R	4	3.94	0.35	-1.28
IPI00376394	Sulfhydryl oxidase 2 precursor	K.TPDQAILWLWK.K	2	3.91	0.45	-3.37
IPI00376394	Sulfhydryl oxidase 2 precursor	R.AFFSSYLK.S	2	2.12	0.17	-1.47
IPI00376394	Sulfhydryl oxidase 2 precursor	R.ALDGDKAFLEK.L	2	2.94	0.30	-2.13
IPI00376394	Sulfhydryl oxidase 2 precursor	R.DNLLDTYSADQGDSSEGGTLAR.G	2	6.07	0.57	-1.61
IPI00376394	Sulfhydryl oxidase 2 precursor	R.DNLLDTYSADQGDSSEGGTLAR.G	3	4.89	0.39	-1.26
IPI00376394	Sulfhydryl oxidase 2 precursor	R.EVILDLIPYESIVVTR.A	2	5.12	0.46	-4.83
IPI00376394	Sulfhydryl oxidase 2 precursor	R.EVILDLIPYESIVVTR.A	3	3.40	0.30	-4.88
IPI00376394	Sulfhydryl oxidase 2 precursor	R.ISGIFLTNHIK.W	2	2.66	0.15	-3.16
IPI00376394	Sulfhydryl oxidase 2 precursor	R.ISGIFLTNHIK.W	3	2.92	0.26	-3.70
IPI00376394	Sulfhydryl oxidase 2 precursor	R.LAGHLSEDPRFPK.L	2	2.76	0.15	-2.72
IPI00376394	Sulfhydryl oxidase 2 precursor	R.LDPIQPSDVLSLLDNR.G	2	4.05	0.47	-4.32
IPI00376394	Sulfhydryl oxidase 2 precursor	R.LDPIQPSDVLSLLDNR.G	3	4.68	0.35	-3.81
IPI00376427	Neural cell adhesion molecule 2 precursor	K.AGEDEKQAFLQVFVQPH.I	2	3.77	0.41	-2.67
IPI00376427	Neural cell adhesion molecule 2 precursor	K.AGEDEKQAFLQVFVQPH.I	3	4.86	0.41	-2.19
IPI00376427	Neural cell adhesion molecule 2 precursor	K.AGEDEKQAFLQVFVQPHIIQLK.N	4	4.52	0.38	-1.64
IPI00376427	Neural cell adhesion molecule 2 precursor	K.GQGDYSKIEIFQTLPVREPSPPSIHGQPSSGK.S	4	5.42	0.43	-6.10
IPI00376427	Neural cell adhesion molecule 2 precursor	K.GQHGSSSLHIK.D	2	3.30	0.33	-2.96
IPI00376427	Neural cell adhesion molecule 2 precursor	K.GQHGSSSLHIKDVK.L	2	4.04	0.42	-4.30
IPI00376427	Neural cell adhesion molecule 2 precursor	K.GQTQEATVVLEIYQK.L	2	3.47	0.31	-5.76
IPI00376427	Neural cell adhesion molecule 2 precursor	K.GSNTELTVR.N	1	1.94	0.17	-1.76
IPI00376427	Neural cell adhesion molecule 2 precursor	K.GSNTELTVR.N	2	2.73	0.17	-1.44

IPI00376427	Neural cell adhesion molecule 2 precursor	K.IEIFQTLPVREPSPPSIHGQPSSGK.S	2	4.03	0.46	-2.67
IPI00376427	Neural cell adhesion molecule 2 precursor	K.IEIFQTLPVREPSPPSIHGQPSSGK.S	3	5.37	0.54	-3.49
IPI00376427	Neural cell adhesion molecule 2 precursor	K.IEIFQTLPVREPSPPSIHGQPSSGK.S	4	3.52	0.35	-2.95
IPI00376427	Neural cell adhesion molecule 2 precursor	K.IIELSQTTAK.V	1	2.24	0.20	-3.97
IPI00376427	Neural cell adhesion molecule 2 precursor	K.IIELSQTTAK.V	2	3.41	0.24	-2.54
IPI00376427	Neural cell adhesion molecule 2 precursor	K.LIEENEKYILK.G	2	3.98	0.23	-3.44
IPI00376427	Neural cell adhesion molecule 2 precursor	K.LSITKQDDGGAPILEYIVK.Y	2	2.80	0.17	-1.68
IPI00376427	Neural cell adhesion molecule 2 precursor	K.LSITKQDDGGAPILEYIVK.Y	3	4.06	0.34	-2.88
IPI00376427	Neural cell adhesion molecule 2 precursor	K.LTFREVVSPQEFK.Q	2	2.97	0.22	-2.76
IPI00376427	Neural cell adhesion molecule 2 precursor	K.LTFREVVSPQEFK.Q	3	4.20	0.29	-2.57
IPI00376427	Neural cell adhesion molecule 2 precursor	K.M*ILEIAPTSDNDFGR.Y	2	3.91	0.35	-4.86
IPI00376427	Neural cell adhesion molecule 2 precursor	K.QAFLQVFVQPH.I	2	2.99	0.38	-4.05
IPI00376427	Neural cell adhesion molecule 2 precursor	K.QDDGGAPILEYIVK.Y	2	4.17	0.37	-3.98
IPI00376427	Neural cell adhesion molecule 2 precursor	K.QGEDAEVVCR.V	2	3.49	0.39	-2.48
IPI00376427	Neural cell adhesion molecule 2 precursor	K.RAVDGFTFTEGDKSPDGR.I	3	3.48	0.41	-3.51
IPI00376427	Neural cell adhesion molecule 2 precursor	K.RAVDGFTFTEGDKSPDGR.I	4	3.09	0.22	-2.67
IPI00376427	Neural cell adhesion molecule 2 precursor	K.SDEGIYR.C	2	1.77	0.07	-3.52
IPI00376427	Neural cell adhesion molecule 2 precursor	K.SFKLSITKQDDGGAPILEYIVK.Y	3	4.60	0.46	-5.13
IPI00376427	Neural cell adhesion molecule 2 precursor	K.SFKLSITKQDDGGAPILEYIVK.Y	4	3.24	0.14	-3.66
IPI00376427	Neural cell adhesion molecule 2 precursor	K.SM*YLDIEYAPK.F	1	2.32	0.23	-3.84
IPI00376427	Neural cell adhesion molecule 2 precursor	K.SM*YLDIEYAPK.F	2	4.15	0.41	-4.03
IPI00376427	Neural cell adhesion molecule 2 precursor	K.VELSVGESK.F	1	2.00	0.30	-4.08
IPI00376427	Neural cell adhesion molecule 2 precursor	K.VELSVGESK.F	2	2.88	0.26	-2.87
IPI00376427	Neural cell adhesion molecule 2 precursor	K.VSFNKPDSHGGVPIHHYQVDVK.E	3	4.13	0.35	-5.10
IPI00376427	Neural cell adhesion molecule 2 precursor	R.ASGSPEPAISWFR.N	2	3.07	0.29	-3.81
IPI00376427	Neural cell adhesion molecule 2 precursor	R.ATNKAGEDEKQAFLQVFVQPH.I	3	4.01	0.46	-3.19
IPI00376427	Neural cell adhesion molecule 2 precursor	R.AVDGFTFTEGDK.S	1	2.30	0.41	-1.77
IPI00376427	Neural cell adhesion molecule 2 precursor	R.AVDGFTFTEGDK.S	2	3.62	0.46	-1.17
IPI00376427	Neural cell adhesion molecule 2 precursor	R.AVDGFTFTEGDKSPDGR.I	2	3.92	0.47	-2.63
IPI00376427	Neural cell adhesion molecule 2 precursor	R.AVDGFTFTEGDKSPDGR.I	3	2.84	0.33	-2.61
IPI00376427	Neural cell adhesion molecule 2 precursor	R.AVDGFTFTEGDKSPDGRIEVK.G	3	3.20	0.25	-3.86
IPI00376427	Neural cell adhesion molecule 2 precursor	R.DIIVIVNVPPAISM*PQK.S	2	3.96	0.32	-6.67
IPI00376427	Neural cell adhesion molecule 2 precursor	R.EVVSPQEFK.Q	1	1.81	0.17	-2.49
IPI00376427	Neural cell adhesion molecule 2 precursor	R.EVVSPQEFK.Q	2	2.27	0.15	-3.90
IPI00376427	Neural cell adhesion molecule 2 precursor	R.EVVSPQEFKQGEDAEVVCR.V	2	4.55	0.51	-2.05
IPI00376427	Neural cell adhesion molecule 2 precursor	R.EVVSPQEFKQGEDAEVVCR.V	3	3.63	0.45	-0.87
IPI00376427	Neural cell adhesion molecule 2 precursor	R.FQEYILALADVPSSPYGVK.I	2	4.65	0.53	-4.43
IPI00376427	Neural cell adhesion molecule 2 precursor	R.FQEYILALADVPSSPYGVK.I	3	3.98	0.37	-3.70
IPI00376427	Neural cell adhesion molecule 2 precursor	R.KM*ILEIAPTSDNDFGR.Y	3	2.90	0.08	-3.11
IPI00376427	Neural cell adhesion molecule 2 precursor	R.LGYSEPTVYEFSM*PPKPNIIK.D	2	4.04	0.45	-1.24
IPI00376427	Neural cell adhesion molecule 2 precursor	R.LGYSEPTVYEFSM*PPKPNIIK.D	3	3.80	0.51	-1.94
IPI00376427	Neural cell adhesion molecule 2 precursor	R.LTIYNANIEDAGIYR.C	2	4.94	0.51	-5.08

IPI00376427	Neural cell adhesion molecule 2 precursor	R.NIINSDGGPYVCR.A	2	3.83	0.42	-4.04
IPI00376427	Neural cell adhesion molecule 2 precursor	R.SRLTIYNANIEDAGIYR.C	2	4.08	0.42	-3.27
IPI00376427	Neural cell adhesion molecule 2 precursor	R.SRLTIYNANIEDAGIYR.C	3	3.18	0.22	-2.89
IPI00376427	Neural cell adhesion molecule 2 precursor	R.VSSSPAPAVSWLYHNEEVTTISDNR.L	2	4.87	0.59	-3.32
IPI00376427	Neural cell adhesion molecule 2 precursor	R.VSSSPAPAVSWLYHNEEVTTISDNR.L	3	3.72	0.43	-4.36
IPI00376587	Uncharacterized protein ENSP00000345065	R.HFKSKTDSNSKK.C	2	1.83	0.19	
IPI00376689	Isoform 1 of Protein KIAA1199 precursor	K.GGALELHGQKK.L	2	2.91	0.12	-2.63
IPI00376689	Isoform 1 of Protein KIAA1199 precursor	K.IFQVVPIPVVK.K	2	3.19	0.32	-4.42
IPI00376689	Isoform 1 of Protein KIAA1199 precursor	K.NDFPSHPLYLEGALTR.S	3	3.39	0.34	-1.24
IPI00376689	Isoform 1 of Protein KIAA1199 precursor	K.SGTVIHSDRFDTYR.S	2	3.09	0.33	-2.43
IPI00376689	Isoform 1 of Protein KIAA1199 precursor	R.ADEGIQPDPYYGLK.Y	2	3.71	0.17	-3.49
IPI00376689	Isoform 1 of Protein KIAA1199 precursor	R.AEVGLLSR.N	2	2.30	0.08	-2.48
IPI00376689	Isoform 1 of Protein KIAA1199 precursor	R.DLSIHHTFSR.C	2	2.69	0.30	-2.99
IPI00376689	Isoform 1 of Protein KIAA1199 precursor	R.FADNGIGLTLASGGTFPYDDGSKQEIK.N	3	3.54	0.24	-2.95
IPI00376689	Isoform 1 of Protein KIAA1199 precursor	R.ILSVAVNDEGSR.N	2	3.13	0.39	-0.96
IPI00376689	Isoform 1 of Protein KIAA1199 precursor	R.KFVALEGR.H	2	1.87	0.15	-1.91
IPI00376689	Isoform 1 of Protein KIAA1199 precursor	R.LVQYLNAVPDGR.I	2	2.54	0.11	-3.24
IPI00376689	Isoform 1 of Protein KIAA1199 precursor	R.NIIVM*GEM*EDK.C	2	2.84	0.21	-3.49
IPI00376689	Isoform 1 of Protein KIAA1199 precursor	R.TLQM*DKVEQSYPGR.S	3	3.17	0.34	-2.81
IPI00377045	Alpha3A	R.FNKTKTFRINQLLQDTPVASPR.S	3	3.41	0.15	
IPI00377077	Isoform 3 of Astrotactin-2 precursor	R.AGAGAGTGAGAAAAAASPGSPGSAGTAAESR.L	3	4.31	0.38	-3.77
IPI00382420	Ig lambda chain V-I region HA	K.SGTSASLAISGLR.S	1	2.82	0.39	
IPI00382420	Ig lambda chain V-I region HA	K.SGTSASLAISGLR.S	2	3.92	0.23	
IPI00382421	Ig lambda chain V-I region NEW	K.SGTSATLGITGLR.T	2	2.63	0.18	
IPI00382442	Ig lambda chain V-V region DEL	-.YVLSQPPSVSVAPGQTAR.I	2	3.38	0.43	
IPI00382442	Ig lambda chain V-V region DEL	R.FSGSNSGNTAALTISR.V	2	3.46	0.21	
IPI00382474	Ig heavy chain V-III region TRO	R.DNAQKSLYLZM*BSLR.T	3	4.53	0.06	
IPI00382476	Ig heavy chain V-III region WEA	-.QVQLVDSGGGLVEPGGSLR.L	2	4.00	0.09	
IPI00382476	Ig heavy chain V-III region WEA	K.NSLYLQMSSLR.A	2	3.54	0.30	
IPI00382478	Ig heavy chain V-III region TIL	C.EVQLLESGGGLVQPGGSLR.L	1	4.02	0.09	
IPI00382478	Ig heavy chain V-III region TIL	C.EVQLLESGGGLVQPGGSLR.L	2	5.62	0.07	
IPI00382478	Ig heavy chain V-III region TIL	R.FTISRDDSK.N	2	2.52	0.15	
IPI00382481	Ig heavy chain V-III region BUT	-.EVQLVETGGGLIQPGGSLR.L	2	5.85	0.23	
IPI00382481	Ig heavy chain V-III region BUT	-.EVQLVETGGGLIQPGGSLR.L	3	4.13	0.05	
IPI00382481	Ig heavy chain V-III region BUT	R.BTVYLQM*BSLR.A	2	2.88	0.14	
IPI00382481	Ig heavy chain V-III region BUT	R.BTVYLQMBSLR.A	2	4.02	0.14	
IPI00382482	Ig heavy chain V-III region CAM	R.DBSKBTLYLQMNSLR.A	2	4.43	0.10	
IPI00382482	Ig heavy chain V-III region CAM	R.LSCAASGFTFSNYAMHWVR.Q	2	3.75	0.31	
IPI00382486	Ig heavy chain V-III region NIE	R.LSCAASGFTFSR.Y	1	2.80	0.05	
IPI00382486	Ig heavy chain V-III region NIE	R.LSCAASGFTFSR.Y	2	4.44	0.08	
IPI00382488	Ig heavy chain V-III region HIL	-.QVKLVQAGGGVVQPGR.S	1	3.69	0.23	
IPI00382488	Ig heavy chain V-III region HIL	-.QVKLVQAGGGVVQPGR.S	2	4.10	0.21	

IPI00382488	Ig heavy chain V-III region HIL	R.TEDTAVYYCAR.D	2	4.05	0.24	
IPI00382493	Ig heavy chain V-III region WAS	C.EVQLLESGGGLVQPGGSLR.L	1	4.02	0.09	
IPI00382493	Ig heavy chain V-III region WAS	C.EVQLLESGGGLVQPGGSLR.L	2	5.62	0.07	
IPI00382493	Ig heavy chain V-III region WAS	K.NTLYLQM*NR.L	2	1.98	0.23	
IPI00382499	Ig heavy chain V-III region JON	-.DVQLVESGGGLVKPGGSLR.L	2	5.73	0.33	
IPI00382499	Ig heavy chain V-III region JON	-.DVQLVESGGGLVKPGGSLR.L	3	5.56	0.18	
IPI00382500	Ig heavy chain V-III region GAL	-.EVQLVESGGDLVQPGR.S	2	3.14	0.24	
IPI00382500	Ig heavy chain V-III region GAL	-.EVQLVESGGDLVQPGR.S	3	4.56	0.09	
IPI00382500	Ig heavy chain V-III region GAL	K.GLEWVANIK.Z	1	2.05	0.11	
IPI00382500	Ig heavy chain V-III region GAL	K.GLEWVANIK.Z	2	3.69	0.25	
IPI00382500	Ig heavy chain V-III region GAL	K.NSLYLQM*NSLR.A	2	3.76	0.14	
IPI00382500	Ig heavy chain V-III region GAL	K.NSLYLQMNSLR.A	1	3.55	0.11	
IPI00382500	Ig heavy chain V-III region GAL	K.NSLYLQMNSLR.A	2	4.01	0.17	
IPI00382500	Ig heavy chain V-III region GAL	R.DNAKNSLYLQM*NSLR.A	2	4.82	0.44	
IPI00382500	Ig heavy chain V-III region GAL	R.DNAKNSLYLQM*NSLR.A	3	4.39	0.37	
IPI00382500	Ig heavy chain V-III region GAL	R.VEDTALYYCAR.D	2	4.55	0.38	
IPI00382515	CDNA FLJ30384 fis, clone BRACE2008114	-.MMSFTCFSTFNFHRTLSPLQVGK.L	3	2.97	0.20	
IPI00382606	Factor VII active site mutant immunoconjugate	C.DKTHTCPPCPAPELLGGPSVFLFPPKPK.D	3	6.55	0.38	
IPI00382606	Factor VII active site mutant immunoconjugate	K.ALPAPIEK.T	1	1.81	0.11	
IPI00382606	Factor VII active site mutant immunoconjugate	K.CKVSNKALPAPIEK.T	2	2.28	0.15	
IPI00382606	Factor VII active site mutant immunoconjugate	K.DTLM*ISR.T	2	2.48	0.09	-3.74
IPI00382606	Factor VII active site mutant immunoconjugate	K.DTLMISR.T	1	2.38	0.13	
IPI00382606	Factor VII active site mutant immunoconjugate	K.DTLMISR.T	2	2.45	0.16	
IPI00382606	Factor VII active site mutant immunoconjugate	K.FNWYVDGVEVHNAK.T	1	3.93	0.41	
IPI00382606	Factor VII active site mutant immunoconjugate	K.FNWYVDGVEVHNAK.T	2	5.51	0.51	
IPI00382606	Factor VII active site mutant immunoconjugate	K.FNWYVDGVEVHNAK.T	3	3.99	0.38	
IPI00382606	Factor VII active site mutant immunoconjugate	K.GFYPSDIAVEWESNGQPENNYK.T	2	4.88	0.31	
IPI00382606	Factor VII active site mutant immunoconjugate	K.GFYPSDIAVEWESNGQPENNYK.T	3	4.56	0.26	
IPI00382606	Factor VII active site mutant immunoconjugate	K.GFYPSDIAVEWESNGQPENNYKTTPPVLDSDGSFFLYSK.L	3	4.64	0.25	
IPI00382606	Factor VII active site mutant immunoconjugate	K.GQPREPQVYTLPPSR.E	2	2.89	0.13	
IPI00382606	Factor VII active site mutant immunoconjugate	K.GQPREPQVYTLPPSR.E	3	2.92	0.17	
IPI00382606	Factor VII active site mutant immunoconjugate	K.GQPREPQVYTLPPSRDELTK.N	3	4.51	0.32	
IPI00382606	Factor VII active site mutant immunoconjugate	K.NQVSLTCLVK.G	1	2.26	0.22	
IPI00382606	Factor VII active site mutant immunoconjugate	K.NQVSLTCLVK.G	2	2.44	0.28	-2.15
IPI00382606	Factor VII active site mutant immunoconjugate	K.SCDKTHTCPPCPAPELLGGPSVFLFPPKPK.D	3	6.71	0.47	
IPI00382606	Factor VII active site mutant immunoconjugate	K.THTCPPCPAPELLGGPSVFLFPPKPK.D	2	3.81	0.39	
IPI00382606	Factor VII active site mutant immunoconjugate	K.THTCPPCPAPELLGGPSVFLFPPKPK.D	3	6.29	0.52	
IPI00382606	Factor VII active site mutant immunoconjugate	K.TKPREEQYNSTYR.V	2	2.99	0.10	
IPI00382606	Factor VII active site mutant immunoconjugate	K.TTPPVLDSDGSFFLYSK.L	1	3.22	0.41	
IPI00382606	Factor VII active site mutant immunoconjugate	K.TTPPVLDSDGSFFLYSK.L	2	3.42	0.37	
IPI00382606	Factor VII active site mutant immunoconjugate	K.TTPPVLDSDGSFFLYSK.L	3	4.11	0.39	
IPI00382606	Factor VII active site mutant immunoconjugate	K.VSNKALPAPIEK.T	2	3.33	0.18	

IPI00382606	Factor VII active site mutant immunoconjugate	K.WYVDGVEVHNAK.T	1	2.91	0.35	
IPI00382606	Factor VII active site mutant immunoconjugate	K.WYVDGVEVHNAK.T	2	3.90	0.46	
IPI00382606	Factor VII active site mutant immunoconjugate	K.WYVDGVEVHNAK.T	3	2.99	0.21	
IPI00382606	Factor VII active site mutant immunoconjugate	R.CPAPELLGGPSVFLFPPKPK.D	2	4.00	0.37	
IPI00382606	Factor VII active site mutant immunoconjugate	R.CPAPELLGGPSVFLFPPKPK.D	3	6.31	0.49	
IPI00382606	Factor VII active site mutant immunoconjugate	R.CPAPELLGGPSVFLFPPKPKDTLM*ISR.T	3	3.77	0.23	
IPI00382606	Factor VII active site mutant immunoconjugate	R.EPQVYTLPPSRDELTK.N	2	3.97	0.21	
IPI00382606	Factor VII active site mutant immunoconjugate	R.EPQVYTLPPSRDELTKNQVSLTCLVK.G	3	4.03	0.23	
IPI00382606	Factor VII active site mutant immunoconjugate	R.TPEVTCVVVDVSHED.P	2	5.50	0.52	
IPI00382606	Factor VII active site mutant immunoconjugate	R.VAQVIIPSTYVPGTTNHDIALLR.L	3	2.74	0.15	-1.66
IPI00382606	Factor VII active site mutant immunoconjugate	R.VVSVLTVLHQDWLNGK.E	1	4.22	0.41	
IPI00382606	Factor VII active site mutant immunoconjugate	R.VVSVLTVLHQDWLNGK.E	2	5.39	0.46	
IPI00382606	Factor VII active site mutant immunoconjugate	R.VVSVLTVLHQDWLNGK.E	3	3.42	0.38	
IPI00382606	Factor VII active site mutant immunoconjugate	R.VVSVLTVLHQDWLNGKEYK.C	2	5.56	0.40	
IPI00382606	Factor VII active site mutant immunoconjugate	R.VVSVLTVLHQDWLNGKEYK.C	3	4.97	0.40	
IPI00382682	Putative matrix cell adhesion molecule-3	R.DTSTSTVYM*DLSSLR.S	2	3.22	0.29	
IPI00382682	Putative matrix cell adhesion molecule-3	R.SDDTAVYFCAR.E	2	3.45	0.27	
IPI00382756	Isoform 2 of Pleiotropic regulator 1	K.EKGPQNATDSYVHKQYPANQGQEVEYFVAGVALTADTKIQR.M	4	4.03	0.23	1.70
IPI00382938	IGLV4-3 protein	K.AAPSVTLFPPSSEELQANK.A	1	4.04	0.50	
IPI00382938	IGLV4-3 protein	K.AAPSVTLFPPSSEELQANK.A	2	3.15	0.34	-1.50
IPI00382938	IGLV4-3 protein	K.AAPSVTLFPPSSEELQANK.A	3	3.32	0.13	
IPI00382938	IGLV4-3 protein	K.ADSSPVKAGVETTTPSK.Q	1	3.85	0.37	
IPI00382938	IGLV4-3 protein	K.ADSSPVKAGVETTTPSK.Q	2	3.50	0.38	
IPI00382938	IGLV4-3 protein	K.ADSSPVKAGVETTTPSK.Q	3	3.46	0.35	
IPI00382938	IGLV4-3 protein	K.ADSSPVKAGVETTTPSKQSNNK.Y	2	5.35	0.42	
IPI00382938	IGLV4-3 protein	K.ADSSPVKAGVETTTPSKQSNNK.Y	3	4.61	0.23	
IPI00382938	IGLV4-3 protein	K.AGVETTTPSK.Q	2	2.62	0.09	-3.23
IPI00382938	IGLV4-3 protein	K.AGVETTTPSKQSNNK.Y	2	4.14	0.32	
IPI00382938	IGLV4-3 protein	K.AGVETTTPSKQSNNKYAASSYLSLTPEQWK.S	3	5.47	0.40	
IPI00382938	IGLV4-3 protein	K.ATLVCLISDFYPGAVTVAWK.A	2	5.07	0.50	
IPI00382938	IGLV4-3 protein	K.ATLVCLISDFYPGAVTVAWK.A	3	3.53	0.31	-3.63
IPI00382938	IGLV4-3 protein	K.ATLVCLISDFYPGAVTVAWKADSSPVK.A	3	3.81	0.20	
IPI00382938	IGLV4-3 protein	K.QSNNKYAASSYLSLTPEQWK.S	2	5.38	0.37	
IPI00382938	IGLV4-3 protein	K.QSNNKYAASSYLSLTPEQWK.S	3	4.26	0.33	
IPI00382938	IGLV4-3 protein	K.YAASSYLSLTPEQWK.S	1	3.49	0.43	
IPI00382938	IGLV4-3 protein	K.YAASSYLSLTPEQWK.S	2	5.52	0.47	
IPI00382938	IGLV4-3 protein	K.YAASSYLSLTPEQWK.S	3	3.44	0.22	
IPI00382938	IGLV4-3 protein	R.FM*GSSSGADR.Y	2	2.49	0.25	
IPI00382938	IGLV4-3 protein	R.SYSCQVTHEGSTVEK.T	1	3.84	0.43	
IPI00382938	IGLV4-3 protein	R.SYSCQVTHEGSTVEK.T	2	3.17	0.41	-4.10
IPI00382938	IGLV4-3 protein	R.SYSCQVTHEGSTVEKTVAPTECS.-	2	5.35	0.45	
IPI00382938	IGLV4-3 protein	R.SYSCQVTHEGSTVEKTVAPTECS.-	3	2.78	0.26	

IPI00383016	Immunglobulin light chain variable region (Fragment)	K.LLIYDNNKRPSGVPDR.F	2	2.91	0.12	
IPI00383032	Isoform 2 of Hepatitis A virus cellular receptor 2 precursor	R.AEVGQNAYLPCFYTPAAPGNLVPVCWGK.G	3	3.71	0.35	-5.43
IPI00383032	Isoform 2 of Hepatitis A virus cellular receptor 2 precursor	R.DVNYWTSR.Y	2	2.22	0.26	-2.19
IPI00383032	Isoform 2 of Hepatitis A virus cellular receptor 2 precursor	R.IQIPGIM*NDEKFNLK.L	2	2.84	0.19	-2.14
IPI00383594	melanoma ubiquitous mutated protein	R.GGNSAQKASLCLNGSSLSEDDTERDMGSK.G	3	2.76	0.24	2.67
IPI00383603	Anti-thyroglobulin light chain variable region (Fragment)	K.VTISCSGSSSNIGK.N	2	4.04	0.49	
IPI00383680	Ribophorin II	K.ASLDRPFTNLESAFYSIVGLSSLGAQVPDAK.K	3	3.07	0.09	-4.42
IPI00383680	Ribophorin II	R.LSKEETVLATVQALQTASHLSQQADLR.S	3	3.22	0.29	-3.05
IPI00383680	Ribophorin II	R.LSKEETVLATVQALQTASHLSQQADLR.S	4	3.63	0.34	-4.68
IPI00383732	VH3 protein (Fragment)	K.GPSVFPLAPSSK.S	1	3.15	0.35	
IPI00383732	VH3 protein (Fragment)	K.GPSVFPLAPSSK.S	2	3.30	0.36	
IPI00383732	VH3 protein (Fragment)	K.NTLYLQM*TSLR.V	2	2.68	0.11	
IPI00383732	VH3 protein (Fragment)	K.NTLYLQMTSLR.V	2	2.59	0.28	
IPI00383732	VH3 protein (Fragment)	R.VEDTAVYYCAK.D	2	3.33	0.21	
IPI00383808	Ig kappa chain V-IV region STH (Fragment)	-.DIVM*TQSPDSLVVSLGER.A	2	4.08	0.23	
IPI00383832	Protein kinase C-binding protein RACK8	-.MGETKIIYHLDGQETPYLVKLPLPAER.V	3	2.91	0.09	-4.40
IPI00383887	Immunoblobulin heavy chain (Fragment)	K.NTLYLQMNSLR.A	1	3.28	0.09	
IPI00383887	Immunoblobulin heavy chain (Fragment)	K.NTLYLQMNSLR.A	2	4.45	0.07	
IPI00383887	Immunoblobulin heavy chain (Fragment)	R.DNSKNTLYLQM*NSLR.A	2	3.02	0.07	
IPI00383887	Immunoblobulin heavy chain (Fragment)	R.DNSKNTLYLQM*NSLR.A	3	3.90	0.23	
IPI00383951	Isoform 3 of Protein sidekick-1 precursor	R.DDSELTTYSSEYK.Y	2	3.44	0.48	-2.06
IPI00383951	Isoform 3 of Protein sidekick-1 precursor	R.M*GALLQR.Q	2	2.22	0.18	-2.78
IPI00383953	VH4 heavy chain variable region precursor (Fragment)	K.LSSVTAADTAVYYCAR.-	2	5.61	0.50	
IPI00383953	VH4 heavy chain variable region precursor (Fragment)	K.LSSVTAADTAVYYCAR.-	3	4.55	0.30	
IPI00384016	Full-length cDNA 5-PRIME end of clone CS0DJ009YL13 of T cells (Jurkat cell line) of Homo sapiens (Fragment)	K.TPAFAESVTEGDVR.W	2	3.90	0.45	-2.89
IPI00384016	Full-length cDNA 5-PRIME end of clone CS0DJ009YL13 of T cells (Jurkat cell line) of Homo sapiens (Fragment)	K.VEGGTPLFTLR.K	2	2.82	0.34	-1.83
IPI00384051	Uncharacterized protein PSME2	R.QNLFQEAEEFLYR.F	2	3.58	0.33	-4.93
IPI00384225	Meteorin precursor	R.AGGALELLLAEGPGPAGGR.C	2	2.78	0.28	-3.71
IPI00384225	Meteorin precursor	R.AGGALELLLAEGPGPAGGR.C	3	2.30	0.16	-3.05
IPI00384391	Myosin-reactive immunoglobulin heavy chain variable region (Fragment)	-.EVQLVESGGGVVQPGGSLR.L	2	3.96	0.22	

IPI00384391	Myosin-reactive immunoglobulin heavy chain variable region (Fragment)	-.EVQLVESGGGVVQPGGSLR.L	3	4.24	0.07	
IPI00384391	Myosin-reactive immunoglobulin heavy chain variable region (Fragment)	K.NSLYLQM*NSLR.A	2	3.76	0.14	
IPI00384391	Myosin-reactive immunoglobulin heavy chain variable region (Fragment)	K.NSLYLQMNSLR.A	1	3.55	0.11	
IPI00384391	Myosin-reactive immunoglobulin heavy chain variable region (Fragment)	K.NSLYLQMNSLR.A	2	4.01	0.17	
IPI00384391	Myosin-reactive immunoglobulin heavy chain variable region (Fragment)	R.AEDTALYYCAK.H	2	3.60	0.41	
IPI00384391	Myosin-reactive immunoglobulin heavy chain variable region (Fragment)	R.DNSKNSLYLQM*NSLR.A	2	4.94	0.16	
IPI00384391	Myosin-reactive immunoglobulin heavy chain variable region (Fragment)	R.DNSKNSLYLQM*NSLR.A	3	4.24	0.22	
IPI00384392	Myosin-reactive immunoglobulin heavy chain variable region (Fragment)	K.NTLYLQMNSLR.A	1	3.28	0.09	
IPI00384392	Myosin-reactive immunoglobulin heavy chain variable region (Fragment)	K.NTLYLQMNSLR.A	2	4.45	0.07	
IPI00384392	Myosin-reactive immunoglobulin heavy chain variable region (Fragment)	R.AEDTAFYYCAR.D	2	3.82	0.39	
IPI00384392	Myosin-reactive immunoglobulin heavy chain variable region (Fragment)	R.DNSKNTLYLQM*NSLR.A	2	3.02	0.07	
IPI00384392	Myosin-reactive immunoglobulin heavy chain variable region (Fragment)	R.DNSKNTLYLQM*NSLR.A	3	3.90	0.23	
IPI00384395	Myosin-reactive immunoglobulin heavy chain variable region	K.NQFSLQLNSVTPEDTAVYYCAR.-	2	4.75	0.24	
IPI00384395	Myosin-reactive immunoglobulin heavy chain variable region	K.SRITINPDTSK.N	2	2.84	0.24	
IPI00384400	Myosin-reactive immunoglobulin heavy chain variable region (Fragment)	-.EVQLVESGGGVVQPGR.S	2	3.37	0.07	
IPI00384400	Myosin-reactive immunoglobulin heavy chain variable region (Fragment)	K.NM*M*DLQMNSLR.A	2	2.86	0.16	
IPI00384402	Myosin-reactive immunoglobulin kappa chain variable region (Fragment)	R.ASQSVSSSYLAWYQQKPGQAPR.L	2	5.52	0.40	
IPI00384402	Myosin-reactive immunoglobulin kappa chain variable region (Fragment)	R.ASQSVSSSYLAWYQQKPGQAPR.L	3	3.59	0.29	
IPI00384402	Myosin-reactive immunoglobulin kappa chain variable region (Fragment)	R.ATGIPDRFSGSGSETDFTLTISR.L	3	3.38	0.11	
IPI00384402	Myosin-reactive immunoglobulin kappa chain variable region (Fragment)	R.FSGSGSETDFTLTISR.L	2	4.26	0.48	
IPI00384404	Rheumatoid factor RF-ET9 (Fragment)	K.NTLYLQM*NSLK.T	2	2.43	0.12	
IPI00384404	Rheumatoid factor RF-ET9 (Fragment)	K.NTLYLQMNSLK.T	2	3.59	0.08	

IPI00384404	Rheumatoid factor RF-ET9 (Fragment)	R.DDSKNTLYLQM*NSLK.T	2	4.53	0.27	
IPI00384404	Rheumatoid factor RF-ET9 (Fragment)	R.DDSKNTLYLQM*NSLK.T	3	3.61	0.23	
IPI00384404	Rheumatoid factor RF-ET9 (Fragment)	R.FTISRDDSK.N	2	2.52	0.15	
IPI00384407	Myosin-reactive immunoglobulin heavy chain variable region (Fragment)	-.EVQLVESGAEVK.K	2	5.25	0.25	
IPI00384407	Myosin-reactive immunoglobulin heavy chain variable region (Fragment)	R.DTSTSTVYMELSSLR.S	2	3.00	0.09	
IPI00384542	Isoform 2 of Nidogen-1 precursor	C.LSRQELFPFGPGQGDLELEDGDDFVSPALELSGALR.F	3	7.07	0.58	-3.86
IPI00384542	Isoform 2 of Nidogen-1 precursor	K.AFLHVPAK.V	2	2.41	0.09	-2.92
IPI00384542	Isoform 2 of Nidogen-1 precursor	K.ALEGLQYPFAVTSYGK.N	2	4.93	0.55	-4.69
IPI00384542	Isoform 2 of Nidogen-1 precursor	K.ESHPGLFPPTFGAVAPFLADLDTTDGLGK.V	3	5.87	0.56	-4.74
IPI00384542	Isoform 2 of Nidogen-1 precursor	K.ETDAFQPHKQTR.L	2	2.79	0.32	-2.24
IPI00384542	Isoform 2 of Nidogen-1 precursor	K.IETSYM*DGTNR.R	2	2.55	0.23	-2.29
IPI00384542	Isoform 2 of Nidogen-1 precursor	K.M*VYWTDITEPSIGR.A	2	4.28	0.40	-2.60
IPI00384542	Isoform 2 of Nidogen-1 precursor	K.NGFSITGGEFTR.Q	2	2.97	0.28	-4.62
IPI00384542	Isoform 2 of Nidogen-1 precursor	K.VIIGLAFDCVDK.M	2	3.99	0.45	-3.02
IPI00384542	Isoform 2 of Nidogen-1 precursor	K.VYYREDLSPSITQR.A	2	4.43	0.45	-2.56
IPI00384542	Isoform 2 of Nidogen-1 precursor	R.AECLNPSQPSR.R	2	3.02	0.24	-1.38
IPI00384542	Isoform 2 of Nidogen-1 precursor	R.ASLHGGEPTTIIRQDLGSPEGIAVDHLGR.N	4	5.50	0.51	-3.12
IPI00384542	Isoform 2 of Nidogen-1 precursor	R.EDLSPSITQR.A	2	2.89	0.22	-3.61
IPI00384542	Isoform 2 of Nidogen-1 precursor	R.EYTVTEPER.D	2	2.29	0.27	-2.63
IPI00384542	Isoform 2 of Nidogen-1 precursor	R.EYTVTEPERDGASPSR.I	3	2.35	0.12	-1.10
IPI00384542	Isoform 2 of Nidogen-1 precursor	R.FYDRSDIDAVYVTTNGIIATSEPPAK.E	3	3.17	0.33	0.39
IPI00384542	Isoform 2 of Nidogen-1 precursor	R.GFPEISFQPSSAVVVTWESVAPYQGPSRDPDQK.G	3	3.34	0.24	-5.37
IPI00384542	Isoform 2 of Nidogen-1 precursor	R.GFPEISFQPSSAVVVTWESVAPYQGPSRDPDQK.G	4	3.34	0.11	-6.30
IPI00384542	Isoform 2 of Nidogen-1 precursor	R.GGADTYSVPSVLSPR.R	2	2.56	0.13	-1.97
IPI00384542	Isoform 2 of Nidogen-1 precursor	R.GIVTDSVR.G	2	2.14	0.17	-2.13
IPI00384542	Isoform 2 of Nidogen-1 precursor	R.GNLYWTDWNR.D	2	2.70	0.29	-1.23
IPI00384542	Isoform 2 of Nidogen-1 precursor	R.IFVGSSQVPIVFENTDLHSYVVM*NHGR.S	4	2.55	0.11	-3.84
IPI00384542	Isoform 2 of Nidogen-1 precursor	R.KALEGLQYPFAVTSYGK.N	2	5.18	0.53	-4.19
IPI00384542	Isoform 2 of Nidogen-1 precursor	R.QAEVTFVGHPGNLVIK.Q	3	2.13	0.14	-2.55
IPI00384542	Isoform 2 of Nidogen-1 precursor	R.QDLGSPEGIAVDHLGR.N	2	2.67	0.30	-2.05
IPI00384542	Isoform 2 of Nidogen-1 precursor	R.QDLGSPEGIAVDHLGR.N	3	2.23	0.16	-0.35
IPI00384542	Isoform 2 of Nidogen-1 precursor	R.QELFPFGPGQGDLELEDGDDFVSPALELSGALR.F	2	2.13	0.37	-2.51
IPI00384542	Isoform 2 of Nidogen-1 precursor	R.QELFPFGPGQGDLELEDGDDFVSPALELSGALR.F	3	5.66	0.49	-3.14
IPI00384542	Isoform 2 of Nidogen-1 precursor	R.QELFPFGPGQGDLELEDGDDFVSPALELSGALR.F	4	4.02	0.39	-2.68
IPI00384542	Isoform 2 of Nidogen-1 precursor	R.RGGADTYSVPSVLSPR.R	2	3.25	0.32	-3.12
IPI00384542	Isoform 2 of Nidogen-1 precursor	R.RVLFETDLVNPR.G	2	3.12	0.35	-4.36
IPI00384542	Isoform 2 of Nidogen-1 precursor	R.RVLFETDLVNPR.G	3	2.48	0.21	-2.61
IPI00384542	Isoform 2 of Nidogen-1 precursor	R.SDIDAVYVTTNGIIATSEPPAK.E	3	3.28	0.06	0.46
IPI00384542	Isoform 2 of Nidogen-1 precursor	R.SFQLAVETFHQQ.H	2	3.81	0.33	-5.92
IPI00384542	Isoform 2 of Nidogen-1 precursor	R.TQFTCECSIGFR.G	2	3.78	0.43	-3.06

IPI00384542	Isoform 2 of Nidogen-1 precursor	R.VLFETDLVNPR.G	2	2.97	0.39	-3.10
IPI00384542	Isoform 2 of Nidogen-1 precursor	R.VPQIPFGSSVHIEPYTELYHYSTSVITSSSTR.E	4	2.86	0.17	-2.70
IPI00384697	Isoform 2 of Serum albumin precursor	A.DLPSLAADFVESKDVCK.N	1	3.67	0.21	
IPI00384697	Isoform 2 of Serum albumin precursor	A.DLPSLAADFVESKDVCK.N	2	5.26	0.48	
IPI00384697	Isoform 2 of Serum albumin precursor	A.KVFDEFKPLVEEPQNLIK.Q	3	6.62	0.37	
IPI00384697	Isoform 2 of Serum albumin precursor	C.FSALEVDETYVPK.E	2	4.90	0.32	
IPI00384697	Isoform 2 of Serum albumin precursor	C.FSALEVDETYVPKEFNAETFTFHADICTLSEK.E	3	6.45	0.49	
IPI00384697	Isoform 2 of Serum albumin precursor	C.FSALEVDETYVPKEFNAETFTFHADICTLSEKER.Q	3	6.06	0.42	
IPI00384697	Isoform 2 of Serum albumin precursor	C.IAEVENDEM*PADLPSLAADFVESK.D	2	5.54	0.49	
IPI00384697	Isoform 2 of Serum albumin precursor	D.LPSLAADFVESKDVCK.N	1	4.13	0.38	
IPI00384697	Isoform 2 of Serum albumin precursor	D.VFLGM*FLYEYAR.R	2	3.66	0.43	-4.13
IPI00384697	Isoform 2 of Serum albumin precursor	E.FAEVSKLVTDLTK.V	2	4.99	0.38	
IPI00384697	Isoform 2 of Serum albumin precursor	E.MPADLPSLAADFVESK.D	1	4.14	0.34	
IPI00384697	Isoform 2 of Serum albumin precursor	E.PQNLIKQNCELFEQLGEYK.F	2	5.83	0.50	
IPI00384697	Isoform 2 of Serum albumin precursor	E.PQNLIKQNCELFEQLGEYKFQNALLVR.Y	3	7.04	0.46	
IPI00384697	Isoform 2 of Serum albumin precursor	E.TFTFHADICTLSEKER.Q	2	4.95	0.39	
IPI00384697	Isoform 2 of Serum albumin precursor	E.VENDEM*PADLPSLAADFVESK.D	2	5.73	0.50	
IPI00384697	Isoform 2 of Serum albumin precursor	H.CIAEVENDEM*PADLPSLAADFVESKDVCK.N	3	5.79	0.36	
IPI00384697	Isoform 2 of Serum albumin precursor	K.ADDKETCFAEEGKK.L	3	4.64	0.28	
IPI00384697	Isoform 2 of Serum albumin precursor	K.ADDKETCFAEEGKKLVAASQAALGL.-	3	4.07	0.24	
IPI00384697	Isoform 2 of Serum albumin precursor	K.AEFAEVSK.L	1	2.70	0.11	
IPI00384697	Isoform 2 of Serum albumin precursor	K.AEFAEVSK.L	2	2.32	0.06	-2.27
IPI00384697	Isoform 2 of Serum albumin precursor	K.AEFAEVSKLVTDLTK.V	1	3.71	0.40	
IPI00384697	Isoform 2 of Serum albumin precursor	K.AEFAEVSKLVTDLTK.V	2	4.59	0.44	-3.50
IPI00384697	Isoform 2 of Serum albumin precursor	K.AEFAEVSKLVTDLTK.V	3	3.57	0.24	-4.12
IPI00384697	Isoform 2 of Serum albumin precursor	K.ATKEQLKAVM*DDFAAFVEK.C	2	6.27	0.48	
IPI00384697	Isoform 2 of Serum albumin precursor	K.ATKEQLKAVM*DDFAAFVEK.C	3	5.05	0.42	
IPI00384697	Isoform 2 of Serum albumin precursor	K.ATKEQLKAVMDDFAAFVEK.C	2	6.09	0.52	
IPI00384697	Isoform 2 of Serum albumin precursor	K.ATKEQLKAVMDDFAAFVEK.C	3	4.63	0.36	
IPI00384697	Isoform 2 of Serum albumin precursor	K.AVM*DDFAAFVEK.C	1	3.46	0.41	
IPI00384697	Isoform 2 of Serum albumin precursor	K.AVM*DDFAAFVEK.C	2	2.72	0.20	-4.62
IPI00384697	Isoform 2 of Serum albumin precursor	K.AVM*DDFAAFVEK.C	3	4.27	0.17	
IPI00384697	Isoform 2 of Serum albumin precursor	K.AVMDDFAAFVEK.C	1	3.38	0.39	
IPI00384697	Isoform 2 of Serum albumin precursor	K.AVMDDFAAFVEK.C	2	4.72	0.45	
IPI00384697	Isoform 2 of Serum albumin precursor	K.AVMDDFAAFVEK.C	3	4.80	0.31	
IPI00384697	Isoform 2 of Serum albumin precursor	K.AVMDDFAAFVEKCCK.A	2	3.36	0.24	
IPI00384697	Isoform 2 of Serum albumin precursor	K.CCAAADPHECYAK.V	1	3.25	0.49	
IPI00384697	Isoform 2 of Serum albumin precursor	K.CCAAADPHECYAK.V	2	5.15	0.46	
IPI00384697	Isoform 2 of Serum albumin precursor	K.CCAAADPHECYAKVFDEFKPLVEEPQNLIK.Q	3	5.66	0.38	
IPI00384697	Isoform 2 of Serum albumin precursor	K.CCTESLVNR.R	1	3.26	0.42	
IPI00384697	Isoform 2 of Serum albumin precursor	K.CCTESLVNR.R	2	3.78	0.39	
IPI00384697	Isoform 2 of Serum albumin precursor	K.CCTESLVNRRPCFSALEVDETYVPK.E	3	4.01	0.32	

IPI00384697	Isoform 2 of Serum albumin precursor	K.DLGEENFK.A	1	2.48	0.19	
IPI00384697	Isoform 2 of Serum albumin precursor	K.DLGEENFK.A	2	2.99	0.11	
IPI00384697	Isoform 2 of Serum albumin precursor	K.DVCKNYAEAK.D	1	2.80	0.28	
IPI00384697	Isoform 2 of Serum albumin precursor	K.DVCKNYAEAK.D	2	3.15	0.28	
IPI00384697	Isoform 2 of Serum albumin precursor	K.DVCKNYAEAK.D	3	2.72	0.23	
IPI00384697	Isoform 2 of Serum albumin precursor	K.DVCKNYAEAKDVFLGM*FLYEYAR.R	2	5.81	0.52	
IPI00384697	Isoform 2 of Serum albumin precursor	K.DVCKNYAEAKDVFLGM*FLYEYAR.R	3	6.16	0.51	
IPI00384697	Isoform 2 of Serum albumin precursor	K.DVCKNYAEAKDVFLGMFLYEYAR.R	2	5.40	0.53	
IPI00384697	Isoform 2 of Serum albumin precursor	K.DVCKNYAEAKDVFLGMFLYEYAR.R	3	5.99	0.53	
IPI00384697	Isoform 2 of Serum albumin precursor	K.DVFLGM*FLYEYAR.R	2	3.99	0.47	-5.06
IPI00384697	Isoform 2 of Serum albumin precursor	K.DVFLGM*FLYEYAR.R	3	5.36	0.36	
IPI00384697	Isoform 2 of Serum albumin precursor	K.DVFLGMFLYEYAR.R	1	4.45	0.40	
IPI00384697	Isoform 2 of Serum albumin precursor	K.DVFLGMFLYEYAR.R	2	3.34	0.41	-5.62
IPI00384697	Isoform 2 of Serum albumin precursor	K.DVFLGMFLYEYAR.R	3	4.99	0.43	
IPI00384697	Isoform 2 of Serum albumin precursor	K.ECCEKPLLEK.S	1	2.75	0.15	
IPI00384697	Isoform 2 of Serum albumin precursor	K.ECCEKPLLEK.S	2	2.89	0.28	
IPI00384697	Isoform 2 of Serum albumin precursor	K.EFNAETFTFHADICTLSEK.E	2	6.58	0.56	
IPI00384697	Isoform 2 of Serum albumin precursor	K.EFNAETFTFHADICTLSEK.E	3	3.55	0.39	
IPI00384697	Isoform 2 of Serum albumin precursor	K.EFNAETFTFHADICTLSEKER.Q	2	5.54	0.57	
IPI00384697	Isoform 2 of Serum albumin precursor	K.EFNAETFTFHADICTLSEKER.Q	3	5.35	0.42	
IPI00384697	Isoform 2 of Serum albumin precursor	K.EQLKAVM*DDFAAFVEK.C	1	3.76	0.46	
IPI00384697	Isoform 2 of Serum albumin precursor	K.EQLKAVM*DDFAAFVEK.C	2	5.62	0.47	
IPI00384697	Isoform 2 of Serum albumin precursor	K.EQLKAVM*DDFAAFVEK.C	3	3.90	0.30	
IPI00384697	Isoform 2 of Serum albumin precursor	K.EQLKAVMDDFAAFVEK.C	1	5.01	0.49	
IPI00384697	Isoform 2 of Serum albumin precursor	K.EQLKAVMDDFAAFVEK.C	2	5.14	0.48	
IPI00384697	Isoform 2 of Serum albumin precursor	K.EQLKAVMDDFAAFVEK.C	3	4.60	0.44	
IPI00384697	Isoform 2 of Serum albumin precursor	K.FQNALLVR.Y	1	1.76	0.09	-1.15
IPI00384697	Isoform 2 of Serum albumin precursor	K.FQNALLVR.Y	2	3.03	0.15	-2.03
IPI00384697	Isoform 2 of Serum albumin precursor	K.HKPKATKEQLK.A	3	2.92	0.14	
IPI00384697	Isoform 2 of Serum albumin precursor	K.KLVAASQAALGL.-	1	3.00	0.31	
IPI00384697	Isoform 2 of Serum albumin precursor	K.KLVAASQAALGL.-	2	3.69	0.36	
IPI00384697	Isoform 2 of Serum albumin precursor	K.KQTALVELVK.H	1	2.79	0.27	
IPI00384697	Isoform 2 of Serum albumin precursor	K.KQTALVELVK.H	2	3.26	0.24	
IPI00384697	Isoform 2 of Serum albumin precursor	K.KVPQVSTPTLVEVSR.N	1	4.15	0.48	
IPI00384697	Isoform 2 of Serum albumin precursor	K.KVPQVSTPTLVEVSR.N	2	3.59	0.45	-4.27
IPI00384697	Isoform 2 of Serum albumin precursor	K.KVPQVSTPTLVEVSR.N	3	4.86	0.49	-3.58
IPI00384697	Isoform 2 of Serum albumin precursor	K.KVPQVSTPTLVEVSRNLGK.V	2	3.37	0.40	
IPI00384697	Isoform 2 of Serum albumin precursor	K.KVPQVSTPTLVEVSRNLGK.V	3	3.70	0.34	
IPI00384697	Isoform 2 of Serum albumin precursor	K.LKECCEKPLLEK.S	1	3.16	0.24	
IPI00384697	Isoform 2 of Serum albumin precursor	K.LKECCEKPLLEK.S	2	4.46	0.29	
IPI00384697	Isoform 2 of Serum albumin precursor	K.LKECCEKPLLEK.S	3	4.40	0.22	
IPI00384697	Isoform 2 of Serum albumin precursor	K.LKECCEKPLLEKSHCIAEVENDEM*PADLPSLAADFVESK.D	3	3.46	0.15	

IPI00384697	Isoform 2 of Serum albumin precursor	K.LKECCEKPLLEKSHCIAEVENDEMPADLPSLAADFVESK.D	3	3.17	0.11	
IPI00384697	Isoform 2 of Serum albumin precursor	K.LVAASQAALGL.-	2	3.53	0.32	-2.35
IPI00384697	Isoform 2 of Serum albumin precursor	K.LVTDLTK.V	1	2.20	0.11	
IPI00384697	Isoform 2 of Serum albumin precursor	K.LVTDLTKVHTECCHGDLLECADDR.A	2	4.79	0.46	
IPI00384697	Isoform 2 of Serum albumin precursor	K.LVTDLTKVHTECCHGDLLECADDR.A	3	7.23	0.53	
IPI00384697	Isoform 2 of Serum albumin precursor	K.LVTDLTKVHTECCHGDLLECADDRADLAK.Y	2	4.02	0.42	
IPI00384697	Isoform 2 of Serum albumin precursor	K.LVTDLTKVHTECCHGDLLECADDRADLAK.Y	3	5.08	0.30	
IPI00384697	Isoform 2 of Serum albumin precursor	K.NYAEAKDVFLGM*FLYEYAR.R	2	5.71	0.54	
IPI00384697	Isoform 2 of Serum albumin precursor	K.NYAEAKDVFLGM*FLYEYAR.R	3	3.08	0.08	-4.06
IPI00384697	Isoform 2 of Serum albumin precursor	K.NYAEAKDVFLGMFLYEYAR.R	2	5.95	0.48	
IPI00384697	Isoform 2 of Serum albumin precursor	K.NYAEAKDVFLGMFLYEYAR.R	3	4.91	0.36	
IPI00384697	Isoform 2 of Serum albumin precursor	K.QNCELFEQLGEYK.F	1	3.85	0.33	
IPI00384697	Isoform 2 of Serum albumin precursor	K.QNCELFEQLGEYK.F	2	3.52	0.44	-2.05
IPI00384697	Isoform 2 of Serum albumin precursor	K.QNCELFEQLGEYK.F	3	4.54	0.25	
IPI00384697	Isoform 2 of Serum albumin precursor	K.QNCELFEQLGEYKFQNALLVR.Y	2	5.42	0.38	
IPI00384697	Isoform 2 of Serum albumin precursor	K.QNCELFEQLGEYKFQNALLVR.Y	3	6.95	0.48	
IPI00384697	Isoform 2 of Serum albumin precursor	K.QTALVELVK.H	1	1.96	0.13	
IPI00384697	Isoform 2 of Serum albumin precursor	K.QTALVELVK.H	2	1.80	0.10	-1.82
IPI00384697	Isoform 2 of Serum albumin precursor	K.RM*PCAEDYLSVVLNQLCVLHEK.T	2	4.36	0.39	
IPI00384697	Isoform 2 of Serum albumin precursor	K.RM*PCAEDYLSVVLNQLCVLHEK.T	3	7.25	0.47	
IPI00384697	Isoform 2 of Serum albumin precursor	K.RM*PCAEDYLSVVLNQLCVLHEKTPVSDR.V	3	4.69	0.39	
IPI00384697	Isoform 2 of Serum albumin precursor	K.RM*PCAEDYLSVVLNQLCVLHEKTPVSDRVTK.C	3	4.04	0.28	
IPI00384697	Isoform 2 of Serum albumin precursor	K.RMPCAEDYLSVVLNQLCVLHEK.T	2	5.25	0.45	
IPI00384697	Isoform 2 of Serum albumin precursor	K.RMPCAEDYLSVVLNQLCVLHEK.T	3	6.52	0.47	
IPI00384697	Isoform 2 of Serum albumin precursor	K.RMPCAEDYLSVVLNQLCVLHEKTPVSDR.V	3	5.15	0.32	
IPI00384697	Isoform 2 of Serum albumin precursor	K.SHCIAEVENDEM*PAD.L	2	5.40	0.43	
IPI00384697	Isoform 2 of Serum albumin precursor	K.SHCIAEVENDEM*PADLPSLAADFVESK.D	2	6.10	0.55	
IPI00384697	Isoform 2 of Serum albumin precursor	K.SHCIAEVENDEM*PADLPSLAADFVESK.D	3	6.12	0.57	-4.57
IPI00384697	Isoform 2 of Serum albumin precursor	K.SHCIAEVENDEM*PADLPSLAADFVESKD.V	3	5.94	0.29	
IPI00384697	Isoform 2 of Serum albumin precursor	K.SHCIAEVENDEM*PADLPSLAADFVESKDVCK.N	2	5.61	0.60	
IPI00384697	Isoform 2 of Serum albumin precursor	K.SHCIAEVENDEM*PADLPSLAADFVESKDVCK.N	3	7.63	0.56	
IPI00384697	Isoform 2 of Serum albumin precursor	K.SHCIAEVENDEM*PADLPSLAADFVESKDVCKNYAEAK.D	3	6.65	0.57	
IPI00384697	Isoform 2 of Serum albumin precursor	K.SHCIAEVENDEMPADLPSLAADFVESK.D	2	6.14	0.54	
IPI00384697	Isoform 2 of Serum albumin precursor	K.SHCIAEVENDEMPADLPSLAADFVESK.D	3	6.62	0.53	
IPI00384697	Isoform 2 of Serum albumin precursor	K.SHCIAEVENDEMPADLPSLAADFVESKDVCK.N	2	5.73	0.58	
IPI00384697	Isoform 2 of Serum albumin precursor	K.SHCIAEVENDEMPADLPSLAADFVESKDVCK.N	3	7.27	0.55	
IPI00384697	Isoform 2 of Serum albumin precursor	K.SHCIAEVENDEMPADLPSLAADFVESKDVCKNYAEAK.D	3	7.14	0.51	
IPI00384697	Isoform 2 of Serum albumin precursor	K.TPVSDRVTK.C	2	2.16	0.06	-2.14
IPI00384697	Isoform 2 of Serum albumin precursor	K.TPVSDRVTKCCTESLVNR.R	3	4.31	0.40	
IPI00384697	Isoform 2 of Serum albumin precursor	K.TYETTLEK.C	1	1.93	0.10	
IPI00384697	Isoform 2 of Serum albumin precursor	K.TYETTLEK.C	2	2.62	0.23	
IPI00384697	Isoform 2 of Serum albumin precursor	K.TYETTLEKCCAAADPHECYAK.V	2	5.25	0.50	

IPI00384697	Isoform 2 of Serum albumin precursor	K.TYETTLEKCCAAADPHECYAK.V	3	5.13	0.32	
IPI00384697	Isoform 2 of Serum albumin precursor	K.VFDEFKPLVEEPQNLIK.Q	2	4.55	0.39	-5.33
IPI00384697	Isoform 2 of Serum albumin precursor	K.VFDEFKPLVEEPQNLIK.Q	3	4.84	0.32	-4.89
IPI00384697	Isoform 2 of Serum albumin precursor	K.VFDEFKPLVEEPQNLIKQNCELFEQLGEYK.F	3	7.59	0.48	
IPI00384697	Isoform 2 of Serum albumin precursor	K.VFDEFKPLVEEPQNLIKQNCELFEQLGEYKFQNALLVR.Y	3	6.11	0.54	
IPI00384697	Isoform 2 of Serum albumin precursor	K.VHTECCHGDLLECADDR.A	2	6.67	0.60	
IPI00384697	Isoform 2 of Serum albumin precursor	K.VHTECCHGDLLECADDR.A	3	5.51	0.45	
IPI00384697	Isoform 2 of Serum albumin precursor	K.VHTECCHGDLLECADDRADLAK.Y	2	5.13	0.43	
IPI00384697	Isoform 2 of Serum albumin precursor	K.VHTECCHGDLLECADDRADLAK.Y	3	6.44	0.50	
IPI00384697	Isoform 2 of Serum albumin precursor	K.VHTECCHGDLLECADDRADLAKYICENQDSISSK.L	3	4.92	0.43	
IPI00384697	Isoform 2 of Serum albumin precursor	K.VPQVSTPTLVEVSR.N	1	3.38	0.40	
IPI00384697	Isoform 2 of Serum albumin precursor	K.VPQVSTPTLVEVSR.N	2	3.92	0.45	
IPI00384697	Isoform 2 of Serum albumin precursor	K.VPQVSTPTLVEVSR.N	3	3.70	0.29	
IPI00384697	Isoform 2 of Serum albumin precursor	K.VPQVSTPTLVEVSRNLGK.V	3	2.66	0.20	
IPI00384697	Isoform 2 of Serum albumin precursor	K.YICENQDSISSK.L	1	3.56	0.39	
IPI00384697	Isoform 2 of Serum albumin precursor	K.YICENQDSISSK.L	2	1.96	0.16	-2.00
IPI00384697	Isoform 2 of Serum albumin precursor	K.YICENQDSISSK.L	3	2.98	0.10	
IPI00384697	Isoform 2 of Serum albumin precursor	K.YICENQDSISSKLK.E	2	4.73	0.43	
IPI00384697	Isoform 2 of Serum albumin precursor	K.YICENQDSISSKLKECCEKPLLEK.S	3	5.80	0.27	
IPI00384697	Isoform 2 of Serum albumin precursor	L.FEQLGEYKFQNALLVR.Y	2	5.33	0.32	
IPI00384697	Isoform 2 of Serum albumin precursor	L.IKQNCELFEQLGEYK.F	2	4.99	0.28	
IPI00384697	Isoform 2 of Serum albumin precursor	L.PSLAADFVESKDVCK.N	1	4.05	0.37	
IPI00384697	Isoform 2 of Serum albumin precursor	L.PSLAADFVESKDVCK.N	2	5.36	0.39	
IPI00384697	Isoform 2 of Serum albumin precursor	M.PADLPSLAADFVESK.D	1	4.03	0.52	
IPI00384697	Isoform 2 of Serum albumin precursor	M.PADLPSLAADFVESK.D	2	5.47	0.47	
IPI00384697	Isoform 2 of Serum albumin precursor	M.PADLPSLAADFVESKDVCK.N	2	6.02	0.51	
IPI00384697	Isoform 2 of Serum albumin precursor	M.PCAEDYLSVVLNQLCVLHEK.T	3	6.35	0.43	
IPI00384697	Isoform 2 of Serum albumin precursor	P.CAEDYLSVVLNQLCVLHEK.T	2	5.18	0.36	
IPI00384697	Isoform 2 of Serum albumin precursor	P.CAEDYLSVVLNQLCVLHEK.T	3	6.03	0.45	
IPI00384697	Isoform 2 of Serum albumin precursor	P.CFSALEVDETYVPK.E	2	5.56	0.47	
IPI00384697	Isoform 2 of Serum albumin precursor	R.ADLAKYICENQDSISSK.L	2	5.03	0.43	
IPI00384697	Isoform 2 of Serum albumin precursor	R.DAHKSEVAHR.F	2	2.69	0.26	
IPI00384697	Isoform 2 of Serum albumin precursor	R.FKDLGEENFK.A	1	3.27	0.31	
IPI00384697	Isoform 2 of Serum albumin precursor	R.FKDLGEENFK.A	2	3.67	0.24	
IPI00384697	Isoform 2 of Serum albumin precursor	R.FKDLGEENFK.A	3	2.78	0.18	-3.67
IPI00384697	Isoform 2 of Serum albumin precursor	R.FPKAEFAEVSK.L	1	3.28	0.29	
IPI00384697	Isoform 2 of Serum albumin precursor	R.FPKAEFAEVSK.L	2	3.86	0.31	
IPI00384697	Isoform 2 of Serum albumin precursor	R.FPKAEFAEVSK.L	3	4.48	0.30	
IPI00384697	Isoform 2 of Serum albumin precursor	R.FPKAEFAEVSKLVTDLTK.V	2	6.07	0.48	
IPI00384697	Isoform 2 of Serum albumin precursor	R.FPKAEFAEVSKLVTDLTK.V	3	5.52	0.39	
IPI00384697	Isoform 2 of Serum albumin precursor	R.HPDYSVVLLLR.L	1	3.42	0.37	
IPI00384697	Isoform 2 of Serum albumin precursor	R.HPDYSVVLLLR.L	2	2.94	0.36	

IPI00384697	Isoform 2 of Serum albumin precursor	R.HPDYSVVLLLR.L	3	4.45	0.12	
IPI00384697	Isoform 2 of Serum albumin precursor	R.LAKTYETTLEK.C	1	2.80	0.23	
IPI00384697	Isoform 2 of Serum albumin precursor	R.LAKTYETTLEK.C	2	3.39	0.31	
IPI00384697	Isoform 2 of Serum albumin precursor	R.LAKTYETTLEK.C	3	2.39	0.11	1.05
IPI00384697	Isoform 2 of Serum albumin precursor	R.LAKTYETTLEKCCAAADPHECYAK.V	3	5.45	0.35	
IPI00384697	Isoform 2 of Serum albumin precursor	R.LSQRFPKAEFAEVSK.L	2	3.95	0.32	
IPI00384697	Isoform 2 of Serum albumin precursor	R.LSQRFPKAEFAEVSK.L	3	5.85	0.39	
IPI00384697	Isoform 2 of Serum albumin precursor	R.LSQRFPKAEFAEVSKLVTDLTK.V	3	7.14	0.47	
IPI00384697	Isoform 2 of Serum albumin precursor	R.M*PCAEDYLSVVLNQLCVLHEK.T	2	5.36	0.46	
IPI00384697	Isoform 2 of Serum albumin precursor	R.M*PCAEDYLSVVLNQLCVLHEK.T	3	4.25	0.37	-4.98
IPI00384697	Isoform 2 of Serum albumin precursor	R.M*PCAEDYLSVVLNQLCVLHEKTPVSDR.V	3	5.69	0.42	
IPI00384697	Isoform 2 of Serum albumin precursor	R.M*PCAEDYLSVVLNQLCVLHEKTPVSDRVTK.C	3	5.84	0.48	
IPI00384697	Isoform 2 of Serum albumin precursor	R.MPCAEDYLSVVLNQLCVLHEK.T	2	5.15	0.43	
IPI00384697	Isoform 2 of Serum albumin precursor	R.MPCAEDYLSVVLNQLCVLHEK.T	3	4.21	0.43	-4.61
IPI00384697	Isoform 2 of Serum albumin precursor	R.MPCAEDYLSVVLNQLCVLHEKTPVSDR.V	3	6.18	0.50	
IPI00384697	Isoform 2 of Serum albumin precursor	R.MPCAEDYLSVVLNQLCVLHEKTPVSDRVTK.C	3	5.28	0.39	
IPI00384697	Isoform 2 of Serum albumin precursor	R.PCFSALEVDETYVPK.E	2	5.95	0.49	
IPI00384697	Isoform 2 of Serum albumin precursor	R.QIKKQTALVELVK.H	3	2.90	0.25	
IPI00384697	Isoform 2 of Serum albumin precursor	R.RHPDYSVVLLLR.L	2	3.78	0.40	
IPI00384697	Isoform 2 of Serum albumin precursor	R.RHPDYSVVLLLR.L	3	5.46	0.33	
IPI00384697	Isoform 2 of Serum albumin precursor	R.RPCFSALEVDETYVPK.E	1	3.97	0.49	
IPI00384697	Isoform 2 of Serum albumin precursor	R.RPCFSALEVDETYVPK.E	2	4.52	0.40	
IPI00384697	Isoform 2 of Serum albumin precursor	R.RPCFSALEVDETYVPK.E	3	3.79	0.26	
IPI00384697	Isoform 2 of Serum albumin precursor	R.RPCFSALEVDETYVPKEFNAETFTFHADICTLSEK.E	3	6.83	0.48	
IPI00384697	Isoform 2 of Serum albumin precursor	R.RPCFSALEVDETYVPKEFNAETFTFHADICTLSEKER.Q	3	6.05	0.47	
IPI00384697	Isoform 2 of Serum albumin precursor	R.VTKCCTESLVNR.R	3	3.44	0.17	
IPI00384697	Isoform 2 of Serum albumin precursor	R.YTKKVPQVSTPTLVEVSR.N	2	4.95	0.33	
IPI00384697	Isoform 2 of Serum albumin precursor	R.YTKKVPQVSTPTLVEVSR.N	3	4.84	0.39	
IPI00384697	Isoform 2 of Serum albumin precursor	V.FDEFKPLVEEPQNLIKQNCELFEQLGEYK.F	3	5.61	0.34	
IPI00384697	Isoform 2 of Serum albumin precursor	V.PKEFNAETFTFHADICTLSEK.E	2	5.73	0.47	
IPI00384722	Isoform 2 of UPF0510 protein C19orf63 precursor	R.LRDVAALNGLYR.V	3	2.53	0.05	-3.07
IPI00384861	Isoform 1 of ARF GTPase-activating protein GIT1	R.SQSDLDDQHDYDSVASDEDTDQEPLRSTGATR.S	3	2.66	0.26	-2.32
IPI00384952	Putative uncharacterized protein DKFZp686K04218 (Fragment)	K.HYTNPSQDVTVPCPVPPPPPCCHPR.L	3	5.66	0.35	
IPI00384952	Putative uncharacterized protein DKFZp686K04218 (Fragment)	K.SAVQGPPER.D	2	2.30	0.12	0.79
IPI00384952	Putative uncharacterized protein DKFZp686K04218 (Fragment)	K.SAVQGPPERDLCGCYSVSSVLPGCAQPWNHGETFTCTAAHPELK.T	3	3.92	0.18	
IPI00384952	Putative uncharacterized protein DKFZp686K04218 (Fragment)	K.SGNTFRPEVHLLPPPSEELALNELVTLTCLAR.G	2	4.32	0.41	
IPI00384952	Putative uncharacterized protein DKFZp686K04218 (Fragment)	K.SGNTFRPEVHLLPPPSEELALNELVTLTCLAR.G	3	6.86	0.60	

IPI00384952	Putative uncharacterized protein DKFZp686K04218 (Fragment)	K.YLTWASR.Q	1	1.98	0.18	
IPI00384952	Putative uncharacterized protein DKFZp686K04218 (Fragment)	K.YLTWASR.Q	2	1.93	0.24	
IPI00384952	Putative uncharacterized protein DKFZp686K04218 (Fragment)	Q.EPSQGTTTFAVTSILR.V	2	3.82	0.43	-5.84
IPI00384952	Putative uncharacterized protein DKFZp686K04218 (Fragment)	R.DASGATFTWTPSSGK.S	2	4.55	0.44	
IPI00384952	Putative uncharacterized protein DKFZp686K04218 (Fragment)	R.DLCGCYSVSSVLPGCAQPWNHGETFTCTAAHPELK.T	3	5.72	0.26	
IPI00384952	Putative uncharacterized protein DKFZp686K04218 (Fragment)	R.EKYLTWASR.Q	1	2.49	0.27	
IPI00384952	Putative uncharacterized protein DKFZp686K04218 (Fragment)	R.GFSPKDVLVR.W	2	2.81	0.13	
IPI00384952	Putative uncharacterized protein DKFZp686K04218 (Fragment)	R.LSISIDTSKNQFSLR.L	2	2.66	0.13	
IPI00384952	Putative uncharacterized protein DKFZp686K04218 (Fragment)	R.NFPPSQDASGDLYTTSSQLTLPATQCPDGK.S	2	3.66	0.39	
IPI00384952	Putative uncharacterized protein DKFZp686K04218 (Fragment)	R.NFPPSQDASGDLYTTSSQLTLPATQCPDGK.S	3	5.98	0.54	
IPI00384952	Putative uncharacterized protein DKFZp686K04218 (Fragment)	R.QEPSQGTTTFAVTSILR.V	2	4.27	0.52	
IPI00384952	Putative uncharacterized protein DKFZp686K04218 (Fragment)	R.QEPSQGTTTFAVTSILR.V	3	4.05	0.27	
IPI00384952	Putative uncharacterized protein DKFZp686K04218 (Fragment)	R.VAAEDWK.K	2	2.23	0.16	
IPI00384952	Putative uncharacterized protein DKFZp686K04218 (Fragment)	R.WLQGSQELPR.E	1	3.00	0.19	
IPI00384952	Putative uncharacterized protein DKFZp686K04218 (Fragment)	R.WLQGSQELPR.E	2	3.80	0.33	
IPI00384952	Putative uncharacterized protein DKFZp686K04218 (Fragment)	R.WLQGSQELPREK.Y	2	2.71	0.15	
IPI00385003	Putative transposase	K.AIAAIDSDSSDGSGQSKLKTFWKGFTILDAIK.N	3	2.43	0.10	0.36
IPI00385007	Putative uncharacterized protein DKFZp686A01208	K.DIPNENEAQFQIR.D	2	3.75	0.30	-4.40
IPI00385007	Putative uncharacterized protein DKFZp686A01208	K.LQSSNIFTVAK.R	2	2.10	0.12	-3.05
IPI00385007	Putative uncharacterized protein DKFZp686A01208	K.M*EPLNNLQVAVK.N	2	3.13	0.30	-3.02
IPI00385007	Putative uncharacterized protein DKFZp686A01208	K.RNVEGQDM*LYQSLK.L	3	2.64	0.29	-1.14
IPI00385007	Putative uncharacterized protein DKFZp686A01208	R.NVEGQDM*LYQSLK.L	2	3.99	0.46	-3.71

IPI00385042	Nucleolar GTP-binding protein 1	K.KAKTMMKNAQK.K	3	3.61	0.06	
IPI00385143	Microfibrillar protein 2 (Fragment)	K.LLIYWASTR.E	2	2.79	0.24	
IPI00385252	Ig kappa chain V-III region GOL	G.EIVLTQSPGTLSLSPGER.A	2	6.33	0.22	
IPI00385252	Ig kappa chain V-III region GOL	R.ATGIPDRFSGSGSGTDFTLTISR.L	2	4.84	0.38	
IPI00385252	Ig kappa chain V-III region GOL	R.ATGIPDRFSGSGSGTDFTLTISR.L	3	3.97	0.23	
IPI00385252	Ig kappa chain V-III region GOL	R.FSGSGSGTDFTLTISR.L	1	2.55	0.22	
IPI00385252	Ig kappa chain V-III region GOL	R.FSGSGSGTDFTLTISR.L	2	4.49	0.53	
IPI00385253	Ig kappa chain V-III region CLL precursor	-.EIVM*TQSPATLSVSPGER.A	2	4.01	0.28	
IPI00385253	Ig kappa chain V-III region CLL precursor	-.EIVM*TQSPATLSVSPGER.A	3	3.27	0.20	
IPI00385253	Ig kappa chain V-III region CLL precursor	-.EIVMTQSPATLSVSPGER.A	2	4.41	0.23	
IPI00385253	Ig kappa chain V-III region CLL precursor	-.EIVMTQSPATLSVSPGER.A	3	4.58	0.17	
IPI00385253	Ig kappa chain V-III region CLL precursor	R.LLIYGASTR.A	2	3.10	0.20	
IPI00385264	Ig mu heavy chain disease protein	H.SILTVSEEEWNTGETYTCVVAHEALPNR.V	3	4.14	0.42	-3.93
IPI00385264	Ig mu heavy chain disease protein	K.GVALHRPDVYLLPPAR.E	2	4.14	0.31	
IPI00385264	Ig mu heavy chain disease protein	K.GVALHRPDVYLLPPAR.E	3	4.42	0.26	
IPI00385264	Ig mu heavy chain disease protein	K.LICQATGFSPR.Q	2	3.30	0.34	
IPI00385264	Ig mu heavy chain disease protein	K.QVGSGVTTDEVEAEAK.E	2	4.83	0.45	
IPI00385264	Ig mu heavy chain disease protein	K.SKLICQATGFSPR.Q	2	3.95	0.38	
IPI00385264	Ig mu heavy chain disease protein	K.SKLICQATGFSPR.Q	3	3.47	0.30	
IPI00385264	Ig mu heavy chain disease protein	K.VSVFVPPRDGFFGNPR.K	3	2.70	0.22	
IPI00385264	Ig mu heavy chain disease protein	K.YVTSAPM*PEPQAPGR.Y	2	3.06	0.40	-4.52
IPI00385264	Ig mu heavy chain disease protein	K.YVTSAPMPEPQAPGR.Y	2	3.15	0.35	
IPI00385264	Ig mu heavy chain disease protein	R.DGFFGNPR.K	2	2.88	0.34	-3.78
IPI00385264	Ig mu heavy chain disease protein	R.FTCTVTHTDLPSPLK.Q	2	4.47	0.36	
IPI00385264	Ig mu heavy chain disease protein	R.FTCTVTHTDLPSPLK.Q	3	3.66	0.36	
IPI00385264	Ig mu heavy chain disease protein	R.GQPLSPEKYVTSAPM*PEPQAPGR.Y	2	3.82	0.40	
IPI00385264	Ig mu heavy chain disease protein	R.TVDKSTGKPTLYNVSLVM*SDTAGTC.Y	3	5.71	0.42	
IPI00385264	Ig mu heavy chain disease protein	R.TVDKSTGKPTLYNVSLVMSDTAGTCY.-	3	4.75	0.35	
IPI00385264	Ig mu heavy chain disease protein	R.VFAIPPSFASIFLTK.S	2	2.74	0.19	-4.97
IPI00385264	Ig mu heavy chain disease protein	R.VFAIPPSFASIFLTK.S	3	4.22	0.32	
IPI00385264	Ig mu heavy chain disease protein	R.YFAHSILTVSEEEWNTGETYTCVVAHEALPNR.V	3	6.30	0.58	-4.67
IPI00385264	Ig mu heavy chain disease protein	R.YFAHSILTVSEEEWNTGETYTCVVAHEALPNR.V	4	3.78	0.24	-4.51
IPI00385480	Caskin-1	K.SIAAMLELSSIGGGGR.A	2	2.79	0.12	-4.59
IPI00385543	Isoform 3 of UPF0469 protein KIAA0907	K.GLTSNKSK.D	2	2.23	0.09	-8.09
IPI00385791	Serologically defined breast cancer antigen NY-BR-87 (Fragment)	R.ARPQDGVR.V	1	1.55	0.12	-7.22
IPI00385918	CDNA FLJ90582 fis, clone PLACE1000442, moderately similar to ZINC FINGER PROTEIN ZFP-36	R.NPM*IVSNVGKPSVVPVRFENMK.E	2	2.94	0.09	
IPI00385980	ROBO2 isoform a	K.EGSQNLLFPNQPQQPNSR.C	2	3.40	0.32	-3.59
IPI00385980	ROBO2 isoform a	K.GNPQPAVFWQK.E	2	2.89	0.31	-3.28
IPI00385980	ROBO2 isoform a	K.TM*STDEGTYM*CIAENR.V	2	4.69	0.57	-4.09

IPI00385980	ROBO2 isoform a	R.CQVQGDPQPTVR.W	2	3.45	0.21	-1.37
IPI00385980	ROBO2 isoform a	R.CSVSPTGDLTITNIQR.S	2	4.13	0.35	-3.27
IPI00385980	ROBO2 isoform a	R.DSDPAELTVFERPTFLR.R	3	2.83	0.23	-4.40
IPI00385980	ROBO2 isoform a	R.GRYDIKDDYTLR.I	2	2.90	0.24	-1.58
IPI00385980	ROBO2 isoform a	R.LRQEDFPPR.I	2	1.74	0.08	-5.77
IPI00385980	ROBO2 isoform a	R.M*LLPSGSLFFLR.I	2	3.22	0.24	-3.41
IPI00385980	ROBO2 isoform a	R.QNPTDVVVAAGEPAILECQPPR.G	3	4.28	0.36	-4.67
IPI00385980	ROBO2 isoform a	R.RPINQVVLEEEAVEFR.C	2	3.58	0.35	-3.87
IPI00385980	ROBO2 isoform a	R.SDAGYYICQALTVAGSILAK.A	2	5.02	0.50	-2.13
IPI00385980	ROBO2 isoform a	R.SDAGYYICQALTVAGSILAK.A	3	3.83	0.36	-1.85
IPI00385980	ROBO2 isoform a	R.SVIIGGLFPGIQYR.V	2	4.12	0.46	-3.67
IPI00385980	ROBO2 isoform a	R.YDIKDDYTLR.I	3	2.35	0.20	-1.85
IPI00385985	Ig lambda chain V-III region LOI	-.YVLTQPPSVSVAPGETAR.L	2	4.95	0.37	
IPI00385985	Ig lambda chain V-III region LOI	R.FSGSNSGNTATLTISR.V	1	3.22	0.40	
IPI00385985	Ig lambda chain V-III region LOI	R.FSGSNSGNTATLTISR.V	2	4.32	0.38	
IPI00386131	Ig kappa chain V-III region IARC/BL41 precursor	R.FSGSGSGTDFTLIISR.L	2	4.55	0.46	
IPI00386133	Ig kappa chain V-IV region B17 precursor	G.DIVM*TQSPDSLAVSLGER.A	2	5.76	0.42	
IPI00386133	Ig kappa chain V-IV region B17 precursor	K.LLIYWASTR.E	2	2.79	0.24	
IPI00386133	Ig kappa chain V-IV region B17 precursor	K.NYLAWYQQKPGQPPK.L	3	2.60	0.33	
IPI00386133	Ig kappa chain V-IV region B17 precursor	K.SSQSILYSSDNK.N	2	2.71	0.12	
IPI00386133	Ig kappa chain V-IV region B17 precursor	Y.GDIVM*TQSPDSLAVSLGER.A	2	5.69	0.50	
IPI00386284	olfactory receptor, family 2, subfamily AK, member 2	K.EVTGAVR.R	1	1.68	0.05	-1.15
IPI00386314	FLJ00064 protein (Fragment)	K.TGQEIPVNVR.F	2	2.28	0.18	-1.12
IPI00386393	CDNA FLJ13729 fis, clone PLACE3000121, weakly similar to VESICULAR TRAFFIC CONTROL PROTEIN SEC15	K.M*KDTSRKNNMFAQFR.K	2	2.19	0.17	-2.54
IPI00386575	Ig lambda chain V-I region EPS	R.VSISCSGSSSNIGK.N	2	3.58	0.34	
IPI00386576	Ig lambda chain V-IV region MOL	-.YELTQPPSVSVSPGQTATISCSGDK.L	3	3.45	0.14	
IPI00386630	TCN2 protein	K.DGETIELR.L	2	2.33	0.07	-3.04
IPI00386630	TCN2 protein	K.TYIDLIFPDCLAPR.V	2	4.10	0.51	-2.03
IPI00386630	TCN2 protein	R.DPNTPLLQGIADYRPK.D	3	2.72	0.15	-2.60
IPI00386630	TCN2 protein	R.LSLEHLNPSIYVGLR.L	2	3.43	0.32	-1.82
IPI00386630	TCN2 protein	R.LSLEHLNPSIYVGLR.L	3	4.65	0.26	-2.36
IPI00386630	TCN2 protein	R.LSSLQAGTKEDLYLHSLK.L	2	4.21	0.50	-3.35
IPI00386630	TCN2 protein	R.LSSLQAGTKEDLYLHSLK.L	3	2.06	0.19	-1.09
IPI00386630	TCN2 protein	R.VHDSVVDK.L	2	2.51	0.22	-2.75
IPI00386754	Isoform 2 of Cysteine-rich with EGF-like domain protein 2 precursor	K.SEYPDLFEWFCVK.T	2	3.12	0.46	-3.06
IPI00386879	CDNA FLJ14473 fis, clone MAMMA1001080, highly similar to Homo sapiens SNC73 protein (SNC73) mRNA	K.GDTFSCMVGHEALPLAFTQK.T	2	4.19	0.19	

IPI00386879	CDNA FLJ14473 fis, clone MAMMA1001080, highly similar to Homo sapiens SNC73 protein (SNC73) mRNA	K.KGDTFSCM*VGHEALPLAFTQK.T	2	4.91	0.41	
IPI00386879	CDNA FLJ14473 fis, clone MAMMA1001080, highly similar to Homo sapiens SNC73 protein (SNC73) mRNA	K.KGDTFSCM*VGHEALPLAFTQK.T	3	3.90	0.44	-2.10
IPI00386879	CDNA FLJ14473 fis, clone MAMMA1001080, highly similar to Homo sapiens SNC73 protein (SNC73) mRNA	K.NSLYLQM*NSLR.A	2	3.76	0.14	
IPI00386879	CDNA FLJ14473 fis, clone MAMMA1001080, highly similar to Homo sapiens SNC73 protein (SNC73) mRNA	K.NSLYLQMNSLR.A	1	3.55	0.11	
IPI00386879	CDNA FLJ14473 fis, clone MAMMA1001080, highly similar to Homo sapiens SNC73 protein (SNC73) mRNA	K.NSLYLQMNSLR.A	2	4.01	0.17	
IPI00386879	CDNA FLJ14473 fis, clone MAMMA1001080, highly similar to Homo sapiens SNC73 protein (SNC73) mRNA	K.SAVQGPPER.D	2	2.30	0.12	0.79
IPI00386879	CDNA FLJ14473 fis, clone MAMMA1001080, highly similar to Homo sapiens SNC73 protein (SNC73) mRNA	K.SAVQGPPERDLCGCYSVSSVLPGCAEPWNHGK.T	3	5.80	0.08	
IPI00386879	CDNA FLJ14473 fis, clone MAMMA1001080, highly similar to Homo sapiens SNC73 protein (SNC73) mRNA	K.SGNTFRPEVHLLPPPSEELALNELVTLTCLAR.G	2	4.32	0.41	
IPI00386879	CDNA FLJ14473 fis, clone MAMMA1001080, highly similar to Homo sapiens SNC73 protein (SNC73) mRNA	K.SGNTFRPEVHLLPPPSEELALNELVTLTCLAR.G	3	6.86	0.60	
IPI00386879	CDNA FLJ14473 fis, clone MAMMA1001080, highly similar to Homo sapiens SNC73 protein (SNC73) mRNA	K.TFTCTAAYPESK.T	1	2.27	0.26	
IPI00386879	CDNA FLJ14473 fis, clone MAMMA1001080, highly similar to Homo sapiens SNC73 protein (SNC73) mRNA	K.TFTCTAAYPESK.T	2	4.10	0.40	
IPI00386879	CDNA FLJ14473 fis, clone MAMMA1001080, highly similar to Homo sapiens SNC73 protein (SNC73) mRNA	K.TFTCTAAYPESKTPLTATLSK.S	2	4.13	0.39	
IPI00386879	CDNA FLJ14473 fis, clone MAMMA1001080, highly similar to Homo sapiens SNC73 protein (SNC73) mRNA	K.TFTCTAAYPESKTPLTATLSK.S	3	4.01	0.44	
IPI00386879	CDNA FLJ14473 fis, clone MAMMA1001080, highly similar to Homo sapiens SNC73 protein (SNC73) mRNA	K.TPLTATLSK.S	1	2.18	0.20	

IPI00386879	CDNA FLJ14473 fis, clone MAMMA1001080, highly similar to Homo sapiens SNC73 protein (SNC73) mRNA	K.TPLTATLSK.S	2	2.50	0.14	
IPI00386879	CDNA FLJ14473 fis, clone MAMMA1001080, highly similar to Homo sapiens SNC73 protein (SNC73) mRNA	K.VFPLSLCSTQPDGNVVIACLVQGFFPQEPLSVTWSESGQGVTAR.N	3	3.85	0.24	
IPI00386879	CDNA FLJ14473 fis, clone MAMMA1001080, highly similar to Homo sapiens SNC73 protein (SNC73) mRNA	K.YLTWASR.Q	1	1.98	0.18	
IPI00386879	CDNA FLJ14473 fis, clone MAMMA1001080, highly similar to Homo sapiens SNC73 protein (SNC73) mRNA	K.YLTWASR.Q	2	1.93	0.24	
IPI00386879	CDNA FLJ14473 fis, clone MAMMA1001080, highly similar to Homo sapiens SNC73 protein (SNC73) mRNA	Q.EPSQGTTTFAVTSILR.V	2	3.82	0.43	-5.84
IPI00386879	CDNA FLJ14473 fis, clone MAMMA1001080, highly similar to Homo sapiens SNC73 protein (SNC73) mRNA	R.DASGVTFTWTPSSGK.S	1	3.53	0.45	
IPI00386879	CDNA FLJ14473 fis, clone MAMMA1001080, highly similar to Homo sapiens SNC73 protein (SNC73) mRNA	R.DASGVTFTWTPSSGK.S	2	5.30	0.49	
IPI00386879	CDNA FLJ14473 fis, clone MAMMA1001080, highly similar to Homo sapiens SNC73 protein (SNC73) mRNA	R.DLCGCYSVSSVLPGCAEPWNHGK.T	2	4.99	0.09	
IPI00386879	CDNA FLJ14473 fis, clone MAMMA1001080, highly similar to Homo sapiens SNC73 protein (SNC73) mRNA	R.DLCGCYSVSSVLPGCAEPWNHGK.T	3	3.41	0.09	
IPI00386879	CDNA FLJ14473 fis, clone MAMMA1001080, highly similar to Homo sapiens SNC73 protein (SNC73) mRNA	R.DNAKNSLYLQM*NSLR.A	2	4.82	0.44	
IPI00386879	CDNA FLJ14473 fis, clone MAMMA1001080, highly similar to Homo sapiens SNC73 protein (SNC73) mRNA	R.DNAKNSLYLQM*NSLR.A	3	4.39	0.37	
IPI00386879	CDNA FLJ14473 fis, clone MAMMA1001080, highly similar to Homo sapiens SNC73 protein (SNC73) mRNA	R.EKYLTWASR.Q	1	2.49	0.27	
IPI00386879	CDNA FLJ14473 fis, clone MAMMA1001080, highly similar to Homo sapiens SNC73 protein (SNC73) mRNA	R.GFSPKDVLVR.W	2	2.81	0.13	
IPI00386879	CDNA FLJ14473 fis, clone MAMMA1001080, highly similar to Homo sapiens SNC73 protein (SNC73) mRNA	R.NFPPSQDASGDLYTTSSQLTLPATQCLAGK.S	2	4.34	0.48	

IPI00386879	CDNA FLJ14473 fis, clone MAMMA1001080, highly similar to Homo sapiens SNC73 protein (SNC73) mRNA	R.NFPPSQDASGDLYTTSSQLTLPATQCLAGK.S	3	5.77	0.57	
IPI00386879	CDNA FLJ14473 fis, clone MAMMA1001080, highly similar to Homo sapiens SNC73 protein (SNC73) mRNA	R.QEPSQGTTTFAVTSILR.V	2	4.27	0.52	
IPI00386879	CDNA FLJ14473 fis, clone MAMMA1001080, highly similar to Homo sapiens SNC73 protein (SNC73) mRNA	R.QEPSQGTTTFAVTSILR.V	3	4.05	0.27	
IPI00386879	CDNA FLJ14473 fis, clone MAMMA1001080, highly similar to Homo sapiens SNC73 protein (SNC73) mRNA	R.VAAEDWK.K	2	2.23	0.16	
IPI00386879	CDNA FLJ14473 fis, clone MAMMA1001080, highly similar to Homo sapiens SNC73 protein (SNC73) mRNA	R.VDDTAVYYCAR.D	2	2.27	0.15	
IPI00386879	CDNA FLJ14473 fis, clone MAMMA1001080, highly similar to Homo sapiens SNC73 protein (SNC73) mRNA	R.WLQGSQELPR.E	1	3.00	0.19	
IPI00386879	CDNA FLJ14473 fis, clone MAMMA1001080, highly similar to Homo sapiens SNC73 protein (SNC73) mRNA	R.WLQGSQELPR.E	2	3.80	0.33	
IPI00386879	CDNA FLJ14473 fis, clone MAMMA1001080, highly similar to Homo sapiens SNC73 protein (SNC73) mRNA	R.WLQGSQELPREK.Y	2	2.71	0.15	
IPI00386879	CDNA FLJ14473 fis, clone MAMMA1001080, highly similar to Homo sapiens SNC73 protein (SNC73) mRNA	W.GQGTLVTVSSASPTSPK.V	2	5.02	0.45	
IPI00387004	PNAS-146	R.M*PADHLHCQGRHSHHTQCPLLHPGCR.Q	3	2.46	0.20	-2.29
IPI00387024	Ig kappa chain V-I region CAR	-.DIQM*TQSPSTLSASVGDR.V	2	5.72	0.23	
IPI00387024	Ig kappa chain V-I region CAR	-.DIQM*TQSPSTLSASVGDR.V	3	3.93	0.31	
IPI00387024	Ig kappa chain V-I region CAR	-.DIQMTQSPSTLSASVGDR.V	2	5.56	0.15	
IPI00387024	Ig kappa chain V-I region CAR	K.VLIYKSSSLESGVPSR.F	2	3.09	0.14	
IPI00387095	Ig kappa chain V-I region Ka	-.DIQM*TQSPSTLSVSVGDR.V	2	3.25	0.08	
IPI00387095	Ig kappa chain V-I region Ka	-.DIQM*TQSPSTLSVSVGDR.V	3	3.37	0.19	
IPI00387096	Ig kappa chain V-I region Kue	K.ASTLETGVPSR.F	2	3.00	0.37	
IPI00387097	Ig kappa chain V-I region Lay	-.DIQM*TQSPSSLSVSVGDR.V	2	5.09	0.31	
IPI00387097	Ig kappa chain V-I region Lay	-.DIQM*TQSPSSLSVSVGDR.V	3	3.32	0.27	
IPI00387097	Ig kappa chain V-I region Lay	R.LLIYGASTR.A	2	3.10	0.20	
IPI00387101	Ig kappa chain V-I region Scw	K.ASTLETGVPSR.F	2	3.00	0.37	
IPI00387105	Ig kappa chain V-I region Mev	-.DVQM*TQSPSSLSASVGDR.V	2	5.41	0.28	
IPI00387105	Ig kappa chain V-I region Mev	-.DVQM*TQSPSSLSASVGDR.V	3	3.44	0.14	
IPI00387105	Ig kappa chain V-I region Mev	-.DVQMTQSPSSLSASVGDR.V	2	5.13	0.23	

IPI00387105	Ig kappa chain V-I region Mev	-.DVQMTQSPSSLSASVGDR.V	3	2.92	0.20	
IPI00387106	Ig kappa chain V-I region Ni	-.DIQM*TQSPSSLSATVGDR.V	2	5.32	0.27	
IPI00387106	Ig kappa chain V-I region Ni	-.DIQM*TQSPSSLSATVGDR.V	3	4.06	0.34	
IPI00387106	Ig kappa chain V-I region Ni	-.DIQMTQSPSSLSATVGDR.V	2	5.31	0.12	
IPI00387110	Ig kappa chain V-II region MIL	-.DIVLTQSPLSLPVTPGEPASISCR.S	2	5.20	0.49	
IPI00387110	Ig kappa chain V-II region MIL	-.DIVLTQSPLSLPVTPGEPASISCR.S	3	4.06	0.36	
IPI00387113	Ig kappa chain V-III region B6	R.FSGSGSGADFTLTISR.L	2	3.79	0.37	
IPI00387115	Ig kappa chain V-III region SIE	G.EIVLTQSPGTLSLSPGER.A	2	6.33	0.22	
IPI00387115	Ig kappa chain V-III region SIE	R.ATGIPDRFSGSGSGTDFTLTISR.L	2	4.84	0.38	
IPI00387115	Ig kappa chain V-III region SIE	R.ATGIPDRFSGSGSGTDFTLTISR.L	3	3.97	0.23	
IPI00387115	Ig kappa chain V-III region SIE	R.FSGSGSGTDFTLTISR.L	1	2.55	0.22	
IPI00387115	Ig kappa chain V-III region SIE	R.FSGSGSGTDFTLTISR.L	2	4.49	0.53	
IPI00387115	Ig kappa chain V-III region SIE	R.LLIYGASSR.A	2	3.35	0.21	
IPI00387116	Ig kappa chain V-III region NG9 precursor (Fragment)	G.EIVLTQSPGTLSLSPGER.A	2	6.33	0.22	
IPI00387116	Ig kappa chain V-III region NG9 precursor (Fragment)	R.ASQSVSSSYLAWYQQKPGQAPR.L	2	5.52	0.40	
IPI00387116	Ig kappa chain V-III region NG9 precursor (Fragment)	R.ASQSVSSSYLAWYQQKPGQAPR.L	3	3.59	0.29	
IPI00387116	Ig kappa chain V-III region NG9 precursor (Fragment)	R.FSGSASGTDFTLTISR.L	2	3.65	0.26	
IPI00387117	Ig kappa chain V-III region Ti	G.EIVLTQSPGTLSLSPGER.A	2	6.33	0.22	
IPI00387117	Ig kappa chain V-III region Ti	R.ATGIPDRFSGSGSGTDFTLTISR.L	2	4.84	0.38	
IPI00387117	Ig kappa chain V-III region Ti	R.ATGIPDRFSGSGSGTDFTLTISR.L	3	3.97	0.23	
IPI00387117	Ig kappa chain V-III region Ti	R.FSGSGSGTDFTLTISR.L	1	2.55	0.22	
IPI00387117	Ig kappa chain V-III region Ti	R.FSGSGSGTDFTLTISR.L	2	4.49	0.53	
IPI00387118	Ig kappa chain V-III region WOL	G.EIVLTQSPGTLSLSPGER.A	2	6.33	0.22	
IPI00387118	Ig kappa chain V-III region WOL	R.ATGIPDRFSGSGSGTDFTLTISR.L	2	4.84	0.38	
IPI00387118	Ig kappa chain V-III region WOL	R.ATGIPDRFSGSGSGTDFTLTISR.L	3	3.97	0.23	
IPI00387118	Ig kappa chain V-III region WOL	R.FSGSGSGTDFTLTISR.L	1	2.55	0.22	
IPI00387118	Ig kappa chain V-III region WOL	R.FSGSGSGTDFTLTISR.L	2	4.49	0.53	
IPI00387118	Ig kappa chain V-III region WOL	R.LLIYGASSR.A	2	3.35	0.21	
IPI00387119	Ig kappa chain V-III region POM	-.EIVM*TQSPVTLSVSPGER.A	2	4.74	0.41	
IPI00387119	Ig kappa chain V-III region POM	-.EIVMTQSPVTLSVSPGER.A	2	4.42	0.31	
IPI00387119	Ig kappa chain V-III region POM	R.LLIYGASTR.A	2	3.10	0.20	
IPI00387120	Ig kappa chain V-IV region Len	G.DIVM*TQSPDSLAVSLGER.A	2	5.76	0.42	
IPI00387120	Ig kappa chain V-IV region Len	K.LLIYWASTR.E	2	2.79	0.24	
IPI00387120	Ig kappa chain V-IV region Len	K.NYLAWYQQKPGQPPK.L	3	2.60	0.33	
IPI00387159	Isoform 1 of Inhibitor of growth protein 3	K.FKM*ELEADNAGITEILERR.S	2	1.45	0.07	-6.41
IPI00387168	Isoform 1 of Proprotein convertase subtilisin/kexin type 9 precursor	-.MGTVSSRRSWWPLPLLLLLLLLLGPAGAR.A	3	3.58	0.09	

IPI00387168	Isoform 1 of Proprotein convertase subtilisin/kexin type 9 precursor	K.GTVSGTLIGLEFIR.K	2	3.37	0.28	-4.23
IPI00387168	Isoform 1 of Proprotein convertase subtilisin/kexin type 9 precursor	K.SQLVQPVGPLVVLLPLAGGYSR.V	2	5.18	0.55	-4.70
IPI00387168	Isoform 1 of Proprotein convertase subtilisin/kexin type 9 precursor	K.SQLVQPVGPLVVLLPLAGGYSR.V	3	3.58	0.32	-4.63
IPI00387168	Isoform 1 of Proprotein convertase subtilisin/kexin type 9 precursor	R.AHNAFGGEGVYAIAR.C	2	4.57	0.58	-3.17
IPI00387168	Isoform 1 of Proprotein convertase subtilisin/kexin type 9 precursor	R.AHNAFGGEGVYAIAR.C	3	3.72	0.21	-1.91
IPI00387168	Isoform 1 of Proprotein convertase subtilisin/kexin type 9 precursor	R.KSQLVQPVGPLVVLLPLAGGYSR.V	3	3.46	0.32	-5.45
IPI00387168	Isoform 1 of Proprotein convertase subtilisin/kexin type 9 precursor	R.SEEDGLAEAPEHGTTATFHR.C	3	2.74	0.37	-1.97
IPI00387168	Isoform 1 of Proprotein convertase subtilisin/kexin type 9 precursor	R.SEEDGLAEAPEHGTTATFHR.C	4	2.15	0.16	-1.14
IPI00394655	Isoform 4 of Neurofascin precursor	A.IEIPM*DPSIQNELTQPPTITK.Q	2	4.08	0.33	-5.69
IPI00394655	Isoform 4 of Neurofascin precursor	A.IEIPM*DPSIQNELTQPPTITK.Q	3	5.49	0.37	-4.35
IPI00394655	Isoform 4 of Neurofascin precursor	C.VASTELDQDLAK.A	2	3.06	0.26	-0.59
IPI00394655	Isoform 4 of Neurofascin precursor	F.RVIAINEVGSSHPSLPSER.Y	3	3.81	0.34	-3.64
IPI00394655	Isoform 4 of Neurofascin precursor	K.AAPYWLDEPKNLILAPGEDGR.L	2	4.06	0.39	-3.92
IPI00394655	Isoform 4 of Neurofascin precursor	K.AKFENFNK.A	2	2.12	0.09	-2.89
IPI00394655	Isoform 4 of Neurofascin precursor	K.AYLTVLADQATPTNR.L	3	3.35	0.21	-2.55
IPI00394655	Isoform 4 of Neurofascin precursor	K.EDDSLTIFGVAER.D	2	4.02	0.40	-4.17
IPI00394655	Isoform 4 of Neurofascin precursor	K.ENLDPVVVQEGAPLTLQCNPPPGLPSPVIFWM*SSSM*EPITQDKR.V	4	3.32	0.16	-7.39
IPI00394655	Isoform 4 of Neurofascin precursor	K.FGTALSNR.I	1	1.84	0.08	-2.90
IPI00394655	Isoform 4 of Neurofascin precursor	K.FGTALSNR.I	2	2.29	0.20	-3.34
IPI00394655	Isoform 4 of Neurofascin precursor	K.GGDLPSDKAK.F	2	2.36	0.05	-1.37
IPI00394655	Isoform 4 of Neurofascin precursor	K.GRPDRPRDLELTDLAER.S	4	3.27	0.20	-2.52
IPI00394655	Isoform 4 of Neurofascin precursor	K.KEDDSLTIFGVAER.D	3	3.42	0.15	0.13
IPI00394655	Isoform 4 of Neurofascin precursor	K.LTVSWLKDDEPLYIGNR.M	2	5.04	0.45	-3.74
IPI00394655	Isoform 4 of Neurofascin precursor	K.LTVSWLKDDEPLYIGNR.M	3	5.39	0.43	-2.28
IPI00394655	Isoform 4 of Neurofascin precursor	K.NLILAPGEDGR.L	1	2.33	0.21	-3.04
IPI00394655	Isoform 4 of Neurofascin precursor	K.NLILAPGEDGR.L	2	2.67	0.12	-2.58
IPI00394655	Isoform 4 of Neurofascin precursor	K.YPGSVNSAVLR.L	1	2.22	0.25	-3.47
IPI00394655	Isoform 4 of Neurofascin precursor	K.YPGSVNSAVLR.L	2	3.22	0.33	-1.52
IPI00394655	Isoform 4 of Neurofascin precursor	R.ANGNPKPTVQWM*VNGEPLQSAPPNPNREVAGDTIIFR.D	4	3.48	0.21	-3.58
IPI00394655	Isoform 4 of Neurofascin precursor	R.DLELTDLAER.S	1	2.84	0.22	-4.11
IPI00394655	Isoform 4 of Neurofascin precursor	R.DLELTDLAER.S	2	3.65	0.31	-4.60
IPI00394655	Isoform 4 of Neurofascin precursor	R.DNILIECEAK.G	2	3.39	0.28	-3.81
IPI00394655	Isoform 4 of Neurofascin precursor	R.DQGSYTCVASTELDQDLAK.A	2	7.00	0.59	-3.32
IPI00394655	Isoform 4 of Neurofascin precursor	R.DQGSYTCVASTELDQDLAK.A	3	4.18	0.48	-3.40

IPI00394655	Isoform 4 of Neurofascin precursor	R.EVAGDTIIFR.D	1	2.83	0.29	-2.15
IPI00394655	Isoform 4 of Neurofascin precursor	R.EVAGDTIIFR.D	2	1.94	0.11	-2.47
IPI00394655	Isoform 4 of Neurofascin precursor	R.EVAGDTIIFRDTQISSR.A	2	2.81	0.18	-1.92
IPI00394655	Isoform 4 of Neurofascin precursor	R.EVAGDTIIFRDTQISSR.A	3	2.41	0.18	-1.92
IPI00394655	Isoform 4 of Neurofascin precursor	R.FHFTHTIQQK.N	2	2.80	0.30	-3.01
IPI00394655	Isoform 4 of Neurofascin precursor	R.GM*DLLLECIASGVPTPDIAWYK.K	2	4.83	0.46	-4.12
IPI00394655	Isoform 4 of Neurofascin precursor	R.GM*DLLLECIASGVPTPDIAWYK.K	3	3.29	0.20	-4.49
IPI00394655	Isoform 4 of Neurofascin precursor	R.GM*DLLLECIASGVPTPDIAWYKK.G	2	4.30	0.53	-4.31
IPI00394655	Isoform 4 of Neurofascin precursor	R.GM*DLLLECIASGVPTPDIAWYKK.G	3	4.79	0.48	-4.02
IPI00394655	Isoform 4 of Neurofascin precursor	R.GMDLLLECIASGVPTPDIAWYKK.G	3	3.52	0.35	-3.68
IPI00394655	Isoform 4 of Neurofascin precursor	R.GTTVQLECR.V	2	2.51	0.19	-0.97
IPI00394655	Isoform 4 of Neurofascin precursor	R.ITNVSEEDSGEYFCLASNK.M	2	6.35	0.61	-4.36
IPI00394655	Isoform 4 of Neurofascin precursor	R.ITNVSEEDSGEYFCLASNK.M	3	3.19	0.36	-2.50
IPI00394655	Isoform 4 of Neurofascin precursor	R.IYRM*PEDQVAR.R	3	2.81	0.18	-2.90
IPI00394655	Isoform 4 of Neurofascin precursor	R.KEDQGIYTCVATNILGK.A	2	4.44	0.40	-3.92
IPI00394655	Isoform 4 of Neurofascin precursor	R.LDCPFFGSPIPTLR.W	2	3.63	0.41	0.22
IPI00394655	Isoform 4 of Neurofascin precursor	R.LSPYVNYQFR.V	1	2.20	0.20	-3.40
IPI00394655	Isoform 4 of Neurofascin precursor	R.LSPYVNYQFR.V	2	2.96	0.44	-2.72
IPI00394655	Isoform 4 of Neurofascin precursor	R.LTWIPGDANNSPITDYVVQFEEDQFQPGVWHDHSK.Y	4	3.11	0.25	-3.67
IPI00394655	Isoform 4 of Neurofascin precursor	R.M*KKEDDSLTIFGVAER.D	2	5.17	0.51	-3.80
IPI00394655	Isoform 4 of Neurofascin precursor	R.M*PEDQVAR.R	2	2.81	0.26	-2.71
IPI00394655	Isoform 4 of Neurofascin precursor	R.SGGRPEEYEGEYQCFAR.N	3	3.19	0.30	-1.96
IPI00394655	Isoform 4 of Neurofascin precursor	R.SGTLVIDFR.S	1	1.61	0.06	-3.58
IPI00394655	Isoform 4 of Neurofascin precursor	R.SGTLVIDFR.S	2	2.68	0.23	-6.00
IPI00394655	Isoform 4 of Neurofascin precursor	R.TPSFM*YPQGTASSQM*VLR.G	2	4.64	0.51	-3.64
IPI00394655	Isoform 4 of Neurofascin precursor	R.TPSFM*YPQGTASSQM*VLR.G	3	4.23	0.37	-3.47
IPI00394655	Isoform 4 of Neurofascin precursor	R.TSGAPPESNPGDVK.G	2	2.88	0.27	-2.95
IPI00394655	Isoform 4 of Neurofascin precursor	R.TSGAPPESNPGDVKGEGTR.K	2	4.49	0.53	-3.13
IPI00394655	Isoform 4 of Neurofascin precursor	R.TSGAPPESNPGDVKGEGTRK.N	2	4.02	0.50	-2.22
IPI00394655	Isoform 4 of Neurofascin precursor	R.TSGAPPESNPGDVKGEGTRK.N	4	2.72	0.31	-1.23
IPI00394655	Isoform 4 of Neurofascin precursor	R.VIAINEVGSSHPSLPSER.Y	2	4.76	0.48	-4.22
IPI00394655	Isoform 4 of Neurofascin precursor	R.VIAINEVGSSHPSLPSER.Y	3	3.16	0.34	-2.98
IPI00394655	Isoform 4 of Neurofascin precursor	R.VQAENDFGKGPEPESVIGYSGEDLPSAPR.R	3	6.03	0.50	-2.75
IPI00394655	Isoform 4 of Neurofascin precursor	R.VQAENDFGKGPEPESVIGYSGEDLPSAPRR.F	3	4.17	0.32	-3.55
IPI00394655	Isoform 4 of Neurofascin precursor	R.VSQGHNGDLYFSNVM*LQDM*QTDYSCNAR.F	3	7.42	0.61	-3.33
IPI00394655	Isoform 4 of Neurofascin precursor	R.YRTSGAPPESNPGDVKGEGTR.K	3	4.48	0.39	-2.89
IPI00394655	Isoform 4 of Neurofascin precursor	R.YVVGQTPVYVPYEIR.V	2	4.79	0.42	-3.80
IPI00394655	Isoform 4 of Neurofascin precursor	W.LKDDEPLYIGNR.M	2	3.77	0.30	-1.91
IPI00394655	Isoform 4 of Neurofascin precursor	W.M*VNGEPLQSAPPNPNR.E	2	3.85	0.40	-1.22
IPI00394712	Granulocyte inhibitory protein	-.DIVM*TQSPGTLSVSPGER.A	2	3.44	0.17	
IPI00394820	Olfactomedin-like protein 1 precursor	K.SAVGNLALR.V	2	1.87	0.07	-2.10
IPI00394870	Brorin precursor	K.LAQAPEQPGQEKR.E	2	3.17	0.42	-3.01

IPI00394870	Brorin precursor	K.LAQAPEQPGQEKR.E	3	3.31	0.20	-3.01
IPI00394879	Leucine-rich repeat-containing protein 9	K.LPEERVKLFSFVKK.T	1	2.36	0.05	
IPI00394992	Isoform 2 of N-acetylmuramoyl-L-alanine amidase precursor	A.SLPLLM*DSVIQALAELEQK.V	2	6.11	0.46	-4.29
IPI00394992	Isoform 2 of N-acetylmuramoyl-L-alanine amidase precursor	A.SLPLLM*DSVIQALAELEQK.V	3	5.28	0.35	-3.10
IPI00394992	Isoform 2 of N-acetylmuramoyl-L-alanine amidase precursor	A.SLPLLM*DSVIQALAELEQKVPAAK.T	2	4.82	0.48	-4.58
IPI00394992	Isoform 2 of N-acetylmuramoyl-L-alanine amidase precursor	A.SLPLLM*DSVIQALAELEQKVPAAK.T	4	4.95	0.44	-2.28
IPI00394992	Isoform 2 of N-acetylmuramoyl-L-alanine amidase precursor	A.SLPLLMDSVIQALAELEQK.V	2	4.45	0.41	-2.33
IPI00394992	Isoform 2 of N-acetylmuramoyl-L-alanine amidase precursor	A.SLPLLMDSVIQALAELEQKVPAAK.T	2	5.34	0.38	-4.42
IPI00394992	Isoform 2 of N-acetylmuramoyl-L-alanine amidase precursor	D.GSPDVTTADIGANTPDATK.G	2	4.15	0.51	-2.32
IPI00394992	Isoform 2 of N-acetylmuramoyl-L-alanine amidase precursor	E.TGDTFPDVVAIAPDVR.A	2	2.90	0.16	-4.28
IPI00394992	Isoform 2 of N-acetylmuramoyl-L-alanine amidase precursor	K.ASLLTM*AFLNGALDGVILGDYLSR.T	2	5.56	0.51	-5.10
IPI00394992	Isoform 2 of N-acetylmuramoyl-L-alanine amidase precursor	K.EFTEAFLGCPAIHPR.C	3	2.72	0.38	-2.71
IPI00394992	Isoform 2 of N-acetylmuramoyl-L-alanine amidase precursor	K.EYGVVLAPDGSTVAVEPLLAGLEAGLQGR.R	2	5.16	0.59	-2.92
IPI00394992	Isoform 2 of N-acetylmuramoyl-L-alanine amidase precursor	K.EYGVVLAPDGSTVAVEPLLAGLEAGLQGR.R	3	6.62	0.54	-4.62
IPI00394992	Isoform 2 of N-acetylmuramoyl-L-alanine amidase precursor	K.GCPDVQASLPDAK.A	2	2.72	0.30	-2.48
IPI00394992	Isoform 2 of N-acetylmuramoyl-L-alanine amidase precursor	K.LLQLPLGFLYVHHTYVPAPPCTDFTR.C	3	3.87	0.33	-4.57
IPI00394992	Isoform 2 of N-acetylmuramoyl-L-alanine amidase precursor	K.LLQLPLGFLYVHHTYVPAPPCTDFTR.C	4	2.63	0.10	-4.36
IPI00394992	Isoform 2 of N-acetylmuramoyl-L-alanine amidase precursor	L.PLLM*DSVIQALAELEQKVPAAK.T	3	3.98	0.43	-1.03
IPI00394992	Isoform 2 of N-acetylmuramoyl-L-alanine amidase precursor	M.AFLNGALDGVILGDYLSR.T	2	5.52	0.53	-6.31
IPI00394992	Isoform 2 of N-acetylmuramoyl-L-alanine amidase precursor	M.AFLNGALDGVILGDYLSR.T	3	4.19	0.29	-3.82
IPI00394992	Isoform 2 of N-acetylmuramoyl-L-alanine amidase precursor	R.AGLLRPDYALLGHR.Q	2	3.86	0.46	-2.08
IPI00394992	Isoform 2 of N-acetylmuramoyl-L-alanine amidase precursor	R.AGLLRPDYALLGHR.Q	3	4.59	0.35	-2.91

IPI00394992	Isoform 2 of N-acetylmuramoyl-L-alanine amidase precursor	R.DGSPDVTTADIGANTPDATK.G	2	6.12	0.59	-2.87
IPI00394992	Isoform 2 of N-acetylmuramoyl-L-alanine amidase precursor	R.DGSPDVTTADIGANTPDATK.G	3	3.31	0.35	-1.24
IPI00394992	Isoform 2 of N-acetylmuramoyl-L-alanine amidase precursor	R.DTLPSCAVR.A	2	2.69	0.27	-3.42
IPI00394992	Isoform 2 of N-acetylmuramoyl-L-alanine amidase precursor	R.EGKEYGVVLAPDGSTVAVEPLLAGLEAGLQGR.R	2	3.34	0.52	-3.71
IPI00394992	Isoform 2 of N-acetylmuramoyl-L-alanine amidase precursor	R.EGKEYGVVLAPDGSTVAVEPLLAGLEAGLQGR.R	3	7.36	0.64	-4.83
IPI00394992	Isoform 2 of N-acetylmuramoyl-L-alanine amidase precursor	R.PSLSHLLSQYYGAGVAR.D	2	4.86	0.49	-5.19
IPI00394992	Isoform 2 of N-acetylmuramoyl-L-alanine amidase precursor	R.PSLSHLLSQYYGAGVAR.D	3	5.19	0.39	-4.26
IPI00394992	Isoform 2 of N-acetylmuramoyl-L-alanine amidase precursor	R.QNGAALTSASILAQQVWGTLVLLQR.L	3	4.32	0.19	-3.01
IPI00394992	Isoform 2 of N-acetylmuramoyl-L-alanine amidase precursor	R.QNGAALTSASILAQQVWGTLVLLQR.L	4	2.99	0.13	-0.95
IPI00394992	Isoform 2 of N-acetylmuramoyl-L-alanine amidase precursor	R.RVINLPLDSM*AAPWETGDTFPDVVAIAPDVR.A	3	5.87	0.53	-3.82
IPI00394992	Isoform 2 of N-acetylmuramoyl-L-alanine amidase precursor	R.TDCPGDALFDLLR.T	2	3.97	0.36	-3.05
IPI00394992	Isoform 2 of N-acetylmuramoyl-L-alanine amidase precursor	R.TFTLLDPK.A	2	2.53	0.25	-2.38
IPI00394992	Isoform 2 of N-acetylmuramoyl-L-alanine amidase precursor	R.TPEPRPSLSHLLSQYYGAGVAR.D	2	3.46	0.36	-4.66
IPI00394992	Isoform 2 of N-acetylmuramoyl-L-alanine amidase precursor	R.TPEPRPSLSHLLSQYYGAGVAR.D	3	5.70	0.49	-4.27
IPI00394992	Isoform 2 of N-acetylmuramoyl-L-alanine amidase precursor	R.TPEPRPSLSHLLSQYYGAGVAR.D	4	3.73	0.36	-3.26
IPI00394992	Isoform 2 of N-acetylmuramoyl-L-alanine amidase precursor	S.LPLLM*DSVIQALAELEQKVPAAK.T	3	5.73	0.49	-3.66
IPI00394992	Isoform 2 of N-acetylmuramoyl-L-alanine amidase precursor	W.ETGDTFPDVVAIAPDVR.A	2	3.69	0.41	-2.18
IPI00394992	Isoform 2 of N-acetylmuramoyl-L-alanine amidase precursor	W.ETGDTFPDVVAIAPDVR.A	3	3.98	0.20	-2.12
IPI00395488	Vasorin precursor	R.ESHVTLASPEETR.C	2	3.58	0.42	-2.34
IPI00395488	Vasorin precursor	R.ESHVTLASPEETR.C	3	2.67	0.15	-1.50
IPI00395488	Vasorin precursor	R.HIQPGAFDTLDR.L	2	3.00	0.37	-2.73
IPI00395488	Vasorin precursor	R.HIQPGAFDTLDR.L	3	2.55	0.11	-3.52
IPI00395488	Vasorin precursor	R.IAQLRPEDLAGLAALQELDVSNLSLQALPGDLSGLFPR.L	3	7.55	0.68	-3.69
IPI00395488	Vasorin precursor	R.IAQLRPEDLAGLAALQELDVSNLSLQALPGDLSGLFPR.L	4	4.75	0.45	-3.32

IPI00395488	Vasorin precursor	R.IAQLRPEDLAGLAALQELDVSNLSLQALPGDLSGLFPR.L	5	3.44	0.31	-4.39
IPI00395488	Vasorin precursor	R.IAQLRPEDLAGLAALQELDVSNLSLQALPGDLSGLFPR.L	6	3.07	0.18	-0.92
IPI00395488	Vasorin precursor	R.IRHIQPGAFDTLDR.L	2	3.74	0.19	-4.05
IPI00395488	Vasorin precursor	R.LAGLGLQQLDEGLFSR.L	2	5.22	0.50	-4.04
IPI00395488	Vasorin precursor	R.LLLLDLSHNSLLALEPGILDTANVEALR.L	3	5.78	0.45	-6.84
IPI00395488	Vasorin precursor	R.LLLLDLSHNSLLALEPGILDTANVEALR.L	4	3.77	0.28	-5.48
IPI00395488	Vasorin precursor	R.LRNLHDLDVSDNQLER.V	2	6.03	0.53	-1.62
IPI00395488	Vasorin precursor	R.LRNLHDLDVSDNQLER.V	3	5.08	0.24	-3.55
IPI00395488	Vasorin precursor	R.NLHDLDVSDNQLER.V	2	4.37	0.51	-0.66
IPI00395488	Vasorin precursor	R.NLHDLDVSDNQLER.V	3	4.08	0.24	-2.02
IPI00395488	Vasorin precursor	R.SLTLGIEPVSPTSLR.V	2	4.48	0.43	-3.29
IPI00395488	Vasorin precursor	R.YLQGSSVQLR.S	1	2.08	0.16	-1.26
IPI00395488	Vasorin precursor	R.YLQGSSVQLR.S	2	3.17	0.27	-1.29
IPI00395866	SCUBE1 protein	K.YALHSDGR.T	2	2.32	0.29	-3.08
IPI00396077	Isoform 1 of E3 ubiquitin-protein ligase Topors	K.HKKHHGDNASRSPVVITIDSDSDK.D	2	2.40	0.14	
IPI00396378	Isoform B1 of Heterogeneous nuclear ribonucleoproteins A2/B1	R.GFGFVTFDDHDPVDKIVLQK.Y	3	3.74	0.40	-5.30
IPI00396378	Isoform B1 of Heterogeneous nuclear ribonucleoproteins A2/B1	R.GFGFVTFSSMAEVDAAMAARPHSIDGR.V	3	2.94	0.21	-3.75
IPI00396378	Isoform B1 of Heterogeneous nuclear ribonucleoproteins A2/B1	R.QEM*QEVQSSR.S	2	2.48	0.26	-2.56
IPI00396383	Isoform 1 of von Willebrand factor A domain-containing protein 1 precursor	R.APTPGTASREP.-	2	2.50	0.32	-0.29
IPI00396383	Isoform 1 of von Willebrand factor A domain-containing protein 1 precursor	R.GSILDAM*RPQQLHATEITSSGFR.L	3	2.51	0.26	-3.24
IPI00396423	Alcadein beta	E.IVHNLDGCEISLVGDDLDPERESLLLDTTSLQQR.G	3	6.33	0.51	-3.46
IPI00396423	Alcadein beta	E.IVHNLDGCEISLVGDDLDPERESLLLDTTSLQQR.G	4	4.55	0.33	-2.95
IPI00396423	Alcadein beta	H.VLSSQQFLHR.G	2	3.20	0.17	-2.00
IPI00396423	Alcadein beta	K.RIEYAPGAGSLALFPGIR.L	2	4.11	0.47	-3.67
IPI00396423	Alcadein beta	K.VHVNPSQSLLTLEGDDVETFNHALQHV.A	3	5.09	0.53	-4.36
IPI00396423	Alcadein beta	K.VHVNPSQSLLTLEGDDVETFNHALQHVAYM*NTLR.F	4	3.87	0.26	-3.40
IPI00396423	Alcadein beta	R.EGLDYRDFESLGK.G	2	2.74	0.26	0.71
IPI00396423	Alcadein beta	R.EGLDYRDFESLGK.G	3	3.04	0.22	-0.86
IPI00396423	Alcadein beta	R.ESLLLDTTSLQQR.G	2	4.18	0.42	-1.92
IPI00396423	Alcadein beta	R.EVIECLYACR.E	2	2.51	0.34	-3.38
IPI00396423	Alcadein beta	R.FATPGVRPLR.L	2	1.91	0.07	-1.49
IPI00396423	Alcadein beta	R.GHQPPPEM*AGHSLASSHR.N	2	3.91	0.49	-4.98
IPI00396423	Alcadein beta	R.IEYAPGAGSLALFPGIR.L	2	3.38	0.31	
IPI00396423	Alcadein beta	R.LHGSGVPFEAVILDKATGEGLIR.A	4	3.64	0.09	-3.07
IPI00396423	Alcadein beta	R.LSCSEM*NGR.Y	2	1.90	0.11	-2.59
IPI00396423	Alcadein beta	R.M*SDEIVHNLDGCEISLVGDDLDPERESLLLDTTSLQQR.G	3	4.84	0.51	-2.40
IPI00396423	Alcadein beta	R.M*SDEIVHNLDGCEISLVGDDLDPERESLLLDTTSLQQR.G	4	5.11	0.26	-5.03

IPI00396423	Alcadein beta	R.YAGEICGFR.L	2	2.50	0.22	-2.12
IPI00396423	Alcadein beta	V.LSSQQFLHR.G	2	3.28	0.23	-1.70
IPI00396930	Uncharacterized protein ENSP00000353216 (Fragment)	R.AEDTAVYYCVK.H	2	4.09	0.12	
IPI00396930	Uncharacterized protein ENSP00000353216 (Fragment)	R.DNANNSPYLQM*NSLR.A	2	2.91	0.13	
IPI00396961	Leucine-rich repeat and fibronectin type-III domain-containing protein 5 precursor	K.DTGAFTCIASNPAGEATQIVDLHIIK.L	3	4.05	0.49	-3.50
IPI00396961	Leucine-rich repeat and fibronectin type-III domain-containing protein 5 precursor	R.LADNFVTNIK.R	2	2.78	0.20	-2.31
IPI00397578	135 kDa protein	T.ELRAEEIETRVTSGSM*EALNLK.Q	2	2.91	0.16	-7.99
IPI00397645	Isoform 2 of Matrix-remodeling-associated protein 7	G.LGELGEPAGPGEPEGPGDPAAAPAEAEEQAVEAR.Q	3	4.85	0.45	-3.09
IPI00397645	Isoform 2 of Matrix-remodeling-associated protein 7	K.M*M*TKEELEEEQRTEE.-	2	3.28	0.49	-4.07
IPI00397645	Isoform 2 of Matrix-remodeling-associated protein 7	K.M*M*TKEELEEEQRTEE.-	3	2.56	0.15	-2.69
IPI00397645	Isoform 2 of Matrix-remodeling-associated protein 7	R.QEEEQDLDGEKGPSSEGPEEEDGEGFSFK.Y	3	5.35	0.41	-1.93
IPI00397949	G protein-coupled receptor 56 isoform b	R.DLQLLSQFLK.H	2	2.88	0.24	-3.76
IPI00398154	actin filament associated protein 1	K.KSSKSEAKGTVSKVTGKKITKIISLGK.K	3	2.94	0.12	
IPI00398229	similar to deubiquitinating enzyme 3	K.NVQYPK.C	1	1.64	0.05	-1.63
IPI00398715	Neuropilin 1	K.EGNKPVLFQGNTNPTDVVVAVFPKPLITR.F	3	3.04	0.35	-4.25
IPI00398715	Neuropilin 1	K.EGNKPVLFQGNTNPTDVVVAVFPKPLITR.F	4	3.51	0.30	-2.50
IPI00398715	Neuropilin 1	K.FVSDYETHGAGFSIR.Y	2	4.43	0.47	-4.03
IPI00398715	Neuropilin 1	K.IAPPPVVSSGPFLFIK.F	2	1.36	0.49	-3.69
IPI00398715	Neuropilin 1	K.IAPPPVVSSGPFLFIK.F	3	4.77	0.46	-1.49
IPI00398715	Neuropilin 1	K.SFEGNNNYDTPELR.T	2	3.75	0.46	-4.94
IPI00398715	Neuropilin 1	R.EWIQVDLGLLR.F	2	3.02	0.36	-2.23
IPI00398715	Neuropilin 1	R.FVTAVGTQGAISK.E	2	4.71	0.37	-3.52
IPI00398715	Neuropilin 1	R.IM*INFNPHFDLEDR.D	3	3.14	0.19	-3.83
IPI00398918	Putative uncharacterized protein DKFZp686I21167	K.FPHPIEISEDVITGPTIK.N	3	2.32	0.16	-2.65
IPI00398918	Putative uncharacterized protein DKFZp686I21167	K.QTQKFPHPIEISEDVITGPTIK.N	3	3.61	0.27	-2.78
IPI00398918	Putative uncharacterized protein DKFZp686I21167	K.SLTFDKEVK.V	2	1.95	0.14	-2.85
IPI00398992	Isoform 1 of Chromodomain-helicase-DNA-binding protein 8	R.SKLYDEESLLSLTMSQDGFP.N	3	3.76	0.23	-1.93
IPI00399089	Mesoderm development candidate 2	R.DGSYAWEIKDFLVGQDR.C	3	3.90	0.30	-4.12
IPI00399089	Mesoderm development candidate 2	R.DYNDADM*AR.L	2	2.29	0.16	-1.96
IPI00399180	Serine/threonine-protein kinase SBK1	K.VDLVVYK.G	2	1.86	0.07	-3.46
IPI00399252	Isoform 1 of Protein Jade-1	K.VNYNQTAVKVPTTPASPVKNWGGFRIPK.K	4	2.90	0.11	-5.97
IPI00399254	Isoform 1 of OTU domain-containing protein 4	K.CQVRLDHNGKFLNADVQGIHSENGPVLVEELGK.K	3	3.84	0.18	0.97
IPI00399296	hypothetical protein LOC390110	R.GKTYMCK.E	1	1.04	0.18	-0.74

IPI00399328	similar to jumonji domain containing 2D	R.GCEAFLRHKVALISPTVLKENGIPFNCM*TQEAG.E	3	3.68	0.14	0.44
IPI00400935	Isoform 1 of Collagen alpha-1(XVI) chain precursor	R.AQGQDGDFVSCIFPVPQLFDLR.W	3	4.48	0.29	-2.62
IPI00400935	Isoform 1 of Collagen alpha-1(XVI) chain precursor	R.LGAAPVTQPTR.R	2	2.67	0.33	-2.07
IPI00400967	KIAA1843 protein (Fragment)	R.YQEQGAK.L	2	1.28	0.12	-2.97
IPI00400986	hypothetical protein LOC85459	P.QQDNLKALQEQLATQR.E	2	3.05	0.28	-5.27
IPI00401264	Thioredoxin domain-containing protein 4 precursor	R.DLAEITTLDR.S	2	3.92	0.36	-3.86
IPI00401264	Thioredoxin domain-containing protein 4 precursor	R.FSQM*LHPIFEEASDVIKEEFPNENQVVFAR.V	4	3.04	0.21	-4.63
IPI00401264	Thioredoxin domain-containing protein 4 precursor	R.HM*YVFGDFKDVLIPGK.L	3	2.84	0.32	-3.80
IPI00401264	Thioredoxin domain-containing protein 4 precursor	R.HM*YVFGDFKDVLIPGK.L	4	3.32	0.26	-3.40
IPI00401283	Multiple epidermal growth factor-like domains 9 precursor	K.VGVIGSICDR.C	2	3.57	0.34	-2.15
IPI00401283	Multiple epidermal growth factor-like domains 9 precursor	R.CPCSAVTSTGSCSIK.S	2	4.10	0.42	-2.39
IPI00401283	Multiple epidermal growth factor-like domains 9 precursor	R.CQDGYYGFSK.N	2	2.30	0.24	-2.33
IPI00401283	Multiple epidermal growth factor-like domains 9 precursor	R.GEPSHPFPR.A	2	2.14	0.36	0.32
IPI00401852	Conserved hypothetical protein	R.KM*PGDM*SSSPR.V	3	2.31	0.18	
IPI00402144	Isoform 1 of Zinc finger protein 555	K.ECGKVFKWPSSLPIHM*R.L	3	2.49	0.21	-2.49
IPI00402157	Cerebellin-3 precursor	R.AAAGGPGGAALGEAPPGR.V	2	4.97	0.50	-3.30
IPI00402157	Cerebellin-3 precursor	R.AAAGGPGGAALGEAPPGR.V	3	3.71	0.19	-2.20
IPI00402157	Cerebellin-3 precursor	R.AAAGGPGGAALGEAPPGRV.A	2	3.70	0.40	-2.41
IPI00402157	Cerebellin-3 precursor	R.ASGSFVAPVR.G	1	1.88	0.22	-2.76
IPI00402157	Cerebellin-3 precursor	R.ASGSFVAPVR.G	2	3.39	0.24	-0.89
IPI00402157	Cerebellin-3 precursor	R.ASGSFVAPVRGVYSFR.F	2	2.20	0.11	-3.35
IPI00402157	Cerebellin-3 precursor	R.EAATSSVLLPLDPGDR.V	2	3.61	0.24	-4.51
IPI00402157	Cerebellin-3 precursor	R.EAATSSVLLPLDPGDRVSLR.L	2	2.28	0.20	-3.16
IPI00402157	Cerebellin-3 precursor	R.EAATSSVLLPLDPGDRVSLR.L	3	2.88	0.28	-2.82
IPI00402293	Arylsulfatase G precursor	K.AFYITGGAR.A	2	2.73	0.25	-2.78
IPI00402293	Arylsulfatase G precursor	R.VLFHPNSGAAGEFGALQTVR.L	3	2.62	0.16	-4.69
IPI00402293	Arylsulfatase G precursor	R.YKAFYITGGAR.A	3	2.87	0.05	-3.99
IPI00409640	Isoform 1 of Lipolysis-stimulated lipoprotein receptor	K.QGNAVTLGDYYQGR.R	2	2.22	0.19	-2.35
IPI00409640	Isoform 1 of Lipolysis-stimulated lipoprotein receptor	R.IQASQQDDSM*R.V	2	3.30	0.40	-3.03
IPI00409640	Isoform 1 of Lipolysis-stimulated lipoprotein receptor	R.SGDLPYDGR.L	2	2.53	0.22	-2.76
IPI00409640	Isoform 1 of Lipolysis-stimulated lipoprotein receptor	R.SRDDLYDQDDSRDFPR.S	3	2.41	0.24	-2.19

IPI00409640	Isoform 1 of Lipolysis-stimulated lipoprotein receptor	R.SRDPHYDDFR.S	2	2.53	0.24	-3.47
IPI00409640	Isoform 1 of Lipolysis-stimulated lipoprotein receptor	R.SVDALDDLTPPSTAESGSR.S	2	4.50	0.31	-5.34
IPI00410013	Isoform 1 of Zinc finger CCCH domain-containing protein 3	R.HSRRAATSPAPGPSDATARSRVSASHGPR.K	3	2.92	0.13	
IPI00410079	Isoform 1 of Protein FAM82C	K.TATALLESPLSATVEDALQSFLK.A	2	5.37	0.59	-4.48
IPI00410079	Isoform 1 of Protein FAM82C	K.TATALLESPLSATVEDALQSFLK.A	3	3.69	0.31	-4.07
IPI00410079	Isoform 1 of Protein FAM82C	K.TATALLESPLSATVEDALQSFLKAEELQPGFSK.A	3	4.10	0.40	-4.11
IPI00410079	Isoform 1 of Protein FAM82C	K.TATALLESPLSATVEDALQSFLKAEELQPGFSK.A	4	3.16	0.14	-4.04
IPI00410093	coiled-coil domain containing 69	R.DRLDEQQRVLEGKNEEALQVLR.A	3	3.58	0.06	
IPI00410122	Isoform 1 of Plexin domain-containing protein 1 precursor	K.IHTILSNTHR.Q	2	2.56	0.15	-4.26
IPI00410122	Isoform 1 of Plexin domain-containing protein 1 precursor	K.TGLSDAFM*ILNPSPDVPESR.R	3	4.35	0.28	-4.20
IPI00410122	Isoform 1 of Plexin domain-containing protein 1 precursor	R.LYGPSEPHSR.E	2	2.40	0.21	-2.91
IPI00410122	Isoform 1 of Plexin domain-containing protein 1 precursor	R.VVEDNHSYYVSR.L	2	3.62	0.36	-1.66
IPI00410122	Isoform 1 of Plexin domain-containing protein 1 precursor	R.VVLSFDFPFYGHPLR.Q	2	1.89	0.18	0.02
IPI00410210	Isoform 2 of Latrophilin-1 precursor	K.LM*EQLLDILDAQLQALRPIER.E	3	2.99	0.11	-4.10
IPI00410210	Isoform 2 of Latrophilin-1 precursor	K.SGETVINTANYHDTSPYR.W	2	5.31	0.62	-3.33
IPI00410210	Isoform 2 of Latrophilin-1 precursor	R.AGLPFGLM*R.R	2	2.48	0.17	-1.32
IPI00410210	Isoform 2 of Latrophilin-1 precursor	R.CPGSDVIM*VENANYGR.T	2	4.08	0.33	-5.50
IPI00410210	Isoform 2 of Latrophilin-1 precursor	R.EEPVSLTFPNPYQFISSVDYNPR.D	3	4.39	0.46	-4.12
IPI00410210	Isoform 2 of Latrophilin-1 precursor	R.ELACEGYPIELR.C	1	1.78	0.35	-3.29
IPI00410210	Isoform 2 of Latrophilin-1 precursor	R.ELACEGYPIELR.C	2	3.96	0.38	-4.14
IPI00410210	Isoform 2 of Latrophilin-1 precursor	R.IKSGETVINTANYHDTSPYR.W	2	5.95	0.60	-1.83
IPI00410210	Isoform 2 of Latrophilin-1 precursor	R.IKSGETVINTANYHDTSPYR.W	3	4.23	0.45	-0.15
IPI00410210	Isoform 2 of Latrophilin-1 precursor	R.IYVM*PWIPYR.T	2	2.78	0.19	-2.17
IPI00410210	Isoform 2 of Latrophilin-1 precursor	R.LPNRVDGTGFVVYDGAVFYNKER.T	4	3.21	0.20	-3.35
IPI00410210	Isoform 2 of Latrophilin-1 precursor	R.LVVSQLNPYTLR.F	1	2.49	0.20	-2.41
IPI00410210	Isoform 2 of Latrophilin-1 precursor	R.LVVSQLNPYTLR.F	2	4.31	0.40	-3.40
IPI00410210	Isoform 2 of Latrophilin-1 precursor	R.SVYVDDDSEAAGNR.V	2	3.58	0.54	-3.78
IPI00410210	Isoform 2 of Latrophilin-1 precursor	R.SVYVDDDSEAAGNRVDYAFNTNANR.E	3	3.69	0.45	-3.03
IPI00410210	Isoform 2 of Latrophilin-1 precursor	R.TDDKICDADPFQM*ENVQCYLPDAFK.I	3	5.37	0.57	-3.51
IPI00410210	Isoform 2 of Latrophilin-1 precursor	R.TDTLTEYASWEDYVAAR.H	2	5.22	0.58	-4.64
IPI00410210	Isoform 2 of Latrophilin-1 precursor	R.TQCVVVAGSDAFPDPCPGTYK.Y	2	5.27	0.57	-3.64
IPI00410210	Isoform 2 of Latrophilin-1 precursor	R.TQCVVVAGSDAFPDPCPGTYK.Y	3	2.89	0.19	-3.30
IPI00410210	Isoform 2 of Latrophilin-1 precursor	R.VDYAFNTNANREEPVSLTFPNPYQFISSVDYNPR.D	3	4.06	0.43	-1.56

IPI00410487	Isoform 1 of Twisted gastrulation protein homolog 1 precursor	K.ALCASDVSK.C	1	2.18	0.28	-1.32
IPI00410487	Isoform 1 of Twisted gastrulation protein homolog 1 precursor	K.ALCASDVSK.C	2	2.14	0.18	-3.57
IPI00410487	Isoform 1 of Twisted gastrulation protein homolog 1 precursor	K.ISCESM*GASK.Y	2	3.03	0.37	-1.72
IPI00410487	Isoform 1 of Twisted gastrulation protein homolog 1 precursor	K.STVEELHEPIPSLFR.A	2	3.21	0.39	-2.17
IPI00410487	Isoform 1 of Twisted gastrulation protein homolog 1 precursor	K.STVEELHEPIPSLFR.A	3	2.67	0.23	-0.07
IPI00410488	Isoform 1 of CD276 antigen precursor	R.SPTGAVEVQVPEDPVVALVGTDATLR.C	3	3.17	0.28	-2.76
IPI00410585	Isoform 1 of Crumbs homolog 2 precursor	R.GGHGLPGAVLPIPGPR.V	3	2.61	0.08	-2.45
IPI00410585	Isoform 1 of Crumbs homolog 2 precursor	R.SDPALYGGVQAAFPGAFSFR.H	2	4.62	0.50	-5.24
IPI00410585	Isoform 1 of Crumbs homolog 2 precursor	R.VALGGLPLPLARPRPGAAPGAR.E	3	2.40	0.20	-3.08
IPI00410585	Isoform 1 of Crumbs homolog 2 precursor	R.WLLWLDGAATPVALR.G	2	4.13	0.49	-4.47
IPI00410588	ADAMTS-like protein 3 precursor	A.EKSPGAYFLPEFALSPQGSFLEDTTGEQFLTYR.Y	3	4.71	0.47	-4.18
IPI00410600	Isoform 3 of Voltage-dependent calcium channel subunit alpha-2/delta-2 precursor	K.ADAELDDPESEDVER.G	2	4.83	0.48	-2.56
IPI00410600	Isoform 3 of Voltage-dependent calcium channel subunit alpha-2/delta-2 precursor	K.EAVQGM*VAK.G	1	1.43	0.21	-3.74
IPI00410600	Isoform 3 of Voltage-dependent calcium channel subunit alpha-2/delta-2 precursor	K.EAVQGM*VAK.G	2	2.35	0.19	-2.35
IPI00410600	Isoform 3 of Voltage-dependent calcium channel subunit alpha-2/delta-2 precursor	K.GYYFEIPSIGAIR.I	2	4.23	0.36	-4.59
IPI00410600	Isoform 3 of Voltage-dependent calcium channel subunit alpha-2/delta-2 precursor	K.IDLYDVR.R	2	2.38	0.25	-2.33
IPI00410600	Isoform 3 of Voltage-dependent calcium channel subunit alpha-2/delta-2 precursor	K.KIDLYDVR.R	2	2.11	0.05	-3.74
IPI00410600	Isoform 3 of Voltage-dependent calcium channel subunit alpha-2/delta-2 precursor	K.VAGDIESLLDR.K	2	1.44	0.20	-2.40
IPI00410600	Isoform 3 of Voltage-dependent calcium channel subunit alpha-2/delta-2 precursor	K.VAGDIESLLDRK.V	2	3.01	0.30	-1.68
IPI00410600	Isoform 3 of Voltage-dependent calcium channel subunit alpha-2/delta-2 precursor	K.VAGDIESLLDRK.V	3	2.51	0.16	-1.19
IPI00410600	Isoform 3 of Voltage-dependent calcium channel subunit alpha-2/delta-2 precursor	K.YFEFLLPSSFESEGHVFIAPR.E	3	3.70	0.37	-2.77
IPI00410600	Isoform 3 of Voltage-dependent calcium channel subunit alpha-2/delta-2 precursor	R.DQDLNTYSLLAVFAATDGGITR.V	2	5.71	0.56	-3.93
IPI00410600	Isoform 3 of Voltage-dependent calcium channel subunit alpha-2/delta-2 precursor	R.DQDLNTYSLLAVFAATDGGITR.V	3	3.40	0.14	-2.35
IPI00410600	Isoform 3 of Voltage-dependent calcium channel subunit alpha-2/delta-2 precursor	R.IFGGVQQLR.E	2	3.58	0.13	-1.94

IPI00410600	Isoform 3 of Voltage-dependent calcium channel subunit alpha-2/delta-2 precursor	R.INTQEYLDVLGRPM*VLAGK.E	2	4.20	0.50	-3.96
IPI00410600	Isoform 3 of Voltage-dependent calcium channel subunit alpha-2/delta-2 precursor	R.INTQEYLDVLGRPM*VLAGK.E	3	3.55	0.24	-0.15
IPI00410600	Isoform 3 of Voltage-dependent calcium channel subunit alpha-2/delta-2 precursor	R.LADAAENFQK.A	2	2.78	0.24	-2.96
IPI00410600	Isoform 3 of Voltage-dependent calcium channel subunit alpha-2/delta-2 precursor	R.LEQEVDGVM*R.I	2	2.49	0.08	-1.66
IPI00410600	Isoform 3 of Voltage-dependent calcium channel subunit alpha-2/delta-2 precursor	R.NLFEVQENEPQKLVEK.V	2	4.39	0.21	-1.40
IPI00410600	Isoform 3 of Voltage-dependent calcium channel subunit alpha-2/delta-2 precursor	R.QDPTLLWQVFGSATGVTR.Y	2	5.02	0.48	-3.41
IPI00410600	Isoform 3 of Voltage-dependent calcium channel subunit alpha-2/delta-2 precursor	R.RLEQEVDGVM*R.I	2	1.73	0.15	-3.57
IPI00410600	Isoform 3 of Voltage-dependent calcium channel subunit alpha-2/delta-2 precursor	R.TLRPAVVGVK.L	2	2.43	0.15	-2.20
IPI00410657	Isoform 2 of mRNA cap guanine-N7 methyltransferase	K.VDDYEHAAKYMKNSQVRLPLGTLSK.S	3	2.47	0.14	-5.91
IPI00410675	Syntaxin-1B	K.TKQELEDLTADIKK.T	3	4.40	0.39	-1.88
IPI00410675	Syntaxin-1B	R.ELHDMFVDMAMLVESQGEMIDR.I	3	3.01	0.26	-2.35
IPI00410714	Hemoglobin subunit alpha	K.FLASVSTVLTSK.Y	2	3.47	0.42	-5.13
IPI00410714	Hemoglobin subunit alpha	K.GHGKKVADALTNAVAHVDDMPNALSALSDLHAHK.L	5	3.66	0.19	-3.33
IPI00410714	Hemoglobin subunit alpha	K.GHGKKVADALTNAVAHVDDMPNALSALSDLHAHK.L	6	2.73	0.11	-3.84
IPI00410714	Hemoglobin subunit alpha	K.KVADALTNAVAHVDDM*PNALSALSDLHAHK.L	3	6.35	0.40	
IPI00410714	Hemoglobin subunit alpha	K.KVADALTNAVAHVDDMPNALSALSDLHAHK.L	3	4.73	0.41	-3.38
IPI00410714	Hemoglobin subunit alpha	K.KVADALTNAVAHVDDMPNALSALSDLHAHK.L	4	6.05	0.44	-3.42
IPI00410714	Hemoglobin subunit alpha	K.KVADALTNAVAHVDDMPNALSALSDLHAHK.L	5	3.36	0.16	-1.77
IPI00410714	Hemoglobin subunit alpha	K.KVADALTNAVAHVDDMPNALSALSDLHAHKLR.V	3	6.04	0.46	-3.98
IPI00410714	Hemoglobin subunit alpha	K.KVADALTNAVAHVDDMPNALSALSDLHAHKLR.V	4	5.62	0.47	-1.59
IPI00410714	Hemoglobin subunit alpha	K.KVADALTNAVAHVDDMPNALSALSDLHAHKLR.V	5	2.45	0.13	-1.64
IPI00410714	Hemoglobin subunit alpha	K.LLSHCLLVTLAAHLPAEFTPAVHASLDK.F	4	4.87	0.46	-4.65
IPI00410714	Hemoglobin subunit alpha	K.TYFPHFDLSHGSAQVK.G	2	4.48	0.54	-3.19
IPI00410714	Hemoglobin subunit alpha	K.TYFPHFDLSHGSAQVK.G	3	2.97	0.35	-3.39
IPI00410714	Hemoglobin subunit alpha	K.TYFPHFDLSHGSAQVK.G	4	1.62	0.18	-3.11
IPI00410714	Hemoglobin subunit alpha	K.TYFPHFDLSHGSAQVKGHGKK.V	3	2.34	0.18	-1.21
IPI00410714	Hemoglobin subunit alpha	K.VADALTNAVAHVDDM*PNALSALSDLHAHK.L	2	5.41	0.52	
IPI00410714	Hemoglobin subunit alpha	K.VADALTNAVAHVDDM*PNALSALSDLHAHK.L	3	7.14	0.48	
IPI00410714	Hemoglobin subunit alpha	K.VADALTNAVAHVDDMPNALSALSDLHAHK.L	3	5.80	0.55	-3.90
IPI00410714	Hemoglobin subunit alpha	K.VADALTNAVAHVDDMPNALSALSDLHAHK.L	4	2.67	0.16	-3.16
IPI00410714	Hemoglobin subunit alpha	K.VADALTNAVAHVDDMPNALSALSDLHAHK.L	5	3.44	0.31	-2.88
IPI00410714	Hemoglobin subunit alpha	K.VGAHAGEYGAEALER.M	1	3.92	0.39	
IPI00410714	Hemoglobin subunit alpha	K.VGAHAGEYGAEALER.M	2	3.86	0.54	-4.08

IPI00410714	Hemoglobin subunit alpha	K.VGAHAGEYGAEALER.M	3	4.67	0.24	-2.78
IPI00410714	Hemoglobin subunit alpha	R.M*FLSFPTTK.T	1	2.23	0.08	-3.24
IPI00410714	Hemoglobin subunit alpha	R.M*FLSFPTTK.T	2	2.78	0.23	-1.48
IPI00410714	Hemoglobin subunit alpha	R.MFLSFPTTK.T	2	2.42	0.20	-3.37
IPI00411656	Isoform 1 of Protein piccolo	G.VTNGWTDSTVSQGITDGEVVDLSTTKSHR.T	3	3.65	0.10	-7.20
IPI00411656	Isoform 1 of Protein piccolo	K.ARHRPHGPLLPTIEDSSEEEELREEEELLK.E	3	1.53	0.11	-5.15
IPI00411656	Isoform 1 of Protein piccolo	K.DVVYK.N	1	1.46	0.08	-3.57
IPI00411674	Isoform 1 of Zinc finger protein 254	K.KRVKLFCM*LSHK.T	2	3.07	0.05	
IPI00411680	Isoform 1 of Protein-L-isoaspartate(D-aspartate) O-methyltransferase	K.ALDVGSGSGILTACFAR.M	2	5.06	0.61	-3.94
IPI00411680	Isoform 1 of Protein-L-isoaspartate(D-aspartate) O-methyltransferase	K.ALDVGSGSGILTACFAR.M	3	2.64	0.13	-2.64
IPI00411680	Isoform 1 of Protein-L-isoaspartate(D-aspartate) O-methyltransferase	K.DDPTLLSSGR.V	2	2.96	0.19	-0.14
IPI00411680	Isoform 1 of Protein-L-isoaspartate(D-aspartate) O-methyltransferase	K.ELVDDSINNVR.K	2	3.16	0.41	-2.15
IPI00411680	Isoform 1 of Protein-L-isoaspartate(D-aspartate) O-methyltransferase	K.M*KPLM*GVIYVPLTDKEK.Q	3	3.41	0.27	-2.63
IPI00411680	Isoform 1 of Protein-L-isoaspartate(D-aspartate) O-methyltransferase	K.TDKVFEVM*LATDR.S	2	3.01	0.30	-0.67
IPI00411680	Isoform 1 of Protein-L-isoaspartate(D-aspartate) O-methyltransferase	K.VFEVM*LATDR.S	2	2.78	0.35	-3.44
IPI00411680	Isoform 1 of Protein-L-isoaspartate(D-aspartate) O-methyltransferase	K.VIGIDHIK.E	1	1.56	0.18	-4.20
IPI00411680	Isoform 1 of Protein-L-isoaspartate(D-aspartate) O-methyltransferase	K.VIGIDHIKELVDDSINNVR.K	3	4.90	0.45	-2.06
IPI00411680	Isoform 1 of Protein-L-isoaspartate(D-aspartate) O-methyltransferase	K.VIGIDHIKELVDDSINNVRKDDPTLLSSGR.V	4	3.13	0.19	-3.66
IPI00411680	Isoform 1 of Protein-L-isoaspartate(D-aspartate) O-methyltransferase	R.KDDPTLLSSGR.V	2	2.59	0.25	-1.27
IPI00411680	Isoform 1 of Protein-L-isoaspartate(D-aspartate) O-methyltransferase	R.LILPVGPAGGNQM*LEQYDKLQDGSIK.M	3	2.38	0.39	-2.96
IPI00411680	Isoform 1 of Protein-L-isoaspartate(D-aspartate) O-methyltransferase	R.LILPVGPAGGNQM*LEQYDKLQDGSIK.M	4	3.91	0.34	-2.36
IPI00411680	Isoform 1 of Protein-L-isoaspartate(D-aspartate) O-methyltransferase	R.M*GYAEEAPYDAIHVGAAAPVVPQALIDQLKPGGR.L	3	6.33	0.56	-2.89
IPI00411680	Isoform 1 of Protein-L-isoaspartate(D-aspartate) O-methyltransferase	R.M*GYAEEAPYDAIHVGAAAPVVPQALIDQLKPGGR.L	4	5.06	0.37	-3.75
IPI00411706	S-formylglutathione hydrolase	K.SVSAFAPICNPVLCPWGK.K	2	3.99	0.43	-2.00
IPI00411706	S-formylglutathione hydrolase	R.M*YSYVTEELPQLINANFPVDPQR.M	3	3.87	0.32	-5.18
IPI00412216	vacuolar protein sorting 13C protein isoform 2B	R.LHLM*ASSGKMFKDGSMNVSVK.L	2	1.87	0.20	3.17
IPI00412264	Pleiotrophin precursor	K.KSDCGEWQWSVCVPTSGDCGLGTR.E	3	5.35	0.21	

IPI00412408	Breast cancer type 2 susceptibility protein	K.SLLYDHENASTLILTPTSK.D	3	3.68	0.08	
IPI00412492	Isoform 1 of Plexin-D1 precursor	K.LFTFDLNPSDDNILK.I	2	2.92	0.31	-4.22
IPI00412492	Isoform 1 of Plexin-D1 precursor	K.SQVFPLSLQLK.G	2	2.09	0.22	-3.10
IPI00412541	Probable G-protein coupled receptor 158 precursor	K.KLYAQLEIYK.R	2	2.23	0.12	
IPI00412987	GMFB protein	K.ETNNAAIIM*K.I	2	2.75	0.21	-2.35
IPI00412987	GMFB protein	K.NKLVQTAELTK.V	2	3.03	0.17	-0.79
IPI00412987	GMFB protein	R.LVQTAELTK.V	1	1.80	0.14	-2.97
IPI00412987	GMFB protein	R.LVQTAELTK.V	2	2.59	0.13	-2.12
IPI00412987	GMFB protein	R.LVVLDEELEGISPDELKDELPER.Q	3	3.50	0.30	-4.38
IPI00412987	GMFB protein	R.LVVLDEELEGISPDELKDELPERQPR.F	3	5.02	0.51	-2.14
IPI00412987	GMFB protein	R.LVVLDEELEGISPDELKDELPERQPR.F	4	3.25	0.25	-2.99
IPI00412988	Isoform 1 of Netrin-G1 precursor	K.LRDFFTVTDLR.I	3	2.82	0.11	-2.59
IPI00412988	Isoform 1 of Netrin-G1 precursor	R.CLCPAAYTGILCEK.L	2	4.21	0.48	-3.02
IPI00412988	Isoform 1 of Netrin-G1 precursor	R.DFFTVTDLR.I	2	2.07	0.23	-1.42
IPI00412988	Isoform 1 of Netrin-G1 precursor	R.FAFFAGPR.L	2	2.91	0.32	-1.49
IPI00412988	Isoform 1 of Netrin-G1 precursor	R.LLRPAVGEIFVDELHLAR.Y	2	3.37	0.36	-5.15
IPI00412988	Isoform 1 of Netrin-G1 precursor	R.LLRPAVGEIFVDELHLAR.Y	3	7.26	0.52	-4.05
IPI00412988	Isoform 1 of Netrin-G1 precursor	R.LLRPAVGEIFVDELHLAR.Y	4	4.89	0.44	-4.03
IPI00412988	Isoform 1 of Netrin-G1 precursor	R.NM*ASLYGGQLDTTK.K	2	3.69	0.44	-5.14
IPI00412988	Isoform 1 of Netrin-G1 precursor	R.NM*ASLYGGQLDTTKK.L	3	1.84	0.14	-0.80
IPI00413344	Cofilin-2	K.QILVGDIGDTVEDPYTSFVK.L	2	5.30	0.52	-5.18
IPI00413344	Cofilin-2	R.YALYDATYETK.E	2	3.27	0.30	-1.92
IPI00413451	Putative uncharacterized protein DKFZp686I04222	K.GNTAAQM*AQILSFNK.S	2	4.91	0.43	-3.01
IPI00413451	Putative uncharacterized protein DKFZp686I04222	K.IAELLSPGSVDPLTR.L	2	3.11	0.31	-3.82
IPI00413451	Putative uncharacterized protein DKFZp686I04222	K.SGGGGDIHQGFQSLLTEVNKTGTQYLLR.V	4	3.02	0.16	-4.32
IPI00413451	Putative uncharacterized protein DKFZp686I04222	R.LDM*MDEEEVEVSLPR.F	2	1.23	0.15	-2.86
IPI00413587	Isoform 1 of BH3-interacting domain death agonist	R.DLATALEQLLQAYPR.D	2	3.22	0.21	-3.51
IPI00413587	Isoform 1 of BH3-interacting domain death agonist	R.DLATALEQLLQAYPR.D	3	4.70	0.43	-2.08
IPI00413587	Isoform 1 of BH3-interacting domain death agonist	R.HLAQVGDSM*DR.S	3	1.95	0.14	-3.98
IPI00413641	Aldose reductase	K.REELFIVSK.L	2	2.46	0.06	-2.43
IPI00413641	Aldose reductase	K.SPPGQVTEAVK.V	2	3.13	0.13	-1.12
IPI00413778	Peptidyl-prolyl cis-trans isomerase	M.GVQVETISPGDGR.T	2	3.50	0.30	-3.68
IPI00413781	chemokine (C-X-C motif) ligand 12 (stromal cell-derived factor 1) isoform gamma	K.ILNTPNCALQIVAR.L	2	3.42	0.24	
IPI00413781	chemokine (C-X-C motif) ligand 12 (stromal cell-derived factor 1) isoform gamma	R.FFESHVAR.A	2	2.60	0.14	
IPI00413826	similar to H3 histone, family 3B	K.RVTIMPKDIQLAHSIR.G	2	2.71	0.06	
IPI00413912	Transmembrane protein 132E precursor	K.SVLATTPVGLR.V	2	2.06	0.16	-2.12
IPI00413912	Transmembrane protein 132E precursor	K.VVSPLTEAVLGETLLTVTEEK.V	3	4.39	0.38	-4.56
IPI00413912	Transmembrane protein 132E precursor	R.AVTELTVIQR.D	2	3.46	0.19	-2.97
IPI00413912	Transmembrane protein 132E precursor	R.DYGLLVSSLDEHVATVTQDR.A	3	3.73	0.39	-3.55

IPI00413912	Transmembrane protein 132E precursor	R.GHGALPDLER.A	2	2.62	0.25	-2.16
IPI00413959	Calsyntenin-1 precursor	D.PPLIALDKDAPLR.F	2	3.96	0.32	-4.13
IPI00413959	Calsyntenin-1 precursor	D.PPLIALDKDAPLR.F	3	3.80	0.38	-2.57
IPI00413959	Calsyntenin-1 precursor	I.PDGVVSVSPK.E	2	3.32	0.40	-1.49
IPI00413959	Calsyntenin-1 precursor	K.AM*QHISYLNSR.Q	2	3.52	0.37	-3.84
IPI00413959	Calsyntenin-1 precursor	K.AM*QHISYLNSR.Q	3	4.15	0.31	-4.11
IPI00413959	Calsyntenin-1 precursor	K.ATVHIQVNDVNEYAPVFK.E	2	5.28	0.56	-4.37
IPI00413959	Calsyntenin-1 precursor	K.ATVHIQVNDVNEYAPVFK.E	3	5.17	0.35	-5.40
IPI00413959	Calsyntenin-1 precursor	K.ATVHIQVNDVNEYAPVFKEK.S	2	5.80	0.50	-5.13
IPI00413959	Calsyntenin-1 precursor	K.ATVHIQVNDVNEYAPVFKEK.S	3	5.92	0.45	-4.24
IPI00413959	Calsyntenin-1 precursor	K.ATVHIQVNDVNEYAPVFKEK.S	4	4.80	0.36	-3.41
IPI00413959	Calsyntenin-1 precursor	K.CFNEATCISVPPVDGYVM*VLQPEEPK.I	3	2.47	0.07	-4.61
IPI00413959	Calsyntenin-1 precursor	K.CSELNGR.Y	1	1.91	0.16	-1.88
IPI00413959	Calsyntenin-1 precursor	K.DYSFTIQAYDCGK.G	2	4.68	0.55	-4.60
IPI00413959	Calsyntenin-1 precursor	K.DYSFTIQAYDCGKGPDGTNVKK.S	3	4.02	0.42	-0.92
IPI00413959	Calsyntenin-1 precursor	K.DYSFTIQAYDCGKGPDGTNVKK.S	4	2.73	0.35	-1.21
IPI00413959	Calsyntenin-1 precursor	K.EGLDLQVLEDSGR.G	1	1.82	0.18	-2.66
IPI00413959	Calsyntenin-1 precursor	K.EGLDLQVLEDSGR.G	2	4.86	0.49	-5.86
IPI00413959	Calsyntenin-1 precursor	K.EPFTISVWM*R.H	2	2.39	0.35	-4.43
IPI00413959	Calsyntenin-1 precursor	K.ETILCSSDKTDM*NR.H	2	3.90	0.45	-1.66
IPI00413959	Calsyntenin-1 precursor	K.ETILCSSDKTDM*NR.H	3	3.03	0.39	-0.98
IPI00413959	Calsyntenin-1 precursor	K.FKLICSELNGR.Y	3	3.78	0.17	-1.61
IPI00413959	Calsyntenin-1 precursor	K.GIEVSSSELGM*TFTGVDTM*ASYEEVLHLLR.Y	3	3.83	0.30	-3.11
IPI00413959	Calsyntenin-1 precursor	K.HKPWLEPTYHGIVTENDNTVLLDPPLIALDKDAPLR.F	4	3.99	0.34	-4.34
IPI00413959	Calsyntenin-1 precursor	K.IHGQNVPFDAVVVDK.S	2	4.98	0.50	-3.62
IPI00413959	Calsyntenin-1 precursor	K.IHGQNVPFDAVVVDK.S	3	3.29	0.29	-2.93
IPI00413959	Calsyntenin-1 precursor	K.IHGQNVPFDAVVVDKSTGEGVIR.S	2	5.01	0.53	-4.89
IPI00413959	Calsyntenin-1 precursor	K.IHGQNVPFDAVVVDKSTGEGVIR.S	3	5.74	0.52	-5.05
IPI00413959	Calsyntenin-1 precursor	K.IHGQNVPFDAVVVDKSTGEGVIR.S	4	4.24	0.43	-5.46
IPI00413959	Calsyntenin-1 precursor	K.ISLSGVHHFAR.A	2	3.16	0.25	-2.13
IPI00413959	Calsyntenin-1 precursor	K.LICSELNGR.Y	1	2.35	0.29	-2.78
IPI00413959	Calsyntenin-1 precursor	K.LICSELNGR.Y	2	2.51	0.22	2.09
IPI00413959	Calsyntenin-1 precursor	K.LNYGKEHQYK.L	1	2.45	0.27	-6.29
IPI00413959	Calsyntenin-1 precursor	K.LNYGKEHQYK.L	2	3.53	0.36	-4.17
IPI00413959	Calsyntenin-1 precursor	K.LTVTAYDCGK.K	1	2.13	0.40	-3.69
IPI00413959	Calsyntenin-1 precursor	K.LTVTAYDCGK.K	2	3.72	0.35	-2.63
IPI00413959	Calsyntenin-1 precursor	K.LTVTAYDCGKK.R	2	2.44	0.21	
IPI00413959	Calsyntenin-1 precursor	K.NTEKLNYGK.E	1	2.52	0.17	-2.09
IPI00413959	Calsyntenin-1 precursor	K.NTEKLNYGK.E	2	2.87	0.30	-1.43
IPI00413959	Calsyntenin-1 precursor	K.NTEKLNYGKEHQYK.L	2	3.65	0.38	-4.44
IPI00413959	Calsyntenin-1 precursor	K.NTEKLNYGKEHQYK.L	3	2.92	0.23	-3.48
IPI00413959	Calsyntenin-1 precursor	K.RATEDVLVK.I	2	2.42	0.09	-0.67

IPI00413959	Calsyntenin-1 precursor	K.STGEGVIR.S	1	1.48	0.05	-2.72
IPI00413959	Calsyntenin-1 precursor	K.STGEGVIR.S	2	2.16	0.07	-2.74
IPI00413959	Calsyntenin-1 precursor	K.SYKATVIEGK.Q	1	2.51	0.07	-1.49
IPI00413959	Calsyntenin-1 precursor	K.SYKATVIEGK.Q	2	2.66	0.17	-3.09
IPI00413959	Calsyntenin-1 precursor	K.VEVNVIHTANPM*EHANH.M	2	4.80	0.54	-3.09
IPI00413959	Calsyntenin-1 precursor	K.VIDCLYTCK.E	1	2.08	0.21	-3.75
IPI00413959	Calsyntenin-1 precursor	K.VIDCLYTCK.E	2	3.65	0.36	-3.48
IPI00413959	Calsyntenin-1 precursor	K.VIDCLYTCKEGLDLQVLEDSGR.G	2	4.80	0.45	-4.58
IPI00413959	Calsyntenin-1 precursor	K.VIDCLYTCKEGLDLQVLEDSGR.G	3	5.50	0.46	-2.92
IPI00413959	Calsyntenin-1 precursor	M.AAQPQFVHPEHR.S	2	3.19	0.37	-4.60
IPI00413959	Calsyntenin-1 precursor	P.DGVVSVSPKEPFTISVWM*R.H	2	3.29	0.26	-4.51
IPI00413959	Calsyntenin-1 precursor	P.DGVVSVSPKEPFTISVWM*R.H	3	3.93	0.41	-1.89
IPI00413959	Calsyntenin-1 precursor	Q.PQFVHPEHR.S	2	2.92	0.32	-3.59
IPI00413959	Calsyntenin-1 precursor	R.AASEFESSEGVFLFPELR.I	2	4.71	0.45	-8.90
IPI00413959	Calsyntenin-1 precursor	R.AASEFESSEGVFLFPELR.I	3	5.03	0.31	-4.67
IPI00413959	Calsyntenin-1 precursor	R.ATEDVLVK.I	1	2.30	0.10	-2.76
IPI00413959	Calsyntenin-1 precursor	R.ATEDVLVK.I	2	2.79	0.10	-2.54
IPI00413959	Calsyntenin-1 precursor	R.GNLAGLTLR.S	1	1.65	0.11	-1.41
IPI00413959	Calsyntenin-1 precursor	R.GNLAGLTLR.S	2	3.34	0.37	-2.13
IPI00413959	Calsyntenin-1 precursor	R.GVQIQAHPSQLVLTLEGEDLGELDK.A	2	5.85	0.47	-2.90
IPI00413959	Calsyntenin-1 precursor	R.GVQIQAHPSQLVLTLEGEDLGELDK.A	3	6.12	0.40	-5.09
IPI00413959	Calsyntenin-1 precursor	R.GVQIQAHPSQLVLTLEGEDLGELDK.A	4	5.78	0.39	-2.81
IPI00413959	Calsyntenin-1 precursor	R.GVQIQAHPSQLVLTLEGEDLGELDKAM*QHISYLNSR.Q	3	4.69	0.55	-3.96
IPI00413959	Calsyntenin-1 precursor	R.GVQIQAHPSQLVLTLEGEDLGELDKAM*QHISYLNSR.Q	4	8.33	0.54	-6.93
IPI00413959	Calsyntenin-1 precursor	R.GVQIQAHPSQLVLTLEGEDLGELDKAM*QHISYLNSR.Q	5	4.44	0.43	-5.15
IPI00413959	Calsyntenin-1 precursor	R.IEYEPGTGALAVFPNIHLETCDEPVASVQATVELETSHIGK.G	4	3.22	0.21	-3.28
IPI00413959	Calsyntenin-1 precursor	R.IPDGVVSVSPK.E	1	2.03	0.33	-3.87
IPI00413959	Calsyntenin-1 precursor	R.IPDGVVSVSPK.E	2	3.14	0.38	-2.96
IPI00413959	Calsyntenin-1 precursor	R.IPDGVVSVSPKEPF.T	2	4.05	0.47	-2.64
IPI00413959	Calsyntenin-1 precursor	R.IPDGVVSVSPKEPFTISVWM*R.H	3	4.68	0.55	-3.17
IPI00413959	Calsyntenin-1 precursor	R.LIFLFRQDPSEEKK.Y	2	2.48	0.10	-2.58
IPI00413959	Calsyntenin-1 precursor	R.LIFLFRQDPSEEKK.Y	3	3.12	0.18	-2.10
IPI00413959	Calsyntenin-1 precursor	R.LKITSTIK.C	1	2.09	0.21	-4.13
IPI00413959	Calsyntenin-1 precursor	R.SFVDLSGHNLANPHPF.A	2	4.17	0.48	-3.37
IPI00413959	Calsyntenin-1 precursor	R.SFVDLSGHNLANPHPF.A	3	4.84	0.50	-2.07
IPI00413959	Calsyntenin-1 precursor	R.SFVDLSGHNLANPHPFAVVPSTA.T	2	4.47	0.57	-3.64
IPI00413959	Calsyntenin-1 precursor	R.SFVDLSGHNLANPHPFAVVPSTAT.V	2	4.76	0.52	-4.11
IPI00413959	Calsyntenin-1 precursor	R.SFVDLSGHNLANPHPFAVVPSTATV.V	2	5.00	0.54	-3.24
IPI00413959	Calsyntenin-1 precursor	R.SFVDLSGHNLANPHPFAVVPSTATVV.I	2	3.98	0.37	-2.45
IPI00413959	Calsyntenin-1 precursor	R.SFVDLSGHNLANPHPFAVVPSTATVVI.V	2	4.17	0.38	-2.51
IPI00413959	Calsyntenin-1 precursor	R.VEAVDADCSPQFSQICSYEIITPDVPFTVDKDGYIK.N	3	5.78	0.61	-3.95
IPI00413959	Calsyntenin-1 precursor	R.VEAVDADCSPQFSQICSYEIITPDVPFTVDKDGYIK.N	4	4.54	0.37	-3.84

IPI00413959	Calsyntenin-1 precursor	R.VEAVDADCSPQFSQICSYEIITPDVPFTVDKDGYIKNTEK.L	4	5.79	0.35	-2.78
IPI00413959	Calsyntenin-1 precursor	R.YISNEFK.V	1	2.32	0.12	-1.62
IPI00413959	Calsyntenin-1 precursor	V.PFDAVVVDK.S	1	2.67	0.19	-5.98
IPI00413959	Calsyntenin-1 precursor	V.PFDAVVVDK.S	2	3.54	0.24	-1.24
IPI00413959	Calsyntenin-1 precursor	W.LEPTYHGIVTENDNTVLLDPPLIALDK.D	3	4.10	0.29	-4.31
IPI00413959	Calsyntenin-1 precursor	W.LEPTYHGIVTENDNTVLLDPPLIALDKDAPLR.F	3	6.13	0.53	-4.49
IPI00413959	Calsyntenin-1 precursor	W.LEPTYHGIVTENDNTVLLDPPLIALDKDAPLR.F	4	4.50	0.42	-3.77
IPI00414249	Isoform 1 of Neurexin-3-alpha precursor	A.PGLGDFLQLHIEQGK.I	2	3.08	0.35	-4.17
IPI00414249	Isoform 1 of Neurexin-3-alpha precursor	G.LEFM*GLPNQWAR.Y	2	4.31	0.34	-3.18
IPI00414249	Isoform 1 of Neurexin-3-alpha precursor	K.AREENVATFR.G	2	3.72	0.23	-2.99
IPI00414249	Isoform 1 of Neurexin-3-alpha precursor	K.CENVATLDPINFETPEAYISLPK.W	2	5.59	0.47	-5.72
IPI00414249	Isoform 1 of Neurexin-3-alpha precursor	K.DFRPNKVSETSR.T	2	1.82	0.13	-2.89
IPI00414249	Isoform 1 of Neurexin-3-alpha precursor	K.DGAVSLVINLGSGAFEAIVEPVNGK.F	3	3.16	0.21	-6.08
IPI00414249	Isoform 1 of Neurexin-3-alpha precursor	K.EVVYKNNDIR.L	2	2.17	0.11	-0.62
IPI00414249	Isoform 1 of Neurexin-3-alpha precursor	K.EVVYKNNDIR.L	3	2.10	0.14	-2.04
IPI00414249	Isoform 1 of Neurexin-3-alpha precursor	K.GDLYM*AGLAQGM*YSNLPK.L	2	5.40	0.57	-3.13
IPI00414249	Isoform 1 of Neurexin-3-alpha precursor	K.GDLYM*AGLAQGM*YSNLPK.L	3	3.88	0.45	-2.41
IPI00414249	Isoform 1 of Neurexin-3-alpha precursor	K.GPETLYAGQK.L	1	2.11	0.26	-1.68
IPI00414249	Isoform 1 of Neurexin-3-alpha precursor	K.GPETLYAGQK.L	2	2.94	0.21	0.06
IPI00414249	Isoform 1 of Neurexin-3-alpha precursor	K.GRLFQGQLSGLYYDGLK.V	3	4.55	0.43	-3.36
IPI00414249	Isoform 1 of Neurexin-3-alpha precursor	K.GYIHYVFDLGNGPNVIK.G	2	4.55	0.44	-1.43
IPI00414249	Isoform 1 of Neurexin-3-alpha precursor	K.GYIHYVFDLGNGPNVIK.G	3	3.88	0.22	-3.69
IPI00414249	Isoform 1 of Neurexin-3-alpha precursor	K.IIM*PM*VM*HTEAEDVSFR.F	2	4.07	0.50	-3.30
IPI00414249	Isoform 1 of Neurexin-3-alpha precursor	K.IIM*PM*VM*HTEAEDVSFR.F	3	2.09	0.32	-2.29
IPI00414249	Isoform 1 of Neurexin-3-alpha precursor	K.IYGEVVFK.C	1	2.06	0.22	-2.31
IPI00414249	Isoform 1 of Neurexin-3-alpha precursor	K.IYGEVVFK.C	2	1.55	0.06	-1.54
IPI00414249	Isoform 1 of Neurexin-3-alpha precursor	K.LCSEDVSQDPGLSH.L	2	4.45	0.58	-5.24
IPI00414249	Isoform 1 of Neurexin-3-alpha precursor	K.LCSEDVSQDPGLSHLM*.M	2	4.70	0.52	-3.39
IPI00414249	Isoform 1 of Neurexin-3-alpha precursor	K.LCSEDVSQDPGLSHLM*M*S.E	2	5.11	0.52	-4.39
IPI00414249	Isoform 1 of Neurexin-3-alpha precursor	K.LM*VNLDCIR.I	2	3.24	0.18	-1.80
IPI00414249	Isoform 1 of Neurexin-3-alpha precursor	K.NLDLKGDLYM*AGLAQGM*YSNLPK.L	2	4.64	0.51	-3.72
IPI00414249	Isoform 1 of Neurexin-3-alpha precursor	K.NLDLKGDLYM*AGLAQGM*YSNLPK.L	3	5.76	0.43	-3.66
IPI00414249	Isoform 1 of Neurexin-3-alpha precursor	K.SADYVNLALK.D	1	2.35	0.24	-3.84
IPI00414249	Isoform 1 of Neurexin-3-alpha precursor	K.SADYVNLALK.D	2	3.96	0.37	-1.89
IPI00414249	Isoform 1 of Neurexin-3-alpha precursor	K.SGGLILYTWPANDRPSTR.S	2	2.76	0.21	-3.81
IPI00414249	Isoform 1 of Neurexin-3-alpha precursor	K.SGGLILYTWPANDRPSTR.S	3	2.88	0.35	-3.79
IPI00414249	Isoform 1 of Neurexin-3-alpha precursor	K.TTSPDGFILFNSGDGNDFIAVELVK.G	2	4.16	0.50	-4.19
IPI00414249	Isoform 1 of Neurexin-3-alpha precursor	K.TTSPDGFILFNSGDGNDFIAVELVK.G	3	3.53	0.34	-4.27
IPI00414249	Isoform 1 of Neurexin-3-alpha precursor	K.VLNM*AAENNPNIK.I	2	4.53	0.36	-2.56
IPI00414249	Isoform 1 of Neurexin-3-alpha precursor	K.VLNM*AAENNPNIK.I	3	3.80	0.23	-0.76
IPI00414249	Isoform 1 of Neurexin-3-alpha precursor	K.VVTQVINGAK.N	1	2.30	0.15	-1.44
IPI00414249	Isoform 1 of Neurexin-3-alpha precursor	K.VVTQVINGAK.N	2	2.99	0.14	-2.87

IPI00414249	Isoform 1 of Neurexin-3-alpha precursor	K.YGNSEPR.L	2	1.92	0.25	-1.41
IPI00414249	Isoform 1 of Neurexin-3-alpha precursor	R.AYGLLVATTSR.D	1	2.21	0.33	-3.49
IPI00414249	Isoform 1 of Neurexin-3-alpha precursor	R.AYGLLVATTSR.D	2	4.29	0.40	-3.80
IPI00414249	Isoform 1 of Neurexin-3-alpha precursor	R.DGFQGCLASVDLNGR.L	2	4.89	0.57	-5.11
IPI00414249	Isoform 1 of Neurexin-3-alpha precursor	R.DGFQGCLASVDLNGRLPDLINDALHR.S	3	5.12	0.47	-2.78
IPI00414249	Isoform 1 of Neurexin-3-alpha precursor	R.DLFIDGR.S	1	2.11	0.13	-1.89
IPI00414249	Isoform 1 of Neurexin-3-alpha precursor	R.DNSNTHSLKVDTK.V	2	3.62	0.42	-3.87
IPI00414249	Isoform 1 of Neurexin-3-alpha precursor	R.DSADTLRLELDGGR.V	3	2.60	0.27	-2.05
IPI00414249	Isoform 1 of Neurexin-3-alpha precursor	R.DSADTLRLELDGGRVK.L	3	4.11	0.27	-1.81
IPI00414249	Isoform 1 of Neurexin-3-alpha precursor	R.EASILSYDGSM*YM*K.I	2	4.21	0.13	-1.99
IPI00414249	Isoform 1 of Neurexin-3-alpha precursor	R.EENVATFR.G	1	2.23	0.18	-3.61
IPI00414249	Isoform 1 of Neurexin-3-alpha precursor	R.EENVATFR.G	2	2.00	0.23	-3.05
IPI00414249	Isoform 1 of Neurexin-3-alpha precursor	R.FSM*DCAETAVLSNK.Q	2	4.81	0.52	-4.57
IPI00414249	Isoform 1 of Neurexin-3-alpha precursor	R.FSM*DCAETAVLSNK.Q	3	2.84	0.28	-3.73
IPI00414249	Isoform 1 of Neurexin-3-alpha precursor	R.GSEYLCYDLSQNPIQSSSDEITLSFK.T	2	4.47	0.54	-4.80
IPI00414249	Isoform 1 of Neurexin-3-alpha precursor	R.GVQM*DAEGPCGERPCENGGICFLLDGHPTCDCSTTGYGGK.L	4	4.67	0.45	-2.22
IPI00414249	Isoform 1 of Neurexin-3-alpha precursor	R.IDSAPGLGDFLQLHIEQGK.I	2	5.03	0.43	-2.90
IPI00414249	Isoform 1 of Neurexin-3-alpha precursor	R.IDSAPGLGDFLQLHIEQGK.I	3	2.74	0.34	-3.15
IPI00414249	Isoform 1 of Neurexin-3-alpha precursor	R.LAVGFSTTVK.D	2	2.73	0.28	-1.95
IPI00414249	Isoform 1 of Neurexin-3-alpha precursor	R.LAVGFSTTVKDGILVR.I	3	2.19	0.19	-2.27
IPI00414249	Isoform 1 of Neurexin-3-alpha precursor	R.LEFHNIETGIM*TEK.R	2	4.01	0.36	-2.47
IPI00414249	Isoform 1 of Neurexin-3-alpha precursor	R.LEFHNIETGIM*TEK.R	3	3.67	0.33	-3.05
IPI00414249	Isoform 1 of Neurexin-3-alpha precursor	R.LEFHNIETGIM*TEKR.Y	2	3.67	0.38	-3.02
IPI00414249	Isoform 1 of Neurexin-3-alpha precursor	R.LEFHNIETGIM*TEKR.Y	3	3.74	0.43	-2.30
IPI00414249	Isoform 1 of Neurexin-3-alpha precursor	R.LFQGQLSGLYYDGLK.V	2	5.21	0.39	-6.20
IPI00414249	Isoform 1 of Neurexin-3-alpha precursor	R.LFQGQLSGLYYDGLK.V	3	4.24	0.36	-3.34
IPI00414249	Isoform 1 of Neurexin-3-alpha precursor	R.LPDLINDALHR.S	2	3.78	0.36	-2.41
IPI00414249	Isoform 1 of Neurexin-3-alpha precursor	R.LPDLINDALHR.S	3	4.14	0.33	-2.11
IPI00414249	Isoform 1 of Neurexin-3-alpha precursor	R.M*GSISFDFR.T	1	1.31	0.22	-3.10
IPI00414249	Isoform 1 of Neurexin-3-alpha precursor	R.M*GSISFDFR.T	2	3.82	0.38	-2.50
IPI00414249	Isoform 1 of Neurexin-3-alpha precursor	R.NGLILHTGK.S	1	2.65	0.14	-3.65
IPI00414249	Isoform 1 of Neurexin-3-alpha precursor	R.NGLILHTGK.S	2	2.77	0.15	-1.89
IPI00414249	Isoform 1 of Neurexin-3-alpha precursor	R.NIIADPVTFK.T	1	2.97	0.31	-2.13
IPI00414249	Isoform 1 of Neurexin-3-alpha precursor	R.NIIADPVTFK.T	2	2.61	0.15	-2.52
IPI00414249	Isoform 1 of Neurexin-3-alpha precursor	R.QLAEM*QNAAGVK.S	1	1.24	0.11	-2.06
IPI00414249	Isoform 1 of Neurexin-3-alpha precursor	R.QLAEM*QNAAGVK.S	2	2.93	0.37	-3.09
IPI00414249	Isoform 1 of Neurexin-3-alpha precursor	R.QLTIFNTQAQIAIGGK.D	2	4.62	0.44	-6.95
IPI00414249	Isoform 1 of Neurexin-3-alpha precursor	R.QLTIFNTQAQIAIGGKDK.G	3	2.53	0.33	-4.21
IPI00414249	Isoform 1 of Neurexin-3-alpha precursor	R.RTPFTASGESEILDLEGDM*YLGGLPENR.A	3	3.70	0.29	-1.88
IPI00414249	Isoform 1 of Neurexin-3-alpha precursor	R.SDLSFQFK.T	1	2.63	0.17	-2.25
IPI00414249	Isoform 1 of Neurexin-3-alpha precursor	R.SDLSFQFK.T	2	2.55	0.09	-2.93
IPI00414249	Isoform 1 of Neurexin-3-alpha precursor	R.SGTISVNSR.R	1	2.12	0.18	-1.75

IPI00414249	Isoform 1 of Neurexin-3-alpha precursor	R.SGTISVNSR.R	2	2.13	0.07	-0.95
IPI00414249	Isoform 1 of Neurexin-3-alpha precursor	R.TPFTASGESEILDLEGDM*YLGGLPENR.A	2	4.48	0.55	-4.54
IPI00414249	Isoform 1 of Neurexin-3-alpha precursor	R.TPFTASGESEILDLEGDM*YLGGLPENR.A	3	4.09	0.37	-4.17
IPI00414249	Isoform 1 of Neurexin-3-alpha precursor	R.TPVNDGKYHVVR.F	2	3.84	0.11	-4.34
IPI00414249	Isoform 1 of Neurexin-3-alpha precursor	R.TTEPNGLILFTHGKPQER.K	2	3.21	0.37	-3.92
IPI00414249	Isoform 1 of Neurexin-3-alpha precursor	R.TTEPNGLILFTHGKPQER.K	3	2.43	0.09	-0.33
IPI00414249	Isoform 1 of Neurexin-3-alpha precursor	R.TTEPNGLILFTHGKPQER.K	4	3.21	0.32	-0.66
IPI00414249	Isoform 1 of Neurexin-3-alpha precursor	R.VPGASEVIR.E	2	1.86	0.06	-2.21
IPI00414249	Isoform 1 of Neurexin-3-alpha precursor	S.EQAREENVATFR.G	2	3.29	0.31	-3.60
IPI00414294	hypothetical protein	R.LAQASCSNYDTPR.W	2	4.22	0.53	-3.12
IPI00414294	hypothetical protein	R.TGPAGGAGAAAR.A	2	3.10	0.38	-1.44
IPI00414320	Annexin A11	R.DAQELYAAGENR.L	2	3.57	0.34	-2.68
IPI00414467	collectin sub-family member 12	K.CYYFSVEK.E	1	1.81	0.23	-3.04
IPI00414467	collectin sub-family member 12	K.CYYFSVEK.E	2	2.13	0.24	0.21
IPI00414467	collectin sub-family member 12	K.EIFEDAK.L	2	1.93	0.12	-3.49
IPI00414467	collectin sub-family member 12	K.LFCEDKSSHLVFINTR.E	3	4.63	0.38	-4.05
IPI00414467	collectin sub-family member 12	K.LFCEDKSSHLVFINTR.E	4	3.26	0.37	-2.58
IPI00414467	collectin sub-family member 12	K.SSHLVFINTR.E	1	2.55	0.24	-4.09
IPI00414467	collectin sub-family member 12	K.SSHLVFINTR.E	2	2.82	0.22	-3.56
IPI00414467	collectin sub-family member 12	K.WLDGTSPDYK.N	2	2.35	0.27	-3.52
IPI00414481	GTF3C1 protein	R.TIKQESGRAGVRPSSSGSAWEACSEAPSK.G	3	2.97	0.07	-4.68
IPI00414676	Heat shock protein HSP 90-beta	K.DLVVLLFETALLSSGFSLEDPQTHSNR.I	3	4.66	0.53	-4.51
IPI00414676	Heat shock protein HSP 90-beta	K.YIDQEELNK.T	2	3.04	0.15	-2.82
IPI00414676	Heat shock protein HSP 90-beta	R.DNSTM*GYM*M*AK.K	2	2.95	0.41	-2.80
IPI00414717	golgi apparatus protein 1	K.ADIFVDPVLHTACALDIK.H	3	3.51	0.33	-3.36
IPI00414717	golgi apparatus protein 1	R.FCENTQAGEGR.V	2	3.45	0.39	-2.07
IPI00414717	golgi apparatus protein 1	R.LLELQYFISR.D	2	3.39	0.24	-3.21
IPI00414717	golgi apparatus protein 1	R.VAELSSDDFHLDR.H	2	4.26	0.40	-3.09
IPI00414717	golgi apparatus protein 1	R.VAELSSDDFHLDR.H	3	2.65	0.20	-1.79
IPI00414896	Isoform 1 of Ribonuclease T2 precursor	K.LGIKPSINYYQVADFK.D	2	4.03	0.43	-4.20
IPI00414896	Isoform 1 of Ribonuclease T2 precursor	K.LGIKPSINYYQVADFK.D	3	5.64	0.42	-1.98
IPI00414896	Isoform 1 of Ribonuclease T2 precursor	K.LGIKPSINYYQVADFKDALAR.V	2	5.49	0.60	-4.60
IPI00414896	Isoform 1 of Ribonuclease T2 precursor	K.LGIKPSINYYQVADFKDALAR.V	3	5.34	0.54	-4.06
IPI00414896	Isoform 1 of Ribonuclease T2 precursor	K.LGIKPSINYYQVADFKDALAR.V	4	5.22	0.37	-6.77
IPI00414896	Isoform 1 of Ribonuclease T2 precursor	K.QEVWLANGAAESR.G	2	3.43	0.36	-3.11
IPI00414896	Isoform 1 of Ribonuclease T2 precursor	R.ELDLNSVLLK.L	1	2.46	0.14	-1.00
IPI00414896	Isoform 1 of Ribonuclease T2 precursor	R.ELDLNSVLLK.L	2	2.81	0.12	-3.59
IPI00414896	Isoform 1 of Ribonuclease T2 precursor	R.SLELYRELDLNSVLLK.L	3	3.49	0.20	-1.97
IPI00414896	Isoform 1 of Ribonuclease T2 precursor	R.VCEDGPVFYPPPK.K	2	3.28	0.38	-3.98
IPI00414896	Isoform 1 of Ribonuclease T2 precursor	R.VYGVIPK.I	1	2.01	0.11	-2.76
IPI00414896	Isoform 1 of Ribonuclease T2 precursor	W.LANGAAESR.G	2	3.01	0.23	-3.23
IPI00414896	Isoform 1 of Ribonuclease T2 precursor	W.PFNLEEIKDLLPEM*R.A	2	3.54	0.40	-2.12

IPI00414896	Isoform 1 of Ribonuclease T2 precursor	W.PFNLEEIKDLLPEM*R.A	3	4.90	0.32	-2.97
IPI00414909	Alpha-N-acetylgalactosaminidase precursor	K.ASALVFFSCR.T	2	2.87	0.19	-0.62
IPI00414909	Alpha-N-acetylgalactosaminidase precursor	K.INQDPLGIQGR.R	2	3.48	0.34	-3.26
IPI00414909	Alpha-N-acetylgalactosaminidase precursor	R.AQM*ALWTVLAAPLLM*STDLR.T	3	4.97	0.43	-5.17
IPI00414909	Alpha-N-acetylgalactosaminidase precursor	R.TISAQNM*DILQNPLM*IK.I	2	4.49	0.36	-4.05
IPI00414984	sarcoglycan, epsilon isoform 1	K.ISLVDKTK.Q	1	2.27	0.16	-3.22
IPI00414984	sarcoglycan, epsilon isoform 1	K.ISLVDKTK.Q	2	2.52	0.12	-3.63
IPI00414984	sarcoglycan, epsilon isoform 1	K.NM*NVEEM*LASEVLGDFLGAVK.N	2	6.15	0.56	-3.13
IPI00414984	sarcoglycan, epsilon isoform 1	K.NM*NVEEM*LASEVLGDFLGAVK.N	3	5.67	0.40	-3.85
IPI00414984	sarcoglycan, epsilon isoform 1	K.QVSTYQEVIR.G	2	2.69	0.21	-2.15
IPI00414984	sarcoglycan, epsilon isoform 1	K.QVSTYQEVIRGEGILPDGGEYKPPSDS.L	3	5.41	0.34	-3.14
IPI00414984	sarcoglycan, epsilon isoform 1	R.EVENPQNQLR.C	2	2.47	0.11	-3.62
IPI00414984	sarcoglycan, epsilon isoform 1	R.GEGILPDGGEYKPPSDS.L	2	3.75	0.44	-2.85
IPI00414984	sarcoglycan, epsilon isoform 1	R.GGRVPLPINDLK.E	2	2.75	0.18	-2.10
IPI00414984	sarcoglycan, epsilon isoform 1	R.TPYSDGVLYGSPTAENVGKPTIIEITAYNR.R	3	4.87	0.45	-3.60
IPI00414984	sarcoglycan, epsilon isoform 1	R.TPYSDGVLYGSPTAENVGKPTIIEITAYNRR.T	3	5.27	0.60	-2.93
IPI00414984	sarcoglycan, epsilon isoform 1	R.TPYSDGVLYGSPTAENVGKPTIIEITAYNRR.T	4	5.17	0.49	-3.60
IPI00414984	sarcoglycan, epsilon isoform 1	R.TQFYIDWCK.I	1	2.32	0.32	-0.67
IPI00414984	sarcoglycan, epsilon isoform 1	R.TQFYIDWCK.I	2	3.22	0.36	-3.07
IPI00414984	sarcoglycan, epsilon isoform 1	R.VPLPINDLK.E	2	2.16	0.07	-1.13
IPI00414984	sarcoglycan, epsilon isoform 1	S.DRNVYPSAGVLFVHVLER.E	2	3.82	0.43	-5.30
IPI00414984	sarcoglycan, epsilon isoform 1	S.DRNVYPSAGVLFVHVLER.E	3	6.28	0.41	-6.24
IPI00415032	Isoform 4 of Neuronal cell adhesion molecule precursor	A.LEVPLDLVQPPTITQQSPK.D	2	5.58	0.48	-4.99
IPI00415032	Isoform 4 of Neuronal cell adhesion molecule precursor	A.LEVPLDLVQPPTITQQSPK.D	3	4.38	0.25	-3.82
IPI00415032	Isoform 4 of Neuronal cell adhesion molecule precursor	A.LEVPLDLVQPPTITQQSPKDYIIDPR.E	2	3.02	0.33	-3.83
IPI00415032	Isoform 4 of Neuronal cell adhesion molecule precursor	A.LEVPLDLVQPPTITQQSPKDYIIDPR.E	3	4.87	0.37	-4.71
IPI00415032	Isoform 4 of Neuronal cell adhesion molecule precursor	E.PFSHYTLNVR.V	2	3.34	0.37	-2.69
IPI00415032	Isoform 4 of Neuronal cell adhesion molecule precursor	H.HQTEVSGTQTTAQLK.L	2	4.78	0.55	-3.18
IPI00415032	Isoform 4 of Neuronal cell adhesion molecule precursor	K.AAPYWITAPQNLVLSPGEDGTLICR.A	2	5.33	0.54	-5.00
IPI00415032	Isoform 4 of Neuronal cell adhesion molecule precursor	K.AAPYWITAPQNLVLSPGEDGTLICR.A	3	6.05	0.45	-4.91
IPI00415032	Isoform 4 of Neuronal cell adhesion molecule precursor	K.AAPYWITAPQNLVLSPGEDGTLICR.A	4	4.08	0.32	-4.52
IPI00415032	Isoform 4 of Neuronal cell adhesion molecule precursor	K.AETYEGVYQCTAR.N	2	4.85	0.53	-3.95

IPI00415032	Isoform 4 of Neuronal cell adhesion molecule precursor	K.ASEPDKNPTAVEGLGSEPDNLVITWKPLNGFESNGPGLQYK.V	3	6.19	0.51	-1.26
IPI00415032	Isoform 4 of Neuronal cell adhesion molecule precursor	K.ASEPDKNPTAVEGLGSEPDNLVITWKPLNGFESNGPGLQYK.V	4	5.14	0.41	-3.83
IPI00415032	Isoform 4 of Neuronal cell adhesion molecule precursor	K.DNRELPSDER.F	2	2.26	0.08	-1.16
IPI00415032	Isoform 4 of Neuronal cell adhesion molecule precursor	K.DSTGTYTCVAR.N	1	2.43	0.41	-2.36
IPI00415032	Isoform 4 of Neuronal cell adhesion molecule precursor	K.DSTGTYTCVAR.N	2	4.21	0.47	-4.76
IPI00415032	Isoform 4 of Neuronal cell adhesion molecule precursor	K.EDGM*LPK.N	1	1.38	0.11	-1.82
IPI00415032	Isoform 4 of Neuronal cell adhesion molecule precursor	K.EELRGNVLSLECIAEGLPTPIIYWAK.E	3	4.42	0.34	-5.09
IPI00415032	Isoform 4 of Neuronal cell adhesion molecule precursor	K.EKLEPITLQSGQSLVLPCRPPIGLPPPIIFWM*DNSFQR.L	3	4.40	0.55	-0.96
IPI00415032	Isoform 4 of Neuronal cell adhesion molecule precursor	K.EKLEPITLQSGQSLVLPCRPPIGLPPPIIFWM*DNSFQR.L	4	6.08	0.49	-4.94
IPI00415032	Isoform 4 of Neuronal cell adhesion molecule precursor	K.EKLEPITLQSGQSLVLPCRPPIGLPPPIIFWM*DNSFQR.L	5	3.82	0.17	-0.45
IPI00415032	Isoform 4 of Neuronal cell adhesion molecule precursor	K.FIIEYEDAM*HKPGLWHHQTEVSGTQTTAQLK.L	3	6.10	0.54	-5.04
IPI00415032	Isoform 4 of Neuronal cell adhesion molecule precursor	K.FIIEYEDAM*HKPGLWHHQTEVSGTQTTAQLK.L	4	5.77	0.46	-4.49
IPI00415032	Isoform 4 of Neuronal cell adhesion molecule precursor	K.GEGPASPDRVFNTPEGVPSAPSSLK.I	2	3.70	0.41	-3.01
IPI00415032	Isoform 4 of Neuronal cell adhesion molecule precursor	K.GEGPASPDRVFNTPEGVPSAPSSLK.I	3	4.79	0.43	-2.81
IPI00415032	Isoform 4 of Neuronal cell adhesion molecule precursor	K.IDGDTIIFSNVQER.S	2	5.23	0.46	-3.56
IPI00415032	Isoform 4 of Neuronal cell adhesion molecule precursor	K.IDGDTIIFSNVQER.S	3	5.17	0.30	-0.47
IPI00415032	Isoform 4 of Neuronal cell adhesion molecule precursor	K.ILTFQGSK.T	2	2.22	0.13	-2.25
IPI00415032	Isoform 4 of Neuronal cell adhesion molecule precursor	K.IVNPTLDSLTLEWDPPSHPNGILTEYTLK.Y	2	4.84	0.53	-3.73
IPI00415032	Isoform 4 of Neuronal cell adhesion molecule precursor	K.IVNPTLDSLTLEWDPPSHPNGILTEYTLK.Y	3	5.21	0.51	-4.58
IPI00415032	Isoform 4 of Neuronal cell adhesion molecule precursor	K.IVNPTLDSLTLEWDPPSHPNGILTEYTLK.Y	4	4.06	0.30	-3.90
IPI00415032	Isoform 4 of Neuronal cell adhesion molecule precursor	K.KILTFQGSK.T	1	2.83	0.24	-4.92

IPI00415032	Isoform 4 of Neuronal cell adhesion molecule precursor	K.LSPYVNYSFR.V	1	2.03	0.14	-2.76
IPI00415032	Isoform 4 of Neuronal cell adhesion molecule precursor	K.LSPYVNYSFR.V	2	3.02	0.41	-3.03
IPI00415032	Isoform 4 of Neuronal cell adhesion molecule precursor	K.NEVHLEIK.D	1	2.25	0.16	-4.23
IPI00415032	Isoform 4 of Neuronal cell adhesion molecule precursor	K.NEVHLEIK.D	2	3.03	0.17	-1.47
IPI00415032	Isoform 4 of Neuronal cell adhesion molecule precursor	K.NEVHLEIKDPTWIVK.Q	3	3.65	0.30	-3.36
IPI00415032	Isoform 4 of Neuronal cell adhesion molecule precursor	K.NLNFSTR.Y	2	2.17	0.14	-2.56
IPI00415032	Isoform 4 of Neuronal cell adhesion molecule precursor	K.PLNGFESNGPGLQYK.V	3	3.53	0.23	-2.06
IPI00415032	Isoform 4 of Neuronal cell adhesion molecule precursor	K.QPEYAVVQR.G	1	2.25	0.34	-3.31
IPI00415032	Isoform 4 of Neuronal cell adhesion molecule precursor	K.QPEYAVVQR.G	2	1.99	0.24	-2.06
IPI00415032	Isoform 4 of Neuronal cell adhesion molecule precursor	K.SLPSEASEQYLTK.A	1	2.36	0.33	-2.91
IPI00415032	Isoform 4 of Neuronal cell adhesion molecule precursor	K.SLPSEASEQYLTK.A	2	4.46	0.39	-6.04
IPI00415032	Isoform 4 of Neuronal cell adhesion molecule precursor	K.SLPSEASEQYLTK.A	3	3.29	0.34	-2.28
IPI00415032	Isoform 4 of Neuronal cell adhesion molecule precursor	K.SVQLSWTPGDDNNSPITK.F	2	5.19	0.57	-4.24
IPI00415032	Isoform 4 of Neuronal cell adhesion molecule precursor	K.THGM*LPGLEPFSHYTLNVR.V	2	4.97	0.56	-5.68
IPI00415032	Isoform 4 of Neuronal cell adhesion molecule precursor	K.THGM*LPGLEPFSHYTLNVR.V	3	4.27	0.47	-3.70
IPI00415032	Isoform 4 of Neuronal cell adhesion molecule precursor	K.TLQIIHVSEADSGNYQCIAK.N	2	5.96	0.62	-3.27
IPI00415032	Isoform 4 of Neuronal cell adhesion molecule precursor	K.TLQIIHVSEADSGNYQCIAK.N	3	5.97	0.45	-4.53
IPI00415032	Isoform 4 of Neuronal cell adhesion molecule precursor	K.VQALNDM*GFAPEPAVVM*GHSGEDLPM*VAPGNVR.V	3	5.08	0.55	-4.04
IPI00415032	Isoform 4 of Neuronal cell adhesion molecule precursor	K.VQALNDM*GFAPEPAVVM*GHSGEDLPM*VAPGNVR.V	4	3.53	0.27	-4.19
IPI00415032	Isoform 4 of Neuronal cell adhesion molecule precursor	K.YIVSGTPTFVPYLIK.V	1	3.11	0.41	-1.34
IPI00415032	Isoform 4 of Neuronal cell adhesion molecule precursor	K.YIVSGTPTFVPYLIK.V	2	5.14	0.51	-5.26

IPI00415032	Isoform 4 of Neuronal cell adhesion molecule precursor	K.YIVSGTPTFVPYLIK.V	3	3.67	0.26	-3.52
IPI00415032	Isoform 4 of Neuronal cell adhesion molecule precursor	L.PSEASEQYLTK.A	2	3.75	0.44	-2.53
IPI00415032	Isoform 4 of Neuronal cell adhesion molecule precursor	L.TNGVPIEIAPDDPSR.K	2	4.14	0.45	-4.34
IPI00415032	Isoform 4 of Neuronal cell adhesion molecule precursor	P.GLEPFSHYTLNVR.V	2	3.39	0.30	-2.09
IPI00415032	Isoform 4 of Neuronal cell adhesion molecule precursor	Q.PPTITQQSPKDYIIDPR.E	2	3.53	0.47	-4.33
IPI00415032	Isoform 4 of Neuronal cell adhesion molecule precursor	R.EDYICYAR.F	2	1.83	0.08	-2.72
IPI00415032	Isoform 4 of Neuronal cell adhesion molecule precursor	R.ENIVIQCEAK.G	1	2.94	0.17	-3.05
IPI00415032	Isoform 4 of Neuronal cell adhesion molecule precursor	R.ENIVIQCEAK.G	2	3.30	0.22	-2.91
IPI00415032	Isoform 4 of Neuronal cell adhesion molecule precursor	R.ERPPTFLTPEGNASNK.E	2	3.21	0.21	-3.57
IPI00415032	Isoform 4 of Neuronal cell adhesion molecule precursor	R.ERPPTFLTPEGNASNKEELR.G	2	4.57	0.36	-5.17
IPI00415032	Isoform 4 of Neuronal cell adhesion molecule precursor	R.ERPPTFLTPEGNASNKEELR.G	3	5.10	0.47	-3.44
IPI00415032	Isoform 4 of Neuronal cell adhesion molecule precursor	R.ERPPTFLTPEGNASNKEELRGNVLSLECIAEGLPTPIIYWAK.E	4	4.38	0.32	-4.01
IPI00415032	Isoform 4 of Neuronal cell adhesion molecule precursor	R.GAAVSNNIVVRPSR.S	2	2.63	0.14	-4.64
IPI00415032	Isoform 4 of Neuronal cell adhesion molecule precursor	R.GAAVSNNIVVRPSR.S	3	3.00	0.35	-3.92
IPI00415032	Isoform 4 of Neuronal cell adhesion molecule precursor	R.GHLQGYR.I	1	1.87	0.08	-5.06
IPI00415032	Isoform 4 of Neuronal cell adhesion molecule precursor	R.GNVLSLECIAEGLPTPIIYWAK.E	2	5.21	0.54	-5.18
IPI00415032	Isoform 4 of Neuronal cell adhesion molecule precursor	R.GNVLSLECIAEGLPTPIIYWAK.E	3	5.02	0.39	-5.31
IPI00415032	Isoform 4 of Neuronal cell adhesion molecule precursor	R.GNVLSLECIAEGLPTPIIYWAKEDGM*LPK.N	3	3.52	0.34	-4.35
IPI00415032	Isoform 4 of Neuronal cell adhesion molecule precursor	R.GSM*VSFECK.V	1	2.54	0.21	-3.09
IPI00415032	Isoform 4 of Neuronal cell adhesion molecule precursor	R.GSM*VSFECK.V	2	2.97	0.26	-1.60
IPI00415032	Isoform 4 of Neuronal cell adhesion molecule precursor	R.ILTPANTLYQVIANR.P	2	4.08	0.48	-3.49

IPI00415032	Isoform 4 of Neuronal cell adhesion molecule precursor	R.ISWLTNGVPIEIAPDDPSR.K	2	4.97	0.37	-3.38
IPI00415032	Isoform 4 of Neuronal cell adhesion molecule precursor	R.ISWLTNGVPIEIAPDDPSR.K	3	3.57	0.06	-3.74
IPI00415032	Isoform 4 of Neuronal cell adhesion molecule precursor	R.ISWLTNGVPIEIAPDDPSRK.I	2	3.68	0.36	-1.53
IPI00415032	Isoform 4 of Neuronal cell adhesion molecule precursor	R.ISWLTNGVPIEIAPDDPSRK.I	3	5.40	0.40	-2.54
IPI00415032	Isoform 4 of Neuronal cell adhesion molecule precursor	R.KIDGDTIIFSNVQER.S	2	5.34	0.51	-5.43
IPI00415032	Isoform 4 of Neuronal cell adhesion molecule precursor	R.KIDGDTIIFSNVQER.S	3	6.06	0.36	-4.35
IPI00415032	Isoform 4 of Neuronal cell adhesion molecule precursor	R.TVYKNFEK.T	1	1.90	0.08	-3.71
IPI00415032	Isoform 4 of Neuronal cell adhesion molecule precursor	R.TVYKNFEK.T	2	2.31	0.15	-1.93
IPI00415032	Isoform 4 of Neuronal cell adhesion molecule precursor	R.VFNTPEGVPSAPSSLK.I	2	4.62	0.44	-5.50
IPI00415032	Isoform 4 of Neuronal cell adhesion molecule precursor	R.VFNTPEGVPSAPSSLK.I	3	2.99	0.11	-2.34
IPI00415032	Isoform 4 of Neuronal cell adhesion molecule precursor	R.VKAAPYWITAPQNLVLSPGEDGTLICR.A	3	5.92	0.48	-2.80
IPI00415032	Isoform 4 of Neuronal cell adhesion molecule precursor	R.VKAAPYWITAPQNLVLSPGEDGTLICR.A	4	2.50	0.11	-3.10
IPI00415032	Isoform 4 of Neuronal cell adhesion molecule precursor	R.VM*AVNSIGK.S	1	2.43	0.18	-3.09
IPI00415032	Isoform 4 of Neuronal cell adhesion molecule precursor	R.VM*AVNSIGK.S	2	2.77	0.20	-2.73
IPI00415032	Isoform 4 of Neuronal cell adhesion molecule precursor	R.VSQGLNGDLYFSNVLPEDTR.E	2	5.50	0.51	-3.83
IPI00415032	Isoform 4 of Neuronal cell adhesion molecule precursor	R.VSQGLNGDLYFSNVLPEDTR.E	3	4.38	0.24	-2.49
IPI00415032	Isoform 4 of Neuronal cell adhesion molecule precursor	R.VSQGLNGDLYFSNVLPEDTREDYICYAR.F	2	1.61	0.09	-3.71
IPI00415032	Isoform 4 of Neuronal cell adhesion molecule precursor	R.VSQGLNGDLYFSNVLPEDTREDYICYAR.F	3	4.89	0.46	-6.46
IPI00415032	Isoform 4 of Neuronal cell adhesion molecule precursor	R.VSQGLNGDLYFSNVLPEDTREDYICYAR.F	4	3.87	0.29	-5.93
IPI00415032	Isoform 4 of Neuronal cell adhesion molecule precursor	R.VVNGKGEGPASPDR.V	2	3.98	0.47	-4.17
IPI00415032	Isoform 4 of Neuronal cell adhesion molecule precursor	R.VVNGKGEGPASPDR.V	3	3.11	0.41	-3.22

IPI00415032	Isoform 4 of Neuronal cell adhesion molecule precursor	R.VVNGKGEGPASPDRVFNTPEGVPSAPSSLK.I	2	3.50	0.52	-3.25
IPI00415032	Isoform 4 of Neuronal cell adhesion molecule precursor	R.VVNGKGEGPASPDRVFNTPEGVPSAPSSLK.I	3	4.69	0.46	-4.11
IPI00415032	Isoform 4 of Neuronal cell adhesion molecule precursor	S.PYVNYSFR.V	2	3.17	0.25	-2.62
IPI00415032	Isoform 4 of Neuronal cell adhesion molecule precursor	T.PGDDNNSPITK.F	2	3.60	0.43	-2.53
IPI00415032	Isoform 4 of Neuronal cell adhesion molecule precursor	W.HHQTEVSGTQTTAQLK.L	2	4.57	0.48	-4.41
IPI00415032	Isoform 4 of Neuronal cell adhesion molecule precursor	W.HHQTEVSGTQTTAQLK.L	3	4.01	0.31	-3.60
IPI00415032	Isoform 4 of Neuronal cell adhesion molecule precursor	W.ITAPQNLVLSPGEDGTLICR.A	2	4.37	0.47	-5.49
IPI00415032	Isoform 4 of Neuronal cell adhesion molecule precursor	W.ITAPQNLVLSPGEDGTLICR.A	3	4.47	0.40	-4.79
IPI00415032	Isoform 4 of Neuronal cell adhesion molecule precursor	W.LTNGVPIEIAPDDPSR.K	2	4.35	0.48	-3.90
IPI00415032	Isoform 4 of Neuronal cell adhesion molecule precursor	W.TPGDDNNSPITK.F	2	3.21	0.43	-0.47
IPI00418125	Isoform 1 of Layilin precursor	K.FIENLLPSDGDFWIGLR.R	2	4.48	0.42	-5.40
IPI00418163	C4B1	A.PFLLQALVR.E	2	3.53	0.25	-4.36
IPI00418163	C4B1	A.PKVVEEQESR.V	2	3.04	0.29	-1.03
IPI00418163	C4B1	D.DPDAPLQPVTPLQLFEGR.R	2	3.34	0.40	-3.65
IPI00418163	C4B1	D.DPDAPLQPVTPLQLFEGR.R	3	4.88	0.46	-2.11
IPI00418163	C4B1	D.HAVDLIQK.G	2	2.94	0.27	-0.14
IPI00418163	C4B1	D.PLDTLGSEGALSPGGVASLLR.L	2	5.16	0.52	-3.43
IPI00418163	C4B1	D.PLDTLGSEGALSPGGVASLLR.L	3	5.06	0.42	-2.98
IPI00418163	C4B1	E.APKVVEEQESR.V	2	3.24	0.37	-3.22
IPI00418163	C4B1	E.APKVVEEQESR.V	3	3.64	0.30	-2.42
IPI00418163	C4B1	F.LSCCQFAESLR.K	2	3.54	0.35	-2.59
IPI00418163	C4B1	K.ADGSYAAWLSR.D	2	3.01	0.24	
IPI00418163	C4B1	K.AEFQDALEK.L	1	2.52	0.13	-4.21
IPI00418163	C4B1	K.AEFQDALEK.L	2	3.23	0.16	-3.14
IPI00418163	C4B1	K.AEFQDALEKLNM*GITDLQGLR.L	2	4.80	0.57	-3.22
IPI00418163	C4B1	K.AEFQDALEKLNM*GITDLQGLR.L	3	6.42	0.49	-5.12
IPI00418163	C4B1	K.AEFQDALEKLNM*GITDLQGLR.L	4	4.59	0.32	-2.23
IPI00418163	C4B1	K.AEFQDALEKLNMGITDLQGLR.L	2	3.83	0.32	
IPI00418163	C4B1	K.AEFQDALEKLNMGITDLQGLR.L	3	5.14	0.35	-4.61
IPI00418163	C4B1	K.AEM*ADQAAAWLTR.Q	2	4.00	0.29	-3.79
IPI00418163	C4B1	K.AEMADQAAAWLTR.Q	2	3.56	0.19	
IPI00418163	C4B1	K.ASAGLLGAHAAAITAYALTLTK.A	2	6.12	0.64	-3.89

IPI00418163	C4B1	K.ASAGLLGAHAAAITAYALTLTK.A	3	4.59	0.52	-5.32
IPI00418163	C4B1	K.ASAGLLGAHAAAITAYALTLTK.A	4	3.88	0.45	-0.90
IPI00418163	C4B1	K.ASAGLLGAHAAAITAYALTLTKAPADLR.G	3	5.69	0.56	-5.20
IPI00418163	C4B1	K.ASAGLLGAHAAAITAYALTLTKAPADLR.G	4	4.41	0.46	-4.73
IPI00418163	C4B1	K.ASSFLGEK.A	1	1.79	0.13	-4.90
IPI00418163	C4B1	K.ASSFLGEK.A	2	2.73	0.16	-3.37
IPI00418163	C4B1	K.DDPDAPLQPVTPLQLFEGR.R	2	4.90	0.57	-4.98
IPI00418163	C4B1	K.DDPDAPLQPVTPLQLFEGR.R	3	3.93	0.32	-3.18
IPI00418163	C4B1	K.DDPDAPLQPVTPLQLFEGRR.N	3	2.03	0.14	-4.63
IPI00418163	C4B1	K.DHAVDLIQK.G	1	3.03	0.30	-2.14
IPI00418163	C4B1	K.DHAVDLIQK.G	2	2.76	0.32	-2.89
IPI00418163	C4B1	K.DVKAAANQM*R.N	2	2.64	0.13	-1.54
IPI00418163	C4B1	K.EGAIHREELVYELNPLDHR.G	2	4.35	0.44	-4.58
IPI00418163	C4B1	K.EGAIHREELVYELNPLDHR.G	3	5.45	0.39	-4.68
IPI00418163	C4B1	K.EGAIHREELVYELNPLDHR.G	4	2.47	0.18	-2.23
IPI00418163	C4B1	K.EGAIHREELVYELNPLDHR.G	5	2.71	0.21	-3.27
IPI00418163	C4B1	K.EGAIHREELVYELNPLDHRG.R	3	4.71	0.30	-2.28
IPI00418163	C4B1	K.EVYM*PSSIFQDDFVIPDISEPGTWK.I	2	4.19	0.53	-3.32
IPI00418163	C4B1	K.EVYM*PSSIFQDDFVIPDISEPGTWK.I	3	2.90	0.17	-4.23
IPI00418163	C4B1	K.FACYYPR.V	1	1.88	0.26	-1.69
IPI00418163	C4B1	K.FACYYPR.V	2	2.69	0.29	-1.19
IPI00418163	C4B1	K.GLCVATPVQLR.V	1	2.09	0.28	-4.05
IPI00418163	C4B1	K.GLCVATPVQLR.V	2	3.26	0.21	-2.73
IPI00418163	C4B1	K.GSVFLRNPSR.N	2	2.67	0.15	-3.14
IPI00418163	C4B1	K.INVKVGGNSK.G	2	2.52	0.17	-2.51
IPI00418163	C4B1	K.ITPGKPYILTVPGHLDEM*QLDIQAR.Y	2	3.47	0.45	-1.39
IPI00418163	C4B1	K.ITPGKPYILTVPGHLDEM*QLDIQAR.Y	3	4.87	0.51	-4.46
IPI00418163	C4B1	K.ITPGKPYILTVPGHLDEM*QLDIQAR.Y	4	3.95	0.28	-4.16
[truncated: 445,991 more chars]
