# Supplementary material for: Establishing the Proteome of Normal Human Cerebrospinal Fluid
Source: PLoS One. 2010 Jun 11;5(6):e10980. doi: 10.1371/journal.pone.0010980 (PMC2881861; doi:10.1371/journal.pone.0010980)
Supplement: Table S2 — Analysis of overlap between proteins identified in normal CSF, plasma and previous CSF (neurologic surrogate-normal) proteomic study. (0.24 MB PDF) [file pone.0010980.s009.pdf]

Supplementary Table 2. Analysis of overlap between proteins identified in normal CSF, plasma and previous CSF (neurologic surrogate-normal) proteomic study

***PNNL* plasma (burn-trauma) proteome**	***Zougman* - neurologic-surrogate normals**	**IPI**	**Protein name**	**Gene symbol**	**unique peptide count**
√	√	IPI00022434	Uncharacterized protein ALB	ALB	189
√	√	IPI00783987	Complement C3 precursor (Fragment)	C3	153
√	√	IPI00418163	C4B1	C4B	153
	√	IPI00032258	Complement C4-A precursor	C4A;C4B	151
√	√	IPI00878517	56 kDa protein	ALB	141
√	√	IPI00006601	Secretogranin-1 precursor	CHGB	133
√	√	IPI00384697	Isoform 2 of Serum albumin precursor	ALB	119
√	√	IPI00022463	Serotransferrin precursor	TF	105
	√	IPI00069058	VGF nerve growth factor inducible precursor	VGF	105
√	√	IPI00798430	Transferrin variant (Fragment)	TF	104
√	√	IPI00478003	Alpha-2-macroglobulin precursor	A2M	97
√	√	IPI00017601	Ceruloplasmin precursor	CP	95
√	√	IPI00292071	Secretogranin-3 precursor	SCG3	90
√	√	IPI00026314	Isoform 1 of Gelsolin precursor	GSN	86
√	√	IPI00029751	Isoform 1 of Contactin-1 precursor	CNTN1	83
		IPI00289501	Neurosecretory protein VGF precursor	VGF	83
√	√	IPI00013179	Prostaglandin-H2 D-isomerase precursor	PTGDS	81
√	√	IPI00514285	Prostaglandin D2 synthase 21kDa	PTGDS	79
√	√	IPI00296777	SPARC-like protein 1 precursor	SPARCL1	79
√	√	IPI00022229	Apolipoprotein B-100 precursor	APOB	79
√	√	IPI00006114	Pigment epithelium-derived factor precursor	SERPINF1	76
√	√	IPI00783390	Isoform 1 of Neural cell adhesion molecule L1-like protein precursor	CHL1	73
√	√	IPI00029739	Isoform 1 of Complement factor H precursor	CFH	73
√	√	IPI00242956	IgGFc-binding protein precursor	FCGBP	73
√	√	IPI00299059	Isoform 2 of Neural cell adhesion molecule L1-like protein precursor	CHL1	72
√	√	IPI00291262	Clusterin precursor	CLU	71
√	√	IPI00794184	97 kDa protein	CP	70
√	√	IPI00788189	similar to Fc fragment of IgG binding protein	FCGBP	68
√	√	IPI00553177	Isoform 1 of Alpha-1-antitrypsin precursor	SERPINA1	68
√	√	IPI00304273	Apolipoprotein A-IV precursor	APOA4	65
√	√	IPI00024284	Basement membrane-specific heparan sulfate proteoglycan core protein precursor	HSPG2	64

√		IPI00883753	NRCAM protein	NRCAM	64
√		IPI00847179	apolipoprotein A-IV precursor	APOA4	64
√	√	IPI00555812	Vitamin D-binding protein precursor	GC	63
√	√	IPI00333776	Isoform 1 of Neuronal cell adhesion molecule precursor	NRCAM	63
√	√	IPI00742696	vitamin D-binding protein precursor	GC	62
√		IPI00415032	Isoform 4 of Neuronal cell adhesion molecule precursor	NRCAM	62
√	√	IPI00072917	alpha 3 type VI collagen isoform 3 precursor	COL6A3	61
√	√	IPI00022488	Hemopexin precursor	HPX	61
√	√	IPI00647027	32 kDa protein	CHGB	60
√	√	IPI00020557	Prolow-density lipoprotein receptor-related protein 1 precursor	LRP1	59
√	√	IPI00241562	reelin isoform a	RELN	59
	√	IPI00607600	amyloid precursor-like protein 1 isoform 1 precursor	APLP1	59
	√	IPI00020012	Amyloid-like protein 1 precursor	APLP1	59
√	√	IPI00032220	Angiotensinogen precursor	AGT	58
√	√	IPI00007257	calsyntenin 1 isoform 2	CLSTN1	57
√	√	IPI00032291	Complement C5 precursor	C5	57
√	√	IPI00156171	Isoform 1 of Ectonucleotide pyrophosphatase/phosphodiesterase family member 2 precu	ENPP2	57
	√	IPI00414249	Isoform 1 of Neurexin-3-alpha precursor	NRXN3	57
		IPI00878576	Autotaxin isoform gamma	-	57
√	√	IPI00413959	Calsyntenin-1 precursor	CLSTN1	56
√	√	IPI00021842	Apolipoprotein E precursor	APOE	56
√	√	IPI00064667	Beta-Ala-His dipeptidase precursor	CNDP1	55
√	√	IPI00303210	Isoform 2 of Ectonucleotide pyrophosphatase/phosphodiesterase family member 2 precu	ENPP2	54
√	√	IPI00006608	Isoform APP770 of Amyloid beta A4 protein precursor (Fragment)	APP	53
	√	IPI00216728	Neurexin 3-alpha	NRXN3	53
	√	IPI00007921	Isoform 1 of Neurexin-2-alpha precursor	NRXN2	51
√	√	IPI00550991	Alpha-1-antichymotrypsin precursor	SERPINA3	50
	√	IPI00009362	Secretogranin-2 precursor	SCG2	50
		IPI00655702	Isoform 5 of Neurofascin precursor	NFASC	50
√	√	IPI00795918	neural cell adhesion molecule 1 isoform 2	NCAM1	49
√	√	IPI00019591	Isoform 1 of Complement factor B precursor (Fragment)	CFB	49
√	√	IPI00290315	Chromogranin-A precursor	CHGA	49
	√	IPI00470535	Dihydropyridine receptor alpha 2 subunit	CACNA2D1	49
√	√	IPI00291136	Collagen alpha-1(VI) chain precursor	COL6A1	48
√	√	IPI00021841	Apolipoprotein A-I precursor	APOA1	48
√	√	IPI00177543	peptidylglycine alpha-amidating monooxygenase isoform a, preproprotein	PAM	48
	√	IPI00394655	Isoform 4 of Neurofascin precursor	NFASC	48
√		IPI00479514	Voltage-dependent calcium channel subunit alpha-2/delta-1 precursor	CACNA2D1	48
√		IPI00639937	B-factor, properdin	CFB	47
√	√	IPI00021000	Isoform A of Osteopontin precursor	SPP1	46

√	√	IPI00478809	Coagulation factor V precursor	F5	46
√	√	IPI00022937	Coagulation factor V	F5	46
√	√	IPI00032179	Antithrombin III variant	SERPINC1	46
√	√	IPI00023814	Isoform 1 of Neogenin precursor	NEO1	45
		IPI00220741	spectrin, alpha, erythrocytic 1	SPTA1	45
√	√	IPI00019580	Plasminogen precursor	PLG	44
√	√	IPI00294193	Isoform 1 of Inter-alpha-trypsin inhibitor heavy chain H4 precursor	ITIH4	44
√	√	IPI00024966	Contactin-2 precursor	CNTN2	44
√	√	IPI00218192	Isoform 2 of Inter-alpha-trypsin inhibitor heavy chain H4 precursor	ITIH4	44
√	√	IPI00218875	Isoform C of Osteopontin precursor	SPP1	43
√	√	IPI00219042	Isoform 3 of Peptidyl-glycine alpha-amidating monooxygenase precursor	PAM	42
√	√	IPI00306339	secreted phosphoprotein 1 isoform b	SPP1	42
√	√	IPI00296608	Complement component C7 precursor	C7	42
		IPI00739237	similar to Complement C3 precursor	LOC653879	42
√	√	IPI00009997	N-acetyllactosaminide beta-1,3-N-acetylglucosaminyltransferase	B3GNT1	41
√	√	IPI00305461	Inter-alpha-trypsin inhibitor heavy chain H2 precursor	ITIH2	41
√	√	IPI00032293	Cystatin-C precursor	CST3	41
√	√	IPI00220644	Isoform M1 of Pyruvate kinase isozymes M1/M2	PKM2	41
	√	IPI00334238	neuronal pentraxin receptor	NPTXR	41
√	√	IPI00853525	Apolipoprotein A1	APOA1	40
√	√	IPI00218874	Isoform B of Osteopontin precursor	SPP1	40
√	√	IPI00215894	Isoform LMW of Kininogen-1 precursor	KNG1	39
√	√	IPI00031030	Isoform 1 of Amyloid-like protein 2 precursor	APLP2	39
√	√	IPI00025276	Isoform XB of Tenascin-X precursor	TNXB	39
√	√	IPI00032328	Isoform HMW of Kininogen-1 precursor	KNG1	37
√	√	IPI00376427	Neural cell adhesion molecule 2 precursor	NCAM2	37
	√	IPI00456623	Isoform 1 of Brevican core protein precursor	BCAN	37
√	√	IPI00019943	Afamin precursor	AFM	36
√	√	IPI00748312	protein tyrosine phosphatase, receptor-type, zeta1 precursor	PTPRZ1	36
√	√	IPI00291866	Plasma protease C1 inhibitor precursor	SERPING1	36
√	√	IPI00021885	Isoform 1 of Fibrinogen alpha chain precursor	FGA	35
√	√	IPI00022395	Complement component C9 precursor	C9	35
√	√	IPI00289831	Isoform PTPS of Receptor-type tyrosine-protein phosphatase S precursor	PTPRS	35
√	√	IPI00027310	Isoform 1 of Multiple epidermal growth factor-like domains 8	MEGF8	34
√	√	IPI00815926	IGHG1 protein	IGHG1	34
√	√	IPI00302641	Protocadherin Fat 2 precursor	FAT2	34
√	√	IPI00017696	Complement C1s subcomponent precursor	C1S	34
√	√	IPI00375547	Protein tyrosine phosphatase receptor type D	PTPRD	34
	√	IPI00015260	Protein kinase C-binding protein NELL2 precursor	NELL2	34
		IPI00607580	multiple EGF-like-domains 8	MEGF8	34

		IPI00784828	Putative uncharacterized protein DKFZp686C11235	-	34
		IPI00784807	Putative uncharacterized protein	-	34
√	√	IPI00002714	Dickkopf-related protein 3 precursor	DKK3	33
√	√	IPI00219798	Isoform 1 of Roundabout homolog 1 precursor	ROBO1	33
√		IPI00426051	Putative uncharacterized protein DKFZp686C15213	-	33
√	√	IPI00296537	Isoform C of Fibulin-1 precursor	FBLN1	32
√	√	IPI00384542	Isoform 2 of Nidogen-1 precursor	NID1	32
√	√	IPI00025465	Mimecan precursor	OGN	32
√	√	IPI00796279	25 kDa protein	SERPINF1	32
√	√	IPI00107831	Receptor-type tyrosine-protein phosphatase F precursor	PTPRF	32
√	√	IPI00761159	IGHM protein	IGHM	32
√	√	IPI00026944	Isoform 1 of Nidogen-1 precursor	NID1	32
√		IPI00472345	IGHG3 protein	IGHG3	32
		IPI00791343	261 kDa protein	-	32
		IPI00784894	Putative uncharacterized protein	-	32
√	√	IPI00018219	Transforming growth factor-beta-induced protein ig-h3 precursor	TGFBI	31
√	√	IPI00298497	Fibrinogen beta chain precursor	FGB	31
√	√	IPI00003802	Alpha-mannosidase 2	MAN2A1	31
√	√	IPI00025257	Semaphorin-7A precursor	SEMA7A	31
√	√	IPI00448925	IGHG1 protein	IGHG1	31
√	√	IPI00328829	inter-alpha trypsin inhibitor heavy chain precursor 5 isoform 1	ITIH5	31
	√	IPI00031121	Carboxypeptidase E precursor	CPE	31
√	√	IPI00022432	Transthyretin precursor	TTR	30
√	√	IPI00641737	Haptoglobin precursor	HP	30
√	√	IPI00816741	Complement component 5 variant (Fragment)	C5	30
√	√	IPI00296534	Isoform D of Fibulin-1 precursor	FBLN1	30
√	√	IPI00218725	laminin alpha 2 subunit isoform b precursor	LAMA2	30
√	√	IPI00027703	Isoform Long of Alpha-mannosidase IIx	MAN2A2	30
√	√	IPI00423463	Putative uncharacterized protein DKFZp686O01196	IGHG1	30
		IPI00784842	Putative uncharacterized protein DKFZp686G11190	IGHV4-31	30
√	√	IPI00219029	Aspartate aminotransferase, cytoplasmic	GOT1	29
√	√	IPI00295542	Nucleobindin-1 precursor	NUCB1	29
√	√	IPI00744835	Isoform Sap-mu-9 of Proactivator polypeptide precursor	PSAP	29
√	√	IPI00012503	Isoform Sap-mu-0 of Proactivator polypeptide precursor	PSAP	29
√	√	IPI00418262	Fructose-bisphosphate aldolase C	ALDOC	29
	√	IPI00159927	Neurocan core protein precursor	NCAN	29
		IPI00784810	IGHV4-31 protein	IGHV4-31	29
		IPI00785084	Immunoglobulin heavy variable 4-31	IGHV4-31	29
√	√	IPI00011229	Cathepsin D precursor	CTSD	28
√	√	IPI00465439	Fructose-bisphosphate aldolase A	ALDOA	28

√	√	IPI00016334	Isoform 1 of Cell surface glycoprotein MUC18 precursor	MCAM	28
√	√	IPI00003362	HSPA5 protein	HSPA5	28
√	√	IPI00853369	Plexin-B2 precursor	PLXNB2	28
√	√	IPI00872363	PTPRD protein	PTPRD	28
√	√	IPI00423466	Putative uncharacterized protein DKFZp686H20196	IGHG1	28
		IPI00790899	55 kDa protein	-	28
√	√	IPI00334282	Protein FAM3C precursor	FAM3C	27
√	√	IPI00019568	Prothrombin precursor (Fragment)	F2	27
√	√	IPI00296165	Complement C1r subcomponent precursor	C1R;C17orf13;/	27
√	√	IPI00829767	Uncharacterized protein IGHG2 (Fragment)	IGHG2	27
√	√	IPI00292950	Serpin peptidase inhibitor, clade D (Heparin cofactor), member 1	SERPIND1	27
√	√	IPI00292530	Inter-alpha-trypsin inhibitor heavy chain H1 precursor	ITIH1	27
√	√	IPI00023673	Galectin-3-binding protein precursor	LGALS3BP	27
√	√	IPI00303963	Complement C2 precursor (Fragment)	C2	27
	√*	IPI00000828	Proenkephalin A precursor	PENK	26
√	√	IPI00828205	IGHM protein	IGHM	26
√	√	IPI00451625	Isoform 2 of Cartilage acidic protein 1 precursor	CRTAC1	26
√	√	IPI00029658	Isoform 1 of EGF-containing fibulin-like extracellular matrix protein 1 precursor	EFEMP1	26
	√	IPI00016150	Neuroserpin precursor	SERPINI1	26
	√	IPI00004433	Contactin-6 precursor	CNTN6	26
√		IPI00012545	Isoform TGN51 of Trans-Golgi network integral membrane protein 2 precursor	TGOLN2	26
√		IPI00025252	Protein disulfide-isomerase A3 precursor	PDIA3	26
√		IPI00220117	Uncharacterized protein CD99	CD99	26
		IPI00784942	Putative uncharacterized protein DKFZp686E23209	-	26
√	√	IPI00022895	Alpha-1B-glycoprotein precursor	A1BG	25
√	√	IPI00394992	Isoform 2 of N-acetylmuramoyl-L-alanine amidase precursor	PGLYRP2	25
√	√	IPI00550558	Protein O-linked-mannose beta-1,2-N-acetylglucosaminyltransferase 1	POMGNT1	25
√	√	IPI00298828	Beta-2-glycoprotein 1 precursor	APOH	25
√	√	IPI00163207	Isoform 1 of N-acetylmuramoyl-L-alanine amidase precursor	PGLYRP2	25
√	√	IPI00009920	Complement component 6 precursor	C6	25
	√	IPI00300241	Leucine-rich repeat-containing protein 4B precursor	LRRC4B	25
√		IPI00386879	CDNA FLJ14473 fis, clone MAMMA1001080, highly similar to Homo sapiens SNC73 prot	IGHV3OR16-13	25
√		IPI00382606	Factor VII active site mutant immunoconjugate	F7	25
	√*	IPI00788835	25 kDa protein	PENK	24
√	√	IPI00015102	Isoform 1 of CD166 antigen precursor	ALCAM	24
√	√	IPI00013976	Laminin subunit beta-1 precursor	LAMB1	24
√	√	IPI00216171	Gamma-enolase	ENO2	24
		IPI00439446	MAN1A1 protein	MAN1A1	24
√	√	IPI00027780	72 kDa type IV collagenase precursor	MMP2	23
√	√	IPI00162735	Isoform 2 of Attractin precursor	ATRN	23

√	√	IPI00002147	Chitinase-3-like protein 1 precursor	CHI3L1	23
√	√	IPI00294395	Complement component C8 beta chain precursor	C8B	23
√	√	IPI00008318	Ephrin type-A receptor 4 precursor	EPHA4	23
√	√	IPI00853454	200 kDa protein	LAMB1	23
√	√	IPI00027087	Isoform 1 of Neural cell adhesion molecule L1 precursor	L1CAM	23
√	√	IPI00029863	SERPINF2 protein	SERPINF2	23
	√	IPI00029343	Isoform 1 of Contactin-associated protein-like 2 precursor	CNTNAP2	23
	√	IPI00430842	IGHA1 protein	IGHV3OR16-13	23
		IPI00744561	IGHA1 protein	IGHV3OR16-13	23
		IPI00784998	Putative uncharacterized protein DKFZp686M24218	-	23
	√*	IPI00166048	Isoform 1 of Cell adhesion molecule 3 precursor	CADM3	22
√	√	IPI00442294	Neurotrimin variant 3	HNT	22
√	√	IPI00296922	Laminin subunit beta-2 precursor	LAMB2	22
√	√	IPI00016915	Insulin-like growth factor-binding protein 7 precursor	IGFBP7	22
√	√	IPI00844156	SERPINC1 protein	SERPINC1	22
√	√	IPI00003351	Extracellular matrix protein 1 precursor	ECM1	22
√	√	IPI00646281	L1 cell adhesion molecule	L1CAM	22
	√	IPI00023845	Kallikrein-6 precursor	KLK6	22
	√	IPI00374563	Agrin precursor	AGRN	22
	√	IPI00002280	ProSAAS precursor	PCSK1N	22
√	√	IPI00298971	Vitronectin precursor	VTN	21
√	√	IPI00029260	Monocyte differentiation antigen CD14 precursor	CD14	21
√	√	IPI00442297	Isoform 2 of Neurotrimin precursor	HNT	21
√	√	IPI00830132	Uncharacterized protein IGHG4 (Fragment)	IGHG4	21
√	√	IPI00872555	cDNA FLJ76262, highly similar to Homo sapiens I factor (complement) (IF), mRNA	CFI	21
√	√	IPI00218733	Superoxide dismutase	SOD1	21
√	√	IPI00515041	Uncharacterized protein CFH	CFH	21
√	√	IPI00019157	Chondroitin sulfate proteoglycan 4 precursor	CSPG4	21
√	√	IPI00465028	Isoform 1 of Triosephosphate isomerase	TPI1	21
	√	IPI00220562	Neuronal pentraxin-1 precursor	NPTX1	21
	√	IPI00166622	similar to CG14446-PA	TMEM132C	21
	√	IPI00787050	similar to neuronal pentraxin I precursor	NPTX1	21
√		IPI00216704	Isoform 2 of Spectrin beta chain, erythrocyte	SPTB	21
√		IPI00647704	CDNA FLJ41552 fis, clone COLON2004478, highly similar to Protein Tro alpha1 H,myelo	IGHV3OR16-13	21
		IPI00784950	Putative uncharacterized protein DKFZp686L19235	LOC10012658	21
		IPI00550731	Putative uncharacterized protein	-	21
		IPI00472249	protein tyrosine phosphatase, receptor type, N polypeptide 2 isoform 2 precursor	PTPRN2	21
√	√	IPI00654888	Plasma kallikrein precursor	KLKB1	20
√	√	IPI00792626	14 kDa protein	TF	20
√	√	IPI00171411	Golgi phosphoprotein 2	GOLM1	20

√	√	IPI00020996	Insulin-like growth factor-binding protein complex acid labile chain precursor	IGFALS	20
√	√	IPI00783399	Isoform 1 of Transmembrane protein 132D precursor	TMEM132D	20
	√	IPI00160552	Isoform 1 of Tenascin-R precursor	TNR	20
	√	IPI00294004	Vitamin K-dependent protein S precursor	PROS1	20
	√	IPI00301865	Isoform 1 of Transmembrane protein 132A precursor	TMEM132A	20
√		IPI00216697	Isoform Er1 of Ankyrin-1	ANK1	20
√		IPI00440577	IGKV2-24 protein	IGKV2-24	20
√		IPI00061977	IGHA1 protein	IGHV3OR16-13	20
√		IPI00855725	Isoform 4 of Trans-Golgi network integral membrane protein 2 precursor	TGOLN2	20
√	√	IPI00020986	Lumican precursor	LUM	19
√	√	IPI00297646	Collagen alpha-1(I) chain precursor	COL1A1	19
√	√	IPI00845354	IGKC protein	IGKC	19
√	√	IPI00169383	Phosphoglycerate kinase 1	PGK1	19
√	√	IPI00004669	Polypeptide N-acetylgalactosaminyltransferase 2	GALNT2	19
√	√	IPI00022426	AMBP protein precursor	AMBP	19
√	√	IPI00022284	Major prion protein precursor	PRNP	19
√	√	IPI00328609	Kallistatin precursor	SERPINA4	19
√	√	IPI00219365	Moesin	MSN	19
√	√	IPI00006662	Apolipoprotein D precursor	APOD	19
√	√	IPI00004656	Beta-2-microglobulin	B2M	19
√	√	IPI00021891	Isoform Gamma-B of Fibrinogen gamma chain precursor	FGG	19
√	√	IPI00018136	Isoform 1 of Vascular cell adhesion protein 1 precursor	VCAM1	19
	√	IPI00001662	Opioid-binding protein/cell adhesion molecule precursor	OPCML	19
	√	IPI00477747	Isoform 1 of Follistatin-related protein 4 precursor	FSTL4	19
	√	IPI00183445	Isoform 1 of Latrophilin-1 precursor	LPHN1	19
		IPI00784985	IGK@ protein	IGK@	19
		IPI00334667	Isoform 2 of Receptor-type tyrosine-protein phosphatase N2 precursor	PTPRN2	19
		IPI00784969	Putative uncharacterized protein	LOC10012658[illegible]	19
		IPI00785067	IGH@ protein	IGH@	19
√	√	IPI00430808	Immunoblobulin light chain (Fragment)	IGKC	18
√	√	IPI00477597	Isoform 1 of Haptoglobin-related protein precursor	HPR	18
√	√	IPI00301579	Epididymal secretory protein E1 precursor	NPC2	18
√	√	IPI00290085	Cadherin-2 precursor	CDH2	18
√	√	IPI00022371	Histidine-rich glycoprotein precursor	HRG	18
√	√	IPI00176221	Neuronal growth regulator 1 precursor	NEGR1	18
√	√	IPI00480183	Protein	PTPRF	18
	√	IPI00855824	dipeptidyl-peptidase 6 isoform 3	DPP6	18
	√	IPI00289083	Isoform 1 of VWFA and cache domain-containing protein 1 precursor	CACHD1	18
	√	IPI00016422	Netrin receptor DCC precursor	DCC	18
	√	IPI00000779	Isoform 1 of ADAM 22 precursor	ADAM22	18

		IPI00784830	CDNA FLJ41981 fis, clone SMINT2011888, highly similar to Protein Tro alpha1 H,myelon	LOC10012658З	18
		IPI00784758	Putative uncharacterized protein DKFZp686M08189	LOC10012658З	18
√	√	IPI00021854	Apolipoprotein A-II precursor	APOA2	17
√	√	IPI00298281	Laminin subunit gamma-1 precursor	LAMC1	17
√	√	IPI00008787	Alpha-N-acetylglucosaminidase precursor	NAGLU	17
√	√	IPI00028911	Dystroglycan precursor	DAG1	17
√	√	IPI00166729	alpha-2-glycoprotein 1, zinc	AZGP1	17
√	√	IPI00022417	Leucine-rich alpha-2-glycoprotein precursor	LRG1	17
√	√	IPI00022431	Alpha-2-HS-glycoprotein precursor	AHSG	17
√	√	IPI00010154	Rab GDP dissociation inhibitor alpha	GDI1	17
√	√	IPI00009028	Tetranectin precursor	CLEC3B	17
√	√	IPI00746963	IGKC protein	IGKC	17
√	√	IPI00003590	Isoform 1 of Sulfhydryl oxidase 1 precursor	QSOX1	17
√	√	IPI00027827	Extracellular superoxide dismutase [Cu-Zn] precursor	SOD3	17
√	√	IPI00219217	L-lactate dehydrogenase B chain	LDHB	17
√	√	IPI00465248	Isoform alpha-enolase of Alpha-enolase	ENO1	17
√	√	IPI00792115	Putative uncharacterized protein DKFZp686H17246	CLEC3B	17
√	√	IPI00410600	Isoform 3 of Voltage-dependent calcium channel subunit alpha-2/delta-2 precursor	CACNA2D2	17
√	√	IPI00298237	Isoform 1 of Tripeptidyl-peptidase 1 precursor	TPP1	17
√	√	IPI00419722	seizure related 6 homolog (mouse)-like 2 isoform 2	SEZ6L2	17
	√	IPI00879665	112 kDa protein	SEZ6L	17
	√	IPI00028908	Nidogen-2 precursor	NID2	17
	√	IPI00252731	Isoform DPPX-S of Dipeptidyl aminopeptidase-like protein 6	DPP6	17
√		IPI00012585	Beta-hexosaminidase beta chain precursor	HEXB	17
√		IPI00021304	Keratin, type II cytoskeletal 2 epidermal	KRT2	17
√		IPI00299024	Brain acid soluble protein 1	BASP1	17
		IPI00784773	Putative uncharacterized protein	-	17
		IPI00784865	IGK@ protein	IGK@	17
		IPI00008603	Actin, aortic smooth muscle	ACTA2	17
√	√	IPI00020599	Calreticulin precursor	CALR	16
√	√	IPI00472961	IGKC protein	IGKC	16
√	√	IPI00011651	Isoform 1 of Receptor-type tyrosine-protein phosphatase gamma precursor	PTPRG	16
√	√	IPI00027851	Beta-hexosaminidase alpha chain precursor	HEXA	16
√	√	IPI00396423	Alcadein beta	CLSTN3	16
√	√	IPI00295741	Cathepsin B precursor	CTSB	16
√	√	IPI00853045	Anti-RhD monoclonal T125 kappa light chain precursor	IGKC	16
√	√	IPI00289819	Cation-independent mannose-6-phosphate receptor precursor	IGF2R	16
√	√	IPI00011252	Complement component C8 alpha chain precursor	C8A	16
√	√	IPI00015688	Glypican-1 precursor	GPC1	16
√	√	IPI00020091	Alpha-1-acid glycoprotein 2 precursor	ORM2	16

√	√	IPI00854806	IGKV1-5 protein	IGKV1-5	16
√	√	IPI00022429	Alpha-1-acid glycoprotein 1 precursor	ORM1	16
√	√	IPI00014572	SPARC precursor	SPARC	16
√	√	IPI00013303	Limbic system-associated membrane protein precursor	LSAMP	16
√	√	IPI00022420	Plasma retinol-binding protein precursor	RBP4	16
√	√	IPI00298793	Beta-mannosidase precursor	MANBA	16
√	√	IPI00001952	Endonuclease domain-containing 1 protein precursor	ENDOD1	16
√	√	IPI00152540	Isoform 1 of CD109 antigen precursor	CD109	16
√	√	IPI00027848	Macrophage mannose receptor 1 precursor	MRC1	16
√	√	IPI00643034	Isoform 1 of Phospholipid transfer protein precursor	PLTP	16
	√	IPI00026946	Neuronal pentraxin-2 precursor	NPTX2	16
	√	IPI00166766	hypothetical protein LOC146556 isoform 2	MGC45438	16
	√	IPI00154734	seizure related 6 homolog isoform 1	SEZ6	16
	√	IPI00410210	Isoform 2 of Latrophilin-1 precursor	LPHN1	16
	√	IPI00008944	Isoform 1 of Neuroendocrine protein 7B2 precursor	SCG5	16
√		IPI00384952	Putative uncharacterized protein DKFZp686K04218 (Fragment)	-	16
		IPI00180240	thymosin-like 3	TMSL3	16
√	√	IPI00176458	protocadherin 1 isoform 2 precursor	PCDH1	15
√	√	IPI00011218	Macrophage colony-stimulating factor 1 receptor precursor	CSF1R	15
√	√	IPI00292946	Thyroxine-binding globulin precursor	SERPINA7	15
√	√	IPI00329775	Isoform 1 of Carboxypeptidase B2 precursor	CPB2	15
√	√	IPI00024046	Cadherin-13 precursor	CDH13	15
√	√	IPI00221224	Aminopeptidase N	ANPEP	15
√	√	IPI00297124	Isoform 1 of Interleukin-6 receptor subunit beta precursor	IL6ST	15
√	√	IPI00297284	Insulin-like growth factor-binding protein 2 precursor	IGFBP2	15
	√	IPI00102543	SLIT and NTRK-like protein 1 precursor	SLITRK1	15
	√	IPI00550162	IGLV3-25 protein	IGLV3-25	15
	√	IPI00165949	Isoform 2 of Endoplasmic reticulum aminopeptidase 1	ERAP1	15
	√	IPI00056478	Isoform 1 of Immunoglobulin superfamily member 8 precursor	IGSF8	15
√		IPI00385264	Ig mu heavy chain disease protein	-	15
√		IPI00418471	Vimentin	VIM	15
√		IPI00178302	Isoform 4 of Semaphorin-6D precursor	SEMA6D	15
		IPI00465016	Isoform 2 of Sulfhydryl oxidase 1 precursor	QSOX1	15
√	√	IPI00104074	Isoform 1 of Scavenger receptor cysteine-rich type 1 protein M130 precursor	CD163	14
√	√	IPI00012887	Cathepsin L1 precursor	CTSL1	14
√	√	IPI00219446	Phosphatidylethanolamine-binding protein 1	PEBP1	14
√	√	IPI00154742	IGL@ protein	IGL@	14
√	√	IPI00009950	Vesicular integral-membrane protein VIP36 precursor	LMAN2	14
√	√	IPI00012303	Selenium-binding protein 1	SELENBP1	14
√	√	IPI00829626	IGL@ protein	IGL@	14

√	√	IPI00005794	60 kDa protein	PGCP	14
√	√	IPI00295832	Oligodendrocyte-myelin glycoprotein precursor	OMG	14
√	√	IPI00004440	Receptor-type tyrosine-protein phosphatase-like N precursor	PTPRN	14
√	√	IPI00030255	Procollagen-lysine,2-oxoglutarate 5-dioxygenase 3 precursor	PLOD3	14
√	√	IPI00027350	Peroxiredoxin-2	PRDX2	14
√	√	IPI00291005	Malate dehydrogenase, cytoplasmic	MDH1	14
√	√	IPI00292791	Contactin-3 precursor	CNTN3	14
√	√	IPI00027192	Procollagen-lysine,2-oxoglutarate 5-dioxygenase 1 precursor	PLOD1	14
√	√	IPI00296141	Dipeptidyl-peptidase 2 precursor	DPP7	14
√	√	IPI00794070	CFI protein	CFI	14
√	√	IPI00009802	Isoform V0 of Versican core protein precursor	VCAN	14
√	√	IPI00465184	Guanine deaminase	GDA	14
	√	IPI00795013	149 kDa protein	LPHN3	14
	√	IPI00385980	ROBO2 isoform a	ROBO2	14
	√	IPI00218046	Heparan-sulfate 6-O-sulfotransferase 3	HS6ST3	14
	√	IPI00022337	Interphotoreceptor retinoid-binding protein precursor	RBP3	14
	√	IPI00103175	Isoform 1 of Soluble calcium-activated nucleotidase 1	CANT1	14
√		IPI00009865	Keratin, type I cytoskeletal 10	KRT10	14
		IPI00552905	Isoform 1 of Proline-rich transmembrane protein 3 precursor	PRRT3	14
		IPI00787853	Inositol monophosphatase 3	IMPAD1	14
	√*	IPI00000130	Somatostatin precursor	SST	13
√	√	IPI00829877	IGL@ protein	IGL@	13
√	√	IPI00013698	Acid ceramidase precursor	ASAH1	13
√	√	IPI00011264	Complement factor H-related protein 1 precursor	CFHR1	13
√	√	IPI00163563	PEBP family protein precursor	PEBP4	13
√	√	IPI00328391	N-acetylgalactosaminyltransferase 7	GALNT7	13
√	√	IPI00026216	Puromycin-sensitive aminopeptidase	NPEPPS	13
√	√	IPI00783492	Isoform 2 of Latent-transforming growth factor beta-binding protein 4 precursor	LTBP4	13
√	√	IPI00816626	PLXNB2 protein	PLXNB2	13
√	√	IPI00027230	Endoplasmin precursor	HSP90B1	13
√	√	IPI00290283	mannan-binding lectin serine protease 1 isoform 2 precursor	MASP1	13
√	√	IPI00376689	Isoform 1 of Protein KIAA1199 precursor	KIAA1199	13
√	√	IPI00419724	semaphorin 4B precursor	SEMA4B	13
√	√	IPI00218732	Serum paraoxonase/arylesterase 1	PON1	13
√	√	IPI00021263	14-3-3 protein zeta/delta	YWHAZ	13
√	√	IPI00031461	Rab GDP dissociation inhibitor beta	GDI2	13
	√	IPI00293836	Isoform 3 of Cell adhesion molecule 2 precursor	CADM2	13
	√	IPI00220334	Isoform 3 of Seizure 6-like protein precursor	SEZ6L	13
	√	IPI00176427	Cell adhesion molecule 4 precursor	CADM4	13
	√	IPI00410714	Hemoglobin subunit alpha	HBA2;HBA1	13

√		IPI00220327	Keratin, type II cytoskeletal 1	KRT1	13
√		IPI00022361	Band 3 anion transport protein	SLC4A1	13
√		IPI00012989	Lysosomal alpha-mannosidase precursor	MAN2B1	13
√		IPI00152418	Decay-accelerating factor splicing variant 4	CD55	13
√		IPI00001593	Lysosomal Pro-X carboxypeptidase precursor	PRCP	13
√		IPI00166392	Immunoglobulin superfamily member 4	CADM1	13
		IPI00304925	Heat shock 70 kDa protein 1	HSPA1A;HSPA	13
		IPI00784519	Putative uncharacterized protein	-	13
		IPI00382938	IGLV4-3 protein	IGLV4-3	13
		IPI00785200	Putative uncharacterized protein	-	13
√	√	IPI00012440	Plasma alpha-L-fucosidase precursor	FUCA2	12
√	√	IPI00289204	Reticulon-4 receptor precursor	RTN4R	12
√	√	IPI00418446	N-acylsphingosine amidohydrolase (acid ceramidase) 1 isoform b	ASAH1	12
√	√	IPI00654755	Hemoglobin subunit beta	HBB	12
√	√	IPI00015881	Isoform 1 of Macrophage colony-stimulating factor 1 precursor	CSF1	12
√	√	IPI00787781	similar to Metalloproteinase inhibitor 2 precursor	TIMP2	12
√	√	IPI00026104	Isoform Long of Iduronate 2-sulfatase precursor	IDS	12
√	√	IPI00000816	14-3-3 protein epsilon	YWHAE	12
√	√	IPI00793166	15 kDa protein	SPARCL1	12
√	√	IPI00299738	Procollagen C-endopeptidase enhancer 1 precursor	PCOLCE	12
√	√	IPI00022822	Isoform 2 of Collagen alpha-1(XVIII) chain precursor	COL18A1	12
√	√	IPI00218413	biotinidase precursor	BTD	12
√	√	IPI00019581	Coagulation factor XII precursor	F12	12
√	√	IPI00006967	Protocadherin-9 precursor	PCDH9	12
√	√	IPI00019576	Coagulation factor X precursor	F10	12
√	√	IPI00028413	Isoform 1 of Inter-alpha-trypsin inhibitor heavy chain H3 precursor	ITIH3	12
√	√	IPI00167093	complement factor H-related 1	CFHR1	12
√	√	IPI00018236	Ganglioside GM2 activator precursor	GM2A	12
√	√	IPI00877029	FGA protein	FGA	12
√	√	IPI00027166	Metalloproteinase inhibitor 2 precursor	TIMP2	12
√	√	IPI00411680	Isoform 1 of Protein-L-isoaspartate(D-aspartate) O-methyltransferase	PCMT1	12
√	√	IPI00852979	hypothetical protein LOC25758	C11orf41	12
	√	IPI00414984	sarcoglycan, epsilon isoform 1	SGCE	12
	√	IPI00791228	glutamate receptor, ionotrophic, AMPA 4 isoform 3 precursor	GRIA4	12
	√	IPI00162547	latrophilin 3 precursor	LPHN3	12
	√	IPI00022608	Sortilin-related receptor precursor	SORL1	12
	√	IPI00024570	Semaphorin-3G precursor	SEMA3G	12
	√	IPI00008994	Isoform 1 of Protein NDRG2	NDRG2	12
√		IPI00157414	Ectonucleotide pyrophosphatase/phosphodiesterase family member 6 precursor	ENPP6	12
√		IPI00443799	hypothetical protein LOC124565 isoform a	MGC15523	12

√		IPI00746623	Hyaluronan-binding protein 2 precursor	HABP2	12
√		IPI00829711	Uncharacterized protein IGHA2 (Fragment)	IGHA2	12
		IPI00785196	Putative uncharacterized protein	-	12
		IPI00743302	intercellular adhesion molecule 5 precursor	ICAM5	12
		IPI00032063	Similar to Candidate tumor suppressor protein	LRP1B	12
		IPI00301395	Probable serine carboxypeptidase CPVL precursor	CPVL	12
√	√	IPI00328746	Reticulon-4 receptor-like 2 precursor	RTN4RL2	11
√	√	IPI00298388	Isoform 1 of Phosphoinositide-3-kinase-interacting protein 1 precursor	PIK3IP1	11
√	√	IPI00026154	Glucosidase 2 subunit beta precursor	PRKCSH	11
√	√	IPI00217966	Isoform 1 of L-lactate dehydrogenase A chain	LDHA	11
√	√	IPI00014048	Ribonuclease pancreatic precursor	RNASE1	11
√	√	IPI00023728	Gamma-glutamyl hydrolase precursor	GGH	11
√	√	IPI00015315	Extracellular matrix protein 2 precursor	ECM2	11
√	√	IPI00247243	31 kDa protein	-	11
√	√	IPI00216318	Isoform Long of 14-3-3 protein beta/alpha	YWHAB	11
√	√	IPI00293748	Isoform 1 of Multiple inositol polyphosphate phosphatase 1 precursor	MINPP1	11
√	√	IPI00829640	IGL@ protein	IGL@	11
√	√	IPI00027493	4F2 cell-surface antigen heavy chain	SLC3A2;LOC4	11
√	√	IPI00026199	Glutathione peroxidase 3 precursor	GPX3	11
√	√	IPI00646304	peptidylprolyl isomerase B precursor	PPIB	11
√	√	IPI00029693	Isoform A22 of Neuropilin-2 precursor	NRP2	11
√	√	IPI00877169	calcium/calmodulin-dependent protein kinase IIA isoform 2	CAMK2A	11
√	√	IPI00010949	Isoform 1 of Sialate O-acetylesterase precursor	SIAE	11
	√	IPI00220342	N(G),N(G)-dimethylarginine dimethylaminohydrolase 1	DDAH1	11
	√	IPI00718977	glutamate receptor, ionotrophic, AMPA 4 isoform 2 precursor	GRIA4	11
	√	IPI00302181	Isoform 1 of Voltage-dependent calcium channel subunit alpha-2/delta-3 precursor	CACNA2D3	11
	√	IPI00465325	leucine-rich repeat neuronal 6A	LINGO1	11
	√	IPI00337548	Cell growth regulator with EF hand domain protein 1	CGREF1	11
√		IPI00032532	Isoform 2 of Growth arrest-specific protein 6 precursor	GAS6	11
√		IPI00643920	Transketolase	TKT	11
√		IPI00743766	Fetuin-B precursor	FETUB	11
√		IPI00000877	Hypoxia up-regulated protein 1 precursor	HYOU1	11
√		IPI00423461	Putative uncharacterized protein DKFZp686C02220 (Fragment)	IGHA2	11
√		IPI00219526	Isoform 1 of Phosphoglucomutase-1	PGM1	11
√		IPI00783665	Laminin subunit alpha-5 precursor	LAMA5	11
		IPI00012102	N-acetylglucosamine-6-sulfatase precursor	GNS	11
		IPI00003921	Isoform 1 of Protein 4.1	EPB41	11
√	√	IPI00011261	Complement component C8 gamma chain precursor	C8G	10
√	√	IPI00879084	20 kDa protein	CP	10
√	√	IPI00029235	Insulin-like growth factor-binding protein 6 precursor	IGFBP6	10

√	√	IPI00395488	Vasorin precursor	VASN	10
√	√	IPI00298994	Talin-1	TLN1	10
√	√	IPI00010471	Plastin-2	LCP1	10
√	√	IPI00298547	Protein DJ-1	PARK7	10
√	√	IPI00220642	14-3-3 protein gamma	YWHAG	10
√	√	IPI00843910	Tissue alpha-L-fucosidase precursor	FUCA1	10
√	√	IPI00002211	Isoform 2 of Semaphorin-6A precursor	SEMA6A	10
√	√	IPI00418531	Isoform 1 of Gliomedin	GLDN	10
√	√	IPI00025426	Pregnancy zone protein precursor	PZP	10
√	√	IPI00022331	Phosphatidylcholine-sterol acyltransferase precursor	LCAT	10
√	√	IPI00010796	Protein disulfide-isomerase precursor	P4HB	10
√	√	IPI00009793	Complement C1r-like protein	C1RL	10
√	√	IPI00297263	Isoform 1 of Protein HEG homolog 1 precursor	HEG1	10
√	√	IPI00871139	92 kDa protein	MASP1	10
	√	IPI00784156	Isoform 1 of AP-2 complex subunit beta-1	AP2B1	10
	√	IPI00103597	Isoform 1 of VPS10 domain-containing receptor SorCS1 precursor	SORCS1	10
	√	IPI00176193	Isoform 1 of Collagen alpha-1(XIV) chain precursor	COL14A1	10
	√	IPI00456736	Isoform 1 of RGM domain family member B precursor	RGMB	10
	√	IPI00010381	VPS10 domain-containing receptor SorCS3 precursor	SORCS3	10
	√	IPI00003919	Glutaminyl-peptide cyclotransferase precursor	QPCT	10
	√	IPI00007236	Isoform 2 of Neuroligin-1 precursor	NLGN1	10
√		IPI00296176	Coagulation factor IX precursor	F9	10
√		IPI00163187	Fascin	FSCN1	10
√		IPI00304962	Collagen alpha-2(I) chain precursor	COL1A2	10
√		IPI00292550	Isoform 1 of Polypeptide N-acetylgalactosaminyltransferase 13	GALNT13	10
√		IPI00027497	Glucose-6-phosphate isomerase	GPI	10
		IPI00815938	IGLV3-21 protein	IGLV3-21	10
		IPI00816555	IGLV2-14 protein	IGLV2-14	10
		IPI00015964	Neuromodulin	GAP43	10
		IPI00299299	Stress 70 protein chaperone microsome-associated 60 kDa protein precursor	STCH	10
√	√	IPI00301459	1-O-acylceramide synthase precursor	LYPLA3	9
√	√	IPI00290856	Lymphatic vessel endothelial hyaluronic acid receptor 1 precursor	LYVE1	9
√	√	IPI00029723	Follistatin-related protein 1 precursor	FSTL1	9
√	√	IPI00297487	Cathepsin H precursor	CTSH	9
√	√	IPI00027482	Corticosteroid-binding globulin precursor	SERPINA6	9
√	√	IPI00215983	Carbonic anhydrase 1	CA1	9
√	√	IPI00790473	12 kDa protein	SERPINF1	9
√	√	IPI00000137	N-acetylglucosamine-1-phosphotransferase subunit gamma precursor	GNPTG	9
√	√	IPI00477992	complement component 1, q subcomponent, B chain precursor	C1QB	9
√	√	IPI00015756	Isoform 1 of Receptor-type tyrosine-protein phosphatase kappa precursor	PTPRK	9

√	√	IPI00002816	Cathepsin F precursor	CTSF	9
√	√	IPI00023014	von Willebrand factor precursor	VWF	9
√	√	IPI00021485	Leucine-rich repeat neuronal protein 1 precursor	LRRN1	9
√	√	IPI00293088	Lysosomal alpha-glucosidase precursor	GAA	9
√	√	IPI00020990	Osteomodulin precursor	OMD	9
√	√	IPI00745660	IGL@ protein	IGL@	9
√	√	IPI00414896	Isoform 1 of Ribonuclease T2 precursor	RNASET2	9
√	√	IPI00023019	Isoform 1 of Sex hormone-binding globulin precursor	SHBG	9
√	√	IPI00002732	EXTL2 protein (Fragment)	EXTL2	9
√	√	IPI00007221	Plasma serine protease inhibitor precursor	SERPINA5	9
√	√	IPI00018769	Thrombospondin-2 precursor	THBS2	9
√	√	IPI00788786	309 kDa protein	VWF	9
√	√	IPI00064607	Isoform 1 of Multiple epidermal growth factor-like domains 10 precursor	MEGF10	9
√	√	IPI00216691	Profilin-1	PFN1	9
√	√	IPI00005142	Isoform 1 of Basic fibroblast growth factor receptor 1 precursor	FGFR1	9
√	√	IPI00001611	Isoform 1 of Insulin-like growth factor II precursor	IGF2	9
√	√	IPI00000874	Peroxiredoxin-1	PRDX1	9
√	√	IPI00163446	IGHD protein	IGHD	9
√	√	IPI00027038	Isoform 1 of V-set and immunoglobulin domain-containing protein 4 precursor	VSIG4	9
√	√	IPI00013682	Isoform 3 of Ecto-ADP-ribosyltransferase 3 precursor	ART3	9
	√	IPI00745363	Immunglobulin heavy chain variable region (Fragment)	LOC652113	9
	√	IPI00106646	45 kDa calcium-binding protein precursor	SDF4	9
	√	IPI00005292	Testican-1 precursor	SPOCK1	9
	√	IPI00292150	Latent-transforming growth factor beta-binding protein 2 precursor	LTBP2	9
	√	IPI00514676	myelin oligodendrocyte glycoprotein isoform beta2 precursor	MOG	9
	√	IPI00376394	Sulfhydryl oxidase 2 precursor	QSOX2	9
	√	IPI00008087	Follistatin-related protein 5 precursor	FSTL5	9
	√	IPI00014592	Chondroadherin precursor	CHAD	9
	√	IPI00183487	Xylosyltransferase 1	XYLT1	9
	√	IPI00024621	Isoform 1 of Olfactomedin-like protein 3 precursor	OLFML3	9
√		IPI00015902	Beta-type platelet-derived growth factor receptor precursor	PDGFRB	9
√		IPI00303071	Cat eye syndrome critical region protein 1 precursor	CECR1	9
√		IPI00470607	family with sequence similarity 20, member C	FAM20C	9
√		IPI00011454	Isoform 2 of Neutral alpha-glucosidase AB precursor	GANAB	9
√		IPI00025110	Isoform 2 of Mesothelin precursor	MSLN	9
√		IPI00291175	Isoform 1 of Vinculin	VCL	9
√		IPI00220292	Isoform 1 of Ecto-ADP-ribosyltransferase 3 precursor	ART3	9
√		IPI00009123	Nucleobindin-2 precursor	NUCB2	9
√		IPI00299571	Isoform 2 of Protein disulfide-isomerase A6 precursor	PDIA6	9
√		IPI00002406	Lutheran blood group glycoprotein precursor	BCAM	9

		IPI00785079	Putative uncharacterized protein	-	9
		IPI00852577	IGLC1 protein	IGLC1	9
		IPI00871326	plexin A1	PLXNA1	9
		IPI00001477	Isoform 1 of Epithelial discoidin domain-containing receptor 1 precursor	DDR1	9
		IPI00795055	CDNA FLJ14022 fis, clone HEMBA1003538, weakly similar to COMPLEMENT C1R COM	C1RL	9
		IPI00011654	Tubulin beta chain	TUBB	9
√	√	IPI00005707	Macrophage mannose receptor 2 precursor	MRC2	8
√	√	IPI00883772	acid alpha-glucosidase preproprotein	GAA	8
√	√	IPI00302592	filamin A, alpha isoform 1	FLNA	8
√	√	IPI00003865	Isoform 1 of Heat shock cognate 71 kDa protein	HSPA8	8
√	√	IPI00003366	Isoform TrkB of BDNF/NT-3 growth factors receptor precursor	NTRK2	8
√	√	IPI00021033	Isoform 1 of Collagen alpha-1(III) chain precursor	COL3A1	8
√	√	IPI00010295	Carboxypeptidase N catalytic chain precursor	CPN1	8
√	√	IPI00165438	Muscle type neuropilin 1	NRP1	8
√	√	IPI00219465	Transcobalamin-2 precursor	TCN2	8
√	√	IPI00002745	Cathepsin Z precursor	CTSZ	8
√	√	IPI00044369	Isoform 1 of Plexin domain-containing protein 2 precursor	PLXDC2	8
√	√	IPI00007778	Di-N-acetylchitobiase precursor	CTBS	8
√	√	IPI00022296	Mast/stem cell growth factor receptor precursor	KIT	8
√	√	IPI00473011	Hemoglobin subunit delta	HBB;HBD	8
√	√	IPI00297224	Sushi domain-containing protein 5	SUSD5	8
√	√	IPI00645206	Isoform 1 of Protocadherin-17 precursor	PCDH17	8
√	√	IPI00219757	Glutathione S-transferase P	GSTP1	8
√	√	IPI00294705	Papilin	PAPLN	8
√	√	IPI00032292	Metalloproteinase inhibitor 1 precursor	TIMP1	8
√	√	IPI00217493	Myoglobin	MB	8
	√	IPI00015346	Cadherin EGF LAG seven-pass G-type receptor 2 precursor	CELSR2	8
	√	IPI00455667	hypothetical protein LOC402665	LOC402665	8
	√	IPI00001895	Isoform 1 of Protocadherin-8 precursor	PCDH8	8
	√	IPI00419585	Peptidyl-prolyl cis-trans isomerase A	PPIA;LOC6541	8
	√	IPI00007798	Thyrotropin-releasing hormone-degrading ectoenzyme	TRHDE	8
	√	IPI00217146	SLIT and NTRK-like protein 4 precursor	SLITRK4	8
	√	IPI00784119	Vacuolar ATP synthase subunit S1 precursor	ATP6AP1	8
	√	IPI00008290	Isoform 1 of Ephrin type-A receptor 5 precursor	EPHA5	8
	√	IPI00152847	WAP, kazal, immunoglobulin, kunitz and NTR domain-containing protein 2 precursor	WFIKKN2	8
√		IPI00003269	hypothetical protein LOC345651	DKFZp686D09	8
√		IPI00009904	Protein disulfide-isomerase A4 precursor	PDIA4	8
√		IPI00007752	Tubulin beta-2C chain	TUBB2C	8
√		IPI00477804	Immunglobulin heavy chain variable region (Fragment)	-	8
√		IPI00328488	Isoform 1 of Epididymis-specific alpha-mannosidase precursor	MAN2B2	8

√		IPI00022810	Dipeptidyl-peptidase 1 precursor	CTSC	8
√		IPI00019906	Isoform 2 of Basigin precursor	BSG	8
		IPI00013475	Tubulin beta-2A chain	TUBB2A	8
		IPI00007702	Heat shock-related 70 kDa protein 2	HSPA2	8
		IPI00329352	Nodal modulator 1 precursor	NOMO1;NOMC	8
		IPI00023598	Tubulin beta-4 chain	TUBB4	8
		IPI00292304	Uncharacterized protein C9orf4	C9orf4	8
		IPI00016870	Zona pellucida sperm-binding protein 2 precursor	ZP2	8
		IPI00854743	Uncharacterized protein ENSP00000375034	-	8
		IPI00220739	Membrane-associated progesterone receptor component 1	PGRMC1	8
		IPI00043756	Isoform 3 of Zinc transporter ZIP12	SLC39A12	8
		IPI00293303	Legumain precursor	LGMN	8
		IPI00292732	fibromodulin precursor	FMOD	8
		IPI00827650	Isoform 3 of CD44 antigen precursor	CD44	8
		IPI00021552	UDP-GalNAc:beta-1,3-N-acetylgalactosaminyltransferase 1	B3GALNT1	8
		IPI00028614	erythrocyte membrane protein band 4.2 isoform 2	EPB42	8
		IPI00004962	Golgi integral membrane protein 4	GOLIM4	8
		IPI00440580	Isoform 1 of Glycoprotein endo-alpha-1,2-mannosidase-like protein	MANEAL	8
		IPI00375205	Isoform 1 of Polypeptide N-acetylgalactosaminyltransferase 10	GALNT10	8
		IPI00432592	126 kDa protein	-	8
	√*	IPI00031769	Esophageal cancer-related gene 4 protein precursor	C2orf40	7
	√*	IPI00045841	Isoform 1 of Low-density lipoprotein receptor-related protein 11 precursor	LRP11	7
√	√	IPI00025864	Cholinesterase precursor	BCHE	7
√	√	IPI00006644	Isoform 2 of Plexin-B1 precursor	PLXNB1	7
√	√	IPI00218493	Hypoxanthine-guanine phosphoribosyltransferase	HPRT1	7
√	√	IPI00022391	Serum amyloid P-component precursor	APCS	7
√	√	IPI00333140	Delta and Notch-like epidermal growth factor-related receptor precursor	DNER	7
√	√	IPI00027466	Carbonic anhydrase 4 precursor	CA4	7
√	√	IPI00019038	Lysozyme C precursor	LYZ	7
√	√	IPI00216298	Thioredoxin	TXN	7
√	√	IPI00029193	Hepatocyte growth factor activator precursor	HGFAC	7
√	√	IPI00165972	Complement factor D preproprotein	CFD	7
√	√	IPI00745313	adipocyte enhancer binding protein 1 precursor	AEBP1	7
√	√	IPI00001734	Isoform 1 of Phosphoserine aminotransferase	PSAT1	7
√	√	IPI00023648	Immunoglobulin superfamily containing leucine-rich repeat protein precursor	ISLR	7
√	√	IPI00006128	Testican-2 precursor	SPOCK2	7
√	√	IPI00299503	Isoform 1 of Phosphatidylinositol-glycan-specific phospholipase D precursor	GPLD1	7
√	√	IPI00022792	Microfibril-associated glycoprotein 4 precursor	MFAP4	7
√	√	IPI00328550	Thrombospondin-4 precursor	THBS4	7
√	√	IPI00000138	Alpha-1,3-mannosyl-glycoprotein 2-beta-N-acetylglucosaminyltransferase	MGAT1	7

√	√	IPI00004946	chemokine (C-X-C motif) ligand 16	CXCL16	7
√	√	IPI00171473	Spondin-1 precursor	SPON1	7
√	√	IPI00216882	mannan-binding lectin serine protease 1 isoform 3	MASP1	7
√	√	IPI00019399	Serum amyloid A-4 protein precursor	SAA4	7
√	√	IPI00304865	transforming growth factor, beta receptor III	TGFBR3	7
√	√	IPI00005908	ADAMTS-1 precursor	ADAMTS1	7
√	√	IPI00024825	Isoform A of Proteoglycan-4 precursor	PRG4	7
√	√	IPI00168479	Isoform 1 of Apolipoprotein A-I-binding protein precursor	APOA1BP	7
√	√	IPI00398715	Neuropilin 1	NRP1	7
	√	IPI00028448	Brain-specific angiogenesis inhibitor 3 precursor	BAI3	7
	√	IPI00002236	Lactadherin precursor	MFGE8	7
	√	IPI00014964	Lymphocyte antigen 6H precursor	LY6H	7
	√	IPI00103471	Thioredoxin-like selenoprotein M precursor	SELM	7
	√	IPI00029046	Uncharacterized protein KIAA0152 precursor	KIAA0152	7
	√	IPI00791134	Calsyntenin 2	CLSTN2	7
	√	IPI00428511	Neurexin-1-beta precursor	NRXN1	7
	√	IPI00412988	Isoform 1 of Netrin-G1 precursor	NTNG1	7
	√	IPI00168884	Renin receptor precursor	ATP6AP2	7
√		IPI00465436	Catalase	CAT	7
√		IPI00246058	PDCD6IP protein	PDCD6IP	7
√		IPI00026270	Carboxypeptidase M precursor	CPM	7
√		IPI00290328	Receptor-type tyrosine-protein phosphatase eta precursor	PTPRJ	7
√		IPI00030075	Fibroleukin precursor	FGL2	7
√		IPI00296099	Thrombospondin-1 precursor	THBS1	7
√		IPI00003648	Isoform Delta of Poliovirus receptor-related protein 1 precursor	PVRL1	7
√		IPI00029605	N-acetylgalactosamine-6-sulfatase precursor	GALNS	7
√		IPI00216983	Carbonic anhydrase 3	CA3	7
√		IPI00025818	Isoform 1 of Polypeptide N-acetylgalactosaminyltransferase 1	GALNT1	7
√		IPI00178767	Acid sphingomyelinase-like phosphodiesterase 3a precursor	SMPDL3A	7
√		IPI00029273	Isoform 1 of Hepatocyte growth factor receptor precursor	MET	7
		IPI00026103	ACHE protein	ACHE	7
		IPI00016679	SLIT and NTRK-like protein 5 precursor	SLITRK5	7
		IPI00168626	Isoform 1 of Putative polypeptide N-acetylgalactosaminyltransferase-like protein 4	GALNTL4	7
		IPI00746388	Ezrin	EZR	7
		IPI00830047	Uncharacterized protein ENSP00000374858 (Fragment)	-	7
		IPI00478483	172 kDa protein	LAMC3	7
		IPI00171410	Isoform 1 of Uncharacterized protein C3orf21	C3orf21	7
		IPI00013569	Isoform 1 of Pappalysin-2 precursor	PAPPA2	7
		IPI00166892	DPPY splice variant c	DPP10	7
		IPI00021903	Isoform Alpha of ADAM 23 precursor	ADAM23	7

		IPI00017569	Fas apoptotic inhibitory molecule 2	FAIM2	7
		IPI00167215	Isoform 1 of Hepatocyte cell adhesion molecule precursor	HEPACAM	7
		IPI00789234	Immunoglobulin V-set domain containing protein	VSTM2A	7
		IPI00183321	Isoform 1 of N-acetylgalactosamine 4-sulfate 6-O-sulfotransferase	GALNAC4S-6S	7
		IPI00012283	Isoform 1 of Semaphorin-3B precursor	SEMA3B	7
	√*	IPI00301961	Neuroendocrine convertase 1 precursor	PCSK1	6
√	√	IPI00025846	Isoform 2A of Desmocollin-2 precursor	DSC2	6
√	√	IPI00011732	Isoform 1 of GDNF family receptor alpha-2 precursor	GFRA2	6
√	√	IPI00329482	Isoform 1 of Laminin subunit alpha-4 precursor	LAMA4	6
√	√	IPI00015049	Isoform 2 of Repulsive guidance molecule A precursor	RGMA	6
√	√	IPI00004503	lysosomal-associated membrane protein 1	LAMP1	6
√	√	IPI00018206	Aspartate aminotransferase, mitochondrial precursor	GOT2	6
√	√	IPI00015911	Dihydrolipoyl dehydrogenase, mitochondrial precursor	DLD	6
√	√	IPI00011140	Protein NOV homolog precursor	NOV	6
√	√	IPI00789954	7 kDa protein	TF	6
√	√	IPI00011605	Cerebellin-1 precursor	CBLN1	6
√	√	IPI00028553	Isoform 2 of Multiple inositol polyphosphate phosphatase 1 precursor	MINPP1	6
√	√	IPI00029061	Selenoprotein P precursor	SEPP1	6
√	√	IPI00301143	Isoform 1 of Peptidase inhibitor 16 precursor	PI16	6
√	√	IPI00060310	Phospholipase D4	PLD4	6
√	√	IPI00295414	Collagen alpha-1(XV) chain precursor	COL15A1	6
√	√	IPI00006154	Isoform Long of Complement factor H-related protein 2 precursor	CFHR2	6
√	√	IPI00168866	MAM domain containing glycosylphosphatidylinositol anchor 1	MDGA1	6
√	√	IPI00017704	Coactosin-like protein	COTL1	6
√	√	IPI00739827	Isoform LAMP-2B of Lysosome-associated membrane glycoprotein 2 precursor	LAMP2	6
√	√	IPI00301255	Immunoglobulin superfamily member 21 precursor	IGSF21	6
√	√	IPI00002525	Neudesin precursor	NENF	6
√	√	IPI00382500	Ig heavy chain V-III region GAL	-	6
√	√	IPI00479116	Carboxypeptidase N subunit 2 precursor	CPN2	6
√	√	IPI00009030	Isoform LAMP-2A of Lysosome-associated membrane glycoprotein 2 precursor	LAMP2	6
√	√	IPI00022892	Thy-1 membrane glycoprotein precursor	THY1	6
√	√	IPI00216602	Isoform 5 of Fibroblast growth factor receptor 2 precursor	FGFR2	6
√	√	IPI00784258	latent transforming growth factor beta binding protein 1 isoform LTBP-1L	LTBP1	6
√	√	IPI00386630	TCN2 protein	TCN2	6
	√	IPI00745251	Mannosyl-oligosaccharide 1,2-alpha-mannosidase IC	MAN1C1	6
	√	IPI00008207	Endoplasmic reticulum mannosyl-oligosaccharide 1,2-alpha-mannosidase	MAN1B1	6
	√	IPI00024601	Carbonic anhydrase-related protein 10	CA10	6
	√	IPI00024034	Cadherin-4 precursor	CDH4	6
	√	IPI00011994	Ectonucleotide pyrophosphatase/phosphodiesterase family member 5 precursor	ENPP5	6
	√	IPI00002925	Cocaine- and amphetamine-regulated transcript protein precursor	CARTPT	6

	√	IPI00176424	Neuroligin-2 precursor	NLGN2	6
	√	IPI00337351	MAM domain-containing glycosylphosphatidylinositol anchor protein 2 precursor	MDGA2	6
	√	IPI00003907	Isoform 1 of Protocadherin gamma C5 precursor	PCDHGC5;PCI	6
	√	IPI00060715	BTB/POZ domain-containing protein KCTD12	KCTD12	6
	√	IPI00402157	Cerebellin-3 precursor	CBLN3	6
	√	IPI00002142	Protocadherin-10 precursor	PCDH10	6
	√	IPI00299086	Syntenin-1	SDCBP	6
√		IPI00029275	Isoform 1 of Melanotransferrin precursor	MFI2	6
√		IPI00374065	similar to melanoma inhibitory activity 3 isoform 1	MIA3	6
√		IPI00306322	Collagen alpha-2(IV) chain precursor	COL4A2	6
√		IPI00030887	Tyrosine-protein kinase receptor TYRO3 precursor	TYRO3	6
√		IPI00719621	Isoform 1 of Plexin-A2 precursor	PLXNA2	6
√		IPI00387168	Isoform 1 of Proprotein convertase subtilisin/kexin type 9 precursor	PCSK9	6
√		IPI00004413	Tumor necrosis factor receptor superfamily member 21 precursor	TNFRSF21	6
√		IPI00027078	Carboxypeptidase D precursor	CPD	6
√		IPI00006971	Isoform 1 of Endosialin precursor	CD248	6
√		IPI00328257	Isoform A of AP-1 complex subunit beta-1	AP1B1	6
√		IPI00019502	Myosin-9	MYH9	6
√		IPI00025204	CD5 antigen-like precursor	CD5L	6
√		IPI00027462	Protein S100-A9	S100A9	6
√		IPI00477611	184 kDa protein	COL5A1	6
√		IPI00216319	14-3-3 protein eta	YWHAH	6
√		IPI00013897	ADAM 10 precursor	ADAM10	6
√		IPI00304840	Isoform 2C2 of Collagen alpha-2(VI) chain precursor	COL6A2	6
√		IPI00007750	Tubulin alpha-4A chain	TUBA4A	6
√		IPI00018534	Histone H2B type 1-L	HIST1H2BL	6
		IPI00016645	Isoform 1 of Ephrin type-A receptor 7 precursor	EPHA7	6
		IPI00102435	collagen, type XXI, alpha 1 precursor	COL21A1	6
		IPI00182438	Isoform 2 of Contactin-5 precursor	CNTN5	6
		IPI00791479	CDNA FLJ90299 fis, clone NT2RP2000514, highly similar to Homo sapiens roundabout 2	ROBO2	6
		IPI00299652	Isoform Long of ADAM 11 precursor	ADAM11	6
		IPI00854709	Uncharacterized protein ENSP00000374799 (Fragment)	-	6
		IPI00165975	Cysteine-rich flanking region, C-terminal domain containing protein	ISLR2	6
		IPI00789847	Protein	-	6
		IPI00642861	CDNA FLJ37558 fis, clone BRCOC1000087	CXorf36	6
		IPI00155729	Plexin-B3 precursor	PLXNB3	6
		IPI00409640	Isoform 1 of Lipolysis-stimulated lipoprotein receptor	LSR	6
		IPI00291807	C3 and PZP-like, alpha-2-macroglobulin domain containing 8	CPAMD8	6
		IPI00829590	Uncharacterized protein ENSP00000375044	-	6
		IPI00295399	Cadherin-10 precursor	CDH10	6

		IPI00453473	Histone H4	HIST2H4A;HIS	6
		IPI00303894	Protein FAM3A precursor	FAM3A	6
		IPI00555693	Isoform 3 of Testican-3 precursor	SPOCK3	6
√	√*	IPI00006166	Probable G-protein coupled receptor 37 precursor	GPR37	5
√	√*	IPI00220827	Thymosin beta-10	TMSB10	5
	√*	IPI00012075	C-type natriuretic peptide precursor	NPPC	5
√	√	IPI00292300	contactin associated protein-like 5	CNTNAP5	5
√	√	IPI00419966	Isoform 2 of Target of Nesh-SH3 precursor	ABI3BP	5
√	√	IPI00186903	Isoform 2 of Apolipoprotein-L1 precursor	APOL1	5
√	√	IPI00430291	Isoform Delta 2 of Calcium/calmodulin-dependent protein kinase type II delta chain	CAMK2D	5
√	√	IPI00019954	Cystatin-M precursor	CST6	5
√	√	IPI00328113	Fibrillin-1 precursor	FBN1	5
√	√	IPI00015351	Isoform 1 of UPF0424 protein C1orf128	C1orf128	5
√	√	IPI00032288	MANSC domain-containing protein 1 precursor	MANSC1	5
√	√	IPI00021856	Apolipoprotein C-II precursor	APOC2	5
√	√	IPI00056357	Uncharacterized protein C19orf10 precursor	C19orf10	5
√	√	IPI00329801	Annexin A5	ANXA5	5
√	√	IPI00020987	Prolargin precursor	PRELP	5
√	√	IPI00018274	Isoform 1 of Epidermal growth factor receptor precursor	EGFR	5
√	√	IPI00294615	Fibulin-5 precursor	FBLN5	5
√	√	IPI00029236	Insulin-like growth factor-binding protein 5 precursor	IGFBP5	5
√	√	IPI00003176	Serine protease HTRA1 precursor	HTRA1	5
√	√	IPI00295386	Carbonyl reductase [NADPH] 1	CBR1	5
√	√	IPI00219131	Isoform 1 of ICOS ligand precursor	ICOSLG	5
√	√	IPI00291006	Malate dehydrogenase, mitochondrial precursor	MDH2	5
√	√	IPI00289058	Ly-6/neurotoxin-like protein 1 precursor	LYNX1	5
√	√	IPI00032311	Lipopolysaccharide-binding protein precursor	LBP	5
√	√	IPI00023824	Fibulin-2 precursor	FBLN2	5
√	√	IPI00007797	Fatty acid-binding protein, epidermal	FABP5;FABP5I	5
√	√	IPI00375364	Isoform 3 of Chitotriosidase-1 precursor	CHIT1	5
√	√	IPI00289870	Isoform C of Protocadherin-7 precursor	PCDH7	5
√	√	IPI00005126	Ephrin-B2 precursor	EFNB2	5
√	√	IPI00031564	Uncharacterized protein C7orf24	C7orf24	5
√	√	IPI00034319	Isoform A of Protein CutA precursor	CUTA	5
√	√	IPI00021857	Apolipoprotein C-III precursor	APOC3	5
√	√	IPI00003102	Ciliary neurotrophic factor receptor alpha precursor	CNTFR	5
√	√	IPI00550363	Transgelin-2	TAGLN2	5
	√	IPI00259102	Mammalian ependymin-related protein 1 precursor	EPDR1	5
	√	IPI00017257	Cathepsin O precursor	CTSO	5
	√	IPI00019533	Chitinase-3-like protein 2 precursor	CHI3L2	5

	√	IPI00879309	Protein	NRXN2	5
	√	IPI00419595	Isoform 1 of Podocalyxin-like protein 2 precursor	PODXL2	5
	√	IPI00216138	Transgelin	TAGLN	5
	√	IPI00024048	Cadherin-15 precursor	CDH15	5
	√	IPI00642632	C7 protein	-	5
	√	IPI00289924	Alpha-2,8-sialyltransferase 8E	ST8SIA5	5
	√	IPI00328243	Phospholipase D3	PLD3	5
	√	IPI00031821	Integral membrane protein 2B	ITM2B	5
	√	IPI00023751	Growth/differentiation factor 8 precursor	MSTN	5
	√	IPI00043215	immunoglobulin superfamily, member 1 isoform 1	IGSF1	5
	√	IPI00412987	GMFB protein	GMFB	5
	√	IPI00302840	Sodium/potassium-transporting ATPase subunit alpha-3	ATP1A3	5
	√	IPI00022333	Brain-specific angiogenesis inhibitor 1 precursor	BAI1	5
√		IPI00385007	Putative uncharacterized protein DKFZp686A01208	AP1B1	5
√		IPI00023807	Semaphorin-4D precursor	SEMA4D	5
√		IPI00008215	NADP-dependent malic enzyme	ME1	5
√		IPI00007240	Coagulation factor XIII B chain precursor	F13B	5
√		IPI00292218	Hepatocyte growth factor-like protein precursor	MST1	5
√		IPI00007199	Protein Z-dependent protease inhibitor precursor	SERPINA10	5
√		IPI00019190	Myocilin precursor	MYOC	5
√		IPI00018305	Insulin-like growth factor-binding protein 3 precursor	IGFBP3	5
√		IPI00029606	Isoform B of ADAM 17 precursor	ADAM17	5
√		IPI00384391	Myosin-reactive immunoglobulin heavy chain variable region (Fragment)	-	5
√		IPI00026050	Ceroid-lipofuscinosis neuronal protein 5	CLN5	5
√		IPI00031510	Semaphorin-3A precursor	SEMA3A	5
√		IPI00016862	Isoform Mitochondrial of Glutathione reductase, mitochondrial precursor	GSR	5
√		IPI00021794	Lysosomal protective protein precursor	CTSA	5
√		IPI00021447	Alpha-amylase 2B precursor	AMY2B	5
√		IPI00008780	Stanniocalcin-2 precursor	STC2	5
√		IPI00296197	Nucleotide exchange factor SIL1 precursor	SIL1	5
√		IPI00009276	Endothelial protein C receptor precursor	PROCR	5
√		IPI00009145	Mannosyl-oligosaccharide 1,2-alpha-mannosidase IB	MAN1A2	5
√		IPI00005474	Phospholysine phosphohistidine inorganic pyrophosphate phosphatase	LHPP	5
√		IPI00030205	Ig kappa chain V-III region HAH precursor	IGKV3-20	5
√		IPI00027377	aggrecan isoform 2 precursor	ACAN	5
√		IPI00029997	6-phosphogluconolactonase	PGLS	5
√		IPI00329685	Putative uncharacterized protein DKFZp686G12235	ARSA	5
√		IPI00218570	Phosphoglycerate mutase 2	PGAM2	5
√		IPI00306710	Isoform 1 of Chordin precursor	CHRD	5
		IPI00431738	X-linked interleukin-1 receptor accessory protein-like 1 precursor	IL1RAPL1	5

IPI00307592	ATP-binding cassette, sub-family A, member 2 isoform a	ABCA2	5
IPI00410122	Isoform 1 of Plexin domain-containing protein 1 precursor	PLXDC1	5
IPI00550115	Isoform 1 of Acid sphingomyelinase-like phosphodiesterase 3b precursor	SMPDL3B	5
IPI00020672	Isoform 1 of Dipeptidyl-peptidase 3	DPP3;BBS1	5
IPI00027174	Isoform 1 of Fibroblast growth factor receptor 3 precursor	FGFR3	5
IPI00829701	Uncharacterized protein ENSP00000375014	-	5
IPI00006803	Carbohydrate sulfotransferase 10	CHST10	5
IPI00107886	Semaphorin 6B isoform 2	SEMA6B	5
IPI00171928	Angiopoietin-related protein 7 precursor	ANGPTL7	5
IPI00304331	Galactosylgalactosylxylosylprotein 3-beta-glucuronosyltransferase 3	B3GAT3	5
IPI00607655	Isoform 2 of Ephrin type-A receptor 7 precursor	EPHA7	5
IPI00816799	Rheumatoid factor D5 light chain (Fragment)	-	5
IPI00328745	Reticulon-4 receptor-like 1 precursor	RTN4RL1	5
IPI00011730	EMILIN-3 precursor	EMILIN3	5
IPI00783689	Immunglobulin heavy chain variable region (Fragment)	-	5
IPI00026237	Myelin-associated glycoprotein precursor	MAG	5
IPI00386133	Ig kappa chain V-IV region B17 precursor	-	5
IPI00296558	Carboxypeptidase-like protein X2 precursor	CPXM2	5
IPI00413912	Transmembrane protein 132E precursor	TMEM132E	5
IPI00063048	Isoform 2 of Beta-galactoside alpha-2,6-sialyltransferase 2	ST6GAL2	5
IPI00003021	Sodium/potassium-transporting ATPase subunit alpha-2 precursor	ATP1A2	5
IPI00027721	Isoform 1 of Alpha-type platelet-derived growth factor receptor precursor	PDGFRA	5
IPI00783287	Immunglobulin heavy chain variable region (Fragment)	-	5
IPI00013096	Isoform 1 of Receptor-type tyrosine-protein phosphatase T precursor	PTPRT	5
IPI00787265	similar to aminopeptidase puromycin sensitive	LOC729034	5
IPI00020906	Inositol monophosphatase	IMPA1	5
IPI00740545	similar to Prostate, ovary, testis expressed protein on chromosome 2 isoform 2	LOC653269	5
IPI00010348	Deoxyribonuclease-2-alpha precursor	DNASE2	5
IPI00294834	Aspartyl/asparaginyl beta-hydroxylase	ASPH	5
IPI00829947	13 kDa protein	-	5
IPI00165044	Isoform 2 of Uncharacterized protein C4orf18	C4orf18	5
IPI00021275	Isoform 1 of Ephrin type-B receptor 2 precursor	EPHB2	5
IPI00478892	Leucine-rich repeats and immunoglobulin-like domains protein 2 precursor	LRIG2	5
IPI00556287	Putative uncharacterized protein	-	5
IPI00854841	Uncharacterized protein ENSP00000375033	-	5
IPI00736885	Ig kappa chain V-II region TEW	LOC440786	5
IPI00016467	SLIT and NTRK-like protein 3 precursor	SLITRK3	5
IPI00827892	VH87-2 protein (Fragment)	-	5
IPI00479997	Stathmin	STMN1	5
IPI00790775	Isoform 3 of Voltage-dependent calcium channel subunit alpha-2/delta-3 precursor	CACNA2D3	5

		IPI00736860	ELK2, member of ETS oncogene family, pseudogene 1	ELK2P1	5
		IPI00017968	ADM precursor	ADM	5
		IPI00867665	Similar to Protein disulfide-isomerase precursor	-	5
		IPI00181174	Isoform 1 of Neuroligin-4, X-linked precursor	NLGN4X	5
		IPI00414467	collectin sub-family member 12	COLEC12	5
		IPI00010790	Biglycan precursor	BGN	5
		IPI00021983	Isoform 1 of Nicastrin precursor	NCSTN	5
		IPI00044743	Isoform 1 of Transmembrane protein 132B	TMEM132B	5
		IPI00005981	Transgelin-3	TAGLN3	5
		IPI00006482	Isoform Long of Sodium/potassium-transporting ATPase subunit alpha-1 precursor	ATP1A1	5
	√*	IPI00026174	Cholecystokinins precursor	CCK	4
√	√	IPI00744692	Transaldolase	TALDO1	4
√	√	IPI00794450	9 kDa protein	LYNX1	4
√	√	IPI00401283	Multiple epidermal growth factor-like domains 9 precursor	MEGF9	4
√	√	IPI00014439	Dihydropteridine reductase	QDPR	4
√	√	IPI00025840	Isoform 1 of Ephrin-A1 precursor	EFNA1	4
√	√	IPI00296058	EGF-containing fibulin-like extracellular matrix protein 2 precursor	EFEMP2	4
√	√	IPI00011302	CD59 glycoprotein precursor	CD59	4
√	√	IPI00021855	Apolipoprotein C-I precursor	APOC1	4
√	√	IPI00178926	immunoglobulin J chain	IGJ	4
√	√	IPI00015525	Multimerin-2 precursor	MMRN2	4
√	√	IPI00296992	AXL receptor tyrosine kinase isoform 1	AXL	4
√	√	IPI00302944	Isoform 4 of Collagen alpha-1(XII) chain precursor	COL12A1	4
√	√	IPI00784430	Similar to Ig kappa chain V-III region VG precursor	IGKV3D-11	4
√	√	IPI00305380	Insulin-like growth factor-binding protein 4 precursor	IGFBP4	4
√	√	IPI00029699	Ribonuclease 4 precursor	RNASE4	4
√	√	IPI00001893	Isoform A of Protocadherin-7 precursor	PCDH7	4
√	√	IPI00012386	Cochlin precursor	COCH	4
√	√	IPI00413451	Putative uncharacterized protein DKFZp686I04222	SERPINB6	4
√	√	IPI00007102	Uncharacterized protein C17orf25	GLOD4	4
√	√	IPI00643348	80 kDa protein	COMP	4
√	√	IPI00741710	Isoform 2 of Sushi, nidogen and EGF-like domain-containing protein 1 precursor	SNED1	4
	√	IPI00168520	Isoform 2 of Matrilin-2 precursor	MATN2	4
	√	IPI00153049	Isoform 2 of Matrix-remodeling-associated protein 8 precursor	MXRA8	4
	√	IPI00432525	Sialic acid-binding Ig-like lectin 14 precursor	SIGLEC14	4
	√	IPI00152850	junctional adhesion molecule 3 precursor	JAM3	4
	√	IPI00217882	Sortilin precursor	SORT1	4
	√	IPI00297188	Brain-specific angiogenesis inhibitor 2 precursor	BAI2	4
	√	IPI00099670	carboxyl ester lipase precursor	CEL	4
	√	IPI00219930	Cellular retinoic acid-binding protein 1	CRABP1	4

	√	IPI00387115	Ig kappa chain V-III region SIE	-	4
	√	IPI00024129	Peptidyl-prolyl cis-trans isomerase C	PPIC	4
	√	IPI00299699	Neural proliferation differentiation and control protein 1 precursor	NPDC1	4
	√	IPI00166339	Isoform 1 of Ephrin type-A receptor 10 precursor	EPHA10	4
	√	IPI00000824	Isoform A of NT-3 growth factor receptor precursor	NTRK3	4
	√	IPI00075248	Calmodulin	CALM3;CALM2	4
	√	IPI00880120	Abhydrolase domain-containing protein 14A	ABHD14A	4
√		IPI00442911	CDNA FLJ26266 fis, clone DMC05613	IGHV4-31	4
√		IPI00008494	Intercellular adhesion molecule 1 precursor	ICAM1	4
√		IPI00023858	Fc-gamma receptor IIIb	FCGR3B	4
√		IPI00027223	Isocitrate dehydrogenase [NADP] cytoplasmic	IDH1	4
√		IPI00410585	Isoform 1 of Crumbs homolog 2 precursor	CRB2	4
√		IPI00299724	Isoform 1 of Signal regulatory protein beta-1 precursor	SIRPB1	4
√		IPI00167710	Isoform 1 of Fibulin-7 precursor	FBLN7	4
√		IPI00218414	Carbonic anhydrase 2	CA2	4
√		IPI00465255	Isoform 1 of Proline-rich acidic protein 1 precursor	PRAP1	4
√		IPI00182944	Isoform 3 of Calcium/calmodulin-dependent protein kinase type II beta chain	CAMK2B	4
√		IPI00103871	Isoform 1 of Roundabout homolog 4 precursor	ROBO4	4
√		IPI00293128	Exostosin-1	EXT1	4
√		IPI00217236	Tubulin-specific chaperone A	TBCA	4
√		IPI00383732	VH3 protein (Fragment)	-	4
√		IPI00414717	golgi apparatus protein 1	GLG1	4
√		IPI00219684	Fatty acid-binding protein, heart	FABP3	4
√		IPI00027341	Macrophage-capping protein	CAPG	4
√		IPI00008223	UV excision repair protein RAD23 homolog B	RAD23B	4
√		IPI00414909	Alpha-N-acetylgalactosaminidase precursor	NAGA	4
√		IPI00021817	Vitamin K-dependent protein C precursor	PROC	4
√		IPI00437751	Isoform Somatic-1 of Angiotensin-converting enzyme, somatic isoform precursor	ACE	4
√		IPI00017567	Isoform Long of Endoglin precursor	ENG	4
√		IPI00219025	Glutaredoxin-1	GLRX	4
√		IPI00010706	Glutathione synthetase	GSS	4
√		IPI00257508	Dihydropyrimidinase-related protein 2	DPYSL2	4
√		IPI00293925	Isoform 1 of Ficolin-3 precursor	FCN3	4
√		IPI00031789	Isoform 1 of Interleukin-1 receptor accessory protein precursor	IL1RAP	4
√		IPI00218474	Beta-enolase	ENO3	4
√		IPI00101608	Isoform 2 of Cysteine-rich with EGF-like domain protein 1 precursor	CRELD1	4
√		IPI00216457	Histone H2A type 2-A	HIST2H2AA3;H	4
√		IPI00747849	Isoform 1 of Sodium/potassium-transporting ATPase subunit beta-1	ATP1B1	4
√		IPI00549330	Myosin-reactive immunoglobulin light chain variable region	IGKV3D-15	4
√		IPI00007425	desmocollin 1 isoform Dsc1b preproprotein	DSC1	4

√	IPI00020984	Calnexin precursor	CANX	4
√	IPI00554786	Thioredoxin reductase 1, cytoplasmic precursor	TXNRD1	4
√	IPI00019359	Keratin, type I cytoskeletal 9	KRT9	4
√	IPI00384404	Rheumatoid factor RF-ET9 (Fragment)	-	4
√	IPI00218834	Low affinity immunoglobulin gamma Fc region receptor III-A precursor	FCGR3A	4
√	IPI00017841	Isoform 1 of Noelin precursor	OLFM1	4
√	IPI00784154	60 kDa heat shock protein, mitochondrial precursor	HSPD1	4
√	IPI00006451	Vesicle-fusing ATPase	NSF	4
√	IPI00025476	Pancreatic alpha-amylase precursor	AMY2A;AMY1E	4
	IPI00827940	Mu-chain precursor (Fragment)	-	4
	IPI00829752	Uncharacterized protein ENSP00000375029	-	4
	IPI00749328	hypothetical protein	LOC729085	4
	IPI00871227	Isoform 1 of Hemicentin-1 precursor	HMCN1	4
	IPI00180707	Isoform 1 of FRAS1-related extracellular matrix protein 2 precursor	FREM2	4
	IPI00005652	Isoform 1 of WSC domain-containing protein 2	WSCD2	4
	IPI00009111	Trophoblast glycoprotein precursor	TPBG	4
	IPI00219301	Myristoylated alanine-rich C-kinase substrate	MARCKS	4
	IPI00007709	Isoform 1 of ADAM 28 precursor	ADAM28	4
	IPI00019209	Semaphorin-3C precursor	SEMA3C	4
	IPI00001506	Neuropeptide Y precursor	NPY	4
	IPI00854667	Uncharacterized protein ENSP00000375015	-	4
	IPI00827560	HRV Fab N27-VL (Fragment)	-	4
	IPI00007249	ectonucleotide pyrophosphatase/phosphodiesterase 4	ENPP4	4
	IPI00031534	Alpha-N-acetylgalactosaminide alpha-2,6-sialyltransferase 1	ST6GALNAC1	4
	IPI00217466	Histone H1.3	HIST1H1D	4
	IPI00643115	Stathmin 1/oncoprotein 18	STMN1	4
	IPI00217759	Isoform 1 of Alpha-(1,3)-fucosyltransferase 11	FUT11	4
	IPI00218345	Isoform 2 of Tubulin alpha-3C/D chain	TUBA3C;TUBA	4
	IPI00478890	Isoform 1 of Testican-3 precursor	SPOCK3	4
	IPI00011518	Isoform A of Beta-secretase 1 precursor	BACE1	4
	IPI00783184	Immunglobulin heavy chain variable region (Fragment)	-	4
	IPI00251507	Isoform IB of Synapsin-1	SYN1	4
	IPI00019501	Ephrin-B3 precursor	EFNB3	4
	IPI00432723	Isoform 1 of Xylosyltransferase 2	XYLT2	4
	IPI00644472	Isoform 2 of Haloacid dehalogenase-like hydrolase domain-containing protein 2	HDHD2	4
	IPI00217465	Histone H1.2	HIST1H1C	4
	IPI00012058	Brain-derived neurotrophic factor precursor	BDNF	4
	IPI00217467	Histone H1.4	HIST1H1E	4
	IPI00847670	Similar to Phosphoglycerate mutase 1	LOC440043	4
	IPI00021833	Isoform Long of Platelet-derived growth factor A chain precursor	PDGFA	4

		IPI00218539	Isoform B of Collagen alpha-1(XI) chain precursor	COL11A1	4
		IPI00300838	Carbohydrate sulfotransferase 8	CHST8	4
		IPI00006657	Protein FAM20B precursor	FAM20B	4
		IPI00033560	Isoform Alpha of Receptor-type tyrosine-protein phosphatase R precursor	PTPRR	4
		IPI00025809	Alpha-1,6-mannosyl-glycoprotein 2-beta-N-acetylglucosaminyltransferase	MGAT2	4
		IPI00026197	Similar to Ig kappa chain V-IV region precursor	IGKV4-1	4
		IPI00023576	Leucine-rich repeat transmembrane neuronal protein 2 precursor	LRRTM2	4
		IPI00215767	Isoform Long of Beta-1,4-galactosyltransferase 1	B4GALT1	4
		IPI00024094	Rhesus blood group-associated glycoprotein	RHAG	4
		IPI00827510	HRV Fab 026-VL (Fragment)	-	4
		IPI00829663	Uncharacterized protein ENSP00000374801	-	4
		IPI00883855	Similar to Hepatitis B virus receptor binding protein	-	4
		IPI00456589	Isoform 1 of Polypeptide N-acetylgalactosaminyltransferase 11	GALNT11	4
		IPI00024036	Cadherin-8 precursor	CDH8	4
		IPI00419720	Dermokine gamma-1	-	4
		IPI00387118	Ig kappa chain V-III region WOL	-	4
		IPI00291395	fibronectin leucine rich transmembrane protein 1	FLRT1	4
		IPI00022367	Isoform 2 of Astrotactin-1 precursor	ASTN1	4
		IPI00002307	Isoform 1 of Neuroligin-3 precursor	NLGN3	4
		IPI00001399	Adherens junction-associated protein 1	AJAP1	4
		IPI00006713	Isoform 1 of DnaJ homolog subfamily C member 3	DNAJC3	4
		IPI00827482	Uncharacterized protein ENSP00000348964 (Fragment)	-	4
		IPI00008107	Leucine-rich repeat and fibronectin type-III domain-containing protein 2 precursor	LRFN2	4
		IPI00783024	Myosin-reactive immunoglobulin heavy chain variable region (Fragment)	-	4
		IPI00162329	Isoform 1 of Transmembrane protein 25 precursor	TMEM25	4
		IPI00217345	Isoform 2 of UDP-GlcNAc:betaGal beta-1,3-N-acetylglucosaminyltransferase 2	B3GNT2	4
		IPI00023643	Sema domain, transmembrane domain (TM), and cytoplasmic domain, (Semaphorin) 6C	SEMA6C	4
		IPI00470484	Isoform 1 of EGF-like, fibronectin type-III and laminin G-like domain-containing protein pr	EGFLAM	4
		IPI00428967	Toll-like receptor adapter molecule 2	TICAM2;TMED	4
		IPI00455739	Isoform 1 of Beta-1,3-N-acetylglucosaminyltransferase lunatic fringe	LFNG	4
		IPI00171412	Isoform 1 of Sulfatase-modifying factor 2 precursor	SUMF2	4
√	√*	IPI00182138	Isoform 2 of Granulins precursor	GRN	3
	√*	IPI00016666	Metallothionein-3	MT3	3
	√*	IPI00029131	Neuroendocrine convertase 2 precursor	PCSK2	3
	√*	IPI00293723	Neurexophilin-4 precursor	NXPH4	3
	√*	IPI00002334	Neuron-specific protein family member 1	D4S234E	3
√	√	IPI00478414	Ventroptin (Fragment)	CHRDL1	3
√	√	IPI00019176	Retinoic acid receptor responder protein 2 precursor	RARRES2	3
√	√	IPI00382481	Ig heavy chain V-III region BUT	-	3
√	√	IPI00022977	Creatine kinase B-type	CKB	3

√	√	IPI00643667	C1q and tumor necrosis factor related protein 3 isoform b	C1QTNF3	3
√	√	IPI00219219	Galectin-1	LGALS1	3
√	√	IPI00293757	Isoform 1 of Netrin receptor UNC5C precursor	UNC5C	3
√	√	IPI00011094	Complement C1q tumor necrosis factor-related protein 4 precursor	C1QTNF4	3
√	√	IPI00018396	Cerebellin-4 precursor	CBLN4	3
√	√	IPI00465315	Cytochrome c	CYCS	3
√	√	IPI00026259	N	AGA	3
√	√	IPI00022394	Complement C1q subcomponent subunit C precursor	C1QC	3
√	√	IPI00024035	Isoform 1 of Cadherin-6 precursor	CDH6	3
√	√	IPI00022392	Complement C1q subcomponent subunit A precursor	C1QA	3
√	√	IPI00299150	Cathepsin S precursor	CTSS	3
√	√	IPI00004114	Ribonuclease K6 precursor	RNASE6	3
√	√	IPI00010896	Chloride intracellular channel protein 1	CLIC1	3
√	√	IPI00024273	Isoform Long of Very low-density lipoprotein receptor precursor	VLDLR	3
√	√	IPI00020977	Isoform 1 of Connective tissue growth factor precursor	CTGF	3
√	√	IPI00012011	Cofilin-1	CFL1	3
√	√	IPI00028931	Desmoglein-2 precursor	DSG2	3
√	√	IPI00328703	Out at first protein homolog precursor	OAF	3
√	√	IPI00030739	Apolipoprotein M	APOM	3
√	√	IPI00031549	Isoform 3A of Desmocollin-3 precursor	DSC3	3
√	√	IPI00293849	Receptor-type tyrosine-protein phosphatase mu precursor	PTPRM	3
√	√	IPI00385252	Ig kappa chain V-III region GOL	-	3
√	√	IPI00789477	Similar to Lactotransferrin precursor	LTF	3
√	√	IPI00219575	Bleomycin hydrolase	BLMH	3
	√	IPI00387120	Ig kappa chain V-IV region Len	-	3
	√	IPI00011662	Kunitz-type protease inhibitor 2 precursor	SPINT2	3
	√	IPI00026991	Polypeptide N-acetylgalactosaminyltransferase 6	GALNT6	3
	√	IPI00030111	Growth/differentiation factor 11 precursor	GDF11	3
	√	IPI00013162	Isoform 1 of OX-2 membrane glycoprotein precursor	CD200	3
	√	IPI00017745	Metalloproteinase inhibitor 4 precursor	TIMP4	3
	√	IPI00220361	Calbindin	CALB1	3
	√	IPI00374914	hypothetical protein	LOC401115	3
	√	IPI00024105	Complement C1q tumor necrosis factor-related protein 5 precursor	C1QTNF5	3
	√	IPI00470625	Neuritin precursor	NRN1	3
	√	IPI00307276	ADAMTS-4 precursor	ADAMTS4	3
√		IPI00383032	Isoform 2 of Hepatitis A virus cellular receptor 2 precursor	HAVCR2	3
√		IPI00219425	Isoform Beta of Poliovirus receptor precursor	PVR	3
√		IPI00012269	Multimerin-1 precursor	MMRN1	3
√		IPI00788824	Light chain Fab	IGLV1-44	3
√		IPI00022959	Isoform 1 of Poliovirus receptor-related protein 3 precursor	PVRL3	3

√	IPI00477714	V3-4 protein	IGLV8-61	3
√	IPI00019449	Non-secretory ribonuclease precursor	RNASE2	3
√	IPI00010182	Isoform a 1 of Acyl-CoA-binding protein	DBI	3
√	IPI00292496	Beta-tubulin 4Q	TUBB8	3
√	IPI00297655	Neurogenic locus notch homolog protein 2 precursor	NOTCH2	3
√	IPI00028015	Isoform 2 of Leukocyte-associated immunoglobulin-like receptor 1 precursor	LAIR1	3
√	IPI00385253	Ig kappa chain V-III region CLL precursor	-	3
√	IPI00179851	NDT80/PhoG like DNA-binding family protein	C11orf9	3
√	IPI00021347	Ubiquitin-conjugating enzyme E2 L3	UBE2L3	3
√	IPI00029928	Elastin	ELN	3
√	IPI00401264	Thioredoxin domain-containing protein 4 precursor	TXNDC4	3
√	IPI00015842	Reticulocalbin-1 precursor	RCN1	3
√	IPI00005222	Ephrin type-B receptor 6 precursor	EPHB6	3
√	IPI00440932	Isoform 1 of ADAM 9 precursor	ADAM9	3
√	IPI00184019	Isoform 3 of Paired immunoglobulin-like type 2 receptor alpha precursor	PILRA	3
√	IPI00414676	Heat shock protein HSP 90-beta	HSP90AB1	3
√	IPI00030871	Pantetheinase precursor	VNN1	3
√	IPI00022674	Isoform 1 of Oncostatin-M specific receptor subunit beta precursor	OSMR	3
√	IPI00000044	Platelet-derived growth factor B chain precursor	PDGFB	3
√	IPI00003799	Isoform 2 of Heme-binding protein 2	HEBP2	3
√	IPI00374732	similar to peptidylprolyl isomerase A isoform 1	PPIAP19	3
√	IPI00289876	Isoform 1 of Syntaxin-7	STX7	3
√	IPI00300725	Keratin, type II cytoskeletal 6A	KRT6A	3
√	IPI00018381	Isoform 1 of Tolloid-like protein 1 precursor	TLL1	3
√	IPI00028082	Reversion-inducing cysteine-rich protein with Kazal motifs precursor	RECK	3
√	IPI00009826	Carboxypeptidase B precursor	CPB1	3
√	IPI00220301	Peroxiredoxin-6	PRDX6	3
√	IPI00006130	Uncharacterized calcium-binding protein KIAA0494	KIAA0494	3
√	IPI00157454	Isoform 1 of Heparan-sulfate 6-O-sulfotransferase 2	HS6ST2	3
√	IPI00062037	Dynein light chain 2, cytoplasmic	DYNLL2	3
√	IPI00019755	Glutathione transferase omega-1	GSTO1	3
√	IPI00220362	10 kDa heat shock protein, mitochondrial	HSPE1	3
√	IPI00739099	Collagen alpha-2(V) chain precursor	COL5A2	3
√	IPI00220766	Lactoylglutathione lyase	GLO1	3
√	IPI00552852	V2-19 protein	IGLV3-27	3
√	IPI00107731	Isoform 6 of Osteoclast associated immunoglobulin-like receptor precursor	OSCAR	3
√	IPI00293464	DNA damage-binding protein 1	DDB1	3
√	IPI00018146	14-3-3 protein theta	YWHAQ	3
√	IPI00029817	Sialidase-1 precursor	NEU1	3
√	IPI00219129	Ribosyldihydronicotinamide dehydrogenase	NQO2	3

√	IPI00184094	UDP-GlcNAc:betaGal beta-1,3-N-acetylglucosaminyltransferase 8	B3GNT8	3
√	IPI00219910	22 kDa protein	-	3
√	IPI00398918	Putative uncharacterized protein DKFZp686I21167	DKFZp686O24	3
√	IPI00552267	Similar to V2-13 protein	IGLV3-19	3
√	IPI00031708	Fumarylacetoacetase	FAH	3
√	IPI00022774	Transitional endoplasmic reticulum ATPase	VCP	3
√	IPI00026240	ADP-ribosyl cyclase 2 precursor	BST1	3
√	IPI00005722	Tyrosine-protein kinase receptor	FLT3	3
√	IPI00169285	Putative phospholipase B-like 2 precursor	P76	3
√	IPI00396378	Isoform B1 of Heterogeneous nuclear ribonucleoproteins A2/B1	HNRNPA2B1	3
√	IPI00299758	Carbohydrate sulfotransferase 12	CHST12	3
√	IPI00007960	Isoform 1 of Periostin precursor	POSTN	3
√	IPI00387117	Ig kappa chain V-III region Ti	IGKV3D-20	3
√	IPI00005809	Serum deprivation-response protein	SDPR	3
√	IPI00008556	Isoform 1 of Coagulation factor XI precursor	F11	3
√	IPI00027463	Protein S100-A6	S100A6	3
√	IPI00297252	Isoform 1 of Extracellular sulfatase Sulf-2 precursor	SULF2	3
√	IPI00303476	ATP synthase subunit beta, mitochondrial precursor	ATP5B	3
√	IPI00410487	Isoform 1 of Twisted gastrulation protein homolog 1 precursor	TWSG1	3
√	IPI00025447	Elongation factor 1-alpha	EEF1A1	3
√	IPI00029168	Apolipoprotein	LPA	3
√	IPI00299145	Keratin, type II cytoskeletal 6C	KRT6C	3
	IPI00005123	Ephrin-A3 precursor	EFNA3	3
	IPI00297181	Cadherin-7 precursor	CDH7	3
	IPI00387119	Ig kappa chain V-III region POM	-	3
	IPI00030882	Isoform Flop of Glutamate receptor 2 precursor	GRIA2	3
	IPI00884080	Similar to Immunglobulin heavy chain variable region	-	3
	IPI00411656	Isoform 1 of Protein piccolo	PCLO	3
	IPI00375879	Uncharacterized protein KIAA1467	KIAA1467	3
	IPI00032050	WW domain-binding protein 2	WBP2	3
	IPI00026285	Sia-alpha-2,3-Gal-beta-1,4-GlcNAc-R:alpha 2,8-sialyltransferase	ST8SIA3	3
	IPI00402293	Arylsulfatase G precursor	ARSG	3
	IPI00384402	Myosin-reactive immunoglobulin kappa chain variable region (Fragment)	-	3
	IPI00829740	V2-6 protein	-	3
	IPI00006444	Isoform 1 of Sodium/potassium/calcium exchanger 2 precursor	SLC24A2	3
	IPI00024929	Adipocyte adhesion molecule precursor	ASAM	3
	IPI00020747	Sodium channel subunit beta-3 precursor	SCN3B	3
	IPI00829812	Uncharacterized protein ENSP00000375011	-	3
	IPI00293971	Sodium/potassium-transporting ATPase subunit beta-2	ATP1B2	3
	IPI00884092	Anti-HER3 scFv (Fragment)	-	3

IPI00022891	ADP/ATP translocase 1	SLC25A4	3
IPI00783393	Immunglobulin heavy chain variable region (Fragment)	-	3
IPI00176398	Isoform 1 of SLIT and NTRK-like protein 6 precursor	SLITRK6	3
IPI00017562	Isoform 2 of Latrophilin-2 precursor	LPHN2	3
IPI00010148	Brain-specific polypeptide PEP-19	PCP4	3
IPI00883879	Similar to Anti-IFN-G scFv	-	3
IPI00299399	Protein S100-B	S100B	3
IPI00022649	Isoform 1 of Solute carrier family 12 member 2	SLC12A2	3
IPI00001633	Leucine-rich repeat transmembrane protein FLRT2 precursor	FLRT2	3
IPI00171438	Thioredoxin domain-containing protein 5 precursor	TXNDC5;MUTE	3
IPI00376131	Similar to Leucine rich repeat neuronal 6C	LINGO3	3
IPI00005517	Ephrin-A5 precursor	EFNA5	3
IPI00827581	Variable immnoglobulin anti-estradiol heavy chain (Fragment)	-	3
IPI00827637	K light chain variable region (Fragment)	-	3
IPI00298650	ADAMTS-8 precursor	ADAMTS8	3
IPI00873863	Brain-derived neurotrophic factor transcript variant 5	BDNF	3
IPI00000760	N(G),N(G)-dimethylarginine dimethylaminohydrolase 2	DDAH2	3
IPI00102575	ATPase family, AAA domain containing 5	ATAD5	3
IPI00472754	Polycystic kidney disease 1-related protein	KIAA0319L	3
IPI00013701	Nociceptin precursor	PNOC	3
IPI00784368	Isoform 1 of Bifunctional heparan sulfate N-deacetylase/N-sulfotransferase 1	NDST1	3
IPI00884389	Similar to Immunglobulin heavy chain variable region	-	3
IPI00854644	Uncharacterized protein ENSP00000374805	-	3
IPI00016621	Adaptor-related protein complex 2, alpha 2 subunit variant (Fragment)	AP2A2	3
IPI00387116	Ig kappa chain V-III region NG9 precursor (Fragment)	-	3
IPI00384392	Myosin-reactive immunoglobulin heavy chain variable region (Fragment)	-	3
IPI00827876	Heavy chain Fab (Fragment)	-	3
IPI00018941	Calcitonin gene-related peptide 2 precursor	CALCB	3
IPI00883765	Similar to Immunglobulin heavy chain variable region	-	3
IPI00748265	Rheumatoid factor RF-ET13	-	3
IPI00006556	hypothetical protein LOC9865	KIAA0644	3
IPI00374590	cancer susceptibility candidate 4 isoform a	CASC4	3
IPI00018860	NKG2D ligand 2 precursor	ULBP2	3
IPI00556391	Actin-like protein (Fragment)	-	3
IPI00883711	Similar to Anti-(ED-B) scFV	-	3
IPI00441344	Beta-galactosidase precursor	GLB1	3
IPI00019862	butyrophilin, subfamily 2, member A1 isoform 2 precursor	BTN2A1	3
IPI00019600	Ubiquitin-conjugating enzyme E2 variant 2	UBE2V2	3
IPI00827829	HRV Fab N8-VL (Fragment)	-	3
IPI00829841	13 kDa protein	-	3

		IPI00031506	Potassium/sodium hyperpolarization-activated cyclic nucleotide-gated channel 1	HCN1	3
		IPI00293539	Isoform 2 of Cadherin-11 precursor	CDH11	3
		IPI00010470	Isoform SNAP-25b of Synaptosomal-associated protein 25	SNAP25	3
		IPI00011899	BMP and activin membrane-bound inhibitor homolog precursor	BAMBI	3
		IPI00024587	D1 dopamine receptor-interacting protein calcyon	CALY	3
		IPI00005516	Leucine-rich repeat-containing protein 4 precursor	LRRC4	3
		IPI00012510	EMILIN-2 precursor	EMILIN2	3
		IPI00217376	Isoform 1 of Sodium channel subunit beta-4 precursor	SCN4B	3
		IPI00553138	Vesicle-associated membrane protein 2	VAMP2	3
		IPI00020396	Isoform PACE4A-I of Proprotein convertase subtilisin/kexin type 6 precursor	PCSK6	3
		IPI00000832	Beta-neoendorphin-dynorphin precursor	PDYN	3
		IPI00760721	13 kDa protein	-	3
		IPI00465377	Isoform 1 of Matrix-remodeling-associated protein 7	MXRA7	3
		IPI00828105	Anti-Mpl scFv (Fragment)	-	3
		IPI00743194	Kappa light chain variable region (Fragment)	-	3
		IPI00387024	Ig kappa chain V-I region CAR	-	3
		IPI00444605	CDNA FLJ45296 fis, clone BRHIP3003340, moderately similar to Actin, alpha skeletal mu	-	3
		IPI00304577	Isoform A of AP-2 complex subunit alpha-1	AP2A1	3
		IPI00301364	Isoform 1 of S-phase kinase-associated protein 1A	SKP1	3
		IPI00030847	Transmembrane 9 superfamily member 3 precursor	TM9SF3	3
		IPI00008533	Isoform Long of Matrix metalloproteinase-17 precursor	MMP17	3
		IPI00657742	Major histocompatibility complex, class I, F	HLA-F	3
		IPI00218667	Stathmin-2	STMN2	3
		IPI00008997	WAP four-disulfide core domain protein 1 precursor	WFDC1	3
		IPI00165125	Isoform 1 of Uncharacterized protein C14orf37 precursor	C14orf37	3
		IPI00012048	Nucleoside diphosphate kinase A	NME2;NME1	3
		IPI00418960	Isoform 3 of Protein NDRG4	NDRG4	3
		IPI00168847	Isoform 2 of Hyaluronidase-1 precursor	HYAL1	3
		IPI00168921	Putative polypeptide N-acetylgalactosaminyltransferase-like protein 3	WBSCR17	3
		IPI00184851	Type 2 lactosamine alpha-2,3-sialyltransferase	ST3GAL6	3
		IPI00027507	Complement factor H-related protein 3 precursor	CFHR3	3
		IPI00081836	Histone H2A type 1-H	HIST1H2AH	3
		IPI00397645	Isoform 2 of Matrix-remodeling-associated protein 7	MXRA7	3
	√*	IPI00220748	Isoform Alpha-7X1A of Integrin alpha-7 precursor	ITGA7	2
	√*	IPI00019771	Fractalkine precursor	CX3CL1	2
	√*	IPI00016014	Isoform 1 of Integral membrane protein 2C	ITM2C	2
	√*	IPI00301180	Isoform 2 of Solute carrier family 12 member 5	SLC12A5	2
√	√	IPI00005038	Ribonuclease UK114	HRSP12	2
√	√	IPI00010402	Putative uncharacterized protein	SH3BGRL3	2
√	√	IPI00303161	Endothelial cell-selective adhesion molecule precursor	ESAM	2

√	√	IPI00022389	Isoform 1 of C-reactive protein precursor	CRP	2
√	√	IPI00419442	IGLV6-57 protein	IGLV6-57	2
√	√	IPI00004573	Polymeric immunoglobulin receptor precursor	PIGR	2
√	√	IPI00257882	Xaa-Pro dipeptidase	PEPD	2
√	√	IPI00293276	Macrophage migration inhibitory factor	MIF	2
√	√	IPI00025812	Carbonic anhydrase-related protein 11 precursor	CA11	2
√	√	IPI00291488	Isoform 1 of WAP four-disulfide core domain protein 2 precursor	WFDC2	2
√	√	IPI00382478	Ig heavy chain V-III region TIL	-	2
√	√	IPI00007047	Protein S100-A8	S100A8	2
√	√	IPI00413641	Aldose reductase	AKR1B1	2
√	√	IPI00027444	Leukocyte elastase inhibitor	SERPINB1	2
√	√	IPI00299083	Junctional adhesion molecule B precursor	JAM2	2
√	√	IPI00299547	Neutrophil gelatinase-associated lipocalin precursor	LCN2	2
√	√	IPI00170814	PTK7 protein tyrosine kinase 7 isoform b precursor	PTK7	2
	√	IPI00009890	Glia-derived nexin precursor	SERPINE2	2
	√	IPI00219468	Isoform IIa of Profilin-2	PFN2	2
	√	IPI00413344	Cofilin-2	CFL2	2
	√	IPI00008586	Isoform 1 of Chondroitin sulfate proteoglycan 5 precursor	CSPG5	2
	√	IPI00006524	Uncharacterized protein KIAA0319 precursor	KIAA0319	2
	√	IPI00026800	Scrapie-responsive protein 1 precursor	SCRG1	2
	√	IPI00216461	Acylphosphatase-2	ACYP2	2
	√	IPI00735451	Uncharacterized protein ENSP00000375035	-	2
	√	IPI00075013	Complement C1q tumor necrosis factor-related protein 1 precursor	C1QTNF1	2
	√	IPI00006009	Isoform 2 of Pleckstrin homology domain-containing family B member 1	PLEKHB1	2
	√	IPI00329332	Syntaxin-12	STX12	2
	√	IPI00384400	Myosin-reactive immunoglobulin heavy chain variable region (Fragment)	-	2
	√	IPI00033466	C-type lectin domain family 11 member A precursor	CLEC11A	2
	√	IPI00289926	leukocyte immunoglobulin-like receptor, subfamily B, member 4 isoform 2	LILRB4	2
	√	IPI00016371	Isoform JM-A of Receptor tyrosine-protein kinase erbB-4 precursor	ERBB4	2
√		IPI00289862	Secernin-1	SCRN1	2
√		IPI00013945	Isoform 1 of Uromodulin precursor	UMOD	2
√		IPI00465261	Isoform 1 of Endoplasmic reticulum aminopeptidase 2	ERAP2	2
√		IPI00215997	CD9 antigen	CD9	2
√		IPI00382476	Ig heavy chain V-III region WEA	-	2
√		IPI00024307	Ephrin-B1 precursor	EFNB1	2
√		IPI00294713	Isoform 1 of Mannan-binding lectin serine protease 2 precursor	MASP2	2
√		IPI00554752	cAMP-dependent protein kinase type II-beta regulatory subunit	PRKAR2B	2
√		IPI00306844	Corticotropin-releasing factor-binding protein precursor	CRHBP	2
√		IPI00384016	Full-length cDNA 5-PRIME end of clone CS0DJ009YL13 of T cells (Jurkat cell line) of Ho	DLST	2
√		IPI00477868	LAMA5 protein	LAMA5	2

√	IPI00022640	Neurogranin	NRGN	2
√	IPI00644346	ADAMTS-like protein 2 precursor	ADAMTSL2	2
√	IPI00027847	Lipoprotein lipase precursor	LPL	2
√	IPI00009867	Keratin, type II cytoskeletal 5	KRT5	2
√	IPI00002966	Heat shock 70 kDa protein 4	HSPA4	2
√	IPI00029756	Proto-oncogene tyrosine-protein kinase MER precursor	MERTK	2
√	IPI00007853	Gamma-interferon-inducible lysosomal thiol reductase precursor	IFI30	2
√	IPI00465322	Uncharacterized protein BOC	BOC	2
√	IPI00103755	Isoform 2 of Netrin receptor UNC5D precursor	UNC5D	2
√	IPI00550533	Isoform 1 of Uncharacterized protein C1orf56 precursor	C1orf56	2
√	IPI00002283	Isoform 2 of Patched domain-containing protein 2	PTCHD2	2
√	IPI00419565	Isoform 1 of Stabilin-1 precursor	STAB1	2
√	IPI00029623	Proteasome subunit alpha type-6	PSMA6	2
√	IPI00171199	Isoform 2 of Proteasome subunit alpha type-3	PSMA3	2
√	IPI00149097	Semaphorin-4A precursor	SEMA4A	2
√	IPI00021834	Isoform Alpha of Tissue factor pathway inhibitor precursor	TFPI	2
√	IPI00001610	Insulin-like growth factor IA precursor	IGF1	2
√	IPI00001592	Isoform 2 of Transmembrane glycoprotein NMB precursor	GPNMB	2
√	IPI00382682	Putative matrix cell adhesion molecule-3	-	2
√	IPI00061354	Isoform 2 of Bromodomain adjacent to zinc finger domain protein 2B	BAZ2B	2
√	IPI00009054	Isoform BMP1-3 of Bone morphogenetic protein 1 precursor	BMP1	2
√	IPI00297779	T-complex protein 1 subunit beta	CCT2	2
√	IPI00168920	collagen, type XXIV, alpha 1	COL24A1	2
√	IPI00215980	Isoform Alpha of Poliovirus receptor-related protein 2 precursor	PVRL2	2
√	IPI00100154	Toll-interacting protein	TOLLIP	2
√	IPI00021997	Protein CREG1 precursor	CREG1	2
√	IPI00418169	annexin A2 isoform 1	ANXA2	2
√	IPI00012315	Nucleoside diphosphate kinase 3	NME3	2
√	IPI00552943	V1-11 protein	IGLV1-36	2
√	IPI00008726	Iron-responsive element-binding protein 2	IREB2	2
√	IPI00021727	C4b-binding protein alpha chain precursor	C4BPA	2
√	IPI00411706	S-formylglutathione hydrolase	ESD	2
√	IPI00027972	Isoform 1 of Leukocyte immunoglobulin-like receptor subfamily A member 2 precursor	LILRA2	2
√	IPI00383951	Isoform 3 of Protein sidekick-1 precursor	SDK1	2
√	IPI00099650	Protein jagged-1 precursor	JAG1	2
√	IPI00008756	Isoform 1 of Bullous pemphigoid antigen 1, isoforms 1/2/3/4/5/8 (Fragment)	DST	2
√	IPI00008164	Prolyl endopeptidase	PREP	2
√	IPI00002511	Cyclic AMP-dependent transcription factor ATF-6 alpha	ATF6	2
√	IPI00009901	Nuclear transport factor 2	NUTF2	2
√	IPI00009477	Intercellular adhesion molecule 2 precursor	ICAM2	2

√	IPI00012792	Cadherin-5 precursor	CDH5	2
√	IPI00412492	Isoform 1 of Plexin-D1 precursor	PLXND1	2
√	IPI00178352	Isoform 1 of Filamin-C	FLNC	2
√	IPI00413587	Isoform 1 of BH3-interacting domain death agonist	BID	2
√	IPI00017334	Prohibitin	PHB	2
√	IPI00044600	VPS10 domain-containing receptor SorCS2 precursor	SORCS2	2
√	IPI00012007	Adenosylhomocysteinase	AHCY	2
√	IPI00441498	Folate receptor alpha precursor	FOLR1	2
√	IPI00004457	Membrane copper amine oxidase	AOC3	2
√	IPI00382493	Ig heavy chain V-III region WAS	-	2
√	IPI00337612	Discoidin, CUB and LCCL domain-containing protein 1 precursor	DCBLD1	2
√	IPI00024466	UDP-glucose ceramide glucosyltransferase-like 1 isoform 1	UGCGL1	2
√	IPI00012009	Isoform 1 of Granulocyte-macrophage colony-stimulating factor receptor alpha chain prec	CSF2RA	2
√	IPI00217963	Keratin, type I cytoskeletal 16	KRT16	2
√	IPI00382488	Ig heavy chain V-III region HIL	-	2
√	IPI00022445	Platelet basic protein precursor	PPBP	2
√	IPI00006510	Tubulin beta-1 chain	TUBB1	2
√	IPI00000914	Isoform 1 of Calcitonin precursor	CALCA	2
√	IPI00299116	Podocalyxin-like protein 1 precursor	PODXL	2
√	IPI00400935	Isoform 1 of Collagen alpha-1(XVI) chain precursor	COL16A1	2
√	IPI00783156	Bone morphogenetic protein receptor type-2 precursor	BMPR2	2
√	IPI00176581	Isoform 1 of Fanconi anemia group M protein	FANCM	2
√	IPI00023191	Target of myb1	TOM1	2
√	IPI00217005	Ankyrin repeat domain-containing protein 18A	ANKRD18A	2
√	IPI00219682	Erythrocyte band 7 integral membrane protein	STOM	2
√	IPI00029700	Isoform Long of Down syndrome cell adhesion molecule precursor	DSCAM	2
√	IPI00024919	Thioredoxin-dependent peroxide reductase, mitochondrial precursor	PRDX3	2
√	IPI00022039	Isoform 3 of SLAM family member 5 precursor	CD84	2
√	IPI00004047	Isoform 1 of Exostosin-2	EXT2	2
√	IPI00387106	Ig kappa chain V-I region Ni	-	2
√	IPI00305975	Spondin-2 precursor	SPON2	2
√	IPI00440493	ATP synthase subunit alpha, mitochondrial precursor	ATP5A1	2
√	IPI00419237	Isoform 1 of Cytosol aminopeptidase	LAP3	2
√	IPI00024067	Isoform 1 of Clathrin heavy chain 1	CLTC	2
√	IPI00218795	L-selectin precursor	SELL	2
√	IPI00382482	Ig heavy chain V-III region CAM	-	2
√	IPI00292657	NADP-dependent leukotriene B4 12-hydroxydehydrogenase	LTB4DH	2
√	IPI00216694	plastin 3	PLS3	2
√	IPI00384395	Myosin-reactive immunoglobulin heavy chain variable region	-	2
√	IPI00023754	Protein kinase C-binding protein NELL1 precursor	NELL1	2

√	IPI00019904	Isoform 1 of Beta-adducin	ADD2	2
	IPI00829827	Uncharacterized protein ENSP00000374804	-	2
	IPI00219806	Protein S100-A7	S100A7	2
	IPI00873344	N8 protein long isoform (Fragment)	TPD52	2
	IPI00552939	Isoform 1 of Complement C1q-like protein 3 precursor	C1QL3	2
	IPI00827939	Anti-mucin1 light chain variable region (Fragment)	-	2
	IPI00065312	N-acetyl-beta-glucosaminyl-glycoprotein 4-beta-N- acetylgalactosaminyltransferase 1	B4GALNT4	2
	IPI00152524	Isoform 3 of Neuropilin and tolloid-like protein 1 precursor	NETO1	2
	IPI00182194	Teneurin-2	ODZ2	2
	IPI00165936	Isoform A of Chloride intracellular channel 6	CLIC6	2
	IPI00783818	Immunglobulin heavy chain variable region (Fragment)	-	2
	IPI00242905	Uncharacterized protein ENSP00000344689 (Fragment)	-	2
	IPI00022890	Ig lambda chain V region 4A precursor	IGLV7-43	2
	IPI00026546	Platelet-activating factor acetylhydrolase IB subunit beta	PAFAH1B2	2
	IPI00293460	ATP-binding cassette sub-family A member 1	ABCA1	2
	IPI00032227	Isoform 1 of Rabphilin-3A	RPH3A	2
	IPI00064377	Tumor necrosis factor receptor superfamily member 19L precursor	RELT	2
	IPI00816775	F5-20 (Fragment)	-	2
	IPI00298476	Isoform 1 of Gremlin-1 precursor	GREM1	2
	IPI00830035	Similar to Anti-streptococcal/anti-myosin immunoglobulin kappa light chain variable regior	-	2
	IPI00002320	Leucine-rich repeat transmembrane protein FLRT3 precursor	FLRT3	2
	IPI00166613	Isoform 1 of Putative polypeptide N-acetylgalactosaminyltransferase-like protein 1	GALNTL1	2
	IPI00001120	CDNA FLJ31810 fis, clone NT2RI2009289, weakly similar to CARBOXYPEPTIDASE N 8	LINGO2	2
	IPI00382442	Ig lambda chain V-V region DEL	-	2
	IPI00008202	Headcase protein homolog	HECA	2
	IPI00024580	Methylcrotonoyl-CoA carboxylase subunit alpha, mitochondrial precursor	MCCC1	2
	IPI00328431	Isoform 1 of Netrin receptor UNC5B precursor	UNC5B	2
	IPI00829834	Ig kappa chain V-III region VH precursor	-	2
	IPI00306576	Arylsulfatase B precursor	ARSB	2
	IPI00854624	Uncharacterized protein ENSP00000375043	-	2
	IPI00003391	Teneurin-1	ODZ1	2
	IPI00178727	Novel protein	C10orf79	2
	IPI00383680	Ribophorin II	RPN2	2
	IPI00000871	Prolactin	PRL	2
	IPI00181079	Meteorin-like protein precursor	METRNL	2
	IPI00020884	Plexin-A3 precursor	PLXNA3	2
	IPI00008148	Isoform 1 of GDNF family receptor alpha-1 precursor	GFRA1	2
	IPI00018246	Isoform 1 of Hexokinase-1	HK1	2
	IPI00827839	VK3 protein (Fragment)	-	2
	IPI00640292	Isoform 1 of Protein G7c precursor	C6orf27	2

IPI00396930	Uncharacterized protein ENSP00000353216 (Fragment)	-	2
IPI00829810	Uncharacterized protein ENSP00000375027	-	2
IPI00001872	Isoform 1 of Protocadherin gamma C3 precursor	PCDHGC3;PCI	2
IPI00175989	Rho family guanine-nucleotide exchange factor	MCF2L2	2
IPI00432766	Isoform 2 of Netrin-G2 precursor	NTNG2	2
IPI00300623	Pro-MCH precursor	PMCH	2
IPI00011865	Isoform 2 of Platelet-derived growth factor D precursor	PDGFD	2
IPI00015479	UPF0454 protein C12orf49 precursor	C12orf49	2
IPI00294619	Protein TFG	TFG	2
IPI00003441	Isoform 1 of Protein C1orf9 precursor	C1orf9	2
IPI00043810	Isoform 1 of Proline-rich transmembrane protein 1	PRRT1	2
IPI00024976	Mitochondrial import receptor subunit TOM22 homolog	TOMM22	2
IPI00550949	Bone morphogenetic protein 7 precursor	BMP7	2
IPI00006034	Cysteine-rich protein 2	CRIP2	2
IPI00297180	Cadherin-9 precursor	CDH9	2
IPI00003527	Ezrin-radixin-moesin-binding phosphoprotein 50	SLC9A3R1	2
IPI00061520	hypothetical protein LOC84752	MGC4655	2
IPI00219420	Structural maintenance of chromosomes protein 3	SMC3	2
IPI00005774	Isoform 1 of Low-density lipoprotein receptor-related protein 8 precursor	LRP8	2
IPI00879950	15 kDa protein	-	2
IPI00004494	Semaphorin-3E precursor	SEMA3E	2
IPI00396383	Isoform 1 of von Willebrand factor A domain-containing protein 1 precursor	VWA1	2
IPI00454695	Histone H2B type 2-C	HIST2H2BC	2
IPI00031718	Isoform 1 of Ectonucleoside triphosphate diphosphohydrolase 4	ENTPD4	2
IPI00021119	Carbohydrate sulfotransferase 1	CHST1	2
IPI00004488	Vacuolar proton pump subunit F	ATP6V1F	2
IPI00514517	V4-1 protein	IGLV5-37	2
IPI00009439	Synaptotagmin-1	SYT1	2
IPI00008504	Carbonic anhydrase 14 precursor	CA14	2
IPI00789259	V1-13 protein (Fragment)	IGLV1-40	2
IPI00017557	Secreted frizzled-related protein 4 precursor	SFRP4	2
IPI00014223	Netrin-G1 ligand precursor	LRRC4C	2
IPI00216699	Isoform 2 of Unc-112-related protein 2	FERMT3	2
IPI00044707	Hyaluronan and proteoglycan link protein 4 precursor	HAPLN4;TM6S	2
IPI00216348	Isoform 2C of Cytoplasmic dynein 1 intermediate chain 2	DYNC1I2	2
IPI00176104	Isoform 1 of SLIT and NTRK-like protein 2 precursor	SLITRK2	2
IPI00328826	Uncharacterized protein CADPS2	CADPS2	2
IPI00151036	RING finger protein 13	RNF13	2
IPI00020407	Alpha-1,6-mannosylglycoprotein 6-beta-N-acetylglucosaminyltransferase A	MGAT5	2
IPI00289802	Isoform 1 of CUB and sushi domain-containing protein 2	CSMD2	2

IPI00328587	Enolase	ENO1P	2
IPI00387105	Ig kappa chain V-I region Mev	-	2
IPI00396961	Leucine-rich repeat and fibronectin type-III domain-containing protein 5 precursor	LRFN5	2
IPI00024689	Aquaporin-1	AQP1	2
IPI00741608	similar to eukaryotic translation initiation factor 5A	EIF5AL3	2
IPI00414294	hypothetical protein	LOC729956	2
IPI00019907	Glypican-3 precursor	GPC3	2
IPI00328680	Multiple coagulation factor deficiency protein 2 precursor	MCFD2	2
IPI00026031	Uncharacterized protein C6orf72 precursor	C6orf72	2
IPI00219067	Glutathione S-transferase Mu 2	GSTM2	2
IPI00410079	Isoform 1 of Protein FAM82C	FAM82C	2
IPI00827929	VH-3 family (VH26)D/J protein (Fragment)	-	2
IPI00019812	Serine/threonine-protein phosphatase 5	PPP5C	2
IPI00010343	Sodium/calcium exchanger 2 precursor	SLC8A2	2
IPI00023942	Isoform 2 of Syndecan-3	SDC3	2
IPI00305833	Smu-1 suppressor of mec-8 and unc-52 protein homolog	SMU1	2
IPI00550746	Nuclear migration protein nudC	NUDC	2
IPI00003470	Ig kappa chain V-I region Wes	-	2
IPI00028387	Isoform 1 of Uncharacterized protein C20orf116 precursor	C20orf116	2
IPI00009396	Isoform 1 of Cannabinoid receptor 1	CNR1	2
IPI00828156	NANUC-1 heavy chain (Fragment)	-	2
IPI00181743	Isoform 1 of BAI1-associated protein 3	BAIAP3	2
IPI00426727	Isoform 1 of Methyl-CpG-binding domain protein 4	MBD4	2
IPI00552937	NHL repeat containing 3 isoform a	NHLRC3	2
IPI00787936	similar to cathepsin L-like protein	LOC644021	2
IPI00031907	Isoform 1 of Transmembrane protein 108 precursor	TMEM108	2
IPI00018879	Alpha-L-iduronidase precursor	IDUA	2
IPI00399089	Mesoderm development candidate 2	MESDC2	2
IPI00387097	Ig kappa chain V-I region Lay	-	2
IPI00007812	Vacuolar ATP synthase subunit B, brain isoform	ATP6V1B2	2
IPI00170692	Vesicle-associated membrane protein-associated protein A	VAPA	2
IPI00854707	Immunglobulin heavy chain variable region (Fragment)	-	2
IPI00788258	similar to lysyl oxidase-like 1 preproprotein	LOXL1	2
IPI00410675	Syntaxin-1B	STX1B	2
IPI00045536	Isoform 3 of Chitinase domain-containing protein 1 precursor	CHID1	2
IPI00019180	Glypican-5 precursor	GPC5	2
IPI00008085	Zinc transporter ZIP10 precursor	SLC39A10	2
IPI00329538	Prostasin precursor	PRSS8	2
IPI00030634	Isoform 1 of Gamma-glutamyltransferase 4 precursor	GGTL3	2
IPI00550677	WSC domain-containing protein 1	WSCD1	2

		IPI00029050	Isoform 1 of Glycosyltransferase-like protein LARGE1	LARGE	2
		IPI00328520	Isoform 2 of Proline-rich transmembrane protein 2	PRRT2	2
		IPI00749514	attractin-like 1	ATRNL1	2
		IPI00220281	Guanine nucleotide-binding protein G(o) subunit alpha 1	GNAO1	2
		IPI00000087	Sodium channel subunit beta-2 precursor	SCN2B	2
		IPI00010810	Electron transfer flavoprotein subunit alpha, mitochondrial precursor	ETFA	2
		IPI00301812	Isoform 1 of SPARC-related modular calcium-binding protein 1 precursor	SMOC1	2
		IPI00027239	Isoform Alpha of Tumor necrosis factor ligand superfamily member 13 precursor	TNFSF12-TNF!	2
		IPI00847652	CDNA FLJ46805 fis, clone TRACH3033535	LOC400891	2
		IPI00250724	Protein kinase-like domain containing protein	C18orf51	2
		IPI00024704	Uronyl 2-sulfotransferase	UST	2
		IPI00646291	Integral membrane protein GPR180 precursor	GPR180	2
		IPI00021199	Stathmin-3	STMN3	2
		IPI00020692	Isoform 1 of Sodium channel protein type 3 subunit alpha	SCN3A	2
		IPI00021091	Isoform 1 of Leucine-rich glioma-inactivated protein 1 precursor	LGI1	2
		IPI00021695	Isoform D of Plasma membrane calcium-transporting ATPase 1	ATP2B1	2
		IPI00413781	chemokine (C-X-C motif) ligand 12 (stromal cell-derived factor 1) isoform gamma	CXCL12	2
		IPI00480159	inositol polyphosphate-5-phosphatase F	INPP5F	2
		IPI00005690	Matrilin-3 precursor	MATN3	2
		IPI00289746	Isoform 2 of Serine/threonine-protein kinase PAK 1	PAK1	2
		IPI00295767	Noelin-2 precursor	OLFM2	2
		IPI00296441	Adenosine deaminase	ADA	2
		IPI00827906	Anti-mucin1 light chain variable region (Fragment)	-	2
		IPI00383887	Immunoblobulin heavy chain (Fragment)	-	2
		IPI00385985	Ig lambda chain V-III region LOI	-	2
		IPI00064652	vascular endothelial growth factor A isoform e precursor	VEGFA	2
		IPI00024572	aspartate beta-hydroxylase isoform e	ASPH	2
		IPI00384407	Myosin-reactive immunoglobulin heavy chain variable region (Fragment)	-	2
		IPI00030431	Isoform 1 of Anthrax toxin receptor 1 precursor	ANTXR1	2
		IPI00220706	Hemoglobin subunit gamma-1	HBG1	2
		IPI00028193	192 kDa protein	KNDC1	2
		IPI00000775	Isoform 1 of Leucine-rich repeats and immunoglobulin-like domains protein 1 precursor	LRIG1	2
		IPI00221117	Acylphosphatase-1	C17orf13;ACYF	2
		IPI00646689	Thioredoxin domain-containing protein 17	TXNDC17	2
		IPI00218319	Isoform 2 of Tropomyosin alpha-3 chain	TPM3	2
√	√*	IPI00004367	FXYD domain-containing ion transport regulator 6 precursor	FXYD6	1
√	√*	IPI00029591	P-selectin glycoprotein ligand 1 precursor	SELPLG	1
√	√*	IPI00552578	Serum amyloid A protein precursor	SAA1;SAA2	1
√	√*	IPI00301288	polydom	SVEP1	1
	√*	IPI00000160	Proopiomelanocortin preproprotein	POMC	1

	√*	IPI00032405	Endothelin B receptor-like protein 2 precursor	GPR37L1	1
	√*	IPI00012948	Proheparin-binding EGF-like growth factor precursor	HBEGF	1
	√*	IPI00307612	Cadherin-20 precursor	CDH20	1
	√*	IPI00025992	Hepcidin precursor	HAMP	1
	√*	IPI00027875	Synaptotagmin-11	SYT11	1
	√*	IPI00010442	Phospholemman precursor	FXYD1;FXYD7	1
√	√	IPI00387113	Ig kappa chain V-III region B6	-	1
√	√	IPI00031411	Cadherin-related tumor suppressor homolog precursor	FAT	1
√	√	IPI00738499	Ferritin light chain	FTL	1
√	√	IPI00797310	14 kDa protein	CLSTN3	1
√	√	IPI00853073	Protein	GPX3	1
√	√	IPI00303318	Protein FAM49B	FAM49B	1
√	√	IPI00025318	SH3 domain-binding glutamic acid-rich-like protein	SH3BGRL	1
√	√	IPI00027547	Dermcidin precursor	DCD	1
√	√	IPI00413778	Peptidyl-prolyl cis-trans isomerase	FKBP1A	1
√	√	IPI00022314	Superoxide dismutase [Mn], mitochondrial precursor	SOD2	1
√	√	IPI00645194	integrin beta 1 isoform 1A precursor	ITGB1	1
√	√	IPI00013299	Neuroblastoma, suppression of tumorigenicity 1	NBL1	1
√	√	IPI00743963	Ig kappa chain V-I region HK101 precursor (Fragment)	IGKC	1
	√	IPI00013933	Isoform DPI of Desmoplakin	DSP	1
	√	IPI00024248	Sodium/iodide cotransporter	SLC5A5	1
	√	IPI00028714	Matrix Gla protein precursor	MGP	1
	√	IPI00063827	Isoform 1 of Abhydrolase domain-containing protein 14B	ABHD14B	1
	√	IPI00215899	Isoform 2 of Sushi repeat-containing protein SRPX precursor	SRPX	1
	√	IPI00412264	Pleiotrophin precursor	PTN	1
	√	IPI00024887	Bone morphogenetic protein 6 precursor	BMP6	1
	√	IPI00005837	Angiopoietin-related protein 1 precursor	ANGPTL1	1
	√	IPI00024138	Uncharacterized protein ENSP00000374816	-	1
	√	IPI00335946	Family with sequence similarity 120B	FAM120B	1
	√	IPI00807609	Aberrant LSLCL	CLEC11A	1
	√	IPI00021900	Tumor necrosis factor ligand superfamily member 12	TNFSF12	1
	√	IPI00026358	Gamma-aminobutyric acid receptor-associated protein-like 2	GABARAPL2	1
	√	IPI00030877	15 kDa selenoprotein isoform 1 precursor	SEP15	1
	√	IPI00000265	Uncharacterized protein C10orf38 precursor	C10orf38	1
	√	IPI00386575	Ig lambda chain V-I region EPS	-	1
	√	IPI00000190	CD81 antigen	CD81	1
	√	IPI00790122	27 kDa protein	TNFSF12	1
	√	IPI00026125	Deoxyribonuclease I-like 1 precursor	DNASE1L1	1
	√	IPI00002818	Isoform 1 of Kallikrein-11 precursor	KLK11	1
	√	IPI00296259	Transmembrane emp24 domain-containing protein 4 precursor	TMED4	1

√	IPI00642645	Methylenetetrahydrofolate reductase	MTHFR	1
√	IPI00386131	Ig kappa chain V-III region IARC/BL41 precursor	-	1
√	IPI00013290	hepatoma-derived growth factor-related protein 2 isoform 1	HDGF2	1
√	IPI00154858	Platelet endothelial aggregation receptor 1 precursor	PEAR1	1
√	IPI00000811	Proteasome subunit beta type-6 precursor	PSMB6	1
√	IPI00005719	Isoform 1 of Ras-related protein Rab-1A	RAB1A	1
√	IPI00217264	Isoform 3 of MAP7 domain-containing protein 3	MAP7D3	1
√	IPI00218628	Isoform 2 of Integrin alpha-IIb precursor	ITGA2B	1
√	IPI00217405	Isoform 1 of E3 ubiquitin-protein ligase UBR1	UBR1	1
√	IPI00554521	Ferritin heavy chain	FTH1	1
√	IPI00456670	Isoform 13 of Peroxisomal N(1)-acetyl-spermine/spermidine oxidase	PAOX	1
√	IPI00002535	FK506-binding protein 2 precursor	FKBP2	1
√	IPI00006900	Something about silencing protein 10	UTP3	1
√	IPI00291922	Proteasome subunit alpha type-5	PSMA5	1
√	IPI00031131	Adipocyte plasma membrane-associated protein	C20orf3	1
√	IPI00017163	Isoform 1 of E3 ubiquitin-protein ligase HECW2	HECW2	1
√	IPI00026665	Glutaminyl-tRNA synthetase	QARS	1
√	IPI00003406	Isoform 1 of Drebrin	DBN1	1
√	IPI00020008	NEDD8 precursor	NEDD8	1
√	IPI00387110	Ig kappa chain V-II region MIL	-	1
√	IPI00376087	putative binding protein 7a5	7A5	1
√	IPI00008282	Isoform 1 of Calcium/calmodulin-dependent 3',5'-cyclic nucleotide phosphodiesterase 1A	PDE1A	1
√	IPI00007277	Isoform 1 of Leucine-rich repeat flightless-interacting protein 2	LRRFIP2	1
√	IPI00179589	Myotrophin	MTPN	1
√	IPI00216914	Vitelline membrane outer layer protein 1 homolog precursor	VMO1	1
√	IPI00399252	Isoform 1 of Protein Jade-1	PHF17	1
√	IPI00144243	Human immunodeficiency virus type I enhancer-binding protein 2	HIVEP2	1
√	IPI00186004	hypothetical protein LOC57730	KIAA1641	1
√	IPI00000070	Low-density lipoprotein receptor precursor	LDLR	1
√	IPI00255145	hypothetical protein	LOC342346	1
√	IPI00552874	V1-3 protein	IGLV2-11	1
√	IPI00010737	Thrombomodulin precursor	THBD	1
√	IPI00168806	Isoform 1 of Myeloid/lymphoid or mixed-lineage leukemia protein 3 homolog	MLL3	1
√	IPI00302850	Small nuclear ribonucleoprotein Sm D1	SNRPD1	1
√	IPI00013681	N-terminally extended type 3 canonical transient receptor potential channel	TRPC3	1
√	IPI00019208	Similar to 60S ribosomal protein L29	-	1
√	IPI00025019	Proteasome subunit beta type-1 precursor	PSMB1	1
√	IPI00013508	Alpha-actinin-1	ACTN1	1
√	IPI00470766	Isoform 1 of Olfactomedin-like protein 2B precursor	OLFML2B	1
√	IPI00023359	Isoform 1 of Malonyl CoA-acyl carrier protein transacylase, mitochondrial precursor	MCAT	1

√	IPI00031696	FAST kinase domain-containing protein 3	FASTKD3	1
√	IPI00038378	Isoform 1 of Enolase-phosphatase E1	ENOPH1	1
√	IPI00029819	Neurogenic locus notch homolog protein 3 precursor	NOTCH3	1
√	IPI00003933	hydroxyacyl glutathione hydrolase isoform 1	HAGH	1
√	IPI00184997	cDNA FLJ78771, highly similar to Homo sapiens discs, large homolog 7 (Drosophila), mF	DLG7	1
√	IPI00004409	Discoidin domain-containing receptor 2 precursor	DDR2	1
√	IPI00556643	Semaphorin 3F variant	SEMA3F	1
√	IPI00000792	Quinone oxidoreductase	CRYZ	1
√	IPI00218130	Glycogen phosphorylase, muscle form	PYGM	1
√	IPI00292393	Sodium channel protein type 4 subunit alpha	SCN4A	1
√	IPI00221332	Uncharacterized protein DNM3	DNM3	1
√	IPI00046057	Isoform 2 of Syntaxin-binding protein 1	STXBP1	1
√	IPI00021364	Properdin precursor	CFP	1
√	IPI00061507	Isoform 3 of Ester hydrolase C11orf54	C11orf54	1
√	IPI00382499	Ig heavy chain V-III region JON	-	1
√	IPI00386754	Isoform 2 of Cysteine-rich with EGF-like domain protein 2 precursor	CRELD2	1
√	IPI00737969	microtubule associated monoxygenase, calponin and LIM domain containing 3	MICAL3	1
√	IPI00442121	delta-aminolevulinic acid dehydratase isoform a	ALAD	1
√	IPI00186826	Ephrin receptor	EPHB4	1
√	IPI00013219	Integrin-linked protein kinase	ILK	1
√	IPI00010207	Ubiquitin-fold modifier 1 precursor	UFM1	1
√	IPI00737429	Teneurin-4	ODZ4	1
√	IPI00004480	ADAM DEC1 precursor	ADAMDEC1	1
√	IPI00412216	vacuolar protein sorting 13C protein isoform 2B	VPS13C	1
√	IPI00005969	F-actin-capping protein subunit alpha-1	CAPZA1	1
√	IPI00060308	Isoform 6 of PDZ and LIM domain protein 7	PDLIM7	1
√	IPI00387101	Ig kappa chain V-I region Scw	-	1
√	IPI00218075	Protein FAM9B	FAM9B	1
√	IPI00002790	Isoform 1 of Protein sel-1 homolog 1 precursor	SEL1L	1
√	IPI00221034	Transcription factor RelB	RELB	1
√	IPI00019372	Serglycin precursor	SRGN	1
√	IPI00215979	Bisphosphoglycerate mutase	BPGM	1
√	IPI00216963	Isoform 9 of CASP8 and FADD-like apoptosis regulator precursor	CFLAR	1
√	IPI00248596	similar to slit homolog 1	ELFN1	1
√	IPI00012391	Isoform Long of Adenomatous polyposis coli protein	APC	1
√	IPI00296337	Isoform 1 of DNA-dependent protein kinase catalytic subunit	PRKDC	1
√	IPI00299147	Small ubiquitin-related modifier 3 precursor	SUMO3	1
√	IPI00435925	PP14214	IGFBP3	1
√	IPI00008274	Adenylyl cyclase-associated protein 1	CAP1	1
√	IPI00410588	ADAMTS-like protein 3 precursor	ADAMTSL3	1

√	IPI00004534	Phosphoribosylformylglycinamidine synthase	PFAS	1
√	IPI00554474	Hypothetical LOC284297	LOC284297	1
√	IPI00479669	Isoform 1 of Uncharacterized protein KIAA0701	UHRF1BP1L	1
√	IPI00017940	LMBR1 domain-containing protein 2	LMBRD2	1
√	IPI00400986	hypothetical protein LOC85459	KIAA1731	1
√	IPI00011643	Isoform 2 of Kunitz-type protease inhibitor 1 precursor	SPINT1	1
√	IPI00748955	platelet glycoprotein Ib alpha polypeptide precursor	GP1BA	1
√	IPI00217781	Similar to expressed sequence AI593442	LOC399947	1
√	IPI00031008	Isoform 1 of Tenascin precursor	TNC	1
√	IPI00103552	Mucin-16	MUC16	1
√	IPI00443909	Isoform 1 of Protein canopy homolog 2 precursor	CNPY2	1
√	IPI00024853	Isoform 1 of Periaxin	PRX	1
√	IPI00101927	Leucine zipper putative tumor suppressor 2	LZTS2	1
√	IPI00470838	Isoform 1 of DENN domain-containing protein 2C	DENND2C	1
√	IPI00008580	Antileukoproteinase precursor	SLPI	1
√	IPI00025869	Alpha-galactosidase A precursor	GLA	1
√	IPI00005531	Isoform 1 of Probable DNA dC->dU-editing enzyme APOBEC-3B	APOBEC3B	1
√	IPI00783313	Glycogen phosphorylase, liver form	PYGL	1
√	IPI00009335	Brain protein 16	C8orf30A	1
√	IPI00387095	Ig kappa chain V-I region Ka	-	1
√	IPI00013860	3-hydroxyisobutyrate dehydrogenase, mitochondrial precursor	HIBADH	1
√	IPI00377045	Alpha3A	LAMA3	1
√	IPI00008433	40S ribosomal protein S5	RPS5	1
√	IPI00219525	6-phosphogluconate dehydrogenase, decarboxylating	PGD	1
√	IPI00020501	Myosin-11	MYH11	1
√	IPI00442865	CDNA FLJ26488 fis, clone KDN05770, highly similar to Bumetanide- sensitive sodium-(p	SLC12A1	1
√	IPI00414320	Annexin A11	ANXA11	1
√	IPI00827584	similar to kinesin family member 27	KIF27	1
√	IPI00295618	Isoform Long of Platelet endothelial cell adhesion molecule precursor	PECAM1	1
√	IPI00010133	Coronin-1A	CORO1A	1
√	IPI00013991	Isoform 1 of Tropomyosin beta chain	TPM2	1
√	IPI00005705	Isoform Gamma-1 of Serine/threonine-protein phosphatase PP1-gamma catalytic subuni	PPP1CC	1
√	IPI00303882	Isoform B of Mannose-6-phosphate receptor-binding protein 1	M6PRBP1	1
√	IPI00154528	Isoform 1 of Structural maintenance of chromosomes protein 6	SMC6	1
√	IPI00216049	Isoform 1 of Heterogeneous nuclear ribonucleoprotein K	HNRPK	1
√	IPI00003111	Ig kappa chain V-I region AU	LOC652694	1
√	IPI00003031	Isoform 2 of Isochorismatase domain-containing protein 2, mitochondrial precursor	ISOC2	1
√	IPI00009532	4-aminobutyrate aminotransferase, mitochondrial precursor	ABAT	1
√	IPI00643937	Methylenetetrahydrofolate dehydrogenase (NADP+ dependent) 1-like	MTHFD1L	1
√	IPI00030876	diaphanous 1 isoform 2	DIAPH1	1

√	IPI00031627	DNA-directed RNA polymerase II subunit RPB1	POLR2A	1
√	IPI00737920	similar to dynein, axonemal, heavy polypeptide 1	DNAH3	1
√	IPI00218914	Retinal dehydrogenase 1	ALDH1A1	1
√	IPI00456578	LOC441054 protein	LOC441054	1
√	IPI00013302	ADAM 15 precursor	ADAM15	1
√	IPI00333197	Isoform 2 of GRIP and coiled-coil domain-containing protein 2	GCC2	1
√	IPI00004346	C-C chemokine receptor type 10	CCR10	1
√	IPI00218131	Protein S100-A12	S100A12	1
√	IPI00440153	68 kDa protein	XRRA1	1
√	IPI00014516	Isoform 1 of Caldesmon	CALD1	1
√	IPI00297208	similar to Myosin-10	KIAA1276	1
√	IPI00025084	Calpain small subunit 1	CAPNS1	1
√	IPI00014899	CDNA FLJ20744 fis, clone HEP06585	BRPF3	1
√	IPI00218398	Matrix metalloproteinase-14 precursor	MMP14	1
√	IPI00013978	Speckle-type POZ protein	SPOP	1
√	IPI00014398	Four and a half LIM domains 1 variant	FHL1	1
√	IPI00874156	Isoform 1 of Ubiquitin thioesterase OTUB1	OTUB1	1
√	IPI00004373	Mannose-binding protein C precursor	MBL2	1
√	IPI00827522	Anti-streptococcal/anti-myosin immunoglobulin lambda light chain variable region (Fragm	IGLV1-44	1
√	IPI00007010	Lysozyme-like protein 6 precursor	LYZL6	1
√	IPI00002412	Palmitoyl-protein thioesterase 1 precursor	PPT1	1
√	IPI00299778	Serum paraoxonase/lactonase 3	PON3	1
√	IPI00451429	NIF3L1 isoform gamma	NIF3L1	1
√	IPI00218407	Fructose-bisphosphate aldolase B	ALDOB	1
√	IPI00010369	Testis-expressed sequence 15 protein	TEX15	1
√	IPI00219005	FK506-binding protein 4	FKBP4	1
√	IPI00013004	Isoform 1 of Pyridoxal kinase	PDXK	1
√	IPI00289334	Isoform 1 of Filamin-B	FLNB	1
√	IPI00021907	Isoform 1 of Myelin basic protein	MBP	1
√	IPI00008303	Isoform 1 of N-acetylglucosamine-1-phosphodiester alpha-N-acetylglucosaminidase prec	NAGPA	1
√	IPI00064935	Alpha-protein kinase 3	ALPK3	1
√	IPI00300407	Syndecan-2 precursor	SDC2	1
√	IPI00061448	13 kDa protein	-	1
√	IPI00550364	Phosphoglucomutase-2	PGM2	1
√	IPI00028450	Isoform 1 of Sodium/calcium exchanger 1 precursor	SLC8A1	1
√	IPI00218465	Phospholipase A-2-activating protein	PLAA	1
√	IPI00217740	C20orf12 protein	C20orf12	1
√	IPI00872550	Uncharacterized protein PRDM2	PRDM2	1
√	IPI00382421	Ig lambda chain V-I region NEW	-	1
√	IPI00289861	Isoform 1 of Zinc finger CCHC domain-containing protein 11	ZCCHC11	1

√	IPI00855918	mucin 5, subtype B, tracheobronchial	MUC5B	1
√	IPI00032313	Protein S100-A4	S100A4	1
√	IPI00027626	T-complex protein 1 subunit zeta	CCT6A	1
√	IPI00001960	Chloride intracellular channel protein 4	CLIC4	1
√	IPI00027009	Isoform 1 of Protein kinase C and casein kinase substrate in neurons protein 2	PACSIN2	1
√	IPI00005132	Guanine nucleotide-binding protein-like 3-like protein	GNL3L	1
√	IPI00215746	Fatty acid-binding protein, adipocyte	FABP4	1
√	IPI00216780	Cartilage intermediate layer protein 2 precursor	CILP2	1
√	IPI00385918	CDNA FLJ90582 fis, clone PLACE1000442, moderately similar to ZINC FINGER PROTE	ZNF627	1
√	IPI00218820	Isoform 3 of Tropomyosin beta chain	TPM2	1
√	IPI00383603	Anti-thyroglobulin light chain variable region (Fragment)	-	1
√	IPI00219622	Proteasome subunit alpha type-2	PSMA2	1
√	IPI00031086	Insulin-like growth factor-binding protein 1 precursor	IGFBP1	1
√	IPI00022462	Transferrin receptor protein 1	TFRC	1
√	IPI00016112	peroxidasin homolog	PXDN	1
√	IPI00010193	Isoform 1 of Interferon-alpha/beta receptor beta chain precursor	IFNAR2	1
√	IPI00021048	Isoform 1 of Myoferlin	FER1L3	1
√	IPI00149375	Isoform 2 of Uncharacterized protein C11orf56	C11orf56	1
√	IPI00015913	5,6-dihydroxyindole-2-carboxylic acid oxidase precursor	TYRP1	1
√	IPI00383016	Immunglobulin light chain variable region (Fragment)	-	1
√	IPI00382486	Ig heavy chain V-III region NIE	-	1
√	IPI00004533	Kinesin-like protein KIF3B	KIF3B	1
√	IPI00020019	Adiponectin precursor	ADIPOQ	1
√	IPI00026241	Bone marrow stromal antigen 2 precursor	BST2	1
√	IPI00010863	Copper transport protein ATOX1	ATOX1	1
√	IPI00218918	Annexin A1	ANXA1	1
√	IPI00295577	Receptor-type tyrosine-protein phosphatase beta precursor	PTPRB	1
√	IPI00029372	Uncharacterized protein C4orf15	C4orf15	1
√	IPI00220271	Alcohol dehydrogenase	AKR1A1	1
√	IPI00306853	Carbohydrate sulfotransferase 3	CHST3	1
√	IPI00019530	Tyrosine-protein kinase receptor Tie-1 precursor	TIE1	1
√	IPI00456683	Isoform 3 of Transcription elongation factor SPT6	SUPT6H	1
√	IPI00005614	Isoform Long of Spectrin beta chain, brain 1	SPTBN1	1
√	IPI00021831	cAMP-dependent protein kinase type I-alpha regulatory subunit	PRKAR1A	1
√	IPI00007682	Vacuolar ATP synthase catalytic subunit A	ATP6V1A	1
√	IPI00024502	Ubiquilin-4	UBQLN4	1
√	IPI00298738	DNA-directed RNA polymerase, mitochondrial precursor	POLRMT	1
√	IPI00335541	Isoform 1 of Protein timeless homolog	TIMELESS	1
√	IPI00444331	Isoform 4 of Histone-lysine N-methyltransferase NSD3	WHSC1L1	1
√	IPI00028600	Isoform 1 of Kallikrein-7 precursor	KLK7	1

√	IPI00456635	Isoform 1 of Protein unc-13 homolog D	UNC13D	1
√	IPI00221178	Isoform 2 of Tumor protein D54	TPD52L2	1
√	IPI00000977	Mitogen-activated protein kinase kinase kinase 11	MAP3K11	1
√	IPI00290854	A-kinase anchor protein 3	AKAP3	1
√	IPI00301058	Vasodilator-stimulated phosphoprotein	VASP	1
√	IPI00853516	dynein, axonemal, heavy chain 17	DNAH17	1
√	IPI00384051	Uncharacterized protein PSME2	PSME2	1
√	IPI00006146	serum amyloid A2	SAA1;SAA2	1
√	IPI00150881	Isoform 1 of Coiled-coil domain-containing protein C6orf204	C6orf204	1
√	IPI00024346	snRNA-activating protein complex subunit 3	SNAPC3	1
√	IPI00296913	ADP-sugar pyrophosphatase	NUDT5	1
√	IPI00018342	Adenylate kinase isoenzyme 1	AK1	1
√	IPI00166075	Leucine-rich repeat LGI family member 3 precursor	LGI3	1
√	IPI00029107	Werner syndrome ATP-dependent helicase	WRN	1
√	IPI00867509	Coronin-1C_i3 protein	CORO1C	1
√	IPI00217871	Delta-1-pyrroline-5-carboxylate dehydrogenase, mitochondrial precursor	ALDH4A1	1
√	IPI00552771	V2-11 protein	IGLV3-16	1
√	IPI00410488	Isoform 1 of CD276 antigen precursor	CD276	1
√	IPI00382474	Ig heavy chain V-III region TRO	-	1
√	IPI00215610	55 kDa erythrocyte membrane protein	MPP1	1
√	IPI00015148	Ras-related protein Rap-1b precursor	RAP1B	1
√	IPI00020431	Isoform 1 of TGF-beta receptor type-2 precursor	TGFBR2	1
√	IPI00410657	Isoform 2 of mRNA cap guanine-N7 methyltransferase	RNMT	1
√	IPI00552591	V1-20 protein	IGLV10-54	1
√	IPI00335168	Isoform Non-muscle of Myosin light polypeptide 6	MYL6	1
√	IPI00002491	Isoform 9 of Sorbin and SH3 domain-containing protein 1	SORBS1	1
√	IPI00005159	Actin-related protein 2	ACTR2	1
√	IPI00647217	Superkiller viralicidic activity 2-like 2	SKIV2L2	1
√	IPI00017529	Isoform 1 of Lymphocyte function-associated antigen 3 precursor	CD58	1
√	IPI00024664	Isoform Long of Ubiquitin carboxyl-terminal hydrolase 5	USP5	1
√	IPI00004798	cDNA FLJ75207	CRISP3	1
√	IPI00012877	Isoform 1 of Interferon-alpha/beta receptor alpha chain precursor	IFNAR1	1
√	IPI00478816	Serine protease inhibitor Kazal-type 5 precursor	SPINK5	1
√	IPI00017256	Ras suppressor protein 1	RSU1	1
√	IPI00023217	Isoform 1 of Ryanodine receptor 2	RYR2	1
√	IPI00477616	Protein phosphatase 2A activator, regulatory subunit 4	PPP2R4	1
√	IPI00479722	Proteasome activator complex subunit 1	PSME1	1
√	IPI00006470	Neuron-specific protein family member 2	HMP19	1
√	IPI00021753	Kinesin-like protein KIF13B	KIF13B	1
√	IPI00027984	Putative uncharacterized protein	DNAJC11	1

√	IPI00221255	Isoform 2 of Myosin light chain kinase, smooth muscle	MYLK	1
√	IPI00300207	Isoform 1 of Uncharacterized protein FLJ44066	LOC91431	1
√	IPI00171678	Dopamine beta-hydroxylase	DBH	1
√	IPI00018909	trefoil factor 3 precursor	TFF3	1
√	IPI00163851	Isoform 1 of Eukaryotic translation initiation factor 2-alpha kinase 4	EIF2AK4	1
√	IPI00014340	Isoform 1 of Protein phosphatase 1 regulatory subunit 12C	PPP1R12C	1
√	IPI00297550	Coagulation factor XIII A chain precursor	F13A1	1
√	IPI00299263	ADP-ribosylation factor GTPase-activating protein 3	ARFGAP3	1
√	IPI00014537	Isoform 1 of Calumenin precursor	CALU	1
√	IPI00243995	Serine/threonine-protein kinase Nek5	NEK5	1
√	IPI00171196	keratin 13 isoform b	KRT13	1
√	IPI00021327	Isoform 1 of Growth factor receptor-bound protein 2	GRB2	1
√	IPI00023505	Low affinity immunoglobulin gamma Fc region receptor II-a precursor	FCGR2A	1
√	IPI00015988	HLA class I histocompatibility antigen, alpha chain G precursor	HLA-G	1
√	IPI00021812	Neuroblast differentiation-associated protein AHNAK	AHNAK	1
√	IPI00219077	Isoform 1 of Leukotriene A-4 hydrolase	LTA4H	1
√	IPI00004084	Isoform 2 of Cyclic AMP-dependent transcription factor ATF-6 beta	CREBL1	1
√	IPI00642259	Dystonin	DST	1
√	IPI00789398	Isoform 3 of Lymphocyte antigen 75 precursor	LY75	1
√	IPI00032826	Hsc70-interacting protein	ST13	1
√	IPI00152326	glutathione S-transferase M1 isoform 2	GSTM1	1
√	IPI00044751	Isoform 1 of M-phase phosphoprotein 1	MPHOSPH1	1
√	IPI00009943	Tumor protein, translationally-controlled 1	TPT1	1
√	IPI00166807	Isoform 3 of Oxidation resistance protein 1	OXR1	1
√	IPI00032187	nischarin	NISCH	1
√	IPI00420014	Isoform 1 of U5 small nuclear ribonucleoprotein 200 kDa helicase	ASCC3L1	1
√	IPI00386314	FLJ00064 protein (Fragment)	CNDP2	1
√	IPI00024107	Isoform 1 of Alpha-synuclein	SNCA	1
	IPI00160369	PRKCA-binding protein	PICK1	1
	IPI00002478	Isoform B of Endothelin-converting enzyme 1	ECE1	1
	IPI00027438	Flotillin-1	FLOT1	1
	IPI00033030	Protein ADRM1	ADRM1	1
	IPI00017964	Small nuclear ribonucleoprotein Sm D3	SNRPD3	1
	IPI00163724	Potassium/sodium hyperpolarization-activated cyclic nucleotide-gated channel 3	HCN3	1
	IPI00478640	Isoform 1 of Transmembrane protein C17orf87	C17orf87	1
	IPI00383953	VH4 heavy chain variable region precursor (Fragment)	-	1
	IPI00011051	T-cell leukemia homeobox protein 1	TLX1	1
	IPI00830051	Similar to Immunolgoobulin heavy chain	LOC90925	1
	IPI00550720	Isoform 1 of Uncharacterized protein C19orf57	C19orf57	1
	IPI00103510	Relaxin receptor 2	RXFP2	1

IPI00373872	polycystin 1-like 2 isoform a	PKD1L2	1
IPI00295172	Ninjurin-1	NINJ1	1
IPI00328522	KTEL motif-containing protein 1	KTELC1	1
IPI00251596	Isoform 1 of Collagen alpha-1(XXIII) chain	COL23A1	1
IPI00550906	Cleavage stimulation factor 64 kDa subunit, tau variant	CSTF2T	1
IPI00550263	Isoform 5 of Serine/threonine-protein kinase MRCK alpha	CDC42BPA	1
IPI00045219	Sorting nexin-18	SNAG1	1
IPI00179473	Isoform 1 of Sequestosome-1	SQSTM1	1
IPI00221235	nucleoporin 160kDa	NUP160	1
IPI00167560	PAP-associated domain-containing protein 4	PAPD4	1
IPI00098902	2-oxoglutarate dehydrogenase E1 component, mitochondrial precursor	OGDH	1
IPI00000959	Isoform 1 of VIP peptides precursor	VIP	1
IPI00289271	Liprin-alpha-2	PPFIA2	1
IPI00216470	Isoform 1 of Phosphatidylinositol-5-phosphate 4-kinase type-2 beta	PIP4K2B	1
IPI00879575	71 kDa protein	-	1
IPI00744825	Conserved hypothetical protein	-	1
IPI00005675	NF-kappa-B-repressing factor	NKRF	1
IPI00217989	Isoform 1 of Protein-associating with the carboxyl-terminal domain of ezrin	SCYL3	1
IPI00829836	Uncharacterized protein ENSP00000374797	-	1
IPI00185661	Ubiquitin carboxyl-terminal hydrolase 32	USP32	1
IPI00217258	CCDC100 protein	CCDC100	1
IPI00307729	ADAMTS-3 precursor	ADAMTS3	1
IPI00604430	Isoform 2 of Receptor expression-enhancing protein 2	REEP2	1
IPI00186621	Orofacial clefting chromosomal breakpoint region 1	OFCC1	1
IPI00295098	Signal recognition particle receptor subunit beta	SRPRB	1
IPI00071824	Isoform 1 of Cytoskeleton-associated protein 2	CKAP2	1
IPI00747494	Glutamate receptor delta-2 subunit precursor	GRID2	1
IPI00302962	Amphiphysin I variant CT4 (Fragment)	AMPH	1
IPI00879409	28 kDa protein	-	1
IPI00005153	Isoform Aa of Odorant-binding protein 2a precursor	OBP2A	1
IPI00169259	Small VCP/p97-interacting protein	SVIP	1
IPI00106506	Isoform 1 of Evolutionarily conserved signaling intermediate in Toll pathway, mitochondria	ECSIT	1
IPI00472332	similar to polyhomeotic 1-like isoform 4	LOC653441	1
IPI00789245	Isoform 2 of Probable organic cation transporter protein C6orf85	SLC22A23	1
IPI00012044	Isoform 1 of Pro-neuregulin-3, membrane-bound isoform precursor	NRG3	1
IPI00290744	Fibronectin type-III domain-containing protein C5orf40	C5orf40	1
IPI00152145	Protein odd-skipped-related 1	OSR1	1
IPI00021770	Isoform 1 of 3-hydroxy-3-methylglutaryl-coenzyme A reductase	HMGCR	1
IPI00465045	DIP2 disco-interacting protein 2 homolog B	DIP2B	1
IPI00018803	homeobox D12	HOXD12	1

IPI00828083	Heavy chain Fab (Fragment)	-	1
IPI00827580	Immunogobulin kappa, VJ region (Fragment)	-	1
IPI00021594	Isoform 1 of Glycosylphosphatidylinositol anchor attachment 1 protein	GPAA1	1
IPI00010360	Isoform 1 of Collagen alpha-3(IV) chain precursor	COL4A3	1
IPI00028381	Isoform 1 of Delta-like protein 2 precursor	DLK2	1
IPI00465044	Protein RCC2	RCC2	1
IPI00013847	Cytochrome b-c1 complex subunit 1, mitochondrial precursor	UQCRC1	1
IPI00180386	Isoform GN-1L of Glycogenin-1	GYG1	1
IPI00001793	Beta-1,3-N-acetylglucosaminyltransferase radical fringe	RFNG	1
IPI00167638	Isoform 1 of GTP-binding protein 10	GTPBP10	1
IPI00827891	Cold agglutinin FS-2 H-chain (Fragment)	-	1
IPI00218292	Isoform Short of Ubiquitin fusion degradation protein 1 homolog	UFD1L	1
IPI00384861	Isoform 1 of ARF GTPase-activating protein GIT1	GIT1	1
IPI00018980	Sodium channel subunit beta-1 precursor	SCN1B	1
IPI00830044	Uncharacterized protein ENSP00000374806	-	1
IPI00024012	Frizzled-7 precursor	FZD7	1
IPI00478860	Glycoprotein endo-alpha-1,2-mannosidase	MANEA	1
IPI00552735	V2-8 protein	IGLV3-12	1
IPI00000104	Isoform 1 of mRNA-capping enzyme	RNGTT	1
IPI00296219	Glutaminase liver isoform, mitochondrial precursor	GLS2	1
IPI00005564	Stanniocalcin-1 precursor	STC1	1
IPI00014850	Astrocytic phosphoprotein PEA-15	PEA15	1
IPI00794679	Major histocompatibility complex, class I, B	MICA;HLA-B;HI	1
IPI00294519	Isoform 1 of Telomerase protein component 1	TEP1	1
IPI00744811	Low-density lipoprotein receptor-related protein 5 precursor	LRP5	1
IPI00031485	Mitochondrial ribosomal protein 63	MRP63	1
IPI00784044	Isoform 1 of Methylcrotonoyl-CoA carboxylase beta chain, mitochondrial precursor	MCCC2	1
IPI00514893	Disheveled-associated activator of morphogenesis 2	DAAM2	1
IPI00429191	Eukaryotic peptide chain release factor subunit 1	ETF1	1
IPI00514594	Isoform 1 of Protein FAM5B precursor	FAM5B	1
IPI00853376	additional sex combs like 3	ASXL3	1
IPI00400967	KIAA1843 protein (Fragment)	KIAA1843	1
IPI00166010	Isoform 1 of CCR4-NOT transcription complex subunit 1	CNOT1	1
IPI00793576	7 kDa protein	PRH2;PRB4;PF	1
IPI00301631	Isoform 1 of Torsin-3A precursor	TOR3A	1
IPI00396077	Isoform 1 of E3 ubiquitin-protein ligase Topors	TOPORS	1
IPI00465178	Isoform 1 of Vacuolar proton translocating ATPase 116 kDa subunit a isoform 1	ATP6V0A1	1
IPI00291463	Radical S-adenosyl methionine domain-containing protein 2	RSAD2	1
IPI00007040	Zinc finger protein 222	ZNF222	1
IPI00220578	Guanine nucleotide-binding protein G	GNAI3	1

IPI00740191	similar to Forkhead box protein L1	LOC651986	1
IPI00044607	Protein phosphatase inhibitor 2-like protein 1	PPP1R2P1	1
IPI00301923	Isoform 1 of Cell division protein kinase 9	CDK9	1
IPI00059395	Kinesin-like protein KIFC2	KIFC2	1
IPI00030319	Forkhead box protein F2	FOXF2	1
IPI00018275	Prion-like protein doppel precursor	PRND	1
IPI00025363	Isoform 1 of Glial fibrillary acidic protein	GFAP	1
IPI00015285	Ethanolamine-phosphate cytidylyltransferase	PCYT2	1
IPI00554799	shadow of prion protein	SPRN	1
IPI00478124	61 kDa protein	UCKL1	1
IPI00304596	Non-POU domain-containing octamer-binding protein	NONO	1
IPI00012895	Isoform 1 of Carbonic anhydrase 12 precursor	CA12	1
IPI00299627	Dual oxidase 2 precursor	DUOX2	1
IPI00023513	Isoform 1 of E3 ubiquitin-protein ligase CHFR	CHFR	1
IPI00394870	Brorin precursor	VWC2	1
IPI00166817	Zinc finger protein 561	ZNF561	1
IPI00375174	Ankyrin repeat and sterile alpha motif domain-containing protein 1B	ANKS1B	1
IPI00296866	interphotoreceptor matrix proteoglycan 2	IMPG2	1
IPI00040730	protocadherin 21 precursor	PCDH21	1
IPI00829980	Myosin-reactive immunoglobulin light chain variable region (Fragment)	-	1
IPI00028561	Kinesin heavy chain isoform 5C	KIF5C	1
IPI00743898	Uncharacterized protein ENSP00000357890 (Fragment)	-	1
IPI00644231	Isoform 1 of Cytoplasmic FMR1-interacting protein 1	CYFIP1	1
IPI00007193	Isoform 2 of Ankyrin repeat domain-containing protein 26	ANKRD26	1
IPI00306959	Keratin, type II cytoskeletal 7	KRT7	1
IPI00816737	Rheumatoid factor D5 heavy chain (Fragment)	-	1
IPI00013466	Arsenical pump-driving ATPase	ASNA1	1
IPI00016605	Uncharacterized protein C1orf123	C1orf123	1
IPI00872861	PTD016 protein	LOC51136	1
IPI00298285	Isoform 1 of Receptor tyrosine-protein kinase erbB-3 precursor	ERBB3	1
IPI00166619	Isoform 2 of Putative transporter SVOPL	SVOPL	1
IPI00029722	Kinesin heavy chain isoform 5A	KIF5A	1
IPI00005128	Isoform 1 of Angiopoietin-2 precursor	ANGPT2	1
IPI00796647	HIG1 domain family, member 1C	HIGD1C	1
IPI00852758	Similar to Ankyrin repeat domain-containing protein 26. Isoform 2	ANKRD18B	1
IPI00023087	Ubiquitin-conjugating enzyme E2 T	UBE2T	1
IPI00297444	Isoform 1 of CD177 antigen precursor	CD177	1
IPI00161119	Isoform 1 of NF-kappa-B inhibitor beta	NFKBIB	1
IPI00016577	CDNA: FLJ22814 fis, clone KAIA3004	CD22	1
IPI00852633	16 kDa protein	RABL2B	1

IPI00163391	Isoform 1 of Putative methyltransferase METT10D	METT10D	1
IPI00299679	Isoform B of Ral guanine nucleotide dissociation stimulator-like 1	RGL1	1
IPI00797699	20 kDa protein	-	1
IPI00333126	Leucine-rich repeat-containing protein 56	LRRC56	1
IPI00794119	13 kDa protein	ABCC8	1
IPI00816274	Chemokine-like factor superfamily 1 transcript variant 26	CMTM1	1
IPI00032830	Isoform 1 of Oligoribonuclease, mitochondrial precursor (Fragment)	REXO2	1
IPI00171737	Isoform 2 of Leucine-rich repeat and death domain-containing protein	LRDD	1
IPI00385003	Putative transposase	TIGD1	1
IPI00029123	Isoform A of Endothelin B receptor precursor	EDNRB	1
IPI00218487	Gap junction alpha-1 protein	GJA1	1
IPI00158992	snRNA-activating protein complex subunit 4	SNAPC4	1
IPI00394712	Granulocyte inhibitory protein	-	1
IPI00001869	Pappalysin-1 precursor	PAPPA	1
IPI00027834	heterogeneous nuclear ribonucleoprotein L isoform a	HNRNPL	1
IPI00062730	Uncharacterized protein C16orf45	C16orf45	1
IPI00298258	UNC13B protein	UNC13B	1
IPI00018352	Ubiquitin carboxyl-terminal hydrolase isozyme L1	UCHL1	1
IPI00744366	Conserved hypothetical protein	-	1
IPI00008905	UDP-glucuronosyltransferase 2B15 precursor	UGT2B15	1
IPI00377077	Isoform 3 of Astrotactin-2 precursor	ASTN2	1
IPI00009294	Cysteine-rich motor neuron 1 protein precursor	CRIM1	1
IPI00186581	amplified in osteosarcoma isoform 2 precursor	OS9	1
IPI00445364	CDNA FLJ44171 fis, clone THYMU2036058	-	1
IPI00021985	transmembrane 9 superfamily protein member 4	TM9SF4	1
IPI00166071	B-cell CLL/lymphoma 6 member B protein	BCL6B	1
IPI00217023	MMAA protein	MMAA	1
IPI00455521	similar to transmembrane protein 46	C22:CTA-250D	1
IPI00034006	Tyrosine-protein phosphatase non-receptor type 23	PTPN23	1
IPI00021274	Ephrin type-A receptor 8 precursor	EPHA8	1
IPI00394820	Olfactomedin-like protein 1 precursor	OLFML1	1
IPI00419253	Isoform 1 of Nck-associated protein 5	NAP5	1
IPI00029012	Eukaryotic translation initiation factor 3 subunit A	EIF3A	1
IPI00043201	Centromere protein J	CENPJ	1
IPI00385042	Nucleolar GTP-binding protein 1	GTPBP4	1
IPI00410013	Isoform 1 of Zinc finger CCCH domain-containing protein 3	ZC3H3	1
IPI00402144	Isoform 1 of Zinc finger protein 555	ZNF555	1
IPI00478986	Similar to 40S ribosomal protein S4	LOC126235	1
IPI00478521	Isoform 1 of UPF0475 protein	RILPL1	1
IPI00420071	microtubule-associated protein 6 isoform 1	MAP6	1

IPI00555614	Heat shock protein 90Bc	HSP90AB3P	1
IPI00026530	Protein ERGIC-53 precursor	LMAN1	1
IPI00025700	Isoform CD6A of T-cell differentiation antigen CD6 precursor	CD6	1
IPI00016949	Isoform 4 of Electrogenic sodium bicarbonate cotransporter 1	SLC4A4	1
IPI00297040	Serine protease inhibitor Kazal-type 6 precursor	SPINK6	1
IPI00454910	Serine/threonine-protein kinase MRCK gamma	CDC42BPG	1
IPI00031547	Desmoglein-3 precursor	DSG3	1
IPI00384722	Isoform 2 of UPF0510 protein C19orf63 precursor	C19orf63	1
IPI00382756	Isoform 2 of Pleiotropic regulator 1	PLRG1	1
IPI00604599	Transmembrane emp24 domain-containing protein 3 precursor	TMED3	1
IPI00743284	Methionine synthase	MTR	1
IPI00021363	Histone demethylase JARID1A	JARID1A	1
IPI00749440	Uncharacterized protein ENSP00000368180	-	1
IPI00216683	M-phase inducer phosphatase 3	CDC25C	1
IPI00015522	Growth/differentiation factor 5 precursor	GDF5	1
IPI00877084	Isoform 1 of Coiled-coil domain-containing protein 144C	CCDC144C	1
IPI00060265	Zinc finger protein 775	ZNF775	1
IPI00024802	TATA-binding protein-associated factor 172	BTAF1	1
IPI00374039	Conserved hypothetical protein	C1orf189	1
IPI00302133	Transient receptor potential cation channel subfamily V member 5	TRPV5	1
IPI00028786	Isoform 3 of Polycystin-1 precursor	PKD1	1
IPI00167254	Isoform 4 of Inactive phospholipase D5	PLD5	1
IPI00013455	CLIP1 protein	CLIP1	1
IPI00026299	Isoform Glycophorin C of Glycophorin-C	GYPC	1
IPI00845508	BAH domain and coiled-coil containing 1	BAHCC1	1
IPI00745300	31 kDa protein	NAT11	1
IPI00009148	Diphosphoinositol polyphosphate phosphohydrolase 1	NUDT3	1
IPI00787932	similar to zinc finger protein 10	hCG_1646157	1
IPI00166161	Protein SIX6OS1	C14orf39	1
IPI00180426	Isoform 3 of G protein-coupled receptor kinase 4	GRK4	1
IPI00382420	Ig lambda chain V-I region HA	-	1
IPI00166553	Isoform 1 of Protein FAM19A2 precursor	FAM19A2	1
IPI00004315	Sialic acid-binding Ig-like lectin 9 precursor	SIGLEC9	1
IPI00796906	8 kDa protein	ABCF3	1
IPI00028932	Microtubule-associated serine/threonine-protein kinase 3	MAST3	1
IPI00018708	Isoform 2 of Centrosomal protein of 63 kDa	CEP63	1
IPI00011578	Isoform 1 of Neuroplastin precursor	NPTN	1
IPI00387159	Isoform 1 of Inhibitor of growth protein 3	ING3	1
IPI00645089	Kv channel interacting protein 1 isoform 3	KCNIP1	1
IPI00167619	Leucine-rich repeat and transmembrane domain-containing protein 2 precursor	LRTM2	1

IPI00374862	Isoform 1 of Kelch-like protein 5	KLHL5	1
IPI00009377	HSPC212	C3orf19	1
IPI00300244	zinc finger, CW type with PWWP domain 1	ZCWPW1	1
IPI00333410	Isoform 1 of Ubiquitin-conjugating enzyme E2 Q1	UBE2Q1	1
IPI00018311	Isoform 2 of Neuroplastin precursor	NPTN	1
IPI00168862	Conserved hypothetical protein	PXT1	1
IPI00025647	Isoform 1 of F-box only protein 21	FBXO21	1
IPI00027429	Putative uncharacterized protein DKFZp547J2313	FABP7	1
IPI00013281	Fukutin-related protein	FKRP	1
IPI00252845	SYT9 protein	SYT9	1
IPI00171611	Histone H3.2	HIST2H3C;HIS	1
IPI00477361	10 kDa protein	SDHALP1	1
IPI00397578	135 kDa protein	PPFIA4	1
IPI00031019	Cystatin-8 precursor	CST8	1
IPI00017659	Protein kinase substrate CapZIP	RCSD1	1
IPI00744706	282 kDa protein	SPTAN1	1
IPI00025094	CDNA: FLJ22037 fis, clone HEP08868 (Fragment)	MYH16	1
IPI00830057	Uncharacterized protein ENSP00000374791	-	1
IPI00023315	Bone morphogenetic protein 3b precursor	GDF10	1
IPI00010895	Tubby-related protein 2	TULP2	1
IPI00739106	similar to ribosomal protein L5 isoform 1	LOC647436	1
IPI00022606	Isoform 1 of Proline-serine-threonine phosphatase-interacting protein 1	PSTPIP1	1
IPI00550232	cardiomyopathy associated 3 isoform 1	XIRP2	1
IPI00295502	Isoform 1 of Protein Wiz	WIZ	1
IPI00216106	Isoform 3 of Obg-like ATPase 1	OLA1	1
IPI00010118	Isoform 1 of Prostate tumor overexpressed gene 1 protein	PTOV1	1
IPI00300990	Isoform 1 of Uncharacterized protein C1orf77	C1orf77	1
IPI00741780	similar to CG4845-PA	LOC652559	1
IPI00454858	similar to alpha 3 type VI collagen isoform 1 precursor	LOC344875	1
IPI00399328	similar to jumonji domain containing 2D	LOC390245	1
IPI00479083	Isoform 2 of Erythroid differentiation-related factor 1	C10orf137	1
IPI00168885	Isoform 1 of Putative ATP-dependent RNA helicase DHX57	DHX57	1
IPI00010575	KIAA1466 protein	KIAA1466	1
IPI00785015	Isoform 1 of Uncharacterized protein KIAA2030	FLJ25778	1
IPI00106502	Kelch-like ECH-associated protein 1	KEAP1	1
IPI00830025	Uncharacterized protein ENSP00000375021	-	1
IPI00187143	Isoform 2 of Ras-related protein Rab-4B	RAB4B	1
IPI00103630	Isoform 2 of Protein phosphatase 1E	PPM1E	1
IPI00216508	Isoform 2 of Sorting nexin-3	SNX3	1
IPI00011400	T-lymphoma invasion and metastasis-inducing protein 1	TIAM1	1

IPI00299485	Complement component C1q receptor precursor	CD93	1
IPI00060146	Isoform 1 of Smith-Magenis syndrome chromosome region candidate gene 7 protein	SMCR7	1
IPI00329688	Protein YIPF3	YIPF3	1
IPI00022989	Isoform Beta-1 of Retinoic acid receptor beta	RARB	1
IPI00152072	hypothetical protein LOC387758	FIBIN	1
IPI00060546	Uncharacterized protein C10orf35	C10orf35	1
IPI00412541	Probable G-protein coupled receptor 158 precursor	GPR158	1
IPI00043978	Isoform 1 of Partitioning-defective 3 homolog B	PARD3B	1
IPI00440221	Putative uncharacterized protein (Fragment)	CDC2L5	1
IPI00011564	Syndecan-4 precursor	SDC4	1
IPI00423683	Isoform 2 of EMI domain-containing protein 1 precursor	EMID1	1
IPI00026612	Isoform Beta-1 of Protein phosphatase 1B	PPM1B	1
IPI00167137	Isoform 3 of SLAM family member 7 precursor	SLAMF7	1
IPI00155447	MMP28 protein	MMP28	1
IPI00001434	Protocadherin beta 14 precursor	PCDHB14	1
IPI00854745	Uncharacterized protein ENSP00000375019	-	1
IPI00043731	CDNA FLJ30671 fis, clone FCBBF1000687, moderately similar to Mus musculus Rap2 in	RUNDC3B	1
IPI00645814	Isoform 2 of MAP7 domain-containing protein 1	MAP7D1	1
IPI00003353	Neuronal protein 3.1	C5orf13	1
IPI00007402	Importin-7	IPO7	1
IPI00152470	Prokineticin receptor 1	PROKR1	1
IPI00006094	Regulating synaptic membrane exocytosis protein 3	RIMS3	1
IPI00169426	Isoform 2 of Cytosolic 5'-nucleotidase 1B	NT5C1B	1
IPI00816794	REV25-2 (Fragment)	-	1
IPI00010346	Neurolysin, mitochondrial precursor	NLN	1
IPI00013216	Origin recognition complex subunit 2	ORC2L	1
IPI00216572	BarH-like homeobox 2	BARX2	1
IPI00644025	Isoform 1 of Synaptic vesicle glycoprotein 2A	SV2A	1
IPI00171647	Isoform 1 of Sialic acid-binding Ig-like lectin 8 precursor	SIGLEC8	1
IPI00020058	Isoform 1 of Copper-transporting ATPase 2	ATP7B	1
IPI00026570	Cytochrome c oxidase polypeptide VIIa-liver/heart, mitochondrial precursor	COX7A2	1
IPI00027726	Isoform 1 of Krueppel-like factor 3	KLF3	1
IPI00064241	Isoform 1 of Zinc finger protein Eos	IKZF4	1
IPI00295503	Isoform 2 of Probable ATP-dependent RNA helicase DDX58	DDX58	1
IPI00307591	Zinc finger protein 609	ZNF609	1
IPI00290292	Rhomboid 5 homolog 1	RHBDF1	1
IPI00003348	Guanine nucleotide-binding protein G(I)/G(S)/G(T) subunit beta-2	GNB2	1
IPI00299435	apolipoprotein F precursor	APOF	1
IPI00029533	Integrin beta-8 precursor	ITGB8	1
IPI00815786	Hexokinase 1 (Fragment)	HK1	1

IPI00375881	Polycystic kidney disease 1-like protein 3	PKD1L3	1
IPI00008998	Protein tyrosine phosphatase-like protein PTPLAD1	PTPLAD1	1
IPI00658112	32 kDa protein	SPEG	1
IPI00479361	Isoform 1 of UDP-GlcNAc:betaGal beta-1,3-N-acetylglucosaminyltransferase 4	B3GNT4	1
IPI00448465	Isoform 1 of Ankyrin repeat domain-containing protein 12	ANKRD12	1
IPI00745122	Conserved hypothetical protein	MGC33894	1
IPI00783471	Immunglobulin heavy chain variable region (Fragment)	-	1
IPI00023542	transmembrane emp24 protein transport domain containing 9	TMED9	1
IPI00383832	Protein kinase C-binding protein RACK8	DVL3	1
IPI00027685	C-C chemokine receptor type 1	CCR1	1
IPI00432755	PPRR6495	FAM124A	1
IPI00796777	17 kDa protein	CRYAA	1
IPI00008894	Carboxypeptidase A4 precursor	CPA4	1
IPI00847759	DENN domain-containing protein 4B	DENND4B	1
IPI00008315	Isoform 1 of Ephrin type-B receptor 1 precursor	EPHB1	1
IPI00031005	Protein kinase-like protein SgK196	FLJ23356	1
IPI00787414	Uncharacterized protein ENSP00000381388	MGC34829	1
IPI00019988	N-sulphoglucosamine sulphohydrolase precursor	SGSH	1
IPI00059164	Galactose-3-O-sulfotransferase 3	GAL3ST3	1
IPI00175654	Probable mast cell antigen 32 homolog precursor	C17orf60	1
IPI00873740	Uncharacterized protein ENSP00000383832 (Fragment)	-	1
IPI00032416	Isoform Long of Protein jagged-2 precursor	JAG2	1
IPI00335589	RNA methyltransferase-like protein 1	RNMTL1	1
IPI00843819	Similar to Dual specificity protein kinase CLK2	-	1
IPI00065276	Isoform 2 of Tether containing UBX domain for GLUT4	ASPSCR1	1
IPI00644522	PNKP protein	PNKP	1
IPI00045511	Isoform 1 of Chloride channel CLIC-like protein 1 precursor	CLCC1	1
IPI00604763	Transmembrane protein 66 precursor	TMEM66	1
IPI00829759	Uncharacterized protein ENSP00000375040	-	1
IPI00235647	similar to fibrillarin	LOC345630	1
IPI00294910	Protein PARM-1 precursor	DKFZP564O08	1
IPI00217652	Isoform 1 of Glycosyltransferase 8 domain-containing protein 3	GLT8D3	1
IPI00470805	Isoform 2 of Mediator of DNA damage checkpoint protein 1	MDC1	1
IPI00167089	Isoform 2 of Activated CDC42 kinase 1	TNK2	1
IPI00385143	Microfibrillar protein 2 (Fragment)	-	1
IPI00783753	UPF0235 protein C15orf40	C15orf40	1
IPI00445716	Isoform 1 of GDNF family receptor alpha-3 precursor	GFRA3	1
IPI00030919	Mitogen-activated protein kinase kinase 1-interacting protein 1	MAP2K1IP1	1
IPI00827745	Isoform 1 of RNA-binding protein 24	RBM24	1
IPI00005776	Nucleotide-binding oligomerization domain-containing protein 1	NOD1	1

IPI00394879	Leucine-rich repeat-containing protein 9	LRRC9	1
IPI00401852	Conserved hypothetical protein	DKFZP434L187	1
IPI00306884	CDNA FLJ11867 fis, clone HEMBA1006976, weakly similar to H.sapiens Gal-beta(1-3/1-4	ST3GAL4	1
IPI00748891	hypothetical protein LOC283635 isoform 1	C14orf24	1
IPI00874023	Uncharacterized protein ENSP00000379699	-	1
IPI00020199	Alpha-2,8-sialyltransferase 8B	ST8SIA2	1
IPI00297714	Gamma-synuclein	SNCG	1
IPI00005158	Lon protease homolog, mitochondrial precursor	LONP1	1
IPI00791513	CDNA FLJ16614 fis, clone TESTI4013365	CTA-216E10.6	1
IPI00013319	Isoform 2 of 43 kDa receptor-associated protein of the synapse	RAPSN	1
IPI00828037	Heavy chain Fab (Fragment)	-	1
IPI00020201	CMP-N-acetylneuraminate-poly-alpha-2,8-sialyltransferase	ST8SIA4	1
IPI00217537	Isoform 1 of Putative Polycomb group protein ASXL1	ASXL1	1
IPI00002884	CDNA: FLJ22222 fis, clone HRC01658	FLJ22222	1
IPI00025092	Myosin-binding protein C, slow-type	MYBPC1	1
IPI00003363	Isoform 1 of Protein phosphatase 1 regulatory subunit 1B	PPP1R1B	1
IPI00022078	Protein NDRG1	NDRG1	1
IPI00290857	Keratin, type II cytoskeletal 3	KRT3	1
IPI00164949	Isoform NELF-C of Negative elongation factor C/D	TH1L	1
IPI00029647	Zymogen granule membrane protein 16 precursor	ZG16	1
IPI00414481	GTF3C1 protein	GTF3C1	1
IPI00640818	Isoform 3 of Neuropathy target esterase	PNPLA6	1
IPI00164861	Isoform 3 of Kinesin-like protein KIF13A	KIF13A	1
IPI00411674	Isoform 1 of Zinc finger protein 254	ZNF254	1
IPI00025365	Isoform Long of Endothelin-3 precursor	EDN3	1
IPI00020131	Son of sevenless homolog 1	SOS1	1
IPI00828191	NANUC-2 heavy chain (Fragment)	-	1
IPI00249982	Isoform 1 of Death-inducer obliterator 1	DIDO1	1
IPI00025473	Beta-1,4 N-acetylgalactosaminyltransferase 1	B4GALNT1	1
IPI00328270	Neuronal PAS domain-containing protein 2	NPAS2	1
IPI00432707	Caspase-12	CASP12	1
IPI00442544	CDNA FLJ27034 fis, clone SLV07984	-	1
IPI00175019	similar to Temporarily Assigned Gene name family member	LOC643677	1
IPI00027898	Isoform A of Uncharacterized protein C21orf70	C21orf70	1
IPI00019485	Isoform 2 of Enoyl-CoA hydratase domain-containing protein 2, mitochondrial precursor	ECHDC2	1
IPI00023340	Histone acetyltransferase MYST3	MYST3	1
IPI00300052	Keratin type II cuticular Hb4	KRT84	1
IPI00383594	melanoma ubiquitous mutated protein	MUM1	1
IPI00019158	ADAM metallopeptidase domain 8 precursor	ADAM8	1
IPI00005605	Isoform 1 of Protein NDRG3	NDRG3	1

IPI00045928	Sodium/hydrogen exchanger 7	SLC9A7	1
IPI00306332	60S ribosomal protein L24	RPL24	1
IPI00015047	8D6 antigen (Fragment)	CD320	1
IPI00001786	Isoform 2 of Ubiquitin carboxyl-terminal hydrolase 36	USP36	1
IPI00745103	similar to melanoma associated antigen (mutated) 1-like 1	LOC728307	1
IPI00827846	Anti-mucin1 heavy chain variable region (Fragment)	-	1
IPI00385791	Serologically defined breast cancer antigen NY-BR-87 (Fragment)	MRPS26	1
IPI00145805	Isoform 1 of TRAF2 and NCK-interacting protein kinase	TNIK	1
IPI00296374	Zinc finger protein-like 1	ZFPL1	1
IPI00021733	Bifunctional heparan sulfate N-deacetylase/N-sulfotransferase 4	NDST4	1
IPI00184884	Non-structural maintenance of chromosomes element 1 homolog	NSMCE1	1
IPI00103874	Isoform 1 of Zinc finger FYVE domain-containing protein 1	ZFYVE1	1
IPI00243221	nardilysin (N-arginine dibasic convertase) isoform a	NRD1	1
IPI00220791	Amphiphysin I variant CT2	AMPH	1
IPI00015983	Sphingosine 1-phosphate receptor Edg-3	EDG3	1
IPI00289965	Potassium voltage-gated channel subfamily C member 3	KCNC3	1
IPI00374129	NLR family, pyrin domain containing 3 isoform b	NLRP3	1
IPI00007617	Olfactory receptor 52A1	OR52A1	1
IPI00297288	Cdc42 GTPase-activating protein	CDGAP	1
IPI00748890	Isoform 1 of Leucine zipper protein 2 precursor	LUZP2	1
IPI00376587	Uncharacterized protein ENSP00000345065	LOC728780	1
IPI00297251	Isoform 2 of Probable E3 ubiquitin-protein ligase MGRN1	MGRN1	1
IPI00438170	Isoform 1 of Sorting nexin-12	SNX12	1
IPI00373823	Cytochrome P450 26C1	CYP26C1	1
IPI00550917	Twinfilin-2	TWF2	1
IPI00301294	Protein FAM134A	FAM134A	1
IPI00021951	Uncharacterized protein KIAA0247 precursor	KIAA0247	1
IPI00815893	Isoform 1 of Chromodomain-helicase-DNA-binding protein 2	CHD2	1
IPI00002243	Isoform 1 of Gamma-glutamyltransferase 5 precursor	GGTLA1	1
IPI00159049	SET-binding protein	SETBP1	1
IPI00297277	Isoform 1 of RING finger protein 150 precursor	RNF150	1
IPI00031765	Isoform 2 of Protocadherin gamma C4 precursor	PCDHGC4	1
IPI00016685	Enamelin precursor	ENAM	1
IPI00002191	Putative uncharacterized protein FLJ12684	-	1
IPI00007928	Pre-mRNA-processing-splicing factor 8	PRPF8	1
IPI00022542	Rho-associated protein kinase 1	ROCK1	1
IPI00028053	Gap junction alpha-9 protein	GJA9	1
IPI00005732	Isoform 1 of Activin receptor type-1B precursor	ACVR1B	1
IPI00293095	Isoform 1 of Coiled-coil domain-containing protein 83	CCDC83	1
IPI00747142	Centaurin-gamma-like family member 6	CTGLF6	1

IPI00040900	Isoform 2 of Heparan sulfate 2-O-sulfotransferase 1	HS2ST1	1
IPI00011515	Protein kinase C and casein kinase substrate in neurons protein 1	PACSIN1	1
IPI00164066	Isoform 4 of Coiled-coil domain-containing protein 136	CCDC136	1
IPI00307702	H53_GS1 (Fragment)	-	1
IPI00307611	Isoform 1 of Microtubule-associated serine/threonine-protein kinase 4	MAST4	1
IPI00014444	Isoform 1 of Protein SERAC1	SERAC1	1
IPI00479217	Isoform Short of Heterogeneous nuclear ribonucleoprotein U	HNRNPU	1
IPI00183206	Isoform 1 of RIM-binding protein 2	RIMBP2	1
IPI00220070	6-phosphofructo-2-kinase/fructose-2,6-biphosphatase 4	PFKFB4	1
IPI00023322	Zinc finger protein ubi-d4	DPF2	1
IPI00784739	Uncharacterized protein C14orf43	C14orf43	1
IPI00738920	similar to CG3104-PA, isoform A	LOC642574	1
IPI00056314	Pre-rRNA-processing protein TSR2 homolog	TSR2	1
IPI00797694	3 kDa protein	-	1
IPI00294210	DNA-binding protein inhibitor ID-2	ID2	1
IPI00787020	similar to Dynamin-1	LOC644153	1
IPI00167941	Midasin	MDN1	1
IPI00016701	P2Y purinoceptor 14	P2RY14	1
IPI00166039	Isoform 1 of Scotin precursor	SCOTIN	1
IPI00009899	Uncharacterized protein C5orf5	C5orf5	1
IPI00005129	Isoform 1 of Secretory carrier-associated membrane protein 1	SCAMP1	1
IPI00217948	FRMD4B protein	FRMD4B	1
IPI00027457	C1q-related factor precursor	C1QL1	1
IPI00002993	Transcription initiation factor TFIID subunit 9	TAF9	1
IPI00555600	Solute carrier family 26, member 1 isoform a variant (Fragment)	IDUA	1
IPI00419221	Membrane-bound O-acyltransferase domain-containing protein 2	MBOAT2	1
IPI00021634	Kinesin light chain 2	KLC2	1
IPI00384225	Meteorin precursor	METRN	1
IPI00375803	Isoform 1 of GON-4-like protein	GON4L	1
IPI00023184	Isoform 1 of Poly [ADP-ribose] polymerase 3	PARP3	1
IPI00872739	Uncharacterized protein C18orf2	C18orf2	1
IPI00241409	hypothetical protein LOC55747	FAM21B	1
IPI00090764	Toll-like receptor 1 precursor	TLR1	1
IPI00141938	H2A histone family, member V isoform 2	H2AFV	1
IPI00024253	Isoform 1 of Fibroblast growth factor 14	FGF14	1
IPI00387096	Ig kappa chain V-I region Kue	-	1
IPI00218637	Major histocompatibility complex, class II, DQ beta 2	HLA-DQB2	1
IPI00216921	Isoform 2 of Stathmin-4	STMN4	1
IPI00011416	Delta(3,5)-Delta(2,4)-dienoyl-CoA isomerase, mitochondrial precursor	ECH1	1
IPI00260755	similar to Rho GTPase activating protein 18	C20orf95	1

IPI00337385	Isoform 1 of Pre-mRNA-processing factor 40 homolog A	PRPF40A	1
IPI00304527	Protein FAM83B	FAM83B	1
IPI00000459	Transmembrane gamma-carboxyglutamic acid protein 1 precursor	PRRG1	1
IPI00030741	Uncharacterized protein C21orf13	LCA5L	1
IPI00399296	hypothetical protein LOC390110	LOC390110	1
IPI00233358	islet cell autoantigen 1,69kDa-like isoform 2	ICA1L	1
IPI00879842	6 kDa protein	-	1
IPI00021476	Eukaryotic translation initiation factor 4E-binding protein 3	EIF4EBP3	1
IPI00028383	Uncharacterized protein C16orf24	C16orf24	1
IPI00001863	Wnt inhibitory factor 1 precursor	WIF1	1
IPI00298337	cDNA FLJ77671	SLC14A1	1
IPI00044842	Isoform 2 of RAB3A-interacting protein	RAB3IP	1
IPI00022055	Histone acetyltransferase PCAF	PCAF	1
IPI00328260	Protein FAN	NSMAF	1
IPI00807418	Isoform 9 of Lymphoid-specific helicase	HELLS	1
IPI00183002	Isoform 1 of Protein phosphatase 1 regulatory subunit 12A	PPP1R12A	1
IPI00163601	Putative uncharacterized protein FLJ10213	FLJ10213	1
IPI00428741	LP2477	FLJ35348	1
IPI00006252	Multisynthetase complex auxiliary component p43	SCYE1	1
IPI00007321	Isoform 1 of Acyl-protein thioesterase 1	LYPLA1	1
IPI00217617	palmitoylated membrane protein 7	MPP7	1
IPI00306850	EGF-like-domain, multiple 3	MEGF6	1
IPI00024662	Chromobox protein homolog 5	CBX5	1
IPI00169331	Phosphatidylcholine:ceramide cholinephosphotransferase 2	SGMS2	1
IPI00783464	dynein heavy chain domain 3	DNAH2	1
IPI00298702	solute carrier family 39 (zinc transporter), member 6 isoform 1	SLC39A6	1
IPI00185088	immunoglobulin superfamily, member 11 isoform b	IGSF11	1
IPI00010405	Isoform Long of Tyrosine-protein kinase transmembrane receptor ROR1 precursor	ROR1	1
IPI00873774	Uncharacterized protein ENSP00000383488 (Fragment)	-	1
IPI00847335	FLJ45422 protein	-	1
IPI00877800	32 kDa protein	-	1
IPI00008497	Ornithine decarboxylase	ODC1	1
IPI00010808	Interferon-gamma receptor alpha chain precursor	IFNGR1	1
IPI00827485	BRE (Fragment)	-	1
IPI00514622	Ran-binding protein 6	RANBP6	1
IPI00009203	Sorting nexin-7	SNX7	1
IPI00790021	Zinc finger protein 652	ZNF652	1
IPI00000027	Pituitary adenylate cyclase-activating polypeptide precursor	ADCYAP1	1
IPI00033600	Isoform 1 of Protein phosphatase 1 regulatory subunit 7	PPP1R7	1
IPI00375746	Isoform 1 of Guanylate-binding protein 6	GBP6	1

IPI00180384	dynein, axonemal, heavy chain 7	DNAH7	1
IPI00176920	Nephrocystin-4	NPHP4	1
IPI00045839	Isoform 3 of Prolyl 3-hydroxylase 1 precursor	LEPRE1	1
IPI00008404	Isoform Long of Segment polarity protein dishevelled homolog DVL-1-like	DVL1L1	1
IPI00658025	Putative novel transcript	-	1
IPI00022958	PRO0149	C16orf72	1
IPI00006987	ATP-dependent RNA helicase DDX24	DDX24	1
IPI00023162	UDP-N-acetylglucosamine 2-epimerase/N-acetylmannosamine kinase	GNE	1
IPI00019146	Isoform 1 of Coxsackievirus and adenovirus receptor precursor	CXADR	1
IPI00789181	115 kDa protein	PLCL1	1
IPI00742725	Conserved hypothetical protein	LOC388564	1
IPI00878755	43 kDa protein	-	1
IPI00152050	ataxin 2-binding protein 1 isoform 3	A2BP1	1
IPI00385480	Caskin-1	CASKIN1	1
IPI00465123	KIAA0415 gene product	KIAA0415	1
IPI00013495	Isoform 2 of ATP-binding cassette sub-family F member 1	ABCF1	1
IPI00398992	Isoform 1 of Chromodomain-helicase-DNA-binding protein 8	CHD8	1
IPI00827724	Rheumatoid factor Vh I region precursor (Fragment)	-	1
IPI00012441	Isoform 1 of Synaptojanin-1	SYNJ1	1
IPI00006054	Syntaphilin	SNPH	1
IPI00008438	40S ribosomal protein S10	RPS10	1
IPI00152849	Isoform 1 of G2/mitotic-specific cyclin-B3	CCNB3	1
IPI00029556	Uncharacterized protein C1orf105	C1orf105	1
IPI00217012	pleckstrin and Sec7 domain containing	PSD	1
IPI00218730	Rod cGMP-specific 3',5'-cyclic phosphodiesterase subunit alpha	PDE6A	1
IPI00215914	ADP-ribosylation factor 1	ARF1	1
IPI00290826	Transmembrane protein 157 precursor	TMEM157	1
IPI00553092	V3-3 protein	IGLV7-46	1
IPI00015980	Isoform 2 of Multiple PDZ domain protein	MPDZ	1
IPI00830122	A30	-	1
IPI00641251	CD320 antigen precursor	CD320	1
IPI00103853	Isoform 1 of Putative ribosome-binding factor A, mitochondrial precursor	C18orf22	1
IPI00301465	14-3-3-associated AKT substrate	HJURP	1
IPI00102678	Isoform 1 of Pecanex-like protein 1	PCNX	1
IPI00020356	331 kDa protein	MAP1A	1
IPI00008226	73 kDa protein	THSD3	1
IPI00010903	Dopey family member 1	DOPEY1	1
IPI00735934	similar to capicua homolog	LOC646070	1
IPI00465234	Cytokine receptor common beta chain precursor	CSF2RB	1
IPI00010303	Serpin B4	SERPINB4	1

IPI00008091	Putative DNA helicase INO80 complex homolog 1	INOC1	1
IPI00329791	cDNA FLJ78679, highly similar to Homo sapiens DEAD (Asp-Glu-Ala-Asp) box polypeptic	DDX46	1
IPI00152344	Pyridoxal phosphate phosphatase PHOSPHO2	PHOSPHO2	1
IPI00465363	Histone H2B type 1-A	HIST1H2BA	1
IPI00028601	Putative metallothionein C20orf127	MT1P3	1
IPI00011781	Ankyrin repeat-containing protein C20orf86 precursor	C20orf86	1
IPI00419908	Uncharacterized protein GPR179	GPR179	1
IPI00479125	SLIT-ROBO Rho GTPase-activating protein 2	SRGAP2	1
IPI00455852	Isoform 1 of Rho guanine nucleotide exchange factor 15	ARHGEF15	1
IPI00174976	Isoform 1 of MAGUK p55 subfamily member 5	MPP5	1
IPI00550876	Protein maestro	MRO	1
IPI00795481	Isoform 1 of Ly6/PLAUR domain-containing protein 1 precursor	LYPD1	1
IPI00064296	PRO0633	-	1
IPI00002459	annexin VI isoform 2	ANXA6	1
IPI00025311	Isoform 1 of Breast carcinoma-amplified sequence 1	BCAS1	1
IPI00216651	Isoform 1 of Interleukin-28 receptor alpha chain precursor	IL28RA	1
IPI00216774	Cerebellin-2	CBLN2	1
IPI00792945	38 kDa protein	CHFR	1
IPI00027464	Calcineurin subunit B isoform 1	PPP3R1	1
IPI00293530	C3a anaphylatoxin chemotactic receptor	C3AR1	1
IPI00399180	Serine/threonine-protein kinase SBK1	SBK1	1
IPI00007512	Glutathione transferase omega-2	GSTO2	1
IPI00016576	Isoform 1 of Grainyhead-like protein 2 homolog	GRHL2	1
IPI00290308	Tribbles homolog 1	TRIB1	1
IPI00005859	Keratin, type II cytoskeletal 75	KRT75	1
IPI00025622	AN1-type zinc finger protein 5	ZFAND5	1
IPI00019901	Isoform 1 of Alpha-adducin	ADD1	1
IPI00102808	Isoform 1 of Dual specificity protein phosphatase 19	DUSP19	1
IPI00073763	Semaphorin-4C precursor	SEMA4C	1
IPI00018208	Tetratricopeptide repeat protein 33	TTC33	1
IPI00166865	CDGSH iron sulfur domain-containing protein 2	CISD2	1
IPI00477468	RNA polymerase-associated protein CTR9 homolog	CTR9	1
IPI00005668	Aldo-keto reductase family 1 member C2	AKR1C2	1
IPI00018098	Isoform 1 of Pre-mRNA-splicing factor 38B	PRPF38B	1
IPI00243338	24 kDa protein	KRT23	1
IPI00786937	similar to deleted in malignant brain tumors 1 isoform b precursor	LOC731940	1
IPI00027744	Isoform 1 of Mineralocorticoid receptor	NR3C2	1
IPI00718806	arylhydrocarbon receptor repressor	AHRR;PDCD6	1
IPI00024032	TBC1 domain family, member 29	TBC1D29	1
IPI00744226	Conserved hypothetical protein	-	1

IPI00792229	20 kDa protein	TATDN1	1
IPI00000144	Oxytocin-neurophysin 1 precursor	OXT	1
IPI00786893	similar to LYRIC/3D3	LOC730296;LC	1
IPI00015954	GTP-binding protein SAR1a	SAR1A	1
IPI00099838	Isoform 1 of Lysophosphatidic acid phosphatase type 6 precursor	ACP6	1
IPI00397949	G protein-coupled receptor 56 isoform b	GPR56	1
IPI00418735	hypothetical protein LOC400566	LOC400566	1
IPI00306413	Tubulin polymerization-promoting protein family member 3	TPPP3	1
IPI00018755	High mobility group protein 1-like 10	HMG1L10	1
IPI00385543	Isoform 3 of UPF0469 protein KIAA0907	KIAA0907	1
IPI00299076	Receptor-binding cancer antigen expressed on SiSo cells (Fragment)	EBAG9	1
IPI00386393	CDNA FLJ13729 fis, clone PLACE3000121, weakly similar to VESICULAR TRAFFIC CO	EXOC6B	1
IPI00878511	45 kDa protein	-	1
IPI00816155	Isoform 2 of Complement C1q-like protein 3 precursor	C1QL3	1
IPI00442564	CDNA FLJ26948 fis, clone RCT08241	-	1
IPI00412408	Breast cancer type 2 susceptibility protein	BRCA2	1
IPI00008422	Isoform 2 of SWI/SNF-related matrix-associated actin-dependent regulator of chromatin s	SMARCAD1	1
IPI00746177	similar to Tubulin alpha-2 chain	LOC730222	1
IPI00550792	Isoform 1 of Bridging integrator 2	BIN2	1
IPI00029175	Strumpellin	KIAA0196	1
IPI00045360	Capicua-like protein/double homeodomain 4 fusion protein	CIC	1
IPI00001755	Glypican-6 precursor	GPC6	1
IPI00749171	Conserved hypothetical protein	LOC340184	1
IPI00033419	Protein Fem-1 homolog b	FEM1B	1
IPI00170635	Secreted and transmembrane protein 1 precursor	SECTM1	1
IPI00830018	Uncharacterized protein ENSP00000374807	-	1
IPI00026230	Heterogeneous nuclear ribonucleoprotein H2	HNRPH2	1
IPI00009365	COX16-like protein C14orf112, mitochondrial precursor	C14orf112	1
IPI00445278	CDNA FLJ44033 fis, clone TESTI4028062	-	1
IPI00644840	Hypothetical protein	LOC255783	1
IPI00301098	Uncharacterized protein C1orf187 precursor	C1orf187	1
IPI00009771	Lamin-B2	LMNB2	1
IPI00444842	CDNA FLJ45125 fis, clone BRAWH3036561	-	1
IPI00023152	Isoform 1 of N-acetylated-alpha-linked acidic dipeptidase-like protein	NAALADL1	1
IPI00296727	Kinesin-like protein KIF2B	KIF2B	1
IPI00005347	Zinc finger Ran-binding domain-containing protein 1	ZRANB1	1
IPI00470913	RANBP2-like and GRIP domain containing 1	RGPD1;RGPD2	1
IPI00023407	Nck-associated protein 1-like	NCKAP1L	1
IPI00306046	Isoform 1 of EGF-like repeat and discoidin I-like domain-containing protein 3 precursor	EDIL3	1
IPI00445315	Protein FAM47C	FAM47C	1

IPI00065931	Isoform 2 of A-kinase anchor protein 13	AKAP13	1
IPI00149044	Isoform 2 of Suppressor of hairy wing homolog 4	ZNF280D	1
IPI00007834	Isoform 1 of Ankyrin-2	ANK2	1
IPI00003971	Isoform RTN1-A of Reticulon-1	RTN1	1
IPI00746681	Similar to Bcl-2-related ovarian killer protein	-	1
IPI00383808	Ig kappa chain V-IV region STH (Fragment)	-	1
IPI00871556	107 kDa protein	ZFYVE28	1
IPI00746666	hypothetical protein	LOC728262	1
IPI00003814	Isoform 1 of Dual specificity mitogen-activated protein kinase kinase 6	MAP2K6	1
IPI00245940	immunoglobulin superfamily 5 like	IGSF5	1
IPI00657699	Protein	MUC19	1
IPI00290094	Splicing factor, arginine/serine-rich 8	SFRS8	1
IPI00413826	similar to H3 histone, family 3B	LOC644914	1
IPI00030706	Activator of 90 kDa heat shock protein ATPase homolog 1	AHSA1	1
IPI00410093	coiled-coil domain containing 69	CCDC69	1
IPI00168404	Zinc finger and BTB domain containing 34	ZBTB34	1
IPI00644766	cDNA FLJ78048, highly similar to Homo sapiens torsin A interacting protein 1, mRNA	TOR1AIP1	1
IPI00167154	Uncharacterized protein MAPKBP1	MAPKBP1	1
IPI00871533	Uncharacterized protein C3orf48 (Fragment)	C3orf48	1
IPI00877615	15 kDa protein	-	1
IPI00030352	Isoform 2 of Growth inhibition and differentiation-related protein 88	C10orf28	1
IPI00221080	Isoform 2 of Parathyroid hormone-related protein precursor	PTHLH	1
IPI00032338	kelch-like 20	KLHL20	1
IPI00293679	Isoform 1 of Potassium voltage-gated channel subfamily KQT member 4	KCNQ4	1
IPI00152769	Isoform 1 of Trpc4-associated protein	TRPC4AP	1
IPI00748682	Pheromone shutdown-related, TraB family protein	-	1
IPI00019888	Succinate-semialdehyde dehydrogenase, mitochondrial precursor	ALDH5A1	1
IPI00291987	Insulin-like growth factor-binding protein-like 1 precursor	IGFBPL1	1
IPI00170766	Isoform 2 of Protein CASC5	CASC5	1
IPI00032597	RNA-binding motif protein, X-linked 2	RBMX2	1
IPI00783855	neighbor of BRCA1 gene 1	NBR1	1
IPI00746987	Ribosomal protein S1 family protein	-	1
IPI00295469	Copine-6	CPNE6	1
IPI00044326	Carbohydrate sulfotransferase 14	CHST14	1
IPI00884004	Rheumatoid factor RF-ET12 (Fragment)	-	1
IPI00478997	V5-6 protein	IGLV4-69	1
IPI00456599	hypothetical protein LOC84792	MGC12966	1
IPI00607831	PRAME family member 3	PRAMEF3	1
IPI00399254	Isoform 1 of OTU domain-containing protein 4	OTUD4	1
IPI00171230	Isoform 2 of ELKS/RAB6-interacting/CAST family member 1	ERC1	1

IPI00719505	RABL2A protein	RABL2A	1
IPI00784880	Cancer/testis antigen 75	LOC440934	1
IPI00741005	similar to MAX-interacting protein isoform 4	MGA	1
IPI00884353	Ets-1 transcript variant ets-1 delta	-	1
IPI00852725	Isoform 7 of Prolactin receptor precursor	PRLR	1
IPI00005600	Isoform 1 of Bifunctional heparan sulfate N-deacetylase/N-sulfotransferase 2	NDST2	1
IPI00791593	8 kDa protein	GLYCAM1	1
IPI00293396	adaptor-related protein complex 1, gamma 1 subunit isoform a	AP1G1	1
IPI00018914	Tyrosine-protein phosphatase non-receptor type 14	PTPN14	1
IPI00185146	Importin-9	IPO9	1
IPI00143753	Isoform 1 of U2-associated protein SR140	SR140	1
IPI00217791	Coiled-coil domain-containing protein 105	CCDC105	1
IPI00167006	Uncharacterized protein C13orf26	C13orf26	1
IPI00028481	Ras-related protein Rab-8A	RAB8A	1
IPI00294215	Uncharacterized protein KIAA0232	KIAA0232	1
IPI00027806	Cysteine-rich secretory protein LCCL domain-containing 1 precursor	CRISPLD1	1
IPI00071185	Isoform SV1 of PITSLRE serine/threonine-protein kinase CDC2L1	CDC2L1	1
IPI00166776	Protein CREG2 precursor	CREG2	1
IPI00853400	Isoform 1 of FK506-binding protein 15	FKBP15	1
IPI00473033	Isoform 1 of Zinc finger protein 69	ZNF69	1
IPI00749245	Secreted frizzled-related protein 1 precursor	SFRP1	1
IPI00152182	Isoform 1 of Kelch domain-containing protein 4	KLHDC4	1
IPI00783604	EPH receptor A6 isoform a	EPHA6	1
IPI00456969	Dynein heavy chain, cytosolic	DYNC1H1	1
IPI00001796	Tumor necrosis factor receptor superfamily, member 18 (Fragment)	TNFRSF18	1
IPI00549972	LIM domain-containing protein 2	LIMD2	1
IPI00604551	Isoform 1 of Cell division cycle-associated protein 7	CDCA7	1
IPI00470468	Isoform 3 of Protein EFR3 homolog A	EFR3A	1
IPI00382515	CDNA FLJ30384 fis, clone BRACE2008114	-	1
IPI00853312	Uncharacterized protein ENSP00000324580	-	1
IPI00644191	70 kDa protein	ZNF90	1
IPI00470490	Isoform 1 of Nuclear receptor coactivator 1	NCOA1	1
IPI00001433	Protocadherin beta 15 precursor	PCDHB15	1
IPI00220156	Isoform B of Transforming growth factor beta-2 precursor	TGFB2	1
IPI00024818	Isoform 1 of Ubiquitin-specific peptidase-like protein 1	USPL1	1
IPI00335437	Ankyrin repeat and zinc finger domain-containing protein 1	ANKZF1	1
IPI00000076	Beta-nerve growth factor precursor	NGFB	1
IPI00290078	keratin 4	KRT4	1
IPI00100980	EH domain-containing protein 2	EHD2	1
IPI00328361	Seryl-tRNA synthetase, mitochondrial precursor	SARS2	1

IPI00022277	Coiled-coil domain-containing protein 56	CCDC56	1
IPI00216288	Isoform 3 of Lethal	L3MBTL	1
IPI00025499	Isoform Tau-F of Microtubule-associated protein tau	MAPT	1
IPI00030757	Isoform LpNPI of ADAMTS-2 precursor	ADAMTS2	1
IPI00787083	similar to peptidylprolyl isomerase A isoform 1	LOC256374	1
IPI00856012	collagen type VI alpha 6	COL6A6	1
IPI00001712	Isoform 1 of Catenin alpha-3	CTNNA3	1
IPI00216592	Isoform C1 of Heterogeneous nuclear ribonucleoproteins C1/C2	HNRNPC	1
IPI00217831	Ankyrin repeat domain-containing protein 13A	ANKRD13A	1
IPI00030037	Agouti-signaling protein precursor	ASIP	1
IPI00013749	Protein kinase C zeta type	PRKCZ	1
IPI00645078	Ubiquitin-like modifier-activating enzyme 1	UBA1	1
IPI00395866	SCUBE1 protein	SCUBE1	1
IPI00240793	Probable phospholipid-transporting ATPase IF	ATP11B	1
IPI00376237	Isoform 2 of Transcription factor LBX2	LBX2	1
IPI00847723	Similar to VH4 heavy chain variable region precursor	-	1
IPI00169115	Olfactory receptor OR9-8	OR13C3	1
IPI00020966	Isoform 1 of Phosphatidylinositol N-acetylglucosaminyltransferase subunit A	PIGA	1
IPI00456827	Protein FAM22G precursor	FAM22G	1
IPI00747657	Similar to Rod cGMP-specific 3',5'-cyclic phosphodiesterase subunit beta precursor	PDE6B	1
IPI00215777	Isoform B of Phosphate carrier protein, mitochondrial precursor	SLC25A3	1
IPI00028912	zinc finger protein 161	VEZF1	1
IPI00003807	Lysosomal acid phosphatase precursor	ACP2	1
IPI00830107	V4-2 protein	IGLV5-45	1
IPI00019242	Matrix metalloproteinase-15 precursor	MMP15	1
IPI00786946	similar to Tektin-3	LOC642249	1
IPI00166257	CDNA FLJ37614 fis, clone BRCOC2011769	-	1
IPI00011592	Cytoplasmic dynein 1 light intermediate chain 2	DYNC1LI2	1
IPI00442745	CDNA FLJ26780 fis, clone PRS03837	-	1
IPI00374301	hypothetical protein	-	1
IPI00024920	ATP synthase subunit delta, mitochondrial precursor	ATP5D	1
IPI00026262	Isoform 1 of Ras GTPase-activating protein 1	RASA1	1
IPI00018843	Isoform 1 of D(3) dopamine receptor	DRD3	1
IPI00027782	Stromelysin-1 precursor	MMP3	1
IPI00030009	Isoform A of Bifunctional 3'-phosphoadenosine 5'-phosphosulfate synthetase 2	PAPSS2	1
IPI00177878	Isoform 3 of Transmembrane protein 16D	TMEM16D	1
IPI00104907	Isoform 1 of Uncharacterized potential DNA-binding protein C14orf106	C14orf106	1
IPI00827978	VL4 protein (Fragment)	-	1
IPI00005107	Niemann-Pick C1 protein precursor	NPC1	1
IPI00152216	Isoform 1 of Protein RIC-3 precursor	RIC3	1

IPI00028520	Isoform 1 of NADH dehydrogenase [ubiquinone] flavoprotein 1, mitochondrial precursor	NDUFV1	1
IPI00290358	Putative uncharacterized protein gs103	LOC283951	1
IPI00013272	Isoform 1 of Golgin subfamily A member 4	GOLGA4	1
IPI00009070	Isoform 1 of HBS1-like protein	HBS1L	1
IPI00386576	Ig lambda chain V-IV region MOL	-	1
IPI00022295	Platelet factor 4 variant precursor	PF4V1	1
IPI00747420	Melanoma-derived protein (Fragment)	-	1
IPI00432226	AVLL5809	-	1
IPI00171874	Ras guanyl-releasing protein 3	RASGRP3	1
IPI00165229	proprotein convertase subtilisin/kexin type 5 preproprotein	PCSK5	1
IPI00006746	Ermin	ERMN	1
IPI00178894	Zinc finger and BTB domain-containing protein 20	ZBTB20	1
IPI00878436	24 kDa protein	SLC2A11	1
IPI00640810	6 kDa protein	CTDP1	1
IPI00845229	Isoform 2 of DEP domain-containing protein 2	DEPDC2	1
IPI00306549	CDNA FLJ11065 fis, clone PLACE1004868, weakly similar to MALE STERILITY PROTEI	MLSTD1	1
IPI00003392	Transmembrane protein 5	TMEM5	1
IPI00293361	Isoform 2 of Small G protein signaling modulator 2	SGSM2	1
IPI00827788	VH-3 family (VH26)D/J protein (Fragment)	-	1
IPI00253281	Isoform 2 of Epidermal growth factor receptor kinase substrate 8-like protein 1	EPS8L1	1
IPI00005607	Isoform 1 of Deleted in bladder cancer protein 1 precursor	DBC1	1
IPI00014371	Cadherin-18 precursor	CDH18	1
IPI00329605	DNA mismatch repair protein Msh3	MSH3	1
IPI00032425	Receptor activity-modifying protein 3 precursor	RAMP3	1
IPI00300020	Excitatory amino acid transporter 2	SLC1A2	1
IPI00001568	Vacuolar proton pump subunit D	ATP6V1D	1
IPI00020470	Isoform 1 of Glycosyltransferase 8 domain-containing protein 1	GLT8D1	1
IPI00718821	Isoform 1 of Uncharacterized protein C19orf55	C19orf55	1
IPI00018387	Furin precursor	FURIN	1
IPI00398229	similar to deubiquitinating enzyme 3	LOC392197	1
IPI00027248	Tumor suppressor candidate 2	TUSC2	1
IPI00438286	Isoform 1 of Protein LAP2	ERBB2IP	1
IPI00023780	Isoform 2 of DnaJ homolog subfamily C member 5	DNAJC5	1
IPI00018027	Isoform 1 of Angiogenic factor with G patch and FHA domains 1	AGGF1	1
IPI00434711	Putative uncharacterized protein FP6679	-	1
IPI00009881	Neuroendocrine secretory protein 55	GNAS	1
IPI00020329	Potassium voltage-gated channel subfamily S member 2	KCNS2	1
IPI00243451	Liver-specific organic anion transporter 3TM12	SLCO1B3;LST-	1
IPI00184650	Class B basic helix-loop-helix protein 4	BHLHB4	1
IPI00328298	Isoform 2 of Structural maintenance of chromosomes protein 4	SMC4	1

IPI00386284	olfactory receptor, family 2, subfamily AK, member 2	OR2AK2	1
IPI00289329	Ephrin type-B receptor 3 precursor	EPHB3	1
IPI00018429	Paired mesoderm homeobox protein 2	PRRX2	1
IPI00032904	Beta-synuclein	SNCB	1
IPI00424119	Frizzled-3 precursor	FZD3	1
IPI00387004	PNAS-146	MCM7	1
IPI00218946	Potassium/sodium hyperpolarization-activated cyclic nucleotide-gated channel 2	HCN2	1
IPI00024766	Plexin-C1 precursor	PLXNC1	1
IPI00009471	WD repeat-containing protein 3	WDR3	1
IPI00455967	Uncharacterized protein ENSP00000353619 (Fragment)	-	1
IPI00186966	Isoform IIA of Myc box-dependent-interacting protein 1	BIN1	1
IPI00045939	2-aminoethanethiol dioxygenase	ADO	1
IPI00165009	Isoform 3 of MBT domain-containing protein 1	MBTD1	1
IPI00374670	hypothetical protein isoform 2	LOC388588	1
IPI00289837	Coiled-coil domain-containing protein 85A	CCDC85A	1
IPI00027264	Calretinin	CALB2	1
IPI00878962	10 kDa protein	-	1
IPI00103891	Putative uncharacterized protein	MTMR14	1
IPI00328709	Gremlin-2 precursor	GREM2	1
IPI00398154	actin filament associated protein 1	AFAP1	1
IPI00291939	Structural maintenance of chromosomes protein 1A	SMC1A	1
IPI00444272	Leukemia inhibitory factor receptor precursor	LIFR	1
IPI00641181	MARCKS-related protein	MARCKSL1	1
IPI00418125	Isoform 1 of Layilin precursor	LAYN	1
IPI00151990	Isoform 1 of Thioredoxin domain-containing protein 15 precursor	TXNDC15	1
IPI00024272	Integral membrane protein DGCR2/IDD precursor	DGCR2	1
IPI00003384	Isoform 1 of Cadherin EGF LAG seven-pass G-type receptor 1 precursor	CELSR1	1
IPI00657936	collagen, type XXVIII precursor	COL28A1	1
IPI00329593	Isoform 2 of ADP-dependent glucokinase	ADPGK	1
IPI00296461	Isoform 1 of Sphingomyelin phosphodiesterase precursor	SMPD1	1
IPI00376383	Centrosomal protein 110kDa	CEP110	1
IPI00015973	Band 4.1-like protein 2	EPB41L2	1
IPI00304789	27 kDa protein	TTLL9	1
IPI00792759	73 kDa protein	THSD4	1
IPI00446588	Isoform 2 of Plexin-A4 precursor	PLXNA4	1
IPI00419836	Isoform 1 of Discoidin, CUB and LCCL domain-containing protein 2 precursor	DCBLD2	1
IPI00217435	Signal peptide, CUB and EGF-like domain-containing protein 1 precursor	SCUBE1	1
IPI00021766	Isoform 1 of Reticulon-4	RTN4	1
IPI00221006	Isoform 4 of Transcription factor 7-like 2	TCF7L2	1

√	detected in proteomic data
√*	detected in peptidomic data
