# Supplementary material for: Establishing the Proteome of Normal Human Cerebrospinal Fluid
Source: PLoS One. 2010 Jun 11;5(6):e10980. doi: 10.1371/journal.pone.0010980 (PMC2881861; doi:10.1371/journal.pone.0010980)
Supplement: Table S3 — Proteins identified and quantified from direct LC-MS analysis of CSF from non-neurologic and neurologic (headache) surrogate-normals. (0.02 MB PDF) [file pone.0010980.s010.pdf]

Supplementary Table 3. Proteins identified and quantified from direct LC-MS analysis of CSF from non-neurologic and neurologic (headache) surrogate-normals.

IPI	Protein Name	Gene	unique peptide count	p-value	q-value	average fold change
IPI00410714	Hemoglobin subunit alpha	HBA2;HBA1	16	4.96E-05	2.27E-03	14.58
IPI00654755	Hemoglobin subunit beta	HBB	7	8.63E-05	2.27E-03	8.93
IPI00654755	Hemoglobin subunit delta	HBB;HBD	7	1.10E-04	2.27E-03	17.46
IPI00021000	Isoform A of Osteopontin precursor	SPP1	10	1.25E-04	2.27E-03	50.56
IPI00298497	Fibrinogen beta chain precursor	FGB	10	2.07E-04	2.94E-03	0.65
IPI00008944	Isoform 1 of Neuroendocrine protein 7B2 precursor	SCG5	4	2.44E-04	2.94E-03	5.35
IPI00022488	Hemopexin precursor	HPX	25	3.89E-04	4.01E-03	1.53
IPI00410714	Hemoglobin subunit zeta	HBZ	2	1.46E-03	1.32E-02	7.96
IPI00026314	Isoform 1 of Gelsolin precursor	GSN	53	2.36E-03	1.90E-02	2.17
IPI00069058	VGF nerve growth factor inducible precursor	VGF	20	3.34E-03	2.41E-02	6.79
IPI00292071	Secretogranin-3 precursor	SCG3	23	3.84E-03	2.53E-02	1.78
IPI00384697	Uncharacterized protein ALB	ALB	83	5.28E-03	2.84E-02	1.58
IPI00296537	Isoform C of Fibulin-1 precursor	FBLN1	4	5.39E-03	2.84E-02	1.73
IPI00029863	SERPINF2 protein	SERPINF2	14	5.50E-03	2.84E-02	2.75
IPI00022417	Leucine-rich alpha-2-glycoprotein precursor	LRG1	6	6.69E-03	2.86E-02	0.55
IPI00641737	Isoform 1 of Haptoglobin-related protein precursor	HPR	7	6.99E-03	2.86E-02	2.37
IPI00102543	SLIT and NTRK-like protein 1 precursor	SLITRK1	3	7.00E-03	2.86E-02	0.06
IPI00553177	PRO2275	SERPINA1	33	7.11E-03	2.86E-02	1.93
IPI00334238	neuronal pentraxin receptor	NPTXR	8	7.77E-03	2.96E-02	0.70
IPI00032179	Antithrombin III variant	SERPINC1	25	8.29E-03	3.00E-02	1.48
IPI00784810	FLJ00385 protein (Fragment)	IGHM	23	9.02E-03	3.10E-02	1.67
IPI00305461	Inter-alpha-trypsin inhibitor heavy chain H2 precursor	ITIH2	16	9.66E-03	3.17E-02	1.52
IPI00550991	Alpha-1-antichymotrypsin precursor	SERPINA3	35	1.20E-02	3.77E-02	5.29
IPI00746813	Chromogranin-A precursor	CHGA	14	1.30E-02	3.93E-02	12.61
IPI00853525	Apolipoprotein A-I precursor	APOA1	49	1.44E-02	4.16E-02	1.42
IPI00456623	Isoform 1 of Brevican core protein precursor	BCAN	3	1.51E-02	4.21E-02	6.07
IPI00006114	Pigment epithelium-derived factor precursor	SERPINF1	33	1.69E-02	4.51E-02	1.41
IPI00742696	vitamin D-binding protein precursor	GC	2	1.75E-02	4.51E-02	1.55
IPI00019399	Serum amyloid A-4 protein precursor	SAA4	3	2.21E-02	5.50E-02	2.06
IPI00021891	Isoform Gamma-B of Fibrinogen gamma chain precursor	FGG	7	2.61E-02	6.30E-02	0.70
IPI00418163	C4B1	C4B	10	2.82E-02	6.43E-02	1.82
IPI00299059	precursor	CHL1	19	2.85E-02	6.43E-02	1.40

IPI00025426	Pregnancy zone protein precursor	PZP	7	3.47E-02	7.59E-02	1.40
IPI00027827	Extracellular superoxide dismutase [Cu-Zn] precursor	SOD3	7	3.74E-02	7.94E-02	1.56
IPI00013303	Limbic system-associated membrane protein precursor	LSAMP	8	4.50E-02	8.98E-02	2.30
IPI00304273	Apolipoprotein A-IV precursor	APOA4	35	4.52E-02	8.98E-02	1.35
IPI00167093	Complement factor H-related protein 1 precursor	CFHR1	2	4.67E-02	8.98E-02	0.26
IPI00007257	calsyntenin 1 isoform 2	CLSTN1	14	4.78E-02	8.98E-02	1.33
IPI00022229	Apolipoprotein B-100 precursor	APOB	2	4.94E-02	8.98E-02	1.66
IPI00011218	Macrophage colony-stimulating factor 1 receptor precursor	CSF1R	3	4.97E-02	8.98E-02	1.57
IPI00022429	Alpha-1-acid glycoprotein 1 precursor	ORM1	9	5.09E-02	8.98E-02	1.44
IPI00006601	Secretogranin-1 precursor	CHGB	40	5.24E-02	8.98E-02	3.86
IPI00016915	Insulin-like growth factor-binding protein 7 precursor	IGFBP7	7	5.53E-02	8.98E-02	1.62
IPI00784589	IGL@ protein	IGL@	16	5.63E-02	8.98E-02	1.36
IPI00013179	Prostaglandin-H2 D-isomerase precursor	PTGDS	28	5.69E-02	8.98E-02	1.33
IPI00334282	Protein FAM3C precursor	FAM3C	8	5.72E-02	8.98E-02	1.49
IPI00641737	HP protein	HP	13	5.99E-02	9.21E-02	1.37
IPI00021857	Apolipoprotein C-III precursor	APOC3	3	6.24E-02	9.25E-02	1.39
IPI00017601	Ceruloplasmin precursor	CP	34	6.30E-02	9.25E-02	1.44
IPI00019568	Prothrombin precursor (Fragment)	F2	7	6.40E-02	9.25E-02	1.65
IPI00845354	IGKV1-5 protein	IGKV1-5	8	6.55E-02	9.28E-02	1.71
IPI00006662	Apolipoprotein D precursor	APOD	9	6.79E-02	9.44E-02	0.85
IPI00217778	Isoform 2 of Phospholipid transfer protein precursor	PLTP	2	7.37E-02	1.01E-01	0.56
IPI00296777	SPARC-like protein 1 precursor	SPARCL1	13	7.81E-02	1.04E-01	1.55
IPI00166729	alpha-2-glycoprotein 1, zinc	AZGP1	9	7.92E-02	1.04E-01	0.81
IPI00296534	Isoform D of Fibulin-1 precursor	FBLN1	2	8.09E-02	1.04E-01	2.08
IPI00426051	Putative uncharacterized protein DKFZp686I04196 (Fragment)	IGHG2	9	8.74E-02	1.10E-01	1.53
IPI00015260	Protein kinase C-binding protein NELL2 precursor	NELL2	10	8.79E-02	1.10E-01	1.53
IPI00019580	Plasminogen precursor	PLG	11	9.07E-02	1.11E-01	1.50
IPI00451624	Isoform 1 of Cartilage acidic protein 1 precursor	CRTAC1	9	9.43E-02	1.12E-01	1.54
IPI00004656	Beta-2-microglobulin	B2M	6	9.47E-02	1.12E-01	0.39
IPI00009997	N-acetyllactosaminide beta-1,3-N-acetylglucosaminyltransferase	B3GNT1	16	9.97E-02	1.16E-01	0.68
IPI00294004	Vitamin K-dependent protein S precursor	PROS1	5	1.05E-01	1.20E-01	1.46
IPI00783987	Complement C3 precursor (Fragment)	C3	99	1.08E-01	1.22E-01	1.34
IPI00296534	Isoform B of Fibulin-1 precursor	FBLN1	6	1.10E-01	1.22E-01	1.35
IPI00795918	isoform precursor	NCAM1	10	1.16E-01	1.25E-01	1.49
IPI00291262	Clusterin precursor	CLU	42	1.16E-01	1.25E-01	0.74
IPI00022463	Serotransferrin precursor	TF	57	1.18E-01	1.26E-01	1.22
IPI00156171	family member 2 precursor	ENPP2	18	1.20E-01	1.26E-01	1.36
IPI00844156	SERPINC1 protein	SERPINC1	5	1.27E-01	1.31E-01	1.47

IPI00032293	Cystatin-C precursor	CST3	30	1.30E-01	1.31E-01	1.27
IPI00019591	Isoform 1 of Complement factor B precursor (Fragment)	CFB	27	1.31E-01	1.31E-01	1.29
IPI00219217	L-lactate dehydrogenase B chain	LDHB	3	1.33E-01	1.32E-01	3.28
IPI00022432	Transthyretin precursor	TTR	25	1.38E-01	1.34E-01	1.97
IPI00477992	complement component 1, q subcomponent, B chain precursor	C1QB	3	1.42E-01	1.34E-01	2.16
IPI00301579	Epididymal secretory protein E1 precursor	NPC2	3	1.43E-01	1.34E-01	1.30
IPI00177543	preproprotein	PAM	4	1.44E-01	1.34E-01	1.21
IPI00218192	Isoform 2 of Inter-alpha-trypsin inhibitor heavy chain H4 precursor	ITIH4	17	1.47E-01	1.34E-01	1.44
IPI00220327	Keratin, type II cytoskeletal 1	KRT1	4	1.47E-01	1.34E-01	0.24
IPI00385252	Ig kappa chain V-III region HAH precursor	IGKV3-20	4	1.51E-01	1.34E-01	1.33
IPI00296608	Complement component C7 precursor	C7	7	1.52E-01	1.34E-01	0.66
IPI00022394	Complement C1q subcomponent subunit C precursor	C1QC	3	1.52E-01	1.34E-01	0.85
IPI00242956	IgGFc-binding protein precursor	FCGBP	2	1.54E-01	1.34E-01	0.36
IPI00218733	Superoxide dismutase	SOD1	7	1.63E-01	1.40E-01	1.54
IPI00418163	Complement C4-A precursor	C4A;C4B	79	1.77E-01	1.51E-01	1.26
IPI00022431	Alpha-2-HS-glycoprotein precursor	AHSG	9	1.84E-01	1.54E-01	1.34
IPI00784810	Factor VII active site mutant immunoconjugate	F7	8	1.87E-01	1.56E-01	1.48
IPI00028911	Dystroglycan precursor	DAG1	5	1.90E-01	1.56E-01	0.69
IPI00478003	Alpha-2-macroglobulin precursor	A2M	52	1.94E-01	1.57E-01	1.31
IPI00296165	Complement C1r subcomponent precursor	CYP1	5	1.97E-01	1.58E-01	0.29
IPI00024966	Contactin-2 precursor	CNTN2	5	2.01E-01	1.60E-01	4.16
IPI00854806	(Fragment)	IGKV1D-8	2	2.11E-01	1.65E-01	1.58
IPI00022420	Plasma retinol-binding protein precursor	RBP4	6	2.13E-01	1.65E-01	1.25
IPI00020091	Alpha-1-acid glycoprotein 2 precursor	ORM2	10	2.14E-01	1.65E-01	1.22
IPI00031121	Carboxypeptidase E precursor	CPE	5	2.31E-01	1.76E-01	1.47
IPI00023673	Galectin-3-binding protein precursor	LGALS3BP	6	2.35E-01	1.77E-01	1.29
IPI00219446	Phosphatidylethanolamine-binding protein 1	PEBP1	4	2.38E-01	1.77E-01	1.62
IPI00064667	Beta-Ala-His dipeptidase precursor	CNDP1	27	2.52E-01	1.84E-01	0.82
IPI00290085	Cadherin-2 precursor	CDH2	6	2.52E-01	1.84E-01	1.37
IPI00023845	Kallikrein-6 precursor	KLK6	6	2.58E-01	1.87E-01	0.93
IPI00742696	Vitamin D-binding protein precursor	GC	16	2.66E-01	1.89E-01	1.32
IPI00180384	dynein, axonemal, heavy chain 7	DNAH7	2	2.67E-01	1.89E-01	2.28
IPI00332887	signal-regulatory protein alpha precursor	SIRPA	2	2.76E-01	1.94E-01	0.93
IPI00006608	Isoform APP770 of Amyloid beta A4 protein precursor (Fragment)	APP	16	2.81E-01	1.95E-01	1.21
IPI00022395	Complement component C9 precursor	C9	6	2.84E-01	1.95E-01	1.18
IPI00025465	Mimecan precursor	OGN	14	2.87E-01	1.96E-01	0.81
IPI00027482	Corticosteroid-binding globulin precursor	SERPINA6	3	2.91E-01	1.96E-01	1.79
IPI00607600	Amyloid-like protein 1 precursor	APLP1	30	3.08E-01	2.06E-01	1.19

IPI00021842	Apolipoprotein E precursor	APOE	50	3.15E-01	2.09E-01	1.13
IPI00022892	Thy-1 membrane glycoprotein precursor	THY1	2	3.32E-01	2.18E-01	0.74
IPI00794070	Complement factor I precursor	CFI	2	3.41E-01	2.22E-01	0.50
IPI00415032	Isoform 1 of Neuronal cell adhesion molecule precursor	NRCAM	24	3.61E-01	2.33E-01	1.13
IPI00291866	Plasma protease C1 inhibitor precursor	SERPING1	15	3.69E-01	2.36E-01	0.84
IPI00783987	similar to Complement C3 precursor	LOC653879	11	3.80E-01	2.41E-01	1.79
IPI00176221	Neuronal growth regulator 1 precursor	NEGR1	3	3.83E-01	2.41E-01	1.75
IPI00002714	Dickkopf-related protein 3 precursor	DKK3	14	3.91E-01	2.44E-01	0.89
IPI00442297	Neurotrimin variant 3	HNT	4	4.06E-01	2.51E-01	1.19
IPI00384697	ALB protein	ALB	3	4.13E-01	2.53E-01	1.28
IPI00003351	Extracellular matrix protein 1 precursor	ECM1	6	4.17E-01	2.53E-01	1.32
IPI00456623	Hyaluronan binding protein (Fragment)	BCAN	4	4.30E-01	2.59E-01	0.72
IPI00007921	Isoform 1 of Neurexin-2-alpha precursor	NRXN2	4	4.40E-01	2.60E-01	1.48
IPI00292530	Inter-alpha-trypsin inhibitor heavy chain H1 precursor	ITIH1	4	4.42E-01	2.60E-01	1.35
IPI00163207	Isoform 1 of N-acetylmuramoyl-L-alanine amidase precursor	PGLYRP2	6	4.43E-01	2.60E-01	0.68
IPI00159927	Neurocan core protein precursor	NCAN	4	4.67E-01	2.72E-01	0.69
IPI00034319	Isoform A of Protein CutA precursor	CUTA	2	4.85E-01	2.78E-01	0.86
IPI00553177	Isoform 1 of Alpha-1-antitrypsin precursor	SERPINA1	35	4.87E-01	2.78E-01	1.17
IPI00029751	Isoform 1 of Contactin-1 precursor	CNTN1	19	4.90E-01	2.78E-01	1.28
IPI00011261	Complement component C8 gamma chain precursor	C8G	2	4.93E-01	2.78E-01	1.18
IPI00001611	Isoform 1 of Insulin-like growth factor II precursor	IGF2	2	5.08E-01	2.84E-01	0.88
IPI00024284	protein precursor	HSPG2	6	5.11E-01	2.84E-01	1.18
IPI00011229	Cathepsin D precursor	CTSD	9	5.18E-01	2.86E-01	1.20
IPI00029260	Monocyte differentiation antigen CD14 precursor	CD14	4	5.22E-01	2.86E-01	0.88
IPI00002147	Chitinase-3-like protein 1 precursor	CHI3L1	8	5.45E-01	2.96E-01	1.46
IPI00787781	Metalloproteinase inhibitor 2 precursor	TIMP2	2	5.54E-01	2.98E-01	1.22
IPI00332887	RcPTPNS1 (Fragment)	-	2	5.57E-01	2.98E-01	0.75
IPI00297160	Isoform 12 of CD44 antigen precursor	CD44	2	5.70E-01	3.01E-01	0.85
IPI00291136	Collagen alpha-1(VI) chain precursor	COL6A1	5	5.70E-01	3.01E-01	0.64
IPI00414249	Neurexin 3-alpha	NRXN3	2	5.86E-01	3.04E-01	1.28
IPI00382420	Ig lambda chain V-I region HA	-	2	5.90E-01	3.04E-01	1.45
IPI00026199	Glutathione peroxidase 3 precursor	GPX3	2	5.91E-01	3.04E-01	0.82
IPI00023814	Isoform 1 of Neogenin precursor	NEO1	4	5.93E-01	3.04E-01	1.79
IPI00021854	Apolipoprotein A-II precursor	APOA2	10	6.18E-01	3.15E-01	1.12
IPI00032328	Isoform HMW of Kininogen-1 precursor	KNG1	11	6.43E-01	3.24E-01	1.09
IPI00009362	Secretogranin-2 precursor	SCG2	13	6.45E-01	3.24E-01	0.90
IPI00022371	Histidine-rich glycoprotein precursor	HRG	7	6.78E-01	3.35E-01	1.10
IPI00298971	Vitronectin precursor	VTN	9	6.81E-01	3.35E-01	1.11

IPI00008318	Ephrin type-A receptor 4 precursor	EPHA4	4	6.82E-01	3.35E-01	1.09
IPI00298828	Beta-2-glycoprotein 1 precursor	APOH	3	6.90E-01	3.37E-01	0.93
IPI00029739	Isoform 1 of Complement factor H precursor	CFH	12	7.07E-01	3.41E-01	1.05
IPI00021885	Isoform 1 of Fibrinogen alpha chain precursor	FGA	15	7.08E-01	3.41E-01	0.93
IPI00020986	Lumican precursor	LUM	7	7.39E-01	3.53E-01	0.95
IPI00029235	Insulin-like growth factor-binding protein 6 precursor	IGFBP6	2	7.42E-01	3.53E-01	1.09
IPI00014964	Lymphocyte antigen 6H precursor	LY6H	2	7.53E-01	3.56E-01	1.23
IPI00217778	45 kDa protein	PLTP	5	7.58E-01	3.56E-01	1.12
IPI00012503	Isoform Sap-mu-0 of Proactivator polypeptide precursor	PSAP	3	7.72E-01	3.60E-01	0.80
IPI00002280	ProSAAS precursor	PCSK1N	10	7.84E-01	3.62E-01	0.97
IPI00792115	Tetranectin precursor	CLEC3B	5	7.86E-01	3.62E-01	1.05
IPI00001662	Opioid-binding protein/cell adhesion molecule precursor	OPCML	4	7.98E-01	3.65E-01	0.95
IPI00784119	Vacuolar ATP synthase subunit S1 precursor	ATP6AP1	2	8.02E-01	3.65E-01	1.04
IPI00017696	Complement C1s subcomponent precursor	C1S	7	8.27E-01	3.73E-01	1.06
IPI00029658	1 precursor	EFEMP1	8	8.32E-01	3.74E-01	0.93
IPI00032220	Angiotensinogen precursor	AGT	22	8.54E-01	3.81E-01	0.97
IPI00297646	Collagen alpha-1(I) chain precursor	COL1A1	2	9.07E-01	4.01E-01	1.03
IPI00022426	AMBP protein precursor	AMBP	2	9.17E-01	4.01E-01	0.94
IPI00022895	Alpha-1B-glycoprotein precursor	A1BG	17	9.18E-01	4.01E-01	1.02
IPI00292950	Serpin peptidase inhibitor, clade D (Heparin cofactor), member 1	SERPIND1	2	9.21E-01	4.01E-01	0.97
IPI00022792	Microfibril-associated glycoprotein 4 precursor	MFAP4	2	9.26E-01	4.01E-01	0.96
IPI00022331	Phosphatidylcholine-sterol acyltransferase precursor	LCAT	4	9.33E-01	4.01E-01	1.04
IPI00386879	IGHA1 protein	13;IGHA1	9	9.38E-01	4.01E-01	0.99
IPI00019943	Afamin precursor	AFM	9	9.48E-01	4.03E-01	0.97
IPI00299738	Procollagen C-endopeptidase enhancer 1 precursor	PCOLCE	2	9.65E-01	4.08E-01	1.02
IPI00004440	Receptor-type tyrosine-protein phosphatase-like N precursor	PTPRN	2			3.71
IPI00241562	reelin isoform a	RELN	3			2.89
IPI00018236	Ganglioside GM2 activator precursor	GM2A	2			2.86
IPI00026946	Neuronal pentraxin-2 precursor	NPTX2	3			1.73
IPI00220562	Neuronal pentraxin-1 precursor	NPTX1	4			1.72
IPI00019581	Coagulation factor XII precursor	F12	2			1.04
IPI00022822	Isoform 2 of Collagen alpha-1(XVIII) chain precursor	COL18A1	2			0.99
IPI00176427	Cell adhesion molecule 4 precursor	CADM4	2			0.91
IPI00000779	Isoform 1 of ADAM 22 precursor	ADAM22	3			0.78
IPI00022937	Coagulation factor V	F5	3			0.75
IPI00018219	Transforming growth factor-beta-induced protein ig-h3 precursor	TGFBI	2			0.62
IPI00009619	Isoform 2 of Cell adhesion molecule 3 precursor	CADM3	3			0.46
IPI00789234	Immunoglobulin V-set domain containing protein	VSTM2A	2			0.46

IPI00328520	Isoform 2 of Proline-rich transmembrane protein 2	PRRT2	2	0.24
